# Deep insights into the gut microbial community of extreme longevity in south Chinese centenarians by ultra-deep metagenomics and large-scale culturomics

**DOI:** 10.1038/s41522-022-00282-3

**Published:** 2022-04-19

**Authors:** Congyong Li, Zhe Luan, Yiming Zhao, Jun Chen, Yanan Yang, Cong Wang, Yujia Jing, Shirui Qi, Zhuanyu Li, Hao Guo, Wenyi Xu, Bowen Zhao, Chongming Wu, Shufang Wang, Yunsheng Yang, Gang Sun

**Affiliations:** 1 Sixth Health Care Department, Second Medical Center of PLA General Hospital, 100853 Beijing, China; 2 Department of Gastroenterology and Hepatology, First Medical Center of PLA General Hospital, 100853 Beijing, China; 3 Department of Gastroenterology and Hepatology, Hainan Hospital of PLA General Hospital, 572013 Sanya, China; 4 Unit 91917, 102401 Beijing, China; 5 School of Chinese Materia Medica, Tianjin University of Traditional Chinese Medicine, 301617 Tianjin, China; 6 Emergency Department, Tianjin First Central Hospital, 300192 Tianjin, China; 7 Beijing QuantiHealth Technology Co., Ltd, 100070 Beijing, China

**Keywords:** Microbiome, Next-generation sequencing, Health care

## Abstract

The gut microbes play important roles in human longevity and the gut microbiota profile of centenarians shows some unique features from young adults. Nowadays, most microbial studies on longevity are commonly based on metagenomic sequencing which may lose information about the functional microbes with extremely low abundance. Here, we combined in-depth metagenomic sequencing and large-scale culturomics to reveal the unique gut microbial structure of a Chinese longevity population, and to explore the possible relationship between intestinal microbes and longevity. Twenty-five healthy Hainan natives were enrolled in the study, including 12 centenarians and 13 senior neighbors. An average of 51.1 Gb raw sequencing data were obtained from individual fecal sample. We assembled 1778 non-redundant metagenomic assembled genomes (MAGs), 33.46% of which cannot be classified into known species. Comparison with the ordinary people in Hainan province, the longevous cohort displayed significantly decreased abundance of butyrate-producing bacteria and largely increased proportion of *Escherichia coli*, *Desulfovibrio piger* and *Methanobrevibacter smithii*. These species showed a constant change with aging. We also isolated 8,030 strains from these samples by large-scale culturomics, most of which belonged to 203 known species as identified by MALDI-TOF. Surprisingly, only 42.17% of the isolated species were also detected by metagenomics, indicating obvious complementarity between these two approaches. Combination of two complement methods, in-depth metagenomic sequencing and culturomics, provides deeper insights into the longevity-related gut microbiota. The uniquely enriched gut microbes in Hainan extreme decades population may help to promote health and longevity.

## Introduction

Nowadays, the world is facing an increasingly serious aging problem. How to extend our health and lifespan is becoming a focus issue. Extreme effort has been paid to explore the underlying mechanisms of aging and attempts to delay its progress by identifying key factors that regulate it^1^. Multiple pathways are implicated in aging, in which genetics is considered to account for 25–30% while environmental factors contribute to other 70–75%^2^. Among various environmental factors, gut microbiota is tightly linked to human health and longevity^3^. Experiments on various animal models showed that gut microbiota plays important roles in the regulation of host lifespan^4–8^. Clinical investigations also reveal that the gut microbial signature of centenarians is distinct from that of common elders^9–13^. Thus, a detailed depicting the gut microbial community in extremely aged and healthy centenarians and identifying key taxa that contribute to the longevity may provide novel strategies to achieve healthy aging.

The diversity and composition of the gut microbiota show a nonlinear relationship with age. The gut microbial communities of the elderly and the young are highly similar, while the gut microbiota of the centenarians displays some differences. In the centenarians, the abundance of *Roseburia* and *Escherichia* was significantly higher, while *Lactobacillus*, *Faecalibacterium*, *Parabacteroides*, *Butyricimonas*, *Coprococcus*, *Megamonas*, *Mitsuokella*, *Sutterella*, and *Akkermansia* were significantly lower in centenarians than the non-centenarians at the genus level^1,10–12^. Currently, most metagenomic studies on longevity were based on 16S rRNA gene amplicon sequencing^12^, which cannot provide functional information. Wu et al.^13^ investigated the gut microbiota structure of centenarians living at Sardinia, Italy using shotgun metagenomics method. Their results showed that the abundance of *Faecalibacterium prausnitzii* and *Eubacterium rectal* decreased, while *Methanobrevibacter smithii* and *Bifidobacterium adolescent is* markedly enriched in centenarians. Functional analysis showed that the centenarians had higher metabolic capacity, especially glycolysis and short-chain fatty acids (SCFAs) fermentation, while the genes encoding carbohydrate degrading enzymes (including fiber and galactose) were low^13^. The changes of gut microbiota may be a result or a contributing factor of aging. Centenarians have reached the limit of their life by adapting, adjusting and resisting the external biological and abiotic challenges. They can maintain intestinal homeostasis through a variety of ways to achieve people’s longevity. However, it is still necessary to further explore the relationship between the gut microbiota and aging.

Although numerous investigations have been performed to explore the relationship between the gut microbiota and longevity, metagenomics is the only technique in most studies used to depict the gut microbial composition. However, the DNA sequencing-based methods have several inherent drawbacks, such as inaccurate assembly results due to DNA process deviation, depth bias, incomplete genomic databases, and inability to detect, in some cases, the causative bacteria with low abundance^14^.Importantly, metagenomic analysis does not provide live microbes that can be further strain characterized or functionally assessed^14^. These disadvantages of metagenomics make the factual intestinal microbiota of the healthy centenarians far from been fully understood. Recent advances in culturomics have realized us that all microbes are culturable using right cultivating conditions^15^, which presents an effective complement to metagenomic sequencing for the comprehensive characterization of the gut microbial composition. Nevertheless, culturomics has been considered as an important approach to describe the gut microbiota^16^. Currently, culturomics and metagenomics exhibit high complementarity as only 15% of the detected species were concurrent for these two techniques^17,18^. However, these results were obtained by normal metagenomic and small-scale culturomics, a combination of ultra-depth metagenomics and large-scale culturomics is still needed to certificate this conclusion.

In this study, we enrolled 25 elderly people from Hainan province, South China, including 12 centenarians, 4 direct descendants of the centenarians, and 9 extremely decades neighbors. Their fecal samples were intensively analyzed by both ultra-depth metagenomic sequencing (51.1 Gb per sample) and large-scale culturomic analysis (321.2 strains per sample). A total of 897 known bacterial species were found by either ultra-depth metagenomics or large-scale culturomics, in which 42.17% of the species were concurrent for these two methods, revealing satisfactory complementarity. We compared the difference between the centenarians and non-centenarians in this Hainan population, and we also compared the microbial composition of this longevous population with that of the ordinary people living in Hainan province. Our results showed that some gut bacterial species changed consistently with aging, implying that they might play a role in maintaining health and longevity.

## Results

### Subject characteristics

A total of 25 elderly Hainan natives, South China, were enrolled in this study which included 12 centenarians, 3 direct descendants of the centenarians, and 10 extremely decades neighbors. All the individuals had an average age of 91.72 years, 14 were male and 11 were female (Table 1). These people were healthy and had no clear disease based on a multidisciplinary health assessment. All subjects had an omnivorous diet and did not consume antibiotics within 3 months before the study. 24 out of the 25 subjects do not drink in the daily life. Generally, there is no significant difference in the common features between the centenarians and the non-centenarians groups except age.

**Table 1:** Demographics of the Hainan longevity cohort.

Characteristics	Centenarians	Non-centenarians	*P* value
Male	8 (66.67%)	6 (46.15%)	0.30
Female	4 (33.33%)	7 (53.85%)	
Age (years)	103.75 ± 1.91	80.62 ± 13.49	<0.0001
BMI	17.96 ± 2.47	21.12 ± 3.07	0.015
Drinking	1/12	0/13	1.00
Dietary habit	Omnivorous	Omnivorous	–
Hypertension	0/12	0/13	1.00
Diabetes mellitus	0/12	0/13	1.00
Coronary heart disease	0/12	0/13	1.00
Autoimmune disease	0/12	0/13	1.00
Kidney disease	0/12	0/13	1.00
Cerebrovascular disease	0/12	0/13	1.00
Cancer	0/12	0/13	1.00

### Metagenomics/sequencing and metagenome-assembled genomes (MAGs)

Ultra-depth metagenomic analysis was performed to describe the gut microbial composition of the Hainan longevity cohort. A total of 1277.7 Gb raw sequence data was obtained with an average sequencing depth of 51.108 Gb per sample. The reads number of individual samples ranged from 242,836,682 to 468,165,554. MetaWRAP pipeline method was adopted to reconstruct the metagenome of the Hainan longevity population, and 2421 metagenome-assembled genomes (MAGs) were obtained after strict quality control (integrity and contamination detection). We assembled these MAGs based on 99% ANI threshold, and acquired 1,159 medium-quality MAGs and 1,262 high-quality MAGs according to Bowers criteria (Additional file 1: Fig. S1; Additional file 2: Table S2).

**Fig. 1:**
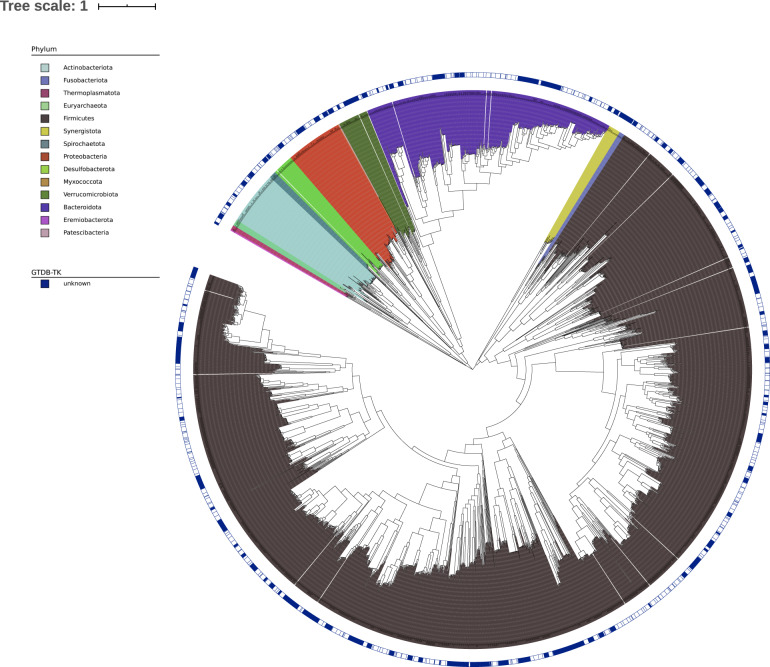
Phylogenetic tree of the gut microbial genome in Hainan longevity cohort. The phylogenetic tree was constructed using 2,421 MAGs. The MAGs were cataloged using GTDB-TK and indicated by different colors. The peripheral blue circle indicates MAGs that cannot be recognized by GTDBTK.

The 2421 MAGs were aligned in GTDBTk database for taxon classification. A total of 717 species, belonging to 371 genera, 99 families, 43 orders, 22 classes, and 14 phyla, were identified by clustering genomic boxes spanning 5% genetic diversity after all-to-all genetic distance quantification. Among the 14 phyla, 12 belonged to bacteria and 2 belonged to archaea. Firmicutes (68.17%, *n* = 1630), Bacteroidetes (19.53%, *n* = 467), Proteobacteria (3.97%, *n* = 95), Actinobacteria (2.76%, *n* = 66), and Verrucomicrobia (2.17%, *n* = 52) were the top 5 bacterial phyla that contained most assembled MAGs, while the 30 assembled archaeal MAGs belonged to Euryarchaeotta (*n* = 23) and Thermoplasmatota (*n* = 7) (Additional file 2: Table S3). We further constructed a phylogenetic tree using the MAG-predicted common core genes. The core gene-derived clustering pattern was highly consistent with the taxonomy assignments, with Firmicutes and Bacteroidales as the two most dominant clusters (Fig. 1). Additionally, 734 MAGs could not be classified at the species level with the currently available reference genome arrays, indicating that a significant proportion of MAGs may be new species. We used average nucleotide identity (ANI) as calculated by FastANI to annotate the taxonomy of MAGs. The MAGs with ANI > 95% are considered as the same species with the closest reference genome, otherwise it will be deemed as a novel species within the genus. These unclassified MAGs contained 339 potential new species (Additional file 2: Table S3).

### The enriched MAGs in centenarians compared to non-centenarians

**Fig. 2:**
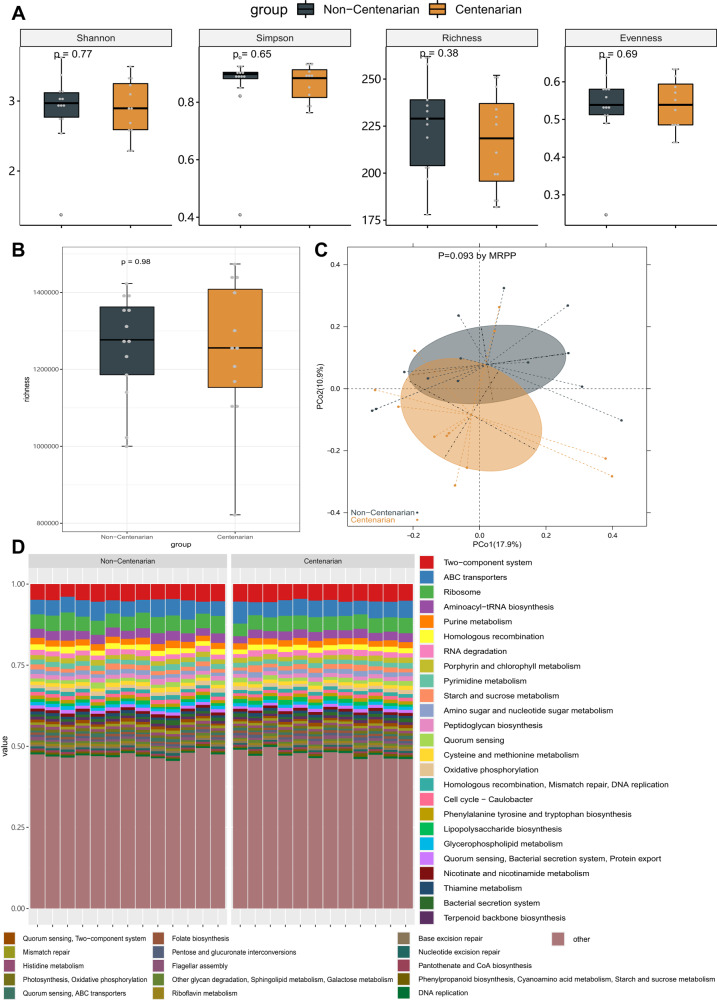
There is no significant difference in the gut microbiota composition between centenarians and non-centenarians. **A** Alpha diversity. **B** Gene richness. **C** Principal coordinate analysis (PCoA) analysis based on the species-level microbial community. The significance was calculated by multi-response permutation procedure (MRPP) analysis. **D** Functional gene profile. There is no significant difference between the two groups.

Species diversity analysis was conducted according to the assembled MAGs. There are no significances of alpha diversity and gene richness between centenarians and non-centenarians (Fig. 2A, B). The principal coordinate analysis (PCoA) of the Bray-Curtis distance based on the species-level microbial community was used to identify the similarities in the intestinal microbial structures of the two groups. Their intestinal microbial community structures and the functional gene profile were both similar between the centenarians and the non-centenarians (Fig. 2C, D).

**Fig. 3:**
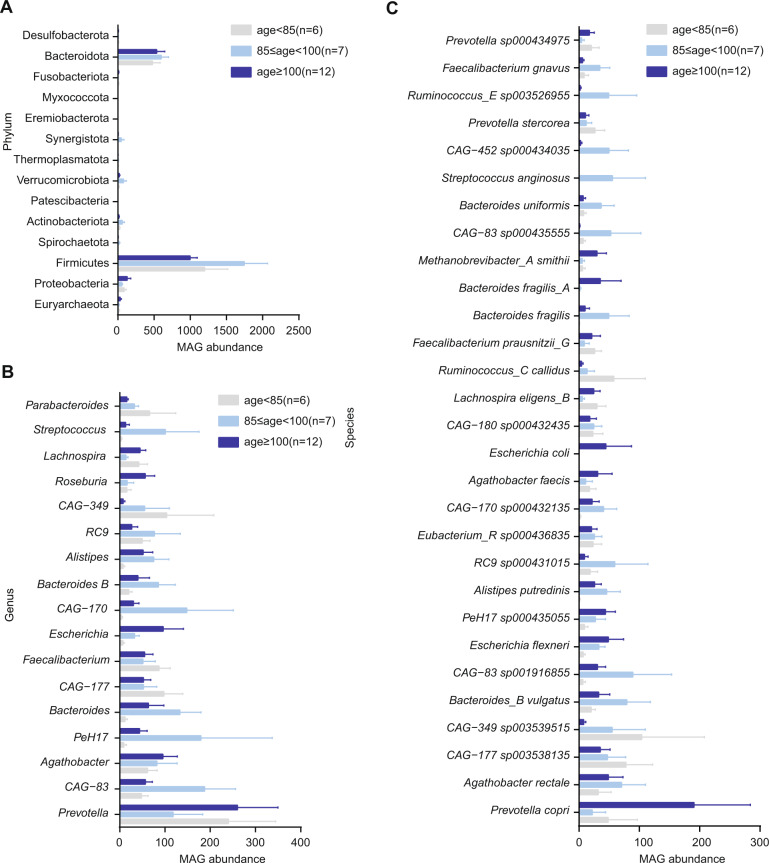
MAGs abundances at phylum, genus and species levels in different age groups. MAGs abundance at the phylum (**A**), genus (**B**) and species (**C**) levels in subjects with ages of <85 (group1), 85–100 (groups), and ≥100 (group3) years. The abundance of MAGs was calculated by metaWRAP. All taxa showed no significance among groups.

To find the key taxa that consistently change with aging, we further divided the Hainan longevity subjects into three subgroups, that were, Group1 contained 6 subjects whose ages below 85 years, Group2 contained 7 subjects whose ages between 85 and 100 years, and Group3 contained 12 centenarians. We compared the abundance of the 2,421 MAGs among the three subgroups. At the phylum level, the people with ages of 85–100 years presented the highest abundance of Firmicutes, Actinobacteria, Bacteroidota, Synergistota, and Verrucomicrobiota, while the people with ages < 85 or >100 revealed a lower abundance of these phyla. Notably, the centenarians contained higher abundance of Proteobacteria and Euryarchaeota (archaebacteria) than the other two groups (Fig. 3A). At the genus level, the abundance of *Agathobacter*, *Escherichia*, and *Roseburia* constantly increased with the ages (Fig. 3B), which mainly attributed to the enrichment of *Agathobacter faecis*, *Agathobacter rectale*, *Escherichia coli*, and *Escherichia flexneri* (Fig. 3C). Additionally, four species *Methanobrevibacter smithii* (archaebacteria), *E. coli*, *Prevotella copri*, and *Bacteroides fragilis* were highly enriched in centenarians (Fig. 3C), suggesting that the enrichment of these species is associated with extremely long life.

### The main taxa that are significantly different between the longevous and ordinary population in Hainan province

**Fig. 4:**
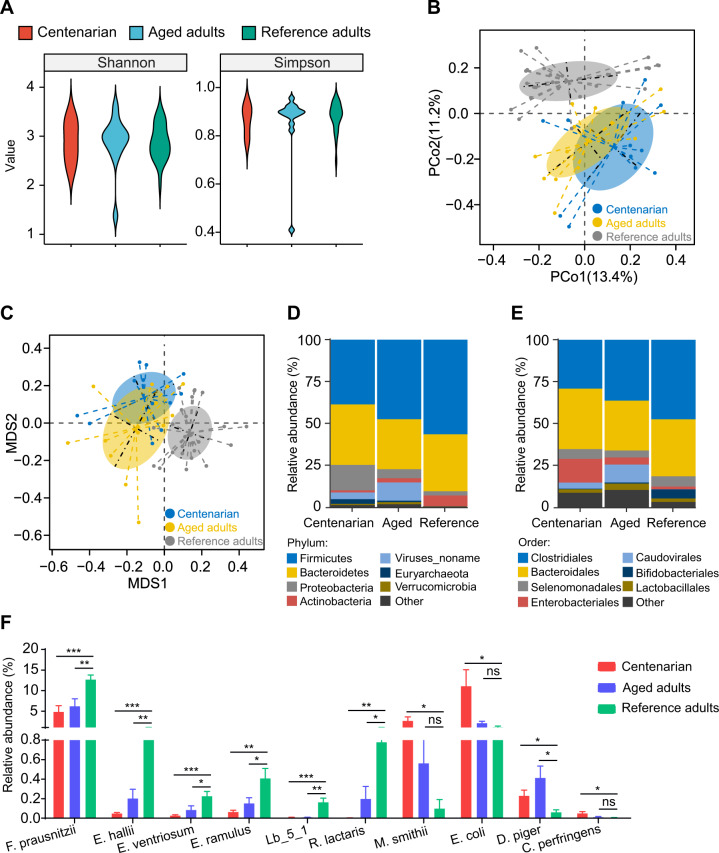
There is significant difference in the gut microbiota between the longevous cohort and the ordinary adults in Hainan China. **A** Alpha diversity. **B** Principal coordinate analysis (PCoA) analysis. **C** Non-metric multidimensional scaling (NMDS) analysis. **D**, **E** The microbial profile at phylum (**D**) and order (**E**) levels. **F** The main species that changes constantly with aging. Lb_5_1 refers to Lachnospiraceae_bacterium_5_1. *P < 0.05, **P < 0.01, ***P < 0.001.

**Fig. 5:**
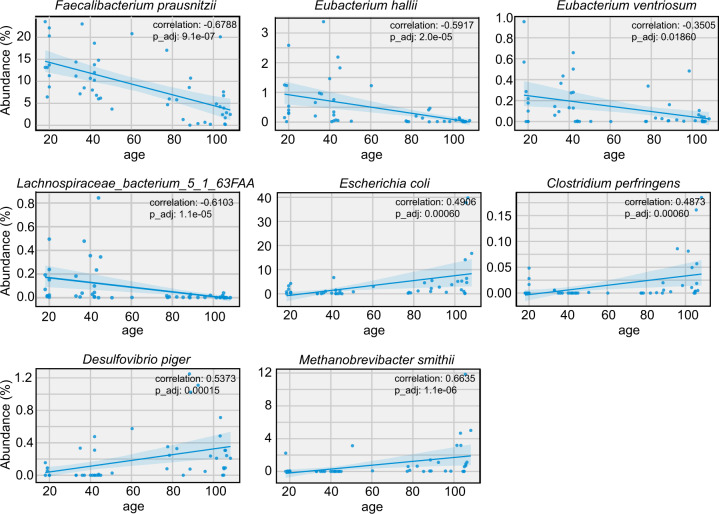
Regression analysis between the abundance of individual species and the age. Non-parameteric spearman correlation analysis was performed to monitor the correlation of individual species and age.

We then compared the metagenomics data of 1778 MAGs in this longevity cohorts with that of the reference people living in Hainan province^19,20^ to find the main taxa that are significantly changed with aging. The centenarians (*n* = 12) and aged adults (*n* = 13) were from our longevity cohort, while the reference adults (*n* = 24, 18–45 years old) were from published papers^21,22^. There is no significant difference in the alpha diversity of gut microbiota among groups (Fig. 4A). PCoA and non-metric multidimensional scaling (NMDS) analysis revealed that the gut microbial structures constantly shifted along aging (Fig. 4B, C). The microbial composition at the phylum level showed that Proteobacteria largely increased while Actinobacteria continually decreased with ages (Fig. 4D), which mainly attributed to the increase of Enterobacteriales and decrease in Bifidobacteriales (Fig. 4E). Inspection of individual gut bacterial species exhibited that the butyrate-producing bacteria such as *Faecalibacterium prausnitzii*, *Eubacterium hallii*, *E. ventriosum*, *E. ramulus*, *Lachnospiraceae_bacterium_5_1* and *Ruminococcus lactaris* were significantly decreased in the aged and centenarian groups, while opportunistic pathogens such as *Escherichia coli*, *Desulfovibrio piger* and *Clostridium perfringens* were increased in the longevity groups (Fig. 4F). Notably, the methanogen *Methanobrevibacter smithii* was enormously presented in the longevity cohort. Although the #38 volunteer is only 50 years old, he possesses 2.23% *M. smithii* in his gut microbial community which is far more than the average abundance (0.098%) of this species in the ordinary population in Hainan province. Regression analysis of the species’ abundance with age revealed that the butyrate-producing species such as *Faecalibacterium prausnitzii*, *Eubacterium hallii*, *Eubacterium ventriosum* and *Lachnospiraceae_bacterium_5_1_63FAA* were negatively related to aging while opportunistic pathogens such as *Escherichia coli*, *Clostridium perfringens*, *Desulfovibrio piger* and methanogen *M. smithii* were significantly positive to age (*q* < 0.05 by Benjamini-Hochberg test) (Fig. 5).

### The sequencing depth is saturated at 50 Gb per sample

**Fig. 6:**
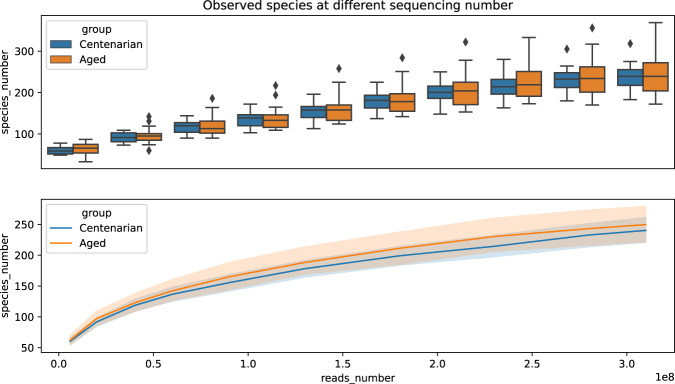
Rarefaction curve as indicated by Shannon or Simpson index. The upper chart shows the observed species at different sequencing numbers in a box plot format and the lower chart indicates the same thing and plots the data in a line regression format. The number of observed species increases rapidly until the sequencing reads becomes 5e7 and after that, the number of observed species still grows.

To verify whether the 50 Gb-depth sequencing is sufficient for a thorough metagenomic analysis, Rarefaction analysis was performed. The Rarefaction curve became flat when sequencing depth beyond 3 × 10^8^ reads (about 40 Gb)/sample (Fig. 6), suggesting that the sequencing depth is nearly saturated and very few new phylotypes can be obtained by additional sequencing. Therefore, new methology should be taken to acquire more compositional bacteria from the fecal materials.

### The gut microbial composition assessed by culturomics

**Fig. 7:**
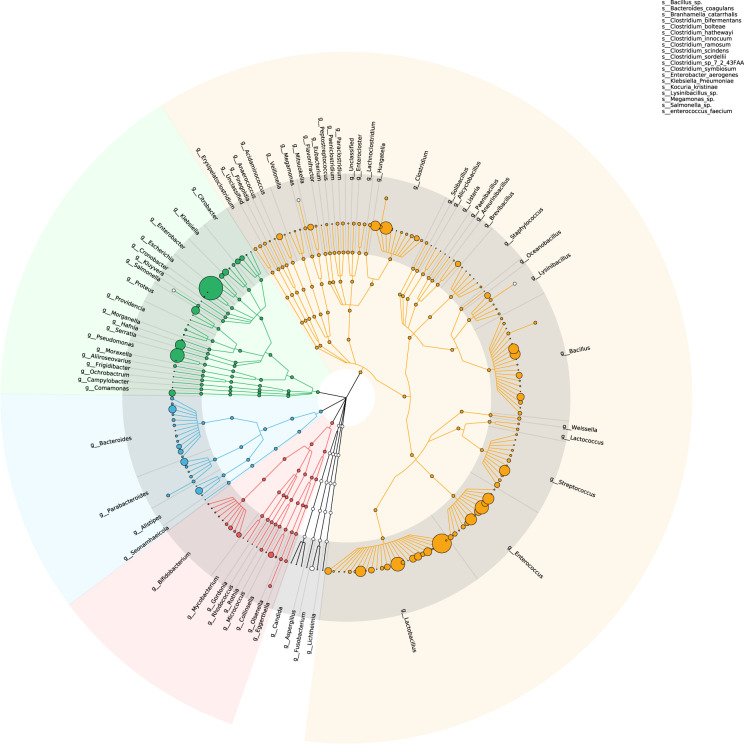
Phylogenetic tree based on the gut bacteria obtained by culturomics. Orange, black, red, blue, and green branches represent Firmicutes, Fusobacteriota, Actinobacteria, Bacteroides, and Proteobacteria, respectively. The area of the circles stands for the number of isolated strains belonging to each species.

We further performed culturomics to gain comprehensive insights into the gut microbiota composition of longevity. To thoroughly isolate the compositional gut bacteria from the fecal samples of Hainan longevity population, 23 types of culture media and 98 pretreatment conditions were utilized, which ultimately provided 8165 bacterial strains. Identification by MALDI-TOF MS showed that 8123 strains belonged to 235 known gut bacterial species and other 42 strains were undefined species (Additional file 2: Table S4). Generally, 6979 strains of 256 species were retracted under anaerobic conditions which contained 41 undefined species, and 1186 strains of 89 species were obtained under microaerobic conditions which contained 1 undefined species (Additional file 2: Table S5). The isolated species belonged to 7 phyla, including Firmicutes (134 species), Proteobacteria (38 species), Bacteroidetes (27 species), Actinobacteria (19 species), Fusobacteria (2 species), Ascomycota (1 species), and Agaricomycotina (1 species). A total of 69 genera were identified in which *Lactobacillus*, *Bacillus*, *Enterococcus*, *Streptococcus* and *Clostridium* were mostly presented (Fig. 7, Additional file 2: Table S5).

The average isolated strain number (322.92 vs. 317.77, *p* = 0.959) and species number (48.00 vs. 53.62, *p* = 0.592) per subject were similar between the centenarians and non-centenarians (Additional file 2: Table S6). However, the average number of *Lactobacillus johnsonii* (3.08 vs. 0.08), *L. kalixensis* (4.58 vs. 0.15), *L. oris* (2.75 vs. 0.23), *L. agilis* (5.67 vs. 0.62), *L. gasseri* (4.42 vs. 0.54) and *Enterococcus dispar* (15.17 vs. 1.92) were much higher in centenarians, while *Brevibacillus borstelensis* (0.00 vs. 4.08), *Mitsuokella multacida* (0.33 vs. 4.62), *Enterococcus asini* (0.25 vs. 3.00), *Bifidobacterium pseudolongum* (0.25 vs. 2.54) and *L. reuteri* (1.33 vs. 8.23) were higher in non-centenarians (Additional file 2: Table S7). In general, centenarians presented higher amount of culturable *Lactobacillus* and *Enterococcus*, which was in accordance with the metagenomic analysis (Fig. 4E).

### Culturomics is an important complement for metagenomics to gain a thorough insight of gut microbiota

**Fig. 8:**
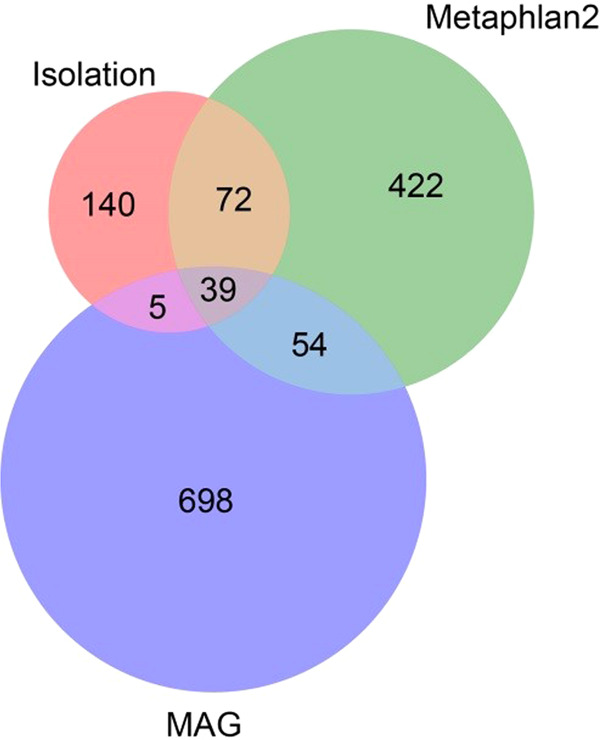
Venndiagram on species identified by metagenomics (metaphlan2 and MAG algorithms) or culturomics.

In this study, a total of 1430 species were identified using culture-independent (metagenomics) or culture-dependent (culturomics) approaches. Specifically, metagenomic studies identified 1290 species among which 587 and 796 species were identified by metaphlan2- and MAG-based methods respectively, while 256 species were identified by culturomics. Only 116 species were identified by both metagenomics and culturomics (Fig. 8). A total of 140 species were solely identified by culturomics (Fig. 8), suggesting that culturomics is an important complement for metagenomics to gain a thorough insight of gut microbiota.

**Fig. 9:**
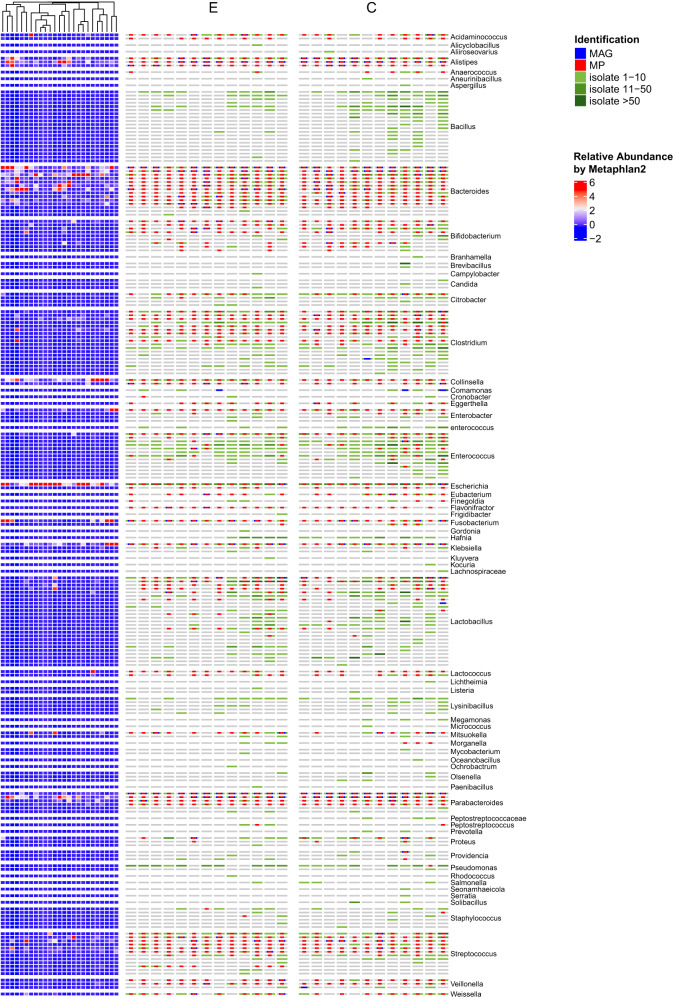
Overview of the isolated strains by culturomics. The right part displayed the isolated strain number of each species in the feces donated by Hainan centenarians (**C**) and non-centenarian elders (**E**). The left part indicates the relative abundance (%) of each species in fecal samples by metagenomic analysis.

To qualify the species we obtained, we matched species information, sample information, and genomic sequence data, after which the metagenome abundance of each strain in metagenome sequencing was mapped. The 144 cultured species were unable to obtain the species abundance information, accounting for 69.90% of the cultural species. Some genera, such as *Bacillus*, *Lysinibacillus*, *Hafnia*, *Pseudomonas*, and *Salmonella*, could not be detected by metagenomics but were frequently isolated by culturomics (Fig. 9), which may be due to their low abundance or difficulty to extract DNA material.

## Discussion

Hainan Island is an ideal area for studying longevity because of its high incidence of centenarians, relatively homogeneous population, lifestyle, and unique diet. Therefore, in-depth investigation on the gut microbiota of Hainan centenarians helps to deepen our understanding of the mechanism of longevity. Although the intestinal microflora is considered to be an important determinant of human health^23^, the structure of intestinal microbiota and the mechanism influencing longevity in long-lived people have not been fully understood. Besides, how to thoroughly profile the composition of the gut microbiota remains a big challenge. The main goal of this study is to explore the intestinal microecological features of the gut microbiota in long-lived people in Hainan through culturomics and in-depth metagenomics. This is, to our best knowledge, the first study to combine the deep metagenomic sequencing of fecal microorganisms with large-scale culturomics to thoroughly depict the metagenomic features of centenarians.

Several studies have reported that the gut microbial structure and function of centenarians exhibit unique features as compared to ordinary adults^1,10–13,24^. Centenarians are commonly featured by decreased alpha diversity, reduced butyrate-producing bacteria such as *Faecalibacterium*, *Roseburia*, *Eubacterium* and *Ruminococcus*, and increased opportunistic pathobionts^25^. It has been proposed that the decreased ratio of *Faecalibacterium* and *Prevotella* and a higher abundance of *Escherichia*, *Akkermansia*, *Christensenellaceae*, and *Lactobacillus* in the centenarians may be beneficial from an immunological and metabolic viewpoint^26^. In our study, the Hainan centenarians displayed a distinct gut microbial community compared with the ordinary adults living in the same region. The alpha diversity of their gut microbiota showed no difference. Similar with the centenarians living in other regions, the Hainan centenarians displayed increased abundance of opportunistic pathogens such as *Escherichia coli*, *Desulfovibrio piger* and *Clostridium perfringens* and reduced butyrate-producing bacteria including *Faecalibacterium*, *Eubacterium* and *Ruminococcus*. We further compared the abundance of these bacteria among persons with ages across 18 to 106 years, in order to find the species whose abundance changes along aging tightly. All the above butyrate producers decreased with aging, especially *Faecalibacterium prausnitzii*. On the contrary, the abundance of opportunistic pathogens such as *E. coli*, *C. perfringens*, *D. piger* and methanogen *M. smithii* were increased with people getting old. Although the enrichment of *M. smithii* in centenarians has been reported previously^13^, our work provided evidence that the enrichment of this methanogen may occur at an earlier stage of longevity (<85 year-old), suggesting that *M. smithii* may play a role in longevity. Overall, our in-depth metagenomic analysis revealed that the Hainan longevity population displays a similar gut microbial feature with other longevities worldwide. The decrease in butyrate producers especially *F. prausnitzii* and *Eubacterium* spp. and the enrichment of *M. smithii* are accompanied with aging, which may play a role in longevity.

Since 2005, metagenomics has enabled large-scale investigations of complex microbiomes^27–29^. High-throughput sequencing is an ideal way to analyze the genomes of all the microbes in the sample, not just those that are easier to cultivate. However, metagenomics sequencing prefers to detect the dominant species, it is difficult to find the vast majority of microorganisms with extremely low abundance. In this study, an ultra-deep metagenomic sequencing was adopted for mining the intestinal microbial gene fragments of the longevities. However, even the sequencing depth exceeds 50 Gb, the number of new MAGs assemblies showed little increase. The result indicates even with ultra-sequencing depth, completing a complete collection of gut microbes would be difficult and result in unaffordable costs. In addition, the deviation and chimera formation in the amplification process may lead to the deviation of the assembly result from the reality, the unspecific extraction of the DNA of the species of interest, the acquired microbial viability cannot be confirmed, and other problems are inevitable. Thus, more measures such as isolation, purification and culture of intestinal microorganisms may be a profound method for discovery of microorganisms with an extremely low abundance.

For comprehensive profiling of the gut microbial structure, microorganisms were enriched immediately in an anaerobic environment after collection and cultured on 23 types of culture media under 98 pretreatment conditions. Our culturomic method enabled analysis of intestinal microbes with an amount >10^2^ per gram of feces, and retrieved 8,165 bacterial strains belonging to 256 species. A total of 116 gut microbial species were found by both culturomics and metagenomics methods, which accounted for 45.31% of cultured species. More than half (140 species) of the retrieved species were solely identified by culturomics and were not detected by the ultra-deep metagenomic sequencing (Fig. 7), suggesting that culturomics is an important complement for metagenomics to gain a thorough insight of gut microbiota. Although the retrieved strain number of gut bacterial species by culturomics could not reflect the actual abundance of each species, the higher availability by culturomics still indicated a relative higher abundance of the species in feces to a certain extent. Our results revealed that the Hainan centenarians presented higher number of cultured strains of *Lactobacillus* and *Enterococcus* spp., suggesting that these two taxa may be more abundance in Hainan centenarians. These findings were in accordance with our metagenomic data as well as previous reports^26^. Therefore, culturomics contributes to additional understanding of the composition of gut microbiota and highlights microbial dark matter. Combined culturomic and metagenomic data can gain a thorough insight into the construction of intestinal bacterial communities.

## Conclusions

By using culturomics and in-depth sequencing of metagenomics, which complement each other, we first showed the characteristic structure of the gut microbiota of health longevity population in Hainan, as well as its unique enriched intestinal microorganisms. Our research is a supplementary for gut microbiome structure of centenarians and longevity.

## Methods

### Subjects preparation and Sample Collection

Twenty-five Chinese people from Hainan province, South China were enrolled in the study, which included twelve centenarians, three offspring of the centenarians and ten longevity neighbors. All the volunteers have no disease by multidisciplinary health assessment. The subjects did not consume antibiotics within three months before the study. In order to verify the data and collect more detailed information, a questionnaire survey was conducted among the population before the sample was collected to obtain information about age and eating habits from the identified subjects. The subjects have all signed a written consent and the project has been approved by the Hainan Branch of the General Hospital of the People’s Liberation Army (PLAGH)’s ethics committee under number 301hn11-2017-03.

Trial registration: ChiCTR2100041983. Registered 10 Jan 2021 - Retrospectively registered, http://www.chictr.org.cn/showproj.aspx?proj=119862.

Fresh feces were collected from each subject on the day of examination by independent defecation or via digital rectal examination, as previously characterized by Luan et al.^30^. A small amount of feces was collected into a sterile anaerobic sealed test tube and stored in a 37 ± 2 °C incubator until inoculated and cultured. At the same time, each sample of fresh feces (5 g) was collected in an OMNIgene-GUT tube (DNA Genotek, Ontario, Canada) and a sterile stool collection container and transported using dry ice. The fecal samples were immediately used for bacterial isolation, and those used for metagenomic sequencing were stored at −80 °C until use.

### Bacterial isolation, purification, culturing and identification

Bacterial culturing was performed using a variety of medium with or without ethanol pretreatment. Briefly, sample processing and culturing took place under anaerobic conditions in a Whitley DG250 workstation at 37 °C using phosphate-buffered saline and culture media incubated under anaerobic conditions for 24 h before use. A total of 23 kinds of culture medium and 36 kinds of culture combinations (Additional file 2: Table S1) were applied to study 29 fecal samples for 42 days. Colonies were picked, re-streaked to purity, and identified using MALDI-TOF mass spectrometry.

### Deep metagenomic sequencing

Microbial DNA was extracted using the QIAamp DNA stool mini kit (QIAGEN, Hilden, Germany) with additional bead-beating and heating steps. The rest of fecal samples were stored at −80 °C for further use. DNA library was constructed using the KAPA HyperPlus Library Preparation kit (KAPA Biosystems) then quantified by KAPA Library Quantification Kits (KAPA Biosystems) according to the manufacturer’s instructions. The insert size of the library was approximately 350 bp, and the library was sequenced by shotgun method to acquire paired-end reads with 150 bp in the forward and afterward directions.

### Bioinformatic analysis

#### Taxonomy and gene functional pathway profiling

The raw data were removed low quality reads using MOCAT2, and sequencing adapters were removed by Cutadapt (version v1.14,-m 30). The reads with quality of less than 20 or the length of less than 30 bp were removed by SolexaQA package. The filtered reads aligned with the human genome(H. sapiens, UCSC hg19) using SOAPaligner (v2.21, -M 4 -l 30 -v 10) to obtain high-quality clean data. The relative abundance of each taxon was calculated by MetaPhlan2 based on default parameters. The relative abundance of gene families and functional pathway were calculated by HUMAnN2. The KO abundance in KEGG catalogs was determined by regroup method in HUMAnN2 package based on the relative abundance of gene families.

#### Metagenomic assembly, binning, and quantification

A metadraw procedure was performed to assemble and binning the high-quality reads. High-quality sequencing data were assembled using metaSPAdes. The assembly results were binned by three tools, which were MaxBin2, metaBAT2 and CONCOCT. The binning results were further processed by a refinement module to obtain the final bin set. The quality of these bins meets the threshold setting of Medium-Quality Draft Metagenome-Assembled Genomes (completeness > 50% and compatibility < 5%). Finally, the relative abundance of bins in each sample was calculated by the quant_bins module, which can avoid sparse read assignment. Afterwards, the bins were de-duplicated by dRep to acquire the representative genomes. Finally, the genomes were classified into different species using GTDBTk, and phylogenetic tree was constructed using Phylophlan.

### Statistical analysis

Data are indicated by the means ± sem. R software (version 3.5.1) was used for the statistical analysis to determine the statistical differences among the groups using parametric methods (ANOVA) and nonparametric statistical methods (Wilcox nonparametric test). For multiple tests, adjusted *p* values were calculated by Benjamini–Hochberg’s correction. Alpha diversity was calculated by vegan package. Principle coordinate Analysis (PCoA) of Bray–Curtis distance was performed by phyloseq and plotted using ggplot2. A *p* < 0.05 was considered as significance.

### Reporting summary

Further information on research design is available in the Nature Research Reporting Summary linked to this article.

### Supplementary information

Reporting Summary

supplementary figure S1

supplementary table

## Data Availability

The sequence data has been deposited in the NCBI Sequence Read Archive (SRA) database (BIOProject: PRJNA772518). All data relevant to the study are included in the article or uploaded as supplementary information.
