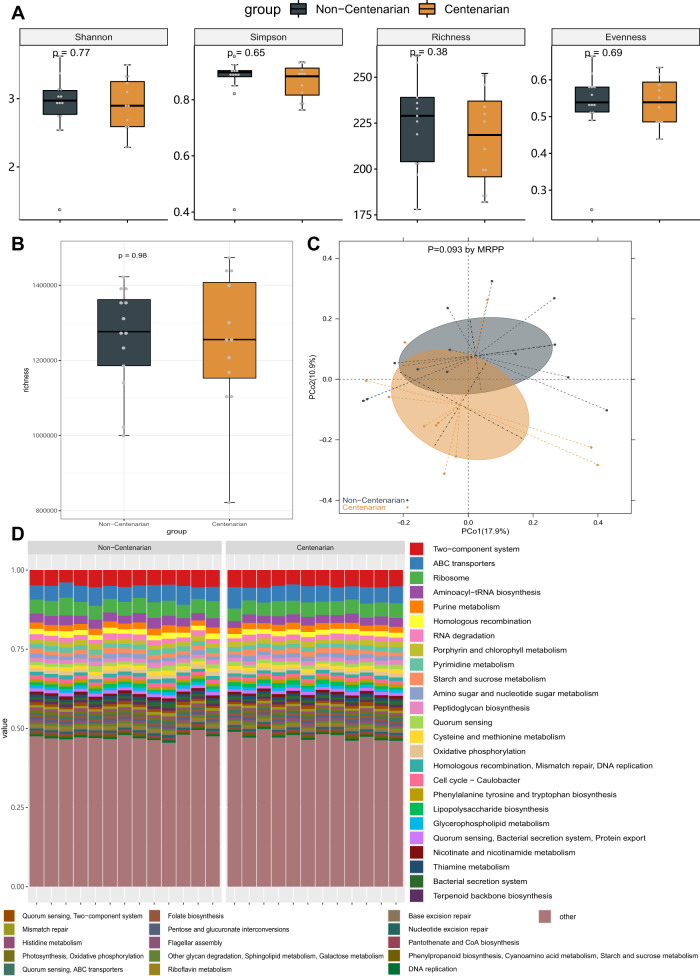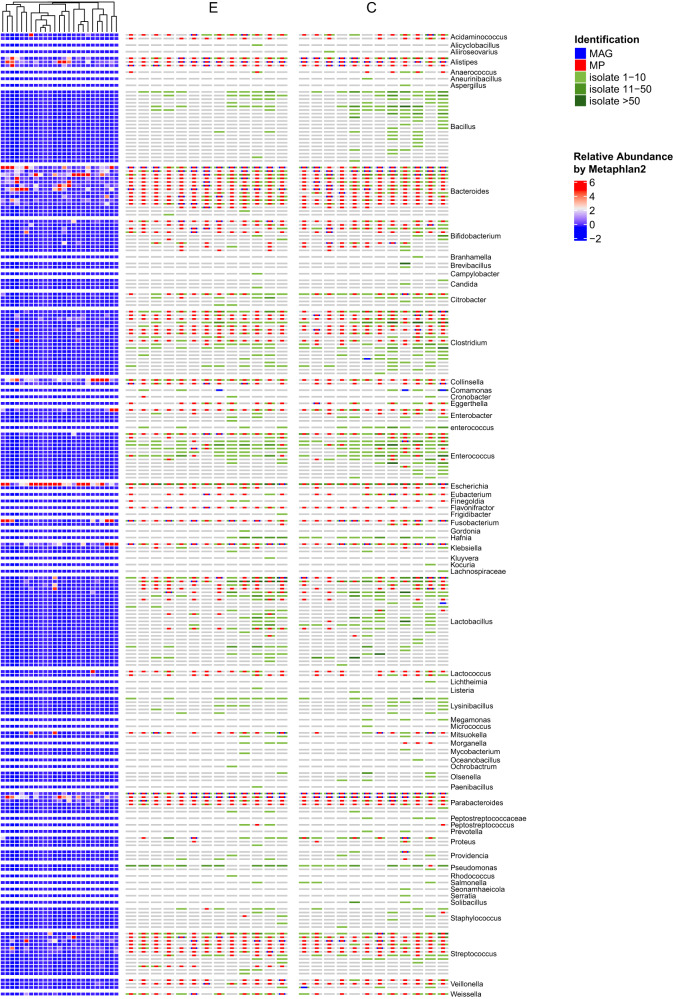# Author Correction: Deep insights into the gut microbial community of extreme longevity in south Chinese centenarians by ultra-deep metagenomics and large-scale culturomics

**DOI:** 10.1038/s41522-023-00456-7

**Published:** 2023-11-28

**Authors:** Congyong Li, Zhe Luan, Yiming Zhao, Jun Chen, Yanan Yang, Cong Wang, Yujia Jing, Shirui Qi, Zhuanyu Li, Hao Guo, Wenyi Xu, Bowen Zhao, Chongming Wu, Shufang Wang, Yunsheng Yang, Gang Sun

**Affiliations:** 1 Sixth Health Care Department, Second Medical Center of PLA General Hospital, 100853 Beijing, China; 2 Department of Gastroenterology and Hepatology, First Medical Center of PLA General Hospital, 100853 Beijing, China; 3 Department of Gastroenterology and Hepatology, Hainan Hospital of PLA General Hospital, 572013 Sanya, China; 4 Unit 91917, 102401 Beijing, China; 5 School of Chinese Materia Medica, Tianjin University of Traditional Chinese Medicine, 301617 Tianjin, China; 6 Emergency Department, Tianjin First Central Hospital, 300192 Tianjin, China; 7 Beijing QuantiHealth Technology Co., Ltd, 100070 Beijing, China

**Keywords:** Microbiome, Next-generation sequencing, Health care

Correction to: *npj Biofilms and Microbiomes* 10.1038/s41522-022-00282-3, published online 19 April 2022

In the original version of this article one female non-centenarian was mistakenly included in the female centenarian cohort. This error alters the numbers presented in the Article slightly, but the conclusions are unaffected. As a result of the error, Table 1, Figure 2 and Figure 9 have been corrected, as well as some minor edits in the text. Both the HTML and PDF versions of the article have been corrected.