# Supplementary material for: Deep insights into the gut microbial community of extreme longevity in south Chinese centenarians by ultra-deep metagenomics and large-scale culturomics
Source: NPJ Biofilms Microbiomes. 2022 Apr 19;8:28. doi: 10.1038/s41522-022-00282-3 (PMC9019030; doi:10.1038/s41522-022-00282-3)
Supplement: Supplementary file 1 — Reporting Summary [file 41522_2022_282_MOESM1_ESM.pdf]

nature portfolio

Corresponding author(s): Gang Sun, Chongming Wu, Yunsheng Yang, Shufang Wang

Last updated by author(s): Jan 28, 2022

Reporting Summary

Nature Portfolio wishes to improve the reproducibility of the work that we publish. This form provides structure for consistency and transparency in reporting. For further information on Nature Portfolio policies, see our Editorial Policies and the Editorial Policy Checklist.

Statistics

For all statistical analyses, confirm that the following items are present in the figure legend, table legend, main text, or Methods section.

n/a	Confirmed	
☐	☒	The exact sample size (n) for each experimental group/condition, given as a discrete number and unit of measurement
☐	☒	A statement on whether measurements were taken from distinct samples or whether the same sample was measured repeatedly
☐	☒	The statistical test(s) used AND whether they are one- or two-sided<br>*Only common tests should be described solely by name; describe more complex techniques in the Methods section.*
☒	☐	A description of all covariates tested
☐	☒	A description of any assumptions or corrections, such as tests of normality and adjustment for multiple comparisons
☐	☒	A full description of the statistical parameters including central tendency (e.g. means) or other basic estimates (e.g. regression coefficient) AND variation (e.g. standard deviation) or associated estimates of uncertainty (e.g. confidence intervals)
☐	☒	For null hypothesis testing, the test statistic (e.g. F, t, r) with confidence intervals, effect sizes, degrees of freedom and P value noted<br>*Give P values as exact values whenever suitable.*
☒	☐	For Bayesian analysis, information on the choice of priors and Markov chain Monte Carlo settings
☒	☐	For hierarchical and complex designs, identification of the appropriate level for tests and full reporting of outcomes
☒	☐	Estimates of effect sizes (e.g. Cohen's d, Pearson's r), indicating how they were calculated

Our web collection on statistics for biologists contains articles on many of the points above.

Software and code

Policy information about availability of computer code

Data collection	No software was used for data collection
Data analysis	Softwares used in this study include MaxBin2 metaBAT2 CONCOCT HUMAnN2 SOAPaligner MOCAT2 and MetaPhlAn2. See the manuscript for detailSoftwares used in this study include MaxBin2 metaBAT2 CONCOCT HUMAnN2 SOAPaligner MOCAT2 and MetaPhlAn2. See the manuscript for detail.

For manuscripts utilizing custom algorithms or software that are central to the research but not yet described in published literature, software must be made available to editors and reviewers. We strongly encourage code deposition in a community repository (e.g. GitHub). See the Nature Portfolio guidelines for submitting code & software for further information.

Data

Policy information about availability of data

All manuscripts must include a data availability statement. This statement should provide the following information, where applicable:

- Accession codes, unique identifiers, or web links for publicly available datasets
- A description of any restrictions on data availability
- For clinical datasets or third party data, please ensure that the statement adheres to our policy

All sequencing data are available from NCBI. (PRJNA772518)

Field-specific reporting

Please select the one below that is the best fit for your research. If you are not sure, read the appropriate sections before making your selection.

☒ Life sciences ☐ Behavioural & social sciences ☐ Ecological, evolutionary & environmental sciences

For a reference copy of the document with all sections, see nature.com/documents/nr-reporting-summary-flat.pdf

Life sciences study design

All studies must disclose on these points even when the disclosure is negative.

Sample size	In this study, all centenarians who met the inclusion criteria in two cities of Hainan province were selected, as well as their relatives and neighbors
Data exclusions	No data were excluded.
Replication	All attempts at replication were successful.
Randomization	The enriched MAGs in centenarians compared to non-centenarians. The main taxa that are significantly different between the longevous and ordinary population in Hainan province. The differences in gut microbes between long-lived and non-long-lived people have been documented in a number of studies.
Blinding	Metagenomic and culture group technical analysts are not clear about grouping information

Reporting for specific materials, systems and methods

We require information from authors about some types of materials, experimental systems and methods used in many studies. Here, indicate whether each material, system or method listed is relevant to your study. If you are not sure if a list item applies to your research, read the appropriate section before selecting a response.

Materials & experimental systems

n/a	Involved in the study	
☒	☐	Antibodies
☒	☐	Eukaryotic cell lines
☒	☐	Palaeontology and archaeology
☒	☐	Animals and other organisms
☐	☒	Human research participants
☐	☒	Clinical data
☒	☐	Dual use research of concern

Methods

n/a	Involved in the study	
☒	☐	ChIP-seq
☒	☐	Flow cytometry
☒	☐	MRI-based neuroimaging

Human research participants

Policy information about studies involving human research participants

Population characteristics	A total of 25 elderly Hainan natives, South China, were enrolled in this study which included 12 centenarians, 4 direct descendants of the centenarians, and 9 extremely decades neighbors. All the individuals had an average age of 91.72 years, 14 were male and 11 were female . These people were healthy and had no clear disease based on a multidisciplinary health assessment.
Recruitment	Thirteen centenarians, three offspring of the centenarians and nine longevity neighbors. The subjects have all signed a written consent.
Ethics oversight	Hainan Branch of the General Hospital of the People's Liberation Army (PLAGH)'s ethics committee under number 301hn11-2017-03

Note that full information on the approval of the study protocol must also be provided in the manuscript.

Clinical data

Policy information about clinical studies

All manuscripts should comply with the ICMJE guidelines for publication of clinical research and a completed CONSORT checklist must be included with all submissions.

Clinical trial registration	ChiCTR2100041983

Study protocol	http://www.chictr.org.cn/showproj.aspx?proj=119862
Data collection	Centenarians refer to those who were born before January 1, 1918 and have lived in Chengmai and Danzhou in Hainan for more than 20 years .Sample collection and data extraction began in September 2018.
Outcomes	Metagenomic and Culturomics analysis results. Culturomics and in-depth sequencing of metagenomics are complement each other.
