# Supplementary material for: Deep insights into the gut microbial community of extreme longevity in south Chinese centenarians by ultra-deep metagenomics and large-scale culturomics
Source: NPJ Biofilms Microbiomes. 2022 Apr 19;8:28. doi: 10.1038/s41522-022-00282-3 (PMC9019030; doi:10.1038/s41522-022-00282-3)
Supplement: Supplementary file 2 — supplementary figure S1 [file 41522_2022_282_MOESM2_ESM.docx]

**Supplementary data**


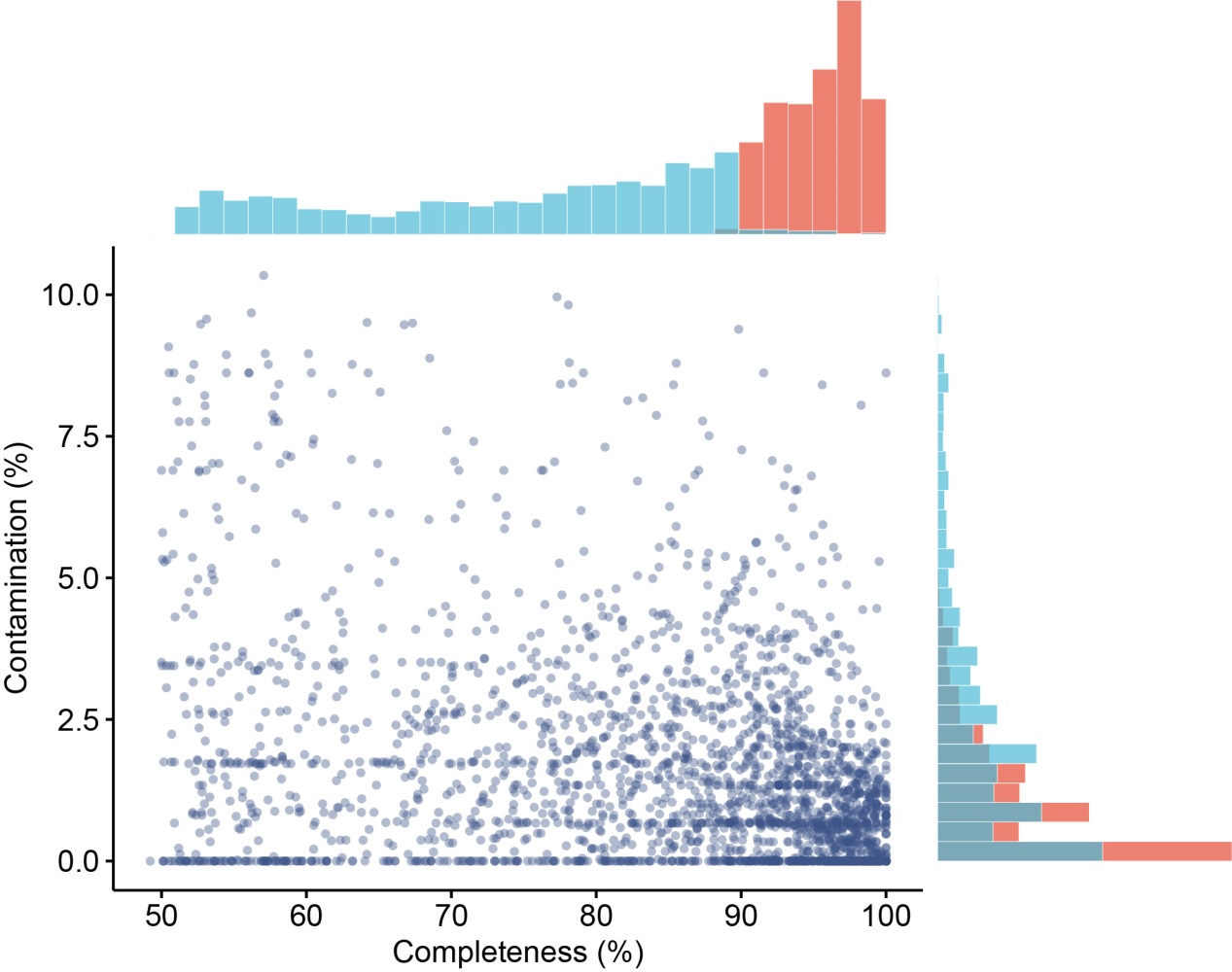


**Supplementary Figure S1. Estimated completeness and contamination of the 2421 MAGs obtained from the fecal microbiome of the Hainan longevity cohort.** The red, light and dark blue columns indicate high-, medium- and low-quality MAGs, respectively. The length of the column indicates the number at each position.
