# Supplementary material for: Deep insights into the gut microbial community of extreme longevity in south Chinese centenarians by ultra-deep metagenomics and large-scale culturomics
Source: NPJ Biofilms Microbiomes. 2022 Apr 19;8:28. doi: 10.1038/s41522-022-00282-3 (PMC9019030; doi:10.1038/s41522-022-00282-3)
Supplement: Supplementary file 3 — supplementary table [file 41522_2022_282_MOESM3_ESM.pdf]

Table S1. Culture medium and pretreatment conditions

No.	Medium
1	BA
2	BHI
3	CBA
4	CMM
5	CMPYG
6	CYCFA
7	DA
8	FA
9	GSYCFA
10	GYCFA
11	IYCFA
12	LGA
13	M17
14	MA
15	MGYCFA
16	MRS
17	PYG
18	RA
19	SA
20	SYCFA
21	TBA
22	TSB

No.	Code	Pretreatment
1	LCLDYZMA30D	storage under 4 dgree for 7 days , then under -20 dgree for 30 days, finally growth in seawater medium for 30 days
2	lcldyz25d	storage under 4 dgree for 7 days , then under -20 dgree for 30 days, finally growth in GCB + 5% blood + 10% rumen juice for 25 days
3	lcldyzma25d	storage under 4 dgree for 7 days , then growth in seawater medium for 25 days
4	LCYZO10D	storage under 4 dgree in BD standard for 10 days , then growth in F culture for 10 days
5	LCYZO13D	storage under 4 dgree in BD standard for 10 days , then growth in F culture for 13 days
6	LCYZO15d	storage under 4 dgree in BD standard for 10 days , then growth in F culture for 15 days
7	LCYZO16d	storage under 4 dgree in BD standard for 10 days , then growth in F culture for 16 days
8	LCYZO17d	storage under 4 dgree in BD standard for 10 days , then growth in F culture for 17 days
9	LCYZO18d	storage under 4 dgree in BD standard for 10 days , then growth in F culture for 18 days
10	LCYZO2D	storage under 4 dgree in BD standard for 10 days , then growth in F culture for 2 days
11	LCYZO3D	storage under 4 dgree in BD standard for 10 days , then growth in F culture for 3 days
12	LCYZO6D	storage under 4 dgree in BD standard for 10 days , then growth in F culture for 6 days
13	LCYZO7D	storage under 4 dgree in BD standard for 10 days , then growth in F culture for 7 days
14	LCYZO8D	storage under 4 dgree in BD standard for 10 days , then growth in F culture for 8 days
15	LCIYCFAYZA0D	growth of the 4 degree stored samples in IYCFA medium for 0 days
16	lcIYCFAYZA10d	growth of the 4 degree stored samples in IYCFA medium for 10 days
17	lcIYCFAYZA14d	growth of the 4 degree stored samples in IYCFA medium for 14 days
18	lcIYCFAYZA16d	growth of the 4 degree stored samples in IYCFA medium for 16 days
19	lcIYCFAYZA17d	growth of the 4 degree stored samples in IYCFA medium for 17 days
20	lcIYCFAYZA18d	growth of the 4 degree stored samples in IYCFA medium for 18 days
21	LCIYCFAYZA1D	growth of the 4 degree stored samples in IYCFA medium for 1 days
22	lcIYCFAYZA20d	growth of the 4 degree stored samples in IYCFA medium for 20 days

23	YCFA

23	lcIYCFAYZA2d	growth of the 4 degree stored samples in IYCFA medium for 2 days
24	LCIYCFAYZA3d	growth of the 4 degree stored samples in IYCFA medium for 3 days
25	lcIYCFA6d	growth of the 4 degree stored samples in IYCFA medium for 6 days
26	lcIYCFAYZA7d	growth of the 4 degree stored samples in IYCFA medium for 7 days
27	lcIYCFAYZA9d	growth of the 4 degree stored samples in IYCFA medium for 9 days
28	LCYZA0D	storage under 4 dgree in Lytic for 10 days , then growth in F +5%Blood+10% Fluid for 0 days
29	lcYZA10d	storage under 4 dgree in Lytic for 10 days , then growth in F +5%Blood+10% Fluid for 10 days
30	lcYZA12d	storage under 4 dgree in Lytic for 10 days , then growth in F +5%Blood+10% Fluid for 12 days
31	lcYZA13d	storage under 4 dgree in Lytic for 10 days , then growth in F +5%Blood+10% Fluid for 13 days
32	lcYZA14d	storage under 4 dgree in Lytic for 10 days , then growth in F +5%Blood+10% Fluid for 14 days
33	lcYZA15d	storage under 4 dgree in Lytic for 10 days , then growth in F +5%Blood+10% Fluid for 15 days
34	lcYZA17d	storage under 4 dgree in Lytic for 10 days , then growth in F +5%Blood+10% Fluid for 17 days
35	lcYZA19d	storage under 4 dgree in Lytic for 10 days , then growth in F +5%Blood+10% Fluid for 19 days
36	LCYZA1D	storage under 4 dgree in Lytic for 10 days , then growth in F +5%Blood+10% Fluid for 1 day
37	lcYZA21d	storage under 4 dgree in Lytic for 10 days , then growth in F +5%Blood+10% Fluid for 21 days
38	LCYZA2D	storage under 4 dgree in Lytic for 10 days , then growth in F +5%Blood+10% Fluid for 2 days
39	LCYZA3D	storage under 4 dgree in Lytic for 10 days , then growth in F +5%Blood+10% Fluid for 3 days
40	lcYZA5d	storage under 4 dgree in Lytic for 10 days , then growth in F +5%Blood+10% Fluid for 5 days

41	lcYZA6d	storage under 4 dgree in Lytic for 10 days , then growth in F +5%Blood+10% Fluid for 6 days
42	lcYZA7d	storage under 4 dgree in Lytic for 10 days , then growth in F +5%Blood+10% Fluid for 7 days
43	lcYZA8d	storage under 4 dgree in Lytic for 10 days , then growth in F +5%Blood+10% Fluid for 8 days
44	lcYZA9d	storage under 4 dgree in Lytic for 10 days , then growth in F +5%Blood+10% Fluid for 9 days
45	LCYZTO25D	growth of the 4 degree stored samples in Versa TREK1 for 25 days
46	LCMAYZO0D	growth of the 4 degree stored samples in seawater medium for 0 days
47	LCMAYZO10D	growth of the 4 degree stored samples in seawater medium for 10 days
48	LCMAYZO13D	growth of the 4 degree stored samples in seawater medium for 13 days
49	LCMAYZO15d	growth of the 4 degree stored samples in seawater medium for 15 days
50	LCMAYZO16d	growth of the 4 degree stored samples in seawater medium for 16 days
51	LCMAYZO17d	growth of the 4 degree stored samples in seawater medium for 17 days
52	LCMAYZO18d	growth of the 4 degree stored samples in seawater medium for 18 days
53	LCMAYZO1D	growth of the 4 degree stored samples in seawater medium for 1 day
54	LCMAYZO20d	growth of the 4 degree stored samples in seawater medium for 20 days
55	LCMAYZO25D	growth of the 4 degree stored samples in seawater medium for 25 days
56	LCMAYZO2D	growth of the 4 degree stored samples in seawater medium for 2 days
57	LCMAYZO3D	growth of the 4 degree stored samples in seawater medium for 3 days
58	LCMAYZO6D	growth of the 4 degree stored samples in seawater medium for 6 days
59	LCMAYZO7D	growth of the 4 degree stored samples in seawater medium for 7 days
60	LCMAYZO8D	growth of the 4 degree stored samples in seawater medium for 8 days
61	ldIYCFAYZA0d	growth of the -20 degree stored samples in IYCFA medium for 0 days
62	ldIYCFAYZA10d	growth of the -20 degree stored samples in IYCFA medium for 10 days
63	ldIYCFAYZA11d	growth of the -20 degree stored samples in IYCFA medium for 11 days

64	ldIYCFAYZA3d	growth of the -20 degree stored samples in IYCFA medium for 3 days
65	ldIYCFAYZA4d	growth of the -20 degree stored samples in IYCFA medium for 4 days
66	ldIYCFAYZA5d	growth of the -20 degree stored samples in IYCFA medium for 5 days
67	ldIYCFAYZA6d	growth of the -20 degree stored samples in IYCFA medium for 6 days
68	ldIYCFAYZA7d	growth of the -20 degree stored samples in IYCFA medium for 7 days
69	ldIYCFAYZA8d	growth of the -20 degree stored samples in IYCFA medium for 8 days
70	ldyza0d	growth of the -20 degree stored samples in Lytic medium for 10 days, then growth in F +5%Blood+10%Fluid for 0 day
71	ldYZA10d	growth of the -20 degree stored samples in Lytic medium for 10 days, then growth in F +5%Blood+11%Fluid for 10 days
72	ldYZA13d	growth of the -20 degree stored samples in Lytic medium for 10 days, then growth in F +5%Blood+11%Fluid for 13 days
73	ldyza15d	growth of the -20 degree stored samples in Lytic medium for 10 days, then growth in F +5%Blood+11%Fluid for 15 days
74	ldYZA17d	growth of the -20 degree stored samples in Lytic medium for 10 days, then growth in F +5%Blood+11%Fluid for 17 days
75	ldyza1d	growth of the -20 degree stored samples in Lytic medium for 10 days, then growth in F +5%Blood+11%Fluid for 1 day
76	ldYZA20d	growth of the -20 degree stored samples in Lytic medium for 10 days, then growth in F +5%Blood+11%Fluid for 20 days
77	ldYZA23d	growth of the -20 degree stored samples in Lytic medium for 10 days, then growth in F +5%Blood+11%Fluid for 23 days
78	ldYZA27d	growth of the -20 degree stored samples in Lytic medium for 10 days, then growth in F +5%Blood+11%Fluid for 27 days
79	LdYZA2d	growth of the -20 degree stored samples in Lytic medium for 10 days, then growth in F +5%Blood+11%Fluid for 2 days
80	ldYZA31d	growth of the -20 degree stored samples in Lytic medium for 10 days, then growth in F +5%Blood+11%Fluid for 31 days
81	ldYZA38d	growth of the -20 degree stored samples in Lytic medium for 10 days, then growth in F +5%Blood+11%Fluid for 38 days

82	ldyza3d	growth of the -20 degree stored samples in Lytic medium for 10 days, then growth in F +5%Blood+11%Fluid for 3 days
83	ldYZA42d	growth of the -20 degree stored samples in Lytic medium for 10 days, then growth in F +5%Blood+11%Fluid for 42 days
84	ldYZA4d	growth of the -20 degree stored samples in Lytic medium for 10 days, then growth in F +5%Blood+11%Fluid for 4 days
85	ldYZA5d	growth of the -20 degree stored samples in Lytic medium for 10 days, then growth in F +5%Blood+11%Fluid for 5 days
86	ldYZA6d	growth of the -20 degree stored samples in Lytic medium for 10 days, then growth in F +5%Blood+11%Fluid for 6 days
87	ldYZA7d	growth of the -20 degree stored samples in Lytic medium for 10 days, then growth in F +5%Blood+11%Fluid for 7 days
88	ldYZA8d	growth of the -20 degree stored samples in Lytic medium for 10 days, then growth in F +5%Blood+11%Fluid for 8 days
89	ldYZA9d	growth of the -20 degree stored samples in Lytic medium for 10 days, then growth in F +5%Blood+11%Fluid for 9 days
90	LDYZTO24D	growth of the -20 degree stored samples in Versa TREK1 for 24 days
91	LDYZTO25D	growth of the -20 degree stored samples in Versa TREK1 for 25 days
92	LDMAYZO24D	growth of the -20 degree stored samples in seawater medium for 24 days
93	LDMAYZO25D	growth of the -20 degree stored samples in seawater medium for 25 days
94	yza	growth of the fresh samples in BD Lytic for 210 days, then growth in F +5%Blood+11%Fluid for 0 day
95	YZTA10d	growth of the fresh samples in BD Lytic for 210 days, then growth in F +5%Blood+11%Fluid for 10 days
96	YZTA3d	growth of the fresh samples in BD Lytic for 210 days, then growth in F +5%Blood+11%Fluid for 3 days
97	YZTA7d	growth of the fresh samples in BD Lytic for 210 days, then growth in F +5%Blood+11%Fluid for 7 days
98	YZTO1d	growth of the fresh samples in Versa TREK1 for 1 day

Table S2. MAGs quanlity

	CheckM_completeness	CheckM_contamination	Quality
F68C_bin.101	97.32	0.34	HQ
F68C_bin.104	93.34	0	HQ
F68C_bin.111	97.65	0.34	HQ
F68C_bin.16	96.64	0	HQ
F68C_bin.19	91.55	3.36	HQ
F68C_bin.2	99.18	0	HQ
F68C_bin.20	91.25	0.74	HQ
F68C_bin.25	97.65	0	HQ
F68C_bin.26	90.91	1.7	HQ
F68C_bin.27	91.16	0.34	HQ
F68C_bin.32	99.3	1.17	HQ
F68C_bin.41	90.2	1.34	HQ
F68C_bin.42	91.1	2.04	HQ
F68C_bin.43	94.6	1.67	HQ
F6D7_bin.32	94.6	1.67	HQ
F68C_bin.44	99.33	0	HQ
F68C_bin.47	98.55	1.76	HQ
F68C_bin.49	96.16	0.19	HQ
F68H_bin.37	95.42	0.56	HQ
F68W_bin.15	94.3	0.19	HQ
F6DH_bin.120	96.53	0.56	HQ
F68C_bin.51	99.06	0	HQ
F6D7_bin.95	99.06	1.89	HQ
F68C_bin.54	98.82	2.4	HQ
F68C_bin.62	94.23	0.67	HQ
F68C_bin.70	96.63	2.08	HQ
F68C_bin.74	95.91	0	HQ
F68C_bin.76	97.47	1.58	HQ
F68C_bin.88	92.7	2.25	HQ
F68C_bin.89	100	0	HQ
F68N_bin.55	94.66	0.28	HQ
F68C_bin.94	97.5	0.62	HQ
F68C_bin.98	96.15	1.88	HQ
F68D_bin.103	97.54	3.74	HQ
F68D_bin.105	96.11	0	HQ
F68D_bin.12	92.52	1.43	HQ
F68D_bin.14	100	0.08	HQ
F68D_bin.19	95.74	0	HQ
F68C_bin.92	100	0	HQ
F68D_bin.21	100	0	HQ
F68G_bin.35	100	0	HQ
F68H_bin.60	100	0.8	HQ
F68J_bin.33	100	0.8	HQ
F68M_bin.53	100	0	HQ
F6D7_bin.99	91.29	1.66	HQ
F6D8_bin.35	100	0	HQ
F6DB_bin.111	100	0	HQ

Table S2. MAGs quanlity

	CheckM_completeness	CheckM_contamination	Quality
F6MX_bin.135	100	0	HQ
F6MY_bin.118	100	0	HQ
F68D_bin.30	98.79	0	HQ
F68D_bin.39	96.98	0.34	HQ
F68D_bin.41	92.6	1.34	HQ
F68D_bin.42	91.33	1.02	HQ
F68D_bin.46	97.52	0	HQ
F68G_bin.21	90.78	0	HQ
F68D_bin.47	99.12	0.58	HQ
F68D_bin.49	93.35	1.61	HQ
F68D_bin.5	96.15	0	HQ
F68D_bin.53	96.5	0.71	HQ
F68D_bin.56	95.66	1.96	HQ
F68D_bin.62	95.07	2.03	HQ
F68D_bin.65	97.82	0	HQ
F68D_bin.68	91.11	2.4	HQ
F68D_bin.69	96.4	0.81	HQ
F68D_bin.7	95.71	0.71	HQ
F68D_bin.70	96.64	0	HQ
F68D_bin.71	98.73	1.74	HQ
F6MY_bin.48	99.33	1.96	HQ
F68D_bin.72	90.73	1.1	HQ
F68D_bin.75	100	0	HQ
F68D_bin.77	93.15	0.81	HQ
F6MX_bin.131	93.15	2.42	HQ
F68D_bin.79	92.62	0.67	HQ
F68D_bin.80	92.84	1.35	HQ
F68D_bin.81	100	0.94	HQ
F68M_bin.7	100	0.94	HQ
F68D_bin.82	99.97	0.9	HQ
F68D_bin.83	94.63	1.34	HQ
F68D_bin.86	93.24	3.94	HQ
F68D_bin.89	97.5	0.38	HQ
F68K_bin.15	99.1	1.47	HQ
F6DE_bin.91	97.12	0.38	HQ
F68D_bin.9	98.31	0	HQ
F68D_bin.90	100	0.7	HQ
F68D_bin.93	97.96	0	HQ
F68D_bin.94	95.39	0.35	HQ
F68D_bin.97	94.36	2.33	HQ
F68D_bin.99	98.96	0	HQ
F68E_bin.10	99.37	1.27	HQ
F68U_bin.24	92.89	0.63	HQ
F6DA_bin.15	91.46	1.18	HQ
F68E_bin.11	96.35	0.38	HQ
F68E_bin.13	94.96	1.35	HQ
F68E_bin.17	95.64	0	HQ

Table S2. MAGs quanlity

	CheckM_completeness	CheckM_contamination	Quality
F68E_bin.18	97.28	4.88	HQ
F68E_bin.19	96.76	2.85	HQ
F68E_bin.20	93.7	0	HQ
F68E_bin.21	97.96	0	HQ
F68E_bin.22	93.02	0.72	HQ
F68E_bin.23	98.66	0	HQ
F68E_bin.27	98.4	0	HQ
F68E_bin.28	95.97	1.34	HQ
F68E_bin.3	98.07	1.45	HQ
F68E_bin.30	99.37	1.9	HQ
F68E_bin.31	100	0.11	HQ
F68E_bin.32	96.24	1.6	HQ
F68E_bin.38	98.2	0.9	HQ
F68E_bin.39	95.32	0.58	HQ
F6DB_bin.86	90.35	1.75	HQ
F68E_bin.4	100	0	HQ
F68E_bin.41	90.27	2.5	HQ
F68E_bin.42	96.94	0.56	HQ
F68E_bin.44	100	0.81	HQ
F68E_bin.45	95.2	1.29	HQ
F68E_bin.47	95.75	0.09	HQ
F68E_bin.6	97.95	0.88	HQ
F68F_bin.1	92.6	1.34	HQ
F68F_bin.10	90.34	1.7	HQ
F68C_bin.79	90.04	0.2	HQ
F68D_bin.8	94.15	0	HQ
F68F_bin.100	98.83	0	HQ
F68H_bin.56	97.58	0	HQ
F68K_bin.52	97	0.58	HQ
F68R_bin.23	95.91	0	HQ
F68T_bin.28	96.2	0.68	HQ
F6DB_bin.110	94.15	0	HQ
F6DG_bin.9	98.45	0	HQ
F6MY_bin.32	97.66	1.56	HQ
F68F_bin.101	95.08	0.69	HQ
F68F_bin.103	91.88	1.5	HQ
F68F_bin.108	100	0	HQ
F68H_bin.73	99.4	0	HQ
F68T_bin.53	99.4	0	HQ
F68F_bin.111	91.83	0	HQ
F68F_bin.16	96.98	0	HQ
F68F_bin.19	97.88	0.3	HQ
F68F_bin.2	91.11	2.54	HQ
F68F_bin.23	100	0.8	HQ
F68F_bin.25	96.92	1.22	HQ
F68F_bin.28	99.35	0.96	HQ
F68F_bin.30	96.64	0.67	HQ

Table S2. MAGs quanlity

	CheckM_completeness	CheckM_contamination	Quality
F68F_bin.33	97.27	0	HQ
F68F_bin.34	96.39	0.63	HQ
F68F_bin.35	100	2.42	HQ
F68F_bin.37	92.08	0.75	HQ
F68F_bin.38	99.19	0	HQ
F68F_bin.39	97.38	1.25	HQ
F68F_bin.4	98.23	0	HQ
F68N_bin.18	90.17	1.65	HQ
F68F_bin.41	98.66	0	HQ
F68K_bin.87	98.66	0	HQ
F68M_bin.11	97.09	0.02	HQ
F68F_bin.43	97.99	0	HQ
F68F_bin.46	97.3	0.84	HQ
F68F_bin.5	99.33	0.83	HQ
F68F_bin.50	90.39	3.3	HQ
F68F_bin.51	95.97	1.2	HQ
F68F_bin.52	97.01	0.8	HQ
F68F_bin.55	97.1	0.24	HQ
F68F_bin.57	97.4	1.25	HQ
F68F_bin.59	92.67	1.08	HQ
F68F_bin.64	96.42	0	HQ
F68F_bin.68	97.58	0.12	HQ
F68F_bin.7	94.35	0	HQ
F68F_bin.70	99.37	0	HQ
F68T_bin.48	92.03	0.63	HQ
F6MX_bin.42	94.94	0	HQ
F68F_bin.74	92.26	1.01	HQ
F68F_bin.77	98.12	0.62	HQ
F68F_bin.81	95.97	1.7	HQ
F68F_bin.82	92.42	2.25	HQ
F68F_bin.84	97.13	1.93	HQ
F68F_bin.85	93.96	0.89	HQ
F68F_bin.89	94.35	1.01	HQ
F68F_bin.9	96.64	0.68	HQ
F6MY_bin.134	95.56	2.27	HQ
F68F_bin.90	98.88	0.37	HQ
F68F_bin.91	96.81	0.34	HQ
F68F_bin.98	95.05	3.77	HQ
F68F_bin.99	98.07	0.6	HQ
F68G_bin.10	92.21	0.67	HQ
F68D_bin.88	90.57	1.84	HQ
F68G_bin.12	95.43	1.19	HQ
F68G_bin.13	97.99	0	HQ
F68G_bin.14	90.56	1.27	HQ
F68G_bin.20	93.09	1.31	HQ
F68G_bin.22	96.81	0.67	HQ
F68G_bin.26	93.96	1.36	HQ

Table S2. MAGs quanlity

	CheckM_completeness	CheckM_contamination	Quality
F68G_bin.27	99.12	0.24	HQ
F68G_bin.28	99.28	0.97	HQ
F68G_bin.29	92.52	0.95	HQ
F68G_bin.31	94.97	4.13	HQ
F68G_bin.33	92	4	HQ
F68G_bin.37	97.05	1.7	HQ
F68G_bin.39	97.78	0.63	HQ
F68G_bin.4	97.6	0	HQ
F68N_bin.63	96.8	0	HQ
F68G_bin.41	92.74	1.88	HQ
F68C_bin.82	96.6	0	HQ
F68G_bin.46	97.27	0	HQ
F68W_bin.72	96.6	0	HQ
F6DH_bin.44	96.6	0	HQ
F68G_bin.47	93.35	0.81	HQ
F68G_bin.50	96.95	0.89	HQ
F68R_bin.64	96.14	4.31	HQ
F6D7_bin.47	98.07	1.21	HQ
F6DG_bin.86	92.18	1.55	HQ
F6MY_bin.45	97.21	0.97	HQ
F68G_bin.52	93.16	0.59	HQ
F68G_bin.53	96.57	3.49	HQ
F68E_bin.14	95.96	0.56	HQ
F68G_bin.54	98.76	0	HQ
F68G_bin.55	92.34	0.03	HQ
F68G_bin.56	99.19	0.81	HQ
F68G_bin.57	95.58	0.63	HQ
F68G_bin.6	98.73	0.32	HQ
F68V_bin.49	91.5	0.63	HQ
F68G_bin.61	96.64	3.25	HQ
F68G_bin.62	91.92	0.74	HQ
F68G_bin.63	92.04	0.67	HQ
F68G_bin.65	92.7	1.97	HQ
F68G_bin.66	91.95	0.67	HQ
F68G_bin.67	95.5	0	HQ
F68G_bin.68	93.85	0.06	HQ
F68G_bin.7	96.45	0.35	HQ
F68G_bin.72	91.28	0.67	HQ
F68G_bin.73	97.32	2.01	HQ
F68G_bin.74	99.46	0	HQ
F6MY_bin.83	93.14	4.01	HQ
F68G_bin.76	91.84	1.87	HQ
F68G_bin.77	90.44	1.01	HQ
F68G_bin.8	97.35	1.61	HQ
F68G_bin.85	95.62	2.27	HQ
F68E_bin.26	94.85	2.26	HQ
F68G_bin.80	98.02	1.17	HQ

Table S2. MAGs quanlity

	CheckM_completeness	CheckM_contamination	Quality
F68H_bin.1	98.5	0	HQ
F68K_bin.41	94.36	0	HQ
F68N_bin.25	97.1	1.57	HQ
F68P_bin.38	96.87	0	HQ
F68U_bin.54	95.86	0.56	HQ
F6MY_bin.44	94.36	0.01	HQ
F68H_bin.11	90.38	1.11	HQ
F68H_bin.14	95.8	0	HQ
F68H_bin.15	100	0.62	HQ
F68H_bin.16	95.42	3.67	HQ
F68H_bin.19	96.59	0.1	HQ
F68H_bin.2	96.14	3.67	HQ
F68H_bin.22	95.21	0.6	HQ
F68G_bin.51	97.36	0.05	HQ
F68H_bin.24	97.36	0	HQ
F68W_bin.7	96.98	0.5	HQ
F6DH_bin.14	96.6	0	HQ
F68H_bin.25	98.66	2.01	HQ
F6DB_bin.82	91.83	3.36	HQ
F68H_bin.28	97.09	0.24	HQ
F68H_bin.29	92.6	1.34	HQ
F68H_bin.3	95.89	1.21	HQ
F68H_bin.30	90.54	0.69	HQ
F68H_bin.31	97.48	1.01	HQ
F68H_bin.39	97.01	2.01	HQ
F68H_bin.4	98.66	0.67	HQ
F68H_bin.40	97.32	0	HQ
F68H_bin.44	94.92	1.83	HQ
F68H_bin.47	98.5	0.62	HQ
F6DB_bin.15	91.92	3.13	HQ
F68H_bin.49	95.19	1.51	HQ
F68H_bin.50	99.18	0.54	HQ
F68H_bin.52	95.48	0.8	HQ
F68H_bin.55	97.99	3.36	HQ
F68H_bin.6	97.27	0	HQ
F68H_bin.63	98.49	0.34	HQ
F68F_bin.65	92.42	0	HQ
F68H_bin.66	97.4	0.74	HQ
F68H_bin.7	99.19	0	HQ
F68H_bin.70	94.48	1.1	HQ
F68H_bin.72	100	1.87	HQ
F68H_bin.79	99.07	0.08	HQ
F68H_bin.80	91.66	3.86	HQ
F68J_bin.10	95.12	0.63	HQ
F68J_bin.11	98.8	0.19	HQ
F68J_bin.14	98.83	1.15	HQ
F68J_bin.16	92.32	0	HQ

Table S2. MAGs quanlity

	CheckM_completeness	CheckM_contamination	Quality
F68J_bin.19	92.54	1.61	HQ
F68D_bin.50	97.62	3.28	HQ
F68G_bin.19	97.98	1.92	HQ
F68J_bin.20	99.23	0.77	HQ
F68J_bin.22	95.77	0	HQ
F68J_bin.23	97.32	0	HQ
F68J_bin.25	97.75	1.12	HQ
F68J_bin.27	91.89	0.13	HQ
F68J_bin.29	95.97	3.86	HQ
F68J_bin.3	98.82	0.29	HQ
F68J_bin.32	91.01	3.08	HQ
F68J_bin.34	97.53	2.01	HQ
F68J_bin.37	90.51	2.93	HQ
F68J_bin.41	95.31	0.56	HQ
F68J_bin.42	93.15	1.61	HQ
F68J_bin.43	92.4	0.7	HQ
F68J_bin.44	99.4	1.2	HQ
F68J_bin.45	94.8	0.04	HQ
F68J_bin.5	97.4	1.25	HQ
F68R_bin.1	91.09	2.71	HQ
F68J_bin.51	94.43	1.86	HQ
F68J_bin.53	97.99	0	HQ
F68J_bin.55	98.32	0	HQ
F68J_bin.59	90.25	0	HQ
F68J_bin.6	94.29	0	HQ
F68J_bin.62	100	0.81	HQ
F68J_bin.65	99.33	0	HQ
F68C_bin.95	97.75	1.12	HQ
F68D_bin.23	93.26	2.81	HQ
F68J_bin.67	100	1.12	HQ
F68K_bin.38	97.56	2.25	HQ
F68P_bin.50	98.88	1.12	HQ
F6DA_bin.25	98.88	1.12	HQ
F6DG_bin.65	94.38	2.25	HQ
F68J_bin.69	95.63	0.17	HQ
F68J_bin.7	99.42	0	HQ
F68J_bin.72	94.57	0.81	HQ
F68J_bin.74	97.4	1.25	HQ
F68J_bin.76	94.63	0.17	HQ
F68J_bin.79	95.95	0.5	HQ
F68J_bin.81	100	0	HQ
F68J_bin.9	99.24	0	HQ
F68K_bin.1	92.12	0	HQ
F68K_bin.10	93.2	0.34	HQ
F68K_bin.103	90.94	0	HQ
F68K_bin.105	94.86	1.06	HQ
F68K_bin.108	94.27	2.94	HQ

Table S2. MAGs quanlity

	CheckM_completeness	CheckM_contamination	Quality
F68K_bin.18	93.16	2.24	HQ
F68K_bin.2	98.4	2.79	HQ
F68K_bin.24	99.2	0	HQ
F68K_bin.25	98.8	1.2	HQ
F68K_bin.26	91.02	1.63	HQ
F68K_bin.28	97.2	0	HQ
F68R_bin.90	97.43	0.4	HQ
F6DB_bin.61	96.15	0.34	HQ
F68K_bin.3	93.19	1.25	HQ
F68K_bin.32	97.76	0	HQ
F68K_bin.34	92.36	2.68	HQ
F68K_bin.35	98.11	0	HQ
F68K_bin.37	92.74	1.08	HQ
F68K_bin.39	97.32	0	HQ
F68K_bin.44	98.8	0	HQ
F68K_bin.46	93.06	0.67	HQ
F68K_bin.47	93.86	0	HQ
F68K_bin.49	92.36	0	HQ
F68K_bin.50	91.28	0.67	HQ
F68K_bin.51	91.32	2.19	HQ
F68K_bin.54	98.64	0	HQ
F68K_bin.55	94.36	1.13	HQ
F68K_bin.61	97.78	1.31	HQ
F68K_bin.66	96.45	0	HQ
F68K_bin.69	97.16	0	HQ
F6DE_bin.65	93.36	2.33	HQ
F68K_bin.72	91.02	4.11	HQ
F68K_bin.75	99.48	1.45	HQ
F68K_bin.77	97.84	1.38	HQ
F68K_bin.78	93.86	2.35	HQ
F68K_bin.79	97.99	0	HQ
F68K_bin.8	90.08	1.15	HQ
F68K_bin.82	96.63	0.67	HQ
F68K_bin.83	99.33	1.01	HQ
F68K_bin.91	97.32	0.34	HQ
F68K_bin.96	91.13	0.74	HQ
F68K_bin.98	95.3	0	HQ
F68M_bin.12	99.19	1.08	HQ
F68M_bin.13	99.6	1.49	HQ
F68M_bin.15	99.63	0	HQ
F68M_bin.2	99.42	0.13	HQ
F68M_bin.22	96.58	1.35	HQ
F68V_bin.1	90.61	4.05	HQ
F68D_bin.6	95.83	0.48	HQ
F68M_bin.24	100	0	HQ
F68T_bin.26	97.36	1.12	HQ
F6D8_bin.87	97	2	HQ

Table S2. MAGs quanlity

	CheckM_completeness	CheckM_contamination	Quality
F6DA_bin.5	95.43	0	HQ
F6DB_bin.18	94.23	0.16	HQ
F6DE_bin.52	99.6	0.18	HQ
F6DG_bin.62	94.71	4.25	HQ
F6MX_bin.88	99.04	0	HQ
F6MY_bin.85	94.71	0.96	HQ
F68M_bin.26	96.8	0	HQ
F68M_bin.27	90.64	4.01	HQ
F68M_bin.3	90.1	3.58	HQ
F68M_bin.30	94.3	2.55	HQ
F68D_bin.44	97.15	1.14	HQ
F68M_bin.31	96.53	0.67	HQ
F6D9_bin.19	95.02	1.98	HQ
F68M_bin.32	99.19	0.81	HQ
F68M_bin.33	98.93	0	HQ
F68M_bin.34	97.99	2.01	HQ
F68M_bin.35	95.13	0.67	HQ
F68M_bin.37	91.22	0.68	HQ
F68M_bin.39	93.96	1.7	HQ
F68M_bin.40	94.77	2.22	HQ
F68M_bin.41	95.58	0.68	HQ
F68M_bin.42	97.54	0.42	HQ
F6DA_bin.63	91.89	0.84	HQ
F68M_bin.46	95.3	1.34	HQ
F68M_bin.49	90.9	1.24	HQ
F68M_bin.50	98.58	0.71	HQ
F68M_bin.55	98.39	1.61	HQ
F68M_bin.57	97.27	0	HQ
F6MX_bin.4	92.53	1.73	HQ
F68M_bin.6	98.52	0.32	HQ
F68M_bin.60	95.03	0.57	HQ
F68M_bin.61	97.32	0.69	HQ
F68V_bin.26	96.56	1.07	HQ
F6MY_bin.60	94.63	1.46	HQ
F68M_bin.65	92.6	1.34	HQ
F6DB_bin.99	92.6	1.34	HQ
F68M_bin.67	97.57	0.88	HQ
F68M_bin.68	91.53	0	HQ
F68M_bin.70	98.94	1.06	HQ
F68M_bin.72	90.84	4.76	HQ
F68K_bin.63	96.06	0.56	HQ
F68M_bin.73	97.96	0.37	HQ
F6D7_bin.87	93.48	1.6	HQ
F68M_bin.76	96.32	0	HQ
F68M_bin.78	100	0	HQ
F68M_bin.84	96.09	3.34	HQ
F68M_bin.85	99.32	1.02	HQ

Table S2. MAGs quanlity

	CheckM_completeness	CheckM_contamination	Quality
F68M_bin.86	93.15	1.09	HQ
F68N_bin.12	95.91	0	HQ
F68N_bin.16	93.11	0.97	HQ
F68N_bin.2	96.2	0.46	HQ
F68N_bin.21	99.33	0	HQ
F68N_bin.22	98.95	0.02	HQ
F68N_bin.27	93.97	1.83	HQ
F68N_bin.28	96.49	2.42	HQ
F68N_bin.32	98.24	0.29	HQ
F68N_bin.39	94.1	2.04	HQ
F68N_bin.44	97.27	0	HQ
F68N_bin.50	93.66	0.05	HQ
F68N_bin.54	90.09	0.37	HQ
F68N_bin.56	99.33	0	HQ
F68N_bin.57	98.66	1.01	HQ
F68N_bin.58	93.94	0.67	HQ
F68N_bin.6	90.7	2.57	HQ
F68N_bin.60	98.68	0.6	HQ
F68N_bin.64	92.6	1.34	HQ
F68N_bin.65	97.99	0	HQ
F68N_bin.66	93.13	0.98	HQ
F68M_bin.58	100	1.61	HQ
F68N_bin.67	99.19	0.16	HQ
F68N_bin.68	95.17	1.7	HQ
F68N_bin.69	99.59	0	HQ
F68N_bin.73	97.63	0.59	HQ
F68R_bin.15	92.94	1.48	HQ
F6MY_bin.100	94.38	0.59	HQ
F68K_bin.14	96.81	0.97	HQ
F68N_bin.75	97.48	0.34	HQ
F68N_bin.76	93.98	1.87	HQ
F68N_bin.78	97.99	0	HQ
F68N_bin.79	97.99	0	HQ
F6DE_bin.57	97.32	0	HQ
F68P_bin.11	98.21	1.93	HQ
F68P_bin.14	98.75	0.62	HQ
F68H_bin.67	90.6	0.67	HQ
F68P_bin.18	92.62	0	HQ
F68P_bin.20	96.73	4	HQ
F68P_bin.21	94.61	0.17	HQ
F68P_bin.32	95.23	0.19	HQ
F68P_bin.39	99.06	0.31	HQ
F68P_bin.41	93.01	1.02	HQ
F68P_bin.43	92.43	0.91	HQ
F68P_bin.46	98.66	0	HQ
F68P_bin.5	100	0.81	HQ
F68P_bin.55	95.95	0	HQ

Table S2. MAGs quanlity

	CheckM_completeness	CheckM_contamination	Quality
F68P_bin.6	93.5	0	HQ
F68P_bin.7	97.52	1.53	HQ
F68P_bin.9	97.1	0.6	HQ
F68R_bin.103	97.18	0.93	HQ
F68R_bin.109	91.95	0.34	HQ
F68R_bin.11	98.11	0	HQ
F68R_bin.110	93.09	0.67	HQ
F68R_bin.118	92.62	0.67	HQ
F68R_bin.121	93.06	3.36	HQ
F68R_bin.124	91.11	0.19	HQ
F68R_bin.19	92.6	1.34	HQ
F68R_bin.28	94.13	0.02	HQ
F68R_bin.31	99.59	0	HQ
F68D_bin.74	98.39	0	HQ
F68F_bin.18	98.39	0.81	HQ
F68R_bin.34	98.39	0	HQ
F68R_bin.35	96.21	0	HQ
F68R_bin.37	90.79	0.05	HQ
F68R_bin.4	94.65	0.6	HQ
F68R_bin.42	94.22	2.28	HQ
F68R_bin.43	92.21	0.34	HQ
F68R_bin.52	97.32	0	HQ
F68R_bin.56	100	1.69	HQ
F68F_bin.58	98.66	0	HQ
F68R_bin.59	98.66	0	HQ
F68R_bin.6	91.51	0.94	HQ
F68R_bin.62	96.42	0	HQ
F68R_bin.66	91	0.7	HQ
F68R_bin.69	93.35	0.81	HQ
F68D_bin.26	96.6	0	HQ
F68R_bin.7	97.27	0	HQ
F68T_bin.86	95.26	0.44	HQ
F68R_bin.71	95.41	0.34	HQ
F68R_bin.72	96.2	0.71	HQ
F68R_bin.73	94.97	1.34	HQ
F68R_bin.74	92.61	2.94	HQ
F68R_bin.75	90.6	0.67	HQ
F68R_bin.77	91.12	1.27	HQ
F68T_bin.50	90.17	3.8	HQ
F68R_bin.79	98.11	1.86	HQ
F68K_bin.92	98.14	0.62	HQ
F68N_bin.48	98.14	1.24	HQ
F68R_bin.81	98.14	0.62	HQ
F68R_bin.86	98.39	0.81	HQ
F68R_bin.92	97.67	0.24	HQ
F68R_bin.98	95.32	1.57	HQ
F68T_bin.101	99.78	0.67	HQ

Table S2. MAGs quanlity

	CheckM_completeness	CheckM_contamination	Quality
F68T_bin.102	97.7	0.68	HQ
F68T_bin.106	98.66	0.34	HQ
F68T_bin.18	98.16	0.92	HQ
F68T_bin.20	100	0.24	HQ
F68T_bin.21	97.32	0	HQ
F68T_bin.27	92.43	2.7	HQ
F68T_bin.31	95.27	0.59	HQ
F68T_bin.32	97.36	0	HQ
F68K_bin.88	97.99	1.45	HQ
F68T_bin.35	91.32	0	HQ
F68T_bin.37	98.54	0.88	HQ
F68T_bin.4	90.92	1.52	HQ
F68T_bin.40	97.59	0.9	HQ
F68T_bin.41	97.99	1.01	HQ
F68T_bin.42	96.26	0.08	HQ
F68T_bin.44	96.7	1.42	HQ
F68T_bin.45	92.89	1.11	HQ
F68T_bin.46	98.66	0	HQ
F68T_bin.5	99.3	1.19	HQ
F68T_bin.51	97.65	0	HQ
F6D8_bin.27	96.98	0	HQ
F68T_bin.52	94.54	0	HQ
F68T_bin.59	98.77	0	HQ
F68T_bin.66	97.47	0	HQ
F68E_bin.36	90.54	2.27	HQ
F68K_bin.9	99.04	0.96	HQ
F68N_bin.19	94.87	1.92	HQ
F68T_bin.67	99.52	0.96	HQ
F6D7_bin.44	96.79	0.99	HQ
F6DB_bin.23	97.12	0.96	HQ
F6DE_bin.28	99.52	0.96	HQ
F6DG_bin.87	99.52	1.44	HQ
F6MY_bin.39	98.72	1.44	HQ
F68F_bin.73	97.53	0.64	HQ
F68T_bin.69	94.53	0	HQ
F68U_bin.102	95.54	0.57	HQ
F68T_bin.70	98.56	1.53	HQ
F68T_bin.73	96.55	1.02	HQ
F68T_bin.74	97.68	2.85	HQ
F68T_bin.76	97.08	1.25	HQ
F68T_bin.79	95.9	1.57	HQ
F68T_bin.82	90.6	0	HQ
F68T_bin.84	95.39	0.17	HQ
F68T_bin.85	94.41	0	HQ
F68T_bin.88	96.6	0	HQ
F68T_bin.90	97.55	0.2	HQ
F68U_bin.101	96.33	1.61	HQ

Table S2. MAGs quanlity

	CheckM_completeness	CheckM_contamination	Quality
F68U_bin.103	90.2	0.49	HQ
F68F_bin.62	92.37	4.09	HQ
F68U_bin.106	96.84	1.84	HQ
F68U_bin.107	97.12	0.62	HQ
F6DH_bin.110	95	1.38	HQ
F68U_bin.11	98.14	1.55	HQ
F68U_bin.17	93.67	4.02	HQ
F68U_bin.18	98.8	1.8	HQ
F68U_bin.19	97.75	0	HQ
F68U_bin.20	95.33	0	HQ
F68U_bin.21	98.39	4.44	HQ
F68U_bin.23	98.1	1.27	HQ
F6DG_bin.41	94.58	1.27	HQ
F6MY_bin.106	93.88	0.79	HQ
F68U_bin.3	94.1	0.48	HQ
F6DH_bin.9	91.37	2.16	HQ
F68U_bin.30	98.66	0	HQ
F68W_bin.62	98.66	0	HQ
F6DE_bin.59	98.66	0	HQ
F6DH_bin.76	98.66	0.02	HQ
F68U_bin.34	96.23	1.25	HQ
F68U_bin.38	93.46	0	HQ
F68U_bin.39	99.03	2.09	HQ
F68U_bin.40	93.54	1.8	HQ
F68W_bin.9	91.29	3.92	HQ
F6DH_bin.68	91.29	2.69	HQ
F68U_bin.41	96.26	3.34	HQ
F68U_bin.47	98.07	0	HQ
F68U_bin.49	96.31	0.67	HQ
F68U_bin.5	96.08	0	HQ
F6DH_bin.46	94.15	1.08	HQ
F68U_bin.51	91.56	2.62	HQ
F68U_bin.55	100	1.12	HQ
F68U_bin.56	94.62	0.86	HQ
F68U_bin.6	99.23	0	HQ
F68U_bin.61	90.84	2.01	HQ
F68U_bin.65	97.27	0.34	HQ
F68U_bin.66	97.36	0	HQ
F68K_bin.95	97.99	0	HQ
F68U_bin.68	97.99	0	HQ
F68U_bin.7	90.62	1.34	HQ
F68U_bin.70	91.95	0	HQ
F68U_bin.71	92.98	1.75	HQ
F68U_bin.75	94.27	1.04	HQ
F68U_bin.78	93.96	0	HQ
F68U_bin.79	99.69	0.15	HQ
F68U_bin.80	98.11	0.79	HQ

Table S2. MAGs quanlity

	CheckM_completeness	CheckM_contamination	Quality
F68U_bin.81	99.03	1.28	HQ
F68U_bin.91	92.13	0.71	HQ
F68N_bin.31	93.83	0.43	HQ
F68U_bin.96	98.99	0	HQ
F68W_bin.57	98.99	0	HQ
F6DH_bin.64	98.83	0	HQ
F68U_bin.97	93.35	1.48	HQ
F6DH_bin.54	90.93	1.98	HQ
F68U_bin.98	98.66	0.67	HQ
F68W_bin.3	97.32	0.67	HQ
F6DH_bin.5	96.64	0.67	HQ
F68V_bin.108	98.99	1.79	HQ
F68V_bin.109	98.28	0	HQ
F68V_bin.11	93.74	1.08	HQ
F68J_bin.21	97.63	0.67	HQ
F68V_bin.112	97.48	0.34	HQ
F68V_bin.113	93.92	2.93	HQ
F68M_bin.75	95.61	4.9	HQ
F68T_bin.3	96.96	0.38	HQ
F68V_bin.116	99.04	0.38	HQ
F6DA_bin.39	98.53	0.9	HQ
F6DB_bin.31	91.86	3.6	HQ
F6DE_bin.62	95.64	0.38	HQ
F6DG_bin.106	99.23	1.25	HQ
F68V_bin.117	99.33	0.34	HQ
F68V_bin.13	97.76	0	HQ
F68V_bin.14	96.64	0.67	HQ
F68V_bin.15	99.33	1.01	HQ
F68V_bin.2	93.97	2.66	HQ
F68V_bin.20	99.28	1.22	HQ
F68V_bin.22	97.28	0	HQ
F68V_bin.23	97.27	0	HQ
F68V_bin.27	92.26	0	HQ
F68V_bin.28	91.44	0.67	HQ
F68K_bin.21	92.62	1.34	HQ
F68V_bin.31	94.32	1.34	HQ
F68V_bin.35	100	1.21	HQ
F68V_bin.37	99.2	0	HQ
F68V_bin.42	93.29	0.67	HQ
F68C_bin.109	91.97	1.22	HQ
F68V_bin.45	99.42	0.13	HQ
F6MY_bin.77	93.11	1.41	HQ
F68V_bin.47	92.48	2.68	HQ
F68V_bin.50	97.53	0.15	HQ
F68V_bin.52	90.6	0	HQ
F68V_bin.53	94.44	1.05	HQ
F68V_bin.54	93.46	2.2	HQ

Table S2. MAGs quanlity

	CheckM_completeness	CheckM_contamination	Quality
F68V_bin.58	99.33	0.22	HQ
F68V_bin.59	98.6	1.52	HQ
F68V_bin.6	97.1	0	HQ
F68V_bin.60	91.25	2.01	HQ
F68D_bin.91	95.02	0	HQ
F68H_bin.34	97.91	0	HQ
F68M_bin.17	92.57	0	HQ
F68N_bin.70	90.32	0	HQ
F68U_bin.9	96.67	0.56	HQ
F68V_bin.62	98.88	0	HQ
F68V_bin.65	94.58	1.46	HQ
F68V_bin.66	95.36	0.45	HQ
F68V_bin.68	98.99	0.34	HQ
F6MX_bin.81	90.91	1.76	HQ
F68V_bin.71	99.33	0	HQ
F68V_bin.72	94.13	0.67	HQ
F68V_bin.74	95.9	0.95	HQ
F68V_bin.76	97.79	0	HQ
F68V_bin.8	98.66	0	HQ
F68V_bin.83	95.51	0.81	HQ
F68V_bin.84	96.42	0	HQ
F68V_bin.85	97.32	0.67	HQ
F68V_bin.93	91.34	0.58	HQ
F68W_bin.1	96.57	0.48	HQ
F68U_bin.35	91.03	0	HQ
F68W_bin.100	91.03	0	HQ
F6DH_bin.113	91.03	0	HQ
F68W_bin.101	97.03	0.36	HQ
F6DH_bin.50	94.37	0	HQ
F68W_bin.108	95.25	0.87	HQ
F6DH_bin.32	93.83	0.63	HQ
F68W_bin.12	90.6	0	HQ
F6DH_bin.57	90.6	1.34	HQ
F68W_bin.13	94.33	1.53	HQ
F68W_bin.17	94.51	1.96	HQ
F6DH_bin.128	91.35	2.23	HQ
F68J_bin.57	96.23	0	HQ
F68K_bin.58	96.53	0.24	HQ
F68W_bin.30	99.06	0	HQ
F6DH_bin.79	99.06	0	HQ
F68W_bin.36	94.63	2.03	HQ
F68W_bin.37	93.82	0	HQ
F6DH_bin.82	93.26	1.12	HQ
F68W_bin.38	94.03	0.3	HQ
F68U_bin.37	94.17	0.02	HQ
F68W_bin.54	98.02	0.47	HQ
F6DH_bin.69	93.93	0	HQ

Table S2. MAGs quanlity

	CheckM_completeness	CheckM_contamination	Quality
F68W_bin.6	93.59	3.13	HQ
F68W_bin.77	94.19	2.04	HQ
F6DH_bin.102	92.38	2.45	HQ
F68W_bin.78	97.45	0	HQ
F6DH_bin.88	97.45	0	HQ
F68W_bin.8	94.8	1.87	HQ
F68W_bin.80	99.28	0	HQ
F6DH_bin.90	98.79	0	HQ
F68W_bin.87	94.91	0.5	HQ
F6DH_bin.72	96.04	0.88	HQ
F68W_bin.88	90.82	2.34	HQ
F68W_bin.90	92.03	1.34	HQ
F6D7_bin.1	91.61	0.81	HQ
F6D7_bin.10	92.08	1	HQ
F6D7_bin.107	95.71	3.75	HQ
F6D7_bin.11	91.93	0	HQ
F6D7_bin.13	95.3	3.69	HQ
F6D7_bin.14	97.96	0	HQ
F6D7_bin.15	92.38	0.11	HQ
F6D7_bin.16	96.61	2.66	HQ
F6D7_bin.17	93.4	2.01	HQ
F6D7_bin.2	97.97	0	HQ
F68D_bin.52	93.2	2.03	HQ
F6D7_bin.22	93.88	1.35	HQ
F6D7_bin.26	98.81	0.59	HQ
F6D7_bin.27	94.59	0.68	HQ
F6D7_bin.28	98.66	1.01	HQ
F6D7_bin.3	99.28	1.93	HQ
F6D7_bin.30	96.29	1.72	HQ
F6D7_bin.31	92.42	3.37	HQ
F6D7_bin.33	97.04	1.21	HQ
F6D7_bin.35	95.89	0.95	HQ
F6D7_bin.38	93.82	0.56	HQ
F6D7_bin.4	95.97	0	HQ
F6D7_bin.40	91.59	0.04	HQ
F6D7_bin.43	95.97	0	HQ
F6D7_bin.46	93.61	0.63	HQ
F6D7_bin.48	95.57	0.63	HQ
F6D7_bin.5	99.54	2.63	HQ
F6D7_bin.51	96.53	0.68	HQ
F6D7_bin.52	97.99	0	HQ
F6D7_bin.54	97.99	0.13	HQ
F6D7_bin.56	96.58	0	HQ
F6D7_bin.58	97.99	0	HQ
F6D7_bin.59	95.01	2.17	HQ
F6D7_bin.6	97.27	0.34	HQ
F6D7_bin.73	99.33	0	HQ

Table S2. MAGs quanlity

	CheckM_completeness	CheckM_contamination	Quality
F6D7_bin.74	93.76	4.37	HQ
F68C_bin.66	92.6	1.34	HQ
F6D7_bin.77	92.6	1.34	HQ
F6D7_bin.81	93.89	2.48	HQ
F6D7_bin.82	97.65	1.34	HQ
F6D7_bin.83	95.56	0.05	HQ
F6D7_bin.84	98.66	0	HQ
F6D7_bin.86	96.42	0	HQ
F6D7_bin.90	96.08	0.37	HQ
F6D7_bin.94	99.08	0.07	HQ
F6D7_bin.97	90.19	2.24	HQ
F68R_bin.36	97.99	0.67	HQ
F6D8_bin.11	96.64	0	HQ
F6D8_bin.14	97.18	0.72	HQ
F6D8_bin.17	99.38	0.9	HQ
F6D8_bin.18	95.8	0	HQ
F68M_bin.36	97.32	0.36	HQ
F6D8_bin.19	97.99	0	HQ
F6D8_bin.20	98.83	0	HQ
F6D8_bin.21	96.5	1.3	HQ
F6D8_bin.22	95.93	0	HQ
F6D8_bin.25	93.23	1.64	HQ
F68R_bin.80	92.6	0	HQ
F6D8_bin.26	98.64	0	HQ
F6D8_bin.3	95.65	2.22	HQ
F6D8_bin.30	90.71	0	HQ
F6D8_bin.37	99.18	0	HQ
F6D8_bin.38	96.64	0.73	HQ
F6D8_bin.41	96.26	2.08	HQ
F6D8_bin.42	97.04	0.74	HQ
F6D8_bin.43	99.33	0	HQ
F6D8_bin.47	97.82	0	HQ
F6D8_bin.49	96.49	4	HQ
F6D8_bin.5	95.74	3.9	HQ
F6D8_bin.50	92.4	0.7	HQ
F6D8_bin.51	94.41	1.57	HQ
F6D8_bin.52	97.27	0	HQ
F6D8_bin.54	100	0.81	HQ
F6D8_bin.55	96.09	1.34	HQ
F6D8_bin.57	92.95	0	HQ
F6D8_bin.58	93.96	0.84	HQ
F6DG_bin.43	99.06	2.36	HQ
F6D8_bin.6	92.6	1.34	HQ
F68D_bin.92	97.58	0.81	HQ
F68N_bin.3	99.19	1.61	HQ
F6D8_bin.63	99.19	0.81	HQ
F6D8_bin.64	93.29	0.34	HQ

Table S2. MAGs quanlity

	CheckM_completeness	CheckM_contamination	Quality
F6D8_bin.66	96.62	2.24	HQ
F6D8_bin.68	99.33	1.01	HQ
F6D8_bin.7	92.7	4.49	HQ
F6D8_bin.8	100	0	HQ
F6D8_bin.84	99.28	2.9	HQ
F6D8_bin.86	95.56	0.05	HQ
F6D8_bin.9	98.66	0	HQ
F6D8_bin.91	99.52	1.2	HQ
F6D8_bin.92	97.32	0	HQ
F6D8_bin.93	97.9	1.17	HQ
F68G_bin.49	92.6	1.34	HQ
F6D9_bin.1	92.6	1.34	HQ
F6D9_bin.10	99.36	4.46	HQ
F6D9_bin.11	91.07	2.68	HQ
F6D9_bin.12	99.9	1.29	HQ
F6D9_bin.14	96.77	1.73	HQ
F6D9_bin.15	100	0.7	HQ
F6D9_bin.17	99.33	0.67	HQ
F6D9_bin.18	93.29	1.34	HQ
F6D9_bin.2	98.66	0.67	HQ
F6D9_bin.23	97.58	0	HQ
F6D9_bin.25	94.28	0.56	HQ
F6D9_bin.29	93.27	1.98	HQ
F6D9_bin.3	99.28	0	HQ
F6D9_bin.30	93.29	1.34	HQ
F6D9_bin.33	95.75	0	HQ
F6D9_bin.35	99.4	0.6	HQ
F68T_bin.7	96.27	0.54	HQ
F6D9_bin.39	98.92	0.27	HQ
F68D_bin.3	92.07	2.88	HQ
F6D8_bin.45	97.28	1.68	HQ
F6D9_bin.4	98.56	1.6	HQ
F6D9_bin.40	96.92	1.58	HQ
F6D9_bin.41	97.44	0.67	HQ
F6D9_bin.42	97.27	0	HQ
F6DA_bin.48	97.27	0	HQ
F6D9_bin.45	94.43	1.41	HQ
F6D9_bin.48	93.81	1.64	HQ
F6D9_bin.5	97.54	0.09	HQ
F6D9_bin.50	99.33	0.67	HQ
F6D9_bin.53	97.65	0.34	HQ
F6DH_bin.51	97.48	0.34	HQ
F6D9_bin.54	93.96	0	HQ
F6D9_bin.59	96.23	0.5	HQ
F6D9_bin.63	94.09	0.71	HQ
F6D9_bin.64	97.05	0	HQ
F6D9_bin.65	99.33	0.34	HQ

Table S2. MAGs quanlity

	CheckM_completeness	CheckM_contamination	Quality
F6D9_bin.69	98.65	0.9	HQ
F6D9_bin.7	97.32	0.67	HQ
F6D9_bin.71	95.95	0	HQ
F6D9_bin.73	96.24	0	HQ
F6D9_bin.74	94.56	0.62	HQ
F6D9_bin.77	98.66	0.34	HQ
F6D9_bin.78	91.55	1.28	HQ
F68N_bin.9	91.53	0	HQ
F6D9_bin.79	100	0	HQ
F6DB_bin.63	94.92	0	HQ
F68E_bin.34	100	0.13	HQ
F68M_bin.1	100	0	HQ
F68V_bin.38	100	0	HQ
F6D9_bin.8	100	0	HQ
F68N_bin.15	100	0	HQ
F68R_bin.18	100	0	HQ
F68T_bin.78	97.6	0	HQ
F68U_bin.100	100	0	HQ
F6D9_bin.81	100	0	HQ
F6D9_bin.82	97.32	0	HQ
F6D9_bin.84	94.06	2.1	HQ
F6D9_bin.89	95.75	1.34	HQ
F6D9_bin.93	100	1.24	HQ
F6DA_bin.13	97.73	0.84	HQ
F6DA_bin.18	99.03	0	HQ
F6DA_bin.19	97.99	0.34	HQ
F6DA_bin.22	97.6	0.6	HQ
F68K_bin.94	93.26	2.95	HQ
F68U_bin.48	99.24	0.74	HQ
F6DA_bin.29	99.62	0.1	HQ
F6DA_bin.3	91.1	1.52	HQ
F6DA_bin.32	95.99	0	HQ
F6DA_bin.33	93.96	1.69	HQ
F6DA_bin.35	94.28	4.36	HQ
F6DA_bin.36	93.6	2.41	HQ
F6DA_bin.37	91.93	1.34	HQ
F68N_bin.29	91.05	3.27	HQ
F68P_bin.10	99.02	0.9	HQ
F68U_bin.36	95.19	0.38	HQ
F6DA_bin.41	99.04	0.13	HQ
F6DA_bin.43	97.32	0	HQ
F6DA_bin.44	93.67	0.63	HQ
F6DA_bin.45	96.44	0.19	HQ
F6DA_bin.47	91.08	2.42	HQ
F6DA_bin.49	98.66	0	HQ
F6DA_bin.51	98.49	2.72	HQ
F6DA_bin.53	92.51	0	HQ

Table S2. MAGs quanlity

	CheckM_completeness	CheckM_contamination	Quality
F6DA_bin.55	94.57	0.17	HQ
F6DA_bin.57	93.1	1.34	HQ
F6DA_bin.60	92.2	0.02	HQ
F6DA_bin.61	99.06	0	HQ
F6DA_bin.64	94.06	0.56	HQ
F6DA_bin.66	90.77	0.97	HQ
F6DA_bin.69	92.62	0.67	HQ
F6DA_bin.7	97.99	0.34	HQ
F6MY_bin.22	90.49	1.85	HQ
F6DA_bin.70	97.99	0	HQ
F6DA_bin.72	100	1.27	HQ
F6DA_bin.75	93.89	3.6	HQ
F6DA_bin.76	99.33	0	HQ
F6DA_bin.77	100	0.48	HQ
F6DA_bin.79	90.27	0	HQ
F6DA_bin.82	98.14	1.55	HQ
F6DA_bin.85	96.18	0.32	HQ
F6DA_bin.86	91.3	0	HQ
F6DA_bin.87	92.36	0	HQ
F6DA_bin.88	97.32	0	HQ
F68C_bin.22	95.97	0	HQ
F68N_bin.72	95.82	0.03	HQ
F6DA_bin.9	95.97	0	HQ
F6DA_bin.93	97.32	0	HQ
F6D9_bin.68	94.23	2.15	HQ
F6DA_bin.95	97.99	0	HQ
F6DA_bin.97	91.78	2.17	HQ
F6DB_bin.10	97.63	3.45	HQ
F68R_bin.33	98.37	1.53	HQ
F68T_bin.63	100	1.34	HQ
F6DB_bin.102	99.35	0.19	HQ
F6DB_bin.103	97.99	0	HQ
F6DB_bin.105	93.24	2.36	HQ
F6DB_bin.107	93.62	2.01	HQ
F68R_bin.61	93.93	1.68	HQ
F6DB_bin.109	97.32	0	HQ
F6MY_bin.91	98.64	0.67	HQ
F6DB_bin.11	94.1	1.27	HQ
F6DB_bin.113	93.74	0	HQ
F6DB_bin.114	93.92	1.35	HQ
F6DB_bin.116	90.4	4.11	HQ
F6DB_bin.117	98.66	0.89	HQ
F6DB_bin.12	98.66	0	HQ
F6DB_bin.13	99.14	1.88	HQ
F6DB_bin.14	91.95	0.75	HQ
F6DB_bin.16	100	0.48	HQ
F6DB_bin.19	96.58	0.28	HQ

Table S2. MAGs quanlity

	CheckM_completeness	CheckM_contamination	Quality
F6DB_bin.2	97.27	0	HQ
F6DB_bin.21	96.12	1.52	HQ
F6DB_bin.24	98.39	0	HQ
F6DB_bin.29	95.97	0.94	HQ
F6DB_bin.3	93.25	2.53	HQ
F6DB_bin.32	98.75	0.62	HQ
F6DB_bin.33	97.83	0	HQ
F6DB_bin.36	96.5	1.52	HQ
F6DB_bin.38	99.37	0	HQ
F6DB_bin.43	100	0.23	HQ
F6DB_bin.44	97.99	0	HQ
F6DB_bin.45	95.3	0.67	HQ
F6MY_bin.18	91.37	0.67	HQ
F6DB_bin.47	98.63	0	HQ
F6DB_bin.48	96.85	1.2	HQ
F6DE_bin.77	100	2.08	HQ
F6DB_bin.51	97.32	0	HQ
F6DB_bin.54	98.66	0	HQ
F6DB_bin.56	99.29	0	HQ
F6DB_bin.58	90.56	2.91	HQ
F6DB_bin.6	99.33	1.34	HQ
F68N_bin.5	92.06	1.08	HQ
F6DB_bin.65	98.11	0.47	HQ
F6DB_bin.66	90.86	2.8	HQ
F6DB_bin.67	92.62	1.68	HQ
F6DB_bin.68	97.52	0	HQ
F68R_bin.65	95.97	0	HQ
F68V_bin.10	97.32	0.73	HQ
F6D7_bin.55	93.29	0	HQ
F6DB_bin.7	97.99	0	HQ
F6MY_bin.34	93.29	0	HQ
F68N_bin.23	92.8	2.24	HQ
F6DB_bin.74	93.96	2.01	HQ
F6DB_bin.76	99.33	0	HQ
F6D7_bin.98	93.74	0	HQ
F6DA_bin.6	92.31	0	HQ
F6DB_bin.79	97.63	0	HQ
F6MX_bin.121	99.7	0.59	HQ
F6DB_bin.81	94.76	2.69	HQ
F6DB_bin.83	98.66	0	HQ
F6DB_bin.87	93.89	2.03	HQ
F6MX_bin.7	92.54	2.03	HQ
F6DB_bin.88	94.37	0.96	HQ
F6DB_bin.9	94.9	0	HQ
F6D9_bin.91	93.66	2.15	HQ
F6DB_bin.90	99.98	0.6	HQ
F6DB_bin.91	93.1	3.92	HQ

Table S2. MAGs quanlity

	CheckM_completeness	CheckM_contamination	Quality
F6DB_bin.93	92.23	2.73	HQ
F68D_bin.4	98.53	0	HQ
F6DB_bin.95	98.53	0	HQ
F6D8_bin.40	92.54	0.16	HQ
F6DB_bin.97	97.56	0.16	HQ
F6DB_bin.98	98.21	1.87	HQ
F6DE_bin.10	95.27	1.87	HQ
F6DE_bin.11	99.63	0.07	HQ
F68F_bin.107	94.23	0	HQ
F68R_bin.78	91.27	0.96	HQ
F6D8_bin.29	98	0.48	HQ
F6DB_bin.64	100	0.48	HQ
F6DE_bin.14	98.56	0	HQ
F6MX_bin.22	98.56	0	HQ
F6MY_bin.7	98.56	0.48	HQ
F6DE_bin.15	94.18	3.55	HQ
F6DE_bin.16	92.01	3.94	HQ
F6DE_bin.17	91.94	0.03	HQ
F6DG_bin.37	93.68	3.28	HQ
F6DE_bin.19	96.63	0	HQ
F68G_bin.40	96.06	0.67	HQ
F6DE_bin.2	99.33	0	HQ
F6DB_bin.84	94.63	3.02	HQ
F6DE_bin.21	92.17	2.07	HQ
F6DB_bin.53	90.27	1.39	HQ
F6DE_bin.23	99.31	0	HQ
F6DE_bin.3	99.33	0	HQ
F6DE_bin.30	95.24	0.68	HQ
F6DE_bin.32	98.73	0.48	HQ
F6DE_bin.33	98.49	0	HQ
F6DE_bin.34	99.85	2.1	HQ
F6DE_bin.36	97.27	0	HQ
F6DE_bin.38	98.6	1.17	HQ
F6DE_bin.39	94.63	0	HQ
F6DE_bin.4	98.88	1.12	HQ
F6DE_bin.42	96.43	2.55	HQ
F6DE_bin.43	100	1.2	HQ
F6DE_bin.47	98.66	0	HQ
F6MY_bin.54	91.95	0	HQ
F6DE_bin.49	97.8	0.67	HQ
F6DE_bin.5	97.82	0.02	HQ
F6DE_bin.50	99.33	1.33	HQ
F68H_bin.78	99.67	0.12	HQ
F68J_bin.52	92.07	3.09	HQ
F68N_bin.13	99.85	1.36	HQ
F68V_bin.92	99.47	0.04	HQ
F6DE_bin.51	99.97	0.07	HQ

Table S2. MAGs quanlity

	CheckM_completeness	CheckM_contamination	Quality
F6MX_bin.52	92.17	3.86	HQ
F6DE_bin.54	93.29	2.01	HQ
F6DE_bin.6	98.63	0.68	HQ
F6DE_bin.67	91.53	1.87	HQ
F6DE_bin.68	91.72	0	HQ
F6DE_bin.70	96.33	1.18	HQ
F6DE_bin.72	91.98	2.48	HQ
F6DE_bin.74	93.19	2.13	HQ
F6DE_bin.78	99.74	0	HQ
F6DE_bin.8	92.09	3.64	HQ
F68D_bin.2	93.84	0.74	HQ
F68M_bin.62	91.84	0	HQ
F68V_bin.4	92.18	0.68	HQ
F6DE_bin.82	96.71	0.57	HQ
F6DE_bin.84	98.76	0.74	HQ
F6DE_bin.85	93.29	0.67	HQ
F6DE_bin.9	97.9	0.7	HQ
F68C_bin.18	97.85	0.57	HQ
F68F_bin.44	98.39	0	HQ
F68H_bin.21	91.32	0.81	HQ
F68K_bin.85	98.39	0	HQ
F68N_bin.53	97.85	0.54	HQ
F68R_bin.29	98.92	0	HQ
F68T_bin.29	97.85	0	HQ
F68U_bin.105	96.24	0	HQ
F6D7_bin.96	95.43	2.15	HQ
F6D8_bin.62	99.46	0	HQ
F6DA_bin.40	97.85	0	HQ
F6DB_bin.73	99.46	0	HQ
F6DE_bin.90	99.46	0	HQ
F6DG_bin.55	97.31	0	HQ
F6MX_bin.119	98.39	0	HQ
F6MY_bin.116	98.92	0	HQ
F6DG_bin.1	91.38	0.63	HQ
F6DG_bin.100	97.83	0.48	HQ
F6DG_bin.101	95.55	0.71	HQ
F6DG_bin.104	97.09	2.01	HQ
F6DG_bin.14	97.2	0.93	HQ
F6DG_bin.15	97.36	0	HQ
F6DG_bin.18	97.32	0.67	HQ
F6DG_bin.2	91.56	0.34	HQ
F6DG_bin.20	94.56	3.54	HQ
F6DG_bin.21	96.81	0	HQ
F6DG_bin.23	96.62	0.6	HQ
F6DG_bin.24	91.97	0	HQ
F6DG_bin.29	92.62	0	HQ
F6DG_bin.3	94.01	1.84	HQ

Table S2. MAGs quanlity

	CheckM_completeness	CheckM_contamination	Quality
F6DG_bin.30	95.76	2.11	HQ
F6DG_bin.32	94.53	1.02	HQ
F68P_bin.22	98.66	1.34	HQ
F6DG_bin.34	98.32	0	HQ
F6DG_bin.35	97.32	0	HQ
F6DG_bin.39	94.71	0.48	HQ
F6DG_bin.40	92.62	0.67	HQ
F6DG_bin.48	96.64	0	HQ
F6DG_bin.49	92.18	1.01	HQ
F68G_bin.71	94.3	0.06	HQ
F6DG_bin.50	97.99	0	HQ
F6DG_bin.51	98.66	0.67	HQ
F6DA_bin.31	97.34	0.34	HQ
F6DG_bin.52	98.66	0.15	HQ
F6DG_bin.56	93.62	0	HQ
F6DG_bin.63	98.49	0	HQ
F6DG_bin.64	99.33	0.67	HQ
F6DG_bin.66	98.14	1.55	HQ
F6DG_bin.67	93.47	2.73	HQ
F6DG_bin.70	93.06	3.02	HQ
F6DG_bin.72	91.31	0.11	HQ
F6DG_bin.73	90.06	0.58	HQ
F6DG_bin.75	97.87	0	HQ
F6DG_bin.76	95.86	1.16	HQ
F6DG_bin.77	97.44	1.63	HQ
F6DG_bin.79	91.55	2.5	HQ
F6D8_bin.32	92.49	2.16	HQ
F6DG_bin.80	98.38	0.48	HQ
F6DG_bin.83	96.91	0.19	HQ
F68G_bin.43	98.27	0.13	HQ
F68H_bin.5	97	3.79	HQ
F68T_bin.22	97.5	0.38	HQ
F6D7_bin.92	95.76	1.63	HQ
F6DB_bin.1	98.27	0.19	HQ
F6DG_bin.90	99.04	0	HQ
F6DG_bin.92	91.71	1.03	HQ
F6DG_bin.96	91.11	0.4	HQ
F68R_bin.119	92.84	3.42	HQ
F68W_bin.81	93.58	2.07	HQ
F6D9_bin.57	93.92	2.7	HQ
F6DH_bin.10	94.26	2.03	HQ
F6MY_bin.42	92.57	2.03	HQ
F68W_bin.24	90.02	0.67	HQ
F6DH_bin.101	95.7	0.34	HQ
F68W_bin.28	94.94	0	HQ
F6DH_bin.108	96.64	0	HQ
F68W_bin.42	94.54	0.45	HQ

Table S2. MAGs quanlity

	CheckM_completeness	CheckM_contamination	Quality
F6DH_bin.11	97.99	0.45	HQ
F68W_bin.68	96.42	0	HQ
F6DH_bin.115	96.42	0	HQ
F6DH_bin.116	94.91	0.67	HQ
F68W_bin.103	94.3	2.01	HQ
F6DH_bin.122	99.33	0	HQ
F68W_bin.46	94.92	0.74	HQ
F6DH_bin.16	95	0.74	HQ
F6DH_bin.17	92.23	0	HQ
F6DH_bin.2	98.66	0	HQ
F6DH_bin.21	99.33	0	HQ
F6DH_bin.25	91.08	3.17	HQ
F6DH_bin.29	91.55	0	HQ
F68W_bin.40	90.46	0	HQ
F6DH_bin.31	98.32	0.81	HQ
F6DH_bin.33	93.67	0.63	HQ
F68W_bin.109	93.96	1.34	HQ
F6DH_bin.35	95.3	1.34	HQ
F68U_bin.29	92.75	0.86	HQ
F68W_bin.10	94.96	0.37	HQ
F6DH_bin.36	97.26	0.22	HQ
F6DH_bin.43	97.49	2.42	HQ
F6DH_bin.52	93.45	2.81	HQ
F68W_bin.26	96.05	0.38	HQ
F6DH_bin.6	96.86	0.38	HQ
F6DH_bin.61	93.04	1.53	HQ
F68W_bin.66	95.83	1.88	HQ
F6DH_bin.66	95.83	1.88	HQ
F68W_bin.52	96.58	1.36	HQ
F6DH_bin.71	98.66	0	HQ
F6DH_bin.78	96.5	0.23	HQ
F6DH_bin.85	91.75	1.23	HQ
F6DH_bin.87	96.1	0	HQ
F6DH_bin.93	96.22	1.27	HQ
F68W_bin.69	97.32	0.67	HQ
F6DH_bin.95	95.97	0	HQ
F6MX_bin.1	92.34	0.03	HQ
F6MX_bin.100	93.33	0	HQ
F6MX_bin.101	97.58	1.69	HQ
F6MX_bin.106	91.38	3.63	HQ
F6MX_bin.107	92.23	0	HQ
F6MX_bin.108	93.68	2.84	HQ
F6MX_bin.111	91.94	0.7	HQ
F6MX_bin.115	96.51	0.05	HQ
F6MX_bin.116	92.46	1.34	HQ
F6DE_bin.37	97.96	1.36	HQ
F6MX_bin.117	97.96	1.36	HQ

Table S2. MAGs quanlity

	CheckM_completeness	CheckM_contamination	Quality
F6MX_bin.124	92.39	2.35	HQ
F6MX_bin.125	90.99	2.01	HQ
F6MX_bin.126	95.24	0	HQ
F6MX_bin.127	97.83	0	HQ
F6MX_bin.128	92.49	2.4	HQ
F6MX_bin.129	93.88	0.34	HQ
F6MX_bin.14	95.61	0.57	HQ
F6MX_bin.15	97.58	2.42	HQ
F6MX_bin.20	90.44	0	HQ
F6MX_bin.24	92.7	3.93	HQ
F6MX_bin.26	98.99	0.96	HQ
F6DA_bin.16	90.88	1.45	HQ
F6MX_bin.32	98.6	1.4	HQ
F6MX_bin.35	95.85	0	HQ
F6MX_bin.36	93.48	0	HQ
F6MX_bin.44	96.26	2.09	HQ
F6MX_bin.48	90.37	1.9	HQ
F6MX_bin.51	98.97	0.2	HQ
F68C_bin.48	98.2	1.2	HQ
F68U_bin.88	98.8	1.2	HQ
F68W_bin.14	98.2	1.2	HQ
F6D7_bin.7	98.2	1.2	HQ
F6DH_bin.89	99.4	1.4	HQ
F6MX_bin.53	99.2	1.2	HQ
F6MX_bin.54	93.86	0.29	HQ
F6MX_bin.56	98.32	0.67	HQ
F6D7_bin.24	94.55	0	HQ
F6MX_bin.58	95.36	0	HQ
F6MX_bin.60	94.74	0	HQ
F6MX_bin.67	95.97	0.54	HQ
F6MX_bin.68	96.42	0.22	HQ
F6MX_bin.70	90.81	1.34	HQ
F6MX_bin.71	92.6	1.34	HQ
F6MX_bin.74	94.04	1.56	HQ
F6MX_bin.79	96.74	1.77	HQ
F6MX_bin.84	94.55	1.35	HQ
F6MX_bin.91	96.77	1.88	HQ
F6MX_bin.92	96.98	0	HQ
F6MX_bin.96	95.95	0.48	HQ
F68J_bin.56	95.56	0.87	HQ
F68M_bin.63	95.53	0.63	HQ
F6MX_bin.98	95.53	0.63	HQ
F6MX_bin.99	90.3	3.23	HQ
F6MY_bin.103	94.18	0	HQ
F6MY_bin.110	92.6	1.34	HQ
F68K_bin.4	96.38	0	HQ
F6MY_bin.113	96.86	0	HQ

Table S2. MAGs quanlity

	CheckM_completeness	CheckM_contamination	Quality
F6MY_bin.114	90.6	0.67	HQ
F6MY_bin.119	91.28	0.67	HQ
F68H_bin.12	96.64	0.69	HQ
F6MY_bin.12	100	0.67	HQ
F6MY_bin.120	97.99	0	HQ
F6MY_bin.123	93.68	0.69	HQ
F6MY_bin.124	95.89	0	HQ
F6MY_bin.125	96.64	0	HQ
F6MY_bin.126	92.22	2.76	HQ
F6MY_bin.129	98.66	3.02	HQ
F6MY_bin.130	96.51	0.22	HQ
F6MY_bin.136	93.55	0	HQ
F6MY_bin.15	94.41	0.67	HQ
F68D_bin.24	98.71	1.02	HQ
F6MY_bin.25	99.62	0	HQ
F6MY_bin.28	96.41	0	HQ
F6MY_bin.36	94.41	0.93	HQ
F6MY_bin.40	94.63	1.34	HQ
F6MY_bin.47	98.39	0.81	HQ
F68K_bin.99	92.01	0	HQ
F6MY_bin.52	97.41	0	HQ
F6MY_bin.56	97.9	1.17	HQ
F6MY_bin.58	95.86	0.63	HQ
F6MY_bin.61	96.32	0.67	HQ
F6MY_bin.65	96.64	0.67	HQ
F6MY_bin.66	97.28	1.05	HQ
F6MY_bin.70	93.02	3.75	HQ
F6MY_bin.75	91.39	0	HQ
F6MY_bin.78	100	0.89	HQ
F6MY_bin.81	91.15	2.74	HQ
F6D7_bin.9	93.33	2.67	HQ
F6MY_bin.87	92.42	1.12	HQ
F6MY_bin.88	96.6	0	HQ
F6MY_bin.90	96.5	1.3	HQ
F68P_bin.44	98.01	1.6	HQ
F68T_bin.62	96.58	0.75	HQ
F6MY_bin.94	98.05	0.23	HQ
F6MY_bin.98	92.35	0.6	HQ
F68G_bin.24	90.8	1.89	HQ
F6MY_bin.99	98.11	1.42	HQ
F68C_bin.102	71.57	1.76	MQ
F68C_bin.103	58.33	1.84	MQ
F68C_bin.107	78.62	1.9	MQ
F6D8_bin.83	78.62	3.24	MQ
F6MX_bin.64	78.62	1.9	MQ
F68C_bin.108	66.71	0.84	MQ
F68C_bin.112	53.71	0.67	MQ

Table S2. MAGs quanlity

	CheckM_completeness	CheckM_contamination	Quality
F68C_bin.12	83.88	1.93	MQ
F68C_bin.14	73.04	0	MQ
F68C_bin.15	72.28	3.58	MQ
F68C_bin.17	87.33	2.68	MQ
F6D7_bin.102	63.16	0	MQ
F6DE_bin.1	75.5	3.97	MQ
F68F_bin.78	76.21	0	MQ
F68K_bin.70	76.9	1.72	MQ
F6DB_bin.41	58.1	3.45	MQ
F6MY_bin.95	52.07	0	MQ
F68C_bin.23	72.45	4.31	MQ
F68C_bin.24	69.12	0.86	MQ
F68C_bin.28	79.83	3.6	MQ
F68C_bin.29	78.77	3.03	MQ
F68C_bin.3	80.17	4.73	MQ
F68C_bin.30	73.22	2.64	MQ
F68C_bin.31	81.83	4.13	MQ
F68C_bin.34	71.64	4.97	MQ
F68C_bin.36	80	1.45	MQ
F68C_bin.37	83.44	2.22	MQ
F68C_bin.40	76.67	2.89	MQ
F68C_bin.45	89.26	0	MQ
F68J_bin.83	89.57	1.05	MQ
F68C_bin.50	67.55	4.09	MQ
F68H_bin.20	86.79	2.78	MQ
F6D7_bin.21	53.4	3.16	MQ
F68C_bin.55	69.3	3.51	MQ
F68C_bin.59	59.31	0	MQ
F68C_bin.6	81.56	0	MQ
F68C_bin.60	85.58	0.81	MQ
F68C_bin.63	81.88	0	MQ
F6D7_bin.100	79.19	0	MQ
F68C_bin.64	52.47	1.79	MQ
F68C_bin.68	61.38	0	MQ
F68C_bin.7	71.73	0	MQ
F68C_bin.73	68.83	2.78	MQ
F68C_bin.78	78.21	1.03	MQ
F68C_bin.8	69.7	1.17	MQ
F68C_bin.80	77.05	0	MQ
F68C_bin.81	82.59	0	MQ
F68C_bin.86	76.46	4.53	MQ
F68C_bin.87	82.89	0.67	MQ
F68C_bin.9	78.48	0	MQ
F68C_bin.90	69.6	4.5	MQ
F68C_bin.93	86.23	3.13	MQ
F68D_bin.10	86.88	2.63	MQ
F68D_bin.100	79.73	2.55	MQ

Table S2. MAGs quanlity

	CheckM_completeness	CheckM_contamination	Quality
F68D_bin.101	57.53	3.51	MQ
F68D_bin.102	74.29	2.52	MQ
F68M_bin.28	52.63	0	MQ
F68D_bin.107	89.17	3.32	MQ
F68D_bin.108	68.16	0	MQ
F68D_bin.11	89.04	4.57	MQ
F68D_bin.110	62.55	4.22	MQ
F68D_bin.112	67.05	2.25	MQ
F68D_bin.113	79.48	3.98	MQ
F68D_bin.13	85.85	1.34	MQ
F68D_bin.16	87.18	0.4	MQ
F68D_bin.17	88.75	1.81	MQ
F68D_bin.18	88.63	2.35	MQ
F6DE_bin.20	52.5	1.77	MQ
F68D_bin.20	83.63	2.38	MQ
F68D_bin.22	89.26	0.67	MQ
F68D_bin.25	85.19	0.67	MQ
F68D_bin.27	84.96	0.67	MQ
F68D_bin.31	64.75	3.3	MQ
F68D_bin.32	56.61	0.86	MQ
F68D_bin.35	52.61	2.59	MQ
F68D_bin.36	77	0	MQ
F68M_bin.9	82.36	1.78	MQ
F6DA_bin.23	54.55	1.34	MQ
F68D_bin.37	86.06	0.22	MQ
F68D_bin.38	69.83	1.72	MQ
F68D_bin.51	71.99	0	MQ
F68D_bin.54	82.27	2.13	MQ
F68D_bin.55	77.66	2.58	MQ
F68D_bin.58	84.54	1.68	MQ
F6DG_bin.8	85.65	4.16	MQ
F68D_bin.59	54.75	0	MQ
F68D_bin.61	85.04	4.14	MQ
F68D_bin.63	70.37	1.36	MQ
F68D_bin.64	58.5	0	MQ
F68D_bin.66	72.98	4.09	MQ
F68M_bin.20	79.3	4.11	MQ
F68D_bin.78	76.91	2.32	MQ
F68F_bin.116	56.82	2.03	MQ
F68D_bin.84	67.9	1.72	MQ
F68D_bin.85	52.54	2.68	MQ
F6D8_bin.85	86.55	2.78	MQ
F68D_bin.98	84.97	1.72	MQ
F68E_bin.1	87.64	4.33	MQ
F68C_bin.72	66.58	2.24	MQ
F68E_bin.15	52.39	0	MQ
F68E_bin.16	65.86	1.72	MQ

Table S2. MAGs quanlity

	CheckM_completeness	CheckM_contamination	Quality
F68E_bin.2	89.86	0.63	MQ
F68E_bin.24	82.22	3.41	MQ
F68E_bin.25	52.95	0	MQ
F68E_bin.29	82.79	2.85	MQ
F68E_bin.35	84.54	0	MQ
F68E_bin.37	66.59	3.51	MQ
F68E_bin.40	67.41	0.86	MQ
F6DA_bin.94	88.88	2.72	MQ
F6MX_bin.102	89.65	0.19	MQ
F68E_bin.48	88.14	0.96	MQ
F68E_bin.7	70	4.32	MQ
F68M_bin.5	52.37	0.12	MQ
F68N_bin.24	89.41	1.95	MQ
F68F_bin.104	80.44	0.83	MQ
F68F_bin.109	65.75	0	MQ
F68F_bin.110	78.01	4.26	MQ
F68F_bin.113	54.73	1.06	MQ
F68F_bin.115	57.18	2.63	MQ
F68F_bin.13	89.77	4.83	MQ
F68F_bin.14	56.14	1.75	MQ
F68F_bin.17	75.59	3.24	MQ
F68F_bin.20	55.68	0.67	MQ
F68F_bin.22	81.8	3.4	MQ
F68F_bin.26	76.14	3.02	MQ
F68F_bin.27	86.24	2.22	MQ
F6D7_bin.19	77.4	1.4	MQ
F68F_bin.29	52.59	0	MQ
F68F_bin.3	69.98	2.85	MQ
F68R_bin.32	61.32	4.66	MQ
F68F_bin.31	71.48	0	MQ
F68F_bin.32	81.76	4.24	MQ
F68R_bin.107	81.29	1.9	MQ
F68V_bin.89	85.94	1.59	MQ
F68K_bin.48	73.05	1.41	MQ
F68F_bin.36	52.19	3.16	MQ
F68H_bin.51	82.08	0.41	MQ
F68K_bin.16	88.5	0.71	MQ
F6DB_bin.77	88.25	0.24	MQ
F68F_bin.40	58.04	0	MQ
F6MX_bin.65	60.4	0	MQ
F68F_bin.42	61.32	0.48	MQ
F68F_bin.47	72.87	1.27	MQ
F68F_bin.49	80.98	2.37	MQ
F68F_bin.53	86.58	0.67	MQ
F68F_bin.54	57.14	0	MQ
F68F_bin.56	66.11	0	MQ
F68F_bin.63	66.07	1.79	MQ

Table S2. MAGs quanlity

	CheckM_completeness	CheckM_contamination	Quality
F68F_bin.66	86.81	3.22	MQ
F68F_bin.67	85.68	0	MQ
F68F_bin.75	62.34	0	MQ
F68F_bin.76	89.54	3.65	MQ
F68F_bin.79	59.94	4.17	MQ
F68F_bin.80	78.34	3.09	MQ
F68F_bin.86	76.12	2.46	MQ
F68F_bin.87	66.26	2.07	MQ
F68F_bin.88	88.36	3.01	MQ
F68V_bin.87	84.43	3.06	MQ
F6DE_bin.88	80.37	0	MQ
F68F_bin.92	88.81	4.44	MQ
F68F_bin.93	82.22	1.81	MQ
F68F_bin.94	56.03	1.72	MQ
F68F_bin.95	56.55	1.72	MQ
F68F_bin.96	67.73	1.08	MQ
F68F_bin.97	84.27	0.03	MQ
F68G_bin.1	56.31	1.75	MQ
F68G_bin.11	62.67	2.15	MQ
F68P_bin.51	69.75	0	MQ
F68G_bin.15	89.04	0	MQ
F68G_bin.16	85.57	0.67	MQ
F68G_bin.17	77.3	1.93	MQ
F68G_bin.18	89.86	4.08	MQ
F68D_bin.15	88.39	1.34	MQ
F68G_bin.2	85.32	0.67	MQ
F68G_bin.23	86.26	2.01	MQ
F68G_bin.25	61.85	3.74	MQ
F68G_bin.3	66.75	1	MQ
F68G_bin.32	62.73	0.37	MQ
F68G_bin.34	85.32	0.79	MQ
F68G_bin.38	78.84	0.37	MQ
F68D_bin.96	50.73	0	MQ
F68R_bin.120	85.59	2.84	MQ
F68E_bin.49	51.96	3.51	MQ
F68G_bin.42	88.61	0	MQ
F68G_bin.44	81.38	3.01	MQ
F68U_bin.111	59.44	4.39	MQ
F68G_bin.58	50.16	0	MQ
F68G_bin.59	77.16	0.79	MQ
F68M_bin.83	89.89	3.38	MQ
F68G_bin.60	86.3	1.8	MQ
F68H_bin.81	56.68	0.36	MQ
F68G_bin.64	71.75	0.62	MQ
F68G_bin.70	87.76	1.36	MQ
F68G_bin.75	87.91	0.22	MQ
F68G_bin.78	87.27	3.16	MQ

Table S2. MAGs quanlity

	CheckM_completeness	CheckM_contamination	Quality
F68G_bin.81	74.6	1.79	MQ
F68G_bin.86	74.93	2.4	MQ
F68G_bin.89	51.99	3.55	MQ
F68G_bin.9	83.43	2.33	MQ
F68C_bin.85	56.9	0	MQ
F68D_bin.95	82.76	1.72	MQ
F68F_bin.72	83.71	0.94	MQ
F68M_bin.81	81.87	2.16	MQ
F68R_bin.97	64.32	0.56	MQ
F68T_bin.39	65.52	0	MQ
F68V_bin.99	82.76	1.72	MQ
F68W_bin.97	64.66	0.86	MQ
F6D7_bin.71	51.72	2.59	MQ
F6D8_bin.31	89.84	3.76	MQ
F6DE_bin.61	67.24	0	MQ
F68H_bin.17	57.81	0.88	MQ
F68H_bin.18	80.63	1.19	MQ
F68H_bin.26	84.91	2.35	MQ
F68H_bin.27	78.04	1.79	MQ
F68H_bin.32	69.87	0.73	MQ
F68H_bin.38	82.42	0.67	MQ
F68H_bin.41	50.67	1.75	MQ
F68H_bin.42	83.6	2.17	MQ
F6D9_bin.27	50	3.51	MQ
F68H_bin.45	86.95	0.57	MQ
F68H_bin.58	80.86	4.31	MQ
F68H_bin.59	78.97	0	MQ
F68H_bin.61	78.06	0.67	MQ
F68H_bin.62	63.81	1.1	MQ
F68H_bin.64	65.25	4.11	MQ
F68H_bin.68	58.62	0	MQ
F68H_bin.71	89.04	1.48	MQ
F68H_bin.75	86.97	0.34	MQ
F68H_bin.76	57.38	0	MQ
F68H_bin.77	67.99	1.42	MQ
F68H_bin.82	58.66	2.66	MQ
F68H_bin.9	55.26	1.75	MQ
F68J_bin.12	88.79	4.48	MQ
F68J_bin.13	82.59	3.11	MQ
F68J_bin.15	83	3.91	MQ
F68J_bin.17	73.6	1.51	MQ
F68J_bin.18	74.55	0	MQ
F6D9_bin.83	82.48	2.66	MQ
F68J_bin.24	77.93	1.79	MQ
F68C_bin.65	56.74	2.36	MQ
F68J_bin.26	80.16	2.93	MQ
F6D7_bin.23	74.9	3.76	MQ

Table S2. MAGs quanlity

	CheckM_completeness	CheckM_contamination	Quality
F68J_bin.28	67.86	1.79	MQ
F68J_bin.30	79.73	0.88	MQ
F6DE_bin.80	81.66	1.48	MQ
F68J_bin.31	81.76	2.02	MQ
F68J_bin.36	82.91	1.45	MQ
F68J_bin.38	85.22	1.46	MQ
F68J_bin.4	89.82	0.71	MQ
F68J_bin.40	54.9	0	MQ
F6DH_bin.112	89.41	2.85	MQ
F68J_bin.47	88.48	0.73	MQ
F68J_bin.48	66.79	0.67	MQ
F68J_bin.50	51.57	2.9	MQ
F68J_bin.54	82.34	1.81	MQ
F68J_bin.58	53.75	0	MQ
F6DB_bin.52	54.81	0	MQ
F68J_bin.60	58.93	1.72	MQ
F68J_bin.63	89.4	4.13	MQ
F68J_bin.64	89.44	0.71	MQ
F68J_bin.66	83.3	3.19	MQ
F68G_bin.84	51.88	1.72	MQ
F68T_bin.33	56.22	0.94	MQ
F68J_bin.68	57.37	0	MQ
F68E_bin.8	75.24	1.16	MQ
F68H_bin.53	82.75	2.92	MQ
F68P_bin.49	88.06	0.58	MQ
F6D9_bin.20	51.72	0	MQ
F6DG_bin.99	75.57	1.38	MQ
F6MY_bin.74	76.02	0	MQ
F68J_bin.71	74.45	0	MQ
F68R_bin.16	88.9	1.34	MQ
F68J_bin.73	65.23	1.75	MQ
F68J_bin.75	55.23	0.02	MQ
F68J_bin.77	79.81	1.91	MQ
F68J_bin.78	73.67	0.49	MQ
F68J_bin.8	82.29	0.85	MQ
F68J_bin.80	70.09	0.32	MQ
F68J_bin.82	86.67	2.14	MQ
F68J_bin.84	55.16	1.72	MQ
F68J_bin.85	69.48	2.64	MQ
F68J_bin.86	55.46	0.56	MQ
F68J_bin.87	62.47	0.06	MQ
F68K_bin.100	85.69	2.36	MQ
F68K_bin.107	69.8	4.03	MQ
F68K_bin.109	63.75	1.56	MQ
F68J_bin.46	56.14	0	MQ
F68K_bin.11	78.95	0	MQ
F68R_bin.14	69.96	1.23	MQ

Table S2. MAGs quanlity

	CheckM_completeness	CheckM_contamination	Quality
F68K_bin.110	67.86	0	MQ
F68K_bin.112	64.19	2.45	MQ
F68K_bin.113	62.14	0.94	MQ
F68K_bin.12	56.03	3.45	MQ
F68K_bin.17	89.55	0.38	MQ
F68K_bin.19	66.89	2.58	MQ
F68K_bin.20	88.89	0.24	MQ
F68K_bin.22	72.24	0	MQ
F68K_bin.23	62.07	0	MQ
F68K_bin.29	53.43	0	MQ
F68R_bin.95	87.73	3.76	MQ
F68V_bin.119	57.35	1.08	MQ
F6MX_bin.110	81.83	0.99	MQ
F68K_bin.30	82.65	2.85	MQ
F68K_bin.31	88.02	1.01	MQ
F68K_bin.40	80.59	0.11	MQ
F6DB_bin.89	53.57	1.44	MQ
F6DG_bin.12	89.34	0.88	MQ
F68K_bin.45	84.85	2.91	MQ
F6D7_bin.57	73.28	1.72	MQ
F68K_bin.5	73.29	0.68	MQ
F68K_bin.53	64.62	1.75	MQ
F68K_bin.57	54.39	1.75	MQ
F68K_bin.59	84.78	1.34	MQ
F68K_bin.6	85.23	0	MQ
F68K_bin.62	84.53	0.5	MQ
F68K_bin.65	67.68	1.18	MQ
F68K_bin.68	55.6	3.7	MQ
F68U_bin.85	57.89	0	MQ
F68K_bin.73	62.07	1.42	MQ
F68K_bin.76	83.96	2.07	MQ
F68P_bin.54	69.58	2.32	MQ
F68K_bin.80	78.25	0.64	MQ
F68K_bin.81	53.68	0	MQ
F68K_bin.84	85.45	2.67	MQ
F68K_bin.86	54.78	1.72	MQ
F68K_bin.89	79.81	0.67	MQ
F68K_bin.90	76.29	0.84	MQ
F68K_bin.97	50.16	1.75	MQ
F68M_bin.16	88.46	0	MQ
F68M_bin.18	54.48	3.79	MQ
F68M_bin.19	86.46	1.59	MQ
F6MX_bin.57	50.94	0	MQ
F68M_bin.23	68.21	3.26	MQ
F68F_bin.11	85.48	3.94	MQ
F68N_bin.37	54.31	2.59	MQ
F68R_bin.38	71.55	3.45	MQ

Table S2. MAGs quanlity

	CheckM_completeness	CheckM_contamination	Quality
F6D7_bin.39	88.94	3.22	MQ
F68M_bin.25	65.96	0.27	MQ
F6DB_bin.60	70.67	1.68	MQ
F6MX_bin.109	59.64	3.61	MQ
F68M_bin.29	70.6	2.72	MQ
F6MX_bin.76	72.01	0.67	MQ
F6MX_bin.55	77.34	0.68	MQ
F68M_bin.4	56.24	0.85	MQ
F68D_bin.60	86.09	1.74	MQ
F68M_bin.43	52.8	1.22	MQ
F68M_bin.44	83.43	3.23	MQ
F68M_bin.48	68.42	0.38	MQ
F68M_bin.52	85.21	0.67	MQ
F68M_bin.59	58.86	1.16	MQ
F6MX_bin.41	72.86	2.94	MQ
F68H_bin.35	86.29	2.82	MQ
F68M_bin.71	61.21	0.86	MQ
F68C_bin.46	67.9	0.37	MQ
F68M_bin.74	67.6	2.68	MQ
F68M_bin.77	84.37	0.83	MQ
F68M_bin.79	75.27	0	MQ
F68M_bin.8	71.19	3.44	MQ
F68D_bin.29	54.63	2.63	MQ
F68M_bin.87	52.32	1.59	MQ
F68M_bin.88	79.62	2.09	MQ
F68M_bin.89	52.14	0.83	MQ
F68N_bin.1	76.61	0.12	MQ
F68N_bin.10	79.53	1.34	MQ
F68N_bin.11	66.18	2.97	MQ
F68N_bin.20	82.46	0.29	MQ
F68W_bin.22	74.55	1.35	MQ
F6DH_bin.19	69.58	3.03	MQ
F68N_bin.30	73.79	1.72	MQ
F68N_bin.40	74.94	3.19	MQ
F68N_bin.42	57.5	1.79	MQ
F68N_bin.45	75.24	0.68	MQ
F68N_bin.46	82.58	0	MQ
F68N_bin.47	80.08	1.7	MQ
F68N_bin.51	61.36	0	MQ
F68N_bin.62	60.51	0.63	MQ
F68N_bin.71	55.32	0.58	MQ
F68N_bin.74	79.87	1.96	MQ
F68N_bin.77	88.93	0	MQ
F68N_bin.8	86.65	0.82	MQ
F68P_bin.1	80.33	0.52	MQ
F68U_bin.84	89.9	3.46	MQ
F68P_bin.12	76.85	1.59	MQ

Table S2. MAGs quanlity

	CheckM_completeness	CheckM_contamination	Quality
F68P_bin.13	53.61	0	MQ
F68P_bin.15	69.86	0.32	MQ
F68P_bin.16	69.72	0.67	MQ
F68P_bin.17	80.54	0	MQ
F68R_bin.46	54.36	0	MQ
F68V_bin.124	51.23	0	MQ
F68P_bin.25	88.11	0.38	MQ
F68P_bin.26	82.49	0.67	MQ
F68P_bin.27	53.02	2.12	MQ
F68P_bin.28	77.84	0.9	MQ
F68P_bin.29	79.56	2.87	MQ
F68P_bin.31	79.45	0.63	MQ
F68P_bin.33	73.01	3.02	MQ
F68P_bin.35	62.34	0.37	MQ
F68P_bin.37	76.21	2.07	MQ
F68P_bin.4	87.92	3.02	MQ
F68P_bin.42	85.06	1.22	MQ
F68G_bin.83	89.98	0	MQ
F68P_bin.56	76.29	2.23	MQ
F68P_bin.57	54.31	1.72	MQ
F68P_bin.58	51.69	0	MQ
F68P_bin.59	78.11	0.74	MQ
F68P_bin.61	88.79	0.73	MQ
F68P_bin.8	82.57	1.25	MQ
F68R_bin.100	53.62	4.96	MQ
F68R_bin.101	64.04	0	MQ
F68R_bin.106	71.32	0	MQ
F68C_bin.39	78.12	0.89	MQ
F68R_bin.108	78.12	0.89	MQ
F6MX_bin.118	78.12	1.57	MQ
F68R_bin.112	75.52	3.57	MQ
F68R_bin.113	59.59	0.88	MQ
F68R_bin.114	58.36	1.79	MQ
F68R_bin.115	77.29	3.47	MQ
F68R_bin.116	74.52	3.13	MQ
F68R_bin.117	88.18	0.51	MQ
F68R_bin.122	72.03	0.37	MQ
F68R_bin.123	75.59	0	MQ
F68R_bin.125	86.34	1.51	MQ
F68R_bin.127	88.38	4.25	MQ
F68R_bin.128	66.52	1.69	MQ
F68R_bin.13	75.62	2.96	MQ
F68R_bin.2	88.59	0.67	MQ
F68R_bin.21	77.93	0.27	MQ
F68R_bin.25	68.45	2.59	MQ
F68R_bin.27	89.08	0.97	MQ
F68U_bin.53	81.24	0.22	MQ

Table S2. MAGs quanlity

	CheckM_completeness	CheckM_contamination	Quality
F68R_bin.30	72.44	1.95	MQ
F68R_bin.39	89.04	0	MQ
F68R_bin.41	84.27	1.6	MQ
F68R_bin.44	68.93	2.35	MQ
F68R_bin.49	65.31	0	MQ
F68R_bin.53	63.67	1.75	MQ
F68R_bin.55	66.26	0.7	MQ
F68R_bin.58	53.68	0	MQ
F68R_bin.60	86.25	0.81	MQ
F68D_bin.1	88.69	1.9	MQ
F68R_bin.63	79.3	2.98	MQ
F68R_bin.67	56.41	1.5	MQ
F68R_bin.68	59.48	3.45	MQ
F68R_bin.70	69.19	0	MQ
F6MY_bin.108	50.08	0	MQ
F68G_bin.36	83.54	0.63	MQ
F6D8_bin.67	71.39	1.75	MQ
F6D9_bin.70	59.65	0	MQ
F68R_bin.8	86.4	0.47	MQ
F6D7_bin.101	81.03	2.59	MQ
F6DB_bin.20	53.1	1.72	MQ
F6DE_bin.48	80.43	2.93	MQ
F6MY_bin.59	58.39	0	MQ
F68R_bin.82	89.04	3.1	MQ
F68R_bin.83	65.52	1.75	MQ
F68R_bin.84	57.74	0.08	MQ
F68R_bin.88	81.37	3.58	MQ
F68R_bin.89	53.18	4.76	MQ
F68R_bin.9	54.39	0	MQ
F68R_bin.91	59.49	0.94	MQ
F68R_bin.93	50.18	0	MQ
F68R_bin.99	80.7	0	MQ
F68T_bin.1	86.07	0.89	MQ
F68T_bin.10	86.59	0.35	MQ
F68T_bin.100	81.57	2.54	MQ
F68T_bin.104	66.45	1.75	MQ
F68T_bin.105	58.52	3.14	MQ
F68T_bin.12	82.59	2.18	MQ
F68T_bin.13	84.56	1.01	MQ
F68T_bin.17	50.34	3.06	MQ
F68R_bin.57	88.07	2.48	MQ
F68T_bin.19	86.82	1.24	MQ
F68T_bin.2	75.07	3.22	MQ
F68T_bin.23	73.83	0	MQ
F68T_bin.24	58.83	0.9	MQ
F68T_bin.25	80.04	2.46	MQ
F68T_bin.34	68.77	0.16	MQ

Table S2. MAGs quanlity

	CheckM_completeness	CheckM_contamination	Quality
F68T_bin.36	89.37	0	MQ
F68T_bin.47	88.76	1.69	MQ
F68K_bin.111	71.6	0.63	MQ
F68T_bin.54	78.91	0	MQ
F6DB_bin.8	70.25	0.63	MQ
F68T_bin.55	76.58	2.79	MQ
F68T_bin.56	79.31	1.92	MQ
F68T_bin.57	87.87	2.74	MQ
F68T_bin.58	87.75	1.23	MQ
F68T_bin.6	84.8	2.01	MQ
F68T_bin.61	83.23	1.29	MQ
F68T_bin.65	77.12	1.68	MQ
F68N_bin.59	81.38	0	MQ
F6D7_bin.53	61.78	3.45	MQ
F6DB_bin.26	60.78	3.09	MQ
F68T_bin.71	81.7	1.21	MQ
F68T_bin.72	50.05	3.45	MQ
F68T_bin.75	75.83	0.67	MQ
F68T_bin.83	73.32	0.47	MQ
F68T_bin.87	55.93	0.19	MQ
F68T_bin.89	74.37	1.21	MQ
F68R_bin.22	62.67	1.03	MQ
F68T_bin.91	61.4	0	MQ
F68T_bin.92	76.67	0	MQ
F68T_bin.94	73.48	2.78	MQ
F68T_bin.95	52.64	0.5	MQ
F68T_bin.98	88.08	0.84	MQ
F68T_bin.99	61.4	0	MQ
F68W_bin.70	77.9	3.45	MQ
F6DH_bin.27	89.81	2.79	MQ
F68U_bin.104	88.5	4.36	MQ
F68W_bin.20	89.79	1.25	MQ
F68U_bin.108	82.73	3.82	MQ
F68U_bin.112	57.72	3.86	MQ
F68U_bin.113	61.8	4.77	MQ
F68U_bin.13	63.04	0.94	MQ
F68U_bin.14	68.99	2.49	MQ
F68U_bin.15	89.51	0.63	MQ
F68U_bin.16	87.58	0.67	MQ
F68W_bin.73	57.02	0	MQ
F6DH_bin.4	85.41	2.37	MQ
F68U_bin.2	60.67	0	MQ
F68U_bin.22	67.25	1.51	MQ
F68K_bin.43	85.56	2.74	MQ
F68P_bin.52	89.83	1.03	MQ
F68R_bin.48	68.3	2.44	MQ
F68U_bin.27	83.07	2.58	MQ

Table S2. MAGs quanlity

	CheckM_completeness	CheckM_contamination	Quality
F68U_bin.28	79.2	4.65	MQ
F68D_bin.43	74.07	0.48	MQ
F68W_bin.4	83.15	2.24	MQ
F68U_bin.31	78.8	0.63	MQ
F68U_bin.32	63.33	2.33	MQ
F68U_bin.33	57.89	0	MQ
F68U_bin.43	70.67	1.75	MQ
F6DH_bin.34	77.4	3.54	MQ
F68U_bin.44	86.57	0	MQ
F68U_bin.45	80.01	1.7	MQ
F68W_bin.35	58.57	3.76	MQ
F6DH_bin.86	59.23	3.69	MQ
F68U_bin.50	81.17	0.75	MQ
F68U_bin.52	59.65	0	MQ
F68U_bin.57	60.45	2.9	MQ
F68U_bin.58	71.53	1.75	MQ
F6DH_bin.100	74.66	4.74	MQ
F6DH_bin.47	88.88	1.96	MQ
F68U_bin.63	87.26	1.73	MQ
F68W_bin.63	80.89	2.78	MQ
F68U_bin.64	81.31	4.46	MQ
F68U_bin.67	80.02	1.01	MQ
F68U_bin.72	59.29	0	MQ
F68U_bin.74	74.46	2.57	MQ
F68U_bin.76	81.72	0.29	MQ
F68U_bin.77	69.48	0	MQ
F68W_bin.92	56.9	3.45	MQ
F6DH_bin.42	60.5	1.72	MQ
F68C_bin.100	89.24	2.93	MQ
F68T_bin.103	71.98	1.04	MQ
F68D_bin.106	77.91	1.24	MQ
F68U_bin.83	80.47	0.79	MQ
F68U_bin.86	68.38	2.3	MQ
F68U_bin.89	80.23	0.54	MQ
F68U_bin.90	76.49	1.01	MQ
F68U_bin.93	84.57	4.05	MQ
F68W_bin.55	62.53	3.02	MQ
F68U_bin.99	73.1	0.4	MQ
F68V_bin.100	70.75	0	MQ
F68V_bin.101	84	4	MQ
F68V_bin.102	67.53	2.96	MQ
F68V_bin.103	79.46	0.67	MQ
F68V_bin.104	51.52	0	MQ
F68V_bin.105	76.88	3.23	MQ
F68M_bin.54	76.4	1.23	MQ
F68V_bin.106	89.89	2.15	MQ
F6DE_bin.7	79.83	0.22	MQ

Table S2. MAGs quanlity

	CheckM_completeness	CheckM_contamination	Quality
F6MY_bin.27	68.94	0.67	MQ
F68M_bin.69	86.21	1.88	MQ
F68V_bin.111	73.42	3.36	MQ
F68V_bin.114	88.93	0.67	MQ
F68E_bin.9	57.07	0	MQ
F68N_bin.36	88.68	1.67	MQ
F68P_bin.3	68.26	0	MQ
F6D7_bin.34	88.83	0.58	MQ
F6MY_bin.51	86.73	1.74	MQ
F68V_bin.12	75.21	0.4	MQ
F68V_bin.121	55.59	1.23	MQ
F68V_bin.123	50.7	2.24	MQ
F68V_bin.125	52.93	0	MQ
F68V_bin.16	81.98	3.69	MQ
F68V_bin.17	83.76	0	MQ
F68V_bin.19	61.97	3.51	MQ
F68V_bin.21	82.41	1.21	MQ
F68V_bin.24	81.65	1.34	MQ
F68V_bin.25	74.27	1.27	MQ
F68D_bin.34	54.02	0	MQ
F68V_bin.29	72.22	1.17	MQ
F68K_bin.67	80.52	2.91	MQ
F68V_bin.3	87.45	1.48	MQ
F6DG_bin.89	81.37	4.81	MQ
F6MY_bin.4	89.26	1.34	MQ
F68V_bin.32	74.54	3.15	MQ
F68V_bin.33	77.04	2.01	MQ
F68V_bin.36	89.93	1.34	MQ
F68V_bin.40	56.79	1.72	MQ
F6DE_bin.94	54.35	1.9	MQ
F68V_bin.44	63.79	0.86	MQ
F68V_bin.46	84.99	2.25	MQ
F68V_bin.48	80.31	1.01	MQ
F68V_bin.5	78.06	0	MQ
F68V_bin.51	57.05	1.72	MQ
F6MY_bin.97	62.28	3.38	MQ
F68M_bin.80	71.64	0.88	MQ
F68V_bin.55	81.81	4.26	MQ
F68V_bin.56	78.73	1.34	MQ
F68E_bin.46	78.79	0	MQ
F68K_bin.102	67.45	2.42	MQ
F6D9_bin.55	63.2	0	MQ
F6MX_bin.114	56.9	0	MQ
F68V_bin.67	52.72	3.51	MQ
F68V_bin.69	84.18	1.06	MQ
F68V_bin.7	85.09	0.31	MQ
F68V_bin.70	53.33	1.13	MQ

Table S2. MAGs quanlity

	CheckM_completeness	CheckM_contamination	Quality
F68V_bin.75	89.46	0.42	MQ
F6MX_bin.83	60.39	2.82	MQ
F68V_bin.77	89.26	1.4	MQ
F68V_bin.79	84.27	4.33	MQ
F68V_bin.80	71.26	0.53	MQ
F68V_bin.81	55.35	2.9	MQ
F68V_bin.82	87.03	1.01	MQ
F68V_bin.9	56.2	3.02	MQ
F68V_bin.91	88.44	1.63	MQ
F68T_bin.97	84.8	0	MQ
F68V_bin.94	62.43	1.53	MQ
F68V_bin.96	71.77	2.69	MQ
F68V_bin.97	89.61	3.13	MQ
F68V_bin.98	68.34	2.01	MQ
F6MY_bin.72	61.09	2.46	MQ
F68U_bin.26	78.71	0	MQ
F68W_bin.110	68.63	0.51	MQ
F6DH_bin.59	70.28	2.63	MQ
F6DH_bin.12	86.96	2.68	MQ
F68W_bin.16	80.44	4.33	MQ
F68W_bin.19	86.8	0	MQ
F6DH_bin.67	81.71	0	MQ
F68W_bin.2	83.86	0.3	MQ
F6DH_bin.119	62.23	3.45	MQ
F68W_bin.23	87.25	0.67	MQ
F6DH_bin.55	66.11	0	MQ
F68U_bin.109	68.84	0.37	MQ
F68W_bin.25	84.76	1.11	MQ
F68W_bin.31	85.49	1.87	MQ
F6DH_bin.45	89.26	4.7	MQ
F6DH_bin.99	89.37	2.43	MQ
F68W_bin.41	56.4	3.36	MQ
F68W_bin.44	83.9	4.99	MQ
F68W_bin.45	88.26	0.67	MQ
F6DH_bin.91	71.48	0.67	MQ
F68W_bin.48	59.48	0.86	MQ
F6DH_bin.62	52.83	1.34	MQ
F68W_bin.51	83.82	0.17	MQ
F6DH_bin.127	82.51	0	MQ
F68W_bin.56	75.48	1.55	MQ
F6DH_bin.28	78.9	2.49	MQ
F68W_bin.58	55.33	0	MQ
F68W_bin.59	70.54	0	MQ
F6DH_bin.20	57.31	0	MQ
F68U_bin.12	70.39	0	MQ
F6DH_bin.1	74.14	3.45	MQ
F68W_bin.61	81.97	1.62	MQ

Table S2. MAGs quanlity

	CheckM_completeness	CheckM_contamination	Quality
F68W_bin.65	55.6	3.61	MQ
F6DH_bin.24	51.9	4.75	MQ
F68U_bin.10	85.23	0	MQ
F68W_bin.67	89.93	0	MQ
F6DH_bin.8	86.58	0	MQ
F68W_bin.71	53.45	1.72	MQ
F68W_bin.75	52.79	0.81	MQ
F68W_bin.79	74.25	0.89	MQ
F6DH_bin.41	78.39	4.08	MQ
F6DH_bin.56	80.92	0	MQ
F68W_bin.82	89.26	0.67	MQ
F6DH_bin.104	89.26	1.01	MQ
F68W_bin.83	79.62	3.95	MQ
F68W_bin.85	85.76	1.43	MQ
F6DH_bin.38	76.49	2.38	MQ
F6DH_bin.73	56.52	0	MQ
F68W_bin.89	73.82	2.01	MQ
F6DH_bin.94	82.47	0.82	MQ
F68U_bin.73	89.19	0.68	MQ
F68W_bin.93	89.19	0.68	MQ
F6DH_bin.58	89.19	0.68	MQ
F68W_bin.95	86.9	3.15	MQ
F6DH_bin.97	80.36	4.49	MQ
F6D7_bin.103	87.08	1.12	MQ
F6D7_bin.104	78.87	3.75	MQ
F6D7_bin.105	80.02	2.01	MQ
F6D7_bin.109	56.67	3.37	MQ
F68V_bin.39	88.79	0.02	MQ
F6D7_bin.18	66.81	1.48	MQ
F68H_bin.8	87.53	4.05	MQ
F6D7_bin.29	89.26	1.01	MQ
F68M_bin.14	88.23	4.05	MQ
F6MX_bin.25	72.14	1.05	MQ
F6D7_bin.36	61.05	0	MQ
F6D7_bin.37	85.52	2.62	MQ
F6D7_bin.41	70.42	0.77	MQ
F6D7_bin.45	87.38	2.15	MQ
F68C_bin.96	87.4	2.53	MQ
F6D7_bin.49	79.03	0.05	MQ
F6D7_bin.50	89.6	1.68	MQ
F6MY_bin.80	53.25	0.56	MQ
F6D7_bin.60	75	1.25	MQ
F6D7_bin.61	80.24	0.03	MQ
F6D7_bin.62	67.89	3.51	MQ
F6D7_bin.63	81.31	2.57	MQ
F6D7_bin.66	88.37	1.42	MQ
F6D7_bin.68	59.26	1.13	MQ

Table S2. MAGs quanlity

	CheckM_completeness	CheckM_contamination	Quality
F6D7_bin.69	68.59	0	MQ
F6D7_bin.70	79.08	2.35	MQ
F68K_bin.93	72.93	0.4	MQ
F6D7_bin.76	85.01	0.34	MQ
F6D7_bin.78	81.34	2.49	MQ
F6D7_bin.79	86.52	0.34	MQ
F6D7_bin.8	64.83	1.34	MQ
F6D7_bin.80	50.33	3.45	MQ
F6D7_bin.85	88.26	0	MQ
F6D7_bin.88	55.49	1.72	MQ
F6D7_bin.89	57.27	0	MQ
F68C_bin.58	67.02	0.12	MQ
F6D7_bin.93	85.23	1.04	MQ
F6D8_bin.1	78.64	0	MQ
F6D8_bin.10	85.2	2.18	MQ
F6D8_bin.12	87.3	0.67	MQ
F6D8_bin.15	71.17	0	MQ
F6D8_bin.2	74.34	1.72	MQ
F6D8_bin.23	57.14	0	MQ
F6D8_bin.24	77.84	2.4	MQ
F6D8_bin.34	82.66	0.67	MQ
F6D8_bin.36	86.34	1.85	MQ
F68D_bin.87	74.14	0	MQ
F6D8_bin.39	79.4	1.12	MQ
F6D8_bin.4	89.26	0.17	MQ
F6DA_bin.83	85.44	2.88	MQ
F6D8_bin.48	87.92	1.48	MQ
F68M_bin.21	88.53	3.91	MQ
F68V_bin.88	60.34	0	MQ
F6DE_bin.26	51.72	0	MQ
F6DB_bin.49	86.11	0	MQ
F6D9_bin.44	72.68	2.57	MQ
F6D8_bin.56	64.17	0.12	MQ
F68R_bin.26	80.28	0.67	MQ
F6D8_bin.59	88.12	0.09	MQ
F6DG_bin.53	84.56	4.36	MQ
F6D8_bin.65	80.7	0	MQ
F6D8_bin.69	53.4	1.61	MQ
F6D8_bin.70	88.11	0	MQ
F6D8_bin.71	81.9	1.85	MQ
F6D8_bin.72	83.82	2.68	MQ
F6D8_bin.73	68.1	1.89	MQ
F6D8_bin.75	79.19	0.34	MQ
F6D8_bin.77	54.1	3.33	MQ
F6D8_bin.78	52.19	4.35	MQ
F6D8_bin.79	80.27	2.59	MQ
F6D8_bin.80	75.72	0.95	MQ

Table S2. MAGs quanlity

	CheckM_completeness	CheckM_contamination	Quality
F6D8_bin.94	56.79	1.68	MQ
F6D8_bin.95	59.87	1.88	MQ
F6D8_bin.98	58.32	1.85	MQ
F6D9_bin.21	70.17	3.45	MQ
F6D9_bin.24	89.92	0.18	MQ
F6D9_bin.26	88.1	2.74	MQ
F6D9_bin.31	77.07	1.72	MQ
F6D9_bin.32	76.12	4.03	MQ
F6D9_bin.34	84.76	0.17	MQ
F6D9_bin.38	87.94	0	MQ
F6D9_bin.43	79.06	0.37	MQ
F6D9_bin.46	80.95	0	MQ
F6D9_bin.47	69.51	0.43	MQ
F6D9_bin.49	52.6	1.34	MQ
F6D9_bin.51	62.21	0.95	MQ
F68C_bin.75	53.45	1.72	MQ
F68D_bin.104	54.58	3.45	MQ
F68T_bin.8	85.34	0.25	MQ
F6D9_bin.52	86.18	0.37	MQ
F68W_bin.102	73.66	0	MQ
F6D9_bin.56	53.23	0	MQ
F6D9_bin.58	50.86	3.45	MQ
F6D9_bin.6	76.18	0.49	MQ
F6D9_bin.61	56.9	1.72	MQ
F68M_bin.66	82.91	0	MQ
F6D9_bin.66	85.29	3.16	MQ
F6D9_bin.72	72.19	1.12	MQ
F6D9_bin.75	89.61	4.58	MQ
F6D9_bin.76	81.84	1.08	MQ
F68N_bin.26	70	1.72	MQ
F6D9_bin.80	55.93	0	MQ
F68F_bin.8	87.99	1.89	MQ
F68W_bin.64	83.64	2.01	MQ
F6D9_bin.85	82.21	0	MQ
F6DH_bin.75	78.41	1.81	MQ
F6D9_bin.86	89.87	1.05	MQ
F6D9_bin.87	89.26	1.29	MQ
F6D9_bin.88	55.59	1.12	MQ
F6D9_bin.9	88.36	0.67	MQ
F6D9_bin.92	81.85	2.36	MQ
F6D9_bin.94	77.07	1.56	MQ
F6D9_bin.96	61.11	1.01	MQ
F6DA_bin.11	77.87	1.37	MQ
F6DG_bin.11	56.63	3.37	MQ
F6DA_bin.12	84.3	1.44	MQ
F6DA_bin.14	83.47	1.73	MQ
F6DA_bin.17	71.29	0	MQ

Table S2. MAGs quanlity

	CheckM_completeness	CheckM_contamination	Quality
F6DA_bin.2	84.12	3.76	MQ
F6DA_bin.20	66.18	2.4	MQ
F6DA_bin.21	79.94	4.01	MQ
F6DA_bin.27	88.28	1.34	MQ
F6DA_bin.28	87.76	0.68	MQ
F68T_bin.15	84.69	1.71	MQ
F6D8_bin.16	76.68	2.44	MQ
F6D9_bin.60	82.26	2.44	MQ
F6DA_bin.30	84.1	1.43	MQ
F6DE_bin.87	78.59	1.34	MQ
F6DA_bin.38	86	2.63	MQ
F6DA_bin.4	80.57	0.24	MQ
F6MX_bin.39	89.94	2.08	MQ
F6DA_bin.42	58.91	0	MQ
F6DA_bin.46	66.94	0.81	MQ
F6DA_bin.50	58.62	0	MQ
F6DA_bin.52	89.47	0.68	MQ
F6DA_bin.54	87.12	0.84	MQ
F68R_bin.3	51.75	0	MQ
F6DB_bin.92	88.61	2.68	MQ
F6DA_bin.58	76.67	3.51	MQ
F6DA_bin.62	67.14	2.5	MQ
F6DA_bin.65	76.48	0.67	MQ
F6DA_bin.67	72.99	0	MQ
F6DA_bin.68	83.97	2.85	MQ
F6DA_bin.71	83.89	0.67	MQ
F6DA_bin.74	77.92	1.34	MQ
F68R_bin.104	84.99	3.38	MQ
F68H_bin.46	87.54	1.68	MQ
F6DA_bin.78	81.99	0.34	MQ
F6DA_bin.80	62.28	4.39	MQ
F6DA_bin.81	86.98	0.56	MQ
F6DA_bin.84	84.91	3.33	MQ
F6DG_bin.7	82	2.55	MQ
F68F_bin.69	87.02	0.34	MQ
F6DA_bin.89	55.94	0.67	MQ
F68H_bin.10	87.82	1.41	MQ
F68J_bin.35	89.14	0	MQ
F6DA_bin.90	73.69	2.74	MQ
F6DA_bin.92	83.1	1.72	MQ
F6DA_bin.96	86.79	0	MQ
F68D_bin.73	53.49	2.63	MQ
F6DB_bin.101	78.79	0.67	MQ
F6DG_bin.31	74.3	1	MQ
F68R_bin.5	82.7	1.34	MQ
F6DB_bin.108	86.98	0.59	MQ
F6DB_bin.115	79.71	3.1	MQ

Table S2. MAGs quanlity

	CheckM_completeness	CheckM_contamination	Quality
F68P_bin.23	60.71	1.79	MQ
F6D8_bin.82	51.25	0	MQ
F6DB_bin.22	87.32	1.4	MQ
F6DB_bin.27	72.55	1.02	MQ
F6DB_bin.28	75.2	0.68	MQ
F6DB_bin.30	85.53	1.84	MQ
F6DB_bin.34	70.67	1.27	MQ
F6DB_bin.35	54.9	0	MQ
F6DB_bin.37	85.99	0	MQ
F68T_bin.68	78.45	2.91	MQ
F6DB_bin.4	86.79	0.67	MQ
F6MY_bin.135	82.53	0.67	MQ
F6DB_bin.40	89.47	0	MQ
F6DB_bin.42	71.72	2.5	MQ
F68V_bin.126	52.15	0.86	MQ
F6DB_bin.46	77.31	1.72	MQ
F6DB_bin.5	89.53	0.73	MQ
F6DB_bin.50	83.63	1.51	MQ
F6DB_bin.55	75.94	0.49	MQ
F6DB_bin.57	67.59	0	MQ
F6DE_bin.89	53.45	0.86	MQ
F6DB_bin.59	68.62	2.65	MQ
F6DB_bin.69	89.59	4.95	MQ
F6D8_bin.97	58.06	2.11	MQ
F68C_bin.77	51.38	0	MQ
F68F_bin.83	85.23	0.67	MQ
F68J_bin.1	74.38	1.34	MQ
F68T_bin.14	85.91	0.67	MQ
F6DB_bin.70	84.56	0	MQ
F6DE_bin.35	87.25	0.67	MQ
F6DB_bin.72	83.67	0	MQ
F68C_bin.113	68.56	3.51	MQ
F6DB_bin.75	77.01	1.01	MQ
F68G_bin.45	82.26	1.41	MQ
F68M_bin.45	82.83	0.59	MQ
F68P_bin.53	71.74	4.06	MQ
F6DE_bin.55	74.52	0.66	MQ
F6DG_bin.36	89.74	0.95	MQ
F6DB_bin.80	74.48	0	MQ
F6DB_bin.85	84.9	1.75	MQ
F68C_bin.33	83.93	4.39	MQ
F68U_bin.114	82.78	1.35	MQ
F6DE_bin.76	81.38	1.72	MQ
F6DB_bin.96	89.86	2.02	MQ
F6DE_bin.12	86.09	1.81	MQ
F6DE_bin.13	73.71	1.57	MQ
F68D_bin.48	58.17	2.64	MQ

Table S2. MAGs quanlity

	CheckM_completeness	CheckM_contamination	Quality
F68E_bin.5	87.71	1.39	MQ
F68K_bin.56	80.09	1.6	MQ
F6DA_bin.10	88.28	2.08	MQ
F6DH_bin.131	51.18	0.23	MQ
F6DE_bin.22	85.23	0.21	MQ
F68T_bin.30	78.05	2.53	MQ
F6DE_bin.24	58.79	4.31	MQ
F6DE_bin.29	75.84	3.69	MQ
F68V_bin.78	60.66	3.45	MQ
F68V_bin.107	86.83	1.5	MQ
F6DE_bin.40	89.26	0	MQ
F6DE_bin.41	70.89	0	MQ
F6DE_bin.44	89.52	0.81	MQ
F6DE_bin.45	78.68	1.93	MQ
F6DE_bin.46	78.52	1.16	MQ
F68C_bin.110	86.43	3.66	MQ
F68F_bin.105	77	2.1	MQ
F6D7_bin.65	62.93	1.72	MQ
F6DB_bin.17	61.94	2.51	MQ
F6D7_bin.12	85.19	2.74	MQ
F6DE_bin.58	88.99	3.23	MQ
F6DG_bin.28	64.98	1.72	MQ
F6DE_bin.60	77.63	4.7	MQ
F6D8_bin.96	54.02	1.92	MQ
F6DE_bin.63	89.13	1.84	MQ
F6DE_bin.64	67.67	0	MQ
F68H_bin.36	70.08	3.32	MQ
F68R_bin.24	85.4	0.27	MQ
F68V_bin.18	84.08	2.01	MQ
F6DA_bin.98	51.28	0.12	MQ
F6DE_bin.69	84.94	1.63	MQ
F6DE_bin.71	68.68	1.79	MQ
F6DE_bin.75	84.34	1.46	MQ
F6DB_bin.112	85.04	1.52	MQ
F6DG_bin.59	89.54	0.56	MQ
F6DE_bin.79	88.87	4.72	MQ
F6DE_bin.81	82.12	0	MQ
F68G_bin.79	89.28	2.78	MQ
F68K_bin.64	85.47	0.45	MQ
F68T_bin.96	82.97	1.52	MQ
F6DB_bin.39	87.84	0.79	MQ
F6DE_bin.83	88.02	2.56	MQ
F68D_bin.57	89.96	0.02	MQ
F68E_bin.43	84.91	0.56	MQ
F68V_bin.73	65.67	0	MQ
F6D9_bin.16	62.63	2.33	MQ
F6DE_bin.92	70.7	2.63	MQ

Table S2. MAGs quanlity

	CheckM_completeness	CheckM_contamination	Quality
F6DE_bin.93	72.28	3.57	MQ
F6DG_bin.10	86.58	1.01	MQ
F6DG_bin.102	87.76	0	MQ
F6DG_bin.105	51.67	4.47	MQ
F6DG_bin.107	55.3	3.52	MQ
F6DG_bin.108	73.29	2.01	MQ
F6DG_bin.16	58.62	1.72	MQ
F6DG_bin.17	85.23	0	MQ
F6DG_bin.22	86.47	1.34	MQ
F6DG_bin.25	50.61	3.45	MQ
F6DG_bin.26	82.21	0	MQ
F6DG_bin.27	77.19	0	MQ
F6DG_bin.33	50.94	4.31	MQ
F6DG_bin.4	59.44	0	MQ
F6DB_bin.71	76.45	2.35	MQ
F6DG_bin.44	80.57	0	MQ
F6DG_bin.45	71.74	1.45	MQ
F6DG_bin.46	61.82	0.82	MQ
F6DG_bin.47	82.49	1.8	MQ
F6DG_bin.5	68.11	1.27	MQ
F6DG_bin.57	89.87	0	MQ
F6DG_bin.58	56.47	0.78	MQ
F6DG_bin.6	57.71	0	MQ
F6DG_bin.60	65	4.92	MQ
F6DG_bin.74	78.87	2.01	MQ
F6DG_bin.78	55.15	0.79	MQ
F6DG_bin.82	61.72	0.36	MQ
F68P_bin.45	85.92	3.14	MQ
F6DG_bin.85	65.67	0.32	MQ
F6DG_bin.93	86.11	1.81	MQ
F6DG_bin.94	86.39	1.03	MQ
F6DG_bin.95	62.52	4.03	MQ
F6DG_bin.98	89.92	1.61	MQ
F68W_bin.39	76.42	0	MQ
F6DH_bin.105	81.7	0	MQ
F6DH_bin.107	57.76	1.75	MQ
F6DH_bin.109	82.33	1.14	MQ
F68W_bin.106	88.59	2.72	MQ
F6DH_bin.111	83.08	0	MQ
F68W_bin.111	54.38	1.49	MQ
F6DG_bin.61	68.96	1.9	MQ
F6DH_bin.117	81.24	0	MQ
F6DH_bin.121	53.62	2.59	MQ
F6DH_bin.124	59.04	3.92	MQ
F6DH_bin.125	60.5	0.21	MQ
F6DH_bin.126	84.24	0.63	MQ
F68K_bin.7	74.6	0	MQ

Table S2. MAGs quanlity

	CheckM_completeness	CheckM_contamination	Quality
F68U_bin.8	72.41	1.72	MQ
F68W_bin.74	73.58	0	MQ
F6DH_bin.15	79.41	0	MQ
F68W_bin.11	83.66	2.35	MQ
F6DH_bin.18	81.21	0.81	MQ
F68U_bin.87	85.91	0	MQ
F68W_bin.107	82.38	0	MQ
F6D8_bin.89	68.68	4.39	MQ
F6D9_bin.36	67.72	2.37	MQ
F68U_bin.25	85.07	1.74	MQ
F68W_bin.34	85.59	0.67	MQ
F68W_bin.29	74.39	1.77	MQ
F6DH_bin.23	75.68	1.77	MQ
F68W_bin.99	89.54	0	MQ
F68W_bin.98	52.96	1.01	MQ
F6DH_bin.30	55.82	0	MQ
F68W_bin.60	55.72	0	MQ
F68U_bin.95	87.69	0.93	MQ
F68W_bin.27	87.64	1.02	MQ
F6DH_bin.37	89.25	0.23	MQ
F6DH_bin.39	81.7	3.32	MQ
F68W_bin.32	79.34	2.03	MQ
F6DH_bin.40	88.31	1.69	MQ
F6MY_bin.111	75.58	1.69	MQ
F68W_bin.86	71.85	3.32	MQ
F6DH_bin.48	80.3	1.34	MQ
F68U_bin.46	81.19	1.72	MQ
F68W_bin.5	61.32	1.72	MQ
F6DH_bin.53	64.64	3.57	MQ
F68W_bin.76	79.29	0.59	MQ
F6DH_bin.60	81.66	0.59	MQ
F6DH_bin.63	56.41	1.91	MQ
F6DH_bin.65	73	0	MQ
F6DH_bin.70	77.55	1.24	MQ
F6DH_bin.74	52.59	3.45	MQ
F68W_bin.50	69.49	2.59	MQ
F6DH_bin.77	85.94	2.03	MQ
F68W_bin.84	58.18	0	MQ
F6DH_bin.80	54.37	2.46	MQ
F6DH_bin.81	69.98	3.43	MQ
F6DH_bin.83	85.09	4.28	MQ
F6DH_bin.84	50.88	1.75	MQ
F6DH_bin.92	56.11	1.01	MQ
F6MX_bin.10	79.57	2.01	MQ
F6MX_bin.103	86.44	3.58	MQ
F6MX_bin.105	88.38	1.88	MQ
F6MX_bin.11	76.96	0.84	MQ

Table S2. MAGs quanlity

	CheckM_completeness	CheckM_contamination	Quality
F6MX_bin.112	86.88	0.17	MQ
F6MX_bin.12	83.64	0	MQ
F6MX_bin.120	57.89	1.75	MQ
F6MX_bin.122	88.14	0	MQ
F6MX_bin.132	89.26	0	MQ
F6MX_bin.133	65.48	0.68	MQ
F6MX_bin.134	84.84	4.37	MQ
F6MX_bin.136	89.09	0.67	MQ
F6MX_bin.137	50.89	0.67	MQ
F6MX_bin.138	72.4	4.7	MQ
F68C_bin.91	77.92	0	MQ
F6MX_bin.16	89.26	1.34	MQ
F6MX_bin.17	57.97	3.19	MQ
F6MX_bin.2	59.76	0	MQ
F6MX_bin.21	86.19	1.89	MQ
F6DG_bin.103	88.2	3.37	MQ
F6MX_bin.27	84.42	0.49	MQ
F6MX_bin.28	54.15	0	MQ
F6MX_bin.29	84.6	2.36	MQ
F68F_bin.6	86.24	3.36	MQ
F6MX_bin.3	86.91	0.67	MQ
F6MX_bin.30	89.14	0	MQ
F6MX_bin.31	74.71	0.67	MQ
F6MX_bin.33	59.23	4.38	MQ
F6MX_bin.34	72.87	0.22	MQ
F6MX_bin.37	52.59	2.35	MQ
F6MX_bin.43	67.65	1.11	MQ
F6MX_bin.45	88.98	0.1	MQ
F68N_bin.4	84.28	0.34	MQ
F6MX_bin.46	87.02	0	MQ
F6MX_bin.47	87.7	4.87	MQ
F6MX_bin.49	74.52	0.71	MQ
F6MX_bin.5	89.35	1.17	MQ
F6MX_bin.50	86.36	3.54	MQ
F6MX_bin.6	62.09	2.9	MQ
F6MX_bin.62	86.02	0	MQ
F68R_bin.40	79.69	0.22	MQ
F68V_bin.110	79.02	2.91	MQ
F6D8_bin.60	79.69	0.22	MQ
F6MX_bin.66	80.36	0.22	MQ
F6DG_bin.97	84.11	2.57	MQ
F6MX_bin.73	87.25	1.01	MQ
F6MX_bin.75	50.52	0	MQ
F6MX_bin.77	63.91	0.37	MQ
F6MX_bin.78	78.79	1.34	MQ
F6MY_bin.3	73.62	1.86	MQ
F6MX_bin.82	70.05	3.25	MQ

Table S2. MAGs quanlity

	CheckM_completeness	CheckM_contamination	Quality
F6MX_bin.85	54.7	1.75	MQ
F6MX_bin.89	80.5	2.18	MQ
F6MX_bin.9	60.34	1.72	MQ
F6MX_bin.90	68.64	2.31	MQ
F6MX_bin.93	52.51	4.98	MQ
F6MX_bin.94	77.07	0	MQ
F6MX_bin.97	73.08	0.71	MQ
F6MY_bin.1	83.9	0.68	MQ
F6MY_bin.10	85.12	2.42	MQ
F6MY_bin.101	67.48	0	MQ
F6MY_bin.102	83.66	0.53	MQ
F6MY_bin.104	86.8	1.57	MQ
F6MY_bin.105	74.96	0.67	MQ
F6MY_bin.107	52.38	0.81	MQ
F6MY_bin.109	65.52	1.72	MQ
F6MY_bin.112	68.6	0	MQ
F68H_bin.69	86.23	0.48	MQ
F68K_bin.36	75.06	0.37	MQ
F6D8_bin.28	81.2	0.37	MQ
F6MY_bin.117	87.23	0.93	MQ
F6MY_bin.122	58.62	0	MQ
F6MY_bin.127	53.36	0.34	MQ
F6MY_bin.128	67.41	1.57	MQ
F6MY_bin.13	75.89	3.44	MQ
F6MY_bin.132	88.73	2.41	MQ
F6MY_bin.133	64.86	1.75	MQ
F6MY_bin.138	58.28	1.45	MQ
F6MY_bin.139	71.35	0.74	MQ
F6MY_bin.14	77.3	2.84	MQ
F6MY_bin.16	80.93	0.96	MQ
F6MY_bin.17	52.14	0	MQ
F6MY_bin.19	88.5	0.94	MQ
F6MY_bin.2	53.65	3.78	MQ
F6MY_bin.20	60.34	0	MQ
F6MY_bin.23	53.63	3.57	MQ
F6MY_bin.26	68.45	2.59	MQ
F6MY_bin.29	70.16	2.59	MQ
F6MY_bin.30	68.57	0	MQ
F6MY_bin.31	88.36	0.87	MQ
F6MY_bin.37	62.6	3.51	MQ
F68U_bin.4	83.67	0.67	MQ
F6MY_bin.38	83.33	0	MQ
F68J_bin.2	74.9	1.73	MQ
F6MY_bin.49	89.26	0	MQ
F6MY_bin.53	52.68	0	MQ
F6MY_bin.55	78.15	2.13	MQ
F6MY_bin.57	87.9	2.01	MQ

Table S2. MAGs quanlity

	CheckM_completeness	CheckM_contamination	Quality
F6MY_bin.6	59.99	2.55	MQ
F6MY_bin.62	80.72	2.21	MQ
F6MY_bin.63	80.51	2.18	MQ
F6MY_bin.67	60.6	0.69	MQ
F6MY_bin.68	79.75	2.13	MQ
F6MY_bin.69	58.39	0	MQ
F6MY_bin.76	80.96	1.68	MQ
F6MY_bin.79	89.57	0.71	MQ
F6MY_bin.8	73.95	0.74	MQ
F68T_bin.43	59.42	2.34	MQ
F6MY_bin.82	71.83	1.89	MQ
F6MY_bin.84	74.35	0.17	MQ
F6MY_bin.86	85.14	2.27	MQ
F68V_bin.63	85.67	0	MQ
F6MX_bin.104	87.92	1.12	MQ
F6MY_bin.89	77.91	3.74	MQ
F6MY_bin.9	88.68	0.38	MQ
F6MY_bin.93	89.57	4.76	MQ
F68D_bin.40	70.33	2.59	MQ
F68G_bin.82	87.66	1.65	MQ
F68H_bin.33	56.54	1.72	MQ
F6D7_bin.20	52.76	0.86	MQ
F6DG_bin.38	79.46	4.14	MQ
F6MX_bin.113	72.14	0	MQ

Table S3. Taxonomic classifiation of the 2,421 detected MAGs

	Domain	Phylum	Class	Order	Family	Genus	Species	RefGenome	primary_cluster	secondary_cluster	repr_genome	genome	CheckM_completeness	CheckM_contamination	
F68V_bin.37	d__Archaea	p__Euryarchaeota	c__Methanobacteria	o__Methanobacteriales	f__Methanobacteriaceae	g__Methanobrevibacter_A	s__	N/A	187	187_0	F68V_bin.37.fa	F68V_bin.37.fa	99.2	0	1
F68C_bin.92	d__Archaea	p__Euryarchaeota	c__Methanobacteria	o__Methanobacteriales	f__Methanobacteriaceae	g__Methanobrevibacter_A	s__Methanobrevibacter_A smithii	GCF_000016525.1	186	186_2	F68D_bin.21.fa	F68C_bin.92.fa	100	0	1
F68D_bin.21	d__Archaea	p__Euryarchaeota	c__Methanobacteria	o__Methanobacteriales	f__Methanobacteriaceae	g__Methanobrevibacter_A	s__Methanobrevibacter_A smithii	GCF_000016525.1	186	186_2	F68D_bin.21.fa	F68D_bin.21.fa	100	0	1
F68G_bin.35	d__Archaea	p__Euryarchaeota	c__Methanobacteria	o__Methanobacteriales	f__Methanobacteriaceae	g__Methanobrevibacter_A	s__Methanobrevibacter_A smithii	GCF_000016525.1	186	186_2	F68D_bin.21.fa	F68G_bin.35.fa	100	0	1
F68H_bin.60	d__Archaea	p__Euryarchaeota	c__Methanobacteria	o__Methanobacteriales	f__Methanobacteriaceae	g__Methanobrevibacter_A	s__Methanobrevibacter_A smithii	GCF_000016525.1	186	186_2	F68D_bin.21.fa	F68H_bin.60.fa	100	0.8	1
F68J_bin.33	d__Archaea	p__Euryarchaeota	c__Methanobacteria	o__Methanobacteriales	f__Methanobacteriaceae	g__Methanobrevibacter_A	s__Methanobrevibacter_A smithii	GCF_000016525.1	186	186_2	F68D_bin.21.fa	F68J_bin.33.fa	100	0.8	1

Table S3. Taxonomic classifiation of the 2,421 detected MAGs

	Domain	Phylum	Class	Order	Family	Genus	Species	RefGenome	primary_cluster	secondary_cluster	repr_genome	genome	CheckM_completeness	CheckM_contamination	
F68M_bin.53	d__Archaea	p__Euryarchaeota	c__Methanobacteria	o__Methanobacteriales	f__Methanobacteriaceae	g__Methanobrevibacter_A	s__Methanobrevibacter_A smithii	GCF_000016525.1	186	186_2	F68D_bin.21.fa	F68M_bin.53.fa	100	0	1
F6D7_bin.99	d__Archaea	p__Euryarchaeota	c__Methanobacteria	o__Methanobacteriales	f__Methanobacteriaceae	g__Methanobrevibacter_A	s__Methanobrevibacter_A smithii	GCF_000016525.1	186	186_2	F68D_bin.21.fa	F6D7_bin.99.fa	91.29	1.66	1
F6D8_bin.35	d__Archaea	p__Euryarchaeota	c__Methanobacteria	o__Methanobacteriales	f__Methanobacteriaceae	g__Methanobrevibacter_A	s__Methanobrevibacter_A smithii	GCF_000016525.1	186	186_2	F68D_bin.21.fa	F6D8_bin.35.fa	100	0	1
F6DB_bin.111	d__Archaea	p__Euryarchaeota	c__Methanobacteria	o__Methanobacteriales	f__Methanobacteriaceae	g__Methanobrevibacter_A	s__Methanobrevibacter_A smithii	GCF_000016525.1	186	186_2	F68D_bin.21.fa	F6DB_bin.111.fa	100	0	1
F6MX_bin.135	d__Archaea	p__Euryarchaeota	c__Methanobacteria	o__Methanobacteriales	f__Methanobacteriaceae	g__Methanobrevibacter_A	s__Methanobrevibacter_A smithii	GCF_000016525.1	186	186_2	F68D_bin.21.fa	F6MX_bin.135.fa	100	0	1
F6MY_bin.118	d__Archaea	p__Euryarchaeota	c__Methanobacteria	o__Methanobacteriales	f__Methanobacteriaceae	g__Methanobrevibacter_A	s__Methanobrevibacter_A smithii	GCF_000016525.1	186	186_2	F68D_bin.21.fa	F6MY_bin.118.fa	100	0	1

Table S3. Taxonomic classifiation of the 2,421 detected MAGs

	Domain	Phylum	Class	Order	Family	Genus	Species	RefGenome	primary_cluster	secondary_cluster	repr_genome	genome	CheckM_completeness	CheckM_contamination	
F68N_bin.15	d__Archaea	p__Euryarchaeota	c__Methanobacteria	o__Methanobacteriales	f__Methanobacteriaceae	g__Methanobrevibacter_A	s__Methanobrevibacter_A smithii	GCF_000016525.1	186	186_1	F6D9_bin.81.fa	F68N_bin.15.fa	100	0	1
F68R_bin.18	d__Archaea	p__Euryarchaeota	c__Methanobacteria	o__Methanobacteriales	f__Methanobacteriaceae	g__Methanobrevibacter_A	s__Methanobrevibacter_A smithii	GCF_000016525.1	186	186_1	F6D9_bin.81.fa	F68R_bin.18.fa	100	0	1
F68T_bin.78	d__Archaea	p__Euryarchaeota	c__Methanobacteria	o__Methanobacteriales	f__Methanobacteriaceae	g__Methanobrevibacter_A	s__Methanobrevibacter_A smithii	GCF_000016525.1	186	186_1	F6D9_bin.81.fa	F68T_bin.78.fa	97.6	0	1
F68U_bin.100	d__Archaea	p__Euryarchaeota	c__Methanobacteria	o__Methanobacteriales	f__Methanobacteriaceae	g__Methanobrevibacter_A	s__Methanobrevibacter_A smithii	GCF_000016525.1	186	186_1	F6D9_bin.81.fa	F68U_bin.100.fa	100	0	1
F6D9_bin.81	d__Archaea	p__Euryarchaeota	c__Methanobacteria	o__Methanobacteriales	f__Methanobacteriaceae	g__Methanobrevibacter_A	s__Methanobrevibacter_A smithii	GCF_000016525.1	186	186_1	F6D9_bin.81.fa	F6D9_bin.81.fa	100	0	1
F68F_bin.23	d__Archaea	p__Euryarchaeota	c__Methanobacteria	o__Methanobacteriales	f__Methanobacteriaceae	g__Methanobrevibacter_A	s__Methanobrevibacter_A woesei	GCA_003111605.1	185	185_1	F68F_bin.23.fa	F68F_bin.23.fa	100	0.8	1

Table S3. Taxonomic classifiation of the 2,421 detected MAGs

	Domain	Phylum	Class	Order	Family	Genus	Species	RefGenome	primary_cluster	secondary_cluster	repr_genome	genome	CheckM_completeness	CheckM_contamination	
F68V_bin.12	d__Archaea	p__Euryarchaeota	c__Methanobacteria	o__Methanobacteriales	f__Methanobacteriaceae	g__Methanobrevibacter_A	s__Methanobrevibacter_A woesei	GCA_003111605.1	185	185_2	F68V_bin.12.fa	F68V_bin.12.fa	75.21	0.4	1
F68D_bin.96	d__Archaea	p__Euryarchaeota	c__Methanobacteria	o__Methanobacteriales	f__Methanobacteriaceae	g__Methanosphaera	s__Methanosphaera stadtmanae	GCF_000012545.1	198	198_1	F68G_bin.4.fa	F68D_bin.96.fa	50.73	0	1
F68G_bin.4	d__Archaea	p__Euryarchaeota	c__Methanobacteria	o__Methanobacteriales	f__Methanobacteriaceae	g__Methanosphaera	s__Methanosphaera stadtmanae	GCF_000012545.1	198	198_1	F68G_bin.4.fa	F68G_bin.4.fa	97.6	0	1
F68N_bin.63	d__Archaea	p__Euryarchaeota	c__Methanobacteria	o__Methanobacteriales	f__Methanobacteriaceae	g__Methanosphaera	s__Methanosphaera stadtmanae	GCF_000012545.1	198	198_1	F68G_bin.4.fa	F68N_bin.63.fa	96.8	0	1
F68R_bin.120	d__Archaea	p__Euryarchaeota	c__Methanobacteria	o__Methanobacteriales	f__Methanobacteriaceae	g__Methanosphaera	s__Methanosphaera stadtmanae	GCF_000012545.1	198	198_1	F68G_bin.4.fa	F68R_bin.120.fa	85.59	2.84	1
F68G_bin.8	d__Archaea	p__Thermoplasmatota	c__Thermoplasmata	o__Methanomassiliicoccales	f__Methanomethylophilaceae	g__Methanomethylophilus	s__Methanomethylophilus alvus	GCF_000300255.2	244	244_2	F68G_bin.8.fa	F68G_bin.8.fa	97.35	1.61	1

Table S3. Taxonomic classifiation of the 2,421 detected MAGs

	Domain	Phylum	Class	Order	Family	Genus	Species	RefGenome	primary_cluster	secondary_cluster	repr_genome	genome	CheckM_completeness	CheckM_contamination	
F68D_bin.74	d__Archaea	p__Thermoplasmatota	c__Thermoplasmata	o__Methanomassiliicoccales	f__Methanomethylophilaceae	g__Methanomethylophilus	s__Methanomethylophilus alvus	GCF_000300255.2	244	244_1	F68R_bin.34.fa	F68D_bin.74.fa	98.39	0	1
F68F_bin.18	d__Archaea	p__Thermoplasmatota	c__Thermoplasmata	o__Methanomassiliicoccales	f__Methanomethylophilaceae	g__Methanomethylophilus	s__Methanomethylophilus alvus	GCF_000300255.2	244	244_1	F68R_bin.34.fa	F68F_bin.18.fa	98.39	0.81	1
F68R_bin.34	d__Archaea	p__Thermoplasmatota	c__Thermoplasmata	o__Methanomassiliicoccales	f__Methanomethylophilaceae	g__Methanomethylophilus	s__Methanomethylophilus alvus	GCF_000300255.2	244	244_1	F68R_bin.34.fa	F68R_bin.34.fa	98.39	0	1
F68G_bin.3	d__Archaea	p__Thermoplasmatota	c__Thermoplasmata	o__Methanomassiliicoccales	f__Methanomethylophilaceae	g__UBA71	s__	N/A	245	245_0	F68G_bin.3.fa	F68G_bin.3.fa	66.75	1	2
F68M_bin.6	d__Archaea	p__Thermoplasmatota	c__Thermoplasmata	o__Methanomassiliicoccales	f__Methanomethylophilaceae	g__UBA71	s__	N/A	246	246_0	F68M_bin.6.fa	F68M_bin.6.fa	98.52	0.32	3
F68T_bin.56	d__Archaea	p__Thermoplasmatota	c__Thermoplasmata	o__Methanomassiliicoccales	f__Methanomethylophilaceae	g__UBA71	s__	N/A	247	247_0	F68T_bin.56.fa	F68T_bin.56.fa	79.31	1.92	4

Table S3. Taxonomic classifiation of the 2,421 detected MAGs

	Domain	Phylum	Class	Order	Family	Genus	Species	RefGenome	primary_cluster	secondary_cluster	repr_genome	genome	CheckM_completeness	CheckM_contamination	
F68V_bin.102	d__Bacteria	p__Actinobacteriota	c__Actinobacteria	o__Actinomycetales	f__Actinomycetaceae	g__Pauljensenia	s__Pauljensenia sp001838165	GCF_001838165.1	468	468_0	F68V_bin.102.fa	F68V_bin.102.fa	67.53	2.96	4
F68H_bin.72	d__Bacteria	p__Actinobacteriota	c__Actinobacteria	o__Actinomycetales	f__Bifidobacteriaceae	g__Bifidobacterium	s__Bifidobacterium adolescentis	GCF_000010425.1	525	525_0	F68H_bin.72.fa	F68H_bin.72.fa	100	1.87	4
F68T_bin.18	d__Bacteria	p__Actinobacteriota	c__Actinobacteria	o__Actinomycetales	f__Bifidobacteriaceae	g__Bifidobacterium	s__Bifidobacterium bifidum	GCF_001025135.1	526	526_1	F68T_bin.18.fa	F68T_bin.18.fa	98.16	0.92	4
F6DB_bin.30	d__Bacteria	p__Actinobacteriota	c__Actinobacteria	o__Actinomycetales	f__Bifidobacteriaceae	g__Bifidobacterium	s__Bifidobacterium bifidum	GCF_001025135.1	526	526_2	F6DB_bin.30.fa	F6DB_bin.30.fa	85.53	1.84	4
F68M_bin.14	d__Bacteria	p__Actinobacteriota	c__Actinobacteria	o__Actinomycetales	f__Bifidobacteriaceae	g__Bifidobacterium	s__Bifidobacterium dentium	GCF_001042595.1	523	523_1	F6D7_bin.30.fa	F68M_bin.14.fa	88.23	4.05	4
F6D7_bin.30	d__Bacteria	p__Actinobacteriota	c__Actinobacteria	o__Actinomycetales	f__Bifidobacteriaceae	g__Bifidobacterium	s__Bifidobacterium dentium	GCF_001042595.1	523	523_1	F6D7_bin.30.fa	F6D7_bin.30.fa	96.29	1.72	4
F6MX_bin.25	d__Bacteria	p__Actinobacteriota	c__Actinobacteria	o__Actinomycetales	f__Bifidobacteriaceae	g__Bifidobacterium	s__Bifidobacterium dentium	GCF_001042595.1	523	523_1	F6D7_bin.30.fa	F6MX_bin.25.fa	72.14	1.05	4

Table S3. Taxonomic classifiation of the 2,421 detected MAGs

	Domain	Phylum	Class	Order	Family	Genus	Species	RefGenome	primary_cluster	secondary_cluster	repr_genome	genome	CheckM_completeness	CheckM_contamination	
F68T_bin.30	d__Bacteria	p__Actinobacteriota	c__Actinobacteria	o__Actinomycetales	f__Bifidobacteriaceae	g__Bifidobacterium	s__Bifidobacterium longum	GCF_000196555.1	527	527_1	F6DE_bin.23.fa	F68T_bin.30.fa	78.05	2.53	4
F6DB_bin.53	d__Bacteria	p__Actinobacteriota	c__Actinobacteria	o__Actinomycetales	f__Bifidobacteriaceae	g__Bifidobacterium	s__Bifidobacterium longum	GCF_000196555.1	527	527_1	F6DE_bin.23.fa	F6DB_bin.53.fa	90.27	1.39	4
F6DE_bin.23	d__Bacteria	p__Actinobacteriota	c__Actinobacteria	o__Actinomycetales	f__Bifidobacteriaceae	g__Bifidobacterium	s__Bifidobacterium longum	GCF_000196555.1	527	527_1	F6DE_bin.23.fa	F6DE_bin.23.fa	99.31	0	4
F68V_bin.66	d__Bacteria	p__Actinobacteriota	c__Actinobacteria	o__Actinomycetales	f__Bifidobacteriaceae	g__Bifidobacterium	s__Bifidobacterium pseudocatenulatum	GCF_001025215.1	524	524_1	F68V_bin.66.fa	F68V_bin.66.fa	95.36	0.45	4
F6DB_bin.43	d__Bacteria	p__Actinobacteriota	c__Actinobacteria	o__Actinomycetales	f__Bifidobacteriaceae	g__Bifidobacterium	s__Bifidobacterium pseudocatenulatum	GCF_001025215.1	524	524_2	F6DB_bin.43.fa	F6DB_bin.43.fa	100	0.23	4
F6DE_bin.50	d__Bacteria	p__Actinobacteriota	c__Actinobacteria	o__Actinomycetales	f__Micrococcaceae	g__Rothia	s__Rothia mucilaginosa_B	GCF_001548235.1	383	383_0	F6DE_bin.50.fa	F6DE_bin.50.fa	99.33	1.33	4

Table S3. Taxonomic classifiation of the 2,421 detected MAGs

	Domain	Phylum	Class	Order	Family	Genus	Species	RefGenome	primary_cluster	secondary_cluster	repr_genome	genome	CheckM_completeness	CheckM_contamination	
F68V_bin.69	d__Bacteria	p__Actinobacteriota	c__Actinobacteria	o__Mycobacteriales	f__Mycobacteriaceae	g__Corynebacterium	s__Corynebacterium argentoratense	GCF_000590555.1	21	21_1	F68V_bin.69.fa	F68V_bin.69.fa	84.18	1.06	4
F6MX_bin.115	d__Bacteria	p__Actinobacteriota	c__Actinobacteria	o__Mycobacteriales	f__Mycobacteriaceae	g__Corynebacterium	s__Corynebacterium argentoratense	GCF_000590555.1	21	21_2	F6MX_bin.115.fa	F6MX_bin.115.fa	96.51	0.05	4
F68M_bin.13	d__Bacteria	p__Actinobacteriota	c__Coriobacteriia	o__Coriobacteriales	f__Atopobiaceae	g__	s__	N/A	512	512_0	F68M_bin.13.fa	F68M_bin.13.fa	99.6	1.49	5
F68M_bin.44	d__Bacteria	p__Actinobacteriota	c__Coriobacteriia	o__Coriobacteriales	f__Atopobiaceae	g__Olsenella	s__Olsenella phocaeensis	GCF_900120385.1	658	658_0	F68M_bin.44.fa	F68M_bin.44.fa	83.43	3.23	5
F68M_bin.58	d__Bacteria	p__Actinobacteriota	c__Coriobacteriia	o__Coriobacteriales	f__Atopobiaceae	g__Olsenella_B	s__Olsenella_B sp000752675	GCF_000752675.2	511	511_1	F68N_bin.67.fa	F68M_bin.58.fa	100	1.61	5
F68N_bin.67	d__Bacteria	p__Actinobacteriota	c__Coriobacteriia	o__Coriobacteriales	f__Atopobiaceae	g__Olsenella_B	s__Olsenella_B sp000752675	GCF_000752675.2	511	511_1	F68N_bin.67.fa	F68N_bin.67.fa	99.19	0.16	5
F6MY_bin.6	d__Bacteria	p__Actinobacteriota	c__Coriobacteriia	o__Coriobacteriales	f__Atopobiaceae	g__Olsenella_B	s__Olsenella_B sp000752675	GCF_000752675.2	511	511_2	F6MY_bin.6.fa	F6MY_bin.6.fa	59.99	2.55	5

Table S3. Taxonomic classifiation of the 2,421 detected MAGs

	Domain	Phylum	Class	Order	Family	Genus	Species	RefGenome	primary_cluster	secondary_cluster	repr_genome	genome	CheckM_completeness	CheckM_contamination	
F68C_bin.29	d__Bacteria	p__Actinobacteriota	c__Coriobacteriia	o__Coriobacteriales	f__Coriobacteriaceae	g__Collinsella	s__	N/A	514	514_17	F68C_bin.29.fa	F68C_bin.29.fa	78.77	3.03	6
F68R_bin.74	d__Bacteria	p__Actinobacteriota	c__Coriobacteriia	o__Coriobacteriales	f__Coriobacteriaceae	g__Collinsella	s__	N/A	514	514_5	F68R_bin.74.fa	F68R_bin.74.fa	92.61	2.94	7
F68U_bin.63	d__Bacteria	p__Actinobacteriota	c__Coriobacteriia	o__Coriobacteriales	f__Coriobacteriaceae	g__Collinsella	s__	N/A	514	514_6	F68U_bin.63.fa	F68U_bin.63.fa	87.26	1.73	8
F68W_bin.63	d__Bacteria	p__Actinobacteriota	c__Coriobacteriia	o__Coriobacteriales	f__Coriobacteriaceae	g__Collinsella	s__	N/A	514	514_6	F68U_bin.63.fa	F68W_bin.63.fa	80.89	2.78	9
F6DB_bin.96	d__Bacteria	p__Actinobacteriota	c__Coriobacteriia	o__Coriobacteriales	f__Coriobacteriaceae	g__Collinsella	s__	N/A	514	514_2	F6DB_bin.96.fa	F6DB_bin.96.fa	89.86	2.02	10
F6DE_bin.44	d__Bacteria	p__Actinobacteriota	c__Coriobacteriia	o__Coriobacteriales	f__Coriobacteriaceae	g__Collinsella	s__	N/A	514	514_19	F6DE_bin.44.fa	F6DE_bin.44.fa	89.52	0.81	11
F6MY_bin.78	d__Bacteria	p__Actinobacteriota	c__Coriobacteriia	o__Coriobacteriales	f__Coriobacteriaceae	g__Collinsella	s__	N/A	514	514_18	F6MY_bin.78.fa	F6MY_bin.78.fa	100	0.89	12
F68K_bin.51	d__Bacteria	p__Actinobacteriota	c__Coriobacteriia	o__Coriobacteriales	f__Coriobacteriaceae	g__Collinsella	s__	N/A	516	516_1	F68K_bin.51.fa	F68K_bin.51.fa	91.32	2.19	13
F68D_bin.30	d__Bacteria	p__Actinobacteriota	c__Coriobacteriia	o__Coriobacteriales	f__Coriobacteriaceae	g__Collinsella	s__	N/A	517	517_0	F68D_bin.30.fa	F68D_bin.30.fa	98.79	0	14

Table S3. Taxonomic classifiation of the 2,421 detected MAGs

	Domain	Phylum	Class	Order	Family	Genus	Species	RefGenome	primary_cluster	secondary_cluster	repr_genome	genome	CheckM_completeness	CheckM_contamination	
F6MX_bin.15	d__Bacteria	p__Actinobacteriota	c__Coriobacteriia	o__Coriobacteriales	f__Coriobacteriaceae	g__Collinsella	s__Collinsella bouchesdurhonensis	GCF_900155365.1	513	513_1	F6MX_bin.15.fa	F6MX_bin.15.fa	97.58	2.42	14
F6MY_bin.107	d__Bacteria	p__Actinobacteriota	c__Coriobacteriia	o__Coriobacteriales	f__Coriobacteriaceae	g__Collinsella	s__Collinsella bouchesdurhonensis	GCF_900155365.1	513	513_2	F6MY_bin.107.fa	F6MY_bin.107.fa	52.38	0.81	14
F68U_bin.51	d__Bacteria	p__Actinobacteriota	c__Coriobacteriia	o__Coriobacteriales	f__Coriobacteriaceae	g__Collinsella	s__Collinsella intestinalis	GCF_000156175.1	516	516_2	F68U_bin.51.fa	F68U_bin.51.fa	91.56	2.62	14
F68D_bin.11	d__Bacteria	p__Actinobacteriota	c__Coriobacteriia	o__Coriobacteriales	f__Coriobacteriaceae	g__Collinsella	s__Collinsella sp002232035	GCF_002232035.1	514	514_16	F68D_bin.11.fa	F68D_bin.11.fa	89.04	4.57	14
F68E_bin.44	d__Bacteria	p__Actinobacteriota	c__Coriobacteriia	o__Coriobacteriales	f__Coriobacteriaceae	g__Collinsella	s__Collinsella sp002232035	GCF_002232035.1	514	514_7	F68E_bin.44.fa	F68E_bin.44.fa	100	0.81	14
F68F_bin.89	d__Bacteria	p__Actinobacteriota	c__Coriobacteriia	o__Coriobacteriales	f__Coriobacteriaceae	g__Collinsella	s__Collinsella sp002232035	GCF_002232035.1	514	514_14	F68F_bin.89.fa	F68F_bin.89.fa	94.35	1.01	14
F68G_bin.11	d__Bacteria	p__Actinobacteriota	c__Coriobacteriia	o__Coriobacteriales	f__Coriobacteriaceae	g__Collinsella	s__Collinsella sp002232035	GCF_002232035.1	514	514_3	F68G_bin.11.fa	F68G_bin.11.fa	62.67	2.15	14

Table S3. Taxonomic classifiation of the 2,421 detected MAGs

	Domain	Phylum	Class	Order	Family	Genus	Species	RefGenome	primary_cluster	secondary_cluster	repr_genome	genome	CheckM_completeness	CheckM_contamination	
F68J_bin.62	d__Bacteria	p__Actinobacteriota	c__Coriobacteriia	o__Coriobacteriales	f__Coriobacteriaceae	g__Collinsella	s__Collinsella sp002232035	GCF_002232035.1	514	514_12	F68J_bin.62.fa	F68J_bin.62.fa	100	0.81	14
F68K_bin.37	d__Bacteria	p__Actinobacteriota	c__Coriobacteriia	o__Coriobacteriales	f__Coriobacteriaceae	g__Collinsella	s__Collinsella sp002232035	GCF_002232035.1	514	514_9	F68K_bin.37.fa	F68K_bin.37.fa	92.74	1.08	14
F68M_bin.55	d__Bacteria	p__Actinobacteriota	c__Coriobacteriia	o__Coriobacteriales	f__Coriobacteriaceae	g__Collinsella	s__Collinsella sp002232035	GCF_002232035.1	514	514_11	F68M_bin.55.fa	F68M_bin.55.fa	98.39	1.61	14
F68P_bin.5	d__Bacteria	p__Actinobacteriota	c__Coriobacteriia	o__Coriobacteriales	f__Coriobacteriaceae	g__Collinsella	s__Collinsella sp002232035	GCF_002232035.1	514	514_4	F68P_bin.5.fa	F68P_bin.5.fa	100	0.81	14
F68V_bin.35	d__Bacteria	p__Actinobacteriota	c__Coriobacteriia	o__Coriobacteriales	f__Coriobacteriaceae	g__Collinsella	s__Collinsella sp002232035	GCF_002232035.1	514	514_8	F68V_bin.35.fa	F68V_bin.35.fa	100	1.21	14
F6D7_bin.16	d__Bacteria	p__Actinobacteriota	c__Coriobacteriia	o__Coriobacteriales	f__Coriobacteriaceae	g__Collinsella	s__Collinsella sp002232035	GCF_002232035.1	514	514_10	F6D7_bin.16.fa	F6D7_bin.16.fa	96.61	2.66	14
F6D8_bin.54	d__Bacteria	p__Actinobacteriota	c__Coriobacteriia	o__Coriobacteriales	f__Coriobacteriaceae	g__Collinsella	s__Collinsella sp002232035	GCF_002232035.1	514	514_15	F6D8_bin.54.fa	F6D8_bin.54.fa	100	0.81	14

Table S3. Taxonomic classifiation of the 2,421 detected MAGs

	Domain	Phylum	Class	Order	Family	Genus	Species	RefGenome	primary_cluster	secondary_cluster	repr_genome	genome	CheckM_completeness	CheckM_contamination	
F6D9_bin.14	d__Bacteria	p__Actinobacteriota	c__Coriobacteriia	o__Coriobacteriales	f__Coriobacteriaceae	g__Collinsella	s__Collinsella sp002232035	GCF_002232035.1	514	514_13	F6D9_bin.14.fa	F6D9_bin.14.fa	96.77	1.73	14
F6DA_bin.46	d__Bacteria	p__Actinobacteriota	c__Coriobacteriia	o__Coriobacteriales	f__Coriobacteriaceae	g__Collinsella	s__Collinsella sp002232035	GCF_002232035.1	514	514_1	F6DA_bin.46.fa	F6DA_bin.46.fa	66.94	0.81	14
F68D_bin.5	d__Bacteria	p__Actinobacteriota	c__Coriobacteriia	o__Coriobacteriales	f__Coriobacteriaceae	g__Collinsella	s__Collinsella tanakaei	GCF_000225705.1	515	515_3	F68D_bin.5.fa	F68D_bin.5.fa	96.15	0	14
F68F_bin.35	d__Bacteria	p__Actinobacteriota	c__Coriobacteriia	o__Coriobacteriales	f__Coriobacteriaceae	g__Collinsella	s__Collinsella tanakaei	GCF_000225705.1	515	515_1	F68F_bin.35.fa	F68F_bin.35.fa	100	2.42	14
F68K_bin.48	d__Bacteria	p__Actinobacteriota	c__Coriobacteriia	o__Coriobacteriales	f__Coriobacteriaceae	g__Collinsella	s__Collinsella tanakaei	GCF_000225705.1	515	515_1	F68F_bin.35.fa	F68K_bin.48.fa	73.05	1.41	14
F6DE_bin.43	d__Bacteria	p__Actinobacteriota	c__Coriobacteriia	o__Coriobacteriales	f__Coriobacteriaceae	g__Collinsella	s__Collinsella tanakaei	GCF_000225705.1	515	515_2	F6DE_bin.43.fa	F6DE_bin.43.fa	100	1.2	14
F68C_bin.102	d__Bacteria	p__Actinobacteriota	c__Coriobacteriia	o__Coriobacteriales	f__Eggerthellaceae	g__	s__	N/A	518	518_0	F68C_bin.102.fa	F68C_bin.102.fa	71.57	1.76	15
F6DB_bin.28	d__Bacteria	p__Actinobacteriota	c__Coriobacteriia	o__Coriobacteriales	f__Eggerthellaceae	g__	s__	N/A	531	531_3	F6DB_bin.28.fa	F6DB_bin.28.fa	75.2	0.68	16

Table S3. Taxonomic classifiation of the 2,421 detected MAGs

	Domain	Phylum	Class	Order	Family	Genus	Species	RefGenome	primary_cluster	secondary_cluster	repr_genome	genome	CheckM_completeness	CheckM_contamination	
F6DH_bin.81	d__Bacteria	p__Actinobacteriota	c__Coriobacteriia	o__Coriobacteriales	f__Eggerthellaceae	g__	s__	N/A	531	531_1	F6DH_bin.81.fa	F6DH_bin.81.fa	69.98	3.43	17
F6MX_bin.2	d__Bacteria	p__Actinobacteriota	c__Coriobacteriia	o__Coriobacteriales	f__Eggerthellaceae	g__	s__	N/A	531	531_2	F6MX_bin.2.fa	F6MX_bin.2.fa	59.76	0	18
F68F_bin.63	d__Bacteria	p__Actinobacteriota	c__Coriobacteriia	o__Coriobacteriales	f__Eggerthellaceae	g__Adlercreutzia	s__Adlercreutzia equolifaciens	GCF_000478885.1	520	520_0	F68F_bin.63.fa	F68F_bin.63.fa	66.07	1.79	18
F68T_bin.70	d__Bacteria	p__Actinobacteriota	c__Coriobacteriia	o__Coriobacteriales	f__Eggerthellaceae	g__CAG-1427	s__CAG-1427 sp000431675	GCA_000431675.1	530	530_0	F68T_bin.70.fa	F68T_bin.70.fa	98.56	1.53	18
F68F_bin.68	d__Bacteria	p__Actinobacteriota	c__Coriobacteriia	o__Coriobacteriales	f__Eggerthellaceae	g__CAG-1427	s__CAG-1427 sp000435475	GCA_000435475.1	529	529_3	F68F_bin.68.fa	F68F_bin.68.fa	97.58	0.12	18
F68N_bin.1	d__Bacteria	p__Actinobacteriota	c__Coriobacteriia	o__Coriobacteriales	f__Eggerthellaceae	g__CAG-1427	s__CAG-1427 sp000435475	GCA_000435475.1	529	529_5	F68N_bin.1.fa	F68N_bin.1.fa	76.61	0.12	18
F68T_bin.71	d__Bacteria	p__Actinobacteriota	c__Coriobacteriia	o__Coriobacteriales	f__Eggerthellaceae	g__CAG-1427	s__CAG-1427 sp000435475	GCA_000435475.1	529	529_1	F68T_bin.71.fa	F68T_bin.71.fa	81.7	1.21	18
F6D7_bin.93	d__Bacteria	p__Actinobacteriota	c__Coriobacteriia	o__Coriobacteriales	f__Eggerthellaceae	g__CAG-1427	s__CAG-1427 sp000435475	GCA_000435475.1	529	529_2	F6D7_bin.93.fa	F6D7_bin.93.fa	85.23	1.04	18

Table S3. Taxonomic classifiation of the 2,421 detected MAGs

	Domain	Phylum	Class	Order	Family	Genus	Species	RefGenome	primary_cluster	secondary_cluster	repr_genome	genome	CheckM_completeness	CheckM_contamination	
F6MX_bin.51	d__Bacteria	p__Actinobacteriota	c__Coriobacteriia	o__Coriobacteriales	f__Eggerthellaceae	g__CAG-1427	s__CAG-1427 sp000435475	GCA_000435475.1	529	529_4	F6MX_bin.51.fa	F6MX_bin.51.fa	98.97	0.2	18
F68E_bin.29	d__Bacteria	p__Actinobacteriota	c__Coriobacteriia	o__Coriobacteriales	f__Eggerthellaceae	g__Eggerthella	s__Eggerthella lenta	GCF_000024265.1	519	519_0	F68E_bin.29.fa	F68E_bin.29.fa	82.79	2.85	18
F68K_bin.86	d__Bacteria	p__Actinobacteriota	c__Coriobacteriia	o__Coriobacteriales	f__Eggerthellaceae	g__Raoultibacter	s__	N/A	522	522_0	F68K_bin.86.fa	F68K_bin.86.fa	54.78	1.72	19
F68F_bin.96	d__Bacteria	p__Actinobacteriota	c__Coriobacteriia	o__Coriobacteriales	f__Eggerthellaceae	g__Senegalimassilia	s__Senegalimassilia anaerobia	GCF_000236865.1	532	532_0	F68F_bin.96.fa	F68F_bin.96.fa	67.73	1.08	19
F6DG_bin.4	d__Bacteria	p__Actinobacteriota	c__Coriobacteriia	o__Coriobacteriales	f__Eggerthellaceae	g__Slackia_A	s__	N/A	528	528_1	F6DG_bin.4.fa	F6DG_bin.4.fa	59.44	0	20
F6DH_bin.18	d__Bacteria	p__Actinobacteriota	c__Coriobacteriia	o__Coriobacteriales	f__Eggerthellaceae	g__Slackia_A	s__	N/A	528	528_2	F6DH_bin.18.fa	F6DH_bin.18.fa	81.21	0.81	21
F68V_bin.105	d__Bacteria	p__Actinobacteriota	c__Coriobacteriia	o__Coriobacteriales	f__QAMH01	g__QAMH01	s__	N/A	521	521_0	F68V_bin.105.fa	F68V_bin.105.fa	76.88	3.23	22
F68F_bin.29	d__Bacteria	p__Bacteroidota	c__Bacteroidia	o__Bacteroidales	f__Bacteroidaceae	g__	s__	N/A	75	75_0	F68F_bin.29.fa	F68F_bin.29.fa	52.59	0	23
F6DH_bin.121	d__Bacteria	p__Bacteroidota	c__Bacteroidia	o__Bacteroidales	f__Bacteroidaceae	g__	s__	N/A	409	409_0	F6DH_bin.121.fa	F6DH_bin.121.fa	53.62	2.59	24

Table S3. Taxonomic classifiation of the 2,421 detected MAGs

	Domain	Phylum	Class	Order	Family	Genus	Species	RefGenome	primary_cluster	secondary_cluster	repr_genome	genome	CheckM_completeness	CheckM_contamination	
F68C_bin.80	d__Bacteria	p__Bacteroidota	c__Bacteroidia	o__Bacteroidales	f__Bacteroidaceae	g__43-108	s__43-108 sp001915545	GCA_001915545.1	74	74_0	F68C_bin.80.fa	F68C_bin.80.fa	77.05	0	24
F6DB_bin.88	d__Bacteria	p__Bacteroidota	c__Bacteroidia	o__Bacteroidales	f__Bacteroidaceae	g__Bacteroides	s__Bacteroides caccae	GCF_002222615.2	92	92_1	F6DB_bin.88.fa	F6DB_bin.88.fa	94.37	0.96	24
F6DE_bin.76	d__Bacteria	p__Bacteroidota	c__Bacteroidia	o__Bacteroidales	f__Bacteroidaceae	g__Bacteroides	s__Bacteroides caccae	GCF_002222615.2	92	92_1	F6DB_bin.88.fa	F6DE_bin.76.fa	81.38	1.72	24
F68C_bin.75	d__Bacteria	p__Bacteroidota	c__Bacteroidia	o__Bacteroidales	f__Bacteroidaceae	g__Bacteroides	s__Bacteroides eggerthii	GCF_000155815.1	96	96_1	F6D9_bin.52.fa	F68C_bin.75.fa	53.45	1.72	24
F68D_bin.104	d__Bacteria	p__Bacteroidota	c__Bacteroidia	o__Bacteroidales	f__Bacteroidaceae	g__Bacteroides	s__Bacteroides eggerthii	GCF_000155815.1	96	96_1	F6D9_bin.52.fa	F68D_bin.104.fa	54.58	3.45	24
F68T_bin.8	d__Bacteria	p__Bacteroidota	c__Bacteroidia	o__Bacteroidales	f__Bacteroidaceae	g__Bacteroides	s__Bacteroides eggerthii	GCF_000155815.1	96	96_1	F6D9_bin.52.fa	F68T_bin.8.fa	85.34	0.25	24
F6D9_bin.52	d__Bacteria	p__Bacteroidota	c__Bacteroidia	o__Bacteroidales	f__Bacteroidaceae	g__Bacteroides	s__Bacteroides eggerthii	GCF_000155815.1	96	96_1	F6D9_bin.52.fa	F6D9_bin.52.fa	86.18	0.37	24
F68D_bin.38	d__Bacteria	p__Bacteroidota	c__Bacteroidia	o__Bacteroidales	f__Bacteroidaceae	g__Bacteroides	s__Bacteroides fluxus	GCF_000195635.1	97	97_0	F68D_bin.38.fa	F68D_bin.38.fa	69.83	1.72	24

Table S3. Taxonomic classifiation of the 2,421 detected MAGs

	Domain	Phylum	Class	Order	Family	Genus	Species	RefGenome	primary_cluster	secondary_cluster	repr_genome	genome	CheckM_completeness	CheckM_contamination	
F68D_bin.91	d__Bacteria	p__Bacteroidota	c__Bacteroidia	o__Bacteroidales	f__Bacteroidaceae	g__Bacteroides	s__Bacteroides fragilis	GCF_000025985.1	86	86_1	F68V_bin.62.fa	F68D_bin.91.fa	95.02	0	24
F68E_bin.46	d__Bacteria	p__Bacteroidota	c__Bacteroidia	o__Bacteroidales	f__Bacteroidaceae	g__Bacteroides	s__Bacteroides fragilis	GCF_000025985.1	86	86_1	F68V_bin.62.fa	F68E_bin.46.fa	78.79	0	24
F68H_bin.34	d__Bacteria	p__Bacteroidota	c__Bacteroidia	o__Bacteroidales	f__Bacteroidaceae	g__Bacteroides	s__Bacteroides fragilis	GCF_000025985.1	86	86_1	F68V_bin.62.fa	F68H_bin.34.fa	97.91	0	24
F68K_bin.102	d__Bacteria	p__Bacteroidota	c__Bacteroidia	o__Bacteroidales	f__Bacteroidaceae	g__Bacteroides	s__Bacteroides fragilis	GCF_000025985.1	86	86_1	F68V_bin.62.fa	F68K_bin.102.fa	67.45	2.42	24
F68M_bin.17	d__Bacteria	p__Bacteroidota	c__Bacteroidia	o__Bacteroidales	f__Bacteroidaceae	g__Bacteroides	s__Bacteroides fragilis	GCF_000025985.1	86	86_1	F68V_bin.62.fa	F68M_bin.17.fa	92.57	0	24
F68N_bin.70	d__Bacteria	p__Bacteroidota	c__Bacteroidia	o__Bacteroidales	f__Bacteroidaceae	g__Bacteroides	s__Bacteroides fragilis	GCF_000025985.1	86	86_1	F68V_bin.62.fa	F68N_bin.70.fa	90.32	0	24
F68U_bin.9	d__Bacteria	p__Bacteroidota	c__Bacteroidia	o__Bacteroidales	f__Bacteroidaceae	g__Bacteroides	s__Bacteroides fragilis	GCF_000025985.1	86	86_1	F68V_bin.62.fa	F68U_bin.9.fa	96.67	0.56	24

Table S3. Taxonomic classifiation of the 2,421 detected MAGs

	Domain	Phylum	Class	Order	Family	Genus	Species	RefGenome	primary_cluster	secondary_cluster	repr_genome	genome	CheckM_completeness	CheckM_contamination	
F68V_bin.62	d__Bacteria	p__Bacteroidota	c__Bacteroidia	o__Bacteroidales	f__Bacteroidaceae	g__Bacteroides	s__Bacteroides fragilis	GCF_000025985.1	86	86_1	F68V_bin.62.fa	F68V_bin.62.fa	98.88	0	24
F6D9_bin.55	d__Bacteria	p__Bacteroidota	c__Bacteroidia	o__Bacteroidales	f__Bacteroidaceae	g__Bacteroides	s__Bacteroides fragilis	GCF_000025985.1	86	86_1	F68V_bin.62.fa	F6D9_bin.55.fa	63.2	0	24
F6MX_bin.114	d__Bacteria	p__Bacteroidota	c__Bacteroidia	o__Bacteroidales	f__Bacteroidaceae	g__Bacteroides	s__Bacteroides fragilis	GCF_000025985.1	86	86_1	F68V_bin.62.fa	F6MX_bin.114.fa	56.9	0	24
F68G_bin.32	d__Bacteria	p__Bacteroidota	c__Bacteroidia	o__Bacteroidales	f__Bacteroidaceae	g__Bacteroides	s__Bacteroides fragilis_A	GCF_002849695.1	87	87_2	F68G_bin.32.fa	F68G_bin.32.fa	62.73	0.37	24
F6DB_bin.57	d__Bacteria	p__Bacteroidota	c__Bacteroidia	o__Bacteroidales	f__Bacteroidaceae	g__Bacteroides	s__Bacteroides fragilis_A	GCF_002849695.1	87	87_1	F6DB_bin.57.fa	F6DB_bin.57.fa	67.59	0	24
F6DE_bin.89	d__Bacteria	p__Bacteroidota	c__Bacteroidia	o__Bacteroidales	f__Bacteroidaceae	g__Bacteroides	s__Bacteroides fragilis_A	GCF_002849695.1	87	87_1	F6DB_bin.57.fa	F6DE_bin.89.fa	53.45	0.86	24
F68M_bin.27	d__Bacteria	p__Bacteroidota	c__Bacteroidia	o__Bacteroidales	f__Bacteroidaceae	g__Bacteroides	s__Bacteroides intestinalis	GCF_000172175.1	101	101_1	F68M_bin.27.fa	F68M_bin.27.fa	90.64	4.01	24

Table S3. Taxonomic classifiation of the 2,421 detected MAGs

	Domain	Phylum	Class	Order	Family	Genus	Species	RefGenome	primary_cluster	secondary_cluster	repr_genome	genome	CheckM_completeness	CheckM_contamination	
F6DB_bin.60	d__Bacteria	p__Bacteroidota	c__Bacteroidia	o__Bacteroidales	f__Bacteroidaceae	g__Bacteroides	s__Bacteroides intestinalis	GCF_000172175.1	101	101_1	F68M_bin.27.fa	F6DB_bin.60.fa	70.67	1.68	24
F6MX_bin.109	d__Bacteria	p__Bacteroidota	c__Bacteroidia	o__Bacteroidales	f__Bacteroidaceae	g__Bacteroides	s__Bacteroides intestinalis	GCF_000172175.1	101	101_1	F68M_bin.27.fa	F6MX_bin.109.fa	59.64	3.61	24
F6DG_bin.16	d__Bacteria	p__Bacteroidota	c__Bacteroidia	o__Bacteroidales	f__Bacteroidaceae	g__Bacteroides	s__Bacteroides nordii	GCF_000613465.1	103	103_0	F6DG_bin.16.fa	F6DG_bin.16.fa	58.62	1.72	24
F68F_bin.94	d__Bacteria	p__Bacteroidota	c__Bacteroidia	o__Bacteroidales	f__Bacteroidaceae	g__Bacteroides	s__Bacteroides ovatus	GCF_001314995.1	94	94_3	F68F_bin.94.fa	F68F_bin.94.fa	56.03	1.72	24
F6DE_bin.15	d__Bacteria	p__Bacteroidota	c__Bacteroidia	o__Bacteroidales	f__Bacteroidaceae	g__Bacteroides	s__Bacteroides salyersiae	GCF_000381365.1	102	102_0	F6DE_bin.15.fa	F6DE_bin.15.fa	94.18	3.55	24
F68C_bin.20	d__Bacteria	p__Bacteroidota	c__Bacteroidia	o__Bacteroidales	f__Bacteroidaceae	g__Bacteroides	s__Bacteroides stercoris	GCF_000154525.1	95	95_1	F68C_bin.20.fa	F68C_bin.20.fa	91.25	0.74	24
F68F_bin.78	d__Bacteria	p__Bacteroidota	c__Bacteroidia	o__Bacteroidales	f__Bacteroidaceae	g__Bacteroides	s__Bacteroides stercoris	GCF_000154525.1	95	95_1	F68C_bin.20.fa	F68F_bin.78.fa	76.21	0	24
F68K_bin.70	d__Bacteria	p__Bacteroidota	c__Bacteroidia	o__Bacteroidales	f__Bacteroidaceae	g__Bacteroides	s__Bacteroides stercoris	GCF_000154525.1	95	95_1	F68C_bin.20.fa	F68K_bin.70.fa	76.9	1.72	24

Table S3. Taxonomic classifiation of the 2,421 detected MAGs

	Domain	Phylum	Class	Order	Family	Genus	Species	RefGenome	primary_cluster	secondary_cluster	repr_genome	genome	CheckM_completeness	CheckM_contamination	
F6DB_bin.41	d__Bacteria	p__Bacteroidota	c__Bacteroidia	o__Bacteroidales	f__Bacteroidaceae	g__Bacteroides	s__Bacteroides stercoris	GCF_000154525.1	95	95_1	F68C_bin.20.fa	F6DB_bin.41.fa	58.1	3.45	24
F6MY_bin.95	d__Bacteria	p__Bacteroidota	c__Bacteroidia	o__Bacteroidales	f__Bacteroidaceae	g__Bacteroides	s__Bacteroides stercoris	GCF_000154525.1	95	95_1	F68C_bin.20.fa	F6MY_bin.95.fa	52.07	0	24
F68E_bin.16	d__Bacteria	p__Bacteroidota	c__Bacteroidia	o__Bacteroidales	f__Bacteroidaceae	g__Bacteroides	s__Bacteroides stercoris	GCF_000154525.1	95	95_3	F68E_bin.16.fa	F68E_bin.16.fa	65.86	1.72	24
F68R_bin.57	d__Bacteria	p__Bacteroidota	c__Bacteroidia	o__Bacteroidales	f__Bacteroidaceae	g__Bacteroides	s__Bacteroides stercoris	GCF_000154525.1	95	95_2	F68T_bin.19.fa	F68R_bin.57.fa	88.07	2.48	24
F68T_bin.19	d__Bacteria	p__Bacteroidota	c__Bacteroidia	o__Bacteroidales	f__Bacteroidaceae	g__Bacteroides	s__Bacteroides stercoris	GCF_000154525.1	95	95_2	F68T_bin.19.fa	F68T_bin.19.fa	86.82	1.24	24
F68E_bin.18	d__Bacteria	p__Bacteroidota	c__Bacteroidia	o__Bacteroidales	f__Bacteroidaceae	g__Bacteroides	s__Bacteroides thetaiotaomicron	GCF_000011065.1	93	93_1	F68E_bin.18.fa	F68E_bin.18.fa	97.28	4.88	24
F6DE_bin.83	d__Bacteria	p__Bacteroidota	c__Bacteroidia	o__Bacteroidales	f__Bacteroidaceae	g__Bacteroides	s__Bacteroides thetaiotaomicron	GCF_000011065.1	93	93_2	F6DE_bin.83.fa	F6DE_bin.83.fa	88.02	2.56	24

Table S3. Taxonomic classifiation of the 2,421 detected MAGs

	Domain	Phylum	Class	Order	Family	Genus	Species	RefGenome	primary_cluster	secondary_cluster	repr_genome	genome	CheckM_completeness	CheckM_contamination	
F68D_bin.19	d__Bacteria	p__Bacteroidota	c__Bacteroidia	o__Bacteroidales	f__Bacteroidaceae	g__Bacteroides	s__Bacteroides uniformis	GCF_000154205.1	98	98_2	F68D_bin.19.fa	F68D_bin.19.fa	95.74	0	24
F6DE_bin.20	d__Bacteria	p__Bacteroidota	c__Bacteroidia	o__Bacteroidales	f__Bacteroidaceae	g__Bacteroides	s__Bacteroides uniformis	GCF_000154205.1	98	98_2	F68D_bin.19.fa	F6DE_bin.20.fa	52.5	1.77	24
F68E_bin.40	d__Bacteria	p__Bacteroidota	c__Bacteroidia	o__Bacteroidales	f__Bacteroidaceae	g__Bacteroides	s__Bacteroides uniformis	GCF_000154205.1	98	98_6	F68E_bin.40.fa	F68E_bin.40.fa	67.41	0.86	24
F68H_bin.68	d__Bacteria	p__Bacteroidota	c__Bacteroidia	o__Bacteroidales	f__Bacteroidaceae	g__Bacteroides	s__Bacteroides uniformis	GCF_000154205.1	98	98_4	F68H_bin.68.fa	F68H_bin.68.fa	58.62	0	24
F68F_bin.73	d__Bacteria	p__Bacteroidota	c__Bacteroidia	o__Bacteroidales	f__Bacteroidaceae	g__Bacteroides	s__Bacteroides uniformis	GCF_000154205.1	98	98_3	F68T_bin.69.fa	F68F_bin.73.fa	97.53	0.64	24
F68N_bin.59	d__Bacteria	p__Bacteroidota	c__Bacteroidia	o__Bacteroidales	f__Bacteroidaceae	g__Bacteroides	s__Bacteroides uniformis	GCF_000154205.1	98	98_3	F68T_bin.69.fa	F68N_bin.59.fa	81.38	0	24
F68T_bin.69	d__Bacteria	p__Bacteroidota	c__Bacteroidia	o__Bacteroidales	f__Bacteroidaceae	g__Bacteroides	s__Bacteroides uniformis	GCF_000154205.1	98	98_3	F68T_bin.69.fa	F68T_bin.69.fa	94.53	0	24

Table S3. Taxonomic classifiation of the 2,421 detected MAGs

	Domain	Phylum	Class	Order	Family	Genus	Species	RefGenome	primary_cluster	secondary_cluster	repr_genome	genome	CheckM_completeness	CheckM_contamination	
F68U_bin.102	d__Bacteria	p__Bacteroidota	c__Bacteroidia	o__Bacteroidales	f__Bacteroidaceae	g__Bacteroides	s__Bacteroides uniformis	GCF_000154205.1	98	98_3	F68T_bin.69.fa	F68U_bin.102.fa	95.54	0.57	24
F6D7_bin.53	d__Bacteria	p__Bacteroidota	c__Bacteroidia	o__Bacteroidales	f__Bacteroidaceae	g__Bacteroides	s__Bacteroides uniformis	GCF_000154205.1	98	98_3	F68T_bin.69.fa	F6D7_bin.53.fa	61.78	3.45	24
F6DB_bin.26	d__Bacteria	p__Bacteroidota	c__Bacteroidia	o__Bacteroidales	f__Bacteroidaceae	g__Bacteroides	s__Bacteroides uniformis	GCF_000154205.1	98	98_3	F68T_bin.69.fa	F6DB_bin.26.fa	60.78	3.09	24
F68V_bin.75	d__Bacteria	p__Bacteroidota	c__Bacteroidia	o__Bacteroidales	f__Bacteroidaceae	g__Bacteroides	s__Bacteroides uniformis	GCF_000154205.1	98	98_1	F68V_bin.75.fa	F68V_bin.75.fa	89.46	0.42	24
F6MX_bin.83	d__Bacteria	p__Bacteroidota	c__Bacteroidia	o__Bacteroidales	f__Bacteroidaceae	g__Bacteroides	s__Bacteroides uniformis	GCF_000154205.1	98	98_1	F68V_bin.75.fa	F6MX_bin.83.fa	60.39	2.82	24
F6DA_bin.92	d__Bacteria	p__Bacteroidota	c__Bacteroidia	o__Bacteroidales	f__Bacteroidaceae	g__Bacteroides	s__Bacteroides uniformis	GCF_000154205.1	98	98_5	F6DA_bin.92.fa	F6DA_bin.92.fa	83.1	1.72	24
F6MY_bin.109	d__Bacteria	p__Bacteroidota	c__Bacteroidia	o__Bacteroidales	f__Bacteroidaceae	g__Bacteroides	s__Bacteroides uniformis	GCF_000154205.1	98	98_7	F6MY_bin.109.fa	F6MY_bin.109.fa	65.52	1.72	24

Table S3. Taxonomic classifiation of the 2,421 detected MAGs

	Domain	Phylum	Class	Order	Family	Genus	Species	RefGenome	primary_cluster	secondary_cluster	repr_genome	genome	CheckM_completeness	CheckM_contamination	
F68T_bin.34	d__Bacteria	p__Bacteroidota	c__Bacteroidia	o__Bacteroidales	f__Bacteroidaceae	g__Bacteroides	s__Bacteroides xylanisolvens	GCA_000210075.1	94	94_1	F68T_bin.34.fa	F68T_bin.34.fa	68.77	0.16	24
F6MX_bin.9	d__Bacteria	p__Bacteroidota	c__Bacteroidia	o__Bacteroidales	f__Bacteroidaceae	g__Bacteroides	s__Bacteroides xylanisolvens	GCA_000210075.1	94	94_2	F6MX_bin.9.fa	F6MX_bin.9.fa	60.34	1.72	24
F6D9_bin.56	d__Bacteria	p__Bacteroidota	c__Bacteroidia	o__Bacteroidales	f__Bacteroidaceae	g__Bacteroides_A	s__	N/A	91	91_0	F6D9_bin.56.fa	F6D9_bin.56.fa	53.23	0	25
F68K_bin.99	d__Bacteria	p__Bacteroidota	c__Bacteroidia	o__Bacteroidales	f__Bacteroidaceae	g__Bacteroides_A	s__Bacteroides_A coprocola	GCF_000154845.1	89	89_1	F6MY_bin.52.fa	F68K_bin.99.fa	92.01	0	25
F6MY_bin.52	d__Bacteria	p__Bacteroidota	c__Bacteroidia	o__Bacteroidales	f__Bacteroidaceae	g__Bacteroides_A	s__Bacteroides_A coprocola	GCF_000154845.1	89	89_1	F6MY_bin.52.fa	F6MY_bin.52.fa	97.41	0	25
F68F_bin.90	d__Bacteria	p__Bacteroidota	c__Bacteroidia	o__Bacteroidales	f__Bacteroidaceae	g__Bacteroides_A	s__Bacteroides_A plebeius_A	GCA_000433715.1	90	90_2	F68F_bin.90.fa	F68F_bin.90.fa	98.88	0.37	25
F6DA_bin.11	d__Bacteria	p__Bacteroidota	c__Bacteroidia	o__Bacteroidales	f__Bacteroidaceae	g__Bacteroides_A	s__Bacteroides_A plebeius_A	GCA_000433715.1	90	90_1	F6DA_bin.11.fa	F6DA_bin.11.fa	77.87	1.37	25

Table S3. Taxonomic classifiation of the 2,421 detected MAGs

	Domain	Phylum	Class	Order	Family	Genus	Species	RefGenome	primary_cluster	secondary_cluster	repr_genome	genome	CheckM_completeness	CheckM_contamination	
F6DG_bin.11	d__Bacteria	p__Bacteroidota	c__Bacteroidia	o__Bacteroidales	f__Bacteroidaceae	g__Bacteroides_A	s__Bacteroides_A plebeius_A	GCA_000433715.1	90	90_1	F6DA_bin.11.fa	F6DG_bin.11.fa	56.63	3.37	25
F68J_bin.45	d__Bacteria	p__Bacteroidota	c__Bacteroidia	o__Bacteroidales	f__Bacteroidaceae	g__Bacteroides_A	s__Bacteroides_A sp000434735	GCA_000434735.1	88	88_1	F68J_bin.45.fa	F68J_bin.45.fa	94.8	0.04	25
F6DH_bin.112	d__Bacteria	p__Bacteroidota	c__Bacteroidia	o__Bacteroidales	f__Bacteroidaceae	g__Bacteroides_A	s__Bacteroides_A sp000434735	GCA_000434735.1	88	88_1	F68J_bin.45.fa	F6DH_bin.112.fa	89.41	2.85	25
F68E_bin.42	d__Bacteria	p__Bacteroidota	c__Bacteroidia	o__Bacteroidales	f__Bacteroidaceae	g__Bacteroides_B	s__Bacteroides_B massiliensis	GCF_000382445.1	99	99_1	F68E_bin.42.fa	F68E_bin.42.fa	96.94	0.56	25
F6MX_bin.102	d__Bacteria	p__Bacteroidota	c__Bacteroidia	o__Bacteroidales	f__Bacteroidaceae	g__Bacteroides_B	s__Bacteroides_B massiliensis	GCF_000382445.1	99	99_1	F68E_bin.42.fa	F6MX_bin.102.fa	89.65	0.19	25
F68C_bin.85	d__Bacteria	p__Bacteroidota	c__Bacteroidia	o__Bacteroidales	f__Bacteroidaceae	g__Bacteroides_B	s__Bacteroides_B vulgatus	GCF_000012825.1	100	100_1	F68H_bin.1.fa	F68C_bin.85.fa	56.9	0	25

Table S3. Taxonomic classifiation of the 2,421 detected MAGs

	Domain	Phylum	Class	Order	Family	Genus	Species	RefGenome	primary_cluster	secondary_cluster	repr_genome	genome	CheckM_completeness	CheckM_contamination	
F68D_bin.95	d__Bacteria	p__Bacteroidota	c__Bacteroidia	o__Bacteroidales	f__Bacteroidaceae	g__Bacteroides_B	s__Bacteroides_B vulgatus	GCF_000012825.1	100	100_1	F68H_bin.1.fa	F68D_bin.95.fa	82.76	1.72	25
F68E_bin.26	d__Bacteria	p__Bacteroidota	c__Bacteroidia	o__Bacteroidales	f__Bacteroidaceae	g__Bacteroides_B	s__Bacteroides_B vulgatus	GCF_000012825.1	100	100_1	F68H_bin.1.fa	F68E_bin.26.fa	94.85	2.26	25
F68F_bin.72	d__Bacteria	p__Bacteroidota	c__Bacteroidia	o__Bacteroidales	f__Bacteroidaceae	g__Bacteroides_B	s__Bacteroides_B vulgatus	GCF_000012825.1	100	100_1	F68H_bin.1.fa	F68F_bin.72.fa	83.71	0.94	25
F68G_bin.80	d__Bacteria	p__Bacteroidota	c__Bacteroidia	o__Bacteroidales	f__Bacteroidaceae	g__Bacteroides_B	s__Bacteroides_B vulgatus	GCF_000012825.1	100	100_1	F68H_bin.1.fa	F68G_bin.80.fa	98.02	1.17	25
F68H_bin.1	d__Bacteria	p__Bacteroidota	c__Bacteroidia	o__Bacteroidales	f__Bacteroidaceae	g__Bacteroides_B	s__Bacteroides_B vulgatus	GCF_000012825.1	100	100_1	F68H_bin.1.fa	F68H_bin.1.fa	98.5	0	25
F68K_bin.41	d__Bacteria	p__Bacteroidota	c__Bacteroidia	o__Bacteroidales	f__Bacteroidaceae	g__Bacteroides_B	s__Bacteroides_B vulgatus	GCF_000012825.1	100	100_1	F68H_bin.1.fa	F68K_bin.41.fa	94.36	0	25

Table S3. Taxonomic classifiation of the 2,421 detected MAGs

	Domain	Phylum	Class	Order	Family	Genus	Species	RefGenome	primary_cluster	secondary_cluster	repr_genome	genome	CheckM_completeness	CheckM_contamination	
F68M_bin.81	d__Bacteria	p__Bacteroidota	c__Bacteroidia	o__Bacteroidales	f__Bacteroidaceae	g__Bacteroides_B	s__Bacteroides_B vulgatus	GCF_000012825.1	100	100_1	F68H_bin.1.fa	F68M_bin.81.fa	81.87	2.16	25
F68N_bin.25	d__Bacteria	p__Bacteroidota	c__Bacteroidia	o__Bacteroidales	f__Bacteroidaceae	g__Bacteroides_B	s__Bacteroides_B vulgatus	GCF_000012825.1	100	100_1	F68H_bin.1.fa	F68N_bin.25.fa	97.1	1.57	25
F68P_bin.38	d__Bacteria	p__Bacteroidota	c__Bacteroidia	o__Bacteroidales	f__Bacteroidaceae	g__Bacteroides_B	s__Bacteroides_B vulgatus	GCF_000012825.1	100	100_1	F68H_bin.1.fa	F68P_bin.38.fa	96.87	0	25
F68R_bin.97	d__Bacteria	p__Bacteroidota	c__Bacteroidia	o__Bacteroidales	f__Bacteroidaceae	g__Bacteroides_B	s__Bacteroides_B vulgatus	GCF_000012825.1	100	100_1	F68H_bin.1.fa	F68R_bin.97.fa	64.32	0.56	25
F68T_bin.39	d__Bacteria	p__Bacteroidota	c__Bacteroidia	o__Bacteroidales	f__Bacteroidaceae	g__Bacteroides_B	s__Bacteroides_B vulgatus	GCF_000012825.1	100	100_1	F68H_bin.1.fa	F68T_bin.39.fa	65.52	0	25
F68U_bin.54	d__Bacteria	p__Bacteroidota	c__Bacteroidia	o__Bacteroidales	f__Bacteroidaceae	g__Bacteroides_B	s__Bacteroides_B vulgatus	GCF_000012825.1	100	100_1	F68H_bin.1.fa	F68U_bin.54.fa	95.86	0.56	25

Table S3. Taxonomic classifiation of the 2,421 detected MAGs

	Domain	Phylum	Class	Order	Family	Genus	Species	RefGenome	primary_cluster	secondary_cluster	repr_genome	genome	CheckM_completeness	CheckM_contamination	
F68V_bin.99	d__Bacteria	p__Bacteroidota	c__Bacteroidia	o__Bacteroidales	f__Bacteroidaceae	g__Bacteroides_B	s__Bacteroides_B vulgatus	GCF_000012825.1	100	100_1	F68H_bin.1.fa	F68V_bin.99.fa	82.76	1.72	25
F68W_bin.97	d__Bacteria	p__Bacteroidota	c__Bacteroidia	o__Bacteroidales	f__Bacteroidaceae	g__Bacteroides_B	s__Bacteroides_B vulgatus	GCF_000012825.1	100	100_1	F68H_bin.1.fa	F68W_bin.97.fa	64.66	0.86	25
F6D7_bin.71	d__Bacteria	p__Bacteroidota	c__Bacteroidia	o__Bacteroidales	f__Bacteroidaceae	g__Bacteroides_B	s__Bacteroides_B vulgatus	GCF_000012825.1	100	100_1	F68H_bin.1.fa	F6D7_bin.71.fa	51.72	2.59	25
F6D8_bin.31	d__Bacteria	p__Bacteroidota	c__Bacteroidia	o__Bacteroidales	f__Bacteroidaceae	g__Bacteroides_B	s__Bacteroides_B vulgatus	GCF_000012825.1	100	100_1	F68H_bin.1.fa	F6D8_bin.31.fa	89.84	3.76	25
F6DE_bin.61	d__Bacteria	p__Bacteroidota	c__Bacteroidia	o__Bacteroidales	f__Bacteroidaceae	g__Bacteroides_B	s__Bacteroides_B vulgatus	GCF_000012825.1	100	100_1	F68H_bin.1.fa	F6DE_bin.61.fa	67.24	0	25
F6MY_bin.44	d__Bacteria	p__Bacteroidota	c__Bacteroidia	o__Bacteroidales	f__Bacteroidaceae	g__Bacteroides_B	s__Bacteroides_B vulgatus	GCF_000012825.1	100	100_1	F68H_bin.1.fa	F6MY_bin.44.fa	94.36	0.01	25

Table S3. Taxonomic classifiation of the 2,421 detected MAGs

	Domain	Phylum	Class	Order	Family	Genus	Species	RefGenome	primary_cluster	secondary_cluster	repr_genome	genome	CheckM_completeness	CheckM_contamination	
F68F_bin.36	d__Bacteria	p__Bacteroidota	c__Bacteroidia	o__Bacteroidales	f__Bacteroidaceae	g__CAG-617	s__	N/A	22	22_0	F68F_bin.36.fa	F68F_bin.36.fa	52.19	3.16	26
F68K_bin.22	d__Bacteria	p__Bacteroidota	c__Bacteroidia	o__Bacteroidales	f__Bacteroidaceae	g__Paraprevotella	s__	N/A	286	286_0	F68K_bin.22.fa	F68K_bin.22.fa	72.24	0	27
F68E_bin.27	d__Bacteria	p__Bacteroidota	c__Bacteroidia	o__Bacteroidales	f__Bacteroidaceae	g__Paraprevotella	s__Paraprevotella clara	GCF_000233955.1	73	73_6	F68E_bin.27.fa	F68E_bin.27.fa	98.4	0	27
F68T_bin.102	d__Bacteria	p__Bacteroidota	c__Bacteroidia	o__Bacteroidales	f__Bacteroidaceae	g__Paraprevotella	s__Paraprevotella clara	GCF_000233955.1	73	73_4	F68T_bin.102.fa	F68T_bin.102.fa	97.7	0.68	27
F6DA_bin.64	d__Bacteria	p__Bacteroidota	c__Bacteroidia	o__Bacteroidales	f__Bacteroidaceae	g__Paraprevotella	s__Paraprevotella clara	GCF_000233955.1	73	73_7	F6DA_bin.64.fa	F6DA_bin.64.fa	94.06	0.56	27
F68P_bin.45	d__Bacteria	p__Bacteroidota	c__Bacteroidia	o__Bacteroidales	f__Bacteroidaceae	g__Paraprevotella	s__Paraprevotella clara	GCF_000233955.1	73	73_3	F6DG_bin.83.fa	F68P_bin.45.fa	85.92	3.14	27
F6DG_bin.83	d__Bacteria	p__Bacteroidota	c__Bacteroidia	o__Bacteroidales	f__Bacteroidaceae	g__Paraprevotella	s__Paraprevotella clara	GCF_000233955.1	73	73_3	F6DG_bin.83.fa	F6DG_bin.83.fa	96.91	0.19	27
F6MY_bin.20	d__Bacteria	p__Bacteroidota	c__Bacteroidia	o__Bacteroidales	f__Bacteroidaceae	g__Paraprevotella	s__Paraprevotella clara	GCF_000233955.1	73	73_5	F6MY_bin.20.fa	F6MY_bin.20.fa	60.34	0	27

Table S3. Taxonomic classifiation of the 2,421 detected MAGs

	Domain	Phylum	Class	Order	Family	Genus	Species	RefGenome	primary_cluster	secondary_cluster	repr_genome	genome	CheckM_completeness	CheckM_contamination	
F6D8_bin.41	d__Bacteria	p__Bacteroidota	c__Bacteroidia	o__Bacteroidales	f__Bacteroidaceae	g__Paraprevotella	s__Paraprevotella xylaniphila	GCF_000205165.1	73	73_1	F6D8_bin.41.fa	F6D8_bin.41.fa	96.26	2.08	27
F6DE_bin.84	d__Bacteria	p__Bacteroidota	c__Bacteroidia	o__Bacteroidales	f__Bacteroidaceae	g__Paraprevotella	s__Paraprevotella xylaniphila	GCF_000205165.1	73	73_2	F6DE_bin.84.fa	F6DE_bin.84.fa	98.76	0.74	27
F68T_bin.87	d__Bacteria	p__Bacteroidota	c__Bacteroidia	o__Bacteroidales	f__Bacteroidaceae	g__Prevotella	s__	N/A	15	15_0	F68T_bin.87.fa	F68T_bin.87.fa	55.93	0.19	28
F68U_bin.72	d__Bacteria	p__Bacteroidota	c__Bacteroidia	o__Bacteroidales	f__Bacteroidaceae	g__Prevotella	s__	N/A	46	46_0	F68U_bin.72.fa	F68U_bin.72.fa	59.29	0	29
F6D8_bin.73	d__Bacteria	p__Bacteroidota	c__Bacteroidia	o__Bacteroidales	f__Bacteroidaceae	g__Prevotella	s__	N/A	48	48_2	F6D8_bin.73.fa	F6D8_bin.73.fa	68.1	1.89	30
F68C_bin.108	d__Bacteria	p__Bacteroidota	c__Bacteroidia	o__Bacteroidales	f__Bacteroidaceae	g__Prevotella	s__	N/A	48	48_1	F68C_bin.108.fa	F68C_bin.108.fa	66.71	0.84	31
F6D9_bin.58	d__Bacteria	p__Bacteroidota	c__Bacteroidia	o__Bacteroidales	f__Bacteroidaceae	g__Prevotella	s__	N/A	48	48_3	F6D9_bin.58.fa	F6D9_bin.58.fa	50.86	3.45	32
F68H_bin.62	d__Bacteria	p__Bacteroidota	c__Bacteroidia	o__Bacteroidales	f__Bacteroidaceae	g__Prevotella	s__	N/A	49	49_1	F68H_bin.62.fa	F68H_bin.62.fa	63.81	1.1	33
F68K_bin.100	d__Bacteria	p__Bacteroidota	c__Bacteroidia	o__Bacteroidales	f__Bacteroidaceae	g__Prevotella	s__	N/A	49	49_2	F68K_bin.100.fa	F68K_bin.100.fa	85.69	2.36	34

Table S3. Taxonomic classifiation of the 2,421 detected MAGs

	Domain	Phylum	Class	Order	Family	Genus	Species	RefGenome	primary_cluster	secondary_cluster	repr_genome	genome	CheckM_completeness	CheckM_contamination	
F68P_bin.27	d__Bacteria	p__Bacteroidota	c__Bacteroidia	o__Bacteroidales	f__Bacteroidaceae	g__Prevotella	s__	N/A	50	50_1	F68P_bin.27.fa	F68P_bin.27.fa	53.02	2.12	35
F68W_bin.71	d__Bacteria	p__Bacteroidota	c__Bacteroidia	o__Bacteroidales	f__Bacteroidaceae	g__Prevotella	s__	N/A	50	50_2	F68W_bin.71.fa	F68W_bin.71.fa	53.45	1.72	36
F68P_bin.57	d__Bacteria	p__Bacteroidota	c__Bacteroidia	o__Bacteroidales	f__Bacteroidaceae	g__Prevotella	s__	N/A	51	51_0	F68P_bin.57.fa	F68P_bin.57.fa	54.31	1.72	37
F68K_bin.36	d__Bacteria	p__Bacteroidota	c__Bacteroidia	o__Bacteroidales	f__Bacteroidaceae	g__Prevotella	s__	N/A	57	57_1	F6MY_bin.117.fa	F68K_bin.36.fa	75.06	0.37	38
F6D8_bin.28	d__Bacteria	p__Bacteroidota	c__Bacteroidia	o__Bacteroidales	f__Bacteroidaceae	g__Prevotella	s__	N/A	57	57_1	F6MY_bin.117.fa	F6D8_bin.28.fa	81.2	0.37	39
F6MY_bin.117	d__Bacteria	p__Bacteroidota	c__Bacteroidia	o__Bacteroidales	f__Bacteroidaceae	g__Prevotella	s__	N/A	57	57_1	F6MY_bin.117.fa	F6MY_bin.117.fa	87.23	0.93	40
F68D_bin.113	d__Bacteria	p__Bacteroidota	c__Bacteroidia	o__Bacteroidales	f__Bacteroidaceae	g__Prevotella	s__	N/A	59	59_1	F68D_bin.113.fa	F68D_bin.113.fa	79.48	3.98	41
F68G_bin.38	d__Bacteria	p__Bacteroidota	c__Bacteroidia	o__Bacteroidales	f__Bacteroidaceae	g__Prevotella	s__	N/A	59	59_2	F68G_bin.38.fa	F68G_bin.38.fa	78.84	0.37	42
F68H_bin.11	d__Bacteria	p__Bacteroidota	c__Bacteroidia	o__Bacteroidales	f__Bacteroidaceae	g__Prevotella	s__	N/A	61	61_4	F68H_bin.11.fa	F68H_bin.11.fa	90.38	1.11	43
F68K_bin.49	d__Bacteria	p__Bacteroidota	c__Bacteroidia	o__Bacteroidales	f__Bacteroidaceae	g__Prevotella	s__	N/A	61	61_2	F68K_bin.49.fa	F68K_bin.49.fa	92.36	0	44

Table S3. Taxonomic classifiation of the 2,421 detected MAGs

	Domain	Phylum	Class	Order	Family	Genus	Species	RefGenome	primary_cluster	secondary_cluster	repr_genome	genome	CheckM_completeness	CheckM_contamination	
F6D7_bin.57	d__Bacteria	p__Bacteroidota	c__Bacteroidia	o__Bacteroidales	f__Bacteroidaceae	g__Prevotella	s__	N/A	61	61_2	F68K_bin.49.fa	F6D7_bin.57.fa	73.28	1.72	45
F68N_bin.66	d__Bacteria	p__Bacteroidota	c__Bacteroidia	o__Bacteroidales	f__Bacteroidaceae	g__Prevotella	s__	N/A	61	61_5	F68N_bin.66.fa	F68N_bin.66.fa	93.13	0.98	46
F68R_bin.37	d__Bacteria	p__Bacteroidota	c__Bacteroidia	o__Bacteroidales	f__Bacteroidaceae	g__Prevotella	s__	N/A	61	61_3	F68R_bin.37.fa	F68R_bin.37.fa	90.79	0.05	47
F68U_bin.46	d__Bacteria	p__Bacteroidota	c__Bacteroidia	o__Bacteroidales	f__Bacteroidaceae	g__Prevotella	s__	N/A	61	61_1	F6DH_bin.52.fa	F68U_bin.46.fa	81.19	1.72	48
F68W_bin.5	d__Bacteria	p__Bacteroidota	c__Bacteroidia	o__Bacteroidales	f__Bacteroidaceae	g__Prevotella	s__	N/A	61	61_1	F6DH_bin.52.fa	F68W_bin.5.fa	61.32	1.72	49
F6DH_bin.52	d__Bacteria	p__Bacteroidota	c__Bacteroidia	o__Bacteroidales	f__Bacteroidaceae	g__Prevotella	s__	N/A	61	61_1	F6DH_bin.52.fa	F6DH_bin.52.fa	93.45	2.81	50
F68K_bin.96	d__Bacteria	p__Bacteroidota	c__Bacteroidia	o__Bacteroidales	f__Bacteroidaceae	g__Prevotella	s__	N/A	63	63_2	F68K_bin.96.fa	F68K_bin.96.fa	91.13	0.74	51
F68P_bin.8	d__Bacteria	p__Bacteroidota	c__Bacteroidia	o__Bacteroidales	f__Bacteroidaceae	g__Prevotella	s__	N/A	63	63_3	F68P_bin.8.fa	F68P_bin.8.fa	82.57	1.25	52
F68U_bin.109	d__Bacteria	p__Bacteroidota	c__Bacteroidia	o__Bacteroidales	f__Bacteroidaceae	g__Prevotella	s__	N/A	63	63_1	F68W_bin.25.fa	F68U_bin.109.fa	68.84	0.37	53
F68W_bin.25	d__Bacteria	p__Bacteroidota	c__Bacteroidia	o__Bacteroidales	f__Bacteroidaceae	g__Prevotella	s__	N/A	63	63_1	F68W_bin.25.fa	F68W_bin.25.fa	84.76	1.11	54

Table S3. Taxonomic classifiation of the 2,421 detected MAGs

	Domain	Phylum	Class	Order	Family	Genus	Species	RefGenome	primary_cluster	secondary_cluster	repr_genome	genome	CheckM_completeness	CheckM_contamination	
F6DH_bin.124	d__Bacteria	p__Bacteroidota	c__Bacteroidia	o__Bacteroidales	f__Bacteroidaceae	g__Prevotella	s__	N/A	65	65_0	F6DH_bin.124.fa	F6DH_bin.124.fa	59.04	3.92	55
F68J_bin.11	d__Bacteria	p__Bacteroidota	c__Bacteroidia	o__Bacteroidales	f__Bacteroidaceae	g__Prevotella	s__	N/A	67	67_2	F68J_bin.11.fa	F68J_bin.11.fa	98.8	0.19	56
F68U_bin.95	d__Bacteria	p__Bacteroidota	c__Bacteroidia	o__Bacteroidales	f__Bacteroidaceae	g__Prevotella	s__	N/A	67	67_1	F6DH_bin.37.fa	F68U_bin.95.fa	87.69	0.93	57
F68W_bin.27	d__Bacteria	p__Bacteroidota	c__Bacteroidia	o__Bacteroidales	f__Bacteroidaceae	g__Prevotella	s__	N/A	67	67_1	F6DH_bin.37.fa	F68W_bin.27.fa	87.64	1.02	58
F6DH_bin.37	d__Bacteria	p__Bacteroidota	c__Bacteroidia	o__Bacteroidales	f__Bacteroidaceae	g__Prevotella	s__	N/A	67	67_1	F6DH_bin.37.fa	F6DH_bin.37.fa	89.25	0.23	59
F68E_bin.13	d__Bacteria	p__Bacteroidota	c__Bacteroidia	o__Bacteroidales	f__Bacteroidaceae	g__Prevotella	s__Prevotella copri	GCF_000157935.1	47	47_4	F68E_bin.13.fa	F68E_bin.13.fa	94.96	1.35	59
F68K_bin.90	d__Bacteria	p__Bacteroidota	c__Bacteroidia	o__Bacteroidales	f__Bacteroidaceae	g__Prevotella	s__Prevotella copri	GCF_000157935.1	47	47_8	F68K_bin.90.fa	F68K_bin.90.fa	76.29	0.84	59
F68P_bin.13	d__Bacteria	p__Bacteroidota	c__Bacteroidia	o__Bacteroidales	f__Bacteroidaceae	g__Prevotella	s__Prevotella copri	GCF_000157935.1	47	47_1	F68P_bin.13.fa	F68P_bin.13.fa	53.61	0	59
F6D9_bin.29	d__Bacteria	p__Bacteroidota	c__Bacteroidia	o__Bacteroidales	f__Bacteroidaceae	g__Prevotella	s__Prevotella copri	GCF_000157935.1	47	47_2	F6D9_bin.29.fa	F6D9_bin.29.fa	93.27	1.98	59
F6DA_bin.50	d__Bacteria	p__Bacteroidota	c__Bacteroidia	o__Bacteroidales	f__Bacteroidaceae	g__Prevotella	s__Prevotella copri	GCF_000157935.1	47	47_6	F6DA_bin.50.fa	F6DA_bin.50.fa	58.62	0	59

Table S3. Taxonomic classifiation of the 2,421 detected MAGs

	Domain	Phylum	Class	Order	Family	Genus	Species	RefGenome	primary_cluster	secondary_cluster	repr_genome	genome	CheckM_completeness	CheckM_contamination	
F6DB_bin.80	d__Bacteria	p__Bacteroidota	c__Bacteroidia	o__Bacteroidales	f__Bacteroidaceae	g__Prevotella	s__Prevotella copri	GCF_000157935.1	47	47_3	F6DB_bin.80.fa	F6DB_bin.80.fa	74.48	0	59
F6DG_bin.82	d__Bacteria	p__Bacteroidota	c__Bacteroidia	o__Bacteroidales	f__Bacteroidaceae	g__Prevotella	s__Prevotella copri	GCF_000157935.1	47	47_7	F6DG_bin.82.fa	F6DG_bin.82.fa	61.72	0.36	59
F6MY_bin.31	d__Bacteria	p__Bacteroidota	c__Bacteroidia	o__Bacteroidales	f__Bacteroidaceae	g__Prevotella	s__Prevotella copri	GCF_000157935.1	47	47_5	F6MY_bin.31.fa	F6MY_bin.31.fa	88.36	0.87	59
F68F_bin.95	d__Bacteria	p__Bacteroidota	c__Bacteroidia	o__Bacteroidales	f__Bacteroidaceae	g__Prevotella	s__Prevotella copri_A	GCF_002224675.1	49	49_5	F68F_bin.95.fa	F68F_bin.95.fa	56.55	1.72	59
F68W_bin.58	d__Bacteria	p__Bacteroidota	c__Bacteroidia	o__Bacteroidales	f__Bacteroidaceae	g__Prevotella	s__Prevotella copri_A	GCF_002224675.1	49	49_3	F68W_bin.58.fa	F68W_bin.58.fa	55.33	0	59
F6DG_bin.78	d__Bacteria	p__Bacteroidota	c__Bacteroidia	o__Bacteroidales	f__Bacteroidaceae	g__Prevotella	s__Prevotella copri_A	GCF_002224675.1	49	49_4	F6DG_bin.78.fa	F6DG_bin.78.fa	55.15	0.79	59
F68F_bin.104	d__Bacteria	p__Bacteroidota	c__Bacteroidia	o__Bacteroidales	f__Bacteroidaceae	g__Prevotella	s__Prevotella sp000434515	GCA_000434515.1	53	53_2	F68F_bin.104.fa	F68F_bin.104.fa	80.44	0.83	59
F68U_bin.77	d__Bacteria	p__Bacteroidota	c__Bacteroidia	o__Bacteroidales	f__Bacteroidaceae	g__Prevotella	s__Prevotella sp000434515	GCA_000434515.1	53	53_1	F68U_bin.77.fa	F68U_bin.77.fa	69.48	0	59
F68W_bin.92	d__Bacteria	p__Bacteroidota	c__Bacteroidia	o__Bacteroidales	f__Bacteroidaceae	g__Prevotella	s__Prevotella sp000434515	GCA_000434515.1	53	53_1	F68U_bin.77.fa	F68W_bin.92.fa	56.9	3.45	59

Table S3. Taxonomic classifiation of the 2,421 detected MAGs

	Domain	Phylum	Class	Order	Family	Genus	Species	RefGenome	primary_cluster	secondary_cluster	repr_genome	genome	CheckM_completeness	CheckM_contamination	
F6DH_bin.42	d__Bacteria	p__Bacteroidota	c__Bacteroidia	o__Bacteroidales	f__Bacteroidaceae	g__Prevotella	s__Prevotella sp000434515	GCA_000434515.1	53	53_1	F68U_bin.77.fa	F6DH_bin.42.fa	60.5	1.72	59
F68E_bin.23	d__Bacteria	p__Bacteroidota	c__Bacteroidia	o__Bacteroidales	f__Bacteroidaceae	g__Prevotella	s__Prevotella sp000434975	GCA_000434975.1	60	60_11	F68E_bin.23.fa	F68E_bin.23.fa	98.66	0	59
F68G_bin.62	d__Bacteria	p__Bacteroidota	c__Bacteroidia	o__Bacteroidales	f__Bacteroidaceae	g__Prevotella	s__Prevotella sp000434975	GCA_000434975.1	60	60_8	F68G_bin.62.fa	F68G_bin.62.fa	91.92	0.74	59
F68F_bin.65	d__Bacteria	p__Bacteroidota	c__Bacteroidia	o__Bacteroidales	f__Bacteroidaceae	g__Prevotella	s__Prevotella sp000434975	GCA_000434975.1	60	60_7	F68H_bin.66.fa	F68F_bin.65.fa	92.42	0	59
F68H_bin.66	d__Bacteria	p__Bacteroidota	c__Bacteroidia	o__Bacteroidales	f__Bacteroidaceae	g__Prevotella	s__Prevotella sp000434975	GCA_000434975.1	60	60_7	F68H_bin.66.fa	F68H_bin.66.fa	97.4	0.74	59
F68R_bin.103	d__Bacteria	p__Bacteroidota	c__Bacteroidia	o__Bacteroidales	f__Bacteroidaceae	g__Prevotella	s__Prevotella sp000434975	GCA_000434975.1	60	60_10	F68R_bin.103.fa	F68R_bin.103.fa	97.18	0.93	59
F6D9_bin.73	d__Bacteria	p__Bacteroidota	c__Bacteroidia	o__Bacteroidales	f__Bacteroidaceae	g__Prevotella	s__Prevotella sp000434975	GCA_000434975.1	60	60_9	F6D9_bin.73.fa	F6D9_bin.73.fa	96.24	0	59

Table S3. Taxonomic classifiation of the 2,421 detected MAGs

	Domain	Phylum	Class	Order	Family	Genus	Species	RefGenome	primary_cluster	secondary_cluster	repr_genome	genome	CheckM_completeness	CheckM_contamination	
F68U_bin.29	d__Bacteria	p__Bacteroidota	c__Bacteroidia	o__Bacteroidales	f__Bacteroidaceae	g__Prevotella	s__Prevotella sp000434975	GCA_000434975.1	60	60_6	F6DH_bin.36.fa	F68U_bin.29.fa	92.75	0.86	59
F68W_bin.10	d__Bacteria	p__Bacteroidota	c__Bacteroidia	o__Bacteroidales	f__Bacteroidaceae	g__Prevotella	s__Prevotella sp000434975	GCA_000434975.1	60	60_6	F6DH_bin.36.fa	F68W_bin.10.fa	94.96	0.37	59
F6DH_bin.36	d__Bacteria	p__Bacteroidota	c__Bacteroidia	o__Bacteroidales	f__Bacteroidaceae	g__Prevotella	s__Prevotella sp000434975	GCA_000434975.1	60	60_6	F6DH_bin.36.fa	F6DH_bin.36.fa	97.26	0.22	59
F68H_bin.58	d__Bacteria	p__Bacteroidota	c__Bacteroidia	o__Bacteroidales	f__Bacteroidaceae	g__Prevotella	s__Prevotella sp000436035	GCA_000436035.1	52	52_1	F68H_bin.58.fa	F68H_bin.58.fa	80.86	4.31	59
F68J_bin.51	d__Bacteria	p__Bacteroidota	c__Bacteroidia	o__Bacteroidales	f__Bacteroidaceae	g__Prevotella	s__Prevotella sp000436035	GCA_000436035.1	52	52_2	F68J_bin.51.fa	F68J_bin.51.fa	94.43	1.86	59
F68J_bin.41	d__Bacteria	p__Bacteroidota	c__Bacteroidia	o__Bacteroidales	f__Bacteroidaceae	g__Prevotella	s__Prevotella sp000436915	GCA_000436915.1	66	66_1	F68J_bin.41.fa	F68J_bin.41.fa	95.31	0.56	59
F68P_bin.20	d__Bacteria	p__Bacteroidota	c__Bacteroidia	o__Bacteroidales	f__Bacteroidaceae	g__Prevotella	s__Prevotella sp000436915	GCA_000436915.1	66	66_2	F68P_bin.20.fa	F68P_bin.20.fa	96.73	4	59

Table S3. Taxonomic classifiation of the 2,421 detected MAGs

	Domain	Phylum	Class	Order	Family	Genus	Species	RefGenome	primary_cluster	secondary_cluster	repr_genome	genome	CheckM_completeness	CheckM_contamination	
F68R_bin.124	d__Bacteria	p__Bacteroidota	c__Bacteroidia	o__Bacteroidales	f__Bacteroidaceae	g__Prevotella	s__Prevotella sp000436915	GCA_000436915.1	66	66_3	F68R_bin.124.fa	F68R_bin.124.fa	91.11	0.19	59
F68K_bin.8	d__Bacteria	p__Bacteroidota	c__Bacteroidia	o__Bacteroidales	f__Bacteroidaceae	g__Prevotella	s__Prevotella sp000834015	GCF_000834015.1	68	68_3	F68K_bin.8.fa	F68K_bin.8.fa	90.08	1.15	59
F68N_bin.45	d__Bacteria	p__Bacteroidota	c__Bacteroidia	o__Bacteroidales	f__Bacteroidaceae	g__Prevotella	s__Prevotella sp000834015	GCF_000834015.1	68	68_1	F68N_bin.45.fa	F68N_bin.45.fa	75.24	0.68	59
F6D9_bin.43	d__Bacteria	p__Bacteroidota	c__Bacteroidia	o__Bacteroidales	f__Bacteroidaceae	g__Prevotella	s__Prevotella sp000834015	GCF_000834015.1	68	68_2	F6D9_bin.43.fa	F6D9_bin.43.fa	79.06	0.37	59
F68J_bin.78	d__Bacteria	p__Bacteroidota	c__Bacteroidia	o__Bacteroidales	f__Bacteroidaceae	g__Prevotella	s__Prevotella sp002251385	GCF_002251385.1	55	55_3	F68J_bin.78.fa	F68J_bin.78.fa	73.67	0.49	59
F68R_bin.122	d__Bacteria	p__Bacteroidota	c__Bacteroidia	o__Bacteroidales	f__Bacteroidaceae	g__Prevotella	s__Prevotella sp002251385	GCF_002251385.1	55	55_2	F68R_bin.122.fa	F68R_bin.122.fa	72.03	0.37	59
F6D9_bin.6	d__Bacteria	p__Bacteroidota	c__Bacteroidia	o__Bacteroidales	f__Bacteroidaceae	g__Prevotella	s__Prevotella sp002251385	GCF_002251385.1	55	55_4	F6D9_bin.6.fa	F6D9_bin.6.fa	76.18	0.49	59

Table S3. Taxonomic classifiation of the 2,421 detected MAGs

	Domain	Phylum	Class	Order	Family	Genus	Species	RefGenome	primary_cluster	secondary_cluster	repr_genome	genome	CheckM_completeness	CheckM_contamination	
F68K_bin.7	d__Bacteria	p__Bacteroidota	c__Bacteroidia	o__Bacteroidales	f__Bacteroidaceae	g__Prevotella	s__Prevotella sp002251385	GCF_002251385.1	55	55_1	F6DH_bin.15.fa	F68K_bin.7.fa	74.6	0	59
F68U_bin.8	d__Bacteria	p__Bacteroidota	c__Bacteroidia	o__Bacteroidales	f__Bacteroidaceae	g__Prevotella	s__Prevotella sp002251385	GCF_002251385.1	55	55_1	F6DH_bin.15.fa	F68U_bin.8.fa	72.41	1.72	59
F68W_bin.74	d__Bacteria	p__Bacteroidota	c__Bacteroidia	o__Bacteroidales	f__Bacteroidaceae	g__Prevotella	s__Prevotella sp002251385	GCF_002251385.1	55	55_1	F6DH_bin.15.fa	F68W_bin.74.fa	73.58	0	59
F6DH_bin.15	d__Bacteria	p__Bacteroidota	c__Bacteroidia	o__Bacteroidales	f__Bacteroidaceae	g__Prevotella	s__Prevotella sp002251385	GCF_002251385.1	55	55_1	F6DH_bin.15.fa	F6DH_bin.15.fa	79.41	0	59
F68D_bin.59	d__Bacteria	p__Bacteroidota	c__Bacteroidia	o__Bacteroidales	f__Bacteroidaceae	g__Prevotella	s__Prevotella sp002265625	GCF_002265625.1	62	62_4	F68D_bin.59.fa	F68D_bin.59.fa	54.75	0	59
F68H_bin.45	d__Bacteria	p__Bacteroidota	c__Bacteroidia	o__Bacteroidales	f__Bacteroidaceae	g__Prevotella	s__Prevotella sp002265625	GCF_002265625.1	62	62_2	F68H_bin.45.fa	F68H_bin.45.fa	86.95	0.57	59
F68P_bin.59	d__Bacteria	p__Bacteroidota	c__Bacteroidia	o__Bacteroidales	f__Bacteroidaceae	g__Prevotella	s__Prevotella sp002265625	GCF_002265625.1	62	62_5	F68P_bin.59.fa	F68P_bin.59.fa	78.11	0.74	59

Table S3. Taxonomic classifiation of the 2,421 detected MAGs

	Domain	Phylum	Class	Order	Family	Genus	Species	RefGenome	primary_cluster	secondary_cluster	repr_genome	genome	CheckM_completeness	CheckM_contamination	
F68U_bin.103	d__Bacteria	p__Bacteroidota	c__Bacteroidia	o__Bacteroidales	f__Bacteroidaceae	g__Prevotella	s__Prevotella sp002265625	GCF_002265625.1	62	62_1	F68U_bin.103.fa	F68U_bin.103.fa	90.2	0.49	59
F68W_bin.70	d__Bacteria	p__Bacteroidota	c__Bacteroidia	o__Bacteroidales	f__Bacteroidaceae	g__Prevotella	s__Prevotella sp002265625	GCF_002265625.1	62	62_1	F68U_bin.103.fa	F68W_bin.70.fa	77.9	3.45	59
F6DH_bin.27	d__Bacteria	p__Bacteroidota	c__Bacteroidia	o__Bacteroidales	f__Bacteroidaceae	g__Prevotella	s__Prevotella sp002265625	GCF_002265625.1	62	62_1	F68U_bin.103.fa	F6DH_bin.27.fa	89.81	2.79	59
F6MX_bin.27	d__Bacteria	p__Bacteroidota	c__Bacteroidia	o__Bacteroidales	f__Bacteroidaceae	g__Prevotella	s__Prevotella sp002265625	GCF_002265625.1	62	62_3	F6MX_bin.27.fa	F6MX_bin.27.fa	84.42	0.49	59
F6D9_bin.45	d__Bacteria	p__Bacteroidota	c__Bacteroidia	o__Bacteroidales	f__Bacteroidaceae	g__Prevotella	s__Prevotella sp002300055	GCA_002300055.1	58	58_0	F6D9_bin.45.fa	F6D9_bin.45.fa	94.43	1.41	59
F68C_bin.74	d__Bacteria	p__Bacteroidota	c__Bacteroidia	o__Bacteroidales	f__Bacteroidaceae	g__Prevotella	s__Prevotella sp003447235	GCA_003447235.1	56	56_2	F68C_bin.74.fa	F68C_bin.74.fa	95.91	0	59
F68D_bin.88	d__Bacteria	p__Bacteroidota	c__Bacteroidia	o__Bacteroidales	f__Bacteroidaceae	g__Prevotella	s__Prevotella sp003447235	GCA_003447235.1	56	56_3	F68G_bin.12.fa	F68D_bin.88.fa	90.57	1.84	59

Table S3. Taxonomic classifiation of the 2,421 detected MAGs

	Domain	Phylum	Class	Order	Family	Genus	Species	RefGenome	primary_cluster	secondary_cluster	repr_genome	genome	CheckM_completeness	CheckM_contamination	
F68G_bin.12	d__Bacteria	p__Bacteroidota	c__Bacteroidia	o__Bacteroidales	f__Bacteroidaceae	g__Prevotella	s__Prevotella sp003447235	GCA_003447235.1	56	56_3	F68G_bin.12.fa	F68G_bin.12.fa	95.43	1.19	59
F68P_bin.51	d__Bacteria	p__Bacteroidota	c__Bacteroidia	o__Bacteroidales	f__Bacteroidaceae	g__Prevotella	s__Prevotella sp003447235	GCA_003447235.1	56	56_3	F68G_bin.12.fa	F68P_bin.51.fa	69.75	0	59
F68J_bin.22	d__Bacteria	p__Bacteroidota	c__Bacteroidia	o__Bacteroidales	f__Bacteroidaceae	g__Prevotella	s__Prevotella sp003447235	GCA_003447235.1	56	56_4	F68J_bin.22.fa	F68J_bin.22.fa	95.77	0	59
F68M_bin.67	d__Bacteria	p__Bacteroidota	c__Bacteroidia	o__Bacteroidales	f__Bacteroidaceae	g__Prevotella	s__Prevotella sp003447235	GCA_003447235.1	56	56_6	F68M_bin.67.fa	F68M_bin.67.fa	97.57	0.88	59
F68U_bin.26	d__Bacteria	p__Bacteroidota	c__Bacteroidia	o__Bacteroidales	f__Bacteroidaceae	g__Prevotella	s__Prevotella sp003447235	GCA_003447235.1	56	56_1	F68W_bin.101.fa	F68U_bin.26.fa	78.71	0	59
F68W_bin.101	d__Bacteria	p__Bacteroidota	c__Bacteroidia	o__Bacteroidales	f__Bacteroidaceae	g__Prevotella	s__Prevotella sp003447235	GCA_003447235.1	56	56_1	F68W_bin.101.fa	F68W_bin.101.fa	97.03	0.36	59
F6DH_bin.50	d__Bacteria	p__Bacteroidota	c__Bacteroidia	o__Bacteroidales	f__Bacteroidaceae	g__Prevotella	s__Prevotella sp003447235	GCA_003447235.1	56	56_1	F68W_bin.101.fa	F6DH_bin.50.fa	94.37	0	59

Table S3. Taxonomic classifiation of the 2,421 detected MAGs

	Domain	Phylum	Class	Order	Family	Genus	Species	RefGenome	primary_cluster	secondary_cluster	repr_genome	genome	CheckM_completeness	CheckM_contamination	
F6MX_bin.36	d__Bacteria	p__Bacteroidota	c__Bacteroidia	o__Bacteroidales	f__Bacteroidaceae	g__Prevotella	s__Prevotella sp003447235	GCA_003447235.1	56	56_5	F6MX_bin.36.fa	F6MX_bin.36.fa	93.48	0	59
F68H_bin.19	d__Bacteria	p__Bacteroidota	c__Bacteroidia	o__Bacteroidales	f__Bacteroidaceae	g__Prevotella	s__Prevotella sp900290275	GCF_900290275.1	64	64_2	F68H_bin.19.fa	F68H_bin.19.fa	96.59	0.1	59
F68U_bin.5	d__Bacteria	p__Bacteroidota	c__Bacteroidia	o__Bacteroidales	f__Bacteroidaceae	g__Prevotella	s__Prevotella sp900290275	GCF_900290275.1	64	64_1	F68U_bin.5.fa	F68U_bin.5.fa	96.08	0	59
F6DH_bin.46	d__Bacteria	p__Bacteroidota	c__Bacteroidia	o__Bacteroidales	f__Bacteroidaceae	g__Prevotella	s__Prevotella sp900290275	GCF_900290275.1	64	64_1	F68U_bin.5.fa	F6DH_bin.46.fa	94.15	1.08	59
F68C_bin.6	d__Bacteria	p__Bacteroidota	c__Bacteroidia	o__Bacteroidales	f__Bacteroidaceae	g__Prevotella	s__Prevotella sp900313215	GCA_900313215.1	45	45_1	F68C_bin.6.fa	F68C_bin.6.fa	81.56	0	59
F68H_bin.70	d__Bacteria	p__Bacteroidota	c__Bacteroidia	o__Bacteroidales	f__Bacteroidaceae	g__Prevotella	s__Prevotella sp900313215	GCA_900313215.1	45	45_3	F68H_bin.70.fa	F68H_bin.70.fa	94.48	1.1	59
F68K_bin.84	d__Bacteria	p__Bacteroidota	c__Bacteroidia	o__Bacteroidales	f__Bacteroidaceae	g__Prevotella	s__Prevotella sp900313215	GCA_900313215.1	45	45_2	F68K_bin.84.fa	F68K_bin.84.fa	85.45	2.67	59

Table S3. Taxonomic classifiation of the 2,421 detected MAGs

	Domain	Phylum	Class	Order	Family	Genus	Species	RefGenome	primary_cluster	secondary_cluster	repr_genome	genome	CheckM_completeness	CheckM_contamination	
F68N_bin.47	d__Bacteria	p__Bacteroidota	c__Bacteroidia	o__Bacteroidales	f__Bacteroidaceae	g__Prevotella	s__Prevotella sp900313215	GCA_900313215.1	45	45_4	F68N_bin.47.fa	F68N_bin.47.fa	80.08	1.7	59
F68P_bin.1	d__Bacteria	p__Bacteroidota	c__Bacteroidia	o__Bacteroidales	f__Bacteroidaceae	g__Prevotella	s__Prevotella sp900313215	GCA_900313215.1	45	45_6	F68P_bin.1.fa	F68P_bin.1.fa	80.33	0.52	59
F68U_bin.84	d__Bacteria	p__Bacteroidota	c__Bacteroidia	o__Bacteroidales	f__Bacteroidaceae	g__Prevotella	s__Prevotella sp900313215	GCA_900313215.1	45	45_6	F68P_bin.1.fa	F68U_bin.84.fa	89.9	3.46	59
F68W_bin.2	d__Bacteria	p__Bacteroidota	c__Bacteroidia	o__Bacteroidales	f__Bacteroidaceae	g__Prevotella	s__Prevotella sp900313215	GCA_900313215.1	45	45_5	F68W_bin.2.fa	F68W_bin.2.fa	83.86	0.3	59
F6DH_bin.119	d__Bacteria	p__Bacteroidota	c__Bacteroidia	o__Bacteroidales	f__Bacteroidaceae	g__Prevotella	s__Prevotella sp900313215	GCA_900313215.1	45	45_5	F68W_bin.2.fa	F6DH_bin.119.fa	62.23	3.45	59
F68C_bin.46	d__Bacteria	p__Bacteroidota	c__Bacteroidia	o__Bacteroidales	f__Bacteroidaceae	g__Prevotella	s__Prevotella stercorea	GCF_000235885.1	60	60_1	F68M_bin.73.fa	F68C_bin.46.fa	67.9	0.37	59
F68K_bin.63	d__Bacteria	p__Bacteroidota	c__Bacteroidia	o__Bacteroidales	f__Bacteroidaceae	g__Prevotella	s__Prevotella stercorea	GCF_000235885.1	60	60_1	F68M_bin.73.fa	F68K_bin.63.fa	96.06	0.56	59

Table S3. Taxonomic classifiation of the 2,421 detected MAGs

	Domain	Phylum	Class	Order	Family	Genus	Species	RefGenome	primary_cluster	secondary_cluster	repr_genome	genome	CheckM_completeness	CheckM_contamination	
F68M_bin.73	d__Bacteria	p__Bacteroidota	c__Bacteroidia	o__Bacteroidales	f__Bacteroidaceae	g__Prevotella	s__Prevotella stercorea	GCF_000235885.1	60	60_1	F68M_bin.73.fa	F68M_bin.73.fa	97.96	0.37	59
F6D7_bin.87	d__Bacteria	p__Bacteroidota	c__Bacteroidia	o__Bacteroidales	f__Bacteroidaceae	g__Prevotella	s__Prevotella stercorea	GCF_000235885.1	60	60_1	F68M_bin.73.fa	F6D7_bin.87.fa	93.48	1.6	59
F68N_bin.54	d__Bacteria	p__Bacteroidota	c__Bacteroidia	o__Bacteroidales	f__Bacteroidaceae	g__Prevotella	s__Prevotella stercorea	GCF_000235885.1	60	60_2	F68N_bin.54.fa	F68N_bin.54.fa	90.09	0.37	59
F68P_bin.35	d__Bacteria	p__Bacteroidota	c__Bacteroidia	o__Bacteroidales	f__Bacteroidaceae	g__Prevotella	s__Prevotella stercorea	GCF_000235885.1	60	60_5	F68P_bin.35.fa	F68P_bin.35.fa	62.34	0.37	59
F6D8_bin.18	d__Bacteria	p__Bacteroidota	c__Bacteroidia	o__Bacteroidales	f__Bacteroidaceae	g__Prevotella	s__Prevotella stercorea	GCF_000235885.1	60	60_3	F6D8_bin.18.fa	F6D8_bin.18.fa	95.8	0	59
F6DB_bin.37	d__Bacteria	p__Bacteroidota	c__Bacteroidia	o__Bacteroidales	f__Bacteroidaceae	g__Prevotella	s__Prevotella stercorea	GCF_000235885.1	60	60_4	F6DB_bin.37.fa	F6DB_bin.37.fa	85.99	0	59
F68D_bin.42	d__Bacteria	p__Bacteroidota	c__Bacteroidia	o__Bacteroidales	f__Bacteroidaceae	g__Prevotellamassilia	s__	N/A	54	54_3	F68D_bin.42.fa	F68D_bin.42.fa	91.33	1.02	60
F68R_bin.70	d__Bacteria	p__Bacteroidota	c__Bacteroidia	o__Bacteroidales	f__Bacteroidaceae	g__Prevotellamassilia	s__	N/A	54	54_4	F68R_bin.70.fa	F68R_bin.70.fa	69.19	0	61

Table S3. Taxonomic classifiation of the 2,421 detected MAGs

	Domain	Phylum	Class	Order	Family	Genus	Species	RefGenome	primary_cluster	secondary_cluster	repr_genome	genome	CheckM_completeness	CheckM_contamination	
F68U_bin.12	d__Bacteria	p__Bacteroidota	c__Bacteroidia	o__Bacteroidales	f__Bacteroidaceae	g__Prevotellamassilia	s__	N/A	54	54_1	F68W_bin.6.fa	F68U_bin.12.fa	70.39	0	62
F68W_bin.6	d__Bacteria	p__Bacteroidota	c__Bacteroidia	o__Bacteroidales	f__Bacteroidaceae	g__Prevotellamassilia	s__	N/A	54	54_1	F68W_bin.6.fa	F68W_bin.6.fa	93.59	3.13	63
F6DH_bin.1	d__Bacteria	p__Bacteroidota	c__Bacteroidia	o__Bacteroidales	f__Bacteroidaceae	g__Prevotellamassilia	s__	N/A	54	54_1	F68W_bin.6.fa	F6DH_bin.1.fa	74.14	3.45	64
F68C_bin.58	d__Bacteria	p__Bacteroidota	c__Bacteroidia	o__Bacteroidales	f__Bacteroidaceae	g__Prevotellamassilia	s__	N/A	54	54_2	F6D7_bin.90.fa	F68C_bin.58.fa	67.02	0.12	65
F6D7_bin.90	d__Bacteria	p__Bacteroidota	c__Bacteroidia	o__Bacteroidales	f__Bacteroidaceae	g__Prevotellamassilia	s__	N/A	54	54_2	F6D7_bin.90.fa	F6D7_bin.90.fa	96.08	0.37	66
F6D8_bin.56	d__Bacteria	p__Bacteroidota	c__Bacteroidia	o__Bacteroidales	f__Bacteroidaceae	g__Prevotellamassilia	s__	N/A	54	54_5	F6D8_bin.56.fa	F6D8_bin.56.fa	64.17	0.12	67
F68U_bin.37	d__Bacteria	p__Bacteroidota	c__Bacteroidia	o__Bacteroidales	f__Bacteroidaceae	g__Prevotellamassilia	s__	N/A	70	70_1	F68W_bin.54.fa	F68U_bin.37.fa	94.17	0.02	68
F68W_bin.54	d__Bacteria	p__Bacteroidota	c__Bacteroidia	o__Bacteroidales	f__Bacteroidaceae	g__Prevotellamassilia	s__	N/A	70	70_1	F68W_bin.54.fa	F68W_bin.54.fa	98.02	0.47	69
F6DH_bin.69	d__Bacteria	p__Bacteroidota	c__Bacteroidia	o__Bacteroidales	f__Bacteroidaceae	g__Prevotellamassilia	s__	N/A	70	70_1	F68W_bin.54.fa	F6DH_bin.69.fa	93.93	0	70
F68U_bin.38	d__Bacteria	p__Bacteroidota	c__Bacteroidia	o__Bacteroidales	f__Bacteroidaceae	g__Prevotellamassilia	s__	N/A	71	71_1	F68U_bin.38.fa	F68U_bin.38.fa	93.46	0	71

Table S3. Taxonomic classifiation of the 2,421 detected MAGs

	Domain	Phylum	Class	Order	Family	Genus	Species	RefGenome	primary_cluster	secondary_cluster	repr_genome	genome	CheckM_completeness	CheckM_contamination	
F6DA_bin.45	d__Bacteria	p__Bacteroidota	c__Bacteroidia	o__Bacteroidales	f__Bacteroidaceae	g__Prevotellamassilia	s__	N/A	71	71_3	F6DA_bin.45.fa	F6DA_bin.45.fa	96.44	0.19	72
F6DG_bin.21	d__Bacteria	p__Bacteroidota	c__Bacteroidia	o__Bacteroidales	f__Bacteroidaceae	g__Prevotellamassilia	s__	N/A	71	71_2	F6DG_bin.21.fa	F6DG_bin.21.fa	96.81	0	73
F68J_bin.75	d__Bacteria	p__Bacteroidota	c__Bacteroidia	o__Bacteroidales	f__Bacteroidaceae	g__Prevotellamassilia	s__Prevotellamassilia timonensis	GCF_900106785.1	72	72_4	F68J_bin.75.fa	F68J_bin.75.fa	55.23	0.02	73
F68N_bin.50	d__Bacteria	p__Bacteroidota	c__Bacteroidia	o__Bacteroidales	f__Bacteroidaceae	g__Prevotellamassilia	s__Prevotellamassilia timonensis	GCF_900106785.1	72	72_3	F68N_bin.50.fa	F68N_bin.50.fa	93.66	0.05	73
F68P_bin.32	d__Bacteria	p__Bacteroidota	c__Bacteroidia	o__Bacteroidales	f__Bacteroidaceae	g__Prevotellamassilia	s__Prevotellamassilia timonensis	GCF_900106785.1	72	72_1	F68P_bin.32.fa	F68P_bin.32.fa	95.23	0.19	73
F6MX_bin.74	d__Bacteria	p__Bacteroidota	c__Bacteroidia	o__Bacteroidales	f__Bacteroidaceae	g__Prevotellamassilia	s__Prevotellamassilia timonensis	GCF_900106785.1	72	72_2	F6MX_bin.74.fa	F6MX_bin.74.fa	94.04	1.56	73
F68C_bin.49	d__Bacteria	p__Bacteroidota	c__Bacteroidia	o__Bacteroidales	f__Bacteroidaceae	g__UBA4372	s__	N/A	69	69_1	F68C_bin.49.fa	F68C_bin.49.fa	96.16	0.19	74

Table S3. Taxonomic classifiation of the 2,421 detected MAGs

	Domain	Phylum	Class	Order	Family	Genus	Species	RefGenome	primary_cluster	secondary_cluster	repr_genome	genome	CheckM_completeness	CheckM_contamination	
F68H_bin.37	d__Bacteria	p__Bacteroidota	c__Bacteroidia	o__Bacteroidales	f__Bacteroidaceae	g__UBA4372	s__	N/A	69	69_1	F68C_bin.49.fa	F68H_bin.37.fa	95.42	0.56	75
F68J_bin.83	d__Bacteria	p__Bacteroidota	c__Bacteroidia	o__Bacteroidales	f__Bacteroidaceae	g__UBA4372	s__	N/A	69	69_1	F68C_bin.49.fa	F68J_bin.83.fa	89.57	1.05	76
F68W_bin.15	d__Bacteria	p__Bacteroidota	c__Bacteroidia	o__Bacteroidales	f__Bacteroidaceae	g__UBA4372	s__	N/A	69	69_1	F68C_bin.49.fa	F68W_bin.15.fa	94.3	0.19	77
F6DH_bin.120	d__Bacteria	p__Bacteroidota	c__Bacteroidia	o__Bacteroidales	f__Bacteroidaceae	g__UBA4372	s__	N/A	69	69_1	F68C_bin.49.fa	F6DH_bin.120.fa	96.53	0.56	78
F6D9_bin.25	d__Bacteria	p__Bacteroidota	c__Bacteroidia	o__Bacteroidales	f__Bacteroidaceae	g__UBA4372	s__	N/A	69	69_2	F6D9_bin.25.fa	F6D9_bin.25.fa	94.28	0.56	79
F68F_bin.93	d__Bacteria	p__Bacteroidota	c__Bacteroidia	o__Bacteroidales	f__Bacteroidaceae	g__UBA6398	s__	N/A	16	16_6	F68F_bin.93.fa	F68F_bin.93.fa	82.22	1.81	80
F68M_bin.88	d__Bacteria	p__Bacteroidota	c__Bacteroidia	o__Bacteroidales	f__Bacteroidaceae	g__UBA6398	s__	N/A	16	16_5	F68M_bin.88.fa	F68M_bin.88.fa	79.62	2.09	81
F6D7_bin.10	d__Bacteria	p__Bacteroidota	c__Bacteroidia	o__Bacteroidales	f__Bacteroidaceae	g__UBA6398	s__	N/A	16	16_1	F6D7_bin.10.fa	F6D7_bin.10.fa	92.08	1	82
F6D8_bin.95	d__Bacteria	p__Bacteroidota	c__Bacteroidia	o__Bacteroidales	f__Bacteroidaceae	g__UBA6398	s__	N/A	16	16_2	F6D8_bin.95.fa	F6D8_bin.95.fa	59.87	1.88	83
F6DB_bin.55	d__Bacteria	p__Bacteroidota	c__Bacteroidia	o__Bacteroidales	f__Bacteroidaceae	g__UBA6398	s__	N/A	16	16_3	F6DB_bin.55.fa	F6DB_bin.55.fa	75.94	0.49	84

Table S3. Taxonomic classifiation of the 2,421 detected MAGs

	Domain	Phylum	Class	Order	Family	Genus	Species	RefGenome	primary_cluster	secondary_cluster	repr_genome	genome	CheckM_completeness	CheckM_contamination	
F6MX_bin.77	d__Bacteria	p__Bacteroidota	c__Bacteroidia	o__Bacteroidales	f__Bacteroidaceae	g__UBA6398	s__	N/A	16	16_4	F6MX_bin.77.fa	F6MX_bin.77.fa	63.91	0.37	85
F6MY_bin.8	d__Bacteria	p__Bacteroidota	c__Bacteroidia	o__Bacteroidales	f__Bacteroidaceae	g__UBA6398	s__	N/A	16	16_7	F6MY_bin.8.fa	F6MY_bin.8.fa	73.95	0.74	86
F68K_bin.17	d__Bacteria	p__Bacteroidota	c__Bacteroidia	o__Bacteroidales	f__Barnesiellaceae	g__Barnesiella	s__Barnesiella intestinihominis	GCF_000296465.1	116	116_5	F68K_bin.17.fa	F68K_bin.17.fa	89.55	0.38	86
F68T_bin.59	d__Bacteria	p__Bacteroidota	c__Bacteroidia	o__Bacteroidales	f__Barnesiellaceae	g__Barnesiella	s__Barnesiella intestinihominis	GCF_000296465.1	116	116_2	F68T_bin.59.fa	F68T_bin.59.fa	98.77	0	86
F6DE_bin.33	d__Bacteria	p__Bacteroidota	c__Bacteroidia	o__Bacteroidales	f__Barnesiellaceae	g__Barnesiella	s__Barnesiella intestinihominis	GCF_000296465.1	116	116_3	F6DE_bin.33.fa	F6DE_bin.33.fa	98.49	0	86
F6DG_bin.15	d__Bacteria	p__Bacteroidota	c__Bacteroidia	o__Bacteroidales	f__Barnesiellaceae	g__Barnesiella	s__Barnesiella intestinihominis	GCF_000296465.1	116	116_4	F6DG_bin.15.fa	F6DG_bin.15.fa	97.36	0	86
F68D_bin.24	d__Bacteria	p__Bacteroidota	c__Bacteroidia	o__Bacteroidales	f__Barnesiellaceae	g__Barnesiella	s__Barnesiella intestinihominis	GCF_000296465.1	116	116_1	F6MY_bin.25.fa	F68D_bin.24.fa	98.71	1.02	86

Table S3. Taxonomic classifiation of the 2,421 detected MAGs

	Domain	Phylum	Class	Order	Family	Genus	Species	RefGenome	primary_cluster	secondary_cluster	repr_genome	genome	CheckM_completeness	CheckM_contamination	
F6MY_bin.25	d__Bacteria	p__Bacteroidota	c__Bacteroidia	o__Bacteroidales	f__Barnesiellaceae	g__Barnesiella	s__Barnesiella intestinihominis	GCF_000296465.1	116	116_1	F6MY_bin.25.fa	F6MY_bin.25.fa	99.62	0	86
F6DH_bin.83	d__Bacteria	p__Bacteroidota	c__Bacteroidia	o__Bacteroidales	f__Marinifilaceae	g__Butyricimonas	s__Butyricimonas sp002161485	GCF_002161485.1	78	78_10	F6DH_bin.83.fa	F6DH_bin.83.fa	85.09	4.28	86
F6MX_bin.67	d__Bacteria	p__Bacteroidota	c__Bacteroidia	o__Bacteroidales	f__Marinifilaceae	g__Butyricimonas	s__Butyricimonas sp900184685	GCF_900184685.1	79	79_0	F6MX_bin.67.fa	F6MX_bin.67.fa	95.97	0.54	86
F6D7_bin.12	d__Bacteria	p__Bacteroidota	c__Bacteroidia	o__Bacteroidales	f__Marinifilaceae	g__Butyricimonas	s__Butyricimonas sp900258545	GCF_900258545.1	77	77_1	F6DE_bin.58.fa	F6D7_bin.12.fa	85.19	2.74	86
F6DE_bin.58	d__Bacteria	p__Bacteroidota	c__Bacteroidia	o__Bacteroidales	f__Marinifilaceae	g__Butyricimonas	s__Butyricimonas sp900258545	GCF_900258545.1	77	77_1	F6DE_bin.58.fa	F6DE_bin.58.fa	88.99	3.23	86
F6DG_bin.28	d__Bacteria	p__Bacteroidota	c__Bacteroidia	o__Bacteroidales	f__Marinifilaceae	g__Butyricimonas	s__Butyricimonas sp900258545	GCF_900258545.1	77	77_1	F6DE_bin.58.fa	F6DG_bin.28.fa	64.98	1.72	86

Table S3. Taxonomic classifiation of the 2,421 detected MAGs

	Domain	Phylum	Class	Order	Family	Genus	Species	RefGenome	primary_cluster	secondary_cluster	repr_genome	genome	CheckM_completeness	CheckM_contamination	
F6DA_bin.2	d__Bacteria	p__Bacteroidota	c__Bacteroidia	o__Bacteroidales	f__Marinifilaceae	g__Butyricimonas	s__Butyricimonas synergistica_A	GCA_001915615.1	78	78_9	F6DA_bin.2.fa	F6DA_bin.2.fa	84.12	3.76	86
F68C_bin.24	d__Bacteria	p__Bacteroidota	c__Bacteroidia	o__Bacteroidales	f__Marinifilaceae	g__Butyricimonas	s__Butyricimonas virosa	GCF_000519105.1	78	78_7	F68C_bin.24.fa	F68C_bin.24.fa	69.12	0.86	86
F68E_bin.20	d__Bacteria	p__Bacteroidota	c__Bacteroidia	o__Bacteroidales	f__Marinifilaceae	g__Butyricimonas	s__Butyricimonas virosa	GCF_000519105.1	78	78_3	F68E_bin.20.fa	F68E_bin.20.fa	93.7	0	86
F68G_bin.74	d__Bacteria	p__Bacteroidota	c__Bacteroidia	o__Bacteroidales	f__Marinifilaceae	g__Butyricimonas	s__Butyricimonas virosa	GCF_000519105.1	78	78_1	F68G_bin.74.fa	F68G_bin.74.fa	99.46	0	86
F6MY_bin.83	d__Bacteria	p__Bacteroidota	c__Bacteroidia	o__Bacteroidales	f__Marinifilaceae	g__Butyricimonas	s__Butyricimonas virosa	GCF_000519105.1	78	78_1	F68G_bin.74.fa	F6MY_bin.83.fa	93.14	4.01	86
F68H_bin.64	d__Bacteria	p__Bacteroidota	c__Bacteroidia	o__Bacteroidales	f__Marinifilaceae	g__Butyricimonas	s__Butyricimonas virosa	GCF_000519105.1	78	78_4	F68H_bin.64.fa	F68H_bin.64.fa	65.25	4.11	86
F68K_bin.3	d__Bacteria	p__Bacteroidota	c__Bacteroidia	o__Bacteroidales	f__Marinifilaceae	g__Butyricimonas	s__Butyricimonas virosa	GCF_000519105.1	78	78_2	F68K_bin.3.fa	F68K_bin.3.fa	93.19	1.25	86

Table S3. Taxonomic classifiation of the 2,421 detected MAGs

	Domain	Phylum	Class	Order	Family	Genus	Species	RefGenome	primary_cluster	secondary_cluster	repr_genome	genome	CheckM_completeness	CheckM_contamination	
F68R_bin.95	d__Bacteria	p__Bacteroidota	c__Bacteroidia	o__Bacteroidales	f__Marinifilaceae	g__Butyricimonas	s__Butyricimonas virosa	GCF_000519105.1	78	78_2	F68K_bin.3.fa	F68R_bin.95.fa	87.73	3.76	86
F68V_bin.119	d__Bacteria	p__Bacteroidota	c__Bacteroidia	o__Bacteroidales	f__Marinifilaceae	g__Butyricimonas	s__Butyricimonas virosa	GCF_000519105.1	78	78_2	F68K_bin.3.fa	F68V_bin.119.fa	57.35	1.08	86
F6MX_bin.110	d__Bacteria	p__Bacteroidota	c__Bacteroidia	o__Bacteroidales	f__Marinifilaceae	g__Butyricimonas	s__Butyricimonas virosa	GCF_000519105.1	78	78_2	F68K_bin.3.fa	F6MX_bin.110.fa	81.83	0.99	86
F68U_bin.21	d__Bacteria	p__Bacteroidota	c__Bacteroidia	o__Bacteroidales	f__Marinifilaceae	g__Butyricimonas	s__Butyricimonas virosa	GCF_000519105.1	78	78_5	F68U_bin.21.fa	F68U_bin.21.fa	98.39	4.44	86
F6DB_bin.66	d__Bacteria	p__Bacteroidota	c__Bacteroidia	o__Bacteroidales	f__Marinifilaceae	g__Butyricimonas	s__Butyricimonas virosa	GCF_000519105.1	78	78_8	F6DB_bin.66.fa	F6DB_bin.66.fa	90.86	2.8	86
F6DE_bin.69	d__Bacteria	p__Bacteroidota	c__Bacteroidia	o__Bacteroidales	f__Marinifilaceae	g__Butyricimonas	s__Butyricimonas virosa	GCF_000519105.1	78	78_6	F6DE_bin.69.fa	F6DE_bin.69.fa	84.94	1.63	86
F68C_bin.18	d__Bacteria	p__Bacteroidota	c__Bacteroidia	o__Bacteroidales	f__Marinifilaceae	g__Odoribacter	s__Odoribacter splanchnicus	GCF_000190535.1	76	76_1	F6DE_bin.90.fa	F68C_bin.18.fa	97.85	0.57	86

Table S3. Taxonomic classifiation of the 2,421 detected MAGs

	Domain	Phylum	Class	Order	Family	Genus	Species	RefGenome	primary_cluster	secondary_cluster	repr_genome	genome	CheckM_completeness	CheckM_contamination	
F68D_bin.57	d__Bacteria	p__Bacteroidota	c__Bacteroidia	o__Bacteroidales	f__Marinifilaceae	g__Odoribacter	s__Odoribacter splanchnicus	GCF_000190535.1	76	76_1	F6DE_bin.90.fa	F68D_bin.57.fa	89.96	0.02	86
F68E_bin.43	d__Bacteria	p__Bacteroidota	c__Bacteroidia	o__Bacteroidales	f__Marinifilaceae	g__Odoribacter	s__Odoribacter splanchnicus	GCF_000190535.1	76	76_1	F6DE_bin.90.fa	F68E_bin.43.fa	84.91	0.56	86
F68F_bin.44	d__Bacteria	p__Bacteroidota	c__Bacteroidia	o__Bacteroidales	f__Marinifilaceae	g__Odoribacter	s__Odoribacter splanchnicus	GCF_000190535.1	76	76_1	F6DE_bin.90.fa	F68F_bin.44.fa	98.39	0	86
F68H_bin.21	d__Bacteria	p__Bacteroidota	c__Bacteroidia	o__Bacteroidales	f__Marinifilaceae	g__Odoribacter	s__Odoribacter splanchnicus	GCF_000190535.1	76	76_1	F6DE_bin.90.fa	F68H_bin.21.fa	91.32	0.81	86
F68K_bin.85	d__Bacteria	p__Bacteroidota	c__Bacteroidia	o__Bacteroidales	f__Marinifilaceae	g__Odoribacter	s__Odoribacter splanchnicus	GCF_000190535.1	76	76_1	F6DE_bin.90.fa	F68K_bin.85.fa	98.39	0	86
F68N_bin.53	d__Bacteria	p__Bacteroidota	c__Bacteroidia	o__Bacteroidales	f__Marinifilaceae	g__Odoribacter	s__Odoribacter splanchnicus	GCF_000190535.1	76	76_1	F6DE_bin.90.fa	F68N_bin.53.fa	97.85	0.54	86
F68R_bin.29	d__Bacteria	p__Bacteroidota	c__Bacteroidia	o__Bacteroidales	f__Marinifilaceae	g__Odoribacter	s__Odoribacter splanchnicus	GCF_000190535.1	76	76_1	F6DE_bin.90.fa	F68R_bin.29.fa	98.92	0	86

Table S3. Taxonomic classifiation of the 2,421 detected MAGs

	Domain	Phylum	Class	Order	Family	Genus	Species	RefGenome	primary_cluster	secondary_cluster	repr_genome	genome	CheckM_completeness	CheckM_contamination	
F68T_bin.29	d__Bacteria	p__Bacteroidota	c__Bacteroidia	o__Bacteroidales	f__Marinifilaceae	g__Odoribacter	s__Odoribacter splanchnicus	GCF_000190535.1	76	76_1	F6DE_bin.90.fa	F68T_bin.29.fa	97.85	0	86
F68U_bin.105	d__Bacteria	p__Bacteroidota	c__Bacteroidia	o__Bacteroidales	f__Marinifilaceae	g__Odoribacter	s__Odoribacter splanchnicus	GCF_000190535.1	76	76_1	F6DE_bin.90.fa	F68U_bin.105.fa	96.24	0	86
F68V_bin.73	d__Bacteria	p__Bacteroidota	c__Bacteroidia	o__Bacteroidales	f__Marinifilaceae	g__Odoribacter	s__Odoribacter splanchnicus	GCF_000190535.1	76	76_1	F6DE_bin.90.fa	F68V_bin.73.fa	65.67	0	86
F6D7_bin.96	d__Bacteria	p__Bacteroidota	c__Bacteroidia	o__Bacteroidales	f__Marinifilaceae	g__Odoribacter	s__Odoribacter splanchnicus	GCF_000190535.1	76	76_1	F6DE_bin.90.fa	F6D7_bin.96.fa	95.43	2.15	86
F6D8_bin.62	d__Bacteria	p__Bacteroidota	c__Bacteroidia	o__Bacteroidales	f__Marinifilaceae	g__Odoribacter	s__Odoribacter splanchnicus	GCF_000190535.1	76	76_1	F6DE_bin.90.fa	F6D8_bin.62.fa	99.46	0	86
F6D9_bin.16	d__Bacteria	p__Bacteroidota	c__Bacteroidia	o__Bacteroidales	f__Marinifilaceae	g__Odoribacter	s__Odoribacter splanchnicus	GCF_000190535.1	76	76_1	F6DE_bin.90.fa	F6D9_bin.16.fa	62.63	2.33	86
F6DA_bin.40	d__Bacteria	p__Bacteroidota	c__Bacteroidia	o__Bacteroidales	f__Marinifilaceae	g__Odoribacter	s__Odoribacter splanchnicus	GCF_000190535.1	76	76_1	F6DE_bin.90.fa	F6DA_bin.40.fa	97.85	0	86

Table S3. Taxonomic classifiation of the 2,421 detected MAGs

	Domain	Phylum	Class	Order	Family	Genus	Species	RefGenome	primary_cluster	secondary_cluster	repr_genome	genome	CheckM_completeness	CheckM_contamination	
F6DB_bin.73	d__Bacteria	p__Bacteroidota	c__Bacteroidia	o__Bacteroidales	f__Marinifilaceae	g__Odoribacter	s__Odoribacter splanchnicus	GCF_000190535.1	76	76_1	F6DE_bin.90.fa	F6DB_bin.73.fa	99.46	0	86
F6DE_bin.90	d__Bacteria	p__Bacteroidota	c__Bacteroidia	o__Bacteroidales	f__Marinifilaceae	g__Odoribacter	s__Odoribacter splanchnicus	GCF_000190535.1	76	76_1	F6DE_bin.90.fa	F6DE_bin.90.fa	99.46	0	86
F6DG_bin.55	d__Bacteria	p__Bacteroidota	c__Bacteroidia	o__Bacteroidales	f__Marinifilaceae	g__Odoribacter	s__Odoribacter splanchnicus	GCF_000190535.1	76	76_1	F6DE_bin.90.fa	F6DG_bin.55.fa	97.31	0	86
F6MX_bin.119	d__Bacteria	p__Bacteroidota	c__Bacteroidia	o__Bacteroidales	f__Marinifilaceae	g__Odoribacter	s__Odoribacter splanchnicus	GCF_000190535.1	76	76_1	F6DE_bin.90.fa	F6MX_bin.119.fa	98.39	0	86
F6MY_bin.116	d__Bacteria	p__Bacteroidota	c__Bacteroidia	o__Bacteroidales	f__Marinifilaceae	g__Odoribacter	s__Odoribacter splanchnicus	GCF_000190535.1	76	76_1	F6DE_bin.90.fa	F6MY_bin.116.fa	98.92	0	86
F68J_bin.27	d__Bacteria	p__Bacteroidota	c__Bacteroidia	o__Bacteroidales	f__Muribaculaceae	g__C941	s__	N/A	264	264_2	F68J_bin.27.fa	F68J_bin.27.fa	91.89	0.13	87
F68P_bin.29	d__Bacteria	p__Bacteroidota	c__Bacteroidia	o__Bacteroidales	f__Muribaculaceae	g__C941	s__	N/A	264	264_3	F68P_bin.29.fa	F68P_bin.29.fa	79.56	2.87	88
F68W_bin.87	d__Bacteria	p__Bacteroidota	c__Bacteroidia	o__Bacteroidales	f__Muribaculaceae	g__C941	s__	N/A	264	264_1	F68W_bin.87.fa	F68W_bin.87.fa	94.91	0.5	89

Table S3. Taxonomic classifiation of the 2,421 detected MAGs

	Domain	Phylum	Class	Order	Family	Genus	Species	RefGenome	primary_cluster	secondary_cluster	repr_genome	genome	CheckM_completeness	CheckM_contamination	
F6DH_bin.72	d__Bacteria	p__Bacteroidota	c__Bacteroidia	o__Bacteroidales	f__Muribaculaceae	g__C941	s__	N/A	264	264_1	F68W_bin.87.fa	F6DH_bin.72.fa	96.04	0.88	90
F6D9_bin.59	d__Bacteria	p__Bacteroidota	c__Bacteroidia	o__Bacteroidales	f__Muribaculaceae	g__C941	s__	N/A	264	264_4	F6D9_bin.59.fa	F6D9_bin.59.fa	96.23	0.5	91
F68K_bin.62	d__Bacteria	p__Bacteroidota	c__Bacteroidia	o__Bacteroidales	f__Muribaculaceae	g__CAG-279	s__	N/A	114	114_3	F68K_bin.62.fa	F68K_bin.62.fa	84.53	0.5	92
F6DE_bin.67	d__Bacteria	p__Bacteroidota	c__Bacteroidia	o__Bacteroidales	f__Muribaculaceae	g__CAG-279	s__	N/A	114	114_1	F6DE_bin.67.fa	F6DE_bin.67.fa	91.53	1.87	93
F6MY_bin.9	d__Bacteria	p__Bacteroidota	c__Bacteroidia	o__Bacteroidales	f__Muribaculaceae	g__CAG-279	s__	N/A	114	114_2	F6MY_bin.9.fa	F6MY_bin.9.fa	88.68	0.38	94
F6MX_bin.90	d__Bacteria	p__Bacteroidota	c__Bacteroidia	o__Bacteroidales	f__Muribaculaceae	g__CAG-279	s__	N/A	38	38_0	F6MX_bin.90.fa	F6MX_bin.90.fa	68.64	2.31	95
F68F_bin.37	d__Bacteria	p__Bacteroidota	c__Bacteroidia	o__Bacteroidales	f__Muribaculaceae	g__CAG-279	s__CAG-279 sp000437795	GCA_000437795.1	113	113_2	F68F_bin.37.fa	F68F_bin.37.fa	92.08	0.75	95
F68H_bin.51	d__Bacteria	p__Bacteroidota	c__Bacteroidia	o__Bacteroidales	f__Muribaculaceae	g__CAG-279	s__CAG-279 sp000437795	GCA_000437795.1	113	113_2	F68F_bin.37.fa	F68H_bin.51.fa	82.08	0.41	95
F68J_bin.85	d__Bacteria	p__Bacteroidota	c__Bacteroidia	o__Bacteroidales	f__Muribaculaceae	g__CAG-279	s__CAG-279 sp000437795	GCA_000437795.1	113	113_6	F68J_bin.85.fa	F68J_bin.85.fa	69.48	2.64	95

Table S3. Taxonomic classifiation of the 2,421 detected MAGs

	Domain	Phylum	Class	Order	Family	Genus	Species	RefGenome	primary_cluster	secondary_cluster	repr_genome	genome	CheckM_completeness	CheckM_contamination	
F68N_bin.46	d__Bacteria	p__Bacteroidota	c__Bacteroidia	o__Bacteroidales	f__Muribaculaceae	g__CAG-279	s__CAG-279 sp000437795	GCA_000437795.1	113	113_9	F68N_bin.46.fa	F68N_bin.46.fa	82.58	0	95
F68P_bin.25	d__Bacteria	p__Bacteroidota	c__Bacteroidia	o__Bacteroidales	f__Muribaculaceae	g__CAG-279	s__CAG-279 sp000437795	GCA_000437795.1	113	113_12	F68P_bin.25.fa	F68P_bin.25.fa	88.11	0.38	95
F68R_bin.106	d__Bacteria	p__Bacteroidota	c__Bacteroidia	o__Bacteroidales	f__Muribaculaceae	g__CAG-279	s__CAG-279 sp000437795	GCA_000437795.1	113	113_11	F68R_bin.106.fa	F68R_bin.106.fa	71.32	0	95
F68T_bin.32	d__Bacteria	p__Bacteroidota	c__Bacteroidia	o__Bacteroidales	f__Muribaculaceae	g__CAG-279	s__CAG-279 sp000437795	GCA_000437795.1	113	113_8	F68T_bin.32.fa	F68T_bin.32.fa	97.36	0	95
F68U_bin.50	d__Bacteria	p__Bacteroidota	c__Bacteroidia	o__Bacteroidales	f__Muribaculaceae	g__CAG-279	s__CAG-279 sp000437795	GCA_000437795.1	113	113_4	F68U_bin.50.fa	F68U_bin.50.fa	81.17	0.75	95
F6D8_bin.70	d__Bacteria	p__Bacteroidota	c__Bacteroidia	o__Bacteroidales	f__Muribaculaceae	g__CAG-279	s__CAG-279 sp000437795	GCA_000437795.1	113	113_10	F6D8_bin.70.fa	F6D8_bin.70.fa	88.11	0	95
F6D9_bin.94	d__Bacteria	p__Bacteroidota	c__Bacteroidia	o__Bacteroidales	f__Muribaculaceae	g__CAG-279	s__CAG-279 sp000437795	GCA_000437795.1	113	113_13	F6D9_bin.94.fa	F6D9_bin.94.fa	77.07	1.56	95

Table S3. Taxonomic classifiation of the 2,421 detected MAGs

	Domain	Phylum	Class	Order	Family	Genus	Species	RefGenome	primary_cluster	secondary_cluster	repr_genome	genome	CheckM_completeness	CheckM_contamination	
F6DA_bin.96	d__Bacteria	p__Bacteroidota	c__Bacteroidia	o__Bacteroidales	f__Muribaculaceae	g__CAG-279	s__CAG-279 sp000437795	GCA_000437795.1	113	113_7	F6DA_bin.96.fa	F6DA_bin.96.fa	86.79	0	95
F6DG_bin.44	d__Bacteria	p__Bacteroidota	c__Bacteroidia	o__Bacteroidales	f__Muribaculaceae	g__CAG-279	s__CAG-279 sp000437795	GCA_000437795.1	113	113_5	F6DG_bin.44.fa	F6DG_bin.44.fa	80.57	0	95
F68W_bin.39	d__Bacteria	p__Bacteroidota	c__Bacteroidia	o__Bacteroidales	f__Muribaculaceae	g__CAG-279	s__CAG-279 sp000437795	GCA_000437795.1	113	113_1	F6DH_bin.105.fa	F68W_bin.39.fa	76.42	0	95
F6DH_bin.105	d__Bacteria	p__Bacteroidota	c__Bacteroidia	o__Bacteroidales	f__Muribaculaceae	g__CAG-279	s__CAG-279 sp000437795	GCA_000437795.1	113	113_1	F6DH_bin.105.fa	F6DH_bin.105.fa	81.7	0	95
F68C_bin.91	d__Bacteria	p__Bacteroidota	c__Bacteroidia	o__Bacteroidales	f__Muribaculaceae	g__CAG-279	s__CAG-279 sp000437795	GCA_000437795.1	113	113_3	F6MX_bin.14.fa	F68C_bin.91.fa	77.92	0	95
F6MX_bin.14	d__Bacteria	p__Bacteroidota	c__Bacteroidia	o__Bacteroidales	f__Muribaculaceae	g__CAG-279	s__CAG-279 sp000437795	GCA_000437795.1	113	113_3	F6MX_bin.14.fa	F6MX_bin.14.fa	95.61	0.57	95
F68E_bin.11	d__Bacteria	p__Bacteroidota	c__Bacteroidia	o__Bacteroidales	f__Muribaculaceae	g__CAG-485	s__	N/A	117	117_0	F68E_bin.11.fa	F68E_bin.11.fa	96.35	0.38	96
F6DB_bin.14	d__Bacteria	p__Bacteroidota	c__Bacteroidia	o__Bacteroidales	f__Muribaculaceae	g__CAG-485	s__	N/A	17	17_0	F6DB_bin.14.fa	F6DB_bin.14.fa	91.95	0.75	97

Table S3. Taxonomic classifiation of the 2,421 detected MAGs

	Domain	Phylum	Class	Order	Family	Genus	Species	RefGenome	primary_cluster	secondary_cluster	repr_genome	genome	CheckM_completeness	CheckM_contamination	
F68G_bin.51	d__Bacteria	p__Bacteroidota	c__Bacteroidia	o__Bacteroidales	f__Muribaculaceae	g__CAG-873	s__CAG-873 sp001701165	GCA_001701165.1	115	115_1	F68H_bin.24.fa	F68G_bin.51.fa	97.36	0.05	97
F68H_bin.24	d__Bacteria	p__Bacteroidota	c__Bacteroidia	o__Bacteroidales	f__Muribaculaceae	g__CAG-873	s__CAG-873 sp001701165	GCA_001701165.1	115	115_1	F68H_bin.24.fa	F68H_bin.24.fa	97.36	0	97
F68W_bin.7	d__Bacteria	p__Bacteroidota	c__Bacteroidia	o__Bacteroidales	f__Muribaculaceae	g__CAG-873	s__CAG-873 sp001701165	GCA_001701165.1	115	115_1	F68H_bin.24.fa	F68W_bin.7.fa	96.98	0.5	97
F6DH_bin.14	d__Bacteria	p__Bacteroidota	c__Bacteroidia	o__Bacteroidales	f__Muribaculaceae	g__CAG-873	s__CAG-873 sp001701165	GCA_001701165.1	115	115_1	F68H_bin.24.fa	F6DH_bin.14.fa	96.6	0	97
F68G_bin.67	d__Bacteria	p__Bacteroidota	c__Bacteroidia	o__Bacteroidales	f__P3	g__UBA3388	s__	N/A	25	25_0	F68G_bin.67.fa	F68G_bin.67.fa	95.5	0	98
F68T_bin.20	d__Bacteria	p__Bacteroidota	c__Bacteroidia	o__Bacteroidales	f__Rikenellaceae	g__Alistipes	s__	N/A	34	34_0	F68T_bin.20.fa	F68T_bin.20.fa	100	0.24	99
F6MY_bin.29	d__Bacteria	p__Bacteroidota	c__Bacteroidia	o__Bacteroidales	f__Rikenellaceae	g__Alistipes	s__	N/A	35	35_0	F6MY_bin.29.fa	F6MY_bin.29.fa	70.16	2.59	100
F6MY_bin.66	d__Bacteria	p__Bacteroidota	c__Bacteroidia	o__Bacteroidales	f__Rikenellaceae	g__Alistipes	s__	N/A	36	36_0	F6MY_bin.66.fa	F6MY_bin.66.fa	97.28	1.05	101

Table S3. Taxonomic classifiation of the 2,421 detected MAGs

	Domain	Phylum	Class	Order	Family	Genus	Species	RefGenome	primary_cluster	secondary_cluster	repr_genome	genome	CheckM_completeness	CheckM_contamination	
F68K_bin.80	d__Bacteria	p__Bacteroidota	c__Bacteroidia	o__Bacteroidales	f__Rikenellaceae	g__Alistipes	s__Alistipes finegoldii	GCF_000265365.1	32	32_2	F68K_bin.80.fa	F68K_bin.80.fa	78.25	0.64	101
F68U_bin.6	d__Bacteria	p__Bacteroidota	c__Bacteroidia	o__Bacteroidales	f__Rikenellaceae	g__Alistipes	s__Alistipes finegoldii	GCF_000265365.1	32	32_3	F68U_bin.6.fa	F68U_bin.6.fa	99.23	0	101
F6DB_bin.112	d__Bacteria	p__Bacteroidota	c__Bacteroidia	o__Bacteroidales	f__Rikenellaceae	g__Alistipes	s__Alistipes finegoldii	GCF_000265365.1	32	32_1	F6DE_bin.78.fa	F6DB_bin.112.fa	85.04	1.52	101
F6DE_bin.78	d__Bacteria	p__Bacteroidota	c__Bacteroidia	o__Bacteroidales	f__Rikenellaceae	g__Alistipes	s__Alistipes finegoldii	GCF_000265365.1	32	32_1	F6DE_bin.78.fa	F6DE_bin.78.fa	99.74	0	101
F6DG_bin.59	d__Bacteria	p__Bacteroidota	c__Bacteroidia	o__Bacteroidales	f__Rikenellaceae	g__Alistipes	s__Alistipes finegoldii	GCF_000265365.1	32	32_1	F6DE_bin.78.fa	F6DG_bin.59.fa	89.54	0.56	101
F68D_bin.48	d__Bacteria	p__Bacteroidota	c__Bacteroidia	o__Bacteroidales	f__Rikenellaceae	g__Alistipes	s__Alistipes obesi	GCF_000311925.1	29	29_1	F6DE_bin.14.fa	F68D_bin.48.fa	58.17	2.64	101
F68E_bin.5	d__Bacteria	p__Bacteroidota	c__Bacteroidia	o__Bacteroidales	f__Rikenellaceae	g__Alistipes	s__Alistipes obesi	GCF_000311925.1	29	29_1	F6DE_bin.14.fa	F68E_bin.5.fa	87.71	1.39	101
F68F_bin.107	d__Bacteria	p__Bacteroidota	c__Bacteroidia	o__Bacteroidales	f__Rikenellaceae	g__Alistipes	s__Alistipes obesi	GCF_000311925.1	29	29_1	F6DE_bin.14.fa	F68F_bin.107.fa	94.23	0	101

Table S3. Taxonomic classifiation of the 2,421 detected MAGs

	Domain	Phylum	Class	Order	Family	Genus	Species	RefGenome	primary_cluster	secondary_cluster	repr_genome	genome	CheckM_completeness	CheckM_contamination	
F68K_bin.56	d__Bacteria	p__Bacteroidota	c__Bacteroidia	o__Bacteroidales	f__Rikenellaceae	g__Alistipes	s__Alistipes obesi	GCF_000311925.1	29	29_1	F6DE_bin.14.fa	F68K_bin.56.fa	80.09	1.6	101
F68R_bin.78	d__Bacteria	p__Bacteroidota	c__Bacteroidia	o__Bacteroidales	f__Rikenellaceae	g__Alistipes	s__Alistipes obesi	GCF_000311925.1	29	29_1	F6DE_bin.14.fa	F68R_bin.78.fa	91.27	0.96	101
F6D8_bin.29	d__Bacteria	p__Bacteroidota	c__Bacteroidia	o__Bacteroidales	f__Rikenellaceae	g__Alistipes	s__Alistipes obesi	GCF_000311925.1	29	29_1	F6DE_bin.14.fa	F6D8_bin.29.fa	98	0.48	101
F6DA_bin.10	d__Bacteria	p__Bacteroidota	c__Bacteroidia	o__Bacteroidales	f__Rikenellaceae	g__Alistipes	s__Alistipes obesi	GCF_000311925.1	29	29_1	F6DE_bin.14.fa	F6DA_bin.10.fa	88.28	2.08	101
F6DB_bin.64	d__Bacteria	p__Bacteroidota	c__Bacteroidia	o__Bacteroidales	f__Rikenellaceae	g__Alistipes	s__Alistipes obesi	GCF_000311925.1	29	29_1	F6DE_bin.14.fa	F6DB_bin.64.fa	100	0.48	101
F6DE_bin.14	d__Bacteria	p__Bacteroidota	c__Bacteroidia	o__Bacteroidales	f__Rikenellaceae	g__Alistipes	s__Alistipes obesi	GCF_000311925.1	29	29_1	F6DE_bin.14.fa	F6DE_bin.14.fa	98.56	0	101
F6MX_bin.22	d__Bacteria	p__Bacteroidota	c__Bacteroidia	o__Bacteroidales	f__Rikenellaceae	g__Alistipes	s__Alistipes obesi	GCF_000311925.1	29	29_1	F6DE_bin.14.fa	F6MX_bin.22.fa	98.56	0	101
F6MY_bin.7	d__Bacteria	p__Bacteroidota	c__Bacteroidia	o__Bacteroidales	f__Rikenellaceae	g__Alistipes	s__Alistipes obesi	GCF_000311925.1	29	29_1	F6DE_bin.14.fa	F6MY_bin.7.fa	98.56	0.48	101
F68E_bin.41	d__Bacteria	p__Bacteroidota	c__Bacteroidia	o__Bacteroidales	f__Rikenellaceae	g__Alistipes	s__Alistipes onderdonkii	GCF_000374505.1	33	33_1	F68E_bin.41.fa	F68E_bin.41.fa	90.27	2.5	101

Table S3. Taxonomic classifiation of the 2,421 detected MAGs

	Domain	Phylum	Class	Order	Family	Genus	Species	RefGenome	primary_cluster	secondary_cluster	repr_genome	genome	CheckM_completeness	CheckM_contamination	
F6DA_bin.94	d__Bacteria	p__Bacteroidota	c__Bacteroidia	o__Bacteroidales	f__Rikenellaceae	g__Alistipes	s__Alistipes onderdonkii	GCF_000374505.1	33	33_1	F68E_bin.41.fa	F6DA_bin.94.fa	88.88	2.72	101
F68V_bin.79	d__Bacteria	p__Bacteroidota	c__Bacteroidia	o__Bacteroidales	f__Rikenellaceae	g__Alistipes	s__Alistipes onderdonkii	GCF_000374505.1	33	33_2	F68V_bin.79.fa	F68V_bin.79.fa	84.27	4.33	101
F6DE_bin.32	d__Bacteria	p__Bacteroidota	c__Bacteroidia	o__Bacteroidales	f__Rikenellaceae	g__Alistipes	s__Alistipes onderdonkii	GCF_000374505.1	33	33_5	F6DE_bin.32.fa	F6DE_bin.32.fa	98.73	0.48	101
F6D8_bin.32	d__Bacteria	p__Bacteroidota	c__Bacteroidia	o__Bacteroidales	f__Rikenellaceae	g__Alistipes	s__Alistipes onderdonkii	GCF_000374505.1	33	33_4	F6DG_bin.80.fa	F6D8_bin.32.fa	92.49	2.16	101
F6DG_bin.80	d__Bacteria	p__Bacteroidota	c__Bacteroidia	o__Bacteroidales	f__Rikenellaceae	g__Alistipes	s__Alistipes onderdonkii	GCF_000374505.1	33	33_4	F6DG_bin.80.fa	F6DG_bin.80.fa	98.38	0.48	101
F6MX_bin.26	d__Bacteria	p__Bacteroidota	c__Bacteroidia	o__Bacteroidales	f__Rikenellaceae	g__Alistipes	s__Alistipes onderdonkii	GCF_000374505.1	33	33_3	F6MX_bin.26.fa	F6MX_bin.26.fa	98.99	0.96	101
F68E_bin.36	d__Bacteria	p__Bacteroidota	c__Bacteroidia	o__Bacteroidales	f__Rikenellaceae	g__Alistipes	s__Alistipes putredinis	GCF_000154465.1	28	28_2	F68T_bin.67.fa	F68E_bin.36.fa	90.54	2.27	101

Table S3. Taxonomic classifiation of the 2,421 detected MAGs

	Domain	Phylum	Class	Order	Family	Genus	Species	RefGenome	primary_cluster	secondary_cluster	repr_genome	genome	CheckM_completeness	CheckM_contamination	
F68K_bin.9	d__Bacteria	p__Bacteroidota	c__Bacteroidia	o__Bacteroidales	f__Rikenellaceae	g__Alistipes	s__Alistipes putredinis	GCF_000154465.1	28	28_2	F68T_bin.67.fa	F68K_bin.9.fa	99.04	0.96	101
F68N_bin.19	d__Bacteria	p__Bacteroidota	c__Bacteroidia	o__Bacteroidales	f__Rikenellaceae	g__Alistipes	s__Alistipes putredinis	GCF_000154465.1	28	28_2	F68T_bin.67.fa	F68N_bin.19.fa	94.87	1.92	101
F68T_bin.67	d__Bacteria	p__Bacteroidota	c__Bacteroidia	o__Bacteroidales	f__Rikenellaceae	g__Alistipes	s__Alistipes putredinis	GCF_000154465.1	28	28_2	F68T_bin.67.fa	F68T_bin.67.fa	99.52	0.96	101
F6D7_bin.44	d__Bacteria	p__Bacteroidota	c__Bacteroidia	o__Bacteroidales	f__Rikenellaceae	g__Alistipes	s__Alistipes putredinis	GCF_000154465.1	28	28_2	F68T_bin.67.fa	F6D7_bin.44.fa	96.79	0.99	101
F6DB_bin.23	d__Bacteria	p__Bacteroidota	c__Bacteroidia	o__Bacteroidales	f__Rikenellaceae	g__Alistipes	s__Alistipes putredinis	GCF_000154465.1	28	28_2	F68T_bin.67.fa	F6DB_bin.23.fa	97.12	0.96	101
F6DE_bin.28	d__Bacteria	p__Bacteroidota	c__Bacteroidia	o__Bacteroidales	f__Rikenellaceae	g__Alistipes	s__Alistipes putredinis	GCF_000154465.1	28	28_2	F68T_bin.67.fa	F6DE_bin.28.fa	99.52	0.96	101
F6DG_bin.87	d__Bacteria	p__Bacteroidota	c__Bacteroidia	o__Bacteroidales	f__Rikenellaceae	g__Alistipes	s__Alistipes putredinis	GCF_000154465.1	28	28_2	F68T_bin.67.fa	F6DG_bin.87.fa	99.52	1.44	101

Table S3. Taxonomic classifiation of the 2,421 detected MAGs

	Domain	Phylum	Class	Order	Family	Genus	Species	RefGenome	primary_cluster	secondary_cluster	repr_genome	genome	CheckM_completeness	CheckM_contamination	
F6MY_bin.39	d__Bacteria	p__Bacteroidota	c__Bacteroidia	o__Bacteroidales	f__Rikenellaceae	g__Alistipes	s__Alistipes putredinis	GCF_000154465.1	28	28_2	F68T_bin.67.fa	F6MY_bin.39.fa	98.72	1.44	101
F68D_bin.3	d__Bacteria	p__Bacteroidota	c__Bacteroidia	o__Bacteroidales	f__Rikenellaceae	g__Alistipes	s__Alistipes putredinis	GCF_000154465.1	28	28_1	F6D9_bin.4.fa	F68D_bin.3.fa	92.07	2.88	101
F6D8_bin.45	d__Bacteria	p__Bacteroidota	c__Bacteroidia	o__Bacteroidales	f__Rikenellaceae	g__Alistipes	s__Alistipes putredinis	GCF_000154465.1	28	28_1	F6D9_bin.4.fa	F6D8_bin.45.fa	97.28	1.68	101
F6D9_bin.4	d__Bacteria	p__Bacteroidota	c__Bacteroidia	o__Bacteroidales	f__Rikenellaceae	g__Alistipes	s__Alistipes putredinis	GCF_000154465.1	28	28_1	F6D9_bin.4.fa	F6D9_bin.4.fa	98.56	1.6	101
F68C_bin.3	d__Bacteria	p__Bacteroidota	c__Bacteroidia	o__Bacteroidales	f__Rikenellaceae	g__Alistipes	s__Alistipes senegalensis	GCF_000312145.1	30	30_2	F68C_bin.3.fa	F68C_bin.3.fa	80.17	4.73	101
F68W_bin.95	d__Bacteria	p__Bacteroidota	c__Bacteroidia	o__Bacteroidales	f__Rikenellaceae	g__Alistipes	s__Alistipes senegalensis	GCF_000312145.1	30	30_1	F68W_bin.95.fa	F68W_bin.95.fa	86.9	3.15	101
F6DH_bin.97	d__Bacteria	p__Bacteroidota	c__Bacteroidia	o__Bacteroidales	f__Rikenellaceae	g__Alistipes	s__Alistipes senegalensis	GCF_000312145.1	30	30_1	F68W_bin.95.fa	F6DH_bin.97.fa	80.36	4.49	101
F68G_bin.89	d__Bacteria	p__Bacteroidota	c__Bacteroidia	o__Bacteroidales	f__Rikenellaceae	g__Alistipes	s__Alistipes shahii	GCF_000210575.1	31	31_2	F68G_bin.89.fa	F68G_bin.89.fa	51.99	3.55	101

Table S3. Taxonomic classifiation of the 2,421 detected MAGs

	Domain	Phylum	Class	Order	Family	Genus	Species	RefGenome	primary_cluster	secondary_cluster	repr_genome	genome	CheckM_completeness	CheckM_contamination	
F68D_bin.6	d__Bacteria	p__Bacteroidota	c__Bacteroidia	o__Bacteroidales	f__Rikenellaceae	g__Alistipes	s__Alistipes shahii	GCF_000210575.1	31	31_1	F68M_bin.24.fa	F68D_bin.6.fa	95.83	0.48	101
F68F_bin.11	d__Bacteria	p__Bacteroidota	c__Bacteroidia	o__Bacteroidales	f__Rikenellaceae	g__Alistipes	s__Alistipes shahii	GCF_000210575.1	31	31_1	F68M_bin.24.fa	F68F_bin.11.fa	85.48	3.94	101
F68M_bin.24	d__Bacteria	p__Bacteroidota	c__Bacteroidia	o__Bacteroidales	f__Rikenellaceae	g__Alistipes	s__Alistipes shahii	GCF_000210575.1	31	31_1	F68M_bin.24.fa	F68M_bin.24.fa	100	0	101
F68N_bin.37	d__Bacteria	p__Bacteroidota	c__Bacteroidia	o__Bacteroidales	f__Rikenellaceae	g__Alistipes	s__Alistipes shahii	GCF_000210575.1	31	31_1	F68M_bin.24.fa	F68N_bin.37.fa	54.31	2.59	101
F68R_bin.38	d__Bacteria	p__Bacteroidota	c__Bacteroidia	o__Bacteroidales	f__Rikenellaceae	g__Alistipes	s__Alistipes shahii	GCF_000210575.1	31	31_1	F68M_bin.24.fa	F68R_bin.38.fa	71.55	3.45	101
F68T_bin.26	d__Bacteria	p__Bacteroidota	c__Bacteroidia	o__Bacteroidales	f__Rikenellaceae	g__Alistipes	s__Alistipes shahii	GCF_000210575.1	31	31_1	F68M_bin.24.fa	F68T_bin.26.fa	97.36	1.12	101
F6D7_bin.39	d__Bacteria	p__Bacteroidota	c__Bacteroidia	o__Bacteroidales	f__Rikenellaceae	g__Alistipes	s__Alistipes shahii	GCF_000210575.1	31	31_1	F68M_bin.24.fa	F6D7_bin.39.fa	88.94	3.22	101
F6D8_bin.87	d__Bacteria	p__Bacteroidota	c__Bacteroidia	o__Bacteroidales	f__Rikenellaceae	g__Alistipes	s__Alistipes shahii	GCF_000210575.1	31	31_1	F68M_bin.24.fa	F6D8_bin.87.fa	97	2	101
F6DA_bin.5	d__Bacteria	p__Bacteroidota	c__Bacteroidia	o__Bacteroidales	f__Rikenellaceae	g__Alistipes	s__Alistipes shahii	GCF_000210575.1	31	31_1	F68M_bin.24.fa	F6DA_bin.5.fa	95.43	0	101
F6DB_bin.18	d__Bacteria	p__Bacteroidota	c__Bacteroidia	o__Bacteroidales	f__Rikenellaceae	g__Alistipes	s__Alistipes shahii	GCF_000210575.1	31	31_1	F68M_bin.24.fa	F6DB_bin.18.fa	94.23	0.16	101

Table S3. Taxonomic classifiation of the 2,421 detected MAGs

	Domain	Phylum	Class	Order	Family	Genus	Species	RefGenome	primary_cluster	secondary_cluster	repr_genome	genome	CheckM_completeness	CheckM_contamination	
F6DE_bin.52	d__Bacteria	p__Bacteroidota	c__Bacteroidia	o__Bacteroidales	f__Rikenellaceae	g__Alistipes	s__Alistipes shahii	GCF_000210575.1	31	31_1	F68M_bin.24.fa	F6DE_bin.52.fa	99.6	0.18	101
F6DG_bin.62	d__Bacteria	p__Bacteroidota	c__Bacteroidia	o__Bacteroidales	f__Rikenellaceae	g__Alistipes	s__Alistipes shahii	GCF_000210575.1	31	31_1	F68M_bin.24.fa	F6DG_bin.62.fa	94.71	4.25	101
F6MX_bin.88	d__Bacteria	p__Bacteroidota	c__Bacteroidia	o__Bacteroidales	f__Rikenellaceae	g__Alistipes	s__Alistipes shahii	GCF_000210575.1	31	31_1	F68M_bin.24.fa	F6MX_bin.88.fa	99.04	0	101
F6MY_bin.85	d__Bacteria	p__Bacteroidota	c__Bacteroidia	o__Bacteroidales	f__Rikenellaceae	g__Alistipes	s__Alistipes shahii	GCF_000210575.1	31	31_1	F68M_bin.24.fa	F6MY_bin.85.fa	94.71	0.96	101
F68E_bin.48	d__Bacteria	p__Bacteroidota	c__Bacteroidia	o__Bacteroidales	f__Rikenellaceae	g__Alistipes_A	s__	N/A	104	104_6	F68E_bin.48.fa	F68E_bin.48.fa	88.14	0.96	102
F68F_bin.51	d__Bacteria	p__Bacteroidota	c__Bacteroidia	o__Bacteroidales	f__Rikenellaceae	g__Alistipes_A	s__	N/A	104	104_1	F68F_bin.51.fa	F68F_bin.51.fa	95.97	1.2	103
F6D8_bin.91	d__Bacteria	p__Bacteroidota	c__Bacteroidia	o__Bacteroidales	f__Rikenellaceae	g__Alistipes_A	s__	N/A	104	104_4	F6D8_bin.91.fa	F6D8_bin.91.fa	99.52	1.2	104
F6DA_bin.77	d__Bacteria	p__Bacteroidota	c__Bacteroidia	o__Bacteroidales	f__Rikenellaceae	g__Alistipes_A	s__	N/A	104	104_2	F6DA_bin.77.fa	F6DA_bin.77.fa	100	0.48	105
F6DB_bin.16	d__Bacteria	p__Bacteroidota	c__Bacteroidia	o__Bacteroidales	f__Rikenellaceae	g__Alistipes_A	s__	N/A	104	104_5	F6DB_bin.16.fa	F6DB_bin.16.fa	100	0.48	106
F6DG_bin.39	d__Bacteria	p__Bacteroidota	c__Bacteroidia	o__Bacteroidales	f__Rikenellaceae	g__Alistipes_A	s__	N/A	104	104_3	F6DG_bin.39.fa	F6DG_bin.39.fa	94.71	0.48	107

Table S3. Taxonomic classifiation of the 2,421 detected MAGs

	Domain	Phylum	Class	Order	Family	Genus	Species	RefGenome	primary_cluster	secondary_cluster	repr_genome	genome	CheckM_completeness	CheckM_contamination	
F6MY_bin.16	d__Bacteria	p__Bacteroidota	c__Bacteroidia	o__Bacteroidales	f__Rikenellaceae	g__Alistipes_A	s__	N/A	106	106_0	F6MY_bin.16.fa	F6MY_bin.16.fa	80.93	0.96	108
F68K_bin.44	d__Bacteria	p__Bacteroidota	c__Bacteroidia	o__Bacteroidales	f__Rikenellaceae	g__Alistipes_A	s__Alistipes_A ihumii	GCF_000321205.1	27	27_1	F68K_bin.44.fa	F68K_bin.44.fa	98.8	0	108
F6DB_bin.89	d__Bacteria	p__Bacteroidota	c__Bacteroidia	o__Bacteroidales	f__Rikenellaceae	g__Alistipes_A	s__Alistipes_A ihumii	GCF_000321205.1	27	27_1	F68K_bin.44.fa	F6DB_bin.89.fa	53.57	1.44	108
F6DG_bin.12	d__Bacteria	p__Bacteroidota	c__Bacteroidia	o__Bacteroidales	f__Rikenellaceae	g__Alistipes_A	s__Alistipes_A ihumii	GCF_000321205.1	27	27_1	F68K_bin.44.fa	F6DG_bin.12.fa	89.34	0.88	108
F6DE_bin.42	d__Bacteria	p__Bacteroidota	c__Bacteroidia	o__Bacteroidales	f__Rikenellaceae	g__Alistipes_A	s__Alistipes_A indistinctus	GCF_000231275.1	105	105_0	F6DE_bin.42.fa	F6DE_bin.42.fa	96.43	2.55	108
F6DB_bin.48	d__Bacteria	p__Bacteroidota	c__Bacteroidia	o__Bacteroidales	f__Rikenellaceae	g__Alistipes_A	s__Alistipes_A sp900240235	GCF_900240235.1	26	26_1	F6DB_bin.48.fa	F6DB_bin.48.fa	96.85	1.2	108
F6DE_bin.77	d__Bacteria	p__Bacteroidota	c__Bacteroidia	o__Bacteroidales	f__Rikenellaceae	g__Alistipes_A	s__Alistipes_A sp900240235	GCF_900240235.1	26	26_1	F6DB_bin.48.fa	F6DE_bin.77.fa	100	2.08	108
F68V_bin.25	d__Bacteria	p__Bacteroidota	c__Bacteroidia	o__Bacteroidales	f__Rikenellaceae	g__Rikenella	s__	N/A	174	174_0	F68V_bin.25.fa	F68V_bin.25.fa	74.27	1.27	109
F6D8_bin.96	d__Bacteria	p__Bacteroidota	c__Bacteroidia	o__Bacteroidales	f__Rikenellaceae	g__Tidjanibacter	s__Tidjanibacter inops	GCF_000786575.1	173	173_1	F6DE_bin.63.fa	F6D8_bin.96.fa	54.02	1.92	109

Table S3. Taxonomic classifiation of the 2,421 detected MAGs

	Domain	Phylum	Class	Order	Family	Genus	Species	RefGenome	primary_cluster	secondary_cluster	repr_genome	genome	CheckM_completeness	CheckM_contamination	
F6DE_bin.63	d__Bacteria	p__Bacteroidota	c__Bacteroidia	o__Bacteroidales	f__Rikenellaceae	g__Tidjanibacter	s__Tidjanibacter inops	GCF_000786575.1	173	173_1	F6DE_bin.63.fa	F6DE_bin.63.fa	89.13	1.84	109
F68D_bin.50	d__Bacteria	p__Bacteroidota	c__Bacteroidia	o__Bacteroidales	f__Tannerellaceae	g__Parabacteroides	s__	N/A	112	112_1	F68J_bin.20.fa	F68D_bin.50.fa	97.62	3.28	110
F68G_bin.19	d__Bacteria	p__Bacteroidota	c__Bacteroidia	o__Bacteroidales	f__Tannerellaceae	g__Parabacteroides	s__	N/A	112	112_1	F68J_bin.20.fa	F68G_bin.19.fa	97.98	1.92	111
F68J_bin.20	d__Bacteria	p__Bacteroidota	c__Bacteroidia	o__Bacteroidales	f__Tannerellaceae	g__Parabacteroides	s__	N/A	112	112_1	F68J_bin.20.fa	F68J_bin.20.fa	99.23	0.77	112
F6D9_bin.83	d__Bacteria	p__Bacteroidota	c__Bacteroidia	o__Bacteroidales	f__Tannerellaceae	g__Parabacteroides	s__	N/A	112	112_1	F68J_bin.20.fa	F6D9_bin.83.fa	82.48	2.66	113
F6DE_bin.24	d__Bacteria	p__Bacteroidota	c__Bacteroidia	o__Bacteroidales	f__Tannerellaceae	g__Parabacteroides	s__	N/A	111	111_0	F6DE_bin.24.fa	F6DE_bin.24.fa	58.79	4.31	114
F68D_bin.89	d__Bacteria	p__Bacteroidota	c__Bacteroidia	o__Bacteroidales	f__Tannerellaceae	g__Parabacteroides	s__Parabacteroides distasonis	GCF_000012845.1	107	107_7	F68D_bin.89.fa	F68D_bin.89.fa	97.5	0.38	114
F68K_bin.15	d__Bacteria	p__Bacteroidota	c__Bacteroidia	o__Bacteroidales	f__Tannerellaceae	g__Parabacteroides	s__Parabacteroides distasonis	GCF_000012845.1	107	107_7	F68D_bin.89.fa	F68K_bin.15.fa	99.1	1.47	114

Table S3. Taxonomic classifiation of the 2,421 detected MAGs

	Domain	Phylum	Class	Order	Family	Genus	Species	RefGenome	primary_cluster	secondary_cluster	repr_genome	genome	CheckM_completeness	CheckM_contamination	
F6D8_bin.85	d__Bacteria	p__Bacteroidota	c__Bacteroidia	o__Bacteroidales	f__Tannerellaceae	g__Parabacteroides	s__Parabacteroides distasonis	GCF_000012845.1	107	107_7	F68D_bin.89.fa	F6D8_bin.85.fa	86.55	2.78	114
F6DE_bin.91	d__Bacteria	p__Bacteroidota	c__Bacteroidia	o__Bacteroidales	f__Tannerellaceae	g__Parabacteroides	s__Parabacteroides distasonis	GCF_000012845.1	107	107_7	F68D_bin.89.fa	F6DE_bin.91.fa	97.12	0.38	114
F68E_bin.32	d__Bacteria	p__Bacteroidota	c__Bacteroidia	o__Bacteroidales	f__Tannerellaceae	g__Parabacteroides	s__Parabacteroides distasonis	GCF_000012845.1	107	107_10	F68E_bin.32.fa	F68E_bin.32.fa	96.24	1.6	114
F68F_bin.28	d__Bacteria	p__Bacteroidota	c__Bacteroidia	o__Bacteroidales	f__Tannerellaceae	g__Parabacteroides	s__Parabacteroides distasonis	GCF_000012845.1	107	107_8	F68F_bin.28.fa	F68F_bin.28.fa	99.35	0.96	114
F68M_bin.2	d__Bacteria	p__Bacteroidota	c__Bacteroidia	o__Bacteroidales	f__Tannerellaceae	g__Parabacteroides	s__Parabacteroides distasonis	GCF_000012845.1	107	107_4	F68M_bin.2.fa	F68M_bin.2.fa	99.42	0.13	114
F68R_bin.79	d__Bacteria	p__Bacteroidota	c__Bacteroidia	o__Bacteroidales	f__Tannerellaceae	g__Parabacteroides	s__Parabacteroides distasonis	GCF_000012845.1	107	107_6	F68R_bin.79.fa	F68R_bin.79.fa	98.11	1.86	114

Table S3. Taxonomic classifiation of the 2,421 detected MAGs

	Domain	Phylum	Class	Order	Family	Genus	Species	RefGenome	primary_cluster	secondary_cluster	repr_genome	genome	CheckM_completeness	CheckM_contamination	
F68C_bin.109	d__Bacteria	p__Bacteroidota	c__Bacteroidia	o__Bacteroidales	f__Tannerellaceae	g__Parabacteroides	s__Parabacteroides distasonis	GCF_000012845.1	107	107_1	F68V_bin.45.fa	F68C_bin.109.fa	91.97	1.22	114
F68V_bin.45	d__Bacteria	p__Bacteroidota	c__Bacteroidia	o__Bacteroidales	f__Tannerellaceae	g__Parabacteroides	s__Parabacteroides distasonis	GCF_000012845.1	107	107_1	F68V_bin.45.fa	F68V_bin.45.fa	99.42	0.13	114
F6MY_bin.77	d__Bacteria	p__Bacteroidota	c__Bacteroidia	o__Bacteroidales	f__Tannerellaceae	g__Parabacteroides	s__Parabacteroides distasonis	GCF_000012845.1	107	107_1	F68V_bin.45.fa	F6MY_bin.77.fa	93.11	1.41	114
F68W_bin.110	d__Bacteria	p__Bacteroidota	c__Bacteroidia	o__Bacteroidales	f__Tannerellaceae	g__Parabacteroides	s__Parabacteroides distasonis	GCF_000012845.1	107	107_3	F68W_bin.110.fa	F68W_bin.110.fa	68.63	0.51	114
F6DH_bin.59	d__Bacteria	p__Bacteroidota	c__Bacteroidia	o__Bacteroidales	f__Tannerellaceae	g__Parabacteroides	s__Parabacteroides distasonis	GCF_000012845.1	107	107_3	F68W_bin.110.fa	F6DH_bin.59.fa	70.28	2.63	114
F6D9_bin.69	d__Bacteria	p__Bacteroidota	c__Bacteroidia	o__Bacteroidales	f__Tannerellaceae	g__Parabacteroides	s__Parabacteroides distasonis	GCF_000012845.1	107	107_9	F6D9_bin.69.fa	F6D9_bin.69.fa	98.65	0.9	114

Table S3. Taxonomic classifiation of the 2,421 detected MAGs

	Domain	Phylum	Class	Order	Family	Genus	Species	RefGenome	primary_cluster	secondary_cluster	repr_genome	genome	CheckM_completeness	CheckM_contamination	
F68N_bin.29	d__Bacteria	p__Bacteroidota	c__Bacteroidia	o__Bacteroidales	f__Tannerellaceae	g__Parabacteroides	s__Parabacteroides distasonis	GCF_000012845.1	107	107_5	F6DA_bin.41.fa	F68N_bin.29.fa	91.05	3.27	114
F68P_bin.10	d__Bacteria	p__Bacteroidota	c__Bacteroidia	o__Bacteroidales	f__Tannerellaceae	g__Parabacteroides	s__Parabacteroides distasonis	GCF_000012845.1	107	107_5	F6DA_bin.41.fa	F68P_bin.10.fa	99.02	0.9	114
F68U_bin.36	d__Bacteria	p__Bacteroidota	c__Bacteroidia	o__Bacteroidales	f__Tannerellaceae	g__Parabacteroides	s__Parabacteroides distasonis	GCF_000012845.1	107	107_5	F6DA_bin.41.fa	F68U_bin.36.fa	95.19	0.38	114
F6DA_bin.41	d__Bacteria	p__Bacteroidota	c__Bacteroidia	o__Bacteroidales	f__Tannerellaceae	g__Parabacteroides	s__Parabacteroides distasonis	GCF_000012845.1	107	107_5	F6DA_bin.41.fa	F6DA_bin.41.fa	99.04	0.13	114
F6MX_bin.39	d__Bacteria	p__Bacteroidota	c__Bacteroidia	o__Bacteroidales	f__Tannerellaceae	g__Parabacteroides	s__Parabacteroides distasonis	GCF_000012845.1	107	107_5	F6DA_bin.41.fa	F6MX_bin.39.fa	89.94	2.08	114
F68G_bin.43	d__Bacteria	p__Bacteroidota	c__Bacteroidia	o__Bacteroidales	f__Tannerellaceae	g__Parabacteroides	s__Parabacteroides distasonis	GCF_000012845.1	107	107_2	F6DG_bin.90.fa	F68G_bin.43.fa	98.27	0.13	114

Table S3. Taxonomic classifiation of the 2,421 detected MAGs

	Domain	Phylum	Class	Order	Family	Genus	Species	RefGenome	primary_cluster	secondary_cluster	repr_genome	genome	CheckM_completeness	CheckM_contamination	
F68H_bin.5	d__Bacteria	p__Bacteroidota	c__Bacteroidia	o__Bacteroidales	f__Tannerellaceae	g__Parabacteroides	s__Parabacteroides distasonis	GCF_000012845.1	107	107_2	F6DG_bin.90.fa	F68H_bin.5.fa	97	3.79	114
F68T_bin.22	d__Bacteria	p__Bacteroidota	c__Bacteroidia	o__Bacteroidales	f__Tannerellaceae	g__Parabacteroides	s__Parabacteroides distasonis	GCF_000012845.1	107	107_2	F6DG_bin.90.fa	F68T_bin.22.fa	97.5	0.38	114
F6D7_bin.92	d__Bacteria	p__Bacteroidota	c__Bacteroidia	o__Bacteroidales	f__Tannerellaceae	g__Parabacteroides	s__Parabacteroides distasonis	GCF_000012845.1	107	107_2	F6DG_bin.90.fa	F6D7_bin.92.fa	95.76	1.63	114
F6DB_bin.1	d__Bacteria	p__Bacteroidota	c__Bacteroidia	o__Bacteroidales	f__Tannerellaceae	g__Parabacteroides	s__Parabacteroides distasonis	GCF_000012845.1	107	107_2	F6DG_bin.90.fa	F6DB_bin.1.fa	98.27	0.19	114
F6DG_bin.90	d__Bacteria	p__Bacteroidota	c__Bacteroidia	o__Bacteroidales	f__Tannerellaceae	g__Parabacteroides	s__Parabacteroides distasonis	GCF_000012845.1	107	107_2	F6DG_bin.90.fa	F6DG_bin.90.fa	99.04	0	114
F68V_bin.44	d__Bacteria	p__Bacteroidota	c__Bacteroidia	o__Bacteroidales	f__Tannerellaceae	g__Parabacteroides	s__Parabacteroides goldsteinii	GCF_000969835.1	109	109_2	F68V_bin.44.fa	F68V_bin.44.fa	63.79	0.86	114

Table S3. Taxonomic classifiation of the 2,421 detected MAGs

	Domain	Phylum	Class	Order	Family	Genus	Species	RefGenome	primary_cluster	secondary_cluster	repr_genome	genome	CheckM_completeness	CheckM_contamination	
F68W_bin.26	d__Bacteria	p__Bacteroidota	c__Bacteroidia	o__Bacteroidales	f__Tannerellaceae	g__Parabacteroides	s__Parabacteroides goldsteinii	GCF_000969835.1	109	109_1	F6DH_bin.6.fa	F68W_bin.26.fa	96.05	0.38	114
F6DH_bin.6	d__Bacteria	p__Bacteroidota	c__Bacteroidia	o__Bacteroidales	f__Tannerellaceae	g__Parabacteroides	s__Parabacteroides goldsteinii	GCF_000969835.1	109	109_1	F6DH_bin.6.fa	F6DH_bin.6.fa	96.86	0.38	114
F68J_bin.14	d__Bacteria	p__Bacteroidota	c__Bacteroidia	o__Bacteroidales	f__Tannerellaceae	g__Parabacteroides	s__Parabacteroides gordonii	GCF_000969825.1	110	110_0	F68J_bin.14.fa	F68J_bin.14.fa	98.83	1.15	114
F68R_bin.68	d__Bacteria	p__Bacteroidota	c__Bacteroidia	o__Bacteroidales	f__Tannerellaceae	g__Parabacteroides	s__Parabacteroides merdae	GCF_000154105.1	108	108_2	F68R_bin.68.fa	F68R_bin.68.fa	59.48	3.45	114
F68E_bin.9	d__Bacteria	p__Bacteroidota	c__Bacteroidia	o__Bacteroidales	f__Tannerellaceae	g__Parabacteroides	s__Parabacteroides merdae	GCF_000154105.1	108	108_1	F68V_bin.116.fa	F68E_bin.9.fa	57.07	0	114
F68M_bin.75	d__Bacteria	p__Bacteroidota	c__Bacteroidia	o__Bacteroidales	f__Tannerellaceae	g__Parabacteroides	s__Parabacteroides merdae	GCF_000154105.1	108	108_1	F68V_bin.116.fa	F68M_bin.75.fa	95.61	4.9	114
F68N_bin.36	d__Bacteria	p__Bacteroidota	c__Bacteroidia	o__Bacteroidales	f__Tannerellaceae	g__Parabacteroides	s__Parabacteroides merdae	GCF_000154105.1	108	108_1	F68V_bin.116.fa	F68N_bin.36.fa	88.68	1.67	114

Table S3. Taxonomic classifiation of the 2,421 detected MAGs

	Domain	Phylum	Class	Order	Family	Genus	Species	RefGenome	primary_cluster	secondary_cluster	repr_genome	genome	CheckM_completeness	CheckM_contamination	
F68P_bin.3	d__Bacteria	p__Bacteroidota	c__Bacteroidia	o__Bacteroidales	f__Tannerellaceae	g__Parabacteroides	s__Parabacteroides merdae	GCF_000154105.1	108	108_1	F68V_bin.116.fa	F68P_bin.3.fa	68.26	0	114
F68T_bin.3	d__Bacteria	p__Bacteroidota	c__Bacteroidia	o__Bacteroidales	f__Tannerellaceae	g__Parabacteroides	s__Parabacteroides merdae	GCF_000154105.1	108	108_1	F68V_bin.116.fa	F68T_bin.3.fa	96.96	0.38	114
F68V_bin.116	d__Bacteria	p__Bacteroidota	c__Bacteroidia	o__Bacteroidales	f__Tannerellaceae	g__Parabacteroides	s__Parabacteroides merdae	GCF_000154105.1	108	108_1	F68V_bin.116.fa	F68V_bin.116.fa	99.04	0.38	114
F6D7_bin.34	d__Bacteria	p__Bacteroidota	c__Bacteroidia	o__Bacteroidales	f__Tannerellaceae	g__Parabacteroides	s__Parabacteroides merdae	GCF_000154105.1	108	108_1	F68V_bin.116.fa	F6D7_bin.34.fa	88.83	0.58	114
F6DA_bin.39	d__Bacteria	p__Bacteroidota	c__Bacteroidia	o__Bacteroidales	f__Tannerellaceae	g__Parabacteroides	s__Parabacteroides merdae	GCF_000154105.1	108	108_1	F68V_bin.116.fa	F6DA_bin.39.fa	98.53	0.9	114
F6DB_bin.31	d__Bacteria	p__Bacteroidota	c__Bacteroidia	o__Bacteroidales	f__Tannerellaceae	g__Parabacteroides	s__Parabacteroides merdae	GCF_000154105.1	108	108_1	F68V_bin.116.fa	F6DB_bin.31.fa	91.86	3.6	114
F6DE_bin.62	d__Bacteria	p__Bacteroidota	c__Bacteroidia	o__Bacteroidales	f__Tannerellaceae	g__Parabacteroides	s__Parabacteroides merdae	GCF_000154105.1	108	108_1	F68V_bin.116.fa	F6DE_bin.62.fa	95.64	0.38	114

Table S3. Taxonomic classifiation of the 2,421 detected MAGs

	Domain	Phylum	Class	Order	Family	Genus	Species	RefGenome	primary_cluster	secondary_cluster	repr_genome	genome	CheckM_completeness	CheckM_contamination	
F6DG_bin.106	d__Bacteria	p__Bacteroidota	c__Bacteroidia	o__Bacteroidales	f__Tannerellaceae	g__Parabacteroides	s__Parabacteroides merdae	GCF_000154105.1	108	108_1	F68V_bin.116.fa	F6DG_bin.106.fa	99.23	1.25	114
F6MY_bin.51	d__Bacteria	p__Bacteroidota	c__Bacteroidia	o__Bacteroidales	f__Tannerellaceae	g__Parabacteroides	s__Parabacteroides merdae	GCF_000154105.1	108	108_1	F68V_bin.116.fa	F6MY_bin.51.fa	86.73	1.74	114
F6D9_bin.26	d__Bacteria	p__Bacteroidota	c__Bacteroidia	o__Bacteroidales	f__Tannerellaceae	g__Parabacteroides	s__Parabacteroides merdae	GCF_000154105.1	108	108_3	F6D9_bin.26.fa	F6D9_bin.26.fa	88.1	2.74	114
F68U_bin.35	d__Bacteria	p__Bacteroidota	c__Bacteroidia	o__Bacteroidales	f__UBA932	g__RC9	s__	N/A	42	42_1	F68W_bin.100.fa	F68U_bin.35.fa	91.03	0	115
F68W_bin.100	d__Bacteria	p__Bacteroidota	c__Bacteroidia	o__Bacteroidales	f__UBA932	g__RC9	s__	N/A	42	42_1	F68W_bin.100.fa	F68W_bin.100.fa	91.03	0	116
F6DH_bin.113	d__Bacteria	p__Bacteroidota	c__Bacteroidia	o__Bacteroidales	f__UBA932	g__RC9	s__	N/A	42	42_1	F68W_bin.100.fa	F6DH_bin.113.fa	91.03	0	117
F6D9_bin.38	d__Bacteria	p__Bacteroidota	c__Bacteroidia	o__Bacteroidales	f__UBA932	g__RC9	s__	N/A	24	24_0	F6D9_bin.38.fa	F6D9_bin.38.fa	87.94	0	118
F6D9_bin.51	d__Bacteria	p__Bacteroidota	c__Bacteroidia	o__Bacteroidales	f__UBA932	g__RC9	s__	N/A	41	41_0	F6D9_bin.51.fa	F6D9_bin.51.fa	62.21	0.95	119
F68G_bin.34	d__Bacteria	p__Bacteroidota	c__Bacteroidia	o__Bacteroidales	f__UBA932	g__RC9	s__RC9 sp000431015	GCA_000431015.1	43	43_3	F68G_bin.34.fa	F68G_bin.34.fa	85.32	0.79	119

Table S3. Taxonomic classifiation of the 2,421 detected MAGs

	Domain	Phylum	Class	Order	Family	Genus	Species	RefGenome	primary_cluster	secondary_cluster	repr_genome	genome	CheckM_completeness	CheckM_contamination	
F68D_bin.43	d__Bacteria	p__Bacteroidota	c__Bacteroidia	o__Bacteroidales	f__UBA932	g__RC9	s__RC9 sp000431015	GCA_000431015.1	43	43_1	F68U_bin.3.fa	F68D_bin.43.fa	74.07	0.48	119
F68U_bin.3	d__Bacteria	p__Bacteroidota	c__Bacteroidia	o__Bacteroidales	f__UBA932	g__RC9	s__RC9 sp000431015	GCA_000431015.1	43	43_1	F68U_bin.3.fa	F68U_bin.3.fa	94.1	0.48	119
F68W_bin.4	d__Bacteria	p__Bacteroidota	c__Bacteroidia	o__Bacteroidales	f__UBA932	g__RC9	s__RC9 sp000431015	GCA_000431015.1	43	43_1	F68U_bin.3.fa	F68W_bin.4.fa	83.15	2.24	119
F6DH_bin.9	d__Bacteria	p__Bacteroidota	c__Bacteroidia	o__Bacteroidales	f__UBA932	g__RC9	s__RC9 sp000431015	GCA_000431015.1	43	43_1	F68U_bin.3.fa	F6DH_bin.9.fa	91.37	2.16	119
F68J_bin.56	d__Bacteria	p__Bacteroidota	c__Bacteroidia	o__Bacteroidales	f__UBA932	g__RC9	s__RC9 sp000431015	GCA_000431015.1	43	43_2	F6MX_bin.98.fa	F68J_bin.56.fa	95.56	0.87	119
F68M_bin.63	d__Bacteria	p__Bacteroidota	c__Bacteroidia	o__Bacteroidales	f__UBA932	g__RC9	s__RC9 sp000431015	GCA_000431015.1	43	43_2	F6MX_bin.98.fa	F68M_bin.63.fa	95.53	0.63	119
F6MX_bin.98	d__Bacteria	p__Bacteroidota	c__Bacteroidia	o__Bacteroidales	f__UBA932	g__RC9	s__RC9 sp000431015	GCA_000431015.1	43	43_2	F6MX_bin.98.fa	F6MX_bin.98.fa	95.53	0.63	119
F68C_bin.43	d__Bacteria	p__Bacteroidota	c__Bacteroidia	o__Bacteroidales	f__UBA932	g__RC9	s__RC9 sp000433355	GCA_000433355.1	44	44_2	F68C_bin.43.fa	F68C_bin.43.fa	94.6	1.67	119
F6D7_bin.32	d__Bacteria	p__Bacteroidota	c__Bacteroidia	o__Bacteroidales	f__UBA932	g__RC9	s__RC9 sp000433355	GCA_000433355.1	44	44_2	F68C_bin.43.fa	F6D7_bin.32.fa	94.6	1.67	119
F68D_bin.20	d__Bacteria	p__Bacteroidota	c__Bacteroidia	o__Bacteroidales	f__UBA932	g__RC9	s__RC9 sp000433355	GCA_000433355.1	44	44_3	F68D_bin.20.fa	F68D_bin.20.fa	83.63	2.38	119

Table S3. Taxonomic classifiation of the 2,421 detected MAGs

	Domain	Phylum	Class	Order	Family	Genus	Species	RefGenome	primary_cluster	secondary_cluster	repr_genome	genome	CheckM_completeness	CheckM_contamination	
F68G_bin.17	d__Bacteria	p__Bacteroidota	c__Bacteroidia	o__Bacteroidales	f__UBA932	g__RC9	s__RC9 sp000433355	GCA_000433355.1	44	44_4	F68G_bin.17.fa	F68G_bin.17.fa	77.3	1.93	119
F68H_bin.44	d__Bacteria	p__Bacteroidota	c__Bacteroidia	o__Bacteroidales	f__UBA932	g__RC9	s__RC9 sp000433355	GCA_000433355.1	44	44_5	F68H_bin.44.fa	F68H_bin.44.fa	94.92	1.83	119
F68N_bin.27	d__Bacteria	p__Bacteroidota	c__Bacteroidia	o__Bacteroidales	f__UBA932	g__RC9	s__RC9 sp000433355	GCA_000433355.1	44	44_1	F68N_bin.27.fa	F68N_bin.27.fa	93.97	1.83	119
F68W_bin.22	d__Bacteria	p__Bacteroidota	c__Bacteroidia	o__Bacteroidales	f__UBA932	g__RC9	s__RC9 sp000433355	GCA_000433355.1	44	44_1	F68N_bin.27.fa	F68W_bin.22.fa	74.55	1.35	119
F6DH_bin.19	d__Bacteria	p__Bacteroidota	c__Bacteroidia	o__Bacteroidales	f__UBA932	g__RC9	s__RC9 sp000433355	GCA_000433355.1	44	44_1	F68N_bin.27.fa	F6DH_bin.19.fa	69.58	3.03	119
F68T_bin.4	d__Bacteria	p__Bacteroidota	c__Bacteroidia	o__Bacteroidales	f__UBA932	g__RC9	s__RC9 sp000433355	GCA_000433355.1	44	44_6	F68T_bin.4.fa	F68T_bin.4.fa	90.92	1.52	119
F68D_bin.12	d__Bacteria	p__Bacteroidota	c__Bacteroidia	o__Bacteroidales	f__UBA932	g__RC9	s__RC9 sp000434935	GCA_000434935.1	40	40_4	F68D_bin.12.fa	F68D_bin.12.fa	92.52	1.43	119
F68G_bin.83	d__Bacteria	p__Bacteroidota	c__Bacteroidia	o__Bacteroidales	f__UBA932	g__RC9	s__RC9 sp000434935	GCA_000434935.1	40	40_3	F68P_bin.55.fa	F68G_bin.83.fa	89.98	0	119
F68P_bin.55	d__Bacteria	p__Bacteroidota	c__Bacteroidia	o__Bacteroidales	f__UBA932	g__RC9	s__RC9 sp000434935	GCA_000434935.1	40	40_3	F68P_bin.55.fa	F68P_bin.55.fa	95.95	0	119
F68W_bin.108	d__Bacteria	p__Bacteroidota	c__Bacteroidia	o__Bacteroidales	f__UBA932	g__RC9	s__RC9 sp000434935	GCA_000434935.1	40	40_1	F68W_bin.108.fa	F68W_bin.108.fa	95.25	0.87	119

Table S3. Taxonomic classifiation of the 2,421 detected MAGs

	Domain	Phylum	Class	Order	Family	Genus	Species	RefGenome	primary_cluster	secondary_cluster	repr_genome	genome	CheckM_completeness	CheckM_contamination	
F6DH_bin.32	d__Bacteria	p__Bacteroidota	c__Bacteroidia	o__Bacteroidales	f__UBA932	g__RC9	s__RC9 sp000434935	GCA_000434935.1	40	40_1	F68W_bin.108.fa	F6DH_bin.32.fa	93.83	0.63	119
F6D9_bin.71	d__Bacteria	p__Bacteroidota	c__Bacteroidia	o__Bacteroidales	f__UBA932	g__RC9	s__RC9 sp000434935	GCA_000434935.1	40	40_2	F6D9_bin.71.fa	F6D9_bin.71.fa	95.95	0	119
F6MX_bin.96	d__Bacteria	p__Bacteroidota	c__Bacteroidia	o__Bacteroidales	f__UBA932	g__RC9	s__RC9 sp000434935	GCA_000434935.1	40	40_5	F6MX_bin.96.fa	F6MX_bin.96.fa	95.95	0.48	119
F68G_bin.31	d__Bacteria	p__Bacteroidota	c__Bacteroidia	o__Bacteroidales	f__UBA932	g__RC9	s__RC9 sp000435075	GCA_000435075.1	39	39_2	F68G_bin.31.fa	F68G_bin.31.fa	94.97	4.13	119
F68J_bin.79	d__Bacteria	p__Bacteroidota	c__Bacteroidia	o__Bacteroidales	f__UBA932	g__RC9	s__RC9 sp000435075	GCA_000435075.1	39	39_3	F68J_bin.79.fa	F68J_bin.79.fa	95.95	0.5	119
F68W_bin.46	d__Bacteria	p__Bacteroidota	c__Bacteroidia	o__Bacteroidales	f__UBA932	g__RC9	s__RC9 sp000435075	GCA_000435075.1	39	39_1	F6DH_bin.16.fa	F68W_bin.46.fa	94.92	0.74	119
F6DH_bin.16	d__Bacteria	p__Bacteroidota	c__Bacteroidia	o__Bacteroidales	f__UBA932	g__RC9	s__RC9 sp000435075	GCA_000435075.1	39	39_1	F6DH_bin.16.fa	F6DH_bin.16.fa	95	0.74	119
F68M_bin.76	d__Bacteria	p__Bacteroidota	c__Bacteroidia	o__Flavobacteriales	f__UBA1820	g__UBA1820	s__	N/A	439	439_3	F68M_bin.76.fa	F68M_bin.76.fa	96.32	0	120
F68V_bin.76	d__Bacteria	p__Bacteroidota	c__Bacteroidia	o__Flavobacteriales	f__UBA1820	g__UBA1820	s__	N/A	439	439_1	F68V_bin.76.fa	F68V_bin.76.fa	97.79	0	121
F6MY_bin.139	d__Bacteria	p__Bacteroidota	c__Bacteroidia	o__Flavobacteriales	f__UBA1820	g__UBA1820	s__	N/A	439	439_2	F6MY_bin.139.fa	F6MY_bin.139.fa	71.35	0.74	122

Table S3. Taxonomic classifiation of the 2,421 detected MAGs

	Domain	Phylum	Class	Order	Family	Genus	Species	RefGenome	primary_cluster	secondary_cluster	repr_genome	genome	CheckM_completeness	CheckM_contamination	
F68D_bin.4	d__Bacteria	p__Bacteroidota	c__Bacteroidia	o__Flavobacteriales	f__UBA1820	g__UBA1820	s__UBA1820 sp003150615	GCA_003150615.1	14	14_1	F6DB_bin.95.fa	F68D_bin.4.fa	98.53	0	122
F6DB_bin.95	d__Bacteria	p__Bacteroidota	c__Bacteroidia	o__Flavobacteriales	f__UBA1820	g__UBA1820	s__UBA1820 sp003150615	GCA_003150615.1	14	14_1	F6DB_bin.95.fa	F6DB_bin.95.fa	98.53	0	122
F68M_bin.87	d__Bacteria	p__Desulfobacterota_A	c__Desulfovibrionia	o__Desulfovibrionales	f__Desulfovibrionaceae	g__	s__	N/A	449	449_0	F68M_bin.87.fa	F68M_bin.87.fa	52.32	1.59	123
F68E_bin.45	d__Bacteria	p__Desulfobacterota_A	c__Desulfovibrionia	o__Desulfovibrionales	f__Desulfovibrionaceae	g__Bilophila	s__	N/A	547	547_0	F68E_bin.45.fa	F68E_bin.45.fa	95.2	1.29	124
F68T_bin.31	d__Bacteria	p__Desulfobacterota_A	c__Desulfovibrionia	o__Desulfovibrionales	f__Desulfovibrionaceae	g__Bilophila	s__Bilophila wadsworthia	GCF_000701705.1	546	546_2	F68T_bin.31.fa	F68T_bin.31.fa	95.27	0.59	124
F68G_bin.45	d__Bacteria	p__Desulfobacterota_A	c__Desulfovibrionia	o__Desulfovibrionales	f__Desulfovibrionaceae	g__Bilophila	s__Bilophila wadsworthia	GCF_000701705.1	546	546_1	F6DB_bin.79.fa	F68G_bin.45.fa	82.26	1.41	124
F68M_bin.45	d__Bacteria	p__Desulfobacterota_A	c__Desulfovibrionia	o__Desulfovibrionales	f__Desulfovibrionaceae	g__Bilophila	s__Bilophila wadsworthia	GCF_000701705.1	546	546_1	F6DB_bin.79.fa	F68M_bin.45.fa	82.83	0.59	124

Table S3. Taxonomic classifiation of the 2,421 detected MAGs

	Domain	Phylum	Class	Order	Family	Genus	Species	RefGenome	primary_cluster	secondary_cluster	repr_genome	genome	CheckM_completeness	CheckM_contamination	
F68P_bin.53	d__Bacteria	p__Desulfobacterota_A	c__Desulfovibrionia	o__Desulfovibrionales	f__Desulfovibrionaceae	g__Bilophila	s__Bilophila wadsworthia	GCF_000701705.1	546	546_1	F6DB_bin.79.fa	F68P_bin.53.fa	71.74	4.06	124
F6D7_bin.98	d__Bacteria	p__Desulfobacterota_A	c__Desulfovibrionia	o__Desulfovibrionales	f__Desulfovibrionaceae	g__Bilophila	s__Bilophila wadsworthia	GCF_000701705.1	546	546_1	F6DB_bin.79.fa	F6D7_bin.98.fa	93.74	0	124
F6DA_bin.6	d__Bacteria	p__Desulfobacterota_A	c__Desulfovibrionia	o__Desulfovibrionales	f__Desulfovibrionaceae	g__Bilophila	s__Bilophila wadsworthia	GCF_000701705.1	546	546_1	F6DB_bin.79.fa	F6DA_bin.6.fa	92.31	0	124
F6DB_bin.79	d__Bacteria	p__Desulfobacterota_A	c__Desulfovibrionia	o__Desulfovibrionales	f__Desulfovibrionaceae	g__Bilophila	s__Bilophila wadsworthia	GCF_000701705.1	546	546_1	F6DB_bin.79.fa	F6DB_bin.79.fa	97.63	0	124
F6DE_bin.55	d__Bacteria	p__Desulfobacterota_A	c__Desulfovibrionia	o__Desulfovibrionales	f__Desulfovibrionaceae	g__Bilophila	s__Bilophila wadsworthia	GCF_000701705.1	546	546_1	F6DB_bin.79.fa	F6DE_bin.55.fa	74.52	0.66	124
F6DG_bin.36	d__Bacteria	p__Desulfobacterota_A	c__Desulfovibrionia	o__Desulfovibrionales	f__Desulfovibrionaceae	g__Bilophila	s__Bilophila wadsworthia	GCF_000701705.1	546	546_1	F6DB_bin.79.fa	F6DG_bin.36.fa	89.74	0.95	124
F6MX_bin.121	d__Bacteria	p__Desulfobacterota_A	c__Desulfovibrionia	o__Desulfovibrionales	f__Desulfovibrionaceae	g__Bilophila	s__Bilophila wadsworthia	GCF_000701705.1	546	546_1	F6DB_bin.79.fa	F6MX_bin.121.fa	99.7	0.59	124

Table S3. Taxonomic classifiation of the 2,421 detected MAGs

	Domain	Phylum	Class	Order	Family	Genus	Species	RefGenome	primary_cluster	secondary_cluster	repr_genome	genome	CheckM_completeness	CheckM_contamination	
F68C_bin.54	d__Bacteria	p__Desulfobacterota_A	c__Desulfovibrionia	o__Desulfovibrionales	f__Desulfovibrionaceae	g__Desulfovibrio	s__Desulfovibrio piger	GCF_000156375.1	548	548_4	F68C_bin.54.fa	F68C_bin.54.fa	98.82	2.4	124
F68H_bin.20	d__Bacteria	p__Desulfobacterota_A	c__Desulfovibrionia	o__Desulfovibrionales	f__Desulfovibrionaceae	g__Desulfovibrio	s__Desulfovibrio piger	GCF_000156375.1	548	548_4	F68C_bin.54.fa	F68H_bin.20.fa	86.79	2.78	124
F6D7_bin.21	d__Bacteria	p__Desulfobacterota_A	c__Desulfovibrionia	o__Desulfovibrionales	f__Desulfovibrionaceae	g__Desulfovibrio	s__Desulfovibrio piger	GCF_000156375.1	548	548_4	F68C_bin.54.fa	F6D7_bin.21.fa	53.4	3.16	124
F68D_bin.107	d__Bacteria	p__Desulfobacterota_A	c__Desulfovibrionia	o__Desulfovibrionales	f__Desulfovibrionaceae	g__Desulfovibrio	s__Desulfovibrio piger	GCF_000156375.1	548	548_12	F68D_bin.107.fa	F68D_bin.107.fa	89.17	3.32	124
F68F_bin.25	d__Bacteria	p__Desulfobacterota_A	c__Desulfovibrionia	o__Desulfovibrionales	f__Desulfovibrionaceae	g__Desulfovibrio	s__Desulfovibrio piger	GCF_000156375.1	548	548_7	F68F_bin.25.fa	F68F_bin.25.fa	96.92	1.22	124
F68G_bin.52	d__Bacteria	p__Desulfobacterota_A	c__Desulfovibrionia	o__Desulfovibrionales	f__Desulfovibrionaceae	g__Desulfovibrio	s__Desulfovibrio piger	GCF_000156375.1	548	548_8	F68G_bin.52.fa	F68G_bin.52.fa	93.16	0.59	124
F68M_bin.30	d__Bacteria	p__Desulfobacterota_A	c__Desulfovibrionia	o__Desulfovibrionales	f__Desulfovibrionaceae	g__Desulfovibrio	s__Desulfovibrio piger	GCF_000156375.1	548	548_11	F68M_bin.30.fa	F68M_bin.30.fa	94.3	2.55	124

Table S3. Taxonomic classifiation of the 2,421 detected MAGs

	Domain	Phylum	Class	Order	Family	Genus	Species	RefGenome	primary_cluster	secondary_cluster	repr_genome	genome	CheckM_completeness	CheckM_contamination	
F68N_bin.73	d__Bacteria	p__Desulfobacterota_A	c__Desulfovibrionia	o__Desulfovibrionales	f__Desulfovibrionaceae	g__Desulfovibrio	s__Desulfovibrio piger	GCF_000156375.1	548	548_6	F68N_bin.73.fa	F68N_bin.73.fa	97.63	0.59	124
F68R_bin.15	d__Bacteria	p__Desulfobacterota_A	c__Desulfovibrionia	o__Desulfovibrionales	f__Desulfovibrionaceae	g__Desulfovibrio	s__Desulfovibrio piger	GCF_000156375.1	548	548_6	F68N_bin.73.fa	F68R_bin.15.fa	92.94	1.48	124
F6MY_bin.100	d__Bacteria	p__Desulfobacterota_A	c__Desulfovibrionia	o__Desulfovibrionales	f__Desulfovibrionaceae	g__Desulfovibrio	s__Desulfovibrio piger	GCF_000156375.1	548	548_6	F68N_bin.73.fa	F6MY_bin.100.fa	94.38	0.59	124
F68U_bin.101	d__Bacteria	p__Desulfobacterota_A	c__Desulfovibrionia	o__Desulfovibrionales	f__Desulfovibrionaceae	g__Desulfovibrio	s__Desulfovibrio piger	GCF_000156375.1	548	548_2	F68U_bin.101.fa	F68U_bin.101.fa	96.33	1.61	124
F68V_bin.74	d__Bacteria	p__Desulfobacterota_A	c__Desulfovibrionia	o__Desulfovibrionales	f__Desulfovibrionaceae	g__Desulfovibrio	s__Desulfovibrio piger	GCF_000156375.1	548	548_5	F68V_bin.74.fa	F68V_bin.74.fa	95.9	0.95	124
F6D9_bin.74	d__Bacteria	p__Desulfobacterota_A	c__Desulfovibrionia	o__Desulfovibrionales	f__Desulfovibrionaceae	g__Desulfovibrio	s__Desulfovibrio piger	GCF_000156375.1	548	548_3	F6D9_bin.74.fa	F6D9_bin.74.fa	94.56	0.62	124
F6DB_bin.108	d__Bacteria	p__Desulfobacterota_A	c__Desulfovibrionia	o__Desulfovibrionales	f__Desulfovibrionaceae	g__Desulfovibrio	s__Desulfovibrio piger	GCF_000156375.1	548	548_10	F6DB_bin.108.fa	F6DB_bin.108.fa	86.98	0.59	124

Table S3. Taxonomic classifiation of the 2,421 detected MAGs

	Domain	Phylum	Class	Order	Family	Genus	Species	RefGenome	primary_cluster	secondary_cluster	repr_genome	genome	CheckM_completeness	CheckM_contamination	
F6DG_bin.67	d__Bacteria	p__Desulfobacterota_A	c__Desulfovibrionia	o__Desulfovibrionales	f__Desulfovibrionaceae	g__Desulfovibrio	s__Desulfovibrio piger	GCF_000156375.1	548	548_9	F6DG_bin.67.fa	F6DG_bin.67.fa	93.47	2.73	124
F68W_bin.76	d__Bacteria	p__Desulfobacterota_A	c__Desulfovibrionia	o__Desulfovibrionales	f__Desulfovibrionaceae	g__Desulfovibrio	s__Desulfovibrio piger	GCF_000156375.1	548	548_1	F6DH_bin.60.fa	F68W_bin.76.fa	79.29	0.59	124
F6DH_bin.60	d__Bacteria	p__Desulfobacterota_A	c__Desulfovibrionia	o__Desulfovibrionales	f__Desulfovibrionaceae	g__Desulfovibrio	s__Desulfovibrio piger	GCF_000156375.1	548	548_1	F6DH_bin.60.fa	F6DH_bin.60.fa	81.66	0.59	124
F6MX_bin.82	d__Bacteria	p__Desulfobacterota_A	c__Desulfovibrionia	o__Desulfovibrionales	f__Desulfovibrionaceae	g__Desulfovibrio	s__Desulfovibrio piger	GCF_000156375.1	548	548_13	F6MX_bin.82.fa	F6MX_bin.82.fa	70.05	3.25	124
F6DE_bin.70	d__Bacteria	p__Desulfobacterota_A	c__Desulfovibrionia	o__Desulfovibrionales	f__Desulfovibrionaceae	g__Desulfovibrio	s__Desulfovibrio sp900319575	GCA_900319575.1	381	381_0	F6DE_bin.70.fa	F6DE_bin.70.fa	96.33	1.18	124
F68C_bin.17	d__Bacteria	p__Desulfobacterota_A	c__Desulfovibrionia	o__Desulfovibrionales	f__Desulfovibrionaceae	g__Mailhella	s__	N/A	551	551_0	F68C_bin.17.fa	F68C_bin.17.fa	87.33	2.68	125
F68F_bin.62	d__Bacteria	p__Desulfobacterota_A	c__Desulfovibrionia	o__Desulfovibrionales	f__Desulfovibrionaceae	g__Mailhella	s__	N/A	549	549_1	F68U_bin.106.fa	F68F_bin.62.fa	92.37	4.09	126

Table S3. Taxonomic classifiation of the 2,421 detected MAGs

	Domain	Phylum	Class	Order	Family	Genus	Species	RefGenome	primary_cluster	secondary_cluster	repr_genome	genome	CheckM_completeness	CheckM_contamination	
F68U_bin.106	d__Bacteria	p__Desulfobacterota_A	c__Desulfovibrionia	o__Desulfovibrionales	f__Desulfovibrionaceae	g__Mailhella	s__	N/A	549	549_1	F68U_bin.106.fa	F68U_bin.106.fa	96.84	1.84	127
F68H_bin.82	d__Bacteria	p__Desulfobacterota_A	c__Desulfovibrionia	o__Desulfovibrionales	f__Desulfovibrionaceae	g__Mailhella	s__Mailhella massiliensis	GCF_900155525.1	550	550_0	F68H_bin.82.fa	F68H_bin.82.fa	58.66	2.66	127
F68D_bin.73	d__Bacteria	p__Desulfobacterota_A	c__Desulfovibrionia	o__Desulfovibrionales	f__Desulfovibrionaceae	g__Mailhella	s__Mailhella sp003150275	GCA_003150275.1	382	382_1	F6DA_bin.97.fa	F68D_bin.73.fa	53.49	2.63	127
F6DA_bin.97	d__Bacteria	p__Desulfobacterota_A	c__Desulfovibrionia	o__Desulfovibrionales	f__Desulfovibrionaceae	g__Mailhella	s__Mailhella sp003150275	GCA_003150275.1	382	382_1	F6DA_bin.97.fa	F6DA_bin.97.fa	91.78	2.17	127
F68U_bin.44	d__Bacteria	p__Eremiobacterota	c__UBP9	o__UBA4705	f__UBA4705	g__	s__	N/A	509	509_0	F68U_bin.44.fa	F68U_bin.44.fa	86.57	0	128
F68J_bin.57	d__Bacteria	p__Firmicutes	c__Bacilli	o__Erysipelotrichales	f__Erysipelatoclostridiaceae	g__Catenibacterium	s__Catenibacterium mitsuokai	GCF_000173795.1	210	210_1	F68W_bin.30.fa	F68J_bin.57.fa	96.23	0	128
F68K_bin.58	d__Bacteria	p__Firmicutes	c__Bacilli	o__Erysipelotrichales	f__Erysipelatoclostridiaceae	g__Catenibacterium	s__Catenibacterium mitsuokai	GCF_000173795.1	210	210_1	F68W_bin.30.fa	F68K_bin.58.fa	96.53	0.24	128

Table S3. Taxonomic classifiation of the 2,421 detected MAGs

	Domain	Phylum	Class	Order	Family	Genus	Species	RefGenome	primary_cluster	secondary_cluster	repr_genome	genome	CheckM_completeness	CheckM_contamination	
F68W_bin.30	d__Bacteria	p__Firmicutes	c__Bacili	o__Erysipelotrichales	f__Erysipelatoclostridiaceae	g__Catenibacterium	s__Catenibacterium mitsuokai	GCF_000173795.1	210	210_1	F68W_bin.30.fa	F68W_bin.30.fa	99.06	0	128
F6DH_bin.79	d__Bacteria	p__Firmicutes	c__Bacili	o__Erysipelotrichales	f__Erysipelatoclostridiaceae	g__Catenibacterium	s__Catenibacterium mitsuokai	GCF_000173795.1	210	210_1	F68W_bin.30.fa	F6DH_bin.79.fa	99.06	0	128
F6MX_bin.21	d__Bacteria	p__Firmicutes	c__Bacili	o__Erysipelotrichales	f__Erysipelatoclostridiaceae	g__Catenibacterium	s__Catenibacterium mitsuokai	GCF_000173795.1	210	210_2	F6MX_bin.21.fa	F6MX_bin.21.fa	86.19	1.89	128
F68D_bin.31	d__Bacteria	p__Firmicutes	c__Bacili	o__Erysipelotrichales	f__Erysipelatoclostridiaceae	g__Catenibacterium	s__Catenibacterium sp000437715	GCA_000437715.1	210	210_4	F68D_bin.31.fa	F68D_bin.31.fa	64.75	3.3	128
F68U_bin.80	d__Bacteria	p__Firmicutes	c__Bacili	o__Erysipelotrichales	f__Erysipelatoclostridiaceae	g__Catenibacterium	s__Catenibacterium sp000437715	GCA_000437715.1	210	210_6	F68U_bin.80.fa	F68U_bin.80.fa	98.11	0.79	128
F6D7_bin.68	d__Bacteria	p__Firmicutes	c__Bacili	o__Erysipelotrichales	f__Erysipelatoclostridiaceae	g__Catenibacterium	s__Catenibacterium sp000437715	GCA_000437715.1	210	210_5	F6D7_bin.68.fa	F6D7_bin.68.fa	59.26	1.13	128

Table S3. Taxonomic classifiation of the 2,421 detected MAGs

	Domain	Phylum	Class	Order	Family	Genus	Species	RefGenome	primary_cluster	secondary_cluster	repr_genome	genome	CheckM_completeness	CheckM_contamination	
F68N_bin.5	d__Bacteria	p__Firmicutes	c__Bacilli	o__Erysipelotrichales	f__Erysipelatoclostridiaceae	g__Catenibacterium	s__Catenibacterium sp000437715	GCA_000437715.1	210	210_3	F6DB_bin.65.fa	F68N_bin.5.fa	92.06	1.08	128
F6DB_bin.65	d__Bacteria	p__Firmicutes	c__Bacilli	o__Erysipelotrichales	f__Erysipelatoclostridiaceae	g__Catenibacterium	s__Catenibacterium sp000437715	GCA_000437715.1	210	210_3	F6DB_bin.65.fa	F6DB_bin.65.fa	98.11	0.47	128
F68D_bin.81	d__Bacteria	p__Firmicutes	c__Bacilli	o__Erysipelotrichales	f__Erysipelatoclostridiaceae	g__Coprobacillus	s__Coprobacillus cateniformis	GCA_001312445.1	214	214_1	F68D_bin.81.fa	F68D_bin.81.fa	100	0.94	128
F68M_bin.7	d__Bacteria	p__Firmicutes	c__Bacilli	o__Erysipelotrichales	f__Erysipelatoclostridiaceae	g__Coprobacillus	s__Coprobacillus cateniformis	GCA_001312445.1	214	214_1	F68D_bin.81.fa	F68M_bin.7.fa	100	0.94	128
F68E_bin.34	d__Bacteria	p__Firmicutes	c__Bacilli	o__Erysipelotrichales	f__Erysipelatoclostridiaceae	g__Erysipelatoclostridium	s__Erysipelatoclostridium ramosum	GCF_000154485.1	211	211_1	F6D9_bin.8.fa	F68E_bin.34.fa	100	0.13	128
F68M_bin.1	d__Bacteria	p__Firmicutes	c__Bacilli	o__Erysipelotrichales	f__Erysipelatoclostridiaceae	g__Erysipelatoclostridium	s__Erysipelatoclostridium ramosum	GCF_000154485.1	211	211_1	F6D9_bin.8.fa	F68M_bin.1.fa	100	0	128

Table S3. Taxonomic classifiation of the 2,421 detected MAGs

	Domain	Phylum	Class	Order	Family	Genus	Species	RefGenome	primary_cluster	secondary_cluster	repr_genome	genome	CheckM_completeness	CheckM_contamination	
F68V_bin.38	d__Bacteria	p__Firmicutes	c__Bacilli	o__Erysipelotrichales	f__Erysipelatoclostridiaceae	g__Erysipelatoclostridium	s__Erysipelatoclostridium ramosum	GCF_000154485.1	211	211_1	F6D9_bin.8.fa	F68V_bin.38.fa	100	0	128
F6D9_bin.8	d__Bacteria	p__Firmicutes	c__Bacilli	o__Erysipelotrichales	f__Erysipelatoclostridiaceae	g__Erysipelatoclostridium	s__Erysipelatoclostridium ramosum	GCF_000154485.1	211	211_1	F6D9_bin.8.fa	F6D9_bin.8.fa	100	0	128
F68C_bin.51	d__Bacteria	p__Firmicutes	c__Bacilli	o__Erysipelotrichales	f__Erysipelatoclostridiaceae	g__Erysipelatoclostridium	s__Erysipelatoclostridium sp000752095	GCF_000752095.2	213	213_4	F68C_bin.51.fa	F68C_bin.51.fa	99.06	0	128
F6D7_bin.95	d__Bacteria	p__Firmicutes	c__Bacilli	o__Erysipelotrichales	f__Erysipelatoclostridiaceae	g__Erysipelatoclostridium	s__Erysipelatoclostridium sp000752095	GCF_000752095.2	213	213_4	F68C_bin.51.fa	F6D7_bin.95.fa	99.06	1.89	128
F68F_bin.98	d__Bacteria	p__Firmicutes	c__Bacilli	o__Erysipelotrichales	f__Erysipelatoclostridiaceae	g__Erysipelatoclostridium	s__Erysipelatoclostridium sp000752095	GCF_000752095.2	213	213_8	F68F_bin.98.fa	F68F_bin.98.fa	95.05	3.77	128

Table S3. Taxonomic classifiation of the 2,421 detected MAGs

	Domain	Phylum	Class	Order	Family	Genus	Species	RefGenome	primary_cluster	secondary_cluster	repr_genome	genome	CheckM_completeness	CheckM_contamination	
F68J_bin.59	d__Bacteria	p__Firmicutes	c__Bacilli	o__Erysipelotrichales	f__Erysipelatoclostridiaceae	g__Erysipelatoclostridium	s__Erysipelatoclostridium sp000752095	GCF_000752095.2	213	213_1	F68J_bin.59.fa	F68J_bin.59.fa	90.25	0	128
F6DB_bin.52	d__Bacteria	p__Firmicutes	c__Bacilli	o__Erysipelotrichales	f__Erysipelatoclostridiaceae	g__Erysipelatoclostridium	s__Erysipelatoclostridium sp000752095	GCF_000752095.2	213	213_1	F68J_bin.59.fa	F6DB_bin.52.fa	54.81	0	128
F68P_bin.39	d__Bacteria	p__Firmicutes	c__Bacilli	o__Erysipelotrichales	f__Erysipelatoclostridiaceae	g__Erysipelatoclostridium	s__Erysipelatoclostridium sp000752095	GCF_000752095.2	213	213_2	F68P_bin.39.fa	F68P_bin.39.fa	99.06	0.31	128
F68R_bin.6	d__Bacteria	p__Firmicutes	c__Bacilli	o__Erysipelotrichales	f__Erysipelatoclostridiaceae	g__Erysipelatoclostridium	s__Erysipelatoclostridium sp000752095	GCF_000752095.2	213	213_3	F68R_bin.6.fa	F68R_bin.6.fa	91.51	0.94	128
F68U_bin.13	d__Bacteria	p__Firmicutes	c__Bacilli	o__Erysipelotrichales	f__Erysipelatoclostridiaceae	g__Erysipelatoclostridium	s__Erysipelatoclostridium sp000752095	GCF_000752095.2	213	213_9	F68U_bin.13.fa	F68U_bin.13.fa	63.04	0.94	128

Table S3. Taxonomic classifiation of the 2,421 detected MAGs

	Domain	Phylum	Class	Order	Family	Genus	Species	RefGenome	primary_cluster	secondary_cluster	repr_genome	genome	CheckM_completeness	CheckM_contamination	
F6D8_bin.59	d__Bacteria	p__Firmicutes	c__Bacilli	o__Erysipelotrichales	f__Erysipelatoclostridiaceae	g__Erysipelatoclostridium	s__Erysipelatoclostridium sp000752095	GCF_000752095.2	213	213_5	F6D8_bin.59.fa	F6D8_bin.59.fa	88.12	0.09	128
F6DG_bin.43	d__Bacteria	p__Firmicutes	c__Bacilli	o__Erysipelotrichales	f__Erysipelatoclostridiaceae	g__Erysipelatoclostridium	s__Erysipelatoclostridium sp000752095	GCF_000752095.2	213	213_5	F6D8_bin.59.fa	F6DG_bin.43.fa	99.06	2.36	128
F6DA_bin.61	d__Bacteria	p__Firmicutes	c__Bacilli	o__Erysipelotrichales	f__Erysipelatoclostridiaceae	g__Erysipelatoclostridium	s__Erysipelatoclostridium sp000752095	GCF_000752095.2	213	213_6	F6DA_bin.61.fa	F6DA_bin.61.fa	99.06	0	128
F6MY_bin.19	d__Bacteria	p__Firmicutes	c__Bacilli	o__Erysipelotrichales	f__Erysipelatoclostridiaceae	g__Erysipelatoclostridium	s__Erysipelatoclostridium sp000752095	GCF_000752095.2	213	213_7	F6MY_bin.19.fa	F6MY_bin.19.fa	88.5	0.94	128
F68J_bin.68	d__Bacteria	p__Firmicutes	c__Bacilli	o__Erysipelotrichales	f__Erysipelatoclostridiaceae	g__Erysipelatoclostridium	s__Erysipelatoclostridium sp003024675	GCF_003024675.1	212	212_1	F68J_bin.68.fa	F68J_bin.68.fa	57.37	0	128

Table S3. Taxonomic classifiation of the 2,421 detected MAGs

	Domain	Phylum	Class	Order	Family	Genus	Species	RefGenome	primary_cluster	secondary_cluster	repr_genome	genome	CheckM_completeness	CheckM_contamination	
F68T_bin.44	d__Bacteria	p__Firmicutes	c__Bacilli	o__Erysipelotrichales	f__Erysipelatoclostridiaceae	g__Erysipelatoclostridium	s__Erysipelatoclostridium sp003024675	GCF_003024675.1	212	212_2	F68T_bin.44.fa	F68T_bin.44.fa	96.7	1.42	128
F68J_bin.31	d__Bacteria	p__Firmicutes	c__Bacilli	o__Erysipelotrichales	f__Erysipelatoclostridiaceae	g__Massiliomicrobiota	s__Massiliomicrobiota sp002160815	GCF_002160815.1	215	215_0	F68J_bin.31.fa	F68J_bin.31.fa	81.76	2.02	128
F68J_bin.63	d__Bacteria	p__Firmicutes	c__Bacilli	o__Erysipelotrichales	f__Erysipelotrichaceae	g__	s__	N/A	217	217_1	F68J_bin.63.fa	F68J_bin.63.fa	89.4	4.13	129
F68R_bin.89	d__Bacteria	p__Firmicutes	c__Bacilli	o__Erysipelotrichales	f__Erysipelotrichaceae	g__	s__	N/A	217	217_2	F68R_bin.89.fa	F68R_bin.89.fa	53.18	4.76	130
F6MX_bin.48	d__Bacteria	p__Firmicutes	c__Bacilli	o__Erysipelotrichales	f__Erysipelotrichaceae	g__	s__	N/A	595	595_0	F6MX_bin.48.fa	F6MX_bin.48.fa	90.37	1.9	131
F68E_bin.47	d__Bacteria	p__Firmicutes	c__Bacilli	o__Erysipelotrichales	f__Erysipelotrichaceae	g__Absiella	s__Absiella innocuum	GCF_000371425.1	438	438_2	F68E_bin.47.fa	F68E_bin.47.fa	95.75	0.09	131
F68G_bin.24	d__Bacteria	p__Firmicutes	c__Bacilli	o__Erysipelotrichales	f__Erysipelotrichaceae	g__Absiella	s__Absiella innocuum	GCF_000371425.1	438	438_1	F6MY_bin.99.fa	F68G_bin.24.fa	90.8	1.89	131

Table S3. Taxonomic classifiation of the 2,421 detected MAGs

	Domain	Phylum	Class	Order	Family	Genus	Species	RefGenome	primary_cluster	secondary_cluster	repr_genome	genome	CheckM_completeness	CheckM_contamination	
F6MX_bin.113	d__Bacteria	p__Firmicutes	c__Bacilli	o__Erysipelotrichales	f__Erysipelotrichaceae	g__Absiella	s__Absiella innocuum	GCF_000371425.1	438	438_1	F6MY_bin.99.fa	F6MX_bin.113.fa	72.14	0	131
F6MY_bin.99	d__Bacteria	p__Firmicutes	c__Bacilli	o__Erysipelotrichales	f__Erysipelotrichaceae	g__Absiella	s__Absiella innocuum	GCF_000371425.1	438	438_1	F6MY_bin.99.fa	F6MY_bin.99.fa	98.11	1.42	131
F68M_bin.29	d__Bacteria	p__Firmicutes	c__Bacilli	o__Erysipelotrichales	f__Erysipelotrichaceae	g__CAG-536	s__	N/A	209	209_0	F68M_bin.29.fa	F68M_bin.29.fa	70.6	2.72	132
F68K_bin.65	d__Bacteria	p__Firmicutes	c__Bacilli	o__Erysipelotrichales	f__Erysipelotrichaceae	g__Dielma	s__Dielma fastidiosa	GCF_000313565.1	6	6_0	F68K_bin.65.fa	F68K_bin.65.fa	67.68	1.18	132
F68F_bin.59	d__Bacteria	p__Firmicutes	c__Bacilli	o__Erysipelotrichales	f__Erysipelotrichaceae	g__Faecalitalea	s__Faecalitalea cylindroides	GCF_000469305.1	208	208_0	F68F_bin.59.fa	F68F_bin.59.fa	92.67	1.08	132
F68D_bin.101	d__Bacteria	p__Firmicutes	c__Bacilli	o__Erysipelotrichales	f__Erysipelotrichaceae	g__Holdemanella	s__	N/A	206	206_1	F68D_bin.101.fa	F68D_bin.101.fa	57.53	3.51	133
F68F_bin.46	d__Bacteria	p__Firmicutes	c__Bacilli	o__Erysipelotrichales	f__Erysipelotrichaceae	g__Holdemanella	s__	N/A	206	206_2	F68F_bin.46.fa	F68F_bin.46.fa	97.3	0.84	134

Table S3. Taxonomic classifiation of the 2,421 detected MAGs

	Domain	Phylum	Class	Order	Family	Genus	Species	RefGenome	primary_cluster	secondary_cluster	repr_genome	genome	CheckM_completeness	CheckM_contamination	
F68K_bin.77	d__Bacteria	p__Firmicutes	c__Bacilli	o__Erysipelotrichales	f__Erysipelotrichaceae	g__Holdemanella	s__	N/A	207	207_14	F68K_bin.77.fa	F68K_bin.77.fa	97.84	1.38	135
F68N_bin.51	d__Bacteria	p__Firmicutes	c__Bacilli	o__Erysipelotrichales	f__Erysipelotrichaceae	g__Holdemanella	s__	N/A	207	207_11	F68N_bin.51.fa	F68N_bin.51.fa	61.36	0	136
F68H_bin.16	d__Bacteria	p__Firmicutes	c__Bacilli	o__Erysipelotrichales	f__Erysipelotrichaceae	g__Holdemanella	s__Holdemanella biformis	GCF_000156655.1	207	207_5	F68H_bin.16.fa	F68H_bin.16.fa	95.42	3.67	136
F68J_bin.73	d__Bacteria	p__Firmicutes	c__Bacilli	o__Erysipelotrichales	f__Erysipelotrichaceae	g__Holdemanella	s__Holdemanella biformis	GCF_000156655.1	207	207_2	F68J_bin.73.fa	F68J_bin.73.fa	65.23	1.75	136
F68M_bin.84	d__Bacteria	p__Firmicutes	c__Bacilli	o__Erysipelotrichales	f__Erysipelotrichaceae	g__Holdemanella	s__Holdemanella biformis	GCF_000156655.1	207	207_4	F68M_bin.84.fa	F68M_bin.84.fa	96.09	3.34	136
F68R_bin.63	d__Bacteria	p__Firmicutes	c__Bacilli	o__Erysipelotrichales	f__Erysipelotrichaceae	g__Holdemanella	s__Holdemanella biformis	GCF_000156655.1	207	207_7	F68R_bin.63.fa	F68R_bin.63.fa	79.3	2.98	136

Table S3. Taxonomic classifiation of the 2,421 detected MAGs

	Domain	Phylum	Class	Order	Family	Genus	Species	RefGenome	primary_cluster	secondary_cluster	repr_genome	genome	CheckM_completeness	CheckM_contamination	
F68U_bin.58	d__Bacteria	p__Firmicutes	c__Bacilli	o__Erysipelotrichales	f__Erysipelotrichaceae	g__Holdemanella	s__Holdemanella biformis	GCF_000156655.1	207	207_1	F68U_bin.58.fa	F68U_bin.58.fa	71.53	1.75	136
F6DH_bin.100	d__Bacteria	p__Firmicutes	c__Bacilli	o__Erysipelotrichales	f__Erysipelotrichaceae	g__Holdemanella	s__Holdemanella biformis	GCF_000156655.1	207	207_1	F68U_bin.58.fa	F6DH_bin.100.fa	74.66	4.74	136
F6DG_bin.101	d__Bacteria	p__Firmicutes	c__Bacilli	o__Erysipelotrichales	f__Erysipelotrichaceae	g__Holdemanella	s__Holdemanella biformis	GCF_000156655.1	207	207_3	F6DG_bin.101.fa	F6DG_bin.101.fa	95.55	0.71	136
F6MY_bin.133	d__Bacteria	p__Firmicutes	c__Bacilli	o__Erysipelotrichales	f__Erysipelotrichaceae	g__Holdemanella	s__Holdemanella biformis	GCF_000156655.1	207	207_6	F6MY_bin.133.fa	F6MY_bin.133.fa	64.86	1.75	136
F68D_bin.61	d__Bacteria	p__Firmicutes	c__Bacilli	o__Erysipelotrichales	f__Erysipelotrichaceae	g__Holdemanella	s__Holdemanella sp002299315	GCA_002299315.1	207	207_10	F68D_bin.61.fa	F68D_bin.61.fa	85.04	4.14	136
F68J_bin.12	d__Bacteria	p__Firmicutes	c__Bacilli	o__Erysipelotrichales	f__Erysipelotrichaceae	g__Holdemanella	s__Holdemanella sp002299315	GCA_002299315.1	207	207_9	F68J_bin.12.fa	F68J_bin.12.fa	88.79	4.48	136

Table S3. Taxonomic classifiation of the 2,421 detected MAGs

	Domain	Phylum	Class	Order	Family	Genus	Species	RefGenome	primary_cluster	secondary_cluster	repr_genome	genome	CheckM_completeness	CheckM_contamination	
F68U_bin.43	d__Bacteria	p__Firmicutes	c__Bacilli	o__Erysipelotrichales	f__Erysipelotrichaceae	g__Holdemanella	s__Holdemanella sp002299315	GCA_002299315.1	207	207_8	F68U_bin.43.fa	F68U_bin.43.fa	70.67	1.75	136
F6DH_bin.34	d__Bacteria	p__Firmicutes	c__Bacilli	o__Erysipelotrichales	f__Erysipelotrichaceae	g__Holdemanella	s__Holdemanella sp002299315	GCA_002299315.1	207	207_8	F68U_bin.43.fa	F6DH_bin.34.fa	77.4	3.54	136
F6DB_bin.50	d__Bacteria	p__Firmicutes	c__Bacilli	o__Erysipelotrichales	f__Erysipelotrichaceae	g__Holdemanella	s__Holdemanella sp002299315	GCA_002299315.1	207	207_13	F6DB_bin.50.fa	F6DB_bin.50.fa	83.63	1.51	136
F6MX_bin.50	d__Bacteria	p__Firmicutes	c__Bacilli	o__Erysipelotrichales	f__Erysipelotrichaceae	g__Holdemanella	s__Holdemanella sp002299315	GCA_002299315.1	207	207_12	F6MX_bin.50.fa	F6MX_bin.50.fa	86.36	3.54	136
F68G_bin.29	d__Bacteria	p__Firmicutes	c__Bacilli	o__Erysipelotrichales	f__Erysipelotrichaceae	g__Solobacterium	s__	N/A	205	205_2	F68G_bin.29.fa	F68G_bin.29.fa	92.52	0.95	137
F68J_bin.6	d__Bacteria	p__Firmicutes	c__Bacilli	o__Erysipelotrichales	f__Erysipelotrichaceae	g__Solobacterium	s__	N/A	205	205_3	F68J_bin.6.fa	F68J_bin.6.fa	94.29	0	138

Table S3. Taxonomic classifiation of the 2,421 detected MAGs

	Domain	Phylum	Class	Order	Family	Genus	Species	RefGenome	primary_cluster	secondary_cluster	repr_genome	genome	CheckM_completeness	CheckM_contamination	
F68W_bin.85	d__Bacteria	p__Firmicutes	c__Bacilli	o__Erysipelotrichales	f__Erysipelotrichaceae	g__Solobacterium	s__	N/A	205	205_1	F68W_bin.85.fa	F68W_bin.85.fa	85.76	1.43	139
F6DH_bin.38	d__Bacteria	p__Firmicutes	c__Bacilli	o__Erysipelotrichales	f__Erysipelotrichaceae	g__Solobacterium	s__	N/A	205	205_1	F68W_bin.85.fa	F6DH_bin.38.fa	76.49	2.38	140
F68D_bin.7	d__Bacteria	p__Firmicutes	c__Bacilli	o__Erysipelotrichales	f__Erysipelotrichaceae	g__UBA636	s__	N/A	216	216_3	F68D_bin.7.fa	F68D_bin.7.fa	95.71	0.71	141
F68H_bin.18	d__Bacteria	p__Firmicutes	c__Bacilli	o__Erysipelotrichales	f__Erysipelotrichaceae	g__UBA636	s__	N/A	216	216_5	F68H_bin.18.fa	F68H_bin.18.fa	80.63	1.19	142
F68J_bin.82	d__Bacteria	p__Firmicutes	c__Bacilli	o__Erysipelotrichales	f__Erysipelotrichaceae	g__UBA636	s__	N/A	216	216_4	F68J_bin.82.fa	F68J_bin.82.fa	86.67	2.14	143
F68U_bin.57	d__Bacteria	p__Firmicutes	c__Bacilli	o__Erysipelotrichales	f__Erysipelotrichaceae	g__UBA636	s__	N/A	216	216_1	F68U_bin.57.fa	F68U_bin.57.fa	60.45	2.9	144
F6MX_bin.49	d__Bacteria	p__Firmicutes	c__Bacilli	o__Erysipelotrichales	f__Erysipelotrichaceae	g__UBA636	s__	N/A	216	216_2	F6MX_bin.49.fa	F6MX_bin.49.fa	74.52	0.71	145
F68M_bin.25	d__Bacteria	p__Firmicutes	c__Bacilli	o__Lactobacillales	f__Aerococcaceae	g__Granulicatella	s__	N/A	151	151_0	F68M_bin.25.fa	F68M_bin.25.fa	65.96	0.27	146

Table S3. Taxonomic classifiation of the 2,421 detected MAGs

	Domain	Phylum	Class	Order	Family	Genus	Species	RefGenome	primary_cluster	secondary_cluster	repr_genome	genome	CheckM_completeness	CheckM_contamination	
F68M_bin.15	d__Bacteria	p__Firmicutes	c__Bacilli	o__Lactobacillales	f__Enterococcaceae	g__Enterococcus	s__Enterococcus faecalis	GCF_000392875.1	150	150_1	F68M_bin.15.fa	F68M_bin.15.fa	99.63	0	146
F6DE_bin.11	d__Bacteria	p__Firmicutes	c__Bacilli	o__Lactobacillales	f__Enterococcaceae	g__Enterococcus	s__Enterococcus faecalis	GCF_000392875.1	150	150_2	F6DE_bin.11.fa	F6DE_bin.11.fa	99.63	0.07	146
F68T_bin.55	d__Bacteria	p__Firmicutes	c__Bacilli	o__Lactobacillales	f__Enterococcaceae	g__Enterococcus_D	s__Enterococcus_D casseliflavus	GCF_001544095.1	10	10_0	F68T_bin.55.fa	F68T_bin.55.fa	76.58	2.79	146
F6D9_bin.84	d__Bacteria	p__Firmicutes	c__Bacilli	o__Lactobacillales	f__Enterococcaceae	g__Enterococcus_D	s__Enterococcus_D gallinarum	GCF_001544275.1	11	11_0	F6D9_bin.84.fa	F6D9_bin.84.fa	94.06	2.1	146
F68D_bin.68	d__Bacteria	p__Firmicutes	c__Bacilli	o__Lactobacillales	f__Lactobacillaceae	g__Lactobacillus	s__	N/A	378	378_3	F68D_bin.68.fa	F68D_bin.68.fa	91.11	2.4	147
F68R_bin.11	d__Bacteria	p__Firmicutes	c__Bacilli	o__Lactobacillales	f__Lactobacillaceae	g__Lactobacillus	s__	N/A	378	378_1	F68R_bin.11.fa	F68R_bin.11.fa	98.11	0	148
F68T_bin.45	d__Bacteria	p__Firmicutes	c__Bacilli	o__Lactobacillales	f__Lactobacillaceae	g__Lactobacillus	s__	N/A	378	378_4	F68T_bin.45.fa	F68T_bin.45.fa	92.89	1.11	149

Table S3. Taxonomic classifiation of the 2,421 detected MAGs

	Domain	Phylum	Class	Order	Family	Genus	Species	RefGenome	primary_cluster	secondary_cluster	repr_genome	genome	CheckM_completeness	CheckM_contamination	
F6D8_bin.20	d__Bacteria	p__Firmicutes	c__Bacilli	o__Lactobacillales	f__Lactobacillaceae	g__Lactobacillus	s__	N/A	378	378_2	F6D8_bin.20.fa	F6D8_bin.20.fa	98.83	0	150
F68H_bin.40	d__Bacteria	p__Firmicutes	c__Bacilli	o__Lactobacillales	f__Lactobacillaceae	g__Lactobacillus	s__Lactobacillus amylovorus	GCF_002706375.1	379	379_2	F68H_bin.40.fa	F68H_bin.40.fa	97.32	0	150
F68D_bin.69	d__Bacteria	p__Firmicutes	c__Bacilli	o__Lactobacillales	f__Lactobacillaceae	g__Lactobacillus	s__Lactobacillus kitasatonis	GCF_000615285.1	379	379_1	F68D_bin.69.fa	F68D_bin.69.fa	96.4	0.81	150
F68T_bin.79	d__Bacteria	p__Firmicutes	c__Bacilli	o__Lactobacillales	f__Lactobacillaceae	g__Lactobacillus_B	s__Lactobacillus_B salivarius	GCF_001435955.1	171	171_0	F68T_bin.79.fa	F68T_bin.79.fa	95.9	1.57	150
F68J_bin.77	d__Bacteria	p__Firmicutes	c__Bacilli	o__Lactobacillales	f__Lactobacillaceae	g__Lactobacillus_H	s__Lactobacillus_H fermentum	GCF_000159215.1	12	12_0	F68J_bin.77.fa	F68J_bin.77.fa	79.81	1.91	150
F68H_bin.50	d__Bacteria	p__Firmicutes	c__Bacilli	o__Lactobacillales	f__Lactobacillaceae	g__Lactobacillus_H	s__Lactobacillus_H mucosae	GCF_001436025.1	377	377_1	F68H_bin.50.fa	F68H_bin.50.fa	99.18	0.54	150
F68M_bin.3	d__Bacteria	p__Firmicutes	c__Bacilli	o__Lactobacillales	f__Lactobacillaceae	g__Lactobacillus_H	s__Lactobacillus_H mucosae	GCF_001436025.1	377	377_4	F68M_bin.3.fa	F68M_bin.3.fa	90.1	3.58	150

Table S3. Taxonomic classifiation of the 2,421 detected MAGs

	Domain	Phylum	Class	Order	Family	Genus	Species	RefGenome	primary_cluster	secondary_cluster	repr_genome	genome	CheckM_completeness	CheckM_contamination	
F68R_bin.55	d__Bacteria	p__Firmicutes	c__Bacilli	o__Lactobacillales	f__Lactobacillaceae	g__Lactobacillus_H	s__Lactobacillus_H mucosae	GCF_001436025.1	377	377_2	F68R_bin.55.fa	F68R_bin.55.fa	66.26	0.7	150
F68D_bin.87	d__Bacteria	p__Firmicutes	c__Bacilli	o__Lactobacillales	f__Lactobacillaceae	g__Lactobacillus_H	s__Lactobacillus_H mucosae	GCF_001436025.1	377	377_3	F6D8_bin.37.fa	F68D_bin.87.fa	74.14	0	150
F6D8_bin.37	d__Bacteria	p__Firmicutes	c__Bacilli	o__Lactobacillales	f__Lactobacillaceae	g__Lactobacillus_H	s__Lactobacillus_H mucosae	GCF_001436025.1	377	377_3	F6D8_bin.37.fa	F6D8_bin.37.fa	99.18	0	150
F6D9_bin.96	d__Bacteria	p__Firmicutes	c__Bacilli	o__Lactobacillales	f__Lactobacillaceae	g__Weissella	s__Weissella cibaria	GCF_000193635.1	153	153_0	F6D9_bin.96.fa	F6D9_bin.96.fa	61.11	1.01	150
F68R_bin.22	d__Bacteria	p__Firmicutes	c__Bacilli	o__Lactobacillales	f__Lactobacillaceae	g__Weissella	s__Weissella confusa	GCF_001436895.1	152	152_1	F68T_bin.90.fa	F68R_bin.22.fa	62.67	1.03	150
F68T_bin.90	d__Bacteria	p__Firmicutes	c__Bacilli	o__Lactobacillales	f__Lactobacillaceae	g__Weissella	s__Weissella confusa	GCF_001436895.1	152	152_1	F68T_bin.90.fa	F68T_bin.90.fa	97.55	0.2	150
F68J_bin.15	d__Bacteria	p__Firmicutes	c__Bacilli	o__Lactobacillales	f__Streptococcaceae	g__Lactococcus	s__Lactococcus petauri	GCF_002154895.1	164	164_0	F68J_bin.15.fa	F68J_bin.15.fa	83	3.91	150
F68M_bin.43	d__Bacteria	p__Firmicutes	c__Bacilli	o__Lactobacillales	f__Streptococcaceae	g__Streptococcus	s__	N/A	159	159_0	F68M_bin.43.fa	F68M_bin.43.fa	52.8	1.22	151
F6MX_bin.93	d__Bacteria	p__Firmicutes	c__Bacilli	o__Lactobacillales	f__Streptococcaceae	g__Streptococcus	s__	N/A	154	154_2	F6MX_bin.93.fa	F6MX_bin.93.fa	52.51	4.98	152

Table S3. Taxonomic classifiation of the 2,421 detected MAGs

	Domain	Phylum	Class	Order	Family	Genus	Species	RefGenome	primary_cluster	secondary_cluster	repr_genome	genome	CheckM_completeness	CheckM_contamination	
F68M_bin.77	d__Bacteria	p__Firmicutes	c__Bacilli	o__Lactobacillales	f__Streptococcaceae	g__Streptococcus	s__Streptococcus anginosus	GCF_002088025.1	156	156_1	F68M_bin.77.fa	F68M_bin.77.fa	84.37	0.83	152
F6MX_bin.79	d__Bacteria	p__Firmicutes	c__Bacilli	o__Lactobacillales	f__Streptococcaceae	g__Streptococcus	s__Streptococcus anginosus	GCF_002088025.1	156	156_2	F6MX_bin.79.fa	F6MX_bin.79.fa	96.74	1.77	152
F68N_bin.2	d__Bacteria	p__Firmicutes	c__Bacilli	o__Lactobacillales	f__Streptococcaceae	g__Streptococcus	s__Streptococcus gallolyticus	GCF_002000985.1	162	162_2	F68N_bin.2.fa	F68N_bin.2.fa	96.2	0.46	152
F68C_bin.89	d__Bacteria	p__Firmicutes	c__Bacilli	o__Lactobacillales	f__Streptococcaceae	g__Streptococcus	s__Streptococcus infantarius	GCF_000154985.1	161	161_2	F68C_bin.89.fa	F68C_bin.89.fa	100	0	152
F68N_bin.55	d__Bacteria	p__Firmicutes	c__Bacilli	o__Lactobacillales	f__Streptococcaceae	g__Streptococcus	s__Streptococcus infantarius	GCF_000154985.1	161	161_2	F68C_bin.89.fa	F68N_bin.55.fa	94.66	0.28	152
F68R_bin.33	d__Bacteria	p__Firmicutes	c__Bacilli	o__Lactobacillales	f__Streptococcaceae	g__Streptococcus	s__Streptococcus infantarius	GCF_000154985.1	161	161_1	F6DB_bin.102.fa	F68R_bin.33.fa	98.37	1.53	152

Table S3. Taxonomic classifiation of the 2,421 detected MAGs

	Domain	Phylum	Class	Order	Family	Genus	Species	RefGenome	primary_cluster	secondary_cluster	repr_genome	genome	CheckM_completeness	CheckM_contamination	
F68T_bin.63	d__Bacteria	p__Firmicutes	c__Bacilli	o__Lactobacillales	f__Streptococcaceae	g__Streptococcus	s__Streptococcus infantarius	GCF_000154985.1	161	161_1	F6DB_bin.102.fa	F68T_bin.63.fa	100	1.34	152
F6DB_bin.102	d__Bacteria	p__Firmicutes	c__Bacilli	o__Lactobacillales	f__Streptococcaceae	g__Streptococcus	s__Streptococcus infantarius	GCF_000154985.1	161	161_1	F6DB_bin.102.fa	F6DB_bin.102.fa	99.35	0.19	152
F6DG_bin.31	d__Bacteria	p__Firmicutes	c__Bacilli	o__Lactobacillales	f__Streptococcaceae	g__Streptococcus	s__Streptococcus infantarius	GCF_000154985.1	161	161_1	F6DB_bin.102.fa	F6DG_bin.31.fa	74.3	1	152
F6D7_bin.37	d__Bacteria	p__Firmicutes	c__Bacilli	o__Lactobacillales	f__Streptococcaceae	g__Streptococcus	s__Streptococcus parasanguinis_B	GCF_000963275.1	158	158_0	F6D7_bin.37.fa	F6D7_bin.37.fa	85.52	2.62	152
F68D_bin.75	d__Bacteria	p__Firmicutes	c__Bacilli	o__Lactobacillales	f__Streptococcaceae	g__Streptococcus	s__Streptococcus pasteurianus	GCF_900478025.1	162	162_1	F68D_bin.75.fa	F68D_bin.75.fa	100	0	152
F68N_bin.11	d__Bacteria	p__Firmicutes	c__Bacilli	o__Lactobacillales	f__Streptococcaceae	g__Streptococcus	s__Streptococcus salivarius	GCF_000785515.1	154	154_3	F68N_bin.11.fa	F68N_bin.11.fa	66.18	2.97	152

Table S3. Taxonomic classifiation of the 2,421 detected MAGs

	Domain	Phylum	Class	Order	Family	Genus	Species	RefGenome	primary_cluster	secondary_cluster	repr_genome	genome	CheckM_completeness	CheckM_contamination	
F68T_bin.17	d__Bacteria	p__Firmicutes	c__Bacilli	o__Lactobacillales	f__Streptococcaceae	g__Streptococcus	s__Streptococcus salivarius	GCF_000785515.1	154	154_1	F68T_bin.17.fa	F68T_bin.17.fa	50.34	3.06	152
F68M_bin.71	d__Bacteria	p__Firmicutes	c__Bacilli	o__Lactobacillales	f__Streptococcaceae	g__Streptococcus	s__Streptococcus sinensis	GCF_000767835.1	160	160_0	F68M_bin.71.fa	F68M_bin.71.fa	61.21	0.86	152
F68M_bin.48	d__Bacteria	p__Firmicutes	c__Bacilli	o__Lactobacillales	f__Streptococcaceae	g__Streptococcus	s__Streptococcus sp001587175	GCF_001587175.1	157	157_0	F68M_bin.48.fa	F68M_bin.48.fa	68.42	0.38	152
F68J_bin.32	d__Bacteria	p__Firmicutes	c__Bacilli	o__Lactobacillales	f__Streptococcaceae	g__Streptococcus	s__Streptococcus sp002300045	GCA_002300045.1	163	163_0	F68J_bin.32.fa	F68J_bin.32.fa	91.01	3.08	152
F68V_bin.50	d__Bacteria	p__Firmicutes	c__Bacilli	o__Lactobacillales	f__Streptococcaceae	g__Streptococcus	s__Streptococcus thermophilus	GCF_000014485.1	155	155_0	F68V_bin.50.fa	F68V_bin.50.fa	97.53	0.15	152
F6D7_bin.103	d__Bacteria	p__Firmicutes	c__Bacilli	o__RF39	f__CAG-1000	g__CAG-460	s__	N/A	243	243_0	F6D7_bin.103.fa	F6D7_bin.103.fa	87.08	1.12	153

Table S3. Taxonomic classifiation of the 2,421 detected MAGs

	Domain	Phylum	Class	Order	Family	Genus	Species	RefGenome	primary_cluster	secondary_cluster	repr_genome	genome	CheckM_completeness	CheckM_contamination	
F68F_bin.110	d__Bacteria	p__Firmicutes	c__Bacilli	o__RF39	f__CAG-1000	g__CAG-533	s__CAG-533 sp000434495	GCA_000434495.1	234	234_0	F68F_bin.110.fa	F68F_bin.110.fa	78.01	4.26	153
F6D7_bin.109	d__Bacteria	p__Firmicutes	c__Bacilli	o__RF39	f__CAG-302	g__	s__	N/A	172	172_0	F6D7_bin.109.fa	F6D7_bin.109.fa	56.67	3.37	154
F68U_bin.40	d__Bacteria	p__Firmicutes	c__Bacilli	o__RF39	f__CAG-302	g__CAG-302	s__	N/A	236	236_1	F68U_bin.40.fa	F68U_bin.40.fa	93.54	1.8	155
F68W_bin.9	d__Bacteria	p__Firmicutes	c__Bacilli	o__RF39	f__CAG-302	g__CAG-302	s__	N/A	236	236_1	F68U_bin.40.fa	F68W_bin.9.fa	91.29	3.92	156
F6DH_bin.68	d__Bacteria	p__Firmicutes	c__Bacilli	o__RF39	f__CAG-302	g__CAG-302	s__	N/A	236	236_1	F68U_bin.40.fa	F6DH_bin.68.fa	91.29	2.69	157
F6D9_bin.72	d__Bacteria	p__Firmicutes	c__Bacilli	o__RF39	f__CAG-302	g__CAG-302	s__	N/A	236	236_2	F6D9_bin.72.fa	F6D9_bin.72.fa	72.19	1.12	158
F68V_bin.63	d__Bacteria	p__Firmicutes	c__Bacilli	o__RF39	f__CAG-302	g__CAG-302	s__CAG-302 sp000431795	GCA_000431795.1	235	235_1	F6MY_bin.87.fa	F68V_bin.63.fa	85.67	0	158
F6D7_bin.9	d__Bacteria	p__Firmicutes	c__Bacilli	o__RF39	f__CAG-302	g__CAG-302	s__CAG-302 sp000431795	GCA_000431795.1	235	235_1	F6MY_bin.87.fa	F6D7_bin.9.fa	93.33	2.67	158
F6MX_bin.104	d__Bacteria	p__Firmicutes	c__Bacilli	o__RF39	f__CAG-302	g__CAG-302	s__CAG-302 sp000431795	GCA_000431795.1	235	235_1	F6MY_bin.87.fa	F6MX_bin.104.fa	87.92	1.12	158
F6MY_bin.87	d__Bacteria	p__Firmicutes	c__Bacilli	o__RF39	f__CAG-302	g__CAG-302	s__CAG-302 sp000431795	GCA_000431795.1	235	235_1	F6MY_bin.87.fa	F6MY_bin.87.fa	92.42	1.12	158

Table S3. Taxonomic classifiation of the 2,421 detected MAGs

	Domain	Phylum	Class	Order	Family	Genus	Species	RefGenome	primary_cluster	secondary_cluster	repr_genome	genome	CheckM_completeness	CheckM_contamination	
F68F_bin.82	d__Bacteria	p__Firmicutes	c__Bacilli	o__RF39	f__CAG-302	g__CAG-302	s__CAG-302 sp001916775	GCA_001916775.1	237	237_3	F68F_bin.82.fa	F68F_bin.82.fa	92.42	2.25	158
F68G_bin.33	d__Bacteria	p__Firmicutes	c__Bacilli	o__RF39	f__CAG-302	g__CAG-302	s__CAG-302 sp001916775	GCA_001916775.1	237	237_5	F68G_bin.33.fa	F68G_bin.33.fa	92	4	158
F6D7_bin.31	d__Bacteria	p__Firmicutes	c__Bacilli	o__RF39	f__CAG-302	g__CAG-302	s__CAG-302 sp001916775	GCA_001916775.1	237	237_4	F6D7_bin.31.fa	F6D7_bin.31.fa	92.42	3.37	158
F6D8_bin.7	d__Bacteria	p__Firmicutes	c__Bacilli	o__RF39	f__CAG-302	g__CAG-302	s__CAG-302 sp001916775	GCA_001916775.1	237	237_2	F6D8_bin.7.fa	F6D8_bin.7.fa	92.7	4.49	158
F6DG_bin.103	d__Bacteria	p__Firmicutes	c__Bacilli	o__RF39	f__CAG-302	g__CAG-302	s__CAG-302 sp001916775	GCA_001916775.1	237	237_1	F6MX_bin.24.fa	F6DG_bin.103.fa	88.2	3.37	158
F6MX_bin.24	d__Bacteria	p__Firmicutes	c__Bacilli	o__RF39	f__CAG-302	g__CAG-302	s__CAG-302 sp001916775	GCA_001916775.1	237	237_1	F6MX_bin.24.fa	F6MX_bin.24.fa	92.7	3.93	158
F68V_bin.101	d__Bacteria	p__Firmicutes	c__Bacilli	o__RF39	f__CAG-302	g__CAG-914	s__CAG-914 sp000437895	GCA_000437895.1	240	240_0	F68V_bin.101.fa	F68V_bin.101.fa	84	4	158
F6D7_bin.38	d__Bacteria	p__Firmicutes	c__Bacilli	o__RF39	f__CAG-433	g__CAG-433	s__	N/A	238	238_0	F6D7_bin.38.fa	F6D7_bin.38.fa	93.82	0.56	159

Table S3. Taxonomic classifiation of the 2,421 detected MAGs

	Domain	Phylum	Class	Order	Family	Genus	Species	RefGenome	primary_cluster	secondary_cluster	repr_genome	genome	CheckM_completeness	CheckM_contamination	
F68C_bin.88	d__Bacteria	p__Firmicutes	c__Bacilli	o__RF39	f__CAG-433	g__CAG-594	s__CAG-594 sp000434835	GCA_000434835.1	233	233_1	F68C_bin.88.fa	F68C_bin.88.fa	92.7	2.25	159
F6D8_bin.39	d__Bacteria	p__Firmicutes	c__Bacilli	o__RF39	f__CAG-433	g__CAG-594	s__CAG-594 sp000434835	GCA_000434835.1	233	233_2	F6D8_bin.39.fa	F6D8_bin.39.fa	79.4	1.12	159
F6D8_bin.77	d__Bacteria	p__Firmicutes	c__Bacilli	o__RF39	f__CAG-611	g__CAG-611	s__	N/A	239	239_2	F6D8_bin.77.fa	F6D8_bin.77.fa	54.1	3.33	160
F68V_bin.46	d__Bacteria	p__Firmicutes	c__Bacilli	o__RF39	f__CAG-611	g__CAG-611	s__CAG-611 sp000434175	GCA_000434175.1	239	239_1	F68V_bin.46.fa	F68V_bin.46.fa	84.99	2.25	160
F68W_bin.37	d__Bacteria	p__Firmicutes	c__Bacilli	o__RF39	f__CAG-822	g__UBA5026	s__	N/A	242	242_1	F68W_bin.37.fa	F68W_bin.37.fa	93.82	0	161
F6DH_bin.82	d__Bacteria	p__Firmicutes	c__Bacilli	o__RF39	f__CAG-822	g__UBA5026	s__	N/A	242	242_1	F68W_bin.37.fa	F6DH_bin.82.fa	93.26	1.12	162
F6MX_bin.100	d__Bacteria	p__Firmicutes	c__Bacilli	o__RF39	f__UBA5348	g__CAG-1193	s__	N/A	241	241_0	F6MX_bin.100.fa	F6MX_bin.100.fa	93.33	0	163
F68J_bin.16	d__Bacteria	p__Firmicutes	c__Bacilli	o__RFN20	f__CAG-288	g__UBA7642	s__	N/A	426	426_0	F68J_bin.16.fa	F68J_bin.16.fa	92.32	0	164
F68J_bin.81	d__Bacteria	p__Firmicutes	c__Bacilli	o__RFN20	f__CAG-449	g__CAG-449	s__	N/A	118	118_0	F68J_bin.81.fa	F68J_bin.81.fa	100	0	165
F68J_bin.25	d__Bacteria	p__Firmicutes	c__Bacilli	o__RFN20	f__CAG-826	g__UBA733	s__	N/A	9	9_1	F68J_bin.25.fa	F68J_bin.25.fa	97.75	1.12	166
F68U_bin.19	d__Bacteria	p__Firmicutes	c__Bacilli	o__RFN20	f__CAG-826	g__UBA733	s__	N/A	9	9_4	F68U_bin.19.fa	F68U_bin.19.fa	97.75	0	167
F68W_bin.78	d__Bacteria	p__Firmicutes	c__Bacilli	o__RFN20	f__CAG-826	g__UBA733	s__	N/A	9	9_3	F68W_bin.78.fa	F68W_bin.78.fa	97.45	0	168

Table S3. Taxonomic classifiation of the 2,421 detected MAGs

	Domain	Phylum	Class	Order	Family	Genus	Species	RefGenome	primary_cluster	secondary_cluster	repr_genome	genome	CheckM_completeness	CheckM_contamination	
F6DH_bin.88	d__Bacteria	p__Firmicutes	c__Bacilli	o__RFN20	f__CAG-826	g__UBA733	s__	N/A	9	9_3	F68W_bin.78.fa	F6DH_bin.88.fa	97.45	0	169
F6D9_bin.92	d__Bacteria	p__Firmicutes	c__Bacilli	o__RFN20	f__CAG-826	g__UBA733	s__	N/A	9	9_2	F6D9_bin.92.fa	F6D9_bin.92.fa	81.85	2.36	170
F68M_bin.49	d__Bacteria	p__Firmicutes	c__Clostridia	o__	f__	g__	s__	N/A	368	368_0	F68M_bin.49.fa	F68M_bin.49.fa	90.9	1.24	171
F6MX_bin.28	d__Bacteria	p__Firmicutes	c__Clostridia	o__	f__	g__	s__	N/A	665	665_0	F6MX_bin.28.fa	F6MX_bin.28.fa	54.15	0	172
F6D7_bin.36	d__Bacteria	p__Firmicutes	c__Clostridia	o__4C28d-15	f__	g__	s__	N/A	510	510_0	F6D7_bin.36.fa	F6D7_bin.36.fa	61.05	0	173
F68G_bin.55	d__Bacteria	p__Firmicutes	c__Clostridia	o__4C28d-15	f__CAG-314	g__CAG-1435	s__CAG-1435 sp003537755	GCA_003537755.1	231	231_1	F68G_bin.55.fa	F68G_bin.55.fa	92.34	0.03	173
F6MX_bin.1	d__Bacteria	p__Firmicutes	c__Clostridia	o__4C28d-15	f__CAG-314	g__CAG-1435	s__CAG-1435 sp003537755	GCA_003537755.1	231	231_2	F6MX_bin.1.fa	F6MX_bin.1.fa	92.34	0.03	173
F68D_bin.77	d__Bacteria	p__Firmicutes	c__Clostridia	o__4C28d-15	f__CAG-314	g__CAG-314	s__CAG-314 sp000437915	GCA_000437915.1	500	500_1	F68D_bin.77.fa	F68D_bin.77.fa	93.15	0.81	173
F6MX_bin.131	d__Bacteria	p__Firmicutes	c__Clostridia	o__4C28d-15	f__CAG-314	g__CAG-314	s__CAG-314 sp000437915	GCA_000437915.1	500	500_1	F68D_bin.77.fa	F6MX_bin.131.fa	93.15	2.42	173
F6D7_bin.40	d__Bacteria	p__Firmicutes	c__Clostridia	o__4C28d-15	f__CAG-552	g__	s__	N/A	23	23_0	F6D7_bin.40.fa	F6D7_bin.40.fa	91.59	0.04	174
F68F_bin.97	d__Bacteria	p__Firmicutes	c__Clostridia	o__4C28d-15	f__CAG-727	g__	s__	N/A	327	327_0	F68F_bin.97.fa	F68F_bin.97.fa	84.27	0.03	175

Table S3. Taxonomic classifiation of the 2,421 detected MAGs

	Domain	Phylum	Class	Order	Family	Genus	Species	RefGenome	primary_cluster	secondary_cluster	repr_genome	genome	CheckM_completeness	CheckM_contamination	
F68D_bin.29	d__Bacteria	p__Firmicutes	c__Clostridia	o__4C28d-15	f__CAG-727	g__	s__	N/A	501	501_1	F68M_bin.86.fa	F68D_bin.29.fa	54.63	2.63	176
F68M_bin.86	d__Bacteria	p__Firmicutes	c__Clostridia	o__4C28d-15	f__CAG-727	g__	s__	N/A	501	501_1	F68M_bin.86.fa	F68M_bin.86.fa	93.15	1.09	177
F6D7_bin.49	d__Bacteria	p__Firmicutes	c__Clostridia	o__4C28d-15	f__CAG-727	g__	s__	N/A	416	416_0	F6D7_bin.49.fa	F6D7_bin.49.fa	79.03	0.05	178
F6D7_bin.61	d__Bacteria	p__Firmicutes	c__Clostridia	o__4C28d-15	f__CAG-727	g__	s__	N/A	328	328_0	F6D7_bin.61.fa	F6D7_bin.61.fa	80.24	0.03	179
F6D7_bin.83	d__Bacteria	p__Firmicutes	c__Clostridia	o__4C28d-15	f__CAG-727	g__	s__	N/A	325	325_0	F6D7_bin.83.fa	F6D7_bin.83.fa	95.56	0.05	180
F6D8_bin.86	d__Bacteria	p__Firmicutes	c__Clostridia	o__4C28d-15	f__CAG-727	g__	s__	N/A	326	326_0	F6D8_bin.86.fa	F6D8_bin.86.fa	95.56	0.05	181
F6DE_bin.17	d__Bacteria	p__Firmicutes	c__Clostridia	o__4C28d-15	f__CAG-727	g__	s__	N/A	324	324_1	F6DE_bin.17.fa	F6DE_bin.17.fa	91.94	0.03	182
F6DG_bin.37	d__Bacteria	p__Firmicutes	c__Clostridia	o__4C28d-15	f__CAG-727	g__	s__	N/A	324	324_1	F6DE_bin.17.fa	F6DG_bin.37.fa	93.68	3.28	183
F6DH_bin.131	d__Bacteria	p__Firmicutes	c__Clostridia	o__4C28d-15	f__CAG-727	g__	s__	N/A	324	324_1	F6DE_bin.17.fa	F6DH_bin.131.fa	51.18	0.23	184
F68F_bin.27	d__Bacteria	p__Firmicutes	c__Clostridia	o__4C28d-15	f__CAG-727	g__QALS01	s__	N/A	542	542_1	F68F_bin.27.fa	F68F_bin.27.fa	86.24	2.22	185
F6D7_bin.19	d__Bacteria	p__Firmicutes	c__Clostridia	o__4C28d-15	f__CAG-727	g__QALS01	s__	N/A	542	542_1	F68F_bin.27.fa	F6D7_bin.19.fa	77.4	1.4	186
F6D7_bin.43	d__Bacteria	p__Firmicutes	c__Clostridia	o__4C28d-15	f__CAG-727	g__QALS01	s__QALS01 sp003150575	GCA_003150575.1	543	543_2	F6D7_bin.43.fa	F6D7_bin.43.fa	95.97	0	186
F6DG_bin.61	d__Bacteria	p__Firmicutes	c__Clostridia	o__4C28d-15	f__CAG-727	g__QALS01	s__QALS01 sp003150575	GCA_003150575.1	543	543_1	F6DH_bin.117.fa	F6DG_bin.61.fa	68.96	1.9	186

Table S3. Taxonomic classifiation of the 2,421 detected MAGs

	Domain	Phylum	Class	Order	Family	Genus	Species	RefGenome	primary_cluster	secondary_cluster	repr_genome	genome	CheckM_completeness	CheckM_contamination	
F6DH_bin.117	d__Bacteria	p__Firmicutes	c__Clostridia	o__4C28d-15	f__CAG-727	g__QALS01	s__QALS01 sp003150575	GCA_003150575.1	543	543_1	F6DH_bin.117.fa	F6DH_bin.117.fa	81.24	0	186
F68J_bin.42	d__Bacteria	p__Firmicutes	c__Clostridia	o__4C28d-15	f__CAG-727	g__UBA10281	s__	N/A	536	536_1	F68J_bin.42.fa	F68J_bin.42.fa	93.15	1.61	187
F6MX_bin.106	d__Bacteria	p__Firmicutes	c__Clostridia	o__4C28d-15	f__CAG-727	g__UBA10281	s__	N/A	536	536_2	F6MX_bin.106.fa	F6MX_bin.106.fa	91.38	3.63	188
F68D_bin.49	d__Bacteria	p__Firmicutes	c__Clostridia	o__4C28d-15	f__CAG-727	g__UBA10281	s__UBA10281 sp000437515	GCA_000437515.1	535	535_3	F68D_bin.49.fa	F68D_bin.49.fa	93.35	1.61	188
F68G_bin.47	d__Bacteria	p__Firmicutes	c__Clostridia	o__4C28d-15	f__CAG-727	g__UBA10281	s__UBA10281 sp000437515	GCA_000437515.1	535	535_1	F68G_bin.47.fa	F68G_bin.47.fa	93.35	0.81	188
F68J_bin.19	d__Bacteria	p__Firmicutes	c__Clostridia	o__4C28d-15	f__CAG-727	g__UBA10281	s__UBA10281 sp000437515	GCA_000437515.1	535	535_2	F68J_bin.19.fa	F68J_bin.19.fa	92.54	1.61	188
F68R_bin.69	d__Bacteria	p__Firmicutes	c__Clostridia	o__4C28d-15	f__CAG-727	g__UBA10281	s__UBA10281 sp000437515	GCA_000437515.1	535	535_4	F68R_bin.69.fa	F68R_bin.69.fa	93.35	0.81	188
F68F_bin.7	d__Bacteria	p__Firmicutes	c__Clostridia	o__4C28d-15	f__CAG-727	g__UBA7597	s__	N/A	534	534_4	F68F_bin.7.fa	F68F_bin.7.fa	94.35	0	189
F68P_bin.16	d__Bacteria	p__Firmicutes	c__Clostridia	o__4C28d-15	f__CAG-727	g__UBA7597	s__	N/A	534	534_1	F68P_bin.16.fa	F68P_bin.16.fa	69.72	0.67	190
F68R_bin.60	d__Bacteria	p__Firmicutes	c__Clostridia	o__4C28d-15	f__CAG-727	g__UBA7597	s__	N/A	534	534_3	F68R_bin.60.fa	F68R_bin.60.fa	86.25	0.81	191

Table S3. Taxonomic classifiation of the 2,421 detected MAGs

	Domain	Phylum	Class	Order	Family	Genus	Species	RefGenome	primary_cluster	secondary_cluster	repr_genome	genome	CheckM_completeness	CheckM_contamination	
F6MY_bin.136	d__Bacteria	p__Firmicutes	c__Clostridia	o__4C28d-15	f__CAG-727	g__UBA7597	s__	N/A	534	534_2	F6MY_bin.136.fa	F6MY_bin.136.fa	93.55	0	192
F68C_bin.22	d__Bacteria	p__Firmicutes	c__Clostridia	o__4C28d-15	f__CAG-727	g__UBA7597	s__UBA7597 sp003448195	GCA_003448195.1	533	533_1	F6DA_bin.9.fa	F68C_bin.22.fa	95.97	0	192
F68H_bin.10	d__Bacteria	p__Firmicutes	c__Clostridia	o__4C28d-15	f__CAG-727	g__UBA7597	s__UBA7597 sp003448195	GCA_003448195.1	533	533_1	F6DA_bin.9.fa	F68H_bin.10.fa	87.82	1.41	192
F68J_bin.35	d__Bacteria	p__Firmicutes	c__Clostridia	o__4C28d-15	f__CAG-727	g__UBA7597	s__UBA7597 sp003448195	GCA_003448195.1	533	533_1	F6DA_bin.9.fa	F68J_bin.35.fa	89.14	0	192
F68N_bin.72	d__Bacteria	p__Firmicutes	c__Clostridia	o__4C28d-15	f__CAG-727	g__UBA7597	s__UBA7597 sp003448195	GCA_003448195.1	533	533_1	F6DA_bin.9.fa	F68N_bin.72.fa	95.82	0.03	192
F6DA_bin.9	d__Bacteria	p__Firmicutes	c__Clostridia	o__4C28d-15	f__CAG-727	g__UBA7597	s__UBA7597 sp003448195	GCA_003448195.1	533	533_1	F6DA_bin.9.fa	F6DA_bin.9.fa	95.97	0	192
F68H_bin.3	d__Bacteria	p__Firmicutes	c__Clostridia	o__4C28d-15	f__CAG-917	g__CAG-349	s__CAG-349 sp003539515	GCA_003539515.1	333	333_3	F68H_bin.3.fa	F68H_bin.3.fa	95.89	1.21	192
F68J_bin.43	d__Bacteria	p__Firmicutes	c__Clostridia	o__4C28d-15	f__CAG-917	g__CAG-349	s__CAG-349 sp003539515	GCA_003539515.1	333	333_5	F68J_bin.43.fa	F68J_bin.43.fa	92.4	0.7	192

Table S3. Taxonomic classifiation of the 2,421 detected MAGs

	Domain	Phylum	Class	Order	Family	Genus	Species	RefGenome	primary_cluster	secondary_cluster	repr_genome	genome	CheckM_completeness	CheckM_contamination	
F68R_bin.66	d__Bacteria	p__Firmicutes	c__Clostridia	o__4C28d-15	f__CAG-917	g__CAG-349	s__CAG-349 sp003539515	GCA_003539515.1	333	333_1	F68R_bin.66.fa	F68R_bin.66.fa	91	0.7	192
F6D8_bin.50	d__Bacteria	p__Firmicutes	c__Clostridia	o__4C28d-15	f__CAG-917	g__CAG-349	s__CAG-349 sp003539515	GCA_003539515.1	333	333_2	F6D8_bin.50.fa	F6D8_bin.50.fa	92.4	0.7	192
F6D9_bin.61	d__Bacteria	p__Firmicutes	c__Clostridia	o__4C28d-15	f__CAG-917	g__CAG-349	s__CAG-349 sp003539515	GCA_003539515.1	333	333_4	F6D9_bin.61.fa	F6D9_bin.61.fa	56.9	1.72	192
F6MX_bin.111	d__Bacteria	p__Firmicutes	c__Clostridia	o__4C28d-15	f__CAG-917	g__CAG-349	s__CAG-349 sp003539515	GCA_003539515.1	333	333_6	F6MX_bin.111.fa	F6MX_bin.111.fa	91.94	0.7	192
F68C_bin.70	d__Bacteria	p__Firmicutes	c__Clostridia	o__Christensenellales	f__CAG-138	g__	s__	N/A	440	440_7	F68C_bin.70.fa	F68C_bin.70.fa	96.63	2.08	193
F68D_bin.55	d__Bacteria	p__Firmicutes	c__Clostridia	o__Christensenellales	f__CAG-138	g__	s__	N/A	440	440_2	F68D_bin.55.fa	F68D_bin.55.fa	77.66	2.58	194
F68F_bin.92	d__Bacteria	p__Firmicutes	c__Clostridia	o__Christensenellales	f__CAG-138	g__	s__	N/A	440	440_4	F68F_bin.92.fa	F68F_bin.92.fa	88.81	4.44	195
F68G_bin.86	d__Bacteria	p__Firmicutes	c__Clostridia	o__Christensenellales	f__CAG-138	g__	s__	N/A	440	440_6	F68G_bin.86.fa	F68G_bin.86.fa	74.93	2.4	196
F68V_bin.96	d__Bacteria	p__Firmicutes	c__Clostridia	o__Christensenellales	f__CAG-138	g__	s__	N/A	440	440_9	F68V_bin.96.fa	F68V_bin.96.fa	71.77	2.69	197

Table S3. Taxonomic classifiation of the 2,421 detected MAGs

	Domain	Phylum	Class	Order	Family	Genus	Species	RefGenome	primary_cluster	secondary_cluster	repr_genome	genome	CheckM_completeness	CheckM_contamination	
F68W_bin.56	d__Bacteria	p__Firmicutes	c__Clostridia	o__Christensenellales	f__CAG-138	g__	s__	N/A	440	440_1	F68W_bin.56.fa	F68W_bin.56.fa	75.48	1.55	198
F6DH_bin.28	d__Bacteria	p__Firmicutes	c__Clostridia	o__Christensenellales	f__CAG-138	g__	s__	N/A	440	440_1	F68W_bin.56.fa	F6DH_bin.28.fa	78.9	2.49	199
F6D8_bin.25	d__Bacteria	p__Firmicutes	c__Clostridia	o__Christensenellales	f__CAG-138	g__	s__	N/A	440	440_5	F6D8_bin.25.fa	F6D8_bin.25.fa	93.23	1.64	200
F6MX_bin.91	d__Bacteria	p__Firmicutes	c__Clostridia	o__Christensenellales	f__CAG-138	g__	s__	N/A	440	440_3	F6MX_bin.91.fa	F6MX_bin.91.fa	96.77	1.88	201
F6MY_bin.36	d__Bacteria	p__Firmicutes	c__Clostridia	o__Christensenellales	f__CAG-138	g__	s__	N/A	440	440_8	F6MY_bin.36.fa	F6MY_bin.36.fa	94.41	0.93	202
F68V_bin.17	d__Bacteria	p__Firmicutes	c__Clostridia	o__Christensenellales	f__CAG-138	g__CAG-1024	s__	N/A	567	567_0	F68V_bin.17.fa	F68V_bin.17.fa	83.76	0	203
F68V_bin.5	d__Bacteria	p__Firmicutes	c__Clostridia	o__Christensenellales	f__CAG-138	g__CAG-1024	s__	N/A	568	568_0	F68V_bin.5.fa	F68V_bin.5.fa	78.06	0	204
F68V_bin.83	d__Bacteria	p__Firmicutes	c__Clostridia	o__Christensenellales	f__CAG-138	g__PeH17	s__	N/A	480	480_0	F68V_bin.83.fa	F68V_bin.83.fa	95.51	0.81	205
F68D_bin.41	d__Bacteria	p__Firmicutes	c__Clostridia	o__Christensenellales	f__CAG-138	g__PeH17	s__PeH17 sp000435055	GCA_000435055.1	479	479_4	F68D_bin.41.fa	F68D_bin.41.fa	92.6	1.34	205

Table S3. Taxonomic classifiation of the 2,421 detected MAGs

	Domain	Phylum	Class	Order	Family	Genus	Species	RefGenome	primary_cluster	secondary_cluster	repr_genome	genome	CheckM_completeness	CheckM_contamination	
F68F_bin.1	d__Bacteria	p__Firmicutes	c__Clostridia	o__Christensenellales	f__CAG-138	g__PeH17	s__PeH17 sp000435055	GCA_000435055.1	479	479_12	F68F_bin.1.fa	F68F_bin.1.fa	92.6	1.34	205
F68H_bin.29	d__Bacteria	p__Firmicutes	c__Clostridia	o__Christensenellales	f__CAG-138	g__PeH17	s__PeH17 sp000435055	GCA_000435055.1	479	479_7	F68H_bin.29.fa	F68H_bin.29.fa	92.6	1.34	205
F68M_bin.65	d__Bacteria	p__Firmicutes	c__Clostridia	o__Christensenellales	f__CAG-138	g__PeH17	s__PeH17 sp000435055	GCA_000435055.1	479	479_2	F68M_bin.65.fa	F68M_bin.65.fa	92.6	1.34	205
F6DB_bin.99	d__Bacteria	p__Firmicutes	c__Clostridia	o__Christensenellales	f__CAG-138	g__PeH17	s__PeH17 sp000435055	GCA_000435055.1	479	479_2	F68M_bin.65.fa	F6DB_bin.99.fa	92.6	1.34	205
F68N_bin.64	d__Bacteria	p__Firmicutes	c__Clostridia	o__Christensenellales	f__CAG-138	g__PeH17	s__PeH17 sp000435055	GCA_000435055.1	479	479_5	F68N_bin.64.fa	F68N_bin.64.fa	92.6	1.34	205
F68R_bin.19	d__Bacteria	p__Firmicutes	c__Clostridia	o__Christensenellales	f__CAG-138	g__PeH17	s__PeH17 sp000435055	GCA_000435055.1	479	479_3	F68R_bin.19.fa	F68R_bin.19.fa	92.6	1.34	205
F68V_bin.60	d__Bacteria	p__Firmicutes	c__Clostridia	o__Christensenellales	f__CAG-138	g__PeH17	s__PeH17 sp000435055	GCA_000435055.1	479	479_13	F68V_bin.60.fa	F68V_bin.60.fa	91.25	2.01	205

Table S3. Taxonomic classifiation of the 2,421 detected MAGs

	Domain	Phylum	Class	Order	Family	Genus	Species	RefGenome	primary_cluster	secondary_cluster	repr_genome	genome	CheckM_completeness	CheckM_contamination	
F68C_bin.66	d__Bacteria	p__Firmicutes	c__Clostridia	o__Christensenellales	f__CAG-138	g__PeH17	s__PeH17 sp000435055	GCA_000435055.1	479	479_6	F6D7_bin.77.fa	F68C_bin.66.fa	92.6	1.34	205
F6D7_bin.77	d__Bacteria	p__Firmicutes	c__Clostridia	o__Christensenellales	f__CAG-138	g__PeH17	s__PeH17 sp000435055	GCA_000435055.1	479	479_6	F6D7_bin.77.fa	F6D7_bin.77.fa	92.6	1.34	205
F6D8_bin.6	d__Bacteria	p__Firmicutes	c__Clostridia	o__Christensenellales	f__CAG-138	g__PeH17	s__PeH17 sp000435055	GCA_000435055.1	479	479_11	F6D8_bin.6.fa	F6D8_bin.6.fa	92.6	1.34	205
F68G_bin.49	d__Bacteria	p__Firmicutes	c__Clostridia	o__Christensenellales	f__CAG-138	g__PeH17	s__PeH17 sp000435055	GCA_000435055.1	479	479_1	F6D9_bin.1.fa	F68G_bin.49.fa	92.6	1.34	205
F6D9_bin.1	d__Bacteria	p__Firmicutes	c__Clostridia	o__Christensenellales	f__CAG-138	g__PeH17	s__PeH17 sp000435055	GCA_000435055.1	479	479_1	F6D9_bin.1.fa	F6D9_bin.1.fa	92.6	1.34	205
F6DA_bin.37	d__Bacteria	p__Firmicutes	c__Clostridia	o__Christensenellales	f__CAG-138	g__PeH17	s__PeH17 sp000435055	GCA_000435055.1	479	479_10	F6DA_bin.37.fa	F6DA_bin.37.fa	91.93	1.34	205
F6MX_bin.71	d__Bacteria	p__Firmicutes	c__Clostridia	o__Christensenellales	f__CAG-138	g__PeH17	s__PeH17 sp000435055	GCA_000435055.1	479	479_9	F6MX_bin.71.fa	F6MX_bin.71.fa	92.6	1.34	205

Table S3. Taxonomic classifiation of the 2,421 detected MAGs

	Domain	Phylum	Class	Order	Family	Genus	Species	RefGenome	primary_cluster	secondary_cluster	repr_genome	genome	CheckM_completeness	CheckM_contamination	
F6MY_bin.110	d__Bacteria	p__Firmicutes	c__Clostridia	o__Christensenellales	f__CAG-138	g__PeH17	s__PeH17 sp000435055	GCA_000435055.1	479	479_8	F6MY_bin.110.fa	F6MY_bin.110.fa	92.6	1.34	205
F6D9_bin.23	d__Bacteria	p__Firmicutes	c__Clostridia	o__Christensenellales	f__CAG-138	g__Phil1	s__	N/A	230	230_0	F6D9_bin.23.fa	F6D9_bin.23.fa	97.58	0	206
F68F_bin.38	d__Bacteria	p__Firmicutes	c__Clostridia	o__Christensenellales	f__CAG-138	g__Phil1	s__Phil1 sp001940855	GCA_001940855.1	229	229_8	F68F_bin.38.fa	F68F_bin.38.fa	99.19	0	206
F68G_bin.56	d__Bacteria	p__Firmicutes	c__Clostridia	o__Christensenellales	f__CAG-138	g__Phil1	s__Phil1 sp001940855	GCA_001940855.1	229	229_9	F68G_bin.56.fa	F68G_bin.56.fa	99.19	0.81	206
F68H_bin.7	d__Bacteria	p__Firmicutes	c__Clostridia	o__Christensenellales	f__CAG-138	g__Phil1	s__Phil1 sp001940855	GCA_001940855.1	229	229_10	F68H_bin.7.fa	F68H_bin.7.fa	99.19	0	206
F68M_bin.32	d__Bacteria	p__Firmicutes	c__Clostridia	o__Christensenellales	f__CAG-138	g__Phil1	s__Phil1 sp001940855	GCA_001940855.1	229	229_4	F68M_bin.32.fa	F68M_bin.32.fa	99.19	0.81	206
F68R_bin.86	d__Bacteria	p__Firmicutes	c__Clostridia	o__Christensenellales	f__CAG-138	g__Phil1	s__Phil1 sp001940855	GCA_001940855.1	229	229_6	F68R_bin.86.fa	F68R_bin.86.fa	98.39	0.81	206
F6D7_bin.33	d__Bacteria	p__Firmicutes	c__Clostridia	o__Christensenellales	f__CAG-138	g__Phil1	s__Phil1 sp001940855	GCA_001940855.1	229	229_5	F6D7_bin.33.fa	F6D7_bin.33.fa	97.04	1.21	206

Table S3. Taxonomic classifiation of the 2,421 detected MAGs

	Domain	Phylum	Class	Order	Family	Genus	Species	RefGenome	primary_cluster	secondary_cluster	repr_genome	genome	CheckM_completeness	CheckM_contamination	
F68D_bin.92	d__Bacteria	p__Firmicutes	c__Clostridia	o__Christensenellales	f__CAG-138	g__Phil1	s__Phil1 sp001940855	GCA_001940855.1	229	229_3	F6D8_bin.63.fa	F68D_bin.92.fa	97.58	0.81	206
F68N_bin.3	d__Bacteria	p__Firmicutes	c__Clostridia	o__Christensenellales	f__CAG-138	g__Phil1	s__Phil1 sp001940855	GCA_001940855.1	229	229_3	F6D8_bin.63.fa	F68N_bin.3.fa	99.19	1.61	206
F6D8_bin.63	d__Bacteria	p__Firmicutes	c__Clostridia	o__Christensenellales	f__CAG-138	g__Phil1	s__Phil1 sp001940855	GCA_001940855.1	229	229_3	F6D8_bin.63.fa	F6D8_bin.63.fa	99.19	0.81	206
F6DB_bin.24	d__Bacteria	p__Firmicutes	c__Clostridia	o__Christensenellales	f__CAG-138	g__Phil1	s__Phil1 sp001940855	GCA_001940855.1	229	229_1	F6DB_bin.24.fa	F6DB_bin.24.fa	98.39	0	206
F6MX_bin.62	d__Bacteria	p__Firmicutes	c__Clostridia	o__Christensenellales	f__CAG-138	g__Phil1	s__Phil1 sp001940855	GCA_001940855.1	229	229_2	F6MX_bin.62.fa	F6MX_bin.62.fa	86.02	0	206
F6MY_bin.47	d__Bacteria	p__Firmicutes	c__Clostridia	o__Christensenellales	f__CAG-138	g__Phil1	s__Phil1 sp001940855	GCA_001940855.1	229	229_7	F6MY_bin.47.fa	F6MY_bin.47.fa	98.39	0.81	206
F68D_bin.78	d__Bacteria	p__Firmicutes	c__Clostridia	o__Christensenellales	f__CAG-138	g__UBA1685	s__UBA1685 sp002320595	GCA_002320595.1	37	37_1	F68D_bin.78.fa	F68D_bin.78.fa	76.91	2.32	206

Table S3. Taxonomic classifiation of the 2,421 detected MAGs

	Domain	Phylum	Class	Order	Family	Genus	Species	RefGenome	primary_cluster	secondary_cluster	repr_genome	genome	CheckM_completeness	CheckM_contamination	
F6MX_bin.32	d__Bacteria	p__Firmicutes	c__Clostridia	o__Christensenellales	f__CAG-138	g__UBA1685	s__UBA1685 sp002320595	GCA_002320595.1	37	37_2	F6MX_bin.32.fa	F6MX_bin.32.fa	98.6	1.4	206
F68V_bin.11	d__Bacteria	p__Firmicutes	c__Clostridia	o__Christensenellales	f__CAG-138	g__UBA5394	s__UBA5394 sp003150565	GCA_003150565.1	184	184_0	F68V_bin.11.fa	F68V_bin.11.fa	93.74	1.08	206
F6MX_bin.6	d__Bacteria	p__Firmicutes	c__Clostridia	o__Christensenellales	f__CAG-74	g__	s__	N/A	5	5_0	F6MX_bin.6.fa	F6MX_bin.6.fa	62.09	2.9	207
F68G_bin.9	d__Bacteria	p__Firmicutes	c__Clostridia	o__Christensenellales	f__CAG-74	g__	s__	N/A	85	85_0	F68G_bin.9.fa	F68G_bin.9.fa	83.43	2.33	208
F68D_bin.112	d__Bacteria	p__Firmicutes	c__Clostridia	o__Christensenellales	f__CAG-74	g__	s__	N/A	284	284_1	F68D_bin.112.fa	F68D_bin.112.fa	67.05	2.25	209
F6MY_bin.130	d__Bacteria	p__Firmicutes	c__Clostridia	o__Christensenellales	f__CAG-74	g__	s__	N/A	284	284_2	F6MY_bin.130.fa	F6MY_bin.130.fa	96.51	0.22	210
F68G_bin.53	d__Bacteria	p__Firmicutes	c__Clostridia	o__Christensenellales	f__CAG-74	g__	s__	N/A	433	433_0	F68G_bin.53.fa	F68G_bin.53.fa	96.57	3.49	211
F68V_bin.67	d__Bacteria	p__Firmicutes	c__Clostridia	o__Christensenellales	f__CAG-74	g__	s__	N/A	552	552_1	F68V_bin.67.fa	F68V_bin.67.fa	52.72	3.51	212
F6MX_bin.134	d__Bacteria	p__Firmicutes	c__Clostridia	o__Christensenellales	f__CAG-74	g__	s__	N/A	552	552_2	F6MX_bin.134.fa	F6MX_bin.134.fa	84.84	4.37	213

Table S3. Taxonomic classifiation of the 2,421 detected MAGs

	Domain	Phylum	Class	Order	Family	Genus	Species	RefGenome	primary_cluster	secondary_cluster	repr_genome	genome	CheckM_completeness	CheckM_contamination	
F68M_bin.12	d__Bacteria	p__Firmicutes	c__Clostridia	o__Christensenellales	f__CAG-74	g__	s__	N/A	553	553_0	F68M_bin.12.fa	F68M_bin.12.fa	99.19	1.08	214
F68V_bin.70	d__Bacteria	p__Firmicutes	c__Clostridia	o__Christensenellales	f__CAG-74	g__	s__	N/A	555	555_0	F68V_bin.70.fa	F68V_bin.70.fa	53.33	1.13	215
F68F_bin.115	d__Bacteria	p__Firmicutes	c__Clostridia	o__Christensenellales	f__CAG-74	g__	s__	N/A	556	556_1	F68F_bin.115.fa	F68F_bin.115.fa	57.18	2.63	216
F6DG_bin.98	d__Bacteria	p__Firmicutes	c__Clostridia	o__Christensenellales	f__CAG-74	g__	s__	N/A	556	556_2	F6DG_bin.98.fa	F6DG_bin.98.fa	89.92	1.61	217
F68K_bin.26	d__Bacteria	p__Firmicutes	c__Clostridia	o__Christensenellales	f__CAG-74	g__	s__	N/A	557	557_1	F68K_bin.26.fa	F68K_bin.26.fa	91.02	1.63	218
F68U_bin.89	d__Bacteria	p__Firmicutes	c__Clostridia	o__Christensenellales	f__CAG-74	g__	s__	N/A	557	557_2	F68U_bin.89.fa	F68U_bin.89.fa	80.23	0.54	219
F68D_bin.97	d__Bacteria	p__Firmicutes	c__Clostridia	o__Christensenellales	f__CAG-74	g__	s__	N/A	558	558_2	F68D_bin.97.fa	F68D_bin.97.fa	94.36	2.33	220
F68F_bin.79	d__Bacteria	p__Firmicutes	c__Clostridia	o__Christensenellales	f__CAG-74	g__	s__	N/A	558	558_8	F68F_bin.79.fa	F68F_bin.79.fa	59.94	4.17	221
F68R_bin.21	d__Bacteria	p__Firmicutes	c__Clostridia	o__Christensenellales	f__CAG-74	g__	s__	N/A	558	558_7	F68R_bin.21.fa	F68R_bin.21.fa	77.93	0.27	222
F6D7_bin.60	d__Bacteria	p__Firmicutes	c__Clostridia	o__Christensenellales	f__CAG-74	g__	s__	N/A	558	558_3	F6D7_bin.60.fa	F6D7_bin.60.fa	75	1.25	223

Table S3. Taxonomic classifiation of the 2,421 detected MAGs

	Domain	Phylum	Class	Order	Family	Genus	Species	RefGenome	primary_cluster	secondary_cluster	repr_genome	genome	CheckM_completeness	CheckM_contamination	
F6D8_bin.21	d__Bacteria	p__Firmicutes	c__Clostridia	o__Christensenellales	f__CAG-74	g__	s__	N/A	558	558_4	F6D8_bin.21.fa	F6D8_bin.21.fa	96.5	1.3	224
F6DB_bin.29	d__Bacteria	p__Firmicutes	c__Clostridia	o__Christensenellales	f__CAG-74	g__	s__	N/A	558	558_6	F6DB_bin.29.fa	F6DB_bin.29.fa	95.97	0.94	225
F68W_bin.84	d__Bacteria	p__Firmicutes	c__Clostridia	o__Christensenellales	f__CAG-74	g__	s__	N/A	558	558_1	F6DH_bin.78.fa	F68W_bin.84.fa	58.18	0	226
F6DH_bin.78	d__Bacteria	p__Firmicutes	c__Clostridia	o__Christensenellales	f__CAG-74	g__	s__	N/A	558	558_1	F6DH_bin.78.fa	F6DH_bin.78.fa	96.5	0.23	227
F6MY_bin.90	d__Bacteria	p__Firmicutes	c__Clostridia	o__Christensenellales	f__CAG-74	g__	s__	N/A	558	558_5	F6MY_bin.90.fa	F6MY_bin.90.fa	96.5	1.3	228
F68D_bin.72	d__Bacteria	p__Firmicutes	c__Clostridia	o__Christensenellales	f__CAG-74	g__	s__	N/A	663	663_4	F68D_bin.72.fa	F68D_bin.72.fa	90.73	1.1	229
F68G_bin.41	d__Bacteria	p__Firmicutes	c__Clostridia	o__Christensenellales	f__CAG-74	g__	s__	N/A	663	663_5	F68G_bin.41.fa	F68G_bin.41.fa	92.74	1.88	230
F68W_bin.65	d__Bacteria	p__Firmicutes	c__Clostridia	o__Christensenellales	f__CAG-74	g__	s__	N/A	663	663_1	F68W_bin.65.fa	F68W_bin.65.fa	55.6	3.61	231
F6DH_bin.24	d__Bacteria	p__Firmicutes	c__Clostridia	o__Christensenellales	f__CAG-74	g__	s__	N/A	663	663_1	F68W_bin.65.fa	F6DH_bin.24.fa	51.9	4.75	232
F6DB_bin.81	d__Bacteria	p__Firmicutes	c__Clostridia	o__Christensenellales	f__CAG-74	g__	s__	N/A	663	663_2	F6DB_bin.81.fa	F6DB_bin.81.fa	94.76	2.69	233

Table S3. Taxonomic classifiation of the 2,421 detected MAGs

	Domain	Phylum	Class	Order	Family	Genus	Species	RefGenome	primary_cluster	secondary_cluster	repr_genome	genome	CheckM_completeness	CheckM_contamination	
F6MX_bin.99	d__Bacteria	p__Firmicutes	c__Clostridia	o__Christensenellales	f__CAG-74	g__	s__	N/A	663	663_3	F6MX_bin.99.fa	F6MX_bin.99.fa	90.3	3.23	234
F68C_bin.32	d__Bacteria	p__Firmicutes	c__Clostridia	o__Christensenellales	f__CAG-74	g__UBA11524	s__UBA11524 sp000437595	GCA_000437595.1	554	554_9	F68C_bin.32.fa	F68C_bin.32.fa	99.3	1.17	234
F68T_bin.5	d__Bacteria	p__Firmicutes	c__Clostridia	o__Christensenellales	f__CAG-74	g__UBA11524	s__UBA11524 sp000437595	GCA_000437595.1	554	554_7	F68T_bin.5.fa	F68T_bin.5.fa	99.3	1.19	234
F68V_bin.59	d__Bacteria	p__Firmicutes	c__Clostridia	o__Christensenellales	f__CAG-74	g__UBA11524	s__UBA11524 sp000437595	GCA_000437595.1	554	554_1	F68V_bin.59.fa	F68V_bin.59.fa	98.6	1.52	234
F6D8_bin.93	d__Bacteria	p__Firmicutes	c__Clostridia	o__Christensenellales	f__CAG-74	g__UBA11524	s__UBA11524 sp000437595	GCA_000437595.1	554	554_3	F6D8_bin.93.fa	F6D8_bin.93.fa	97.9	1.17	234
F6DB_bin.36	d__Bacteria	p__Firmicutes	c__Clostridia	o__Christensenellales	f__CAG-74	g__UBA11524	s__UBA11524 sp000437595	GCA_000437595.1	554	554_8	F6DB_bin.36.fa	F6DB_bin.36.fa	96.5	1.52	234
F6DE_bin.38	d__Bacteria	p__Firmicutes	c__Clostridia	o__Christensenellales	f__CAG-74	g__UBA11524	s__UBA11524 sp000437595	GCA_000437595.1	554	554_2	F6DE_bin.38.fa	F6DE_bin.38.fa	98.6	1.17	234
F6DG_bin.14	d__Bacteria	p__Firmicutes	c__Clostridia	o__Christensenellales	f__CAG-74	g__UBA11524	s__UBA11524 sp000437595	GCA_000437595.1	554	554_6	F6DG_bin.14.fa	F6DG_bin.14.fa	97.2	0.93	234

Table S3. Taxonomic classifiation of the 2,421 detected MAGs

	Domain	Phylum	Class	Order	Family	Genus	Species	RefGenome	primary_cluster	secondary_cluster	repr_genome	genome	CheckM_completeness	CheckM_contamination	
F6MX_bin.5	d__Bacteria	p__Firmicutes	c__Clostridia	o__Christensenellales	f__CAG-74	g__UBA11524	s__UBA11524 sp000437595	GCA_000437595.1	554	554_4	F6MX_bin.5.fa	F6MX_bin.5.fa	89.35	1.17	234
F6MY_bin.56	d__Bacteria	p__Firmicutes	c__Clostridia	o__Christensenellales	f__CAG-74	g__UBA11524	s__UBA11524 sp000437595	GCA_000437595.1	554	554_5	F6MY_bin.56.fa	F6MY_bin.56.fa	97.9	1.17	234
F68T_bin.104	d__Bacteria	p__Firmicutes	c__Clostridia	o__Christensenellales	f__Christensenellaceae	g__	s__	N/A	398	398_0	F68T_bin.104.fa	F68T_bin.104.fa	66.45	1.75	235
F6DE_bin.9	d__Bacteria	p__Firmicutes	c__Clostridia	o__Christensenellales	f__Christensenellaceae	g__	s__	N/A	410	410_0	F6DE_bin.9.fa	F6DE_bin.9.fa	97.9	0.7	236
F68K_bin.112	d__Bacteria	p__Firmicutes	c__Clostridia	o__Christensenellales	f__Christensenellaceae	g__QANA01	s__	N/A	397	397_0	F68K_bin.112.fa	F68K_bin.112.fa	64.19	2.45	237
F6MX_bin.45	d__Bacteria	p__Firmicutes	c__Clostridia	o__Christensenellales	f__QALW01	g__QALW01	s__	N/A	399	399_0	F6MX_bin.45.fa	F6MX_bin.45.fa	88.98	0.1	238
F68G_bin.58	d__Bacteria	p__Firmicutes	c__Clostridia	o__Christensenellales	f__UBA1750	g__	s__	N/A	559	559_0	F68G_bin.58.fa	F68G_bin.58.fa	50.16	0	239
F6MX_bin.101	d__Bacteria	p__Firmicutes	c__Clostridia	o__Christensenellales	f__UBA1750	g__	s__	N/A	560	560_0	F6MX_bin.101.fa	F6MX_bin.101.fa	97.58	1.69	240

Table S3. Taxonomic classifiation of the 2,421 detected MAGs

	Domain	Phylum	Class	Order	Family	Genus	Species	RefGenome	primary_cluster	secondary_cluster	repr_genome	genome	CheckM_completeness	CheckM_contamination	
F68G_bin.65	d__Bacteria	p__Firmicutes	c__Clostridia	o__Clostridiales	f__Clostridiaceae	g__Clostridium	s__	N/A	192	192_0	F68G_bin.65.fa	F68G_bin.65.fa	92.7	1.97	241
F6D8_bin.40	d__Bacteria	p__Firmicutes	c__Clostridia	o__Clostridiales	f__Clostridiaceae	g__Clostridium	s__	N/A	191	191_1	F6DB_bin.97.fa	F6D8_bin.40.fa	92.54	0.16	242
F6DB_bin.97	d__Bacteria	p__Firmicutes	c__Clostridia	o__Clostridiales	f__Clostridiaceae	g__Clostridium	s__	N/A	191	191_1	F6DB_bin.97.fa	F6DB_bin.97.fa	97.56	0.16	243
F6DG_bin.24	d__Bacteria	p__Firmicutes	c__Clostridia	o__Clostridiales	f__Clostridiaceae	g__Clostridium	s__	N/A	193	193_0	F6DG_bin.24.fa	F6DG_bin.24.fa	91.97	0	244
F6MY_bin.126	d__Bacteria	p__Firmicutes	c__Clostridia	o__Clostridiales	f__Clostridiaceae	g__Clostridium	s__Clostridium paraputrificum	GCF_900447045.1	194	194_0	F6MY_bin.126.fa	F6MY_bin.126.fa	92.22	2.76	244
F68D_bin.110	d__Bacteria	p__Firmicutes	c__Clostridia	o__Clostridiales	f__Clostridiaceae	g__Clostridium_P	s__Clostridium_P perfringens	GCF_000013285.1	203	203_0	F68D_bin.110.fa	F68D_bin.110.fa	62.55	4.22	244
F68V_bin.123	d__Bacteria	p__Firmicutes	c__Clostridia	o__Eubacteriales	f__Eubacteriaceae	g__Eubacterium	s__Eubacterium limosum_A	GCF_001481725.1	169	169_1	F68V_bin.123.fa	F68V_bin.123.fa	50.7	2.24	244
F6DE_bin.92	d__Bacteria	p__Firmicutes	c__Clostridia	o__Eubacteriales	f__Eubacteriaceae	g__Eubacterium	s__Eubacterium limosum_A	GCF_001481725.1	169	169_2	F6DE_bin.92.fa	F6DE_bin.92.fa	70.7	2.63	244

Table S3. Taxonomic classifiation of the 2,421 detected MAGs

	Domain	Phylum	Class	Order	Family	Genus	Species	RefGenome	primary_cluster	secondary_cluster	repr_genome	genome	CheckM_completeness	CheckM_contamination	
F6DB_bin.117	d__Bacteria	p__Firmicutes	c__Clostridia	o__Lachnospirales	f__	g__	s__	N/A	475	475_2	F6DB_bin.117.fa	F6DB_bin.117.fa	98.66	0.89	245
F68E_bin.35	d__Bacteria	p__Firmicutes	c__Clostridia	o__Lachnospirales	f__Anaerotignaceae	g__	s__	N/A	257	257_0	F68E_bin.35.fa	F68E_bin.35.fa	84.54	0	246
F68K_bin.18	d__Bacteria	p__Firmicutes	c__Clostridia	o__Lachnospirales	f__Anaerotignaceae	g__	s__	N/A	258	258_0	F68K_bin.18.fa	F68K_bin.18.fa	93.16	2.24	247
F6D7_bin.107	d__Bacteria	p__Firmicutes	c__Clostridia	o__Lachnospirales	f__Anaerotignaceae	g__	s__	N/A	475	475_1	F6D7_bin.107.fa	F6D7_bin.107.fa	95.71	3.75	248
F6DG_bin.92	d__Bacteria	p__Firmicutes	c__Clostridia	o__Lachnospirales	f__Anaerotignaceae	g__Anaerotignum	s__	N/A	442	442_0	F6DG_bin.92.fa	F6DG_bin.92.fa	91.71	1.03	249
F68E_bin.17	d__Bacteria	p__Firmicutes	c__Clostridia	o__Lachnospirales	f__Anaerotignaceae	g__Anaerotignum	s__Anaerotignum lactatifermentans	GCF_900142265.1	441	441_0	F68E_bin.17.fa	F68E_bin.17.fa	95.64	0	249
F68C_bin.16	d__Bacteria	p__Firmicutes	c__Clostridia	o__Lachnospirales	f__Anaerotignaceae	g__Anaerotignum	s__Anaerotignum sp000436415	GCA_000436415.1	445	445_8	F68C_bin.16.fa	F68C_bin.16.fa	96.64	0	249
F68F_bin.16	d__Bacteria	p__Firmicutes	c__Clostridia	o__Lachnospirales	f__Anaerotignaceae	g__Anaerotignum	s__Anaerotignum sp000436415	GCA_000436415.1	445	445_1	F68F_bin.16.fa	F68F_bin.16.fa	96.98	0	249

Table S3. Taxonomic classifiation of the 2,421 detected MAGs

	Domain	Phylum	Class	Order	Family	Genus	Species	RefGenome	primary_cluster	secondary_cluster	repr_genome	genome	CheckM_completeness	CheckM_contamination	
F68H_bin.32	d__Bacteria	p__Firmicutes	c__Clostridia	o__Lachnospirales	f__Anaerotignaceae	g__Anaerotignum	s__Anaerotignum sp000436415	GCA_000436415.1	445	445_3	F68H_bin.32.fa	F68H_bin.32.fa	69.87	0.73	249
F68J_bin.29	d__Bacteria	p__Firmicutes	c__Clostridia	o__Lachnospirales	f__Anaerotignaceae	g__Anaerotignum	s__Anaerotignum sp000436415	GCA_000436415.1	445	445_2	F68J_bin.29.fa	F68J_bin.29.fa	95.97	3.86	249
F68K_bin.79	d__Bacteria	p__Firmicutes	c__Clostridia	o__Lachnospirales	f__Anaerotignaceae	g__Anaerotignum	s__Anaerotignum sp000436415	GCA_000436415.1	445	445_10	F68K_bin.79.fa	F68K_bin.79.fa	97.99	0	249
F68N_bin.65	d__Bacteria	p__Firmicutes	c__Clostridia	o__Lachnospirales	f__Anaerotignaceae	g__Anaerotignum	s__Anaerotignum sp000436415	GCA_000436415.1	445	445_12	F68N_bin.65.fa	F68N_bin.65.fa	97.99	0	249
F68P_bin.6	d__Bacteria	p__Firmicutes	c__Clostridia	o__Lachnospirales	f__Anaerotignaceae	g__Anaerotignum	s__Anaerotignum sp000436415	GCA_000436415.1	445	445_11	F68P_bin.6.fa	F68P_bin.6.fa	93.5	0	249
F68U_bin.70	d__Bacteria	p__Firmicutes	c__Clostridia	o__Lachnospirales	f__Anaerotignaceae	g__Anaerotignum	s__Anaerotignum sp000436415	GCA_000436415.1	445	445_14	F68U_bin.70.fa	F68U_bin.70.fa	91.95	0	249

Table S3. Taxonomic classifiation of the 2,421 detected MAGs

	Domain	Phylum	Class	Order	Family	Genus	Species	RefGenome	primary_cluster	secondary_cluster	repr_genome	genome	CheckM_completeness	CheckM_contamination	
F6D7_bin.52	d__Bacteria	p__Firmicutes	c__Clostridia	o__Lachnospirales	f__Anaerotignaceae	g__Anaerotignum	s__Anaerotignum sp000436415	GCA_000436415.1	445	445_5	F6D7_bin.52.fa	F6D7_bin.52.fa	97.99	0	249
F6D8_bin.57	d__Bacteria	p__Firmicutes	c__Clostridia	o__Lachnospirales	f__Anaerotignaceae	g__Anaerotignum	s__Anaerotignum sp000436415	GCA_000436415.1	445	445_6	F6D8_bin.57.fa	F6D8_bin.57.fa	92.95	0	249
F6DA_bin.70	d__Bacteria	p__Firmicutes	c__Clostridia	o__Lachnospirales	f__Anaerotignaceae	g__Anaerotignum	s__Anaerotignum sp000436415	GCA_000436415.1	445	445_7	F6DA_bin.70.fa	F6DA_bin.70.fa	97.99	0	249
F68R_bin.5	d__Bacteria	p__Firmicutes	c__Clostridia	o__Lachnospirales	f__Anaerotignaceae	g__Anaerotignum	s__Anaerotignum sp000436415	GCA_000436415.1	445	445_13	F6DB_bin.103.fa	F68R_bin.5.fa	82.7	1.34	249
F6DB_bin.103	d__Bacteria	p__Firmicutes	c__Clostridia	o__Lachnospirales	f__Anaerotignaceae	g__Anaerotignum	s__Anaerotignum sp000436415	GCA_000436415.1	445	445_13	F6DB_bin.103.fa	F6DB_bin.103.fa	97.99	0	249
F68G_bin.71	d__Bacteria	p__Firmicutes	c__Clostridia	o__Lachnospirales	f__Anaerotignaceae	g__Anaerotignum	s__Anaerotignum sp000436415	GCA_000436415.1	445	445_4	F6DG_bin.50.fa	F68G_bin.71.fa	94.3	0.06	249

Table S3. Taxonomic classifiation of the 2,421 detected MAGs

	Domain	Phylum	Class	Order	Family	Genus	Species	RefGenome	primary_cluster	secondary_cluster	repr_genome	genome	CheckM_completeness	CheckM_contamination	
F6DG_bin.50	d__Bacteria	p__Firmicutes	c__Clostridia	o__Lachnospirales	f__Anaerotignaceae	g__Anaerotignum	s__Anaerotignum sp000436415	GCA_000436415.1	445	445_4	F6DG_bin.50.fa	F6DG_bin.50.fa	97.99	0	249
F68W_bin.28	d__Bacteria	p__Firmicutes	c__Clostridia	o__Lachnospirales	f__Anaerotignaceae	g__Anaerotignum	s__Anaerotignum sp000436415	GCA_000436415.1	445	445_9	F6DH_bin.108.fa	F68W_bin.28.fa	94.94	0	249
F6DH_bin.108	d__Bacteria	p__Firmicutes	c__Clostridia	o__Lachnospirales	f__Anaerotignaceae	g__Anaerotignum	s__Anaerotignum sp000436415	GCA_000436415.1	445	445_9	F6DH_bin.108.fa	F6DH_bin.108.fa	96.64	0	249
F68C_bin.55	d__Bacteria	p__Firmicutes	c__Clostridia	o__Lachnospirales	f__Lachnospiraceae	g__	s__	N/A	317	317_1	F68C_bin.55.fa	F68C_bin.55.fa	69.3	3.51	250
F68F_bin.14	d__Bacteria	p__Firmicutes	c__Clostridia	o__Lachnospirales	f__Lachnospiraceae	g__	s__	N/A	317	317_3	F68F_bin.14.fa	F68F_bin.14.fa	56.14	1.75	251
F68U_bin.31	d__Bacteria	p__Firmicutes	c__Clostridia	o__Lachnospirales	f__Lachnospiraceae	g__	s__	N/A	317	317_5	F68U_bin.31.fa	F68U_bin.31.fa	78.8	0.63	252
F6DA_bin.21	d__Bacteria	p__Firmicutes	c__Clostridia	o__Lachnospirales	f__Lachnospiraceae	g__	s__	N/A	317	317_4	F6DA_bin.21.fa	F6DA_bin.21.fa	79.94	4.01	253
F6DH_bin.84	d__Bacteria	p__Firmicutes	c__Clostridia	o__Lachnospirales	f__Lachnospiraceae	g__	s__	N/A	317	317_2	F6DH_bin.84.fa	F6DH_bin.84.fa	50.88	1.75	254

Table S3. Taxonomic classifiation of the 2,421 detected MAGs

	Domain	Phylum	Class	Order	Family	Genus	Species	RefGenome	primary_cluster	secondary_cluster	repr_genome	genome	CheckM_completeness	CheckM_contamination	
F68G_bin.50	d__Bacteria	p__Firmicutes	c__Clostridia	o__Lachnospirales	f__Lachnospiraceae	g__	s__	N/A	349	349_1	F68G_bin.50.fa	F68G_bin.50.fa	96.95	0.89	255
F68R_bin.64	d__Bacteria	p__Firmicutes	c__Clostridia	o__Lachnospirales	f__Lachnospiraceae	g__	s__	N/A	349	349_1	F68G_bin.50.fa	F68R_bin.64.fa	96.14	4.31	256
F68U_bin.111	d__Bacteria	p__Firmicutes	c__Clostridia	o__Lachnospirales	f__Lachnospiraceae	g__	s__	N/A	349	349_1	F68G_bin.50.fa	F68U_bin.111.fa	59.44	4.39	257
F6D7_bin.47	d__Bacteria	p__Firmicutes	c__Clostridia	o__Lachnospirales	f__Lachnospiraceae	g__	s__	N/A	349	349_1	F68G_bin.50.fa	F6D7_bin.47.fa	98.07	1.21	258
F6DG_bin.86	d__Bacteria	p__Firmicutes	c__Clostridia	o__Lachnospirales	f__Lachnospiraceae	g__	s__	N/A	349	349_1	F68G_bin.50.fa	F6DG_bin.86.fa	92.18	1.55	259
F6MY_bin.45	d__Bacteria	p__Firmicutes	c__Clostridia	o__Lachnospirales	f__Lachnospiraceae	g__	s__	N/A	349	349_1	F68G_bin.50.fa	F6MY_bin.45.fa	97.21	0.97	260
F68K_bin.20	d__Bacteria	p__Firmicutes	c__Clostridia	o__Lachnospirales	f__Lachnospiraceae	g__	s__	N/A	350	350_0	F68K_bin.20.fa	F68K_bin.20.fa	88.89	0.24	261
F6D7_bin.3	d__Bacteria	p__Firmicutes	c__Clostridia	o__Lachnospirales	f__Lachnospiraceae	g__Acetatifactor	s__Acetatifactor sp003447295	GCA_003447295.1	367	367_19	F6D7_bin.3.fa	F6D7_bin.3.fa	99.28	1.93	261
F68K_bin.55	d__Bacteria	p__Firmicutes	c__Clostridia	o__Lachnospirales	f__Lachnospiraceae	g__Acetatifactor	s__Acetatifactor sp900066365	GCA_900066365.1	367	367_13	F68K_bin.55.fa	F68K_bin.55.fa	94.36	1.13	261

Table S3. Taxonomic classifiation of the 2,421 detected MAGs

	Domain	Phylum	Class	Order	Family	Genus	Species	RefGenome	primary_cluster	secondary_cluster	repr_genome	genome	CheckM_completeness	CheckM_contamination	
F68R_bin.27	d__Bacteria	p__Firmicutes	c__Clostridia	o__Lachnospirales	f__Lachnospiraceae	g__Acetatifactor	s__Acetatifactor sp900066365	GCA_900066365.1	367	367_11	F68R_bin.27.fa	F68R_bin.27.fa	89.08	0.97	261
F68T_bin.89	d__Bacteria	p__Firmicutes	c__Clostridia	o__Lachnospirales	f__Lachnospiraceae	g__Acetatifactor	s__Acetatifactor sp900066365	GCA_900066365.1	367	367_16	F68T_bin.89.fa	F68T_bin.89.fa	74.37	1.21	261
F68U_bin.39	d__Bacteria	p__Firmicutes	c__Clostridia	o__Lachnospirales	f__Lachnospiraceae	g__Acetatifactor	s__Acetatifactor sp900066365	GCA_900066365.1	367	367_17	F68U_bin.39.fa	F68U_bin.39.fa	99.03	2.09	261
F68V_bin.53	d__Bacteria	p__Firmicutes	c__Clostridia	o__Lachnospirales	f__Lachnospiraceae	g__Acetatifactor	s__Acetatifactor sp900066365	GCA_900066365.1	367	367_12	F68V_bin.53.fa	F68V_bin.53.fa	94.44	1.05	261
F6D8_bin.78	d__Bacteria	p__Firmicutes	c__Clostridia	o__Lachnospirales	f__Lachnospiraceae	g__Acetatifactor	s__Acetatifactor sp900066365	GCA_900066365.1	367	367_18	F6D8_bin.78.fa	F6D8_bin.78.fa	52.19	4.35	261
F6DA_bin.62	d__Bacteria	p__Firmicutes	c__Clostridia	o__Lachnospirales	f__Lachnospiraceae	g__Acetatifactor	s__Acetatifactor sp900066365	GCA_900066365.1	367	367_14	F6DA_bin.62.fa	F6DA_bin.62.fa	67.14	2.5	261

Table S3. Taxonomic classifiation of the 2,421 detected MAGs

	Domain	Phylum	Class	Order	Family	Genus	Species	RefGenome	primary_cluster	secondary_cluster	repr_genome	genome	CheckM_completeness	CheckM_contamination	
F6DE_bin.45	d__Bacteria	p__Firmicutes	c__Clostridia	o__Lachnospirales	f__Lachnospiraceae	g__Acetatifactor	s__Acetatifactor sp900066365	GCA_900066365.1	367	367_15	F6DE_bin.45.fa	F6DE_bin.45.fa	78.68	1.93	261
F68C_bin.36	d__Bacteria	p__Firmicutes	c__Clostridia	o__Lachnospirales	f__Lachnospiraceae	g__Acetatifactor	s__Acetatifactor sp900066565	GCA_900066565.1	367	367_1	F68C_bin.36.fa	F68C_bin.36.fa	80	1.45	261
F68F_bin.84	d__Bacteria	p__Firmicutes	c__Clostridia	o__Lachnospirales	f__Lachnospiraceae	g__Acetatifactor	s__Acetatifactor sp900066565	GCA_900066565.1	367	367_9	F68F_bin.84.fa	F68F_bin.84.fa	97.13	1.93	261
F68N_bin.40	d__Bacteria	p__Firmicutes	c__Clostridia	o__Lachnospirales	f__Lachnospiraceae	g__Acetatifactor	s__Acetatifactor sp900066565	GCA_900066565.1	367	367_7	F68N_bin.40.fa	F68N_bin.40.fa	74.94	3.19	261
F68P_bin.11	d__Bacteria	p__Firmicutes	c__Clostridia	o__Lachnospirales	f__Lachnospiraceae	g__Acetatifactor	s__Acetatifactor sp900066565	GCA_900066565.1	367	367_6	F68P_bin.11.fa	F68P_bin.11.fa	98.21	1.93	261
F68R_bin.49	d__Bacteria	p__Firmicutes	c__Clostridia	o__Lachnospirales	f__Lachnospiraceae	g__Acetatifactor	s__Acetatifactor sp900066565	GCA_900066565.1	367	367_3	F68R_bin.49.fa	F68R_bin.49.fa	65.31	0	261

Table S3. Taxonomic classifiation of the 2,421 detected MAGs

	Domain	Phylum	Class	Order	Family	Genus	Species	RefGenome	primary_cluster	secondary_cluster	repr_genome	genome	CheckM_completeness	CheckM_contamination	
F68T_bin.61	d__Bacteria	p__Firmicutes	c__Clostridia	o__Lachnospirales	f__Lachnospiraceae	g__Acetatifactor	s__Acetatifactor sp900066565	GCA_900066565.1	367	367_10	F68T_bin.61.fa	F68T_bin.61.fa	83.23	1.29	261
F6DA_bin.47	d__Bacteria	p__Firmicutes	c__Clostridia	o__Lachnospirales	f__Lachnospiraceae	g__Acetatifactor	s__Acetatifactor sp900066565	GCA_900066565.1	367	367_5	F6DA_bin.47.fa	F6DA_bin.47.fa	91.08	2.42	261
F6DB_bin.42	d__Bacteria	p__Firmicutes	c__Clostridia	o__Lachnospirales	f__Lachnospiraceae	g__Acetatifactor	s__Acetatifactor sp900066565	GCA_900066565.1	367	367_2	F6DB_bin.42.fa	F6DB_bin.42.fa	71.72	2.5	261
F6DG_bin.79	d__Bacteria	p__Firmicutes	c__Clostridia	o__Lachnospirales	f__Lachnospiraceae	g__Acetatifactor	s__Acetatifactor sp900066565	GCA_900066565.1	367	367_4	F6DG_bin.79.fa	F6DG_bin.79.fa	91.55	2.5	261
F6MY_bin.10	d__Bacteria	p__Firmicutes	c__Clostridia	o__Lachnospirales	f__Lachnospiraceae	g__Acetatifactor	s__Acetatifactor sp900066565	GCA_900066565.1	367	367_8	F6MY_bin.10.fa	F6MY_bin.10.fa	85.12	2.42	261
F68F_bin.55	d__Bacteria	p__Firmicutes	c__Clostridia	o__Lachnospirales	f__Lachnospiraceae	g__Agathobacter	s__	N/A	340	340_1	F68F_bin.55.fa	F68F_bin.55.fa	97.1	0.24	262
F68N_bin.28	d__Bacteria	p__Firmicutes	c__Clostridia	o__Lachnospirales	f__Lachnospiraceae	g__Agathobacter	s__	N/A	340	340_4	F68N_bin.28.fa	F68N_bin.28.fa	96.49	2.42	263

Table S3. Taxonomic classifiation of the 2,421 detected MAGs

	Domain	Phylum	Class	Order	Family	Genus	Species	RefGenome	primary_cluster	secondary_cluster	repr_genome	genome	CheckM_completeness	CheckM_contamination	
F68R_bin.93	d__Bacteria	p__Firmicutes	c__Clostridia	o__Lachnospirales	f__Lachnospiraceae	g__Agathobacter	s__	N/A	340	340_2	F68R_bin.93.fa	F68R_bin.93.fa	50.18	0	264
F68V_bin.9	d__Bacteria	p__Firmicutes	c__Clostridia	o__Lachnospirales	f__Lachnospiraceae	g__Agathobacter	s__	N/A	340	340_3	F68V_bin.9.fa	F68V_bin.9.fa	56.2	3.02	265
F68C_bin.47	d__Bacteria	p__Firmicutes	c__Clostridia	o__Lachnospirales	f__Lachnospiraceae	g__Agathobacter	s__Agathobacter faecis	GCF_001405615.1	339	339_7	F68C_bin.47.fa	F68C_bin.47.fa	98.55	1.76	265
F68E_bin.3	d__Bacteria	p__Firmicutes	c__Clostridia	o__Lachnospirales	f__Lachnospiraceae	g__Agathobacter	s__Agathobacter faecis	GCF_001405615.1	339	339_9	F68E_bin.3.fa	F68E_bin.3.fa	98.07	1.45	265
F68H_bin.2	d__Bacteria	p__Firmicutes	c__Clostridia	o__Lachnospirales	f__Lachnospiraceae	g__Agathobacter	s__Agathobacter faecis	GCF_001405615.1	339	339_3	F68H_bin.2.fa	F68H_bin.2.fa	96.14	3.67	265
F68R_bin.58	d__Bacteria	p__Firmicutes	c__Clostridia	o__Lachnospirales	f__Lachnospiraceae	g__Agathobacter	s__Agathobacter faecis	GCF_001405615.1	339	339_6	F68R_bin.58.fa	F68R_bin.58.fa	53.68	0	265
F68T_bin.103	d__Bacteria	p__Firmicutes	c__Clostridia	o__Lachnospirales	f__Lachnospiraceae	g__Agathobacter	s__Agathobacter faecis	GCF_001405615.1	339	339_1	F68U_bin.81.fa	F68T_bin.103.fa	71.98	1.04	265
F68U_bin.81	d__Bacteria	p__Firmicutes	c__Clostridia	o__Lachnospirales	f__Lachnospiraceae	g__Agathobacter	s__Agathobacter faecis	GCF_001405615.1	339	339_1	F68U_bin.81.fa	F68U_bin.81.fa	99.03	1.28	265

Table S3. Taxonomic classifiation of the 2,421 detected MAGs

	Domain	Phylum	Class	Order	Family	Genus	Species	RefGenome	primary_cluster	secondary_cluster	repr_genome	genome	CheckM_completeness	CheckM_contamination	
F6D8_bin.66	d__Bacteria	p__Firmicutes	c__Clostridia	o__Lachnospirales	f__Lachnospiraceae	g__Agathobacter	s__Agathobacter faecis	GCF_001405615.1	339	339_4	F6D8_bin.66.fa	F6D8_bin.66.fa	96.62	2.24	265
F68N_bin.26	d__Bacteria	p__Firmicutes	c__Clostridia	o__Lachnospirales	f__Lachnospiraceae	g__Agathobacter	s__Agathobacter faecis	GCF_001405615.1	339	339_2	F6D9_bin.78.fa	F68N_bin.26.fa	70	1.72	265
F6D9_bin.78	d__Bacteria	p__Firmicutes	c__Clostridia	o__Lachnospirales	f__Lachnospiraceae	g__Agathobacter	s__Agathobacter faecis	GCF_001405615.1	339	339_2	F6D9_bin.78.fa	F6D9_bin.78.fa	91.55	1.28	265
F6DA_bin.12	d__Bacteria	p__Firmicutes	c__Clostridia	o__Lachnospirales	f__Lachnospiraceae	g__Agathobacter	s__Agathobacter faecis	GCF_001405615.1	339	339_8	F6DA_bin.12.fa	F6DA_bin.12.fa	84.3	1.44	265
F6MY_bin.70	d__Bacteria	p__Firmicutes	c__Clostridia	o__Lachnospirales	f__Lachnospiraceae	g__Agathobacter	s__Agathobacter faecis	GCF_001405615.1	339	339_5	F6MY_bin.70.fa	F6MY_bin.70.fa	93.02	3.75	265
F68C_bin.2	d__Bacteria	p__Firmicutes	c__Clostridia	o__Lachnospirales	f__Lachnospiraceae	g__Agathobacter	s__Agathobacter rectale	GCF_000020605.1	341	341_4	F68C_bin.2.fa	F68C_bin.2.fa	99.18	0	265
F6D7_bin.102	d__Bacteria	p__Firmicutes	c__Clostridia	o__Lachnospirales	f__Lachnospiraceae	g__Agathobacter	s__Agathobacter rectale	GCF_000020605.1	341	341_4	F68C_bin.2.fa	F6D7_bin.102.fa	63.16	0	265

Table S3. Taxonomic classifiation of the 2,421 detected MAGs

	Domain	Phylum	Class	Order	Family	Genus	Species	RefGenome	primary_cluster	secondary_cluster	repr_genome	genome	CheckM_completeness	CheckM_contamination	
F6DE_bin.1	d__Bacteria	p__Firmicutes	c__Clostridia	o__Lachnospirales	f__Lachnospiraceae	g__Agathobacter	s__Agathobacter rectale	GCF_000020605.1	341	341_4	F68C_bin.2.fa	F6DE_bin.1.fa	75.5	3.97	265
F68G_bin.28	d__Bacteria	p__Firmicutes	c__Clostridia	o__Lachnospirales	f__Lachnospiraceae	g__Agathobacter	s__Agathobacter rectale	GCF_000020605.1	341	341_12	F68G_bin.28.fa	F68G_bin.28.fa	99.28	0.97	265
F68R_bin.92	d__Bacteria	p__Firmicutes	c__Clostridia	o__Lachnospirales	f__Lachnospiraceae	g__Agathobacter	s__Agathobacter rectale	GCF_000020605.1	341	341_6	F68R_bin.92.fa	F68R_bin.92.fa	97.67	0.24	265
F68T_bin.23	d__Bacteria	p__Firmicutes	c__Clostridia	o__Lachnospirales	f__Lachnospiraceae	g__Agathobacter	s__Agathobacter rectale	GCF_000020605.1	341	341_3	F68T_bin.23.fa	F68T_bin.23.fa	73.83	0	265
F68U_bin.47	d__Bacteria	p__Firmicutes	c__Clostridia	o__Lachnospirales	f__Lachnospiraceae	g__Agathobacter	s__Agathobacter rectale	GCF_000020605.1	341	341_8	F68U_bin.47.fa	F68U_bin.47.fa	98.07	0	265
F68V_bin.6	d__Bacteria	p__Firmicutes	c__Clostridia	o__Lachnospirales	f__Lachnospiraceae	g__Agathobacter	s__Agathobacter rectale	GCF_000020605.1	341	341_5	F68V_bin.6.fa	F68V_bin.6.fa	97.1	0	265
F68W_bin.80	d__Bacteria	p__Firmicutes	c__Clostridia	o__Lachnospirales	f__Lachnospiraceae	g__Agathobacter	s__Agathobacter rectale	GCF_000020605.1	341	341_2	F68W_bin.80.fa	F68W_bin.80.fa	99.28	0	265

Table S3. Taxonomic classifiation of the 2,421 detected MAGs

	Domain	Phylum	Class	Order	Family	Genus	Species	RefGenome	primary_cluster	secondary_cluster	repr_genome	genome	CheckM_completeness	CheckM_contamination	
F6DH_bin.90	d__Bacteria	p__Firmicutes	c__Clostridia	o__Lachnospirales	f__Lachnospiraceae	g__Agathobacter	s__Agathobacter rectale	GCF_000020605.1	341	341_2	F68W_bin.80.fa	F6DH_bin.90.fa	98.79	0	265
F6D9_bin.3	d__Bacteria	p__Firmicutes	c__Clostridia	o__Lachnospirales	f__Lachnospiraceae	g__Agathobacter	s__Agathobacter rectale	GCF_000020605.1	341	341_10	F6D9_bin.3.fa	F6D9_bin.3.fa	99.28	0	265
F6DA_bin.18	d__Bacteria	p__Firmicutes	c__Clostridia	o__Lachnospirales	f__Lachnospiraceae	g__Agathobacter	s__Agathobacter rectale	GCF_000020605.1	341	341_9	F6DA_bin.18.fa	F6DA_bin.18.fa	99.03	0	265
F6DB_bin.33	d__Bacteria	p__Firmicutes	c__Clostridia	o__Lachnospirales	f__Lachnospiraceae	g__Agathobacter	s__Agathobacter rectale	GCF_000020605.1	341	341_7	F6DB_bin.33.fa	F6DB_bin.33.fa	97.83	0	265
F6DG_bin.96	d__Bacteria	p__Firmicutes	c__Clostridia	o__Lachnospirales	f__Lachnospiraceae	g__Agathobacter	s__Agathobacter rectale	GCF_000020605.1	341	341_11	F6DG_bin.96.fa	F6DG_bin.96.fa	91.11	0.4	265
F68H_bin.69	d__Bacteria	p__Firmicutes	c__Clostridia	o__Lachnospirales	f__Lachnospiraceae	g__Agathobacter	s__Agathobacter rectale	GCF_000020605.1	341	341_1	F6MY_bin.113.fa	F68H_bin.69.fa	86.23	0.48	265
F68K_bin.4	d__Bacteria	p__Firmicutes	c__Clostridia	o__Lachnospirales	f__Lachnospiraceae	g__Agathobacter	s__Agathobacter rectale	GCF_000020605.1	341	341_1	F6MY_bin.113.fa	F68K_bin.4.fa	96.38	0	265

Table S3. Taxonomic classifiation of the 2,421 detected MAGs

	Domain	Phylum	Class	Order	Family	Genus	Species	RefGenome	primary_cluster	secondary_cluster	repr_genome	genome	CheckM_completeness	CheckM_contamination	
F6MY_bin.113	d__Bacteria	p__Firmicutes	c__Clostridia	o__Lachnospirales	f__Lachnospiraceae	g__Agathobacter	s__Agathobacter rectale	GCF_000020605.1	341	341_1	F6MY_bin.113.fa	F6MY_bin.113.fa	96.86	0	265
F6D9_bin.10	d__Bacteria	p__Firmicutes	c__Clostridia	o__Lachnospirales	f__Lachnospiraceae	g__Blautia	s__Blautia hansenii	GCF_002222595.2	299	299_0	F6D9_bin.10.fa	F6D9_bin.10.fa	99.36	4.46	265
F68K_bin.40	d__Bacteria	p__Firmicutes	c__Clostridia	o__Lachnospirales	f__Lachnospiraceae	g__Blautia	s__Blautia sp000436935	GCA_000436935.1	319	319_1	F68K_bin.40.fa	F68K_bin.40.fa	80.59	0.11	265
F68P_bin.56	d__Bacteria	p__Firmicutes	c__Clostridia	o__Lachnospirales	f__Lachnospiraceae	g__Blautia	s__Blautia sp000436935	GCA_000436935.1	319	319_5	F68P_bin.56.fa	F68P_bin.56.fa	76.29	2.23	265
F68U_bin.75	d__Bacteria	p__Firmicutes	c__Clostridia	o__Lachnospirales	f__Lachnospiraceae	g__Blautia	s__Blautia sp000436935	GCA_000436935.1	319	319_4	F68U_bin.75.fa	F68U_bin.75.fa	94.27	1.04	265
F6D7_bin.78	d__Bacteria	p__Firmicutes	c__Clostridia	o__Lachnospirales	f__Lachnospiraceae	g__Blautia	s__Blautia sp000436935	GCA_000436935.1	319	319_2	F6D7_bin.78.fa	F6D7_bin.78.fa	81.34	2.49	265
F6DA_bin.85	d__Bacteria	p__Firmicutes	c__Clostridia	o__Lachnospirales	f__Lachnospiraceae	g__Blautia	s__Blautia sp000436935	GCA_000436935.1	319	319_3	F6DA_bin.85.fa	F6DA_bin.85.fa	96.18	0.32	265

Table S3. Taxonomic classifiation of the 2,421 detected MAGs

	Domain	Phylum	Class	Order	Family	Genus	Species	RefGenome	primary_cluster	secondary_cluster	repr_genome	genome	CheckM_completeness	CheckM_contamination	
F6DG_bin.7	d__Bacteria	p__Firmicutes	c__Clostridia	o__Lachnospirales	f__Lachnospiraceae	g__Blautia	s__Blautia sp000436935	GCA_000436935.1	319	319_3	F6DA_bin.85.fa	F6DG_bin.7.fa	82	2.55	265
F68E_bin.15	d__Bacteria	p__Firmicutes	c__Clostridia	o__Lachnospirales	f__Lachnospiraceae	g__Blautia	s__Blautia sp001304935	GCA_001304935.1	357	357_0	F68E_bin.15.fa	F68E_bin.15.fa	52.39	0	265
F6DH_bin.63	d__Bacteria	p__Firmicutes	c__Clostridia	o__Lachnospirales	f__Lachnospiraceae	g__Blautia_A	s__	N/A	311	311_0	F6DH_bin.63.fa	F6DH_bin.63.fa	56.41	1.91	266
F68E_bin.7	d__Bacteria	p__Firmicutes	c__Clostridia	o__Lachnospirales	f__Lachnospiraceae	g__Blautia_A	s__	N/A	313	313_3	F68E_bin.7.fa	F68E_bin.7.fa	70	4.32	267
F6D9_bin.48	d__Bacteria	p__Firmicutes	c__Clostridia	o__Lachnospirales	f__Lachnospiraceae	g__Blautia_A	s__	N/A	313	313_1	F6D9_bin.48.fa	F6D9_bin.48.fa	93.81	1.64	268
F6DG_bin.5	d__Bacteria	p__Firmicutes	c__Clostridia	o__Lachnospirales	f__Lachnospiraceae	g__Blautia_A	s__	N/A	313	313_2	F6DG_bin.5.fa	F6DG_bin.5.fa	68.11	1.27	269
F6D9_bin.86	d__Bacteria	p__Firmicutes	c__Clostridia	o__Lachnospirales	f__Lachnospiraceae	g__Blautia_A	s__	N/A	338	338_0	F6D9_bin.86.fa	F6D9_bin.86.fa	89.87	1.05	270
F68K_bin.30	d__Bacteria	p__Firmicutes	c__Clostridia	o__Lachnospirales	f__Lachnospiraceae	g__Blautia_A	s__Blautia_A massiliensis	GCF_001487165.1	315	315_0	F68K_bin.30.fa	F68K_bin.30.fa	82.65	2.85	270
F6DB_bin.34	d__Bacteria	p__Firmicutes	c__Clostridia	o__Lachnospirales	f__Lachnospiraceae	g__Blautia_A	s__Blautia_A obeum	GCF_000153905.1	310	310_0	F6DB_bin.34.fa	F6DB_bin.34.fa	70.67	1.27	270

Table S3. Taxonomic classifiation of the 2,421 detected MAGs

	Domain	Phylum	Class	Order	Family	Genus	Species	RefGenome	primary_cluster	secondary_cluster	repr_genome	genome	CheckM_completeness	CheckM_contamination	
F68D_bin.62	d__Bacteria	p__Firmicutes	c__Clostridia	o__Lachnospirales	f__Lachnospiraceae	g__Blautia_A	s__Blautia_A obeum_B	GCA_000210015.1	309	309_1	F68D_bin.62.fa	F68D_bin.62.fa	95.07	2.03	270
F6DB_bin.35	d__Bacteria	p__Firmicutes	c__Clostridia	o__Lachnospirales	f__Lachnospiraceae	g__Blautia_A	s__Blautia_A obeum_B	GCA_000210015.1	309	309_2	F6DB_bin.35.fa	F6DB_bin.35.fa	54.9	0	270
F68G_bin.14	d__Bacteria	p__Firmicutes	c__Clostridia	o__Lachnospirales	f__Lachnospiraceae	g__Blautia_A	s__Blautia_A sp000285855	GCA_000285855.2	312	312_1	F68G_bin.14.fa	F68G_bin.14.fa	90.56	1.27	270
F68K_bin.97	d__Bacteria	p__Firmicutes	c__Clostridia	o__Lachnospirales	f__Lachnospiraceae	g__Blautia_A	s__Blautia_A sp000285855	GCA_000285855.2	312	312_2	F68K_bin.97.fa	F68K_bin.97.fa	50.16	1.75	270
F68F_bin.103	d__Bacteria	p__Firmicutes	c__Clostridia	o__Lachnospirales	f__Lachnospiraceae	g__Blautia_A	s__Blautia_A sp900066165	GCA_900066165.1	307	307_4	F68F_bin.103.fa	F68F_bin.103.fa	91.88	1.5	270
F68G_bin.1	d__Bacteria	p__Firmicutes	c__Clostridia	o__Lachnospirales	f__Lachnospiraceae	g__Blautia_A	s__Blautia_A sp900066165	GCA_900066165.1	307	307_3	F68G_bin.1.fa	F68G_bin.1.fa	56.31	1.75	270
F68H_bin.17	d__Bacteria	p__Firmicutes	c__Clostridia	o__Lachnospirales	f__Lachnospiraceae	g__Blautia_A	s__Blautia_A sp900066165	GCA_900066165.1	307	307_1	F68H_bin.17.fa	F68H_bin.17.fa	57.81	0.88	270
F68K_bin.68	d__Bacteria	p__Firmicutes	c__Clostridia	o__Lachnospirales	f__Lachnospiraceae	g__Blautia_A	s__Blautia_A sp900066165	GCA_900066165.1	307	307_5	F68K_bin.68.fa	F68K_bin.68.fa	55.6	3.7	270

Table S3. Taxonomic classifiation of the 2,421 detected MAGs

	Domain	Phylum	Class	Order	Family	Genus	Species	RefGenome	primary_cluster	secondary_cluster	repr_genome	genome	CheckM_completeness	CheckM_contamination	
F6DA_bin.17	d__Bacteria	p__Firmicutes	c__Clostridia	o__Lachnospirales	f__Lachnospiraceae	g__Blautia_A	s__Blautia_A sp900066165	GCA_900066165.1	307	307_6	F6DA_bin.17.fa	F6DA_bin.17.fa	71.29	0	270
F6DG_bin.105	d__Bacteria	p__Firmicutes	c__Clostridia	o__Lachnospirales	f__Lachnospiraceae	g__Blautia_A	s__Blautia_A sp900066165	GCA_900066165.1	307	307_2	F6DG_bin.105.fa	F6DG_bin.105.fa	51.67	4.47	270
F6DA_bin.54	d__Bacteria	p__Firmicutes	c__Clostridia	o__Lachnospirales	f__Lachnospiraceae	g__Blautia_A	s__Blautia_A wexlerae	GCF_000484655.1	314	314_2	F6DA_bin.54.fa	F6DA_bin.54.fa	87.12	0.84	270
F68W_bin.98	d__Bacteria	p__Firmicutes	c__Clostridia	o__Lachnospirales	f__Lachnospiraceae	g__Blautia_A	s__Blautia_A wexlerae	GCF_000484655.1	314	314_1	F6DH_bin.30.fa	F68W_bin.98.fa	52.96	1.01	270
F6DH_bin.30	d__Bacteria	p__Firmicutes	c__Clostridia	o__Lachnospirales	f__Lachnospiraceae	g__Blautia_A	s__Blautia_A wexlerae	GCF_000484655.1	314	314_1	F6DH_bin.30.fa	F6DH_bin.30.fa	55.82	0	270
F6DG_bin.2	d__Bacteria	p__Firmicutes	c__Clostridia	o__Lachnospirales	f__Lachnospiraceae	g__Butyrivibrio_A	s__Butyrivibrio_A crossotus	GCF_000156015.1	335	335_0	F6DG_bin.2.fa	F6DG_bin.2.fa	91.56	0.34	270
F68H_bin.12	d__Bacteria	p__Firmicutes	c__Clostridia	o__Lachnospirales	f__Lachnospiraceae	g__Butyrivibrio_A	s__Butyrivibrio_A sp000431815	GCA_000431815.1	360	360_1	F6MY_bin.12.fa	F68H_bin.12.fa	96.64	0.69	270

Table S3. Taxonomic classifiation of the 2,421 detected MAGs

	Domain	Phylum	Class	Order	Family	Genus	Species	RefGenome	primary_cluster	secondary_cluster	repr_genome	genome	CheckM_completeness	CheckM_contamination	
F6MY_bin.12	d__Bacteria	p__Firmicutes	c__Clostridia	o__Lachnospirales	f__Lachnospiraceae	g__Butyrivibrio_A	s__Butyrivibrio_A sp000431815	GCA_000431815.1	360	360_1	F6MY_bin.12.fa	F6MY_bin.12.fa	100	0.67	270
F68C_bin.63	d__Bacteria	p__Firmicutes	c__Clostridia	o__Lachnospirales	f__Lachnospiraceae	g__CAG-127	s__CAG-127 sp900319515	GCA_900319515.1	372	372_3	F68C_bin.63.fa	F68C_bin.63.fa	81.88	0	270
F6D7_bin.100	d__Bacteria	p__Firmicutes	c__Clostridia	o__Lachnospirales	f__Lachnospiraceae	g__CAG-127	s__CAG-127 sp900319515	GCA_900319515.1	372	372_3	F68C_bin.63.fa	F6D7_bin.100.fa	79.19	0	270
F68H_bin.4	d__Bacteria	p__Firmicutes	c__Clostridia	o__Lachnospirales	f__Lachnospiraceae	g__CAG-127	s__CAG-127 sp900319515	GCA_900319515.1	372	372_7	F68H_bin.4.fa	F68H_bin.4.fa	98.66	0.67	270
F68K_bin.6	d__Bacteria	p__Firmicutes	c__Clostridia	o__Lachnospirales	f__Lachnospiraceae	g__CAG-127	s__CAG-127 sp900319515	GCA_900319515.1	372	372_4	F68K_bin.6.fa	F68K_bin.6.fa	85.23	0	270
F68M_bin.72	d__Bacteria	p__Firmicutes	c__Clostridia	o__Lachnospirales	f__Lachnospiraceae	g__CAG-127	s__CAG-127 sp900319515	GCA_900319515.1	372	372_9	F68M_bin.72.fa	F68M_bin.72.fa	90.84	4.76	270
F68F_bin.58	d__Bacteria	p__Firmicutes	c__Clostridia	o__Lachnospirales	f__Lachnospiraceae	g__CAG-127	s__CAG-127 sp900319515	GCA_900319515.1	372	372_6	F68R_bin.59.fa	F68F_bin.58.fa	98.66	0	270

Table S3. Taxonomic classifiation of the 2,421 detected MAGs

	Domain	Phylum	Class	Order	Family	Genus	Species	RefGenome	primary_cluster	secondary_cluster	repr_genome	genome	CheckM_completeness	CheckM_contamination	
F68R_bin.59	d__Bacteria	p__Firmicutes	c__Clostridia	o__Lachnospirales	f__Lachnospiraceae	g__CAG-127	s__CAG-127 sp900319515	GCA_900319515.1	372	372_6	F68R_bin.59.fa	F68R_bin.59.fa	98.66	0	270
F68V_bin.7	d__Bacteria	p__Firmicutes	c__Clostridia	o__Lachnospirales	f__Lachnospiraceae	g__CAG-127	s__CAG-127 sp900319515	GCA_900319515.1	372	372_2	F68V_bin.7.fa	F68V_bin.7.fa	85.09	0.31	270
F68U_bin.10	d__Bacteria	p__Firmicutes	c__Clostridia	o__Lachnospirales	f__Lachnospiraceae	g__CAG-127	s__CAG-127 sp900319515	GCA_900319515.1	372	372_1	F68W_bin.67.fa	F68U_bin.10.fa	85.23	0	270
F68W_bin.67	d__Bacteria	p__Firmicutes	c__Clostridia	o__Lachnospirales	f__Lachnospiraceae	g__CAG-127	s__CAG-127 sp900319515	GCA_900319515.1	372	372_1	F68W_bin.67.fa	F68W_bin.67.fa	89.93	0	270
F6DH_bin.8	d__Bacteria	p__Firmicutes	c__Clostridia	o__Lachnospirales	f__Lachnospiraceae	g__CAG-127	s__CAG-127 sp900319515	GCA_900319515.1	372	372_1	F68W_bin.67.fa	F6DH_bin.8.fa	86.58	0	270
F6DA_bin.79	d__Bacteria	p__Firmicutes	c__Clostridia	o__Lachnospirales	f__Lachnospiraceae	g__CAG-127	s__CAG-127 sp900319515	GCA_900319515.1	372	372_8	F6DA_bin.79.fa	F6DA_bin.79.fa	90.27	0	270
F6DB_bin.44	d__Bacteria	p__Firmicutes	c__Clostridia	o__Lachnospirales	f__Lachnospiraceae	g__CAG-127	s__CAG-127 sp900319515	GCA_900319515.1	372	372_5	F6DB_bin.44.fa	F6DB_bin.44.fa	97.99	0	270

Table S3. Taxonomic classifiation of the 2,421 detected MAGs

	Domain	Phylum	Class	Order	Family	Genus	Species	RefGenome	primary_cluster	secondary_cluster	repr_genome	genome	CheckM_completeness	CheckM_contamination	
F68V_bin.20	d__Bacteria	p__Firmicutes	c__Clostridia	o__Lachnospirales	f__Lachnospiraceae	g__CAG-45	s__CAG-45 sp900066395	GCA_900066395.1	342	342_1	F68V_bin.20.fa	F68V_bin.20.fa	99.28	1.22	270
F6D8_bin.42	d__Bacteria	p__Firmicutes	c__Clostridia	o__Lachnospirales	f__Lachnospiraceae	g__CAG-45	s__CAG-45 sp900066395	GCA_900066395.1	342	342_2	F6D8_bin.42.fa	F6D8_bin.42.fa	97.04	0.74	270
F6DH_bin.61	d__Bacteria	p__Firmicutes	c__Clostridia	o__Lachnospirales	f__Lachnospiraceae	g__CAG-45	s__CAG-45 sp900066395	GCA_900066395.1	342	342_3	F6DH_bin.61.fa	F6DH_bin.61.fa	93.04	1.53	270
F68G_bin.85	d__Bacteria	p__Firmicutes	c__Clostridia	o__Lachnospirales	f__Lachnospiraceae	g__CAG-510	s__CAG-510 sp000434615	GCA_000434615.1	356	356_1	F68G_bin.85.fa	F68G_bin.85.fa	95.62	2.27	270
F68R_bin.9	d__Bacteria	p__Firmicutes	c__Clostridia	o__Lachnospirales	f__Lachnospiraceae	g__CAG-510	s__CAG-510 sp000434615	GCA_000434615.1	356	356_2	F68R_bin.9.fa	F68R_bin.9.fa	54.39	0	270
F68C_bin.31	d__Bacteria	p__Firmicutes	c__Clostridia	o__Lachnospirales	f__Lachnospiraceae	g__CAG-56	s__CAG-56 sp900066615	GCA_900066615.1	290	290_3	F68C_bin.31.fa	F68C_bin.31.fa	81.83	4.13	270
F68R_bin.72	d__Bacteria	p__Firmicutes	c__Clostridia	o__Lachnospirales	f__Lachnospiraceae	g__CAG-56	s__CAG-56 sp900066615	GCA_900066615.1	290	290_1	F68R_bin.72.fa	F68R_bin.72.fa	96.2	0.71	270

Table S3. Taxonomic classifiation of the 2,421 detected MAGs

	Domain	Phylum	Class	Order	Family	Genus	Species	RefGenome	primary_cluster	secondary_cluster	repr_genome	genome	CheckM_completeness	CheckM_contamination	
F6MY_bin.108	d__Bacteria	p__Firmicutes	c__Clostridia	o__Lachnospirales	f__Lachnospiraceae	g__CAG-56	s__CAG-56 sp900066615	GCA_900066615.1	290	290_1	F68R_bin.72.fa	F6MY_bin.108.fa	50.08	0	270
F68K_bin.111	d__Bacteria	p__Firmicutes	c__Clostridia	o__Lachnospirales	f__Lachnospiraceae	g__CAG-56	s__CAG-56 sp900066615	GCA_900066615.1	290	290_2	F68T_bin.54.fa	F68K_bin.111.fa	71.6	0.63	270
F68T_bin.54	d__Bacteria	p__Firmicutes	c__Clostridia	o__Lachnospirales	f__Lachnospiraceae	g__CAG-56	s__CAG-56 sp900066615	GCA_900066615.1	290	290_2	F68T_bin.54.fa	F68T_bin.54.fa	78.91	0	270
F6DB_bin.8	d__Bacteria	p__Firmicutes	c__Clostridia	o__Lachnospirales	f__Lachnospiraceae	g__CAG-56	s__CAG-56 sp900066615	GCA_900066615.1	290	290_2	F68T_bin.54.fa	F6DB_bin.8.fa	70.25	0.63	270
F68P_bin.46	d__Bacteria	p__Firmicutes	c__Clostridia	o__Lachnospirales	f__Lachnospiraceae	g__CAG-603	s__CAG-603 sp900066105	GCA_900066105.1	361	361_1	F68P_bin.46.fa	F68P_bin.46.fa	98.66	0	270
F6D8_bin.9	d__Bacteria	p__Firmicutes	c__Clostridia	o__Lachnospirales	f__Lachnospiraceae	g__CAG-603	s__CAG-603 sp900066105	GCA_900066105.1	361	361_2	F6D8_bin.9.fa	F6D8_bin.9.fa	98.66	0	270
F6D8_bin.43	d__Bacteria	p__Firmicutes	c__Clostridia	o__Lachnospirales	f__Lachnospiraceae	g__CAG-632	s__CAG-632 sp000431515	GCA_000431515.1	269	269_0	F6D8_bin.43.fa	F6D8_bin.43.fa	99.33	0	270

Table S3. Taxonomic classifiation of the 2,421 detected MAGs

	Domain	Phylum	Class	Order	Family	Genus	Species	RefGenome	primary_cluster	secondary_cluster	repr_genome	genome	CheckM_completeness	CheckM_contamination	
F68M_bin.60	d__Bacteria	p__Firmicutes	c__Clostridia	o__Lachnospirales	f__Lachnospiraceae	g__CAG-791	s__CAG-791 sp000431495	GCA_000431495.1	18	18_1	F68M_bin.60.fa	F68M_bin.60.fa	95.03	0.57	270
F6MX_bin.41	d__Bacteria	p__Firmicutes	c__Clostridia	o__Lachnospirales	f__Lachnospiraceae	g__CAG-791	s__CAG-791 sp000431495	GCA_000431495.1	18	18_1	F68M_bin.60.fa	F6MX_bin.41.fa	72.86	2.94	270
F68F_bin.70	d__Bacteria	p__Firmicutes	c__Clostridia	o__Lachnospirales	f__Lachnospiraceae	g__CAG-81	s__	N/A	272	272_2	F68F_bin.70.fa	F68F_bin.70.fa	99.37	0	271
F68T_bin.48	d__Bacteria	p__Firmicutes	c__Clostridia	o__Lachnospirales	f__Lachnospiraceae	g__CAG-81	s__	N/A	272	272_2	F68F_bin.70.fa	F68T_bin.48.fa	92.03	0.63	272
F6MX_bin.42	d__Bacteria	p__Firmicutes	c__Clostridia	o__Lachnospirales	f__Lachnospiraceae	g__CAG-81	s__	N/A	272	272_2	F68F_bin.70.fa	F6MX_bin.42.fa	94.94	0	273
F68W_bin.60	d__Bacteria	p__Firmicutes	c__Clostridia	o__Lachnospirales	f__Lachnospiraceae	g__CAG-81	s__	N/A	273	273_1	F6DH_bin.33.fa	F68W_bin.60.fa	55.72	0	274
F6DH_bin.33	d__Bacteria	p__Firmicutes	c__Clostridia	o__Lachnospirales	f__Lachnospiraceae	g__CAG-81	s__	N/A	273	273_1	F6DH_bin.33.fa	F6DH_bin.33.fa	93.67	0.63	275
F68K_bin.43	d__Bacteria	p__Firmicutes	c__Clostridia	o__Lachnospirales	f__Lachnospiraceae	g__CAG-81	s__CAG-81 sp000435795	GCA_000435795.1	272	272_1	F68U_bin.23.fa	F68K_bin.43.fa	85.56	2.74	275
F68P_bin.52	d__Bacteria	p__Firmicutes	c__Clostridia	o__Lachnospirales	f__Lachnospiraceae	g__CAG-81	s__CAG-81 sp000435795	GCA_000435795.1	272	272_1	F68U_bin.23.fa	F68P_bin.52.fa	89.83	1.03	275

Table S3. Taxonomic classifiation of the 2,421 detected MAGs

	Domain	Phylum	Class	Order	Family	Genus	Species	RefGenome	primary_cluster	secondary_cluster	repr_genome	genome	CheckM_completeness	CheckM_contamination	
F68R_bin.48	d__Bacteria	p__Firmicutes	c__Clostridia	o__Lachnospirales	f__Lachnospiraceae	g__CAG-81	s__CAG-81 sp000435795	GCA_000435795.1	272	272_1	F68U_bin.23.fa	F68R_bin.48.fa	68.3	2.44	275
F68U_bin.23	d__Bacteria	p__Firmicutes	c__Clostridia	o__Lachnospirales	f__Lachnospiraceae	g__CAG-81	s__CAG-81 sp000435795	GCA_000435795.1	272	272_1	F68U_bin.23.fa	F68U_bin.23.fa	98.1	1.27	275
F6DG_bin.41	d__Bacteria	p__Firmicutes	c__Clostridia	o__Lachnospirales	f__Lachnospiraceae	g__CAG-81	s__CAG-81 sp000435795	GCA_000435795.1	272	272_1	F68U_bin.23.fa	F6DG_bin.41.fa	94.58	1.27	275
F6MY_bin.106	d__Bacteria	p__Firmicutes	c__Clostridia	o__Lachnospirales	f__Lachnospiraceae	g__CAG-81	s__CAG-81 sp000435795	GCA_000435795.1	272	272_1	F68U_bin.23.fa	F6MY_bin.106.fa	93.88	0.79	275
F68R_bin.53	d__Bacteria	p__Firmicutes	c__Clostridia	o__Lachnospirales	f__Lachnospiraceae	g__CAG-81	s__CAG-81 sp900066055	GCA_900066055.1	274	274_7	F68R_bin.53.fa	F68R_bin.53.fa	63.67	1.75	275
F68E_bin.30	d__Bacteria	p__Firmicutes	c__Clostridia	o__Lachnospirales	f__Lachnospiraceae	g__CAG-81	s__CAG-81 sp900066535	GCA_900066535.1	274	274_3	F68E_bin.30.fa	F68E_bin.30.fa	99.37	1.9	275
F68N_bin.62	d__Bacteria	p__Firmicutes	c__Clostridia	o__Lachnospirales	f__Lachnospiraceae	g__CAG-81	s__CAG-81 sp900066535	GCA_900066535.1	274	274_1	F68N_bin.62.fa	F68N_bin.62.fa	60.51	0.63	275

Table S3. Taxonomic classifiation of the 2,421 detected MAGs

	Domain	Phylum	Class	Order	Family	Genus	Species	RefGenome	primary_cluster	secondary_cluster	repr_genome	genome	CheckM_completeness	CheckM_contamination	
F68P_bin.58	d__Bacteria	p__Firmicutes	c__Clostridia	o__Lachnospirales	f__Lachnospiraceae	g__CAG-81	s__CAG-81 sp900066535	GCA_900066535.1	274	274_4	F68P_bin.58.fa	F68P_bin.58.fa	51.69	0	275
F68T_bin.66	d__Bacteria	p__Firmicutes	c__Clostridia	o__Lachnospirales	f__Lachnospiraceae	g__CAG-81	s__CAG-81 sp900066535	GCA_900066535.1	274	274_2	F68T_bin.66.fa	F68T_bin.66.fa	97.47	0	275
F68U_bin.56	d__Bacteria	p__Firmicutes	c__Clostridia	o__Lachnospirales	f__Lachnospiraceae	g__CAG-81	s__CAG-81 sp900066535	GCA_900066535.1	274	274_6	F68U_bin.56.fa	F68U_bin.56.fa	94.62	0.86	275
F6DA_bin.90	d__Bacteria	p__Firmicutes	c__Clostridia	o__Lachnospirales	f__Lachnospiraceae	g__CAG-81	s__CAG-81 sp900066535	GCA_900066535.1	274	274_5	F6DA_bin.90.fa	F6DA_bin.90.fa	73.69	2.74	275
F6DG_bin.102	d__Bacteria	p__Firmicutes	c__Clostridia	o__Lachnospirales	f__Lachnospiraceae	g__CAG-81	s__CAG-81 sp900066785	GCA_900066785.1	274	274_8	F6DG_bin.102.fa	F6DG_bin.102.fa	87.76	0	275
F68T_bin.101	d__Bacteria	p__Firmicutes	c__Clostridia	o__Lachnospirales	f__Lachnospiraceae	g__CAG-882	s__CAG-882 sp003486385	GCA_003486385.1	334	334_3	F68T_bin.101.fa	F68T_bin.101.fa	99.78	0.67	275
F6D8_bin.55	d__Bacteria	p__Firmicutes	c__Clostridia	o__Lachnospirales	f__Lachnospiraceae	g__CAG-882	s__CAG-882 sp003486385	GCA_003486385.1	334	334_1	F6D8_bin.55.fa	F6D8_bin.55.fa	96.09	1.34	275

Table S3. Taxonomic classifiation of the 2,421 detected MAGs

	Domain	Phylum	Class	Order	Family	Genus	Species	RefGenome	primary_cluster	secondary_cluster	repr_genome	genome	CheckM_completeness	CheckM_contamination	
F6DE_bin.13	d__Bacteria	p__Firmicutes	c__Clostridia	o__Lachnospirales	f__Lachnospiraceae	g__CAG-882	s__CAG-882 sp003486385	GCA_003486385.1	334	334_2	F6DE_bin.13.fa	F6DE_bin.13.fa	73.71	1.57	275
F68V_bin.94	d__Bacteria	p__Firmicutes	c__Clostridia	o__Lachnospirales	f__Lachnospiraceae	g__CAG-95	s__CAG-95 sp000438155	GCA_000438155.1	366	366_1	F68V_bin.94.fa	F68V_bin.94.fa	62.43	1.53	275
F6DB_bin.85	d__Bacteria	p__Firmicutes	c__Clostridia	o__Lachnospirales	f__Lachnospiraceae	g__CAG-95	s__CAG-95 sp000438155	GCA_000438155.1	366	366_2	F6DB_bin.85.fa	F6DB_bin.85.fa	84.9	1.75	275
F6DG_bin.45	d__Bacteria	p__Firmicutes	c__Clostridia	o__Lachnospirales	f__Lachnospiraceae	g__CAG-95	s__CAG-95 sp900066375	GCA_900066375.1	316	316_0	F6DG_bin.45.fa	F6DG_bin.45.fa	71.74	1.45	275
F68E_bin.37	d__Bacteria	p__Firmicutes	c__Clostridia	o__Lachnospirales	f__Lachnospiraceae	g__Clostridium_M	s__	N/A	262	262_0	F68E_bin.37.fa	F68E_bin.37.fa	66.59	3.51	276
F6D9_bin.66	d__Bacteria	p__Firmicutes	c__Clostridia	o__Lachnospirales	f__Lachnospiraceae	g__Clostridium_M	s__	N/A	321	321_0	F6D9_bin.66.fa	F6D9_bin.66.fa	85.29	3.16	277
F6DB_bin.38	d__Bacteria	p__Firmicutes	c__Clostridia	o__Lachnospirales	f__Lachnospiraceae	g__Clostridium_M	s__	N/A	263	263_0	F6DB_bin.38.fa	F6DB_bin.38.fa	99.37	0	278
F6MX_bin.120	d__Bacteria	p__Firmicutes	c__Clostridia	o__Lachnospirales	f__Lachnospiraceae	g__Clostridium_M	s__Clostridium_M asparagiforme	GCF_000158075.1	562	562_0	F6MX_bin.120.fa	F6MX_bin.120.fa	57.89	1.75	278

Table S3. Taxonomic classifiation of the 2,421 detected MAGs

	Domain	Phylum	Class	Order	Family	Genus	Species	RefGenome	primary_cluster	secondary_cluster	repr_genome	genome	CheckM_completeness	CheckM_contamination	
F68E_bin.14	d__Bacteria	p__Firmicutes	c__Clostridia	o__Lachnospirales	f__Lachnospiraceae	g__Clostridium_M	s__Clostridium_M bolteae	GCF_002234575.2	261	261_1	F68G_bin.54.fa	F68E_bin.14.fa	95.96	0.56	278
F68G_bin.54	d__Bacteria	p__Firmicutes	c__Clostridia	o__Lachnospirales	f__Lachnospiraceae	g__Clostridium_M	s__Clostridium_M bolteae	GCF_002234575.2	261	261_1	F68G_bin.54.fa	F68G_bin.54.fa	98.76	0	278
F68C_bin.72	d__Bacteria	p__Firmicutes	c__Clostridia	o__Lachnospirales	f__Lachnospiraceae	g__Clostridium_M	s__Clostridium_M sp000431375	GCA_000431375.1	320	320_1	F68E_bin.10.fa	F68C_bin.72.fa	66.58	2.24	278
F68E_bin.10	d__Bacteria	p__Firmicutes	c__Clostridia	o__Lachnospirales	f__Lachnospiraceae	g__Clostridium_M	s__Clostridium_M sp000431375	GCA_000431375.1	320	320_1	F68E_bin.10.fa	F68E_bin.10.fa	99.37	1.27	278
F68U_bin.24	d__Bacteria	p__Firmicutes	c__Clostridia	o__Lachnospirales	f__Lachnospiraceae	g__Clostridium_M	s__Clostridium_M sp000431375	GCA_000431375.1	320	320_1	F68E_bin.10.fa	F68U_bin.24.fa	92.89	0.63	278
F6DA_bin.15	d__Bacteria	p__Firmicutes	c__Clostridia	o__Lachnospirales	f__Lachnospiraceae	g__Clostridium_M	s__Clostridium_M sp000431375	GCA_000431375.1	320	320_1	F68E_bin.10.fa	F6DA_bin.15.fa	91.46	1.18	278

Table S3. Taxonomic classifiation of the 2,421 detected MAGs

	Domain	Phylum	Class	Order	Family	Genus	Species	RefGenome	primary_cluster	secondary_cluster	repr_genome	genome	CheckM_completeness	CheckM_contamination	
F6D7_bin.62	d__Bacteria	p__Firmicutes	c__Clostridia	o__Lachnospirales	f__Lachnospiraceae	g__Clostridium_M	s__Clostridium_M sp000431375	GCA_000431375.1	320	320_2	F6D7_bin.62.fa	F6D7_bin.62.fa	67.89	3.51	278
F68E_bin.2	d__Bacteria	p__Firmicutes	c__Clostridia	o__Lachnospirales	f__Lachnospiraceae	g__Clostridium_Q	s__Clostridium_Q sp003024715	GCF_003024715.1	275	275_11	F68E_bin.2.fa	F68E_bin.2.fa	89.86	0.63	278
F68F_bin.75	d__Bacteria	p__Firmicutes	c__Clostridia	o__Lachnospirales	f__Lachnospiraceae	g__Clostridium_Q	s__Clostridium_Q sp003024715	GCF_003024715.1	275	275_3	F68F_bin.75.fa	F68F_bin.75.fa	62.34	0	278
F68G_bin.78	d__Bacteria	p__Firmicutes	c__Clostridia	o__Lachnospirales	f__Lachnospiraceae	g__Clostridium_Q	s__Clostridium_Q sp003024715	GCF_003024715.1	275	275_8	F68G_bin.78.fa	F68G_bin.78.fa	87.27	3.16	278
F68K_bin.61	d__Bacteria	p__Firmicutes	c__Clostridia	o__Lachnospirales	f__Lachnospiraceae	g__Clostridium_Q	s__Clostridium_Q sp003024715	GCF_003024715.1	275	275_1	F68K_bin.61.fa	F68K_bin.61.fa	97.78	1.31	278
F68N_bin.22	d__Bacteria	p__Firmicutes	c__Clostridia	o__Lachnospirales	f__Lachnospiraceae	g__Clostridium_Q	s__Clostridium_Q sp003024715	GCF_003024715.1	275	275_10	F68N_bin.22.fa	F68N_bin.22.fa	98.95	0.02	278

Table S3. Taxonomic classifiation of the 2,421 detected MAGs

	Domain	Phylum	Class	Order	Family	Genus	Species	RefGenome	primary_cluster	secondary_cluster	repr_genome	genome	CheckM_completeness	CheckM_contamination	
F68P_bin.31	d__Bacteria	p__Firmicutes	c__Clostridia	o__Lachnospirales	f__Lachnospiraceae	g__Clostridium_Q	s__Clostridium_Q sp003024715	GCF_003024715.1	275	275_12	F68P_bin.31.fa	F68P_bin.31.fa	79.45	0.63	278
F68U_bin.33	d__Bacteria	p__Firmicutes	c__Clostridia	o__Lachnospirales	f__Lachnospiraceae	g__Clostridium_Q	s__Clostridium_Q sp003024715	GCF_003024715.1	275	275_4	F68U_bin.33.fa	F68U_bin.33.fa	57.89	0	278
F6D7_bin.18	d__Bacteria	p__Firmicutes	c__Clostridia	o__Lachnospirales	f__Lachnospiraceae	g__Clostridium_Q	s__Clostridium_Q sp003024715	GCF_003024715.1	275	275_13	F6D7_bin.18.fa	F6D7_bin.18.fa	66.81	1.48	278
F6DA_bin.44	d__Bacteria	p__Firmicutes	c__Clostridia	o__Lachnospirales	f__Lachnospiraceae	g__Clostridium_Q	s__Clostridium_Q sp003024715	GCF_003024715.1	275	275_6	F6DA_bin.44.fa	F6DA_bin.44.fa	93.67	0.63	278
F6DB_bin.11	d__Bacteria	p__Firmicutes	c__Clostridia	o__Lachnospirales	f__Lachnospiraceae	g__Clostridium_Q	s__Clostridium_Q sp003024715	GCF_003024715.1	275	275_2	F6DB_bin.11.fa	F6DB_bin.11.fa	94.1	1.27	278
F6DG_bin.1	d__Bacteria	p__Firmicutes	c__Clostridia	o__Lachnospirales	f__Lachnospiraceae	g__Clostridium_Q	s__Clostridium_Q sp003024715	GCF_003024715.1	275	275_9	F6DG_bin.1.fa	F6DG_bin.1.fa	91.38	0.63	278

Table S3. Taxonomic classifiation of the 2,421 detected MAGs

	Domain	Phylum	Class	Order	Family	Genus	Species	RefGenome	primary_cluster	secondary_cluster	repr_genome	genome	CheckM_completeness	CheckM_contamination	
F6DH_bin.93	d__Bacteria	p__Firmicutes	c__Clostridia	o__Lachnospirales	f__Lachnospiraceae	g__Clostridium_Q	s__Clostridium_Q sp003024715	GCF_003024715.1	275	275_5	F6DH_bin.93.fa	F6DH_bin.93.fa	96.22	1.27	278
F6MY_bin.102	d__Bacteria	p__Firmicutes	c__Clostridia	o__Lachnospirales	f__Lachnospiraceae	g__Clostridium_Q	s__Clostridium_Q sp003024715	GCF_003024715.1	275	275_7	F6MY_bin.102.fa	F6MY_bin.102.fa	83.66	0.53	278
F68E_bin.49	d__Bacteria	p__Firmicutes	c__Clostridia	o__Lachnospirales	f__Lachnospiraceae	g__Clostridium_Q	s__Clostridium_Q symbiosum	GCF_000466485.1	329	329_1	F68G_bin.42.fa	F68E_bin.49.fa	51.96	3.51	278
F68G_bin.42	d__Bacteria	p__Firmicutes	c__Clostridia	o__Lachnospirales	f__Lachnospiraceae	g__Clostridium_Q	s__Clostridium_Q symbiosum	GCF_000466485.1	329	329_1	F68G_bin.42.fa	F68G_bin.42.fa	88.61	0	278
F68R_bin.113	d__Bacteria	p__Firmicutes	c__Clostridia	o__Lachnospirales	f__Lachnospiraceae	g__COE1	s__COE1 sp001916965	GCA_001916965.1	371	371_1	F68R_bin.113.fa	F68R_bin.113.fa	59.59	0.88	278
F6DG_bin.76	d__Bacteria	p__Firmicutes	c__Clostridia	o__Lachnospirales	f__Lachnospiraceae	g__COE1	s__COE1 sp001916965	GCA_001916965.1	371	371_2	F6DG_bin.76.fa	F6DG_bin.76.fa	95.86	1.16	278
F68K_bin.83	d__Bacteria	p__Firmicutes	c__Clostridia	o__Lachnospirales	f__Lachnospiraceae	g__Coprococcus	s__Coprococcus eutactus	GCF_000154425.1	370	370_7	F68K_bin.83.fa	F68K_bin.83.fa	99.33	1.01	278

Table S3. Taxonomic classifiation of the 2,421 detected MAGs

	Domain	Phylum	Class	Order	Family	Genus	Species	RefGenome	primary_cluster	secondary_cluster	repr_genome	genome	CheckM_completeness	CheckM_contamination	
F6D7_bin.73	d__Bacteria	p__Firmicutes	c__Clostridia	o__Lachnospirales	f__Lachnospiraceae	g__Coprococcus	s__Coprococcus eutactus	GCF_000154425.1	370	370_8	F6D7_bin.73.fa	F6D7_bin.73.fa	99.33	0	278
F6D9_bin.17	d__Bacteria	p__Firmicutes	c__Clostridia	o__Lachnospirales	f__Lachnospiraceae	g__Coprococcus	s__Coprococcus eutactus	GCF_000154425.1	370	370_9	F6D9_bin.17.fa	F6D9_bin.17.fa	99.33	0.67	278
F68V_bin.117	d__Bacteria	p__Firmicutes	c__Clostridia	o__Lachnospirales	f__Lachnospiraceae	g__Coprococcus	s__Coprococcus eutactus A	GCF_001404675.1	370	370_2	F68V_bin.117.fa	F68V_bin.117.fa	99.33	0.34	278
F6D8_bin.12	d__Bacteria	p__Firmicutes	c__Clostridia	o__Lachnospirales	f__Lachnospiraceae	g__Coprococcus	s__Coprococcus eutactus A	GCF_001404675.1	370	370_5	F6D8_bin.12.fa	F6D8_bin.12.fa	87.3	0.67	278
F68H_bin.46	d__Bacteria	p__Firmicutes	c__Clostridia	o__Lachnospirales	f__Lachnospiraceae	g__Coprococcus	s__Coprococcus eutactus A	GCF_001404675.1	370	370_3	F6DA_bin.76.fa	F68H_bin.46.fa	87.54	1.68	278
F6DA_bin.76	d__Bacteria	p__Firmicutes	c__Clostridia	o__Lachnospirales	f__Lachnospiraceae	g__Coprococcus	s__Coprococcus eutactus A	GCF_001404675.1	370	370_3	F6DA_bin.76.fa	F6DA_bin.76.fa	99.33	0	278
F6DB_bin.76	d__Bacteria	p__Firmicutes	c__Clostridia	o__Lachnospirales	f__Lachnospiraceae	g__Coprococcus	s__Coprococcus eutactus A	GCF_001404675.1	370	370_4	F6DB_bin.76.fa	F6DB_bin.76.fa	99.33	0	278

Table S3. Taxonomic classifiation of the 2,421 detected MAGs

	Domain	Phylum	Class	Order	Family	Genus	Species	RefGenome	primary_cluster	secondary_cluster	repr_genome	genome	CheckM_completeness	CheckM_contamination	
F68U_bin.25	d__Bacteria	p__Firmicutes	c__Clostridia	o__Lachnospirales	f__Lachnospiraceae	g__Coprococcus	s__Coprococcus eutactus_A	GCF_001404675.1	370	370_1	F6DH_bin.21.fa	F68U_bin.25.fa	85.07	1.74	278
F68W_bin.34	d__Bacteria	p__Firmicutes	c__Clostridia	o__Lachnospirales	f__Lachnospiraceae	g__Coprococcus	s__Coprococcus eutactus_A	GCF_001404675.1	370	370_1	F6DH_bin.21.fa	F68W_bin.34.fa	85.59	0.67	278
F6DH_bin.21	d__Bacteria	p__Firmicutes	c__Clostridia	o__Lachnospirales	f__Lachnospiraceae	g__Coprococcus	s__Coprococcus eutactus_A	GCF_001404675.1	370	370_1	F6DH_bin.21.fa	F6DH_bin.21.fa	99.33	0	278
F6MY_bin.28	d__Bacteria	p__Firmicutes	c__Clostridia	o__Lachnospirales	f__Lachnospiraceae	g__Coprococcus	s__Coprococcus eutactus_A	GCF_001404675.1	370	370_6	F6MY_bin.28.fa	F6MY_bin.28.fa	96.41	0	278
F6MX_bin.132	d__Bacteria	p__Firmicutes	c__Clostridia	o__Lachnospirales	f__Lachnospiraceae	g__Coprococcus	s__Coprococcus sp000154245	GCF_000154245.1	369	369_9	F6MX_bin.132.fa	F6MX_bin.132.fa	89.26	0	278
F68G_bin.40	d__Bacteria	p__Firmicutes	c__Clostridia	o__Lachnospirales	f__Lachnospiraceae	g__Coprococcus	s__Coprococcus sp000433075	GCA_000433075.1	373	373_2	F6DE_bin.2.fa	F68G_bin.40.fa	96.06	0.67	278
F6DE_bin.2	d__Bacteria	p__Firmicutes	c__Clostridia	o__Lachnospirales	f__Lachnospiraceae	g__Coprococcus	s__Coprococcus sp000433075	GCA_000433075.1	373	373_2	F6DE_bin.2.fa	F6DE_bin.2.fa	99.33	0	278

Table S3. Taxonomic classifiation of the 2,421 detected MAGs

	Domain	Phylum	Class	Order	Family	Genus	Species	RefGenome	primary_cluster	secondary_cluster	repr_genome	genome	CheckM_completeness	CheckM_contamination	
F68W_bin.103	d__Bacteria	p__Firmicutes	c__Clostridia	o__Lachnospirales	f__Lachnospiraceae	g__Coprococcus	s__Coprococcus sp000433075	GCA_000433075.1	373	373_1	F6DH_bin.122.fa	F68W_bin.103.fa	94.3	2.01	278
F6DH_bin.122	d__Bacteria	p__Firmicutes	c__Clostridia	o__Lachnospirales	f__Lachnospiraceae	g__Coprococcus	s__Coprococcus sp000433075	GCA_000433075.1	373	373_1	F6DH_bin.122.fa	F6DH_bin.122.fa	99.33	0	278
F68C_bin.44	d__Bacteria	p__Firmicutes	c__Clostridia	o__Lachnospirales	f__Lachnospiraceae	g__Coprococcus	s__Coprococcus sp900066115	GCA_900066115.1	369	369_2	F68C_bin.44.fa	F68C_bin.44.fa	99.33	0	278
F68K_bin.98	d__Bacteria	p__Firmicutes	c__Clostridia	o__Lachnospirales	f__Lachnospiraceae	g__Coprococcus	s__Coprococcus sp900066115	GCA_900066115.1	369	369_6	F68K_bin.98.fa	F68K_bin.98.fa	95.3	0	278
F68N_bin.21	d__Bacteria	p__Firmicutes	c__Clostridia	o__Lachnospirales	f__Lachnospiraceae	g__Coprococcus	s__Coprococcus sp900066115	GCA_900066115.1	369	369_4	F68N_bin.21.fa	F68N_bin.21.fa	99.33	0	278
F68V_bin.58	d__Bacteria	p__Firmicutes	c__Clostridia	o__Lachnospirales	f__Lachnospiraceae	g__Coprococcus	s__Coprococcus sp900066115	GCA_900066115.1	369	369_5	F68V_bin.58.fa	F68V_bin.58.fa	99.33	0.22	278
F6D7_bin.97	d__Bacteria	p__Firmicutes	c__Clostridia	o__Lachnospirales	f__Lachnospiraceae	g__Coprococcus	s__Coprococcus sp900066115	GCA_900066115.1	369	369_7	F6D7_bin.97.fa	F6D7_bin.97.fa	90.19	2.24	278

Table S3. Taxonomic classifiation of the 2,421 detected MAGs

	Domain	Phylum	Class	Order	Family	Genus	Species	RefGenome	primary_cluster	secondary_cluster	repr_genome	genome	CheckM_completeness	CheckM_contamination	
F68R_bin.26	d__Bacteria	p__Firmicutes	c__Clostridia	o__Lachnospirales	f__Lachnospiraceae	g__Coprococcus	s__Coprococcus sp900066115	GCA_900066115.1	369	369_1	F6D8_bin.58.fa	F68R_bin.26.fa	80.28	0.67	278
F6D8_bin.58	d__Bacteria	p__Firmicutes	c__Clostridia	o__Lachnospirales	f__Lachnospiraceae	g__Coprococcus	s__Coprococcus sp900066115	GCA_900066115.1	369	369_1	F6D8_bin.58.fa	F6D8_bin.58.fa	93.96	0.84	278
F6D9_bin.11	d__Bacteria	p__Firmicutes	c__Clostridia	o__Lachnospirales	f__Lachnospiraceae	g__Coprococcus	s__Coprococcus sp900066115	GCA_900066115.1	369	369_3	F6D9_bin.11.fa	F6D9_bin.11.fa	91.07	2.68	278
F6MY_bin.13	d__Bacteria	p__Firmicutes	c__Clostridia	o__Lachnospirales	f__Lachnospiraceae	g__Coprococcus	s__Coprococcus sp900066115	GCA_900066115.1	369	369_8	F6MY_bin.13.fa	F6MY_bin.13.fa	75.89	3.44	278
F68J_bin.34	d__Bacteria	p__Firmicutes	c__Clostridia	o__Lachnospirales	f__Lachnospiraceae	g__Coprococcus_A	s__Coprococcus_A catus	GCA_000210555.1	353	353_2	F68J_bin.34.fa	F68J_bin.34.fa	97.53	2.01	278
F68M_bin.59	d__Bacteria	p__Firmicutes	c__Clostridia	o__Lachnospirales	f__Lachnospiraceae	g__Coprococcus_A	s__Coprococcus_A catus	GCA_000210555.1	353	353_5	F68M_bin.59.fa	F68M_bin.59.fa	58.86	1.16	278
F68T_bin.12	d__Bacteria	p__Firmicutes	c__Clostridia	o__Lachnospirales	f__Lachnospiraceae	g__Coprococcus_A	s__Coprococcus_A catus	GCA_000210555.1	353	353_6	F68T_bin.12.fa	F68T_bin.12.fa	82.59	2.18	278

Table S3. Taxonomic classifiation of the 2,421 detected MAGs

	Domain	Phylum	Class	Order	Family	Genus	Species	RefGenome	primary_cluster	secondary_cluster	repr_genome	genome	CheckM_completeness	CheckM_contamination	
F68U_bin.61	d__Bacteria	p__Firmicutes	c__Clostridia	o__Lachnospirales	f__Lachnospiraceae	g__Coprococcus_A	s__Coprococcus_A catus	GCA_000210555.1	353	353_1	F68U_bin.61.fa	F68U_bin.61.fa	90.84	2.01	278
F6DH_bin.47	d__Bacteria	p__Firmicutes	c__Clostridia	o__Lachnospirales	f__Lachnospiraceae	g__Coprococcus_A	s__Coprococcus_A catus	GCA_000210555.1	353	353_1	F68U_bin.61.fa	F6DH_bin.47.fa	88.88	1.96	278
F6D7_bin.82	d__Bacteria	p__Firmicutes	c__Clostridia	o__Lachnospirales	f__Lachnospiraceae	g__Coprococcus_A	s__Coprococcus_A catus	GCA_000210555.1	353	353_3	F6D7_bin.82.fa	F6D7_bin.82.fa	97.65	1.34	278
F68C_bin.113	d__Bacteria	p__Firmicutes	c__Clostridia	o__Lachnospirales	f__Lachnospiraceae	g__Coprococcus_A	s__Coprococcus_A catus	GCA_000210555.1	353	353_4	F6DB_bin.74.fa	F68C_bin.113.fa	68.56	3.51	278
F6DB_bin.74	d__Bacteria	p__Firmicutes	c__Clostridia	o__Lachnospirales	f__Lachnospiraceae	g__Coprococcus_A	s__Coprococcus_A catus	GCA_000210555.1	353	353_4	F6DB_bin.74.fa	F6DB_bin.74.fa	93.96	2.01	278
F68P_bin.42	d__Bacteria	p__Firmicutes	c__Clostridia	o__Lachnospirales	f__Lachnospiraceae	g__Coprococcus_B	s__	N/A	304	304_1	F68P_bin.42.fa	F68P_bin.42.fa	85.06	1.22	279
F6DG_bin.27	d__Bacteria	p__Firmicutes	c__Clostridia	o__Lachnospirales	f__Lachnospiraceae	g__Coprococcus_B	s__	N/A	304	304_2	F6DG_bin.27.fa	F6DG_bin.27.fa	77.19	0	280
F68C_bin.8	d__Bacteria	p__Firmicutes	c__Clostridia	o__Lachnospirales	f__Lachnospiraceae	g__Coprococcus_B	s__Coprococcus_B comes	GCF_000155875.1	287	287_1	F68C_bin.8.fa	F68C_bin.8.fa	69.7	1.17	280

Table S3. Taxonomic classifiation of the 2,421 detected MAGs

	Domain	Phylum	Class	Order	Family	Genus	Species	RefGenome	primary_cluster	secondary_cluster	repr_genome	genome	CheckM_completeness	CheckM_contamination	
F68K_bin.109	d__Bacteria	p__Firmicutes	c__Clostridia	o__Lachnospirales	f__Lachnospiraceae	g__Coprococcus_B	s__Coprococcus_B comes	GCF_000155875.1	287	287_5	F68K_bin.109.fa	F68K_bin.109.fa	63.75	1.56	280
F68U_bin.71	d__Bacteria	p__Firmicutes	c__Clostridia	o__Lachnospirales	f__Lachnospiraceae	g__Coprococcus_B	s__Coprococcus_B comes	GCF_000155875.1	287	287_3	F68U_bin.71.fa	F68U_bin.71.fa	92.98	1.75	280
F6DA_bin.38	d__Bacteria	p__Firmicutes	c__Clostridia	o__Lachnospirales	f__Lachnospiraceae	g__Coprococcus_B	s__Coprococcus_B comes	GCF_000155875.1	287	287_2	F6DA_bin.38.fa	F6DA_bin.38.fa	86	2.63	280
F6DG_bin.73	d__Bacteria	p__Firmicutes	c__Clostridia	o__Lachnospirales	f__Lachnospiraceae	g__Coprococcus_B	s__Coprococcus_B comes	GCF_000155875.1	287	287_6	F6DG_bin.73.fa	F6DG_bin.73.fa	90.06	0.58	280
F6DH_bin.25	d__Bacteria	p__Firmicutes	c__Clostridia	o__Lachnospirales	f__Lachnospiraceae	g__Coprococcus_B	s__Coprococcus_B comes	GCF_000155875.1	287	287_4	F6DH_bin.25.fa	F6DH_bin.25.fa	91.08	3.17	280
F68E_bin.39	d__Bacteria	p__Firmicutes	c__Clostridia	o__Lachnospirales	f__Lachnospiraceae	g__Dorea	s__	N/A	293	293_1	F68E_bin.39.fa	F68E_bin.39.fa	95.32	0.58	281
F6DB_bin.86	d__Bacteria	p__Firmicutes	c__Clostridia	o__Lachnospirales	f__Lachnospiraceae	g__Dorea	s__	N/A	293	293_1	F68E_bin.39.fa	F6DB_bin.86.fa	90.35	1.75	282
F68C_bin.86	d__Bacteria	p__Firmicutes	c__Clostridia	o__Lachnospirales	f__Lachnospiraceae	g__Dorea	s__Dorea formicigenerans	GCF_000169235.1	288	288_4	F68C_bin.86.fa	F68C_bin.86.fa	76.46	4.53	282

Table S3. Taxonomic classifiation of the 2,421 detected MAGs

	Domain	Phylum	Class	Order	Family	Genus	Species	RefGenome	primary_cluster	secondary_cluster	repr_genome	genome	CheckM_completeness	CheckM_contamination	
F68E_bin.6	d__Bacteria	p__Firmicutes	c__Clostridia	o__Lachnospirales	f__Lachnospiraceae	g__Dorea	s__Dorea formicigenerans	GCF_000169235.1	288	288_6	F68E_bin.6.fa	F68E_bin.6.fa	97.95	0.88	282
F68J_bin.9	d__Bacteria	p__Firmicutes	c__Clostridia	o__Lachnospirales	f__Lachnospiraceae	g__Dorea	s__Dorea formicigenerans	GCF_000169235.1	288	288_5	F68J_bin.9.fa	F68J_bin.9.fa	99.24	0	282
F68V_bin.16	d__Bacteria	p__Firmicutes	c__Clostridia	o__Lachnospirales	f__Lachnospiraceae	g__Dorea	s__Dorea formicigenerans	GCF_000169235.1	288	288_1	F68V_bin.16.fa	F68V_bin.16.fa	81.98	3.69	282
F68W_bin.44	d__Bacteria	p__Firmicutes	c__Clostridia	o__Lachnospirales	f__Lachnospiraceae	g__Dorea	s__Dorea formicigenerans	GCF_000169235.1	288	288_2	F68W_bin.44.fa	F68W_bin.44.fa	83.9	4.99	282
F68T_bin.43	d__Bacteria	p__Firmicutes	c__Clostridia	o__Lachnospirales	f__Lachnospiraceae	g__Dorea	s__Dorea formicigenerans	GCF_000169235.1	288	288_3	F6MY_bin.82.fa	F68T_bin.43.fa	59.42	2.34	282
F6MY_bin.82	d__Bacteria	p__Firmicutes	c__Clostridia	o__Lachnospirales	f__Lachnospiraceae	g__Dorea	s__Dorea formicigenerans	GCF_000169235.1	288	288_3	F6MY_bin.82.fa	F6MY_bin.82.fa	71.83	1.89	282

Table S3. Taxonomic classifiation of the 2,421 detected MAGs

	Domain	Phylum	Class	Order	Family	Genus	Species	RefGenome	primary_cluster	secondary_cluster	repr_genome	genome	CheckM_completeness	CheckM_contamination	
F68J_bin.40	d__Bacteria	p__Firmicutes	c__Clostridia	o__Lachnospirales	f__Lachnospiraceae	g__Dorea	s__Dorea longicatena	GCF_000154065.1	289	289_10	F68J_bin.40.fa	F68J_bin.40.fa	54.9	0	282
F68U_bin.52	d__Bacteria	p__Firmicutes	c__Clostridia	o__Lachnospirales	f__Lachnospiraceae	g__Dorea	s__Dorea longicatena	GCF_000154065.1	289	289_9	F68U_bin.52.fa	F68U_bin.52.fa	59.65	0	282
F68W_bin.88	d__Bacteria	p__Firmicutes	c__Clostridia	o__Lachnospirales	f__Lachnospiraceae	g__Dorea	s__Dorea longicatena	GCF_000154065.1	289	289_8	F68W_bin.88.fa	F68W_bin.88.fa	90.82	2.34	282
F6DH_bin.73	d__Bacteria	p__Firmicutes	c__Clostridia	o__Lachnospirales	f__Lachnospiraceae	g__Dorea	s__Dorea longicatena	GCF_000154065.1	289	289_8	F68W_bin.88.fa	F6DH_bin.73.fa	56.52	0	282
F68C_bin.34	d__Bacteria	p__Firmicutes	c__Clostridia	o__Lachnospirales	f__Lachnospiraceae	g__Dorea	s__Dorea longicatena_B	GCF_001404875.1	289	289_3	F68C_bin.34.fa	F68C_bin.34.fa	71.64	4.97	282
F68K_bin.29	d__Bacteria	p__Firmicutes	c__Clostridia	o__Lachnospirales	f__Lachnospiraceae	g__Dorea	s__Dorea longicatena_B	GCF_001404875.1	289	289_7	F68K_bin.29.fa	F68K_bin.29.fa	53.43	0	282
F68T_bin.92	d__Bacteria	p__Firmicutes	c__Clostridia	o__Lachnospirales	f__Lachnospiraceae	g__Dorea	s__Dorea longicatena_B	GCF_001404875.1	289	289_1	F68T_bin.92.fa	F68T_bin.92.fa	76.67	0	282

Table S3. Taxonomic classifiation of the 2,421 detected MAGs

	Domain	Phylum	Class	Order	Family	Genus	Species	RefGenome	primary_cluster	secondary_cluster	repr_genome	genome	CheckM_completeness	CheckM_contamination	
F6DA_bin.66	d__Bacteria	p__Firmicutes	c__Clostridia	o__Lachnospirales	f__Lachnospiraceae	g__Dorea	s__Dorea longicatena_B	GCF_001404875.1	289	289_2	F6DA_bin.66.fa	F6DA_bin.66.fa	90.77	0.97	282
F6DB_bin.40	d__Bacteria	p__Firmicutes	c__Clostridia	o__Lachnospirales	f__Lachnospiraceae	g__Dorea	s__Dorea longicatena_B	GCF_001404875.1	289	289_5	F6DB_bin.40.fa	F6DB_bin.40.fa	89.47	0	282
F6DE_bin.16	d__Bacteria	p__Firmicutes	c__Clostridia	o__Lachnospirales	f__Lachnospiraceae	g__Dorea	s__Dorea longicatena_B	GCF_001404875.1	289	289_4	F6DE_bin.16.fa	F6DE_bin.16.fa	92.01	3.94	282
F6DG_bin.56	d__Bacteria	p__Firmicutes	c__Clostridia	o__Lachnospirales	f__Lachnospiraceae	g__Dorea	s__Dorea longicatena_B	GCF_001404875.1	289	289_6	F6DG_bin.56.fa	F6DG_bin.56.fa	93.62	0	282
F68D_bin.66	d__Bacteria	p__Firmicutes	c__Clostridia	o__Lachnospirales	f__Lachnospiraceae	g__Dorea	s__Dorea sp900066555	GCA_900066555.1	291	291_1	F68D_bin.66.fa	F68D_bin.66.fa	72.98	4.09	282
F6DG_bin.6	d__Bacteria	p__Firmicutes	c__Clostridia	o__Lachnospirales	f__Lachnospiraceae	g__Dorea	s__Dorea sp900066555	GCA_900066555.1	291	291_2	F6DG_bin.6.fa	F6DG_bin.6.fa	57.71	0	282
F68V_bin.19	d__Bacteria	p__Firmicutes	c__Clostridia	o__Lachnospirales	f__Lachnospiraceae	g__Eisenbergiella	s__	N/A	348	348_0	F68V_bin.19.fa	F68V_bin.19.fa	61.97	3.51	283

Table S3. Taxonomic classifiation of the 2,421 detected MAGs

	Domain	Phylum	Class	Order	Family	Genus	Species	RefGenome	primary_cluster	secondary_cluster	repr_genome	genome	CheckM_completeness	CheckM_contamination	
F68M_bin.80	d__Bacteria	p__Firmicutes	c__Clostridia	o__Lachnospirales	f__Lachnospiraceae	g__Eisenbergiella	s__Eisenbergiella massiliensis	GCF_900243045.1	346	346_1	F68V_bin.54.fa	F68M_bin.80.fa	71.64	0.88	283
F68V_bin.54	d__Bacteria	p__Firmicutes	c__Clostridia	o__Lachnospirales	f__Lachnospiraceae	g__Eisenbergiella	s__Eisenbergiella massiliensis	GCF_900243045.1	346	346_1	F68V_bin.54.fa	F68V_bin.54.fa	93.46	2.2	283
F68K_bin.76	d__Bacteria	p__Firmicutes	c__Clostridia	o__Lachnospirales	f__Lachnospiraceae	g__Eisenbergiella	s__Eisenbergiella sp900066775	GCA_900066775.1	454	454_1	F68K_bin.76.fa	F68K_bin.76.fa	83.96	2.07	283
F6DG_bin.74	d__Bacteria	p__Firmicutes	c__Clostridia	o__Lachnospirales	f__Lachnospiraceae	g__Eisenbergiella	s__Eisenbergiella sp900066775	GCA_900066775.1	454	454_2	F6DG_bin.74.fa	F6DG_bin.74.fa	78.87	2.01	283
F68D_bin.103	d__Bacteria	p__Firmicutes	c__Clostridia	o__Lachnospirales	f__Lachnospiraceae	g__Eisenbergiella	s__Eisenbergiella tayi	GCF_001881565.1	347	347_1	F68D_bin.103.fa	F68D_bin.103.fa	97.54	3.74	283
F68M_bin.28	d__Bacteria	p__Firmicutes	c__Clostridia	o__Lachnospirales	f__Lachnospiraceae	g__Eisenbergiella	s__Eisenbergiella tayi	GCF_001881565.1	347	347_1	F68D_bin.103.fa	F68M_bin.28.fa	52.63	0	283
F68R_bin.67	d__Bacteria	p__Firmicutes	c__Clostridia	o__Lachnospirales	f__Lachnospiraceae	g__Eubacterium_E	s__	N/A	323	323_2	F68R_bin.67.fa	F68R_bin.67.fa	56.41	1.5	284

Table S3. Taxonomic classifiation of the 2,421 detected MAGs

	Domain	Phylum	Class	Order	Family	Genus	Species	RefGenome	primary_cluster	secondary_cluster	repr_genome	genome	CheckM_completeness	CheckM_contamination	
F6DG_bin.60	d__Bacteria	p__Firmicutes	c__Clostridia	o__Lachnospirales	f__Lachnospiraceae	g__Eubacterium_E	s__	N/A	323	323_3	F6DG_bin.60.fa	F6DG_bin.60.fa	65	4.92	285
F68F_bin.85	d__Bacteria	p__Firmicutes	c__Clostridia	o__Lachnospirales	f__Lachnospiraceae	g__Eubacterium_E	s__Eubacterium_E hallii	GCF_000173975.1	322	322_1	F68F_bin.85.fa	F68F_bin.85.fa	93.96	0.89	285
F68K_bin.107	d__Bacteria	p__Firmicutes	c__Clostridia	o__Lachnospirales	f__Lachnospiraceae	g__Eubacterium_E	s__Eubacterium_E hallii	GCF_000173975.1	322	322_2	F68K_bin.107.fa	F68K_bin.107.fa	69.8	4.03	285
F68W_bin.36	d__Bacteria	p__Firmicutes	c__Clostridia	o__Lachnospirales	f__Lachnospiraceae	g__Eubacterium_E	s__Eubacterium_E hallii_A	GCF_900209925.1	323	323_1	F68W_bin.36.fa	F68W_bin.36.fa	94.63	2.03	285
F6DH_bin.45	d__Bacteria	p__Firmicutes	c__Clostridia	o__Lachnospirales	f__Lachnospiraceae	g__Eubacterium_E	s__Eubacterium_E hallii_A	GCF_900209925.1	323	323_1	F68W_bin.36.fa	F6DH_bin.45.fa	89.26	4.7	285
F68T_bin.84	d__Bacteria	p__Firmicutes	c__Clostridia	o__Lachnospirales	f__Lachnospiraceae	g__Eubacterium_F	s__Eubacterium_F sp003491505	GCA_003491505.1	467	467_0	F68T_bin.84.fa	F68T_bin.84.fa	95.39	0.17	285
F68V_bin.52	d__Bacteria	p__Firmicutes	c__Clostridia	o__Lachnospirales	f__Lachnospiraceae	g__Eubacterium_G	s__Eubacterium_G sp000434315	GCA_000434315.1	266	266_1	F68V_bin.52.fa	F68V_bin.52.fa	90.6	0	285

Table S3. Taxonomic classifiation of the 2,421 detected MAGs

	Domain	Phylum	Class	Order	Family	Genus	Species	RefGenome	primary_cluster	secondary_cluster	repr_genome	genome	CheckM_completeness	CheckM_contamination	
F6MY_bin.97	d__Bacteria	p__Firmicutes	c__Clostridia	o__Lachnospirales	f__Lachnospiraceae	g__Eubacterium_G	s__Eubacterium_G sp000434315	GCA_000434315.1	266	266_1	F68V_bin.52.fa	F6MY_bin.97.fa	62.28	3.38	285
F6DB_bin.6	d__Bacteria	p__Firmicutes	c__Clostridia	o__Lachnospirales	f__Lachnospiraceae	g__Eubacterium_G	s__Eubacterium_G sp000434315	GCA_000434315.1	266	266_2	F6DB_bin.6.fa	F6DB_bin.6.fa	99.33	1.34	285
F6DE_bin.12	d__Bacteria	p__Firmicutes	c__Clostridia	o__Lachnospirales	f__Lachnospiraceae	g__Eubacterium_G	s__Eubacterium_G sp000434315	GCA_000434315.1	266	266_3	F6DE_bin.12.fa	F6DE_bin.12.fa	86.09	1.81	285
F68V_bin.108	d__Bacteria	p__Firmicutes	c__Clostridia	o__Lachnospirales	f__Lachnospiraceae	g__Eubacterium_G	s__Eubacterium_G sp000435815	GCA_000435815.1	267	267_0	F68V_bin.108.fa	F68V_bin.108.fa	98.99	1.79	285
F68K_bin.89	d__Bacteria	p__Firmicutes	c__Clostridia	o__Lachnospirales	f__Lachnospiraceae	g__Eubacterium_G	s__Eubacterium_G ventriosum	GCF_000153885.1	265	265_2	F68K_bin.89.fa	F68K_bin.89.fa	79.81	0.67	285
F68V_bin.39	d__Bacteria	p__Firmicutes	c__Clostridia	o__Lachnospirales	f__Lachnospiraceae	g__Eubacterium_G	s__Eubacterium_G ventriosum	GCF_000153885.1	265	265_1	F6D7_bin.15.fa	F68V_bin.39.fa	88.79	0.02	285

Table S3. Taxonomic classifiation of the 2,421 detected MAGs

	Domain	Phylum	Class	Order	Family	Genus	Species	RefGenome	primary_cluster	secondary_cluster	repr_genome	genome	CheckM_completeness	CheckM_contamination	
F6D7_bin.15	d__Bacteria	p__Firmicutes	c__Clostridia	o__Lachnospirales	f__Lachnospiraceae	g__Eubacterium_G	s__Eubacterium_G ventriosum	GCF_000153885.1	265	265_1	F6D7_bin.15.fa	F6D7_bin.15.fa	92.38	0.11	285
F68M_bin.26	d__Bacteria	p__Firmicutes	c__Clostridia	o__Lachnospirales	f__Lachnospiraceae	g__Eubacterium_H	s__	N/A	302	302_0	F68M_bin.26.fa	F68M_bin.26.fa	96.8	0	286
F6DH_bin.109	d__Bacteria	p__Firmicutes	c__Clostridia	o__Lachnospirales	f__Lachnospiraceae	g__Eubacterium_I	s__Eubacterium_I ramulus_A	GCF_003122485.1	281	281_0	F6DH_bin.109.fa	F6DH_bin.109.fa	82.33	1.14	286
F6DH_bin.80	d__Bacteria	p__Firmicutes	c__Clostridia	o__Lachnospirales	f__Lachnospiraceae	g__Faecalicatena	s__	N/A	295	295_2	F6DH_bin.80.fa	F6DH_bin.80.fa	54.37	2.46	287
F68C_bin.30	d__Bacteria	p__Firmicutes	c__Clostridia	o__Lachnospirales	f__Lachnospiraceae	g__Faecalicatena	s__Faecalicatena faecis	GCF_001312505.1	297	297_1	F68C_bin.30.fa	F68C_bin.30.fa	73.22	2.64	287
F68D_bin.47	d__Bacteria	p__Firmicutes	c__Clostridia	o__Lachnospirales	f__Lachnospiraceae	g__Faecalicatena	s__Faecalicatena faecis	GCF_001312505.1	297	297_5	F68D_bin.47.fa	F68D_bin.47.fa	99.12	0.58	287
F68N_bin.20	d__Bacteria	p__Firmicutes	c__Clostridia	o__Lachnospirales	f__Lachnospiraceae	g__Faecalicatena	s__Faecalicatena faecis	GCF_001312505.1	297	297_3	F68N_bin.20.fa	F68N_bin.20.fa	82.46	0.29	287

Table S3. Taxonomic classifiation of the 2,421 detected MAGs

	Domain	Phylum	Class	Order	Family	Genus	Species	RefGenome	primary_cluster	secondary_cluster	repr_genome	genome	CheckM_completeness	CheckM_contamination	
F6DA_bin.42	d__Bacteria	p__Firmicutes	c__Clostridia	o__Lachnospirales	f__Lachnospiraceae	g__Faecalicatena	s__Faecalicatena faecis	GCF_001312505.1	297	297_2	F6DA_bin.42.fa	F6DA_bin.42.fa	58.91	0	287
F6MX_bin.54	d__Bacteria	p__Firmicutes	c__Clostridia	o__Lachnospirales	f__Lachnospiraceae	g__Faecalicatena	s__Faecalicatena faecis	GCF_001312505.1	297	297_4	F6MX_bin.54.fa	F6MX_bin.54.fa	93.86	0.29	287
F6MY_bin.122	d__Bacteria	p__Firmicutes	c__Clostridia	o__Lachnospirales	f__Lachnospiraceae	g__Faecalicatena	s__Faecalicatena faecis	GCF_001312505.1	297	297_6	F6MY_bin.122.fa	F6MY_bin.122.fa	58.62	0	287
F68E_bin.8	d__Bacteria	p__Firmicutes	c__Clostridia	o__Lachnospirales	f__Lachnospiraceae	g__Faecalicatena	s__Faecalicatena gnavus	GCF_002959615.1	294	294_1	F68J_bin.7.fa	F68E_bin.8.fa	75.24	1.16	287
F68H_bin.53	d__Bacteria	p__Firmicutes	c__Clostridia	o__Lachnospirales	f__Lachnospiraceae	g__Faecalicatena	s__Faecalicatena gnavus	GCF_002959615.1	294	294_1	F68J_bin.7.fa	F68H_bin.53.fa	82.75	2.92	287
F68J_bin.7	d__Bacteria	p__Firmicutes	c__Clostridia	o__Lachnospirales	f__Lachnospiraceae	g__Faecalicatena	s__Faecalicatena gnavus	GCF_002959615.1	294	294_1	F68J_bin.7.fa	F68J_bin.7.fa	99.42	0	287
F68P_bin.49	d__Bacteria	p__Firmicutes	c__Clostridia	o__Lachnospirales	f__Lachnospiraceae	g__Faecalicatena	s__Faecalicatena gnavus	GCF_002959615.1	294	294_1	F68J_bin.7.fa	F68P_bin.49.fa	88.06	0.58	287

Table S3. Taxonomic classifiation of the 2,421 detected MAGs

	Domain	Phylum	Class	Order	Family	Genus	Species	RefGenome	primary_cluster	secondary_cluster	repr_genome	genome	CheckM_completeness	CheckM_contamination	
F6D9_bin.20	d__Bacteria	p__Firmicutes	c__Clostridia	o__Lachnospirales	f__Lachnospiraceae	g__Faecalicatena	s__Faecalicatena gnavus	GCF_002959615.1	294	294_1	F68J_bin.7.fa	F6D9_bin.20.fa	51.72	0	287
F6DG_bin.99	d__Bacteria	p__Firmicutes	c__Clostridia	o__Lachnospirales	f__Lachnospiraceae	g__Faecalicatena	s__Faecalicatena gnavus	GCF_002959615.1	294	294_1	F68J_bin.7.fa	F6DG_bin.99.fa	75.57	1.38	287
F6MY_bin.74	d__Bacteria	p__Firmicutes	c__Clostridia	o__Lachnospirales	f__Lachnospiraceae	g__Faecalicatena	s__Faecalicatena gnavus	GCF_002959615.1	294	294_1	F68J_bin.7.fa	F6MY_bin.74.fa	76.02	0	287
F68M_bin.21	d__Bacteria	p__Firmicutes	c__Clostridia	o__Lachnospirales	f__Lachnospiraceae	g__Faecalicatena	s__Faecalicatena gnavus	GCF_002959615.1	294	294_2	F6D8_bin.49.fa	F68M_bin.21.fa	88.53	3.91	287
F68V_bin.88	d__Bacteria	p__Firmicutes	c__Clostridia	o__Lachnospirales	f__Lachnospiraceae	g__Faecalicatena	s__Faecalicatena gnavus	GCF_002959615.1	294	294_2	F6D8_bin.49.fa	F68V_bin.88.fa	60.34	0	287
F6D8_bin.49	d__Bacteria	p__Firmicutes	c__Clostridia	o__Lachnospirales	f__Lachnospiraceae	g__Faecalicatena	s__Faecalicatena gnavus	GCF_002959615.1	294	294_2	F6D8_bin.49.fa	F6D8_bin.49.fa	96.49	4	287
F6DE_bin.26	d__Bacteria	p__Firmicutes	c__Clostridia	o__Lachnospirales	f__Lachnospiraceae	g__Faecalicatena	s__Faecalicatena gnavus	GCF_002959615.1	294	294_2	F6D8_bin.49.fa	F6DE_bin.26.fa	51.72	0	287

Table S3. Taxonomic classifiation of the 2,421 detected MAGs

	Domain	Phylum	Class	Order	Family	Genus	Species	RefGenome	primary_cluster	secondary_cluster	repr_genome	genome	CheckM_completeness	CheckM_contamination	
F68T_bin.97	d__Bacteria	p__Firmicutes	c__Clostridia	o__Lachnospirales	f__Lachnospiraceae	g__Faecalicatena	s__Faecalicatena lactaris	GCF_000155205.1	296	296_1	F68V_bin.93.fa	F68T_bin.97.fa	84.8	0	287
F68V_bin.93	d__Bacteria	p__Firmicutes	c__Clostridia	o__Lachnospirales	f__Lachnospiraceae	g__Faecalicatena	s__Faecalicatena lactaris	GCF_000155205.1	296	296_1	F68V_bin.93.fa	F68V_bin.93.fa	91.34	0.58	287
F6DG_bin.46	d__Bacteria	p__Firmicutes	c__Clostridia	o__Lachnospirales	f__Lachnospiraceae	g__Faecalicatena	s__Faecalicatena sp900066545	GCA_900066545.1	295	295_1	F6DG_bin.46.fa	F6DG_bin.46.fa	61.82	0.82	287
F68C_bin.79	d__Bacteria	p__Firmicutes	c__Clostridia	o__Lachnospirales	f__Lachnospiraceae	g__Faecalicatena	s__Faecalicatena torques	GCF_000153925.1	292	292_1	F68F_bin.100.fa	F68C_bin.79.fa	90.04	0.2	287
F68D_bin.8	d__Bacteria	p__Firmicutes	c__Clostridia	o__Lachnospirales	f__Lachnospiraceae	g__Faecalicatena	s__Faecalicatena torques	GCF_000153925.1	292	292_1	F68F_bin.100.fa	F68D_bin.8.fa	94.15	0	287
F68F_bin.100	d__Bacteria	p__Firmicutes	c__Clostridia	o__Lachnospirales	f__Lachnospiraceae	g__Faecalicatena	s__Faecalicatena torques	GCF_000153925.1	292	292_1	F68F_bin.100.fa	F68F_bin.100.fa	98.83	0	287
F68H_bin.56	d__Bacteria	p__Firmicutes	c__Clostridia	o__Lachnospirales	f__Lachnospiraceae	g__Faecalicatena	s__Faecalicatena torques	GCF_000153925.1	292	292_1	F68F_bin.100.fa	F68H_bin.56.fa	97.58	0	287

Table S3. Taxonomic classifiation of the 2,421 detected MAGs

	Domain	Phylum	Class	Order	Family	Genus	Species	RefGenome	primary_cluster	secondary_cluster	repr_genome	genome	CheckM_completeness	CheckM_contamination	
F68K_bin.52	d__Bacteria	p__Firmicutes	c__Clostridia	o__Lachnospirales	f__Lachnospiraceae	g__Faecalicatena	s__Faecalicatena torques	GCF_000153925.1	292	292_1	F68F_bin.100.fa	F68K_bin.52.fa	97	0.58	287
F68M_bin.5	d__Bacteria	p__Firmicutes	c__Clostridia	o__Lachnospirales	f__Lachnospiraceae	g__Faecalicatena	s__Faecalicatena torques	GCF_000153925.1	292	292_1	F68F_bin.100.fa	F68M_bin.5.fa	52.37	0.12	287
F68N_bin.24	d__Bacteria	p__Firmicutes	c__Clostridia	o__Lachnospirales	f__Lachnospiraceae	g__Faecalicatena	s__Faecalicatena torques	GCF_000153925.1	292	292_1	F68F_bin.100.fa	F68N_bin.24.fa	89.41	1.95	287
F68R_bin.23	d__Bacteria	p__Firmicutes	c__Clostridia	o__Lachnospirales	f__Lachnospiraceae	g__Faecalicatena	s__Faecalicatena torques	GCF_000153925.1	292	292_1	F68F_bin.100.fa	F68R_bin.23.fa	95.91	0	287
F68T_bin.28	d__Bacteria	p__Firmicutes	c__Clostridia	o__Lachnospirales	f__Lachnospiraceae	g__Faecalicatena	s__Faecalicatena torques	GCF_000153925.1	292	292_1	F68F_bin.100.fa	F68T_bin.28.fa	96.2	0.68	287
F6DB_bin.110	d__Bacteria	p__Firmicutes	c__Clostridia	o__Lachnospirales	f__Lachnospiraceae	g__Faecalicatena	s__Faecalicatena torques	GCF_000153925.1	292	292_1	F68F_bin.100.fa	F6DB_bin.110.fa	94.15	0	287
F6DG_bin.9	d__Bacteria	p__Firmicutes	c__Clostridia	o__Lachnospirales	f__Lachnospiraceae	g__Faecalicatena	s__Faecalicatena torques	GCF_000153925.1	292	292_1	F68F_bin.100.fa	F6DG_bin.9.fa	98.45	0	287

Table S3. Taxonomic classifiation of the 2,421 detected MAGs

	Domain	Phylum	Class	Order	Family	Genus	Species	RefGenome	primary_cluster	secondary_cluster	repr_genome	genome	CheckM_completeness	CheckM_contamination	
F6MY_bin.32	d__Bacteria	p__Firmicutes	c__Clostridia	o__Lachnospirales	f__Lachnospiraceae	g__Faecalicatena	s__Faecalicatena torques	GCF_000153925.1	292	292_1	F68F_bin.100.fa	F6MY_bin.32.fa	97.66	1.56	287
F68C_bin.7	d__Bacteria	p__Firmicutes	c__Clostridia	o__Lachnospirales	f__Lachnospiraceae	g__Fusicatenibacter	s__Fusicatenibacter saccharivorans	GCF_001405555.1	270	270_2	F68C_bin.7.fa	F68C_bin.7.fa	71.73	0	287
F68F_bin.34	d__Bacteria	p__Firmicutes	c__Clostridia	o__Lachnospirales	f__Lachnospiraceae	g__Fusicatenibacter	s__Fusicatenibacter saccharivorans	GCF_001405555.1	270	270_7	F68F_bin.34.fa	F68F_bin.34.fa	96.39	0.63	287
F68R_bin.107	d__Bacteria	p__Firmicutes	c__Clostridia	o__Lachnospirales	f__Lachnospiraceae	g__Fusicatenibacter	s__Fusicatenibacter saccharivorans	GCF_001405555.1	270	270_7	F68F_bin.34.fa	F68R_bin.107.fa	81.29	1.9	287
F68V_bin.89	d__Bacteria	p__Firmicutes	c__Clostridia	o__Lachnospirales	f__Lachnospiraceae	g__Fusicatenibacter	s__Fusicatenibacter saccharivorans	GCF_001405555.1	270	270_7	F68F_bin.34.fa	F68V_bin.89.fa	85.94	1.59	287
F68H_bin.41	d__Bacteria	p__Firmicutes	c__Clostridia	o__Lachnospirales	f__Lachnospiraceae	g__Fusicatenibacter	s__Fusicatenibacter saccharivorans	GCF_001405555.1	270	270_5	F68H_bin.41.fa	F68H_bin.41.fa	50.67	1.75	287

Table S3. Taxonomic classifiation of the 2,421 detected MAGs

	Domain	Phylum	Class	Order	Family	Genus	Species	RefGenome	primary_cluster	secondary_cluster	repr_genome	genome	CheckM_completeness	CheckM_contamination	
F68J_bin.10	d__Bacteria	p__Firmicutes	c__Clostridia	o__Lachnospirales	f__Lachnospiraceae	g__Fusicatenibacter	s__Fusicatenibacter saccharivorans	GCF_001405555.1	270	270_10	F68J_bin.10.fa	F68J_bin.10.fa	95.12	0.63	287
F68K_bin.72	d__Bacteria	p__Firmicutes	c__Clostridia	o__Lachnospirales	f__Lachnospiraceae	g__Fusicatenibacter	s__Fusicatenibacter saccharivorans	GCF_001405555.1	270	270_4	F68K_bin.72.fa	F68K_bin.72.fa	91.02	4.11	287
F68U_bin.85	d__Bacteria	p__Firmicutes	c__Clostridia	o__Lachnospirales	f__Lachnospiraceae	g__Fusicatenibacter	s__Fusicatenibacter saccharivorans	GCF_001405555.1	270	270_4	F68K_bin.72.fa	F68U_bin.85.fa	57.89	0	287
F68P_bin.15	d__Bacteria	p__Firmicutes	c__Clostridia	o__Lachnospirales	f__Lachnospiraceae	g__Fusicatenibacter	s__Fusicatenibacter saccharivorans	GCF_001405555.1	270	270_9	F68P_bin.15.fa	F68P_bin.15.fa	69.86	0.32	287
F68T_bin.74	d__Bacteria	p__Firmicutes	c__Clostridia	o__Lachnospirales	f__Lachnospiraceae	g__Fusicatenibacter	s__Fusicatenibacter saccharivorans	GCF_001405555.1	270	270_3	F68T_bin.74.fa	F68T_bin.74.fa	97.68	2.85	287
F68W_bin.17	d__Bacteria	p__Firmicutes	c__Clostridia	o__Lachnospirales	f__Lachnospiraceae	g__Fusicatenibacter	s__Fusicatenibacter saccharivorans	GCF_001405555.1	270	270_1	F68W_bin.17.fa	F68W_bin.17.fa	94.51	1.96	287

Table S3. Taxonomic classifiation of the 2,421 detected MAGs

	Domain	Phylum	Class	Order	Family	Genus	Species	RefGenome	primary_cluster	secondary_cluster	repr_genome	genome	CheckM_completeness	CheckM_contamination	
F6DH_bin.128	d__Bacteria	p__Firmicutes	c__Clostridia	o__Lachnospirales	f__Lachnospiraceae	g__Fusicatenibacter	s__Fusicatenibacter saccharivorans	GCF_001405555.1	270	270_1	F68W_bin.17.fa	F6DH_bin.128.fa	91.35	2.23	287
F6D7_bin.46	d__Bacteria	p__Firmicutes	c__Clostridia	o__Lachnospirales	f__Lachnospiraceae	g__Fusicatenibacter	s__Fusicatenibacter saccharivorans	GCF_001405555.1	270	270_6	F6D7_bin.46.fa	F6D7_bin.46.fa	93.61	0.63	287
F6DB_bin.3	d__Bacteria	p__Firmicutes	c__Clostridia	o__Lachnospirales	f__Lachnospiraceae	g__Fusicatenibacter	s__Fusicatenibacter saccharivorans	GCF_001405555.1	270	270_8	F6DB_bin.3.fa	F6DB_bin.3.fa	93.25	2.53	287
F6DG_bin.30	d__Bacteria	p__Firmicutes	c__Clostridia	o__Lachnospirales	f__Lachnospiraceae	g__Fusicatenibacter	s__Fusicatenibacter saccharivorans	GCF_001405555.1	270	270_11	F6DG_bin.30.fa	F6DG_bin.30.fa	95.76	2.11	287
F68T_bin.2	d__Bacteria	p__Firmicutes	c__Clostridia	o__Lachnospirales	f__Lachnospiraceae	g__GCA-900066575	s__	N/A	177	177_0	F68T_bin.2.fa	F68T_bin.2.fa	75.07	3.22	288
F68D_bin.71	d__Bacteria	p__Firmicutes	c__Clostridia	o__Lachnospirales	f__Lachnospiraceae	g__Hungatella	s__Hungatella effluvii	GCF_003201875.1	331	331_1	F68D_bin.71.fa	F68D_bin.71.fa	98.73	1.74	288
F68M_bin.20	d__Bacteria	p__Firmicutes	c__Clostridia	o__Lachnospirales	f__Lachnospiraceae	g__Hungatella	s__Hungatella effluvii	GCF_003201875.1	331	331_1	F68D_bin.71.fa	F68M_bin.20.fa	79.3	4.11	288

Table S3. Taxonomic classifiation of the 2,421 detected MAGs

	Domain	Phylum	Class	Order	Family	Genus	Species	RefGenome	primary_cluster	secondary_cluster	repr_genome	genome	CheckM_completeness	CheckM_contamination	
F6MY_bin.48	d__Bacteria	p__Firmicutes	c__Clostridia	o__Lachnospirales	f__Lachnospiraceae	g__Hungatella	s__Hungatella effluvii	GCF_003201875.1	331	331_1	F68D_bin.71.fa	F6MY_bin.48.fa	99.33	1.96	288
F6MX_bin.85	d__Bacteria	p__Firmicutes	c__Clostridia	o__Lachnospirales	f__Lachnospiraceae	g__Hungatella	s__Hungatella effluvii	GCF_003201875.1	331	331_2	F6MX_bin.85.fa	F6MX_bin.85.fa	54.7	1.75	288
F68G_bin.6	d__Bacteria	p__Firmicutes	c__Clostridia	o__Lachnospirales	f__Lachnospiraceae	g__Hungatella	s__Hungatella hathewayi	GCA_000433395.1	330	330_1	F68G_bin.6.fa	F68G_bin.6.fa	98.73	0.32	288
F68M_bin.83	d__Bacteria	p__Firmicutes	c__Clostridia	o__Lachnospirales	f__Lachnospiraceae	g__Hungatella	s__Hungatella hathewayi	GCA_000433395.1	330	330_1	F68G_bin.6.fa	F68M_bin.83.fa	89.89	3.38	288
F68V_bin.49	d__Bacteria	p__Firmicutes	c__Clostridia	o__Lachnospirales	f__Lachnospiraceae	g__Hungatella	s__Hungatella hathewayi	GCA_000433395.1	330	330_1	F68G_bin.6.fa	F68V_bin.49.fa	91.5	0.63	288
F68C_bin.37	d__Bacteria	p__Firmicutes	c__Clostridia	o__Lachnospirales	f__Lachnospiraceae	g__Hungatella_A	s__	N/A	306	306_1	F68C_bin.37.fa	F68C_bin.37.fa	83.44	2.22	289
F68R_bin.83	d__Bacteria	p__Firmicutes	c__Clostridia	o__Lachnospirales	f__Lachnospiraceae	g__Hungatella_A	s__	N/A	306	306_3	F68R_bin.83.fa	F68R_bin.83.fa	65.52	1.75	290
F6DH_bin.107	d__Bacteria	p__Firmicutes	c__Clostridia	o__Lachnospirales	f__Lachnospiraceae	g__Hungatella_A	s__	N/A	306	306_4	F6DH_bin.107.fa	F6DH_bin.107.fa	57.76	1.75	291

Table S3. Taxonomic classifiation of the 2,421 detected MAGs

	Domain	Phylum	Class	Order	Family	Genus	Species	RefGenome	primary_cluster	secondary_cluster	repr_genome	genome	CheckM_completeness	CheckM_contamination	
F6MY_bin.58	d__Bacteria	p__Firmicutes	c__Clostridia	o__Lachnospirales	f__Lachnospiraceae	g__Hungatella_A	s__	N/A	306	306_2	F6MY_bin.58.fa	F6MY_bin.58.fa	95.86	0.63	292
F68D_bin.56	d__Bacteria	p__Firmicutes	c__Clostridia	o__Lachnospirales	f__Lachnospiraceae	g__Hungatella_A	s__Hungatella_A hathewayi	GCF_000235505.1	332	332_0	F68D_bin.56.fa	F68D_bin.56.fa	95.66	1.96	292
F6MY_bin.37	d__Bacteria	p__Firmicutes	c__Clostridia	o__Lachnospirales	f__Lachnospiraceae	g__KLE1615	s__	N/A	285	285_7	F6MY_bin.37.fa	F6MY_bin.37.fa	62.6	3.51	293
F68C_bin.76	d__Bacteria	p__Firmicutes	c__Clostridia	o__Lachnospirales	f__Lachnospiraceae	g__KLE1615	s__KLE1615 sp900066985	GCA_900066985.1	285	285_2	F68C_bin.76.fa	F68C_bin.76.fa	97.47	1.58	293
F68F_bin.88	d__Bacteria	p__Firmicutes	c__Clostridia	o__Lachnospirales	f__Lachnospiraceae	g__KLE1615	s__KLE1615 sp900066985	GCA_900066985.1	285	285_4	F68F_bin.88.fa	F68F_bin.88.fa	88.36	3.01	293
F68H_bin.9	d__Bacteria	p__Firmicutes	c__Clostridia	o__Lachnospirales	f__Lachnospiraceae	g__KLE1615	s__KLE1615 sp900066985	GCA_900066985.1	285	285_12	F68H_bin.9.fa	F68H_bin.9.fa	55.26	1.75	293
F68R_bin.99	d__Bacteria	p__Firmicutes	c__Clostridia	o__Lachnospirales	f__Lachnospiraceae	g__KLE1615	s__KLE1615 sp900066985	GCA_900066985.1	285	285_10	F68R_bin.99.fa	F68R_bin.99.fa	80.7	0	293
F68T_bin.94	d__Bacteria	p__Firmicutes	c__Clostridia	o__Lachnospirales	f__Lachnospiraceae	g__KLE1615	s__KLE1615 sp900066985	GCA_900066985.1	285	285_5	F68T_bin.94.fa	F68T_bin.94.fa	73.48	2.78	293

Table S3. Taxonomic classifiation of the 2,421 detected MAGs

	Domain	Phylum	Class	Order	Family	Genus	Species	RefGenome	primary_cluster	secondary_cluster	repr_genome	genome	CheckM_completeness	CheckM_contamination	
F68U_bin.17	d__Bacteria	p__Firmicutes	c__Clostridia	o__Lachnospirales	f__Lachnospiraceae	g__KLE1615	s__KLE1615 sp900066985	GCA_900066985.1	285	285_1	F68U_bin.17.fa	F68U_bin.17.fa	93.67	4.02	293
F68W_bin.73	d__Bacteria	p__Firmicutes	c__Clostridia	o__Lachnospirales	f__Lachnospiraceae	g__KLE1615	s__KLE1615 sp900066985	GCA_900066985.1	285	285_1	F68U_bin.17.fa	F68W_bin.73.fa	57.02	0	293
F6DH_bin.4	d__Bacteria	p__Firmicutes	c__Clostridia	o__Lachnospirales	f__Lachnospiraceae	g__KLE1615	s__KLE1615 sp900066985	GCA_900066985.1	285	285_1	F68U_bin.17.fa	F6DH_bin.4.fa	85.41	2.37	293
F6D7_bin.35	d__Bacteria	p__Firmicutes	c__Clostridia	o__Lachnospirales	f__Lachnospiraceae	g__KLE1615	s__KLE1615 sp900066985	GCA_900066985.1	285	285_9	F6D7_bin.35.fa	F6D7_bin.35.fa	95.89	0.95	293
F6D8_bin.3	d__Bacteria	p__Firmicutes	c__Clostridia	o__Lachnospirales	f__Lachnospiraceae	g__KLE1615	s__KLE1615 sp900066985	GCA_900066985.1	285	285_11	F6D8_bin.3.fa	F6D8_bin.3.fa	95.65	2.22	293
F6D9_bin.40	d__Bacteria	p__Firmicutes	c__Clostridia	o__Lachnospirales	f__Lachnospiraceae	g__KLE1615	s__KLE1615 sp900066985	GCA_900066985.1	285	285_3	F6D9_bin.40.fa	F6D9_bin.40.fa	96.92	1.58	293
F6DB_bin.116	d__Bacteria	p__Firmicutes	c__Clostridia	o__Lachnospirales	f__Lachnospiraceae	g__KLE1615	s__KLE1615 sp900066985	GCA_900066985.1	285	285_8	F6DB_bin.116.fa	F6DB_bin.116.fa	90.4	4.11	293

Table S3. Taxonomic classifiation of the 2,421 detected MAGs

	Domain	Phylum	Class	Order	Family	Genus	Species	RefGenome	primary_cluster	secondary_cluster	repr_genome	genome	CheckM_completeness	CheckM_contamination	
F6DG_bin.47	d__Bacteria	p__Firmicutes	c__Clostridia	o__Lachnospirales	f__Lachnospiraceae	g__KLE1615	s__KLE1615 sp900066985	GCA_900066985.1	285	285_6	F6DG_bin.47.fa	F6DG_bin.47.fa	82.49	1.8	293
F68P_bin.21	d__Bacteria	p__Firmicutes	c__Clostridia	o__Lachnospirales	f__Lachnospiraceae	g__Lachnospira	s__Lachnospira eligens	GCF_000146185.1	363	363_12	F68P_bin.21.fa	F68P_bin.21.fa	94.61	0.17	293
F68C_bin.81	d__Bacteria	p__Firmicutes	c__Clostridia	o__Lachnospirales	f__Lachnospiraceae	g__Lachnospira	s__Lachnospira eligens_B	GCA_000434415.1	363	363_9	F68C_bin.81.fa	F68C_bin.81.fa	82.59	0	293
F68N_bin.57	d__Bacteria	p__Firmicutes	c__Clostridia	o__Lachnospirales	f__Lachnospiraceae	g__Lachnospira	s__Lachnospira eligens_B	GCA_000434415.1	363	363_7	F68N_bin.57.fa	F68N_bin.57.fa	98.66	1.01	293
F68U_bin.93	d__Bacteria	p__Firmicutes	c__Clostridia	o__Lachnospirales	f__Lachnospiraceae	g__Lachnospira	s__Lachnospira eligens_B	GCA_000434415.1	363	363_10	F68U_bin.93.fa	F68U_bin.93.fa	84.57	4.05	293
F68V_bin.85	d__Bacteria	p__Firmicutes	c__Clostridia	o__Lachnospirales	f__Lachnospiraceae	g__Lachnospira	s__Lachnospira eligens_B	GCA_000434415.1	363	363_5	F68V_bin.85.fa	F68V_bin.85.fa	97.32	0.67	293
F6D7_bin.58	d__Bacteria	p__Firmicutes	c__Clostridia	o__Lachnospirales	f__Lachnospiraceae	g__Lachnospira	s__Lachnospira eligens_B	GCA_000434415.1	363	363_11	F6D7_bin.58.fa	F6D7_bin.58.fa	97.99	0	293
F68M_bin.36	d__Bacteria	p__Firmicutes	c__Clostridia	o__Lachnospirales	f__Lachnospiraceae	g__Lachnospira	s__Lachnospira eligens_B	GCA_000434415.1	363	363_3	F6D8_bin.19.fa	F68M_bin.36.fa	97.32	0.36	293

Table S3. Taxonomic classifiation of the 2,421 detected MAGs

	Domain	Phylum	Class	Order	Family	Genus	Species	RefGenome	primary_cluster	secondary_cluster	repr_genome	genome	CheckM_completeness	CheckM_contamination	
F6D8_bin.19	d__Bacteria	p__Firmicutes	c__Clostridia	o__Lachnospirales	f__Lachnospiraceae	g__Lachnospira	s__Lachnospira eligens_B	GCA_000434415.1	363	363_3	F6D8_bin.19.fa	F6D8_bin.19.fa	97.99	0	293
F68R_bin.61	d__Bacteria	p__Firmicutes	c__Clostridia	o__Lachnospirales	f__Lachnospiraceae	g__Lachnospira	s__Lachnospira eligens_B	GCA_000434415.1	363	363_4	F6DB_bin.109.fa	F68R_bin.61.fa	93.93	1.68	293
F6DB_bin.109	d__Bacteria	p__Firmicutes	c__Clostridia	o__Lachnospirales	f__Lachnospiraceae	g__Lachnospira	s__Lachnospira eligens_B	GCA_000434415.1	363	363_4	F6DB_bin.109.fa	F6DB_bin.109.fa	97.32	0	293
F6MY_bin.91	d__Bacteria	p__Firmicutes	c__Clostridia	o__Lachnospirales	f__Lachnospiraceae	g__Lachnospira	s__Lachnospira eligens_B	GCA_000434415.1	363	363_4	F6DB_bin.109.fa	F6MY_bin.91.fa	98.64	0.67	293
F6DE_bin.5	d__Bacteria	p__Firmicutes	c__Clostridia	o__Lachnospirales	f__Lachnospiraceae	g__Lachnospira	s__Lachnospira eligens_B	GCA_000434415.1	363	363_8	F6DE_bin.5.fa	F6DE_bin.5.fa	97.82	0.02	293
F6DA_bin.31	d__Bacteria	p__Firmicutes	c__Clostridia	o__Lachnospirales	f__Lachnospiraceae	g__Lachnospira	s__Lachnospira eligens_B	GCA_000434415.1	363	363_2	F6DG_bin.52.fa	F6DA_bin.31.fa	97.34	0.34	293
F6DG_bin.52	d__Bacteria	p__Firmicutes	c__Clostridia	o__Lachnospirales	f__Lachnospiraceae	g__Lachnospira	s__Lachnospira eligens_B	GCA_000434415.1	363	363_2	F6DG_bin.52.fa	F6DG_bin.52.fa	98.66	0.15	293

Table S3. Taxonomic classifiation of the 2,421 detected MAGs

	Domain	Phylum	Class	Order	Family	Genus	Species	RefGenome	primary_cluster	secondary_cluster	repr_genome	genome	CheckM_completeness	CheckM_contamination	
F6MX_bin.12	d__Bacteria	p__Firmicutes	c__Clostridia	o__Lachnospirales	f__Lachnospiraceae	g__Lachnospira	s__Lachnospira eligens_B	GCA_000434415.1	363	363_6	F6MX_bin.12.fa	F6MX_bin.12.fa	83.64	0	293
F68C_bin.9	d__Bacteria	p__Firmicutes	c__Clostridia	o__Lachnospirales	f__Lachnospiraceae	g__Lachnospira	s__Lachnospira rogosae	GCF_900112995.1	365	365_3	F68C_bin.9.fa	F68C_bin.9.fa	78.48	0	293
F68D_bin.51	d__Bacteria	p__Firmicutes	c__Clostridia	o__Lachnospirales	f__Lachnospiraceae	g__Lachnospira	s__Lachnospira rogosae	GCF_900112995.1	365	365_6	F68D_bin.51.fa	F68D_bin.51.fa	71.99	0	293
F68N_bin.56	d__Bacteria	p__Firmicutes	c__Clostridia	o__Lachnospirales	f__Lachnospiraceae	g__Lachnospira	s__Lachnospira rogosae	GCF_900112995.1	365	365_5	F68N_bin.56.fa	F68N_bin.56.fa	99.33	0	293
F68T_bin.99	d__Bacteria	p__Firmicutes	c__Clostridia	o__Lachnospirales	f__Lachnospiraceae	g__Lachnospira	s__Lachnospira rogosae	GCF_900112995.1	365	365_2	F68T_bin.99.fa	F68T_bin.99.fa	61.4	0	293
F68U_bin.66	d__Bacteria	p__Firmicutes	c__Clostridia	o__Lachnospirales	f__Lachnospiraceae	g__Lachnospira	s__Lachnospira rogosae	GCF_900112995.1	365	365_7	F68U_bin.66.fa	F68U_bin.66.fa	97.36	0	293
F68R_bin.3	d__Bacteria	p__Firmicutes	c__Clostridia	o__Lachnospirales	f__Lachnospiraceae	g__Lachnospira	s__Lachnospira rogosae	GCF_900112995.1	365	365_1	F6DA_bin.57.fa	F68R_bin.3.fa	51.75	0	293
F6DA_bin.57	d__Bacteria	p__Firmicutes	c__Clostridia	o__Lachnospirales	f__Lachnospiraceae	g__Lachnospira	s__Lachnospira rogosae	GCF_900112995.1	365	365_1	F6DA_bin.57.fa	F6DA_bin.57.fa	93.1	1.34	293
F6DB_bin.92	d__Bacteria	p__Firmicutes	c__Clostridia	o__Lachnospirales	f__Lachnospiraceae	g__Lachnospira	s__Lachnospira rogosae	GCF_900112995.1	365	365_1	F6DA_bin.57.fa	F6DB_bin.92.fa	88.61	2.68	293
F6DE_bin.49	d__Bacteria	p__Firmicutes	c__Clostridia	o__Lachnospirales	f__Lachnospiraceae	g__Lachnospira	s__Lachnospira rogosae	GCF_900112995.1	365	365_4	F6DE_bin.49.fa	F6DE_bin.49.fa	97.8	0.67	293

Table S3. Taxonomic classifiation of the 2,421 detected MAGs

	Domain	Phylum	Class	Order	Family	Genus	Species	RefGenome	primary_cluster	secondary_cluster	repr_genome	genome	CheckM_completeness	CheckM_contamination	
F68H_bin.25	d__Bacteria	p__Firmicutes	c__Clostridia	o__Lachnospirales	f__Lachnospiraceae	g__Lachnospira	s__Lachnospira sp000436475	GCA_000436475.1	364	364_5	F68H_bin.25.fa	F68H_bin.25.fa	98.66	2.01	293
F6DB_bin.82	d__Bacteria	p__Firmicutes	c__Clostridia	o__Lachnospirales	f__Lachnospiraceae	g__Lachnospira	s__Lachnospira sp000436475	GCA_000436475.1	364	364_5	F68H_bin.25.fa	F6DB_bin.82.fa	91.83	3.36	293
F68K_bin.53	d__Bacteria	p__Firmicutes	c__Clostridia	o__Lachnospirales	f__Lachnospiraceae	g__Lachnospira	s__Lachnospira sp000436475	GCA_000436475.1	364	364_4	F68K_bin.53.fa	F68K_bin.53.fa	64.62	1.75	293
F6D9_bin.50	d__Bacteria	p__Firmicutes	c__Clostridia	o__Lachnospirales	f__Lachnospiraceae	g__Lachnospira	s__Lachnospira sp000436475	GCA_000436475.1	364	364_6	F6D9_bin.50.fa	F6D9_bin.50.fa	99.33	0.67	293
F6DA_bin.58	d__Bacteria	p__Firmicutes	c__Clostridia	o__Lachnospirales	f__Lachnospiraceae	g__Lachnospira	s__Lachnospira sp000436475	GCA_000436475.1	364	364_1	F6DA_bin.58.fa	F6DA_bin.58.fa	76.67	3.51	293
F6DG_bin.18	d__Bacteria	p__Firmicutes	c__Clostridia	o__Lachnospirales	f__Lachnospiraceae	g__Lachnospira	s__Lachnospira sp000436475	GCA_000436475.1	364	364_2	F6DG_bin.18.fa	F6DG_bin.18.fa	97.32	0.67	293
F68W_bin.52	d__Bacteria	p__Firmicutes	c__Clostridia	o__Lachnospirales	f__Lachnospiraceae	g__Lachnospira	s__Lachnospira sp000436475	GCA_000436475.1	364	364_3	F6DH_bin.71.fa	F68W_bin.52.fa	96.58	1.36	293

Table S3. Taxonomic classifiation of the 2,421 detected MAGs

	Domain	Phylum	Class	Order	Family	Genus	Species	RefGenome	primary_cluster	secondary_cluster	repr_genome	genome	CheckM_completeness	CheckM_contamination	
F6DH_bin.71	d__Bacteria	p__Firmicutes	c__Clostridia	o__Lachnospirales	f__Lachnospiraceae	g__Lachnospira	s__Lachnospira sp000436475	GCA_000436475.1	364	364_3	F6DH_bin.71.fa	F6DH_bin.71.fa	98.66	0	293
F6MY_bin.120	d__Bacteria	p__Firmicutes	c__Clostridia	o__Lachnospirales	f__Lachnospiraceae	g__Lachnospira	s__Lachnospira sp000436475	GCA_000436475.1	364	364_7	F6MY_bin.120.fa	F6MY_bin.120.fa	97.99	0	293
F68F_bin.10	d__Bacteria	p__Firmicutes	c__Clostridia	o__Lachnospirales	f__Lachnospiraceae	g__Lachnospira	s__Lachnospira sp000437735	GCA_000437735.1	359	359_8	F68F_bin.10.fa	F68F_bin.10.fa	90.34	1.7	293
F68H_bin.63	d__Bacteria	p__Firmicutes	c__Clostridia	o__Lachnospirales	f__Lachnospiraceae	g__Lachnospira	s__Lachnospira sp000437735	GCA_000437735.1	359	359_4	F68H_bin.63.fa	F68H_bin.63.fa	98.49	0.34	293
F68R_bin.98	d__Bacteria	p__Firmicutes	c__Clostridia	o__Lachnospirales	f__Lachnospiraceae	g__Lachnospira	s__Lachnospira sp000437735	GCA_000437735.1	359	359_9	F68R_bin.98.fa	F68R_bin.98.fa	95.32	1.57	293
F68T_bin.52	d__Bacteria	p__Firmicutes	c__Clostridia	o__Lachnospirales	f__Lachnospiraceae	g__Lachnospira	s__Lachnospira sp000437735	GCA_000437735.1	359	359_3	F68T_bin.52.fa	F68T_bin.52.fa	94.54	0	293
F68U_bin.22	d__Bacteria	p__Firmicutes	c__Clostridia	o__Lachnospirales	f__Lachnospiraceae	g__Lachnospira	s__Lachnospira sp000437735	GCA_000437735.1	359	359_10	F68U_bin.22.fa	F68U_bin.22.fa	67.25	1.51	293

Table S3. Taxonomic classifiation of the 2,421 detected MAGs

	Domain	Phylum	Class	Order	Family	Genus	Species	RefGenome	primary_cluster	secondary_cluster	repr_genome	genome	CheckM_completeness	CheckM_contamination	
F6D7_bin.63	d__Bacteria	p__Firmicutes	c__Clostridia	o__Lachnospirales	f__Lachnospiraceae	g__Lachnospira	s__Lachnospira sp000437735	GCA_000437735.1	359	359_1	F6D7_bin.63.fa	F6D7_bin.63.fa	81.31	2.57	293
F6D8_bin.15	d__Bacteria	p__Firmicutes	c__Clostridia	o__Lachnospirales	f__Lachnospiraceae	g__Lachnospira	s__Lachnospira sp000437735	GCA_000437735.1	359	359_6	F6D8_bin.15.fa	F6D8_bin.15.fa	71.17	0	293
F6DA_bin.32	d__Bacteria	p__Firmicutes	c__Clostridia	o__Lachnospirales	f__Lachnospiraceae	g__Lachnospira	s__Lachnospira sp000437735	GCA_000437735.1	359	359_7	F6DA_bin.32.fa	F6DA_bin.32.fa	95.99	0	293
F6DG_bin.63	d__Bacteria	p__Firmicutes	c__Clostridia	o__Lachnospirales	f__Lachnospiraceae	g__Lachnospira	s__Lachnospira sp000437735	GCA_000437735.1	359	359_11	F6DG_bin.63.fa	F6DG_bin.63.fa	98.49	0	293
F6DH_bin.87	d__Bacteria	p__Firmicutes	c__Clostridia	o__Lachnospirales	f__Lachnospiraceae	g__Lachnospira	s__Lachnospira sp000437735	GCA_000437735.1	359	359_2	F6DH_bin.87.fa	F6DH_bin.87.fa	96.1	0	293
F6MY_bin.132	d__Bacteria	p__Firmicutes	c__Clostridia	o__Lachnospirales	f__Lachnospiraceae	g__Lachnospira	s__Lachnospira sp000437735	GCA_000437735.1	359	359_5	F6MY_bin.132.fa	F6MY_bin.132.fa	88.73	2.41	293
F68W_bin.24	d__Bacteria	p__Firmicutes	c__Clostridia	o__Lachnospirales	f__Lachnospiraceae	g__Lachnospira	s__Lachnospira sp003537285	GCA_003537285.1	363	363_1	F6DH_bin.101.fa	F68W_bin.24.fa	90.02	0.67	293

Table S3. Taxonomic classifiation of the 2,421 detected MAGs

	Domain	Phylum	Class	Order	Family	Genus	Species	RefGenome	primary_cluster	secondary_cluster	repr_genome	genome	CheckM_completeness	CheckM_contamination	
F6DH_bin.101	d__Bacteria	p__Firmicutes	c__Clostridia	o__Lachnospirales	f__Lachnospiraceae	g__Lachnospira	s__Lachnospira sp003537285	GCA_003537285.1	363	363_1	F6DH_bin.101.fa	F6DH_bin.101.fa	95.7	0.34	293
F68D_bin.53	d__Bacteria	p__Firmicutes	c__Clostridia	o__Lachnospirales	f__Lachnospiraceae	g__Oribacterium	s__	N/A	19	19_1	F68D_bin.53.fa	F68D_bin.53.fa	96.5	0.71	294
F68G_bin.57	d__Bacteria	p__Firmicutes	c__Clostridia	o__Lachnospirales	f__Lachnospiraceae	g__Oribacterium	s__	N/A	19	19_2	F68G_bin.57.fa	F68G_bin.57.fa	95.58	0.63	295
F6MX_bin.35	d__Bacteria	p__Firmicutes	c__Clostridia	o__Lachnospirales	f__Lachnospiraceae	g__Oribacterium	s__	N/A	19	19_3	F6MX_bin.35.fa	F6MX_bin.35.fa	95.85	0	296
F68R_bin.82	d__Bacteria	p__Firmicutes	c__Clostridia	o__Lachnospirales	f__Lachnospiraceae	g__Roseburia	s__	N/A	337	337_3	F68R_bin.82.fa	F68R_bin.82.fa	89.04	3.1	297
F6D8_bin.84	d__Bacteria	p__Firmicutes	c__Clostridia	o__Lachnospirales	f__Lachnospiraceae	g__Roseburia	s__	N/A	337	337_4	F6D8_bin.84.fa	F6D8_bin.84.fa	99.28	2.9	298
F6D9_bin.76	d__Bacteria	p__Firmicutes	c__Clostridia	o__Lachnospirales	f__Lachnospiraceae	g__Roseburia	s__	N/A	337	337_1	F6D9_bin.76.fa	F6D9_bin.76.fa	81.84	1.08	299
F6DG_bin.100	d__Bacteria	p__Firmicutes	c__Clostridia	o__Lachnospirales	f__Lachnospiraceae	g__Roseburia	s__	N/A	337	337_2	F6DG_bin.100.fa	F6DG_bin.100.fa	97.83	0.48	300
F68F_bin.99	d__Bacteria	p__Firmicutes	c__Clostridia	o__Lachnospirales	f__Lachnospiraceae	g__Roseburia	s__	N/A	343	343_5	F68F_bin.99.fa	F68F_bin.99.fa	98.07	0.6	301
F68P_bin.9	d__Bacteria	p__Firmicutes	c__Clostridia	o__Lachnospirales	f__Lachnospiraceae	g__Roseburia	s__	N/A	343	343_8	F68P_bin.9.fa	F68P_bin.9.fa	97.1	0.6	302

Table S3. Taxonomic classifiation of the 2,421 detected MAGs

	Domain	Phylum	Class	Order	Family	Genus	Species	RefGenome	primary_cluster	secondary_cluster	repr_genome	genome	CheckM_completeness	CheckM_contamination	
F68R_bin.4	d__Bacteria	p__Firmicutes	c__Clostridia	o__Lachnospirales	f__Lachnospiraceae	g__Roseburia	s__	N/A	343	343_1	F68R_bin.4.fa	F68R_bin.4.fa	94.65	0.6	303
F6D7_bin.59	d__Bacteria	p__Firmicutes	c__Clostridia	o__Lachnospirales	f__Lachnospiraceae	g__Roseburia	s__	N/A	343	343_3	F6D7_bin.59.fa	F6D7_bin.59.fa	95.01	2.17	304
F6D8_bin.65	d__Bacteria	p__Firmicutes	c__Clostridia	o__Lachnospirales	f__Lachnospiraceae	g__Roseburia	s__	N/A	343	343_6	F6D8_bin.65.fa	F6D8_bin.65.fa	80.7	0	305
F6DA_bin.80	d__Bacteria	p__Firmicutes	c__Clostridia	o__Lachnospirales	f__Lachnospiraceae	g__Roseburia	s__	N/A	343	343_2	F6DA_bin.80.fa	F6DA_bin.80.fa	62.28	4.39	306
F6DG_bin.23	d__Bacteria	p__Firmicutes	c__Clostridia	o__Lachnospirales	f__Lachnospiraceae	g__Roseburia	s__	N/A	343	343_4	F6DG_bin.23.fa	F6DG_bin.23.fa	96.62	0.6	307
F6MY_bin.98	d__Bacteria	p__Firmicutes	c__Clostridia	o__Lachnospirales	f__Lachnospiraceae	g__Roseburia	s__	N/A	343	343_7	F6MY_bin.98.fa	F6MY_bin.98.fa	92.35	0.6	308
F6D9_bin.80	d__Bacteria	p__Firmicutes	c__Clostridia	o__Lachnospirales	f__Lachnospiraceae	g__Roseburia	s__	N/A	351	351_0	F6D9_bin.80.fa	F6D9_bin.80.fa	55.93	0	309
F6MX_bin.127	d__Bacteria	p__Firmicutes	c__Clostridia	o__Lachnospirales	f__Lachnospiraceae	g__Roseburia	s__	N/A	352	352_0	F6MX_bin.127.fa	F6MX_bin.127.fa	97.83	0	310
F68C_bin.12	d__Bacteria	p__Firmicutes	c__Clostridia	o__Lachnospirales	f__Lachnospiraceae	g__Roseburia	s__Roseburia hominis	GCF_000225345.1	282	282_4	F68C_bin.12.fa	F68C_bin.12.fa	83.88	1.93	310
F68H_bin.28	d__Bacteria	p__Firmicutes	c__Clostridia	o__Lachnospirales	f__Lachnospiraceae	g__Roseburia	s__Roseburia hominis	GCF_000225345.1	282	282_3	F68H_bin.28.fa	F68H_bin.28.fa	97.09	0.24	310

Table S3. Taxonomic classifiation of the 2,421 detected MAGs

	Domain	Phylum	Class	Order	Family	Genus	Species	RefGenome	primary_cluster	secondary_cluster	repr_genome	genome	CheckM_completeness	CheckM_contamination	
F68K_bin.57	d__Bacteria	p__Firmicutes	c__Clostridia	o__Lachnospirales	f__Lachnospiraceae	g__Roseburia	s__Roseburia hominis	GCF_000225345.1	282	282_7	F68K_bin.57.fa	F68K_bin.57.fa	54.39	1.75	310
F68R_bin.109	d__Bacteria	p__Firmicutes	c__Clostridia	o__Lachnospirales	f__Lachnospiraceae	g__Roseburia	s__Roseburia hominis	GCF_000225345.1	282	282_6	F68R_bin.109.fa	F68R_bin.109.fa	91.95	0.34	310
F68T_bin.47	d__Bacteria	p__Firmicutes	c__Clostridia	o__Lachnospirales	f__Lachnospiraceae	g__Roseburia	s__Roseburia hominis	GCF_000225345.1	282	282_5	F68T_bin.47.fa	F68T_bin.47.fa	88.76	1.69	310
F6D8_bin.14	d__Bacteria	p__Firmicutes	c__Clostridia	o__Lachnospirales	f__Lachnospiraceae	g__Roseburia	s__Roseburia hominis	GCF_000225345.1	282	282_1	F6D8_bin.14.fa	F6D8_bin.14.fa	97.18	0.72	310
F6MY_bin.112	d__Bacteria	p__Firmicutes	c__Clostridia	o__Lachnospirales	f__Lachnospiraceae	g__Roseburia	s__Roseburia hominis	GCF_000225345.1	282	282_2	F6MY_bin.112.fa	F6MY_bin.112.fa	68.6	0	310
F68T_bin.91	d__Bacteria	p__Firmicutes	c__Clostridia	o__Lachnospirales	f__Lachnospiraceae	g__Roseburia	s__Roseburia intestinalis	GCF_000156535.1	343	343_11	F68T_bin.91.fa	F68T_bin.91.fa	61.4	0	310
F68V_bin.21	d__Bacteria	p__Firmicutes	c__Clostridia	o__Lachnospirales	f__Lachnospiraceae	g__Roseburia	s__Roseburia intestinalis	GCF_000156535.1	343	343_9	F68V_bin.21.fa	F68V_bin.21.fa	82.41	1.21	310
F6DA_bin.30	d__Bacteria	p__Firmicutes	c__Clostridia	o__Lachnospirales	f__Lachnospiraceae	g__Roseburia	s__Roseburia intestinalis	GCF_000156535.1	343	343_10	F6DA_bin.30.fa	F6DA_bin.30.fa	84.1	1.43	310
F68H_bin.42	d__Bacteria	p__Firmicutes	c__Clostridia	o__Lachnospirales	f__Lachnospiraceae	g__Roseburia	s__Roseburia inulinivorans	GCF_000174195.1	337	337_5	F68H_bin.42.fa	F68H_bin.42.fa	83.6	2.17	310

Table S3. Taxonomic classifiation of the 2,421 detected MAGs

	Domain	Phylum	Class	Order	Family	Genus	Species	RefGenome	primary_cluster	secondary_cluster	repr_genome	genome	CheckM_completeness	CheckM_contamination	
F6D9_bin.27	d__Bacteria	p__Firmicutes	c__Clostridia	o__Lachnospirales	f__Lachnospiraceae	g__Roseburia	s__Roseburia inulinivorans	GCF_000174195.1	337	337_5	F68H_bin.42.fa	F6D9_bin.27.fa	50	3.51	310
F68N_bin.16	d__Bacteria	p__Firmicutes	c__Clostridia	o__Lachnospirales	f__Lachnospiraceae	g__Roseburia	s__Roseburia inulinivorans	GCF_000174195.1	337	337_7	F68N_bin.16.fa	F68N_bin.16.fa	93.11	0.97	310
F68R_bin.101	d__Bacteria	p__Firmicutes	c__Clostridia	o__Lachnospirales	f__Lachnospiraceae	g__Roseburia	s__Roseburia inulinivorans	GCF_000174195.1	337	337_6	F68R_bin.101.fa	F68R_bin.101.fa	64.04	0	310
F68T_bin.36	d__Bacteria	p__Firmicutes	c__Clostridia	o__Lachnospirales	f__Lachnospiraceae	g__Roseburia	s__Roseburia inulinivorans	GCF_000174195.1	337	337_9	F68T_bin.36.fa	F68T_bin.36.fa	89.37	0	310
F6DA_bin.3	d__Bacteria	p__Firmicutes	c__Clostridia	o__Lachnospirales	f__Lachnospiraceae	g__Roseburia	s__Roseburia inulinivorans	GCF_000174195.1	337	337_8	F6DA_bin.3.fa	F6DA_bin.3.fa	91.1	1.52	310
F68U_bin.15	d__Bacteria	p__Firmicutes	c__Clostridia	o__Lachnospirales	f__Lachnospiraceae	g__Ruminococcus_A	s__Ruminococcus_A sp000437095	GCA_000437095.1	305	305_4	F68U_bin.15.fa	F68U_bin.15.fa	89.51	0.63	310
F68C_bin.96	d__Bacteria	p__Firmicutes	c__Clostridia	o__Lachnospirales	f__Lachnospiraceae	g__Ruminococcus_A	s__Ruminococcus_A sp003011855	GCF_003011855.1	305	305_1	F6D7_bin.48.fa	F68C_bin.96.fa	87.4	2.53	310

Table S3. Taxonomic classifiation of the 2,421 detected MAGs

	Domain	Phylum	Class	Order	Family	Genus	Species	RefGenome	primary_cluster	secondary_cluster	repr_genome	genome	CheckM_completeness	CheckM_contamination	
F6D7_bin.48	d__Bacteria	p__Firmicutes	c__Clostridia	o__Lachnospirales	f__Lachnospiraceae	g__Ruminococcus_A	s__Ruminococcus_A sp003011855	GCF_003011855.1	305	305_1	F6D7_bin.48.fa	F6D7_bin.48.fa	95.57	0.63	310
F6DH_bin.126	d__Bacteria	p__Firmicutes	c__Clostridia	o__Lachnospirales	f__Lachnospiraceae	g__Ruminococcus_A	s__Ruminococcus_A sp003011855	GCF_003011855.1	305	305_2	F6DH_bin.126.fa	F6DH_bin.126.fa	84.24	0.63	310
F6MY_bin.89	d__Bacteria	p__Firmicutes	c__Clostridia	o__Lachnospirales	f__Lachnospiraceae	g__Ruminococcus_A	s__Ruminococcus_A sp003011855	GCF_003011855.1	305	305_3	F6MY_bin.89.fa	F6MY_bin.89.fa	77.91	3.74	310
F68T_bin.37	d__Bacteria	p__Firmicutes	c__Clostridia	o__Lachnospirales	f__Lachnospiraceae	g__Sellimonas	s__Sellimonas intestinalis	GCF_001280875.1	303	303_0	F68T_bin.37.fa	F68T_bin.37.fa	98.54	0.88	310
F68T_bin.25	d__Bacteria	p__Firmicutes	c__Clostridia	o__Lachnospirales	f__Lachnospiraceae	g__TF01-11	s__TF01-11 sp001414325	GCF_001414325.1	476	476_0	F68T_bin.25.fa	F68T_bin.25.fa	80.04	2.46	310
F68U_bin.87	d__Bacteria	p__Firmicutes	c__Clostridia	o__Lachnospirales	f__Lachnospiraceae	g__TF01-11	s__TF01-11 sp001916135	GCA_001916135.1	376	376_1	F6DH_bin.2.fa	F68U_bin.87.fa	85.91	0	310
F68W_bin.107	d__Bacteria	p__Firmicutes	c__Clostridia	o__Lachnospirales	f__Lachnospiraceae	g__TF01-11	s__TF01-11 sp001916135	GCA_001916135.1	376	376_1	F6DH_bin.2.fa	F68W_bin.107.fa	82.38	0	310

Table S3. Taxonomic classifiation of the 2,421 detected MAGs

	Domain	Phylum	Class	Order	Family	Genus	Species	RefGenome	primary_cluster	secondary_cluster	repr_genome	genome	CheckM_completeness	CheckM_contamination	
F6D8_bin.89	d__Bacteria	p__Firmicutes	c__Clostridia	o__Lachnospirales	f__Lachnospiraceae	g__TF01-11	s__TF01-11 sp001916135	GCA_001916135.1	376	376_1	F6DH_bin.2.fa	F6D8_bin.89.fa	68.68	4.39	310
F6D9_bin.36	d__Bacteria	p__Firmicutes	c__Clostridia	o__Lachnospirales	f__Lachnospiraceae	g__TF01-11	s__TF01-11 sp001916135	GCA_001916135.1	376	376_1	F6DH_bin.2.fa	F6D9_bin.36.fa	67.72	2.37	310
F6DH_bin.2	d__Bacteria	p__Firmicutes	c__Clostridia	o__Lachnospirales	f__Lachnospiraceae	g__TF01-11	s__TF01-11 sp001916135	GCA_001916135.1	376	376_1	F6DH_bin.2.fa	F6DH_bin.2.fa	98.66	0	310
F68R_bin.65	d__Bacteria	p__Firmicutes	c__Clostridia	o__Lachnospirales	f__Lachnospiraceae	g__TF01-11	s__TF01-11 sp003529475	GCA_003529475.1	375	375_1	F6DB_bin.7.fa	F68R_bin.65.fa	95.97	0	310
F68V_bin.10	d__Bacteria	p__Firmicutes	c__Clostridia	o__Lachnospirales	f__Lachnospiraceae	g__TF01-11	s__TF01-11 sp003529475	GCA_003529475.1	375	375_1	F6DB_bin.7.fa	F68V_bin.10.fa	97.32	0.73	310
F6D7_bin.55	d__Bacteria	p__Firmicutes	c__Clostridia	o__Lachnospirales	f__Lachnospiraceae	g__TF01-11	s__TF01-11 sp003529475	GCA_003529475.1	375	375_1	F6DB_bin.7.fa	F6D7_bin.55.fa	93.29	0	310
F6D8_bin.97	d__Bacteria	p__Firmicutes	c__Clostridia	o__Lachnospirales	f__Lachnospiraceae	g__TF01-11	s__TF01-11 sp003529475	GCA_003529475.1	375	375_1	F6DB_bin.7.fa	F6D8_bin.97.fa	58.06	2.11	310

Table S3. Taxonomic classifiation of the 2,421 detected MAGs

	Domain	Phylum	Class	Order	Family	Genus	Species	RefGenome	primary_cluster	secondary_cluster	repr_genome	genome	CheckM_completeness	CheckM_contamination	
F6DB_bin.7	d__Bacteria	p__Firmicutes	c__Clostridia	o__Lachnospirales	f__Lachnospiraceae	g__TF01-11	s__TF01-11 sp003529475	GCA_003529475.1	375	375_1	F6DB_bin.7.fa	F6DB_bin.7.fa	97.99	0	310
F6MY_bin.34	d__Bacteria	p__Firmicutes	c__Clostridia	o__Lachnospirales	f__Lachnospiraceae	g__TF01-11	s__TF01-11 sp003529475	GCA_003529475.1	375	375_1	F6DB_bin.7.fa	F6MY_bin.34.fa	93.29	0	310
F68K_bin.47	d__Bacteria	p__Firmicutes	c__Clostridia	o__Lachnospirales	f__Lachnospiraceae	g__Tyzzerella	s__Tyzzerella nexilis	GCF_000156035.2	300	300_2	F68K_bin.47.fa	F68K_bin.47.fa	93.86	0	310
F68G_bin.36	d__Bacteria	p__Firmicutes	c__Clostridia	o__Lachnospirales	f__Lachnospiraceae	g__Tyzzerella	s__Tyzzerella nexilis	GCF_000156035.2	300	300_1	F68R_bin.77.fa	F68G_bin.36.fa	83.54	0.63	310
F68R_bin.77	d__Bacteria	p__Firmicutes	c__Clostridia	o__Lachnospirales	f__Lachnospiraceae	g__Tyzzerella	s__Tyzzerella nexilis	GCF_000156035.2	300	300_1	F68R_bin.77.fa	F68R_bin.77.fa	91.12	1.27	310
F68T_bin.50	d__Bacteria	p__Firmicutes	c__Clostridia	o__Lachnospirales	f__Lachnospiraceae	g__Tyzzerella	s__Tyzzerella nexilis	GCF_000156035.2	300	300_1	F68R_bin.77.fa	F68T_bin.50.fa	90.17	3.8	310
F6D8_bin.67	d__Bacteria	p__Firmicutes	c__Clostridia	o__Lachnospirales	f__Lachnospiraceae	g__Tyzzerella	s__Tyzzerella nexilis	GCF_000156035.2	300	300_1	F68R_bin.77.fa	F6D8_bin.67.fa	71.39	1.75	310
F6D9_bin.70	d__Bacteria	p__Firmicutes	c__Clostridia	o__Lachnospirales	f__Lachnospiraceae	g__Tyzzerella	s__Tyzzerella nexilis	GCF_000156035.2	300	300_1	F68R_bin.77.fa	F6D9_bin.70.fa	59.65	0	310
F6DE_bin.46	d__Bacteria	p__Firmicutes	c__Clostridia	o__Lachnospirales	f__Lachnospiraceae	g__Tyzzerella	s__Tyzzerella sp000209385	GCF_000209385.2	301	301_0	F6DE_bin.46.fa	F6DE_bin.46.fa	78.52	1.16	310

Table S3. Taxonomic classifiation of the 2,421 detected MAGs

	Domain	Phylum	Class	Order	Family	Genus	Species	RefGenome	primary_cluster	secondary_cluster	repr_genome	genome	CheckM_completeness	CheckM_contamination	
F68M_bin.66	d__Bacteria	p__Firmicutes	c__Clostridia	o__Lachnospirales	f__Lachnospiraceae	g__Tyzzerella	s__Tyzzerella sp000411335	GCF_000411335.1	298	298_1	F6D9_bin.63.fa	F68M_bin.66.fa	82.91	0	310
F6D9_bin.63	d__Bacteria	p__Firmicutes	c__Clostridia	o__Lachnospirales	f__Lachnospiraceae	g__Tyzzerella	s__Tyzzerella sp000411335	GCF_000411335.1	298	298_1	F6D9_bin.63.fa	F6D9_bin.63.fa	94.09	0.71	310
F68R_bin.30	d__Bacteria	p__Firmicutes	c__Clostridia	o__Lachnospirales	f__Lachnospiraceae	g__UBA11774	s__UBA11774 sp003507655	GCA_003507655.1	280	280_2	F68R_bin.30.fa	F68R_bin.30.fa	72.44	1.95	310
F68V_bin.104	d__Bacteria	p__Firmicutes	c__Clostridia	o__Lachnospirales	f__Lachnospiraceae	g__UBA11774	s__UBA11774 sp003507655	GCA_003507655.1	280	280_4	F68V_bin.104.fa	F68V_bin.104.fa	51.52	0	310
F68W_bin.42	d__Bacteria	p__Firmicutes	c__Clostridia	o__Lachnospirales	f__Lachnospiraceae	g__UBA11774	s__UBA11774 sp003507655	GCA_003507655.1	280	280_1	F6DH_bin.11.fa	F68W_bin.42.fa	94.54	0.45	310
F6DH_bin.11	d__Bacteria	p__Firmicutes	c__Clostridia	o__Lachnospirales	f__Lachnospiraceae	g__UBA11774	s__UBA11774 sp003507655	GCA_003507655.1	280	280_1	F6DH_bin.11.fa	F6DH_bin.11.fa	97.99	0.45	310
F6MX_bin.44	d__Bacteria	p__Firmicutes	c__Clostridia	o__Lachnospirales	f__Lachnospiraceae	g__UBA11774	s__UBA11774 sp003507655	GCA_003507655.1	280	280_3	F6MX_bin.44.fa	F6MX_bin.44.fa	96.26	2.09	310
F68J_bin.46	d__Bacteria	p__Firmicutes	c__Clostridia	o__Lachnospirales	f__Lachnospiraceae	g__UBA4285	s__	N/A	145	145_1	F68K_bin.11.fa	F68J_bin.46.fa	56.14	0	311

Table S3. Taxonomic classifiation of the 2,421 detected MAGs

	Domain	Phylum	Class	Order	Family	Genus	Species	RefGenome	primary_cluster	secondary_cluster	repr_genome	genome	CheckM_completeness	CheckM_contamination	
F68K_bin.11	d__Bacteria	p__Firmicutes	c__Clostridia	o__Lachnospirales	f__Lachnospiraceae	g__UBA4285	s__	N/A	145	145_1	F68K_bin.11.fa	F68K_bin.11.fa	78.95	0	312
F68R_bin.14	d__Bacteria	p__Firmicutes	c__Clostridia	o__Lachnospirales	f__Lachnospiraceae	g__UBA4285	s__	N/A	145	145_1	F68K_bin.11.fa	F68R_bin.14.fa	69.96	1.23	313
F68E_bin.19	d__Bacteria	p__Firmicutes	c__Clostridia	o__Lachnospirales	f__Lachnospiraceae	g__UBA9502	s__	N/A	276	276_1	F68E_bin.19.fa	F68E_bin.19.fa	96.76	2.85	314
F68F_bin.47	d__Bacteria	p__Firmicutes	c__Clostridia	o__Lachnospirales	f__Lachnospiraceae	g__UBA9502	s__	N/A	276	276_2	F68F_bin.47.fa	F68F_bin.47.fa	72.87	1.27	315
F68F_bin.56	d__Bacteria	p__Firmicutes	c__Clostridia	o__Lachnospirales	f__Lachnospiraceae	g__UBA9502	s__	N/A	308	308_0	F68F_bin.56.fa	F68F_bin.56.fa	66.11	0	316
F68J_bin.80	d__Bacteria	p__Firmicutes	c__Clostridia	o__Lachnospirales	f__Lachnospiraceae	g__UBA7182	s__	N/A	318	318_1	F68J_bin.80.fa	F68J_bin.80.fa	70.09	0.32	317
F6DH_bin.125	d__Bacteria	p__Firmicutes	c__Clostridia	o__Lachnospirales	f__Lachnospiraceae	g__UBA7182	s__	N/A	318	318_2	F6DH_bin.125.fa	F6DH_bin.125.fa	60.5	0.21	318
F68V_bin.2	d__Bacteria	p__Firmicutes	c__Clostridia	o__Lachnospirales	f__Lachnospiraceae	g__UBA7160	s__	N/A	354	354_0	F68V_bin.2.fa	F68V_bin.2.fa	93.97	2.66	319
F68V_bin.71	d__Bacteria	p__Firmicutes	c__Clostridia	o__Lachnospirales	f__Lachnospiraceae	g__UBA7096	s__	N/A	355	355_0	F68V_bin.71.fa	F68V_bin.71.fa	99.33	0	320
F68E_bin.25	d__Bacteria	p__Firmicutes	c__Clostridia	o__Lachnospirales	f__Lachnospiraceae	g__UBA9502	s__UBA9502 sp003506385	GCA_003506385.1	277	277_0	F68E_bin.25.fa	F68E_bin.25.fa	52.95	0	320

Table S3. Taxonomic classifiation of the 2,421 detected MAGs

	Domain	Phylum	Class	Order	Family	Genus	Species	RefGenome	primary_cluster	secondary_cluster	repr_genome	genome	CheckM_completeness	CheckM_contamination	
F68D_bin.37	d__Bacteria	p__Firmicutes	c__Clostridia	o__Monoglobales	f__	g__	s__	N/A	496	496_0	F68D_bin.37.fa	F68D_bin.37.fa	86.06	0.22	321
F6D7_bin.104	d__Bacteria	p__Firmicutes	c__Clostridia	o__Monoglobales	f__	g__	s__	N/A	418	418_0	F6D7_bin.104.fa	F6D7_bin.104.fa	78.87	3.75	322
F6D7_bin.2	d__Bacteria	p__Firmicutes	c__Clostridia	o__Monoglobales	f__	g__	s__	N/A	421	421_0	F6D7_bin.2.fa	F6D7_bin.2.fa	97.97	0	323
F6DA_bin.81	d__Bacteria	p__Firmicutes	c__Clostridia	o__Monoglobales	f__	g__	s__	N/A	176	176_0	F6DA_bin.81.fa	F6DA_bin.81.fa	86.98	0.56	324
F68V_bin.13	d__Bacteria	p__Firmicutes	c__Clostridia	o__Monoglobales	f__Monoglobaceae	g__	s__	N/A	419	419_0	F68V_bin.13.fa	F68V_bin.13.fa	97.76	0	325
F68W_bin.31	d__Bacteria	p__Firmicutes	c__Clostridia	o__Monoglobales	f__UBA1381	g__	s__	N/A	420	420_0	F68W_bin.31.fa	F68W_bin.31.fa	85.49	1.87	326
F68E_bin.28	d__Bacteria	p__Firmicutes	c__Clostridia	o__Monoglobales	f__UBA1381	g__CAG-41	s__CAG-41 sp900066215	GCA_900066215.1	175	175_1	F68E_bin.28.fa	F68E_bin.28.fa	95.97	1.34	326
F68G_bin.73	d__Bacteria	p__Firmicutes	c__Clostridia	o__Monoglobales	f__UBA1381	g__CAG-41	s__CAG-41 sp900066215	GCA_900066215.1	175	175_2	F68G_bin.73.fa	F68G_bin.73.fa	97.32	2.01	326
F68K_bin.31	d__Bacteria	p__Firmicutes	c__Clostridia	o__Monoglobales	f__UBA1381	g__CAG-41	s__CAG-41 sp900066215	GCA_900066215.1	175	175_4	F68K_bin.31.fa	F68K_bin.31.fa	88.02	1.01	326

Table S3. Taxonomic classifiation of the 2,421 detected MAGs

	Domain	Phylum	Class	Order	Family	Genus	Species	RefGenome	primary_cluster	secondary_cluster	repr_genome	genome	CheckM_completeness	CheckM_contamination	
F68T_bin.41	d__Bacteria	p__Firmicutes	c__Clostridia	o__Monoglobales	f__UBA1381	g__CAG-41	s__CAG-41 sp900066215	GCA_900066215.1	175	175_3	F68T_bin.41.fa	F68T_bin.41.fa	97.99	1.01	326
F6DA_bin.35	d__Bacteria	p__Firmicutes	c__Clostridia	o__Monoglobales	f__UBA1381	g__CAG-41	s__CAG-41 sp900066215	GCA_900066215.1	175	175_5	F6DA_bin.35.fa	F6DA_bin.35.fa	94.28	4.36	326
F68D_bin.65	d__Bacteria	p__Firmicutes	c__Clostridia	o__Oscillospirales	f__	g__	s__	N/A	448	448_0	F68D_bin.65.fa	F68D_bin.65.fa	97.82	0	327
F68V_bin.97	d__Bacteria	p__Firmicutes	c__Clostridia	o__Oscillospirales	f__	g__	s__	N/A	571	571_0	F68V_bin.97.fa	F68V_bin.97.fa	89.61	3.13	328
F68D_bin.36	d__Bacteria	p__Firmicutes	c__Clostridia	o__Oscillospirales	f__Acutalibacteraceae	g__	s__	N/A	183	183_1	F68D_bin.36.fa	F68D_bin.36.fa	77	0	329
F68M_bin.9	d__Bacteria	p__Firmicutes	c__Clostridia	o__Oscillospirales	f__Acutalibacteraceae	g__	s__	N/A	183	183_1	F68D_bin.36.fa	F68M_bin.9.fa	82.36	1.78	330
F6DA_bin.23	d__Bacteria	p__Firmicutes	c__Clostridia	o__Oscillospirales	f__Acutalibacteraceae	g__	s__	N/A	183	183_1	F68D_bin.36.fa	F6DA_bin.23.fa	54.55	1.34	331
F68J_bin.48	d__Bacteria	p__Firmicutes	c__Clostridia	o__Oscillospirales	f__Acutalibacteraceae	g__	s__	N/A	232	232_0	F68J_bin.48.fa	F68J_bin.48.fa	66.79	0.67	332
F6DA_bin.43	d__Bacteria	p__Firmicutes	c__Clostridia	o__Oscillospirales	f__Acutalibacteraceae	g__	s__	N/A	412	412_0	F6DA_bin.43.fa	F6DA_bin.43.fa	97.32	0	333

Table S3. Taxonomic classifiation of the 2,421 detected MAGs

	Domain	Phylum	Class	Order	Family	Genus	Species	RefGenome	primary_cluster	secondary_cluster	repr_genome	genome	CheckM_completeness	CheckM_contamination	
F68V_bin.14	d__Bacteria	p__Firmicutes	c__Clostridia	o__Oscillospirales	f__Acutalibacteraceae	g__	s__	N/A	423	423_0	F68V_bin.14.fa	F68V_bin.14.fa	96.64	0.67	334
F68V_bin.24	d__Bacteria	p__Firmicutes	c__Clostridia	o__Oscillospirales	f__Acutalibacteraceae	g__	s__	N/A	424	424_0	F68V_bin.24.fa	F68V_bin.24.fa	81.65	1.34	335
F6DE_bin.39	d__Bacteria	p__Firmicutes	c__Clostridia	o__Oscillospirales	f__Acutalibacteraceae	g__	s__	N/A	425	425_0	F6DE_bin.39.fa	F6DE_bin.39.fa	94.63	0	336
F6DA_bin.71	d__Bacteria	p__Firmicutes	c__Clostridia	o__Oscillospirales	f__Acutalibacteraceae	g__	s__	N/A	437	437_0	F6DA_bin.71.fa	F6DA_bin.71.fa	83.89	0.67	337
F68M_bin.54	d__Bacteria	p__Firmicutes	c__Clostridia	o__Oscillospirales	f__Acutalibacteraceae	g__	s__	N/A	466	466_1	F68V_bin.106.fa	F68M_bin.54.fa	76.4	1.23	338
F68V_bin.106	d__Bacteria	p__Firmicutes	c__Clostridia	o__Oscillospirales	f__Acutalibacteraceae	g__	s__	N/A	466	466_1	F68V_bin.106.fa	F68V_bin.106.fa	89.89	2.15	339
F6DE_bin.7	d__Bacteria	p__Firmicutes	c__Clostridia	o__Oscillospirales	f__Acutalibacteraceae	g__	s__	N/A	466	466_1	F68V_bin.106.fa	F6DE_bin.7.fa	79.83	0.22	340
F6MY_bin.27	d__Bacteria	p__Firmicutes	c__Clostridia	o__Oscillospirales	f__Acutalibacteraceae	g__	s__	N/A	466	466_1	F68V_bin.106.fa	F6MY_bin.27.fa	68.94	0.67	341
F68D_bin.63	d__Bacteria	p__Firmicutes	c__Clostridia	o__Oscillospirales	f__Acutalibacteraceae	g__	s__	N/A	497	497_0	F68D_bin.63.fa	F68D_bin.63.fa	70.37	1.36	342
F68F_bin.81	d__Bacteria	p__Firmicutes	c__Clostridia	o__Oscillospirales	f__Acutalibacteraceae	g__	s__	N/A	498	498_0	F68F_bin.81.fa	F68F_bin.81.fa	95.97	1.7	343

Table S3. Taxonomic classifiation of the 2,421 detected MAGs

	Domain	Phylum	Class	Order	Family	Genus	Species	RefGenome	primary_cluster	secondary_cluster	repr_genome	genome	CheckM_completeness	CheckM_contamination	
F68V_bin.125	d__Bacteria	p__Firmicutes	c__Clostridia	o__Oscillospirales	f__Acutalibacteraceae	g__	s__	N/A	499	499_0	F68V_bin.125.fa	F68V_bin.125.fa	52.93	0	344
F68M_bin.61	d__Bacteria	p__Firmicutes	c__Clostridia	o__Oscillospirales	f__Acutalibacteraceae	g__	s__	N/A	563	563_1	F68M_bin.61.fa	F68M_bin.61.fa	97.32	0.69	345
F68V_bin.26	d__Bacteria	p__Firmicutes	c__Clostridia	o__Oscillospirales	f__Acutalibacteraceae	g__	s__	N/A	563	563_1	F68M_bin.61.fa	F68V_bin.26.fa	96.56	1.07	346
F6MY_bin.60	d__Bacteria	p__Firmicutes	c__Clostridia	o__Oscillospirales	f__Acutalibacteraceae	g__	s__	N/A	563	563_1	F68M_bin.61.fa	F6MY_bin.60.fa	94.63	1.46	347
F68D_bin.58	d__Bacteria	p__Firmicutes	c__Clostridia	o__Oscillospirales	f__Acutalibacteraceae	g__	s__	N/A	564	564_1	F68D_bin.58.fa	F68D_bin.58.fa	84.54	1.68	348
F6DG_bin.8	d__Bacteria	p__Firmicutes	c__Clostridia	o__Oscillospirales	f__Acutalibacteraceae	g__	s__	N/A	564	564_1	F68D_bin.58.fa	F6DG_bin.8.fa	85.65	4.16	349
F6D9_bin.7	d__Bacteria	p__Firmicutes	c__Clostridia	o__Oscillospirales	f__Acutalibacteraceae	g__	s__	N/A	565	565_1	F6D9_bin.7.fa	F6D9_bin.7.fa	97.32	0.67	350
F6DE_bin.60	d__Bacteria	p__Firmicutes	c__Clostridia	o__Oscillospirales	f__Acutalibacteraceae	g__	s__	N/A	565	565_2	F6DE_bin.60.fa	F6DE_bin.60.fa	77.63	4.7	351
F6MY_bin.65	d__Bacteria	p__Firmicutes	c__Clostridia	o__Oscillospirales	f__Acutalibacteraceae	g__	s__	N/A	572	572_0	F6MY_bin.65.fa	F6MY_bin.65.fa	96.64	0.67	352
F68M_bin.52	d__Bacteria	p__Firmicutes	c__Clostridia	o__Oscillospirales	f__Acutalibacteraceae	g__Acutalibacter	s__	N/A	602	602_0	F68M_bin.52.fa	F68M_bin.52.fa	85.21	0.67	353

Table S3. Taxonomic classifiation of the 2,421 detected MAGs

	Domain	Phylum	Class	Order	Family	Genus	Species	RefGenome	primary_cluster	secondary_cluster	repr_genome	genome	CheckM_completeness	CheckM_contamination	
F6D9_bin.64	d__Bacteria	p__Firmicutes	c__Clostridia	o__Oscillospirales	f__Acutalibacteraceae	g__CAG-177	s__CAG-177 sp000431775	GCA_000431775.1	482	482_0	F6D9_bin.64.fa	F6D9_bin.64.fa	97.05	0	353
F68N_bin.44	d__Bacteria	p__Firmicutes	c__Clostridia	o__Oscillospirales	f__Acutalibacteraceae	g__CAG-177	s__CAG-177 sp003514385	GCA_003514385.1	649	649_12	F68N_bin.44.fa	F68N_bin.44.fa	97.27	0	353
F68V_bin.23	d__Bacteria	p__Firmicutes	c__Clostridia	o__Oscillospirales	f__Acutalibacteraceae	g__CAG-177	s__CAG-177 sp003514385	GCA_003514385.1	649	649_13	F68V_bin.23.fa	F68V_bin.23.fa	97.27	0	353
F6D7_bin.6	d__Bacteria	p__Firmicutes	c__Clostridia	o__Oscillospirales	f__Acutalibacteraceae	g__CAG-177	s__CAG-177 sp003514385	GCA_003514385.1	649	649_14	F6D7_bin.6.fa	F6D7_bin.6.fa	97.27	0.34	353
F6D9_bin.42	d__Bacteria	p__Firmicutes	c__Clostridia	o__Oscillospirales	f__Acutalibacteraceae	g__CAG-177	s__CAG-177 sp003514385	GCA_003514385.1	649	649_10	F6D9_bin.42.fa	F6D9_bin.42.fa	97.27	0	353
F6DA_bin.48	d__Bacteria	p__Firmicutes	c__Clostridia	o__Oscillospirales	f__Acutalibacteraceae	g__CAG-177	s__CAG-177 sp003514385	GCA_003514385.1	649	649_10	F6D9_bin.42.fa	F6DA_bin.48.fa	97.27	0	353
F6DB_bin.2	d__Bacteria	p__Firmicutes	c__Clostridia	o__Oscillospirales	f__Acutalibacteraceae	g__CAG-177	s__CAG-177 sp003514385	GCA_003514385.1	649	649_15	F6DB_bin.2.fa	F6DB_bin.2.fa	97.27	0	353

Table S3. Taxonomic classifiation of the 2,421 detected MAGs

	Domain	Phylum	Class	Order	Family	Genus	Species	RefGenome	primary_cluster	secondary_cluster	repr_genome	genome	CheckM_completeness	CheckM_contamination	
F6DE_bin.36	d__Bacteria	p__Firmicutes	c__Clostridia	o__Oscillospirales	f__Acutalibacteraceae	g__CAG-177	s__CAG-177 sp003514385	GCA_003514385.1	649	649_11	F6DE_bin.36.fa	F6DE_bin.36.fa	97.27	0	353
F6MX_bin.112	d__Bacteria	p__Firmicutes	c__Clostridia	o__Oscillospirales	f__Acutalibacteraceae	g__CAG-177	s__CAG-177 sp003514385	GCA_003514385.1	649	649_16	F6MX_bin.112.fa	F6MX_bin.112.fa	86.88	0.17	353
F68F_bin.33	d__Bacteria	p__Firmicutes	c__Clostridia	o__Oscillospirales	f__Acutalibacteraceae	g__CAG-177	s__CAG-177 sp003538135	GCA_003538135.1	649	649_9	F68F_bin.33.fa	F68F_bin.33.fa	97.27	0	353
F68C_bin.82	d__Bacteria	p__Firmicutes	c__Clostridia	o__Oscillospirales	f__Acutalibacteraceae	g__CAG-177	s__CAG-177 sp003538135	GCA_003538135.1	649	649_2	F68G_bin.46.fa	F68C_bin.82.fa	96.6	0	353
F68G_bin.46	d__Bacteria	p__Firmicutes	c__Clostridia	o__Oscillospirales	f__Acutalibacteraceae	g__CAG-177	s__CAG-177 sp003538135	GCA_003538135.1	649	649_2	F68G_bin.46.fa	F68G_bin.46.fa	97.27	0	353
F68W_bin.72	d__Bacteria	p__Firmicutes	c__Clostridia	o__Oscillospirales	f__Acutalibacteraceae	g__CAG-177	s__CAG-177 sp003538135	GCA_003538135.1	649	649_2	F68G_bin.46.fa	F68W_bin.72.fa	96.6	0	353
F6DH_bin.44	d__Bacteria	p__Firmicutes	c__Clostridia	o__Oscillospirales	f__Acutalibacteraceae	g__CAG-177	s__CAG-177 sp003538135	GCA_003538135.1	649	649_2	F68G_bin.46.fa	F6DH_bin.44.fa	96.6	0	353

Table S3. Taxonomic classifiation of the 2,421 detected MAGs

	Domain	Phylum	Class	Order	Family	Genus	Species	RefGenome	primary_cluster	secondary_cluster	repr_genome	genome	CheckM_completeness	CheckM_contamination	
F68H_bin.6	d__Bacteria	p__Firmicutes	c__Clostridia	o__Oscillospirales	f__Acutalibacteraceae	g__CAG-177	s__CAG-177 sp003538135	GCA_003538135.1	649	649_4	F68H_bin.6.fa	F68H_bin.6.fa	97.27	0	353
F68J_bin.71	d__Bacteria	p__Firmicutes	c__Clostridia	o__Oscillospirales	f__Acutalibacteraceae	g__CAG-177	s__CAG-177 sp003538135	GCA_003538135.1	649	649_7	F68J_bin.71.fa	F68J_bin.71.fa	74.45	0	353
F68M_bin.57	d__Bacteria	p__Firmicutes	c__Clostridia	o__Oscillospirales	f__Acutalibacteraceae	g__CAG-177	s__CAG-177 sp003538135	GCA_003538135.1	649	649_1	F68M_bin.57.fa	F68M_bin.57.fa	97.27	0	353
F6MX_bin.4	d__Bacteria	p__Firmicutes	c__Clostridia	o__Oscillospirales	f__Acutalibacteraceae	g__CAG-177	s__CAG-177 sp003538135	GCA_003538135.1	649	649_1	F68M_bin.57.fa	F6MX_bin.4.fa	92.53	1.73	353
F68D_bin.26	d__Bacteria	p__Firmicutes	c__Clostridia	o__Oscillospirales	f__Acutalibacteraceae	g__CAG-177	s__CAG-177 sp003538135	GCA_003538135.1	649	649_3	F68R_bin.7.fa	F68D_bin.26.fa	96.6	0	353
F68R_bin.7	d__Bacteria	p__Firmicutes	c__Clostridia	o__Oscillospirales	f__Acutalibacteraceae	g__CAG-177	s__CAG-177 sp003538135	GCA_003538135.1	649	649_3	F68R_bin.7.fa	F68R_bin.7.fa	97.27	0	353
F68T_bin.86	d__Bacteria	p__Firmicutes	c__Clostridia	o__Oscillospirales	f__Acutalibacteraceae	g__CAG-177	s__CAG-177 sp003538135	GCA_003538135.1	649	649_3	F68R_bin.7.fa	F68T_bin.86.fa	95.26	0.44	353

Table S3. Taxonomic classifiation of the 2,421 detected MAGs

	Domain	Phylum	Class	Order	Family	Genus	Species	RefGenome	primary_cluster	secondary_cluster	repr_genome	genome	CheckM_completeness	CheckM_contamination	
F68U_bin.65	d__Bacteria	p__Firmicutes	c__Clostridia	o__Oscillospirales	f__Acutalibacteraceae	g__CAG-177	s__CAG-177 sp003538135	GCA_003538135.1	649	649_8	F68U_bin.65.fa	F68U_bin.65.fa	97.27	0.34	353
F6D8_bin.22	d__Bacteria	p__Firmicutes	c__Clostridia	o__Oscillospirales	f__Acutalibacteraceae	g__CAG-177	s__CAG-177 sp003538135	GCA_003538135.1	649	649_6	F6D8_bin.22.fa	F6D8_bin.22.fa	95.93	0	353
F6MY_bin.88	d__Bacteria	p__Firmicutes	c__Clostridia	o__Oscillospirales	f__Acutalibacteraceae	g__CAG-177	s__CAG-177 sp003538135	GCA_003538135.1	649	649_5	F6MY_bin.88.fa	F6MY_bin.88.fa	96.6	0	353
F6MX_bin.92	d__Bacteria	p__Firmicutes	c__Clostridia	o__Oscillospirales	f__Acutalibacteraceae	g__CAG-180	s__	N/A	478	478_0	F6MX_bin.92.fa	F6MX_bin.92.fa	96.98	0	354
F68C_bin.111	d__Bacteria	p__Firmicutes	c__Clostridia	o__Oscillospirales	f__Acutalibacteraceae	g__CAG-180	s__CAG-180 sp000432435	GCA_000432435.1	477	477_6	F68C_bin.111.fa	F68C_bin.111.fa	97.65	0.34	354
F68F_bin.91	d__Bacteria	p__Firmicutes	c__Clostridia	o__Oscillospirales	f__Acutalibacteraceae	g__CAG-180	s__CAG-180 sp000432435	GCA_000432435.1	477	477_9	F68F_bin.91.fa	F68F_bin.91.fa	96.81	0.34	354
F6DE_bin.88	d__Bacteria	p__Firmicutes	c__Clostridia	o__Oscillospirales	f__Acutalibacteraceae	g__CAG-180	s__CAG-180 sp000432435	GCA_000432435.1	477	477_9	F68F_bin.91.fa	F6DE_bin.88.fa	80.37	0	354
F68G_bin.22	d__Bacteria	p__Firmicutes	c__Clostridia	o__Oscillospirales	f__Acutalibacteraceae	g__CAG-180	s__CAG-180 sp000432435	GCA_000432435.1	477	477_1	F68G_bin.22.fa	F68G_bin.22.fa	96.81	0.67	354

Table S3. Taxonomic classifiation of the 2,421 detected MAGs

	Domain	Phylum	Class	Order	Family	Genus	Species	RefGenome	primary_cluster	secondary_cluster	repr_genome	genome	CheckM_completeness	CheckM_contamination	
F68H_bin.31	d__Bacteria	p__Firmicutes	c__Clostridia	o__Oscillospirales	f__Acutalibacteraceae	g__CAG-180	s__CAG-180 sp000432435	GCA_000432435.1	477	477_7	F68H_bin.31.fa	F68H_bin.31.fa	97.48	1.01	354
F68K_bin.14	d__Bacteria	p__Firmicutes	c__Clostridia	o__Oscillospirales	f__Acutalibacteraceae	g__CAG-180	s__CAG-180 sp000432435	GCA_000432435.1	477	477_4	F68N_bin.75.fa	F68K_bin.14.fa	96.81	0.97	354
F68N_bin.75	d__Bacteria	p__Firmicutes	c__Clostridia	o__Oscillospirales	f__Acutalibacteraceae	g__CAG-180	s__CAG-180 sp000432435	GCA_000432435.1	477	477_4	F68N_bin.75.fa	F68N_bin.75.fa	97.48	0.34	354
F68R_bin.43	d__Bacteria	p__Firmicutes	c__Clostridia	o__Oscillospirales	f__Acutalibacteraceae	g__CAG-180	s__CAG-180 sp000432435	GCA_000432435.1	477	477_10	F68R_bin.43.fa	F68R_bin.43.fa	92.21	0.34	354
F68J_bin.21	d__Bacteria	p__Firmicutes	c__Clostridia	o__Oscillospirales	f__Acutalibacteraceae	g__CAG-180	s__CAG-180 sp000432435	GCA_000432435.1	477	477_5	F68V_bin.112.fa	F68J_bin.21.fa	97.63	0.67	354
F68V_bin.112	d__Bacteria	p__Firmicutes	c__Clostridia	o__Oscillospirales	f__Acutalibacteraceae	g__CAG-180	s__CAG-180 sp000432435	GCA_000432435.1	477	477_5	F68V_bin.112.fa	F68V_bin.112.fa	97.48	0.34	354
F68W_bin.102	d__Bacteria	p__Firmicutes	c__Clostridia	o__Oscillospirales	f__Acutalibacteraceae	g__CAG-180	s__CAG-180 sp000432435	GCA_000432435.1	477	477_3	F6D9_bin.53.fa	F68W_bin.102.fa	73.66	0	354

Table S3. Taxonomic classifiation of the 2,421 detected MAGs

	Domain	Phylum	Class	Order	Family	Genus	Species	RefGenome	primary_cluster	secondary_cluster	repr_genome	genome	CheckM_completeness	CheckM_contamination	
F6D9_bin.53	d__Bacteria	p__Firmicutes	c__Clostridia	o__Oscillospirales	f__Acutalibacteraceae	g__CAG-180	s__CAG-180 sp000432435	GCA_000432435.1	477	477_3	F6D9_bin.53.fa	F6D9_bin.53.fa	97.65	0.34	354
F6DH_bin.51	d__Bacteria	p__Firmicutes	c__Clostridia	o__Oscillospirales	f__Acutalibacteraceae	g__CAG-180	s__CAG-180 sp000432435	GCA_000432435.1	477	477_3	F6D9_bin.53.fa	F6DH_bin.51.fa	97.48	0.34	354
F6DA_bin.67	d__Bacteria	p__Firmicutes	c__Clostridia	o__Oscillospirales	f__Acutalibacteraceae	g__CAG-180	s__CAG-180 sp000432435	GCA_000432435.1	477	477_8	F6DA_bin.67.fa	F6DA_bin.67.fa	72.99	0	354
F6MY_bin.127	d__Bacteria	p__Firmicutes	c__Clostridia	o__Oscillospirales	f__Acutalibacteraceae	g__CAG-180	s__CAG-180 sp000432435	GCA_000432435.1	477	477_2	F6MY_bin.127.fa	F6MY_bin.127.fa	53.36	0.34	354
F68C_bin.101	d__Bacteria	p__Firmicutes	c__Clostridia	o__Oscillospirales	f__Acutalibacteraceae	g__CAG-217	s__CAG-217 sp000436335	GCA_000436335.1	507	507_4	F68C_bin.101.fa	F68C_bin.101.fa	97.32	0.34	354
F68K_bin.39	d__Bacteria	p__Firmicutes	c__Clostridia	o__Oscillospirales	f__Acutalibacteraceae	g__CAG-217	s__CAG-217 sp000436335	GCA_000436335.1	507	507_1	F68K_bin.39.fa	F68K_bin.39.fa	97.32	0	354
F68R_bin.52	d__Bacteria	p__Firmicutes	c__Clostridia	o__Oscillospirales	f__Acutalibacteraceae	g__CAG-217	s__CAG-217 sp000436335	GCA_000436335.1	507	507_9	F68R_bin.52.fa	F68R_bin.52.fa	97.32	0	354

Table S3. Taxonomic classifiation of the 2,421 detected MAGs

	Domain	Phylum	Class	Order	Family	Genus	Species	RefGenome	primary_cluster	secondary_cluster	repr_genome	genome	CheckM_completeness	CheckM_contamination	
F68T_bin.21	d__Bacteria	p__Firmicutes	c__Clostridia	o__Oscillospirales	f__Acutalibacteraceae	g__CAG-217	s__CAG-217 sp000436335	GCA_000436335.1	507	507_8	F68T_bin.21.fa	F68T_bin.21.fa	97.32	0	354
F6D7_bin.89	d__Bacteria	p__Firmicutes	c__Clostridia	o__Oscillospirales	f__Acutalibacteraceae	g__CAG-217	s__CAG-217 sp000436335	GCA_000436335.1	507	507_11	F6D7_bin.89.fa	F6D7_bin.89.fa	57.27	0	354
F6D8_bin.92	d__Bacteria	p__Firmicutes	c__Clostridia	o__Oscillospirales	f__Acutalibacteraceae	g__CAG-217	s__CAG-217 sp000436335	GCA_000436335.1	507	507_3	F6D8_bin.92.fa	F6D8_bin.92.fa	97.32	0	354
F6D9_bin.82	d__Bacteria	p__Firmicutes	c__Clostridia	o__Oscillospirales	f__Acutalibacteraceae	g__CAG-217	s__CAG-217 sp000436335	GCA_000436335.1	507	507_2	F6D9_bin.82.fa	F6D9_bin.82.fa	97.32	0	354
F6DA_bin.93	d__Bacteria	p__Firmicutes	c__Clostridia	o__Oscillospirales	f__Acutalibacteraceae	g__CAG-217	s__CAG-217 sp000436335	GCA_000436335.1	507	507_7	F6DA_bin.93.fa	F6DA_bin.93.fa	97.32	0	354
F6DG_bin.35	d__Bacteria	p__Firmicutes	c__Clostridia	o__Oscillospirales	f__Acutalibacteraceae	g__CAG-217	s__CAG-217 sp000436335	GCA_000436335.1	507	507_10	F6DG_bin.35.fa	F6DG_bin.35.fa	97.32	0	354
F68W_bin.69	d__Bacteria	p__Firmicutes	c__Clostridia	o__Oscillospirales	f__Acutalibacteraceae	g__CAG-217	s__CAG-217 sp000436335	GCA_000436335.1	507	507_5	F6DH_bin.95.fa	F68W_bin.69.fa	97.32	0.67	354

Table S3. Taxonomic classifiation of the 2,421 detected MAGs

	Domain	Phylum	Class	Order	Family	Genus	Species	RefGenome	primary_cluster	secondary_cluster	repr_genome	genome	CheckM_completeness	CheckM_contamination	
F6DH_bin.95	d__Bacteria	p__Firmicutes	c__Clostridia	o__Oscillospirales	f__Acutalibacteraceae	g__CAG-217	s__CAG-217 sp000436335	GCA_000436335.1	507	507_5	F6DH_bin.95.fa	F6DH_bin.95.fa	95.97	0	354
F6MY_bin.63	d__Bacteria	p__Firmicutes	c__Clostridia	o__Oscillospirales	f__Acutalibacteraceae	g__CAG-217	s__CAG-217 sp000436335	GCA_000436335.1	507	507_6	F6MY_bin.63.fa	F6MY_bin.63.fa	80.51	2.18	354
F68F_bin.64	d__Bacteria	p__Firmicutes	c__Clostridia	o__Oscillospirales	f__Acutalibacteraceae	g__CAG-488	s__CAG-488 sp000434055	GCA_000434055.1	259	259_4	F68F_bin.64.fa	F68F_bin.64.fa	96.42	0	354
F68G_bin.15	d__Bacteria	p__Firmicutes	c__Clostridia	o__Oscillospirales	f__Acutalibacteraceae	g__CAG-488	s__CAG-488 sp000434055	GCA_000434055.1	259	259_3	F68G_bin.15.fa	F68G_bin.15.fa	89.04	0	354
F68D_bin.1	d__Bacteria	p__Firmicutes	c__Clostridia	o__Oscillospirales	f__Acutalibacteraceae	g__CAG-488	s__CAG-488 sp000434055	GCA_000434055.1	259	259_2	F68R_bin.62.fa	F68D_bin.1.fa	88.69	1.9	354
F68R_bin.62	d__Bacteria	p__Firmicutes	c__Clostridia	o__Oscillospirales	f__Acutalibacteraceae	g__CAG-488	s__CAG-488 sp000434055	GCA_000434055.1	259	259_2	F68R_bin.62.fa	F68R_bin.62.fa	96.42	0	354
F68V_bin.84	d__Bacteria	p__Firmicutes	c__Clostridia	o__Oscillospirales	f__Acutalibacteraceae	g__CAG-488	s__CAG-488 sp000434055	GCA_000434055.1	259	259_5	F68V_bin.84.fa	F68V_bin.84.fa	96.42	0	354

Table S3. Taxonomic classifiation of the 2,421 detected MAGs

	Domain	Phylum	Class	Order	Family	Genus	Species	RefGenome	primary_cluster	secondary_cluster	repr_genome	genome	CheckM_completeness	CheckM_contamination	
F6D7_bin.86	d__Bacteria	p__Firmicutes	c__Clostridia	o__Oscillospirales	f__Acutalibacteraceae	g__CAG-488	s__CAG-488 sp000434055	GCA_000434055.1	259	259_6	F6D7_bin.86.fa	F6D7_bin.86.fa	96.42	0	354
F68W_bin.68	d__Bacteria	p__Firmicutes	c__Clostridia	o__Oscillospirales	f__Acutalibacteraceae	g__CAG-488	s__CAG-488 sp000434055	GCA_000434055.1	259	259_1	F6DH_bin.115.fa	F68W_bin.68.fa	96.42	0	354
F6DH_bin.115	d__Bacteria	p__Firmicutes	c__Clostridia	o__Oscillospirales	f__Acutalibacteraceae	g__CAG-488	s__CAG-488 sp000434055	GCA_000434055.1	259	259_1	F6DH_bin.115.fa	F6DH_bin.115.fa	96.42	0	354
F68V_bin.78	d__Bacteria	p__Firmicutes	c__Clostridia	o__Oscillospirales	f__Acutalibacteraceae	g__Clostridium_A	s__Clostridium_A leptum	GCF_000154345.1	490	490_1	F6DE_bin.3.fa	F68V_bin.78.fa	60.66	3.45	354
F6DE_bin.3	d__Bacteria	p__Firmicutes	c__Clostridia	o__Oscillospirales	f__Acutalibacteraceae	g__Clostridium_A	s__Clostridium_A leptum	GCF_000154345.1	490	490_1	F6DE_bin.3.fa	F6DE_bin.3.fa	99.33	0	354
F68J_bin.55	d__Bacteria	p__Firmicutes	c__Clostridia	o__Oscillospirales	f__Acutalibacteraceae	g__Eubacterium_R	s__Eubacterium_R sp000434995	GCA_000434995.1	446	446_1	F68J_bin.55.fa	F68J_bin.55.fa	98.32	0	354
F68V_bin.8	d__Bacteria	p__Firmicutes	c__Clostridia	o__Oscillospirales	f__Acutalibacteraceae	g__Eubacterium_R	s__Eubacterium_R sp000434995	GCA_000434995.1	446	446_2	F68V_bin.8.fa	F68V_bin.8.fa	98.66	0	354

Table S3. Taxonomic classifiation of the 2,421 detected MAGs

	Domain	Phylum	Class	Order	Family	Genus	Species	RefGenome	primary_cluster	secondary_cluster	repr_genome	genome	CheckM_completeness	CheckM_contamination	
F68F_bin.41	d__Bacteria	p__Firmicutes	c__Clostridia	o__Oscillospirales	f__Acutalibacteraceae	g__Eubacterium_R	s__Eubacterium_R sp000436835	GCA_000436835.1	447	447_1	F68F_bin.41.fa	F68F_bin.41.fa	98.66	0	354
F68K_bin.87	d__Bacteria	p__Firmicutes	c__Clostridia	o__Oscillospirales	f__Acutalibacteraceae	g__Eubacterium_R	s__Eubacterium_R sp000436835	GCA_000436835.1	447	447_1	F68F_bin.41.fa	F68K_bin.87.fa	98.66	0	354
F68M_bin.11	d__Bacteria	p__Firmicutes	c__Clostridia	o__Oscillospirales	f__Acutalibacteraceae	g__Eubacterium_R	s__Eubacterium_R sp000436835	GCA_000436835.1	447	447_1	F68F_bin.41.fa	F68M_bin.11.fa	97.09	0.02	354
F6MX_bin.65	d__Bacteria	p__Firmicutes	c__Clostridia	o__Oscillospirales	f__Acutalibacteraceae	g__Eubacterium_R	s__Eubacterium_R sp000436835	GCA_000436835.1	447	447_1	F68F_bin.41.fa	F6MX_bin.65.fa	60.4	0	354
F68N_bin.78	d__Bacteria	p__Firmicutes	c__Clostridia	o__Oscillospirales	f__Acutalibacteraceae	g__Eubacterium_R	s__Eubacterium_R sp000436835	GCA_000436835.1	447	447_9	F68N_bin.78.fa	F68N_bin.78.fa	97.99	0	354
F68U_bin.30	d__Bacteria	p__Firmicutes	c__Clostridia	o__Oscillospirales	f__Acutalibacteraceae	g__Eubacterium_R	s__Eubacterium_R sp000436835	GCA_000436835.1	447	447_5	F68U_bin.30.fa	F68U_bin.30.fa	98.66	0	354

Table S3. Taxonomic classifiation of the 2,421 detected MAGs

	Domain	Phylum	Class	Order	Family	Genus	Species	RefGenome	primary_cluster	secondary_cluster	repr_genome	genome	CheckM_completeness	CheckM_contamination	
F68W_bin.62	d__Bacteria	p__Firmicutes	c__Clostridia	o__Oscillospirales	f__Acutalibacteraceae	g__Eubacterium_R	s__Eubacterium_R sp000436835	GCA_000436835.1	447	447_5	F68U_bin.30.fa	F68W_bin.62.fa	98.66	0	354
F6DE_bin.59	d__Bacteria	p__Firmicutes	c__Clostridia	o__Oscillospirales	f__Acutalibacteraceae	g__Eubacterium_R	s__Eubacterium_R sp000436835	GCA_000436835.1	447	447_5	F68U_bin.30.fa	F6DE_bin.59.fa	98.66	0	354
F6DH_bin.76	d__Bacteria	p__Firmicutes	c__Clostridia	o__Oscillospirales	f__Acutalibacteraceae	g__Eubacterium_R	s__Eubacterium_R sp000436835	GCA_000436835.1	447	447_5	F68U_bin.30.fa	F6DH_bin.76.fa	98.66	0.02	354
F68R_bin.36	d__Bacteria	p__Firmicutes	c__Clostridia	o__Oscillospirales	f__Acutalibacteraceae	g__Eubacterium_R	s__Eubacterium_R sp000436835	GCA_000436835.1	447	447_3	F6D8_bin.11.fa	F68R_bin.36.fa	97.99	0.67	354
F6D8_bin.11	d__Bacteria	p__Firmicutes	c__Clostridia	o__Oscillospirales	f__Acutalibacteraceae	g__Eubacterium_R	s__Eubacterium_R sp000436835	GCA_000436835.1	447	447_3	F6D8_bin.11.fa	F6D8_bin.11.fa	96.64	0	354
F6D9_bin.2	d__Bacteria	p__Firmicutes	c__Clostridia	o__Oscillospirales	f__Acutalibacteraceae	g__Eubacterium_R	s__Eubacterium_R sp000436835	GCA_000436835.1	447	447_6	F6D9_bin.2.fa	F6D9_bin.2.fa	98.66	0.67	354

Table S3. Taxonomic classifiation of the 2,421 detected MAGs

	Domain	Phylum	Class	Order	Family	Genus	Species	RefGenome	primary_cluster	secondary_cluster	repr_genome	genome	CheckM_completeness	CheckM_contamination	
F6DA_bin.49	d__Bacteria	p__Firmicutes	c__Clostridia	o__Oscillospirales	f__Acutalibacteraceae	g__Eubacterium_R	s__Eubacterium_R sp000436835	GCA_000436835.1	447	447_4	F6DA_bin.49.fa	F6DA_bin.49.fa	98.66	0	354
F6DB_bin.12	d__Bacteria	p__Firmicutes	c__Clostridia	o__Oscillospirales	f__Acutalibacteraceae	g__Eubacterium_R	s__Eubacterium_R sp000436835	GCA_000436835.1	447	447_7	F6DB_bin.12.fa	F6DB_bin.12.fa	98.66	0	354
F68P_bin.22	d__Bacteria	p__Firmicutes	c__Clostridia	o__Oscillospirales	f__Acutalibacteraceae	g__Eubacterium_R	s__Eubacterium_R sp000436835	GCA_000436835.1	447	447_8	F6DG_bin.34.fa	F68P_bin.22.fa	98.66	1.34	354
F6DG_bin.34	d__Bacteria	p__Firmicutes	c__Clostridia	o__Oscillospirales	f__Acutalibacteraceae	g__Eubacterium_R	s__Eubacterium_R sp000436835	GCA_000436835.1	447	447_8	F6DG_bin.34.fa	F6DG_bin.34.fa	98.32	0	354
F6MY_bin.129	d__Bacteria	p__Firmicutes	c__Clostridia	o__Oscillospirales	f__Acutalibacteraceae	g__Eubacterium_R	s__Eubacterium_R sp000436835	GCA_000436835.1	447	447_2	F6MY_bin.129.fa	F6MY_bin.129.fa	98.66	3.02	354
F68V_bin.68	d__Bacteria	p__Firmicutes	c__Clostridia	o__Oscillospirales	f__Acutalibacteraceae	g__GCA-900066995	s__GCA-900066995 sp900291955	GCF_900291955.1	165	165_1	F68V_bin.68.fa	F68V_bin.68.fa	98.99	0.34	354

Table S3. Taxonomic classifiation of the 2,421 detected MAGs

	Domain	Phylum	Class	Order	Family	Genus	Species	RefGenome	primary_cluster	secondary_cluster	repr_genome	genome	CheckM_completeness	CheckM_contamination	
F6MX_bin.81	d__Bacteria	p__Firmicutes	c__Clostridia	o__Oscillospirales	f__Acutalibacteraceae	g__GCA-900066995	s__GCA-900066995 sp900291955	GCF_900291955.1	165	165_1	F68V_bin.68.fa	F6MX_bin.81.fa	90.91	1.76	354
F6D8_bin.75	d__Bacteria	p__Firmicutes	c__Clostridia	o__Oscillospirales	f__Acutalibacteraceae	g__GCA-900066995	s__GCA-900066995 sp900291955	GCF_900291955.1	165	165_3	F6D8_bin.75.fa	F6D8_bin.75.fa	79.19	0.34	354
F6D9_bin.65	d__Bacteria	p__Firmicutes	c__Clostridia	o__Oscillospirales	f__Acutalibacteraceae	g__GCA-900066995	s__GCA-900066995 sp900291955	GCF_900291955.1	165	165_2	F6D9_bin.65.fa	F6D9_bin.65.fa	99.33	0.34	354
F68K_bin.91	d__Bacteria	p__Firmicutes	c__Clostridia	o__Oscillospirales	f__Acutalibacteraceae	g__Ruminococcus_E	s__	N/A	167	167_6	F68K_bin.91.fa	F68K_bin.91.fa	97.32	0.34	355
F68M_bin.89	d__Bacteria	p__Firmicutes	c__Clostridia	o__Oscillospirales	f__Acutalibacteraceae	g__Ruminococcus_E	s__	N/A	167	167_1	F68M_bin.89.fa	F68M_bin.89.fa	52.14	0.83	356
F6D9_bin.77	d__Bacteria	p__Firmicutes	c__Clostridia	o__Oscillospirales	f__Acutalibacteraceae	g__Ruminococcus_E	s__	N/A	167	167_7	F6D9_bin.77.fa	F6D9_bin.77.fa	98.66	0.34	357
F6DA_bin.19	d__Bacteria	p__Firmicutes	c__Clostridia	o__Oscillospirales	f__Acutalibacteraceae	g__Ruminococcus_E	s__	N/A	167	167_8	F6DA_bin.19.fa	F6DA_bin.19.fa	97.99	0.34	358

Table S3. Taxonomic classifiation of the 2,421 detected MAGs

	Domain	Phylum	Class	Order	Family	Genus	Species	RefGenome	primary_cluster	secondary_cluster	repr_genome	genome	CheckM_completeness	CheckM_contamination	
F68C_bin.78	d__Bacteria	p__Firmicutes	c__Clostridia	o__Oscillospirales	f__Acutalibacteraceae	g__Ruminococcus_E	s__Ruminococcus_E bromii_B	GCF_002834235.1	166	166_5	F68C_bin.78.fa	F68C_bin.78.fa	78.21	1.03	358
F68F_bin.86	d__Bacteria	p__Firmicutes	c__Clostridia	o__Oscillospirales	f__Acutalibacteraceae	g__Ruminococcus_E	s__Ruminococcus_E bromii_B	GCF_002834235.1	166	166_6	F68F_bin.86.fa	F68F_bin.86.fa	76.12	2.46	358
F68G_bin.26	d__Bacteria	p__Firmicutes	c__Clostridia	o__Oscillospirales	f__Acutalibacteraceae	g__Ruminococcus_E	s__Ruminococcus_E bromii_B	GCF_002834235.1	166	166_3	F68G_bin.26.fa	F68G_bin.26.fa	93.96	1.36	358
F68H_bin.39	d__Bacteria	p__Firmicutes	c__Clostridia	o__Oscillospirales	f__Acutalibacteraceae	g__Ruminococcus_E	s__Ruminococcus_E bromii_B	GCF_002834235.1	166	166_1	F68H_bin.39.fa	F68H_bin.39.fa	97.01	2.01	358
F68V_bin.15	d__Bacteria	p__Firmicutes	c__Clostridia	o__Oscillospirales	f__Acutalibacteraceae	g__Ruminococcus_E	s__Ruminococcus_E bromii_B	GCF_002834235.1	166	166_2	F68V_bin.15.fa	F68V_bin.15.fa	99.33	1.01	358
F6DG_bin.64	d__Bacteria	p__Firmicutes	c__Clostridia	o__Oscillospirales	f__Acutalibacteraceae	g__Ruminococcus_E	s__Ruminococcus_E bromii_B	GCF_002834235.1	166	166_4	F6DG_bin.64.fa	F6DG_bin.64.fa	99.33	0.67	358

Table S3. Taxonomic classifiation of the 2,421 detected MAGs

	Domain	Phylum	Class	Order	Family	Genus	Species	RefGenome	primary_cluster	secondary_cluster	repr_genome	genome	CheckM_completeness	CheckM_contamination	
F68D_bin.39	d__Bacteria	p__Firmicutes	c__Clostridia	o__Oscillospirales	f__Acutalibacteraceae	g__Ruminococcus_E	s__Ruminococcus_E sp003526955	GCA_003526955.1	167	167_3	F68D_bin.39.fa	F68D_bin.39.fa	96.98	0.34	358
F68G_bin.61	d__Bacteria	p__Firmicutes	c__Clostridia	o__Oscillospirales	f__Acutalibacteraceae	g__Ruminococcus_E	s__Ruminococcus_E sp003526955	GCA_003526955.1	167	167_5	F68G_bin.61.fa	F68G_bin.61.fa	96.64	3.25	358
F68T_bin.106	d__Bacteria	p__Firmicutes	c__Clostridia	o__Oscillospirales	f__Acutalibacteraceae	g__Ruminococcus_E	s__Ruminococcus_E sp003526955	GCA_003526955.1	167	167_2	F68T_bin.106.fa	F68T_bin.106.fa	98.66	0.34	358
F6MX_bin.94	d__Bacteria	p__Firmicutes	c__Clostridia	o__Oscillospirales	f__Acutalibacteraceae	g__Ruminococcus_E	s__Ruminococcus_E sp003526955	GCA_003526955.1	167	167_4	F6MX_bin.94.fa	F6MX_bin.94.fa	77.07	0	358
F68T_bin.46	d__Bacteria	p__Firmicutes	c__Clostridia	o__Oscillospirales	f__Acutalibacteraceae	g__Ruminococcus_E	s__Ruminococcus_E sp900314705	GCA_900314705.1	168	168_2	F68T_bin.46.fa	F68T_bin.46.fa	98.66	0	358
F6D9_bin.87	d__Bacteria	p__Firmicutes	c__Clostridia	o__Oscillospirales	f__Acutalibacteraceae	g__Ruminococcus_E	s__Ruminococcus_E sp900314705	GCA_900314705.1	168	168_4	F6D9_bin.87.fa	F6D9_bin.87.fa	89.26	1.29	358

Table S3. Taxonomic classifiation of the 2,421 detected MAGs

	Domain	Phylum	Class	Order	Family	Genus	Species	RefGenome	primary_cluster	secondary_cluster	repr_genome	genome	CheckM_completeness	CheckM_contamination	
F6DA_bin.7	d__Bacteria	p__Firmicutes	c__Clostridia	o__Oscillospirales	f__Acutalibacteraceae	g__Ruminococcus_E	s__Ruminococcus_E sp900314705	GCA_900314705.1	168	168_1	F6DA_bin.7.fa	F6DA_bin.7.fa	97.99	0.34	358
F6MY_bin.22	d__Bacteria	p__Firmicutes	c__Clostridia	o__Oscillospirales	f__Acutalibacteraceae	g__Ruminococcus_E	s__Ruminococcus_E sp900314705	GCA_900314705.1	168	168_1	F6DA_bin.7.fa	F6MY_bin.22.fa	90.49	1.85	358
F6DB_bin.83	d__Bacteria	p__Firmicutes	c__Clostridia	o__Oscillospirales	f__Acutalibacteraceae	g__Ruminococcus_E	s__Ruminococcus_E sp900314705	GCA_900314705.1	168	168_3	F6DB_bin.83.fa	F6DB_bin.83.fa	98.66	0	358
F68G_bin.60	d__Bacteria	p__Firmicutes	c__Clostridia	o__Oscillospirales	f__Acutalibacteraceae	g__UBA1417	s__UBA1417 sp003531055	GCA_003531055.1	489	489_1	F68G_bin.60.fa	F68G_bin.60.fa	86.3	1.8	358
F68J_bin.24	d__Bacteria	p__Firmicutes	c__Clostridia	o__Oscillospirales	f__Acutalibacteraceae	g__UBA1417	s__UBA1417 sp003531055	GCA_003531055.1	489	489_4	F68J_bin.24.fa	F68J_bin.24.fa	77.93	1.79	358
F68K_bin.113	d__Bacteria	p__Firmicutes	c__Clostridia	o__Oscillospirales	f__Acutalibacteraceae	g__UBA1417	s__UBA1417 sp003531055	GCA_003531055.1	489	489_5	F68K_bin.113.fa	F68K_bin.113.fa	62.14	0.94	358
F68N_bin.10	d__Bacteria	p__Firmicutes	c__Clostridia	o__Oscillospirales	f__Acutalibacteraceae	g__UBA1417	s__UBA1417 sp003531055	GCA_003531055.1	489	489_9	F68N_bin.10.fa	F68N_bin.10.fa	79.53	1.34	358

Table S3. Taxonomic classifiation of the 2,421 detected MAGs

	Domain	Phylum	Class	Order	Family	Genus	Species	RefGenome	primary_cluster	secondary_cluster	repr_genome	genome	CheckM_completeness	CheckM_contamination	
F68P_bin.26	d__Bacteria	p__Firmicutes	c__Clostridia	o__Oscillospirales	f__Acutalibacteraceae	g__UBA1417	s__UBA1417 sp003531055	GCA_003531055.1	489	489_7	F68P_bin.26.fa	F68P_bin.26.fa	82.49	0.67	358
F68T_bin.6	d__Bacteria	p__Firmicutes	c__Clostridia	o__Oscillospirales	f__Acutalibacteraceae	g__UBA1417	s__UBA1417 sp003531055	GCA_003531055.1	489	489_6	F68T_bin.6.fa	F68T_bin.6.fa	84.8	2.01	358
F6D8_bin.94	d__Bacteria	p__Firmicutes	c__Clostridia	o__Oscillospirales	f__Acutalibacteraceae	g__UBA1417	s__UBA1417 sp003531055	GCA_003531055.1	489	489_2	F6D8_bin.94.fa	F6D8_bin.94.fa	56.79	1.68	358
F6DE_bin.47	d__Bacteria	p__Firmicutes	c__Clostridia	o__Oscillospirales	f__Acutalibacteraceae	g__UBA1417	s__UBA1417 sp003531055	GCA_003531055.1	489	489_8	F6DE_bin.47.fa	F6DE_bin.47.fa	98.66	0	358
F6MY_bin.54	d__Bacteria	p__Firmicutes	c__Clostridia	o__Oscillospirales	f__Acutalibacteraceae	g__UBA1417	s__UBA1417 sp003531055	GCA_003531055.1	489	489_8	F6DE_bin.47.fa	F6MY_bin.54.fa	91.95	0	358
F68W_bin.40	d__Bacteria	p__Firmicutes	c__Clostridia	o__Oscillospirales	f__Acutalibacteraceae	g__UBA1417	s__UBA1417 sp003531055	GCA_003531055.1	489	489_3	F6DH_bin.31.fa	F68W_bin.40.fa	90.46	0	358
F6DH_bin.31	d__Bacteria	p__Firmicutes	c__Clostridia	o__Oscillospirales	f__Acutalibacteraceae	g__UBA1417	s__UBA1417 sp003531055	GCA_003531055.1	489	489_3	F6DH_bin.31.fa	F6DH_bin.31.fa	98.32	0.81	358
F68D_bin.83	d__Bacteria	p__Firmicutes	c__Clostridia	o__Oscillospirales	f__Acutalibacteraceae	g__UBA737	s__	N/A	417	417_9	F68D_bin.83.fa	F68D_bin.83.fa	94.63	1.34	359

Table S3. Taxonomic classifiation of the 2,421 detected MAGs

	Domain	Phylum	Class	Order	Family	Genus	Species	RefGenome	primary_cluster	secondary_cluster	repr_genome	genome	CheckM_completeness	CheckM_contamination	
F68F_bin.74	d__Bacteria	p__Firmicutes	c__Clostridia	o__Oscillospirales	f__Acutalibacteraceae	g__UBA737	s__	N/A	417	417_4	F68F_bin.74.fa	F68F_bin.74.fa	92.26	1.01	360
F68M_bin.46	d__Bacteria	p__Firmicutes	c__Clostridia	o__Oscillospirales	f__Acutalibacteraceae	g__UBA737	s__	N/A	417	417_11	F68M_bin.46.fa	F68M_bin.46.fa	95.3	1.34	361
F68N_bin.30	d__Bacteria	p__Firmicutes	c__Clostridia	o__Oscillospirales	f__Acutalibacteraceae	g__UBA737	s__	N/A	417	417_6	F68N_bin.30.fa	F68N_bin.30.fa	73.79	1.72	362
F68R_bin.73	d__Bacteria	p__Firmicutes	c__Clostridia	o__Oscillospirales	f__Acutalibacteraceae	g__UBA737	s__	N/A	417	417_5	F68R_bin.73.fa	F68R_bin.73.fa	94.97	1.34	363
F68U_bin.112	d__Bacteria	p__Firmicutes	c__Clostridia	o__Oscillospirales	f__Acutalibacteraceae	g__UBA737	s__	N/A	417	417_8	F68U_bin.112.fa	F68U_bin.112.fa	57.72	3.86	364
F68W_bin.41	d__Bacteria	p__Firmicutes	c__Clostridia	o__Oscillospirales	f__Acutalibacteraceae	g__UBA737	s__	N/A	417	417_12	F68W_bin.41.fa	F68W_bin.41.fa	56.4	3.36	365
F6D9_bin.49	d__Bacteria	p__Firmicutes	c__Clostridia	o__Oscillospirales	f__Acutalibacteraceae	g__UBA737	s__	N/A	417	417_10	F6D9_bin.49.fa	F6D9_bin.49.fa	52.6	1.34	366
F6DA_bin.4	d__Bacteria	p__Firmicutes	c__Clostridia	o__Oscillospirales	f__Acutalibacteraceae	g__UBA737	s__	N/A	417	417_2	F6DA_bin.4.fa	F6DA_bin.4.fa	80.57	0.24	367
F6DE_bin.54	d__Bacteria	p__Firmicutes	c__Clostridia	o__Oscillospirales	f__Acutalibacteraceae	g__UBA737	s__	N/A	417	417_3	F6DE_bin.54.fa	F6DE_bin.54.fa	93.29	2.01	368
F6MX_bin.70	d__Bacteria	p__Firmicutes	c__Clostridia	o__Oscillospirales	f__Acutalibacteraceae	g__UBA737	s__	N/A	417	417_7	F6MX_bin.70.fa	F6MX_bin.70.fa	90.81	1.34	369

Table S3. Taxonomic classifiation of the 2,421 detected MAGs

	Domain	Phylum	Class	Order	Family	Genus	Species	RefGenome	primary_cluster	secondary_cluster	repr_genome	genome	CheckM_completeness	CheckM_contamination	
F68J_bin.2	d__Bacteria	p__Firmicutes	c__Clostridia	o__Oscillospirales	f__Acutalibacteraceae	g__UBA737	s__	N/A	417	417_1	F6MY_bin.40.fa	F68J_bin.2.fa	74.9	1.73	370
F6MY_bin.40	d__Bacteria	p__Firmicutes	c__Clostridia	o__Oscillospirales	f__Acutalibacteraceae	g__UBA737	s__	N/A	417	417_1	F6MY_bin.40.fa	F6MY_bin.40.fa	94.63	1.34	371
F6D7_bin.4	d__Bacteria	p__Firmicutes	c__Clostridia	o__Oscillospirales	f__Acutalibacteraceae	g__UBA737	s__	N/A	508	508_0	F6D7_bin.4.fa	F6D7_bin.4.fa	95.97	0	372
F68J_bin.36	d__Bacteria	p__Firmicutes	c__Clostridia	o__Oscillospirales	f__Butyricicoccaceae	g__	s__	N/A	434	434_0	F68J_bin.36.fa	F68J_bin.36.fa	82.91	1.45	373
F6MY_bin.84	d__Bacteria	p__Firmicutes	c__Clostridia	o__Oscillospirales	f__Butyricicoccaceae	g__	s__	N/A	566	566_0	F6MY_bin.84.fa	F6MY_bin.84.fa	74.35	0.17	374
F68J_bin.54	d__Bacteria	p__Firmicutes	c__Clostridia	o__Oscillospirales	f__Butyricicoccaceae	g__Agathobaculum	s__	N/A	491	491_2	F68J_bin.54.fa	F68J_bin.54.fa	82.34	1.81	375
F68F_bin.69	d__Bacteria	p__Firmicutes	c__Clostridia	o__Oscillospirales	f__Butyricicoccaceae	g__Agathobaculum	s__	N/A	491	491_1	F6DA_bin.87.fa	F68F_bin.69.fa	87.02	0.34	376
F6DA_bin.87	d__Bacteria	p__Firmicutes	c__Clostridia	o__Oscillospirales	f__Butyricicoccaceae	g__Agathobaculum	s__	N/A	491	491_1	F6DA_bin.87.fa	F6DA_bin.87.fa	92.36	0	377
F68E_bin.24	d__Bacteria	p__Firmicutes	c__Clostridia	o__Oscillospirales	f__Butyricicoccaceae	g__Agathobaculum	s__Agathobaculum butyriciproducens	GCA_003096535.1	492	492_9	F68E_bin.24.fa	F68E_bin.24.fa	82.22	3.41	377

Table S3. Taxonomic classifiation of the 2,421 detected MAGs

	Domain	Phylum	Class	Order	Family	Genus	Species	RefGenome	primary_cluster	secondary_cluster	repr_genome	genome	CheckM_completeness	CheckM_contamination	
F68F_bin.17	d__Bacteria	p__Firmicutes	c__Clostridia	o__Oscillospirales	f__Butyricicoccaceae	g__Agathobaculum	s__Agathobaculum butyriciproducens	GCA_003096535.1	492	492_3	F68F_bin.17.fa	F68F_bin.17.fa	75.59	3.24	377
F68K_bin.82	d__Bacteria	p__Firmicutes	c__Clostridia	o__Oscillospirales	f__Butyricicoccaceae	g__Agathobaculum	s__Agathobaculum butyriciproducens	GCA_003096535.1	492	492_4	F68K_bin.82.fa	F68K_bin.82.fa	96.63	0.67	377
F68P_bin.37	d__Bacteria	p__Firmicutes	c__Clostridia	o__Oscillospirales	f__Butyricicoccaceae	g__Agathobaculum	s__Agathobaculum butyriciproducens	GCA_003096535.1	492	492_5	F68P_bin.37.fa	F68P_bin.37.fa	76.21	2.07	377
F68T_bin.105	d__Bacteria	p__Firmicutes	c__Clostridia	o__Oscillospirales	f__Butyricicoccaceae	g__Agathobaculum	s__Agathobaculum butyriciproducens	GCA_003096535.1	492	492_1	F68T_bin.105.fa	F68T_bin.105.fa	58.52	3.14	377
F6D9_bin.41	d__Bacteria	p__Firmicutes	c__Clostridia	o__Oscillospirales	f__Butyricicoccaceae	g__Agathobaculum	s__Agathobaculum butyriciproducens	GCA_003096535.1	492	492_7	F6D9_bin.41.fa	F6D9_bin.41.fa	97.44	0.67	377

Table S3. Taxonomic classifiation of the 2,421 detected MAGs

	Domain	Phylum	Class	Order	Family	Genus	Species	RefGenome	primary_cluster	secondary_cluster	repr_genome	genome	CheckM_completeness	CheckM_contamination	
F6DG_bin.51	d__Bacteria	p__Firmicutes	c__Clostridia	o__Oscillospirales	f__Butyricicoccaceae	g__Agathobaculum	s__Agathobaculum butyriciproducens	GCA_003096535.1	492	492_8	F6DG_bin.51.fa	F6DG_bin.51.fa	98.66	0.67	377
F6DH_bin.92	d__Bacteria	p__Firmicutes	c__Clostridia	o__Oscillospirales	f__Butyricicoccaceae	g__Agathobaculum	s__Agathobaculum butyriciproducens	GCA_003096535.1	492	492_2	F6DH_bin.92.fa	F6DH_bin.92.fa	56.11	1.01	377
F6MY_bin.55	d__Bacteria	p__Firmicutes	c__Clostridia	o__Oscillospirales	f__Butyricicoccaceae	g__Agathobaculum	s__Agathobaculum butyriciproducens	GCA_003096535.1	492	492_6	F6MY_bin.55.fa	F6MY_bin.55.fa	78.15	2.13	377
F68J_bin.86	d__Bacteria	p__Firmicutes	c__Clostridia	o__Oscillospirales	f__CAG-272	g__	s__	N/A	278	278_2	F68J_bin.86.fa	F68J_bin.86.fa	55.46	0.56	378
F68R_bin.71	d__Bacteria	p__Firmicutes	c__Clostridia	o__Oscillospirales	f__CAG-272	g__	s__	N/A	278	278_3	F68R_bin.71.fa	F68R_bin.71.fa	95.41	0.34	379
F68W_bin.61	d__Bacteria	p__Firmicutes	c__Clostridia	o__Oscillospirales	f__CAG-272	g__	s__	N/A	278	278_5	F68W_bin.61.fa	F68W_bin.61.fa	81.97	1.62	380
F6D7_bin.51	d__Bacteria	p__Firmicutes	c__Clostridia	o__Oscillospirales	f__CAG-272	g__	s__	N/A	278	278_1	F6D7_bin.51.fa	F6D7_bin.51.fa	96.53	0.68	381

Table S3. Taxonomic classifiation of the 2,421 detected MAGs

	Domain	Phylum	Class	Order	Family	Genus	Species	RefGenome	primary_cluster	secondary_cluster	repr_genome	genome	CheckM_completeness	CheckM_contamination	
F6MY_bin.80	d__Bacteria	p__Firmicutes	c__Clostridia	o__Oscillospirales	f__CAG-272	g__	s__	N/A	278	278_1	F6D7_bin.51.fa	F6MY_bin.80.fa	53.25	0.56	382
F6DA_bin.55	d__Bacteria	p__Firmicutes	c__Clostridia	o__Oscillospirales	f__CAG-272	g__	s__	N/A	278	278_4	F6DA_bin.55.fa	F6DA_bin.55.fa	94.57	0.17	383
F68C_bin.19	d__Bacteria	p__Firmicutes	c__Clostridia	o__Oscillospirales	f__CAG-272	g__	s__	N/A	336	336_4	F68C_bin.19.fa	F68C_bin.19.fa	91.55	3.36	384
F68G_bin.70	d__Bacteria	p__Firmicutes	c__Clostridia	o__Oscillospirales	f__CAG-272	g__	s__	N/A	336	336_2	F68G_bin.70.fa	F68G_bin.70.fa	87.76	1.36	385
F68H_bin.36	d__Bacteria	p__Firmicutes	c__Clostridia	o__Oscillospirales	f__CAG-272	g__	s__	N/A	336	336_3	F6DE_bin.68.fa	F68H_bin.36.fa	70.08	3.32	386
F68R_bin.24	d__Bacteria	p__Firmicutes	c__Clostridia	o__Oscillospirales	f__CAG-272	g__	s__	N/A	336	336_3	F6DE_bin.68.fa	F68R_bin.24.fa	85.4	0.27	387
F68V_bin.18	d__Bacteria	p__Firmicutes	c__Clostridia	o__Oscillospirales	f__CAG-272	g__	s__	N/A	336	336_3	F6DE_bin.68.fa	F68V_bin.18.fa	84.08	2.01	388
F6DA_bin.98	d__Bacteria	p__Firmicutes	c__Clostridia	o__Oscillospirales	f__CAG-272	g__	s__	N/A	336	336_3	F6DE_bin.68.fa	F6DA_bin.98.fa	51.28	0.12	389
F6DE_bin.68	d__Bacteria	p__Firmicutes	c__Clostridia	o__Oscillospirales	f__CAG-272	g__	s__	N/A	336	336_3	F6DE_bin.68.fa	F6DE_bin.68.fa	91.72	0	390
F68W_bin.99	d__Bacteria	p__Firmicutes	c__Clostridia	o__Oscillospirales	f__CAG-272	g__	s__	N/A	336	336_1	F6DH_bin.29.fa	F68W_bin.99.fa	89.54	0	391

Table S3. Taxonomic classifiation of the 2,421 detected MAGs

	Domain	Phylum	Class	Order	Family	Genus	Species	RefGenome	primary_cluster	secondary_cluster	repr_genome	genome	CheckM_completeness	CheckM_contamination	
F6DH_bin.29	d__Bacteria	p__Firmicutes	c__Clostridia	o__Oscillospirales	f__CAG-272	g__	s__	N/A	336	336_1	F6DH_bin.29.fa	F6DH_bin.29.fa	91.55	0	392
F6MX_bin.125	d__Bacteria	p__Firmicutes	c__Clostridia	o__Oscillospirales	f__CAG-272	g__	s__	N/A	336	336_5	F6MX_bin.125.fa	F6MX_bin.125.fa	90.99	2.01	393
F68M_bin.8	d__Bacteria	p__Firmicutes	c__Clostridia	o__Oscillospirales	f__CAG-272	g__	s__	N/A	344	344_1	F68M_bin.8.fa	F68M_bin.8.fa	71.19	3.44	394
F6MY_bin.15	d__Bacteria	p__Firmicutes	c__Clostridia	o__Oscillospirales	f__CAG-272	g__	s__	N/A	344	344_2	F6MY_bin.15.fa	F6MY_bin.15.fa	94.41	0.67	395
F68D_bin.27	d__Bacteria	p__Firmicutes	c__Clostridia	o__Oscillospirales	f__CAG-272	g__	s__	N/A	345	345_0	F68D_bin.27.fa	F68D_bin.27.fa	84.96	0.67	396
F68C_bin.112	d__Bacteria	p__Firmicutes	c__Clostridia	o__Oscillospirales	f__CAG-272	g__	s__	N/A	362	362_4	F68C_bin.112.fa	F68C_bin.112.fa	53.71	0.67	397
F68J_bin.72	d__Bacteria	p__Firmicutes	c__Clostridia	o__Oscillospirales	f__CAG-272	g__	s__	N/A	362	362_2	F68J_bin.72.fa	F68J_bin.72.fa	94.57	0.81	398
F68R_bin.16	d__Bacteria	p__Firmicutes	c__Clostridia	o__Oscillospirales	f__CAG-272	g__	s__	N/A	362	362_2	F68J_bin.72.fa	F68R_bin.16.fa	88.9	1.34	399
F6D7_bin.17	d__Bacteria	p__Firmicutes	c__Clostridia	o__Oscillospirales	f__CAG-272	g__	s__	N/A	362	362_3	F6D7_bin.17.fa	F6D7_bin.17.fa	93.4	2.01	400
F68W_bin.111	d__Bacteria	p__Firmicutes	c__Clostridia	o__Oscillospirales	f__CAG-272	g__	s__	N/A	362	362_1	F6DH_bin.116.fa	F68W_bin.111.fa	54.38	1.49	401

Table S3. Taxonomic classifiation of the 2,421 detected MAGs

	Domain	Phylum	Class	Order	Family	Genus	Species	RefGenome	primary_cluster	secondary_cluster	repr_genome	genome	CheckM_completeness	CheckM_contamination	
F6DH_bin.116	d__Bacteria	p__Firmicutes	c__Clostridia	o__Oscillospirales	f__CAG-272	g__	s__	N/A	362	362_1	F6DH_bin.116.fa	F6DH_bin.116.fa	94.91	0.67	402
F68V_bin.47	d__Bacteria	p__Firmicutes	c__Clostridia	o__Oscillospirales	f__CAG-272	g__	s__	N/A	413	413_0	F68V_bin.47.fa	F68V_bin.47.fa	92.48	2.68	403
F68G_bin.75	d__Bacteria	p__Firmicutes	c__Clostridia	o__Oscillospirales	f__CAG-272	g__	s__	N/A	429	429_4	F68G_bin.75.fa	F68G_bin.75.fa	87.91	0.22	404
F68D_bin.60	d__Bacteria	p__Firmicutes	c__Clostridia	o__Oscillospirales	f__CAG-272	g__	s__	N/A	429	429_1	F68M_bin.42.fa	F68D_bin.60.fa	86.09	1.74	405
F68M_bin.42	d__Bacteria	p__Firmicutes	c__Clostridia	o__Oscillospirales	f__CAG-272	g__	s__	N/A	429	429_1	F68M_bin.42.fa	F68M_bin.42.fa	97.54	0.42	406
F6DA_bin.63	d__Bacteria	p__Firmicutes	c__Clostridia	o__Oscillospirales	f__CAG-272	g__	s__	N/A	429	429_1	F68M_bin.42.fa	F6DA_bin.63.fa	91.89	0.84	407
F68R_bin.8	d__Bacteria	p__Firmicutes	c__Clostridia	o__Oscillospirales	f__CAG-272	g__	s__	N/A	429	429_5	F68R_bin.8.fa	F68R_bin.8.fa	86.4	0.47	408
F6MX_bin.60	d__Bacteria	p__Firmicutes	c__Clostridia	o__Oscillospirales	f__CAG-272	g__	s__	N/A	429	429_2	F6MX_bin.60.fa	F6MX_bin.60.fa	94.74	0	409
F6MY_bin.123	d__Bacteria	p__Firmicutes	c__Clostridia	o__Oscillospirales	f__CAG-272	g__	s__	N/A	429	429_3	F6MY_bin.123.fa	F6MY_bin.123.fa	93.68	0.69	410
F6D7_bin.66	d__Bacteria	p__Firmicutes	c__Clostridia	o__Oscillospirales	f__CAG-272	g__	s__	N/A	435	435_0	F6D7_bin.66.fa	F6D7_bin.66.fa	88.37	1.42	411

Table S3. Taxonomic classifiation of the 2,421 detected MAGs

	Domain	Phylum	Class	Order	Family	Genus	Species	RefGenome	primary_cluster	secondary_cluster	repr_genome	genome	CheckM_completeness	CheckM_contamination	
F6D8_bin.51	d__Bacteria	p__Firmicutes	c__Clostridia	o__Oscillospirales	f__CAG-272	g__	s__	N/A	455	455_1	F6D8_bin.51.fa	F6D8_bin.51.fa	94.41	1.57	412
F6DB_bin.49	d__Bacteria	p__Firmicutes	c__Clostridia	o__Oscillospirales	f__CAG-272	g__	s__	N/A	455	455_1	F6D8_bin.51.fa	F6DB_bin.49.fa	86.11	0	413
F6DG_bin.10	d__Bacteria	p__Firmicutes	c__Clostridia	o__Oscillospirales	f__CAG-272	g__	s__	N/A	455	455_2	F6DG_bin.10.fa	F6DG_bin.10.fa	86.58	1.01	414
F6DB_bin.59	d__Bacteria	p__Firmicutes	c__Clostridia	o__Oscillospirales	f__CAG-272	g__	s__	N/A	469	469_0	F6DB_bin.59.fa	F6DB_bin.59.fa	68.62	2.65	415
F68V_bin.72	d__Bacteria	p__Firmicutes	c__Clostridia	o__Oscillospirales	f__CAG-272	g__CAG-272	s__CAG-272 sp000433515	GCA_000433515.1	358	358_0	F68V_bin.72.fa	F68V_bin.72.fa	94.13	0.67	415
F68J_bin.47	d__Bacteria	p__Firmicutes	c__Clostridia	o__Oscillospirales	f__CAG-272	g__CAG-390	s__CAG-390 sp000437015	GCA_000437015.1	481	481_0	F68J_bin.47.fa	F68J_bin.47.fa	88.48	0.73	415
F68C_bin.104	d__Bacteria	p__Firmicutes	c__Clostridia	o__Oscillospirales	f__CAG-272	g__CAG-448	s__CAG-448 sp003150135	GCA_003150135.1	427	427_2	F68C_bin.104.fa	F68C_bin.104.fa	93.34	0	415
F68D_bin.18	d__Bacteria	p__Firmicutes	c__Clostridia	o__Oscillospirales	f__CAG-272	g__CAG-448	s__CAG-448 sp003150135	GCA_003150135.1	427	427_8	F68D_bin.18.fa	F68D_bin.18.fa	88.63	2.35	415

Table S3. Taxonomic classifiation of the 2,421 detected MAGs

	Domain	Phylum	Class	Order	Family	Genus	Species	RefGenome	primary_cluster	secondary_cluster	repr_genome	genome	CheckM_completeness	CheckM_contamination	
F68F_bin.49	d__Bacteria	p__Firmicutes	c__Clostridia	o__Oscillospirales	f__CAG-272	g__CAG-448	s__CAG-448 sp003150135	GCA_003150135.1	427	427_3	F68F_bin.49.fa	F68F_bin.49.fa	80.98	2.37	415
F68G_bin.10	d__Bacteria	p__Firmicutes	c__Clostridia	o__Oscillospirales	f__CAG-272	g__CAG-448	s__CAG-448 sp003150135	GCA_003150135.1	427	427_4	F68G_bin.10.fa	F68G_bin.10.fa	92.21	0.67	415
F68H_bin.49	d__Bacteria	p__Firmicutes	c__Clostridia	o__Oscillospirales	f__CAG-272	g__CAG-448	s__CAG-448 sp003150135	GCA_003150135.1	427	427_6	F68H_bin.49.fa	F68H_bin.49.fa	95.19	1.51	415
F68J_bin.87	d__Bacteria	p__Firmicutes	c__Clostridia	o__Oscillospirales	f__CAG-272	g__CAG-448	s__CAG-448 sp003150135	GCA_003150135.1	427	427_5	F68J_bin.87.fa	F68J_bin.87.fa	62.47	0.06	415
F6DA_bin.53	d__Bacteria	p__Firmicutes	c__Clostridia	o__Oscillospirales	f__CAG-272	g__CAG-448	s__CAG-448 sp003150135	GCA_003150135.1	427	427_7	F6DA_bin.53.fa	F6DA_bin.53.fa	92.51	0	415
F6D7_bin.24	d__Bacteria	p__Firmicutes	c__Clostridia	o__Oscillospirales	f__CAG-272	g__CAG-448	s__CAG-448 sp003150135	GCA_003150135.1	427	427_1	F6MX_bin.58.fa	F6D7_bin.24.fa	94.55	0	415
F6MX_bin.58	d__Bacteria	p__Firmicutes	c__Clostridia	o__Oscillospirales	f__CAG-272	g__CAG-448	s__CAG-448 sp003150135	GCA_003150135.1	427	427_1	F6MX_bin.58.fa	F6MX_bin.58.fa	95.36	0	415

Table S3. Taxonomic classifiation of the 2,421 detected MAGs

	Domain	Phylum	Class	Order	Family	Genus	Species	RefGenome	primary_cluster	secondary_cluster	repr_genome	genome	CheckM_completeness	CheckM_contamination	
F6MY_bin.103	d__Bacteria	p__Firmicutes	c__Clostridia	o__Oscillospirales	f__CAG-272	g__CAG-448	s__CAG-448 sp003150135	GCA_003150135.1	427	427_9	F6MY_bin.103.fa	F6MY_bin.103.fa	94.18	0	415
F6MX_bin.122	d__Bacteria	p__Firmicutes	c__Clostridia	o__Oscillospirales	f__CAG-272	g__CAG-724	s__CAG-724 sp003524145	GCA_003524145.1	405	405_0	F6MX_bin.122.fa	F6MX_bin.122.fa	88.14	0	415
F68F_bin.111	d__Bacteria	p__Firmicutes	c__Clostridia	o__Oscillospirales	f__CAG-272	g__CAG-841	s__	N/A	431	431_0	F68F_bin.111.fa	F68F_bin.111.fa	91.83	0	416
F68C_bin.26	d__Bacteria	p__Firmicutes	c__Clostridia	o__Oscillospirales	f__CAG-272	g__Firm-07	s__	N/A	436	436_1	F68C_bin.26.fa	F68C_bin.26.fa	90.91	1.7	417
F68F_bin.101	d__Bacteria	p__Firmicutes	c__Clostridia	o__Oscillospirales	f__CAG-272	g__Firm-07	s__	N/A	436	436_3	F68F_bin.101.fa	F68F_bin.101.fa	95.08	0.69	418
F68R_bin.110	d__Bacteria	p__Firmicutes	c__Clostridia	o__Oscillospirales	f__CAG-272	g__Firm-07	s__	N/A	436	436_4	F68R_bin.110.fa	F68R_bin.110.fa	93.09	0.67	419
F6D8_bin.79	d__Bacteria	p__Firmicutes	c__Clostridia	o__Oscillospirales	f__CAG-272	g__Firm-07	s__	N/A	436	436_2	F6D8_bin.79.fa	F6D8_bin.79.fa	80.27	2.59	420
F6DA_bin.16	d__Bacteria	p__Firmicutes	c__Clostridia	o__Oscillospirales	f__CAG-272	g__UBA11512	s__UBA11512 sp003522145	GCA_003522145.1	444	444_1	F6MX_bin.30.fa	F6DA_bin.16.fa	90.88	1.45	420
F6MX_bin.30	d__Bacteria	p__Firmicutes	c__Clostridia	o__Oscillospirales	f__CAG-272	g__UBA11512	s__UBA11512 sp003522145	GCA_003522145.1	444	444_1	F6MX_bin.30.fa	F6MX_bin.30.fa	89.14	0	420

Table S3. Taxonomic classifiation of the 2,421 detected MAGs

	Domain	Phylum	Class	Order	Family	Genus	Species	RefGenome	primary_cluster	secondary_cluster	repr_genome	genome	CheckM_completeness	CheckM_contamination	
F68J_bin.4	d__Bacteria	p__Firmicutes	c__Clostridia	o__Oscillospirales	f__CAG-272	g__UBA1740	s__	N/A	411	411_0	F68J_bin.4.fa	F68J_bin.4.fa	89.82	0.71	421
F68F_bin.87	d__Bacteria	p__Firmicutes	c__Clostridia	o__Oscillospirales	f__CAG-382	g__	s__	N/A	406	406_0	F68F_bin.87.fa	F68F_bin.87.fa	66.26	2.07	422
F68W_bin.83	d__Bacteria	p__Firmicutes	c__Clostridia	o__Oscillospirales	f__CAG-382	g__	s__	N/A	407	407_0	F68W_bin.83.fa	F68W_bin.83.fa	79.62	3.95	423
F6DG_bin.107	d__Bacteria	p__Firmicutes	c__Clostridia	o__Oscillospirales	f__CAG-382	g__	s__	N/A	408	408_0	F6DG_bin.107.fa	F6DG_bin.107.fa	55.3	3.52	424
F6MX_bin.20	d__Bacteria	p__Firmicutes	c__Clostridia	o__Oscillospirales	f__CAG-382	g__	s__	N/A	414	414_0	F6MX_bin.20.fa	F6MX_bin.20.fa	90.44	0	425
F68G_bin.63	d__Bacteria	p__Firmicutes	c__Clostridia	o__Oscillospirales	f__CAG-382	g__UBA1206	s__UBA1206 sp000433115	GCA_000433115.1	430	430_1	F68G_bin.63.fa	F68G_bin.63.fa	92.04	0.67	425
F68H_bin.81	d__Bacteria	p__Firmicutes	c__Clostridia	o__Oscillospirales	f__CAG-382	g__UBA1206	s__UBA1206 sp000433115	GCA_000433115.1	430	430_1	F68G_bin.63.fa	F68H_bin.81.fa	56.68	0.36	425
F68M_bin.19	d__Bacteria	p__Firmicutes	c__Clostridia	o__Oscillospirales	f__CAG-382	g__UCG-010	s__UCG-010 sp003150115	GCA_003150115.1	659	659_1	F68M_bin.19.fa	F68M_bin.19.fa	86.46	1.59	425
F6MX_bin.57	d__Bacteria	p__Firmicutes	c__Clostridia	o__Oscillospirales	f__CAG-382	g__UCG-010	s__UCG-010 sp003150115	GCA_003150115.1	659	659_1	F68M_bin.19.fa	F6MX_bin.57.fa	50.94	0	425

Table S3. Taxonomic classifiation of the 2,421 detected MAGs

	Domain	Phylum	Class	Order	Family	Genus	Species	RefGenome	primary_cluster	secondary_cluster	repr_genome	genome	CheckM_completeness	CheckM_contamination	
F68F_bin.30	d__Bacteria	p__Firmicutes	c__Clostridia	o__Oscillospirales	f__Oscillospiraceae	g__	s__	N/A	607	607_1	F68F_bin.30.fa	F68F_bin.30.fa	96.64	0.67	426
F68R_bin.32	d__Bacteria	p__Firmicutes	c__Clostridia	o__Oscillospirales	f__Oscillospiraceae	g__	s__	N/A	607	607_1	F68F_bin.30.fa	F68R_bin.32.fa	61.32	4.66	427
F68J_bin.17	d__Bacteria	p__Firmicutes	c__Clostridia	o__Oscillospirales	f__Oscillospiraceae	g__	s__	N/A	664	664_0	F68J_bin.17.fa	F68J_bin.17.fa	73.6	1.51	428
F68J_bin.65	d__Bacteria	p__Firmicutes	c__Clostridia	o__Oscillospirales	f__Oscillospiraceae	g__	s__	N/A	633	633_0	F68J_bin.65.fa	F68J_bin.65.fa	99.33	0	429
F68J_bin.84	d__Bacteria	p__Firmicutes	c__Clostridia	o__Oscillospirales	f__Oscillospiraceae	g__	s__	N/A	657	657_0	F68J_bin.84.fa	F68J_bin.84.fa	55.16	1.72	430
F68U_bin.104	d__Bacteria	p__Firmicutes	c__Clostridia	o__Oscillospirales	f__Oscillospiraceae	g__	s__	N/A	606	606_1	F68U_bin.104.fa	F68U_bin.104.fa	88.5	4.36	431
F6D8_bin.68	d__Bacteria	p__Firmicutes	c__Clostridia	o__Oscillospirales	f__Oscillospiraceae	g__	s__	N/A	606	606_2	F6D8_bin.68.fa	F6D8_bin.68.fa	99.33	1.01	432
F6MX_bin.37	d__Bacteria	p__Firmicutes	c__Clostridia	o__Oscillospirales	f__Oscillospiraceae	g__	s__	N/A	639	639_0	F6MX_bin.37.fa	F6MX_bin.37.fa	52.59	2.35	433
F68C_bin.62	d__Bacteria	p__Firmicutes	c__Clostridia	o__Oscillospirales	f__Oscillospiraceae	g__CAG-103	s__	N/A	652	652_4	F68C_bin.62.fa	F68C_bin.62.fa	94.23	0.67	434
F68P_bin.17	d__Bacteria	p__Firmicutes	c__Clostridia	o__Oscillospirales	f__Oscillospiraceae	g__CAG-103	s__	N/A	652	652_1	F68P_bin.17.fa	F68P_bin.17.fa	80.54	0	435

Table S3. Taxonomic classifiation of the 2,421 detected MAGs

	Domain	Phylum	Class	Order	Family	Genus	Species	RefGenome	primary_cluster	secondary_cluster	repr_genome	genome	CheckM_completeness	CheckM_contamination	
F68R_bin.75	d__Bacteria	p__Firmicutes	c__Clostridia	o__Oscillospirales	f__Oscillospiraceae	g__CAG-103	s__	N/A	652	652_5	F68R_bin.75.fa	F68R_bin.75.fa	90.6	0.67	436
F68T_bin.58	d__Bacteria	p__Firmicutes	c__Clostridia	o__Oscillospirales	f__Oscillospiraceae	g__CAG-103	s__	N/A	652	652_7	F68T_bin.58.fa	F68T_bin.58.fa	87.75	1.23	437
F68U_bin.49	d__Bacteria	p__Firmicutes	c__Clostridia	o__Oscillospirales	f__Oscillospiraceae	g__CAG-103	s__	N/A	652	652_3	F68U_bin.49.fa	F68U_bin.49.fa	96.31	0.67	438
F68W_bin.35	d__Bacteria	p__Firmicutes	c__Clostridia	o__Oscillospirales	f__Oscillospiraceae	g__CAG-103	s__	N/A	652	652_3	F68U_bin.49.fa	F68W_bin.35.fa	58.57	3.76	439
F6DH_bin.86	d__Bacteria	p__Firmicutes	c__Clostridia	o__Oscillospirales	f__Oscillospiraceae	g__CAG-103	s__	N/A	652	652_3	F68U_bin.49.fa	F6DH_bin.86.fa	59.23	3.69	440
F68V_bin.29	d__Bacteria	p__Firmicutes	c__Clostridia	o__Oscillospirales	f__Oscillospiraceae	g__CAG-103	s__	N/A	652	652_9	F68V_bin.29.fa	F68V_bin.29.fa	72.22	1.17	441
F6DB_bin.22	d__Bacteria	p__Firmicutes	c__Clostridia	o__Oscillospirales	f__Oscillospiraceae	g__CAG-103	s__	N/A	652	652_8	F6DB_bin.22.fa	F6DB_bin.22.fa	87.32	1.4	442
F6DB_bin.5	d__Bacteria	p__Firmicutes	c__Clostridia	o__Oscillospirales	f__Oscillospiraceae	g__CAG-103	s__	N/A	652	652_16	F6DB_bin.5.fa	F6DB_bin.5.fa	89.53	0.73	443
F68F_bin.6	d__Bacteria	p__Firmicutes	c__Clostridia	o__Oscillospirales	f__Oscillospiraceae	g__CAG-103	s__	N/A	652	652_6	F6MX_bin.3.fa	F68F_bin.6.fa	86.24	3.36	444
F6MX_bin.3	d__Bacteria	p__Firmicutes	c__Clostridia	o__Oscillospirales	f__Oscillospiraceae	g__CAG-103	s__	N/A	652	652_6	F6MX_bin.3.fa	F6MX_bin.3.fa	86.91	0.67	445

Table S3. Taxonomic classifiation of the 2,421 detected MAGs

	Domain	Phylum	Class	Order	Family	Genus	Species	RefGenome	primary_cluster	secondary_cluster	repr_genome	genome	CheckM_completeness	CheckM_contamination	
F6MY_bin.76	d__Bacteria	p__Firmicutes	c__Clostridia	o__Oscillospirales	f__Oscillospiraceae	g__CAG-103	s__	N/A	652	652_2	F6MY_bin.76.fa	F6MY_bin.76.fa	80.96	1.68	446
F68K_bin.103	d__Bacteria	p__Firmicutes	c__Clostridia	o__Oscillospirales	f__Oscillospiraceae	g__CAG-103	s__CAG-103 sp000432375	GCA_000432375.1	652	652_13	F68K_bin.103.fa	F68K_bin.103.fa	90.94	0	446
F6D8_bin.64	d__Bacteria	p__Firmicutes	c__Clostridia	o__Oscillospirales	f__Oscillospiraceae	g__CAG-103	s__CAG-103 sp000432375	GCA_000432375.1	652	652_10	F6D8_bin.64.fa	F6D8_bin.64.fa	93.29	0.34	446
F6DG_bin.53	d__Bacteria	p__Firmicutes	c__Clostridia	o__Oscillospirales	f__Oscillospiraceae	g__CAG-103	s__CAG-103 sp000432375	GCA_000432375.1	652	652_10	F6D8_bin.64.fa	F6DG_bin.53.fa	84.56	4.36	446
F68F_bin.8	d__Bacteria	p__Firmicutes	c__Clostridia	o__Oscillospirales	f__Oscillospiraceae	g__CAG-103	s__CAG-103 sp000432375	GCA_000432375.1	652	652_11	F6D9_bin.85.fa	F68F_bin.8.fa	87.99	1.89	446
F68W_bin.64	d__Bacteria	p__Firmicutes	c__Clostridia	o__Oscillospirales	f__Oscillospiraceae	g__CAG-103	s__CAG-103 sp000432375	GCA_000432375.1	652	652_11	F6D9_bin.85.fa	F68W_bin.64.fa	83.64	2.01	446
F6D9_bin.85	d__Bacteria	p__Firmicutes	c__Clostridia	o__Oscillospirales	f__Oscillospiraceae	g__CAG-103	s__CAG-103 sp000432375	GCA_000432375.1	652	652_11	F6D9_bin.85.fa	F6D9_bin.85.fa	82.21	0	446
F6DH_bin.75	d__Bacteria	p__Firmicutes	c__Clostridia	o__Oscillospirales	f__Oscillospiraceae	g__CAG-103	s__CAG-103 sp000432375	GCA_000432375.1	652	652_11	F6D9_bin.85.fa	F6DH_bin.75.fa	78.41	1.81	446

Table S3. Taxonomic classifiation of the 2,421 detected MAGs

	Domain	Phylum	Class	Order	Family	Genus	Species	RefGenome	primary_cluster	secondary_cluster	repr_genome	genome	CheckM_completeness	CheckM_contamination	
F6DA_bin.27	d__Bacteria	p__Firmicutes	c__Clostridia	o__Oscillospirales	f__Oscillospiraceae	g__CAG-103	s__CAG-103 sp000432375	GCA_000432375.1	652	652_14	F6DA_bin.27.fa	F6DA_bin.27.fa	88.28	1.34	446
F6MY_bin.93	d__Bacteria	p__Firmicutes	c__Clostridia	o__Oscillospirales	f__Oscillospiraceae	g__CAG-103	s__CAG-103 sp000432375	GCA_000432375.1	652	652_12	F6MY_bin.93.fa	F6MY_bin.93.fa	89.57	4.76	446
F68H_bin.26	d__Bacteria	p__Firmicutes	c__Clostridia	o__Oscillospirales	f__Oscillospiraceae	g__CAG-103	s__CAG-103 sp900317855	GCA_900317855.1	652	652_15	F68H_bin.26.fa	F68H_bin.26.fa	84.91	2.35	446
F6DE_bin.75	d__Bacteria	p__Firmicutes	c__Clostridia	o__Oscillospirales	f__Oscillospiraceae	g__CAG-110	s__	N/A	545	545_0	F6DE_bin.75.fa	F6DE_bin.75.fa	84.34	1.46	447
F68K_bin.67	d__Bacteria	p__Firmicutes	c__Clostridia	o__Oscillospirales	f__Oscillospiraceae	g__CAG-110	s__	N/A	604	604_1	F68V_bin.3.fa	F68K_bin.67.fa	80.52	2.91	448
F68V_bin.3	d__Bacteria	p__Firmicutes	c__Clostridia	o__Oscillospirales	f__Oscillospiraceae	g__CAG-110	s__	N/A	604	604_1	F68V_bin.3.fa	F68V_bin.3.fa	87.45	1.48	449
F68V_bin.33	d__Bacteria	p__Firmicutes	c__Clostridia	o__Oscillospirales	f__Oscillospiraceae	g__CAG-110	s__	N/A	605	605_0	F68V_bin.33.fa	F68V_bin.33.fa	77.04	2.01	450
F68K_bin.28	d__Bacteria	p__Firmicutes	c__Clostridia	o__Oscillospirales	f__Oscillospiraceae	g__CAG-110	s__	N/A	608	608_4	F68K_bin.28.fa	F68K_bin.28.fa	97.2	0	451
F68R_bin.90	d__Bacteria	p__Firmicutes	c__Clostridia	o__Oscillospirales	f__Oscillospiraceae	g__CAG-110	s__	N/A	608	608_4	F68K_bin.28.fa	F68R_bin.90.fa	97.43	0.4	452

Table S3. Taxonomic classifiation of the 2,421 detected MAGs

	Domain	Phylum	Class	Order	Family	Genus	Species	RefGenome	primary_cluster	secondary_cluster	repr_genome	genome	CheckM_completeness	CheckM_contamination	
F6DB_bin.61	d__Bacteria	p__Firmicutes	c__Clostridia	o__Oscillospirales	f__Oscillospiraceae	g__CAG-110	s__	N/A	608	608_4	F68K_bin.28.fa	F6DB_bin.61.fa	96.15	0.34	453
F6D8_bin.36	d__Bacteria	p__Firmicutes	c__Clostridia	o__Oscillospirales	f__Oscillospiraceae	g__CAG-110	s__	N/A	608	608_1	F6D8_bin.36.fa	F6D8_bin.36.fa	86.34	1.85	454
F6DG_bin.75	d__Bacteria	p__Firmicutes	c__Clostridia	o__Oscillospirales	f__Oscillospiraceae	g__CAG-110	s__	N/A	608	608_2	F6DG_bin.75.fa	F6DG_bin.75.fa	97.87	0	455
F6DH_bin.85	d__Bacteria	p__Firmicutes	c__Clostridia	o__Oscillospirales	f__Oscillospiraceae	g__CAG-110	s__	N/A	608	608_3	F6DH_bin.85.fa	F6DH_bin.85.fa	91.75	1.23	456
F6MY_bin.61	d__Bacteria	p__Firmicutes	c__Clostridia	o__Oscillospirales	f__Oscillospiraceae	g__CAG-110	s__	N/A	608	608_5	F6MY_bin.61.fa	F6MY_bin.61.fa	96.32	0.67	457
F68W_bin.11	d__Bacteria	p__Firmicutes	c__Clostridia	o__Oscillospirales	f__Oscillospiraceae	g__CAG-110	s__	N/A	609	609_1	F6DH_bin.17.fa	F68W_bin.11.fa	83.66	2.35	458
F6DH_bin.17	d__Bacteria	p__Firmicutes	c__Clostridia	o__Oscillospirales	f__Oscillospiraceae	g__CAG-110	s__	N/A	609	609_1	F6DH_bin.17.fa	F6DH_bin.17.fa	92.23	0	459
F68F_bin.50	d__Bacteria	p__Firmicutes	c__Clostridia	o__Oscillospirales	f__Oscillospiraceae	g__CAG-110	s__	N/A	610	610_5	F68F_bin.50.fa	F68F_bin.50.fa	90.39	3.3	460
F6DB_bin.113	d__Bacteria	p__Firmicutes	c__Clostridia	o__Oscillospirales	f__Oscillospiraceae	g__CAG-110	s__	N/A	610	610_6	F6DB_bin.113.fa	F6DB_bin.113.fa	93.74	0	461
F68C_bin.93	d__Bacteria	p__Firmicutes	c__Clostridia	o__Oscillospirales	f__Oscillospiraceae	g__CAG-110	s__	N/A	611	611_5	F68C_bin.93.fa	F68C_bin.93.fa	86.23	3.13	462

Table S3. Taxonomic classifiation of the 2,421 detected MAGs

	Domain	Phylum	Class	Order	Family	Genus	Species	RefGenome	primary_cluster	secondary_cluster	repr_genome	genome	CheckM_completeness	CheckM_contamination	
F68D_bin.64	d__Bacteria	p__Firmicutes	c__Clostridia	o__Oscillospirales	f__Oscillospiraceae	g__CAG-110	s__	N/A	611	611_3	F68D_bin.64.fa	F68D_bin.64.fa	58.5	0	463
F68G_bin.20	d__Bacteria	p__Firmicutes	c__Clostridia	o__Oscillospirales	f__Oscillospiraceae	g__CAG-110	s__	N/A	611	611_4	F68G_bin.20.fa	F68G_bin.20.fa	93.09	1.31	464
F6DG_bin.70	d__Bacteria	p__Firmicutes	c__Clostridia	o__Oscillospirales	f__Oscillospiraceae	g__CAG-110	s__	N/A	611	611_1	F6DG_bin.70.fa	F6DG_bin.70.fa	93.06	3.02	465
F6MY_bin.30	d__Bacteria	p__Firmicutes	c__Clostridia	o__Oscillospirales	f__Oscillospiraceae	g__CAG-110	s__	N/A	611	611_2	F6MY_bin.30.fa	F6MY_bin.30.fa	68.57	0	466
F68R_bin.42	d__Bacteria	p__Firmicutes	c__Clostridia	o__Oscillospirales	f__Oscillospiraceae	g__CAG-110	s__	N/A	612	612_1	F68R_bin.42.fa	F68R_bin.42.fa	94.22	2.28	467
F6D8_bin.34	d__Bacteria	p__Firmicutes	c__Clostridia	o__Oscillospirales	f__Oscillospiraceae	g__CAG-110	s__	N/A	612	612_3	F6D8_bin.34.fa	F6D8_bin.34.fa	82.66	0.67	468
F6MY_bin.17	d__Bacteria	p__Firmicutes	c__Clostridia	o__Oscillospirales	f__Oscillospiraceae	g__CAG-110	s__	N/A	612	612_2	F6MY_bin.17.fa	F6MY_bin.17.fa	52.14	0	469
F68D_bin.10	d__Bacteria	p__Firmicutes	c__Clostridia	o__Oscillospirales	f__Oscillospiraceae	g__CAG-110	s__	N/A	624	624_1	F68D_bin.10.fa	F68D_bin.10.fa	86.88	2.63	470
F6D9_bin.89	d__Bacteria	p__Firmicutes	c__Clostridia	o__Oscillospirales	f__Oscillospiraceae	g__CAG-110	s__	N/A	624	624_3	F6D9_bin.89.fa	F6D9_bin.89.fa	95.75	1.34	471
F6MX_bin.89	d__Bacteria	p__Firmicutes	c__Clostridia	o__Oscillospirales	f__Oscillospiraceae	g__CAG-110	s__	N/A	624	624_2	F6MX_bin.89.fa	F6MX_bin.89.fa	80.5	2.18	472

Table S3. Taxonomic classifiation of the 2,421 detected MAGs

	Domain	Phylum	Class	Order	Family	Genus	Species	RefGenome	primary_cluster	secondary_cluster	repr_genome	genome	CheckM_completeness	CheckM_contamination	
F68M_bin.18	d__Bacteria	p__Firmicutes	c__Clostridia	o__Oscillospirales	f__Oscillospiraceae	g__CAG-110	s__	N/A	625	625_0	F68M_bin.18.fa	F68M_bin.18.fa	54.48	3.79	473
F68K_bin.32	d__Bacteria	p__Firmicutes	c__Clostridia	o__Oscillospirales	f__Oscillospiraceae	g__CAG-110	s__CAG-110 sp000434635	GCA_000434635.1	610	610_4	F68K_bin.32.fa	F68K_bin.32.fa	97.76	0	473
F6D8_bin.69	d__Bacteria	p__Firmicutes	c__Clostridia	o__Oscillospirales	f__Oscillospiraceae	g__CAG-110	s__CAG-110 sp000434635	GCA_000434635.1	610	610_1	F6D8_bin.69.fa	F6D8_bin.69.fa	53.4	1.61	473
F6MX_bin.107	d__Bacteria	p__Firmicutes	c__Clostridia	o__Oscillospirales	f__Oscillospiraceae	g__CAG-110	s__CAG-110 sp000434635	GCA_000434635.1	610	610_2	F6MX_bin.107.fa	F6MX_bin.107.fa	92.23	0	473
F6MY_bin.105	d__Bacteria	p__Firmicutes	c__Clostridia	o__Oscillospirales	f__Oscillospiraceae	g__CAG-110	s__CAG-110 sp000434635	GCA_000434635.1	610	610_3	F6MY_bin.105.fa	F6MY_bin.105.fa	74.96	0.67	473
F68K_bin.21	d__Bacteria	p__Firmicutes	c__Clostridia	o__Oscillospirales	f__Oscillospiraceae	g__CAG-110	s__CAG-110 sp000435995	GCA_000435995.1	544	544_1	F68V_bin.31.fa	F68K_bin.21.fa	92.62	1.34	473
F68V_bin.31	d__Bacteria	p__Firmicutes	c__Clostridia	o__Oscillospirales	f__Oscillospiraceae	g__CAG-110	s__CAG-110 sp000435995	GCA_000435995.1	544	544_1	F68V_bin.31.fa	F68V_bin.31.fa	94.32	1.34	473
F6DG_bin.89	d__Bacteria	p__Firmicutes	c__Clostridia	o__Oscillospirales	f__Oscillospiraceae	g__CAG-110	s__CAG-110 sp000435995	GCA_000435995.1	544	544_1	F68V_bin.31.fa	F6DG_bin.89.fa	81.37	4.81	473

Table S3. Taxonomic classifiation of the 2,421 detected MAGs

	Domain	Phylum	Class	Order	Family	Genus	Species	RefGenome	primary_cluster	secondary_cluster	repr_genome	genome	CheckM_completeness	CheckM_contamination	
F6MY_bin.4	d__Bacteria	p__Firmicutes	c__Clostridia	o__Oscillospirales	f__Oscillospiraceae	g__CAG-110	s__CAG-110 sp000435995	GCA_000435995.1	544	544_1	F68V_bin.31.fa	F6MY_bin.4.fa	89.26	1.34	473
F68C_bin.45	d__Bacteria	p__Firmicutes	c__Clostridia	o__Oscillospirales	f__Oscillospiraceae	g__CAG-110	s__CAG-110 sp003525905	GCA_003525905.1	623	623_1	F68C_bin.45.fa	F68C_bin.45.fa	89.26	0	473
F68D_bin.17	d__Bacteria	p__Firmicutes	c__Clostridia	o__Oscillospirales	f__Oscillospiraceae	g__CAG-110	s__CAG-110 sp003525905	GCA_003525905.1	623	623_12	F68D_bin.17.fa	F68D_bin.17.fa	88.75	1.81	473
F68F_bin.80	d__Bacteria	p__Firmicutes	c__Clostridia	o__Oscillospirales	f__Oscillospiraceae	g__CAG-110	s__CAG-110 sp003525905	GCA_003525905.1	623	623_16	F68F_bin.80.fa	F68F_bin.80.fa	78.34	3.09	473
F68K_bin.50	d__Bacteria	p__Firmicutes	c__Clostridia	o__Oscillospirales	f__Oscillospiraceae	g__CAG-110	s__CAG-110 sp003525905	GCA_003525905.1	623	623_11	F68K_bin.50.fa	F68K_bin.50.fa	91.28	0.67	473
F68M_bin.74	d__Bacteria	p__Firmicutes	c__Clostridia	o__Oscillospirales	f__Oscillospiraceae	g__CAG-110	s__CAG-110 sp003525905	GCA_003525905.1	623	623_2	F68M_bin.74.fa	F68M_bin.74.fa	67.6	2.68	473
F68T_bin.1	d__Bacteria	p__Firmicutes	c__Clostridia	o__Oscillospirales	f__Oscillospiraceae	g__CAG-110	s__CAG-110 sp003525905	GCA_003525905.1	623	623_3	F68T_bin.1.fa	F68T_bin.1.fa	86.07	0.89	473

Table S3. Taxonomic classifiation of the 2,421 detected MAGs

	Domain	Phylum	Class	Order	Family	Genus	Species	RefGenome	primary_cluster	secondary_cluster	repr_genome	genome	CheckM_completeness	CheckM_contamination	
F68V_bin.28	d__Bacteria	p__Firmicutes	c__Clostridia	o__Oscillospirales	f__Oscillospiraceae	g__CAG-110	s__CAG-110 sp003525905	GCA_003525905.1	623	623_9	F68V_bin.28.fa	F68V_bin.28.fa	91.44	0.67	473
F6D8_bin.48	d__Bacteria	p__Firmicutes	c__Clostridia	o__Oscillospirales	f__Oscillospiraceae	g__CAG-110	s__CAG-110 sp003525905	GCA_003525905.1	623	623_14	F6D8_bin.48.fa	F6D8_bin.48.fa	87.92	1.48	473
F6D9_bin.34	d__Bacteria	p__Firmicutes	c__Clostridia	o__Oscillospirales	f__Oscillospiraceae	g__CAG-110	s__CAG-110 sp003525905	GCA_003525905.1	623	623_5	F6D9_bin.34.fa	F6D9_bin.34.fa	84.76	0.17	473
F6DA_bin.65	d__Bacteria	p__Firmicutes	c__Clostridia	o__Oscillospirales	f__Oscillospiraceae	g__CAG-110	s__CAG-110 sp003525905	GCA_003525905.1	623	623_4	F6DA_bin.65.fa	F6DA_bin.65.fa	76.48	0.67	473
F6DB_bin.72	d__Bacteria	p__Firmicutes	c__Clostridia	o__Oscillospirales	f__Oscillospiraceae	g__CAG-110	s__CAG-110 sp003525905	GCA_003525905.1	623	623_15	F6DB_bin.72.fa	F6DB_bin.72.fa	83.67	0	473
F6DE_bin.40	d__Bacteria	p__Firmicutes	c__Clostridia	o__Oscillospirales	f__Oscillospiraceae	g__CAG-110	s__CAG-110 sp003525905	GCA_003525905.1	623	623_6	F6DE_bin.40.fa	F6DE_bin.40.fa	89.26	0	473
F6DG_bin.29	d__Bacteria	p__Firmicutes	c__Clostridia	o__Oscillospirales	f__Oscillospiraceae	g__CAG-110	s__CAG-110 sp003525905	GCA_003525905.1	623	623_7	F6DG_bin.29.fa	F6DG_bin.29.fa	92.62	0	473

Table S3. Taxonomic classifiation of the 2,421 detected MAGs

	Domain	Phylum	Class	Order	Family	Genus	Species	RefGenome	primary_cluster	secondary_cluster	repr_genome	genome	CheckM_completeness	CheckM_contamination	
F6DH_bin.70	d__Bacteria	p__Firmicutes	c__Clostridia	o__Oscillospirales	f__Oscillospiraceae	g__CAG-110	s__CAG-110 sp003525905	GCA_003525905.1	623	623_13	F6DH_bin.70.fa	F6DH_bin.70.fa	77.55	1.24	473
F6MX_bin.11	d__Bacteria	p__Firmicutes	c__Clostridia	o__Oscillospirales	f__Oscillospiraceae	g__CAG-110	s__CAG-110 sp003525905	GCA_003525905.1	623	623_8	F6MX_bin.11.fa	F6MX_bin.11.fa	76.96	0.84	473
F6MY_bin.49	d__Bacteria	p__Firmicutes	c__Clostridia	o__Oscillospirales	f__Oscillospiraceae	g__CAG-110	s__CAG-110 sp003525905	GCA_003525905.1	623	623_10	F6MY_bin.49.fa	F6MY_bin.49.fa	89.26	0	473
F68W_bin.109	d__Bacteria	p__Firmicutes	c__Clostridia	o__Oscillospirales	f__Oscillospiraceae	g__CAG-170	s__	N/A	615	615_1	F6DH_bin.35.fa	F68W_bin.109.fa	93.96	1.34	474
F6DH_bin.35	d__Bacteria	p__Firmicutes	c__Clostridia	o__Oscillospirales	f__Oscillospiraceae	g__CAG-170	s__	N/A	615	615_1	F6DH_bin.35.fa	F6DH_bin.35.fa	95.3	1.34	475
F68K_bin.59	d__Bacteria	p__Firmicutes	c__Clostridia	o__Oscillospirales	f__Oscillospiraceae	g__CAG-170	s__	N/A	618	618_3	F68K_bin.59.fa	F68K_bin.59.fa	84.78	1.34	476
F68W_bin.16	d__Bacteria	p__Firmicutes	c__Clostridia	o__Oscillospirales	f__Oscillospiraceae	g__CAG-170	s__	N/A	618	618_1	F68W_bin.16.fa	F68W_bin.16.fa	80.44	4.33	477
F6DB_bin.91	d__Bacteria	p__Firmicutes	c__Clostridia	o__Oscillospirales	f__Oscillospiraceae	g__CAG-170	s__	N/A	618	618_2	F6DB_bin.91.fa	F6DB_bin.91.fa	93.1	3.92	478
F6DG_bin.22	d__Bacteria	p__Firmicutes	c__Clostridia	o__Oscillospirales	f__Oscillospiraceae	g__CAG-170	s__	N/A	618	618_4	F6DG_bin.22.fa	F6DG_bin.22.fa	86.47	1.34	479

Table S3. Taxonomic classifiation of the 2,421 detected MAGs

	Domain	Phylum	Class	Order	Family	Genus	Species	RefGenome	primary_cluster	secondary_cluster	repr_genome	genome	CheckM_completeness	CheckM_contamination	
F6DE_bin.85	d__Bacteria	p__Firmicutes	c__Clostridia	o__Oscillospirales	f__Oscillospiraceae	g__CAG-170	s__	N/A	619	619_0	F6DE_bin.85.fa	F6DE_bin.85.fa	93.29	0.67	480
F68D_bin.25	d__Bacteria	p__Firmicutes	c__Clostridia	o__Oscillospirales	f__Oscillospiraceae	g__CAG-170	s__CAG-170 sp000432135	GCA_000432135.1	614	614_6	F68D_bin.25.fa	F68D_bin.25.fa	85.19	0.67	480
F68F_bin.13	d__Bacteria	p__Firmicutes	c__Clostridia	o__Oscillospirales	f__Oscillospiraceae	g__CAG-170	s__CAG-170 sp000432135	GCA_000432135.1	614	614_8	F68F_bin.13.fa	F68F_bin.13.fa	89.77	4.83	480
F68G_bin.23	d__Bacteria	p__Firmicutes	c__Clostridia	o__Oscillospirales	f__Oscillospiraceae	g__CAG-170	s__CAG-170 sp000432135	GCA_000432135.1	614	614_9	F68G_bin.23.fa	F68G_bin.23.fa	86.26	2.01	480
F68H_bin.80	d__Bacteria	p__Firmicutes	c__Clostridia	o__Oscillospirales	f__Oscillospiraceae	g__CAG-170	s__CAG-170 sp000432135	GCA_000432135.1	614	614_4	F68H_bin.80.fa	F68H_bin.80.fa	91.66	3.86	480
F68M_bin.39	d__Bacteria	p__Firmicutes	c__Clostridia	o__Oscillospirales	f__Oscillospiraceae	g__CAG-170	s__CAG-170 sp000432135	GCA_000432135.1	614	614_10	F68M_bin.39.fa	F68M_bin.39.fa	93.96	1.7	480
F68R_bin.88	d__Bacteria	p__Firmicutes	c__Clostridia	o__Oscillospirales	f__Oscillospiraceae	g__CAG-170	s__CAG-170 sp000432135	GCA_000432135.1	614	614_11	F68R_bin.88.fa	F68R_bin.88.fa	81.37	3.58	480
F68T_bin.72	d__Bacteria	p__Firmicutes	c__Clostridia	o__Oscillospirales	f__Oscillospiraceae	g__CAG-170	s__CAG-170 sp000432135	GCA_000432135.1	614	614_12	F68T_bin.72.fa	F68T_bin.72.fa	50.05	3.45	480

Table S3. Taxonomic classifiation of the 2,421 detected MAGs

	Domain	Phylum	Class	Order	Family	Genus	Species	RefGenome	primary_cluster	secondary_cluster	repr_genome	genome	CheckM_completeness	CheckM_contamination	
F68U_bin.97	d__Bacteria	p__Firmicutes	c__Clostridia	o__Oscillospirales	f__Oscillospiraceae	g__CAG-170	s__CAG-170 sp000432135	GCA_000432135.1	614	614_1	F68U_bin.97.fa	F68U_bin.97.fa	93.35	1.48	480
F68W_bin.55	d__Bacteria	p__Firmicutes	c__Clostridia	o__Oscillospirales	f__Oscillospiraceae	g__CAG-170	s__CAG-170 sp000432135	GCA_000432135.1	614	614_1	F68U_bin.97.fa	F68W_bin.55.fa	62.53	3.02	480
F6DH_bin.54	d__Bacteria	p__Firmicutes	c__Clostridia	o__Oscillospirales	f__Oscillospiraceae	g__CAG-170	s__CAG-170 sp000432135	GCA_000432135.1	614	614_1	F68U_bin.97.fa	F6DH_bin.54.fa	90.93	1.98	480
F68V_bin.114	d__Bacteria	p__Firmicutes	c__Clostridia	o__Oscillospirales	f__Oscillospiraceae	g__CAG-170	s__CAG-170 sp000432135	GCA_000432135.1	614	614_3	F68V_bin.114.fa	F68V_bin.114.fa	88.93	0.67	480
F6D9_bin.30	d__Bacteria	p__Firmicutes	c__Clostridia	o__Oscillospirales	f__Oscillospiraceae	g__CAG-170	s__CAG-170 sp000432135	GCA_000432135.1	614	614_7	F6D9_bin.30.fa	F6D9_bin.30.fa	93.29	1.34	480
F6DB_bin.84	d__Bacteria	p__Firmicutes	c__Clostridia	o__Oscillospirales	f__Oscillospiraceae	g__CAG-170	s__CAG-170 sp000432135	GCA_000432135.1	614	614_2	F6DE_bin.21.fa	F6DB_bin.84.fa	94.63	3.02	480
F6DE_bin.21	d__Bacteria	p__Firmicutes	c__Clostridia	o__Oscillospirales	f__Oscillospiraceae	g__CAG-170	s__CAG-170 sp000432135	GCA_000432135.1	614	614_2	F6DE_bin.21.fa	F6DE_bin.21.fa	92.17	2.07	480

Table S3. Taxonomic classifiation of the 2,421 detected MAGs

	Domain	Phylum	Class	Order	Family	Genus	Species	RefGenome	primary_cluster	secondary_cluster	repr_genome	genome	CheckM_completeness	CheckM_contamination	
F6MX_bin.124	d__Bacteria	p__Firmicutes	c__Clostridia	o__Oscillospirales	f__Oscillospiraceae	g__CAG-170	s__CAG-170 sp000432135	GCA_000432135.1	614	614_5	F6MX_bin.124.fa	F6MX_bin.124.fa	92.39	2.35	480
F68K_bin.34	d__Bacteria	p__Firmicutes	c__Clostridia	o__Oscillospirales	f__Oscillospiraceae	g__CAG-170	s__CAG-170 sp000436735	GCA_000436735.1	616	616_3	F68K_bin.34.fa	F68K_bin.34.fa	92.36	2.68	480
F6D7_bin.50	d__Bacteria	p__Firmicutes	c__Clostridia	o__Oscillospirales	f__Oscillospiraceae	g__CAG-170	s__CAG-170 sp000436735	GCA_000436735.1	616	616_5	F6D7_bin.50.fa	F6D7_bin.50.fa	89.6	1.68	480
F6D9_bin.18	d__Bacteria	p__Firmicutes	c__Clostridia	o__Oscillospirales	f__Oscillospiraceae	g__CAG-170	s__CAG-170 sp000436735	GCA_000436735.1	616	616_2	F6D9_bin.18.fa	F6D9_bin.18.fa	93.29	1.34	480
F6DA_bin.89	d__Bacteria	p__Firmicutes	c__Clostridia	o__Oscillospirales	f__Oscillospiraceae	g__CAG-170	s__CAG-170 sp000436735	GCA_000436735.1	616	616_6	F6DA_bin.89.fa	F6DA_bin.89.fa	55.94	0.67	480
F6DB_bin.67	d__Bacteria	p__Firmicutes	c__Clostridia	o__Oscillospirales	f__Oscillospiraceae	g__CAG-170	s__CAG-170 sp000436735	GCA_000436735.1	616	616_4	F6DB_bin.67.fa	F6DB_bin.67.fa	92.62	1.68	480
F68W_bin.86	d__Bacteria	p__Firmicutes	c__Clostridia	o__Oscillospirales	f__Oscillospiraceae	g__CAG-170	s__CAG-170 sp000436735	GCA_000436735.1	616	616_1	F6DH_bin.48.fa	F68W_bin.86.fa	71.85	3.32	480

Table S3. Taxonomic classifiation of the 2,421 detected MAGs

	Domain	Phylum	Class	Order	Family	Genus	Species	RefGenome	primary_cluster	secondary_cluster	repr_genome	genome	CheckM_completeness	CheckM_contamination	
F6DH_bin.48	d__Bacteria	p__Firmicutes	c__Clostridia	o__Oscillospirales	f__Oscillospiraceae	g__CAG-170	s__CAG-170 sp000436735	GCA_000436735.1	616	616_1	F6DH_bin.48.fa	F6DH_bin.48.fa	80.3	1.34	480
F68G_bin.44	d__Bacteria	p__Firmicutes	c__Clostridia	o__Oscillospirales	f__Oscillospiraceae	g__CAG-170	s__CAG-170 sp002404795	GCA_002404795.1	613	613_3	F68G_bin.44.fa	F68G_bin.44.fa	81.38	3.01	480
F6D8_bin.10	d__Bacteria	p__Firmicutes	c__Clostridia	o__Oscillospirales	f__Oscillospiraceae	g__CAG-170	s__CAG-170 sp002404795	GCA_002404795.1	613	613_1	F6D8_bin.10.fa	F6D8_bin.10.fa	85.2	2.18	480
F6DG_bin.94	d__Bacteria	p__Firmicutes	c__Clostridia	o__Oscillospirales	f__Oscillospiraceae	g__CAG-170	s__CAG-170 sp002404795	GCA_002404795.1	613	613_2	F6DG_bin.94.fa	F6DG_bin.94.fa	86.39	1.03	480
F6D7_bin.80	d__Bacteria	p__Firmicutes	c__Clostridia	o__Oscillospirales	f__Oscillospiraceae	g__CAG-170	s__CAG-170 sp003516765	GCA_003516765.1	617	617_1	F6D7_bin.80.fa	F6D7_bin.80.fa	50.33	3.45	480
F6MY_bin.26	d__Bacteria	p__Firmicutes	c__Clostridia	o__Oscillospirales	f__Oscillospiraceae	g__CAG-170	s__CAG-170 sp003516765	GCA_003516765.1	617	617_2	F6MY_bin.26.fa	F6MY_bin.26.fa	68.45	2.59	480
F68C_bin.50	d__Bacteria	p__Firmicutes	c__Clostridia	o__Oscillospirales	f__Oscillospiraceae	g__CAG-83	s__	N/A	640	640_4	F68C_bin.50.fa	F68C_bin.50.fa	67.55	4.09	481
F68J_bin.66	d__Bacteria	p__Firmicutes	c__Clostridia	o__Oscillospirales	f__Oscillospiraceae	g__CAG-83	s__	N/A	640	640_2	F68J_bin.66.fa	F68J_bin.66.fa	83.3	3.19	482

Table S3. Taxonomic classifiation of the 2,421 detected MAGs

	Domain	Phylum	Class	Order	Family	Genus	Species	RefGenome	primary_cluster	secondary_cluster	repr_genome	genome	CheckM_completeness	CheckM_contamination	
F6DA_bin.36	d__Bacteria	p__Firmicutes	c__Clostridia	o__Oscillospirales	f__Oscillospiraceae	g__CAG-83	s__	N/A	640	640_1	F6DA_bin.36.fa	F6DA_bin.36.fa	93.6	2.41	483
F6DE_bin.87	d__Bacteria	p__Firmicutes	c__Clostridia	o__Oscillospirales	f__Oscillospiraceae	g__CAG-83	s__	N/A	640	640_1	F6DA_bin.36.fa	F6DE_bin.87.fa	78.59	1.34	484
F6DG_bin.49	d__Bacteria	p__Firmicutes	c__Clostridia	o__Oscillospirales	f__Oscillospiraceae	g__CAG-83	s__	N/A	640	640_3	F6DG_bin.49.fa	F6DG_bin.49.fa	92.18	1.01	485
F68J_bin.50	d__Bacteria	p__Firmicutes	c__Clostridia	o__Oscillospirales	f__Oscillospiraceae	g__CAG-83	s__	N/A	643	643_2	F68J_bin.50.fa	F68J_bin.50.fa	51.57	2.9	486
F68C_bin.90	d__Bacteria	p__Firmicutes	c__Clostridia	o__Oscillospirales	f__Oscillospiraceae	g__CAG-83	s__	N/A	644	644_3	F68C_bin.90.fa	F68C_bin.90.fa	69.6	4.5	487
F68W_bin.45	d__Bacteria	p__Firmicutes	c__Clostridia	o__Oscillospirales	f__Oscillospiraceae	g__CAG-83	s__	N/A	644	644_1	F68W_bin.45.fa	F68W_bin.45.fa	88.26	0.67	488
F6DH_bin.91	d__Bacteria	p__Firmicutes	c__Clostridia	o__Oscillospirales	f__Oscillospiraceae	g__CAG-83	s__	N/A	644	644_1	F68W_bin.45.fa	F6DH_bin.91.fa	71.48	0.67	489
F6MX_bin.16	d__Bacteria	p__Firmicutes	c__Clostridia	o__Oscillospirales	f__Oscillospiraceae	g__CAG-83	s__	N/A	644	644_2	F6MX_bin.16.fa	F6MX_bin.16.fa	89.26	1.34	490
F68W_bin.90	d__Bacteria	p__Firmicutes	c__Clostridia	o__Oscillospirales	f__Oscillospiraceae	g__CAG-83	s__	N/A	645	645_1	F68W_bin.90.fa	F68W_bin.90.fa	92.03	1.34	491
F6DH_bin.94	d__Bacteria	p__Firmicutes	c__Clostridia	o__Oscillospirales	f__Oscillospiraceae	g__CAG-83	s__	N/A	645	645_1	F68W_bin.90.fa	F6DH_bin.94.fa	82.47	0.82	492

Table S3. Taxonomic classifiation of the 2,421 detected MAGs

	Domain	Phylum	Class	Order	Family	Genus	Species	RefGenome	primary_cluster	secondary_cluster	repr_genome	genome	CheckM_completeness	CheckM_contamination	
F6DB_bin.107	d__Bacteria	p__Firmicutes	c__Clostridia	o__Oscillospirales	f__Oscillospiraceae	g__CAG-83	s__	N/A	645	645_2	F6DB_bin.107.fa	F6DB_bin.107.fa	93.62	2.01	493
F68D_bin.79	d__Bacteria	p__Firmicutes	c__Clostridia	o__Oscillospirales	f__Oscillospiraceae	g__CAG-83	s__	N/A	646	646_4	F68D_bin.79.fa	F68D_bin.79.fa	92.62	0.67	494
F68H_bin.71	d__Bacteria	p__Firmicutes	c__Clostridia	o__Oscillospirales	f__Oscillospiraceae	g__CAG-83	s__	N/A	646	646_1	F68H_bin.71.fa	F68H_bin.71.fa	89.04	1.48	495
F68R_bin.118	d__Bacteria	p__Firmicutes	c__Clostridia	o__Oscillospirales	f__Oscillospiraceae	g__CAG-83	s__	N/A	646	646_2	F68R_bin.118.fa	F68R_bin.118.fa	92.62	0.67	496
F68V_bin.42	d__Bacteria	p__Firmicutes	c__Clostridia	o__Oscillospirales	f__Oscillospiraceae	g__CAG-83	s__	N/A	646	646_3	F68V_bin.42.fa	F68V_bin.42.fa	93.29	0.67	497
F68J_bin.38	d__Bacteria	p__Firmicutes	c__Clostridia	o__Oscillospirales	f__Oscillospiraceae	g__CAG-83	s__CAG-83 sp000431575	GCA_000431575.1	642	642_1	F68J_bin.38.fa	F68J_bin.38.fa	85.22	1.46	497
F68K_bin.46	d__Bacteria	p__Firmicutes	c__Clostridia	o__Oscillospirales	f__Oscillospiraceae	g__CAG-83	s__CAG-83 sp000431575	GCA_000431575.1	642	642_6	F68K_bin.46.fa	F68K_bin.46.fa	93.06	0.67	497
F68P_bin.4	d__Bacteria	p__Firmicutes	c__Clostridia	o__Oscillospirales	f__Oscillospiraceae	g__CAG-83	s__CAG-83 sp000431575	GCA_000431575.1	642	642_3	F68P_bin.4.fa	F68P_bin.4.fa	87.92	3.02	497
F6D7_bin.8	d__Bacteria	p__Firmicutes	c__Clostridia	o__Oscillospirales	f__Oscillospiraceae	g__CAG-83	s__CAG-83 sp000431575	GCA_000431575.1	642	642_7	F6D7_bin.8.fa	F6D7_bin.8.fa	64.83	1.34	497

Table S3. Taxonomic classifiation of the 2,421 detected MAGs

	Domain	Phylum	Class	Order	Family	Genus	Species	RefGenome	primary_cluster	secondary_cluster	repr_genome	genome	CheckM_completeness	CheckM_contamination	
F6DA_bin.69	d__Bacteria	p__Firmicutes	c__Clostridia	o__Oscillospirales	f__Oscillospiraceae	g__CAG-83	s__CAG-83 sp000431575	GCA_000431575.1	642	642_4	F6DA_bin.69.fa	F6DA_bin.69.fa	92.62	0.67	497
F6DE_bin.93	d__Bacteria	p__Firmicutes	c__Clostridia	o__Oscillospirales	f__Oscillospiraceae	g__CAG-83	s__CAG-83 sp000431575	GCA_000431575.1	642	642_2	F6DE_bin.93.fa	F6DE_bin.93.fa	72.28	3.57	497
F6DB_bin.71	d__Bacteria	p__Firmicutes	c__Clostridia	o__Oscillospirales	f__Oscillospiraceae	g__CAG-83	s__CAG-83 sp000431575	GCA_000431575.1	642	642_5	F6DG_bin.40.fa	F6DB_bin.71.fa	76.45	2.35	497
F6DG_bin.40	d__Bacteria	p__Firmicutes	c__Clostridia	o__Oscillospirales	f__Oscillospiraceae	g__CAG-83	s__CAG-83 sp000431575	GCA_000431575.1	642	642_5	F6DG_bin.40.fa	F6DG_bin.40.fa	92.62	0.67	497
F68F_bin.53	d__Bacteria	p__Firmicutes	c__Clostridia	o__Oscillospirales	f__Oscillospiraceae	g__CAG-83	s__CAG-83 sp000435555	GCA_000435555.1	647	647_5	F68F_bin.53.fa	F68F_bin.53.fa	86.58	0.67	497
F68T_bin.98	d__Bacteria	p__Firmicutes	c__Clostridia	o__Oscillospirales	f__Oscillospiraceae	g__CAG-83	s__CAG-83 sp000435555	GCA_000435555.1	647	647_1	F68T_bin.98.fa	F68T_bin.98.fa	88.08	0.84	497
F68U_bin.16	d__Bacteria	p__Firmicutes	c__Clostridia	o__Oscillospirales	f__Oscillospiraceae	g__CAG-83	s__CAG-83 sp000435555	GCA_000435555.1	647	647_2	F68U_bin.16.fa	F68U_bin.16.fa	87.58	0.67	497

Table S3. Taxonomic classifiation of the 2,421 detected MAGs

	Domain	Phylum	Class	Order	Family	Genus	Species	RefGenome	primary_cluster	secondary_cluster	repr_genome	genome	CheckM_completeness	CheckM_contamination	
F68V_bin.77	d__Bacteria	p__Firmicutes	c__Clostridia	o__Oscillospirales	f__Oscillospiraceae	g__CAG-83	s__CAG-83 sp000435555	GCA_000435555.1	647	647_7	F68V_bin.77.fa	F68V_bin.77.fa	89.26	1.4	497
F6D7_bin.29	d__Bacteria	p__Firmicutes	c__Clostridia	o__Oscillospirales	f__Oscillospiraceae	g__CAG-83	s__CAG-83 sp000435555	GCA_000435555.1	647	647_3	F6D7_bin.29.fa	F6D7_bin.29.fa	89.26	1.01	497
F6MX_bin.73	d__Bacteria	p__Firmicutes	c__Clostridia	o__Oscillospirales	f__Oscillospiraceae	g__CAG-83	s__CAG-83 sp000435555	GCA_000435555.1	647	647_6	F6MX_bin.73.fa	F6MX_bin.73.fa	87.25	1.01	497
F6MY_bin.104	d__Bacteria	p__Firmicutes	c__Clostridia	o__Oscillospirales	f__Oscillospiraceae	g__CAG-83	s__CAG-83 sp000435555	GCA_000435555.1	647	647_4	F6MY_bin.104.fa	F6MY_bin.104.fa	86.8	1.57	497
F6D9_bin.21	d__Bacteria	p__Firmicutes	c__Clostridia	o__Oscillospirales	f__Oscillospiraceae	g__CAG-83	s__CAG-83 sp000435975	GCA_000435975.1	643	643_16	F6D9_bin.21.fa	F6D9_bin.21.fa	70.17	3.45	497
F68D_bin.22	d__Bacteria	p__Firmicutes	c__Clostridia	o__Oscillospirales	f__Oscillospiraceae	g__CAG-83	s__CAG-83 sp001916855	GCA_001916855.1	643	643_12	F68D_bin.22.fa	F68D_bin.22.fa	89.26	0.67	497
F68F_bin.31	d__Bacteria	p__Firmicutes	c__Clostridia	o__Oscillospirales	f__Oscillospiraceae	g__CAG-83	s__CAG-83 sp001916855	GCA_001916855.1	643	643_6	F68F_bin.31.fa	F68F_bin.31.fa	71.48	0	497

Table S3. Taxonomic classifiation of the 2,421 detected MAGs

	Domain	Phylum	Class	Order	Family	Genus	Species	RefGenome	primary_cluster	secondary_cluster	repr_genome	genome	CheckM_completeness	CheckM_contamination	
F68G_bin.72	d__Bacteria	p__Firmicutes	c__Clostridia	o__Oscillospirales	f__Oscillospiraceae	g__CAG-83	s__CAG-83 sp001916855	GCA_001916855.1	643	643_9	F68G_bin.72.fa	F68G_bin.72.fa	91.28	0.67	497
F68H_bin.30	d__Bacteria	p__Firmicutes	c__Clostridia	o__Oscillospirales	f__Oscillospiraceae	g__CAG-83	s__CAG-83 sp001916855	GCA_001916855.1	643	643_10	F68H_bin.30.fa	F68H_bin.30.fa	90.54	0.69	497
F68M_bin.35	d__Bacteria	p__Firmicutes	c__Clostridia	o__Oscillospirales	f__Oscillospiraceae	g__CAG-83	s__CAG-83 sp001916855	GCA_001916855.1	643	643_4	F68M_bin.35.fa	F68M_bin.35.fa	95.13	0.67	497
F6MX_bin.76	d__Bacteria	p__Firmicutes	c__Clostridia	o__Oscillospirales	f__Oscillospiraceae	g__CAG-83	s__CAG-83 sp001916855	GCA_001916855.1	643	643_4	F68M_bin.35.fa	F6MX_bin.76.fa	72.01	0.67	497
F68N_bin.77	d__Bacteria	p__Firmicutes	c__Clostridia	o__Oscillospirales	f__Oscillospiraceae	g__CAG-83	s__CAG-83 sp001916855	GCA_001916855.1	643	643_7	F68N_bin.77.fa	F68N_bin.77.fa	88.93	0	497
F68T_bin.82	d__Bacteria	p__Firmicutes	c__Clostridia	o__Oscillospirales	f__Oscillospiraceae	g__CAG-83	s__CAG-83 sp001916855	GCA_001916855.1	643	643_8	F68T_bin.82.fa	F68T_bin.82.fa	90.6	0	497
F68V_bin.36	d__Bacteria	p__Firmicutes	c__Clostridia	o__Oscillospirales	f__Oscillospiraceae	g__CAG-83	s__CAG-83 sp001916855	GCA_001916855.1	643	643_15	F68V_bin.36.fa	F68V_bin.36.fa	89.93	1.34	497

Table S3. Taxonomic classifiation of the 2,421 detected MAGs

	Domain	Phylum	Class	Order	Family	Genus	Species	RefGenome	primary_cluster	secondary_cluster	repr_genome	genome	CheckM_completeness	CheckM_contamination	
F6D7_bin.13	d__Bacteria	p__Firmicutes	c__Clostridia	o__Oscillospirales	f__Oscillospiraceae	g__CAG-83	s__CAG-83 sp001916855	GCA_001916855.1	643	643_14	F6D7_bin.13.fa	F6D7_bin.13.fa	95.3	3.69	497
F6D8_bin.4	d__Bacteria	p__Firmicutes	c__Clostridia	o__Oscillospirales	f__Oscillospiraceae	g__CAG-83	s__CAG-83 sp001916855	GCA_001916855.1	643	643_13	F6D8_bin.4.fa	F6D8_bin.4.fa	89.26	0.17	497
F6D9_bin.32	d__Bacteria	p__Firmicutes	c__Clostridia	o__Oscillospirales	f__Oscillospiraceae	g__CAG-83	s__CAG-83 sp001916855	GCA_001916855.1	643	643_3	F6D9_bin.32.fa	F6D9_bin.32.fa	76.12	4.03	497
F6DE_bin.22	d__Bacteria	p__Firmicutes	c__Clostridia	o__Oscillospirales	f__Oscillospiraceae	g__CAG-83	s__CAG-83 sp001916855	GCA_001916855.1	643	643_5	F6DE_bin.22.fa	F6DE_bin.22.fa	85.23	0.21	497
F68W_bin.50	d__Bacteria	p__Firmicutes	c__Clostridia	o__Oscillospirales	f__Oscillospiraceae	g__CAG-83	s__CAG-83 sp001916855	GCA_001916855.1	643	643_1	F6DH_bin.77.fa	F68W_bin.50.fa	69.49	2.59	497
F6DH_bin.77	d__Bacteria	p__Firmicutes	c__Clostridia	o__Oscillospirales	f__Oscillospiraceae	g__CAG-83	s__CAG-83 sp001916855	GCA_001916855.1	643	643_1	F6DH_bin.77.fa	F6DH_bin.77.fa	85.94	2.03	497
F6MY_bin.119	d__Bacteria	p__Firmicutes	c__Clostridia	o__Oscillospirales	f__Oscillospiraceae	g__CAG-83	s__CAG-83 sp001916855	GCA_001916855.1	643	643_11	F6MY_bin.119.fa	F6MY_bin.119.fa	91.28	0.67	497

Table S3. Taxonomic classifiation of the 2,421 detected MAGs

	Domain	Phylum	Class	Order	Family	Genus	Species	RefGenome	primary_cluster	secondary_cluster	repr_genome	genome	CheckM_completeness	CheckM_contamination	
F68C_bin.60	d__Bacteria	p__Firmicutes	c__Clostridia	o__Oscillospirales	f__Oscillospiraceae	g__CAG-83	s__CAG-83 sp003487665	GCA_003487665.1	648	648_1	F68C_bin.60.fa	F68C_bin.60.fa	85.58	0.81	497
F68U_bin.7	d__Bacteria	p__Firmicutes	c__Clostridia	o__Oscillospirales	f__Oscillospiraceae	g__CAG-83	s__CAG-83 sp003487665	GCA_003487665.1	648	648_2	F68U_bin.7.fa	F68U_bin.7.fa	90.62	1.34	497
F6DA_bin.68	d__Bacteria	p__Firmicutes	c__Clostridia	o__Oscillospirales	f__Oscillospiraceae	g__CAG-83	s__CAG-83 sp003487665	GCA_003487665.1	648	648_3	F6DA_bin.68.fa	F6DA_bin.68.fa	83.97	2.85	497
F68D_bin.32	d__Bacteria	p__Firmicutes	c__Clostridia	o__Oscillospirales	f__Oscillospiraceae	g__CAG-83	s__CAG-83 sp003539495	GCA_003539495.1	641	641_3	F68D_bin.32.fa	F68D_bin.32.fa	56.61	0.86	497
F68G_bin.66	d__Bacteria	p__Firmicutes	c__Clostridia	o__Oscillospirales	f__Oscillospiraceae	g__CAG-83	s__CAG-83 sp003539495	GCA_003539495.1	641	641_1	F68G_bin.66.fa	F68G_bin.66.fa	91.95	0.67	497
F68J_bin.64	d__Bacteria	p__Firmicutes	c__Clostridia	o__Oscillospirales	f__Oscillospiraceae	g__CAG-83	s__CAG-83 sp003539495	GCA_003539495.1	641	641_2	F68J_bin.64.fa	F68J_bin.64.fa	89.44	0.71	497
F6DG_bin.48	d__Bacteria	p__Firmicutes	c__Clostridia	o__Oscillospirales	f__Oscillospiraceae	g__ER4	s__	N/A	622	622_11	F6DG_bin.48.fa	F6DG_bin.48.fa	96.64	0	498
F68H_bin.55	d__Bacteria	p__Firmicutes	c__Clostridia	o__Oscillospirales	f__Oscillospiraceae	g__ER4	s__ER4 sp000765235	GCF_000765235.1	622	622_5	F68H_bin.55.fa	F68H_bin.55.fa	97.99	3.36	498

Table S3. Taxonomic classifiation of the 2,421 detected MAGs

	Domain	Phylum	Class	Order	Family	Genus	Species	RefGenome	primary_cluster	secondary_cluster	repr_genome	genome	CheckM_completeness	CheckM_contamination	
F68R_bin.121	d__Bacteria	p__Firmicutes	c__Clostridia	o__Oscillospirales	f__Oscillospiraceae	g__ER4	s__ER4 sp000765235	GCF_000765235.1	622	622_2	F68R_bin.121.fa	F68R_bin.121.fa	93.06	3.36	498
F68C_bin.100	d__Bacteria	p__Firmicutes	c__Clostridia	o__Oscillospirales	f__Oscillospiraceae	g__ER4	s__ER4 sp000765235	GCF_000765235.1	622	622_3	F68U_bin.78.fa	F68C_bin.100.fa	89.24	2.93	498
F68U_bin.78	d__Bacteria	p__Firmicutes	c__Clostridia	o__Oscillospirales	f__Oscillospiraceae	g__ER4	s__ER4 sp000765235	GCF_000765235.1	622	622_3	F68U_bin.78.fa	F68U_bin.78.fa	93.96	0	498
F68V_bin.98	d__Bacteria	p__Firmicutes	c__Clostridia	o__Oscillospirales	f__Oscillospiraceae	g__ER4	s__ER4 sp000765235	GCF_000765235.1	622	622_4	F68V_bin.98.fa	F68V_bin.98.fa	68.34	2.01	498
F6MY_bin.72	d__Bacteria	p__Firmicutes	c__Clostridia	o__Oscillospirales	f__Oscillospiraceae	g__ER4	s__ER4 sp000765235	GCF_000765235.1	622	622_4	F68V_bin.98.fa	F6MY_bin.72.fa	61.09	2.46	498
F68W_bin.75	d__Bacteria	p__Firmicutes	c__Clostridia	o__Oscillospirales	f__Oscillospiraceae	g__ER4	s__ER4 sp000765235	GCF_000765235.1	622	622_6	F68W_bin.75.fa	F68W_bin.75.fa	52.79	0.81	498
F6D7_bin.84	d__Bacteria	p__Firmicutes	c__Clostridia	o__Oscillospirales	f__Oscillospiraceae	g__ER4	s__ER4 sp000765235	GCF_000765235.1	622	622_7	F6D7_bin.84.fa	F6D7_bin.84.fa	98.66	0	498
F6D8_bin.47	d__Bacteria	p__Firmicutes	c__Clostridia	o__Oscillospirales	f__Oscillospiraceae	g__ER4	s__ER4 sp000765235	GCF_000765235.1	622	622_1	F6D8_bin.47.fa	F6D8_bin.47.fa	97.82	0	498
F6DA_bin.83	d__Bacteria	p__Firmicutes	c__Clostridia	o__Oscillospirales	f__Oscillospiraceae	g__ER4	s__ER4 sp000765235	GCF_000765235.1	622	622_1	F6D8_bin.47.fa	F6DA_bin.83.fa	85.44	2.88	498
F6D9_bin.33	d__Bacteria	p__Firmicutes	c__Clostridia	o__Oscillospirales	f__Oscillospiraceae	g__ER4	s__ER4 sp000765235	GCF_000765235.1	622	622_8	F6D9_bin.33.fa	F6D9_bin.33.fa	95.75	0	498

Table S3. Taxonomic classifiation of the 2,421 detected MAGs

	Domain	Phylum	Class	Order	Family	Genus	Species	RefGenome	primary_cluster	secondary_cluster	repr_genome	genome	CheckM_completeness	CheckM_contamination	
F6DB_bin.54	d__Bacteria	p__Firmicutes	c__Clostridia	o__Oscillospirales	f__Oscillospiraceae	g__ER4	s__ER4 sp000765235	GCF_000765235.1	622	622_9	F6DB_bin.54.fa	F6DB_bin.54.fa	98.66	0	498
F6DE_bin.79	d__Bacteria	p__Firmicutes	c__Clostridia	o__Oscillospirales	f__Oscillospiraceae	g__ER4	s__ER4 sp000765235	GCF_000765235.1	622	622_10	F6DE_bin.79.fa	F6DE_bin.79.fa	88.87	4.72	498
F68D_bin.70	d__Bacteria	p__Firmicutes	c__Clostridia	o__Oscillospirales	f__Oscillospiraceae	g__ER4	s__ER4 sp003522105	GCA_003522105.1	621	621_1	F68D_bin.70.fa	F68D_bin.70.fa	96.64	0	498
F6MX_bin.136	d__Bacteria	p__Firmicutes	c__Clostridia	o__Oscillospirales	f__Oscillospiraceae	g__ER4	s__ER4 sp003522105	GCA_003522105.1	621	621_2	F6MX_bin.136.fa	F6MX_bin.136.fa	89.09	0.67	498
F68C_bin.28	d__Bacteria	p__Firmicutes	c__Clostridia	o__Oscillospirales	f__Oscillospiraceae	g__ER4	s__ER4 sp900317525	GCA_900317525.1	620	620_1	F68C_bin.28.fa	F68C_bin.28.fa	79.83	3.6	498
F68F_bin.26	d__Bacteria	p__Firmicutes	c__Clostridia	o__Oscillospirales	f__Oscillospiraceae	g__ER4	s__ER4 sp900317525	GCA_900317525.1	620	620_7	F68F_bin.26.fa	F68F_bin.26.fa	76.14	3.02	498
F68H_bin.75	d__Bacteria	p__Firmicutes	c__Clostridia	o__Oscillospirales	f__Oscillospiraceae	g__ER4	s__ER4 sp900317525	GCA_900317525.1	620	620_4	F68H_bin.75.fa	F68H_bin.75.fa	86.97	0.34	498
F68R_bin.28	d__Bacteria	p__Firmicutes	c__Clostridia	o__Oscillospirales	f__Oscillospiraceae	g__ER4	s__ER4 sp900317525	GCA_900317525.1	620	620_3	F68R_bin.28.fa	F68R_bin.28.fa	94.13	0.02	498
F68U_bin.53	d__Bacteria	p__Firmicutes	c__Clostridia	o__Oscillospirales	f__Oscillospiraceae	g__ER4	s__ER4 sp900317525	GCA_900317525.1	620	620_3	F68R_bin.28.fa	F68U_bin.53.fa	81.24	0.22	498
F68T_bin.13	d__Bacteria	p__Firmicutes	c__Clostridia	o__Oscillospirales	f__Oscillospiraceae	g__ER4	s__ER4 sp900317525	GCA_900317525.1	620	620_6	F68T_bin.13.fa	F68T_bin.13.fa	84.56	1.01	498

Table S3. Taxonomic classifiation of the 2,421 detected MAGs

	Domain	Phylum	Class	Order	Family	Genus	Species	RefGenome	primary_cluster	secondary_cluster	repr_genome	genome	CheckM_completeness	CheckM_contamination	
F6D8_bin.30	d__Bacteria	p__Firmicutes	c__Clostridia	o__Oscillospirales	f__Oscillospiraceae	g__ER4	s__ER4 sp900317525	GCA_900317525.1	620	620_5	F6D8_bin.30.fa	F6D8_bin.30.fa	90.71	0	498
F6MY_bin.128	d__Bacteria	p__Firmicutes	c__Clostridia	o__Oscillospirales	f__Oscillospiraceae	g__ER4	s__ER4 sp900317525	GCA_900317525.1	620	620_2	F6MY_bin.128.fa	F6MY_bin.128.fa	67.41	1.57	498
F68D_bin.102	d__Bacteria	p__Firmicutes	c__Clostridia	o__Oscillospirales	f__Oscillospiraceae	g__F23-B02	s__F23-B02 sp001916715	GCA_001916715.1	460	460_0	F68D_bin.102.fa	F68D_bin.102.fa	74.29	2.52	498
F68J_bin.30	d__Bacteria	p__Firmicutes	c__Clostridia	o__Oscillospirales	f__Oscillospiraceae	g__Flavonifractor	s__Flavonifractor plautii	GCF_000239295.1	561	561_1	F68J_bin.30.fa	F68J_bin.30.fa	79.73	0.88	498
F6DE_bin.80	d__Bacteria	p__Firmicutes	c__Clostridia	o__Oscillospirales	f__Oscillospiraceae	g__Flavonifractor	s__Flavonifractor plautii	GCF_000239295.1	561	561_1	F68J_bin.30.fa	F6DE_bin.80.fa	81.66	1.48	498
F68J_bin.58	d__Bacteria	p__Firmicutes	c__Clostridia	o__Oscillospirales	f__Oscillospiraceae	g__Lawsonibacter	s__	N/A	626	626_0	F68J_bin.58.fa	F68J_bin.58.fa	53.75	0	499
F68C_bin.59	d__Bacteria	p__Firmicutes	c__Clostridia	o__Oscillospirales	f__Oscillospiraceae	g__Lawsonibacter	s__	N/A	630	630_4	F68C_bin.59.fa	F68C_bin.59.fa	59.31	0	500
F68J_bin.76	d__Bacteria	p__Firmicutes	c__Clostridia	o__Oscillospirales	f__Oscillospiraceae	g__Lawsonibacter	s__	N/A	630	630_1	F68J_bin.76.fa	F68J_bin.76.fa	94.63	0.17	501
F6D7_bin.45	d__Bacteria	p__Firmicutes	c__Clostridia	o__Oscillospirales	f__Oscillospiraceae	g__Lawsonibacter	s__	N/A	630	630_2	F6D7_bin.45.fa	F6D7_bin.45.fa	87.38	2.15	502

Table S3. Taxonomic classifiation of the 2,421 detected MAGs

	Domain	Phylum	Class	Order	Family	Genus	Species	RefGenome	primary_cluster	secondary_cluster	repr_genome	genome	CheckM_completeness	CheckM_contamination	
F6DG_bin.33	d__Bacteria	p__Firmicutes	c__Clostridia	o__Oscillospirales	f__Oscillospiraceae	g__Lawsonibacter	s__	N/A	630	630_3	F6DG_bin.33.fa	F6DG_bin.33.fa	50.94	4.31	503
F6DH_bin.74	d__Bacteria	p__Firmicutes	c__Clostridia	o__Oscillospirales	f__Oscillospiraceae	g__Lawsonibacter	s__	N/A	630	630_5	F6DH_bin.74.fa	F6DH_bin.74.fa	52.59	3.45	504
F68T_bin.57	d__Bacteria	p__Firmicutes	c__Clostridia	o__Oscillospirales	f__Oscillospiraceae	g__Lawsonibacter	s__	N/A	631	631_0	F68T_bin.57.fa	F68T_bin.57.fa	87.87	2.74	505
F6MX_bin.34	d__Bacteria	p__Firmicutes	c__Clostridia	o__Oscillospirales	f__Oscillospiraceae	g__Lawsonibacter	s__Lawsonibacter asaccharolyticus	GCF_003112755.1	632	632_0	F6MX_bin.34.fa	F6MX_bin.34.fa	72.87	0.22	505
F68F_bin.5	d__Bacteria	p__Firmicutes	c__Clostridia	o__Oscillospirales	f__Oscillospiraceae	g__Lawsonibacter	s__Lawsonibacter sp000177015	GCF_000177015.3	628	628_1	F68F_bin.5.fa	F68F_bin.5.fa	99.33	0.83	505
F68K_bin.45	d__Bacteria	p__Firmicutes	c__Clostridia	o__Oscillospirales	f__Oscillospiraceae	g__Lawsonibacter	s__Lawsonibacter sp000177015	GCF_000177015.3	628	628_2	F68K_bin.45.fa	F68K_bin.45.fa	84.85	2.91	505
F6DB_bin.75	d__Bacteria	p__Firmicutes	c__Clostridia	o__Oscillospirales	f__Oscillospiraceae	g__Lawsonibacter	s__Lawsonibacter sp900066645	GCA_900066645.1	627	627_0	F6DB_bin.75.fa	F6DB_bin.75.fa	77.01	1.01	505

Table S3. Taxonomic classifiation of the 2,421 detected MAGs

	Domain	Phylum	Class	Order	Family	Genus	Species	RefGenome	primary_cluster	secondary_cluster	repr_genome	genome	CheckM_completeness	CheckM_contamination	
F68K_bin.35	d__Bacteria	p__Firmicutes	c__Clostridia	o__Oscillospirales	f__Oscillospiraceae	g__Lawsonibacter	s__Lawsonibacter sp900066825	GCA_900066825.1	629	629_1	F68K_bin.35.fa	F68K_bin.35.fa	98.11	0	505
F6DG_bin.95	d__Bacteria	p__Firmicutes	c__Clostridia	o__Oscillospirales	f__Oscillospiraceae	g__Lawsonibacter	s__Lawsonibacter sp900066825	GCA_900066825.1	629	629_2	F6DG_bin.95.fa	F6DG_bin.95.fa	62.52	4.03	505
F6MX_bin.10	d__Bacteria	p__Firmicutes	c__Clostridia	o__Oscillospirales	f__Oscillospiraceae	g__Oscillibacter	s__	N/A	635	635_0	F6MX_bin.10.fa	F6MX_bin.10.fa	79.57	2.01	506
F68D_bin.35	d__Bacteria	p__Firmicutes	c__Clostridia	o__Oscillospirales	f__Oscillospiraceae	g__Oscillibacter	s__	N/A	636	636_6	F68D_bin.35.fa	F68D_bin.35.fa	52.61	2.59	507
F6MY_bin.2	d__Bacteria	p__Firmicutes	c__Clostridia	o__Oscillospirales	f__Oscillospiraceae	g__Oscillibacter	s__	N/A	636	636_11	F6MY_bin.2.fa	F6MY_bin.2.fa	53.65	3.78	508
F6DG_bin.25	d__Bacteria	p__Firmicutes	c__Clostridia	o__Oscillospirales	f__Oscillospiraceae	g__Oscillibacter	s__	N/A	637	637_0	F6DG_bin.25.fa	F6DG_bin.25.fa	50.61	3.45	509
F68J_bin.23	d__Bacteria	p__Firmicutes	c__Clostridia	o__Oscillospirales	f__Oscillospiraceae	g__Oscillibacter	s__	N/A	638	638_4	F68J_bin.23.fa	F68J_bin.23.fa	97.32	0	510
F68R_bin.91	d__Bacteria	p__Firmicutes	c__Clostridia	o__Oscillospirales	f__Oscillospiraceae	g__Oscillibacter	s__	N/A	638	638_2	F68R_bin.91.fa	F68R_bin.91.fa	59.49	0.94	511
F68U_bin.90	d__Bacteria	p__Firmicutes	c__Clostridia	o__Oscillospirales	f__Oscillospiraceae	g__Oscillibacter	s__	N/A	638	638_3	F68U_bin.90.fa	F68U_bin.90.fa	76.49	1.01	512

Table S3. Taxonomic classifiation of the 2,421 detected MAGs

	Domain	Phylum	Class	Order	Family	Genus	Species	RefGenome	primary_cluster	secondary_cluster	repr_genome	genome	CheckM_completeness	CheckM_contamination	
F68W_bin.38	d__Bacteria	p__Firmicutes	c__Clostridia	o__Oscillospirales	f__Oscillospiraceae	g__Oscillibacter	s__	N/A	638	638_1	F68W_bin.38.fa	F68W_bin.38.fa	94.03	0.3	513
F6DH_bin.99	d__Bacteria	p__Firmicutes	c__Clostridia	o__Oscillospirales	f__Oscillospiraceae	g__Oscillibacter	s__	N/A	638	638_1	F68W_bin.38.fa	F6DH_bin.99.fa	89.37	2.43	514
F68C_bin.15	d__Bacteria	p__Firmicutes	c__Clostridia	o__Oscillospirales	f__Oscillospiraceae	g__Oscillibacter	s__Oscillibacter sp001916835	GCA_001916835.1	636	636_14	F68C_bin.15.fa	F68C_bin.15.fa	72.28	3.58	514
F68J_bin.60	d__Bacteria	p__Firmicutes	c__Clostridia	o__Oscillospirales	f__Oscillospiraceae	g__Oscillibacter	s__Oscillibacter sp001916835	GCA_001916835.1	636	636_4	F68J_bin.60.fa	F68J_bin.60.fa	58.93	1.72	514
F68K_bin.78	d__Bacteria	p__Firmicutes	c__Clostridia	o__Oscillospirales	f__Oscillospiraceae	g__Oscillibacter	s__Oscillibacter sp001916835	GCA_001916835.1	636	636_1	F68K_bin.78.fa	F68K_bin.78.fa	93.86	2.35	514
F68P_bin.54	d__Bacteria	p__Firmicutes	c__Clostridia	o__Oscillospirales	f__Oscillospiraceae	g__Oscillibacter	s__Oscillibacter sp001916835	GCA_001916835.1	636	636_1	F68K_bin.78.fa	F68P_bin.54.fa	69.58	2.32	514
F68N_bin.6	d__Bacteria	p__Firmicutes	c__Clostridia	o__Oscillospirales	f__Oscillospiraceae	g__Oscillibacter	s__Oscillibacter sp001916835	GCA_001916835.1	636	636_5	F68N_bin.6.fa	F68N_bin.6.fa	90.7	2.57	514

Table S3. Taxonomic classifiation of the 2,421 detected MAGs

	Domain	Phylum	Class	Order	Family	Genus	Species	RefGenome	primary_cluster	secondary_cluster	repr_genome	genome	CheckM_completeness	CheckM_contamination	
F68R_bin.44	d__Bacteria	p__Firmicutes	c__Clostridia	o__Oscillospirales	f__Oscillospiraceae	g__Oscillibacter	s__Oscillibacter sp001916835	GCA_001916835.1	636	636_13	F68R_bin.44.fa	F68R_bin.44.fa	68.93	2.35	514
F68T_bin.75	d__Bacteria	p__Firmicutes	c__Clostridia	o__Oscillospirales	f__Oscillospiraceae	g__Oscillibacter	s__Oscillibacter sp001916835	GCA_001916835.1	636	636_2	F68T_bin.75.fa	F68T_bin.75.fa	75.83	0.67	514
F68U_bin.86	d__Bacteria	p__Firmicutes	c__Clostridia	o__Oscillospirales	f__Oscillospiraceae	g__Oscillibacter	s__Oscillibacter sp001916835	GCA_001916835.1	636	636_8	F68U_bin.86.fa	F68U_bin.86.fa	68.38	2.3	514
F68W_bin.48	d__Bacteria	p__Firmicutes	c__Clostridia	o__Oscillospirales	f__Oscillospiraceae	g__Oscillibacter	s__Oscillibacter sp001916835	GCA_001916835.1	636	636_12	F68W_bin.48.fa	F68W_bin.48.fa	59.48	0.86	514
F6DH_bin.62	d__Bacteria	p__Firmicutes	c__Clostridia	o__Oscillospirales	f__Oscillospiraceae	g__Oscillibacter	s__Oscillibacter sp001916835	GCA_001916835.1	636	636_12	F68W_bin.48.fa	F6DH_bin.62.fa	52.83	1.34	514
F6D8_bin.72	d__Bacteria	p__Firmicutes	c__Clostridia	o__Oscillospirales	f__Oscillospiraceae	g__Oscillibacter	s__Oscillibacter sp001916835	GCA_001916835.1	636	636_15	F6D8_bin.72.fa	F6D8_bin.72.fa	83.82	2.68	514

Table S3. Taxonomic classifiation of the 2,421 detected MAGs

	Domain	Phylum	Class	Order	Family	Genus	Species	RefGenome	primary_cluster	secondary_cluster	repr_genome	genome	CheckM_completeness	CheckM_contamination	
F6D9_bin.9	d__Bacteria	p__Firmicutes	c__Clostridia	o__Oscillospirales	f__Oscillospiraceae	g__Oscillibacter	s__Oscillibacter sp001916835	GCA_001916835.1	636	636_16	F6D9_bin.9.fa	F6D9_bin.9.fa	88.36	0.67	514
F6DA_bin.84	d__Bacteria	p__Firmicutes	c__Clostridia	o__Oscillospirales	f__Oscillospiraceae	g__Oscillibacter	s__Oscillibacter sp001916835	GCA_001916835.1	636	636_9	F6DA_bin.84.fa	F6DA_bin.84.fa	84.91	3.33	514
F6DB_bin.58	d__Bacteria	p__Firmicutes	c__Clostridia	o__Oscillospirales	f__Oscillospiraceae	g__Oscillibacter	s__Oscillibacter sp001916835	GCA_001916835.1	636	636_7	F6DB_bin.58.fa	F6DB_bin.58.fa	90.56	2.91	514
F6DG_bin.104	d__Bacteria	p__Firmicutes	c__Clostridia	o__Oscillospirales	f__Oscillospiraceae	g__Oscillibacter	s__Oscillibacter sp001916835	GCA_001916835.1	636	636_3	F6DG_bin.104.fa	F6DG_bin.104.fa	97.09	2.01	514
F6MX_bin.129	d__Bacteria	p__Firmicutes	c__Clostridia	o__Oscillospirales	f__Oscillospiraceae	g__Oscillibacter	s__Oscillibacter sp001916835	GCA_001916835.1	636	636_10	F6MX_bin.129.fa	F6MX_bin.129.fa	93.88	0.34	514
F68T_bin.68	d__Bacteria	p__Firmicutes	c__Clostridia	o__Oscillospirales	f__Oscillospiraceae	g__Oscillibacter	s__Oscillibacter sp900066435	GCA_900066435.1	634	634_1	F6DB_bin.4.fa	F68T_bin.68.fa	78.45	2.91	514

Table S3. Taxonomic classifiation of the 2,421 detected MAGs

	Domain	Phylum	Class	Order	Family	Genus	Species	RefGenome	primary_cluster	secondary_cluster	repr_genome	genome	CheckM_completeness	CheckM_contamination	
F6DB_bin.4	d__Bacteria	p__Firmicutes	c__Clostridia	o__Oscillospirales	f__Oscillospiraceae	g__Oscillibacter	s__Oscillibacter sp900066435	GCA_900066435.1	634	634_1	F6DB_bin.4.fa	F6DB_bin.4.fa	86.79	0.67	514
F6MY_bin.135	d__Bacteria	p__Firmicutes	c__Clostridia	o__Oscillospirales	f__Oscillospiraceae	g__Oscillibacter	s__Oscillibacter sp900066435	GCA_900066435.1	634	634_1	F6DB_bin.4.fa	F6MY_bin.135.fa	82.53	0.67	514
F68D_bin.98	d__Bacteria	p__Firmicutes	c__Clostridia	o__Oscillospirales	f__Oscillospiraceae	g__Ruminiclostridium_C	s__	N/A	598	598_5	F68D_bin.98.fa	F68D_bin.98.fa	84.97	1.72	515
F68G_bin.16	d__Bacteria	p__Firmicutes	c__Clostridia	o__Oscillospirales	f__Oscillospiraceae	g__Ruminiclostridium_C	s__	N/A	598	598_3	F68G_bin.16.fa	F68G_bin.16.fa	85.57	0.67	516
F68H_bin.38	d__Bacteria	p__Firmicutes	c__Clostridia	o__Oscillospirales	f__Oscillospiraceae	g__Ruminiclostridium_C	s__	N/A	598	598_7	F68H_bin.38.fa	F68H_bin.38.fa	82.42	0.67	517
F68P_bin.33	d__Bacteria	p__Firmicutes	c__Clostridia	o__Oscillospirales	f__Oscillospiraceae	g__Ruminiclostridium_C	s__	N/A	598	598_8	F68P_bin.33.fa	F68P_bin.33.fa	73.01	3.02	518
F68V_bin.81	d__Bacteria	p__Firmicutes	c__Clostridia	o__Oscillospirales	f__Oscillospiraceae	g__Ruminiclostridium_C	s__	N/A	598	598_4	F68V_bin.81.fa	F68V_bin.81.fa	55.35	2.9	519

Table S3. Taxonomic classifiation of the 2,421 detected MAGs

	Domain	Phylum	Class	Order	Family	Genus	Species	RefGenome	primary_cluster	secondary_cluster	repr_genome	genome	CheckM_completeness	CheckM_contamination	
F68W_bin.23	d__Bacteria	p__Firmicutes	c__Clostridia	o__Oscillospirales	f__Oscillospiraceae	g__Ruminiclostridium_C	s__	N/A	598	598_6	F68W_bin.23.fa	F68W_bin.23.fa	87.25	0.67	520
F6DH_bin.55	d__Bacteria	p__Firmicutes	c__Clostridia	o__Oscillospirales	f__Oscillospiraceae	g__Ruminiclostridium_C	s__	N/A	598	598_6	F68W_bin.23.fa	F6DH_bin.55.fa	66.11	0	521
F6DG_bin.26	d__Bacteria	p__Firmicutes	c__Clostridia	o__Oscillospirales	f__Oscillospiraceae	g__Ruminiclostridium_C	s__	N/A	598	598_1	F6DG_bin.26.fa	F6DG_bin.26.fa	82.21	0	522
F6MY_bin.67	d__Bacteria	p__Firmicutes	c__Clostridia	o__Oscillospirales	f__Oscillospiraceae	g__Ruminiclostridium_C	s__	N/A	598	598_2	F6MY_bin.67.fa	F6MY_bin.67.fa	60.6	0.69	523
F68M_bin.34	d__Bacteria	p__Firmicutes	c__Clostridia	o__Oscillospirales	f__Oscillospiraceae	g__Ruminiclostridium_C	s__	N/A	600	600_0	F68M_bin.34.fa	F68M_bin.34.fa	97.99	2.01	524
F6MX_bin.56	d__Bacteria	p__Firmicutes	c__Clostridia	o__Oscillospirales	f__Oscillospiraceae	g__Ruminiclostridium_C	s__	N/A	601	601_0	F6MX_bin.56.fa	F6MX_bin.56.fa	98.32	0.67	525
F6D7_bin.70	d__Bacteria	p__Firmicutes	c__Clostridia	o__Oscillospirales	f__Oscillospiraceae	g__Ruminiclostridium_C	s__Ruminiclostridium_C sp000435295	GCA_000435295.1	599	599_2	F6D7_bin.70.fa	F6D7_bin.70.fa	79.08	2.35	525

Table S3. Taxonomic classifiation of the 2,421 detected MAGs

	Domain	Phylum	Class	Order	Family	Genus	Species	RefGenome	primary_cluster	secondary_cluster	repr_genome	genome	CheckM_completeness	CheckM_contamination	
F68C_bin.77	d__Bacteria	p__Firmicutes	c__Clostridia	o__Oscillospirales	f__Oscillospiraceae	g__Ruminiclostridium_C	s__Ruminiclostridium_C sp000435295	GCA_000435295.1	599	599_1	F6DB_bin.70.fa	F68C_bin.77.fa	51.38	0	525
F68F_bin.83	d__Bacteria	p__Firmicutes	c__Clostridia	o__Oscillospirales	f__Oscillospiraceae	g__Ruminiclostridium_C	s__Ruminiclostridium_C sp000435295	GCA_000435295.1	599	599_1	F6DB_bin.70.fa	F68F_bin.83.fa	85.23	0.67	525
F68J_bin.1	d__Bacteria	p__Firmicutes	c__Clostridia	o__Oscillospirales	f__Oscillospiraceae	g__Ruminiclostridium_C	s__Ruminiclostridium_C sp000435295	GCA_000435295.1	599	599_1	F6DB_bin.70.fa	F68J_bin.1.fa	74.38	1.34	525
F68N_bin.23	d__Bacteria	p__Firmicutes	c__Clostridia	o__Oscillospirales	f__Oscillospiraceae	g__Ruminiclostridium_C	s__Ruminiclostridium_C sp000435295	GCA_000435295.1	599	599_1	F6DB_bin.70.fa	F68N_bin.23.fa	92.8	2.24	525
F68T_bin.14	d__Bacteria	p__Firmicutes	c__Clostridia	o__Oscillospirales	f__Oscillospiraceae	g__Ruminiclostridium_C	s__Ruminiclostridium_C sp000435295	GCA_000435295.1	599	599_1	F6DB_bin.70.fa	F68T_bin.14.fa	85.91	0.67	525

Table S3. Taxonomic classifiation of the 2,421 detected MAGs

	Domain	Phylum	Class	Order	Family	Genus	Species	RefGenome	primary_cluster	secondary_cluster	repr_genome	genome	CheckM_completeness	CheckM_contamination	
F6DB_bin.70	d__Bacteria	p__Firmicutes	c__Clostridia	o__Oscillospirales	f__Oscillospiraceae	g__Ruminiclostridium_C	s__Ruminiclostridium_C sp000435295	GCA_000435295.1	599	599_1	F6DB_bin.70.fa	F6DB_bin.70.fa	84.56	0	525
F6DE_bin.35	d__Bacteria	p__Firmicutes	c__Clostridia	o__Oscillospirales	f__Oscillospiraceae	g__Ruminiclostridium_C	s__Ruminiclostridium_C sp000435295	GCA_000435295.1	599	599_1	F6DB_bin.70.fa	F6DE_bin.35.fa	87.25	0.67	525
F68D_bin.16	d__Bacteria	p__Firmicutes	c__Clostridia	o__Oscillospirales	f__Oscillospiraceae	g__UBA1777	s__	N/A	654	654_2	F68D_bin.16.fa	F68D_bin.16.fa	87.18	0.4	526
F68R_bin.125	d__Bacteria	p__Firmicutes	c__Clostridia	o__Oscillospirales	f__Oscillospiraceae	g__UBA1777	s__	N/A	654	654_4	F68R_bin.125.fa	F68R_bin.125.fa	86.34	1.51	527
F68W_bin.51	d__Bacteria	p__Firmicutes	c__Clostridia	o__Oscillospirales	f__Oscillospiraceae	g__UBA1777	s__	N/A	654	654_1	F68W_bin.51.fa	F68W_bin.51.fa	83.82	0.17	528
F6DH_bin.127	d__Bacteria	p__Firmicutes	c__Clostridia	o__Oscillospirales	f__Oscillospiraceae	g__UBA1777	s__	N/A	654	654_1	F68W_bin.51.fa	F6DH_bin.127.fa	82.51	0	529
F6D7_bin.85	d__Bacteria	p__Firmicutes	c__Clostridia	o__Oscillospirales	f__Oscillospiraceae	g__UBA1777	s__	N/A	654	654_3	F6D7_bin.85.fa	F6D7_bin.85.fa	88.26	0	530
F6D9_bin.46	d__Bacteria	p__Firmicutes	c__Clostridia	o__Oscillospirales	f__Oscillospiraceae	g__UBA1777	s__	N/A	654	654_5	F6D9_bin.46.fa	F6D9_bin.46.fa	80.95	0	531

Table S3. Taxonomic classifiation of the 2,421 detected MAGs

	Domain	Phylum	Class	Order	Family	Genus	Species	RefGenome	primary_cluster	secondary_cluster	repr_genome	genome	CheckM_completeness	CheckM_contamination	
F68D_bin.100	d__Bacteria	p__Firmicutes	c__Clostridia	o__Oscillospirales	f__Oscillospiraceae	g__UBA1777	s__UBA1777 sp003150355	GCA_003150355.1	653	653_4	F68D_bin.100.fa	F68D_bin.100.fa	79.73	2.55	531
F68W_bin.12	d__Bacteria	p__Firmicutes	c__Clostridia	o__Oscillospirales	f__Oscillospiraceae	g__UBA1777	s__UBA1777 sp003150355	GCA_003150355.1	653	653_1	F68W_bin.12.fa	F68W_bin.12.fa	90.6	0	531
F6DH_bin.57	d__Bacteria	p__Firmicutes	c__Clostridia	o__Oscillospirales	f__Oscillospiraceae	g__UBA1777	s__UBA1777 sp003150355	GCA_003150355.1	653	653_1	F68W_bin.12.fa	F6DH_bin.57.fa	90.6	1.34	531
F6D8_bin.2	d__Bacteria	p__Firmicutes	c__Clostridia	o__Oscillospirales	f__Oscillospiraceae	g__UBA1777	s__UBA1777 sp003150355	GCA_003150355.1	653	653_3	F6D8_bin.2.fa	F6D8_bin.2.fa	74.34	1.72	531
F6MY_bin.79	d__Bacteria	p__Firmicutes	c__Clostridia	o__Oscillospirales	f__Oscillospiraceae	g__UBA1777	s__UBA1777 sp003150355	GCA_003150355.1	653	653_2	F6MY_bin.79.fa	F6MY_bin.79.fa	89.57	0.71	531
F68D_bin.15	d__Bacteria	p__Firmicutes	c__Clostridia	o__Oscillospirales	f__Oscillospiraceae	g__UBA5446	s__	N/A	650	650_1	F68G_bin.2.fa	F68D_bin.15.fa	88.39	1.34	532
F68G_bin.2	d__Bacteria	p__Firmicutes	c__Clostridia	o__Oscillospirales	f__Oscillospiraceae	g__UBA5446	s__	N/A	650	650_1	F68G_bin.2.fa	F68G_bin.2.fa	85.32	0.67	533
F68V_bin.82	d__Bacteria	p__Firmicutes	c__Clostridia	o__Oscillospirales	f__Oscillospiraceae	g__UBA5446	s__	N/A	650	650_2	F68V_bin.82.fa	F68V_bin.82.fa	87.03	1.01	534

Table S3. Taxonomic classifiation of the 2,421 detected MAGs

	Domain	Phylum	Class	Order	Family	Genus	Species	RefGenome	primary_cluster	secondary_cluster	repr_genome	genome	CheckM_completeness	CheckM_contamination	
F68D_bin.34	d__Bacteria	p__Firmicutes	c__Clostridia	o__Oscillospirales	f__Oscillospiraceae	g__UBA5446	s__	N/A	651	651_1	F68V_bin.27.fa	F68D_bin.34.fa	54.02	0	535
F68V_bin.27	d__Bacteria	p__Firmicutes	c__Clostridia	o__Oscillospirales	f__Oscillospiraceae	g__UBA5446	s__	N/A	651	651_1	F68V_bin.27.fa	F68V_bin.27.fa	92.26	0	536
F68V_bin.51	d__Bacteria	p__Firmicutes	c__Clostridia	o__Oscillospirales	f__Oscillospiraceae	g__UBA738	s__	N/A	656	656_0	F68V_bin.51.fa	F68V_bin.51.fa	57.05	1.72	537
F68C_bin.23	d__Bacteria	p__Firmicutes	c__Clostridia	o__Oscillospirales	f__Oscillospiraceae	g__UBA738	s__UBA738 sp003522945	GCA_003522945.1	655	655_10	F68C_bin.23.fa	F68C_bin.23.fa	72.45	4.31	537
F68D_bin.54	d__Bacteria	p__Firmicutes	c__Clostridia	o__Oscillospirales	f__Oscillospiraceae	g__UBA738	s__UBA738 sp003522945	GCA_003522945.1	655	655_1	F68D_bin.54.fa	F68D_bin.54.fa	82.27	2.13	537
F68F_bin.3	d__Bacteria	p__Firmicutes	c__Clostridia	o__Oscillospirales	f__Oscillospiraceae	g__UBA738	s__UBA738 sp003522945	GCA_003522945.1	655	655_12	F68F_bin.3.fa	F68F_bin.3.fa	69.98	2.85	537
F68G_bin.77	d__Bacteria	p__Firmicutes	c__Clostridia	o__Oscillospirales	f__Oscillospiraceae	g__UBA738	s__UBA738 sp003522945	GCA_003522945.1	655	655_6	F68G_bin.77.fa	F68G_bin.77.fa	90.44	1.01	537
F68H_bin.61	d__Bacteria	p__Firmicutes	c__Clostridia	o__Oscillospirales	f__Oscillospiraceae	g__UBA738	s__UBA738 sp003522945	GCA_003522945.1	655	655_2	F68H_bin.61.fa	F68H_bin.61.fa	78.06	0.67	537

Table S3. Taxonomic classifiation of the 2,421 detected MAGs

	Domain	Phylum	Class	Order	Family	Genus	Species	RefGenome	primary_cluster	secondary_cluster	repr_genome	genome	CheckM_completeness	CheckM_contamination	
F68M_bin.16	d__Bacteria	p__Firmicutes	c__Clostridia	o__Oscillospirales	f__Oscillospiraceae	g__UBA738	s__UBA738 sp003522945	GCA_003522945.1	655	655_8	F68M_bin.16.fa	F68M_bin.16.fa	88.46	0	537
F68R_bin.2	d__Bacteria	p__Firmicutes	c__Clostridia	o__Oscillospirales	f__Oscillospiraceae	g__UBA738	s__UBA738 sp003522945	GCA_003522945.1	655	655_11	F68R_bin.2.fa	F68R_bin.2.fa	88.59	0.67	537
F68W_bin.82	d__Bacteria	p__Firmicutes	c__Clostridia	o__Oscillospirales	f__Oscillospiraceae	g__UBA738	s__UBA738 sp003522945	GCA_003522945.1	655	655_3	F68W_bin.82.fa	F68W_bin.82.fa	89.26	0.67	537
F6DH_bin.104	d__Bacteria	p__Firmicutes	c__Clostridia	o__Oscillospirales	f__Oscillospiraceae	g__UBA738	s__UBA738 sp003522945	GCA_003522945.1	655	655_3	F68W_bin.82.fa	F6DH_bin.104.fa	89.26	1.01	537
F6D7_bin.79	d__Bacteria	p__Firmicutes	c__Clostridia	o__Oscillospirales	f__Oscillospiraceae	g__UBA738	s__UBA738 sp003522945	GCA_003522945.1	655	655_4	F6D7_bin.79.fa	F6D7_bin.79.fa	86.52	0.34	537
F6D8_bin.80	d__Bacteria	p__Firmicutes	c__Clostridia	o__Oscillospirales	f__Oscillospiraceae	g__UBA738	s__UBA738 sp003522945	GCA_003522945.1	655	655_9	F6D8_bin.80.fa	F6D8_bin.80.fa	75.72	0.95	537
F6DA_bin.74	d__Bacteria	p__Firmicutes	c__Clostridia	o__Oscillospirales	f__Oscillospiraceae	g__UBA738	s__UBA738 sp003522945	GCA_003522945.1	655	655_5	F6DA_bin.74.fa	F6DA_bin.74.fa	77.92	1.34	537

Table S3. Taxonomic classifiation of the 2,421 detected MAGs

	Domain	Phylum	Class	Order	Family	Genus	Species	RefGenome	primary_cluster	secondary_cluster	repr_genome	genome	CheckM_completeness	CheckM_contamination	
F6MY_bin.114	d__Bacteria	p__Firmicutes	c__Clostridia	o__Oscillospirales	f__Oscillospiraceae	g__UBA738	s__UBA738 sp003522945	GCA_003522945.1	655	655_7	F6MY_bin.114.fa	F6MY_bin.114.fa	90.6	0.67	537
F6MX_bin.137	d__Bacteria	p__Firmicutes	c__Clostridia	o__Oscillospirales	f__QAMX01	g__QAMX01	s__QAMX01 sp003149835	GCA_003149835.1	575	575_0	F6MX_bin.137.fa	F6MX_bin.137.fa	50.89	0.67	537
F6MY_bin.75	d__Bacteria	p__Firmicutes	c__Clostridia	o__Oscillospirales	f__Ruminococcaceae	g__	s__	N/A	179	179_0	F6MY_bin.75.fa	F6MY_bin.75.fa	91.39	0	538
F68G_bin.81	d__Bacteria	p__Firmicutes	c__Clostridia	o__Oscillospirales	f__Ruminococcaceae	g__	s__	N/A	415	415_0	F68G_bin.81.fa	F68G_bin.81.fa	74.6	1.79	539
F68T_bin.100	d__Bacteria	p__Firmicutes	c__Clostridia	o__Oscillospirales	f__Ruminococcaceae	g__	s__	N/A	461	461_0	F68T_bin.100.fa	F68T_bin.100.fa	81.57	2.54	540
F68V_bin.40	d__Bacteria	p__Firmicutes	c__Clostridia	o__Oscillospirales	f__Ruminococcaceae	g__	s__	N/A	463	463_1	F68V_bin.40.fa	F68V_bin.40.fa	56.79	1.72	541
F6DE_bin.94	d__Bacteria	p__Firmicutes	c__Clostridia	o__Oscillospirales	f__Ruminococcaceae	g__	s__	N/A	463	463_1	F68V_bin.40.fa	F6DE_bin.94.fa	54.35	1.9	542
F68J_bin.28	d__Bacteria	p__Firmicutes	c__Clostridia	o__Oscillospirales	f__Ruminococcaceae	g__	s__	N/A	576	576_1	F68J_bin.28.fa	F68J_bin.28.fa	67.86	1.79	543
F6MY_bin.53	d__Bacteria	p__Firmicutes	c__Clostridia	o__Oscillospirales	f__Ruminococcaceae	g__	s__	N/A	576	576_2	F6MY_bin.53.fa	F6MY_bin.53.fa	52.68	0	544

Table S3. Taxonomic classifiation of the 2,421 detected MAGs

	Domain	Phylum	Class	Order	Family	Genus	Species	RefGenome	primary_cluster	secondary_cluster	repr_genome	genome	CheckM_completeness	CheckM_contamination	
F6DG_bin.72	d__Bacteria	p__Firmicutes	c__Clostridia	o__Oscillospirales	f__Ruminococcaceae	g__	s__	N/A	578	578_0	F6DG_bin.72.fa	F6DG_bin.72.fa	91.31	0.11	545
F6DG_bin.108	d__Bacteria	p__Firmicutes	c__Clostridia	o__Oscillospirales	f__Ruminococcaceae	g__	s__	N/A	596	596_0	F6DG_bin.108.fa	F6DG_bin.108.fa	73.29	2.01	546
F6DA_bin.33	d__Bacteria	p__Firmicutes	c__Clostridia	o__Oscillospirales	f__Ruminococcaceae	g__	s__	N/A	603	603_0	F6DA_bin.33.fa	F6DA_bin.33.fa	93.96	1.69	547
F68U_bin.113	d__Bacteria	p__Firmicutes	c__Clostridia	o__Oscillospirales	f__Ruminococcaceae	g__Anaerotruncus	s__Anaerotruncus colihominis	GCF_000154565.1	453	453_0	F68U_bin.113.fa	F68U_bin.113.fa	61.8	4.77	547
F6MX_bin.17	d__Bacteria	p__Firmicutes	c__Clostridia	o__Oscillospirales	f__Ruminococcaceae	g__Anaerotruncus	s__Anaerotruncus rubiinfantis	GCF_900078395.1	451	451_0	F6MX_bin.17.fa	F6MX_bin.17.fa	57.97	3.19	547
F68M_bin.23	d__Bacteria	p__Firmicutes	c__Clostridia	o__Oscillospirales	f__Ruminococcaceae	g__Anaerotruncus	s__Anaerotruncus sp900199635	GCF_900199635.1	452	452_0	F68M_bin.23.fa	F68M_bin.23.fa	68.21	3.26	547
F68C_bin.14	d__Bacteria	p__Firmicutes	c__Clostridia	o__Oscillospirales	f__Ruminococcaceae	g__Angelakisella	s__	N/A	593	593_3	F68C_bin.14.fa	F68C_bin.14.fa	73.04	0	548
F68F_bin.67	d__Bacteria	p__Firmicutes	c__Clostridia	o__Oscillospirales	f__Ruminococcaceae	g__Angelakisella	s__	N/A	593	593_7	F68F_bin.67.fa	F68F_bin.67.fa	85.68	0	549

Table S3. Taxonomic classifiation of the 2,421 detected MAGs

	Domain	Phylum	Class	Order	Family	Genus	Species	RefGenome	primary_cluster	secondary_cluster	repr_genome	genome	CheckM_completeness	CheckM_contamination	
F68G_bin.64	d__Bacteria	p__Firmicutes	c__Clostridia	o__Oscillospirales	f__Ruminococcaceae	g__Angelakisella	s__	N/A	593	593_5	F68G_bin.64.fa	F68G_bin.64.fa	71.75	0.62	550
F68H_bin.59	d__Bacteria	p__Firmicutes	c__Clostridia	o__Oscillospirales	f__Ruminococcaceae	g__Angelakisella	s__	N/A	593	593_4	F68H_bin.59.fa	F68H_bin.59.fa	78.97	0	551
F68K_bin.93	d__Bacteria	p__Firmicutes	c__Clostridia	o__Oscillospirales	f__Ruminococcaceae	g__Angelakisella	s__	N/A	593	593_1	F6D7_bin.76.fa	F68K_bin.93.fa	72.93	0.4	552
F6D7_bin.76	d__Bacteria	p__Firmicutes	c__Clostridia	o__Oscillospirales	f__Ruminococcaceae	g__Angelakisella	s__	N/A	593	593_1	F6D7_bin.76.fa	F6D7_bin.76.fa	85.01	0.34	553
F6D8_bin.1	d__Bacteria	p__Firmicutes	c__Clostridia	o__Oscillospirales	f__Ruminococcaceae	g__Angelakisella	s__	N/A	593	593_8	F6D8_bin.1.fa	F6D8_bin.1.fa	78.64	0	554
F6DA_bin.78	d__Bacteria	p__Firmicutes	c__Clostridia	o__Oscillospirales	f__Ruminococcaceae	g__Angelakisella	s__	N/A	593	593_9	F6DA_bin.78.fa	F6DA_bin.78.fa	81.99	0.34	555
F6DE_bin.64	d__Bacteria	p__Firmicutes	c__Clostridia	o__Oscillospirales	f__Ruminococcaceae	g__Angelakisella	s__	N/A	593	593_10	F6DE_bin.64.fa	F6DE_bin.64.fa	67.67	0	556
F68N_bin.4	d__Bacteria	p__Firmicutes	c__Clostridia	o__Oscillospirales	f__Ruminococcaceae	g__Angelakisella	s__	N/A	593	593_6	F6MX_bin.46.fa	F68N_bin.4.fa	84.28	0.34	557
F6MX_bin.46	d__Bacteria	p__Firmicutes	c__Clostridia	o__Oscillospirales	f__Ruminococcaceae	g__Angelakisella	s__	N/A	593	593_6	F6MX_bin.46.fa	F6MX_bin.46.fa	87.02	0	558
F68U_bin.4	d__Bacteria	p__Firmicutes	c__Clostridia	o__Oscillospirales	f__Ruminococcaceae	g__Angelakisella	s__	N/A	593	593_2	F6MY_bin.38.fa	F68U_bin.4.fa	83.67	0.67	559

Table S3. Taxonomic classifiation of the 2,421 detected MAGs

	Domain	Phylum	Class	Order	Family	Genus	Species	RefGenome	primary_cluster	secondary_cluster	repr_genome	genome	CheckM_completeness	CheckM_contamination	
F6MY_bin.38	d__Bacteria	p__Firmicutes	c__Clostridia	o__Oscillospirales	f__Ruminococcaceae	g__Angelakisella	s__	N/A	593	593_2	F6MY_bin.38.fa	F6MY_bin.38.fa	83.33	0	560
F68D_bin.13	d__Bacteria	p__Firmicutes	c__Clostridia	o__Oscillospirales	f__Ruminococcaceae	g__Angelakisella	s__	N/A	594	594_3	F68D_bin.13.fa	F68D_bin.13.fa	85.85	1.34	561
F68V_bin.56	d__Bacteria	p__Firmicutes	c__Clostridia	o__Oscillospirales	f__Ruminococcaceae	g__Angelakisella	s__	N/A	594	594_2	F68V_bin.56.fa	F68V_bin.56.fa	78.73	1.34	562
F68W_bin.19	d__Bacteria	p__Firmicutes	c__Clostridia	o__Oscillospirales	f__Ruminococcaceae	g__Angelakisella	s__	N/A	594	594_1	F68W_bin.19.fa	F68W_bin.19.fa	86.8	0	563
F6DH_bin.67	d__Bacteria	p__Firmicutes	c__Clostridia	o__Oscillospirales	f__Ruminococcaceae	g__Angelakisella	s__	N/A	594	594_1	F68W_bin.19.fa	F6DH_bin.67.fa	81.71	0	564
F68U_bin.98	d__Bacteria	p__Firmicutes	c__Clostridia	o__Oscillospirales	f__Ruminococcaceae	g__CAG-115	s__CAG-115 sp003507295	GCA_003507295.1	181	181_1	F68U_bin.98.fa	F68U_bin.98.fa	98.66	0.67	564
F68W_bin.3	d__Bacteria	p__Firmicutes	c__Clostridia	o__Oscillospirales	f__Ruminococcaceae	g__CAG-115	s__CAG-115 sp003507295	GCA_003507295.1	181	181_1	F68U_bin.98.fa	F68W_bin.3.fa	97.32	0.67	564
F6DH_bin.5	d__Bacteria	p__Firmicutes	c__Clostridia	o__Oscillospirales	f__Ruminococcaceae	g__CAG-115	s__CAG-115 sp003507295	GCA_003507295.1	181	181_1	F68U_bin.98.fa	F6DH_bin.5.fa	96.64	0.67	564
F6D7_bin.28	d__Bacteria	p__Firmicutes	c__Clostridia	o__Oscillospirales	f__Ruminococcaceae	g__CAG-115	s__CAG-115 sp003507295	GCA_003507295.1	181	181_2	F6D7_bin.28.fa	F6D7_bin.28.fa	98.66	1.01	564

Table S3. Taxonomic classifiation of the 2,421 detected MAGs

	Domain	Phylum	Class	Order	Family	Genus	Species	RefGenome	primary_cluster	secondary_cluster	repr_genome	genome	CheckM_completeness	CheckM_contamination	
F6D8_bin.52	d__Bacteria	p__Firmicutes	c__Clostridia	o__Oscillospirales	f__Ruminococcaceae	g__CAG-353	s__CAG-353 sp900066885	GCA_900066885.1	494	494_1	F6D8_bin.52.fa	F6D8_bin.52.fa	97.27	0	564
F6D9_bin.44	d__Bacteria	p__Firmicutes	c__Clostridia	o__Oscillospirales	f__Ruminococcaceae	g__CAG-353	s__CAG-353 sp900066885	GCA_900066885.1	494	494_1	F6D8_bin.52.fa	F6D9_bin.44.fa	72.68	2.57	564
F68V_bin.126	d__Bacteria	p__Firmicutes	c__Clostridia	o__Oscillospirales	f__Ruminococcaceae	g__D5	s__D5 sp900113995	GCF_900113995.1	450	450_1	F6DB_bin.45.fa	F68V_bin.126.fa	52.15	0.86	564
F6DB_bin.45	d__Bacteria	p__Firmicutes	c__Clostridia	o__Oscillospirales	f__Ruminococcaceae	g__D5	s__D5 sp900113995	GCF_900113995.1	450	450_1	F6DB_bin.45.fa	F6DB_bin.45.fa	95.3	0.67	564
F6MY_bin.18	d__Bacteria	p__Firmicutes	c__Clostridia	o__Oscillospirales	f__Ruminococcaceae	g__D5	s__D5 sp900113995	GCF_900113995.1	450	450_1	F6DB_bin.45.fa	F6MY_bin.18.fa	91.37	0.67	564
F68T_bin.95	d__Bacteria	p__Firmicutes	c__Clostridia	o__Oscillospirales	f__Ruminococcaceae	g__Faecalibacterium	s__	N/A	580	580_2	F68T_bin.95.fa	F68T_bin.95.fa	52.64	0.5	565
F6D7_bin.74	d__Bacteria	p__Firmicutes	c__Clostridia	o__Oscillospirales	f__Ruminococcaceae	g__Faecalibacterium	s__	N/A	580	580_13	F6D7_bin.74.fa	F6D7_bin.74.fa	93.76	4.37	566
F68C_bin.27	d__Bacteria	p__Firmicutes	c__Clostridia	o__Oscillospirales	f__Ruminococcaceae	g__Faecalibacterium	s__	N/A	582	582_6	F68C_bin.27.fa	F68C_bin.27.fa	91.16	0.34	567
F68F_bin.40	d__Bacteria	p__Firmicutes	c__Clostridia	o__Oscillospirales	f__Ruminococcaceae	g__Faecalibacterium	s__	N/A	582	582_7	F68F_bin.40.fa	F68F_bin.40.fa	58.04	0	568

Table S3. Taxonomic classifiation of the 2,421 detected MAGs

	Domain	Phylum	Class	Order	Family	Genus	Species	RefGenome	primary_cluster	secondary_cluster	repr_genome	genome	CheckM_completeness	CheckM_contamination	
F68K_bin.110	d__Bacteria	p__Firmicutes	c__Clostridia	o__Oscillospirales	f__Ruminococcaceae	g__Faecalibacterium	s__	N/A	582	582_1	F68K_bin.110.fa	F68K_bin.110.fa	67.86	0	569
F68N_bin.76	d__Bacteria	p__Firmicutes	c__Clostridia	o__Oscillospirales	f__Ruminococcaceae	g__Faecalibacterium	s__	N/A	582	582_4	F68N_bin.76.fa	F68N_bin.76.fa	93.98	1.87	570
F68U_bin.45	d__Bacteria	p__Firmicutes	c__Clostridia	o__Oscillospirales	f__Ruminococcaceae	g__Faecalibacterium	s__	N/A	582	582_5	F68U_bin.45.fa	F68U_bin.45.fa	80.01	1.7	571
F6DA_bin.28	d__Bacteria	p__Firmicutes	c__Clostridia	o__Oscillospirales	f__Ruminococcaceae	g__Faecalibacterium	s__	N/A	582	582_8	F6DA_bin.28.fa	F6DA_bin.28.fa	87.76	0.68	572
F6DE_bin.41	d__Bacteria	p__Firmicutes	c__Clostridia	o__Oscillospirales	f__Ruminococcaceae	g__Faecalibacterium	s__	N/A	582	582_2	F6DE_bin.41.fa	F6DE_bin.41.fa	70.89	0	573
F6DH_bin.53	d__Bacteria	p__Firmicutes	c__Clostridia	o__Oscillospirales	f__Ruminococcaceae	g__Faecalibacterium	s__	N/A	582	582_3	F6DH_bin.53.fa	F6DH_bin.53.fa	64.64	3.57	574
F6MY_bin.101	d__Bacteria	p__Firmicutes	c__Clostridia	o__Oscillospirales	f__Ruminococcaceae	g__Faecalibacterium	s__	N/A	583	583_11	F6MY_bin.101.fa	F6MY_bin.101.fa	67.48	0	575
F68W_bin.8	d__Bacteria	p__Firmicutes	c__Clostridia	o__Oscillospirales	f__Ruminococcaceae	g__Faecalibacterium	s__	N/A	584	584_1	F68W_bin.8.fa	F68W_bin.8.fa	94.8	1.87	576
F6DH_bin.56	d__Bacteria	p__Firmicutes	c__Clostridia	o__Oscillospirales	f__Ruminococcaceae	g__Faecalibacterium	s__	N/A	584	584_1	F68W_bin.8.fa	F6DH_bin.56.fa	80.92	0	577

Table S3. Taxonomic classifiation of the 2,421 detected MAGs

	Domain	Phylum	Class	Order	Family	Genus	Species	RefGenome	primary_cluster	secondary_cluster	repr_genome	genome	CheckM_completeness	CheckM_contamination	
F68F_bin.54	d__Bacteria	p__Firmicutes	c__Clostridia	o__Oscillospirales	f__Ruminococcaceae	g__Faecalibacterium	s__Faecalibacterium prausnitzii_C	GCF_000162015.1	581	581_5	F68F_bin.54.fa	F68F_bin.54.fa	57.14	0	577
F68H_bin.27	d__Bacteria	p__Firmicutes	c__Clostridia	o__Oscillospirales	f__Ruminococcaceae	g__Faecalibacterium	s__Faecalibacterium prausnitzii_C	GCF_000162015.1	581	581_4	F68H_bin.27.fa	F68H_bin.27.fa	78.04	1.79	577
F68K_bin.5	d__Bacteria	p__Firmicutes	c__Clostridia	o__Oscillospirales	f__Ruminococcaceae	g__Faecalibacterium	s__Faecalibacterium prausnitzii_C	GCF_000162015.1	581	581_1	F68K_bin.5.fa	F68K_bin.5.fa	73.29	0.68	577
F68N_bin.42	d__Bacteria	p__Firmicutes	c__Clostridia	o__Oscillospirales	f__Ruminococcaceae	g__Faecalibacterium	s__Faecalibacterium prausnitzii_C	GCF_000162015.1	581	581_6	F68N_bin.42.fa	F68N_bin.42.fa	57.5	1.79	577
F68P_bin.7	d__Bacteria	p__Firmicutes	c__Clostridia	o__Oscillospirales	f__Ruminococcaceae	g__Faecalibacterium	s__Faecalibacterium prausnitzii_C	GCF_000162015.1	581	581_2	F68P_bin.7.fa	F68P_bin.7.fa	97.52	1.53	577
F68U_bin.64	d__Bacteria	p__Firmicutes	c__Clostridia	o__Oscillospirales	f__Ruminococcaceae	g__Faecalibacterium	s__Faecalibacterium prausnitzii_C	GCF_000162015.1	581	581_8	F68U_bin.64.fa	F68U_bin.64.fa	81.31	4.46	577

Table S3. Taxonomic classifiation of the 2,421 detected MAGs

	Domain	Phylum	Class	Order	Family	Genus	Species	RefGenome	primary_cluster	secondary_cluster	repr_genome	genome	CheckM_completeness	CheckM_contamination	
F6D7_bin.41	d__Bacteria	p__Firmicutes	c__Clostridia	o__Oscillospirales	f__Ruminococcaceae	g__Faecalibacterium	s__Faecalibacterium prausnitzii_C	GCF_000162015.1	581	581_7	F6D7_bin.41.fa	F6D7_bin.41.fa	70.42	0.77	577
F6DA_bin.51	d__Bacteria	p__Firmicutes	c__Clostridia	o__Oscillospirales	f__Ruminococcaceae	g__Faecalibacterium	s__Faecalibacterium prausnitzii_C	GCF_000162015.1	581	581_9	F6DA_bin.51.fa	F6DA_bin.51.fa	98.49	2.72	577
F6DB_bin.98	d__Bacteria	p__Firmicutes	c__Clostridia	o__Oscillospirales	f__Ruminococcaceae	g__Faecalibacterium	s__Faecalibacterium prausnitzii_C	GCF_000162015.1	581	581_3	F6DB_bin.98.fa	F6DB_bin.98.fa	98.21	1.87	577
F6DG_bin.85	d__Bacteria	p__Firmicutes	c__Clostridia	o__Oscillospirales	f__Ruminococcaceae	g__Faecalibacterium	s__Faecalibacterium prausnitzii_C	GCF_000162015.1	581	581_10	F6DG_bin.85.fa	F6DG_bin.85.fa	65.67	0.32	577
F68P_bin.41	d__Bacteria	p__Firmicutes	c__Clostridia	o__Oscillospirales	f__Ruminococcaceae	g__Faecalibacterium	s__Faecalibacterium prausnitzii_D	GCF_002549755.1	584	584_2	F68P_bin.41.fa	F68P_bin.41.fa	93.01	1.02	577
F68R_bin.112	d__Bacteria	p__Firmicutes	c__Clostridia	o__Oscillospirales	f__Ruminococcaceae	g__Faecalibacterium	s__Faecalibacterium prausnitzii_D	GCF_002549755.1	584	584_3	F68R_bin.112.fa	F68R_bin.112.fa	75.52	3.57	577

Table S3. Taxonomic classifiation of the 2,421 detected MAGs

	Domain	Phylum	Class	Order	Family	Genus	Species	RefGenome	primary_cluster	secondary_cluster	repr_genome	genome	CheckM_completeness	CheckM_contamination	
F6D8_bin.23	d__Bacteria	p__Firmicutes	c__Clostridia	o__Oscillospirales	f__Ruminococcaceae	g__Faecalibacterium	s__Faecalibacterium prausnitzii_D	GCF_002549755.1	584	584_4	F6D8_bin.23.fa	F6D8_bin.23.fa	57.14	0	577
F68C_bin.64	d__Bacteria	p__Firmicutes	c__Clostridia	o__Oscillospirales	f__Ruminococcaceae	g__Faecalibacterium	s__Faecalibacterium prausnitzii_E	GCF_002550035.1	579	579_1	F68C_bin.64.fa	F68C_bin.64.fa	52.47	1.79	577
F6MY_bin.69	d__Bacteria	p__Firmicutes	c__Clostridia	o__Oscillospirales	f__Ruminococcaceae	g__Faecalibacterium	s__Faecalibacterium prausnitzii_E	GCF_002550035.1	579	579_2	F6MY_bin.69.fa	F6MY_bin.69.fa	58.39	0	577
F68C_bin.103	d__Bacteria	p__Firmicutes	c__Clostridia	o__Oscillospirales	f__Ruminococcaceae	g__Faecalibacterium	s__Faecalibacterium prausnitzii_G	GCA_003293635.1	580	580_10	F68C_bin.103.fa	F68C_bin.103.fa	58.33	1.84	577
F68E_bin.1	d__Bacteria	p__Firmicutes	c__Clostridia	o__Oscillospirales	f__Ruminococcaceae	g__Faecalibacterium	s__Faecalibacterium prausnitzii_G	GCA_003293635.1	580	580_5	F68E_bin.1.fa	F68E_bin.1.fa	87.64	4.33	577
F68F_bin.66	d__Bacteria	p__Firmicutes	c__Clostridia	o__Oscillospirales	f__Ruminococcaceae	g__Faecalibacterium	s__Faecalibacterium prausnitzii_G	GCA_003293635.1	580	580_3	F68F_bin.66.fa	F68F_bin.66.fa	86.81	3.22	577

Table S3. Taxonomic classifiation of the 2,421 detected MAGs

	Domain	Phylum	Class	Order	Family	Genus	Species	RefGenome	primary_cluster	secondary_cluster	repr_genome	genome	CheckM_completeness	CheckM_contamination	
F68G_bin.25	d__Bacteria	p__Firmicutes	c__Clostridia	o__Oscillospirales	f__Ruminococcaceae	g__Faecalibacterium	s__Faecalibacterium prausnitzii_G	GCA_003293635.1	580	580_9	F68G_bin.25.fa	F68G_bin.25.fa	61.85	3.74	577
F68J_bin.69	d__Bacteria	p__Firmicutes	c__Clostridia	o__Oscillospirales	f__Ruminococcaceae	g__Faecalibacterium	s__Faecalibacterium prausnitzii_G	GCA_003293635.1	580	580_12	F68J_bin.69.fa	F68J_bin.69.fa	95.63	0.17	577
F68K_bin.73	d__Bacteria	p__Firmicutes	c__Clostridia	o__Oscillospirales	f__Ruminococcaceae	g__Faecalibacterium	s__Faecalibacterium prausnitzii_G	GCA_003293635.1	580	580_1	F68K_bin.73.fa	F68K_bin.73.fa	62.07	1.42	577
F68N_bin.39	d__Bacteria	p__Firmicutes	c__Clostridia	o__Oscillospirales	f__Ruminococcaceae	g__Faecalibacterium	s__Faecalibacterium prausnitzii_G	GCA_003293635.1	580	580_4	F68N_bin.39.fa	F68N_bin.39.fa	94.1	2.04	577
F68P_bin.43	d__Bacteria	p__Firmicutes	c__Clostridia	o__Oscillospirales	f__Ruminococcaceae	g__Faecalibacterium	s__Faecalibacterium prausnitzii_G	GCA_003293635.1	580	580_11	F68P_bin.43.fa	F68P_bin.43.fa	92.43	0.91	577
F68R_bin.114	d__Bacteria	p__Firmicutes	c__Clostridia	o__Oscillospirales	f__Ruminococcaceae	g__Faecalibacterium	s__Faecalibacterium prausnitzii_G	GCA_003293635.1	580	580_6	F68R_bin.114.fa	F68R_bin.114.fa	58.36	1.79	577

Table S3. Taxonomic classifiation of the 2,421 detected MAGs

	Domain	Phylum	Class	Order	Family	Genus	Species	RefGenome	primary_cluster	secondary_cluster	repr_genome	genome	CheckM_completeness	CheckM_contamination	
F6DB_bin.69	d__Bacteria	p__Firmicutes	c__Clostridia	o__Oscillospirales	f__Ruminococcaceae	g__Faecalibacterium	s__Faecalibacterium prausnitzii_G	GCA_003293635.1	580	580_7	F6DB_bin.69.fa	F6DB_bin.69.fa	89.59	4.95	577
F6DE_bin.8	d__Bacteria	p__Firmicutes	c__Clostridia	o__Oscillospirales	f__Ruminococcaceae	g__Faecalibacterium	s__Faecalibacterium prausnitzii_G	GCA_003293635.1	580	580_8	F6DE_bin.8.fa	F6DE_bin.8.fa	92.09	3.64	577
F6DA_bin.13	d__Bacteria	p__Firmicutes	c__Clostridia	o__Oscillospirales	f__Ruminococcaceae	g__Faecalibacterium	s__Faecalibacterium prausnitzii_J	GCA_003287405.1	583	583_10	F6DA_bin.13.fa	F6DA_bin.13.fa	97.73	0.84	577
F68C_bin.73	d__Bacteria	p__Firmicutes	c__Clostridia	o__Oscillospirales	f__Ruminococcaceae	g__Faecalibacterium	s__Faecalibacterium prausnitzii_K	GCA_003312465.1	583	583_4	F68C_bin.73.fa	F68C_bin.73.fa	68.83	2.78	577
F68G_bin.76	d__Bacteria	p__Firmicutes	c__Clostridia	o__Oscillospirales	f__Ruminococcaceae	g__Faecalibacterium	s__Faecalibacterium prausnitzii_K	GCA_003312465.1	583	583_5	F68G_bin.76.fa	F68G_bin.76.fa	91.84	1.87	577
F68K_bin.1	d__Bacteria	p__Firmicutes	c__Clostridia	o__Oscillospirales	f__Ruminococcaceae	g__Faecalibacterium	s__Faecalibacterium prausnitzii_K	GCA_003312465.1	583	583_9	F68K_bin.1.fa	F68K_bin.1.fa	92.12	0	577

Table S3. Taxonomic classifiation of the 2,421 detected MAGs

	Domain	Phylum	Class	Order	Family	Genus	Species	RefGenome	primary_cluster	secondary_cluster	repr_genome	genome	CheckM_completeness	CheckM_contamination	
F68R_bin.117	d__Bacteria	p__Firmicutes	c__Clostridia	o__Oscillospirales	f__Ruminococcaceae	g__Faecalibacterium	s__Faecalibacterium prausnitzii_K	GCA_003312465.1	583	583_6	F68R_bin.117.fa	F68R_bin.117.fa	88.18	0.51	577
F68U_bin.28	d__Bacteria	p__Firmicutes	c__Clostridia	o__Oscillospirales	f__Ruminococcaceae	g__Faecalibacterium	s__Faecalibacterium prausnitzii_K	GCA_003312465.1	583	583_7	F68U_bin.28.fa	F68U_bin.28.fa	79.2	4.65	577
F68W_bin.13	d__Bacteria	p__Firmicutes	c__Clostridia	o__Oscillospirales	f__Ruminococcaceae	g__Faecalibacterium	s__Faecalibacterium prausnitzii_K	GCA_003312465.1	583	583_8	F68W_bin.13.fa	F68W_bin.13.fa	94.33	1.53	577
F6DH_bin.12	d__Bacteria	p__Firmicutes	c__Clostridia	o__Oscillospirales	f__Ruminococcaceae	g__Faecalibacterium	s__Faecalibacterium prausnitzii_K	GCA_003312465.1	583	583_8	F68W_bin.13.fa	F6DH_bin.12.fa	86.96	2.68	577
F6D8_bin.82	d__Bacteria	p__Firmicutes	c__Clostridia	o__Oscillospirales	f__Ruminococcaceae	g__Faecalibacterium	s__Faecalibacterium prausnitzii_K	GCA_003312465.1	583	583_1	F6DB_bin.21.fa	F6D8_bin.82.fa	51.25	0	577
F6DB_bin.21	d__Bacteria	p__Firmicutes	c__Clostridia	o__Oscillospirales	f__Ruminococcaceae	g__Faecalibacterium	s__Faecalibacterium prausnitzii_K	GCA_003312465.1	583	583_1	F6DB_bin.21.fa	F6DB_bin.21.fa	96.12	1.52	577

Table S3. Taxonomic classifiation of the 2,421 detected MAGs

	Domain	Phylum	Class	Order	Family	Genus	Species	RefGenome	primary_cluster	secondary_cluster	repr_genome	genome	CheckM_completeness	CheckM_contamination	
F6DE_bin.10	d__Bacteria	p__Firmicutes	c__Clostridia	o__Oscillospirales	f__Ruminococcaceae	g__Faecalibacterium	s__Faecalibacterium prausnitzii_K	GCA_003312465.1	583	583_2	F6DE_bin.10.fa	F6DE_bin.10.fa	95.27	1.87	577
F6MY_bin.23	d__Bacteria	p__Firmicutes	c__Clostridia	o__Oscillospirales	f__Ruminococcaceae	g__Faecalibacterium	s__Faecalibacterium prausnitzii_K	GCA_003312465.1	583	583_3	F6MY_bin.23.fa	F6MY_bin.23.fa	53.63	3.57	577
F68D_bin.85	d__Bacteria	p__Firmicutes	c__Clostridia	o__Oscillospirales	f__Ruminococcaceae	g__Fournierella	s__	N/A	577	577_3	F68D_bin.85.fa	F68D_bin.85.fa	52.54	2.68	578
F68M_bin.85	d__Bacteria	p__Firmicutes	c__Clostridia	o__Oscillospirales	f__Ruminococcaceae	g__Fournierella	s__	N/A	577	577_1	F68M_bin.85.fa	F68M_bin.85.fa	99.32	1.02	579
F6MX_bin.126	d__Bacteria	p__Firmicutes	c__Clostridia	o__Oscillospirales	f__Ruminococcaceae	g__Fournierella	s__	N/A	577	577_2	F6MX_bin.126.fa	F6MX_bin.126.fa	95.24	0	580
F68F_bin.76	d__Bacteria	p__Firmicutes	c__Clostridia	o__Oscillospirales	f__Ruminococcaceae	g__Gemmiger	s__	N/A	586	586_3	F68F_bin.76.fa	F68F_bin.76.fa	89.54	3.65	581
F6D7_bin.1	d__Bacteria	p__Firmicutes	c__Clostridia	o__Oscillospirales	f__Ruminococcaceae	g__Gemmiger	s__	N/A	586	586_4	F6D7_bin.1.fa	F6D7_bin.1.fa	91.61	0.81	582
F6DB_bin.115	d__Bacteria	p__Firmicutes	c__Clostridia	o__Oscillospirales	f__Ruminococcaceae	g__Gemmiger	s__	N/A	586	586_1	F6DB_bin.115.fa	F6DB_bin.115.fa	79.71	3.1	583
F6MY_bin.86	d__Bacteria	p__Firmicutes	c__Clostridia	o__Oscillospirales	f__Ruminococcaceae	g__Gemmiger	s__	N/A	586	586_2	F6MY_bin.86.fa	F6MY_bin.86.fa	85.14	2.27	584

Table S3. Taxonomic classifiation of the 2,421 detected MAGs

	Domain	Phylum	Class	Order	Family	Genus	Species	RefGenome	primary_cluster	secondary_cluster	repr_genome	genome	CheckM_completeness	CheckM_contamination	
F68F_bin.22	d__Bacteria	p__Firmicutes	c__Clostridia	o__Oscillospirales	f__Ruminococcaceae	g__Gemmiger	s__	N/A	587	587_3	F68F_bin.22.fa	F68F_bin.22.fa	81.8	3.4	585
F68V_bin.100	d__Bacteria	p__Firmicutes	c__Clostridia	o__Oscillospirales	f__Ruminococcaceae	g__Gemmiger	s__	N/A	587	587_2	F68V_bin.100.fa	F68V_bin.100.fa	70.75	0	586
F68W_bin.77	d__Bacteria	p__Firmicutes	c__Clostridia	o__Oscillospirales	f__Ruminococcaceae	g__Gemmiger	s__	N/A	587	587_1	F68W_bin.77.fa	F68W_bin.77.fa	94.19	2.04	587
F6DH_bin.102	d__Bacteria	p__Firmicutes	c__Clostridia	o__Oscillospirales	f__Ruminococcaceae	g__Gemmiger	s__	N/A	587	587_1	F68W_bin.77.fa	F6DH_bin.102.fa	92.38	2.45	588
F68R_bin.127	d__Bacteria	p__Firmicutes	c__Clostridia	o__Oscillospirales	f__Ruminococcaceae	g__Gemmiger	s__	N/A	589	589_5	F68R_bin.127.fa	F68R_bin.127.fa	88.38	4.25	589
F68T_bin.88	d__Bacteria	p__Firmicutes	c__Clostridia	o__Oscillospirales	f__Ruminococcaceae	g__Gemmiger	s__	N/A	589	589_1	F68T_bin.88.fa	F68T_bin.88.fa	96.6	0	590
F68W_bin.59	d__Bacteria	p__Firmicutes	c__Clostridia	o__Oscillospirales	f__Ruminococcaceae	g__Gemmiger	s__	N/A	589	589_4	F68W_bin.59.fa	F68W_bin.59.fa	70.54	0	591
F6DH_bin.20	d__Bacteria	p__Firmicutes	c__Clostridia	o__Oscillospirales	f__Ruminococcaceae	g__Gemmiger	s__	N/A	589	589_4	F68W_bin.59.fa	F6DH_bin.20.fa	57.31	0	592
F6DB_bin.27	d__Bacteria	p__Firmicutes	c__Clostridia	o__Oscillospirales	f__Ruminococcaceae	g__Gemmiger	s__	N/A	589	589_2	F6DB_bin.27.fa	F6DB_bin.27.fa	72.55	1.02	593
F6DE_bin.30	d__Bacteria	p__Firmicutes	c__Clostridia	o__Oscillospirales	f__Ruminococcaceae	g__Gemmiger	s__	N/A	589	589_3	F6DE_bin.30.fa	F6DE_bin.30.fa	95.24	0.68	594

Table S3. Taxonomic classifiation of the 2,421 detected MAGs

	Domain	Phylum	Class	Order	Family	Genus	Species	RefGenome	primary_cluster	secondary_cluster	repr_genome	genome	CheckM_completeness	CheckM_contamination	
F68C_bin.40	d__Bacteria	p__Firmicutes	c__Clostridia	o__Oscillospirales	f__Ruminococcaceae	g__Gemmiger	s__	N/A	590	590_0	F68C_bin.40.fa	F68C_bin.40.fa	76.67	2.89	595
F68D_bin.108	d__Bacteria	p__Firmicutes	c__Clostridia	o__Oscillospirales	f__Ruminococcaceae	g__Gemmiger	s__Gemmiger formicilis	GCF_900167555.1	588	588_4	F68D_bin.108.fa	F68D_bin.108.fa	68.16	0	595
F68K_bin.10	d__Bacteria	p__Firmicutes	c__Clostridia	o__Oscillospirales	f__Ruminococcaceae	g__Gemmiger	s__Gemmiger formicilis	GCF_900167555.1	588	588_3	F68K_bin.10.fa	F68K_bin.10.fa	93.2	0.34	595
F68P_bin.12	d__Bacteria	p__Firmicutes	c__Clostridia	o__Oscillospirales	f__Ruminococcaceae	g__Gemmiger	s__Gemmiger formicilis	GCF_900167555.1	588	588_5	F68P_bin.12.fa	F68P_bin.12.fa	76.85	1.59	595
F6DA_bin.52	d__Bacteria	p__Firmicutes	c__Clostridia	o__Oscillospirales	f__Ruminococcaceae	g__Gemmiger	s__Gemmiger formicilis	GCF_900167555.1	588	588_1	F6DA_bin.52.fa	F6DA_bin.52.fa	89.47	0.68	595
F6MY_bin.1	d__Bacteria	p__Firmicutes	c__Clostridia	o__Oscillospirales	f__Ruminococcaceae	g__Gemmiger	s__Gemmiger formicilis	GCF_900167555.1	588	588_2	F6MY_bin.1.fa	F6MY_bin.1.fa	83.9	0.68	595
F68C_bin.42	d__Bacteria	p__Firmicutes	c__Clostridia	o__Oscillospirales	f__Ruminococcaceae	g__Gemmiger	s__Gemmiger sp003476825	GCA_003476825.1	585	585_5	F68C_bin.42.fa	F68C_bin.42.fa	91.1	2.04	595
F68F_bin.9	d__Bacteria	p__Firmicutes	c__Clostridia	o__Oscillospirales	f__Ruminococcaceae	g__Gemmiger	s__Gemmiger sp003476825	GCA_003476825.1	585	585_3	F68F_bin.9.fa	F68F_bin.9.fa	96.64	0.68	595

Table S3. Taxonomic classifiation of the 2,421 detected MAGs

	Domain	Phylum	Class	Order	Family	Genus	Species	RefGenome	primary_cluster	secondary_cluster	repr_genome	genome	CheckM_completeness	CheckM_contamination	
F68V_bin.87	d__Bacteria	p__Firmicutes	c__Clostridia	o__Oscillospirales	f__Ruminococcaceae	g__Gemmiger	s__Gemmiger sp003476825	GCA_003476825.1	585	585_3	F68F_bin.9.fa	F68V_bin.87.fa	84.43	3.06	595
F6MY_bin.134	d__Bacteria	p__Firmicutes	c__Clostridia	o__Oscillospirales	f__Ruminococcaceae	g__Gemmiger	s__Gemmiger sp003476825	GCA_003476825.1	585	585_3	F68F_bin.9.fa	F6MY_bin.134.fa	95.56	2.27	595
F68R_bin.116	d__Bacteria	p__Firmicutes	c__Clostridia	o__Oscillospirales	f__Ruminococcaceae	g__Gemmiger	s__Gemmiger sp003476825	GCA_003476825.1	585	585_4	F68R_bin.116.fa	F68R_bin.116.fa	74.52	3.13	595
F68W_bin.79	d__Bacteria	p__Firmicutes	c__Clostridia	o__Oscillospirales	f__Ruminococcaceae	g__Gemmiger	s__Gemmiger sp003476825	GCA_003476825.1	585	585_1	F68W_bin.79.fa	F68W_bin.79.fa	74.25	0.89	595
F6DH_bin.41	d__Bacteria	p__Firmicutes	c__Clostridia	o__Oscillospirales	f__Ruminococcaceae	g__Gemmiger	s__Gemmiger sp003476825	GCA_003476825.1	585	585_1	F68W_bin.79.fa	F6DH_bin.41.fa	78.39	4.08	595
F68P_bin.23	d__Bacteria	p__Firmicutes	c__Clostridia	o__Oscillospirales	f__Ruminococcaceae	g__Gemmiger	s__Gemmiger sp003476825	GCA_003476825.1	585	585_2	F6DB_bin.19.fa	F68P_bin.23.fa	60.71	1.79	595
F6DB_bin.19	d__Bacteria	p__Firmicutes	c__Clostridia	o__Oscillospirales	f__Ruminococcaceae	g__Gemmiger	s__Gemmiger sp003476825	GCA_003476825.1	585	585_2	F6DB_bin.19.fa	F6DB_bin.19.fa	96.58	0.28	595

Table S3. Taxonomic classifiation of the 2,421 detected MAGs

	Domain	Phylum	Class	Order	Family	Genus	Species	RefGenome	primary_cluster	secondary_cluster	repr_genome	genome	CheckM_completeness	CheckM_contamination	
F6DG_bin.32	d__Bacteria	p__Firmicutes	c__Clostridia	o__Oscillospirales	f__Ruminococcaceae	g__Gemmiger	s__Gemmiger sp003476825	GCA_003476825.1	585	585_6	F6DG_bin.32.fa	F6DG_bin.32.fa	94.53	1.02	595
F68F_bin.20	d__Bacteria	p__Firmicutes	c__Clostridia	o__Oscillospirales	f__Ruminococcaceae	g__Negativibacillus	s__Negativibacillus massiliensis	GCF_900148495.1	465	465_5	F68F_bin.20.fa	F68F_bin.20.fa	55.68	0.67	595
F68N_bin.58	d__Bacteria	p__Firmicutes	c__Clostridia	o__Oscillospirales	f__Ruminococcaceae	g__Negativibacillus	s__Negativibacillus massiliensis	GCF_900148495.1	465	465_2	F68N_bin.58.fa	F68N_bin.58.fa	93.94	0.67	595
F68P_bin.28	d__Bacteria	p__Firmicutes	c__Clostridia	o__Oscillospirales	f__Ruminococcaceae	g__Negativibacillus	s__Negativibacillus massiliensis	GCF_900148495.1	465	465_3	F68P_bin.28.fa	F68P_bin.28.fa	77.84	0.9	595
F68K_bin.88	d__Bacteria	p__Firmicutes	c__Clostridia	o__Oscillospirales	f__Ruminococcaceae	g__Negativibacillus	s__Negativibacillus massiliensis	GCF_900148495.1	465	465_1	F68T_bin.35.fa	F68K_bin.88.fa	97.99	1.45	595
F68T_bin.35	d__Bacteria	p__Firmicutes	c__Clostridia	o__Oscillospirales	f__Ruminococcaceae	g__Negativibacillus	s__Negativibacillus massiliensis	GCF_900148495.1	465	465_1	F68T_bin.35.fa	F68T_bin.35.fa	91.32	0	595

Table S3. Taxonomic classifiation of the 2,421 detected MAGs

	Domain	Phylum	Class	Order	Family	Genus	Species	RefGenome	primary_cluster	secondary_cluster	repr_genome	genome	CheckM_completeness	CheckM_contamination	
F68U_bin.74	d__Bacteria	p__Firmicutes	c__Clostridia	o__Oscillospirales	f__Ruminococcaceae	g__Negativibacillus	s__Negativibacillus massiliensis	GCF_900148495.1	465	465_6	F68U_bin.74.fa	F68U_bin.74.fa	74.46	2.57	595
F6MX_bin.116	d__Bacteria	p__Firmicutes	c__Clostridia	o__Oscillospirales	f__Ruminococcaceae	g__Negativibacillus	s__Negativibacillus massiliensis	GCF_900148495.1	465	465_7	F6MX_bin.116.fa	F6MX_bin.116.fa	92.46	1.34	595
F6MY_bin.57	d__Bacteria	p__Firmicutes	c__Clostridia	o__Oscillospirales	f__Ruminococcaceae	g__Negativibacillus	s__Negativibacillus massiliensis	GCF_900148495.1	465	465_4	F6MY_bin.57.fa	F6MY_bin.57.fa	87.9	2.01	595
F68C_bin.41	d__Bacteria	p__Firmicutes	c__Clostridia	o__Oscillospirales	f__Ruminococcaceae	g__Negativibacillus	s__Negativibacillus sp000435195	GCA_000435195.1	464	464_1	F68C_bin.41.fa	F68C_bin.41.fa	90.2	1.34	595
F68R_bin.115	d__Bacteria	p__Firmicutes	c__Clostridia	o__Oscillospirales	f__Ruminococcaceae	g__Negativibacillus	s__Negativibacillus sp000435195	GCA_000435195.1	464	464_2	F68R_bin.115.fa	F68R_bin.115.fa	77.29	3.47	595
F68C_bin.25	d__Bacteria	p__Firmicutes	c__Clostridia	o__Oscillospirales	f__Ruminococcaceae	g__Ruminiclostridium_E	s__Ruminiclostridium_E siraeum	GCF_000154325.1	182	182_3	F68C_bin.25.fa	F68C_bin.25.fa	97.65	0	595

Table S3. Taxonomic classifiation of the 2,421 detected MAGs

	Domain	Phylum	Class	Order	Family	Genus	Species	RefGenome	primary_cluster	secondary_cluster	repr_genome	genome	CheckM_completeness	CheckM_contamination	
F68M_bin.33	d__Bacteria	p__Firmicutes	c__Clostridia	o__Oscillospirales	f__Ruminococcaceae	g__Ruminiclostridium_E	s__Ruminiclostridium_E siraeum	GCF_000154325.1	182	182_5	F68M_bin.33.fa	F68M_bin.33.fa	98.93	0	595
F68T_bin.51	d__Bacteria	p__Firmicutes	c__Clostridia	o__Oscillospirales	f__Ruminococcaceae	g__Ruminiclostridium_E	s__Ruminiclostridium_E siraeum	GCF_000154325.1	182	182_1	F68T_bin.51.fa	F68T_bin.51.fa	97.65	0	595
F6D8_bin.27	d__Bacteria	p__Firmicutes	c__Clostridia	o__Oscillospirales	f__Ruminococcaceae	g__Ruminiclostridium_E	s__Ruminiclostridium_E siraeum	GCF_000154325.1	182	182_1	F68T_bin.51.fa	F6D8_bin.27.fa	96.98	0	595
F68N_bin.31	d__Bacteria	p__Firmicutes	c__Clostridia	o__Oscillospirales	f__Ruminococcaceae	g__Ruminiclostridium_E	s__Ruminiclostridium_E siraeum	GCF_000154325.1	182	182_2	F68U_bin.96.fa	F68N_bin.31.fa	93.83	0.43	595
F68U_bin.96	d__Bacteria	p__Firmicutes	c__Clostridia	o__Oscillospirales	f__Ruminococcaceae	g__Ruminiclostridium_E	s__Ruminiclostridium_E siraeum	GCF_000154325.1	182	182_2	F68U_bin.96.fa	F68U_bin.96.fa	98.99	0	595
F68W_bin.57	d__Bacteria	p__Firmicutes	c__Clostridia	o__Oscillospirales	f__Ruminococcaceae	g__Ruminiclostridium_E	s__Ruminiclostridium_E siraeum	GCF_000154325.1	182	182_2	F68U_bin.96.fa	F68W_bin.57.fa	98.99	0	595

Table S3. Taxonomic classifiation of the 2,421 detected MAGs

	Domain	Phylum	Class	Order	Family	Genus	Species	RefGenome	primary_cluster	secondary_cluster	repr_genome	genome	CheckM_completeness	CheckM_contamination	
F6DH_bin.64	d__Bacteria	p__Firmicutes	c__Clostridia	o__Oscillospirales	f__Ruminococcaceae	g__Ruminiclostridium_E	s__Ruminiclostridium_E siraeum	GCF_000154325.1	182	182_2	F68U_bin.96.fa	F6DH_bin.64.fa	98.83	0	595
F6D9_bin.54	d__Bacteria	p__Firmicutes	c__Clostridia	o__Oscillospirales	f__Ruminococcaceae	g__Ruminiclostridium_E	s__Ruminiclostridium_E siraeum	GCF_000154325.1	182	182_4	F6D9_bin.54.fa	F6D9_bin.54.fa	93.96	0	595
F68P_bin.61	d__Bacteria	p__Firmicutes	c__Clostridia	o__Oscillospirales	f__Ruminococcaceae	g__Ruminococcus	s__	N/A	374	374_0	F68P_bin.61.fa	F68P_bin.61.fa	88.79	0.73	596
F68R_bin.80	d__Bacteria	p__Firmicutes	c__Clostridia	o__Oscillospirales	f__Ruminococcaceae	g__Ruminococcus	s__	N/A	493	493_1	F6D8_bin.26.fa	F68R_bin.80.fa	92.6	0	597
F6D8_bin.26	d__Bacteria	p__Firmicutes	c__Clostridia	o__Oscillospirales	f__Ruminococcaceae	g__Ruminococcus	s__	N/A	493	493_1	F6D8_bin.26.fa	F6D8_bin.26.fa	98.64	0	598
F68G_bin.13	d__Bacteria	p__Firmicutes	c__Clostridia	o__Oscillospirales	f__Ruminococcaceae	g__Ruminococcus	s__Ruminococcus sp002438605	GCA_002438605.1	487	487_0	F68G_bin.13.fa	F68G_bin.13.fa	97.99	0	598
F6D8_bin.38	d__Bacteria	p__Firmicutes	c__Clostridia	o__Oscillospirales	f__Ruminococcaceae	g__Ruminococcus_C	s__	N/A	483	483_6	F6D8_bin.38.fa	F6D8_bin.38.fa	96.64	0.73	599

Table S3. Taxonomic classifiation of the 2,421 detected MAGs

	Domain	Phylum	Class	Order	Family	Genus	Species	RefGenome	primary_cluster	secondary_cluster	repr_genome	genome	CheckM_completeness	CheckM_contamination	
F68F_bin.43	d__Bacteria	p__Firmicutes	c__Clostridia	o__Oscillospirales	f__Ruminococcaceae	g__Ruminococcus_C	s__Ruminococcus_C callidus	GCF_000468015.1	485	485_3	F68F_bin.43.fa	F68F_bin.43.fa	97.99	0	599
F68G_bin.68	d__Bacteria	p__Firmicutes	c__Clostridia	o__Oscillospirales	f__Ruminococcaceae	g__Ruminococcus_C	s__Ruminococcus_C callidus	GCF_000468015.1	485	485_8	F68G_bin.68.fa	F68G_bin.68.fa	93.85	0.06	599
F68J_bin.53	d__Bacteria	p__Firmicutes	c__Clostridia	o__Oscillospirales	f__Ruminococcaceae	g__Ruminococcus_C	s__Ruminococcus_C callidus	GCF_000468015.1	485	485_9	F68J_bin.53.fa	F68J_bin.53.fa	97.99	0	599
F68K_bin.81	d__Bacteria	p__Firmicutes	c__Clostridia	o__Oscillospirales	f__Ruminococcaceae	g__Ruminococcus_C	s__Ruminococcus_C callidus	GCF_000468015.1	485	485_6	F68K_bin.81.fa	F68K_bin.81.fa	53.68	0	599
F68N_bin.79	d__Bacteria	p__Firmicutes	c__Clostridia	o__Oscillospirales	f__Ruminococcaceae	g__Ruminococcus_C	s__Ruminococcus_C callidus	GCF_000468015.1	485	485_4	F68N_bin.79.fa	F68N_bin.79.fa	97.99	0	599
F6DE_bin.57	d__Bacteria	p__Firmicutes	c__Clostridia	o__Oscillospirales	f__Ruminococcaceae	g__Ruminococcus_C	s__Ruminococcus_C callidus	GCF_000468015.1	485	485_4	F68N_bin.79.fa	F6DE_bin.57.fa	97.32	0	599

Table S3. Taxonomic classifiation of the 2,421 detected MAGs

	Domain	Phylum	Class	Order	Family	Genus	Species	RefGenome	primary_cluster	secondary_cluster	repr_genome	genome	CheckM_completeness	CheckM_contamination	
F68U_bin.99	d__Bacteria	p__Firmicutes	c__Clostridia	o__Oscillospirales	f__Ruminococcaceae	g__Ruminococcus_C	s__Ruminococcus_C callidus	GCF_000468015.1	485	485_1	F68U_bin.99.fa	F68U_bin.99.fa	73.1	0.4	599
F68W_bin.89	d__Bacteria	p__Firmicutes	c__Clostridia	o__Oscillospirales	f__Ruminococcaceae	g__Ruminococcus_C	s__Ruminococcus_C callidus	GCF_000468015.1	485	485_7	F68W_bin.89.fa	F68W_bin.89.fa	73.82	2.01	599
F6D9_bin.47	d__Bacteria	p__Firmicutes	c__Clostridia	o__Oscillospirales	f__Ruminococcaceae	g__Ruminococcus_C	s__Ruminococcus_C callidus	GCF_000468015.1	485	485_10	F6D9_bin.47.fa	F6D9_bin.47.fa	69.51	0.43	599
F6DA_bin.88	d__Bacteria	p__Firmicutes	c__Clostridia	o__Oscillospirales	f__Ruminococcaceae	g__Ruminococcus_C	s__Ruminococcus_C callidus	GCF_000468015.1	485	485_5	F6DA_bin.88.fa	F6DA_bin.88.fa	97.32	0	599
F6MX_bin.47	d__Bacteria	p__Firmicutes	c__Clostridia	o__Oscillospirales	f__Ruminococcaceae	g__Ruminococcus_C	s__Ruminococcus_C callidus	GCF_000468015.1	485	485_2	F6MX_bin.47.fa	F6MX_bin.47.fa	87.7	4.87	599
F68K_bin.95	d__Bacteria	p__Firmicutes	c__Clostridia	o__Oscillospirales	f__Ruminococcaceae	g__Ruminococcus_C	s__Ruminococcus_C sp000433635	GCA_000433635.1	483	483_4	F68U_bin.68.fa	F68K_bin.95.fa	97.99	0	599

Table S3. Taxonomic classifiation of the 2,421 detected MAGs

	Domain	Phylum	Class	Order	Family	Genus	Species	RefGenome	primary_cluster	secondary_cluster	repr_genome	genome	CheckM_completeness	CheckM_contamination	
F68U_bin.68	d__Bacteria	p__Firmicutes	c__Clostridia	o__Oscillospirales	f__Ruminococcaceae	g__Ruminococcus_C	s__Ruminococcus_C sp000433635	GCA_000433635.1	483	483_4	F68U_bin.68.fa	F68U_bin.68.fa	97.99	0	599
F6D7_bin.54	d__Bacteria	p__Firmicutes	c__Clostridia	o__Oscillospirales	f__Ruminococcaceae	g__Ruminococcus_C	s__Ruminococcus_C sp000433635	GCA_000433635.1	483	483_5	F6D7_bin.54.fa	F6D7_bin.54.fa	97.99	0.13	599
F6D9_bin.68	d__Bacteria	p__Firmicutes	c__Clostridia	o__Oscillospirales	f__Ruminococcaceae	g__Ruminococcus_C	s__Ruminococcus_C sp000433635	GCA_000433635.1	483	483_1	F6DA_bin.95.fa	F6D9_bin.68.fa	94.23	2.15	599
F6DA_bin.95	d__Bacteria	p__Firmicutes	c__Clostridia	o__Oscillospirales	f__Ruminococcaceae	g__Ruminococcus_C	s__Ruminococcus_C sp000433635	GCA_000433635.1	483	483_1	F6DA_bin.95.fa	F6DA_bin.95.fa	97.99	0	599
F6DB_bin.51	d__Bacteria	p__Firmicutes	c__Clostridia	o__Oscillospirales	f__Ruminococcaceae	g__Ruminococcus_C	s__Ruminococcus_C sp000433635	GCA_000433635.1	483	483_2	F6DB_bin.51.fa	F6DB_bin.51.fa	97.32	0	599
F6MY_bin.125	d__Bacteria	p__Firmicutes	c__Clostridia	o__Oscillospirales	f__Ruminococcaceae	g__Ruminococcus_C	s__Ruminococcus_C sp000433635	GCA_000433635.1	483	483_3	F6MY_bin.125.fa	F6MY_bin.125.fa	96.64	0	599

Table S3. Taxonomic classifiation of the 2,421 detected MAGs

	Domain	Phylum	Class	Order	Family	Genus	Species	RefGenome	primary_cluster	secondary_cluster	repr_genome	genome	CheckM_completeness	CheckM_contamination	
F68C_bin.87	d__Bacteria	p__Firmicutes	c__Clostridia	o__Oscillospirales	f__Ruminococcaceae	g__Ruminococcus_C	s__Ruminococcus_C sp000437175	GCA_000437175.1	486	486_0	F68C_bin.87.fa	F68C_bin.87.fa	82.89	0.67	599
F6DG_bin.17	d__Bacteria	p__Firmicutes	c__Clostridia	o__Oscillospirales	f__Ruminococcaceae	g__Ruminococcus_C	s__Ruminococcus_C sp000437255	GCA_000437255.1	488	488_0	F6DG_bin.17.fa	F6DG_bin.17.fa	85.23	0	599
F68H_bin.67	d__Bacteria	p__Firmicutes	c__Clostridia	o__Oscillospirales	f__Ruminococcaceae	g__Ruminococcus_C	s__Ruminococcus_C sp000980705	GCA_000980705.1	484	484_1	F68P_bin.18.fa	F68H_bin.67.fa	90.6	0.67	599
F68P_bin.18	d__Bacteria	p__Firmicutes	c__Clostridia	o__Oscillospirales	f__Ruminococcaceae	g__Ruminococcus_C	s__Ruminococcus_C sp000980705	GCA_000980705.1	484	484_1	F68P_bin.18.fa	F68P_bin.18.fa	92.62	0	599
F68R_bin.46	d__Bacteria	p__Firmicutes	c__Clostridia	o__Oscillospirales	f__Ruminococcaceae	g__Ruminococcus_C	s__Ruminococcus_C sp000980705	GCA_000980705.1	484	484_1	F68P_bin.18.fa	F68R_bin.46.fa	54.36	0	599
F68V_bin.124	d__Bacteria	p__Firmicutes	c__Clostridia	o__Oscillospirales	f__Ruminococcaceae	g__Ruminococcus_C	s__Ruminococcus_C sp000980705	GCA_000980705.1	484	484_1	F68P_bin.18.fa	F68V_bin.124.fa	51.23	0	599

Table S3. Taxonomic classifiation of the 2,421 detected MAGs

	Domain	Phylum	Class	Order	Family	Genus	Species	RefGenome	primary_cluster	secondary_cluster	repr_genome	genome	CheckM_completeness	CheckM_contamination	
F68F_bin.109	d__Bacteria	p__Firmicutes	c__Clostridia	o__Oscillospirales	f__Ruminococcaceae	g__Ruminococcus_D	s__Ruminococcus_D bicirculans	GCF_000723465.1	495	495_1	F68F_bin.109.fa	F68F_bin.109.fa	65.75	0	599
F68H_bin.52	d__Bacteria	p__Firmicutes	c__Clostridia	o__Oscillospirales	f__Ruminococcaceae	g__Ruminococcus_D	s__Ruminococcus_D bicirculans	GCF_000723465.1	495	495_6	F68H_bin.52.fa	F68H_bin.52.fa	95.48	0.8	599
F68N_bin.71	d__Bacteria	p__Firmicutes	c__Clostridia	o__Oscillospirales	f__Ruminococcaceae	g__Ruminococcus_D	s__Ruminococcus_D bicirculans	GCF_000723465.1	495	495_9	F68N_bin.71.fa	F68N_bin.71.fa	55.32	0.58	599
F68R_bin.39	d__Bacteria	p__Firmicutes	c__Clostridia	o__Oscillospirales	f__Ruminococcaceae	g__Ruminococcus_D	s__Ruminococcus_D bicirculans	GCF_000723465.1	495	495_2	F68R_bin.39.fa	F68R_bin.39.fa	89.04	0	599
F6D7_bin.56	d__Bacteria	p__Firmicutes	c__Clostridia	o__Oscillospirales	f__Ruminococcaceae	g__Ruminococcus_D	s__Ruminococcus_D bicirculans	GCF_000723465.1	495	495_7	F6D7_bin.56.fa	F6D7_bin.56.fa	96.58	0	599
F6D8_bin.24	d__Bacteria	p__Firmicutes	c__Clostridia	o__Oscillospirales	f__Ruminococcaceae	g__Ruminococcus_D	s__Ruminococcus_D bicirculans	GCF_000723465.1	495	495_3	F6D8_bin.24.fa	F6D8_bin.24.fa	77.84	2.4	599

Table S3. Taxonomic classifiation of the 2,421 detected MAGs

	Domain	Phylum	Class	Order	Family	Genus	Species	RefGenome	primary_cluster	secondary_cluster	repr_genome	genome	CheckM_completeness	CheckM_contamination	
F6DB_bin.47	d__Bacteria	p__Firmicutes	c__Clostridia	o__Oscillospirales	f__Ruminococcaceae	g__Ruminococcus_D	s__Ruminococcus_D bicirculans	GCF_000723465.1	495	495_4	F6DB_bin.47.fa	F6DB_bin.47.fa	98.63	0	599
F6DE_bin.6	d__Bacteria	p__Firmicutes	c__Clostridia	o__Oscillospirales	f__Ruminococcaceae	g__Ruminococcus_D	s__Ruminococcus_D bicirculans	GCF_000723465.1	495	495_5	F6DE_bin.6.fa	F6DE_bin.6.fa	98.63	0.68	599
F6MY_bin.124	d__Bacteria	p__Firmicutes	c__Clostridia	o__Oscillospirales	f__Ruminococcaceae	g__Ruminococcus_D	s__Ruminococcus_D bicirculans	GCF_000723465.1	495	495_8	F6MY_bin.124.fa	F6MY_bin.124.fa	95.89	0	599
F68D_bin.2	d__Bacteria	p__Firmicutes	c__Clostridia	o__Oscillospirales	f__Ruminococcaceae	g__Ruthenibacterium	s__Ruthenibacterium lactatiformans	GCF_000949455.1	283	283_1	F6DE_bin.82.fa	F68D_bin.2.fa	93.84	0.74	599
F68G_bin.79	d__Bacteria	p__Firmicutes	c__Clostridia	o__Oscillospirales	f__Ruminococcaceae	g__Ruthenibacterium	s__Ruthenibacterium lactatiformans	GCF_000949455.1	283	283_1	F6DE_bin.82.fa	F68G_bin.79.fa	89.28	2.78	599
F68K_bin.64	d__Bacteria	p__Firmicutes	c__Clostridia	o__Oscillospirales	f__Ruminococcaceae	g__Ruthenibacterium	s__Ruthenibacterium lactatiformans	GCF_000949455.1	283	283_1	F6DE_bin.82.fa	F68K_bin.64.fa	85.47	0.45	599

Table S3. Taxonomic classifiation of the 2,421 detected MAGs

	Domain	Phylum	Class	Order	Family	Genus	Species	RefGenome	primary_cluster	secondary_cluster	repr_genome	genome	CheckM_completeness	CheckM_contamination	
F68M_bin.62	d__Bacteria	p__Firmicutes	c__Clostridia	o__Oscillospirales	f__Ruminococcaceae	g__Ruthenibacterium	s__Ruthenibacterium lactatiformans	GCF_000949455.1	283	283_1	F6DE_bin.82.fa	F68M_bin.62.fa	91.84	0	599
F68T_bin.96	d__Bacteria	p__Firmicutes	c__Clostridia	o__Oscillospirales	f__Ruminococcaceae	g__Ruthenibacterium	s__Ruthenibacterium lactatiformans	GCF_000949455.1	283	283_1	F6DE_bin.82.fa	F68T_bin.96.fa	82.97	1.52	599
F68V_bin.4	d__Bacteria	p__Firmicutes	c__Clostridia	o__Oscillospirales	f__Ruminococcaceae	g__Ruthenibacterium	s__Ruthenibacterium lactatiformans	GCF_000949455.1	283	283_1	F6DE_bin.82.fa	F68V_bin.4.fa	92.18	0.68	599
F6DB_bin.39	d__Bacteria	p__Firmicutes	c__Clostridia	o__Oscillospirales	f__Ruminococcaceae	g__Ruthenibacterium	s__Ruthenibacterium lactatiformans	GCF_000949455.1	283	283_1	F6DE_bin.82.fa	F6DB_bin.39.fa	87.84	0.79	599
F6DE_bin.82	d__Bacteria	p__Firmicutes	c__Clostridia	o__Oscillospirales	f__Ruminococcaceae	g__Ruthenibacterium	s__Ruthenibacterium lactatiformans	GCF_000949455.1	283	283_1	F6DE_bin.82.fa	F6DE_bin.82.fa	96.71	0.57	599
F68T_bin.85	d__Bacteria	p__Firmicutes	c__Clostridia	o__Oscillospirales	f__Ruminococcaceae	g__UBA1394	s__	N/A	139	139_0	F68T_bin.85.fa	F68T_bin.85.fa	94.41	0	600
F6DG_bin.97	d__Bacteria	p__Firmicutes	c__Clostridia	o__Oscillospirales	f__Ruminococcaceae	g__UBA866	s__	N/A	180	180_1	F6MX_bin.68.fa	F6DG_bin.97.fa	84.11	2.57	601

Table S3. Taxonomic classifiation of the 2,421 detected MAGs

	Domain	Phylum	Class	Order	Family	Genus	Species	RefGenome	primary_cluster	secondary_cluster	repr_genome	genome	CheckM_completeness	CheckM_contamination	
F6MX_bin.68	d__Bacteria	p__Firmicutes	c__Clostridia	o__Oscillospirales	f__Ruminococcaceae	g__UBA866	s__	N/A	180	180_1	F6MX_bin.68.fa	F6MX_bin.68.fa	96.42	0.22	602
F6MY_bin.68	d__Bacteria	p__Firmicutes	c__Clostridia	o__Oscillospirales	f__Ruminococcaceae	g__UBA866	s__	N/A	180	180_2	F6MY_bin.68.fa	F6MY_bin.68.fa	79.75	2.13	603
F68V_bin.121	d__Bacteria	p__Firmicutes	c__Clostridia	o__Oscillospirales	f__UBA644	g__	s__	N/A	80	80_0	F68V_bin.121.fa	F68V_bin.121.fa	55.59	1.23	604
F68V_bin.48	d__Bacteria	p__Firmicutes	c__Clostridia	o__Oscillospirales	f__UBA644	g__UBA644	s__UBA644 sp002299265	GCA_002299265.1	574	574_0	F68V_bin.48.fa	F68V_bin.48.fa	80.31	1.01	604
F68G_bin.7	d__Bacteria	p__Firmicutes	c__Clostridia	o__Peptostreptococcales	f__Anaerovoracaceae	g__	s__	N/A	123	123_1	F68G_bin.7.fa	F68G_bin.7.fa	96.45	0.35	605
F68H_bin.77	d__Bacteria	p__Firmicutes	c__Clostridia	o__Peptostreptococcales	f__Anaerovoracaceae	g__	s__	N/A	123	123_2	F68H_bin.77.fa	F68H_bin.77.fa	67.99	1.42	606
F68V_bin.55	d__Bacteria	p__Firmicutes	c__Clostridia	o__Peptostreptococcales	f__Anaerovoracaceae	g__	s__	N/A	123	123_3	F68V_bin.55.fa	F68V_bin.55.fa	81.81	4.26	607
F68F_bin.4	d__Bacteria	p__Firmicutes	c__Clostridia	o__Peptostreptococcales	f__Anaerovoracaceae	g__	s__	N/A	127	127_1	F68F_bin.4.fa	F68F_bin.4.fa	98.23	0	608

Table S3. Taxonomic classifiation of the 2,421 detected MAGs

	Domain	Phylum	Class	Order	Family	Genus	Species	RefGenome	primary_cluster	secondary_cluster	repr_genome	genome	CheckM_completeness	CheckM_contamination	
F68K_bin.16	d__Bacteria	p__Firmicutes	c__Clostridia	o__Peptostreptococcales	f__Anaerovoracaceae	g__	s__	N/A	127	127_1	F68F_bin.4.fa	F68K_bin.16.fa	88.5	0.71	609
F68N_bin.18	d__Bacteria	p__Firmicutes	c__Clostridia	o__Peptostreptococcales	f__Anaerovoracaceae	g__	s__	N/A	127	127_1	F68F_bin.4.fa	F68N_bin.18.fa	90.17	1.65	610
F6DB_bin.77	d__Bacteria	p__Firmicutes	c__Clostridia	o__Peptostreptococcales	f__Anaerovoracaceae	g__	s__	N/A	127	127_1	F68F_bin.4.fa	F6DB_bin.77.fa	88.25	0.24	611
F6MX_bin.75	d__Bacteria	p__Firmicutes	c__Clostridia	o__Peptostreptococcales	f__Anaerovoracaceae	g__	s__	N/A	271	271_0	F6MX_bin.75.fa	F6MX_bin.75.fa	50.52	0	612
F68U_bin.91	d__Bacteria	p__Firmicutes	c__Clostridia	o__Peptostreptococcales	f__Anaerovoracaceae	g__	s__	N/A	573	573_0	F68U_bin.91.fa	F68U_bin.91.fa	92.13	0.71	613
F6MX_bin.97	d__Bacteria	p__Firmicutes	c__Clostridia	o__Peptostreptococcales	f__Anaerovoracaceae	g__CAG-145	s__	N/A	189	189_0	F6MX_bin.97.fa	F6MX_bin.97.fa	73.08	0.71	614
F68V_bin.80	d__Bacteria	p__Firmicutes	c__Clostridia	o__Peptostreptococcales	f__Anaerovoracaceae	g__CAG-145	s__	N/A	190	190_0	F68V_bin.80.fa	F68V_bin.80.fa	71.26	0.53	615

Table S3. Taxonomic classifiation of the 2,421 detected MAGs

	Domain	Phylum	Class	Order	Family	Genus	Species	RefGenome	primary_cluster	secondary_cluster	repr_genome	genome	CheckM_completeness	CheckM_contamination	
F68D_bin.94	d__Bacteria	p__Firmicutes	c__Clostridia	o__Peptostreptococcales	f__Anaerovoracaceae	g__CAG-145	s__	N/A	456	456_3	F68D_bin.94.fa	F68D_bin.94.fa	95.39	0.35	616
F68K_bin.105	d__Bacteria	p__Firmicutes	c__Clostridia	o__Peptostreptococcales	f__Anaerovoracaceae	g__CAG-145	s__	N/A	456	456_7	F68K_bin.105.fa	F68K_bin.105.fa	94.86	1.06	617
F6DA_bin.60	d__Bacteria	p__Firmicutes	c__Clostridia	o__Peptostreptococcales	f__Anaerovoracaceae	g__CAG-145	s__	N/A	456	456_5	F6DA_bin.60.fa	F6DA_bin.60.fa	92.2	0.02	618
F6DG_bin.57	d__Bacteria	p__Firmicutes	c__Clostridia	o__Peptostreptococcales	f__Anaerovoracaceae	g__CAG-145	s__	N/A	456	456_6	F6DG_bin.57.fa	F6DG_bin.57.fa	89.87	0	619
F68W_bin.106	d__Bacteria	p__Firmicutes	c__Clostridia	o__Peptostreptococcales	f__Anaerovoracaceae	g__CAG-145	s__	N/A	456	456_4	F6DH_bin.111.fa	F68W_bin.106.fa	88.59	2.72	620
F6DH_bin.111	d__Bacteria	p__Firmicutes	c__Clostridia	o__Peptostreptococcales	f__Anaerovoracaceae	g__CAG-145	s__	N/A	456	456_4	F6DH_bin.111.fa	F6DH_bin.111.fa	83.08	0	621
F6DH_bin.65	d__Bacteria	p__Firmicutes	c__Clostridia	o__Peptostreptococcales	f__Anaerovoracaceae	g__CAG-145	s__	N/A	456	456_1	F6DH_bin.65.fa	F6DH_bin.65.fa	73	0	622

Table S3. Taxonomic classifiation of the 2,421 detected MAGs

	Domain	Phylum	Class	Order	Family	Genus	Species	RefGenome	primary_cluster	secondary_cluster	repr_genome	genome	CheckM_completeness	CheckM_contamination	
F6MX_bin.108	d__Bacteria	p__Firmicutes	c__Clostridia	o__Peptostreptococcales	f__Anaerovoracaceae	g__CAG-145	s__	N/A	456	456_2	F6MX_bin.108.fa	F6MX_bin.108.fa	93.68	2.84	623
F6DB_bin.68	d__Bacteria	p__Firmicutes	c__Clostridia	o__Peptostreptococcales	f__Anaerovoracaceae	g__CAG-145	s__	N/A	457	457_0	F6DB_bin.68.fa	F6DB_bin.68.fa	97.52	0	624
F68R_bin.100	d__Bacteria	p__Firmicutes	c__Clostridia	o__Peptostreptococcales	f__Anaerovoracaceae	g__CAG-145	s__	N/A	458	458_1	F68R_bin.100.fa	F68R_bin.100.fa	53.62	4.96	625
F68T_bin.83	d__Bacteria	p__Firmicutes	c__Clostridia	o__Peptostreptococcales	f__Anaerovoracaceae	g__CAG-145	s__	N/A	458	458_2	F68T_bin.83.fa	F68T_bin.83.fa	73.32	0.47	626
F68K_bin.69	d__Bacteria	p__Firmicutes	c__Clostridia	o__Peptostreptococcales	f__Anaerovoracaceae	g__CAG-145	s__CAG-145 sp000435715	GCA_000435715.1	188	188_1	F68K_bin.69.fa	F68K_bin.69.fa	97.16	0	626
F6DE_bin.65	d__Bacteria	p__Firmicutes	c__Clostridia	o__Peptostreptococcales	f__Anaerovoracaceae	g__CAG-145	s__CAG-145 sp000435715	GCA_000435715.1	188	188_1	F68K_bin.69.fa	F6DE_bin.65.fa	93.36	2.33	626
F6DB_bin.9	d__Bacteria	p__Firmicutes	c__Clostridia	o__Peptostreptococcales	f__Anaerovoracaceae	g__CAG-145	s__CAG-145 sp000435715	GCA_000435715.1	188	188_2	F6DB_bin.9.fa	F6DB_bin.9.fa	94.9	0	626

Table S3. Taxonomic classifiation of the 2,421 detected MAGs

	Domain	Phylum	Class	Order	Family	Genus	Species	RefGenome	primary_cluster	secondary_cluster	repr_genome	genome	CheckM_completeness	CheckM_contamination	
F6DG_bin.58	d__Bacteria	p__Firmicutes	c__Clostridia	o__Peptostreptococcales	f__Anaerovoracaceae	g__CAG-145	s__CAG-145 sp000435715	GCA_000435715.1	188	188_3	F6DG_bin.58.fa	F6DG_bin.58.fa	56.47	0.78	626
F68M_bin.70	d__Bacteria	p__Firmicutes	c__Clostridia	o__Peptostreptococcales	f__Anaerovoracaceae	g__Eubacterium_A	s__	N/A	569	569_0	F68M_bin.70.fa	F68M_bin.70.fa	98.94	1.06	627
F68N_bin.68	d__Bacteria	p__Firmicutes	c__Clostridia	o__Peptostreptococcales	f__Anaerovoracaceae	g__Eubacterium_A	s__	N/A	570	570_0	F68N_bin.68.fa	F68N_bin.68.fa	95.17	1.7	628
F68K_bin.66	d__Bacteria	p__Firmicutes	c__Clostridia	o__Peptostreptococcales	f__Anaerovoracaceae	g__Eubacterium_M	s__	N/A	7	7_0	F68K_bin.66.fa	F68K_bin.66.fa	96.45	0	629
F6DB_bin.56	d__Bacteria	p__Firmicutes	c__Clostridia	o__Peptostreptococcales	f__Anaerovoracaceae	g__Mogibacterium	s__	N/A	661	661_0	F6DB_bin.56.fa	F6DB_bin.56.fa	99.29	0	630
F68D_bin.46	d__Bacteria	p__Firmicutes	c__Clostridia	o__Peptostreptococcales	f__Anaerovoracaceae	g__Mogibacterium	s__Mogibacterium sp002299625	GCA_002299625.1	268	268_1	F68D_bin.46.fa	F68D_bin.46.fa	97.52	0	630
F68G_bin.21	d__Bacteria	p__Firmicutes	c__Clostridia	o__Peptostreptococcales	f__Anaerovoracaceae	g__Mogibacterium	s__Mogibacterium sp002299625	GCA_002299625.1	268	268_1	F68D_bin.46.fa	F68G_bin.21.fa	90.78	0	630

Table S3. Taxonomic classifiation of the 2,421 detected MAGs

	Domain	Phylum	Class	Order	Family	Genus	Species	RefGenome	primary_cluster	secondary_cluster	repr_genome	genome	CheckM_completeness	CheckM_contamination	
F68M_bin.50	d__Bacteria	p__Firmicutes	c__Clostridia	o__Peptostreptococcales	f__Anaerovoracaceae	g__Mogibacterium	s__Mogibacterium sp900315625	GCA_900315625.1	660	660_1	F68M_bin.50.fa	F68M_bin.50.fa	98.58	0.71	630
F6D8_bin.5	d__Bacteria	p__Firmicutes	c__Clostridia	o__Peptostreptococcales	f__Anaerovoracaceae	g__Mogibacterium	s__Mogibacterium sp900315625	GCA_900315625.1	660	660_2	F6D8_bin.5.fa	F6D8_bin.5.fa	95.74	3.9	630
F68F_bin.113	d__Bacteria	p__Firmicutes	c__Clostridia	o__Peptostreptococcales	f__Anaerovoracaceae	g__UBA1191	s__	N/A	380	380_0	F68F_bin.113.fa	F68F_bin.113.fa	54.73	1.06	631
F6D9_bin.24	d__Bacteria	p__Firmicutes	c__Clostridia	o__Peptostreptococcales	f__Anaerovoracaceae	g__UBA1191	s__	N/A	124	124_0	F6D9_bin.24.fa	F6D9_bin.24.fa	89.92	0.18	632
F6DE_bin.72	d__Bacteria	p__Firmicutes	c__Clostridia	o__Peptostreptococcales	f__Anaerovoracaceae	g__UBA1191	s__	N/A	125	125_0	F6DE_bin.72.fa	F6DE_bin.72.fa	91.98	2.48	633
F6DE_bin.81	d__Bacteria	p__Firmicutes	c__Clostridia	o__Peptostreptococcales	f__Anaerovoracaceae	g__UBA1191	s__	N/A	459	459_0	F6DE_bin.81.fa	F6DE_bin.81.fa	82.12	0	634
F6DE_bin.74	d__Bacteria	p__Firmicutes	c__Clostridia	o__Peptostreptococcales	f__Anaerovoracaceae	g__UBA1191	s__UBA1191 sp900066305	GCA_900066305.1	126	126_0	F6DE_bin.74.fa	F6DE_bin.74.fa	93.19	2.13	634

Table S3. Taxonomic classifiation of the 2,421 detected MAGs

	Domain	Phylum	Class	Order	Family	Genus	Species	RefGenome	primary_cluster	secondary_cluster	repr_genome	genome	CheckM_completeness	CheckM_contamination	
F68T_bin.27	d__Bacteria	p__Firmicutes	c__Clostridia	o__Peptostreptococcales	f__Peptostreptococcaceae	g__Intestinibacter	s__Intestinibacter bartlettii	GCF_000154445.1	195	195_1	F68T_bin.27.fa	F68T_bin.27.fa	92.43	2.7	634
F6DG_bin.20	d__Bacteria	p__Firmicutes	c__Clostridia	o__Peptostreptococcales	f__Peptostreptococcaceae	g__Intestinibacter	s__Intestinibacter bartlettii	GCF_000154445.1	195	195_2	F6DG_bin.20.fa	F6DG_bin.20.fa	94.56	3.54	634
F6D9_bin.75	d__Bacteria	p__Firmicutes	c__Clostridia	o__Peptostreptococcales	f__Peptostreptococcaceae	g__Romboutsia	s__Romboutsia timonensis	GCF_900106845.1	196	196_0	F6D9_bin.75.fa	F6D9_bin.75.fa	89.61	4.58	634
F68V_bin.103	d__Bacteria	p__Firmicutes	c__Clostridia	o__TANB77	f__CAG-508	g__CAG-245	s__CAG-245 sp000435175	GCA_000435175.1	220	220_2	F68V_bin.103.fa	F68V_bin.103.fa	79.46	0.67	634
F6DB_bin.101	d__Bacteria	p__Firmicutes	c__Clostridia	o__TANB77	f__CAG-508	g__CAG-245	s__CAG-245 sp000435175	GCA_000435175.1	220	220_3	F6DB_bin.101.fa	F6DB_bin.101.fa	78.79	0.67	634
F6MX_bin.78	d__Bacteria	p__Firmicutes	c__Clostridia	o__TANB77	f__CAG-508	g__CAG-245	s__CAG-245 sp000435175	GCA_000435175.1	220	220_1	F6MX_bin.78.fa	F6MX_bin.78.fa	78.79	1.34	634
F6MY_bin.3	d__Bacteria	p__Firmicutes	c__Clostridia	o__TANB77	f__CAG-508	g__CAG-245	s__CAG-245 sp000435175	GCA_000435175.1	220	220_1	F6MX_bin.78.fa	F6MY_bin.3.fa	73.62	1.86	634

Table S3. Taxonomic classifiation of the 2,421 detected MAGs

	Domain	Phylum	Class	Order	Family	Genus	Species	RefGenome	primary_cluster	secondary_cluster	repr_genome	genome	CheckM_completeness	CheckM_contamination	
F68V_bin.111	d__Bacteria	p__Firmicutes	c__Clostridia	o__TANB77	f__CAG-508	g__CAG-269	s__CAG-269 sp000431335	GCA_000431335.1	226	226_0	F68V_bin.111.fa	F68V_bin.111.fa	73.42	3.36	634
F6DE_bin.71	d__Bacteria	p__Firmicutes	c__Clostridia	o__TANB77	f__CAG-508	g__CAG-269	s__CAG-269 sp000435535	GCA_000435535.1	225	225_0	F6DE_bin.71.fa	F6DE_bin.71.fa	68.68	1.79	634
F68V_bin.32	d__Bacteria	p__Firmicutes	c__Clostridia	o__TANB77	f__CAG-508	g__CAG-269	s__CAG-269 sp000437215	GCA_000437215.1	228	228_0	F68V_bin.32.fa	F68V_bin.32.fa	74.54	3.15	634
F6MY_bin.62	d__Bacteria	p__Firmicutes	c__Clostridia	o__TANB77	f__CAG-508	g__CAG-269	s__CAG-269 sp000438255	GCA_000438255.1	224	224_0	F6MY_bin.62.fa	F6MY_bin.62.fa	80.72	2.21	634
F68T_bin.65	d__Bacteria	p__Firmicutes	c__Clostridia	o__TANB77	f__CAG-508	g__CAG-269	s__CAG-269 sp001916035	GCA_001916035.1	227	227_0	F68T_bin.65.fa	F68T_bin.65.fa	77.12	1.68	634
F6MY_bin.14	d__Bacteria	p__Firmicutes	c__Clostridia	o__TANB77	f__CAG-508	g__CAG-269	s__CAG-269 sp001916065	GCA_001916065.1	223	223_0	F6MY_bin.14.fa	F6MY_bin.14.fa	77.3	2.84	634
F68C_bin.39	d__Bacteria	p__Firmicutes	c__Clostridia	o__TANB77	f__CAG-508	g__CAG-273	s__CAG-273 sp000437855	GCA_000437855.1	219	219_1	F68R_bin.108.fa	F68C_bin.39.fa	78.12	0.89	634

Table S3. Taxonomic classifiation of the 2,421 detected MAGs

	Domain	Phylum	Class	Order	Family	Genus	Species	RefGenome	primary_cluster	secondary_cluster	repr_genome	genome	CheckM_completeness	CheckM_contamination	
F68R_bin.108	d__Bacteria	p__Firmicutes	c__Clostridia	o__TANB77	f__CAG-508	g__CAG-273	s__CAG-273 sp000437855	GCA_000437855.1	219	219_1	F68R_bin.108.fa	F68R_bin.108.fa	78.12	0.89	634
F6MX_bin.118	d__Bacteria	p__Firmicutes	c__Clostridia	o__TANB77	f__CAG-508	g__CAG-273	s__CAG-273 sp000437855	GCA_000437855.1	219	219_1	F68R_bin.108.fa	F6MX_bin.118.fa	78.12	1.57	634
F6MX_bin.31	d__Bacteria	p__Firmicutes	c__Clostridia	o__TANB77	f__CAG-508	g__CAG-273	s__CAG-273 sp000438355	GCA_000438355.1	197	197_0	F6MX_bin.31.fa	F6MX_bin.31.fa	74.71	0.67	634
F68R_bin.40	d__Bacteria	p__Firmicutes	c__Clostridia	o__TANB77	f__CAG-508	g__CAG-452	s__CAG-452 sp000434035	GCA_000434035.1	218	218_1	F6MX_bin.66.fa	F68R_bin.40.fa	79.69	0.22	634
F68V_bin.110	d__Bacteria	p__Firmicutes	c__Clostridia	o__TANB77	f__CAG-508	g__CAG-452	s__CAG-452 sp000434035	GCA_000434035.1	218	218_1	F6MX_bin.66.fa	F68V_bin.110.fa	79.02	2.91	634
F6D8_bin.60	d__Bacteria	p__Firmicutes	c__Clostridia	o__TANB77	f__CAG-508	g__CAG-452	s__CAG-452 sp000434035	GCA_000434035.1	218	218_1	F6MX_bin.66.fa	F6D8_bin.60.fa	79.69	0.22	634
F6MX_bin.66	d__Bacteria	p__Firmicutes	c__Clostridia	o__TANB77	f__CAG-508	g__CAG-452	s__CAG-452 sp000434035	GCA_000434035.1	218	218_1	F6MX_bin.66.fa	F6MX_bin.66.fa	80.36	0.22	634

Table S3. Taxonomic classifiation of the 2,421 detected MAGs

	Domain	Phylum	Class	Order	Family	Genus	Species	RefGenome	primary_cluster	secondary_cluster	repr_genome	genome	CheckM_completeness	CheckM_contamination	
F68C_bin.107	d__Bacteria	p__Firmicutes	c__Clostridia	o__TANB77	f__CAG-508	g__CAG-492	s__CAG-492 sp000434015	GCA_000434015.1	221	221_1	F68C_bin.107.fa	F68C_bin.107.fa	78.62	1.9	634
F6D8_bin.83	d__Bacteria	p__Firmicutes	c__Clostridia	o__TANB77	f__CAG-508	g__CAG-492	s__CAG-492 sp000434015	GCA_000434015.1	221	221_1	F68C_bin.107.fa	F6D8_bin.83.fa	78.62	3.24	634
F6MX_bin.64	d__Bacteria	p__Firmicutes	c__Clostridia	o__TANB77	f__CAG-508	g__CAG-492	s__CAG-492 sp000434015	GCA_000434015.1	221	221_1	F68C_bin.107.fa	F6MX_bin.64.fa	78.62	1.9	634
F68U_bin.67	d__Bacteria	p__Firmicutes	c__Clostridia	o__TANB77	f__CAG-508	g__CAG-492	s__CAG-492 sp000434335	GCA_000434335.1	222	222_1	F68U_bin.67.fa	F68U_bin.67.fa	80.02	1.01	634
F6D7_bin.105	d__Bacteria	p__Firmicutes	c__Clostridia	o__TANB77	f__CAG-508	g__CAG-492	s__CAG-492 sp000434335	GCA_000434335.1	222	222_2	F6D7_bin.105.fa	F6D7_bin.105.fa	80.02	2.01	634
F68V_bin.65	d__Bacteria	p__Firmicutes	c__Clostridia	o__UBA1212	f__UBA1255	g__	s__	N/A	178	178_0	F68V_bin.65.fa	F68V_bin.65.fa	94.58	1.46	635
F68V_bin.91	d__Bacteria	p__Firmicutes	c__Dehalobacteriia	o__UBA7702	f__UBA7702	g__	s__	N/A	462	462_0	F68V_bin.91.fa	F68V_bin.91.fa	88.44	1.63	636
F68D_bin.84	d__Bacteria	p__Firmicutes	c__Peptococcia	o__Peptococcales	f__Peptococcaceae	g__	s__	N/A	279	279_1	F68D_bin.84.fa	F68D_bin.84.fa	67.9	1.72	637
F68K_bin.23	d__Bacteria	p__Firmicutes	c__Peptococcia	o__Peptococcales	f__Peptococcaceae	g__	s__	N/A	279	279_3	F68K_bin.23.fa	F68K_bin.23.fa	62.07	0	638

Table S3. Taxonomic classifiation of the 2,421 detected MAGs

	Domain	Phylum	Class	Order	Family	Genus	Species	RefGenome	primary_cluster	secondary_cluster	repr_genome	genome	CheckM_completeness	CheckM_contamination	
F6MX_bin.43	d__Bacteria	p__Firmicutes	c__Peptococcia	o__Peptococcales	f__Peptococcaceae	g__	s__	N/A	279	279_2	F6MX_bin.43.fa	F6MX_bin.43.fa	67.65	1.11	639
F68F_bin.19	d__Bacteria	p__Firmicutes	c__Negativicutes	o__Acidaminococcales	f__Acidaminococcaceae	g__	s__	N/A	538	538_1	F68F_bin.19.fa	F68F_bin.19.fa	97.88	0.3	640
F68K_bin.75	d__Bacteria	p__Firmicutes	c__Negativicutes	o__Acidaminococcales	f__Acidaminococcaceae	g__	s__	N/A	538	538_2	F68K_bin.75.fa	F68K_bin.75.fa	99.48	1.45	641
F68D_bin.105	d__Bacteria	p__Firmicutes	c__Negativicutes	o__Acidaminococcales	f__Acidaminococcaceae	g__Acidaminococcus	s__	N/A	387	387_1	F68D_bin.105.fa	F68D_bin.105.fa	96.11	0	642
F68G_bin.37	d__Bacteria	p__Firmicutes	c__Negativicutes	o__Acidaminococcales	f__Acidaminococcaceae	g__Acidaminococcus	s__	N/A	387	387_2	F68G_bin.37.fa	F68G_bin.37.fa	97.05	1.7	643
F68N_bin.60	d__Bacteria	p__Firmicutes	c__Negativicutes	o__Acidaminococcales	f__Acidaminococcaceae	g__Acidaminococcus	s__	N/A	422	422_0	F68N_bin.60.fa	F68N_bin.60.fa	98.68	0.6	644
F68F_bin.108	d__Bacteria	p__Firmicutes	c__Negativicutes	o__Acidaminococcales	f__Acidaminococcaceae	g__Acidaminococcus	s__Acidaminococcus intestini	GCF_000425045.1	386	386_1	F68F_bin.108.fa	F68F_bin.108.fa	100	0	644
F68H_bin.73	d__Bacteria	p__Firmicutes	c__Negativicutes	o__Acidaminococcales	f__Acidaminococcaceae	g__Acidaminococcus	s__Acidaminococcus intestini	GCF_000425045.1	386	386_1	F68F_bin.108.fa	F68H_bin.73.fa	99.4	0	644

Table S3. Taxonomic classifiation of the 2,421 detected MAGs

	Domain	Phylum	Class	Order	Family	Genus	Species	RefGenome	primary_cluster	secondary_cluster	repr_genome	genome	CheckM_completeness	CheckM_contamination	
F68T_bin.53	d__Bacteria	p__Firmicutes	c__Negativicutes	o__Acidaminococcales	f__Acidaminococcaceae	g__Acidaminococcus	s__Acidaminococcus intestini	GCF_000425045.1	386	386_1	F68F_bin.108.fa	F68T_bin.53.fa	99.4	0	644
F68H_bin.47	d__Bacteria	p__Firmicutes	c__Negativicutes	o__Acidaminococcales	f__Acidaminococcaceae	g__Acidaminococcus	s__Acidaminococcus massiliensis	GCF_900095825.1	388	388_1	F68H_bin.47.fa	F68H_bin.47.fa	98.5	0.62	644
F6DB_bin.15	d__Bacteria	p__Firmicutes	c__Negativicutes	o__Acidaminococcales	f__Acidaminococcaceae	g__Acidaminococcus	s__Acidaminococcus massiliensis	GCF_900095825.1	388	388_1	F68H_bin.47.fa	F6DB_bin.15.fa	91.92	3.13	644
F6D9_bin.35	d__Bacteria	p__Firmicutes	c__Negativicutes	o__Acidaminococcales	f__Acidaminococcaceae	g__Acidaminococcus	s__Acidaminococcus sp000437815	GCA_000437815.1	389	389_0	F6D9_bin.35.fa	F6D9_bin.35.fa	99.4	0.6	644
F68V_bin.107	d__Bacteria	p__Firmicutes	c__Negativicutes	o__Acidaminococcales	f__Acidaminococcaceae	g__Phascolarctobacterium	s__Phascolarctobacterium faecium	GCA_003269275.1	537	537_1	F6DE_bin.34.fa	F68V_bin.107.fa	86.83	1.5	644
F6DE_bin.34	d__Bacteria	p__Firmicutes	c__Negativicutes	o__Acidaminococcales	f__Acidaminococcaceae	g__Phascolarctobacterium	s__Phascolarctobacterium faecium	GCA_003269275.1	537	537_1	F6DE_bin.34.fa	F6DE_bin.34.fa	99.85	2.1	644

Table S3. Taxonomic classifiation of the 2,421 detected MAGs

	Domain	Phylum	Class	Order	Family	Genus	Species	RefGenome	primary_cluster	secondary_cluster	repr_genome	genome	CheckM_completeness	CheckM_contamination	
F68F_bin.52	d__Bacteria	p__Firmicutes	c__Negativicutes	o__Acidaminococcales	f__Acidaminococcaceae	g__Phascolarctobacterium_A	s__	N/A	541	541_0	F68F_bin.52.fa	F68F_bin.52.fa	97.01	0.8	645
F68M_bin.40	d__Bacteria	p__Firmicutes	c__Negativicutes	o__Acidaminococcales	f__Acidaminococcaceae	g__Phascolarctobacterium_A	s__	N/A	539	539_8	F68M_bin.40.fa	F68M_bin.40.fa	94.77	2.22	646
F68R_bin.41	d__Bacteria	p__Firmicutes	c__Negativicutes	o__Acidaminococcales	f__Acidaminococcaceae	g__Phascolarctobacterium_A	s__	N/A	539	539_7	F68R_bin.41.fa	F68R_bin.41.fa	84.27	1.6	647
F68T_bin.40	d__Bacteria	p__Firmicutes	c__Negativicutes	o__Acidaminococcales	f__Acidaminococcaceae	g__Phascolarctobacterium_A	s__	N/A	539	539_6	F68T_bin.40.fa	F68T_bin.40.fa	97.59	0.9	648
F6DA_bin.22	d__Bacteria	p__Firmicutes	c__Negativicutes	o__Acidaminococcales	f__Acidaminococcaceae	g__Phascolarctobacterium_A	s__	N/A	540	540_0	F6DA_bin.22.fa	F6DA_bin.22.fa	97.6	0.6	649
F68E_bin.38	d__Bacteria	p__Firmicutes	c__Negativicutes	o__Acidaminococcales	f__Acidaminococcaceae	g__Phascolarctobacterium_A	s__Phascolarctobacterium_A succinatutens	GCF_000188175.1	539	539_9	F68E_bin.38.fa	F68E_bin.38.fa	98.2	0.9	649

Table S3. Taxonomic classifiation of the 2,421 detected MAGs

	Domain	Phylum	Class	Order	Family	Genus	Species	RefGenome	primary_cluster	secondary_cluster	repr_genome	genome	CheckM_completeness	CheckM_contamination	
F68J_bin.44	d__Bacteria	p__Firmicutes	c__Negativicutes	o__Acidaminococcales	f__Acidaminococcaceae	g__Phascolarctobacterium_A	s__Phascolarctobacterium_A succinatutens	GCF_000188175.1	539	539_5	F68J_bin.44.fa	F68J_bin.44.fa	99.4	1.2	649
F6D8_bin.17	d__Bacteria	p__Firmicutes	c__Negativicutes	o__Acidaminococcales	f__Acidaminococcaceae	g__Phascolarctobacterium_A	s__Phascolarctobacterium_A succinatutens	GCF_000188175.1	539	539_3	F6D8_bin.17.fa	F6D8_bin.17.fa	99.38	0.9	649
F6D9_bin.91	d__Bacteria	p__Firmicutes	c__Negativicutes	o__Acidaminococcales	f__Acidaminococcaceae	g__Phascolarctobacterium_A	s__Phascolarctobacterium_A succinatutens	GCF_000188175.1	539	539_1	F6DB_bin.90.fa	F6D9_bin.91.fa	93.66	2.15	649
F6DB_bin.90	d__Bacteria	p__Firmicutes	c__Negativicutes	o__Acidaminococcales	f__Acidaminococcaceae	g__Phascolarctobacterium_A	s__Phascolarctobacterium_A succinatutens	GCF_000188175.1	539	539_1	F6DB_bin.90.fa	F6DB_bin.90.fa	99.98	0.6	649
F6DG_bin.3	d__Bacteria	p__Firmicutes	c__Negativicutes	o__Acidaminococcales	f__Acidaminococcaceae	g__Phascolarctobacterium_A	s__Phascolarctobacterium_A succinatutens	GCF_000188175.1	539	539_4	F6DG_bin.3.fa	F6DG_bin.3.fa	94.01	1.84	649

Table S3. Taxonomic classifiation of the 2,421 detected MAGs

	Domain	Phylum	Class	Order	Family	Genus	Species	RefGenome	primary_cluster	secondary_cluster	repr_genome	genome	CheckM_completeness	CheckM_contamination	
F68C_bin.48	d__Bacteria	p__Firmicutes	c__Negativicutes	o__Acidaminococcales	f__Acidaminococcaceae	g__Phascolarctobacterium_A	s__Phascolarctobacterium_A succinatutens	GCF_000188175.1	539	539_2	F6MX_bin.53.fa	F68C_bin.48.fa	98.2	1.2	649
F68U_bin.88	d__Bacteria	p__Firmicutes	c__Negativicutes	o__Acidaminococcales	f__Acidaminococcaceae	g__Phascolarctobacterium_A	s__Phascolarctobacterium_A succinatutens	GCF_000188175.1	539	539_2	F6MX_bin.53.fa	F68U_bin.88.fa	98.8	1.2	649
F68W_bin.14	d__Bacteria	p__Firmicutes	c__Negativicutes	o__Acidaminococcales	f__Acidaminococcaceae	g__Phascolarctobacterium_A	s__Phascolarctobacterium_A succinatutens	GCF_000188175.1	539	539_2	F6MX_bin.53.fa	F68W_bin.14.fa	98.2	1.2	649
F6D7_bin.7	d__Bacteria	p__Firmicutes	c__Negativicutes	o__Acidaminococcales	f__Acidaminococcaceae	g__Phascolarctobacterium_A	s__Phascolarctobacterium_A succinatutens	GCF_000188175.1	539	539_2	F6MX_bin.53.fa	F6D7_bin.7.fa	98.2	1.2	649
F6DH_bin.89	d__Bacteria	p__Firmicutes	c__Negativicutes	o__Acidaminococcales	f__Acidaminococcaceae	g__Phascolarctobacterium_A	s__Phascolarctobacterium_A succinatutens	GCF_000188175.1	539	539_2	F6MX_bin.53.fa	F6DH_bin.89.fa	99.4	1.4	649

Table S3. Taxonomic classifiation of the 2,421 detected MAGs

	Domain	Phylum	Class	Order	Family	Genus	Species	RefGenome	primary_cluster	secondary_cluster	repr_genome	genome	CheckM_completeness	CheckM_contamination	
F6MX_bin.53	d__Bacteria	p__Firmicutes	c__Negativicutes	o__Acidaminococcales	f__Acidaminococcaceae	g__Phascolarctobacterium_A	s__Phascolarctobacterium_A succinatutens	GCF_000188175.1	539	539_2	F6MX_bin.53.fa	F6MX_bin.53.fa	99.2	1.2	649
F68K_bin.2	d__Bacteria	p__Firmicutes	c__Negativicutes	o__Selenomonadales	f__Selenomonadaceae	g__Anaerovibrio	s__	N/A	443	443_1	F68K_bin.2.fa	F68K_bin.2.fa	98.4	2.79	650
F6D7_bin.5	d__Bacteria	p__Firmicutes	c__Negativicutes	o__Selenomonadales	f__Selenomonadaceae	g__Anaerovibrio	s__	N/A	443	443_2	F6D7_bin.5.fa	F6D7_bin.5.fa	99.54	2.63	651
F68E_bin.22	d__Bacteria	p__Firmicutes	c__Negativicutes	o__Selenomonadales	f__Selenomonadaceae	g__Megamonas	s__Megamonas funiformis	GCF_000245775.1	170	170_1	F68E_bin.22.fa	F68E_bin.22.fa	93.02	0.72	651
F68K_bin.19	d__Bacteria	p__Firmicutes	c__Negativicutes	o__Selenomonadales	f__Selenomonadaceae	g__Megamonas	s__Megamonas funiformis	GCF_000245775.1	170	170_3	F68K_bin.19.fa	F68K_bin.19.fa	66.89	2.58	651
F68U_bin.41	d__Bacteria	p__Firmicutes	c__Negativicutes	o__Selenomonadales	f__Selenomonadaceae	g__Megamonas	s__Megamonas funiformis	GCF_000245775.1	170	170_2	F68U_bin.41.fa	F68U_bin.41.fa	96.26	3.34	651
F6DA_bin.72	d__Bacteria	p__Firmicutes	c__Negativicutes	o__Selenomonadales	f__Selenomonadaceae	g__Megamonas	s__Megamonas funiformis	GCF_000245775.1	170	170_4	F6DA_bin.72.fa	F6DA_bin.72.fa	100	1.27	651
F68D_bin.14	d__Bacteria	p__Firmicutes	c__Negativicutes	o__Selenomonadales	f__Selenomonadaceae	g__Mitsuokella	s__	N/A	591	591_1	F68D_bin.14.fa	F68D_bin.14.fa	100	0.08	652

Table S3. Taxonomic classifiation of the 2,421 detected MAGs

	Domain	Phylum	Class	Order	Family	Genus	Species	RefGenome	primary_cluster	secondary_cluster	repr_genome	genome	CheckM_completeness	CheckM_contamination	
F68T_bin.42	d__Bacteria	p__Firmicutes	c__Negativicutes	o__Selenomonadales	f__Selenomonadaceae	g__Mitsuokella	s__	N/A	591	591_2	F68T_bin.42.fa	F68T_bin.42.fa	96.26	0.08	653
F68H_bin.79	d__Bacteria	p__Firmicutes	c__Negativicutes	o__Selenomonadales	f__Selenomonadaceae	g__Mitsuokella	s__Mitsuokella multacida	GCF_000155955.1	592	592_4	F68H_bin.79.fa	F68H_bin.79.fa	99.07	0.08	653
F68N_bin.8	d__Bacteria	p__Firmicutes	c__Negativicutes	o__Selenomonadales	f__Selenomonadaceae	g__Mitsuokella	s__Mitsuokella multacida	GCF_000155955.1	592	592_1	F68N_bin.8.fa	F68N_bin.8.fa	86.65	0.82	653
F68R_bin.84	d__Bacteria	p__Firmicutes	c__Negativicutes	o__Selenomonadales	f__Selenomonadaceae	g__Mitsuokella	s__Mitsuokella multacida	GCF_000155955.1	592	592_3	F68R_bin.84.fa	F68R_bin.84.fa	57.74	0.08	653
F68U_bin.108	d__Bacteria	p__Firmicutes	c__Negativicutes	o__Selenomonadales	f__Selenomonadaceae	g__Mitsuokella	s__Mitsuokella multacida	GCF_000155955.1	592	592_2	F68U_bin.108.fa	F68U_bin.108.fa	82.73	3.82	653
F68G_bin.59	d__Bacteria	p__Firmicutes	c__Negativicutes	o__Veillonellales	f__Dialisteraceae	g__Allisonella	s__Allisonella histaminiformans	GCF_900103425.1	390	390_1	F68G_bin.59.fa	F68G_bin.59.fa	77.16	0.79	653
F68U_bin.27	d__Bacteria	p__Firmicutes	c__Negativicutes	o__Veillonellales	f__Dialisteraceae	g__Allisonella	s__Allisonella histaminiformans	GCF_900103425.1	390	390_2	F68U_bin.27.fa	F68U_bin.27.fa	83.07	2.58	653

Table S3. Taxonomic classifiation of the 2,421 detected MAGs

	Domain	Phylum	Class	Order	Family	Genus	Species	RefGenome	primary_cluster	secondary_cluster	repr_genome	genome	CheckM_completeness	CheckM_contamination	
F68D_bin.99	d__Bacteria	p__Firmicutes	c__Negativicutes	o__Veillonellales	f__Dialisteraceae	g__Dialister	s__	N/A	391	391_1	F68D_bin.99.fa	F68D_bin.99.fa	98.96	0	654
F68H_bin.14	d__Bacteria	p__Firmicutes	c__Negativicutes	o__Veillonellales	f__Dialisteraceae	g__Dialister	s__	N/A	391	391_4	F68H_bin.14.fa	F68H_bin.14.fa	95.8	0	655
F68K_bin.54	d__Bacteria	p__Firmicutes	c__Negativicutes	o__Veillonellales	f__Dialisteraceae	g__Dialister	s__	N/A	391	391_5	F68K_bin.54.fa	F68K_bin.54.fa	98.64	0	656
F68N_bin.69	d__Bacteria	p__Firmicutes	c__Negativicutes	o__Veillonellales	f__Dialisteraceae	g__Dialister	s__	N/A	391	391_3	F68N_bin.69.fa	F68N_bin.69.fa	99.59	0	657
F68R_bin.31	d__Bacteria	p__Firmicutes	c__Negativicutes	o__Veillonellales	f__Dialisteraceae	g__Dialister	s__	N/A	391	391_2	F68R_bin.31.fa	F68R_bin.31.fa	99.59	0	658
F68G_bin.39	d__Bacteria	p__Firmicutes	c__Negativicutes	o__Veillonellales	f__Dialisteraceae	g__Dialister	s__Dialister sp000434475	GCA_000434475.1	392	392_0	F68G_bin.39.fa	F68G_bin.39.fa	97.78	0.63	658
F68E_bin.4	d__Bacteria	p__Firmicutes	c__Negativicutes	o__Veillonellales	f__Megasphaeraceae	g__Anaeroglobus	s__Anaeroglobus micronuciformis	GCF_000165735.1	396	396_0	F68E_bin.4.fa	F68E_bin.4.fa	100	0	658
F68F_bin.2	d__Bacteria	p__Firmicutes	c__Negativicutes	o__Veillonellales	f__Megasphaeraceae	g__Caecibacter	s__	N/A	393	393_3	F68F_bin.2.fa	F68F_bin.2.fa	91.11	2.54	659
F6D9_bin.12	d__Bacteria	p__Firmicutes	c__Negativicutes	o__Veillonellales	f__Megasphaeraceae	g__Caecibacter	s__	N/A	393	393_1	F6D9_bin.12.fa	F6D9_bin.12.fa	99.9	1.29	660

Table S3. Taxonomic classifiation of the 2,421 detected MAGs

	Domain	Phylum	Class	Order	Family	Genus	Species	RefGenome	primary_cluster	secondary_cluster	repr_genome	genome	CheckM_completeness	CheckM_contamination	
F6MY_bin.138	d__Bacteria	p__Firmicutes	c__Negativicutes	o__Veillonellales	f__Megasphaeraceae	g__Caecibacter	s__	N/A	393	393_2	F6MY_bin.138.fa	F6MY_bin.138.fa	58.28	1.45	661
F68N_bin.12	d__Bacteria	p__Firmicutes	c__Negativicutes	o__Veillonellales	f__Megasphaeraceae	g__Megasphaera	s__Megasphaera elsdenii	GCF_003010495.1	395	395_4	F68N_bin.12.fa	F68N_bin.12.fa	95.91	0	661
F68H_bin.22	d__Bacteria	p__Firmicutes	c__Negativicutes	o__Veillonellales	f__Megasphaeraceae	g__Megasphaera	s__Megasphaera sp000417505	GCF_000417505.1	395	395_1	F68H_bin.22.fa	F68H_bin.22.fa	95.21	0.6	661
F68R_bin.35	d__Bacteria	p__Firmicutes	c__Negativicutes	o__Veillonellales	f__Megasphaeraceae	g__Megasphaera	s__Megasphaera sp000417505	GCF_000417505.1	395	395_3	F68R_bin.35.fa	F68R_bin.35.fa	96.21	0	661
F68T_bin.10	d__Bacteria	p__Firmicutes	c__Negativicutes	o__Veillonellales	f__Megasphaeraceae	g__Megasphaera	s__Megasphaera sp000417505	GCF_000417505.1	395	395_2	F68T_bin.10.fa	F68T_bin.10.fa	86.59	0.35	661
F68K_bin.25	d__Bacteria	p__Firmicutes	c__Negativicutes	o__Veillonellales	f__Megasphaeraceae	g__Megasphaera	s__Megasphaera sp900066485	GCA_900066485.1	394	394_1	F68K_bin.25.fa	F68K_bin.25.fa	98.8	1.2	661
F68U_bin.18	d__Bacteria	p__Firmicutes	c__Negativicutes	o__Veillonellales	f__Megasphaeraceae	g__Megasphaera	s__Megasphaera sp900066485	GCA_900066485.1	394	394_2	F68U_bin.18.fa	F68U_bin.18.fa	98.8	1.8	661
F68R_bin.123	d__Bacteria	p__Firmicutes	c__Negativicutes	o__Veillonellales	f__Veillonellaceae	g__Veillonella	s__Veillonella atypica	GCF_002959915.1	147	147_0	F68R_bin.123.fa	F68R_bin.123.fa	75.59	0	661

Table S3. Taxonomic classifiation of the 2,421 detected MAGs

	Domain	Phylum	Class	Order	Family	Genus	Species	RefGenome	primary_cluster	secondary_cluster	repr_genome	genome	CheckM_completeness	CheckM_contamination	
F6DE_bin.29	d__Bacteria	p__Firmicutes	c__Negativicutes	o__Veillonellales	f__Veillonellaceae	g__Veillonella	s__Veillonella parvula	GCF_000024945.1	148	148_0	F6DE_bin.29.fa	F6DE_bin.29.fa	75.84	3.69	661
F6D8_bin.8	d__Bacteria	p__Firmicutes	c__Negativicutes	o__Veillonellales	f__Veillonellaceae	g__Veillonella_A	s__Veillonella_A magna	GCF_000428745.1	8	8_0	F6D8_bin.8.fa	F6D8_bin.8.fa	100	0	661
F6DE_bin.19	d__Bacteria	p__Fusobacteriota	c__Fusobacteriia	o__Fusobacteriales	f__Fusobacteriaceae	g__Cetobacterium_A	s__	N/A	204	204_0	F6DE_bin.19.fa	F6DE_bin.19.fa	96.63	0	662
F6D9_bin.88	d__Bacteria	p__Fusobacteriota	c__Fusobacteriia	o__Fusobacteriales	f__Fusobacteriaceae	g__Fusobacterium_A	s__	N/A	201	201_2	F6D9_bin.88.fa	F6D9_bin.88.fa	55.59	1.12	663
F6DE_bin.4	d__Bacteria	p__Fusobacteriota	c__Fusobacteriia	o__Fusobacteriales	f__Fusobacteriaceae	g__Fusobacterium_A	s__	N/A	202	202_0	F6DE_bin.4.fa	F6DE_bin.4.fa	98.88	1.12	664
F68C_bin.95	d__Bacteria	p__Fusobacteriota	c__Fusobacteriia	o__Fusobacteriales	f__Fusobacteriaceae	g__Fusobacterium_A	s__Fusobacterium_A mortiferum	GCF_000158195.2	201	201_1	F68J_bin.67.fa	F68C_bin.95.fa	97.75	1.12	664
F68D_bin.23	d__Bacteria	p__Fusobacteriota	c__Fusobacteriia	o__Fusobacteriales	f__Fusobacteriaceae	g__Fusobacterium_A	s__Fusobacterium_A mortiferum	GCF_000158195.2	201	201_1	F68J_bin.67.fa	F68D_bin.23.fa	93.26	2.81	664
F68G_bin.84	d__Bacteria	p__Fusobacteriota	c__Fusobacteriia	o__Fusobacteriales	f__Fusobacteriaceae	g__Fusobacterium_A	s__Fusobacterium_A mortiferum	GCF_000158195.2	201	201_1	F68J_bin.67.fa	F68G_bin.84.fa	51.88	1.72	664

Table S3. Taxonomic classifiation of the 2,421 detected MAGs

	Domain	Phylum	Class	Order	Family	Genus	Species	RefGenome	primary_cluster	secondary_cluster	repr_genome	genome	CheckM_completeness	CheckM_contamination	
F68J_bin.67	d__Bacteria	p__Fusobacteriota	c__Fusobacteriia	o__Fusobacteriales	f__Fusobacteriaceae	g__Fusobacterium_A	s__Fusobacterium_A mortiferum	GCF_000158195.2	201	201_1	F68J_bin.67.fa	F68J_bin.67.fa	100	1.12	664
F68K_bin.38	d__Bacteria	p__Fusobacteriota	c__Fusobacteriia	o__Fusobacteriales	f__Fusobacteriaceae	g__Fusobacterium_A	s__Fusobacterium_A mortiferum	GCF_000158195.2	201	201_1	F68J_bin.67.fa	F68K_bin.38.fa	97.56	2.25	664
F68P_bin.50	d__Bacteria	p__Fusobacteriota	c__Fusobacteriia	o__Fusobacteriales	f__Fusobacteriaceae	g__Fusobacterium_A	s__Fusobacterium_A mortiferum	GCF_000158195.2	201	201_1	F68J_bin.67.fa	F68P_bin.50.fa	98.88	1.12	664
F68T_bin.33	d__Bacteria	p__Fusobacteriota	c__Fusobacteriia	o__Fusobacteriales	f__Fusobacteriaceae	g__Fusobacterium_A	s__Fusobacterium_A mortiferum	GCF_000158195.2	201	201_1	F68J_bin.67.fa	F68T_bin.33.fa	56.22	0.94	664
F6DA_bin.25	d__Bacteria	p__Fusobacteriota	c__Fusobacteriia	o__Fusobacteriales	f__Fusobacteriaceae	g__Fusobacterium_A	s__Fusobacterium_A mortiferum	GCF_000158195.2	201	201_1	F68J_bin.67.fa	F6DA_bin.25.fa	98.88	1.12	664
F6DG_bin.65	d__Bacteria	p__Fusobacteriota	c__Fusobacteriia	o__Fusobacteriales	f__Fusobacteriaceae	g__Fusobacterium_A	s__Fusobacterium_A mortiferum	GCF_000158195.2	201	201_1	F68J_bin.67.fa	F6DG_bin.65.fa	94.38	2.25	664

Table S3. Taxonomic classifiation of the 2,421 detected MAGs

	Domain	Phylum	Class	Order	Family	Genus	Species	RefGenome	primary_cluster	secondary_cluster	repr_genome	genome	CheckM_completeness	CheckM_contamination	
F68R_bin.56	d__Bacteria	p__Fusobacteriota	c__Fusobacteriia	o__Fusobacteriales	f__Fusobacteriaceae	g__Fusobacterium_B	s__	N/A	199	199_0	F68R_bin.56.fa	F68R_bin.56.fa	100	1.69	665
F68U_bin.55	d__Bacteria	p__Fusobacteriota	c__Fusobacteriia	o__Fusobacteriales	f__Fusobacteriaceae	g__Fusobacterium_B	s__	N/A	200	200_0	F68U_bin.55.fa	F68U_bin.55.fa	100	1.12	666
F68W_bin.29	d__Bacteria	p__Myxococcota	c__Bradimonadia	o__Bradymonadales	f__UBA4248	g__UBA4248	s__	N/A	4	4_1	F6DH_bin.23.fa	F68W_bin.29.fa	74.39	1.77	667
F6DH_bin.23	d__Bacteria	p__Myxococcota	c__Bradimonadia	o__Bradymonadales	f__UBA4248	g__UBA4248	s__	N/A	4	4_1	F6DH_bin.23.fa	F6DH_bin.23.fa	75.68	1.77	668
F68M_bin.4	d__Bacteria	p__Patescibacteria	c__Saccharimonadia	o__Saccharimonadales	f__Saccharimonadaceae	g__	s__	N/A	119	119_0	F68M_bin.4.fa	F68M_bin.4.fa	56.24	0.85	669
F68U_bin.14	d__Bacteria	p__Proteobacteria	c__Alphaproteobacteria	o__RF32	f__CAG-977	g__CAG-977	s__	N/A	3	3_0	F68U_bin.14.fa	F68U_bin.14.fa	68.99	2.49	670
F68D_bin.106	d__Bacteria	p__Proteobacteria	c__Alphaproteobacteria	o__RF32	f__CAG-977	g__CAG-977	s__CAG-977 sp000434295	GCA_000434295.1	1	1_1	F68U_bin.83.fa	F68D_bin.106.fa	77.91	1.24	670
F68U_bin.83	d__Bacteria	p__Proteobacteria	c__Alphaproteobacteria	o__RF32	f__CAG-977	g__CAG-977	s__CAG-977 sp000434295	GCA_000434295.1	1	1_1	F68U_bin.83.fa	F68U_bin.83.fa	80.47	0.79	670
F68U_bin.76	d__Bacteria	p__Proteobacteria	c__Alphaproteobacteria	o__RF32	f__CAG-977	g__UBA11549	s__	N/A	2	2_1	F68U_bin.76.fa	F68U_bin.76.fa	81.72	0.29	671

Table S3. Taxonomic classifiation of the 2,421 detected MAGs

	Domain	Phylum	Class	Order	Family	Genus	Species	RefGenome	primary_cluster	secondary_cluster	repr_genome	genome	CheckM_completeness	CheckM_contamination	
F6D7_bin.88	d__Bacteria	p__Proteobacteria	c__Alphaproteobacteria	o__RF32	f__CAG-977	g__UBA11549	s__	N/A	2	2_2	F6D7_bin.88.fa	F6D7_bin.88.fa	55.49	1.72	672
F68K_bin.108	d__Bacteria	p__Proteobacteria	c__Gammaproteobacteria	o__Burkholderiales	f__Burkholderiaceae	g__CAG-521	s__	N/A	248	248_1	F68K_bin.108.fa	F68K_bin.108.fa	94.27	2.94	673
F6MX_bin.33	d__Bacteria	p__Proteobacteria	c__Gammaproteobacteria	o__Burkholderiales	f__Burkholderiaceae	g__CAG-521	s__	N/A	248	248_2	F6MX_bin.33.fa	F6MX_bin.33.fa	59.23	4.38	674
F68U_bin.34	d__Bacteria	p__Proteobacteria	c__Gammaproteobacteria	o__Burkholderiales	f__Burkholderiaceae	g__CAG-521	s__	N/A	250	250_0	F68U_bin.34.fa	F68U_bin.34.fa	96.23	1.25	675
F6DA_bin.14	d__Bacteria	p__Proteobacteria	c__Gammaproteobacteria	o__Burkholderiales	f__Burkholderiaceae	g__CAG-521	s__	N/A	256	256_0	F6DA_bin.14.fa	F6DA_bin.14.fa	83.47	1.73	676
F68C_bin.98	d__Bacteria	p__Proteobacteria	c__Gammaproteobacteria	o__Burkholderiales	f__Burkholderiaceae	g__CAG-521	s__CAG-521 sp000437635	GCA_000437635.1	249	249_7	F68C_bin.98.fa	F68C_bin.98.fa	96.15	1.88	676
F68F_bin.57	d__Bacteria	p__Proteobacteria	c__Gammaproteobacteria	o__Burkholderiales	f__Burkholderiaceae	g__CAG-521	s__CAG-521 sp000437635	GCA_000437635.1	249	249_4	F68F_bin.57.fa	F68F_bin.57.fa	97.4	1.25	676

Table S3. Taxonomic classifiation of the 2,421 detected MAGs

	Domain	Phylum	Class	Order	Family	Genus	Species	RefGenome	primary_cluster	secondary_cluster	repr_genome	genome	CheckM_completeness	CheckM_contamination	
F68J_bin.74	d__Bacteria	p__Proteobacteria	c__Gammaproteobacteria	o__Burkholderiales	f__Burkholderiaceae	g__CAG-521	s__CAG-521 sp000437635	GCA_000437635.1	249	249_9	F68J_bin.74.fa	F68J_bin.74.fa	97.4	1.25	676
F68N_bin.74	d__Bacteria	p__Proteobacteria	c__Gammaproteobacteria	o__Burkholderiales	f__Burkholderiaceae	g__CAG-521	s__CAG-521 sp000437635	GCA_000437635.1	249	249_5	F68N_bin.74.fa	F68N_bin.74.fa	79.87	1.96	676
F68T_bin.76	d__Bacteria	p__Proteobacteria	c__Gammaproteobacteria	o__Burkholderiales	f__Burkholderiaceae	g__CAG-521	s__CAG-521 sp000437635	GCA_000437635.1	249	249_8	F68T_bin.76.fa	F68T_bin.76.fa	97.08	1.25	676
F68U_bin.2	d__Bacteria	p__Proteobacteria	c__Gammaproteobacteria	o__Burkholderiales	f__Burkholderiaceae	g__CAG-521	s__CAG-521 sp000437635	GCA_000437635.1	249	249_2	F68U_bin.2.fa	F68U_bin.2.fa	60.67	0	676
F68W_bin.66	d__Bacteria	p__Proteobacteria	c__Gammaproteobacteria	o__Burkholderiales	f__Burkholderiaceae	g__CAG-521	s__CAG-521 sp000437635	GCA_000437635.1	249	249_1	F6DH_bin.66.fa	F68W_bin.66.fa	95.83	1.88	676
F6DH_bin.66	d__Bacteria	p__Proteobacteria	c__Gammaproteobacteria	o__Burkholderiales	f__Burkholderiaceae	g__CAG-521	s__CAG-521 sp000437635	GCA_000437635.1	249	249_1	F6DH_bin.66.fa	F6DH_bin.66.fa	95.83	1.88	676
F6MX_bin.105	d__Bacteria	p__Proteobacteria	c__Gammaproteobacteria	o__Burkholderiales	f__Burkholderiaceae	g__CAG-521	s__CAG-521 sp000437635	GCA_000437635.1	249	249_3	F6MX_bin.105.fa	F6MX_bin.105.fa	88.38	1.88	676

Table S3. Taxonomic classifiation of the 2,421 detected MAGs

	Domain	Phylum	Class	Order	Family	Genus	Species	RefGenome	primary_cluster	secondary_cluster	repr_genome	genome	CheckM_completeness	CheckM_contamination	
F6MY_bin.81	d__Bacteria	p__Proteobacteria	c__Gammaproteobacteria	o__Burkholderiales	f__Burkholderiaceae	g__CAG-521	s__CAG-521 sp000437635	GCA_000437635.1	249	249_6	F6MY_bin.81.fa	F6MY_bin.81.fa	91.15	2.74	676
F6DG_bin.93	d__Bacteria	p__Proteobacteria	c__Gammaproteobacteria	o__Burkholderiales	f__Burkholderiaceae	g__CAG-521	s__CAG-521 sp002329575	GCA_002329575.1	251	251_0	F6DG_bin.93.fa	F6DG_bin.93.fa	86.11	1.81	676
F68D_bin.44	d__Bacteria	p__Proteobacteria	c__Gammaproteobacteria	o__Burkholderiales	f__Burkholderiaceae	g__Comamonas	s__Comamonas kerstersii	GCF_002056725.1	432	432_1	F68M_bin.31.fa	F68D_bin.44.fa	97.15	1.14	676
F68M_bin.31	d__Bacteria	p__Proteobacteria	c__Gammaproteobacteria	o__Burkholderiales	f__Burkholderiaceae	g__Comamonas	s__Comamonas kerstersii	GCF_002056725.1	432	432_1	F68M_bin.31.fa	F68M_bin.31.fa	96.53	0.67	676
F6D9_bin.19	d__Bacteria	p__Proteobacteria	c__Gammaproteobacteria	o__Burkholderiales	f__Burkholderiaceae	g__Comamonas	s__Comamonas kerstersii	GCF_002056725.1	432	432_1	F68M_bin.31.fa	F6D9_bin.19.fa	95.02	1.98	676
F68C_bin.94	d__Bacteria	p__Proteobacteria	c__Gammaproteobacteria	o__Burkholderiales	f__Burkholderiaceae	g__Duodenibacillus	s__	N/A	252	252_5	F68C_bin.94.fa	F68C_bin.94.fa	97.5	0.62	677
F68F_bin.77	d__Bacteria	p__Proteobacteria	c__Gammaproteobacteria	o__Burkholderiales	f__Burkholderiaceae	g__Duodenibacillus	s__	N/A	252	252_4	F68F_bin.77.fa	F68F_bin.77.fa	98.12	0.62	678

Table S3. Taxonomic classifiation of the 2,421 detected MAGs

	Domain	Phylum	Class	Order	Family	Genus	Species	RefGenome	primary_cluster	secondary_cluster	repr_genome	genome	CheckM_completeness	CheckM_contamination	
F68P_bin.14	d__Bacteria	p__Proteobacteria	c__Gammaproteobacteria	o__Burkholderiales	f__Burkholderiaceae	g__Duodenibacillus	s__	N/A	252	252_2	F68P_bin.14.fa	F68P_bin.14.fa	98.75	0.62	679
F68T_bin.24	d__Bacteria	p__Proteobacteria	c__Gammaproteobacteria	o__Burkholderiales	f__Burkholderiaceae	g__Duodenibacillus	s__	N/A	252	252_6	F68T_bin.24.fa	F68T_bin.24.fa	58.83	0.9	680
F68U_bin.107	d__Bacteria	p__Proteobacteria	c__Gammaproteobacteria	o__Burkholderiales	f__Burkholderiaceae	g__Duodenibacillus	s__	N/A	252	252_1	F68U_bin.107.fa	F68U_bin.107.fa	97.12	0.62	681
F68W_bin.20	d__Bacteria	p__Proteobacteria	c__Gammaproteobacteria	o__Burkholderiales	f__Burkholderiaceae	g__Duodenibacillus	s__	N/A	252	252_1	F68U_bin.107.fa	F68W_bin.20.fa	89.79	1.25	682
F6DH_bin.110	d__Bacteria	p__Proteobacteria	c__Gammaproteobacteria	o__Burkholderiales	f__Burkholderiaceae	g__Duodenibacillus	s__	N/A	252	252_1	F68U_bin.107.fa	F6DH_bin.110.fa	95	1.38	683
F6DB_bin.32	d__Bacteria	p__Proteobacteria	c__Gammaproteobacteria	o__Burkholderiales	f__Burkholderiaceae	g__Duodenibacillus	s__	N/A	252	252_3	F6DB_bin.32.fa	F6DB_bin.32.fa	98.75	0.62	684
F68C_bin.68	d__Bacteria	p__Proteobacteria	c__Gammaproteobacteria	o__Burkholderiales	f__Burkholderiaceae	g__Duodenibacillus	s__	N/A	253	253_0	F68C_bin.68.fa	F68C_bin.68.fa	61.38	0	685

Table S3. Taxonomic classifiation of the 2,421 detected MAGs

	Domain	Phylum	Class	Order	Family	Genus	Species	RefGenome	primary_cluster	secondary_cluster	repr_genome	genome	CheckM_completeness	CheckM_contamination	
F68J_bin.5	d__Bacteria	p__Proteobacteria	c__Gammaproteobacteria	o__Burkholderiales	f__Burkholderiaceae	g__Duodenibacillus	s__	N/A	254	254_1	F68J_bin.5.fa	F68J_bin.5.fa	97.4	1.25	686
F68R_bin.1	d__Bacteria	p__Proteobacteria	c__Gammaproteobacteria	o__Burkholderiales	f__Burkholderiaceae	g__Duodenibacillus	s__	N/A	254	254_1	F68J_bin.5.fa	F68R_bin.1.fa	91.09	2.71	687
F68G_bin.27	d__Bacteria	p__Proteobacteria	c__Gammaproteobacteria	o__Burkholderiales	f__Burkholderiaceae	g__Oxalobacter	s__	N/A	384	384_1	F68G_bin.27.fa	F68G_bin.27.fa	99.12	0.24	688
F68W_bin.1	d__Bacteria	p__Proteobacteria	c__Gammaproteobacteria	o__Burkholderiales	f__Burkholderiaceae	g__Oxalobacter	s__	N/A	384	384_2	F68W_bin.1.fa	F68W_bin.1.fa	96.57	0.48	689
F68F_bin.42	d__Bacteria	p__Proteobacteria	c__Gammaproteobacteria	o__Burkholderiales	f__Burkholderiaceae	g__Oxalobacter	s__	N/A	385	385_0	F68F_bin.42.fa	F68F_bin.42.fa	61.32	0.48	690
F68E_bin.31	d__Bacteria	p__Proteobacteria	c__Gammaproteobacteria	o__Burkholderiales	f__Burkholderiaceae	g__Parasutterella	s__Parasutterella excrementihominis	GCF_000205025.1	255	255_0	F68E_bin.31.fa	F68E_bin.31.fa	100	0.11	690
F68F_bin.39	d__Bacteria	p__Proteobacteria	c__Gammaproteobacteria	o__Burkholderiales	f__Burkholderiaceae	g__Sutterella	s__	N/A	471	471_1	F68F_bin.39.fa	F68F_bin.39.fa	97.38	1.25	691

Table S3. Taxonomic classifiation of the 2,421 detected MAGs

	Domain	Phylum	Class	Order	Family	Genus	Species	RefGenome	primary_cluster	secondary_cluster	repr_genome	genome	CheckM_completeness	CheckM_contamination	
F68U_bin.32	d__Bacteria	p__Proteobacteria	c__Gammaproteobacteria	o__Burkholderiales	f__Burkholderiaceae	g__Sutterella	s__	N/A	471	471_2	F68U_bin.32.fa	F68U_bin.32.fa	63.33	2.33	692
F6D7_bin.69	d__Bacteria	p__Proteobacteria	c__Gammaproteobacteria	o__Burkholderiales	f__Burkholderiaceae	g__Sutterella	s__	N/A	474	474_0	F6D7_bin.69.fa	F6D7_bin.69.fa	68.59	0	693
F68U_bin.11	d__Bacteria	p__Proteobacteria	c__Gammaproteobacteria	o__Burkholderiales	f__Burkholderiaceae	g__Sutterella	s__Sutterella parvirubra	GCF_000250875.1	472	472_1	F68U_bin.11.fa	F68U_bin.11.fa	98.14	1.55	693
F6DA_bin.82	d__Bacteria	p__Proteobacteria	c__Gammaproteobacteria	o__Burkholderiales	f__Burkholderiaceae	g__Sutterella	s__Sutterella parvirubra	GCF_000250875.1	472	472_2	F6DA_bin.82.fa	F6DA_bin.82.fa	98.14	1.55	693
F6DG_bin.66	d__Bacteria	p__Proteobacteria	c__Gammaproteobacteria	o__Burkholderiales	f__Burkholderiaceae	g__Sutterella	s__Sutterella parvirubra	GCF_000250875.1	472	472_3	F6DG_bin.66.fa	F6DG_bin.66.fa	98.14	1.55	693
F68H_bin.15	d__Bacteria	p__Proteobacteria	c__Gammaproteobacteria	o__Burkholderiales	f__Burkholderiaceae	g__Sutterella	s__Sutterella wadsworthensis_A	GCF_000297775.1	473	473_2	F68H_bin.15.fa	F68H_bin.15.fa	100	0.62	693
F68K_bin.92	d__Bacteria	p__Proteobacteria	c__Gammaproteobacteria	o__Burkholderiales	f__Burkholderiaceae	g__Sutterella	s__Sutterella wadsworthensis_A	GCF_000297775.1	473	473_1	F68R_bin.81.fa	F68K_bin.92.fa	98.14	0.62	693

Table S3. Taxonomic classifiation of the 2,421 detected MAGs

	Domain	Phylum	Class	Order	Family	Genus	Species	RefGenome	primary_cluster	secondary_cluster	repr_genome	genome	CheckM_completeness	CheckM_contamination	
F68N_bin.48	d__Bacteria	p__Proteobacteria	c__Gammaproteobacteria	o__Burkholderiales	f__Burkholderiaceae	g__Sutterella	s__Sutterella wadsworthensis_A	GCF_000297775.1	473	473_1	F68R_bin.81.fa	F68N_bin.48.fa	98.14	1.24	693
F68R_bin.81	d__Bacteria	p__Proteobacteria	c__Gammaproteobacteria	o__Burkholderiales	f__Burkholderiaceae	g__Sutterella	s__Sutterella wadsworthensis_A	GCF_000297775.1	473	473_1	F68R_bin.81.fa	F68R_bin.81.fa	98.14	0.62	693
F6D7_bin.101	d__Bacteria	p__Proteobacteria	c__Gammaproteobacteria	o__Burkholderiales	f__Burkholderiaceae	g__Sutterella	s__Sutterella wadsworthensis_A	GCF_000297775.1	473	473_1	F68R_bin.81.fa	F6D7_bin.101.fa	81.03	2.59	693
F6DB_bin.20	d__Bacteria	p__Proteobacteria	c__Gammaproteobacteria	o__Burkholderiales	f__Burkholderiaceae	g__Sutterella	s__Sutterella wadsworthensis_A	GCF_000297775.1	473	473_1	F68R_bin.81.fa	F6DB_bin.20.fa	53.1	1.72	693
F6DE_bin.48	d__Bacteria	p__Proteobacteria	c__Gammaproteobacteria	o__Burkholderiales	f__Burkholderiaceae	g__Sutterella	s__Sutterella wadsworthensis_A	GCF_000297775.1	473	473_1	F68R_bin.81.fa	F6DE_bin.48.fa	80.43	2.93	693
F6MY_bin.59	d__Bacteria	p__Proteobacteria	c__Gammaproteobacteria	o__Burkholderiales	f__Burkholderiaceae	g__Sutterella	s__Sutterella wadsworthensis_A	GCF_000297775.1	473	473_1	F68R_bin.81.fa	F6MY_bin.59.fa	58.39	0	693

Table S3. Taxonomic classifiation of the 2,421 detected MAGs

	Domain	Phylum	Class	Order	Family	Genus	Species	RefGenome	primary_cluster	secondary_cluster	repr_genome	genome	CheckM_completeness	CheckM_contamination	
F6D9_bin.93	d__Bacteria	p__Proteobacteria	c__Gammaproteobacteria	o__Burkholderiales	f__Burkholderiaceae	g__Sutterella	s__Sutterella wadsworthensis_A	GCF_000297775.1	473	473_3	F6D9_bin.93.fa	F6D9_bin.93.fa	100	1.24	693
F6D7_bin.11	d__Bacteria	p__Proteobacteria	c__Gammaproteobacteria	o__Burkholderiales	f__Burkholderiaceae	g__Sutterella	s__Sutterella wadsworthensis_B	GCF_000411515.1	470	470_1	F6D7_bin.11.fa	F6D7_bin.11.fa	91.93	0	693
F6DA_bin.86	d__Bacteria	p__Proteobacteria	c__Gammaproteobacteria	o__Burkholderiales	f__Burkholderiaceae	g__Sutterella	s__Sutterella wadsworthensis_B	GCF_000411515.1	470	470_2	F6DA_bin.86.fa	F6DA_bin.86.fa	91.3	0	693
F6MX_bin.138	d__Bacteria	p__Proteobacteria	c__Gammaproteobacteria	o__Enterobacterales	f__Aeromonadaceae	g__Aeromonas	s__Aeromonas enteropelogenes	GCF_000819845.1	597	597_0	F6MX_bin.138.fa	F6MX_bin.138.fa	72.4	4.7	693
F6D9_bin.31	d__Bacteria	p__Proteobacteria	c__Gammaproteobacteria	o__Enterobacterales	f__Enterobacteriaceae	g__Citrobacter	s__Citrobacter freundii	GCF_000759735.1	122	122_0	F6D9_bin.31.fa	F6D9_bin.31.fa	77.07	1.72	693
F68D_bin.82	d__Bacteria	p__Proteobacteria	c__Gammaproteobacteria	o__Enterobacterales	f__Enterobacteriaceae	g__Escherichia	s__Escherichia coli	GCF_000690815.1	121	121_4	F68D_bin.82.fa	F68D_bin.82.fa	99.97	0.9	693

Table S3. Taxonomic classifiation of the 2,421 detected MAGs

	Domain	Phylum	Class	Order	Family	Genus	Species	RefGenome	primary_cluster	secondary_cluster	repr_genome	genome	CheckM_completeness	CheckM_contamination	
F68R_bin.13	d__Bacteria	p__Proteobacteria	c__Gammaproteobacteria	o__Enterobacterales	f__Enterobacteriaceae	g__Escherichia	s__Escherichia coli	GCF_000690815.1	121	121_5	F68R_bin.13.fa	F68R_bin.13.fa	75.62	2.96	693
F6DH_bin.43	d__Bacteria	p__Proteobacteria	c__Gammaproteobacteria	o__Enterobacterales	f__Enterobacteriaceae	g__Escherichia	s__Escherichia coli_D	GCF_000026325.1	121	121_1	F6DH_bin.43.fa	F6DH_bin.43.fa	97.49	2.42	693
F6MY_bin.111	d__Bacteria	p__Proteobacteria	c__Gammaproteobacteria	o__Enterobacterales	f__Enterobacteriaceae	g__Escherichia	s__Escherichia coli_D	GCF_000026325.1	121	121_1	F6DH_bin.43.fa	F6MY_bin.111.fa	75.58	1.69	693
F68K_bin.94	d__Bacteria	p__Proteobacteria	c__Gammaproteobacteria	o__Enterobacterales	f__Enterobacteriaceae	g__Escherichia	s__Escherichia flexneri	GCA_002950215.1	121	121_2	F6DA_bin.29.fa	F68K_bin.94.fa	93.26	2.95	693
F68T_bin.15	d__Bacteria	p__Proteobacteria	c__Gammaproteobacteria	o__Enterobacterales	f__Enterobacteriaceae	g__Escherichia	s__Escherichia flexneri	GCA_002950215.1	121	121_2	F6DA_bin.29.fa	F68T_bin.15.fa	84.69	1.71	693
F68U_bin.48	d__Bacteria	p__Proteobacteria	c__Gammaproteobacteria	o__Enterobacterales	f__Enterobacteriaceae	g__Escherichia	s__Escherichia flexneri	GCA_002950215.1	121	121_2	F6DA_bin.29.fa	F68U_bin.48.fa	99.24	0.74	693
F6D8_bin.16	d__Bacteria	p__Proteobacteria	c__Gammaproteobacteria	o__Enterobacterales	f__Enterobacteriaceae	g__Escherichia	s__Escherichia flexneri	GCA_002950215.1	121	121_2	F6DA_bin.29.fa	F6D8_bin.16.fa	76.68	2.44	693

Table S3. Taxonomic classifiation of the 2,421 detected MAGs

	Domain	Phylum	Class	Order	Family	Genus	Species	RefGenome	primary_cluster	secondary_cluster	repr_genome	genome	CheckM_completeness	CheckM_contamination	
F6D9_bin.60	d__Bacteria	p__Proteobacteria	c__Gammaproteobacteria	o__Enterobacterales	f__Enterobacteriaceae	g__Escherichia	s__Escherichia flexneri	GCA_002950215.1	121	121_2	F6DA_bin.29.fa	F6D9_bin.60.fa	82.26	2.44	693
F6DA_bin.29	d__Bacteria	p__Proteobacteria	c__Gammaproteobacteria	o__Enterobacterales	f__Enterobacteriaceae	g__Escherichia	s__Escherichia flexneri	GCA_002950215.1	121	121_2	F6DA_bin.29.fa	F6DA_bin.29.fa	99.62	0.1	693
F68C_bin.110	d__Bacteria	p__Proteobacteria	c__Gammaproteobacteria	o__Enterobacterales	f__Enterobacteriaceae	g__Escherichia	s__Escherichia flexneri	GCA_002950215.1	121	121_3	F6DE_bin.51.fa	F68C_bin.110.fa	86.43	3.66	693
F68F_bin.105	d__Bacteria	p__Proteobacteria	c__Gammaproteobacteria	o__Enterobacterales	f__Enterobacteriaceae	g__Escherichia	s__Escherichia flexneri	GCA_002950215.1	121	121_3	F6DE_bin.51.fa	F68F_bin.105.fa	77	2.1	693
F68H_bin.78	d__Bacteria	p__Proteobacteria	c__Gammaproteobacteria	o__Enterobacterales	f__Enterobacteriaceae	g__Escherichia	s__Escherichia flexneri	GCA_002950215.1	121	121_3	F6DE_bin.51.fa	F68H_bin.78.fa	99.67	0.12	693
F68J_bin.52	d__Bacteria	p__Proteobacteria	c__Gammaproteobacteria	o__Enterobacterales	f__Enterobacteriaceae	g__Escherichia	s__Escherichia flexneri	GCA_002950215.1	121	121_3	F6DE_bin.51.fa	F68J_bin.52.fa	92.07	3.09	693
F68N_bin.13	d__Bacteria	p__Proteobacteria	c__Gammaproteobacteria	o__Enterobacterales	f__Enterobacteriaceae	g__Escherichia	s__Escherichia flexneri	GCA_002950215.1	121	121_3	F6DE_bin.51.fa	F68N_bin.13.fa	99.85	1.36	693

Table S3. Taxonomic classifiation of the 2,421 detected MAGs

	Domain	Phylum	Class	Order	Family	Genus	Species	RefGenome	primary_cluster	secondary_cluster	repr_genome	genome	CheckM_completeness	CheckM_contamination	
F68V_bin.92	d__Bacteria	p__Proteobacteria	c__Gammaproteobacteria	o__Enterobacterales	f__Enterobacteriaceae	g__Escherichia	s__Escherichia flexneri	GCA_002950215.1	121	121_3	F6DE_bin.51.fa	F68V_bin.92.fa	99.47	0.04	693
F6D7_bin.65	d__Bacteria	p__Proteobacteria	c__Gammaproteobacteria	o__Enterobacterales	f__Enterobacteriaceae	g__Escherichia	s__Escherichia flexneri	GCA_002950215.1	121	121_3	F6DE_bin.51.fa	F6D7_bin.65.fa	62.93	1.72	693
F6DB_bin.17	d__Bacteria	p__Proteobacteria	c__Gammaproteobacteria	o__Enterobacterales	f__Enterobacteriaceae	g__Escherichia	s__Escherichia flexneri	GCA_002950215.1	121	121_3	F6DE_bin.51.fa	F6DB_bin.17.fa	61.94	2.51	693
F6DE_bin.51	d__Bacteria	p__Proteobacteria	c__Gammaproteobacteria	o__Enterobacterales	f__Enterobacteriaceae	g__Escherichia	s__Escherichia flexneri	GCA_002950215.1	121	121_3	F6DE_bin.51.fa	F6DE_bin.51.fa	99.97	0.07	693
F6MX_bin.52	d__Bacteria	p__Proteobacteria	c__Gammaproteobacteria	o__Enterobacterales	f__Enterobacteriaceae	g__Escherichia	s__Escherichia flexneri	GCA_002950215.1	121	121_3	F6DE_bin.51.fa	F6MX_bin.52.fa	92.17	3.86	693
F68K_bin.12	d__Bacteria	p__Proteobacteria	c__Gammaproteobacteria	o__Enterobacterales	f__Enterobacteriaceae	g__Klebsiella	s__Klebsiella pneumoniae	GCF_000742135.1	120	120_3	F68K_bin.12.fa	F68K_bin.12.fa	56.03	3.45	693
F68R_bin.25	d__Bacteria	p__Proteobacteria	c__Gammaproteobacteria	o__Enterobacterales	f__Enterobacteriaceae	g__Klebsiella	s__Klebsiella pneumoniae	GCF_000742135.1	120	120_2	F68R_bin.25.fa	F68R_bin.25.fa	68.45	2.59	693

Table S3. Taxonomic classifiation of the 2,421 detected MAGs

	Domain	Phylum	Class	Order	Family	Genus	Species	RefGenome	primary_cluster	secondary_cluster	repr_genome	genome	CheckM_completeness	CheckM_contamination	
F68D_bin.40	d__Bacteria	p__Proteobacteria	c__Gammaproteobacteria	o__Enterobacterales	f__Enterobacteriaceae	g__Klebsiella	s__Klebsiella pneumoniae	GCF_000742135.1	120	120_1	F6MY_bin.94.fa	F68D_bin.40.fa	70.33	2.59	693
F68G_bin.82	d__Bacteria	p__Proteobacteria	c__Gammaproteobacteria	o__Enterobacterales	f__Enterobacteriaceae	g__Klebsiella	s__Klebsiella pneumoniae	GCF_000742135.1	120	120_1	F6MY_bin.94.fa	F68G_bin.82.fa	87.66	1.65	693
F68H_bin.33	d__Bacteria	p__Proteobacteria	c__Gammaproteobacteria	o__Enterobacterales	f__Enterobacteriaceae	g__Klebsiella	s__Klebsiella pneumoniae	GCF_000742135.1	120	120_1	F6MY_bin.94.fa	F68H_bin.33.fa	56.54	1.72	693
F68P_bin.44	d__Bacteria	p__Proteobacteria	c__Gammaproteobacteria	o__Enterobacterales	f__Enterobacteriaceae	g__Klebsiella	s__Klebsiella pneumoniae	GCF_000742135.1	120	120_1	F6MY_bin.94.fa	F68P_bin.44.fa	98.01	1.6	693
F68T_bin.62	d__Bacteria	p__Proteobacteria	c__Gammaproteobacteria	o__Enterobacterales	f__Enterobacteriaceae	g__Klebsiella	s__Klebsiella pneumoniae	GCF_000742135.1	120	120_1	F6MY_bin.94.fa	F68T_bin.62.fa	96.58	0.75	693
F6D7_bin.20	d__Bacteria	p__Proteobacteria	c__Gammaproteobacteria	o__Enterobacterales	f__Enterobacteriaceae	g__Klebsiella	s__Klebsiella pneumoniae	GCF_000742135.1	120	120_1	F6MY_bin.94.fa	F6D7_bin.20.fa	52.76	0.86	693
F6DG_bin.38	d__Bacteria	p__Proteobacteria	c__Gammaproteobacteria	o__Enterobacterales	f__Enterobacteriaceae	g__Klebsiella	s__Klebsiella pneumoniae	GCF_000742135.1	120	120_1	F6MY_bin.94.fa	F6DG_bin.38.fa	79.46	4.14	693

Table S3. Taxonomic classifiation of the 2,421 detected MAGs

	Domain	Phylum	Class	Order	Family	Genus	Species	RefGenome	primary_cluster	secondary_cluster	repr_genome	genome	CheckM_completeness	CheckM_contamination	
F6MY_bin.94	d__Bacteria	p__Proteobacteria	c__Gammaproteobacteria	o__Enterobacterales	f__Enterobacteriaceae	g__Klebsiella	s__Klebsiella pneumoniae	GCF_000742135.1	120	120_1	F6MY_bin.94.fa	F6MY_bin.94.fa	98.05	0.23	693
F68T_bin.7	d__Bacteria	p__Proteobacteria	c__Gammaproteobacteria	o__Enterobacterales	f__Enterobacteriaceae	g__Proteus	s__Proteus mirabilis	GCF_000160755.1	128	128_1	F6D9_bin.39.fa	F68T_bin.7.fa	96.27	0.54	693
F6D9_bin.39	d__Bacteria	p__Proteobacteria	c__Gammaproteobacteria	o__Enterobacterales	f__Enterobacteriaceae	g__Proteus	s__Proteus mirabilis	GCF_000160755.1	128	128_1	F6D9_bin.39.fa	F6D9_bin.39.fa	98.92	0.27	693
F6D9_bin.5	d__Bacteria	p__Proteobacteria	c__Gammaproteobacteria	o__Enterobacterales	f__Enterobacteriaceae	g__Providencia	s__Providencia alcalifaciens_A	GCF_000527275.1	129	129_0	F6D9_bin.5.fa	F6D9_bin.5.fa	97.54	0.09	693
F68T_bin.73	d__Bacteria	p__Proteobacteria	c__Gammaproteobacteria	o__Enterobacterales	f__Pasteurellaceae	g__Haemophilus_D	s__Haemophilus_D parainfluenzae	GCF_000191405.1	149	149_0	F68T_bin.73.fa	F68T_bin.73.fa	96.55	1.02	693
F6D7_bin.26	d__Bacteria	p__Proteobacteria	c__Gammaproteobacteria	o__Enterobacterales	f__Succinivibrionaceae	g__Succinivibrio	s__	N/A	144	144_0	F6D7_bin.26.fa	F6D7_bin.26.fa	98.81	0.59	694
F68J_bin.3	d__Bacteria	p__Proteobacteria	c__Gammaproteobacteria	o__Enterobacterales	f__Succinivibrionaceae	g__Succinivibrio	s__Succinivibrio sp000431835	GCA_000431835.1	143	143_3	F68J_bin.3.fa	F68J_bin.3.fa	98.82	0.29	694

Table S3. Taxonomic classifiation of the 2,421 detected MAGs

	Domain	Phylum	Class	Order	Family	Genus	Species	RefGenome	primary_cluster	secondary_cluster	repr_genome	genome	CheckM_completeness	CheckM_contamination	
F68N_bin.32	d__Bacteria	p__Proteobacteria	c__Gammaproteobacteria	o__Enterobacterales	f__Succinivibrionaceae	g__Succinivibrio	s__Succinivibrio sp000431835	GCA_000431835.1	143	143_1	F68N_bin.32.fa	F68N_bin.32.fa	98.24	0.29	694
F6D7_bin.94	d__Bacteria	p__Proteobacteria	c__Gammaproteobacteria	o__Enterobacterales	f__Succinivibrionaceae	g__Succinivibrio	s__Succinivibrio sp000431835	GCA_000431835.1	143	143_2	F6D7_bin.94.fa	F6D7_bin.94.fa	99.08	0.07	694
F68K_bin.24	d__Bacteria	p__Spirochaetota	c__Brachyspirae	o__Brachyspirales	f__Brachyspiraceae	g__Brachyspira	s__Brachyspira pilosicoli	GCF_000325665.1	146	146_0	F68K_bin.24.fa	F68K_bin.24.fa	99.2	0	694
F6DB_bin.10	d__Bacteria	p__Spirochaetota	c__Spirochaetia	o__Sphaerochaetales	f__Sphaerochaetaceae	g__RUG023	s__	N/A	13	13_0	F6DB_bin.10.fa	F6DB_bin.10.fa	97.63	3.45	695
F68U_bin.20	d__Bacteria	p__Spirochaetota	c__Spirochaetia	o__Sphaerochaetales	f__Sphaerochaetaceae	g__UBA5920	s__	N/A	20	20_0	F68U_bin.20.fa	F68U_bin.20.fa	95.33	0	696
F68J_bin.13	d__Bacteria	p__Spirochaetota	c__Spirochaetia	o__Treponematales	f__Treponemataceae	g__Treponema_D	s__	N/A	662	662_1	F68J_bin.13.fa	F68J_bin.13.fa	82.59	3.11	697
F6DG_bin.77	d__Bacteria	p__Spirochaetota	c__Spirochaetia	o__Treponematales	f__Treponemataceae	g__Treponema_D	s__	N/A	662	662_2	F6DG_bin.77.fa	F6DG_bin.77.fa	97.44	1.63	698
F68J_bin.18	d__Bacteria	p__Spirochaetota	c__Spirochaetia	o__Treponematales	f__Treponemataceae	g__Treponema_D	s__Treponema_D berlinense	GCF_900167025.1	260	260_0	F68J_bin.18.fa	F68J_bin.18.fa	74.55	0	698

Table S3. Taxonomic classifiation of the 2,421 detected MAGs

	Domain	Phylum	Class	Order	Family	Genus	Species	RefGenome	primary_cluster	secondary_cluster	repr_genome	genome	CheckM_completeness	CheckM_contamination	
F68D_bin.90	d__Bacteria	p__Spirochaetota	c__Spirochaetia	o__Treponematales	f__Treponemataceae	g__Treponema_D	s__Treponema_D succinifaciens	GCF_000195275.1	662	662_3	F68D_bin.90.fa	F68D_bin.90.fa	100	0.7	698
F6D9_bin.15	d__Bacteria	p__Spirochaetota	c__Spirochaetia	o__Treponematales	f__Treponemataceae	g__Treponema_D	s__Treponema_D succinifaciens	GCF_000195275.1	662	662_4	F6D9_bin.15.fa	F6D9_bin.15.fa	100	0.7	698
F68H_bin.35	d__Bacteria	p__Synergistota	c__Synergistia	o__Synergistales	f__Dethiosulfovibrionaceae	g__Pyramidobacter	s__Pyramidobacter piscolens	GCF_000177335.1	84	84_1	F68M_bin.68.fa	F68H_bin.35.fa	86.29	2.82	698
F68M_bin.68	d__Bacteria	p__Synergistota	c__Synergistia	o__Synergistales	f__Dethiosulfovibrionaceae	g__Pyramidobacter	s__Pyramidobacter piscolens	GCF_000177335.1	84	84_1	F68M_bin.68.fa	F68M_bin.68.fa	91.53	0	698
F6D8_bin.98	d__Bacteria	p__Synergistota	c__Synergistia	o__Synergistales	f__Dethiosulfovibrionaceae	g__Pyramidobacter	s__Pyramidobacter piscolens	GCF_000177335.1	84	84_3	F6D8_bin.98.fa	F6D8_bin.98.fa	58.32	1.85	698
F68N_bin.9	d__Bacteria	p__Synergistota	c__Synergistia	o__Synergistales	f__Dethiosulfovibrionaceae	g__Pyramidobacter	s__Pyramidobacter piscolens	GCF_000177335.1	84	84_2	F6D9_bin.79.fa	F68N_bin.9.fa	91.53	0	698

Table S3. Taxonomic classifiation of the 2,421 detected MAGs

	Domain	Phylum	Class	Order	Family	Genus	Species	RefGenome	primary_cluster	secondary_cluster	repr_genome	genome	CheckM_completeness	CheckM_contamination	
F6D9_bin.79	d__Bacteria	p__Synergistota	c__Synergistia	o__Synergistales	f__Dethiosulfovibrionaceae	g__Pyramidobacter	s__Pyramidobacter piscolens	GCF_000177335.1	84	84_2	F6D9_bin.79.fa	F6D9_bin.79.fa	100	0	698
F6DB_bin.63	d__Bacteria	p__Synergistota	c__Synergistia	o__Synergistales	f__Dethiosulfovibrionaceae	g__Pyramidobacter	s__Pyramidobacter piscolens	GCF_000177335.1	84	84_2	F6D9_bin.79.fa	F6DB_bin.63.fa	94.92	0	698
F68D_bin.9	d__Bacteria	p__Synergistota	c__Synergistia	o__Synergistales	f__Synergistaceae	g__An23	s__	N/A	404	404_7	F68D_bin.9.fa	F68D_bin.9.fa	98.31	0	699
F68F_bin.32	d__Bacteria	p__Synergistota	c__Synergistia	o__Synergistales	f__Synergistaceae	g__An23	s__	N/A	404	404_5	F68F_bin.32.fa	F68F_bin.32.fa	81.76	4.24	700
F68H_bin.76	d__Bacteria	p__Synergistota	c__Synergistia	o__Synergistales	f__Synergistaceae	g__An23	s__	N/A	404	404_6	F68H_bin.76.fa	F68H_bin.76.fa	57.38	0	701
F68M_bin.79	d__Bacteria	p__Synergistota	c__Synergistia	o__Synergistales	f__Synergistaceae	g__An23	s__	N/A	404	404_1	F68M_bin.79.fa	F68M_bin.79.fa	75.27	0	702
F68R_bin.128	d__Bacteria	p__Synergistota	c__Synergistia	o__Synergistales	f__Synergistaceae	g__An23	s__	N/A	404	404_3	F68R_bin.128.fa	F68R_bin.128.fa	66.52	1.69	703
F68U_bin.79	d__Bacteria	p__Synergistota	c__Synergistia	o__Synergistales	f__Synergistaceae	g__An23	s__	N/A	404	404_4	F68U_bin.79.fa	F68U_bin.79.fa	99.69	0.15	704
F6D8_bin.71	d__Bacteria	p__Synergistota	c__Synergistia	o__Synergistales	f__Synergistaceae	g__An23	s__	N/A	404	404_2	F6D8_bin.71.fa	F6D8_bin.71.fa	81.9	1.85	705

Table S3. Taxonomic classifiation of the 2,421 detected MAGs

	Domain	Phylum	Class	Order	Family	Genus	Species	RefGenome	primary_cluster	secondary_cluster	repr_genome	genome	CheckM_completeness	CheckM_contamination	
F6DB_bin.46	d__Bacteria	p__Synergistota	c__Synergistia	o__Synergistales	f__Synergistaceae	g__Cloacibacillus	s__Cloacibacillus evryensis	GCF_000585335.1	402	402_0	F6DB_bin.46.fa	F6DB_bin.46.fa	77.31	1.72	705
F68M_bin.69	d__Bacteria	p__Synergistota	c__Synergistia	o__Synergistales	f__Synergistaceae	g__Cloacibacillus	s__Cloacibacillus porcorum	GCF_001701045.1	401	401_1	F68V_bin.109.fa	F68M_bin.69.fa	86.21	1.88	705
F68V_bin.109	d__Bacteria	p__Synergistota	c__Synergistia	o__Synergistales	f__Synergistaceae	g__Cloacibacillus	s__Cloacibacillus porcorum	GCF_001701045.1	401	401_1	F68V_bin.109.fa	F68V_bin.109.fa	98.28	0	705
F6DB_bin.13	d__Bacteria	p__Synergistota	c__Synergistia	o__Synergistales	f__Synergistaceae	g__Cloacibacillus	s__Cloacibacillus porcorum	GCF_001701045.1	401	401_2	F6DB_bin.13.fa	F6DB_bin.13.fa	99.14	1.88	705
F68M_bin.78	d__Bacteria	p__Synergistota	c__Synergistia	o__Synergistales	f__Synergistaceae	g__Synergistes	s__Synergistes jonesii	GCF_000712295.1	403	403_0	F68M_bin.78.fa	F68M_bin.78.fa	100	0	705
F68U_bin.73	d__Bacteria	p__Verrucomicrobiota	c__Lentisphaeria	o__UBA1407	f__UBA1407	g__UBA1724	s__	N/A	400	400_1	F68W_bin.93.fa	F68U_bin.73.fa	89.19	0.68	706
F68W_bin.93	d__Bacteria	p__Verrucomicrobiota	c__Lentisphaeria	o__UBA1407	f__UBA1407	g__UBA1724	s__	N/A	400	400_1	F68W_bin.93.fa	F68W_bin.93.fa	89.19	0.68	707

Table S3. Taxonomic classifiation of the 2,421 detected MAGs

	Domain	Phylum	Class	Order	Family	Genus	Species	RefGenome	primary_cluster	secondary_cluster	repr_genome	genome	CheckM_completeness	CheckM_contamination	
F6DH_bin.58	d__Bacteria	p__Verrucomicrobiota	c__Lentisphaeria	o__UBA1407	f__UBA1407	g__UBA1724	s__	N/A	400	400_1	F68W_bin.93.fa	F6DH_bin.58.fa	89.19	0.68	708
F6MX_bin.29	d__Bacteria	p__Verrucomicrobiota	c__Lentisphaeria	o__Victivallales	f__UBA1829	g__	s__	N/A	142	142_0	F6MX_bin.29.fa	F6MX_bin.29.fa	84.6	2.36	709
F68R_bin.104	d__Bacteria	p__Verrucomicrobiota	c__Lentisphaeria	o__Victivallales	f__UBA1829	g__UBA11452	s__UBA11452 sp003526375	GCA_003526375.1	138	138_1	F6DA_bin.75.fa	F68R_bin.104.fa	84.99	3.38	709
F6DA_bin.75	d__Bacteria	p__Verrucomicrobiota	c__Lentisphaeria	o__Victivallales	f__UBA1829	g__UBA11452	s__UBA11452 sp003526375	GCA_003526375.1	138	138_1	F6DA_bin.75.fa	F6DA_bin.75.fa	93.89	3.6	709
F6DH_bin.39	d__Bacteria	p__Verrucomicrobiota	c__Lentisphaeria	o__Victivallales	f__UBA1829	g__UBA11452	s__UBA11452 sp003526375	GCA_003526375.1	138	138_2	F6DH_bin.39.fa	F6DH_bin.39.fa	81.7	3.32	709
F68J_bin.8	d__Bacteria	p__Verrucomicrobiota	c__Lentisphaeria	o__Victivallales	f__UBA1829	g__UBA1829	s__	N/A	140	140_2	F68J_bin.8.fa	F68J_bin.8.fa	82.29	0.85	710
F68W_bin.32	d__Bacteria	p__Verrucomicrobiota	c__Lentisphaeria	o__Victivallales	f__UBA1829	g__UBA1829	s__	N/A	140	140_1	F6DH_bin.40.fa	F68W_bin.32.fa	79.34	2.03	711
F6DH_bin.40	d__Bacteria	p__Verrucomicrobiota	c__Lentisphaeria	o__Victivallales	f__UBA1829	g__UBA1829	s__	N/A	140	140_1	F6DH_bin.40.fa	F6DH_bin.40.fa	88.31	1.69	712
F6D7_bin.81	d__Bacteria	p__Verrucomicrobiota	c__Lentisphaeria	o__Victivallales	f__UBA1829	g__UBA1829	s__	N/A	141	141_0	F6D7_bin.81.fa	F6D7_bin.81.fa	93.89	2.48	713

Table S3. Taxonomic classifiation of the 2,421 detected MAGs

	Domain	Phylum	Class	Order	Family	Genus	Species	RefGenome	primary_cluster	secondary_cluster	repr_genome	genome	CheckM_completeness	CheckM_contamination	
F68D_bin.80	d__Bacteria	p__Verrucomicrobiota	c__Lentisphaeria	o__Victivallales	f__Victivallaceae	g__	s__	N/A	130	130_1	F68D_bin.80.fa	F68D_bin.80.fa	92.84	1.35	714
F68F_bin.116	d__Bacteria	p__Verrucomicrobiota	c__Lentisphaeria	o__Victivallales	f__Victivallaceae	g__	s__	N/A	130	130_1	F68D_bin.80.fa	F68F_bin.116.fa	56.82	2.03	715
F68J_bin.37	d__Bacteria	p__Verrucomicrobiota	c__Lentisphaeria	o__Victivallales	f__Victivallaceae	g__	s__	N/A	131	131_2	F68J_bin.37.fa	F68J_bin.37.fa	90.51	2.93	716
F68C_bin.33	d__Bacteria	p__Verrucomicrobiota	c__Lentisphaeria	o__Victivallales	f__Victivallaceae	g__	s__	N/A	131	131_1	F6DB_bin.87.fa	F68C_bin.33.fa	83.93	4.39	717
F68U_bin.114	d__Bacteria	p__Verrucomicrobiota	c__Lentisphaeria	o__Victivallales	f__Victivallaceae	g__	s__	N/A	131	131_1	F6DB_bin.87.fa	F68U_bin.114.fa	82.78	1.35	718
F6DB_bin.87	d__Bacteria	p__Verrucomicrobiota	c__Lentisphaeria	o__Victivallales	f__Victivallaceae	g__	s__	N/A	131	131_1	F6DB_bin.87.fa	F6DB_bin.87.fa	93.89	2.03	719
F6MX_bin.7	d__Bacteria	p__Verrucomicrobiota	c__Lentisphaeria	o__Victivallales	f__Victivallaceae	g__	s__	N/A	131	131_1	F6DB_bin.87.fa	F6MX_bin.7.fa	92.54	2.03	720
F6MX_bin.103	d__Bacteria	p__Verrucomicrobiota	c__Lentisphaeria	o__Victivallales	f__Victivallaceae	g__	s__	N/A	136	136_0	F6MX_bin.103.fa	F6MX_bin.103.fa	86.44	3.58	721
F6MX_bin.133	d__Bacteria	p__Verrucomicrobiota	c__Lentisphaeria	o__Victivallales	f__Victivallaceae	g__	s__	N/A	428	428_0	F6MX_bin.133.fa	F6MX_bin.133.fa	65.48	0.68	722
F68D_bin.86	d__Bacteria	p__Verrucomicrobiota	c__Lentisphaeria	o__Victivallales	f__Victivallaceae	g__Victivallis	s__	N/A	134	134_1	F68D_bin.86.fa	F68D_bin.86.fa	93.24	3.94	723

Table S3. Taxonomic classifiation of the 2,421 detected MAGs

	Domain	Phylum	Class	Order	Family	Genus	Species	RefGenome	primary_cluster	secondary_cluster	repr_genome	genome	CheckM_completeness	CheckM_contamination	
F6DB_bin.105	d__Bacteria	p__Verrucomicrobiota	c__Lentisphaeria	o__Victivallales	f__Victivallaceae	g__Victivallis	s__	N/A	135	135_0	F6DB_bin.105.fa	F6DB_bin.105.fa	93.24	2.36	724
F6DB_bin.93	d__Bacteria	p__Verrucomicrobiota	c__Lentisphaeria	o__Victivallales	f__Victivallaceae	g__Victivallis	s__	N/A	137	137_0	F6DB_bin.93.fa	F6DB_bin.93.fa	92.23	2.73	725
F6MX_bin.128	d__Bacteria	p__Verrucomicrobiota	c__Lentisphaeria	o__Victivallales	f__Victivallaceae	g__Victivallis	s__	N/A	134	134_2	F6MX_bin.128.fa	F6MX_bin.128.fa	92.49	2.4	726
F68R_bin.119	d__Bacteria	p__Verrucomicrobiota	c__Lentisphaeria	o__Victivallales	f__Victivallaceae	g__Victivallis	s__Victivallis sp002998355	GCF_002998355.1	132	132_1	F6DH_bin.10.fa	F68R_bin.119.fa	92.84	3.42	726
F68W_bin.81	d__Bacteria	p__Verrucomicrobiota	c__Lentisphaeria	o__Victivallales	f__Victivallaceae	g__Victivallis	s__Victivallis sp002998355	GCF_002998355.1	132	132_1	F6DH_bin.10.fa	F68W_bin.81.fa	93.58	2.07	726
F6D9_bin.57	d__Bacteria	p__Verrucomicrobiota	c__Lentisphaeria	o__Victivallales	f__Victivallaceae	g__Victivallis	s__Victivallis sp002998355	GCF_002998355.1	132	132_1	F6DH_bin.10.fa	F6D9_bin.57.fa	93.92	2.7	726
F6DH_bin.10	d__Bacteria	p__Verrucomicrobiota	c__Lentisphaeria	o__Victivallales	f__Victivallaceae	g__Victivallis	s__Victivallis sp002998355	GCF_002998355.1	132	132_1	F6DH_bin.10.fa	F6DH_bin.10.fa	94.26	2.03	726
F6MY_bin.42	d__Bacteria	p__Verrucomicrobiota	c__Lentisphaeria	o__Victivallales	f__Victivallaceae	g__Victivallis	s__Victivallis sp002998355	GCF_002998355.1	132	132_1	F6DH_bin.10.fa	F6MY_bin.42.fa	92.57	2.03	726

Table S3. Taxonomic classifiation of the 2,421 detected MAGs

	Domain	Phylum	Class	Order	Family	Genus	Species	RefGenome	primary_cluster	secondary_cluster	repr_genome	genome	CheckM_completeness	CheckM_contamination	
F68G_bin.18	d__Bacteria	p__Verrucomicrobiota	c__Lentisphaeria	o__Victivallales	f__Victivallaceae	g__Victivallis	s__Victivallis vadensis	GCA_003096415.1	133	133_2	F68G_bin.18.fa	F68G_bin.18.fa	89.86	4.08	726
F68C_bin.65	d__Bacteria	p__Verrucomicrobiota	c__Lentisphaeria	o__Victivallales	f__Victivallaceae	g__Victivallis	s__Victivallis vadensis	GCA_003096415.1	133	133_1	F68J_bin.26.fa	F68C_bin.65.fa	56.74	2.36	726
F68J_bin.26	d__Bacteria	p__Verrucomicrobiota	c__Lentisphaeria	o__Victivallales	f__Victivallaceae	g__Victivallis	s__Victivallis vadensis	GCA_003096415.1	133	133_1	F68J_bin.26.fa	F68J_bin.26.fa	80.16	2.93	726
F6D7_bin.23	d__Bacteria	p__Verrucomicrobiota	c__Lentisphaeria	o__Victivallales	f__Victivallaceae	g__Victivallis	s__Victivallis vadensis	GCA_003096415.1	133	133_1	F68J_bin.26.fa	F6D7_bin.23.fa	74.9	3.76	726
F68V_bin.113	d__Bacteria	p__Verrucomicrobiota	c__Lentisphaeria	o__Victivallales	f__Victivallaceae	g__Victivallis	s__Victivallis vadensis	GCA_003096415.1	133	133_3	F68V_bin.113.fa	F68V_bin.113.fa	93.92	2.93	726
F6DA_bin.20	d__Bacteria	p__Verrucomicrobiota	c__Lentisphaeria	o__Victivallales	f__Victivallaceae	g__Victivallis	s__Victivallis vadensis	GCA_003096415.1	133	133_4	F6DA_bin.20.fa	F6DA_bin.20.fa	66.18	2.4	726
F68M_bin.22	d__Bacteria	p__Verrucomicrobiota	c__Verrucomicrobiae	o__Opitutales	f__CAG-312	g__	s__	N/A	502	502_1	F68M_bin.22.fa	F68M_bin.22.fa	96.58	1.35	727
F68V_bin.1	d__Bacteria	p__Verrucomicrobiota	c__Verrucomicrobiae	o__Opitutales	f__CAG-312	g__	s__	N/A	502	502_1	F68M_bin.22.fa	F68V_bin.1.fa	90.61	4.05	728

Table S3. Taxonomic classifiation of the 2,421 detected MAGs

	Domain	Phylum	Class	Order	Family	Genus	Species	RefGenome	primary_cluster	secondary_cluster	repr_genome	genome	CheckM_completeness	CheckM_contamination	
F6DB_bin.114	d__Bacteria	p__Verrucomicrobiota	c__Verrucomicrobiae	o__Opitutales	f__CAG-312	g__	s__	N/A	505	505_0	F6DB_bin.114.fa	F6DB_bin.114.fa	93.92	1.35	729
F68M_bin.37	d__Bacteria	p__Verrucomicrobiota	c__Verrucomicrobiae	o__Opitutales	f__CAG-312	g__CAG-312	s__	N/A	503	503_1	F68M_bin.37.fa	F68M_bin.37.fa	91.22	0.68	730
F6MX_bin.55	d__Bacteria	p__Verrucomicrobiota	c__Verrucomicrobiae	o__Opitutales	f__CAG-312	g__CAG-312	s__	N/A	503	503_1	F68M_bin.37.fa	F6MX_bin.55.fa	77.34	0.68	731
F6D7_bin.27	d__Bacteria	p__Verrucomicrobiota	c__Verrucomicrobiae	o__Opitutales	f__CAG-312	g__CAG-312	s__	N/A	506	506_0	F6D7_bin.27.fa	F6D7_bin.27.fa	94.59	0.68	732
F68D_bin.52	d__Bacteria	p__Verrucomicrobiota	c__Verrucomicrobiae	o__Opitutales	f__CAG-312	g__CAG-312	s__CAG-312 sp000438015	GCA_000438015.1	504	504_1	F6D7_bin.22.fa	F68D_bin.52.fa	93.2	2.03	732
F68H_bin.8	d__Bacteria	p__Verrucomicrobiota	c__Verrucomicrobiae	o__Opitutales	f__CAG-312	g__CAG-312	s__CAG-312 sp000438015	GCA_000438015.1	504	504_1	F6D7_bin.22.fa	F68H_bin.8.fa	87.53	4.05	732
F6D7_bin.22	d__Bacteria	p__Verrucomicrobiota	c__Verrucomicrobiae	o__Opitutales	f__CAG-312	g__CAG-312	s__CAG-312 sp000438015	GCA_000438015.1	504	504_1	F6D7_bin.22.fa	F6D7_bin.22.fa	93.88	1.35	732
F6MX_bin.84	d__Bacteria	p__Verrucomicrobiota	c__Verrucomicrobiae	o__Opitutales	f__CAG-312	g__CAG-312	s__CAG-312 sp000438015	GCA_000438015.1	504	504_2	F6MX_bin.84.fa	F6MX_bin.84.fa	94.55	1.35	732
F6DE_bin.37	d__Bacteria	p__Verrucomicrobiota	c__Verrucomicrobiae	o__Verrucomicrobiales	f__Akkermansiaceae	g__Akkermansia	s__	N/A	83	83_1	F6MX_bin.117.fa	F6DE_bin.37.fa	97.96	1.36	733

Table S3. Taxonomic classifiation of the 2,421 detected MAGs

	Domain	Phylum	Class	Order	Family	Genus	Species	RefGenome	primary_cluster	secondary_cluster	repr_genome	genome	CheckM_completeness	CheckM_contamination	
F6MX_bin.117	d__Bacteria	p__Verrucomicrobiota	c__Verrucomicrobiae	o__Verrucomicrobiales	f__Akkermansiaceae	g__Akkermansia	s__	N/A	83	83_1	F6MX_bin.117.fa	F6MX_bin.117.fa	97.96	1.36	734
F68D_bin.93	d__Bacteria	p__Verrucomicrobiota	c__Verrucomicrobiae	o__Verrucomicrobiales	f__Akkermansiaceae	g__Akkermansia	s__Akkermansia muciniphila	GCF_000020225.1	82	82_1	F68D_bin.93.fa	F68D_bin.93.fa	97.96	0	734
F68E_bin.21	d__Bacteria	p__Verrucomicrobiota	c__Verrucomicrobiae	o__Verrucomicrobiales	f__Akkermansiaceae	g__Akkermansia	s__Akkermansia muciniphila	GCF_000020225.1	82	82_3	F68E_bin.21.fa	F68E_bin.21.fa	97.96	0	734
F6D7_bin.14	d__Bacteria	p__Verrucomicrobiota	c__Verrucomicrobiae	o__Verrucomicrobiales	f__Akkermansiaceae	g__Akkermansia	s__Akkermansia muciniphila	GCF_000020225.1	82	82_2	F6D7_bin.14.fa	F6D7_bin.14.fa	97.96	0	734
F68M_bin.41	d__Bacteria	p__Verrucomicrobiota	c__Verrucomicrobiae	o__Verrucomicrobiales	f__Akkermansiaceae	g__Akkermansia	s__Akkermansia muciniphila_B	GCF_002885095.1	81	81_1	F68M_bin.41.fa	F68M_bin.41.fa	95.58	0.68	734
F68V_bin.22	d__Bacteria	p__Verrucomicrobiota	c__Verrucomicrobiae	o__Verrucomicrobiales	f__Akkermansiaceae	g__Akkermansia	s__Akkermansia muciniphila_B	GCF_002885095.1	81	81_2	F68V_bin.22.fa	F68V_bin.22.fa	97.28	0	734

Table S4. Isolated strains by culturomics

bacteriaId	*phylum*	*class*	*order*	*family*	*genus*	*species*
1	*Actinobacteria*	*Actinobacteria*	*Bifidobacteriales*	*Bifidobacteriaceae*	*Bifidobacterium*	*Bifidobacterium*
2	*Actinobacteria*	*Actinobacteria*	*Bifidobacteriales*	*Bifidobacteriaceae*	*Bifidobacterium*	*Bifidobacterium*
3	*Actinobacteria*	*Actinobacteria*	*Bifidobacteriales*	*Bifidobacteriaceae*	*Bifidobacterium*	*Bifidobacterium*
4	*Actinobacteria*	*Actinobacteria*	*Bifidobacteriales*	*Bifidobacteriaceae*	*Bifidobacterium*	*Bifidobacterium*
5	*Actinobacteria*	*Actinobacteria*	*Bifidobacteriales*	*Bifidobacteriaceae*	*Bifidobacterium*	*Bifidobacterium*
6	*Actinobacteria*	*Actinobacteria*	*Bifidobacteriales*	*Bifidobacteriaceae*	*Bifidobacterium*	*Bifidobacterium*
7	*Actinobacteria*	*Actinobacteria*	*Bifidobacteriales*	*Bifidobacteriaceae*	*Bifidobacterium*	*Bifidobacterium*
8	*Actinobacteria*	*Actinobacteria*	*Bifidobacteriales*	*Bifidobacteriaceae*	*Bifidobacterium*	*Bifidobacterium*
9	*Actinobacteria*	*Actinobacteria*	*Bifidobacteriales*	*Bifidobacteriaceae*	*Bifidobacterium*	*Bifidobacterium*
10	*Actinobacteria*	*Actinobacteria*	*Bifidobacteriales*	*Bifidobacteriaceae*	*Bifidobacterium*	*Bifidobacterium*
11	*Actinobacteria*	*Actinobacteria*	*Bifidobacteriales*	*Bifidobacteriaceae*	*Bifidobacterium*	*Bifidobacterium*
12	*Actinobacteria*	*Actinobacteria*	*Bifidobacteriales*	*Bifidobacteriaceae*	*Bifidobacterium*	*Bifidobacterium*
13	*Actinobacteria*	*Actinobacteria*	*Bifidobacteriales*	*Bifidobacteriaceae*	*Bifidobacterium*	*Bifidobacterium*
14	*Actinobacteria*	*Actinobacteria*	*Bifidobacteriales*	*Bifidobacteriaceae*	*Bifidobacterium*	*Bifidobacterium*
15	*Actinobacteria*	*Actinobacteria*	*Bifidobacteriales*	*Bifidobacteriaceae*	*Bifidobacterium*	*Bifidobacterium*
16	*Actinobacteria*	*Actinobacteria*	*Bifidobacteriales*	*Bifidobacteriaceae*	*Bifidobacterium*	*Bifidobacterium*
17	*Actinobacteria*	*Actinobacteria*	*Bifidobacteriales*	*Bifidobacteriaceae*	*Bifidobacterium*	*Bifidobacterium*
18	*Actinobacteria*	*Actinobacteria*	*Bifidobacteriales*	*Bifidobacteriaceae*	*Bifidobacterium*	*Bifidobacterium*
19	*Actinobacteria*	*Actinobacteria*	*Bifidobacteriales*	*Bifidobacteriaceae*	*Bifidobacterium*	*Bifidobacterium*
20	*Actinobacteria*	*Actinobacteria*	*Bifidobacteriales*	*Bifidobacteriaceae*	*Bifidobacterium*	*Bifidobacterium*
21	*Actinobacteria*	*Actinobacteria*	*Bifidobacteriales*	*Bifidobacteriaceae*	*Bifidobacterium*	*Bifidobacterium*
22	*Actinobacteria*	*Actinobacteria*	*Bifidobacteriales*	*Bifidobacteriaceae*	*Bifidobacterium*	*Bifidobacterium*
23	*Actinobacteria*	*Actinobacteria*	*Bifidobacteriales*	*Bifidobacteriaceae*	*Bifidobacterium*	*Bifidobacterium*
24	*Actinobacteria*	*Actinobacteria*	*Bifidobacteriales*	*Bifidobacteriaceae*	*Bifidobacterium*	*Bifidobacterium*
25	*Actinobacteria*	*Actinobacteria*	*Bifidobacteriales*	*Bifidobacteriaceae*	*Bifidobacterium*	*Bifidobacterium*
26	*Actinobacteria*	*Actinobacteria*	*Bifidobacteriales*	*Bifidobacteriaceae*	*Bifidobacterium*	*Bifidobacterium*
27	*Actinobacteria*	*Actinobacteria*	*Bifidobacteriales*	*Bifidobacteriaceae*	*Bifidobacterium*	*Bifidobacterium*
28	*Actinobacteria*	*Actinobacteria*	*Bifidobacteriales*	*Bifidobacteriaceae*	*Bifidobacterium*	*Bifidobacterium*

Table S4. Isolated strains by culturomics

bacteriaId	*phylum*	*class*	*order*	*family*	*genus*	*species*
29	*Actinobacteria*	*Actinobacteria*	*Bifidobacteriales*	*Bifidobacteriaceae*	*Bifidobacterium*	*Bifidobacterium*
30	*Actinobacteria*	*Actinobacteria*	*Bifidobacteriales*	*Bifidobacteriaceae*	*Bifidobacterium*	*Bifidobacterium*
31	*Actinobacteria*	*Actinobacteria*	*Bifidobacteriales*	*Bifidobacteriaceae*	*Bifidobacterium*	*Bifidobacterium*
32	*Actinobacteria*	*Actinobacteria*	*Bifidobacteriales*	*Bifidobacteriaceae*	*Bifidobacterium*	*Bifidobacterium*
33	*Actinobacteria*	*Actinobacteria*	*Bifidobacteriales*	*Bifidobacteriaceae*	*Bifidobacterium*	*Bifidobacterium*
34	*Actinobacteria*	*Actinobacteria*	*Bifidobacteriales*	*Bifidobacteriaceae*	*Bifidobacterium*	*Bifidobacterium*
35	*Actinobacteria*	*Actinobacteria*	*Bifidobacteriales*	*Bifidobacteriaceae*	*Bifidobacterium*	*Bifidobacterium*
36	*Actinobacteria*	*Actinobacteria*	*Bifidobacteriales*	*Bifidobacteriaceae*	*Bifidobacterium*	*Bifidobacterium*
37	*Actinobacteria*	*Actinobacteria*	*Bifidobacteriales*	*Bifidobacteriaceae*	*Bifidobacterium*	*Bifidobacterium*
38	*Actinobacteria*	*Actinobacteria*	*Bifidobacteriales*	*Bifidobacteriaceae*	*Bifidobacterium*	*Bifidobacterium*
39	*Actinobacteria*	*Actinobacteria*	*Bifidobacteriales*	*Bifidobacteriaceae*	*Bifidobacterium*	*Bifidobacterium*
40	*Actinobacteria*	*Actinobacteria*	*Bifidobacteriales*	*Bifidobacteriaceae*	*Bifidobacterium*	*Bifidobacterium*
41	*Actinobacteria*	*Actinobacteria*	*Bifidobacteriales*	*Bifidobacteriaceae*	*Bifidobacterium*	*Bifidobacterium*
42	*Actinobacteria*	*Actinobacteria*	*Bifidobacteriales*	*Bifidobacteriaceae*	*Bifidobacterium*	*Bifidobacterium*
43	*Actinobacteria*	*Actinobacteria*	*Bifidobacteriales*	*Bifidobacteriaceae*	*Bifidobacterium*	*Bifidobacterium*
44	*Actinobacteria*	*Actinobacteria*	*Bifidobacteriales*	*Bifidobacteriaceae*	*Bifidobacterium*	*Bifidobacterium*
45	*Actinobacteria*	*Actinobacteria*	*Bifidobacteriales*	*Bifidobacteriaceae*	*Bifidobacterium*	*Bifidobacterium*
46	*Actinobacteria*	*Actinobacteria*	*Bifidobacteriales*	*Bifidobacteriaceae*	*Bifidobacterium*	*Bifidobacterium*
47	*Actinobacteria*	*Actinobacteria*	*Bifidobacteriales*	*Bifidobacteriaceae*	*Bifidobacterium*	*Bifidobacterium*
48	*Actinobacteria*	*Actinobacteria*	*Bifidobacteriales*	*Bifidobacteriaceae*	*Bifidobacterium*	*Bifidobacterium*
49	*Actinobacteria*	*Actinobacteria*	*Bifidobacteriales*	*Bifidobacteriaceae*	*Bifidobacterium*	*Bifidobacterium*
50	*Actinobacteria*	*Actinobacteria*	*Bifidobacteriales*	*Bifidobacteriaceae*	*Bifidobacterium*	*Bifidobacterium*
51	*Actinobacteria*	*Actinobacteria*	*Bifidobacteriales*	*Bifidobacteriaceae*	*Bifidobacterium*	*Bifidobacterium*
52	*Actinobacteria*	*Actinobacteria*	*Bifidobacteriales*	*Bifidobacteriaceae*	*Bifidobacterium*	*Bifidobacterium*
53	*Actinobacteria*	*Actinobacteria*	*Bifidobacteriales*	*Bifidobacteriaceae*	*Bifidobacterium*	*Bifidobacterium*
54	*Actinobacteria*	*Actinobacteria*	*Bifidobacteriales*	*Bifidobacteriaceae*	*Bifidobacterium*	*Bifidobacterium*
55	*Actinobacteria*	*Actinobacteria*	*Bifidobacteriales*	*Bifidobacteriaceae*	*Bifidobacterium*	*Bifidobacterium*
56	*Actinobacteria*	*Actinobacteria*	*Bifidobacteriales*	*Bifidobacteriaceae*	*Bifidobacterium*	*Bifidobacterium*

Table S4. Isolated strains by culturomics

bacterialId	*phylum*	*class*	*order*	*family*	*genus*	*species*
57	*Actinobacteria*	*Actinobacteria*	*Bifidobacteriales*	*Bifidobacteriaceae*	*Bifidobacterium*	*Bifidobacterium*
58	*Actinobacteria*	*Actinobacteria*	*Bifidobacteriales*	*Bifidobacteriaceae*	*Bifidobacterium*	*Bifidobacterium*
59	*Actinobacteria*	*Actinobacteria*	*Bifidobacteriales*	*Bifidobacteriaceae*	*Bifidobacterium*	*Bifidobacterium*
60	*Actinobacteria*	*Actinobacteria*	*Bifidobacteriales*	*Bifidobacteriaceae*	*Bifidobacterium*	*Bifidobacterium*
61	*Actinobacteria*	*Actinobacteria*	*Bifidobacteriales*	*Bifidobacteriaceae*	*Bifidobacterium*	*Bifidobacterium*
62	*Actinobacteria*	*Actinobacteria*	*Bifidobacteriales*	*Bifidobacteriaceae*	*Bifidobacterium*	*Bifidobacterium*
63	*Actinobacteria*	*Actinobacteria*	*Bifidobacteriales*	*Bifidobacteriaceae*	*Bifidobacterium*	*Bifidobacterium*
64	*Actinobacteria*	*Actinobacteria*	*Bifidobacteriales*	*Bifidobacteriaceae*	*Bifidobacterium*	*Bifidobacterium*
65	*Actinobacteria*	*Actinobacteria*	*Bifidobacteriales*	*Bifidobacteriaceae*	*Bifidobacterium*	*Bifidobacterium*
66	*Actinobacteria*	*Actinobacteria*	*Bifidobacteriales*	*Bifidobacteriaceae*	*Bifidobacterium*	*Bifidobacterium*
67	*Actinobacteria*	*Actinobacteria*	*Bifidobacteriales*	*Bifidobacteriaceae*	*Bifidobacterium*	*Bifidobacterium*
68	*Actinobacteria*	*Actinobacteria*	*Bifidobacteriales*	*Bifidobacteriaceae*	*Bifidobacterium*	*Bifidobacterium*
69	*Actinobacteria*	*Actinobacteria*	*Bifidobacteriales*	*Bifidobacteriaceae*	*Bifidobacterium*	*Bifidobacterium*
70	*Actinobacteria*	*Actinobacteria*	*Bifidobacteriales*	*Bifidobacteriaceae*	*Bifidobacterium*	*Bifidobacterium*
71	*Actinobacteria*	*Actinobacteria*	*Bifidobacteriales*	*Bifidobacteriaceae*	*Bifidobacterium*	*Bifidobacterium*
72	*Actinobacteria*	*Actinobacteria*	*Bifidobacteriales*	*Bifidobacteriaceae*	*Bifidobacterium*	*Bifidobacterium*
73	*Actinobacteria*	*Actinobacteria*	*Bifidobacteriales*	*Bifidobacteriaceae*	*Bifidobacterium*	*Bifidobacterium*
74	*Actinobacteria*	*Actinobacteria*	*Bifidobacteriales*	*Bifidobacteriaceae*	*Bifidobacterium*	*Bifidobacterium*
75	*Actinobacteria*	*Actinobacteria*	*Bifidobacteriales*	*Bifidobacteriaceae*	*Bifidobacterium*	*Bifidobacterium*
76	*Actinobacteria*	*Actinobacteria*	*Bifidobacteriales*	*Bifidobacteriaceae*	*Bifidobacterium*	*Bifidobacterium*
77	*Actinobacteria*	*Actinobacteria*	*Bifidobacteriales*	*Bifidobacteriaceae*	*Bifidobacterium*	*Bifidobacterium*
78	*Actinobacteria*	*Actinobacteria*	*Bifidobacteriales*	*Bifidobacteriaceae*	*Bifidobacterium*	*Bifidobacterium*
79	*Actinobacteria*	*Actinobacteria*	*Bifidobacteriales*	*Bifidobacteriaceae*	*Bifidobacterium*	*Bifidobacterium*
80	*Actinobacteria*	*Actinobacteria*	*Bifidobacteriales*	*Bifidobacteriaceae*	*Bifidobacterium*	*Bifidobacterium*
81	*Actinobacteria*	*Actinobacteria*	*Bifidobacteriales*	*Bifidobacteriaceae*	*Bifidobacterium*	*Bifidobacterium*
82	*Actinobacteria*	*Actinobacteria*	*Bifidobacteriales*	*Bifidobacteriaceae*	*Bifidobacterium*	*Bifidobacterium*
83	*Actinobacteria*	*Actinobacteria*	*Bifidobacteriales*	*Bifidobacteriaceae*	*Bifidobacterium*	*Bifidobacterium*
84	*Actinobacteria*	*Actinobacteria*	*Bifidobacteriales*	*Bifidobacteriaceae*	*Bifidobacterium*	*Bifidobacterium*

Table S4. Isolated strains by culturomics

bacteriaId	*phylum*	*class*	*order*	*family*	*genus*	*species*
85	*Actinobacteria*	*Actinobacteria*	*Bifidobacteriales*	*Bifidobacteriaceae*	*Bifidobacterium*	*Bifidobacterium*
86	*Actinobacteria*	*Actinobacteria*	*Bifidobacteriales*	*Bifidobacteriaceae*	*Bifidobacterium*	*Bifidobacterium*
87	*Actinobacteria*	*Actinobacteria*	*Bifidobacteriales*	*Bifidobacteriaceae*	*Bifidobacterium*	*Bifidobacterium*
88	*Actinobacteria*	*Actinobacteria*	*Bifidobacteriales*	*Bifidobacteriaceae*	*Bifidobacterium*	*Bifidobacterium*
89	*Actinobacteria*	*Actinobacteria*	*Bifidobacteriales*	*Bifidobacteriaceae*	*Bifidobacterium*	*Bifidobacterium*
90	*Actinobacteria*	*Actinobacteria*	*Bifidobacteriales*	*Bifidobacteriaceae*	*Bifidobacterium*	*Bifidobacterium*
91	*Actinobacteria*	*Actinobacteria*	*Bifidobacteriales*	*Bifidobacteriaceae*	*Bifidobacterium*	*Bifidobacterium*
92	*Actinobacteria*	*Actinobacteria*	*Corynebacteriales*	*Corynebacteriaceae*	*Corynebacterium*	*Corynebacterium*
93	*Actinobacteria*	*Actinobacteria*	*Micrococcales*	*Micrococcaceae*	*Kocuria*	*Kocuria kristinae*
94	*Actinobacteria*	*Actinobacteria*	*Micrococcales*	*Micrococcaceae*	*Kocuria*	*Kocuria kristinae*
95	*Actinobacteria*	*Actinobacteria*	*Micrococcales*	*Micrococcaceae*	*Micrococcus*	*Micrococcus*
96	*Actinobacteria*	*Actinobacteria*	*Micrococcales*	*Micrococcaceae*	*Micrococcus*	*Micrococcus*
97	*Actinobacteria*	*Actinobacteria*	*Micrococcales*	*Micrococcaceae*	*Micrococcus*	*Micrococcus*
98	*Actinobacteria*	*Actinobacteria*	*Micrococcales*	*Micrococcaceae*	*Micrococcus*	*Micrococcus*
99	*Actinobacteria*	*Coriobacteriia*	*Coriobacteriales*	*Atopobiaceae*	*Olsenella*	*Olsenella*
100	*Actinobacteria*	*Coriobacteriia*	*Coriobacteriales*	*Atopobiaceae*	*Olsenella*	*Olsenella*
101	*Actinobacteria*	*Coriobacteriia*	*Coriobacteriales*	*Atopobiaceae*	*Olsenella*	*Olsenella*
102	*Actinobacteria*	*Coriobacteriia*	*Coriobacteriales*	*Atopobiaceae*	*Olsenella*	*Olsenella*
103	*Actinobacteria*	*Coriobacteriia*	*Coriobacteriales*	*Atopobiaceae*	*Olsenella*	*Olsenella*
104	*Actinobacteria*	*Coriobacteriia*	*Coriobacteriales*	*Atopobiaceae*	*Olsenella*	*Olsenella*
105	*Actinobacteria*	*Coriobacteriia*	*Coriobacteriales*	*Atopobiaceae*	*Olsenella*	*Olsenella*
106	*Actinobacteria*	*Coriobacteriia*	*Coriobacteriales*	*Atopobiaceae*	*Olsenella*	*Olsenella*
107	*Actinobacteria*	*Coriobacteriia*	*Coriobacteriales*	*Atopobiaceae*	*Olsenella*	*Olsenella*
108	*Actinobacteria*	*Coriobacteriia*	*Coriobacteriales*	*Atopobiaceae*	*Olsenella*	*Olsenella*
109	*Actinobacteria*	*Coriobacteriia*	*Coriobacteriales*	*Atopobiaceae*	*Olsenella*	*Olsenella*
110	*Actinobacteria*	*Coriobacteriia*	*Coriobacteriales*	*Atopobiaceae*	*Olsenella*	*Olsenella*
111	*Actinobacteria*	*Coriobacteriia*	*Coriobacteriales*	*Atopobiaceae*	*Olsenella*	*Olsenella*
112	*Actinobacteria*	*Coriobacteriia*	*Coriobacteriales*	*Coriobacteriaceae*	*Collinsella*	*Collinsella*

Table S4. Isolated strains by culturomics

bacteriaId	*phylum*	*class*	*order*	*family*	*genus*	*species*
113	*Actinobacteria*	*Coriobacteriia*	*Coriobacteriales*	*Coriobacteriaceae*	*Collinsella*	*Collinsella*
114	*Actinobacteria*	*Coriobacteriia*	*Coriobacteriales*	*Coriobacteriaceae*	*Collinsella*	*Collinsella*
115	*Actinobacteria*	*Coriobacteriia*	*Coriobacteriales*	*Coriobacteriaceae*	*Collinsella*	*Collinsella*
116	*Actinobacteria*	*Coriobacteriia*	*Coriobacteriales*	*Coriobacteriaceae*	*Collinsella*	*Collinsella*
117	*Actinobacteria*	*Coriobacteriia*	*Coriobacteriales*	*Coriobacteriaceae*	*Collinsella*	*Collinsella*
118	*Actinobacteria*	*Coriobacteriia*	*Coriobacteriales*	*Coriobacteriaceae*	*Collinsella*	*Collinsella*
119	*Actinobacteria*	*Coriobacteriia*	*Coriobacteriales*	*Coriobacteriaceae*	*Collinsella*	*Collinsella*
120	*Actinobacteria*	*Coriobacteriia*	*Coriobacteriales*	*Coriobacteriaceae*	*Collinsella*	*Collinsella*
121	*Actinobacteria*	*Coriobacteriia*	*Coriobacteriales*	*Coriobacteriaceae*	*Collinsella*	*Collinsella*
122	*Actinobacteria*	*Coriobacteriia*	*Coriobacteriales*	*Coriobacteriaceae*	*Collinsella*	*Collinsella*
123	*Actinobacteria*	*Coriobacteriia*	*Coriobacteriales*	*Coriobacteriaceae*	*Collinsella*	*Collinsella*
124	*Actinobacteria*	*Coriobacteriia*	*Coriobacteriales*	*Coriobacteriaceae*	*Collinsella*	*Collinsella*
125	*Actinobacteria*	*Coriobacteriia*	*Coriobacteriales*	*Coriobacteriaceae*	*Collinsella*	*Collinsella*
126	*Actinobacteria*	*Coriobacteriia*	*Coriobacteriales*	*Coriobacteriaceae*	*Collinsella*	*Collinsella*
127	*Actinobacteria*	*Coriobacteriia*	*Coriobacteriales*	*Coriobacteriaceae*	*Collinsella*	*Collinsella*
128	*Actinobacteria*	*Coriobacteriia*	*Coriobacteriales*	*Coriobacteriaceae*	*Collinsella*	*Collinsella*
129	*Actinobacteria*	*Coriobacteriia*	*Coriobacteriales*	*Coriobacteriaceae*	*Collinsella*	*Collinsella*
130	*Actinobacteria*	*Coriobacteriia*	*Coriobacteriales*	*Coriobacteriaceae*	*Collinsella*	*Collinsella*
131	*Actinobacteria*	*Coriobacteriia*	*Coriobacteriales*	*Coriobacteriaceae*	*Collinsella*	*Collinsella*
132	*Actinobacteria*	*Coriobacteriia*	*Coriobacteriales*	*Coriobacteriaceae*	*Collinsella*	*Collinsella*
133	*Actinobacteria*	*Coriobacteriia*	*Coriobacteriales*	*Coriobacteriaceae*	*Collinsella*	*Collinsella*
134	*Actinobacteria*	*Coriobacteriia*	*Coriobacteriales*	*Coriobacteriaceae*	*Collinsella*	*Collinsella*
135	*Actinobacteria*	*Coriobacteriia*	*Coriobacteriales*	*Coriobacteriaceae*	*Collinsella*	*Collinsella*
136	*Actinobacteria*	*Coriobacteriia*	*Coriobacteriales*	*Coriobacteriaceae*	*Collinsella*	*Collinsella*
137	*Actinobacteria*	*Coriobacteriia*	*Coriobacteriales*	*Coriobacteriaceae*	*Collinsella*	*Collinsella*
138	*Actinobacteria*	*Coriobacteriia*	*Coriobacteriales*	*Coriobacteriaceae*	*Collinsella*	*Collinsella*
139	*Actinobacteria*	*Coriobacteriia*	*Coriobacteriales*	*Coriobacteriaceae*	*Collinsella*	*Collinsella*
140	*Actinobacteria*	*Coriobacteriia*	*Coriobacteriales*	*Coriobacteriaceae*	*Collinsella*	*Collinsella*

Table S4. Isolated strains by culturomics

bacteriaId	*phylum*	*class*	*order*	*family*	*genus*	*species*
141	*Actinobacteria*	*Coriobacteriia*	*Coriobacteriales*	*Coriobacteriaceae*	*Collinsella*	*Collinsella*
142	*Actinobacteria*	*Coriobacteriia*	*Coriobacteriales*	*Coriobacteriaceae*	*Collinsella*	*Collinsella*
143	*Actinobacteria*	*Coriobacteriia*	*Coriobacteriales*	*Coriobacteriaceae*	*Collinsella*	*Collinsella*
144	*Actinobacteria*	*Coriobacteriia*	*Coriobacteriales*	*Coriobacteriaceae*	*Collinsella*	*Collinsella*
145	*Actinobacteria*	*Coriobacteriia*	*Coriobacteriales*	*Coriobacteriaceae*	*Collinsella*	*Collinsella*
146	*Actinobacteria*	*Coriobacteriia*	*Coriobacteriales*	*Coriobacteriaceae*	*Collinsella*	*Collinsella*
147	*Actinobacteria*	*Coriobacteriia*	*Coriobacteriales*	*Coriobacteriaceae*	*Collinsella*	*Collinsella*
148	*Actinobacteria*	*Coriobacteriia*	*Coriobacteriales*	*Coriobacteriaceae*	*Collinsella*	*Collinsella*
149	*Actinobacteria*	*Coriobacteriia*	*Coriobacteriales*	*Coriobacteriaceae*	*Collinsella*	*Collinsella*
150	*Actinobacteria*	*Coriobacteriia*	*Coriobacteriales*	*Coriobacteriaceae*	*Collinsella*	*Collinsella*
151	*Actinobacteria*	*Coriobacteriia*	*Coriobacteriales*	*Coriobacteriaceae*	*Collinsella*	*Collinsella*
152	*Actinobacteria*	*Coriobacteriia*	*Coriobacteriales*	*Coriobacteriaceae*	*Collinsella*	*Collinsella*
153	*Actinobacteria*	*Coriobacteriia*	*Coriobacteriales*	*Coriobacteriaceae*	*Collinsella*	*Collinsella*
154	*Actinobacteria*	*Coriobacteriia*	*Coriobacteriales*	*Coriobacteriaceae*	*Collinsella*	*Collinsella*
155	*Actinobacteria*	*Coriobacteriia*	*Coriobacteriales*	*Coriobacteriaceae*	*Collinsella*	*Collinsella*
156	*Actinobacteria*	*Coriobacteriia*	*Coriobacteriales*	*Coriobacteriaceae*	*Collinsella*	*Collinsella*
157	*Actinobacteria*	*Coriobacteriia*	*Coriobacteriales*	*Coriobacteriaceae*	*Collinsella*	*Collinsella*
158	*Actinobacteria*	*Coriobacteriia*	*Coriobacteriales*	*Coriobacteriaceae*	*Collinsella*	*Collinsella*
159	*Actinobacteria*	*Coriobacteriia*	*Coriobacteriales*	*Coriobacteriaceae*	*Collinsella*	*Collinsella*
160	*Actinobacteria*	*Coriobacteriia*	*Coriobacteriales*	*Coriobacteriaceae*	*Collinsella*	*Collinsella*
161	*Actinobacteria*	*Coriobacteriia*	*Coriobacteriales*	*Coriobacteriaceae*	*Collinsella*	*Collinsella*
162	*Actinobacteria*	*Coriobacteriia*	*Coriobacteriales*	*Coriobacteriaceae*	*Collinsella*	*Collinsella*
163	*Actinobacteria*	*Coriobacteriia*	*Coriobacteriales*	*Coriobacteriaceae*	*Collinsella*	*Collinsella*
164	*Actinobacteria*	*Coriobacteriia*	*Coriobacteriales*	*Coriobacteriaceae*	*Collinsella*	*Collinsella*
165	*Actinobacteria*	*Coriobacteriia*	*Coriobacteriales*	*Coriobacteriaceae*	*Collinsella*	*Collinsella*
166	*Actinobacteria*	*Coriobacteriia*	*Coriobacteriales*	*Coriobacteriaceae*	*Collinsella*	*Collinsella*
167	*Actinobacteria*	*Coriobacteriia*	*Coriobacteriales*	*Coriobacteriaceae*	*Collinsella*	*Collinsella*
168	*Actinobacteria*	*Coriobacteriia*	*Coriobacteriales*	*Coriobacteriaceae*	*Collinsella*	*Collinsella*

Table S4. Isolated strains by culturomics

bacteriaId	*phylum*	*class*	*order*	*family*	*genus*	*species*
169	*Actinobacteria*	*Coriobacteriia*	*Coriobacteriales*	*Coriobacteriaceae*	*Collinsella*	*Collinsella*
170	*Actinobacteria*	*Coriobacteriia*	*Coriobacteriales*	*Coriobacteriaceae*	*Collinsella*	*Collinsella*
171	*Actinobacteria*	*Coriobacteriia*	*Coriobacteriales*	*Coriobacteriaceae*	*Collinsella*	*Collinsella*
172	*Actinobacteria*	*Coriobacteriia*	*Coriobacteriales*	*Coriobacteriaceae*	*Collinsella*	*Collinsella tanakaei*
173	*Actinobacteria*	*Coriobacteriia*	*Coriobacteriales*	*Coriobacteriaceae*	*Collinsella*	*Collinsella tanakaei*
174	*Actinobacteria*	*Coriobacteriia*	*Coriobacteriales*	*Coriobacteriaceae*	*Collinsella*	*Collinsella tanakaei*
175	*Actinobacteria*	*Coriobacteriia*	*Coriobacteriales*	*Coriobacteriaceae*	*Collinsella*	*Collinsella tanakaei*
176	*Actinobacteria*	*Coriobacteriia*	*Coriobacteriales*	*Coriobacteriaceae*	*Collinsella*	*Collinsella tanakaei*
177	*Actinobacteria*	*Coriobacteriia*	*Coriobacteriales*	*Coriobacteriaceae*	*Enorma*	*Enorma timonensis*
178	*Actinobacteria*	*Coriobacteriia*	*Coriobacteriales*	*Coriobacteriaceae*	*Enorma*	*Enorma timonensis*
179	*Actinobacteria*	*Coriobacteriia*	*Coriobacteriales*	*Coriobacteriaceae*	*Enorma*	*Enorma timonensis*
180	*Actinobacteria*	*Coriobacteriia*	*Eggerthellales*	*Eggerthellaceae*	*Eggerthella*	*Eggerthella lenta*
181	*Actinobacteria*	*Coriobacteriia*	*Eggerthellales*	*Eggerthellaceae*	*Eggerthella*	*Eggerthella lenta*
182	*Actinobacteria*	*Coriobacteriia*	*Eggerthellales*	*Eggerthellaceae*	*Eggerthella*	*Eggerthella lenta*
183	*Actinobacteria*	*Coriobacteriia*	*Eggerthellales*	*Eggerthellaceae*	*Eggerthella*	*Eggerthella lenta*
184	*Actinobacteria*	*Coriobacteriia*	*Eggerthellales*	*Eggerthellaceae*	*Eggerthella*	*Eggerthella lenta*
185	*Actinobacteria*	*Coriobacteriia*	*Eggerthellales*	*Eggerthellaceae*	*Eggerthella*	*Eggerthella lenta*
186	*Actinobacteria*	*Coriobacteriia*	*Eggerthellales*	*Eggerthellaceae*	*Eggerthella*	*Eggerthella lenta*
187	*Actinobacteria*	*Coriobacteriia*	*Eggerthellales*	*Eggerthellaceae*	*Eggerthella*	*Eggerthella lenta*
188	*Actinobacteria*	*Coriobacteriia*	*Eggerthellales*	*Eggerthellaceae*	*Eggerthella*	*Eggerthella lenta*
189	*Actinobacteria*	*Coriobacteriia*	*Eggerthellales*	*Eggerthellaceae*	*Eggerthella*	*Eggerthella lenta*
190	*Actinobacteria*	*Coriobacteriia*	*Eggerthellales*	*Eggerthellaceae*	*Eggerthella*	*Eggerthella lenta*
191	*Actinobacteria*	*Coriobacteriia*	*Eggerthellales*	*Eggerthellaceae*	*Eggerthella*	*Eggerthella lenta*
192	*Actinobacteria*	*Coriobacteriia*	*Eggerthellales*	*Eggerthellaceae*	*Eggerthella*	*Eggerthella lenta*
193	*Actinobacteria*	*Coriobacteriia*	*Eggerthellales*	*Eggerthellaceae*	*Eggerthella*	*Eggerthella lenta*
194	*Actinobacteria*	*Coriobacteriia*	*Eggerthellales*	*Eggerthellaceae*	*Eggerthella*	*Eggerthella lenta*
195	*Actinobacteria*	*Coriobacteriia*	*Eggerthellales*	*Eggerthellaceae*	*Eggerthella*	*Eggerthella lenta*
196	*Actinobacteria*	*Coriobacteriia*	*Eggerthellales*	*Eggerthellaceae*	*Eggerthella*	*Eggerthella lenta*

Table S4. Isolated strains by culturomics

bacterialId	*phylum*	*class*	*order*	*family*	*genus*	*species*
197	*Actinobacteria*	*Coriobacteriia*	*Eggerthellales*	*Eggerthellaceae*	*Eggerthella*	*Eggerthella lenta*
198	*Actinobacteria*	*Coriobacteriia*	*Eggerthellales*	*Eggerthellaceae*	*Eggerthella*	*Eggerthella lenta*
199	*Actinobacteria*	*Coriobacteriia*	*Eggerthellales*	*Eggerthellaceae*	*Eggerthella*	*Eggerthella lenta*
200	*Agaricomycotina*	*Tremellomycetes*	*Tremellales*	*Cryptococcaceae*	*Cryptococcus*	*Cryptococcus*
201	*Ascomycota*	*Eurotiomycetes*	*Eurotiales*	*Aspergillaceae*	*Penicillium*	*Penicillium*
202	*Bacteroidetes*	*Bacteroidia*	*Bacteroidales*	*Bacteroidaceae*	*Bacteroides*	*Bacteroides caccae*
203	*Bacteroidetes*	*Bacteroidia*	*Bacteroidales*	*Bacteroidaceae*	*Bacteroides*	*Bacteroides caccae*
204	*Bacteroidetes*	*Bacteroidia*	*Bacteroidales*	*Bacteroidaceae*	*Bacteroides*	*Bacteroides caccae*
205	*Bacteroidetes*	*Bacteroidia*	*Bacteroidales*	*Bacteroidaceae*	*Bacteroides*	*Bacteroides caccae*
206	*Bacteroidetes*	*Bacteroidia*	*Bacteroidales*	*Bacteroidaceae*	*Bacteroides*	*Bacteroides caccae*
207	*Bacteroidetes*	*Bacteroidia*	*Bacteroidales*	*Bacteroidaceae*	*Bacteroides*	*Bacteroides caccae*
208	*Bacteroidetes*	*Bacteroidia*	*Bacteroidales*	*Bacteroidaceae*	*Bacteroides*	*Bacteroides caccae*
209	*Bacteroidetes*	*Bacteroidia*	*Bacteroidales*	*Bacteroidaceae*	*Bacteroides*	*Bacteroides caccae*
210	*Bacteroidetes*	*Bacteroidia*	*Bacteroidales*	*Bacteroidaceae*	*Bacteroides*	*Bacteroides caccae*
211	*Bacteroidetes*	*Bacteroidia*	*Bacteroidales*	*Bacteroidaceae*	*Bacteroides*	*Bacteroides caccae*
212	*Bacteroidetes*	*Bacteroidia*	*Bacteroidales*	*Bacteroidaceae*	*Bacteroides*	*Bacteroides caccae*
213	*Bacteroidetes*	*Bacteroidia*	*Bacteroidales*	*Bacteroidaceae*	*Bacteroides*	*Bacteroides caccae*
214	*Bacteroidetes*	*Bacteroidia*	*Bacteroidales*	*Bacteroidaceae*	*Bacteroides*	*Bacteroides caccae*
215	*Bacteroidetes*	*Bacteroidia*	*Bacteroidales*	*Bacteroidaceae*	*Bacteroides*	*Bacteroides caccae*
216	*Bacteroidetes*	*Bacteroidia*	*Bacteroidales*	*Bacteroidaceae*	*Bacteroides*	*Bacteroides caccae*
217	*Bacteroidetes*	*Bacteroidia*	*Bacteroidales*	*Bacteroidaceae*	*Bacteroides*	*Bacteroides*
218	*Bacteroidetes*	*Bacteroidia*	*Bacteroidales*	*Bacteroidaceae*	*Bacteroides*	*Bacteroides dorei*
219	*Bacteroidetes*	*Bacteroidia*	*Bacteroidales*	*Bacteroidaceae*	*Bacteroides*	*Bacteroides dorei*
220	*Bacteroidetes*	*Bacteroidia*	*Bacteroidales*	*Bacteroidaceae*	*Bacteroides*	*Bacteroides dorei*
221	*Bacteroidetes*	*Bacteroidia*	*Bacteroidales*	*Bacteroidaceae*	*Bacteroides*	*Bacteroides dorei*
222	*Bacteroidetes*	*Bacteroidia*	*Bacteroidales*	*Bacteroidaceae*	*Bacteroides*	*Bacteroides dorei*
223	*Bacteroidetes*	*Bacteroidia*	*Bacteroidales*	*Bacteroidaceae*	*Bacteroides*	*Bacteroides dorei*
224	*Bacteroidetes*	*Bacteroidia*	*Bacteroidales*	*Bacteroidaceae*	*Bacteroides*	*Bacteroides dorei*

Table S4. Isolated strains by culturomics

bacteriaId	*phylum*	*class*	*order*	*family*	*genus*	*species*
225	*Bacteroidetes*	*Bacteroidia*	*Bacteroidales*	*Bacteroidaceae*	*Bacteroides*	*Bacteroides dorei*
226	*Bacteroidetes*	*Bacteroidia*	*Bacteroidales*	*Bacteroidaceae*	*Bacteroides*	*Bacteroides fluxus*
227	*Bacteroidetes*	*Bacteroidia*	*Bacteroidales*	*Bacteroidaceae*	*Bacteroides*	*Bacteroides fragilis*
228	*Bacteroidetes*	*Bacteroidia*	*Bacteroidales*	*Bacteroidaceae*	*Bacteroides*	*Bacteroides fragilis*
229	*Bacteroidetes*	*Bacteroidia*	*Bacteroidales*	*Bacteroidaceae*	*Bacteroides*	*Bacteroides fragilis*
230	*Bacteroidetes*	*Bacteroidia*	*Bacteroidales*	*Bacteroidaceae*	*Bacteroides*	*Bacteroides fragilis*
231	*Bacteroidetes*	*Bacteroidia*	*Bacteroidales*	*Bacteroidaceae*	*Bacteroides*	*Bacteroides fragilis*
232	*Bacteroidetes*	*Bacteroidia*	*Bacteroidales*	*Bacteroidaceae*	*Bacteroides*	*Bacteroides fragilis*
233	*Bacteroidetes*	*Bacteroidia*	*Bacteroidales*	*Bacteroidaceae*	*Bacteroides*	*Bacteroides fragilis*
234	*Bacteroidetes*	*Bacteroidia*	*Bacteroidales*	*Bacteroidaceae*	*Bacteroides*	*Bacteroides fragilis*
235	*Bacteroidetes*	*Bacteroidia*	*Bacteroidales*	*Bacteroidaceae*	*Bacteroides*	*Bacteroides fragilis*
236	*Bacteroidetes*	*Bacteroidia*	*Bacteroidales*	*Bacteroidaceae*	*Bacteroides*	*Bacteroides fragilis*
237	*Bacteroidetes*	*Bacteroidia*	*Bacteroidales*	*Bacteroidaceae*	*Bacteroides*	*Bacteroides fragilis*
238	*Bacteroidetes*	*Bacteroidia*	*Bacteroidales*	*Bacteroidaceae*	*Bacteroides*	*Bacteroides fragilis*
239	*Bacteroidetes*	*Bacteroidia*	*Bacteroidales*	*Bacteroidaceae*	*Bacteroides*	*Bacteroides fragilis*
240	*Bacteroidetes*	*Bacteroidia*	*Bacteroidales*	*Bacteroidaceae*	*Bacteroides*	*Bacteroides fragilis*
241	*Bacteroidetes*	*Bacteroidia*	*Bacteroidales*	*Bacteroidaceae*	*Bacteroides*	*Bacteroides fragilis*
242	*Bacteroidetes*	*Bacteroidia*	*Bacteroidales*	*Bacteroidaceae*	*Bacteroides*	*Bacteroides fragilis*
243	*Bacteroidetes*	*Bacteroidia*	*Bacteroidales*	*Bacteroidaceae*	*Bacteroides*	*Bacteroides fragilis*
244	*Bacteroidetes*	*Bacteroidia*	*Bacteroidales*	*Bacteroidaceae*	*Bacteroides*	*Bacteroides fragilis*
245	*Bacteroidetes*	*Bacteroidia*	*Bacteroidales*	*Bacteroidaceae*	*Bacteroides*	*Bacteroides fragilis*
246	*Bacteroidetes*	*Bacteroidia*	*Bacteroidales*	*Bacteroidaceae*	*Bacteroides*	*Bacteroides fragilis*
247	*Bacteroidetes*	*Bacteroidia*	*Bacteroidales*	*Bacteroidaceae*	*Bacteroides*	*Bacteroides fragilis*
248	*Bacteroidetes*	*Bacteroidia*	*Bacteroidales*	*Bacteroidaceae*	*Bacteroides*	*Bacteroides fragilis*
249	*Bacteroidetes*	*Bacteroidia*	*Bacteroidales*	*Bacteroidaceae*	*Bacteroides*	*Bacteroides fragilis*
250	*Bacteroidetes*	*Bacteroidia*	*Bacteroidales*	*Bacteroidaceae*	*Bacteroides*	*Bacteroides fragilis*
251	*Bacteroidetes*	*Bacteroidia*	*Bacteroidales*	*Bacteroidaceae*	*Bacteroides*	*Bacteroides fragilis*
252	*Bacteroidetes*	*Bacteroidia*	*Bacteroidales*	*Bacteroidaceae*	*Bacteroides*	*Bacteroides fragilis*

Table S4. Isolated strains by culturomics

bacteriaId	*phylum*	*class*	*order*	*family*	*genus*	*species*
253	*Bacteroidetes*	*Bacteroidia*	*Bacteroidales*	*Bacteroidaceae*	*Bacteroides*	*Bacteroides fragilis*
254	*Bacteroidetes*	*Bacteroidia*	*Bacteroidales*	*Bacteroidaceae*	*Bacteroides*	*Bacteroides fragilis*
255	*Bacteroidetes*	*Bacteroidia*	*Bacteroidales*	*Bacteroidaceae*	*Bacteroides*	*Bacteroides fragilis*
256	*Bacteroidetes*	*Bacteroidia*	*Bacteroidales*	*Bacteroidaceae*	*Bacteroides*	*Bacteroides fragilis*
257	*Bacteroidetes*	*Bacteroidia*	*Bacteroidales*	*Bacteroidaceae*	*Bacteroides*	*Bacteroides fragilis*
258	*Bacteroidetes*	*Bacteroidia*	*Bacteroidales*	*Bacteroidaceae*	*Bacteroides*	*Bacteroides fragilis*
259	*Bacteroidetes*	*Bacteroidia*	*Bacteroidales*	*Bacteroidaceae*	*Bacteroides*	*Bacteroides fragilis*
260	*Bacteroidetes*	*Bacteroidia*	*Bacteroidales*	*Bacteroidaceae*	*Bacteroides*	*Bacteroides fragilis*
261	*Bacteroidetes*	*Bacteroidia*	*Bacteroidales*	*Bacteroidaceae*	*Bacteroides*	*Bacteroides fragilis*
262	*Bacteroidetes*	*Bacteroidia*	*Bacteroidales*	*Bacteroidaceae*	*Bacteroides*	*Bacteroides fragilis*
263	*Bacteroidetes*	*Bacteroidia*	*Bacteroidales*	*Bacteroidaceae*	*Bacteroides*	*Bacteroides fragilis*
264	*Bacteroidetes*	*Bacteroidia*	*Bacteroidales*	*Bacteroidaceae*	*Bacteroides*	*Bacteroides fragilis*
265	*Bacteroidetes*	*Bacteroidia*	*Bacteroidales*	*Bacteroidaceae*	*Bacteroides*	*Bacteroides fragilis*
266	*Bacteroidetes*	*Bacteroidia*	*Bacteroidales*	*Bacteroidaceae*	*Bacteroides*	*Bacteroides fragilis*
267	*Bacteroidetes*	*Bacteroidia*	*Bacteroidales*	*Bacteroidaceae*	*Bacteroides*	*Bacteroides fragilis*
268	*Bacteroidetes*	*Bacteroidia*	*Bacteroidales*	*Bacteroidaceae*	*Bacteroides*	*Bacteroides fragilis*
269	*Bacteroidetes*	*Bacteroidia*	*Bacteroidales*	*Bacteroidaceae*	*Bacteroides*	*Bacteroides fragilis*
270	*Bacteroidetes*	*Bacteroidia*	*Bacteroidales*	*Bacteroidaceae*	*Bacteroides*	*Bacteroides fragilis*
271	*Bacteroidetes*	*Bacteroidia*	*Bacteroidales*	*Bacteroidaceae*	*Bacteroides*	*Bacteroides fragilis*
272	*Bacteroidetes*	*Bacteroidia*	*Bacteroidales*	*Bacteroidaceae*	*Bacteroides*	*Bacteroides fragilis*
273	*Bacteroidetes*	*Bacteroidia*	*Bacteroidales*	*Bacteroidaceae*	*Bacteroides*	*Bacteroides fragilis*
274	*Bacteroidetes*	*Bacteroidia*	*Bacteroidales*	*Bacteroidaceae*	*Bacteroides*	*Bacteroides fragilis*
275	*Bacteroidetes*	*Bacteroidia*	*Bacteroidales*	*Bacteroidaceae*	*Bacteroides*	*Bacteroides fragilis*
276	*Bacteroidetes*	*Bacteroidia*	*Bacteroidales*	*Bacteroidaceae*	*Bacteroides*	*Bacteroides fragilis*
277	*Bacteroidetes*	*Bacteroidia*	*Bacteroidales*	*Bacteroidaceae*	*Bacteroides*	*Bacteroides fragilis*
278	*Bacteroidetes*	*Bacteroidia*	*Bacteroidales*	*Bacteroidaceae*	*Bacteroides*	*Bacteroides fragilis*
279	*Bacteroidetes*	*Bacteroidia*	*Bacteroidales*	*Bacteroidaceae*	*Bacteroides*	*Bacteroides fragilis*
280	*Bacteroidetes*	*Bacteroidia*	*Bacteroidales*	*Bacteroidaceae*	*Bacteroides*	*Bacteroides fragilis*

Table S4. Isolated strains by culturomics

bacteriaId	*phylum*	*class*	*order*	*family*	*genus*	*species*
281	*Bacteroidetes*	*Bacteroidia*	*Bacteroidales*	*Bacteroidaceae*	*Bacteroides*	*Bacteroides fragilis*
282	*Bacteroidetes*	*Bacteroidia*	*Bacteroidales*	*Bacteroidaceae*	*Bacteroides*	*Bacteroides fragilis*
283	*Bacteroidetes*	*Bacteroidia*	*Bacteroidales*	*Bacteroidaceae*	*Bacteroides*	*Bacteroides fragilis*
284	*Bacteroidetes*	*Bacteroidia*	*Bacteroidales*	*Bacteroidaceae*	*Bacteroides*	*Bacteroides fragilis*
285	*Bacteroidetes*	*Bacteroidia*	*Bacteroidales*	*Bacteroidaceae*	*Bacteroides*	*Bacteroides fragilis*
286	*Bacteroidetes*	*Bacteroidia*	*Bacteroidales*	*Bacteroidaceae*	*Bacteroides*	*Bacteroides fragilis*
287	*Bacteroidetes*	*Bacteroidia*	*Bacteroidales*	*Bacteroidaceae*	*Bacteroides*	*Bacteroides fragilis*
288	*Bacteroidetes*	*Bacteroidia*	*Bacteroidales*	*Bacteroidaceae*	*Bacteroides*	*Bacteroides fragilis*
289	*Bacteroidetes*	*Bacteroidia*	*Bacteroidales*	*Bacteroidaceae*	*Bacteroides*	*Bacteroides fragilis*
290	*Bacteroidetes*	*Bacteroidia*	*Bacteroidales*	*Bacteroidaceae*	*Bacteroides*	*Bacteroides fragilis*
291	*Bacteroidetes*	*Bacteroidia*	*Bacteroidales*	*Bacteroidaceae*	*Bacteroides*	*Bacteroides fragilis*
292	*Bacteroidetes*	*Bacteroidia*	*Bacteroidales*	*Bacteroidaceae*	*Bacteroides*	*Bacteroides fragilis*
293	*Bacteroidetes*	*Bacteroidia*	*Bacteroidales*	*Bacteroidaceae*	*Bacteroides*	*Bacteroides fragilis*
294	*Bacteroidetes*	*Bacteroidia*	*Bacteroidales*	*Bacteroidaceae*	*Bacteroides*	*Bacteroides fragilis*
295	*Bacteroidetes*	*Bacteroidia*	*Bacteroidales*	*Bacteroidaceae*	*Bacteroides*	*Bacteroides fragilis*
296	*Bacteroidetes*	*Bacteroidia*	*Bacteroidales*	*Bacteroidaceae*	*Bacteroides*	*Bacteroides fragilis*
297	*Bacteroidetes*	*Bacteroidia*	*Bacteroidales*	*Bacteroidaceae*	*Bacteroides*	*Bacteroides fragilis*
298	*Bacteroidetes*	*Bacteroidia*	*Bacteroidales*	*Bacteroidaceae*	*Bacteroides*	*Bacteroides fragilis*
299	*Bacteroidetes*	*Bacteroidia*	*Bacteroidales*	*Bacteroidaceae*	*Bacteroides*	*Bacteroides fragilis*
300	*Bacteroidetes*	*Bacteroidia*	*Bacteroidales*	*Bacteroidaceae*	*Bacteroides*	*Bacteroides fragilis*
301	*Bacteroidetes*	*Bacteroidia*	*Bacteroidales*	*Bacteroidaceae*	*Bacteroides*	*Bacteroides fragilis*
302	*Bacteroidetes*	*Bacteroidia*	*Bacteroidales*	*Bacteroidaceae*	*Bacteroides*	*Bacteroides fragilis*
303	*Bacteroidetes*	*Bacteroidia*	*Bacteroidales*	*Bacteroidaceae*	*Bacteroides*	*Bacteroides fragilis*
304	*Bacteroidetes*	*Bacteroidia*	*Bacteroidales*	*Bacteroidaceae*	*Bacteroides*	*Bacteroides fragilis*
305	*Bacteroidetes*	*Bacteroidia*	*Bacteroidales*	*Bacteroidaceae*	*Bacteroides*	*Bacteroides fragilis*
306	*Bacteroidetes*	*Bacteroidia*	*Bacteroidales*	*Bacteroidaceae*	*Bacteroides*	*Bacteroides fragilis*
307	*Bacteroidetes*	*Bacteroidia*	*Bacteroidales*	*Bacteroidaceae*	*Bacteroides*	*Bacteroides*
308	*Bacteroidetes*	*Bacteroidia*	*Bacteroidales*	*Bacteroidaceae*	*Bacteroides*	*Bacteroides*

Table S4. Isolated strains by culturomics

bacteriaId	*phylum*	*class*	*order*	*family*	*genus*	*species*
309	*Bacteroidetes*	*Bacteroidia*	*Bacteroidales*	*Bacteroidaceae*	*Bacteroides*	*Bacteroides*
310	*Bacteroidetes*	*Bacteroidia*	*Bacteroidales*	*Bacteroidaceae*	*Bacteroides*	*Bacteroides*
311	*Bacteroidetes*	*Bacteroidia*	*Bacteroidales*	*Bacteroidaceae*	*Bacteroides*	*Bacteroides*
312	*Bacteroidetes*	*Bacteroidia*	*Bacteroidales*	*Bacteroidaceae*	*Bacteroides*	*Bacteroides*
313	*Bacteroidetes*	*Bacteroidia*	*Bacteroidales*	*Bacteroidaceae*	*Bacteroides*	*Bacteroides*
314	*Bacteroidetes*	*Bacteroidia*	*Bacteroidales*	*Bacteroidaceae*	*Bacteroides*	*Bacteroides nordii*
315	*Bacteroidetes*	*Bacteroidia*	*Bacteroidales*	*Bacteroidaceae*	*Bacteroides*	*Bacteroides nordii*
316	*Bacteroidetes*	*Bacteroidia*	*Bacteroidales*	*Bacteroidaceae*	*Bacteroides*	*Bacteroides nordii*
317	*Bacteroidetes*	*Bacteroidia*	*Bacteroidales*	*Bacteroidaceae*	*Bacteroides*	*Bacteroides nordii*
318	*Bacteroidetes*	*Bacteroidia*	*Bacteroidales*	*Bacteroidaceae*	*Bacteroides*	*Bacteroides nordii*
319	*Bacteroidetes*	*Bacteroidia*	*Bacteroidales*	*Bacteroidaceae*	*Bacteroides*	*Bacteroides nordii*
320	*Bacteroidetes*	*Bacteroidia*	*Bacteroidales*	*Bacteroidaceae*	*Bacteroides*	*Bacteroides nordii*
321	*Bacteroidetes*	*Bacteroidia*	*Bacteroidales*	*Bacteroidaceae*	*Bacteroides*	*Bacteroides nordii*
322	*Bacteroidetes*	*Bacteroidia*	*Bacteroidales*	*Bacteroidaceae*	*Bacteroides*	*Bacteroides nordii*
323	*Bacteroidetes*	*Bacteroidia*	*Bacteroidales*	*Bacteroidaceae*	*Bacteroides*	*Bacteroides nordii*
324	*Bacteroidetes*	*Bacteroidia*	*Bacteroidales*	*Bacteroidaceae*	*Bacteroides*	*Bacteroides nordii*
325	*Bacteroidetes*	*Bacteroidia*	*Bacteroidales*	*Bacteroidaceae*	*Bacteroides*	*Bacteroides nordii*
326	*Bacteroidetes*	*Bacteroidia*	*Bacteroidales*	*Bacteroidaceae*	*Bacteroides*	*Bacteroides nordii*
327	*Bacteroidetes*	*Bacteroidia*	*Bacteroidales*	*Bacteroidaceae*	*Bacteroides*	*Bacteroides nordii*
328	*Bacteroidetes*	*Bacteroidia*	*Bacteroidales*	*Bacteroidaceae*	*Bacteroides*	*Bacteroides nordii*
329	*Bacteroidetes*	*Bacteroidia*	*Bacteroidales*	*Bacteroidaceae*	*Bacteroides*	*Bacteroides nordii*
330	*Bacteroidetes*	*Bacteroidia*	*Bacteroidales*	*Bacteroidaceae*	*Bacteroides*	*Bacteroides nordii*
331	*Bacteroidetes*	*Bacteroidia*	*Bacteroidales*	*Bacteroidaceae*	*Bacteroides*	*Bacteroides nordii*
332	*Bacteroidetes*	*Bacteroidia*	*Bacteroidales*	*Bacteroidaceae*	*Bacteroides*	*Bacteroides nordii*
333	*Bacteroidetes*	*Bacteroidia*	*Bacteroidales*	*Bacteroidaceae*	*Bacteroides*	*Bacteroides nordii*
334	*Bacteroidetes*	*Bacteroidia*	*Bacteroidales*	*Bacteroidaceae*	*Bacteroides*	*Bacteroides ovatus*
335	*Bacteroidetes*	*Bacteroidia*	*Bacteroidales*	*Bacteroidaceae*	*Bacteroides*	*Bacteroides ovatus*
336	*Bacteroidetes*	*Bacteroidia*	*Bacteroidales*	*Bacteroidaceae*	*Bacteroides*	*Bacteroides ovatus*

Table S4. Isolated strains by culturomics

bacteriaId	*phylum*	*class*	*order*	*family*	*genus*	*species*
337	*Bacteroidetes*	*Bacteroidia*	*Bacteroidales*	*Bacteroidaceae*	*Bacteroides*	*Bacteroides ovatus*
338	*Bacteroidetes*	*Bacteroidia*	*Bacteroidales*	*Bacteroidaceae*	*Bacteroides*	*Bacteroides ovatus*
339	*Bacteroidetes*	*Bacteroidia*	*Bacteroidales*	*Bacteroidaceae*	*Bacteroides*	*Bacteroides ovatus*
340	*Bacteroidetes*	*Bacteroidia*	*Bacteroidales*	*Bacteroidaceae*	*Bacteroides*	*Bacteroides ovatus*
341	*Bacteroidetes*	*Bacteroidia*	*Bacteroidales*	*Bacteroidaceae*	*Bacteroides*	*Bacteroides ovatus*
342	*Bacteroidetes*	*Bacteroidia*	*Bacteroidales*	*Bacteroidaceae*	*Bacteroides*	*Bacteroides ovatus*
343	*Bacteroidetes*	*Bacteroidia*	*Bacteroidales*	*Bacteroidaceae*	*Bacteroides*	*Bacteroides ovatus*
344	*Bacteroidetes*	*Bacteroidia*	*Bacteroidales*	*Bacteroidaceae*	*Bacteroides*	*Bacteroides ovatus*
345	*Bacteroidetes*	*Bacteroidia*	*Bacteroidales*	*Bacteroidaceae*	*Bacteroides*	*Bacteroides ovatus*
346	*Bacteroidetes*	*Bacteroidia*	*Bacteroidales*	*Bacteroidaceae*	*Bacteroides*	*Bacteroides ovatus*
347	*Bacteroidetes*	*Bacteroidia*	*Bacteroidales*	*Bacteroidaceae*	*Bacteroides*	*Bacteroides*
348	*Bacteroidetes*	*Bacteroidia*	*Bacteroidales*	*Bacteroidaceae*	*Bacteroides*	*Bacteroides*
349	*Bacteroidetes*	*Bacteroidia*	*Bacteroidales*	*Bacteroidaceae*	*Bacteroides*	*Bacteroides*
350	*Bacteroidetes*	*Bacteroidia*	*Bacteroidales*	*Bacteroidaceae*	*Bacteroides*	*Bacteroides*
351	*Bacteroidetes*	*Bacteroidia*	*Bacteroidales*	*Bacteroidaceae*	*Bacteroides*	*Bacteroides*
352	*Bacteroidetes*	*Bacteroidia*	*Bacteroidales*	*Bacteroidaceae*	*Bacteroides*	*Bacteroides*
353	*Bacteroidetes*	*Bacteroidia*	*Bacteroidales*	*Bacteroidaceae*	*Bacteroides*	*Bacteroides*
354	*Bacteroidetes*	*Bacteroidia*	*Bacteroidales*	*Bacteroidaceae*	*Bacteroides*	*Bacteroides*
355	*Bacteroidetes*	*Bacteroidia*	*Bacteroidales*	*Bacteroidaceae*	*Bacteroides*	*Bacteroides*
356	*Bacteroidetes*	*Bacteroidia*	*Bacteroidales*	*Bacteroidaceae*	*Bacteroides*	*Bacteroides*
357	*Bacteroidetes*	*Bacteroidia*	*Bacteroidales*	*Bacteroidaceae*	*Bacteroides*	*Bacteroides*
358	*Bacteroidetes*	*Bacteroidia*	*Bacteroidales*	*Bacteroidaceae*	*Bacteroides*	*Bacteroides*
359	*Bacteroidetes*	*Bacteroidia*	*Bacteroidales*	*Bacteroidaceae*	*Bacteroides*	*Bacteroides*
360	*Bacteroidetes*	*Bacteroidia*	*Bacteroidales*	*Bacteroidaceae*	*Bacteroides*	*Bacteroides*
361	*Bacteroidetes*	*Bacteroidia*	*Bacteroidales*	*Bacteroidaceae*	*Bacteroides*	*Bacteroides*
362	*Bacteroidetes*	*Bacteroidia*	*Bacteroidales*	*Bacteroidaceae*	*Bacteroides*	*Bacteroides*
363	*Bacteroidetes*	*Bacteroidia*	*Bacteroidales*	*Bacteroidaceae*	*Bacteroides*	*Bacteroides*
364	*Bacteroidetes*	*Bacteroidia*	*Bacteroidales*	*Bacteroidaceae*	*Bacteroides*	*Bacteroides*

Table S4. Isolated strains by culturomics

bacteriaId	*phylum*	*class*	*order*	*family*	*genus*	*species*
365	*Bacteroidetes*	*Bacteroidia*	*Bacteroidales*	*Bacteroidaceae*	*Bacteroides*	*Bacteroides*
366	*Bacteroidetes*	*Bacteroidia*	*Bacteroidales*	*Bacteroidaceae*	*Bacteroides*	*Bacteroides*
367	*Bacteroidetes*	*Bacteroidia*	*Bacteroidales*	*Bacteroidaceae*	*Bacteroides*	*Bacteroides*
368	*Bacteroidetes*	*Bacteroidia*	*Bacteroidales*	*Bacteroidaceae*	*Bacteroides*	*Bacteroides*
369	*Bacteroidetes*	*Bacteroidia*	*Bacteroidales*	*Bacteroidaceae*	*Bacteroides*	*Bacteroides*
370	*Bacteroidetes*	*Bacteroidia*	*Bacteroidales*	*Bacteroidaceae*	*Bacteroides*	*Bacteroides*
371	*Bacteroidetes*	*Bacteroidia*	*Bacteroidales*	*Bacteroidaceae*	*Bacteroides*	*Bacteroides*
372	*Bacteroidetes*	*Bacteroidia*	*Bacteroidales*	*Bacteroidaceae*	*Bacteroides*	*Bacteroides*
373	*Bacteroidetes*	*Bacteroidia*	*Bacteroidales*	*Bacteroidaceae*	*Bacteroides*	*Bacteroides*
374	*Bacteroidetes*	*Bacteroidia*	*Bacteroidales*	*Bacteroidaceae*	*Bacteroides*	*Bacteroides*
375	*Bacteroidetes*	*Bacteroidia*	*Bacteroidales*	*Bacteroidaceae*	*Bacteroides*	*Bacteroides*
376	*Bacteroidetes*	*Bacteroidia*	*Bacteroidales*	*Bacteroidaceae*	*Bacteroides*	*Bacteroides*
377	*Bacteroidetes*	*Bacteroidia*	*Bacteroidales*	*Bacteroidaceae*	*Bacteroides*	*Bacteroides*
378	*Bacteroidetes*	*Bacteroidia*	*Bacteroidales*	*Bacteroidaceae*	*Bacteroides*	*Bacteroides*
379	*Bacteroidetes*	*Bacteroidia*	*Bacteroidales*	*Bacteroidaceae*	*Bacteroides*	*Bacteroides*
380	*Bacteroidetes*	*Bacteroidia*	*Bacteroidales*	*Bacteroidaceae*	*Bacteroides*	*Bacteroides*
381	*Bacteroidetes*	*Bacteroidia*	*Bacteroidales*	*Bacteroidaceae*	*Bacteroides*	*Bacteroides*
382	*Bacteroidetes*	*Bacteroidia*	*Bacteroidales*	*Bacteroidaceae*	*Bacteroides*	*Bacteroides*
383	*Bacteroidetes*	*Bacteroidia*	*Bacteroidales*	*Bacteroidaceae*	*Bacteroides*	*Bacteroides*
384	*Bacteroidetes*	*Bacteroidia*	*Bacteroidales*	*Bacteroidaceae*	*Bacteroides*	*Bacteroides*
385	*Bacteroidetes*	*Bacteroidia*	*Bacteroidales*	*Bacteroidaceae*	*Bacteroides*	*Bacteroides*
386	*Bacteroidetes*	*Bacteroidia*	*Bacteroidales*	*Bacteroidaceae*	*Bacteroides*	*Bacteroides*
387	*Bacteroidetes*	*Bacteroidia*	*Bacteroidales*	*Bacteroidaceae*	*Bacteroides*	*Bacteroides*
388	*Bacteroidetes*	*Bacteroidia*	*Bacteroidales*	*Bacteroidaceae*	*Bacteroides*	*Bacteroides*
389	*Bacteroidetes*	*Bacteroidia*	*Bacteroidales*	*Bacteroidaceae*	*Bacteroides*	*Bacteroides*
390	*Bacteroidetes*	*Bacteroidia*	*Bacteroidales*	*Bacteroidaceae*	*Bacteroides*	*Bacteroides*
391	*Bacteroidetes*	*Bacteroidia*	*Bacteroidales*	*Bacteroidaceae*	*Bacteroides*	*Bacteroides*
392	*Bacteroidetes*	*Bacteroidia*	*Bacteroidales*	*Bacteroidaceae*	*Bacteroides*	*Bacteroides*

Table S4. Isolated strains by culturomics

bacteriaId	*phylum*	*class*	*order*	*family*	*genus*	*species*
393	*Bacteroidetes*	*Bacteroidia*	*Bacteroidales*	*Bacteroidaceae*	*Bacteroides*	*Bacteroides*
394	*Bacteroidetes*	*Bacteroidia*	*Bacteroidales*	*Bacteroidaceae*	*Bacteroides*	*Bacteroides*
395	*Bacteroidetes*	*Bacteroidia*	*Bacteroidales*	*Bacteroidaceae*	*Bacteroides*	*Bacteroides*
396	*Bacteroidetes*	*Bacteroidia*	*Bacteroidales*	*Bacteroidaceae*	*Bacteroides*	*Bacteroides*
397	*Bacteroidetes*	*Bacteroidia*	*Bacteroidales*	*Bacteroidaceae*	*Bacteroides*	*Bacteroides*
398	*Bacteroidetes*	*Bacteroidia*	*Bacteroidales*	*Bacteroidaceae*	*Bacteroides*	*Bacteroides*
399	*Bacteroidetes*	*Bacteroidia*	*Bacteroidales*	*Bacteroidaceae*	*Bacteroides*	*Bacteroides*
400	*Bacteroidetes*	*Bacteroidia*	*Bacteroidales*	*Bacteroidaceae*	*Bacteroides*	*Bacteroides*
401	*Bacteroidetes*	*Bacteroidia*	*Bacteroidales*	*Bacteroidaceae*	*Bacteroides*	*Bacteroides*
402	*Bacteroidetes*	*Bacteroidia*	*Bacteroidales*	*Bacteroidaceae*	*Bacteroides*	*Bacteroides*
403	*Bacteroidetes*	*Bacteroidia*	*Bacteroidales*	*Bacteroidaceae*	*Bacteroides*	*Bacteroides*
404	*Bacteroidetes*	*Bacteroidia*	*Bacteroidales*	*Bacteroidaceae*	*Bacteroides*	*Bacteroides*
405	*Bacteroidetes*	*Bacteroidia*	*Bacteroidales*	*Bacteroidaceae*	*Bacteroides*	*Bacteroides*
406	*Bacteroidetes*	*Bacteroidia*	*Bacteroidales*	*Bacteroidaceae*	*Bacteroides*	*Bacteroides*
407	*Bacteroidetes*	*Bacteroidia*	*Bacteroidales*	*Bacteroidaceae*	*Bacteroides*	*Bacteroides*
408	*Bacteroidetes*	*Bacteroidia*	*Bacteroidales*	*Bacteroidaceae*	*Bacteroides*	*Bacteroides*
409	*Bacteroidetes*	*Bacteroidia*	*Bacteroidales*	*Bacteroidaceae*	*Bacteroides*	*Bacteroides*
410	*Bacteroidetes*	*Bacteroidia*	*Bacteroidales*	*Bacteroidaceae*	*Bacteroides*	*Bacteroides*
411	*Bacteroidetes*	*Bacteroidia*	*Bacteroidales*	*Bacteroidaceae*	*Bacteroides*	*Bacteroides*
412	*Bacteroidetes*	*Bacteroidia*	*Bacteroidales*	*Bacteroidaceae*	*Bacteroides*	*Bacteroides*
413	*Bacteroidetes*	*Bacteroidia*	*Bacteroidales*	*Bacteroidaceae*	*Bacteroides*	*Bacteroides*
414	*Bacteroidetes*	*Bacteroidia*	*Bacteroidales*	*Bacteroidaceae*	*Bacteroides*	*Bacteroides*
415	*Bacteroidetes*	*Bacteroidia*	*Bacteroidales*	*Bacteroidaceae*	*Bacteroides*	*Bacteroides*
416	*Bacteroidetes*	*Bacteroidia*	*Bacteroidales*	*Bacteroidaceae*	*Bacteroides*	*Bacteroides*
417	*Bacteroidetes*	*Bacteroidia*	*Bacteroidales*	*Bacteroidaceae*	*Bacteroides*	*Bacteroides*
418	*Bacteroidetes*	*Bacteroidia*	*Bacteroidales*	*Bacteroidaceae*	*Bacteroides*	*Bacteroides*
419	*Bacteroidetes*	*Bacteroidia*	*Bacteroidales*	*Bacteroidaceae*	*Bacteroides*	*Bacteroides*
420	*Bacteroidetes*	*Bacteroidia*	*Bacteroidales*	*Bacteroidaceae*	*Bacteroides*	*Bacteroides*

Table S4. Isolated strains by culturomics

bacteriaId	*phylum*	*class*	*order*	*family*	*genus*	*species*
421	*Bacteroidetes*	*Bacteroidia*	*Bacteroidales*	*Bacteroidaceae*	*Bacteroides*	*Bacteroides*
422	*Bacteroidetes*	*Bacteroidia*	*Bacteroidales*	*Bacteroidaceae*	*Bacteroides*	*Bacteroides*
423	*Bacteroidetes*	*Bacteroidia*	*Bacteroidales*	*Bacteroidaceae*	*Bacteroides*	*Bacteroides*
424	*Bacteroidetes*	*Bacteroidia*	*Bacteroidales*	*Bacteroidaceae*	*Bacteroides*	*Bacteroides*
425	*Bacteroidetes*	*Bacteroidia*	*Bacteroidales*	*Bacteroidaceae*	*Bacteroides*	*Bacteroides*
426	*Bacteroidetes*	*Bacteroidia*	*Bacteroidales*	*Bacteroidaceae*	*Bacteroides*	*Bacteroides*
427	*Bacteroidetes*	*Bacteroidia*	*Bacteroidales*	*Bacteroidaceae*	*Bacteroides*	*Bacteroides*
428	*Bacteroidetes*	*Bacteroidia*	*Bacteroidales*	*Bacteroidaceae*	*Bacteroides*	*Bacteroides*
429	*Bacteroidetes*	*Bacteroidia*	*Bacteroidales*	*Bacteroidaceae*	*Bacteroides*	*Bacteroides*
430	*Bacteroidetes*	*Bacteroidia*	*Bacteroidales*	*Bacteroidaceae*	*Bacteroides*	*Bacteroides*
431	*Bacteroidetes*	*Bacteroidia*	*Bacteroidales*	*Bacteroidaceae*	*Bacteroides*	*Bacteroides*
432	*Bacteroidetes*	*Bacteroidia*	*Bacteroidales*	*Bacteroidaceae*	*Bacteroides*	*Bacteroides*
433	*Bacteroidetes*	*Bacteroidia*	*Bacteroidales*	*Bacteroidaceae*	*Bacteroides*	*Bacteroides*
434	*Bacteroidetes*	*Bacteroidia*	*Bacteroidales*	*Bacteroidaceae*	*Bacteroides*	*Bacteroides*
435	*Bacteroidetes*	*Bacteroidia*	*Bacteroidales*	*Bacteroidaceae*	*Bacteroides*	*Bacteroides*
436	*Bacteroidetes*	*Bacteroidia*	*Bacteroidales*	*Bacteroidaceae*	*Bacteroides*	*Bacteroides*
437	*Bacteroidetes*	*Bacteroidia*	*Bacteroidales*	*Bacteroidaceae*	*Bacteroides*	*Bacteroides*
438	*Bacteroidetes*	*Bacteroidia*	*Bacteroidales*	*Bacteroidaceae*	*Bacteroides*	*Bacteroides*
439	*Bacteroidetes*	*Bacteroidia*	*Bacteroidales*	*Bacteroidaceae*	*Bacteroides*	*Bacteroides*
440	*Bacteroidetes*	*Bacteroidia*	*Bacteroidales*	*Bacteroidaceae*	*Bacteroides*	*Bacteroides*
441	*Bacteroidetes*	*Bacteroidia*	*Bacteroidales*	*Bacteroidaceae*	*Bacteroides*	*Bacteroides*
442	*Bacteroidetes*	*Bacteroidia*	*Bacteroidales*	*Bacteroidaceae*	*Bacteroides*	*Bacteroides*
443	*Bacteroidetes*	*Bacteroidia*	*Bacteroidales*	*Bacteroidaceae*	*Bacteroides*	*Bacteroides*
444	*Bacteroidetes*	*Bacteroidia*	*Bacteroidales*	*Bacteroidaceae*	*Bacteroides*	*Bacteroides*
445	*Bacteroidetes*	*Bacteroidia*	*Bacteroidales*	*Bacteroidaceae*	*Bacteroides*	*Bacteroides*
446	*Bacteroidetes*	*Bacteroidia*	*Bacteroidales*	*Bacteroidaceae*	*Bacteroides*	*Bacteroides*
447	*Bacteroidetes*	*Bacteroidia*	*Bacteroidales*	*Bacteroidaceae*	*Bacteroides*	*Bacteroides*
448	*Bacteroidetes*	*Bacteroidia*	*Bacteroidales*	*Bacteroidaceae*	*Bacteroides*	*Bacteroides*

Table S4. Isolated strains by culturomics

bacteriaId	*phylum*	*class*	*order*	*family*	*genus*	*species*
449	*Bacteroidetes*	*Bacteroidia*	*Bacteroidales*	*Bacteroidaceae*	*Bacteroides*	*Bacteroides*
450	*Bacteroidetes*	*Bacteroidia*	*Bacteroidales*	*Bacteroidaceae*	*Bacteroides*	*Bacteroides*
451	*Bacteroidetes*	*Bacteroidia*	*Bacteroidales*	*Bacteroidaceae*	*Bacteroides*	*Bacteroides*
452	*Bacteroidetes*	*Bacteroidia*	*Bacteroidales*	*Bacteroidaceae*	*Bacteroides*	*Bacteroides*
453	*Bacteroidetes*	*Bacteroidia*	*Bacteroidales*	*Bacteroidaceae*	*Bacteroides*	*Bacteroides*
454	*Bacteroidetes*	*Bacteroidia*	*Bacteroidales*	*Bacteroidaceae*	*Bacteroides*	*Bacteroides*
455	*Bacteroidetes*	*Bacteroidia*	*Bacteroidales*	*Bacteroidaceae*	*Bacteroides*	*Bacteroides*
456	*Bacteroidetes*	*Bacteroidia*	*Bacteroidales*	*Bacteroidaceae*	*Bacteroides*	*Bacteroides*
457	*Bacteroidetes*	*Bacteroidia*	*Bacteroidales*	*Bacteroidaceae*	*Bacteroides*	*Bacteroides*
458	*Bacteroidetes*	*Bacteroidia*	*Bacteroidales*	*Bacteroidaceae*	*Bacteroides*	*Bacteroides*
459	*Bacteroidetes*	*Bacteroidia*	*Bacteroidales*	*Bacteroidaceae*	*Bacteroides*	*Bacteroides*
460	*Bacteroidetes*	*Bacteroidia*	*Bacteroidales*	*Bacteroidaceae*	*Bacteroides*	*Bacteroides*
461	*Bacteroidetes*	*Bacteroidia*	*Bacteroidales*	*Bacteroidaceae*	*Bacteroides*	*Bacteroides*
462	*Bacteroidetes*	*Bacteroidia*	*Bacteroidales*	*Bacteroidaceae*	*Bacteroides*	*Bacteroides*
463	*Bacteroidetes*	*Bacteroidia*	*Bacteroidales*	*Bacteroidaceae*	*Bacteroides*	*Bacteroides*
464	*Bacteroidetes*	*Bacteroidia*	*Bacteroidales*	*Bacteroidaceae*	*Bacteroides*	*Bacteroides*
465	*Bacteroidetes*	*Bacteroidia*	*Bacteroidales*	*Bacteroidaceae*	*Bacteroides*	*Bacteroides*
466	*Bacteroidetes*	*Bacteroidia*	*Bacteroidales*	*Bacteroidaceae*	*Bacteroides*	*Bacteroides*
467	*Bacteroidetes*	*Bacteroidia*	*Bacteroidales*	*Bacteroidaceae*	*Bacteroides*	*Bacteroides*
468	*Bacteroidetes*	*Bacteroidia*	*Bacteroidales*	*Bacteroidaceae*	*Bacteroides*	*Bacteroides*
469	*Bacteroidetes*	*Bacteroidia*	*Bacteroidales*	*Bacteroidaceae*	*Bacteroides*	*Bacteroides*
470	*Bacteroidetes*	*Bacteroidia*	*Bacteroidales*	*Bacteroidaceae*	*Bacteroides*	*Bacteroides*
471	*Bacteroidetes*	*Bacteroidia*	*Bacteroidales*	*Bacteroidaceae*	*Bacteroides*	*Bacteroides*
472	*Bacteroidetes*	*Bacteroidia*	*Bacteroidales*	*Bacteroidaceae*	*Bacteroides*	*Bacteroides*
473	*Bacteroidetes*	*Bacteroidia*	*Bacteroidales*	*Bacteroidaceae*	*Bacteroides*	*Bacteroides*
474	*Bacteroidetes*	*Bacteroidia*	*Bacteroidales*	*Bacteroidaceae*	*Bacteroides*	*Bacteroides*
475	*Bacteroidetes*	*Bacteroidia*	*Bacteroidales*	*Bacteroidaceae*	*Bacteroides*	*Bacteroides*
476	*Bacteroidetes*	*Bacteroidia*	*Bacteroidales*	*Bacteroidaceae*	*Bacteroides*	*Bacteroides*

Table S4. Isolated strains by culturomics

bacteriaId	*phylum*	*class*	*order*	*family*	*genus*	*species*
477	*Bacteroidetes*	*Bacteroidia*	*Bacteroidales*	*Bacteroidaceae*	*Bacteroides*	*Bacteroides*
478	*Bacteroidetes*	*Bacteroidia*	*Bacteroidales*	*Bacteroidaceae*	*Bacteroides*	*Bacteroides*
479	*Bacteroidetes*	*Bacteroidia*	*Bacteroidales*	*Bacteroidaceae*	*Bacteroides*	*Bacteroides*
480	*Bacteroidetes*	*Bacteroidia*	*Bacteroidales*	*Bacteroidaceae*	*Bacteroides*	*Bacteroides*
481	*Bacteroidetes*	*Bacteroidia*	*Bacteroidales*	*Bacteroidaceae*	*Bacteroides*	*Bacteroides*
482	*Bacteroidetes*	*Bacteroidia*	*Bacteroidales*	*Bacteroidaceae*	*Bacteroides*	*Bacteroides*
483	*Bacteroidetes*	*Bacteroidia*	*Bacteroidales*	*Bacteroidaceae*	*Bacteroides*	*Bacteroides*
484	*Bacteroidetes*	*Bacteroidia*	*Bacteroidales*	*Bacteroidaceae*	*Bacteroides*	*Bacteroides*
485	*Bacteroidetes*	*Bacteroidia*	*Bacteroidales*	*Bacteroidaceae*	*Bacteroides*	*Bacteroides*
486	*Bacteroidetes*	*Bacteroidia*	*Bacteroidales*	*Dysgonomonadacea*	*Dysgonomonas*	*Dysgonomonas*
487	*Bacteroidetes*	*Bacteroidia*	*Bacteroidales*	*Prevotellaceae*	*Prevotella*	*Prevotella sp.*
488	*Bacteroidetes*	*Bacteroidia*	*Bacteroidales*	*Prevotellaceae*	*Prevotella*	*Prevotella sp.*
489	*Bacteroidetes*	*Bacteroidia*	*Bacteroidales*	*Prevotellaceae*	*Prevotella*	*Prevotella sp.*
490	*Bacteroidetes*	*Bacteroidia*	*Bacteroidales*	*Prevotellaceae*	*Prevotella*	*Prevotella sp.*
491	*Bacteroidetes*	*Bacteroidia*	*Bacteroidales*	*Prevotellaceae*	*Prevotella*	*Prevotella sp.*
492	*Bacteroidetes*	*Bacteroidia*	*Bacteroidales*	*Prevotellaceae*	*Prevotella*	*Prevotella sp.*
493	*Bacteroidetes*	*Bacteroidia*	*Bacteroidales*	*Prevotellaceae*	*Prevotella*	*Prevotella sp.*
494	*Bacteroidetes*	*Bacteroidia*	*Bacteroidales*	*Prevotellaceae*	*Prevotella*	*Prevotella sp.*
495	*Bacteroidetes*	*Bacteroidia*	*Bacteroidales*	*Prevotellaceae*	*Prevotella*	*Prevotella sp.*
496	*Bacteroidetes*	*Bacteroidia*	*Bacteroidales*	*Prevotellaceae*	*Prevotella*	*Prevotella sp.*
497	*Bacteroidetes*	*Bacteroidia*	*Bacteroidales*	*Rikenellaceae*	*Alistipes*	*Alistipes finegoldii*
498	*Bacteroidetes*	*Bacteroidia*	*Bacteroidales*	*Rikenellaceae*	*Alistipes*	*Alistipes*
499	*Bacteroidetes*	*Bacteroidia*	*Bacteroidales*	*Rikenellaceae*	*Alistipes*	*Alistipes*
500	*Bacteroidetes*	*Bacteroidia*	*Bacteroidales*	*Rikenellaceae*	*Alistipes*	*Alistipes*
501	*Bacteroidetes*	*Bacteroidia*	*Bacteroidales*	*Rikenellaceae*	*Alistipes*	*Alistipes*
502	*Bacteroidetes*	*Bacteroidia*	*Bacteroidales*	*Rikenellaceae*	*Alistipes*	*Alistipes*
503	*Bacteroidetes*	*Bacteroidia*	*Bacteroidales*	*Rikenellaceae*	*Alistipes*	*Alistipes*
504	*Bacteroidetes*	*Bacteroidia*	*Bacteroidales*	*Rikenellaceae*	*Alistipes*	*Alistipes shahii*

Table S4. Isolated strains by culturomics

bacteriaId	*phylum*	*class*	*order*	*family*	*genus*	*species*
505	*Bacteroidetes*	*Bacteroidia*	*Bacteroidales*	*Tannerellaceae*	*Parabacteroides*	*Parabacteroides*
506	*Bacteroidetes*	*Bacteroidia*	*Bacteroidales*	*Tannerellaceae*	*Parabacteroides*	*Parabacteroides*
507	*Bacteroidetes*	*Bacteroidia*	*Bacteroidales*	*Tannerellaceae*	*Parabacteroides*	*Parabacteroides*
508	*Bacteroidetes*	*Bacteroidia*	*Bacteroidales*	*Tannerellaceae*	*Parabacteroides*	*Parabacteroides*
509	*Bacteroidetes*	*Bacteroidia*	*Bacteroidales*	*Tannerellaceae*	*Parabacteroides*	*Parabacteroides*
510	*Bacteroidetes*	*Bacteroidia*	*Bacteroidales*	*Tannerellaceae*	*Parabacteroides*	*Parabacteroides*
511	*Bacteroidetes*	*Bacteroidia*	*Bacteroidales*	*Tannerellaceae*	*Parabacteroides*	*Parabacteroides*
512	*Bacteroidetes*	*Bacteroidia*	*Bacteroidales*	*Tannerellaceae*	*Parabacteroides*	*Parabacteroides*
513	*Bacteroidetes*	*Bacteroidia*	*Bacteroidales*	*Tannerellaceae*	*Parabacteroides*	*Parabacteroides*
514	*Bacteroidetes*	*Bacteroidia*	*Bacteroidales*	*Tannerellaceae*	*Parabacteroides*	*Parabacteroides*
515	*Bacteroidetes*	*Bacteroidia*	*Bacteroidales*	*Tannerellaceae*	*Parabacteroides*	*Parabacteroides*
516	*Bacteroidetes*	*Bacteroidia*	*Bacteroidales*	*Tannerellaceae*	*Parabacteroides*	*Parabacteroides*
517	*Bacteroidetes*	*Bacteroidia*	*Bacteroidales*	*Tannerellaceae*	*Parabacteroides*	*Parabacteroides*
518	*Bacteroidetes*	*Bacteroidia*	*Bacteroidales*	*Tannerellaceae*	*Parabacteroides*	*Parabacteroides*
519	*Bacteroidetes*	*Bacteroidia*	*Bacteroidales*	*Tannerellaceae*	*Parabacteroides*	*Parabacteroides*
520	*Bacteroidetes*	*Bacteroidia*	*Bacteroidales*	*Tannerellaceae*	*Parabacteroides*	*Parabacteroides*
521	*Bacteroidetes*	*Bacteroidia*	*Bacteroidales*	*Tannerellaceae*	*Parabacteroides*	*Parabacteroides*
522	*Bacteroidetes*	*Bacteroidia*	*Bacteroidales*	*Tannerellaceae*	*Parabacteroides*	*Parabacteroides*
523	*Bacteroidetes*	*Bacteroidia*	*Bacteroidales*	*Tannerellaceae*	*Parabacteroides*	*Parabacteroides*
524	*Bacteroidetes*	*Bacteroidia*	*Bacteroidales*	*Tannerellaceae*	*Parabacteroides*	*Parabacteroides*
525	*Bacteroidetes*	*Bacteroidia*	*Bacteroidales*	*Tannerellaceae*	*Parabacteroides*	*Parabacteroides*
526	*Bacteroidetes*	*Bacteroidia*	*Bacteroidales*	*Tannerellaceae*	*Parabacteroides*	*Parabacteroides*
527	*Bacteroidetes*	*Bacteroidia*	*Bacteroidales*	*Tannerellaceae*	*Parabacteroides*	*Parabacteroides*
528	*Bacteroidetes*	*Bacteroidia*	*Bacteroidales*	*Tannerellaceae*	*Parabacteroides*	*Parabacteroides*
529	*Bacteroidetes*	*Bacteroidia*	*Bacteroidales*	*Tannerellaceae*	*Parabacteroides*	*Parabacteroides*
530	*Bacteroidetes*	*Bacteroidia*	*Bacteroidales*	*Tannerellaceae*	*Parabacteroides*	*Parabacteroides*
531	*Bacteroidetes*	*Bacteroidia*	*Bacteroidales*	*Tannerellaceae*	*Parabacteroides*	*Parabacteroides*
532	*Bacteroidetes*	*Bacteroidia*	*Bacteroidales*	*Tannerellaceae*	*Parabacteroides*	*Parabacteroides*

Table S4. Isolated strains by culturomics

bacterialId	*phylum*	*class*	*order*	*family*	*genus*	*species*
533	*Bacteroidetes*	*Bacteroidia*	*Bacteroidales*	*Tannerellaceae*	*Parabacteroides*	*Parabacteroides*
534	*Bacteroidetes*	*Bacteroidia*	*Bacteroidales*	*Tannerellaceae*	*Parabacteroides*	*Parabacteroides*
535	*Bacteroidetes*	*Bacteroidia*	*Bacteroidales*	*Tannerellaceae*	*Parabacteroides*	*Parabacteroides*
536	*Bacteroidetes*	*Bacteroidia*	*Bacteroidales*	*Tannerellaceae*	*Parabacteroides*	*Parabacteroides*
537	*Bacteroidetes*	*Bacteroidia*	*Bacteroidales*	*Tannerellaceae*	*Parabacteroides*	*Parabacteroides*
538	*Bacteroidetes*	*Bacteroidia*	*Bacteroidales*	*Tannerellaceae*	*Parabacteroides*	*Parabacteroides*
539	*Bacteroidetes*	*Bacteroidia*	*Bacteroidales*	*Tannerellaceae*	*Parabacteroides*	*Parabacteroides*
540	*Bacteroidetes*	*Bacteroidia*	*Bacteroidales*	*Tannerellaceae*	*Parabacteroides*	*Parabacteroides*
541	*Bacteroidetes*	*Bacteroidia*	*Bacteroidales*	*Tannerellaceae*	*Parabacteroides*	*Parabacteroides*
542	*Bacteroidetes*	*Bacteroidia*	*Bacteroidales*	*Tannerellaceae*	*Parabacteroides*	*Parabacteroides*
543	*Bacteroidetes*	*Bacteroidia*	*Bacteroidales*	*Tannerellaceae*	*Parabacteroides*	*Parabacteroides*
544	*Bacteroidetes*	*Bacteroidia*	*Bacteroidales*	*Tannerellaceae*	*Parabacteroides*	*Parabacteroides*
545	*Bacteroidetes*	*Bacteroidia*	*Bacteroidales*	*Tannerellaceae*	*Parabacteroides*	*Parabacteroides*
546	*Bacteroidetes*	*Bacteroidia*	*Bacteroidales*	*Tannerellaceae*	*Parabacteroides*	*Parabacteroides*
547	*Bacteroidetes*	*Bacteroidia*	*Bacteroidales*	*Tannerellaceae*	*Parabacteroides*	*Parabacteroides*
548	*Bacteroidetes*	*Bacteroidia*	*Bacteroidales*	*Tannerellaceae*	*Parabacteroides*	*Parabacteroides*
549	*Bacteroidetes*	*Bacteroidia*	*Bacteroidales*	*Tannerellaceae*	*Parabacteroides*	*Parabacteroides*
550	*Bacteroidetes*	*Bacteroidia*	*Bacteroidales*	*Tannerellaceae*	*Parabacteroides*	*Parabacteroides*
551	*Bacteroidetes*	*Bacteroidia*	*Bacteroidales*	*Tannerellaceae*	*Parabacteroides*	*Parabacteroides*
552	*Bacteroidetes*	*Bacteroidia*	*Bacteroidales*	*Tannerellaceae*	*Parabacteroides*	*Parabacteroides*
553	*Bacteroidetes*	*Bacteroidia*	*Bacteroidales*	*Tannerellaceae*	*Parabacteroides*	*Parabacteroides*
554	*Bacteroidetes*	*Bacteroidia*	*Bacteroidales*	*Tannerellaceae*	*Parabacteroides*	*Parabacteroides*
555	*Bacteroidetes*	*Bacteroidia*	*Bacteroidales*	*Tannerellaceae*	*Parabacteroides*	*Parabacteroides*
556	*Bacteroidetes*	*Bacteroidia*	*Bacteroidales*	*Tannerellaceae*	*Parabacteroides*	*Parabacteroides*
557	*Bacteroidetes*	*Bacteroidia*	*Bacteroidales*	*Tannerellaceae*	*Parabacteroides*	*Parabacteroides*
558	*Bacteroidetes*	*Bacteroidia*	*Bacteroidales*	*Tannerellaceae*	*Parabacteroides*	*Parabacteroides*
559	*Bacteroidetes*	*Bacteroidia*	*Bacteroidales*	*Tannerellaceae*	*Parabacteroides*	*Parabacteroides*
560	*Bacteroidetes*	*Bacteroidia*	*Bacteroidales*	*Tannerellaceae*	*Parabacteroides*	*Parabacteroides*

Table S4. Isolated strains by culturomics

bacteriaId	*phylum*	*class*	*order*	*family*	*genus*	*species*
561	*Bacteroidetes*	*Bacteroidia*	*Bacteroidales*	*Tannerellaceae*	*Parabacteroides*	*Parabacteroides*
562	*Bacteroidetes*	*Bacteroidia*	*Bacteroidales*	*Tannerellaceae*	*Parabacteroides*	*Parabacteroides*
563	*Bacteroidetes*	*Bacteroidia*	*Bacteroidales*	*Tannerellaceae*	*Parabacteroides*	*Parabacteroides*
564	*Bacteroidetes*	*Bacteroidia*	*Bacteroidales*	*Tannerellaceae*	*Parabacteroides*	*Parabacteroides*
565	*Bacteroidetes*	*Bacteroidia*	*Bacteroidales*	*Tannerellaceae*	*Parabacteroides*	*Parabacteroides*
566	*Bacteroidetes*	*Bacteroidia*	*Bacteroidales*	*Tannerellaceae*	*Parabacteroides*	*Parabacteroides*
567	*Bacteroidetes*	*Bacteroidia*	*Bacteroidales*	*Tannerellaceae*	*Parabacteroides*	*Parabacteroides*
568	*Bacteroidetes*	*Bacteroidia*	*Bacteroidales*	*Tannerellaceae*	*Parabacteroides*	*Parabacteroides*
569	*Bacteroidetes*	*Bacteroidia*	*Bacteroidales*	*Tannerellaceae*	*Parabacteroides*	*Parabacteroides*
570	*Bacteroidetes*	*Bacteroidia*	*Bacteroidales*	*Tannerellaceae*	*Parabacteroides*	*Parabacteroides*
571	*Bacteroidetes*	*Bacteroidia*	*Bacteroidales*	*Tannerellaceae*	*Parabacteroides*	*Parabacteroides*
572	*Bacteroidetes*	*Bacteroidia*	*Bacteroidales*	*Tannerellaceae*	*Parabacteroides*	*Parabacteroides*
573	*Bacteroidetes*	*Bacteroidia*	*Bacteroidales*	*Tannerellaceae*	*Parabacteroides*	*Parabacteroides*
574	*Bacteroidetes*	*Bacteroidia*	*Bacteroidales*	*Tannerellaceae*	*Parabacteroides*	*Parabacteroides*
575	*Bacteroidetes*	*Bacteroidia*	*Bacteroidales*	*Tannerellaceae*	*Parabacteroides*	*Parabacteroides*
576	*Bacteroidetes*	*Bacteroidia*	*Bacteroidales*	*Tannerellaceae*	*Parabacteroides*	*Parabacteroides*
577	*Bacteroidetes*	*Bacteroidia*	*Bacteroidales*	*Tannerellaceae*	*Parabacteroides*	*Parabacteroides*
578	*Bacteroidetes*	*Bacteroidia*	*Bacteroidales*	*Tannerellaceae*	*Parabacteroides*	*Parabacteroides*
579	*Bacteroidetes*	*Bacteroidia*	*Bacteroidales*	*Tannerellaceae*	*Parabacteroides*	*Parabacteroides*
580	*Bacteroidetes*	*Bacteroidia*	*Bacteroidales*	*Tannerellaceae*	*Parabacteroides*	*Parabacteroides*
581	*Bacteroidetes*	*Bacteroidia*	*Bacteroidales*	*Tannerellaceae*	*Parabacteroides*	*Parabacteroides*
582	*Bacteroidetes*	*Bacteroidia*	*Bacteroidales*	*Tannerellaceae*	*Parabacteroides*	*Parabacteroides*
583	*Bacteroidetes*	*Bacteroidia*	*Bacteroidales*	*Tannerellaceae*	*Parabacteroides*	*Parabacteroides*
584	*Bacteroidetes*	*Bacteroidia*	*Bacteroidales*	*Tannerellaceae*	*Parabacteroides*	*Parabacteroides*
585	*Bacteroidetes*	*Bacteroidia*	*Bacteroidales*	*Tannerellaceae*	*Parabacteroides*	*Parabacteroides*
586	*Bacteroidetes*	*Bacteroidia*	*Bacteroidales*	*Tannerellaceae*	*Parabacteroides*	*Parabacteroides*
587	*Bacteroidetes*	*Bacteroidia*	*Bacteroidales*	*Tannerellaceae*	*Parabacteroides*	*Parabacteroides*
588	*Bacteroidetes*	*Bacteroidia*	*Bacteroidales*	*Tannerellaceae*	*Parabacteroides*	*Parabacteroides*

Table S4. Isolated strains by culturomics

bacterialId	*phylum*	*class*	*order*	*family*	*genus*	*species*
589	*Bacteroidetes*	*Bacteroidia*	*Bacteroidales*	*Tannerellaceae*	*Parabacteroides*	*Parabacteroides*
590	*Bacteroidetes*	*Bacteroidia*	*Bacteroidales*	*Tannerellaceae*	*Parabacteroides*	*Parabacteroides*
591	*Bacteroidetes*	*Bacteroidia*	*Bacteroidales*	*Tannerellaceae*	*Parabacteroides*	*Parabacteroides*
592	*Bacteroidetes*	*Bacteroidia*	*Bacteroidales*	*Tannerellaceae*	*Parabacteroides*	*Parabacteroides*
593	*Bacteroidetes*	*Bacteroidia*	*Bacteroidales*	*Tannerellaceae*	*Parabacteroides*	*Parabacteroides*
594	*Bacteroidetes*	*Bacteroidia*	*Bacteroidales*	*Tannerellaceae*	*Parabacteroides*	*Parabacteroides*
595	*Bacteroidetes*	*Bacteroidia*	*Bacteroidales*	*Tannerellaceae*	*Parabacteroides*	*Parabacteroides*
596	*Bacteroidetes*	*Bacteroidia*	*Bacteroidales*	*Tannerellaceae*	*Parabacteroides*	*Parabacteroides*
597	*Bacteroidetes*	*Bacteroidia*	*Bacteroidales*	*Tannerellaceae*	*Parabacteroides*	*Parabacteroides*
598	*Bacteroidetes*	*Bacteroidia*	*Bacteroidales*	*Tannerellaceae*	*Parabacteroides*	*Parabacteroides*
599	*Bacteroidetes*	*Bacteroidia*	*Bacteroidales*	*Tannerellaceae*	*Parabacteroides*	*Parabacteroides*
600	*Bacteroidetes*	*Bacteroidia*	*Bacteroidales*	*Tannerellaceae*	*Parabacteroides*	*Parabacteroides*
601	*Bacteroidetes*	*Bacteroidia*	*Bacteroidales*	*Tannerellaceae*	*Parabacteroides*	*Parabacteroides*
602	*Bacteroidetes*	*Bacteroidia*	*Bacteroidales*	*Tannerellaceae*	*Parabacteroides*	*Parabacteroides*
603	*Bacteroidetes*	*Bacteroidia*	*Bacteroidales*	*Tannerellaceae*	*Parabacteroides*	*Parabacteroides*
604	*Bacteroidetes*	*Bacteroidia*	*Bacteroidales*	*Tannerellaceae*	*Parabacteroides*	*Parabacteroides*
605	*Bacteroidetes*	*Bacteroidia*	*Bacteroidales*	*Tannerellaceae*	*Parabacteroides*	*Parabacteroides*
606	*Bacteroidetes*	*Bacteroidia*	*Bacteroidales*	*Tannerellaceae*	*Parabacteroides*	*Parabacteroides*
607	*Bacteroidetes*	*Bacteroidia*	*Bacteroidales*	*Tannerellaceae*	*Parabacteroides*	*Parabacteroides*
608	*Bacteroidetes*	*Bacteroidia*	*Bacteroidales*	*Tannerellaceae*	*Parabacteroides*	*Parabacteroides*
609	*Bacteroidetes*	*Bacteroidia*	*Bacteroidales*	*Tannerellaceae*	*Parabacteroides*	*Parabacteroides*
610	*Bacteroidetes*	*Bacteroidia*	*Bacteroidales*	*Tannerellaceae*	*Parabacteroides*	*Parabacteroides*
611	*Bacteroidetes*	*Bacteroidia*	*Bacteroidales*	*Tannerellaceae*	*Parabacteroides*	*Parabacteroides*
612	*Bacteroidetes*	*Bacteroidia*	*Bacteroidales*	*Tannerellaceae*	*Parabacteroides*	*Parabacteroides*
613	*Bacteroidetes*	*Bacteroidia*	*Bacteroidales*	*Tannerellaceae*	*Parabacteroides*	*Parabacteroides*
614	*Bacteroidetes*	*Bacteroidia*	*Bacteroidales*	*Tannerellaceae*	*Parabacteroides*	*Parabacteroides*
615	*Bacteroidetes*	*Bacteroidia*	*Bacteroidales*	*Tannerellaceae*	*Parabacteroides*	*Parabacteroides*
616	*Bacteroidetes*	*Bacteroidia*	*Bacteroidales*	*Tannerellaceae*	*Parabacteroides*	*Parabacteroides*

Table S4. Isolated strains by culturomics

bacteriald	*phylum*	*class*	*order*	*family*	*genus*	*species*
617	*Bacteroidetes*	*Bacteroidia*	*Bacteroidales*	*Tannerellaceae*	*Parabacteroides*	*Parabacteroides*
618	*Bacteroidetes*	*Bacteroidia*	*Bacteroidales*	*Tannerellaceae*	*Parabacteroides*	*Parabacteroides*
619	*Bacteroidetes*	*Bacteroidia*	*Bacteroidales*	*Tannerellaceae*	*Parabacteroides*	*Parabacteroides*
620	*Bacteroidetes*	*Bacteroidia*	*Bacteroidales*	*Tannerellaceae*	*Parabacteroides*	*Parabacteroides*
621	*Bacteroidetes*	*Bacteroidia*	*Bacteroidales*	*Tannerellaceae*	*Parabacteroides*	*Parabacteroides*
622	*Bacteroidetes*	*Bacteroidia*	*Bacteroidales*	*Tannerellaceae*	*Parabacteroides*	*Parabacteroides*
623	*Bacteroidetes*	*Bacteroidia*	*Bacteroidales*	*Tannerellaceae*	*Parabacteroides*	*Parabacteroides*
624	*Bacteroidetes*	*Bacteroidia*	*Bacteroidales*	*Tannerellaceae*	*Parabacteroides*	*Parabacteroides*
625	*Bacteroidetes*	*Bacteroidia*	*Bacteroidales*	*Tannerellaceae*	*Parabacteroides*	*Parabacteroides*
626	*Bacteroidetes*	*Bacteroidia*	*Bacteroidales*	*Tannerellaceae*	*Parabacteroides*	*Parabacteroides*
627	*Bacteroidetes*	*Bacteroidia*	*Bacteroidales*	*Tannerellaceae*	*Parabacteroides*	*Parabacteroides*
628	*Bacteroidetes*	*Bacteroidia*	*Bacteroidales*	*Tannerellaceae*	*Parabacteroides*	*Parabacteroides*
629	*Bacteroidetes*	*Bacteroidia*	*Bacteroidales*	*Tannerellaceae*	*Parabacteroides*	*Parabacteroides*
630	*Bacteroidetes*	*Bacteroidia*	*Bacteroidales*	*Tannerellaceae*	*Parabacteroides*	*Parabacteroides*
631	*Bacteroidetes*	*Bacteroidia*	*Bacteroidales*	*Tannerellaceae*	*Parabacteroides*	*Parabacteroides*
632	*Bacteroidetes*	*Bacteroidia*	*Bacteroidales*	*Tannerellaceae*	*Parabacteroides*	*Parabacteroides*
633	*Bacteroidetes*	*Bacteroidia*	*Bacteroidales*	*Tannerellaceae*	*Parabacteroides*	*Parabacteroides*
634	*Bacteroidetes*	*Bacteroidia*	*Bacteroidales*	*Tannerellaceae*	*Parabacteroides*	*Parabacteroides*
635	*Bacteroidetes*	*Bacteroidia*	*Bacteroidales*	*Tannerellaceae*	*Parabacteroides*	*Parabacteroides*
636	*Bacteroidetes*	*Bacteroidia*	*Bacteroidales*	*Tannerellaceae*	*Parabacteroides*	*Parabacteroides*
637	*Bacteroidetes*	*Flavobacteriia*	*Flavobacteriales*	*Flavobacteriaceae*	*Seonamhaeicola*	*Seonamhaeicola*
638	*Firmicutes*	*Bacilli*	*Bacillales*	*Bacillaceae*	*Bacillus*	*Bacillus altitudinis*
639	*Firmicutes*	*Bacilli*	*Bacillales*	*Bacillaceae*	*Bacillus*	*Bacillus altitudinis*
640	*Firmicutes*	*Bacilli*	*Bacillales*	*Bacillaceae*	*Bacillus*	*Bacillus altitudinis*
641	*Firmicutes*	*Bacilli*	*Bacillales*	*Bacillaceae*	*Bacillus*	*Bacillus altitudinis*
642	*Firmicutes*	*Bacilli*	*Bacillales*	*Bacillaceae*	*Bacillus*	*Bacillus altitudinis*
643	*Firmicutes*	*Bacilli*	*Bacillales*	*Bacillaceae*	*Bacillus*	*Bacillus altitudinis*
644	*Firmicutes*	*Bacilli*	*Bacillales*	*Bacillaceae*	*Bacillus*	*Bacillus altitudinis*

Table S4. Isolated strains by culturomics

bacteriaId	*phylum*	*class*	*order*	*family*	*genus*	*species*
645	*Firmicutes*	*Bacilli*	*Bacillales*	*Bacillaceae*	*Bacillus*	*Bacillus altitudinis*
646	*Firmicutes*	*Bacilli*	*Bacillales*	*Bacillaceae*	*Bacillus*	*Bacillus altitudinis*
647	*Firmicutes*	*Bacilli*	*Bacillales*	*Bacillaceae*	*Bacillus*	*Bacillus altitudinis*
648	*Firmicutes*	*Bacilli*	*Bacillales*	*Bacillaceae*	*Bacillus*	*Bacillus altitudinis*
649	*Firmicutes*	*Bacilli*	*Bacillales*	*Bacillaceae*	*Bacillus*	*Bacillus altitudinis*
650	*Firmicutes*	*Bacilli*	*Bacillales*	*Bacillaceae*	*Bacillus*	*Bacillus altitudinis*
651	*Firmicutes*	*Bacilli*	*Bacillales*	*Bacillaceae*	*Bacillus*	*Bacillus altitudinis*
652	*Firmicutes*	*Bacilli*	*Bacillales*	*Bacillaceae*	*Bacillus*	*Bacillus altitudinis*
653	*Firmicutes*	*Bacilli*	*Bacillales*	*Bacillaceae*	*Bacillus*	*Bacillus altitudinis*
654	*Firmicutes*	*Bacilli*	*Bacillales*	*Bacillaceae*	*Bacillus*	*Bacillus altitudinis*
655	*Firmicutes*	*Bacilli*	*Bacillales*	*Bacillaceae*	*Bacillus*	*Bacillus altitudinis*
656	*Firmicutes*	*Bacilli*	*Bacillales*	*Bacillaceae*	*Bacillus*	*Bacillus altitudinis*
657	*Firmicutes*	*Bacilli*	*Bacillales*	*Bacillaceae*	*Bacillus*	*Bacillus altitudinis*
658	*Firmicutes*	*Bacilli*	*Bacillales*	*Bacillaceae*	*Bacillus*	*Bacillus altitudinis*
659	*Firmicutes*	*Bacilli*	*Bacillales*	*Bacillaceae*	*Bacillus*	*Bacillus altitudinis*
660	*Firmicutes*	*Bacilli*	*Bacillales*	*Bacillaceae*	*Bacillus*	*Bacillus altitudinis*
661	*Firmicutes*	*Bacilli*	*Bacillales*	*Bacillaceae*	*Bacillus*	*Bacillus altitudinis*
662	*Firmicutes*	*Bacilli*	*Bacillales*	*Bacillaceae*	*Bacillus*	*Bacillus altitudinis*
663	*Firmicutes*	*Bacilli*	*Bacillales*	*Bacillaceae*	*Bacillus*	*Bacillus altitudinis*
664	*Firmicutes*	*Bacilli*	*Bacillales*	*Bacillaceae*	*Bacillus*	*Bacillus altitudinis*
665	*Firmicutes*	*Bacilli*	*Bacillales*	*Bacillaceae*	*Bacillus*	*Bacillus altitudinis*
666	*Firmicutes*	*Bacilli*	*Bacillales*	*Bacillaceae*	*Bacillus*	*Bacillus altitudinis*
667	*Firmicutes*	*Bacilli*	*Bacillales*	*Bacillaceae*	*Bacillus*	*Bacillus altitudinis*
668	*Firmicutes*	*Bacilli*	*Bacillales*	*Bacillaceae*	*Bacillus*	*Bacillus altitudinis*
669	*Firmicutes*	*Bacilli*	*Bacillales*	*Bacillaceae*	*Bacillus*	*Bacillus altitudinis*
670	*Firmicutes*	*Bacilli*	*Bacillales*	*Bacillaceae*	*Bacillus*	*Bacillus*
671	*Firmicutes*	*Bacilli*	*Bacillales*	*Bacillaceae*	*Bacillus*	*Bacillus*
672	*Firmicutes*	*Bacilli*	*Bacillales*	*Bacillaceae*	*Bacillus*	*Bacillus*

Table S4. Isolated strains by culturomics

bacteriaId	*phylum*	*class*	*order*	*family*	*genus*	*species*
673	*Firmicutes*	*Bacilli*	*Bacillales*	*Bacillaceae*	*Bacillus*	*Bacillus*
674	*Firmicutes*	*Bacilli*	*Bacillales*	*Bacillaceae*	*Bacillus*	*Bacillus badius*
675	*Firmicutes*	*Bacilli*	*Bacillales*	*Bacillaceae*	*Bacillus*	*Bacillus badius*
676	*Firmicutes*	*Bacilli*	*Bacillales*	*Bacillaceae*	*Bacillus*	*Bacillus badius*
677	*Firmicutes*	*Bacilli*	*Bacillales*	*Bacillaceae*	*Bacillus*	*Bacillus badius*
678	*Firmicutes*	*Bacilli*	*Bacillales*	*Bacillaceae*	*Bacillus*	*Bacillus badius*
679	*Firmicutes*	*Bacilli*	*Bacillales*	*Bacillaceae*	*Bacillus*	*Bacillus badius*
680	*Firmicutes*	*Bacilli*	*Bacillales*	*Bacillaceae*	*Bacillus*	*Bacillus badius*
681	*Firmicutes*	*Bacilli*	*Bacillales*	*Bacillaceae*	*Bacillus*	*Bacillus badius*
682	*Firmicutes*	*Bacilli*	*Bacillales*	*Bacillaceae*	*Bacillus*	*Bacillus badius*
683	*Firmicutes*	*Bacilli*	*Bacillales*	*Bacillaceae*	*Bacillus*	*Bacillus badius*
684	*Firmicutes*	*Bacilli*	*Bacillales*	*Bacillaceae*	*Bacillus*	*Bacillus badius*
685	*Firmicutes*	*Bacilli*	*Bacillales*	*Bacillaceae*	*Bacillus*	*Bacillus badius*
686	*Firmicutes*	*Bacilli*	*Bacillales*	*Bacillaceae*	*Bacillus*	*Bacillus badius*
687	*Firmicutes*	*Bacilli*	*Bacillales*	*Bacillaceae*	*Bacillus*	*Bacillus badius*
688	*Firmicutes*	*Bacilli*	*Bacillales*	*Bacillaceae*	*Bacillus*	*Bacillus badius*
689	*Firmicutes*	*Bacilli*	*Bacillales*	*Bacillaceae*	*Bacillus*	*Bacillus badius*
690	*Firmicutes*	*Bacilli*	*Bacillales*	*Bacillaceae*	*Bacillus*	*Bacillus badius*
691	*Firmicutes*	*Bacilli*	*Bacillales*	*Bacillaceae*	*Bacillus*	*Bacillus badius*
692	*Firmicutes*	*Bacilli*	*Bacillales*	*Bacillaceae*	*Bacillus*	*Bacillus badius*
693	*Firmicutes*	*Bacilli*	*Bacillales*	*Bacillaceae*	*Bacillus*	*Bacillus badius*
694	*Firmicutes*	*Bacilli*	*Bacillales*	*Bacillaceae*	*Bacillus*	*Bacillus badius*
695	*Firmicutes*	*Bacilli*	*Bacillales*	*Bacillaceae*	*Bacillus*	*Bacillus badius*
696	*Firmicutes*	*Bacilli*	*Bacillales*	*Bacillaceae*	*Bacillus*	*Bacillus badius*
697	*Firmicutes*	*Bacilli*	*Bacillales*	*Bacillaceae*	*Bacillus*	*Bacillus badius*
698	*Firmicutes*	*Bacilli*	*Bacillales*	*Bacillaceae*	*Bacillus*	*Bacillus badius*
699	*Firmicutes*	*Bacilli*	*Bacillales*	*Bacillaceae*	*Bacillus*	*Bacillus badius*
700	*Firmicutes*	*Bacilli*	*Bacillales*	*Bacillaceae*	*Bacillus*	*Bacillus cereus*

Table S4. Isolated strains by culturomics

bacteriaId	*phylum*	*class*	*order*	*family*	*genus*	*species*
701	*Firmicutes*	*Bacilli*	*Bacillales*	*Bacillaceae*	*Bacillus*	*Bacillus cereus*
702	*Firmicutes*	*Bacilli*	*Bacillales*	*Bacillaceae*	*Bacillus*	*Bacillus cereus*
703	*Firmicutes*	*Bacilli*	*Bacillales*	*Bacillaceae*	*Bacillus*	*Bacillus cereus*
704	*Firmicutes*	*Bacilli*	*Bacillales*	*Bacillaceae*	*Bacillus*	*Bacillus cereus*
705	*Firmicutes*	*Bacilli*	*Bacillales*	*Bacillaceae*	*Bacillus*	*Bacillus cereus*
706	*Firmicutes*	*Bacilli*	*Bacillales*	*Bacillaceae*	*Bacillus*	*Bacillus cereus*
707	*Firmicutes*	*Bacilli*	*Bacillales*	*Bacillaceae*	*Bacillus*	*Bacillus cereus*
708	*Firmicutes*	*Bacilli*	*Bacillales*	*Bacillaceae*	*Bacillus*	*Bacillus cereus*
709	*Firmicutes*	*Bacilli*	*Bacillales*	*Bacillaceae*	*Bacillus*	*Bacillus cereus*
710	*Firmicutes*	*Bacilli*	*Bacillales*	*Bacillaceae*	*Bacillus*	*Bacillus cereus*
711	*Firmicutes*	*Bacilli*	*Bacillales*	*Bacillaceae*	*Bacillus*	*Bacillus cereus*
712	*Firmicutes*	*Bacilli*	*Bacillales*	*Bacillaceae*	*Bacillus*	*Bacillus cereus*
713	*Firmicutes*	*Bacilli*	*Bacillales*	*Bacillaceae*	*Bacillus*	*Bacillus cereus*
714	*Firmicutes*	*Bacilli*	*Bacillales*	*Bacillaceae*	*Bacillus*	*Bacillus cereus*
715	*Firmicutes*	*Bacilli*	*Bacillales*	*Bacillaceae*	*Bacillus*	*Bacillus cereus*
716	*Firmicutes*	*Bacilli*	*Bacillales*	*Bacillaceae*	*Bacillus*	*Bacillus cereus*
717	*Firmicutes*	*Bacilli*	*Bacillales*	*Bacillaceae*	*Bacillus*	*Bacillus cereus*
718	*Firmicutes*	*Bacilli*	*Bacillales*	*Bacillaceae*	*Bacillus*	*Bacillus cereus*
719	*Firmicutes*	*Bacilli*	*Bacillales*	*Bacillaceae*	*Bacillus*	*Bacillus cereus*
720	*Firmicutes*	*Bacilli*	*Bacillales*	*Bacillaceae*	*Bacillus*	*Bacillus cereus*
721	*Firmicutes*	*Bacilli*	*Bacillales*	*Bacillaceae*	*Bacillus*	*Bacillus cereus*
722	*Firmicutes*	*Bacilli*	*Bacillales*	*Bacillaceae*	*Bacillus*	*Bacillus cereus*
723	*Firmicutes*	*Bacilli*	*Bacillales*	*Bacillaceae*	*Bacillus*	*Bacillus cereus*
724	*Firmicutes*	*Bacilli*	*Bacillales*	*Bacillaceae*	*Bacillus*	*Bacillus cereus*
725	*Firmicutes*	*Bacilli*	*Bacillales*	*Bacillaceae*	*Bacillus*	*Bacillus cereus*
726	*Firmicutes*	*Bacilli*	*Bacillales*	*Bacillaceae*	*Bacillus*	*Bacillus cereus*
727	*Firmicutes*	*Bacilli*	*Bacillales*	*Bacillaceae*	*Bacillus*	*Bacillus cereus*
728	*Firmicutes*	*Bacilli*	*Bacillales*	*Bacillaceae*	*Bacillus*	*Bacillus cereus*

Table S4. Isolated strains by culturomics

bacteriaId	*phylum*	*class*	*order*	*family*	*genus*	*species*
729	*Firmicutes*	*Bacilli*	*Bacillales*	*Bacillaceae*	*Bacillus*	*Bacillus cereus*
730	*Firmicutes*	*Bacilli*	*Bacillales*	*Bacillaceae*	*Bacillus*	*Bacillus cereus*
731	*Firmicutes*	*Bacilli*	*Bacillales*	*Bacillaceae*	*Bacillus*	*Bacillus cereus*
732	*Firmicutes*	*Bacilli*	*Bacillales*	*Bacillaceae*	*Bacillus*	*Bacillus cereus*
733	*Firmicutes*	*Bacilli*	*Bacillales*	*Bacillaceae*	*Bacillus*	*Bacillus cereus*
734	*Firmicutes*	*Bacilli*	*Bacillales*	*Bacillaceae*	*Bacillus*	*Bacillus cereus*
735	*Firmicutes*	*Bacilli*	*Bacillales*	*Bacillaceae*	*Bacillus*	*Bacillus cereus*
736	*Firmicutes*	*Bacilli*	*Bacillales*	*Bacillaceae*	*Bacillus*	*Bacillus cereus*
737	*Firmicutes*	*Bacilli*	*Bacillales*	*Bacillaceae*	*Bacillus*	*Bacillus cereus*
738	*Firmicutes*	*Bacilli*	*Bacillales*	*Bacillaceae*	*Bacillus*	*Bacillus cereus*
739	*Firmicutes*	*Bacilli*	*Bacillales*	*Bacillaceae*	*Bacillus*	*Bacillus cereus*
740	*Firmicutes*	*Bacilli*	*Bacillales*	*Bacillaceae*	*Bacillus*	*Bacillus cereus*
741	*Firmicutes*	*Bacilli*	*Bacillales*	*Bacillaceae*	*Bacillus*	*Bacillus cereus*
742	*Firmicutes*	*Bacilli*	*Bacillales*	*Bacillaceae*	*Bacillus*	*Bacillus cereus*
743	*Firmicutes*	*Bacilli*	*Bacillales*	*Bacillaceae*	*Bacillus*	*Bacillus cereus*
744	*Firmicutes*	*Bacilli*	*Bacillales*	*Bacillaceae*	*Bacillus*	*Bacillus cereus*
745	*Firmicutes*	*Bacilli*	*Bacillales*	*Bacillaceae*	*Bacillus*	*Bacillus cereus*
746	*Firmicutes*	*Bacilli*	*Bacillales*	*Bacillaceae*	*Bacillus*	*Bacillus cereus*
747	*Firmicutes*	*Bacilli*	*Bacillales*	*Bacillaceae*	*Bacillus*	*Bacillus cereus*
748	*Firmicutes*	*Bacilli*	*Bacillales*	*Bacillaceae*	*Bacillus*	*Bacillus cereus*
749	*Firmicutes*	*Bacilli*	*Bacillales*	*Bacillaceae*	*Bacillus*	*Bacillus cereus*
750	*Firmicutes*	*Bacilli*	*Bacillales*	*Bacillaceae*	*Bacillus*	*Bacillus cereus*
751	*Firmicutes*	*Bacilli*	*Bacillales*	*Bacillaceae*	*Bacillus*	*Bacillus cereus*
752	*Firmicutes*	*Bacilli*	*Bacillales*	*Bacillaceae*	*Bacillus*	*Bacillus cereus*
753	*Firmicutes*	*Bacilli*	*Bacillales*	*Bacillaceae*	*Bacillus*	*Bacillus cereus*
754	*Firmicutes*	*Bacilli*	*Bacillales*	*Bacillaceae*	*Bacillus*	*Bacillus cereus*
755	*Firmicutes*	*Bacilli*	*Bacillales*	*Bacillaceae*	*Bacillus*	*Bacillus cereus*
756	*Firmicutes*	*Bacilli*	*Bacillales*	*Bacillaceae*	*Bacillus*	*Bacillus cereus*

Table S4. Isolated strains by culturomics

bacteriaId	*phylum*	*class*	*order*	*family*	*genus*	*species*
757	*Firmicutes*	*Bacilli*	*Bacillales*	*Bacillaceae*	*Bacillus*	*Bacillus cereus*
758	*Firmicutes*	*Bacilli*	*Bacillales*	*Bacillaceae*	*Bacillus*	*Bacillus cereus*
759	*Firmicutes*	*Bacilli*	*Bacillales*	*Bacillaceae*	*Bacillus*	*Bacillus cereus*
760	*Firmicutes*	*Bacilli*	*Bacillales*	*Bacillaceae*	*Bacillus*	*Bacillus cereus*
761	*Firmicutes*	*Bacilli*	*Bacillales*	*Bacillaceae*	*Bacillus*	*Bacillus cereus*
762	*Firmicutes*	*Bacilli*	*Bacillales*	*Bacillaceae*	*Bacillus*	*Bacillus cereus*
763	*Firmicutes*	*Bacilli*	*Bacillales*	*Bacillaceae*	*Bacillus*	*Bacillus cereus*
764	*Firmicutes*	*Bacilli*	*Bacillales*	*Bacillaceae*	*Bacillus*	*Bacillus cereus*
765	*Firmicutes*	*Bacilli*	*Bacillales*	*Bacillaceae*	*Bacillus*	*Bacillus cereus*
766	*Firmicutes*	*Bacilli*	*Bacillales*	*Bacillaceae*	*Bacillus*	*Bacillus cereus*
767	*Firmicutes*	*Bacilli*	*Bacillales*	*Bacillaceae*	*Bacillus*	*Bacillus cereus*
768	*Firmicutes*	*Bacilli*	*Bacillales*	*Bacillaceae*	*Bacillus*	*Bacillus cereus*
769	*Firmicutes*	*Bacilli*	*Bacillales*	*Bacillaceae*	*Bacillus*	*Bacillus cereus*
770	*Firmicutes*	*Bacilli*	*Bacillales*	*Bacillaceae*	*Bacillus*	*Bacillus cereus*
771	*Firmicutes*	*Bacilli*	*Bacillales*	*Bacillaceae*	*Bacillus*	*Bacillus cereus*
772	*Firmicutes*	*Bacilli*	*Bacillales*	*Bacillaceae*	*Bacillus*	*Bacillus cereus*
773	*Firmicutes*	*Bacilli*	*Bacillales*	*Bacillaceae*	*Bacillus*	*Bacillus cereus*
774	*Firmicutes*	*Bacilli*	*Bacillales*	*Bacillaceae*	*Bacillus*	*Bacillus cereus*
775	*Firmicutes*	*Bacilli*	*Bacillales*	*Bacillaceae*	*Bacillus*	*Bacillus cereus*
776	*Firmicutes*	*Bacilli*	*Bacillales*	*Bacillaceae*	*Bacillus*	*Bacillus cereus*
777	*Firmicutes*	*Bacilli*	*Bacillales*	*Bacillaceae*	*Bacillus*	*Bacillus cereus*
778	*Firmicutes*	*Bacilli*	*Bacillales*	*Bacillaceae*	*Bacillus*	*Bacillus cereus*
779	*Firmicutes*	*Bacilli*	*Bacillales*	*Bacillaceae*	*Bacillus*	*Bacillus cereus*
780	*Firmicutes*	*Bacilli*	*Bacillales*	*Bacillaceae*	*Bacillus*	*Bacillus cereus*
781	*Firmicutes*	*Bacilli*	*Bacillales*	*Bacillaceae*	*Bacillus*	*Bacillus cereus*
782	*Firmicutes*	*Bacilli*	*Bacillales*	*Bacillaceae*	*Bacillus*	*Bacillus cereus*
783	*Firmicutes*	*Bacilli*	*Bacillales*	*Bacillaceae*	*Bacillus*	*Bacillus cereus*
784	*Firmicutes*	*Bacilli*	*Bacillales*	*Bacillaceae*	*Bacillus*	*Bacillus cereus*

Table S4. Isolated strains by culturomics

bacteriaId	*phylum*	*class*	*order*	*family*	*genus*	*species*
785	*Firmicutes*	*Bacilli*	*Bacillales*	*Bacillaceae*	*Bacillus*	*Bacillus cereus*
786	*Firmicutes*	*Bacilli*	*Bacillales*	*Bacillaceae*	*Bacillus*	*Bacillus cereus*
787	*Firmicutes*	*Bacilli*	*Bacillales*	*Bacillaceae*	*Bacillus*	*Bacillus cereus*
788	*Firmicutes*	*Bacilli*	*Bacillales*	*Bacillaceae*	*Bacillus*	*Bacillus cereus*
789	*Firmicutes*	*Bacilli*	*Bacillales*	*Bacillaceae*	*Bacillus*	*Bacillus cereus*
790	*Firmicutes*	*Bacilli*	*Bacillales*	*Bacillaceae*	*Bacillus*	*Bacillus cereus*
791	*Firmicutes*	*Bacilli*	*Bacillales*	*Bacillaceae*	*Bacillus*	*Bacillus cereus*
792	*Firmicutes*	*Bacilli*	*Bacillales*	*Bacillaceae*	*Bacillus*	*Bacillus cereus*
793	*Firmicutes*	*Bacilli*	*Bacillales*	*Bacillaceae*	*Bacillus*	*Bacillus cereus*
794	*Firmicutes*	*Bacilli*	*Bacillales*	*Bacillaceae*	*Bacillus*	*Bacillus coagulans*
795	*Firmicutes*	*Bacilli*	*Bacillales*	*Bacillaceae*	*Bacillus*	*Bacillus firmus*
796	*Firmicutes*	*Bacilli*	*Bacillales*	*Bacillaceae*	*Bacillus*	*Bacillus flexus*
797	*Firmicutes*	*Bacilli*	*Bacillales*	*Bacillaceae*	*Bacillus*	*Bacillus flexus*
798	*Firmicutes*	*Bacilli*	*Bacillales*	*Bacillaceae*	*Bacillus*	*Bacillus flexus*
799	*Firmicutes*	*Bacilli*	*Bacillales*	*Bacillaceae*	*Bacillus*	*Bacillus infantis*
800	*Firmicutes*	*Bacilli*	*Bacillales*	*Bacillaceae*	*Bacillus*	*Bacillus*
801	*Firmicutes*	*Bacilli*	*Bacillales*	*Bacillaceae*	*Bacillus*	*Bacillus*
802	*Firmicutes*	*Bacilli*	*Bacillales*	*Bacillaceae*	*Bacillus*	*Bacillus*
803	*Firmicutes*	*Bacilli*	*Bacillales*	*Bacillaceae*	*Bacillus*	*Bacillus*
804	*Firmicutes*	*Bacilli*	*Bacillales*	*Bacillaceae*	*Bacillus*	*Bacillus*
805	*Firmicutes*	*Bacilli*	*Bacillales*	*Bacillaceae*	*Bacillus*	*Bacillus*
806	*Firmicutes*	*Bacilli*	*Bacillales*	*Bacillaceae*	*Bacillus*	*Bacillus*
807	*Firmicutes*	*Bacilli*	*Bacillales*	*Bacillaceae*	*Bacillus*	*Bacillus*
808	*Firmicutes*	*Bacilli*	*Bacillales*	*Bacillaceae*	*Bacillus*	*Bacillus*
809	*Firmicutes*	*Bacilli*	*Bacillales*	*Bacillaceae*	*Bacillus*	*Bacillus*
810	*Firmicutes*	*Bacilli*	*Bacillales*	*Bacillaceae*	*Bacillus*	*Bacillus*
811	*Firmicutes*	*Bacilli*	*Bacillales*	*Bacillaceae*	*Bacillus*	*Bacillus*
812	*Firmicutes*	*Bacilli*	*Bacillales*	*Bacillaceae*	*Bacillus*	*Bacillus*

Table S4. Isolated strains by culturomics

bacteriaId	*phylum*	*class*	*order*	*family*	*genus*	*species*
813	*Firmicutes*	*Bacilli*	*Bacillales*	*Bacillaceae*	*Bacillus*	*Bacillus*
814	*Firmicutes*	*Bacilli*	*Bacillales*	*Bacillaceae*	*Bacillus*	*Bacillus*
815	*Firmicutes*	*Bacilli*	*Bacillales*	*Bacillaceae*	*Bacillus*	*Bacillus*
816	*Firmicutes*	*Bacilli*	*Bacillales*	*Bacillaceae*	*Bacillus*	*Bacillus*
817	*Firmicutes*	*Bacilli*	*Bacillales*	*Bacillaceae*	*Bacillus*	*Bacillus*
818	*Firmicutes*	*Bacilli*	*Bacillales*	*Bacillaceae*	*Bacillus*	*Bacillus*
819	*Firmicutes*	*Bacilli*	*Bacillales*	*Bacillaceae*	*Bacillus*	*Bacillus*
820	*Firmicutes*	*Bacilli*	*Bacillales*	*Bacillaceae*	*Bacillus*	*Bacillus*
821	*Firmicutes*	*Bacilli*	*Bacillales*	*Bacillaceae*	*Bacillus*	*Bacillus*
822	*Firmicutes*	*Bacilli*	*Bacillales*	*Bacillaceae*	*Bacillus*	*Bacillus*
823	*Firmicutes*	*Bacilli*	*Bacillales*	*Bacillaceae*	*Bacillus*	*Bacillus*
824	*Firmicutes*	*Bacilli*	*Bacillales*	*Bacillaceae*	*Bacillus*	*Bacillus*
825	*Firmicutes*	*Bacilli*	*Bacillales*	*Bacillaceae*	*Bacillus*	*Bacillus*
826	*Firmicutes*	*Bacilli*	*Bacillales*	*Bacillaceae*	*Bacillus*	*Bacillus*
827	*Firmicutes*	*Bacilli*	*Bacillales*	*Bacillaceae*	*Bacillus*	*Bacillus*
828	*Firmicutes*	*Bacilli*	*Bacillales*	*Bacillaceae*	*Bacillus*	*Bacillus*
829	*Firmicutes*	*Bacilli*	*Bacillales*	*Bacillaceae*	*Bacillus*	*Bacillus*
830	*Firmicutes*	*Bacilli*	*Bacillales*	*Bacillaceae*	*Bacillus*	*Bacillus*
831	*Firmicutes*	*Bacilli*	*Bacillales*	*Bacillaceae*	*Bacillus*	*Bacillus*
832	*Firmicutes*	*Bacilli*	*Bacillales*	*Bacillaceae*	*Bacillus*	*Bacillus*
833	*Firmicutes*	*Bacilli*	*Bacillales*	*Bacillaceae*	*Bacillus*	*Bacillus*
834	*Firmicutes*	*Bacilli*	*Bacillales*	*Bacillaceae*	*Bacillus*	*Bacillus*
835	*Firmicutes*	*Bacilli*	*Bacillales*	*Bacillaceae*	*Bacillus*	*Bacillus*
836	*Firmicutes*	*Bacilli*	*Bacillales*	*Bacillaceae*	*Bacillus*	*Bacillus*
837	*Firmicutes*	*Bacilli*	*Bacillales*	*Bacillaceae*	*Bacillus*	*Bacillus*
838	*Firmicutes*	*Bacilli*	*Bacillales*	*Bacillaceae*	*Bacillus*	*Bacillus*
839	*Firmicutes*	*Bacilli*	*Bacillales*	*Bacillaceae*	*Bacillus*	*Bacillus*
840	*Firmicutes*	*Bacilli*	*Bacillales*	*Bacillaceae*	*Bacillus*	*Bacillus*

Table S4. Isolated strains by culturomics

bacteriaId	*phylum*	*class*	*order*	*family*	*genus*	*species*
841	*Firmicutes*	*Bacilli*	*Bacillales*	*Bacillaceae*	*Bacillus*	*Bacillus*
842	*Firmicutes*	*Bacilli*	*Bacillales*	*Bacillaceae*	*Bacillus*	*Bacillus*
843	*Firmicutes*	*Bacilli*	*Bacillales*	*Bacillaceae*	*Bacillus*	*Bacillus*
844	*Firmicutes*	*Bacilli*	*Bacillales*	*Bacillaceae*	*Bacillus*	*Bacillus*
845	*Firmicutes*	*Bacilli*	*Bacillales*	*Bacillaceae*	*Bacillus*	*Bacillus*
846	*Firmicutes*	*Bacilli*	*Bacillales*	*Bacillaceae*	*Bacillus*	*Bacillus*
847	*Firmicutes*	*Bacilli*	*Bacillales*	*Bacillaceae*	*Bacillus*	*Bacillus*
848	*Firmicutes*	*Bacilli*	*Bacillales*	*Bacillaceae*	*Bacillus*	*Bacillus*
849	*Firmicutes*	*Bacilli*	*Bacillales*	*Bacillaceae*	*Bacillus*	*Bacillus*
850	*Firmicutes*	*Bacilli*	*Bacillales*	*Bacillaceae*	*Bacillus*	*Bacillus*
851	*Firmicutes*	*Bacilli*	*Bacillales*	*Bacillaceae*	*Bacillus*	*Bacillus*
852	*Firmicutes*	*Bacilli*	*Bacillales*	*Bacillaceae*	*Bacillus*	*Bacillus*
853	*Firmicutes*	*Bacilli*	*Bacillales*	*Bacillaceae*	*Bacillus*	*Bacillus*
854	*Firmicutes*	*Bacilli*	*Bacillales*	*Bacillaceae*	*Bacillus*	*Bacillus*
855	*Firmicutes*	*Bacilli*	*Bacillales*	*Bacillaceae*	*Bacillus*	*Bacillus*
856	*Firmicutes*	*Bacilli*	*Bacillales*	*Bacillaceae*	*Bacillus*	*Bacillus*
857	*Firmicutes*	*Bacilli*	*Bacillales*	*Bacillaceae*	*Bacillus*	*Bacillus*
858	*Firmicutes*	*Bacilli*	*Bacillales*	*Bacillaceae*	*Bacillus*	*Bacillus*
859	*Firmicutes*	*Bacilli*	*Bacillales*	*Bacillaceae*	*Bacillus*	*Bacillus*
860	*Firmicutes*	*Bacilli*	*Bacillales*	*Bacillaceae*	*Bacillus*	*Bacillus*
861	*Firmicutes*	*Bacilli*	*Bacillales*	*Bacillaceae*	*Bacillus*	*Bacillus*
862	*Firmicutes*	*Bacilli*	*Bacillales*	*Bacillaceae*	*Bacillus*	*Bacillus*
863	*Firmicutes*	*Bacilli*	*Bacillales*	*Bacillaceae*	*Bacillus*	*Bacillus*
864	*Firmicutes*	*Bacilli*	*Bacillales*	*Bacillaceae*	*Bacillus*	*Bacillus mojavensis*
865	*Firmicutes*	*Bacilli*	*Bacillales*	*Bacillaceae*	*Bacillus*	*Bacillus mojavensis*
866	*Firmicutes*	*Bacilli*	*Bacillales*	*Bacillaceae*	*Bacillus*	*Bacillus mojavensis*
867	*Firmicutes*	*Bacilli*	*Bacillales*	*Bacillaceae*	*Bacillus*	*Bacillus*
868	*Firmicutes*	*Bacilli*	*Bacillales*	*Bacillaceae*	*Bacillus*	*Bacillus pumilus*

Table S4. Isolated strains by culturomics

bacteriaId	*phylum*	*class*	*order*	*family*	*genus*	*species*
869	*Firmicutes*	*Bacilli*	*Bacillales*	*Bacillaceae*	*Bacillus*	*Bacillus pumilus*
870	*Firmicutes*	*Bacilli*	*Bacillales*	*Bacillaceae*	*Bacillus*	*Bacillus pumilus*
871	*Firmicutes*	*Bacilli*	*Bacillales*	*Bacillaceae*	*Bacillus*	*Bacillus pumilus*
872	*Firmicutes*	*Bacilli*	*Bacillales*	*Bacillaceae*	*Bacillus*	*Bacillus pumilus*
873	*Firmicutes*	*Bacilli*	*Bacillales*	*Bacillaceae*	*Bacillus*	*Bacillus pumilus*
874	*Firmicutes*	*Bacilli*	*Bacillales*	*Bacillaceae*	*Bacillus*	*Bacillus pumilus*
875	*Firmicutes*	*Bacilli*	*Bacillales*	*Bacillaceae*	*Bacillus*	*Bacillus pumilus*
876	*Firmicutes*	*Bacilli*	*Bacillales*	*Bacillaceae*	*Bacillus*	*Bacillus pumilus*
877	*Firmicutes*	*Bacilli*	*Bacillales*	*Bacillaceae*	*Bacillus*	*Bacillus pumilus*
878	*Firmicutes*	*Bacilli*	*Bacillales*	*Bacillaceae*	*Bacillus*	*Bacillus pumilus*
879	*Firmicutes*	*Bacilli*	*Bacillales*	*Bacillaceae*	*Bacillus*	*Bacillus pumilus*
880	*Firmicutes*	*Bacilli*	*Bacillales*	*Bacillaceae*	*Bacillus*	*Bacillus pumilus*
881	*Firmicutes*	*Bacilli*	*Bacillales*	*Bacillaceae*	*Bacillus*	*Bacillus pumilus*
882	*Firmicutes*	*Bacilli*	*Bacillales*	*Bacillaceae*	*Bacillus*	*Bacillus pumilus*
883	*Firmicutes*	*Bacilli*	*Bacillales*	*Bacillaceae*	*Bacillus*	*Bacillus pumilus*
884	*Firmicutes*	*Bacilli*	*Bacillales*	*Bacillaceae*	*Bacillus*	*Bacillus pumilus*
885	*Firmicutes*	*Bacilli*	*Bacillales*	*Bacillaceae*	*Bacillus*	*Bacillus pumilus*
886	*Firmicutes*	*Bacilli*	*Bacillales*	*Bacillaceae*	*Bacillus*	*Bacillus pumilus*
887	*Firmicutes*	*Bacilli*	*Bacillales*	*Bacillaceae*	*Bacillus*	*Bacillus pumilus*
888	*Firmicutes*	*Bacilli*	*Bacillales*	*Bacillaceae*	*Bacillus*	*Bacillus pumilus*
889	*Firmicutes*	*Bacilli*	*Bacillales*	*Bacillaceae*	*Bacillus*	*Bacillus pumilus*
890	*Firmicutes*	*Bacilli*	*Bacillales*	*Bacillaceae*	*Bacillus*	*Bacillus pumilus*
891	*Firmicutes*	*Bacilli*	*Bacillales*	*Bacillaceae*	*Bacillus*	*Bacillus pumilus*
892	*Firmicutes*	*Bacilli*	*Bacillales*	*Bacillaceae*	*Bacillus*	*Bacillus pumilus*
893	*Firmicutes*	*Bacilli*	*Bacillales*	*Bacillaceae*	*Bacillus*	*Bacillus pumilus*
894	*Firmicutes*	*Bacilli*	*Bacillales*	*Bacillaceae*	*Bacillus*	*Bacillus pumilus*
895	*Firmicutes*	*Bacilli*	*Bacillales*	*Bacillaceae*	*Bacillus*	*Bacillus pumilus*
896	*Firmicutes*	*Bacilli*	*Bacillales*	*Bacillaceae*	*Bacillus*	*Bacillus pumilus*

Table S4. Isolated strains by culturomics

bacteriaId	*phylum*	*class*	*order*	*family*	*genus*	*species*
897	*Firmicutes*	*Bacilli*	*Bacillales*	*Bacillaceae*	*Bacillus*	*Bacillus pumilus*
898	*Firmicutes*	*Bacilli*	*Bacillales*	*Bacillaceae*	*Bacillus*	*Bacillus pumilus*
899	*Firmicutes*	*Bacilli*	*Bacillales*	*Bacillaceae*	*Bacillus*	*Bacillus pumilus*
900	*Firmicutes*	*Bacilli*	*Bacillales*	*Bacillaceae*	*Bacillus*	*Bacillus pumilus*
901	*Firmicutes*	*Bacilli*	*Bacillales*	*Bacillaceae*	*Bacillus*	*Bacillus pumilus*
902	*Firmicutes*	*Bacilli*	*Bacillales*	*Bacillaceae*	*Bacillus*	*Bacillus pumilus*
903	*Firmicutes*	*Bacilli*	*Bacillales*	*Bacillaceae*	*Bacillus*	*Bacillus pumilus*
904	*Firmicutes*	*Bacilli*	*Bacillales*	*Bacillaceae*	*Bacillus*	*Bacillus pumilus*
905	*Firmicutes*	*Bacilli*	*Bacillales*	*Bacillaceae*	*Bacillus*	*Bacillus pumilus*
906	*Firmicutes*	*Bacilli*	*Bacillales*	*Bacillaceae*	*Bacillus*	*Bacillus pumilus*
907	*Firmicutes*	*Bacilli*	*Bacillales*	*Bacillaceae*	*Bacillus*	*Bacillus pumilus*
908	*Firmicutes*	*Bacilli*	*Bacillales*	*Bacillaceae*	*Bacillus*	*Bacillus pumilus*
909	*Firmicutes*	*Bacilli*	*Bacillales*	*Bacillaceae*	*Bacillus*	*Bacillus pumilus*
910	*Firmicutes*	*Bacilli*	*Bacillales*	*Bacillaceae*	*Bacillus*	*Bacillus pumilus*
911	*Firmicutes*	*Bacilli*	*Bacillales*	*Bacillaceae*	*Bacillus*	*Bacillus pumilus*
912	*Firmicutes*	*Bacilli*	*Bacillales*	*Bacillaceae*	*Bacillus*	*Bacillus pumilus*
913	*Firmicutes*	*Bacilli*	*Bacillales*	*Bacillaceae*	*Bacillus*	*Bacillus pumilus*
914	*Firmicutes*	*Bacilli*	*Bacillales*	*Bacillaceae*	*Bacillus*	*Bacillus pumilus*
915	*Firmicutes*	*Bacilli*	*Bacillales*	*Bacillaceae*	*Bacillus*	*Bacillus pumilus*
916	*Firmicutes*	*Bacilli*	*Bacillales*	*Bacillaceae*	*Bacillus*	*Bacillus pumilus*
917	*Firmicutes*	*Bacilli*	*Bacillales*	*Bacillaceae*	*Bacillus*	*Bacillus pumilus*
918	*Firmicutes*	*Bacilli*	*Bacillales*	*Bacillaceae*	*Bacillus*	*Bacillus pumilus*
919	*Firmicutes*	*Bacilli*	*Bacillales*	*Bacillaceae*	*Bacillus*	*Bacillus pumilus*
920	*Firmicutes*	*Bacilli*	*Bacillales*	*Bacillaceae*	*Bacillus*	*Bacillus pumilus*
921	*Firmicutes*	*Bacilli*	*Bacillales*	*Bacillaceae*	*Bacillus*	*Bacillus pumilus*
922	*Firmicutes*	*Bacilli*	*Bacillales*	*Bacillaceae*	*Bacillus*	*Bacillus pumilus*
923	*Firmicutes*	*Bacilli*	*Bacillales*	*Bacillaceae*	*Bacillus*	*Bacillus pumilus*
924	*Firmicutes*	*Bacilli*	*Bacillales*	*Bacillaceae*	*Bacillus*	*Bacillus pumilus*

Table S4. Isolated strains by culturomics

bacteriaId	*phylum*	*class*	*order*	*family*	*genus*	*species*
925	*Firmicutes*	*Bacilli*	*Bacillales*	*Bacillaceae*	*Bacillus*	*Bacillus pumilus*
926	*Firmicutes*	*Bacilli*	*Bacillales*	*Bacillaceae*	*Bacillus*	*Bacillus pumilus*
927	*Firmicutes*	*Bacilli*	*Bacillales*	*Bacillaceae*	*Bacillus*	*Bacillus pumilus*
928	*Firmicutes*	*Bacilli*	*Bacillales*	*Bacillaceae*	*Bacillus*	*Bacillus pumilus*
929	*Firmicutes*	*Bacilli*	*Bacillales*	*Bacillaceae*	*Bacillus*	*Bacillus pumilus*
930	*Firmicutes*	*Bacilli*	*Bacillales*	*Bacillaceae*	*Bacillus*	*Bacillus pumilus*
931	*Firmicutes*	*Bacilli*	*Bacillales*	*Bacillaceae*	*Bacillus*	*Bacillus pumilus*
932	*Firmicutes*	*Bacilli*	*Bacillales*	*Bacillaceae*	*Bacillus*	*Bacillus pumilus*
933	*Firmicutes*	*Bacilli*	*Bacillales*	*Bacillaceae*	*Bacillus*	*Bacillus pumilus*
934	*Firmicutes*	*Bacilli*	*Bacillales*	*Bacillaceae*	*Bacillus*	*Bacillus pumilus*
935	*Firmicutes*	*Bacilli*	*Bacillales*	*Bacillaceae*	*Bacillus*	*Bacillus pumilus*
936	*Firmicutes*	*Bacilli*	*Bacillales*	*Bacillaceae*	*Bacillus*	*Bacillus pumilus*
937	*Firmicutes*	*Bacilli*	*Bacillales*	*Bacillaceae*	*Bacillus*	*Bacillus pumilus*
938	*Firmicutes*	*Bacilli*	*Bacillales*	*Bacillaceae*	*Bacillus*	*Bacillus pumilus*
939	*Firmicutes*	*Bacilli*	*Bacillales*	*Bacillaceae*	*Bacillus*	*Bacillus pumilus*
940	*Firmicutes*	*Bacilli*	*Bacillales*	*Bacillaceae*	*Bacillus*	*Bacillus pumilus*
941	*Firmicutes*	*Bacilli*	*Bacillales*	*Bacillaceae*	*Bacillus*	*Bacillus pumilus*
942	*Firmicutes*	*Bacilli*	*Bacillales*	*Bacillaceae*	*Bacillus*	*Bacillus pumilus*
943	*Firmicutes*	*Bacilli*	*Bacillales*	*Bacillaceae*	*Bacillus*	*Bacillus pumilus*
944	*Firmicutes*	*Bacilli*	*Bacillales*	*Bacillaceae*	*Bacillus*	*Bacillus pumilus*
945	*Firmicutes*	*Bacilli*	*Bacillales*	*Bacillaceae*	*Bacillus*	*Bacillus pumilus*
946	*Firmicutes*	*Bacilli*	*Bacillales*	*Bacillaceae*	*Bacillus*	*Bacillus pumilus*
947	*Firmicutes*	*Bacilli*	*Bacillales*	*Bacillaceae*	*Bacillus*	*Bacillus pumilus*
948	*Firmicutes*	*Bacilli*	*Bacillales*	*Bacillaceae*	*Bacillus*	*Bacillus pumilus*
949	*Firmicutes*	*Bacilli*	*Bacillales*	*Bacillaceae*	*Bacillus*	*Bacillus pumilus*
950	*Firmicutes*	*Bacilli*	*Bacillales*	*Bacillaceae*	*Bacillus*	*Bacillus pumilus*
951	*Firmicutes*	*Bacilli*	*Bacillales*	*Bacillaceae*	*Bacillus*	*Bacillus pumilus*
952	*Firmicutes*	*Bacilli*	*Bacillales*	*Bacillaceae*	*Bacillus*	*Bacillus pumilus*

Table S4. Isolated strains by culturomics

bacteriaId	*phylum*	*class*	*order*	*family*	*genus*	*species*
953	*Firmicutes*	*Bacilli*	*Bacillales*	*Bacillaceae*	*Bacillus*	*Bacillus pumilus*
954	*Firmicutes*	*Bacilli*	*Bacillales*	*Bacillaceae*	*Bacillus*	*Bacillus pumilus*
955	*Firmicutes*	*Bacilli*	*Bacillales*	*Bacillaceae*	*Bacillus*	*Bacillus pumilus*
956	*Firmicutes*	*Bacilli*	*Bacillales*	*Bacillaceae*	*Bacillus*	*Bacillus pumilus*
957	*Firmicutes*	*Bacilli*	*Bacillales*	*Bacillaceae*	*Bacillus*	*Bacillus pumilus*
958	*Firmicutes*	*Bacilli*	*Bacillales*	*Bacillaceae*	*Bacillus*	*Bacillus pumilus*
959	*Firmicutes*	*Bacilli*	*Bacillales*	*Bacillaceae*	*Bacillus*	*Bacillus pumilus*
960	*Firmicutes*	*Bacilli*	*Bacillales*	*Bacillaceae*	*Bacillus*	*Bacillus pumilus*
961	*Firmicutes*	*Bacilli*	*Bacillales*	*Bacillaceae*	*Bacillus*	*Bacillus pumilus*
962	*Firmicutes*	*Bacilli*	*Bacillales*	*Bacillaceae*	*Bacillus*	*Bacillus pumilus*
963	*Firmicutes*	*Bacilli*	*Bacillales*	*Bacillaceae*	*Bacillus*	*Bacillus pumilus*
964	*Firmicutes*	*Bacilli*	*Bacillales*	*Bacillaceae*	*Bacillus*	*Bacillus pumilus*
965	*Firmicutes*	*Bacilli*	*Bacillales*	*Bacillaceae*	*Bacillus*	*Bacillus pumilus*
966	*Firmicutes*	*Bacilli*	*Bacillales*	*Bacillaceae*	*Bacillus*	*Bacillus pumilus*
967	*Firmicutes*	*Bacilli*	*Bacillales*	*Bacillaceae*	*Bacillus*	*Bacillus pumilus*
968	*Firmicutes*	*Bacilli*	*Bacillales*	*Bacillaceae*	*Bacillus*	*Bacillus pumilus*
969	*Firmicutes*	*Bacilli*	*Bacillales*	*Bacillaceae*	*Bacillus*	*Bacillus pumilus*
970	*Firmicutes*	*Bacilli*	*Bacillales*	*Bacillaceae*	*Bacillus*	*Bacillus pumilus*
971	*Firmicutes*	*Bacilli*	*Bacillales*	*Bacillaceae*	*Bacillus*	*Bacillus pumilus*
972	*Firmicutes*	*Bacilli*	*Bacillales*	*Bacillaceae*	*Bacillus*	*Bacillus pumilus*
973	*Firmicutes*	*Bacilli*	*Bacillales*	*Bacillaceae*	*Bacillus*	*Bacillus pumilus*
974	*Firmicutes*	*Bacilli*	*Bacillales*	*Bacillaceae*	*Bacillus*	*Bacillus pumilus*
975	*Firmicutes*	*Bacilli*	*Bacillales*	*Bacillaceae*	*Bacillus*	*Bacillus pumilus*
976	*Firmicutes*	*Bacilli*	*Bacillales*	*Bacillaceae*	*Bacillus*	*Bacillus pumilus*
977	*Firmicutes*	*Bacilli*	*Bacillales*	*Bacillaceae*	*Bacillus*	*Bacillus pumilus*
978	*Firmicutes*	*Bacilli*	*Bacillales*	*Bacillaceae*	*Bacillus*	*Bacillus pumilus*
979	*Firmicutes*	*Bacilli*	*Bacillales*	*Bacillaceae*	*Bacillus*	*Bacillus pumilus*
980	*Firmicutes*	*Bacilli*	*Bacillales*	*Bacillaceae*	*Bacillus*	*Bacillus pumilus*

Table S4. Isolated strains by culturomics

bacteriaId	*phylum*	*class*	*order*	*family*	*genus*	*species*
981	*Firmicutes*	*Bacilli*	*Bacillales*	*Bacillaceae*	*Bacillus*	*Bacillus pumilus*
982	*Firmicutes*	*Bacilli*	*Bacillales*	*Bacillaceae*	*Bacillus*	*Bacillus pumilus*
983	*Firmicutes*	*Bacilli*	*Bacillales*	*Bacillaceae*	*Bacillus*	*Bacillus pumilus*
984	*Firmicutes*	*Bacilli*	*Bacillales*	*Bacillaceae*	*Bacillus*	*Bacillus pumilus*
985	*Firmicutes*	*Bacilli*	*Bacillales*	*Bacillaceae*	*Bacillus*	*Bacillus pumilus*
986	*Firmicutes*	*Bacilli*	*Bacillales*	*Bacillaceae*	*Bacillus*	*Bacillus pumilus*
987	*Firmicutes*	*Bacilli*	*Bacillales*	*Bacillaceae*	*Bacillus*	*Bacillus pumilus*
988	*Firmicutes*	*Bacilli*	*Bacillales*	*Bacillaceae*	*Bacillus*	*Bacillus pumilus*
989	*Firmicutes*	*Bacilli*	*Bacillales*	*Bacillaceae*	*Bacillus*	*Bacillus pumilus*
990	*Firmicutes*	*Bacilli*	*Bacillales*	*Bacillaceae*	*Bacillus*	*Bacillus pumilus*
991	*Firmicutes*	*Bacilli*	*Bacillales*	*Bacillaceae*	*Bacillus*	*Bacillus pumilus*
992	*Firmicutes*	*Bacilli*	*Bacillales*	*Bacillaceae*	*Bacillus*	*Bacillus pumilus*
993	*Firmicutes*	*Bacilli*	*Bacillales*	*Bacillaceae*	*Bacillus*	*Bacillus pumilus*
994	*Firmicutes*	*Bacilli*	*Bacillales*	*Bacillaceae*	*Bacillus*	*Bacillus pumilus*
995	*Firmicutes*	*Bacilli*	*Bacillales*	*Bacillaceae*	*Bacillus*	*Bacillus pumilus*
996	*Firmicutes*	*Bacilli*	*Bacillales*	*Bacillaceae*	*Bacillus*	*Bacillus pumilus*
997	*Firmicutes*	*Bacilli*	*Bacillales*	*Bacillaceae*	*Bacillus*	*Bacillus pumilus*
998	*Firmicutes*	*Bacilli*	*Bacillales*	*Bacillaceae*	*Bacillus*	*Bacillus pumilus*
999	*Firmicutes*	*Bacilli*	*Bacillales*	*Bacillaceae*	*Bacillus*	*Bacillus pumilus*
1000	*Firmicutes*	*Bacilli*	*Bacillales*	*Bacillaceae*	*Bacillus*	*Bacillus pumilus*
1001	*Firmicutes*	*Bacilli*	*Bacillales*	*Bacillaceae*	*Bacillus*	*Bacillus pumilus*
1002	*Firmicutes*	*Bacilli*	*Bacillales*	*Bacillaceae*	*Bacillus*	*Bacillus pumilus*
1003	*Firmicutes*	*Bacilli*	*Bacillales*	*Bacillaceae*	*Bacillus*	*Bacillus pumilus*
1004	*Firmicutes*	*Bacilli*	*Bacillales*	*Bacillaceae*	*Bacillus*	*Bacillus pumilus*
1005	*Firmicutes*	*Bacilli*	*Bacillales*	*Bacillaceae*	*Bacillus*	*Bacillus pumilus*
1006	*Firmicutes*	*Bacilli*	*Bacillales*	*Bacillaceae*	*Bacillus*	*Bacillus pumilus*
1007	*Firmicutes*	*Bacilli*	*Bacillales*	*Bacillaceae*	*Bacillus*	*Bacillus pumilus*
1008	*Firmicutes*	*Bacilli*	*Bacillales*	*Bacillaceae*	*Bacillus*	*Bacillus pumilus*

Table S4. Isolated strains by culturomics

bacterialId	*phylum*	*class*	*order*	*family*	*genus*	*species*
1009	*Firmicutes*	*Bacilli*	*Bacillales*	*Bacillaceae*	*Bacillus*	*Bacillus pumilus*
1010	*Firmicutes*	*Bacilli*	*Bacillales*	*Bacillaceae*	*Bacillus*	*Bacillus pumilus*
1011	*Firmicutes*	*Bacilli*	*Bacillales*	*Bacillaceae*	*Bacillus*	*Bacillus pumilus*
1012	*Firmicutes*	*Bacilli*	*Bacillales*	*Bacillaceae*	*Bacillus*	*Bacillus pumilus*
1013	*Firmicutes*	*Bacilli*	*Bacillales*	*Bacillaceae*	*Bacillus*	*Bacillus pumilus*
1014	*Firmicutes*	*Bacilli*	*Bacillales*	*Bacillaceae*	*Bacillus*	*Bacillus pumilus*
1015	*Firmicutes*	*Bacilli*	*Bacillales*	*Bacillaceae*	*Bacillus*	*Bacillus pumilus*
1016	*Firmicutes*	*Bacilli*	*Bacillales*	*Bacillaceae*	*Bacillus*	*Bacillus pumilus*
1017	*Firmicutes*	*Bacilli*	*Bacillales*	*Bacillaceae*	*Bacillus*	*Bacillus pumilus*
1018	*Firmicutes*	*Bacilli*	*Bacillales*	*Bacillaceae*	*Bacillus*	*Bacillus pumilus*
1019	*Firmicutes*	*Bacilli*	*Bacillales*	*Bacillaceae*	*Bacillus*	*Bacillus pumilus*
1020	*Firmicutes*	*Bacilli*	*Bacillales*	*Bacillaceae*	*Bacillus*	*Bacillus pumilus*
1021	*Firmicutes*	*Bacilli*	*Bacillales*	*Bacillaceae*	*Bacillus*	*Bacillus pumilus*
1022	*Firmicutes*	*Bacilli*	*Bacillales*	*Bacillaceae*	*Bacillus*	*Bacillus pumilus*
1023	*Firmicutes*	*Bacilli*	*Bacillales*	*Bacillaceae*	*Bacillus*	*Bacillus pumilus*
1024	*Firmicutes*	*Bacilli*	*Bacillales*	*Bacillaceae*	*Bacillus*	*Bacillus pumilus*
1025	*Firmicutes*	*Bacilli*	*Bacillales*	*Bacillaceae*	*Bacillus*	*Bacillus pumilus*
1026	*Firmicutes*	*Bacilli*	*Bacillales*	*Bacillaceae*	*Bacillus*	*Bacillus pumilus*
1027	*Firmicutes*	*Bacilli*	*Bacillales*	*Bacillaceae*	*Bacillus*	*Bacillus pumilus*
1028	*Firmicutes*	*Bacilli*	*Bacillales*	*Bacillaceae*	*Bacillus*	*Bacillus pumilus*
1029	*Firmicutes*	*Bacilli*	*Bacillales*	*Bacillaceae*	*Bacillus*	*Bacillus pumilus*
1030	*Firmicutes*	*Bacilli*	*Bacillales*	*Bacillaceae*	*Bacillus*	*Bacillus pumilus*
1031	*Firmicutes*	*Bacilli*	*Bacillales*	*Bacillaceae*	*Bacillus*	*Bacillus pumilus*
1032	*Firmicutes*	*Bacilli*	*Bacillales*	*Bacillaceae*	*Bacillus*	*Bacillus pumilus*
1033	*Firmicutes*	*Bacilli*	*Bacillales*	*Bacillaceae*	*Bacillus*	*Bacillus pumilus*
1034	*Firmicutes*	*Bacilli*	*Bacillales*	*Bacillaceae*	*Bacillus*	*Bacillus pumilus*
1035	*Firmicutes*	*Bacilli*	*Bacillales*	*Bacillaceae*	*Bacillus*	*Bacillus pumilus*
1036	*Firmicutes*	*Bacilli*	*Bacillales*	*Bacillaceae*	*Bacillus*	*Bacillus pumilus*

Table S4. Isolated strains by culturomics

bacteriaId	*phylum*	*class*	*order*	*family*	*genus*	*species*
1037	*Firmicutes*	*Bacilli*	*Bacillales*	*Bacillaceae*	*Bacillus*	*Bacillus pumilus*
1038	*Firmicutes*	*Bacilli*	*Bacillales*	*Bacillaceae*	*Bacillus*	*Bacillus pumilus*
1039	*Firmicutes*	*Bacilli*	*Bacillales*	*Bacillaceae*	*Bacillus*	*Bacillus pumilus*
1040	*Firmicutes*	*Bacilli*	*Bacillales*	*Bacillaceae*	*Bacillus*	*Bacillus pumilus*
1041	*Firmicutes*	*Bacilli*	*Bacillales*	*Bacillaceae*	*Bacillus*	*Bacillus pumilus*
1042	*Firmicutes*	*Bacilli*	*Bacillales*	*Bacillaceae*	*Bacillus*	*Bacillus pumilus*
1043	*Firmicutes*	*Bacilli*	*Bacillales*	*Bacillaceae*	*Bacillus*	*Bacillus pumilus*
1044	*Firmicutes*	*Bacilli*	*Bacillales*	*Bacillaceae*	*Bacillus*	*Bacillus pumilus*
1045	*Firmicutes*	*Bacilli*	*Bacillales*	*Bacillaceae*	*Bacillus*	*Bacillus pumilus*
1046	*Firmicutes*	*Bacilli*	*Bacillales*	*Bacillaceae*	*Bacillus*	*Bacillus pumilus*
1047	*Firmicutes*	*Bacilli*	*Bacillales*	*Bacillaceae*	*Bacillus*	*Bacillus pumilus*
1048	*Firmicutes*	*Bacilli*	*Bacillales*	*Bacillaceae*	*Bacillus*	*Bacillus pumilus*
1049	*Firmicutes*	*Bacilli*	*Bacillales*	*Bacillaceae*	*Bacillus*	*Bacillus pumilus*
1050	*Firmicutes*	*Bacilli*	*Bacillales*	*Bacillaceae*	*Bacillus*	*Bacillus pumilus*
1051	*Firmicutes*	*Bacilli*	*Bacillales*	*Bacillaceae*	*Bacillus*	*Bacillus safensis*
1052	*Firmicutes*	*Bacilli*	*Bacillales*	*Bacillaceae*	*Bacillus*	*Bacillus safensis*
1053	*Firmicutes*	*Bacilli*	*Bacillales*	*Bacillaceae*	*Bacillus*	*Bacillus safensis*
1054	*Firmicutes*	*Bacilli*	*Bacillales*	*Bacillaceae*	*Bacillus*	*Bacillus safensis*
1055	*Firmicutes*	*Bacilli*	*Bacillales*	*Bacillaceae*	*Bacillus*	*Bacillus safensis*
1056	*Firmicutes*	*Bacilli*	*Bacillales*	*Bacillaceae*	*Bacillus*	*Bacillus safensis*
1057	*Firmicutes*	*Bacilli*	*Bacillales*	*Bacillaceae*	*Bacillus*	*Bacillus safensis*
1058	*Firmicutes*	*Bacilli*	*Bacillales*	*Bacillaceae*	*Bacillus*	*Bacillus safensis*
1059	*Firmicutes*	*Bacilli*	*Bacillales*	*Bacillaceae*	*Bacillus*	*Bacillus safensis*
1060	*Firmicutes*	*Bacilli*	*Bacillales*	*Bacillaceae*	*Bacillus*	*Bacillus safensis*
1061	*Firmicutes*	*Bacilli*	*Bacillales*	*Bacillaceae*	*Bacillus*	*Bacillus safensis*
1062	*Firmicutes*	*Bacilli*	*Bacillales*	*Bacillaceae*	*Bacillus*	*Bacillus safensis*
1063	*Firmicutes*	*Bacilli*	*Bacillales*	*Bacillaceae*	*Bacillus*	*Bacillus safensis*
1064	*Firmicutes*	*Bacilli*	*Bacillales*	*Bacillaceae*	*Bacillus*	*Bacillus safensis*

Table S4. Isolated strains by culturomics

bacteriaId	phylum	class	order	family	genus	species
1065	*Firmicutes*	*Bacilli*	*Bacillales*	*Bacillaceae*	*Bacillus*	*Bacillus safensis*
1066	*Firmicutes*	*Bacilli*	*Bacillales*	*Bacillaceae*	*Bacillus*	*Bacillus safensis*
1067	*Firmicutes*	*Bacilli*	*Bacillales*	*Bacillaceae*	*Bacillus*	*Bacillus safensis*
1068	*Firmicutes*	*Bacilli*	*Bacillales*	*Bacillaceae*	*Bacillus*	*Bacillus safensis*
1069	*Firmicutes*	*Bacilli*	*Bacillales*	*Bacillaceae*	*Bacillus*	*Bacillus safensis*
1070	*Firmicutes*	*Bacilli*	*Bacillales*	*Bacillaceae*	*Bacillus*	*Bacillus safensis*
1071	*Firmicutes*	*Bacilli*	*Bacillales*	*Bacillaceae*	*Bacillus*	*Bacillus safensis*
1072	*Firmicutes*	*Bacilli*	*Bacillales*	*Bacillaceae*	*Bacillus*	*Bacillus safensis*
1073	*Firmicutes*	*Bacilli*	*Bacillales*	*Bacillaceae*	*Bacillus*	*Bacillus safensis*
1074	*Firmicutes*	*Bacilli*	*Bacillales*	*Bacillaceae*	*Bacillus*	*Bacillus safensis*
1075	*Firmicutes*	*Bacilli*	*Bacillales*	*Bacillaceae*	*Bacillus*	*Bacillus safensis*
1076	*Firmicutes*	*Bacilli*	*Bacillales*	*Bacillaceae*	*Bacillus*	*Bacillus safensis*
1077	*Firmicutes*	*Bacilli*	*Bacillales*	*Bacillaceae*	*Bacillus*	*Bacillus safensis*
1078	*Firmicutes*	*Bacilli*	*Bacillales*	*Bacillaceae*	*Bacillus*	*Bacillus safensis*
1079	*Firmicutes*	*Bacilli*	*Bacillales*	*Bacillaceae*	*Bacillus*	*Bacillus safensis*
1080	*Firmicutes*	*Bacilli*	*Bacillales*	*Bacillaceae*	*Bacillus*	*Bacillus safensis*
1081	*Firmicutes*	*Bacilli*	*Bacillales*	*Bacillaceae*	*Bacillus*	*Bacillus safensis*
1082	*Firmicutes*	*Bacilli*	*Bacillales*	*Bacillaceae*	*Bacillus*	*Bacillus safensis*
1083	*Firmicutes*	*Bacilli*	*Bacillales*	*Bacillaceae*	*Bacillus*	*Bacillus safensis*
1084	*Firmicutes*	*Bacilli*	*Bacillales*	*Bacillaceae*	*Bacillus*	*Bacillus safensis*
1085	*Firmicutes*	*Bacilli*	*Bacillales*	*Bacillaceae*	*Bacillus*	*Bacillus safensis*
1086	*Firmicutes*	*Bacilli*	*Bacillales*	*Bacillaceae*	*Bacillus*	*Bacillus safensis*
1087	*Firmicutes*	*Bacilli*	*Bacillales*	*Bacillaceae*	*Bacillus*	*Bacillus safensis*
1088	*Firmicutes*	*Bacilli*	*Bacillales*	*Bacillaceae*	*Bacillus*	*Bacillus safensis*
1089	*Firmicutes*	*Bacilli*	*Bacillales*	*Bacillaceae*	*Bacillus*	*Bacillus safensis*
1090	*Firmicutes*	*Bacilli*	*Bacillales*	*Bacillaceae*	*Bacillus*	*Bacillus safensis*
1091	*Firmicutes*	*Bacilli*	*Bacillales*	*Bacillaceae*	*Bacillus*	*Bacillus safensis*
1092	*Firmicutes*	*Bacilli*	*Bacillales*	*Bacillaceae*	*Bacillus*	*Bacillus safensis*

Table S4. Isolated strains by culturomics

bacteriaId	*phylum*	*class*	*order*	*family*	*genus*	*species*
1093	*Firmicutes*	*Bacilli*	*Bacillales*	*Bacillaceae*	*Bacillus*	*Bacillus safensis*
1094	*Firmicutes*	*Bacilli*	*Bacillales*	*Bacillaceae*	*Bacillus*	*Bacillus safensis*
1095	*Firmicutes*	*Bacilli*	*Bacillales*	*Bacillaceae*	*Bacillus*	*Bacillus safensis*
1096	*Firmicutes*	*Bacilli*	*Bacillales*	*Bacillaceae*	*Bacillus*	*Bacillus safensis*
1097	*Firmicutes*	*Bacilli*	*Bacillales*	*Bacillaceae*	*Bacillus*	*Bacillus safensis*
1098	*Firmicutes*	*Bacilli*	*Bacillales*	*Bacillaceae*	*Bacillus*	*Bacillus safensis*
1099	*Firmicutes*	*Bacilli*	*Bacillales*	*Bacillaceae*	*Bacillus*	*Bacillus safensis*
1100	*Firmicutes*	*Bacilli*	*Bacillales*	*Bacillaceae*	*Bacillus*	*Bacillus safensis*
1101	*Firmicutes*	*Bacilli*	*Bacillales*	*Bacillaceae*	*Bacillus*	*Bacillus safensis*
1102	*Firmicutes*	*Bacilli*	*Bacillales*	*Bacillaceae*	*Bacillus*	*Bacillus safensis*
1103	*Firmicutes*	*Bacilli*	*Bacillales*	*Bacillaceae*	*Bacillus*	*Bacillus safensis*
1104	*Firmicutes*	*Bacilli*	*Bacillales*	*Bacillaceae*	*Bacillus*	*Bacillus safensis*
1105	*Firmicutes*	*Bacilli*	*Bacillales*	*Bacillaceae*	*Bacillus*	*Bacillus safensis*
1106	*Firmicutes*	*Bacilli*	*Bacillales*	*Bacillaceae*	*Bacillus*	*Bacillus safensis*
1107	*Firmicutes*	*Bacilli*	*Bacillales*	*Bacillaceae*	*Bacillus*	*Bacillus safensis*
1108	*Firmicutes*	*Bacilli*	*Bacillales*	*Bacillaceae*	*Bacillus*	*Bacillus safensis*
1109	*Firmicutes*	*Bacilli*	*Bacillales*	*Bacillaceae*	*Bacillus*	*Bacillus safensis*
1110	*Firmicutes*	*Bacilli*	*Bacillales*	*Bacillaceae*	*Bacillus*	*Bacillus safensis*
1111	*Firmicutes*	*Bacilli*	*Bacillales*	*Bacillaceae*	*Bacillus*	*Bacillus safensis*
1112	*Firmicutes*	*Bacilli*	*Bacillales*	*Bacillaceae*	*Bacillus*	*Bacillus safensis*
1113	*Firmicutes*	*Bacilli*	*Bacillales*	*Bacillaceae*	*Bacillus*	*Bacillus safensis*
1114	*Firmicutes*	*Bacilli*	*Bacillales*	*Bacillaceae*	*Bacillus*	*Bacillus safensis*
1115	*Firmicutes*	*Bacilli*	*Bacillales*	*Bacillaceae*	*Bacillus*	*Bacillus safensis*
1116	*Firmicutes*	*Bacilli*	*Bacillales*	*Bacillaceae*	*Bacillus*	*Bacillus safensis*
1117	*Firmicutes*	*Bacilli*	*Bacillales*	*Bacillaceae*	*Bacillus*	*Bacillus safensis*
1118	*Firmicutes*	*Bacilli*	*Bacillales*	*Bacillaceae*	*Bacillus*	*Bacillus safensis*
1119	*Firmicutes*	*Bacilli*	*Bacillales*	*Bacillaceae*	*Bacillus*	*Bacillus safensis*
1120	*Firmicutes*	*Bacilli*	*Bacillales*	*Bacillaceae*	*Bacillus*	*Bacillus safensis*

Table S4. Isolated strains by culturomics

bacteriaId	*phylum*	*class*	*order*	*family*	*genus*	*species*
1121	*Firmicutes*	*Bacilli*	*Bacillales*	*Bacillaceae*	*Bacillus*	*Bacillus safensis*
1122	*Firmicutes*	*Bacilli*	*Bacillales*	*Bacillaceae*	*Bacillus*	*Bacillus safensis*
1123	*Firmicutes*	*Bacilli*	*Bacillales*	*Bacillaceae*	*Bacillus*	*Bacillus safensis*
1124	*Firmicutes*	*Bacilli*	*Bacillales*	*Bacillaceae*	*Bacillus*	*Bacillus safensis*
1125	*Firmicutes*	*Bacilli*	*Bacillales*	*Bacillaceae*	*Bacillus*	*Bacillus safensis*
1126	*Firmicutes*	*Bacilli*	*Bacillales*	*Bacillaceae*	*Bacillus*	*Bacillus safensis*
1127	*Firmicutes*	*Bacilli*	*Bacillales*	*Bacillaceae*	*Bacillus*	*Bacillus safensis*
1128	*Firmicutes*	*Bacilli*	*Bacillales*	*Bacillaceae*	*Bacillus*	*Bacillus safensis*
1129	*Firmicutes*	*Bacilli*	*Bacillales*	*Bacillaceae*	*Bacillus*	*Bacillus safensis*
1130	*Firmicutes*	*Bacilli*	*Bacillales*	*Bacillaceae*	*Bacillus*	*Bacillus safensis*
1131	*Firmicutes*	*Bacilli*	*Bacillales*	*Bacillaceae*	*Bacillus*	*Bacillus safensis*
1132	*Firmicutes*	*Bacilli*	*Bacillales*	*Bacillaceae*	*Bacillus*	*Bacillus safensis*
1133	*Firmicutes*	*Bacilli*	*Bacillales*	*Bacillaceae*	*Bacillus*	*Bacillus safensis*
1134	*Firmicutes*	*Bacilli*	*Bacillales*	*Bacillaceae*	*Bacillus*	*Bacillus safensis*
1135	*Firmicutes*	*Bacilli*	*Bacillales*	*Bacillaceae*	*Bacillus*	*Bacillus safensis*
1136	*Firmicutes*	*Bacilli*	*Bacillales*	*Bacillaceae*	*Bacillus*	*Bacillus safensis*
1137	*Firmicutes*	*Bacilli*	*Bacillales*	*Bacillaceae*	*Bacillus*	*Bacillus safensis*
1138	*Firmicutes*	*Bacilli*	*Bacillales*	*Bacillaceae*	*Bacillus*	*Bacillus safensis*
1139	*Firmicutes*	*Bacilli*	*Bacillales*	*Bacillaceae*	*Bacillus*	*Bacillus safensis*
1140	*Firmicutes*	*Bacilli*	*Bacillales*	*Bacillaceae*	*Bacillus*	*Bacillus safensis*
1141	*Firmicutes*	*Bacilli*	*Bacillales*	*Bacillaceae*	*Bacillus*	*Bacillus safensis*
1142	*Firmicutes*	*Bacilli*	*Bacillales*	*Bacillaceae*	*Bacillus*	*Bacillus safensis*
1143	*Firmicutes*	*Bacilli*	*Bacillales*	*Bacillaceae*	*Bacillus*	*Bacillus safensis*
1144	*Firmicutes*	*Bacilli*	*Bacillales*	*Bacillaceae*	*Bacillus*	*Bacillus safensis*
1145	*Firmicutes*	*Bacilli*	*Bacillales*	*Bacillaceae*	*Bacillus*	*Bacillus safensis*
1146	*Firmicutes*	*Bacilli*	*Bacillales*	*Bacillaceae*	*Bacillus*	*Bacillus safensis*
1147	*Firmicutes*	*Bacilli*	*Bacillales*	*Bacillaceae*	*Bacillus*	*Bacillus safensis*
1148	*Firmicutes*	*Bacilli*	*Bacillales*	*Bacillaceae*	*Bacillus*	*Bacillus safensis*

Table S4. Isolated strains by culturomics

bacteriaId	*phylum*	*class*	*order*	*family*	*genus*	*species*
1149	*Firmicutes*	*Bacilli*	*Bacillales*	*Bacillaceae*	*Bacillus*	*Bacillus safensis*
1150	*Firmicutes*	*Bacilli*	*Bacillales*	*Bacillaceae*	*Bacillus*	*Bacillus safensis*
1151	*Firmicutes*	*Bacilli*	*Bacillales*	*Bacillaceae*	*Bacillus*	*Bacillus safensis*
1152	*Firmicutes*	*Bacilli*	*Bacillales*	*Bacillaceae*	*Bacillus*	*Bacillus safensis*
1153	*Firmicutes*	*Bacilli*	*Bacillales*	*Bacillaceae*	*Bacillus*	*Bacillus safensis*
1154	*Firmicutes*	*Bacilli*	*Bacillales*	*Bacillaceae*	*Bacillus*	*Bacillus safensis*
1155	*Firmicutes*	*Bacilli*	*Bacillales*	*Bacillaceae*	*Bacillus*	*Bacillus safensis*
1156	*Firmicutes*	*Bacilli*	*Bacillales*	*Bacillaceae*	*Bacillus*	*Bacillus safensis*
1157	*Firmicutes*	*Bacilli*	*Bacillales*	*Bacillaceae*	*Bacillus*	*Bacillus safensis*
1158	*Firmicutes*	*Bacilli*	*Bacillales*	*Bacillaceae*	*Bacillus*	*Bacillus safensis*
1159	*Firmicutes*	*Bacilli*	*Bacillales*	*Bacillaceae*	*Bacillus*	*Bacillus safensis*
1160	*Firmicutes*	*Bacilli*	*Bacillales*	*Bacillaceae*	*Bacillus*	*Bacillus safensis*
1161	*Firmicutes*	*Bacilli*	*Bacillales*	*Bacillaceae*	*Bacillus*	*Bacillus safensis*
1162	*Firmicutes*	*Bacilli*	*Bacillales*	*Bacillaceae*	*Bacillus*	*Bacillus safensis*
1163	*Firmicutes*	*Bacilli*	*Bacillales*	*Bacillaceae*	*Bacillus*	*Bacillus safensis*
1164	*Firmicutes*	*Bacilli*	*Bacillales*	*Bacillaceae*	*Bacillus*	*Bacillus safensis*
1165	*Firmicutes*	*Bacilli*	*Bacillales*	*Bacillaceae*	*Bacillus*	*Bacillus safensis*
1166	*Firmicutes*	*Bacilli*	*Bacillales*	*Bacillaceae*	*Bacillus*	*Bacillus safensis*
1167	*Firmicutes*	*Bacilli*	*Bacillales*	*Bacillaceae*	*Bacillus*	*Bacillus safensis*
1168	*Firmicutes*	*Bacilli*	*Bacillales*	*Bacillaceae*	*Bacillus*	*Bacillus safensis*
1169	*Firmicutes*	*Bacilli*	*Bacillales*	*Bacillaceae*	*Bacillus*	*Bacillus safensis*
1170	*Firmicutes*	*Bacilli*	*Bacillales*	*Bacillaceae*	*Bacillus*	*Bacillus safensis*
1171	*Firmicutes*	*Bacilli*	*Bacillales*	*Bacillaceae*	*Bacillus*	*Bacillus safensis*
1172	*Firmicutes*	*Bacilli*	*Bacillales*	*Bacillaceae*	*Bacillus*	*Bacillus safensis*
1173	*Firmicutes*	*Bacilli*	*Bacillales*	*Bacillaceae*	*Bacillus*	*Bacillus safensis*
1174	*Firmicutes*	*Bacilli*	*Bacillales*	*Bacillaceae*	*Bacillus*	*Bacillus safensis*
1175	*Firmicutes*	*Bacilli*	*Bacillales*	*Bacillaceae*	*Bacillus*	*Bacillus safensis*
1176	*Firmicutes*	*Bacilli*	*Bacillales*	*Bacillaceae*	*Bacillus*	*Bacillus safensis*

Table S4. Isolated strains by culturomics

bacteriaId	*phylum*	*class*	*order*	*family*	*genus*	*species*
1177	*Firmicutes*	*Bacilli*	*Bacillales*	*Bacillaceae*	*Bacillus*	*Bacillus safensis*
1178	*Firmicutes*	*Bacilli*	*Bacillales*	*Bacillaceae*	*Bacillus*	*Bacillus safensis*
1179	*Firmicutes*	*Bacilli*	*Bacillales*	*Bacillaceae*	*Bacillus*	*Bacillus safensis*
1180	*Firmicutes*	*Bacilli*	*Bacillales*	*Bacillaceae*	*Bacillus*	*Bacillus safensis*
1181	*Firmicutes*	*Bacilli*	*Bacillales*	*Bacillaceae*	*Bacillus*	*Bacillus safensis*
1182	*Firmicutes*	*Bacilli*	*Bacillales*	*Bacillaceae*	*Bacillus*	*Bacillus safensis*
1183	*Firmicutes*	*Bacilli*	*Bacillales*	*Bacillaceae*	*Bacillus*	*Bacillus safensis*
1184	*Firmicutes*	*Bacilli*	*Bacillales*	*Bacillaceae*	*Bacillus*	*Bacillus safensis*
1185	*Firmicutes*	*Bacilli*	*Bacillales*	*Bacillaceae*	*Bacillus*	*Bacillus safensis*
1186	*Firmicutes*	*Bacilli*	*Bacillales*	*Bacillaceae*	*Bacillus*	*Bacillus safensis*
1187	*Firmicutes*	*Bacilli*	*Bacillales*	*Bacillaceae*	*Bacillus*	*Bacillus safensis*
1188	*Firmicutes*	*Bacilli*	*Bacillales*	*Bacillaceae*	*Bacillus*	*Bacillus safensis*
1189	*Firmicutes*	*Bacilli*	*Bacillales*	*Bacillaceae*	*Bacillus*	*Bacillus safensis*
1190	*Firmicutes*	*Bacilli*	*Bacillales*	*Bacillaceae*	*Bacillus*	*Bacillus safensis*
1191	*Firmicutes*	*Bacilli*	*Bacillales*	*Bacillaceae*	*Bacillus*	*Bacillus safensis*
1192	*Firmicutes*	*Bacilli*	*Bacillales*	*Bacillaceae*	*Bacillus*	*Bacillus safensis*
1193	*Firmicutes*	*Bacilli*	*Bacillales*	*Bacillaceae*	*Bacillus*	*Bacillus safensis*
1194	*Firmicutes*	*Bacilli*	*Bacillales*	*Bacillaceae*	*Bacillus*	*Bacillus safensis*
1195	*Firmicutes*	*Bacilli*	*Bacillales*	*Bacillaceae*	*Bacillus*	*Bacillus safensis*
1196	*Firmicutes*	*Bacilli*	*Bacillales*	*Bacillaceae*	*Bacillus*	*Bacillus safensis*
1197	*Firmicutes*	*Bacilli*	*Bacillales*	*Bacillaceae*	*Bacillus*	*Bacillus safensis*
1198	*Firmicutes*	*Bacilli*	*Bacillales*	*Bacillaceae*	*Bacillus*	*Bacillus safensis*
1199	*Firmicutes*	*Bacilli*	*Bacillales*	*Bacillaceae*	*Bacillus*	*Bacillus safensis*
1200	*Firmicutes*	*Bacilli*	*Bacillales*	*Bacillaceae*	*Bacillus*	*Bacillus safensis*
1201	*Firmicutes*	*Bacilli*	*Bacillales*	*Bacillaceae*	*Bacillus*	*Bacillus safensis*
1202	*Firmicutes*	*Bacilli*	*Bacillales*	*Bacillaceae*	*Bacillus*	*Bacillus safensis*
1203	*Firmicutes*	*Bacilli*	*Bacillales*	*Bacillaceae*	*Bacillus*	*Bacillus safensis*
1204	*Firmicutes*	*Bacilli*	*Bacillales*	*Bacillaceae*	*Bacillus*	*Bacillus safensis*

Table S4. Isolated strains by culturomics

bacteriaId	*phylum*	*class*	*order*	*family*	*genus*	*species*
1205	*Firmicutes*	*Bacilli*	*Bacillales*	*Bacillaceae*	*Bacillus*	*Bacillus safensis*
1206	*Firmicutes*	*Bacilli*	*Bacillales*	*Bacillaceae*	*Bacillus*	*Bacillus safensis*
1207	*Firmicutes*	*Bacilli*	*Bacillales*	*Bacillaceae*	*Bacillus*	*Bacillus safensis*
1208	*Firmicutes*	*Bacilli*	*Bacillales*	*Bacillaceae*	*Bacillus*	*Bacillus safensis*
1209	*Firmicutes*	*Bacilli*	*Bacillales*	*Bacillaceae*	*Bacillus*	*Bacillus safensis*
1210	*Firmicutes*	*Bacilli*	*Bacillales*	*Bacillaceae*	*Bacillus*	*Bacillus safensis*
1211	*Firmicutes*	*Bacilli*	*Bacillales*	*Bacillaceae*	*Bacillus*	*Bacillus safensis*
1212	*Firmicutes*	*Bacilli*	*Bacillales*	*Bacillaceae*	*Bacillus*	*Bacillus safensis*
1213	*Firmicutes*	*Bacilli*	*Bacillales*	*Bacillaceae*	*Bacillus*	*Bacillus sonorensis*
1214	*Firmicutes*	*Bacilli*	*Bacillales*	*Bacillaceae*	*Bacillus*	*Bacillus sonorensis*
1215	*Firmicutes*	*Bacilli*	*Bacillales*	*Bacillaceae*	*Bacillus*	*Bacillus sp*
1216	*Firmicutes*	*Bacilli*	*Bacillales*	*Bacillaceae*	*Bacillus*	*Bacillus sp*
1217	*Firmicutes*	*Bacilli*	*Bacillales*	*Bacillaceae*	*Bacillus*	*Bacillus sp*
1218	*Firmicutes*	*Bacilli*	*Bacillales*	*Bacillaceae*	*Bacillus*	*Bacillus sp.*
1219	*Firmicutes*	*Bacilli*	*Bacillales*	*Bacillaceae*	*Bacillus*	*Bacillus sp.*
1220	*Firmicutes*	*Bacilli*	*Bacillales*	*Bacillaceae*	*Bacillus*	*Bacillus sp.*
1221	*Firmicutes*	*Bacilli*	*Bacillales*	*Bacillaceae*	*Bacillus*	*Bacillus sp.*
1222	*Firmicutes*	*Bacilli*	*Bacillales*	*Bacillaceae*	*Bacillus*	*Bacillus sp.*
1223	*Firmicutes*	*Bacilli*	*Bacillales*	*Bacillaceae*	*Bacillus*	*Bacillus subtilis*
1224	*Firmicutes*	*Bacilli*	*Bacillales*	*Bacillaceae*	*Bacillus*	*Bacillus subtilis*
1225	*Firmicutes*	*Bacilli*	*Bacillales*	*Bacillaceae*	*Bacillus*	*Bacillus subtilis*
1226	*Firmicutes*	*Bacilli*	*Bacillales*	*Bacillaceae*	*Bacillus*	*Bacillus subtilis*
1227	*Firmicutes*	*Bacilli*	*Bacillales*	*Bacillaceae*	*Bacillus*	*Bacillus subtilis*
1228	*Firmicutes*	*Bacilli*	*Bacillales*	*Bacillaceae*	*Bacillus*	*Bacillus subtilis*
1229	*Firmicutes*	*Bacilli*	*Bacillales*	*Bacillaceae*	*Bacillus*	*Bacillus subtilis*
1230	*Firmicutes*	*Bacilli*	*Bacillales*	*Bacillaceae*	*Bacillus*	*Bacillus subtilis*
1231	*Firmicutes*	*Bacilli*	*Bacillales*	*Bacillaceae*	*Bacillus*	*Bacillus subtilis*
1232	*Firmicutes*	*Bacilli*	*Bacillales*	*Bacillaceae*	*Bacillus*	*Bacillus subtilis*

Table S4. Isolated strains by culturomics

bacteriaId	*phylum*	*class*	*order*	*family*	*genus*	*species*
1233	*Firmicutes*	*Bacilli*	*Bacillales*	*Bacillaceae*	*Bacillus*	*Bacillus subtilis*
1234	*Firmicutes*	*Bacilli*	*Bacillales*	*Bacillaceae*	*Bacillus*	*Bacillus velezensis*
1235	*Firmicutes*	*Bacilli*	*Bacillales*	*Bacillaceae*	*Bacillus*	*Bacillus*
1236	*Firmicutes*	*Bacilli*	*Bacillales*	*Bacillaceae*	*Bacillus*	*Bacillus*
1237	*Firmicutes*	*Bacilli*	*Bacillales*	*Bacillaceae*	*Bacillus*	*Bacillus*
1238	*Firmicutes*	*Bacilli*	*Bacillales*	*Bacillaceae*	*Bacillus*	*Bacillus*
1239	*Firmicutes*	*Bacilli*	*Bacillales*	*Bacillaceae*	*Bacillus*	*Bacillus*
1240	*Firmicutes*	*Bacilli*	*Bacillales*	*Bacillaceae*	*Bacillus*	*Brevibacillus*
1241	*Firmicutes*	*Bacilli*	*Bacillales*	*Bacillaceae*	*Lysinibacillus*	*Lysinibacillus*
1242	*Firmicutes*	*Bacilli*	*Bacillales*	*Bacillaceae*	*Lysinibacillus*	*Lysinibacillus*
1243	*Firmicutes*	*Bacilli*	*Bacillales*	*Bacillaceae*	*Lysinibacillus*	*Lysinibacillus*
1244	*Firmicutes*	*Bacilli*	*Bacillales*	*Bacillaceae*	*Lysinibacillus*	*Lysinibacillus*
1245	*Firmicutes*	*Bacilli*	*Bacillales*	*Bacillaceae*	*Lysinibacillus*	*Lysinibacillus*
1246	*Firmicutes*	*Bacilli*	*Bacillales*	*Bacillaceae*	*Lysinibacillus*	*Lysinibacillus*
1247	*Firmicutes*	*Bacilli*	*Bacillales*	*Bacillaceae*	*Lysinibacillus*	*Lysinibacillus*
1248	*Firmicutes*	*Bacilli*	*Bacillales*	*Bacillaceae*	*Lysinibacillus*	*Lysinibacillus*
1249	*Firmicutes*	*Bacilli*	*Bacillales*	*Bacillaceae*	*Lysinibacillus*	*Lysinibacillus*
1250	*Firmicutes*	*Bacilli*	*Bacillales*	*Bacillaceae*	*Lysinibacillus*	*Lysinibacillus*
1251	*Firmicutes*	*Bacilli*	*Bacillales*	*Bacillaceae*	*Lysinibacillus*	*Lysinibacillus*
1252	*Firmicutes*	*Bacilli*	*Bacillales*	*Bacillaceae*	*Lysinibacillus*	*Lysinibacillus*
1253	*Firmicutes*	*Bacilli*	*Bacillales*	*Bacillaceae*	*Lysinibacillus*	*Lysinibacillus sp.*
1254	*Firmicutes*	*Bacilli*	*Bacillales*	*Bacillaceae*	*Lysinibacillus*	*Lysinibacillus sp.*
1255	*Firmicutes*	*Bacilli*	*Bacillales*	*Bacillaceae*	*Lysinibacillus*	*Lysinibacillus sp.*
1256	*Firmicutes*	*Bacilli*	*Bacillales*	*Bacillaceae*	*Lysinibacillus*	*Lysinibacillus sp.*
1257	*Firmicutes*	*Bacilli*	*Bacillales*	*Bacillaceae*	*Lysinibacillus*	*Lysinibacillus sp.*
1258	*Firmicutes*	*Bacilli*	*Bacillales*	*Bacillaceae*	*Lysinibacillus*	*Lysinibacillus sp.*
1259	*Firmicutes*	*Bacilli*	*Bacillales*	*Bacillaceae*	*Lysinibacillus*	*Lysinibacillus sp.*
1260	*Firmicutes*	*Bacilli*	*Bacillales*	*Bacillaceae*	*Lysinibacillus*	*Lysinibacillus sp.*

Table S4. Isolated strains by culturomics

bacteriaId	*phylum*	*class*	*order*	*family*	*genus*	*species*
1261	*Firmicutes*	*Bacilli*	*Bacillales*	*Bacillaceae*	*Lysinibacillus*	*Lysinibacillus sp.*
1262	*Firmicutes*	*Bacilli*	*Bacillales*	*Bacillaceae*	*Lysinibacillus*	*Lysinibacillus sp.*
1263	*Firmicutes*	*Bacilli*	*Bacillales*	*Bacillaceae*	*Lysinibacillus*	*Lysinibacillus sp.*
1264	*Firmicutes*	*Bacilli*	*Bacillales*	*Bacillaceae*	*Lysinibacillus*	*Lysinibacillus*
1265	*Firmicutes*	*Bacilli*	*Bacillales*	*Bacillaceae*	*Lysinibacillus*	*Lysinibacillus*
1266	*Firmicutes*	*Bacilli*	*Bacillales*	*Bacillaceae*	*Lysinibacillus*	*Lysinibacillus*
1267	*Firmicutes*	*Bacilli*	*Bacillales*	*Bacillaceae*	*Lysinibacillus*	*Lysinibacillus*
1268	*Firmicutes*	*Bacilli*	*Bacillales*	*Bacillaceae*	*Lysinibacillus*	*Lysinibacillus*
1269	*Firmicutes*	*Bacilli*	*Bacillales*	*Bacillaceae*	*Lysinibacillus*	*Lysinibacillus*
1270	*Firmicutes*	*Bacilli*	*Bacillales*	*Bacillaceae*	*Lysinibacillus*	*Lysinibacillus*
1271	*Firmicutes*	*Bacilli*	*Bacillales*	*Bacillaceae*	*Lysinibacillus*	*Lysinibacillus*
1272	*Firmicutes*	*Bacilli*	*Bacillales*	*Bacillaceae*	*Lysinibacillus*	*Lysinibacillus*
1273	*Firmicutes*	*Bacilli*	*Bacillales*	*Bacillaceae*	*Lysinibacillus*	*Lysinibacillus*
1274	*Firmicutes*	*Bacilli*	*Bacillales*	*Bacillaceae*	*Lysinibacillus*	*Lysinibacillus*
1275	*Firmicutes*	*Bacilli*	*Bacillales*	*Bacillaceae*	*Lysinibacillus*	*Lysinibacillus*
1276	*Firmicutes*	*Bacilli*	*Bacillales*	*Bacillaceae*	*Lysinibacillus*	*Lysinibacillus*
1277	*Firmicutes*	*Bacilli*	*Bacillales*	*Bacillaceae*	*Lysinibacillus*	*Lysinibacillus*
1278	*Firmicutes*	*Bacilli*	*Bacillales*	*Bacillaceae*	*Lysinibacillus*	*Lysinibacillus*
1279	*Firmicutes*	*Bacilli*	*Bacillales*	*Bacillaceae*	*Lysinibacillus*	*Lysinibacillus*
1280	*Firmicutes*	*Bacilli*	*Bacillales*	*Bacillaceae*	*Lysinibacillus*	*Lysinibacillus*
1281	*Firmicutes*	*Bacilli*	*Bacillales*	*Bacillaceae*	*Lysinibacillus*	*Lysinibacillus*
1282	*Firmicutes*	*Bacilli*	*Bacillales*	*Bacillaceae*	*Lysinibacillus*	*Lysinibacillus*
1283	*Firmicutes*	*Bacilli*	*Bacillales*	*Bacillaceae*	*Lysinibacillus*	*Lysinibacillus*
1284	*Firmicutes*	*Bacilli*	*Bacillales*	*Bacillaceae*	*Lysinibacillus*	*Lysinibacillus*
1285	*Firmicutes*	*Bacilli*	*Bacillales*	*Bacillaceae*	*Lysinibacillus*	*Lysinibacillus*
1286	*Firmicutes*	*Bacilli*	*Bacillales*	*Bacillaceae*	*Lysinibacillus*	*Lysinibacillus*
1287	*Firmicutes*	*Bacilli*	*Bacillales*	*Bacillaceae*	*Lysinibacillus*	*Lysinibacillus*
1288	*Firmicutes*	*Bacilli*	*Bacillales*	*Bacillaceae*	*Lysinibacillus*	*Lysinibacillus*

Table S4. Isolated strains by culturomics

bacteriaId	*phylum*	*class*	*order*	*family*	*genus*	*species*
1289	*Firmicutes*	*Bacilli*	*Bacillales*	*Bacillaceae*	*Lysinibacillus*	*Lysinibacillus*
1290	*Firmicutes*	*Bacilli*	*Bacillales*	*Bacillaceae*	*Lysinibacillus*	*Lysinibacillus*
1291	*Firmicutes*	*Bacilli*	*Bacillales*	*Bacillaceae*	*Lysinibacillus*	*Lysinibacillus*
1292	*Firmicutes*	*Bacilli*	*Bacillales*	*Bacillaceae*	*Lysinibacillus*	*Lysinibacillus*
1293	*Firmicutes*	*Bacilli*	*Bacillales*	*Bacillaceae*	*Lysinibacillus*	*Lysinibacillus*
1294	*Firmicutes*	*Bacilli*	*Bacillales*	*Bacillaceae*	*Lysinibacillus*	*Lysinibacillus*
1295	*Firmicutes*	*Bacilli*	*Bacillales*	*Bacillaceae*	*Lysinibacillus*	*Lysinibacillus*
1296	*Firmicutes*	*Bacilli*	*Bacillales*	*Bacillaceae*	*Lysinibacillus*	*Lysinibacillus*
1297	*Firmicutes*	*Bacilli*	*Bacillales*	*Bacillaceae*	*Lysinibacillus*	*Lysinibacillus*
1298	*Firmicutes*	*Bacilli*	*Bacillales*	*Bacillaceae*	*Lysinibacillus*	*Lysinibacillus*
1299	*Firmicutes*	*Bacilli*	*Bacillales*	*Bacillaceae*	*Lysinibacillus*	*Lysinibacillus*
1300	*Firmicutes*	*Bacilli*	*Bacillales*	*Bacillaceae*	*Lysinibacillus*	*Lysinibacillus*
1301	*Firmicutes*	*Bacilli*	*Bacillales*	*Bacillaceae*	*Lysinibacillus*	*Lysinibacillus*
1302	*Firmicutes*	*Bacilli*	*Bacillales*	*Bacillaceae*	*Lysinibacillus*	*Lysinibacillus*
1303	*Firmicutes*	*Bacilli*	*Bacillales*	*Bacillaceae*	*Lysinibacillus*	*Lysinibacillus*
1304	*Firmicutes*	*Bacilli*	*Bacillales*	*Bacillaceae*	*Lysinibacillus*	*Lysinibacillus*
1305	*Firmicutes*	*Bacilli*	*Bacillales*	*Bacillaceae*	*Lysinibacillus*	*Lysinibacillus*
1306	*Firmicutes*	*Bacilli*	*Bacillales*	*Bacillaceae*	*Lysinibacillus*	*Lysinibacillus*
1307	*Firmicutes*	*Bacilli*	*Bacillales*	*Bacillaceae*	*Lysinibacillus*	*Lysinibacillus*
1308	*Firmicutes*	*Bacilli*	*Bacillales*	*Bacillaceae*	*Lysinibacillus*	*Lysinibacillus*
1309	*Firmicutes*	*Bacilli*	*Bacillales*	*Bacillaceae*	*Lysinibacillus*	*Lysinibacillus*
1310	*Firmicutes*	*Bacilli*	*Bacillales*	*Bacillaceae*	*Lysinibacillus*	*Lysinibacillus*
1311	*Firmicutes*	*Bacilli*	*Bacillales*	*Bacillaceae*	*Lysinibacillus*	*Lysinibacillus*
1312	*Firmicutes*	*Bacilli*	*Bacillales*	*Bacillaceae*	*Lysinibacillus*	*Lysinibacillus*
1313	*Firmicutes*	*Bacilli*	*Bacillales*	*Bacillaceae*	*Lysinibacillus*	*Lysinibacillus*
1314	*Firmicutes*	*Bacilli*	*Bacillales*	*Bacillaceae*	*Lysinibacillus*	*Lysinibacillus*
1315	*Firmicutes*	*Bacilli*	*Bacillales*	*Bacillaceae*	*Lysinibacillus*	*Lysinibacillus*
1316	*Firmicutes*	*Bacilli*	*Bacillales*	*Bacillaceae*	*Lysinibacillus*	*Lysinibacillus*

Table S4. Isolated strains by culturomics

bacteriald	*phylum*	*class*	*order*	*family*	*genus*	*species*
1317	*Firmicutes*	*Bacilli*	*Bacillales*	*Bacillaceae*	*Lysinibacillus*	*Lysinibacillus*
1318	*Firmicutes*	*Bacilli*	*Bacillales*	*Bacillaceae*	*Lysinibacillus*	*Lysinibacillus*
1319	*Firmicutes*	*Bacilli*	*Bacillales*	*Bacillaceae*	*Lysinibacillus*	*Lysinibacillus*
1320	*Firmicutes*	*Bacilli*	*Bacillales*	*Bacillaceae*	*Lysinibacillus*	*Lysinibacillus*
1321	*Firmicutes*	*Bacilli*	*Bacillales*	*Bacillaceae*	*Lysinibacillus*	*Lysinibacillus*
1322	*Firmicutes*	*Bacilli*	*Bacillales*	*Bacillaceae*	*Lysinibacillus*	*Lysinibacillus*
1323	*Firmicutes*	*Bacilli*	*Bacillales*	*Bacillaceae*	*Lysinibacillus*	*Lysinibacillus*
1324	*Firmicutes*	*Bacilli*	*Bacillales*	*Bacillaceae*	*Lysinibacillus*	*Lysinibacillus*
1325	*Firmicutes*	*Bacilli*	*Bacillales*	*Bacillaceae*	*Lysinibacillus*	*Lysinibacillus*
1326	*Firmicutes*	*Bacilli*	*Bacillales*	*Bacillaceae*	*Lysinibacillus*	*Lysinibacillus*
1327	*Firmicutes*	*Bacilli*	*Bacillales*	*Bacillaceae*	*Lysinibacillus*	*Lysinibacillus*
1328	*Firmicutes*	*Bacilli*	*Bacillales*	*Bacillaceae*	*Oceanobacillus*	*Oceanobacillus*
1329	*Firmicutes*	*Bacilli*	*Bacillales*	*Bacillales Family*	*Exiguobacterium*	*Exiguobacterium*
1330	*Firmicutes*	*Bacilli*	*Bacillales*	*Paenibacillaceae*	*Aneurinibacillus*	*Aneurinibacillus*
1331	*Firmicutes*	*Bacilli*	*Bacillales*	*Paenibacillaceae*	*Brevibacillus*	*Brevibacillus*
1332	*Firmicutes*	*Bacilli*	*Bacillales*	*Paenibacillaceae*	*Brevibacillus*	*Brevibacillus*
1333	*Firmicutes*	*Bacilli*	*Bacillales*	*Paenibacillaceae*	*Brevibacillus*	*Brevibacillus*
1334	*Firmicutes*	*Bacilli*	*Bacillales*	*Paenibacillaceae*	*Brevibacillus*	*Brevibacillus*
1335	*Firmicutes*	*Bacilli*	*Bacillales*	*Paenibacillaceae*	*Brevibacillus*	*Brevibacillus*
1336	*Firmicutes*	*Bacilli*	*Bacillales*	*Paenibacillaceae*	*Brevibacillus*	*Brevibacillus*
1337	*Firmicutes*	*Bacilli*	*Bacillales*	*Paenibacillaceae*	*Brevibacillus*	*Brevibacillus*
1338	*Firmicutes*	*Bacilli*	*Bacillales*	*Paenibacillaceae*	*Brevibacillus*	*Brevibacillus*
1339	*Firmicutes*	*Bacilli*	*Bacillales*	*Paenibacillaceae*	*Brevibacillus*	*Brevibacillus*
1340	*Firmicutes*	*Bacilli*	*Bacillales*	*Paenibacillaceae*	*Brevibacillus*	*Brevibacillus*
1341	*Firmicutes*	*Bacilli*	*Bacillales*	*Paenibacillaceae*	*Brevibacillus*	*Brevibacillus*
1342	*Firmicutes*	*Bacilli*	*Bacillales*	*Paenibacillaceae*	*Brevibacillus*	*Brevibacillus*
1343	*Firmicutes*	*Bacilli*	*Bacillales*	*Paenibacillaceae*	*Brevibacillus*	*Brevibacillus*
1344	*Firmicutes*	*Bacilli*	*Bacillales*	*Paenibacillaceae*	*Brevibacillus*	*Brevibacillus*

Table S4. Isolated strains by culturomics

bacteriaId	*phylum*	*class*	*order*	*family*	*genus*	*species*
1345	*Firmicutes*	*Bacilli*	*Bacillales*	*Paenibacillaceae*	*Brevibacillus*	*Brevibacillus*
1346	*Firmicutes*	*Bacilli*	*Bacillales*	*Paenibacillaceae*	*Brevibacillus*	*Brevibacillus*
1347	*Firmicutes*	*Bacilli*	*Bacillales*	*Paenibacillaceae*	*Brevibacillus*	*Brevibacillus*
1348	*Firmicutes*	*Bacilli*	*Bacillales*	*Paenibacillaceae*	*Brevibacillus*	*Brevibacillus*
1349	*Firmicutes*	*Bacilli*	*Bacillales*	*Paenibacillaceae*	*Brevibacillus*	*Brevibacillus*
1350	*Firmicutes*	*Bacilli*	*Bacillales*	*Paenibacillaceae*	*Brevibacillus*	*Brevibacillus*
1351	*Firmicutes*	*Bacilli*	*Bacillales*	*Paenibacillaceae*	*Brevibacillus*	*Brevibacillus*
1352	*Firmicutes*	*Bacilli*	*Bacillales*	*Paenibacillaceae*	*Brevibacillus*	*Brevibacillus*
1353	*Firmicutes*	*Bacilli*	*Bacillales*	*Paenibacillaceae*	*Brevibacillus*	*Brevibacillus*
1354	*Firmicutes*	*Bacilli*	*Bacillales*	*Paenibacillaceae*	*Brevibacillus*	*Brevibacillus*
1355	*Firmicutes*	*Bacilli*	*Bacillales*	*Paenibacillaceae*	*Brevibacillus*	*Brevibacillus*
1356	*Firmicutes*	*Bacilli*	*Bacillales*	*Paenibacillaceae*	*Brevibacillus*	*Brevibacillus*
1357	*Firmicutes*	*Bacilli*	*Bacillales*	*Paenibacillaceae*	*Brevibacillus*	*Brevibacillus*
1358	*Firmicutes*	*Bacilli*	*Bacillales*	*Paenibacillaceae*	*Brevibacillus*	*Brevibacillus*
1359	*Firmicutes*	*Bacilli*	*Bacillales*	*Paenibacillaceae*	*Brevibacillus*	*Brevibacillus*
1360	*Firmicutes*	*Bacilli*	*Bacillales*	*Paenibacillaceae*	*Brevibacillus*	*Brevibacillus*
1361	*Firmicutes*	*Bacilli*	*Bacillales*	*Paenibacillaceae*	*Brevibacillus*	*Brevibacillus*
1362	*Firmicutes*	*Bacilli*	*Bacillales*	*Paenibacillaceae*	*Brevibacillus*	*Brevibacillus*
1363	*Firmicutes*	*Bacilli*	*Bacillales*	*Paenibacillaceae*	*Brevibacillus*	*Brevibacillus*
1364	*Firmicutes*	*Bacilli*	*Bacillales*	*Paenibacillaceae*	*Brevibacillus*	*Brevibacillus*
1365	*Firmicutes*	*Bacilli*	*Bacillales*	*Paenibacillaceae*	*Brevibacillus*	*Brevibacillus*
1366	*Firmicutes*	*Bacilli*	*Bacillales*	*Paenibacillaceae*	*Brevibacillus*	*Brevibacillus*
1367	*Firmicutes*	*Bacilli*	*Bacillales*	*Paenibacillaceae*	*Brevibacillus*	*Brevibacillus*
1368	*Firmicutes*	*Bacilli*	*Bacillales*	*Paenibacillaceae*	*Brevibacillus*	*Brevibacillus*
1369	*Firmicutes*	*Bacilli*	*Bacillales*	*Paenibacillaceae*	*Brevibacillus*	*Brevibacillus*
1370	*Firmicutes*	*Bacilli*	*Bacillales*	*Paenibacillaceae*	*Brevibacillus*	*Brevibacillus*
1371	*Firmicutes*	*Bacilli*	*Bacillales*	*Paenibacillaceae*	*Brevibacillus*	*Brevibacillus*
1372	*Firmicutes*	*Bacilli*	*Bacillales*	*Paenibacillaceae*	*Brevibacillus*	*Brevibacillus*

Table S4. Isolated strains by culturomics

bacteriaId	*phylum*	*class*	*order*	*family*	*genus*	*species*
1373	*Firmicutes*	*Bacilli*	*Bacillales*	*Paenibacillaceae*	*Brevibacillus*	*Brevibacillus*
1374	*Firmicutes*	*Bacilli*	*Bacillales*	*Paenibacillaceae*	*Brevibacillus*	*Brevibacillus*
1375	*Firmicutes*	*Bacilli*	*Bacillales*	*Paenibacillaceae*	*Brevibacillus*	*Brevibacillus*
1376	*Firmicutes*	*Bacilli*	*Bacillales*	*Paenibacillaceae*	*Brevibacillus*	*Brevibacillus*
1377	*Firmicutes*	*Bacilli*	*Bacillales*	*Paenibacillaceae*	*Brevibacillus*	*Brevibacillus*
1378	*Firmicutes*	*Bacilli*	*Bacillales*	*Paenibacillaceae*	*Brevibacillus*	*Brevibacillus*
1379	*Firmicutes*	*Bacilli*	*Bacillales*	*Paenibacillaceae*	*Brevibacillus*	*Brevibacillus*
1380	*Firmicutes*	*Bacilli*	*Bacillales*	*Paenibacillaceae*	*Brevibacillus*	*Brevibacillus*
1381	*Firmicutes*	*Bacilli*	*Bacillales*	*Paenibacillaceae*	*Brevibacillus*	*Brevibacillus*
1382	*Firmicutes*	*Bacilli*	*Bacillales*	*Paenibacillaceae*	*Brevibacillus*	*Brevibacillus*
1383	*Firmicutes*	*Bacilli*	*Bacillales*	*Paenibacillaceae*	*Brevibacillus*	*Brevibacillus*
1384	*Firmicutes*	*Bacilli*	*Bacillales*	*Paenibacillaceae*	*Brevibacillus*	*Brevibacillus*
1385	*Firmicutes*	*Bacilli*	*Bacillales*	*Paenibacillaceae*	*Paenibacillus*	*Paenibacillus*
1386	*Firmicutes*	*Bacilli*	*Bacillales*	*Planococcaceae*	*Solibacillus*	*Solibacillus*
1387	*Firmicutes*	*Bacilli*	*Bacillales*	*Planococcaceae*	*Solibacillus*	*Solibacillus*
1388	*Firmicutes*	*Bacilli*	*Bacillales*	*Planococcaceae*	*Solibacillus*	*Solibacillus*
1389	*Firmicutes*	*Bacilli*	*Bacillales*	*Planococcaceae*	*Solibacillus*	*Solibacillus*
1390	*Firmicutes*	*Bacilli*	*Bacillales*	*Planococcaceae*	*Solibacillus*	*Solibacillus*
1391	*Firmicutes*	*Bacilli*	*Bacillales*	*Planococcaceae*	*Solibacillus*	*Solibacillus*
1392	*Firmicutes*	*Bacilli*	*Bacillales*	*Planococcaceae*	*Solibacillus*	*Solibacillus*
1393	*Firmicutes*	*Bacilli*	*Bacillales*	*Planococcaceae*	*Solibacillus*	*Solibacillus*
1394	*Firmicutes*	*Bacilli*	*Bacillales*	*Planococcaceae*	*Solibacillus*	*Solibacillus*
1395	*Firmicutes*	*Bacilli*	*Bacillales*	*Planococcaceae*	*Solibacillus*	*Solibacillus*
1396	*Firmicutes*	*Bacilli*	*Bacillales*	*Planococcaceae*	*Solibacillus*	*Solibacillus*
1397	*Firmicutes*	*Bacilli*	*Bacillales*	*Planococcaceae*	*Solibacillus*	*Solibacillus*
1398	*Firmicutes*	*Bacilli*	*Bacillales*	*Planococcaceae*	*Solibacillus*	*Solibacillus*
1399	*Firmicutes*	*Bacilli*	*Bacillales*	*Planococcaceae*	*Solibacillus*	*Solibacillus*
1400	*Firmicutes*	*Bacilli*	*Bacillales*	*Planococcaceae*	*Solibacillus*	*Solibacillus*

Table S4. Isolated strains by culturomics

bacteriald	*phylum*	*class*	*order*	*family*	*genus*	*species*
1401	*Firmicutes*	*Bacilli*	*Bacillales*	*Staphylococcaceae*	*Staphylococcus*	*Staphylococcus*
1402	*Firmicutes*	*Bacilli*	*Bacillales*	*Staphylococcaceae*	*Staphylococcus*	*Staphylococcus*
1403	*Firmicutes*	*Bacilli*	*Bacillales*	*Staphylococcaceae*	*Staphylococcus*	*Staphylococcus*
1404	*Firmicutes*	*Bacilli*	*Bacillales*	*Staphylococcaceae*	*Staphylococcus*	*Staphylococcus*
1405	*Firmicutes*	*Bacilli*	*Bacillales*	*Staphylococcaceae*	*Staphylococcus*	*Staphylococcus*
1406	*Firmicutes*	*Bacilli*	*Bacillales*	*Staphylococcaceae*	*Staphylococcus*	*Staphylococcus*
1407	*Firmicutes*	*Bacilli*	*Bacillales*	*Staphylococcaceae*	*Staphylococcus*	*Staphylococcus*
1408	*Firmicutes*	*Bacilli*	*Bacillales*	*Staphylococcaceae*	*Staphylococcus*	*Staphylococcus*
1409	*Firmicutes*	*Bacilli*	*Bacillales*	*Staphylococcaceae*	*Staphylococcus*	*Staphylococcus*
1410	*Firmicutes*	*Bacilli*	*Bacillales*	*Staphylococcaceae*	*Staphylococcus*	*Staphylococcus*
1411	*Firmicutes*	*Bacilli*	*Bacillales*	*Staphylococcaceae*	*Staphylococcus*	*Staphylococcus*
1412	*Firmicutes*	*Bacilli*	*Lactobacillales*	*Enterococcaceae*	*Enterococcus*	*Enterococcus asini*
1413	*Firmicutes*	*Bacilli*	*Lactobacillales*	*Enterococcaceae*	*Enterococcus*	*Enterococcus asini*
1414	*Firmicutes*	*Bacilli*	*Lactobacillales*	*Enterococcaceae*	*Enterococcus*	*Enterococcus asini*
1415	*Firmicutes*	*Bacilli*	*Lactobacillales*	*Enterococcaceae*	*Enterococcus*	*Enterococcus asini*
1416	*Firmicutes*	*Bacilli*	*Lactobacillales*	*Enterococcaceae*	*Enterococcus*	*Enterococcus asini*
1417	*Firmicutes*	*Bacilli*	*Lactobacillales*	*Enterococcaceae*	*Enterococcus*	*Enterococcus asini*
1418	*Firmicutes*	*Bacilli*	*Lactobacillales*	*Enterococcaceae*	*Enterococcus*	*Enterococcus asini*
1419	*Firmicutes*	*Bacilli*	*Lactobacillales*	*Enterococcaceae*	*Enterococcus*	*Enterococcus asini*
1420	*Firmicutes*	*Bacilli*	*Lactobacillales*	*Enterococcaceae*	*Enterococcus*	*Enterococcus asini*
1421	*Firmicutes*	*Bacilli*	*Lactobacillales*	*Enterococcaceae*	*Enterococcus*	*Enterococcus asini*
1422	*Firmicutes*	*Bacilli*	*Lactobacillales*	*Enterococcaceae*	*Enterococcus*	*Enterococcus asini*
1423	*Firmicutes*	*Bacilli*	*Lactobacillales*	*Enterococcaceae*	*Enterococcus*	*Enterococcus asini*
1424	*Firmicutes*	*Bacilli*	*Lactobacillales*	*Enterococcaceae*	*Enterococcus*	*Enterococcus asini*
1425	*Firmicutes*	*Bacilli*	*Lactobacillales*	*Enterococcaceae*	*Enterococcus*	*Enterococcus asini*
1426	*Firmicutes*	*Bacilli*	*Lactobacillales*	*Enterococcaceae*	*Enterococcus*	*Enterococcus asini*
1427	*Firmicutes*	*Bacilli*	*Lactobacillales*	*Enterococcaceae*	*Enterococcus*	*Enterococcus asini*
1428	*Firmicutes*	*Bacilli*	*Lactobacillales*	*Enterococcaceae*	*Enterococcus*	*Enterococcus asini*

Table S4. Isolated strains by culturomics

bacteriaId	*phylum*	*class*	*order*	*family*	*genus*	*species*
1429	*Firmicutes*	*Bacilli*	*Lactobacillales*	*Enterococcaceae*	*Enterococcus*	*Enterococcus asini*
1430	*Firmicutes*	*Bacilli*	*Lactobacillales*	*Enterococcaceae*	*Enterococcus*	*Enterococcus asini*
1431	*Firmicutes*	*Bacilli*	*Lactobacillales*	*Enterococcaceae*	*Enterococcus*	*Enterococcus asini*
1432	*Firmicutes*	*Bacilli*	*Lactobacillales*	*Enterococcaceae*	*Enterococcus*	*Enterococcus asini*
1433	*Firmicutes*	*Bacilli*	*Lactobacillales*	*Enterococcaceae*	*Enterococcus*	*Enterococcus asini*
1434	*Firmicutes*	*Bacilli*	*Lactobacillales*	*Enterococcaceae*	*Enterococcus*	*Enterococcus asini*
1435	*Firmicutes*	*Bacilli*	*Lactobacillales*	*Enterococcaceae*	*Enterococcus*	*Enterococcus asini*
1436	*Firmicutes*	*Bacilli*	*Lactobacillales*	*Enterococcaceae*	*Enterococcus*	*Enterococcus asini*
1437	*Firmicutes*	*Bacilli*	*Lactobacillales*	*Enterococcaceae*	*Enterococcus*	*Enterococcus asini*
1438	*Firmicutes*	*Bacilli*	*Lactobacillales*	*Enterococcaceae*	*Enterococcus*	*Enterococcus asini*
1439	*Firmicutes*	*Bacilli*	*Lactobacillales*	*Enterococcaceae*	*Enterococcus*	*Enterococcus asini*
1440	*Firmicutes*	*Bacilli*	*Lactobacillales*	*Enterococcaceae*	*Enterococcus*	*Enterococcus asini*
1441	*Firmicutes*	*Bacilli*	*Lactobacillales*	*Enterococcaceae*	*Enterococcus*	*Enterococcus asini*
1442	*Firmicutes*	*Bacilli*	*Lactobacillales*	*Enterococcaceae*	*Enterococcus*	*Enterococcus asini*
1443	*Firmicutes*	*Bacilli*	*Lactobacillales*	*Enterococcaceae*	*Enterococcus*	*Enterococcus asini*
1444	*Firmicutes*	*Bacilli*	*Lactobacillales*	*Enterococcaceae*	*Enterococcus*	*Enterococcus asini*
1445	*Firmicutes*	*Bacilli*	*Lactobacillales*	*Enterococcaceae*	*Enterococcus*	*Enterococcus asini*
1446	*Firmicutes*	*Bacilli*	*Lactobacillales*	*Enterococcaceae*	*Enterococcus*	*Enterococcus asini*
1447	*Firmicutes*	*Bacilli*	*Lactobacillales*	*Enterococcaceae*	*Enterococcus*	*Enterococcus asini*
1448	*Firmicutes*	*Bacilli*	*Lactobacillales*	*Enterococcaceae*	*Enterococcus*	*Enterococcus asini*
1449	*Firmicutes*	*Bacilli*	*Lactobacillales*	*Enterococcaceae*	*Enterococcus*	*Enterococcus asini*
1450	*Firmicutes*	*Bacilli*	*Lactobacillales*	*Enterococcaceae*	*Enterococcus*	*Enterococcus asini*
1451	*Firmicutes*	*Bacilli*	*Lactobacillales*	*Enterococcaceae*	*Enterococcus*	*Enterococcus asini*
1452	*Firmicutes*	*Bacilli*	*Lactobacillales*	*Enterococcaceae*	*Enterococcus*	*Enterococcus asini*
1453	*Firmicutes*	*Bacilli*	*Lactobacillales*	*Enterococcaceae*	*Enterococcus*	*Enterococcus asini*
1454	*Firmicutes*	*Bacilli*	*Lactobacillales*	*Enterococcaceae*	*Enterococcus*	*Enterococcus avium*
1455	*Firmicutes*	*Bacilli*	*Lactobacillales*	*Enterococcaceae*	*Enterococcus*	*Enterococcus avium*
1456	*Firmicutes*	*Bacilli*	*Lactobacillales*	*Enterococcaceae*	*Enterococcus*	*Enterococcus avium*

Table S4. Isolated strains by culturomics

bacteriaId	*phylum*	*class*	*order*	*family*	*genus*	*species*
1457	*Firmicutes*	*Bacilli*	*Lactobacillales*	*Enterococcaceae*	*Enterococcus*	*Enterococcus avium*
1458	*Firmicutes*	*Bacilli*	*Lactobacillales*	*Enterococcaceae*	*Enterococcus*	*Enterococcus avium*
1459	*Firmicutes*	*Bacilli*	*Lactobacillales*	*Enterococcaceae*	*Enterococcus*	*Enterococcus avium*
1460	*Firmicutes*	*Bacilli*	*Lactobacillales*	*Enterococcaceae*	*Enterococcus*	*Enterococcus avium*
1461	*Firmicutes*	*Bacilli*	*Lactobacillales*	*Enterococcaceae*	*Enterococcus*	*Enterococcus avium*
1462	*Firmicutes*	*Bacilli*	*Lactobacillales*	*Enterococcaceae*	*Enterococcus*	*Enterococcus avium*
1463	*Firmicutes*	*Bacilli*	*Lactobacillales*	*Enterococcaceae*	*Enterococcus*	*Enterococcus avium*
1464	*Firmicutes*	*Bacilli*	*Lactobacillales*	*Enterococcaceae*	*Enterococcus*	*Enterococcus avium*
1465	*Firmicutes*	*Bacilli*	*Lactobacillales*	*Enterococcaceae*	*Enterococcus*	*Enterococcus avium*
1466	*Firmicutes*	*Bacilli*	*Lactobacillales*	*Enterococcaceae*	*Enterococcus*	*Enterococcus avium*
1467	*Firmicutes*	*Bacilli*	*Lactobacillales*	*Enterococcaceae*	*Enterococcus*	*Enterococcus avium*
1468	*Firmicutes*	*Bacilli*	*Lactobacillales*	*Enterococcaceae*	*Enterococcus*	*Enterococcus avium*
1469	*Firmicutes*	*Bacilli*	*Lactobacillales*	*Enterococcaceae*	*Enterococcus*	*Enterococcus avium*
1470	*Firmicutes*	*Bacilli*	*Lactobacillales*	*Enterococcaceae*	*Enterococcus*	*Enterococcus avium*
1471	*Firmicutes*	*Bacilli*	*Lactobacillales*	*Enterococcaceae*	*Enterococcus*	*Enterococcus avium*
1472	*Firmicutes*	*Bacilli*	*Lactobacillales*	*Enterococcaceae*	*Enterococcus*	*Enterococcus avium*
1473	*Firmicutes*	*Bacilli*	*Lactobacillales*	*Enterococcaceae*	*Enterococcus*	*Enterococcus avium*
1474	*Firmicutes*	*Bacilli*	*Lactobacillales*	*Enterococcaceae*	*Enterococcus*	*Enterococcus avium*
1475	*Firmicutes*	*Bacilli*	*Lactobacillales*	*Enterococcaceae*	*Enterococcus*	*Enterococcus avium*
1476	*Firmicutes*	*Bacilli*	*Lactobacillales*	*Enterococcaceae*	*Enterococcus*	*Enterococcus avium*
1477	*Firmicutes*	*Bacilli*	*Lactobacillales*	*Enterococcaceae*	*Enterococcus*	*Enterococcus avium*
1478	*Firmicutes*	*Bacilli*	*Lactobacillales*	*Enterococcaceae*	*Enterococcus*	*Enterococcus avium*
1479	*Firmicutes*	*Bacilli*	*Lactobacillales*	*Enterococcaceae*	*Enterococcus*	*Enterococcus avium*
1480	*Firmicutes*	*Bacilli*	*Lactobacillales*	*Enterococcaceae*	*Enterococcus*	*Enterococcus avium*
1481	*Firmicutes*	*Bacilli*	*Lactobacillales*	*Enterococcaceae*	*Enterococcus*	*Enterococcus avium*
1482	*Firmicutes*	*Bacilli*	*Lactobacillales*	*Enterococcaceae*	*Enterococcus*	*Enterococcus avium*
1483	*Firmicutes*	*Bacilli*	*Lactobacillales*	*Enterococcaceae*	*Enterococcus*	*Enterococcus avium*
1484	*Firmicutes*	*Bacilli*	*Lactobacillales*	*Enterococcaceae*	*Enterococcus*	*Enterococcus avium*

Table S4. Isolated strains by culturomics

bacteriaId	*phylum*	*class*	*order*	*family*	*genus*	*species*
1485	*Firmicutes*	*Bacilli*	*Lactobacillales*	*Enterococcaceae*	*Enterococcus*	*Enterococcus*
1486	*Firmicutes*	*Bacilli*	*Lactobacillales*	*Enterococcaceae*	*Enterococcus*	*Enterococcus*
1487	*Firmicutes*	*Bacilli*	*Lactobacillales*	*Enterococcaceae*	*Enterococcus*	*Enterococcus*
1488	*Firmicutes*	*Bacilli*	*Lactobacillales*	*Enterococcaceae*	*Enterococcus*	*Enterococcus*
1489	*Firmicutes*	*Bacilli*	*Lactobacillales*	*Enterococcaceae*	*Enterococcus*	*Enterococcus*
1490	*Firmicutes*	*Bacilli*	*Lactobacillales*	*Enterococcaceae*	*Enterococcus*	*Enterococcus*
1491	*Firmicutes*	*Bacilli*	*Lactobacillales*	*Enterococcaceae*	*Enterococcus*	*Enterococcus*
1492	*Firmicutes*	*Bacilli*	*Lactobacillales*	*Enterococcaceae*	*Enterococcus*	*Enterococcus*
1493	*Firmicutes*	*Bacilli*	*Lactobacillales*	*Enterococcaceae*	*Enterococcus*	*Enterococcus*
1494	*Firmicutes*	*Bacilli*	*Lactobacillales*	*Enterococcaceae*	*Enterococcus*	*Enterococcus*
1495	*Firmicutes*	*Bacilli*	*Lactobacillales*	*Enterococcaceae*	*Enterococcus*	*Enterococcus*
1496	*Firmicutes*	*Bacilli*	*Lactobacillales*	*Enterococcaceae*	*Enterococcus*	*Enterococcus*
1497	*Firmicutes*	*Bacilli*	*Lactobacillales*	*Enterococcaceae*	*Enterococcus*	*Enterococcus*
1498	*Firmicutes*	*Bacilli*	*Lactobacillales*	*Enterococcaceae*	*Enterococcus*	*Enterococcus*
1499	*Firmicutes*	*Bacilli*	*Lactobacillales*	*Enterococcaceae*	*Enterococcus*	*Enterococcus*
1500	*Firmicutes*	*Bacilli*	*Lactobacillales*	*Enterococcaceae*	*Enterococcus*	*Enterococcus*
1501	*Firmicutes*	*Bacilli*	*Lactobacillales*	*Enterococcaceae*	*Enterococcus*	*Enterococcus*
1502	*Firmicutes*	*Bacilli*	*Lactobacillales*	*Enterococcaceae*	*Enterococcus*	*Enterococcus*
1503	*Firmicutes*	*Bacilli*	*Lactobacillales*	*Enterococcaceae*	*Enterococcus*	*Enterococcus*
1504	*Firmicutes*	*Bacilli*	*Lactobacillales*	*Enterococcaceae*	*Enterococcus*	*Enterococcus*
1505	*Firmicutes*	*Bacilli*	*Lactobacillales*	*Enterococcaceae*	*Enterococcus*	*Enterococcus*
1506	*Firmicutes*	*Bacilli*	*Lactobacillales*	*Enterococcaceae*	*Enterococcus*	*Enterococcus*
1507	*Firmicutes*	*Bacilli*	*Lactobacillales*	*Enterococcaceae*	*Enterococcus*	*Enterococcus*
1508	*Firmicutes*	*Bacilli*	*Lactobacillales*	*Enterococcaceae*	*Enterococcus*	*Enterococcus*
1509	*Firmicutes*	*Bacilli*	*Lactobacillales*	*Enterococcaceae*	*Enterococcus*	*Enterococcus*
1510	*Firmicutes*	*Bacilli*	*Lactobacillales*	*Enterococcaceae*	*Enterococcus*	*Enterococcus*
1511	*Firmicutes*	*Bacilli*	*Lactobacillales*	*Enterococcaceae*	*Enterococcus*	*Enterococcus*
1512	*Firmicutes*	*Bacilli*	*Lactobacillales*	*Enterococcaceae*	*Enterococcus*	*Enterococcus*

Table S4. Isolated strains by culturomics

bacteriaId	*phylum*	*class*	*order*	*family*	*genus*	*species*
1513	*Firmicutes*	*Bacilli*	*Lactobacillales*	*Enterococcaceae*	*Enterococcus*	*Enterococcus*
1514	*Firmicutes*	*Bacilli*	*Lactobacillales*	*Enterococcaceae*	*Enterococcus*	*Enterococcus*
1515	*Firmicutes*	*Bacilli*	*Lactobacillales*	*Enterococcaceae*	*Enterococcus*	*Enterococcus*
1516	*Firmicutes*	*Bacilli*	*Lactobacillales*	*Enterococcaceae*	*Enterococcus*	*Enterococcus*
1517	*Firmicutes*	*Bacilli*	*Lactobacillales*	*Enterococcaceae*	*Enterococcus*	*Enterococcus*
1518	*Firmicutes*	*Bacilli*	*Lactobacillales*	*Enterococcaceae*	*Enterococcus*	*Enterococcus*
1519	*Firmicutes*	*Bacilli*	*Lactobacillales*	*Enterococcaceae*	*Enterococcus*	*Enterococcus*
1520	*Firmicutes*	*Bacilli*	*Lactobacillales*	*Enterococcaceae*	*Enterococcus*	*Enterococcus*
1521	*Firmicutes*	*Bacilli*	*Lactobacillales*	*Enterococcaceae*	*Enterococcus*	*Enterococcus*
1522	*Firmicutes*	*Bacilli*	*Lactobacillales*	*Enterococcaceae*	*Enterococcus*	*Enterococcus*
1523	*Firmicutes*	*Bacilli*	*Lactobacillales*	*Enterococcaceae*	*Enterococcus*	*Enterococcus*
1524	*Firmicutes*	*Bacilli*	*Lactobacillales*	*Enterococcaceae*	*Enterococcus*	*Enterococcus*
1525	*Firmicutes*	*Bacilli*	*Lactobacillales*	*Enterococcaceae*	*Enterococcus*	*Enterococcus*
1526	*Firmicutes*	*Bacilli*	*Lactobacillales*	*Enterococcaceae*	*Enterococcus*	*Enterococcus*
1527	*Firmicutes*	*Bacilli*	*Lactobacillales*	*Enterococcaceae*	*Enterococcus*	*Enterococcus*
1528	*Firmicutes*	*Bacilli*	*Lactobacillales*	*Enterococcaceae*	*Enterococcus*	*Enterococcus*
1529	*Firmicutes*	*Bacilli*	*Lactobacillales*	*Enterococcaceae*	*Enterococcus*	*Enterococcus*
1530	*Firmicutes*	*Bacilli*	*Lactobacillales*	*Enterococcaceae*	*Enterococcus*	*Enterococcus*
1531	*Firmicutes*	*Bacilli*	*Lactobacillales*	*Enterococcaceae*	*Enterococcus*	*Enterococcus*
1532	*Firmicutes*	*Bacilli*	*Lactobacillales*	*Enterococcaceae*	*Enterococcus*	*Enterococcus*
1533	*Firmicutes*	*Bacilli*	*Lactobacillales*	*Enterococcaceae*	*Enterococcus*	*Enterococcus*
1534	*Firmicutes*	*Bacilli*	*Lactobacillales*	*Enterococcaceae*	*Enterococcus*	*Enterococcus*
1535	*Firmicutes*	*Bacilli*	*Lactobacillales*	*Enterococcaceae*	*Enterococcus*	*Enterococcus*
1536	*Firmicutes*	*Bacilli*	*Lactobacillales*	*Enterococcaceae*	*Enterococcus*	*Enterococcus*
1537	*Firmicutes*	*Bacilli*	*Lactobacillales*	*Enterococcaceae*	*Enterococcus*	*Enterococcus*
1538	*Firmicutes*	*Bacilli*	*Lactobacillales*	*Enterococcaceae*	*Enterococcus*	*Enterococcus*
1539	*Firmicutes*	*Bacilli*	*Lactobacillales*	*Enterococcaceae*	*Enterococcus*	*Enterococcus*
1540	*Firmicutes*	*Bacilli*	*Lactobacillales*	*Enterococcaceae*	*Enterococcus*	*Enterococcus*

Table S4. Isolated strains by culturomics

bacteriaId	*phylum*	*class*	*order*	*family*	*genus*	*species*
1541	*Firmicutes*	*Bacilli*	*Lactobacillales*	*Enterococcaceae*	*Enterococcus*	*Enterococcus*
1542	*Firmicutes*	*Bacilli*	*Lactobacillales*	*Enterococcaceae*	*Enterococcus*	*Enterococcus*
1543	*Firmicutes*	*Bacilli*	*Lactobacillales*	*Enterococcaceae*	*Enterococcus*	*Enterococcus*
1544	*Firmicutes*	*Bacilli*	*Lactobacillales*	*Enterococcaceae*	*Enterococcus*	*Enterococcus*
1545	*Firmicutes*	*Bacilli*	*Lactobacillales*	*Enterococcaceae*	*Enterococcus*	*Enterococcus*
1546	*Firmicutes*	*Bacilli*	*Lactobacillales*	*Enterococcaceae*	*Enterococcus*	*Enterococcus*
1547	*Firmicutes*	*Bacilli*	*Lactobacillales*	*Enterococcaceae*	*Enterococcus*	*Enterococcus*
1548	*Firmicutes*	*Bacilli*	*Lactobacillales*	*Enterococcaceae*	*Enterococcus*	*Enterococcus*
1549	*Firmicutes*	*Bacilli*	*Lactobacillales*	*Enterococcaceae*	*Enterococcus*	*Enterococcus*
1550	*Firmicutes*	*Bacilli*	*Lactobacillales*	*Enterococcaceae*	*Enterococcus*	*Enterococcus*
1551	*Firmicutes*	*Bacilli*	*Lactobacillales*	*Enterococcaceae*	*Enterococcus*	*Enterococcus*
1552	*Firmicutes*	*Bacilli*	*Lactobacillales*	*Enterococcaceae*	*Enterococcus*	*Enterococcus*
1553	*Firmicutes*	*Bacilli*	*Lactobacillales*	*Enterococcaceae*	*Enterococcus*	*Enterococcus*
1554	*Firmicutes*	*Bacilli*	*Lactobacillales*	*Enterococcaceae*	*Enterococcus*	*Enterococcus*
1555	*Firmicutes*	*Bacilli*	*Lactobacillales*	*Enterococcaceae*	*Enterococcus*	*Enterococcus*
1556	*Firmicutes*	*Bacilli*	*Lactobacillales*	*Enterococcaceae*	*Enterococcus*	*Enterococcus dispar*
1557	*Firmicutes*	*Bacilli*	*Lactobacillales*	*Enterococcaceae*	*Enterococcus*	*Enterococcus dispar*
1558	*Firmicutes*	*Bacilli*	*Lactobacillales*	*Enterococcaceae*	*Enterococcus*	*Enterococcus dispar*
1559	*Firmicutes*	*Bacilli*	*Lactobacillales*	*Enterococcaceae*	*Enterococcus*	*Enterococcus dispar*
1560	*Firmicutes*	*Bacilli*	*Lactobacillales*	*Enterococcaceae*	*Enterococcus*	*Enterococcus dispar*
1561	*Firmicutes*	*Bacilli*	*Lactobacillales*	*Enterococcaceae*	*Enterococcus*	*Enterococcus dispar*
1562	*Firmicutes*	*Bacilli*	*Lactobacillales*	*Enterococcaceae*	*Enterococcus*	*Enterococcus dispar*
1563	*Firmicutes*	*Bacilli*	*Lactobacillales*	*Enterococcaceae*	*Enterococcus*	*Enterococcus dispar*
1564	*Firmicutes*	*Bacilli*	*Lactobacillales*	*Enterococcaceae*	*Enterococcus*	*Enterococcus dispar*
1565	*Firmicutes*	*Bacilli*	*Lactobacillales*	*Enterococcaceae*	*Enterococcus*	*Enterococcus dispar*
1566	*Firmicutes*	*Bacilli*	*Lactobacillales*	*Enterococcaceae*	*Enterococcus*	*Enterococcus dispar*
1567	*Firmicutes*	*Bacilli*	*Lactobacillales*	*Enterococcaceae*	*Enterococcus*	*Enterococcus dispar*
1568	*Firmicutes*	*Bacilli*	*Lactobacillales*	*Enterococcaceae*	*Enterococcus*	*Enterococcus dispar*

Table S4. Isolated strains by culturomics

bacteriaId	*phylum*	*class*	*order*	*family*	*genus*	*species*
1569	*Firmicutes*	*Bacilli*	*Lactobacillales*	*Enterococcaceae*	*Enterococcus*	*Enterococcus dispar*
1570	*Firmicutes*	*Bacilli*	*Lactobacillales*	*Enterococcaceae*	*Enterococcus*	*Enterococcus dispar*
1571	*Firmicutes*	*Bacilli*	*Lactobacillales*	*Enterococcaceae*	*Enterococcus*	*Enterococcus dispar*
1572	*Firmicutes*	*Bacilli*	*Lactobacillales*	*Enterococcaceae*	*Enterococcus*	*Enterococcus dispar*
1573	*Firmicutes*	*Bacilli*	*Lactobacillales*	*Enterococcaceae*	*Enterococcus*	*Enterococcus dispar*
1574	*Firmicutes*	*Bacilli*	*Lactobacillales*	*Enterococcaceae*	*Enterococcus*	*Enterococcus dispar*
1575	*Firmicutes*	*Bacilli*	*Lactobacillales*	*Enterococcaceae*	*Enterococcus*	*Enterococcus dispar*
1576	*Firmicutes*	*Bacilli*	*Lactobacillales*	*Enterococcaceae*	*Enterococcus*	*Enterococcus dispar*
1577	*Firmicutes*	*Bacilli*	*Lactobacillales*	*Enterococcaceae*	*Enterococcus*	*Enterococcus dispar*
1578	*Firmicutes*	*Bacilli*	*Lactobacillales*	*Enterococcaceae*	*Enterococcus*	*Enterococcus dispar*
1579	*Firmicutes*	*Bacilli*	*Lactobacillales*	*Enterococcaceae*	*Enterococcus*	*Enterococcus dispar*
1580	*Firmicutes*	*Bacilli*	*Lactobacillales*	*Enterococcaceae*	*Enterococcus*	*Enterococcus dispar*
1581	*Firmicutes*	*Bacilli*	*Lactobacillales*	*Enterococcaceae*	*Enterococcus*	*Enterococcus dispar*
1582	*Firmicutes*	*Bacilli*	*Lactobacillales*	*Enterococcaceae*	*Enterococcus*	*Enterococcus dispar*
1583	*Firmicutes*	*Bacilli*	*Lactobacillales*	*Enterococcaceae*	*Enterococcus*	*Enterococcus dispar*
1584	*Firmicutes*	*Bacilli*	*Lactobacillales*	*Enterococcaceae*	*Enterococcus*	*Enterococcus dispar*
1585	*Firmicutes*	*Bacilli*	*Lactobacillales*	*Enterococcaceae*	*Enterococcus*	*Enterococcus dispar*
1586	*Firmicutes*	*Bacilli*	*Lactobacillales*	*Enterococcaceae*	*Enterococcus*	*Enterococcus dispar*
1587	*Firmicutes*	*Bacilli*	*Lactobacillales*	*Enterococcaceae*	*Enterococcus*	*Enterococcus dispar*
1588	*Firmicutes*	*Bacilli*	*Lactobacillales*	*Enterococcaceae*	*Enterococcus*	*Enterococcus dispar*
1589	*Firmicutes*	*Bacilli*	*Lactobacillales*	*Enterococcaceae*	*Enterococcus*	*Enterococcus dispar*
1590	*Firmicutes*	*Bacilli*	*Lactobacillales*	*Enterococcaceae*	*Enterococcus*	*Enterococcus dispar*
1591	*Firmicutes*	*Bacilli*	*Lactobacillales*	*Enterococcaceae*	*Enterococcus*	*Enterococcus dispar*
1592	*Firmicutes*	*Bacilli*	*Lactobacillales*	*Enterococcaceae*	*Enterococcus*	*Enterococcus dispar*
1593	*Firmicutes*	*Bacilli*	*Lactobacillales*	*Enterococcaceae*	*Enterococcus*	*Enterococcus dispar*
1594	*Firmicutes*	*Bacilli*	*Lactobacillales*	*Enterococcaceae*	*Enterococcus*	*Enterococcus dispar*
1595	*Firmicutes*	*Bacilli*	*Lactobacillales*	*Enterococcaceae*	*Enterococcus*	*Enterococcus dispar*
1596	*Firmicutes*	*Bacilli*	*Lactobacillales*	*Enterococcaceae*	*Enterococcus*	*Enterococcus dispar*

Table S4. Isolated strains by culturomics

bacteriaId	*phylum*	*class*	*order*	*family*	*genus*	*species*
1597	*Firmicutes*	*Bacilli*	*Lactobacillales*	*Enterococcaceae*	*Enterococcus*	*Enterococcus dispar*
1598	*Firmicutes*	*Bacilli*	*Lactobacillales*	*Enterococcaceae*	*Enterococcus*	*Enterococcus dispar*
1599	*Firmicutes*	*Bacilli*	*Lactobacillales*	*Enterococcaceae*	*Enterococcus*	*Enterococcus dispar*
1600	*Firmicutes*	*Bacilli*	*Lactobacillales*	*Enterococcaceae*	*Enterococcus*	*Enterococcus dispar*
1601	*Firmicutes*	*Bacilli*	*Lactobacillales*	*Enterococcaceae*	*Enterococcus*	*Enterococcus dispar*
1602	*Firmicutes*	*Bacilli*	*Lactobacillales*	*Enterococcaceae*	*Enterococcus*	*Enterococcus dispar*
1603	*Firmicutes*	*Bacilli*	*Lactobacillales*	*Enterococcaceae*	*Enterococcus*	*Enterococcus dispar*
1604	*Firmicutes*	*Bacilli*	*Lactobacillales*	*Enterococcaceae*	*Enterococcus*	*Enterococcus dispar*
1605	*Firmicutes*	*Bacilli*	*Lactobacillales*	*Enterococcaceae*	*Enterococcus*	*Enterococcus dispar*
1606	*Firmicutes*	*Bacilli*	*Lactobacillales*	*Enterococcaceae*	*Enterococcus*	*Enterococcus dispar*
1607	*Firmicutes*	*Bacilli*	*Lactobacillales*	*Enterococcaceae*	*Enterococcus*	*Enterococcus dispar*
1608	*Firmicutes*	*Bacilli*	*Lactobacillales*	*Enterococcaceae*	*Enterococcus*	*Enterococcus dispar*
1609	*Firmicutes*	*Bacilli*	*Lactobacillales*	*Enterococcaceae*	*Enterococcus*	*Enterococcus dispar*
1610	*Firmicutes*	*Bacilli*	*Lactobacillales*	*Enterococcaceae*	*Enterococcus*	*Enterococcus dispar*
1611	*Firmicutes*	*Bacilli*	*Lactobacillales*	*Enterococcaceae*	*Enterococcus*	*Enterococcus dispar*
1612	*Firmicutes*	*Bacilli*	*Lactobacillales*	*Enterococcaceae*	*Enterococcus*	*Enterococcus dispar*
1613	*Firmicutes*	*Bacilli*	*Lactobacillales*	*Enterococcaceae*	*Enterococcus*	*Enterococcus dispar*
1614	*Firmicutes*	*Bacilli*	*Lactobacillales*	*Enterococcaceae*	*Enterococcus*	*Enterococcus dispar*
1615	*Firmicutes*	*Bacilli*	*Lactobacillales*	*Enterococcaceae*	*Enterococcus*	*Enterococcus dispar*
1616	*Firmicutes*	*Bacilli*	*Lactobacillales*	*Enterococcaceae*	*Enterococcus*	*Enterococcus dispar*
1617	*Firmicutes*	*Bacilli*	*Lactobacillales*	*Enterococcaceae*	*Enterococcus*	*Enterococcus dispar*
1618	*Firmicutes*	*Bacilli*	*Lactobacillales*	*Enterococcaceae*	*Enterococcus*	*Enterococcus dispar*
1619	*Firmicutes*	*Bacilli*	*Lactobacillales*	*Enterococcaceae*	*Enterococcus*	*Enterococcus dispar*
1620	*Firmicutes*	*Bacilli*	*Lactobacillales*	*Enterococcaceae*	*Enterococcus*	*Enterococcus dispar*
1621	*Firmicutes*	*Bacilli*	*Lactobacillales*	*Enterococcaceae*	*Enterococcus*	*Enterococcus dispar*
1622	*Firmicutes*	*Bacilli*	*Lactobacillales*	*Enterococcaceae*	*Enterococcus*	*Enterococcus dispar*
1623	*Firmicutes*	*Bacilli*	*Lactobacillales*	*Enterococcaceae*	*Enterococcus*	*Enterococcus dispar*
1624	*Firmicutes*	*Bacilli*	*Lactobacillales*	*Enterococcaceae*	*Enterococcus*	*Enterococcus dispar*

Table S4. Isolated strains by culturomics

bacteriaId	*phylum*	*class*	*order*	*family*	*genus*	*species*
1625	*Firmicutes*	*Bacilli*	*Lactobacillales*	*Enterococcaceae*	*Enterococcus*	*Enterococcus dispar*
1626	*Firmicutes*	*Bacilli*	*Lactobacillales*	*Enterococcaceae*	*Enterococcus*	*Enterococcus dispar*
1627	*Firmicutes*	*Bacilli*	*Lactobacillales*	*Enterococcaceae*	*Enterococcus*	*Enterococcus dispar*
1628	*Firmicutes*	*Bacilli*	*Lactobacillales*	*Enterococcaceae*	*Enterococcus*	*Enterococcus dispar*
1629	*Firmicutes*	*Bacilli*	*Lactobacillales*	*Enterococcaceae*	*Enterococcus*	*Enterococcus dispar*
1630	*Firmicutes*	*Bacilli*	*Lactobacillales*	*Enterococcaceae*	*Enterococcus*	*Enterococcus dispar*
1631	*Firmicutes*	*Bacilli*	*Lactobacillales*	*Enterococcaceae*	*Enterococcus*	*Enterococcus dispar*
1632	*Firmicutes*	*Bacilli*	*Lactobacillales*	*Enterococcaceae*	*Enterococcus*	*Enterococcus dispar*
1633	*Firmicutes*	*Bacilli*	*Lactobacillales*	*Enterococcaceae*	*Enterococcus*	*Enterococcus dispar*
1634	*Firmicutes*	*Bacilli*	*Lactobacillales*	*Enterococcaceae*	*Enterococcus*	*Enterococcus dispar*
1635	*Firmicutes*	*Bacilli*	*Lactobacillales*	*Enterococcaceae*	*Enterococcus*	*Enterococcus dispar*
1636	*Firmicutes*	*Bacilli*	*Lactobacillales*	*Enterococcaceae*	*Enterococcus*	*Enterococcus dispar*
1637	*Firmicutes*	*Bacilli*	*Lactobacillales*	*Enterococcaceae*	*Enterococcus*	*Enterococcus dispar*
1638	*Firmicutes*	*Bacilli*	*Lactobacillales*	*Enterococcaceae*	*Enterococcus*	*Enterococcus dispar*
1639	*Firmicutes*	*Bacilli*	*Lactobacillales*	*Enterococcaceae*	*Enterococcus*	*Enterococcus dispar*
1640	*Firmicutes*	*Bacilli*	*Lactobacillales*	*Enterococcaceae*	*Enterococcus*	*Enterococcus dispar*
1641	*Firmicutes*	*Bacilli*	*Lactobacillales*	*Enterococcaceae*	*Enterococcus*	*Enterococcus dispar*
1642	*Firmicutes*	*Bacilli*	*Lactobacillales*	*Enterococcaceae*	*Enterococcus*	*Enterococcus dispar*
1643	*Firmicutes*	*Bacilli*	*Lactobacillales*	*Enterococcaceae*	*Enterococcus*	*Enterococcus dispar*
1644	*Firmicutes*	*Bacilli*	*Lactobacillales*	*Enterococcaceae*	*Enterococcus*	*Enterococcus dispar*
1645	*Firmicutes*	*Bacilli*	*Lactobacillales*	*Enterococcaceae*	*Enterococcus*	*Enterococcus dispar*
1646	*Firmicutes*	*Bacilli*	*Lactobacillales*	*Enterococcaceae*	*Enterococcus*	*Enterococcus dispar*
1647	*Firmicutes*	*Bacilli*	*Lactobacillales*	*Enterococcaceae*	*Enterococcus*	*Enterococcus dispar*
1648	*Firmicutes*	*Bacilli*	*Lactobacillales*	*Enterococcaceae*	*Enterococcus*	*Enterococcus dispar*
1649	*Firmicutes*	*Bacilli*	*Lactobacillales*	*Enterococcaceae*	*Enterococcus*	*Enterococcus dispar*
1650	*Firmicutes*	*Bacilli*	*Lactobacillales*	*Enterococcaceae*	*Enterococcus*	*Enterococcus dispar*
1651	*Firmicutes*	*Bacilli*	*Lactobacillales*	*Enterococcaceae*	*Enterococcus*	*Enterococcus dispar*
1652	*Firmicutes*	*Bacilli*	*Lactobacillales*	*Enterococcaceae*	*Enterococcus*	*Enterococcus dispar*

Table S4. Isolated strains by culturomics

bacteriaId	*phylum*	*class*	*order*	*family*	*genus*	*species*
1653	*Firmicutes*	*Bacilli*	*Lactobacillales*	*Enterococcaceae*	*Enterococcus*	*Enterococcus dispar*
1654	*Firmicutes*	*Bacilli*	*Lactobacillales*	*Enterococcaceae*	*Enterococcus*	*Enterococcus dispar*
1655	*Firmicutes*	*Bacilli*	*Lactobacillales*	*Enterococcaceae*	*Enterococcus*	*Enterococcus dispar*
1656	*Firmicutes*	*Bacilli*	*Lactobacillales*	*Enterococcaceae*	*Enterococcus*	*Enterococcus dispar*
1657	*Firmicutes*	*Bacilli*	*Lactobacillales*	*Enterococcaceae*	*Enterococcus*	*Enterococcus dispar*
1658	*Firmicutes*	*Bacilli*	*Lactobacillales*	*Enterococcaceae*	*Enterococcus*	*Enterococcus dispar*
1659	*Firmicutes*	*Bacilli*	*Lactobacillales*	*Enterococcaceae*	*Enterococcus*	*Enterococcus dispar*
1660	*Firmicutes*	*Bacilli*	*Lactobacillales*	*Enterococcaceae*	*Enterococcus*	*Enterococcus dispar*
1661	*Firmicutes*	*Bacilli*	*Lactobacillales*	*Enterococcaceae*	*Enterococcus*	*Enterococcus dispar*
1662	*Firmicutes*	*Bacilli*	*Lactobacillales*	*Enterococcaceae*	*Enterococcus*	*Enterococcus dispar*
1663	*Firmicutes*	*Bacilli*	*Lactobacillales*	*Enterococcaceae*	*Enterococcus*	*Enterococcus dispar*
1664	*Firmicutes*	*Bacilli*	*Lactobacillales*	*Enterococcaceae*	*Enterococcus*	*Enterococcus dispar*
1665	*Firmicutes*	*Bacilli*	*Lactobacillales*	*Enterococcaceae*	*Enterococcus*	*Enterococcus dispar*
1666	*Firmicutes*	*Bacilli*	*Lactobacillales*	*Enterococcaceae*	*Enterococcus*	*Enterococcus dispar*
1667	*Firmicutes*	*Bacilli*	*Lactobacillales*	*Enterococcaceae*	*Enterococcus*	*Enterococcus dispar*
1668	*Firmicutes*	*Bacilli*	*Lactobacillales*	*Enterococcaceae*	*Enterococcus*	*Enterococcus dispar*
1669	*Firmicutes*	*Bacilli*	*Lactobacillales*	*Enterococcaceae*	*Enterococcus*	*Enterococcus dispar*
1670	*Firmicutes*	*Bacilli*	*Lactobacillales*	*Enterococcaceae*	*Enterococcus*	*Enterococcus dispar*
1671	*Firmicutes*	*Bacilli*	*Lactobacillales*	*Enterococcaceae*	*Enterococcus*	*Enterococcus dispar*
1672	*Firmicutes*	*Bacilli*	*Lactobacillales*	*Enterococcaceae*	*Enterococcus*	*Enterococcus dispar*
1673	*Firmicutes*	*Bacilli*	*Lactobacillales*	*Enterococcaceae*	*Enterococcus*	*Enterococcus dispar*
1674	*Firmicutes*	*Bacilli*	*Lactobacillales*	*Enterococcaceae*	*Enterococcus*	*Enterococcus dispar*
1675	*Firmicutes*	*Bacilli*	*Lactobacillales*	*Enterococcaceae*	*Enterococcus*	*Enterococcus dispar*
1676	*Firmicutes*	*Bacilli*	*Lactobacillales*	*Enterococcaceae*	*Enterococcus*	*Enterococcus dispar*
1677	*Firmicutes*	*Bacilli*	*Lactobacillales*	*Enterococcaceae*	*Enterococcus*	*Enterococcus dispar*
1678	*Firmicutes*	*Bacilli*	*Lactobacillales*	*Enterococcaceae*	*Enterococcus*	*Enterococcus dispar*
1679	*Firmicutes*	*Bacilli*	*Lactobacillales*	*Enterococcaceae*	*Enterococcus*	*Enterococcus dispar*
1680	*Firmicutes*	*Bacilli*	*Lactobacillales*	*Enterococcaceae*	*Enterococcus*	*Enterococcus dispar*

Table S4. Isolated strains by culturomics

bacteriaId	*phylum*	*class*	*order*	*family*	*genus*	*species*
1681	*Firmicutes*	*Bacilli*	*Lactobacillales*	*Enterococcaceae*	*Enterococcus*	*Enterococcus dispar*
1682	*Firmicutes*	*Bacilli*	*Lactobacillales*	*Enterococcaceae*	*Enterococcus*	*Enterococcus dispar*
1683	*Firmicutes*	*Bacilli*	*Lactobacillales*	*Enterococcaceae*	*Enterococcus*	*Enterococcus dispar*
1684	*Firmicutes*	*Bacilli*	*Lactobacillales*	*Enterococcaceae*	*Enterococcus*	*Enterococcus dispar*
1685	*Firmicutes*	*Bacilli*	*Lactobacillales*	*Enterococcaceae*	*Enterococcus*	*Enterococcus dispar*
1686	*Firmicutes*	*Bacilli*	*Lactobacillales*	*Enterococcaceae*	*Enterococcus*	*Enterococcus dispar*
1687	*Firmicutes*	*Bacilli*	*Lactobacillales*	*Enterococcaceae*	*Enterococcus*	*Enterococcus dispar*
1688	*Firmicutes*	*Bacilli*	*Lactobacillales*	*Enterococcaceae*	*Enterococcus*	*Enterococcus dispar*
1689	*Firmicutes*	*Bacilli*	*Lactobacillales*	*Enterococcaceae*	*Enterococcus*	*Enterococcus dispar*
1690	*Firmicutes*	*Bacilli*	*Lactobacillales*	*Enterococcaceae*	*Enterococcus*	*Enterococcus dispar*
1691	*Firmicutes*	*Bacilli*	*Lactobacillales*	*Enterococcaceae*	*Enterococcus*	*Enterococcus dispar*
1692	*Firmicutes*	*Bacilli*	*Lactobacillales*	*Enterococcaceae*	*Enterococcus*	*Enterococcus dispar*
1693	*Firmicutes*	*Bacilli*	*Lactobacillales*	*Enterococcaceae*	*Enterococcus*	*Enterococcus dispar*
1694	*Firmicutes*	*Bacilli*	*Lactobacillales*	*Enterococcaceae*	*Enterococcus*	*Enterococcus dispar*
1695	*Firmicutes*	*Bacilli*	*Lactobacillales*	*Enterococcaceae*	*Enterococcus*	*Enterococcus dispar*
1696	*Firmicutes*	*Bacilli*	*Lactobacillales*	*Enterococcaceae*	*Enterococcus*	*Enterococcus dispar*
1697	*Firmicutes*	*Bacilli*	*Lactobacillales*	*Enterococcaceae*	*Enterococcus*	*Enterococcus dispar*
1698	*Firmicutes*	*Bacilli*	*Lactobacillales*	*Enterococcaceae*	*Enterococcus*	*Enterococcus dispar*
1699	*Firmicutes*	*Bacilli*	*Lactobacillales*	*Enterococcaceae*	*Enterococcus*	*Enterococcus dispar*
1700	*Firmicutes*	*Bacilli*	*Lactobacillales*	*Enterococcaceae*	*Enterococcus*	*Enterococcus dispar*
1701	*Firmicutes*	*Bacilli*	*Lactobacillales*	*Enterococcaceae*	*Enterococcus*	*Enterococcus dispar*
1702	*Firmicutes*	*Bacilli*	*Lactobacillales*	*Enterococcaceae*	*Enterococcus*	*Enterococcus dispar*
1703	*Firmicutes*	*Bacilli*	*Lactobacillales*	*Enterococcaceae*	*Enterococcus*	*Enterococcus dispar*
1704	*Firmicutes*	*Bacilli*	*Lactobacillales*	*Enterococcaceae*	*Enterococcus*	*Enterococcus dispar*
1705	*Firmicutes*	*Bacilli*	*Lactobacillales*	*Enterococcaceae*	*Enterococcus*	*Enterococcus dispar*
1706	*Firmicutes*	*Bacilli*	*Lactobacillales*	*Enterococcaceae*	*Enterococcus*	*Enterococcus dispar*
1707	*Firmicutes*	*Bacilli*	*Lactobacillales*	*Enterococcaceae*	*Enterococcus*	*Enterococcus dispar*
1708	*Firmicutes*	*Bacilli*	*Lactobacillales*	*Enterococcaceae*	*Enterococcus*	*Enterococcus dispar*

Table S4. Isolated strains by culturomics

bacterialId	*phylum*	*class*	*order*	*family*	*genus*	*species*
1709	*Firmicutes*	*Bacilli*	*Lactobacillales*	*Enterococcaceae*	*Enterococcus*	*Enterococcus dispar*
1710	*Firmicutes*	*Bacilli*	*Lactobacillales*	*Enterococcaceae*	*Enterococcus*	*Enterococcus dispar*
1711	*Firmicutes*	*Bacilli*	*Lactobacillales*	*Enterococcaceae*	*Enterococcus*	*Enterococcus dispar*
1712	*Firmicutes*	*Bacilli*	*Lactobacillales*	*Enterococcaceae*	*Enterococcus*	*Enterococcus dispar*
1713	*Firmicutes*	*Bacilli*	*Lactobacillales*	*Enterococcaceae*	*Enterococcus*	*Enterococcus dispar*
1714	*Firmicutes*	*Bacilli*	*Lactobacillales*	*Enterococcaceae*	*Enterococcus*	*Enterococcus dispar*
1715	*Firmicutes*	*Bacilli*	*Lactobacillales*	*Enterococcaceae*	*Enterococcus*	*Enterococcus dispar*
1716	*Firmicutes*	*Bacilli*	*Lactobacillales*	*Enterococcaceae*	*Enterococcus*	*Enterococcus dispar*
1717	*Firmicutes*	*Bacilli*	*Lactobacillales*	*Enterococcaceae*	*Enterococcus*	*Enterococcus dispar*
1718	*Firmicutes*	*Bacilli*	*Lactobacillales*	*Enterococcaceae*	*Enterococcus*	*Enterococcus dispar*
1719	*Firmicutes*	*Bacilli*	*Lactobacillales*	*Enterococcaceae*	*Enterococcus*	*Enterococcus dispar*
1720	*Firmicutes*	*Bacilli*	*Lactobacillales*	*Enterococcaceae*	*Enterococcus*	*Enterococcus dispar*
1721	*Firmicutes*	*Bacilli*	*Lactobacillales*	*Enterococcaceae*	*Enterococcus*	*Enterococcus dispar*
1722	*Firmicutes*	*Bacilli*	*Lactobacillales*	*Enterococcaceae*	*Enterococcus*	*Enterococcus dispar*
1723	*Firmicutes*	*Bacilli*	*Lactobacillales*	*Enterococcaceae*	*Enterococcus*	*Enterococcus dispar*
1724	*Firmicutes*	*Bacilli*	*Lactobacillales*	*Enterococcaceae*	*Enterococcus*	*Enterococcus dispar*
1725	*Firmicutes*	*Bacilli*	*Lactobacillales*	*Enterococcaceae*	*Enterococcus*	*Enterococcus dispar*
1726	*Firmicutes*	*Bacilli*	*Lactobacillales*	*Enterococcaceae*	*Enterococcus*	*Enterococcus dispar*
1727	*Firmicutes*	*Bacilli*	*Lactobacillales*	*Enterococcaceae*	*Enterococcus*	*Enterococcus dispar*
1728	*Firmicutes*	*Bacilli*	*Lactobacillales*	*Enterococcaceae*	*Enterococcus*	*Enterococcus dispar*
1729	*Firmicutes*	*Bacilli*	*Lactobacillales*	*Enterococcaceae*	*Enterococcus*	*Enterococcus dispar*
1730	*Firmicutes*	*Bacilli*	*Lactobacillales*	*Enterococcaceae*	*Enterococcus*	*Enterococcus dispar*
1731	*Firmicutes*	*Bacilli*	*Lactobacillales*	*Enterococcaceae*	*Enterococcus*	*Enterococcus dispar*
1732	*Firmicutes*	*Bacilli*	*Lactobacillales*	*Enterococcaceae*	*Enterococcus*	*Enterococcus dispar*
1733	*Firmicutes*	*Bacilli*	*Lactobacillales*	*Enterococcaceae*	*Enterococcus*	*Enterococcus dispar*
1734	*Firmicutes*	*Bacilli*	*Lactobacillales*	*Enterococcaceae*	*Enterococcus*	*Enterococcus dispar*
1735	*Firmicutes*	*Bacilli*	*Lactobacillales*	*Enterococcaceae*	*Enterococcus*	*Enterococcus dispar*
1736	*Firmicutes*	*Bacilli*	*Lactobacillales*	*Enterococcaceae*	*Enterococcus*	*Enterococcus dispar*

Table S4. Isolated strains by culturomics

bacteriaId	*phylum*	*class*	*order*	*family*	*genus*	*species*
1737	*Firmicutes*	*Bacilli*	*Lactobacillales*	*Enterococcaceae*	*Enterococcus*	*Enterococcus dispar*
1738	*Firmicutes*	*Bacilli*	*Lactobacillales*	*Enterococcaceae*	*Enterococcus*	*Enterococcus dispar*
1739	*Firmicutes*	*Bacilli*	*Lactobacillales*	*Enterococcaceae*	*Enterococcus*	*Enterococcus dispar*
1740	*Firmicutes*	*Bacilli*	*Lactobacillales*	*Enterococcaceae*	*Enterococcus*	*Enterococcus dispar*
1741	*Firmicutes*	*Bacilli*	*Lactobacillales*	*Enterococcaceae*	*Enterococcus*	*Enterococcus dispar*
1742	*Firmicutes*	*Bacilli*	*Lactobacillales*	*Enterococcaceae*	*Enterococcus*	*Enterococcus dispar*
1743	*Firmicutes*	*Bacilli*	*Lactobacillales*	*Enterococcaceae*	*Enterococcus*	*Enterococcus dispar*
1744	*Firmicutes*	*Bacilli*	*Lactobacillales*	*Enterococcaceae*	*Enterococcus*	*Enterococcus dispar*
1745	*Firmicutes*	*Bacilli*	*Lactobacillales*	*Enterococcaceae*	*Enterococcus*	*Enterococcus dispar*
1746	*Firmicutes*	*Bacilli*	*Lactobacillales*	*Enterococcaceae*	*Enterococcus*	*Enterococcus dispar*
1747	*Firmicutes*	*Bacilli*	*Lactobacillales*	*Enterococcaceae*	*Enterococcus*	*Enterococcus dispar*
1748	*Firmicutes*	*Bacilli*	*Lactobacillales*	*Enterococcaceae*	*Enterococcus*	*Enterococcus dispar*
1749	*Firmicutes*	*Bacilli*	*Lactobacillales*	*Enterococcaceae*	*Enterococcus*	*Enterococcus dispar*
1750	*Firmicutes*	*Bacilli*	*Lactobacillales*	*Enterococcaceae*	*Enterococcus*	*Enterococcus dispar*
1751	*Firmicutes*	*Bacilli*	*Lactobacillales*	*Enterococcaceae*	*Enterococcus*	*Enterococcus dispar*
1752	*Firmicutes*	*Bacilli*	*Lactobacillales*	*Enterococcaceae*	*Enterococcus*	*Enterococcus dispar*
1753	*Firmicutes*	*Bacilli*	*Lactobacillales*	*Enterococcaceae*	*Enterococcus*	*Enterococcus dispar*
1754	*Firmicutes*	*Bacilli*	*Lactobacillales*	*Enterococcaceae*	*Enterococcus*	*Enterococcus dispar*
1755	*Firmicutes*	*Bacilli*	*Lactobacillales*	*Enterococcaceae*	*Enterococcus*	*Enterococcus dispar*
1756	*Firmicutes*	*Bacilli*	*Lactobacillales*	*Enterococcaceae*	*Enterococcus*	*Enterococcus dispar*
1757	*Firmicutes*	*Bacilli*	*Lactobacillales*	*Enterococcaceae*	*Enterococcus*	*Enterococcus dispar*
1758	*Firmicutes*	*Bacilli*	*Lactobacillales*	*Enterococcaceae*	*Enterococcus*	*Enterococcus dispar*
1759	*Firmicutes*	*Bacilli*	*Lactobacillales*	*Enterococcaceae*	*Enterococcus*	*Enterococcus dispar*
1760	*Firmicutes*	*Bacilli*	*Lactobacillales*	*Enterococcaceae*	*Enterococcus*	*Enterococcus dispar*
1761	*Firmicutes*	*Bacilli*	*Lactobacillales*	*Enterococcaceae*	*Enterococcus*	*Enterococcus dispar*
1762	*Firmicutes*	*Bacilli*	*Lactobacillales*	*Enterococcaceae*	*Enterococcus*	*Enterococcus dispar*
1763	*Firmicutes*	*Bacilli*	*Lactobacillales*	*Enterococcaceae*	*Enterococcus*	*Enterococcus*
1764	*Firmicutes*	*Bacilli*	*Lactobacillales*	*Enterococcaceae*	*Enterococcus*	*Enterococcus*

Table S4. Isolated strains by culturomics

bacterialId	*phylum*	*class*	*order*	*family*	*genus*	*species*
1765	*Firmicutes*	*Bacilli*	*Lactobacillales*	*Enterococcaceae*	*Enterococcus*	*Enterococcus*
1766	*Firmicutes*	*Bacilli*	*Lactobacillales*	*Enterococcaceae*	*Enterococcus*	*Enterococcus*
1767	*Firmicutes*	*Bacilli*	*Lactobacillales*	*Enterococcaceae*	*Enterococcus*	*Enterococcus*
1768	*Firmicutes*	*Bacilli*	*Lactobacillales*	*Enterococcaceae*	*Enterococcus*	*Enterococcus*
1769	*Firmicutes*	*Bacilli*	*Lactobacillales*	*Enterococcaceae*	*Enterococcus*	*Enterococcus*
1770	*Firmicutes*	*Bacilli*	*Lactobacillales*	*Enterococcaceae*	*Enterococcus*	*Enterococcus*
1771	*Firmicutes*	*Bacilli*	*Lactobacillales*	*Enterococcaceae*	*Enterococcus*	*Enterococcus*
1772	*Firmicutes*	*Bacilli*	*Lactobacillales*	*Enterococcaceae*	*Enterococcus*	*Enterococcus*
1773	*Firmicutes*	*Bacilli*	*Lactobacillales*	*Enterococcaceae*	*Enterococcus*	*Enterococcus*
1774	*Firmicutes*	*Bacilli*	*Lactobacillales*	*Enterococcaceae*	*Enterococcus*	*Enterococcus*
1775	*Firmicutes*	*Bacilli*	*Lactobacillales*	*Enterococcaceae*	*Enterococcus*	*Enterococcus*
1776	*Firmicutes*	*Bacilli*	*Lactobacillales*	*Enterococcaceae*	*Enterococcus*	*Enterococcus*
1777	*Firmicutes*	*Bacilli*	*Lactobacillales*	*Enterococcaceae*	*Enterococcus*	*Enterococcus*
1778	*Firmicutes*	*Bacilli*	*Lactobacillales*	*Enterococcaceae*	*Enterococcus*	*Enterococcus*
1779	*Firmicutes*	*Bacilli*	*Lactobacillales*	*Enterococcaceae*	*Enterococcus*	*Enterococcus*
1780	*Firmicutes*	*Bacilli*	*Lactobacillales*	*Enterococcaceae*	*Enterococcus*	*Enterococcus*
1781	*Firmicutes*	*Bacilli*	*Lactobacillales*	*Enterococcaceae*	*Enterococcus*	*Enterococcus*
1782	*Firmicutes*	*Bacilli*	*Lactobacillales*	*Enterococcaceae*	*Enterococcus*	*Enterococcus*
1783	*Firmicutes*	*Bacilli*	*Lactobacillales*	*Enterococcaceae*	*Enterococcus*	*Enterococcus*
1784	*Firmicutes*	*Bacilli*	*Lactobacillales*	*Enterococcaceae*	*Enterococcus*	*Enterococcus*
1785	*Firmicutes*	*Bacilli*	*Lactobacillales*	*Enterococcaceae*	*Enterococcus*	*Enterococcus*
1786	*Firmicutes*	*Bacilli*	*Lactobacillales*	*Enterococcaceae*	*Enterococcus*	*Enterococcus*
1787	*Firmicutes*	*Bacilli*	*Lactobacillales*	*Enterococcaceae*	*Enterococcus*	*Enterococcus*
1788	*Firmicutes*	*Bacilli*	*Lactobacillales*	*Enterococcaceae*	*Enterococcus*	*Enterococcus*
1789	*Firmicutes*	*Bacilli*	*Lactobacillales*	*Enterococcaceae*	*Enterococcus*	*Enterococcus*
1790	*Firmicutes*	*Bacilli*	*Lactobacillales*	*Enterococcaceae*	*Enterococcus*	*Enterococcus*
1791	*Firmicutes*	*Bacilli*	*Lactobacillales*	*Enterococcaceae*	*Enterococcus*	*Enterococcus*
1792	*Firmicutes*	*Bacilli*	*Lactobacillales*	*Enterococcaceae*	*Enterococcus*	*Enterococcus*

Table S4. Isolated strains by culturomics

bacteriaId	*phylum*	*class*	*order*	*family*	*genus*	*species*
1793	*Firmicutes*	*Bacilli*	*Lactobacillales*	*Enterococcaceae*	*Enterococcus*	*Enterococcus*
1794	*Firmicutes*	*Bacilli*	*Lactobacillales*	*Enterococcaceae*	*Enterococcus*	*Enterococcus*
1795	*Firmicutes*	*Bacilli*	*Lactobacillales*	*Enterococcaceae*	*Enterococcus*	*Enterococcus*
1796	*Firmicutes*	*Bacilli*	*Lactobacillales*	*Enterococcaceae*	*Enterococcus*	*Enterococcus*
1797	*Firmicutes*	*Bacilli*	*Lactobacillales*	*Enterococcaceae*	*Enterococcus*	*Enterococcus*
1798	*Firmicutes*	*Bacilli*	*Lactobacillales*	*Enterococcaceae*	*Enterococcus*	*Enterococcus*
1799	*Firmicutes*	*Bacilli*	*Lactobacillales*	*Enterococcaceae*	*Enterococcus*	*Enterococcus*
1800	*Firmicutes*	*Bacilli*	*Lactobacillales*	*Enterococcaceae*	*Enterococcus*	*Enterococcus*
1801	*Firmicutes*	*Bacilli*	*Lactobacillales*	*Enterococcaceae*	*Enterococcus*	*Enterococcus*
1802	*Firmicutes*	*Bacilli*	*Lactobacillales*	*Enterococcaceae*	*Enterococcus*	*Enterococcus*
1803	*Firmicutes*	*Bacilli*	*Lactobacillales*	*Enterococcaceae*	*Enterococcus*	*Enterococcus*
1804	*Firmicutes*	*Bacilli*	*Lactobacillales*	*Enterococcaceae*	*Enterococcus*	*Enterococcus*
1805	*Firmicutes*	*Bacilli*	*Lactobacillales*	*Enterococcaceae*	*Enterococcus*	*Enterococcus*
1806	*Firmicutes*	*Bacilli*	*Lactobacillales*	*Enterococcaceae*	*Enterococcus*	*Enterococcus*
1807	*Firmicutes*	*Bacilli*	*Lactobacillales*	*Enterococcaceae*	*Enterococcus*	*Enterococcus*
1808	*Firmicutes*	*Bacilli*	*Lactobacillales*	*Enterococcaceae*	*Enterococcus*	*Enterococcus*
1809	*Firmicutes*	*Bacilli*	*Lactobacillales*	*Enterococcaceae*	*Enterococcus*	*Enterococcus*
1810	*Firmicutes*	*Bacilli*	*Lactobacillales*	*Enterococcaceae*	*Enterococcus*	*Enterococcus*
1811	*Firmicutes*	*Bacilli*	*Lactobacillales*	*Enterococcaceae*	*Enterococcus*	*Enterococcus*
1812	*Firmicutes*	*Bacilli*	*Lactobacillales*	*Enterococcaceae*	*Enterococcus*	*Enterococcus*
1813	*Firmicutes*	*Bacilli*	*Lactobacillales*	*Enterococcaceae*	*Enterococcus*	*Enterococcus*
1814	*Firmicutes*	*Bacilli*	*Lactobacillales*	*Enterococcaceae*	*Enterococcus*	*Enterococcus*
1815	*Firmicutes*	*Bacilli*	*Lactobacillales*	*Enterococcaceae*	*Enterococcus*	*Enterococcus*
1816	*Firmicutes*	*Bacilli*	*Lactobacillales*	*Enterococcaceae*	*Enterococcus*	*Enterococcus*
1817	*Firmicutes*	*Bacilli*	*Lactobacillales*	*Enterococcaceae*	*Enterococcus*	*Enterococcus*
1818	*Firmicutes*	*Bacilli*	*Lactobacillales*	*Enterococcaceae*	*Enterococcus*	*Enterococcus*
1819	*Firmicutes*	*Bacilli*	*Lactobacillales*	*Enterococcaceae*	*Enterococcus*	*Enterococcus*
1820	*Firmicutes*	*Bacilli*	*Lactobacillales*	*Enterococcaceae*	*Enterococcus*	*Enterococcus*

Table S4. Isolated strains by culturomics

bacteriaId	*phylum*	*class*	*order*	*family*	*genus*	*species*
1821	*Firmicutes*	*Bacilli*	*Lactobacillales*	*Enterococcaceae*	*Enterococcus*	*Enterococcus*
1822	*Firmicutes*	*Bacilli*	*Lactobacillales*	*Enterococcaceae*	*Enterococcus*	*Enterococcus*
1823	*Firmicutes*	*Bacilli*	*Lactobacillales*	*Enterococcaceae*	*Enterococcus*	*Enterococcus*
1824	*Firmicutes*	*Bacilli*	*Lactobacillales*	*Enterococcaceae*	*Enterococcus*	*Enterococcus*
1825	*Firmicutes*	*Bacilli*	*Lactobacillales*	*Enterococcaceae*	*Enterococcus*	*Enterococcus*
1826	*Firmicutes*	*Bacilli*	*Lactobacillales*	*Enterococcaceae*	*Enterococcus*	*Enterococcus*
1827	*Firmicutes*	*Bacilli*	*Lactobacillales*	*Enterococcaceae*	*Enterococcus*	*Enterococcus*
1828	*Firmicutes*	*Bacilli*	*Lactobacillales*	*Enterococcaceae*	*Enterococcus*	*Enterococcus*
1829	*Firmicutes*	*Bacilli*	*Lactobacillales*	*Enterococcaceae*	*Enterococcus*	*Enterococcus*
1830	*Firmicutes*	*Bacilli*	*Lactobacillales*	*Enterococcaceae*	*Enterococcus*	*Enterococcus*
1831	*Firmicutes*	*Bacilli*	*Lactobacillales*	*Enterococcaceae*	*Enterococcus*	*Enterococcus*
1832	*Firmicutes*	*Bacilli*	*Lactobacillales*	*Enterococcaceae*	*Enterococcus*	*Enterococcus*
1833	*Firmicutes*	*Bacilli*	*Lactobacillales*	*Enterococcaceae*	*Enterococcus*	*Enterococcus*
1834	*Firmicutes*	*Bacilli*	*Lactobacillales*	*Enterococcaceae*	*Enterococcus*	*Enterococcus*
1835	*Firmicutes*	*Bacilli*	*Lactobacillales*	*Enterococcaceae*	*Enterococcus*	*Enterococcus*
1836	*Firmicutes*	*Bacilli*	*Lactobacillales*	*Enterococcaceae*	*Enterococcus*	*Enterococcus*
1837	*Firmicutes*	*Bacilli*	*Lactobacillales*	*Enterococcaceae*	*Enterococcus*	*Enterococcus*
1838	*Firmicutes*	*Bacilli*	*Lactobacillales*	*Enterococcaceae*	*Enterococcus*	*Enterococcus*
1839	*Firmicutes*	*Bacilli*	*Lactobacillales*	*Enterococcaceae*	*Enterococcus*	*Enterococcus*
1840	*Firmicutes*	*Bacilli*	*Lactobacillales*	*Enterococcaceae*	*Enterococcus*	*Enterococcus*
1841	*Firmicutes*	*Bacilli*	*Lactobacillales*	*Enterococcaceae*	*Enterococcus*	*Enterococcus*
1842	*Firmicutes*	*Bacilli*	*Lactobacillales*	*Enterococcaceae*	*Enterococcus*	*Enterococcus*
1843	*Firmicutes*	*Bacilli*	*Lactobacillales*	*Enterococcaceae*	*Enterococcus*	*Enterococcus*
1844	*Firmicutes*	*Bacilli*	*Lactobacillales*	*Enterococcaceae*	*Enterococcus*	*Enterococcus*
1845	*Firmicutes*	*Bacilli*	*Lactobacillales*	*Enterococcaceae*	*Enterococcus*	*Enterococcus*
1846	*Firmicutes*	*Bacilli*	*Lactobacillales*	*Enterococcaceae*	*Enterococcus*	*Enterococcus*
1847	*Firmicutes*	*Bacilli*	*Lactobacillales*	*Enterococcaceae*	*Enterococcus*	*Enterococcus*
1848	*Firmicutes*	*Bacilli*	*Lactobacillales*	*Enterococcaceae*	*Enterococcus*	*Enterococcus*

Table S4. Isolated strains by culturomics

bacteriaId	*phylum*	*class*	*order*	*family*	*genus*	*species*
1849	*Firmicutes*	*Bacilli*	*Lactobacillales*	*Enterococcaceae*	*Enterococcus*	*Enterococcus*
1850	*Firmicutes*	*Bacilli*	*Lactobacillales*	*Enterococcaceae*	*Enterococcus*	*Enterococcus*
1851	*Firmicutes*	*Bacilli*	*Lactobacillales*	*Enterococcaceae*	*Enterococcus*	*Enterococcus*
1852	*Firmicutes*	*Bacilli*	*Lactobacillales*	*Enterococcaceae*	*Enterococcus*	*Enterococcus*
1853	*Firmicutes*	*Bacilli*	*Lactobacillales*	*Enterococcaceae*	*Enterococcus*	*Enterococcus*
1854	*Firmicutes*	*Bacilli*	*Lactobacillales*	*Enterococcaceae*	*Enterococcus*	*Enterococcus*
1855	*Firmicutes*	*Bacilli*	*Lactobacillales*	*Enterococcaceae*	*Enterococcus*	*Enterococcus*
1856	*Firmicutes*	*Bacilli*	*Lactobacillales*	*Enterococcaceae*	*Enterococcus*	*Enterococcus*
1857	*Firmicutes*	*Bacilli*	*Lactobacillales*	*Enterococcaceae*	*Enterococcus*	*Enterococcus*
1858	*Firmicutes*	*Bacilli*	*Lactobacillales*	*Enterococcaceae*	*Enterococcus*	*Enterococcus*
1859	*Firmicutes*	*Bacilli*	*Lactobacillales*	*Enterococcaceae*	*Enterococcus*	*Enterococcus*
1860	*Firmicutes*	*Bacilli*	*Lactobacillales*	*Enterococcaceae*	*Enterococcus*	*Enterococcus*
1861	*Firmicutes*	*Bacilli*	*Lactobacillales*	*Enterococcaceae*	*Enterococcus*	*Enterococcus*
1862	*Firmicutes*	*Bacilli*	*Lactobacillales*	*Enterococcaceae*	*Enterococcus*	*Enterococcus*
1863	*Firmicutes*	*Bacilli*	*Lactobacillales*	*Enterococcaceae*	*Enterococcus*	*Enterococcus*
1864	*Firmicutes*	*Bacilli*	*Lactobacillales*	*Enterococcaceae*	*Enterococcus*	*Enterococcus*
1865	*Firmicutes*	*Bacilli*	*Lactobacillales*	*Enterococcaceae*	*Enterococcus*	*Enterococcus*
1866	*Firmicutes*	*Bacilli*	*Lactobacillales*	*Enterococcaceae*	*Enterococcus*	*Enterococcus*
1867	*Firmicutes*	*Bacilli*	*Lactobacillales*	*Enterococcaceae*	*Enterococcus*	*Enterococcus*
1868	*Firmicutes*	*Bacilli*	*Lactobacillales*	*Enterococcaceae*	*Enterococcus*	*Enterococcus*
1869	*Firmicutes*	*Bacilli*	*Lactobacillales*	*Enterococcaceae*	*Enterococcus*	*Enterococcus*
1870	*Firmicutes*	*Bacilli*	*Lactobacillales*	*Enterococcaceae*	*Enterococcus*	*Enterococcus*
1871	*Firmicutes*	*Bacilli*	*Lactobacillales*	*Enterococcaceae*	*Enterococcus*	*Enterococcus*
1872	*Firmicutes*	*Bacilli*	*Lactobacillales*	*Enterococcaceae*	*Enterococcus*	*Enterococcus*
1873	*Firmicutes*	*Bacilli*	*Lactobacillales*	*Enterococcaceae*	*Enterococcus*	*Enterococcus*
1874	*Firmicutes*	*Bacilli*	*Lactobacillales*	*Enterococcaceae*	*Enterococcus*	*Enterococcus*
1875	*Firmicutes*	*Bacilli*	*Lactobacillales*	*Enterococcaceae*	*Enterococcus*	*Enterococcus*
1876	*Firmicutes*	*Bacilli*	*Lactobacillales*	*Enterococcaceae*	*Enterococcus*	*Enterococcus*

Table S4. Isolated strains by culturomics

bacteriaId	*phylum*	*class*	*order*	*family*	*genus*	*species*
1877	*Firmicutes*	*Bacilli*	*Lactobacillales*	*Enterococcaceae*	*Enterococcus*	*Enterococcus*
1878	*Firmicutes*	*Bacilli*	*Lactobacillales*	*Enterococcaceae*	*Enterococcus*	*Enterococcus*
1879	*Firmicutes*	*Bacilli*	*Lactobacillales*	*Enterococcaceae*	*Enterococcus*	*Enterococcus*
1880	*Firmicutes*	*Bacilli*	*Lactobacillales*	*Enterococcaceae*	*Enterococcus*	*Enterococcus*
1881	*Firmicutes*	*Bacilli*	*Lactobacillales*	*Enterococcaceae*	*Enterococcus*	*Enterococcus*
1882	*Firmicutes*	*Bacilli*	*Lactobacillales*	*Enterococcaceae*	*Enterococcus*	*Enterococcus*
1883	*Firmicutes*	*Bacilli*	*Lactobacillales*	*Enterococcaceae*	*Enterococcus*	*Enterococcus*
1884	*Firmicutes*	*Bacilli*	*Lactobacillales*	*Enterococcaceae*	*Enterococcus*	*Enterococcus*
1885	*Firmicutes*	*Bacilli*	*Lactobacillales*	*Enterococcaceae*	*Enterococcus*	*Enterococcus*
1886	*Firmicutes*	*Bacilli*	*Lactobacillales*	*Enterococcaceae*	*Enterococcus*	*Enterococcus*
1887	*Firmicutes*	*Bacilli*	*Lactobacillales*	*Enterococcaceae*	*Enterococcus*	*Enterococcus*
1888	*Firmicutes*	*Bacilli*	*Lactobacillales*	*Enterococcaceae*	*Enterococcus*	*Enterococcus*
1889	*Firmicutes*	*Bacilli*	*Lactobacillales*	*Enterococcaceae*	*Enterococcus*	*Enterococcus*
1890	*Firmicutes*	*Bacilli*	*Lactobacillales*	*Enterococcaceae*	*Enterococcus*	*Enterococcus*
1891	*Firmicutes*	*Bacilli*	*Lactobacillales*	*Enterococcaceae*	*Enterococcus*	*Enterococcus*
1892	*Firmicutes*	*Bacilli*	*Lactobacillales*	*Enterococcaceae*	*Enterococcus*	*Enterococcus*
1893	*Firmicutes*	*Bacilli*	*Lactobacillales*	*Enterococcaceae*	*Enterococcus*	*Enterococcus*
1894	*Firmicutes*	*Bacilli*	*Lactobacillales*	*Enterococcaceae*	*Enterococcus*	*Enterococcus*
1895	*Firmicutes*	*Bacilli*	*Lactobacillales*	*Enterococcaceae*	*Enterococcus*	*Enterococcus*
1896	*Firmicutes*	*Bacilli*	*Lactobacillales*	*Enterococcaceae*	*Enterococcus*	*Enterococcus*
1897	*Firmicutes*	*Bacilli*	*Lactobacillales*	*Enterococcaceae*	*Enterococcus*	*Enterococcus*
1898	*Firmicutes*	*Bacilli*	*Lactobacillales*	*Enterococcaceae*	*Enterococcus*	*Enterococcus*
1899	*Firmicutes*	*Bacilli*	*Lactobacillales*	*Enterococcaceae*	*Enterococcus*	*Enterococcus*
1900	*Firmicutes*	*Bacilli*	*Lactobacillales*	*Enterococcaceae*	*Enterococcus*	*Enterococcus*
1901	*Firmicutes*	*Bacilli*	*Lactobacillales*	*Enterococcaceae*	*Enterococcus*	*Enterococcus*
1902	*Firmicutes*	*Bacilli*	*Lactobacillales*	*Enterococcaceae*	*Enterococcus*	*Enterococcus*
1903	*Firmicutes*	*Bacilli*	*Lactobacillales*	*Enterococcaceae*	*Enterococcus*	*Enterococcus*
1904	*Firmicutes*	*Bacilli*	*Lactobacillales*	*Enterococcaceae*	*Enterococcus*	*Enterococcus*

Table S4. Isolated strains by culturomics

bacteriaId	*phylum*	*class*	*order*	*family*	*genus*	*species*
1905	*Firmicutes*	*Bacilli*	*Lactobacillales*	*Enterococcaceae*	*Enterococcus*	*Enterococcus*
1906	*Firmicutes*	*Bacilli*	*Lactobacillales*	*Enterococcaceae*	*Enterococcus*	*Enterococcus*
1907	*Firmicutes*	*Bacilli*	*Lactobacillales*	*Enterococcaceae*	*Enterococcus*	*Enterococcus*
1908	*Firmicutes*	*Bacilli*	*Lactobacillales*	*Enterococcaceae*	*Enterococcus*	*Enterococcus*
1909	*Firmicutes*	*Bacilli*	*Lactobacillales*	*Enterococcaceae*	*Enterococcus*	*Enterococcus*
1910	*Firmicutes*	*Bacilli*	*Lactobacillales*	*Enterococcaceae*	*Enterococcus*	*Enterococcus*
1911	*Firmicutes*	*Bacilli*	*Lactobacillales*	*Enterococcaceae*	*Enterococcus*	*Enterococcus*
1912	*Firmicutes*	*Bacilli*	*Lactobacillales*	*Enterococcaceae*	*Enterococcus*	*Enterococcus*
1913	*Firmicutes*	*Bacilli*	*Lactobacillales*	*Enterococcaceae*	*Enterococcus*	*Enterococcus*
1914	*Firmicutes*	*Bacilli*	*Lactobacillales*	*Enterococcaceae*	*Enterococcus*	*Enterococcus*
1915	*Firmicutes*	*Bacilli*	*Lactobacillales*	*Enterococcaceae*	*Enterococcus*	*Enterococcus*
1916	*Firmicutes*	*Bacilli*	*Lactobacillales*	*Enterococcaceae*	*Enterococcus*	*Enterococcus*
1917	*Firmicutes*	*Bacilli*	*Lactobacillales*	*Enterococcaceae*	*Enterococcus*	*Enterococcus*
1918	*Firmicutes*	*Bacilli*	*Lactobacillales*	*Enterococcaceae*	*Enterococcus*	*Enterococcus*
1919	*Firmicutes*	*Bacilli*	*Lactobacillales*	*Enterococcaceae*	*Enterococcus*	*Enterococcus*
1920	*Firmicutes*	*Bacilli*	*Lactobacillales*	*Enterococcaceae*	*Enterococcus*	*Enterococcus*
1921	*Firmicutes*	*Bacilli*	*Lactobacillales*	*Enterococcaceae*	*Enterococcus*	*Enterococcus*
1922	*Firmicutes*	*Bacilli*	*Lactobacillales*	*Enterococcaceae*	*Enterococcus*	*Enterococcus*
1923	*Firmicutes*	*Bacilli*	*Lactobacillales*	*Enterococcaceae*	*Enterococcus*	*Enterococcus*
1924	*Firmicutes*	*Bacilli*	*Lactobacillales*	*Enterococcaceae*	*Enterococcus*	*Enterococcus*
1925	*Firmicutes*	*Bacilli*	*Lactobacillales*	*Enterococcaceae*	*Enterococcus*	*Enterococcus*
1926	*Firmicutes*	*Bacilli*	*Lactobacillales*	*Enterococcaceae*	*Enterococcus*	*Enterococcus*
1927	*Firmicutes*	*Bacilli*	*Lactobacillales*	*Enterococcaceae*	*Enterococcus*	*Enterococcus*
1928	*Firmicutes*	*Bacilli*	*Lactobacillales*	*Enterococcaceae*	*Enterococcus*	*Enterococcus*
1929	*Firmicutes*	*Bacilli*	*Lactobacillales*	*Enterococcaceae*	*Enterococcus*	*Enterococcus*
1930	*Firmicutes*	*Bacilli*	*Lactobacillales*	*Enterococcaceae*	*Enterococcus*	*Enterococcus*
1931	*Firmicutes*	*Bacilli*	*Lactobacillales*	*Enterococcaceae*	*Enterococcus*	*Enterococcus*
1932	*Firmicutes*	*Bacilli*	*Lactobacillales*	*Enterococcaceae*	*Enterococcus*	*Enterococcus*

Table S4. Isolated strains by culturomics

bacteriaId	*phylum*	*class*	*order*	*family*	*genus*	*species*
1933	*Firmicutes*	*Bacilli*	*Lactobacillales*	*Enterococcaceae*	*Enterococcus*	*Enterococcus*
1934	*Firmicutes*	*Bacilli*	*Lactobacillales*	*Enterococcaceae*	*Enterococcus*	*Enterococcus*
1935	*Firmicutes*	*Bacilli*	*Lactobacillales*	*Enterococcaceae*	*Enterococcus*	*Enterococcus*
1936	*Firmicutes*	*Bacilli*	*Lactobacillales*	*Enterococcaceae*	*Enterococcus*	*Enterococcus*
1937	*Firmicutes*	*Bacilli*	*Lactobacillales*	*Enterococcaceae*	*Enterococcus*	*Enterococcus*
1938	*Firmicutes*	*Bacilli*	*Lactobacillales*	*Enterococcaceae*	*Enterococcus*	*Enterococcus*
1939	*Firmicutes*	*Bacilli*	*Lactobacillales*	*Enterococcaceae*	*Enterococcus*	*Enterococcus*
1940	*Firmicutes*	*Bacilli*	*Lactobacillales*	*Enterococcaceae*	*Enterococcus*	*Enterococcus*
1941	*Firmicutes*	*Bacilli*	*Lactobacillales*	*Enterococcaceae*	*Enterococcus*	*Enterococcus*
1942	*Firmicutes*	*Bacilli*	*Lactobacillales*	*Enterococcaceae*	*Enterococcus*	*Enterococcus*
1943	*Firmicutes*	*Bacilli*	*Lactobacillales*	*Enterococcaceae*	*Enterococcus*	*Enterococcus*
1944	*Firmicutes*	*Bacilli*	*Lactobacillales*	*Enterococcaceae*	*Enterococcus*	*Enterococcus*
1945	*Firmicutes*	*Bacilli*	*Lactobacillales*	*Enterococcaceae*	*Enterococcus*	*Enterococcus*
1946	*Firmicutes*	*Bacilli*	*Lactobacillales*	*Enterococcaceae*	*Enterococcus*	*Enterococcus*
1947	*Firmicutes*	*Bacilli*	*Lactobacillales*	*Enterococcaceae*	*Enterococcus*	*Enterococcus*
1948	*Firmicutes*	*Bacilli*	*Lactobacillales*	*Enterococcaceae*	*Enterococcus*	*Enterococcus*
1949	*Firmicutes*	*Bacilli*	*Lactobacillales*	*Enterococcaceae*	*Enterococcus*	*Enterococcus*
1950	*Firmicutes*	*Bacilli*	*Lactobacillales*	*Enterococcaceae*	*Enterococcus*	*Enterococcus*
1951	*Firmicutes*	*Bacilli*	*Lactobacillales*	*Enterococcaceae*	*Enterococcus*	*Enterococcus*
1952	*Firmicutes*	*Bacilli*	*Lactobacillales*	*Enterococcaceae*	*Enterococcus*	*Enterococcus*
1953	*Firmicutes*	*Bacilli*	*Lactobacillales*	*Enterococcaceae*	*Enterococcus*	*Enterococcus*
1954	*Firmicutes*	*Bacilli*	*Lactobacillales*	*Enterococcaceae*	*Enterococcus*	*Enterococcus*
1955	*Firmicutes*	*Bacilli*	*Lactobacillales*	*Enterococcaceae*	*Enterococcus*	*Enterococcus*
1956	*Firmicutes*	*Bacilli*	*Lactobacillales*	*Enterococcaceae*	*Enterococcus*	*Enterococcus*
1957	*Firmicutes*	*Bacilli*	*Lactobacillales*	*Enterococcaceae*	*Enterococcus*	*Enterococcus*
1958	*Firmicutes*	*Bacilli*	*Lactobacillales*	*Enterococcaceae*	*Enterococcus*	*Enterococcus*
1959	*Firmicutes*	*Bacilli*	*Lactobacillales*	*Enterococcaceae*	*Enterococcus*	*Enterococcus*
1960	*Firmicutes*	*Bacilli*	*Lactobacillales*	*Enterococcaceae*	*Enterococcus*	*Enterococcus*

Table S4. Isolated strains by culturomics

bacteriaId	*phylum*	*class*	*order*	*family*	*genus*	*species*
1961	*Firmicutes*	*Bacilli*	*Lactobacillales*	*Enterococcaceae*	*Enterococcus*	*Enterococcus*
1962	*Firmicutes*	*Bacilli*	*Lactobacillales*	*Enterococcaceae*	*Enterococcus*	*Enterococcus*
1963	*Firmicutes*	*Bacilli*	*Lactobacillales*	*Enterococcaceae*	*Enterococcus*	*Enterococcus*
1964	*Firmicutes*	*Bacilli*	*Lactobacillales*	*Enterococcaceae*	*Enterococcus*	*Enterococcus*
1965	*Firmicutes*	*Bacilli*	*Lactobacillales*	*Enterococcaceae*	*Enterococcus*	*Enterococcus*
1966	*Firmicutes*	*Bacilli*	*Lactobacillales*	*Enterococcaceae*	*Enterococcus*	*Enterococcus*
1967	*Firmicutes*	*Bacilli*	*Lactobacillales*	*Enterococcaceae*	*Enterococcus*	*Enterococcus*
1968	*Firmicutes*	*Bacilli*	*Lactobacillales*	*Enterococcaceae*	*Enterococcus*	*Enterococcus*
1969	*Firmicutes*	*Bacilli*	*Lactobacillales*	*Enterococcaceae*	*Enterococcus*	*Enterococcus*
1970	*Firmicutes*	*Bacilli*	*Lactobacillales*	*Enterococcaceae*	*Enterococcus*	*Enterococcus*
1971	*Firmicutes*	*Bacilli*	*Lactobacillales*	*Enterococcaceae*	*Enterococcus*	*Enterococcus*
1972	*Firmicutes*	*Bacilli*	*Lactobacillales*	*Enterococcaceae*	*Enterococcus*	*Enterococcus*
1973	*Firmicutes*	*Bacilli*	*Lactobacillales*	*Enterococcaceae*	*Enterococcus*	*Enterococcus*
1974	*Firmicutes*	*Bacilli*	*Lactobacillales*	*Enterococcaceae*	*Enterococcus*	*Enterococcus*
1975	*Firmicutes*	*Bacilli*	*Lactobacillales*	*Enterococcaceae*	*Enterococcus*	*Enterococcus*
1976	*Firmicutes*	*Bacilli*	*Lactobacillales*	*Enterococcaceae*	*Enterococcus*	*Enterococcus*
1977	*Firmicutes*	*Bacilli*	*Lactobacillales*	*Enterococcaceae*	*Enterococcus*	*Enterococcus*
1978	*Firmicutes*	*Bacilli*	*Lactobacillales*	*Enterococcaceae*	*Enterococcus*	*Enterococcus*
1979	*Firmicutes*	*Bacilli*	*Lactobacillales*	*Enterococcaceae*	*Enterococcus*	*Enterococcus*
1980	*Firmicutes*	*Bacilli*	*Lactobacillales*	*Enterococcaceae*	*Enterococcus*	*Enterococcus*
1981	*Firmicutes*	*Bacilli*	*Lactobacillales*	*Enterococcaceae*	*Enterococcus*	*Enterococcus*
1982	*Firmicutes*	*Bacilli*	*Lactobacillales*	*Enterococcaceae*	*Enterococcus*	*Enterococcus*
1983	*Firmicutes*	*Bacilli*	*Lactobacillales*	*Enterococcaceae*	*Enterococcus*	*Enterococcus*
1984	*Firmicutes*	*Bacilli*	*Lactobacillales*	*Enterococcaceae*	*Enterococcus*	*Enterococcus*
1985	*Firmicutes*	*Bacilli*	*Lactobacillales*	*Enterococcaceae*	*Enterococcus*	*Enterococcus*
1986	*Firmicutes*	*Bacilli*	*Lactobacillales*	*Enterococcaceae*	*Enterococcus*	*Enterococcus*
1987	*Firmicutes*	*Bacilli*	*Lactobacillales*	*Enterococcaceae*	*Enterococcus*	*Enterococcus*
1988	*Firmicutes*	*Bacilli*	*Lactobacillales*	*Enterococcaceae*	*Enterococcus*	*Enterococcus*

Table S4. Isolated strains by culturomics

bacteriaId	*phylum*	*class*	*order*	*family*	*genus*	*species*
1989	*Firmicutes*	*Bacilli*	*Lactobacillales*	*Enterococcaceae*	*Enterococcus*	*Enterococcus*
1990	*Firmicutes*	*Bacilli*	*Lactobacillales*	*Enterococcaceae*	*Enterococcus*	*Enterococcus*
1991	*Firmicutes*	*Bacilli*	*Lactobacillales*	*Enterococcaceae*	*Enterococcus*	*Enterococcus*
1992	*Firmicutes*	*Bacilli*	*Lactobacillales*	*Enterococcaceae*	*Enterococcus*	*Enterococcus*
1993	*Firmicutes*	*Bacilli*	*Lactobacillales*	*Enterococcaceae*	*Enterococcus*	*Enterococcus*
1994	*Firmicutes*	*Bacilli*	*Lactobacillales*	*Enterococcaceae*	*Enterococcus*	*Enterococcus*
1995	*Firmicutes*	*Bacilli*	*Lactobacillales*	*Enterococcaceae*	*Enterococcus*	*Enterococcus*
1996	*Firmicutes*	*Bacilli*	*Lactobacillales*	*Enterococcaceae*	*Enterococcus*	*Enterococcus*
1997	*Firmicutes*	*Bacilli*	*Lactobacillales*	*Enterococcaceae*	*Enterococcus*	*Enterococcus*
1998	*Firmicutes*	*Bacilli*	*Lactobacillales*	*Enterococcaceae*	*Enterococcus*	*Enterococcus*
1999	*Firmicutes*	*Bacilli*	*Lactobacillales*	*Enterococcaceae*	*Enterococcus*	*Enterococcus*
2000	*Firmicutes*	*Bacilli*	*Lactobacillales*	*Enterococcaceae*	*Enterococcus*	*Enterococcus*
2001	*Firmicutes*	*Bacilli*	*Lactobacillales*	*Enterococcaceae*	*Enterococcus*	*Enterococcus*
2002	*Firmicutes*	*Bacilli*	*Lactobacillales*	*Enterococcaceae*	*Enterococcus*	*Enterococcus*
2003	*Firmicutes*	*Bacilli*	*Lactobacillales*	*Enterococcaceae*	*Enterococcus*	*Enterococcus*
2004	*Firmicutes*	*Bacilli*	*Lactobacillales*	*Enterococcaceae*	*Enterococcus*	*Enterococcus*
2005	*Firmicutes*	*Bacilli*	*Lactobacillales*	*Enterococcaceae*	*Enterococcus*	*Enterococcus*
2006	*Firmicutes*	*Bacilli*	*Lactobacillales*	*Enterococcaceae*	*Enterococcus*	*Enterococcus*
2007	*Firmicutes*	*Bacilli*	*Lactobacillales*	*Enterococcaceae*	*Enterococcus*	*Enterococcus*
2008	*Firmicutes*	*Bacilli*	*Lactobacillales*	*Enterococcaceae*	*Enterococcus*	*Enterococcus*
2009	*Firmicutes*	*Bacilli*	*Lactobacillales*	*Enterococcaceae*	*Enterococcus*	*Enterococcus*
2010	*Firmicutes*	*Bacilli*	*Lactobacillales*	*Enterococcaceae*	*Enterococcus*	*Enterococcus*
2011	*Firmicutes*	*Bacilli*	*Lactobacillales*	*Enterococcaceae*	*Enterococcus*	*Enterococcus*
2012	*Firmicutes*	*Bacilli*	*Lactobacillales*	*Enterococcaceae*	*Enterococcus*	*Enterococcus*
2013	*Firmicutes*	*Bacilli*	*Lactobacillales*	*Enterococcaceae*	*Enterococcus*	*Enterococcus*
2014	*Firmicutes*	*Bacilli*	*Lactobacillales*	*Enterococcaceae*	*Enterococcus*	*Enterococcus*
2015	*Firmicutes*	*Bacilli*	*Lactobacillales*	*Enterococcaceae*	*Enterococcus*	*Enterococcus*
2016	*Firmicutes*	*Bacilli*	*Lactobacillales*	*Enterococcaceae*	*Enterococcus*	*Enterococcus*

Table S4. Isolated strains by culturomics

bacteriaId	*phylum*	*class*	*order*	*family*	*genus*	*species*
2017	*Firmicutes*	*Bacilli*	*Lactobacillales*	*Enterococcaceae*	*Enterococcus*	*Enterococcus*
2018	*Firmicutes*	*Bacilli*	*Lactobacillales*	*Enterococcaceae*	*Enterococcus*	*Enterococcus*
2019	*Firmicutes*	*Bacilli*	*Lactobacillales*	*Enterococcaceae*	*Enterococcus*	*Enterococcus*
2020	*Firmicutes*	*Bacilli*	*Lactobacillales*	*Enterococcaceae*	*Enterococcus*	*Enterococcus*
2021	*Firmicutes*	*Bacilli*	*Lactobacillales*	*Enterococcaceae*	*Enterococcus*	*Enterococcus*
2022	*Firmicutes*	*Bacilli*	*Lactobacillales*	*Enterococcaceae*	*Enterococcus*	*Enterococcus*
2023	*Firmicutes*	*Bacilli*	*Lactobacillales*	*Enterococcaceae*	*Enterococcus*	*Enterococcus*
2024	*Firmicutes*	*Bacilli*	*Lactobacillales*	*Enterococcaceae*	*Enterococcus*	*Enterococcus*
2025	*Firmicutes*	*Bacilli*	*Lactobacillales*	*Enterococcaceae*	*Enterococcus*	*Enterococcus*
2026	*Firmicutes*	*Bacilli*	*Lactobacillales*	*Enterococcaceae*	*Enterococcus*	*Enterococcus*
2027	*Firmicutes*	*Bacilli*	*Lactobacillales*	*Enterococcaceae*	*Enterococcus*	*Enterococcus*
2028	*Firmicutes*	*Bacilli*	*Lactobacillales*	*Enterococcaceae*	*Enterococcus*	*Enterococcus*
2029	*Firmicutes*	*Bacilli*	*Lactobacillales*	*Enterococcaceae*	*Enterococcus*	*Enterococcus*
2030	*Firmicutes*	*Bacilli*	*Lactobacillales*	*Enterococcaceae*	*Enterococcus*	*Enterococcus*
2031	*Firmicutes*	*Bacilli*	*Lactobacillales*	*Enterococcaceae*	*Enterococcus*	*Enterococcus*
2032	*Firmicutes*	*Bacilli*	*Lactobacillales*	*Enterococcaceae*	*Enterococcus*	*Enterococcus*
2033	*Firmicutes*	*Bacilli*	*Lactobacillales*	*Enterococcaceae*	*Enterococcus*	*Enterococcus*
2034	*Firmicutes*	*Bacilli*	*Lactobacillales*	*Enterococcaceae*	*Enterococcus*	*Enterococcus*
2035	*Firmicutes*	*Bacilli*	*Lactobacillales*	*Enterococcaceae*	*Enterococcus*	*Enterococcus*
2036	*Firmicutes*	*Bacilli*	*Lactobacillales*	*Enterococcaceae*	*Enterococcus*	*Enterococcus*
2037	*Firmicutes*	*Bacilli*	*Lactobacillales*	*Enterococcaceae*	*Enterococcus*	*Enterococcus*
2038	*Firmicutes*	*Bacilli*	*Lactobacillales*	*Enterococcaceae*	*Enterococcus*	*Enterococcus*
2039	*Firmicutes*	*Bacilli*	*Lactobacillales*	*Enterococcaceae*	*Enterococcus*	*Enterococcus*
2040	*Firmicutes*	*Bacilli*	*Lactobacillales*	*Enterococcaceae*	*Enterococcus*	*Enterococcus*
2041	*Firmicutes*	*Bacilli*	*Lactobacillales*	*Enterococcaceae*	*Enterococcus*	*Enterococcus*
2042	*Firmicutes*	*Bacilli*	*Lactobacillales*	*Enterococcaceae*	*Enterococcus*	*Enterococcus*
2043	*Firmicutes*	*Bacilli*	*Lactobacillales*	*Enterococcaceae*	*Enterococcus*	*Enterococcus*
2044	*Firmicutes*	*Bacilli*	*Lactobacillales*	*Enterococcaceae*	*Enterococcus*	*Enterococcus*

Table S4. Isolated strains by culturomics

bacteriaId	*phylum*	*class*	*order*	*family*	*genus*	*species*
2045	*Firmicutes*	*Bacilli*	*Lactobacillales*	*Enterococcaceae*	*Enterococcus*	*Enterococcus*
2046	*Firmicutes*	*Bacilli*	*Lactobacillales*	*Enterococcaceae*	*Enterococcus*	*Enterococcus*
2047	*Firmicutes*	*Bacilli*	*Lactobacillales*	*Enterococcaceae*	*Enterococcus*	*Enterococcus*
2048	*Firmicutes*	*Bacilli*	*Lactobacillales*	*Enterococcaceae*	*Enterococcus*	*Enterococcus*
2049	*Firmicutes*	*Bacilli*	*Lactobacillales*	*Enterococcaceae*	*Enterococcus*	*Enterococcus*
2050	*Firmicutes*	*Bacilli*	*Lactobacillales*	*Enterococcaceae*	*Enterococcus*	*Enterococcus*
2051	*Firmicutes*	*Bacilli*	*Lactobacillales*	*Enterococcaceae*	*Enterococcus*	*Enterococcus*
2052	*Firmicutes*	*Bacilli*	*Lactobacillales*	*Enterococcaceae*	*Enterococcus*	*Enterococcus*
2053	*Firmicutes*	*Bacilli*	*Lactobacillales*	*Enterococcaceae*	*Enterococcus*	*Enterococcus*
2054	*Firmicutes*	*Bacilli*	*Lactobacillales*	*Enterococcaceae*	*Enterococcus*	*Enterococcus*
2055	*Firmicutes*	*Bacilli*	*Lactobacillales*	*Enterococcaceae*	*Enterococcus*	*Enterococcus*
2056	*Firmicutes*	*Bacilli*	*Lactobacillales*	*Enterococcaceae*	*Enterococcus*	*Enterococcus*
2057	*Firmicutes*	*Bacilli*	*Lactobacillales*	*Enterococcaceae*	*Enterococcus*	*Enterococcus*
2058	*Firmicutes*	*Bacilli*	*Lactobacillales*	*Enterococcaceae*	*Enterococcus*	*Enterococcus*
2059	*Firmicutes*	*Bacilli*	*Lactobacillales*	*Enterococcaceae*	*Enterococcus*	*Enterococcus*
2060	*Firmicutes*	*Bacilli*	*Lactobacillales*	*Enterococcaceae*	*Enterococcus*	*Enterococcus*
2061	*Firmicutes*	*Bacilli*	*Lactobacillales*	*Enterococcaceae*	*Enterococcus*	*Enterococcus*
2062	*Firmicutes*	*Bacilli*	*Lactobacillales*	*Enterococcaceae*	*Enterococcus*	*Enterococcus*
2063	*Firmicutes*	*Bacilli*	*Lactobacillales*	*Enterococcaceae*	*Enterococcus*	*Enterococcus*
2064	*Firmicutes*	*Bacilli*	*Lactobacillales*	*Enterococcaceae*	*Enterococcus*	*Enterococcus*
2065	*Firmicutes*	*Bacilli*	*Lactobacillales*	*Enterococcaceae*	*Enterococcus*	*Enterococcus*
2066	*Firmicutes*	*Bacilli*	*Lactobacillales*	*Enterococcaceae*	*Enterococcus*	*Enterococcus*
2067	*Firmicutes*	*Bacilli*	*Lactobacillales*	*Enterococcaceae*	*Enterococcus*	*Enterococcus*
2068	*Firmicutes*	*Bacilli*	*Lactobacillales*	*Enterococcaceae*	*Enterococcus*	*Enterococcus*
2069	*Firmicutes*	*Bacilli*	*Lactobacillales*	*Enterococcaceae*	*Enterococcus*	*Enterococcus*
2070	*Firmicutes*	*Bacilli*	*Lactobacillales*	*Enterococcaceae*	*Enterococcus*	*Enterococcus*
2071	*Firmicutes*	*Bacilli*	*Lactobacillales*	*Enterococcaceae*	*Enterococcus*	*Enterococcus*
2072	*Firmicutes*	*Bacilli*	*Lactobacillales*	*Enterococcaceae*	*Enterococcus*	*Enterococcus*

Table S4. Isolated strains by culturomics

bacteriaId	*phylum*	*class*	*order*	*family*	*genus*	*species*
2073	*Firmicutes*	*Bacilli*	*Lactobacillales*	*Enterococcaceae*	*Enterococcus*	*Enterococcus*
2074	*Firmicutes*	*Bacilli*	*Lactobacillales*	*Enterococcaceae*	*Enterococcus*	*Enterococcus*
2075	*Firmicutes*	*Bacilli*	*Lactobacillales*	*Enterococcaceae*	*Enterococcus*	*Enterococcus*
2076	*Firmicutes*	*Bacilli*	*Lactobacillales*	*Enterococcaceae*	*Enterococcus*	*Enterococcus*
2077	*Firmicutes*	*Bacilli*	*Lactobacillales*	*Enterococcaceae*	*Enterococcus*	*Enterococcus*
2078	*Firmicutes*	*Bacilli*	*Lactobacillales*	*Enterococcaceae*	*Enterococcus*	*Enterococcus*
2079	*Firmicutes*	*Bacilli*	*Lactobacillales*	*Enterococcaceae*	*Enterococcus*	*Enterococcus*
2080	*Firmicutes*	*Bacilli*	*Lactobacillales*	*Enterococcaceae*	*Enterococcus*	*Enterococcus*
2081	*Firmicutes*	*Bacilli*	*Lactobacillales*	*Enterococcaceae*	*Enterococcus*	*Enterococcus*
2082	*Firmicutes*	*Bacilli*	*Lactobacillales*	*Enterococcaceae*	*Enterococcus*	*Enterococcus*
2083	*Firmicutes*	*Bacilli*	*Lactobacillales*	*Enterococcaceae*	*Enterococcus*	*Enterococcus*
2084	*Firmicutes*	*Bacilli*	*Lactobacillales*	*Enterococcaceae*	*Enterococcus*	*Enterococcus*
2085	*Firmicutes*	*Bacilli*	*Lactobacillales*	*Enterococcaceae*	*Enterococcus*	*Enterococcus*
2086	*Firmicutes*	*Bacilli*	*Lactobacillales*	*Enterococcaceae*	*Enterococcus*	*Enterococcus*
2087	*Firmicutes*	*Bacilli*	*Lactobacillales*	*Enterococcaceae*	*Enterococcus*	*Enterococcus*
2088	*Firmicutes*	*Bacilli*	*Lactobacillales*	*Enterococcaceae*	*Enterococcus*	*Enterococcus*
2089	*Firmicutes*	*Bacilli*	*Lactobacillales*	*Enterococcaceae*	*Enterococcus*	*Enterococcus*
2090	*Firmicutes*	*Bacilli*	*Lactobacillales*	*Enterococcaceae*	*Enterococcus*	*Enterococcus*
2091	*Firmicutes*	*Bacilli*	*Lactobacillales*	*Enterococcaceae*	*Enterococcus*	*Enterococcus*
2092	*Firmicutes*	*Bacilli*	*Lactobacillales*	*Enterococcaceae*	*Enterococcus*	*Enterococcus*
2093	*Firmicutes*	*Bacilli*	*Lactobacillales*	*Enterococcaceae*	*Enterococcus*	*Enterococcus*
2094	*Firmicutes*	*Bacilli*	*Lactobacillales*	*Enterococcaceae*	*Enterococcus*	*Enterococcus*
2095	*Firmicutes*	*Bacilli*	*Lactobacillales*	*Enterococcaceae*	*Enterococcus*	*Enterococcus*
2096	*Firmicutes*	*Bacilli*	*Lactobacillales*	*Enterococcaceae*	*Enterococcus*	*Enterococcus*
2097	*Firmicutes*	*Bacilli*	*Lactobacillales*	*Enterococcaceae*	*Enterococcus*	*Enterococcus*
2098	*Firmicutes*	*Bacilli*	*Lactobacillales*	*Enterococcaceae*	*Enterococcus*	*Enterococcus*
2099	*Firmicutes*	*Bacilli*	*Lactobacillales*	*Enterococcaceae*	*Enterococcus*	*Enterococcus*
2100	*Firmicutes*	*Bacilli*	*Lactobacillales*	*Enterococcaceae*	*Enterococcus*	*Enterococcus*

Table S4. Isolated strains by culturomics

bacteriaId	*phylum*	*class*	*order*	*family*	*genus*	*species*
2101	*Firmicutes*	*Bacilli*	*Lactobacillales*	*Enterococcaceae*	*Enterococcus*	*Enterococcus*
2102	*Firmicutes*	*Bacilli*	*Lactobacillales*	*Enterococcaceae*	*Enterococcus*	*Enterococcus*
2103	*Firmicutes*	*Bacilli*	*Lactobacillales*	*Enterococcaceae*	*Enterococcus*	*Enterococcus*
2104	*Firmicutes*	*Bacilli*	*Lactobacillales*	*Enterococcaceae*	*Enterococcus*	*Enterococcus*
2105	*Firmicutes*	*Bacilli*	*Lactobacillales*	*Enterococcaceae*	*Enterococcus*	*Enterococcus*
2106	*Firmicutes*	*Bacilli*	*Lactobacillales*	*Enterococcaceae*	*Enterococcus*	*Enterococcus*
2107	*Firmicutes*	*Bacilli*	*Lactobacillales*	*Enterococcaceae*	*Enterococcus*	*Enterococcus*
2108	*Firmicutes*	*Bacilli*	*Lactobacillales*	*Enterococcaceae*	*Enterococcus*	*Enterococcus*
2109	*Firmicutes*	*Bacilli*	*Lactobacillales*	*Enterococcaceae*	*Enterococcus*	*Enterococcus*
2110	*Firmicutes*	*Bacilli*	*Lactobacillales*	*Enterococcaceae*	*Enterococcus*	*Enterococcus*
2111	*Firmicutes*	*Bacilli*	*Lactobacillales*	*Enterococcaceae*	*Enterococcus*	*Enterococcus*
2112	*Firmicutes*	*Bacilli*	*Lactobacillales*	*Enterococcaceae*	*Enterococcus*	*Enterococcus*
2113	*Firmicutes*	*Bacilli*	*Lactobacillales*	*Enterococcaceae*	*Enterococcus*	*Enterococcus*
2114	*Firmicutes*	*Bacilli*	*Lactobacillales*	*Enterococcaceae*	*Enterococcus*	*Enterococcus*
2115	*Firmicutes*	*Bacilli*	*Lactobacillales*	*Enterococcaceae*	*Enterococcus*	*Enterococcus*
2116	*Firmicutes*	*Bacilli*	*Lactobacillales*	*Enterococcaceae*	*Enterococcus*	*Enterococcus*
2117	*Firmicutes*	*Bacilli*	*Lactobacillales*	*Enterococcaceae*	*Enterococcus*	*Enterococcus*
2118	*Firmicutes*	*Bacilli*	*Lactobacillales*	*Enterococcaceae*	*Enterococcus*	*Enterococcus*
2119	*Firmicutes*	*Bacilli*	*Lactobacillales*	*Enterococcaceae*	*Enterococcus*	*Enterococcus*
2120	*Firmicutes*	*Bacilli*	*Lactobacillales*	*Enterococcaceae*	*Enterococcus*	*Enterococcus*
2121	*Firmicutes*	*Bacilli*	*Lactobacillales*	*Enterococcaceae*	*Enterococcus*	*Enterococcus*
2122	*Firmicutes*	*Bacilli*	*Lactobacillales*	*Enterococcaceae*	*Enterococcus*	*Enterococcus*
2123	*Firmicutes*	*Bacilli*	*Lactobacillales*	*Enterococcaceae*	*Enterococcus*	*Enterococcus*
2124	*Firmicutes*	*Bacilli*	*Lactobacillales*	*Enterococcaceae*	*Enterococcus*	*Enterococcus*
2125	*Firmicutes*	*Bacilli*	*Lactobacillales*	*Enterococcaceae*	*Enterococcus*	*Enterococcus*
2126	*Firmicutes*	*Bacilli*	*Lactobacillales*	*Enterococcaceae*	*Enterococcus*	*Enterococcus*
2127	*Firmicutes*	*Bacilli*	*Lactobacillales*	*Enterococcaceae*	*Enterococcus*	*Enterococcus*
2128	*Firmicutes*	*Bacilli*	*Lactobacillales*	*Enterococcaceae*	*Enterococcus*	*Enterococcus*

Table S4. Isolated strains by culturomics

bacteriaId	*phylum*	*class*	*order*	*family*	*genus*	*species*
2129	*Firmicutes*	*Bacilli*	*Lactobacillales*	*Enterococcaceae*	*Enterococcus*	*Enterococcus*
2130	*Firmicutes*	*Bacilli*	*Lactobacillales*	*Enterococcaceae*	*Enterococcus*	*Enterococcus*
2131	*Firmicutes*	*Bacilli*	*Lactobacillales*	*Enterococcaceae*	*Enterococcus*	*Enterococcus*
2132	*Firmicutes*	*Bacilli*	*Lactobacillales*	*Enterococcaceae*	*Enterococcus*	*Enterococcus*
2133	*Firmicutes*	*Bacilli*	*Lactobacillales*	*Enterococcaceae*	*Enterococcus*	*Enterococcus*
2134	*Firmicutes*	*Bacilli*	*Lactobacillales*	*Enterococcaceae*	*Enterococcus*	*Enterococcus*
2135	*Firmicutes*	*Bacilli*	*Lactobacillales*	*Enterococcaceae*	*Enterococcus*	*Enterococcus*
2136	*Firmicutes*	*Bacilli*	*Lactobacillales*	*Enterococcaceae*	*Enterococcus*	*Enterococcus*
2137	*Firmicutes*	*Bacilli*	*Lactobacillales*	*Enterococcaceae*	*Enterococcus*	*Enterococcus*
2138	*Firmicutes*	*Bacilli*	*Lactobacillales*	*Enterococcaceae*	*Enterococcus*	*Enterococcus*
2139	*Firmicutes*	*Bacilli*	*Lactobacillales*	*Enterococcaceae*	*Enterococcus*	*Enterococcus*
2140	*Firmicutes*	*Bacilli*	*Lactobacillales*	*Enterococcaceae*	*Enterococcus*	*Enterococcus*
2141	*Firmicutes*	*Bacilli*	*Lactobacillales*	*Enterococcaceae*	*Enterococcus*	*Enterococcus*
2142	*Firmicutes*	*Bacilli*	*Lactobacillales*	*Enterococcaceae*	*Enterococcus*	*Enterococcus*
2143	*Firmicutes*	*Bacilli*	*Lactobacillales*	*Enterococcaceae*	*Enterococcus*	*Enterococcus*
2144	*Firmicutes*	*Bacilli*	*Lactobacillales*	*Enterococcaceae*	*Enterococcus*	*Enterococcus*
2145	*Firmicutes*	*Bacilli*	*Lactobacillales*	*Enterococcaceae*	*Enterococcus*	*Enterococcus*
2146	*Firmicutes*	*Bacilli*	*Lactobacillales*	*Enterococcaceae*	*Enterococcus*	*Enterococcus*
2147	*Firmicutes*	*Bacilli*	*Lactobacillales*	*Enterococcaceae*	*Enterococcus*	*Enterococcus*
2148	*Firmicutes*	*Bacilli*	*Lactobacillales*	*Enterococcaceae*	*Enterococcus*	*Enterococcus*
2149	*Firmicutes*	*Bacilli*	*Lactobacillales*	*Enterococcaceae*	*Enterococcus*	*Enterococcus*
2150	*Firmicutes*	*Bacilli*	*Lactobacillales*	*Enterococcaceae*	*Enterococcus*	*Enterococcus*
2151	*Firmicutes*	*Bacilli*	*Lactobacillales*	*Enterococcaceae*	*Enterococcus*	*Enterococcus*
2152	*Firmicutes*	*Bacilli*	*Lactobacillales*	*Enterococcaceae*	*Enterococcus*	*Enterococcus*
2153	*Firmicutes*	*Bacilli*	*Lactobacillales*	*Enterococcaceae*	*Enterococcus*	*Enterococcus*
2154	*Firmicutes*	*Bacilli*	*Lactobacillales*	*Enterococcaceae*	*Enterococcus*	*Enterococcus*
2155	*Firmicutes*	*Bacilli*	*Lactobacillales*	*Enterococcaceae*	*Enterococcus*	*Enterococcus*
2156	*Firmicutes*	*Bacilli*	*Lactobacillales*	*Enterococcaceae*	*Enterococcus*	*Enterococcus*

Table S4. Isolated strains by culturomics

bacteriald	*phylum*	*class*	*order*	*family*	*genus*	*species*
2157	*Firmicutes*	*Bacilli*	*Lactobacillales*	*Enterococcaceae*	*Enterococcus*	*Enterococcus*
2158	*Firmicutes*	*Bacilli*	*Lactobacillales*	*Enterococcaceae*	*Enterococcus*	*Enterococcus*
2159	*Firmicutes*	*Bacilli*	*Lactobacillales*	*Enterococcaceae*	*Enterococcus*	*Enterococcus*
2160	*Firmicutes*	*Bacilli*	*Lactobacillales*	*Enterococcaceae*	*Enterococcus*	*Enterococcus*
2161	*Firmicutes*	*Bacilli*	*Lactobacillales*	*Enterococcaceae*	*Enterococcus*	*Enterococcus*
2162	*Firmicutes*	*Bacilli*	*Lactobacillales*	*Enterococcaceae*	*Enterococcus*	*Enterococcus*
2163	*Firmicutes*	*Bacilli*	*Lactobacillales*	*Enterococcaceae*	*Enterococcus*	*Enterococcus*
2164	*Firmicutes*	*Bacilli*	*Lactobacillales*	*Enterococcaceae*	*Enterococcus*	*Enterococcus*
2165	*Firmicutes*	*Bacilli*	*Lactobacillales*	*Enterococcaceae*	*Enterococcus*	*Enterococcus*
2166	*Firmicutes*	*Bacilli*	*Lactobacillales*	*Enterococcaceae*	*Enterococcus*	*Enterococcus*
2167	*Firmicutes*	*Bacilli*	*Lactobacillales*	*Enterococcaceae*	*Enterococcus*	*Enterococcus*
2168	*Firmicutes*	*Bacilli*	*Lactobacillales*	*Enterococcaceae*	*Enterococcus*	*Enterococcus*
2169	*Firmicutes*	*Bacilli*	*Lactobacillales*	*Enterococcaceae*	*Enterococcus*	*Enterococcus*
2170	*Firmicutes*	*Bacilli*	*Lactobacillales*	*Enterococcaceae*	*Enterococcus*	*Enterococcus*
2171	*Firmicutes*	*Bacilli*	*Lactobacillales*	*Enterococcaceae*	*Enterococcus*	*Enterococcus*
2172	*Firmicutes*	*Bacilli*	*Lactobacillales*	*Enterococcaceae*	*Enterococcus*	*Enterococcus*
2173	*Firmicutes*	*Bacilli*	*Lactobacillales*	*Enterococcaceae*	*Enterococcus*	*Enterococcus*
2174	*Firmicutes*	*Bacilli*	*Lactobacillales*	*Enterococcaceae*	*Enterococcus*	*Enterococcus*
2175	*Firmicutes*	*Bacilli*	*Lactobacillales*	*Enterococcaceae*	*Enterococcus*	*Enterococcus*
2176	*Firmicutes*	*Bacilli*	*Lactobacillales*	*Enterococcaceae*	*Enterococcus*	*Enterococcus*
2177	*Firmicutes*	*Bacilli*	*Lactobacillales*	*Enterococcaceae*	*Enterococcus*	*Enterococcus*
2178	*Firmicutes*	*Bacilli*	*Lactobacillales*	*Enterococcaceae*	*Enterococcus*	*Enterococcus*
2179	*Firmicutes*	*Bacilli*	*Lactobacillales*	*Enterococcaceae*	*Enterococcus*	*Enterococcus*
2180	*Firmicutes*	*Bacilli*	*Lactobacillales*	*Enterococcaceae*	*Enterococcus*	*Enterococcus*
2181	*Firmicutes*	*Bacilli*	*Lactobacillales*	*Enterococcaceae*	*Enterococcus*	*Enterococcus*
2182	*Firmicutes*	*Bacilli*	*Lactobacillales*	*Enterococcaceae*	*Enterococcus*	*Enterococcus*
2183	*Firmicutes*	*Bacilli*	*Lactobacillales*	*Enterococcaceae*	*Enterococcus*	*Enterococcus*
2184	*Firmicutes*	*Bacilli*	*Lactobacillales*	*Enterococcaceae*	*Enterococcus*	*Enterococcus*

Table S4. Isolated strains by culturomics

bacteriald	*phylum*	*class*	*order*	*family*	*genus*	*species*
2185	*Firmicutes*	*Bacilli*	*Lactobacillales*	*Enterococcaceae*	*Enterococcus*	*Enterococcus*
2186	*Firmicutes*	*Bacilli*	*Lactobacillales*	*Enterococcaceae*	*Enterococcus*	*Enterococcus*
2187	*Firmicutes*	*Bacilli*	*Lactobacillales*	*Enterococcaceae*	*Enterococcus*	*Enterococcus*
2188	*Firmicutes*	*Bacilli*	*Lactobacillales*	*Enterococcaceae*	*Enterococcus*	*Enterococcus*
2189	*Firmicutes*	*Bacilli*	*Lactobacillales*	*Enterococcaceae*	*Enterococcus*	*Enterococcus*
2190	*Firmicutes*	*Bacilli*	*Lactobacillales*	*Enterococcaceae*	*Enterococcus*	*Enterococcus*
2191	*Firmicutes*	*Bacilli*	*Lactobacillales*	*Enterococcaceae*	*Enterococcus*	*Enterococcus*
2192	*Firmicutes*	*Bacilli*	*Lactobacillales*	*Enterococcaceae*	*Enterococcus*	*Enterococcus*
2193	*Firmicutes*	*Bacilli*	*Lactobacillales*	*Enterococcaceae*	*Enterococcus*	*Enterococcus*
2194	*Firmicutes*	*Bacilli*	*Lactobacillales*	*Enterococcaceae*	*Enterococcus*	*Enterococcus*
2195	*Firmicutes*	*Bacilli*	*Lactobacillales*	*Enterococcaceae*	*Enterococcus*	*Enterococcus*
2196	*Firmicutes*	*Bacilli*	*Lactobacillales*	*Enterococcaceae*	*Enterococcus*	*Enterococcus*
2197	*Firmicutes*	*Bacilli*	*Lactobacillales*	*Enterococcaceae*	*Enterococcus*	*Enterococcus*
2198	*Firmicutes*	*Bacilli*	*Lactobacillales*	*Enterococcaceae*	*Enterococcus*	*Enterococcus*
2199	*Firmicutes*	*Bacilli*	*Lactobacillales*	*Enterococcaceae*	*Enterococcus*	*Enterococcus*
2200	*Firmicutes*	*Bacilli*	*Lactobacillales*	*Enterococcaceae*	*Enterococcus*	*Enterococcus*
2201	*Firmicutes*	*Bacilli*	*Lactobacillales*	*Enterococcaceae*	*Enterococcus*	*Enterococcus*
2202	*Firmicutes*	*Bacilli*	*Lactobacillales*	*Enterococcaceae*	*Enterococcus*	*Enterococcus*
2203	*Firmicutes*	*Bacilli*	*Lactobacillales*	*Enterococcaceae*	*Enterococcus*	*Enterococcus*
2204	*Firmicutes*	*Bacilli*	*Lactobacillales*	*Enterococcaceae*	*Enterococcus*	*Enterococcus*
2205	*Firmicutes*	*Bacilli*	*Lactobacillales*	*Enterococcaceae*	*Enterococcus*	*Enterococcus*
2206	*Firmicutes*	*Bacilli*	*Lactobacillales*	*Enterococcaceae*	*Enterococcus*	*Enterococcus*
2207	*Firmicutes*	*Bacilli*	*Lactobacillales*	*Enterococcaceae*	*Enterococcus*	*Enterococcus*
2208	*Firmicutes*	*Bacilli*	*Lactobacillales*	*Enterococcaceae*	*Enterococcus*	*Enterococcus*
2209	*Firmicutes*	*Bacilli*	*Lactobacillales*	*Enterococcaceae*	*Enterococcus*	*Enterococcus*
2210	*Firmicutes*	*Bacilli*	*Lactobacillales*	*Enterococcaceae*	*Enterococcus*	*Enterococcus*
2211	*Firmicutes*	*Bacilli*	*Lactobacillales*	*Enterococcaceae*	*Enterococcus*	*Enterococcus*
2212	*Firmicutes*	*Bacilli*	*Lactobacillales*	*Enterococcaceae*	*Enterococcus*	*Enterococcus*

Table S4. Isolated strains by culturomics

bacteriaId	*phylum*	*class*	*order*	*family*	*genus*	*species*
2213	*Firmicutes*	*Bacilli*	*Lactobacillales*	*Enterococcaceae*	*Enterococcus*	*Enterococcus*
2214	*Firmicutes*	*Bacilli*	*Lactobacillales*	*Enterococcaceae*	*Enterococcus*	*Enterococcus*
2215	*Firmicutes*	*Bacilli*	*Lactobacillales*	*Enterococcaceae*	*Enterococcus*	*Enterococcus*
2216	*Firmicutes*	*Bacilli*	*Lactobacillales*	*Enterococcaceae*	*Enterococcus*	*Enterococcus*
2217	*Firmicutes*	*Bacilli*	*Lactobacillales*	*Enterococcaceae*	*Enterococcus*	*Enterococcus*
2218	*Firmicutes*	*Bacilli*	*Lactobacillales*	*Enterococcaceae*	*Enterococcus*	*Enterococcus*
2219	*Firmicutes*	*Bacilli*	*Lactobacillales*	*Enterococcaceae*	*Enterococcus*	*Enterococcus*
2220	*Firmicutes*	*Bacilli*	*Lactobacillales*	*Enterococcaceae*	*Enterococcus*	*Enterococcus*
2221	*Firmicutes*	*Bacilli*	*Lactobacillales*	*Enterococcaceae*	*Enterococcus*	*Enterococcus*
2222	*Firmicutes*	*Bacilli*	*Lactobacillales*	*Enterococcaceae*	*Enterococcus*	*Enterococcus*
2223	*Firmicutes*	*Bacilli*	*Lactobacillales*	*Enterococcaceae*	*Enterococcus*	*Enterococcus*
2224	*Firmicutes*	*Bacilli*	*Lactobacillales*	*Enterococcaceae*	*Enterococcus*	*Enterococcus*
2225	*Firmicutes*	*Bacilli*	*Lactobacillales*	*Enterococcaceae*	*Enterococcus*	*Enterococcus*
2226	*Firmicutes*	*Bacilli*	*Lactobacillales*	*Enterococcaceae*	*Enterococcus*	*Enterococcus*
2227	*Firmicutes*	*Bacilli*	*Lactobacillales*	*Enterococcaceae*	*Enterococcus*	*Enterococcus*
2228	*Firmicutes*	*Bacilli*	*Lactobacillales*	*Enterococcaceae*	*Enterococcus*	*Enterococcus*
2229	*Firmicutes*	*Bacilli*	*Lactobacillales*	*Enterococcaceae*	*Enterococcus*	*Enterococcus*
2230	*Firmicutes*	*Bacilli*	*Lactobacillales*	*Enterococcaceae*	*Enterococcus*	*Enterococcus*
2231	*Firmicutes*	*Bacilli*	*Lactobacillales*	*Enterococcaceae*	*Enterococcus*	*Enterococcus*
2232	*Firmicutes*	*Bacilli*	*Lactobacillales*	*Enterococcaceae*	*Enterococcus*	*Enterococcus*
2233	*Firmicutes*	*Bacilli*	*Lactobacillales*	*Enterococcaceae*	*Enterococcus*	*Enterococcus*
2234	*Firmicutes*	*Bacilli*	*Lactobacillales*	*Enterococcaceae*	*Enterococcus*	*Enterococcus*
2235	*Firmicutes*	*Bacilli*	*Lactobacillales*	*Enterococcaceae*	*Enterococcus*	*Enterococcus*
2236	*Firmicutes*	*Bacilli*	*Lactobacillales*	*Enterococcaceae*	*Enterococcus*	*Enterococcus*
2237	*Firmicutes*	*Bacilli*	*Lactobacillales*	*Enterococcaceae*	*Enterococcus*	*Enterococcus*
2238	*Firmicutes*	*Bacilli*	*Lactobacillales*	*Enterococcaceae*	*Enterococcus*	*Enterococcus*
2239	*Firmicutes*	*Bacilli*	*Lactobacillales*	*Enterococcaceae*	*Enterococcus*	*Enterococcus*
2240	*Firmicutes*	*Bacilli*	*Lactobacillales*	*Enterococcaceae*	*Enterococcus*	*Enterococcus*

Table S4. Isolated strains by culturomics

bacteriaId	*phylum*	*class*	*order*	*family*	*genus*	*species*
2241	*Firmicutes*	*Bacilli*	*Lactobacillales*	*Enterococcaceae*	*Enterococcus*	*Enterococcus*
2242	*Firmicutes*	*Bacilli*	*Lactobacillales*	*Enterococcaceae*	*Enterococcus*	*Enterococcus*
2243	*Firmicutes*	*Bacilli*	*Lactobacillales*	*Enterococcaceae*	*Enterococcus*	*Enterococcus*
2244	*Firmicutes*	*Bacilli*	*Lactobacillales*	*Enterococcaceae*	*Enterococcus*	*Enterococcus*
2245	*Firmicutes*	*Bacilli*	*Lactobacillales*	*Enterococcaceae*	*Enterococcus*	*Enterococcus*
2246	*Firmicutes*	*Bacilli*	*Lactobacillales*	*Enterococcaceae*	*Enterococcus*	*Enterococcus*
2247	*Firmicutes*	*Bacilli*	*Lactobacillales*	*Enterococcaceae*	*Enterococcus*	*Enterococcus*
2248	*Firmicutes*	*Bacilli*	*Lactobacillales*	*Enterococcaceae*	*Enterococcus*	*Enterococcus*
2249	*Firmicutes*	*Bacilli*	*Lactobacillales*	*Enterococcaceae*	*Enterococcus*	*Enterococcus*
2250	*Firmicutes*	*Bacilli*	*Lactobacillales*	*Enterococcaceae*	*Enterococcus*	*Enterococcus*
2251	*Firmicutes*	*Bacilli*	*Lactobacillales*	*Enterococcaceae*	*Enterococcus*	*Enterococcus*
2252	*Firmicutes*	*Bacilli*	*Lactobacillales*	*Enterococcaceae*	*Enterococcus*	*Enterococcus*
2253	*Firmicutes*	*Bacilli*	*Lactobacillales*	*Enterococcaceae*	*Enterococcus*	*Enterococcus*
2254	*Firmicutes*	*Bacilli*	*Lactobacillales*	*Enterococcaceae*	*Enterococcus*	*Enterococcus*
2255	*Firmicutes*	*Bacilli*	*Lactobacillales*	*Enterococcaceae*	*Enterococcus*	*Enterococcus*
2256	*Firmicutes*	*Bacilli*	*Lactobacillales*	*Enterococcaceae*	*Enterococcus*	*Enterococcus*
2257	*Firmicutes*	*Bacilli*	*Lactobacillales*	*Enterococcaceae*	*Enterococcus*	*Enterococcus*
2258	*Firmicutes*	*Bacilli*	*Lactobacillales*	*Enterococcaceae*	*Enterococcus*	*Enterococcus*
2259	*Firmicutes*	*Bacilli*	*Lactobacillales*	*Enterococcaceae*	*Enterococcus*	*Enterococcus*
2260	*Firmicutes*	*Bacilli*	*Lactobacillales*	*Enterococcaceae*	*Enterococcus*	*Enterococcus*
2261	*Firmicutes*	*Bacilli*	*Lactobacillales*	*Enterococcaceae*	*Enterococcus*	*Enterococcus*
2262	*Firmicutes*	*Bacilli*	*Lactobacillales*	*Enterococcaceae*	*Enterococcus*	*Enterococcus*
2263	*Firmicutes*	*Bacilli*	*Lactobacillales*	*Enterococcaceae*	*Enterococcus*	*Enterococcus*
2264	*Firmicutes*	*Bacilli*	*Lactobacillales*	*Enterococcaceae*	*Enterococcus*	*Enterococcus*
2265	*Firmicutes*	*Bacilli*	*Lactobacillales*	*Enterococcaceae*	*Enterococcus*	*Enterococcus*
2266	*Firmicutes*	*Bacilli*	*Lactobacillales*	*Enterococcaceae*	*Enterococcus*	*Enterococcus*
2267	*Firmicutes*	*Bacilli*	*Lactobacillales*	*Enterococcaceae*	*Enterococcus*	*Enterococcus*
2268	*Firmicutes*	*Bacilli*	*Lactobacillales*	*Enterococcaceae*	*Enterococcus*	*Enterococcus*

Table S4. Isolated strains by culturomics

bacteriaId	*phylum*	*class*	*order*	*family*	*genus*	*species*
2269	*Firmicutes*	*Bacilli*	*Lactobacillales*	*Enterococcaceae*	*Enterococcus*	*Enterococcus*
2270	*Firmicutes*	*Bacilli*	*Lactobacillales*	*Enterococcaceae*	*Enterococcus*	*Enterococcus*
2271	*Firmicutes*	*Bacilli*	*Lactobacillales*	*Enterococcaceae*	*Enterococcus*	*Enterococcus*
2272	*Firmicutes*	*Bacilli*	*Lactobacillales*	*Enterococcaceae*	*Enterococcus*	*Enterococcus*
2273	*Firmicutes*	*Bacilli*	*Lactobacillales*	*Enterococcaceae*	*Enterococcus*	*Enterococcus*
2274	*Firmicutes*	*Bacilli*	*Lactobacillales*	*Enterococcaceae*	*Enterococcus*	*Enterococcus*
2275	*Firmicutes*	*Bacilli*	*Lactobacillales*	*Enterococcaceae*	*Enterococcus*	*Enterococcus*
2276	*Firmicutes*	*Bacilli*	*Lactobacillales*	*Enterococcaceae*	*Enterococcus*	*Enterococcus*
2277	*Firmicutes*	*Bacilli*	*Lactobacillales*	*Enterococcaceae*	*Enterococcus*	*Enterococcus*
2278	*Firmicutes*	*Bacilli*	*Lactobacillales*	*Enterococcaceae*	*Enterococcus*	*Enterococcus*
2279	*Firmicutes*	*Bacilli*	*Lactobacillales*	*Enterococcaceae*	*Enterococcus*	*Enterococcus*
2280	*Firmicutes*	*Bacilli*	*Lactobacillales*	*Enterococcaceae*	*Enterococcus*	*Enterococcus*
2281	*Firmicutes*	*Bacilli*	*Lactobacillales*	*Enterococcaceae*	*Enterococcus*	*Enterococcus*
2282	*Firmicutes*	*Bacilli*	*Lactobacillales*	*Enterococcaceae*	*Enterococcus*	*Enterococcus*
2283	*Firmicutes*	*Bacilli*	*Lactobacillales*	*Enterococcaceae*	*Enterococcus*	*Enterococcus*
2284	*Firmicutes*	*Bacilli*	*Lactobacillales*	*Enterococcaceae*	*Enterococcus*	*Enterococcus*
2285	*Firmicutes*	*Bacilli*	*Lactobacillales*	*Enterococcaceae*	*Enterococcus*	*Enterococcus*
2286	*Firmicutes*	*Bacilli*	*Lactobacillales*	*Enterococcaceae*	*Enterococcus*	*Enterococcus*
2287	*Firmicutes*	*Bacilli*	*Lactobacillales*	*Enterococcaceae*	*Enterococcus*	*Enterococcus*
2288	*Firmicutes*	*Bacilli*	*Lactobacillales*	*Enterococcaceae*	*Enterococcus*	*Enterococcus*
2289	*Firmicutes*	*Bacilli*	*Lactobacillales*	*Enterococcaceae*	*Enterococcus*	*Enterococcus*
2290	*Firmicutes*	*Bacilli*	*Lactobacillales*	*Enterococcaceae*	*Enterococcus*	*Enterococcus*
2291	*Firmicutes*	*Bacilli*	*Lactobacillales*	*Enterococcaceae*	*Enterococcus*	*Enterococcus*
2292	*Firmicutes*	*Bacilli*	*Lactobacillales*	*Enterococcaceae*	*Enterococcus*	*Enterococcus*
2293	*Firmicutes*	*Bacilli*	*Lactobacillales*	*Enterococcaceae*	*Enterococcus*	*Enterococcus*
2294	*Firmicutes*	*Bacilli*	*Lactobacillales*	*Enterococcaceae*	*Enterococcus*	*Enterococcus*
2295	*Firmicutes*	*Bacilli*	*Lactobacillales*	*Enterococcaceae*	*Enterococcus*	*Enterococcus*
2296	*Firmicutes*	*Bacilli*	*Lactobacillales*	*Enterococcaceae*	*Enterococcus*	*Enterococcus*

Table S4. Isolated strains by culturomics

bacteriaId	*phylum*	*class*	*order*	*family*	*genus*	*species*
2297	*Firmicutes*	*Bacilli*	*Lactobacillales*	*Enterococcaceae*	*Enterococcus*	*Enterococcus*
2298	*Firmicutes*	*Bacilli*	*Lactobacillales*	*Enterococcaceae*	*Enterococcus*	*Enterococcus*
2299	*Firmicutes*	*Bacilli*	*Lactobacillales*	*Enterococcaceae*	*Enterococcus*	*Enterococcus*
2300	*Firmicutes*	*Bacilli*	*Lactobacillales*	*Enterococcaceae*	*Enterococcus*	*Enterococcus*
2301	*Firmicutes*	*Bacilli*	*Lactobacillales*	*Enterococcaceae*	*Enterococcus*	*Enterococcus*
2302	*Firmicutes*	*Bacilli*	*Lactobacillales*	*Enterococcaceae*	*Enterococcus*	*Enterococcus*
2303	*Firmicutes*	*Bacilli*	*Lactobacillales*	*Enterococcaceae*	*Enterococcus*	*Enterococcus*
2304	*Firmicutes*	*Bacilli*	*Lactobacillales*	*Enterococcaceae*	*Enterococcus*	*Enterococcus*
2305	*Firmicutes*	*Bacilli*	*Lactobacillales*	*Enterococcaceae*	*Enterococcus*	*Enterococcus*
2306	*Firmicutes*	*Bacilli*	*Lactobacillales*	*Enterococcaceae*	*Enterococcus*	*Enterococcus*
2307	*Firmicutes*	*Bacilli*	*Lactobacillales*	*Enterococcaceae*	*Enterococcus*	*Enterococcus*
2308	*Firmicutes*	*Bacilli*	*Lactobacillales*	*Enterococcaceae*	*Enterococcus*	*Enterococcus*
2309	*Firmicutes*	*Bacilli*	*Lactobacillales*	*Enterococcaceae*	*Enterococcus*	*Enterococcus*
2310	*Firmicutes*	*Bacilli*	*Lactobacillales*	*Enterococcaceae*	*Enterococcus*	*Enterococcus*
2311	*Firmicutes*	*Bacilli*	*Lactobacillales*	*Enterococcaceae*	*Enterococcus*	*Enterococcus*
2312	*Firmicutes*	*Bacilli*	*Lactobacillales*	*Enterococcaceae*	*Enterococcus*	*Enterococcus*
2313	*Firmicutes*	*Bacilli*	*Lactobacillales*	*Enterococcaceae*	*Enterococcus*	*Enterococcus*
2314	*Firmicutes*	*Bacilli*	*Lactobacillales*	*Enterococcaceae*	*Enterococcus*	*Enterococcus*
2315	*Firmicutes*	*Bacilli*	*Lactobacillales*	*Enterococcaceae*	*Enterococcus*	*Enterococcus*
2316	*Firmicutes*	*Bacilli*	*Lactobacillales*	*Enterococcaceae*	*Enterococcus*	*Enterococcus*
2317	*Firmicutes*	*Bacilli*	*Lactobacillales*	*Enterococcaceae*	*Enterococcus*	*Enterococcus*
2318	*Firmicutes*	*Bacilli*	*Lactobacillales*	*Enterococcaceae*	*Enterococcus*	*Enterococcus*
2319	*Firmicutes*	*Bacilli*	*Lactobacillales*	*Enterococcaceae*	*Enterococcus*	*Enterococcus*
2320	*Firmicutes*	*Bacilli*	*Lactobacillales*	*Enterococcaceae*	*Enterococcus*	*Enterococcus*
2321	*Firmicutes*	*Bacilli*	*Lactobacillales*	*Enterococcaceae*	*Enterococcus*	*Enterococcus*
2322	*Firmicutes*	*Bacilli*	*Lactobacillales*	*Enterococcaceae*	*Enterococcus*	*Enterococcus*
2323	*Firmicutes*	*Bacilli*	*Lactobacillales*	*Enterococcaceae*	*Enterococcus*	*Enterococcus*
2324	*Firmicutes*	*Bacilli*	*Lactobacillales*	*Enterococcaceae*	*Enterococcus*	*Enterococcus*

Table S4. Isolated strains by culturomics

bacteriaId	*phylum*	*class*	*order*	*family*	*genus*	*species*
2325	*Firmicutes*	*Bacilli*	*Lactobacillales*	*Enterococcaceae*	*Enterococcus*	*Enterococcus*
2326	*Firmicutes*	*Bacilli*	*Lactobacillales*	*Enterococcaceae*	*Enterococcus*	*Enterococcus*
2327	*Firmicutes*	*Bacilli*	*Lactobacillales*	*Enterococcaceae*	*Enterococcus*	*Enterococcus*
2328	*Firmicutes*	*Bacilli*	*Lactobacillales*	*Enterococcaceae*	*Enterococcus*	*Enterococcus*
2329	*Firmicutes*	*Bacilli*	*Lactobacillales*	*Enterococcaceae*	*Enterococcus*	*Enterococcus*
2330	*Firmicutes*	*Bacilli*	*Lactobacillales*	*Enterococcaceae*	*Enterococcus*	*Enterococcus*
2331	*Firmicutes*	*Bacilli*	*Lactobacillales*	*Enterococcaceae*	*Enterococcus*	*Enterococcus*
2332	*Firmicutes*	*Bacilli*	*Lactobacillales*	*Enterococcaceae*	*Enterococcus*	*Enterococcus*
2333	*Firmicutes*	*Bacilli*	*Lactobacillales*	*Enterococcaceae*	*Enterococcus*	*Enterococcus*
2334	*Firmicutes*	*Bacilli*	*Lactobacillales*	*Enterococcaceae*	*Enterococcus*	*Enterococcus*
2335	*Firmicutes*	*Bacilli*	*Lactobacillales*	*Enterococcaceae*	*Enterococcus*	*Enterococcus*
2336	*Firmicutes*	*Bacilli*	*Lactobacillales*	*Enterococcaceae*	*Enterococcus*	*Enterococcus*
2337	*Firmicutes*	*Bacilli*	*Lactobacillales*	*Enterococcaceae*	*Enterococcus*	*Enterococcus*
2338	*Firmicutes*	*Bacilli*	*Lactobacillales*	*Enterococcaceae*	*Enterococcus*	*Enterococcus*
2339	*Firmicutes*	*Bacilli*	*Lactobacillales*	*Enterococcaceae*	*Enterococcus*	*Enterococcus*
2340	*Firmicutes*	*Bacilli*	*Lactobacillales*	*Enterococcaceae*	*Enterococcus*	*Enterococcus*
2341	*Firmicutes*	*Bacilli*	*Lactobacillales*	*Enterococcaceae*	*Enterococcus*	*Enterococcus*
2342	*Firmicutes*	*Bacilli*	*Lactobacillales*	*Enterococcaceae*	*Enterococcus*	*Enterococcus*
2343	*Firmicutes*	*Bacilli*	*Lactobacillales*	*Enterococcaceae*	*Enterococcus*	*Enterococcus*
2344	*Firmicutes*	*Bacilli*	*Lactobacillales*	*Enterococcaceae*	*Enterococcus*	*Enterococcus*
2345	*Firmicutes*	*Bacilli*	*Lactobacillales*	*Enterococcaceae*	*Enterococcus*	*Enterococcus*
2346	*Firmicutes*	*Bacilli*	*Lactobacillales*	*Enterococcaceae*	*Enterococcus*	*Enterococcus*
2347	*Firmicutes*	*Bacilli*	*Lactobacillales*	*Enterococcaceae*	*Enterococcus*	*Enterococcus*
2348	*Firmicutes*	*Bacilli*	*Lactobacillales*	*Enterococcaceae*	*Enterococcus*	*Enterococcus*
2349	*Firmicutes*	*Bacilli*	*Lactobacillales*	*Enterococcaceae*	*Enterococcus*	*Enterococcus*
2350	*Firmicutes*	*Bacilli*	*Lactobacillales*	*Enterococcaceae*	*Enterococcus*	*Enterococcus*
2351	*Firmicutes*	*Bacilli*	*Lactobacillales*	*Enterococcaceae*	*Enterococcus*	*Enterococcus*
2352	*Firmicutes*	*Bacilli*	*Lactobacillales*	*Enterococcaceae*	*Enterococcus*	*Enterococcus*

Table S4. Isolated strains by culturomics

bacteriaId	*phylum*	*class*	*order*	*family*	*genus*	*species*
2353	*Firmicutes*	*Bacilli*	*Lactobacillales*	*Enterococcaceae*	*Enterococcus*	*Enterococcus*
2354	*Firmicutes*	*Bacilli*	*Lactobacillales*	*Enterococcaceae*	*Enterococcus*	*Enterococcus*
2355	*Firmicutes*	*Bacilli*	*Lactobacillales*	*Enterococcaceae*	*Enterococcus*	*Enterococcus*
2356	*Firmicutes*	*Bacilli*	*Lactobacillales*	*Enterococcaceae*	*Enterococcus*	*Enterococcus*
2357	*Firmicutes*	*Bacilli*	*Lactobacillales*	*Enterococcaceae*	*Enterococcus*	*Enterococcus*
2358	*Firmicutes*	*Bacilli*	*Lactobacillales*	*Enterococcaceae*	*Enterococcus*	*Enterococcus*
2359	*Firmicutes*	*Bacilli*	*Lactobacillales*	*Enterococcaceae*	*Enterococcus*	*Enterococcus*
2360	*Firmicutes*	*Bacilli*	*Lactobacillales*	*Enterococcaceae*	*Enterococcus*	*Enterococcus*
2361	*Firmicutes*	*Bacilli*	*Lactobacillales*	*Enterococcaceae*	*Enterococcus*	*Enterococcus*
2362	*Firmicutes*	*Bacilli*	*Lactobacillales*	*Enterococcaceae*	*Enterococcus*	*Enterococcus*
2363	*Firmicutes*	*Bacilli*	*Lactobacillales*	*Enterococcaceae*	*Enterococcus*	*Enterococcus*
2364	*Firmicutes*	*Bacilli*	*Lactobacillales*	*Enterococcaceae*	*Enterococcus*	*Enterococcus*
2365	*Firmicutes*	*Bacilli*	*Lactobacillales*	*Enterococcaceae*	*Enterococcus*	*Enterococcus*
2366	*Firmicutes*	*Bacilli*	*Lactobacillales*	*Enterococcaceae*	*Enterococcus*	*Enterococcus*
2367	*Firmicutes*	*Bacilli*	*Lactobacillales*	*Enterococcaceae*	*Enterococcus*	*Enterococcus*
2368	*Firmicutes*	*Bacilli*	*Lactobacillales*	*Enterococcaceae*	*Enterococcus*	*Enterococcus*
2369	*Firmicutes*	*Bacilli*	*Lactobacillales*	*Enterococcaceae*	*Enterococcus*	*Enterococcus*
2370	*Firmicutes*	*Bacilli*	*Lactobacillales*	*Enterococcaceae*	*Enterococcus*	*Enterococcus*
2371	*Firmicutes*	*Bacilli*	*Lactobacillales*	*Enterococcaceae*	*Enterococcus*	*Enterococcus*
2372	*Firmicutes*	*Bacilli*	*Lactobacillales*	*Enterococcaceae*	*Enterococcus*	*Enterococcus*
2373	*Firmicutes*	*Bacilli*	*Lactobacillales*	*Enterococcaceae*	*Enterococcus*	*Enterococcus*
2374	*Firmicutes*	*Bacilli*	*Lactobacillales*	*Enterococcaceae*	*Enterococcus*	*Enterococcus*
2375	*Firmicutes*	*Bacilli*	*Lactobacillales*	*Enterococcaceae*	*Enterococcus*	*Enterococcus*
2376	*Firmicutes*	*Bacilli*	*Lactobacillales*	*Enterococcaceae*	*Enterococcus*	*Enterococcus*
2377	*Firmicutes*	*Bacilli*	*Lactobacillales*	*Enterococcaceae*	*Enterococcus*	*Enterococcus*
2378	*Firmicutes*	*Bacilli*	*Lactobacillales*	*Enterococcaceae*	*Enterococcus*	*Enterococcus*
2379	*Firmicutes*	*Bacilli*	*Lactobacillales*	*Enterococcaceae*	*Enterococcus*	*Enterococcus*
2380	*Firmicutes*	*Bacilli*	*Lactobacillales*	*Enterococcaceae*	*Enterococcus*	*Enterococcus*

Table S4. Isolated strains by culturomics

bacteriaId	*phylum*	*class*	*order*	*family*	*genus*	*species*
2381	*Firmicutes*	*Bacilli*	*Lactobacillales*	*Enterococcaceae*	*Enterococcus*	*Enterococcus*
2382	*Firmicutes*	*Bacilli*	*Lactobacillales*	*Enterococcaceae*	*Enterococcus*	*Enterococcus*
2383	*Firmicutes*	*Bacilli*	*Lactobacillales*	*Enterococcaceae*	*Enterococcus*	*Enterococcus*
2384	*Firmicutes*	*Bacilli*	*Lactobacillales*	*Enterococcaceae*	*Enterococcus*	*Enterococcus*
2385	*Firmicutes*	*Bacilli*	*Lactobacillales*	*Enterococcaceae*	*Enterococcus*	*Enterococcus*
2386	*Firmicutes*	*Bacilli*	*Lactobacillales*	*Enterococcaceae*	*Enterococcus*	*Enterococcus*
2387	*Firmicutes*	*Bacilli*	*Lactobacillales*	*Enterococcaceae*	*Enterococcus*	*Enterococcus*
2388	*Firmicutes*	*Bacilli*	*Lactobacillales*	*Enterococcaceae*	*Enterococcus*	*Enterococcus*
2389	*Firmicutes*	*Bacilli*	*Lactobacillales*	*Enterococcaceae*	*Enterococcus*	*Enterococcus*
2390	*Firmicutes*	*Bacilli*	*Lactobacillales*	*Enterococcaceae*	*Enterococcus*	*Enterococcus*
2391	*Firmicutes*	*Bacilli*	*Lactobacillales*	*Enterococcaceae*	*Enterococcus*	*Enterococcus*
2392	*Firmicutes*	*Bacilli*	*Lactobacillales*	*Enterococcaceae*	*Enterococcus*	*Enterococcus*
2393	*Firmicutes*	*Bacilli*	*Lactobacillales*	*Enterococcaceae*	*Enterococcus*	*Enterococcus*
2394	*Firmicutes*	*Bacilli*	*Lactobacillales*	*Enterococcaceae*	*Enterococcus*	*Enterococcus*
2395	*Firmicutes*	*Bacilli*	*Lactobacillales*	*Enterococcaceae*	*Enterococcus*	*Enterococcus*
2396	*Firmicutes*	*Bacilli*	*Lactobacillales*	*Enterococcaceae*	*Enterococcus*	*Enterococcus*
2397	*Firmicutes*	*Bacilli*	*Lactobacillales*	*Enterococcaceae*	*Enterococcus*	*Enterococcus*
2398	*Firmicutes*	*Bacilli*	*Lactobacillales*	*Enterococcaceae*	*Enterococcus*	*Enterococcus*
2399	*Firmicutes*	*Bacilli*	*Lactobacillales*	*Enterococcaceae*	*Enterococcus*	*Enterococcus*
2400	*Firmicutes*	*Bacilli*	*Lactobacillales*	*Enterococcaceae*	*Enterococcus*	*Enterococcus*
2401	*Firmicutes*	*Bacilli*	*Lactobacillales*	*Enterococcaceae*	*Enterococcus*	*Enterococcus*
2402	*Firmicutes*	*Bacilli*	*Lactobacillales*	*Enterococcaceae*	*Enterococcus*	*Enterococcus*
2403	*Firmicutes*	*Bacilli*	*Lactobacillales*	*Enterococcaceae*	*Enterococcus*	*Enterococcus*
2404	*Firmicutes*	*Bacilli*	*Lactobacillales*	*Enterococcaceae*	*Enterococcus*	*Enterococcus*
2405	*Firmicutes*	*Bacilli*	*Lactobacillales*	*Enterococcaceae*	*Enterococcus*	*Enterococcus*
2406	*Firmicutes*	*Bacilli*	*Lactobacillales*	*Enterococcaceae*	*Enterococcus*	*Enterococcus gilvus*
2407	*Firmicutes*	*Bacilli*	*Lactobacillales*	*Enterococcaceae*	*Enterococcus*	*Enterococcus gilvus*
2408	*Firmicutes*	*Bacilli*	*Lactobacillales*	*Enterococcaceae*	*Enterococcus*	*Enterococcus gilvus*

Table S4. Isolated strains by culturomics

bacteriaId	*phylum*	*class*	*order*	*family*	*genus*	*species*
2409	*Firmicutes*	*Bacilli*	*Lactobacillales*	*Enterococcaceae*	*Enterococcus*	*Enterococcus hirae*
2410	*Firmicutes*	*Bacilli*	*Lactobacillales*	*Enterococcaceae*	*Enterococcus*	*Enterococcus hirae*
2411	*Firmicutes*	*Bacilli*	*Lactobacillales*	*Enterococcaceae*	*Enterococcus*	*Enterococcus hirae*
2412	*Firmicutes*	*Bacilli*	*Lactobacillales*	*Enterococcaceae*	*Enterococcus*	*Enterococcus hirae*
2413	*Firmicutes*	*Bacilli*	*Lactobacillales*	*Enterococcaceae*	*Enterococcus*	*Enterococcus hirae*
2414	*Firmicutes*	*Bacilli*	*Lactobacillales*	*Enterococcaceae*	*Enterococcus*	*Enterococcus hirae*
2415	*Firmicutes*	*Bacilli*	*Lactobacillales*	*Enterococcaceae*	*Enterococcus*	*Enterococcus hirae*
2416	*Firmicutes*	*Bacilli*	*Lactobacillales*	*Enterococcaceae*	*Enterococcus*	*Enterococcus hirae*
2417	*Firmicutes*	*Bacilli*	*Lactobacillales*	*Enterococcaceae*	*Enterococcus*	*Enterococcus hirae*
2418	*Firmicutes*	*Bacilli*	*Lactobacillales*	*Enterococcaceae*	*Enterococcus*	*Enterococcus hirae*
2419	*Firmicutes*	*Bacilli*	*Lactobacillales*	*Enterococcaceae*	*Enterococcus*	*Enterococcus hirae*
2420	*Firmicutes*	*Bacilli*	*Lactobacillales*	*Enterococcaceae*	*Enterococcus*	*Enterococcus hirae*
2421	*Firmicutes*	*Bacilli*	*Lactobacillales*	*Enterococcaceae*	*Enterococcus*	*Enterococcus hirae*
2422	*Firmicutes*	*Bacilli*	*Lactobacillales*	*Enterococcaceae*	*Enterococcus*	*Enterococcus hirae*
2423	*Firmicutes*	*Bacilli*	*Lactobacillales*	*Enterococcaceae*	*Enterococcus*	*Enterococcus hirae*
2424	*Firmicutes*	*Bacilli*	*Lactobacillales*	*Enterococcaceae*	*Enterococcus*	*Enterococcus hirae*
2425	*Firmicutes*	*Bacilli*	*Lactobacillales*	*Enterococcaceae*	*Enterococcus*	*Enterococcus hirae*
2426	*Firmicutes*	*Bacilli*	*Lactobacillales*	*Enterococcaceae*	*Enterococcus*	*Enterococcus hirae*
2427	*Firmicutes*	*Bacilli*	*Lactobacillales*	*Enterococcaceae*	*Enterococcus*	*Enterococcus hirae*
2428	*Firmicutes*	*Bacilli*	*Lactobacillales*	*Enterococcaceae*	*Enterococcus*	*Enterococcus hirae*
2429	*Firmicutes*	*Bacilli*	*Lactobacillales*	*Enterococcaceae*	*Enterococcus*	*Enterococcus hirae*
2430	*Firmicutes*	*Bacilli*	*Lactobacillales*	*Enterococcaceae*	*Enterococcus*	*Enterococcus hirae*
2431	*Firmicutes*	*Bacilli*	*Lactobacillales*	*Enterococcaceae*	*Enterococcus*	*Enterococcus hirae*
2432	*Firmicutes*	*Bacilli*	*Lactobacillales*	*Enterococcaceae*	*Enterococcus*	*Enterococcus hirae*
2433	*Firmicutes*	*Bacilli*	*Lactobacillales*	*Enterococcaceae*	*Enterococcus*	*Enterococcus hirae*
2434	*Firmicutes*	*Bacilli*	*Lactobacillales*	*Enterococcaceae*	*Enterococcus*	*Enterococcus hirae*
2435	*Firmicutes*	*Bacilli*	*Lactobacillales*	*Enterococcaceae*	*Enterococcus*	*Enterococcus hirae*
2436	*Firmicutes*	*Bacilli*	*Lactobacillales*	*Enterococcaceae*	*Enterococcus*	*Enterococcus hirae*

Table S4. Isolated strains by culturomics

bacteriaId	*phylum*	*class*	*order*	*family*	*genus*	*species*
2437	*Firmicutes*	*Bacilli*	*Lactobacillales*	*Enterococcaceae*	*Enterococcus*	*Enterococcus hirae*
2438	*Firmicutes*	*Bacilli*	*Lactobacillales*	*Enterococcaceae*	*Enterococcus*	*Enterococcus hirae*
2439	*Firmicutes*	*Bacilli*	*Lactobacillales*	*Enterococcaceae*	*Enterococcus*	*Enterococcus hirae*
2440	*Firmicutes*	*Bacilli*	*Lactobacillales*	*Enterococcaceae*	*Enterococcus*	*Enterococcus hirae*
2441	*Firmicutes*	*Bacilli*	*Lactobacillales*	*Enterococcaceae*	*Enterococcus*	*Enterococcus hirae*
2442	*Firmicutes*	*Bacilli*	*Lactobacillales*	*Enterococcaceae*	*Enterococcus*	*Enterococcus hirae*
2443	*Firmicutes*	*Bacilli*	*Lactobacillales*	*Enterococcaceae*	*Enterococcus*	*Enterococcus hirae*
2444	*Firmicutes*	*Bacilli*	*Lactobacillales*	*Enterococcaceae*	*Enterococcus*	*Enterococcus hirae*
2445	*Firmicutes*	*Bacilli*	*Lactobacillales*	*Enterococcaceae*	*Enterococcus*	*Enterococcus hirae*
2446	*Firmicutes*	*Bacilli*	*Lactobacillales*	*Enterococcaceae*	*Enterococcus*	*Enterococcus hirae*
2447	*Firmicutes*	*Bacilli*	*Lactobacillales*	*Enterococcaceae*	*Enterococcus*	*Enterococcus hirae*
2448	*Firmicutes*	*Bacilli*	*Lactobacillales*	*Enterococcaceae*	*Enterococcus*	*Enterococcus hirae*
2449	*Firmicutes*	*Bacilli*	*Lactobacillales*	*Enterococcaceae*	*Enterococcus*	*Enterococcus hirae*
2450	*Firmicutes*	*Bacilli*	*Lactobacillales*	*Enterococcaceae*	*Enterococcus*	*Enterococcus hirae*
2451	*Firmicutes*	*Bacilli*	*Lactobacillales*	*Enterococcaceae*	*Enterococcus*	*Enterococcus hirae*
2452	*Firmicutes*	*Bacilli*	*Lactobacillales*	*Enterococcaceae*	*Enterococcus*	*Enterococcus hirae*
2453	*Firmicutes*	*Bacilli*	*Lactobacillales*	*Enterococcaceae*	*Enterococcus*	*Enterococcus hirae*
2454	*Firmicutes*	*Bacilli*	*Lactobacillales*	*Enterococcaceae*	*Enterococcus*	*Enterococcus hirae*
2455	*Firmicutes*	*Bacilli*	*Lactobacillales*	*Enterococcaceae*	*Enterococcus*	*Enterococcus hirae*
2456	*Firmicutes*	*Bacilli*	*Lactobacillales*	*Enterococcaceae*	*Enterococcus*	*Enterococcus hirae*
2457	*Firmicutes*	*Bacilli*	*Lactobacillales*	*Enterococcaceae*	*Enterococcus*	*Enterococcus hirae*
2458	*Firmicutes*	*Bacilli*	*Lactobacillales*	*Enterococcaceae*	*Enterococcus*	*Enterococcus hirae*
2459	*Firmicutes*	*Bacilli*	*Lactobacillales*	*Enterococcaceae*	*Enterococcus*	*Enterococcus hirae*
2460	*Firmicutes*	*Bacilli*	*Lactobacillales*	*Enterococcaceae*	*Enterococcus*	*Enterococcus hirae*
2461	*Firmicutes*	*Bacilli*	*Lactobacillales*	*Enterococcaceae*	*Enterococcus*	*Enterococcus hirae*
2462	*Firmicutes*	*Bacilli*	*Lactobacillales*	*Enterococcaceae*	*Enterococcus*	*Enterococcus hirae*
2463	*Firmicutes*	*Bacilli*	*Lactobacillales*	*Enterococcaceae*	*Enterococcus*	*Enterococcus hirae*
2464	*Firmicutes*	*Bacilli*	*Lactobacillales*	*Enterococcaceae*	*Enterococcus*	*Enterococcus hirae*

Table S4. Isolated strains by culturomics

bacteriaId	*phylum*	*class*	*order*	*family*	*genus*	*species*
2465	*Firmicutes*	*Bacilli*	*Lactobacillales*	*Enterococcaceae*	*Enterococcus*	*Enterococcus hirae*
2466	*Firmicutes*	*Bacilli*	*Lactobacillales*	*Enterococcaceae*	*Enterococcus*	*Enterococcus hirae*
2467	*Firmicutes*	*Bacilli*	*Lactobacillales*	*Enterococcaceae*	*Enterococcus*	*Enterococcus hirae*
2468	*Firmicutes*	*Bacilli*	*Lactobacillales*	*Enterococcaceae*	*Enterococcus*	*Enterococcus hirae*
2469	*Firmicutes*	*Bacilli*	*Lactobacillales*	*Enterococcaceae*	*Enterococcus*	*Enterococcus hirae*
2470	*Firmicutes*	*Bacilli*	*Lactobacillales*	*Enterococcaceae*	*Enterococcus*	*Enterococcus hirae*
2471	*Firmicutes*	*Bacilli*	*Lactobacillales*	*Enterococcaceae*	*Enterococcus*	*Enterococcus hirae*
2472	*Firmicutes*	*Bacilli*	*Lactobacillales*	*Enterococcaceae*	*Enterococcus*	*Enterococcus hirae*
2473	*Firmicutes*	*Bacilli*	*Lactobacillales*	*Enterococcaceae*	*Enterococcus*	*Enterococcus hirae*
2474	*Firmicutes*	*Bacilli*	*Lactobacillales*	*Enterococcaceae*	*Enterococcus*	*Enterococcus hirae*
2475	*Firmicutes*	*Bacilli*	*Lactobacillales*	*Enterococcaceae*	*Enterococcus*	*Enterococcus hirae*
2476	*Firmicutes*	*Bacilli*	*Lactobacillales*	*Enterococcaceae*	*Enterococcus*	*Enterococcus hirae*
2477	*Firmicutes*	*Bacilli*	*Lactobacillales*	*Enterococcaceae*	*Enterococcus*	*Enterococcus hirae*
2478	*Firmicutes*	*Bacilli*	*Lactobacillales*	*Enterococcaceae*	*Enterococcus*	*Enterococcus hirae*
2479	*Firmicutes*	*Bacilli*	*Lactobacillales*	*Enterococcaceae*	*Enterococcus*	*Enterococcus hirae*
2480	*Firmicutes*	*Bacilli*	*Lactobacillales*	*Enterococcaceae*	*Enterococcus*	*Enterococcus hirae*
2481	*Firmicutes*	*Bacilli*	*Lactobacillales*	*Enterococcaceae*	*Enterococcus*	*Enterococcus hirae*
2482	*Firmicutes*	*Bacilli*	*Lactobacillales*	*Enterococcaceae*	*Enterococcus*	*Enterococcus hirae*
2483	*Firmicutes*	*Bacilli*	*Lactobacillales*	*Enterococcaceae*	*Enterococcus*	*Enterococcus hirae*
2484	*Firmicutes*	*Bacilli*	*Lactobacillales*	*Enterococcaceae*	*Enterococcus*	*Enterococcus hirae*
2485	*Firmicutes*	*Bacilli*	*Lactobacillales*	*Enterococcaceae*	*Enterococcus*	*Enterococcus hirae*
2486	*Firmicutes*	*Bacilli*	*Lactobacillales*	*Enterococcaceae*	*Enterococcus*	*Enterococcus hirae*
2487	*Firmicutes*	*Bacilli*	*Lactobacillales*	*Enterococcaceae*	*Enterococcus*	*Enterococcus hirae*
2488	*Firmicutes*	*Bacilli*	*Lactobacillales*	*Enterococcaceae*	*Enterococcus*	*Enterococcus hirae*
2489	*Firmicutes*	*Bacilli*	*Lactobacillales*	*Enterococcaceae*	*Enterococcus*	*Enterococcus hirae*
2490	*Firmicutes*	*Bacilli*	*Lactobacillales*	*Enterococcaceae*	*Enterococcus*	*Enterococcus hirae*
2491	*Firmicutes*	*Bacilli*	*Lactobacillales*	*Enterococcaceae*	*Enterococcus*	*Enterococcus hirae*
2492	*Firmicutes*	*Bacilli*	*Lactobacillales*	*Enterococcaceae*	*Enterococcus*	*Enterococcus hirae*

Table S4. Isolated strains by culturomics

bacteriaId	*phylum*	*class*	*order*	*family*	*genus*	*species*
2493	*Firmicutes*	*Bacilli*	*Lactobacillales*	*Enterococcaceae*	*Enterococcus*	*Enterococcus hirae*
2494	*Firmicutes*	*Bacilli*	*Lactobacillales*	*Enterococcaceae*	*Enterococcus*	*Enterococcus hirae*
2495	*Firmicutes*	*Bacilli*	*Lactobacillales*	*Enterococcaceae*	*Enterococcus*	*Enterococcus hirae*
2496	*Firmicutes*	*Bacilli*	*Lactobacillales*	*Enterococcaceae*	*Enterococcus*	*Enterococcus hirae*
2497	*Firmicutes*	*Bacilli*	*Lactobacillales*	*Enterococcaceae*	*Enterococcus*	*Enterococcus hirae*
2498	*Firmicutes*	*Bacilli*	*Lactobacillales*	*Enterococcaceae*	*Enterococcus*	*Enterococcus hirae*
2499	*Firmicutes*	*Bacilli*	*Lactobacillales*	*Enterococcaceae*	*Enterococcus*	*Enterococcus hirae*
2500	*Firmicutes*	*Bacilli*	*Lactobacillales*	*Enterococcaceae*	*Enterococcus*	*Enterococcus hirae*
2501	*Firmicutes*	*Bacilli*	*Lactobacillales*	*Enterococcaceae*	*Enterococcus*	*Enterococcus hirae*
2502	*Firmicutes*	*Bacilli*	*Lactobacillales*	*Enterococcaceae*	*Enterococcus*	*Enterococcus hirae*
2503	*Firmicutes*	*Bacilli*	*Lactobacillales*	*Enterococcaceae*	*Enterococcus*	*Enterococcus hirae*
2504	*Firmicutes*	*Bacilli*	*Lactobacillales*	*Enterococcaceae*	*Enterococcus*	*Enterococcus hirae*
2505	*Firmicutes*	*Bacilli*	*Lactobacillales*	*Enterococcaceae*	*Enterococcus*	*Enterococcus hirae*
2506	*Firmicutes*	*Bacilli*	*Lactobacillales*	*Enterococcaceae*	*Enterococcus*	*Enterococcus hirae*
2507	*Firmicutes*	*Bacilli*	*Lactobacillales*	*Enterococcaceae*	*Enterococcus*	*Enterococcus hirae*
2508	*Firmicutes*	*Bacilli*	*Lactobacillales*	*Enterococcaceae*	*Enterococcus*	*Enterococcus hirae*
2509	*Firmicutes*	*Bacilli*	*Lactobacillales*	*Enterococcaceae*	*Enterococcus*	*Enterococcus hirae*
2510	*Firmicutes*	*Bacilli*	*Lactobacillales*	*Enterococcaceae*	*Enterococcus*	*Enterococcus hirae*
2511	*Firmicutes*	*Bacilli*	*Lactobacillales*	*Enterococcaceae*	*Enterococcus*	*Enterococcus hirae*
2512	*Firmicutes*	*Bacilli*	*Lactobacillales*	*Enterococcaceae*	*Enterococcus*	*Enterococcus hirae*
2513	*Firmicutes*	*Bacilli*	*Lactobacillales*	*Enterococcaceae*	*Enterococcus*	*Enterococcus hirae*
2514	*Firmicutes*	*Bacilli*	*Lactobacillales*	*Enterococcaceae*	*Enterococcus*	*Enterococcus hirae*
2515	*Firmicutes*	*Bacilli*	*Lactobacillales*	*Enterococcaceae*	*Enterococcus*	*Enterococcus hirae*
2516	*Firmicutes*	*Bacilli*	*Lactobacillales*	*Enterococcaceae*	*Enterococcus*	*Enterococcus hirae*
2517	*Firmicutes*	*Bacilli*	*Lactobacillales*	*Enterococcaceae*	*Enterococcus*	*Enterococcus hirae*
2518	*Firmicutes*	*Bacilli*	*Lactobacillales*	*Enterococcaceae*	*Enterococcus*	*Enterococcus hirae*
2519	*Firmicutes*	*Bacilli*	*Lactobacillales*	*Enterococcaceae*	*Enterococcus*	*Enterococcus hirae*
2520	*Firmicutes*	*Bacilli*	*Lactobacillales*	*Enterococcaceae*	*Enterococcus*	*Enterococcus hirae*

Table S4. Isolated strains by culturomics

bacteriaId	*phylum*	*class*	*order*	*family*	*genus*	*species*
2521	*Firmicutes*	*Bacilli*	*Lactobacillales*	*Enterococcaceae*	*Enterococcus*	*Enterococcus hirae*
2522	*Firmicutes*	*Bacilli*	*Lactobacillales*	*Enterococcaceae*	*Enterococcus*	*Enterococcus hirae*
2523	*Firmicutes*	*Bacilli*	*Lactobacillales*	*Enterococcaceae*	*Enterococcus*	*Enterococcus hirae*
2524	*Firmicutes*	*Bacilli*	*Lactobacillales*	*Enterococcaceae*	*Enterococcus*	*Enterococcus hirae*
2525	*Firmicutes*	*Bacilli*	*Lactobacillales*	*Enterococcaceae*	*Enterococcus*	*Enterococcus hirae*
2526	*Firmicutes*	*Bacilli*	*Lactobacillales*	*Enterococcaceae*	*Enterococcus*	*Enterococcus hirae*
2527	*Firmicutes*	*Bacilli*	*Lactobacillales*	*Enterococcaceae*	*Enterococcus*	*Enterococcus hirae*
2528	*Firmicutes*	*Bacilli*	*Lactobacillales*	*Enterococcaceae*	*Enterococcus*	*Enterococcus hirae*
2529	*Firmicutes*	*Bacilli*	*Lactobacillales*	*Enterococcaceae*	*Enterococcus*	*Enterococcus hirae*
2530	*Firmicutes*	*Bacilli*	*Lactobacillales*	*Enterococcaceae*	*Enterococcus*	*Enterococcus hirae*
2531	*Firmicutes*	*Bacilli*	*Lactobacillales*	*Enterococcaceae*	*Enterococcus*	*Enterococcus hirae*
2532	*Firmicutes*	*Bacilli*	*Lactobacillales*	*Enterococcaceae*	*Enterococcus*	*Enterococcus hirae*
2533	*Firmicutes*	*Bacilli*	*Lactobacillales*	*Enterococcaceae*	*Enterococcus*	*Enterococcus hirae*
2534	*Firmicutes*	*Bacilli*	*Lactobacillales*	*Enterococcaceae*	*Enterococcus*	*Enterococcus hirae*
2535	*Firmicutes*	*Bacilli*	*Lactobacillales*	*Enterococcaceae*	*Enterococcus*	*Enterococcus hirae*
2536	*Firmicutes*	*Bacilli*	*Lactobacillales*	*Enterococcaceae*	*Enterococcus*	*Enterococcus hirae*
2537	*Firmicutes*	*Bacilli*	*Lactobacillales*	*Enterococcaceae*	*Enterococcus*	*Enterococcus hirae*
2538	*Firmicutes*	*Bacilli*	*Lactobacillales*	*Enterococcaceae*	*Enterococcus*	*Enterococcus hirae*
2539	*Firmicutes*	*Bacilli*	*Lactobacillales*	*Enterococcaceae*	*Enterococcus*	*Enterococcus hirae*
2540	*Firmicutes*	*Bacilli*	*Lactobacillales*	*Enterococcaceae*	*Enterococcus*	*Enterococcus hirae*
2541	*Firmicutes*	*Bacilli*	*Lactobacillales*	*Enterococcaceae*	*Enterococcus*	*Enterococcus hirae*
2542	*Firmicutes*	*Bacilli*	*Lactobacillales*	*Enterococcaceae*	*Enterococcus*	*Enterococcus hirae*
2543	*Firmicutes*	*Bacilli*	*Lactobacillales*	*Enterococcaceae*	*Enterococcus*	*Enterococcus hirae*
2544	*Firmicutes*	*Bacilli*	*Lactobacillales*	*Enterococcaceae*	*Enterococcus*	*Enterococcus hirae*
2545	*Firmicutes*	*Bacilli*	*Lactobacillales*	*Enterococcaceae*	*Enterococcus*	*Enterococcus hirae*
2546	*Firmicutes*	*Bacilli*	*Lactobacillales*	*Enterococcaceae*	*Enterococcus*	*Enterococcus hirae*
2547	*Firmicutes*	*Bacilli*	*Lactobacillales*	*Enterococcaceae*	*Enterococcus*	*Enterococcus hirae*
2548	*Firmicutes*	*Bacilli*	*Lactobacillales*	*Enterococcaceae*	*Enterococcus*	*Enterococcus hirae*

Table S4. Isolated strains by culturomics

bacteriaId	*phylum*	*class*	*order*	*family*	*genus*	*species*
2549	*Firmicutes*	*Bacilli*	*Lactobacillales*	*Enterococcaceae*	*Enterococcus*	*Enterococcus hirae*
2550	*Firmicutes*	*Bacilli*	*Lactobacillales*	*Enterococcaceae*	*Enterococcus*	*Enterococcus hirae*
2551	*Firmicutes*	*Bacilli*	*Lactobacillales*	*Enterococcaceae*	*Enterococcus*	*Enterococcus hirae*
2552	*Firmicutes*	*Bacilli*	*Lactobacillales*	*Enterococcaceae*	*Enterococcus*	*Enterococcus hirae*
2553	*Firmicutes*	*Bacilli*	*Lactobacillales*	*Enterococcaceae*	*Enterococcus*	*Enterococcus hirae*
2554	*Firmicutes*	*Bacilli*	*Lactobacillales*	*Enterococcaceae*	*Enterococcus*	*Enterococcus hirae*
2555	*Firmicutes*	*Bacilli*	*Lactobacillales*	*Enterococcaceae*	*Enterococcus*	*Enterococcus hirae*
2556	*Firmicutes*	*Bacilli*	*Lactobacillales*	*Enterococcaceae*	*Enterococcus*	*Enterococcus hirae*
2557	*Firmicutes*	*Bacilli*	*Lactobacillales*	*Enterococcaceae*	*Enterococcus*	*Enterococcus hirae*
2558	*Firmicutes*	*Bacilli*	*Lactobacillales*	*Enterococcaceae*	*Enterococcus*	*Enterococcus hirae*
2559	*Firmicutes*	*Bacilli*	*Lactobacillales*	*Enterococcaceae*	*Enterococcus*	*Enterococcus hirae*
2560	*Firmicutes*	*Bacilli*	*Lactobacillales*	*Enterococcaceae*	*Enterococcus*	*Enterococcus hirae*
2561	*Firmicutes*	*Bacilli*	*Lactobacillales*	*Enterococcaceae*	*Enterococcus*	*Enterococcus hirae*
2562	*Firmicutes*	*Bacilli*	*Lactobacillales*	*Enterococcaceae*	*Enterococcus*	*Enterococcus hirae*
2563	*Firmicutes*	*Bacilli*	*Lactobacillales*	*Enterococcaceae*	*Enterococcus*	*Enterococcus hirae*
2564	*Firmicutes*	*Bacilli*	*Lactobacillales*	*Enterococcaceae*	*Enterococcus*	*Enterococcus hirae*
2565	*Firmicutes*	*Bacilli*	*Lactobacillales*	*Enterococcaceae*	*Enterococcus*	*Enterococcus hirae*
2566	*Firmicutes*	*Bacilli*	*Lactobacillales*	*Enterococcaceae*	*Enterococcus*	*Enterococcus hirae*
2567	*Firmicutes*	*Bacilli*	*Lactobacillales*	*Enterococcaceae*	*Enterococcus*	*Enterococcus hirae*
2568	*Firmicutes*	*Bacilli*	*Lactobacillales*	*Enterococcaceae*	*Enterococcus*	*Enterococcus hirae*
2569	*Firmicutes*	*Bacilli*	*Lactobacillales*	*Enterococcaceae*	*Enterococcus*	*Enterococcus hirae*
2570	*Firmicutes*	*Bacilli*	*Lactobacillales*	*Enterococcaceae*	*Enterococcus*	*Enterococcus hirae*
2571	*Firmicutes*	*Bacilli*	*Lactobacillales*	*Enterococcaceae*	*Enterococcus*	*Enterococcus hirae*
2572	*Firmicutes*	*Bacilli*	*Lactobacillales*	*Enterococcaceae*	*Enterococcus*	*Enterococcus hirae*
2573	*Firmicutes*	*Bacilli*	*Lactobacillales*	*Enterococcaceae*	*Enterococcus*	*Enterococcus hirae*
2574	*Firmicutes*	*Bacilli*	*Lactobacillales*	*Enterococcaceae*	*Enterococcus*	*Enterococcus hirae*
2575	*Firmicutes*	*Bacilli*	*Lactobacillales*	*Enterococcaceae*	*Enterococcus*	*Enterococcus hirae*
2576	*Firmicutes*	*Bacilli*	*Lactobacillales*	*Enterococcaceae*	*Enterococcus*	*Enterococcus hirae*

Table S4. Isolated strains by culturomics

bacteriaId	*phylum*	*class*	*order*	*family*	*genus*	*species*
2577	*Firmicutes*	*Bacilli*	*Lactobacillales*	*Enterococcaceae*	*Enterococcus*	*Enterococcus hirae*
2578	*Firmicutes*	*Bacilli*	*Lactobacillales*	*Enterococcaceae*	*Enterococcus*	*Enterococcus hirae*
2579	*Firmicutes*	*Bacilli*	*Lactobacillales*	*Enterococcaceae*	*Enterococcus*	*Enterococcus hirae*
2580	*Firmicutes*	*Bacilli*	*Lactobacillales*	*Enterococcaceae*	*Enterococcus*	*Enterococcus hirae*
2581	*Firmicutes*	*Bacilli*	*Lactobacillales*	*Enterococcaceae*	*Enterococcus*	*Enterococcus hirae*
2582	*Firmicutes*	*Bacilli*	*Lactobacillales*	*Enterococcaceae*	*Enterococcus*	*Enterococcus hirae*
2583	*Firmicutes*	*Bacilli*	*Lactobacillales*	*Enterococcaceae*	*Enterococcus*	*Enterococcus hirae*
2584	*Firmicutes*	*Bacilli*	*Lactobacillales*	*Enterococcaceae*	*Enterococcus*	*Enterococcus hirae*
2585	*Firmicutes*	*Bacilli*	*Lactobacillales*	*Enterococcaceae*	*Enterococcus*	*Enterococcus hirae*
2586	*Firmicutes*	*Bacilli*	*Lactobacillales*	*Enterococcaceae*	*Enterococcus*	*Enterococcus hirae*
2587	*Firmicutes*	*Bacilli*	*Lactobacillales*	*Enterococcaceae*	*Enterococcus*	*Enterococcus hirae*
2588	*Firmicutes*	*Bacilli*	*Lactobacillales*	*Enterococcaceae*	*Enterococcus*	*Enterococcus hirae*
2589	*Firmicutes*	*Bacilli*	*Lactobacillales*	*Enterococcaceae*	*Enterococcus*	*Enterococcus hirae*
2590	*Firmicutes*	*Bacilli*	*Lactobacillales*	*Enterococcaceae*	*Enterococcus*	*Enterococcus hirae*
2591	*Firmicutes*	*Bacilli*	*Lactobacillales*	*Enterococcaceae*	*Enterococcus*	*Enterococcus hirae*
2592	*Firmicutes*	*Bacilli*	*Lactobacillales*	*Enterococcaceae*	*Enterococcus*	*Enterococcus hirae*
2593	*Firmicutes*	*Bacilli*	*Lactobacillales*	*Enterococcaceae*	*Enterococcus*	*Enterococcus hirae*
2594	*Firmicutes*	*Bacilli*	*Lactobacillales*	*Enterococcaceae*	*Enterococcus*	*Enterococcus hirae*
2595	*Firmicutes*	*Bacilli*	*Lactobacillales*	*Enterococcaceae*	*Enterococcus*	*Enterococcus hirae*
2596	*Firmicutes*	*Bacilli*	*Lactobacillales*	*Enterococcaceae*	*Enterococcus*	*Enterococcus hirae*
2597	*Firmicutes*	*Bacilli*	*Lactobacillales*	*Enterococcaceae*	*Enterococcus*	*Enterococcus hirae*
2598	*Firmicutes*	*Bacilli*	*Lactobacillales*	*Enterococcaceae*	*Enterococcus*	*Enterococcus hirae*
2599	*Firmicutes*	*Bacilli*	*Lactobacillales*	*Enterococcaceae*	*Enterococcus*	*Enterococcus hirae*
2600	*Firmicutes*	*Bacilli*	*Lactobacillales*	*Enterococcaceae*	*Enterococcus*	*Enterococcus hirae*
2601	*Firmicutes*	*Bacilli*	*Lactobacillales*	*Enterococcaceae*	*Enterococcus*	*Enterococcus hirae*
2602	*Firmicutes*	*Bacilli*	*Lactobacillales*	*Enterococcaceae*	*Enterococcus*	*Enterococcus hirae*
2603	*Firmicutes*	*Bacilli*	*Lactobacillales*	*Enterococcaceae*	*Enterococcus*	*Enterococcus hirae*
2604	*Firmicutes*	*Bacilli*	*Lactobacillales*	*Enterococcaceae*	*Enterococcus*	*Enterococcus hirae*

Table S4. Isolated strains by culturomics

bacteriaId	*phylum*	*class*	*order*	*family*	*genus*	*species*
2605	*Firmicutes*	*Bacilli*	*Lactobacillales*	*Enterococcaceae*	*Enterococcus*	*Enterococcus hirae*
2606	*Firmicutes*	*Bacilli*	*Lactobacillales*	*Enterococcaceae*	*Enterococcus*	*Enterococcus hirae*
2607	*Firmicutes*	*Bacilli*	*Lactobacillales*	*Enterococcaceae*	*Enterococcus*	*Enterococcus hirae*
2608	*Firmicutes*	*Bacilli*	*Lactobacillales*	*Enterococcaceae*	*Enterococcus*	*Enterococcus hirae*
2609	*Firmicutes*	*Bacilli*	*Lactobacillales*	*Enterococcaceae*	*Enterococcus*	*Enterococcus hirae*
2610	*Firmicutes*	*Bacilli*	*Lactobacillales*	*Enterococcaceae*	*Enterococcus*	*Enterococcus hirae*
2611	*Firmicutes*	*Bacilli*	*Lactobacillales*	*Enterococcaceae*	*Enterococcus*	*Enterococcus hirae*
2612	*Firmicutes*	*Bacilli*	*Lactobacillales*	*Enterococcaceae*	*Enterococcus*	*Enterococcus hirae*
2613	*Firmicutes*	*Bacilli*	*Lactobacillales*	*Enterococcaceae*	*Enterococcus*	*Enterococcus hirae*
2614	*Firmicutes*	*Bacilli*	*Lactobacillales*	*Enterococcaceae*	*Enterococcus*	*Enterococcus hirae*
2615	*Firmicutes*	*Bacilli*	*Lactobacillales*	*Enterococcaceae*	*Enterococcus*	*Enterococcus hirae*
2616	*Firmicutes*	*Bacilli*	*Lactobacillales*	*Enterococcaceae*	*Enterococcus*	*Enterococcus hirae*
2617	*Firmicutes*	*Bacilli*	*Lactobacillales*	*Enterococcaceae*	*Enterococcus*	*Enterococcus hirae*
2618	*Firmicutes*	*Bacilli*	*Lactobacillales*	*Enterococcaceae*	*Enterococcus*	*Enterococcus hirae*
2619	*Firmicutes*	*Bacilli*	*Lactobacillales*	*Enterococcaceae*	*Enterococcus*	*Enterococcus hirae*
2620	*Firmicutes*	*Bacilli*	*Lactobacillales*	*Enterococcaceae*	*Enterococcus*	*Enterococcus hirae*
2621	*Firmicutes*	*Bacilli*	*Lactobacillales*	*Enterococcaceae*	*Enterococcus*	*Enterococcus*
2622	*Firmicutes*	*Bacilli*	*Lactobacillales*	*Enterococcaceae*	*Enterococcus*	*Enterococcus*
2623	*Firmicutes*	*Bacilli*	*Lactobacillales*	*Enterococcaceae*	*Enterococcus*	*Enterococcus*
2624	*Firmicutes*	*Bacilli*	*Lactobacillales*	*Enterococcaceae*	*Enterococcus*	*Enterococcus*
2625	*Firmicutes*	*Bacilli*	*Lactobacillales*	*Enterococcaceae*	*Enterococcus*	*Enterococcus*
2626	*Firmicutes*	*Bacilli*	*Lactobacillales*	*Lactobacillaceae*	*Lactobacillus*	*Lactobacillus agilis*
2627	*Firmicutes*	*Bacilli*	*Lactobacillales*	*Lactobacillaceae*	*Lactobacillus*	*Lactobacillus agilis*
2628	*Firmicutes*	*Bacilli*	*Lactobacillales*	*Lactobacillaceae*	*Lactobacillus*	*Lactobacillus agilis*
2629	*Firmicutes*	*Bacilli*	*Lactobacillales*	*Lactobacillaceae*	*Lactobacillus*	*Lactobacillus agilis*
2630	*Firmicutes*	*Bacilli*	*Lactobacillales*	*Lactobacillaceae*	*Lactobacillus*	*Lactobacillus agilis*
2631	*Firmicutes*	*Bacilli*	*Lactobacillales*	*Lactobacillaceae*	*Lactobacillus*	*Lactobacillus agilis*
2632	*Firmicutes*	*Bacilli*	*Lactobacillales*	*Lactobacillaceae*	*Lactobacillus*	*Lactobacillus agilis*

Table S4. Isolated strains by culturomics

bacteriaId	*phylum*	*class*	*order*	*family*	*genus*	*species*
2633	*Firmicutes*	*Bacilli*	*Lactobacillales*	*Lactobacillaceae*	*Lactobacillus*	*Lactobacillus agilis*
2634	*Firmicutes*	*Bacilli*	*Lactobacillales*	*Lactobacillaceae*	*Lactobacillus*	*Lactobacillus agilis*
2635	*Firmicutes*	*Bacilli*	*Lactobacillales*	*Lactobacillaceae*	*Lactobacillus*	*Lactobacillus agilis*
2636	*Firmicutes*	*Bacilli*	*Lactobacillales*	*Lactobacillaceae*	*Lactobacillus*	*Lactobacillus agilis*
2637	*Firmicutes*	*Bacilli*	*Lactobacillales*	*Lactobacillaceae*	*Lactobacillus*	*Lactobacillus agilis*
2638	*Firmicutes*	*Bacilli*	*Lactobacillales*	*Lactobacillaceae*	*Lactobacillus*	*Lactobacillus agilis*
2639	*Firmicutes*	*Bacilli*	*Lactobacillales*	*Lactobacillaceae*	*Lactobacillus*	*Lactobacillus agilis*
2640	*Firmicutes*	*Bacilli*	*Lactobacillales*	*Lactobacillaceae*	*Lactobacillus*	*Lactobacillus agilis*
2641	*Firmicutes*	*Bacilli*	*Lactobacillales*	*Lactobacillaceae*	*Lactobacillus*	*Lactobacillus agilis*
2642	*Firmicutes*	*Bacilli*	*Lactobacillales*	*Lactobacillaceae*	*Lactobacillus*	*Lactobacillus agilis*
2643	*Firmicutes*	*Bacilli*	*Lactobacillales*	*Lactobacillaceae*	*Lactobacillus*	*Lactobacillus agilis*
2644	*Firmicutes*	*Bacilli*	*Lactobacillales*	*Lactobacillaceae*	*Lactobacillus*	*Lactobacillus agilis*
2645	*Firmicutes*	*Bacilli*	*Lactobacillales*	*Lactobacillaceae*	*Lactobacillus*	*Lactobacillus agilis*
2646	*Firmicutes*	*Bacilli*	*Lactobacillales*	*Lactobacillaceae*	*Lactobacillus*	*Lactobacillus agilis*
2647	*Firmicutes*	*Bacilli*	*Lactobacillales*	*Lactobacillaceae*	*Lactobacillus*	*Lactobacillus agilis*
2648	*Firmicutes*	*Bacilli*	*Lactobacillales*	*Lactobacillaceae*	*Lactobacillus*	*Lactobacillus agilis*
2649	*Firmicutes*	*Bacilli*	*Lactobacillales*	*Lactobacillaceae*	*Lactobacillus*	*Lactobacillus agilis*
2650	*Firmicutes*	*Bacilli*	*Lactobacillales*	*Lactobacillaceae*	*Lactobacillus*	*Lactobacillus agilis*
2651	*Firmicutes*	*Bacilli*	*Lactobacillales*	*Lactobacillaceae*	*Lactobacillus*	*Lactobacillus agilis*
2652	*Firmicutes*	*Bacilli*	*Lactobacillales*	*Lactobacillaceae*	*Lactobacillus*	*Lactobacillus agilis*
2653	*Firmicutes*	*Bacilli*	*Lactobacillales*	*Lactobacillaceae*	*Lactobacillus*	*Lactobacillus agilis*
2654	*Firmicutes*	*Bacilli*	*Lactobacillales*	*Lactobacillaceae*	*Lactobacillus*	*Lactobacillus agilis*
2655	*Firmicutes*	*Bacilli*	*Lactobacillales*	*Lactobacillaceae*	*Lactobacillus*	*Lactobacillus agilis*
2656	*Firmicutes*	*Bacilli*	*Lactobacillales*	*Lactobacillaceae*	*Lactobacillus*	*Lactobacillus agilis*
2657	*Firmicutes*	*Bacilli*	*Lactobacillales*	*Lactobacillaceae*	*Lactobacillus*	*Lactobacillus agilis*
2658	*Firmicutes*	*Bacilli*	*Lactobacillales*	*Lactobacillaceae*	*Lactobacillus*	*Lactobacillus agilis*
2659	*Firmicutes*	*Bacilli*	*Lactobacillales*	*Lactobacillaceae*	*Lactobacillus*	*Lactobacillus agilis*
2660	*Firmicutes*	*Bacilli*	*Lactobacillales*	*Lactobacillaceae*	*Lactobacillus*	*Lactobacillus agilis*

Table S4. Isolated strains by culturomics

bacteriaId	*phylum*	*class*	*order*	*family*	*genus*	*species*
2661	*Firmicutes*	*Bacilli*	*Lactobacillales*	*Lactobacillaceae*	*Lactobacillus*	*Lactobacillus agilis*
2662	*Firmicutes*	*Bacilli*	*Lactobacillales*	*Lactobacillaceae*	*Lactobacillus*	*Lactobacillus agilis*
2663	*Firmicutes*	*Bacilli*	*Lactobacillales*	*Lactobacillaceae*	*Lactobacillus*	*Lactobacillus agilis*
2664	*Firmicutes*	*Bacilli*	*Lactobacillales*	*Lactobacillaceae*	*Lactobacillus*	*Lactobacillus agilis*
2665	*Firmicutes*	*Bacilli*	*Lactobacillales*	*Lactobacillaceae*	*Lactobacillus*	*Lactobacillus agilis*
2666	*Firmicutes*	*Bacilli*	*Lactobacillales*	*Lactobacillaceae*	*Lactobacillus*	*Lactobacillus agilis*
2667	*Firmicutes*	*Bacilli*	*Lactobacillales*	*Lactobacillaceae*	*Lactobacillus*	*Lactobacillus agilis*
2668	*Firmicutes*	*Bacilli*	*Lactobacillales*	*Lactobacillaceae*	*Lactobacillus*	*Lactobacillus agilis*
2669	*Firmicutes*	*Bacilli*	*Lactobacillales*	*Lactobacillaceae*	*Lactobacillus*	*Lactobacillus agilis*
2670	*Firmicutes*	*Bacilli*	*Lactobacillales*	*Lactobacillaceae*	*Lactobacillus*	*Lactobacillus agilis*
2671	*Firmicutes*	*Bacilli*	*Lactobacillales*	*Lactobacillaceae*	*Lactobacillus*	*Lactobacillus agilis*
2672	*Firmicutes*	*Bacilli*	*Lactobacillales*	*Lactobacillaceae*	*Lactobacillus*	*Lactobacillus agilis*
2673	*Firmicutes*	*Bacilli*	*Lactobacillales*	*Lactobacillaceae*	*Lactobacillus*	*Lactobacillus agilis*
2674	*Firmicutes*	*Bacilli*	*Lactobacillales*	*Lactobacillaceae*	*Lactobacillus*	*Lactobacillus agilis*
2675	*Firmicutes*	*Bacilli*	*Lactobacillales*	*Lactobacillaceae*	*Lactobacillus*	*Lactobacillus agilis*
2676	*Firmicutes*	*Bacilli*	*Lactobacillales*	*Lactobacillaceae*	*Lactobacillus*	*Lactobacillus agilis*
2677	*Firmicutes*	*Bacilli*	*Lactobacillales*	*Lactobacillaceae*	*Lactobacillus*	*Lactobacillus agilis*
2678	*Firmicutes*	*Bacilli*	*Lactobacillales*	*Lactobacillaceae*	*Lactobacillus*	*Lactobacillus agilis*
2679	*Firmicutes*	*Bacilli*	*Lactobacillales*	*Lactobacillaceae*	*Lactobacillus*	*Lactobacillus agilis*
2680	*Firmicutes*	*Bacilli*	*Lactobacillales*	*Lactobacillaceae*	*Lactobacillus*	*Lactobacillus agilis*
2681	*Firmicutes*	*Bacilli*	*Lactobacillales*	*Lactobacillaceae*	*Lactobacillus*	*Lactobacillus agilis*
2682	*Firmicutes*	*Bacilli*	*Lactobacillales*	*Lactobacillaceae*	*Lactobacillus*	*Lactobacillus agilis*
2683	*Firmicutes*	*Bacilli*	*Lactobacillales*	*Lactobacillaceae*	*Lactobacillus*	*Lactobacillus agilis*
2684	*Firmicutes*	*Bacilli*	*Lactobacillales*	*Lactobacillaceae*	*Lactobacillus*	*Lactobacillus agilis*
2685	*Firmicutes*	*Bacilli*	*Lactobacillales*	*Lactobacillaceae*	*Lactobacillus*	*Lactobacillus agilis*
2686	*Firmicutes*	*Bacilli*	*Lactobacillales*	*Lactobacillaceae*	*Lactobacillus*	*Lactobacillus agilis*
2687	*Firmicutes*	*Bacilli*	*Lactobacillales*	*Lactobacillaceae*	*Lactobacillus*	*Lactobacillus agilis*
2688	*Firmicutes*	*Bacilli*	*Lactobacillales*	*Lactobacillaceae*	*Lactobacillus*	*Lactobacillus agilis*

Table S4. Isolated strains by culturomics

bacteriaId	*phylum*	*class*	*order*	*family*	*genus*	*species*
2689	*Firmicutes*	*Bacilli*	*Lactobacillales*	*Lactobacillaceae*	*Lactobacillus*	*Lactobacillus agilis*
2690	*Firmicutes*	*Bacilli*	*Lactobacillales*	*Lactobacillaceae*	*Lactobacillus*	*Lactobacillus agilis*
2691	*Firmicutes*	*Bacilli*	*Lactobacillales*	*Lactobacillaceae*	*Lactobacillus*	*Lactobacillus agilis*
2692	*Firmicutes*	*Bacilli*	*Lactobacillales*	*Lactobacillaceae*	*Lactobacillus*	*Lactobacillus agilis*
2693	*Firmicutes*	*Bacilli*	*Lactobacillales*	*Lactobacillaceae*	*Lactobacillus*	*Lactobacillus agilis*
2694	*Firmicutes*	*Bacilli*	*Lactobacillales*	*Lactobacillaceae*	*Lactobacillus*	*Lactobacillus agilis*
2695	*Firmicutes*	*Bacilli*	*Lactobacillales*	*Lactobacillaceae*	*Lactobacillus*	*Lactobacillus agilis*
2696	*Firmicutes*	*Bacilli*	*Lactobacillales*	*Lactobacillaceae*	*Lactobacillus*	*Lactobacillus agilis*
2697	*Firmicutes*	*Bacilli*	*Lactobacillales*	*Lactobacillaceae*	*Lactobacillus*	*Lactobacillus agilis*
2698	*Firmicutes*	*Bacilli*	*Lactobacillales*	*Lactobacillaceae*	*Lactobacillus*	*Lactobacillus agilis*
2699	*Firmicutes*	*Bacilli*	*Lactobacillales*	*Lactobacillaceae*	*Lactobacillus*	*Lactobacillus agilis*
2700	*Firmicutes*	*Bacilli*	*Lactobacillales*	*Lactobacillaceae*	*Lactobacillus*	*Lactobacillus agilis*
2701	*Firmicutes*	*Bacilli*	*Lactobacillales*	*Lactobacillaceae*	*Lactobacillus*	*Lactobacillus agilis*
2702	*Firmicutes*	*Bacilli*	*Lactobacillales*	*Lactobacillaceae*	*Lactobacillus*	*Lactobacillus*
2703	*Firmicutes*	*Bacilli*	*Lactobacillales*	*Lactobacillaceae*	*Lactobacillus*	*Lactobacillus*
2704	*Firmicutes*	*Bacilli*	*Lactobacillales*	*Lactobacillaceae*	*Lactobacillus*	*Lactobacillus*
2705	*Firmicutes*	*Bacilli*	*Lactobacillales*	*Lactobacillaceae*	*Lactobacillus*	*Lactobacillus*
2706	*Firmicutes*	*Bacilli*	*Lactobacillales*	*Lactobacillaceae*	*Lactobacillus*	*Lactobacillus*
2707	*Firmicutes*	*Bacilli*	*Lactobacillales*	*Lactobacillaceae*	*Lactobacillus*	*Lactobacillus*
2708	*Firmicutes*	*Bacilli*	*Lactobacillales*	*Lactobacillaceae*	*Lactobacillus*	*Lactobacillus antri*
2709	*Firmicutes*	*Bacilli*	*Lactobacillales*	*Lactobacillaceae*	*Lactobacillus*	*Lactobacillus antri*
2710	*Firmicutes*	*Bacilli*	*Lactobacillales*	*Lactobacillaceae*	*Lactobacillus*	*Lactobacillus brevis*
2711	*Firmicutes*	*Bacilli*	*Lactobacillales*	*Lactobacillaceae*	*Lactobacillus*	*Lactobacillus brevis*
2712	*Firmicutes*	*Bacilli*	*Lactobacillales*	*Lactobacillaceae*	*Lactobacillus*	*Lactobacillus brevis*
2713	*Firmicutes*	*Bacilli*	*Lactobacillales*	*Lactobacillaceae*	*Lactobacillus*	*Lactobacillus casei*
2714	*Firmicutes*	*Bacilli*	*Lactobacillales*	*Lactobacillaceae*	*Lactobacillus*	*Lactobacillus casei*
2715	*Firmicutes*	*Bacilli*	*Lactobacillales*	*Lactobacillaceae*	*Lactobacillus*	*Lactobacillus casei*
2716	*Firmicutes*	*Bacilli*	*Lactobacillales*	*Lactobacillaceae*	*Lactobacillus*	*Lactobacillus casei*

Table S4. Isolated strains by culturomics

bacteriaId	*phylum*	*class*	*order*	*family*	*genus*	*species*
2717	*Firmicutes*	*Bacilli*	*Lactobacillales*	*Lactobacillaceae*	*Lactobacillus*	*Lactobacillus casei*
2718	*Firmicutes*	*Bacilli*	*Lactobacillales*	*Lactobacillaceae*	*Lactobacillus*	*Lactobacillus casei*
2719	*Firmicutes*	*Bacilli*	*Lactobacillales*	*Lactobacillaceae*	*Lactobacillus*	*Lactobacillus casei*
2720	*Firmicutes*	*Bacilli*	*Lactobacillales*	*Lactobacillaceae*	*Lactobacillus*	*Lactobacillus casei*
2721	*Firmicutes*	*Bacilli*	*Lactobacillales*	*Lactobacillaceae*	*Lactobacillus*	*Lactobacillus casei*
2722	*Firmicutes*	*Bacilli*	*Lactobacillales*	*Lactobacillaceae*	*Lactobacillus*	*Lactobacillus casei*
2723	*Firmicutes*	*Bacilli*	*Lactobacillales*	*Lactobacillaceae*	*Lactobacillus*	*Lactobacillus casei*
2724	*Firmicutes*	*Bacilli*	*Lactobacillales*	*Lactobacillaceae*	*Lactobacillus*	*Lactobacillus casei*
2725	*Firmicutes*	*Bacilli*	*Lactobacillales*	*Lactobacillaceae*	*Lactobacillus*	*Lactobacillus casei*
2726	*Firmicutes*	*Bacilli*	*Lactobacillales*	*Lactobacillaceae*	*Lactobacillus*	*Lactobacillus casei*
2727	*Firmicutes*	*Bacilli*	*Lactobacillales*	*Lactobacillaceae*	*Lactobacillus*	*Lactobacillus casei*
2728	*Firmicutes*	*Bacilli*	*Lactobacillales*	*Lactobacillaceae*	*Lactobacillus*	*Lactobacillus casei*
2729	*Firmicutes*	*Bacilli*	*Lactobacillales*	*Lactobacillaceae*	*Lactobacillus*	*Lactobacillus casei*
2730	*Firmicutes*	*Bacilli*	*Lactobacillales*	*Lactobacillaceae*	*Lactobacillus*	*Lactobacillus casei*
2731	*Firmicutes*	*Bacilli*	*Lactobacillales*	*Lactobacillaceae*	*Lactobacillus*	*Lactobacillus casei*
2732	*Firmicutes*	*Bacilli*	*Lactobacillales*	*Lactobacillaceae*	*Lactobacillus*	*Lactobacillus casei*
2733	*Firmicutes*	*Bacilli*	*Lactobacillales*	*Lactobacillaceae*	*Lactobacillus*	*Lactobacillus casei*
2734	*Firmicutes*	*Bacilli*	*Lactobacillales*	*Lactobacillaceae*	*Lactobacillus*	*Lactobacillus casei*
2735	*Firmicutes*	*Bacilli*	*Lactobacillales*	*Lactobacillaceae*	*Lactobacillus*	*Lactobacillus casei*
2736	*Firmicutes*	*Bacilli*	*Lactobacillales*	*Lactobacillaceae*	*Lactobacillus*	*Lactobacillus casei*
2737	*Firmicutes*	*Bacilli*	*Lactobacillales*	*Lactobacillaceae*	*Lactobacillus*	*Lactobacillus casei*
2738	*Firmicutes*	*Bacilli*	*Lactobacillales*	*Lactobacillaceae*	*Lactobacillus*	*Lactobacillus casei*
2739	*Firmicutes*	*Bacilli*	*Lactobacillales*	*Lactobacillaceae*	*Lactobacillus*	*Lactobacillus casei*
2740	*Firmicutes*	*Bacilli*	*Lactobacillales*	*Lactobacillaceae*	*Lactobacillus*	*Lactobacillus casei*
2741	*Firmicutes*	*Bacilli*	*Lactobacillales*	*Lactobacillaceae*	*Lactobacillus*	*Lactobacillus casei*
2742	*Firmicutes*	*Bacilli*	*Lactobacillales*	*Lactobacillaceae*	*Lactobacillus*	*Lactobacillus casei*
2743	*Firmicutes*	*Bacilli*	*Lactobacillales*	*Lactobacillaceae*	*Lactobacillus*	*Lactobacillus casei*
2744	*Firmicutes*	*Bacilli*	*Lactobacillales*	*Lactobacillaceae*	*Lactobacillus*	*Lactobacillus casei*

Table S4. Isolated strains by culturomics

bacteriaId	*phylum*	*class*	*order*	*family*	*genus*	*species*
2745	*Firmicutes*	*Bacilli*	*Lactobacillales*	*Lactobacillaceae*	*Lactobacillus*	*Lactobacillus casei*
2746	*Firmicutes*	*Bacilli*	*Lactobacillales*	*Lactobacillaceae*	*Lactobacillus*	*Lactobacillus casei*
2747	*Firmicutes*	*Bacilli*	*Lactobacillales*	*Lactobacillaceae*	*Lactobacillus*	*Lactobacillus casei*
2748	*Firmicutes*	*Bacilli*	*Lactobacillales*	*Lactobacillaceae*	*Lactobacillus*	*Lactobacillus casei*
2749	*Firmicutes*	*Bacilli*	*Lactobacillales*	*Lactobacillaceae*	*Lactobacillus*	*Lactobacillus casei*
2750	*Firmicutes*	*Bacilli*	*Lactobacillales*	*Lactobacillaceae*	*Lactobacillus*	*Lactobacillus casei*
2751	*Firmicutes*	*Bacilli*	*Lactobacillales*	*Lactobacillaceae*	*Lactobacillus*	*Lactobacillus casei*
2752	*Firmicutes*	*Bacilli*	*Lactobacillales*	*Lactobacillaceae*	*Lactobacillus*	*Lactobacillus casei*
2753	*Firmicutes*	*Bacilli*	*Lactobacillales*	*Lactobacillaceae*	*Lactobacillus*	*Lactobacillus casei*
2754	*Firmicutes*	*Bacilli*	*Lactobacillales*	*Lactobacillaceae*	*Lactobacillus*	*Lactobacillus casei*
2755	*Firmicutes*	*Bacilli*	*Lactobacillales*	*Lactobacillaceae*	*Lactobacillus*	*Lactobacillus casei*
2756	*Firmicutes*	*Bacilli*	*Lactobacillales*	*Lactobacillaceae*	*Lactobacillus*	*Lactobacillus casei*
2757	*Firmicutes*	*Bacilli*	*Lactobacillales*	*Lactobacillaceae*	*Lactobacillus*	*Lactobacillus casei*
2758	*Firmicutes*	*Bacilli*	*Lactobacillales*	*Lactobacillaceae*	*Lactobacillus*	*Lactobacillus casei*
2759	*Firmicutes*	*Bacilli*	*Lactobacillales*	*Lactobacillaceae*	*Lactobacillus*	*Lactobacillus casei*
2760	*Firmicutes*	*Bacilli*	*Lactobacillales*	*Lactobacillaceae*	*Lactobacillus*	*Lactobacillus casei*
2761	*Firmicutes*	*Bacilli*	*Lactobacillales*	*Lactobacillaceae*	*Lactobacillus*	*Lactobacillus casei*
2762	*Firmicutes*	*Bacilli*	*Lactobacillales*	*Lactobacillaceae*	*Lactobacillus*	*Lactobacillus casei*
2763	*Firmicutes*	*Bacilli*	*Lactobacillales*	*Lactobacillaceae*	*Lactobacillus*	*Lactobacillus casei*
2764	*Firmicutes*	*Bacilli*	*Lactobacillales*	*Lactobacillaceae*	*Lactobacillus*	*Lactobacillus casei*
2765	*Firmicutes*	*Bacilli*	*Lactobacillales*	*Lactobacillaceae*	*Lactobacillus*	*Lactobacillus casei*
2766	*Firmicutes*	*Bacilli*	*Lactobacillales*	*Lactobacillaceae*	*Lactobacillus*	*Lactobacillus casei*
2767	*Firmicutes*	*Bacilli*	*Lactobacillales*	*Lactobacillaceae*	*Lactobacillus*	*Lactobacillus casei*
2768	*Firmicutes*	*Bacilli*	*Lactobacillales*	*Lactobacillaceae*	*Lactobacillus*	*Lactobacillus casei*
2769	*Firmicutes*	*Bacilli*	*Lactobacillales*	*Lactobacillaceae*	*Lactobacillus*	*Lactobacillus casei*
2770	*Firmicutes*	*Bacilli*	*Lactobacillales*	*Lactobacillaceae*	*Lactobacillus*	*Lactobacillus casei*
2771	*Firmicutes*	*Bacilli*	*Lactobacillales*	*Lactobacillaceae*	*Lactobacillus*	*Lactobacillus*
2772	*Firmicutes*	*Bacilli*	*Lactobacillales*	*Lactobacillaceae*	*Lactobacillus*	*Lactobacillus*

Table S4. Isolated strains by culturomics

bacteriaId	*phylum*	*class*	*order*	*family*	*genus*	*species*
2773	*Firmicutes*	*Bacilli*	*Lactobacillales*	*Lactobacillaceae*	*Lactobacillus*	*Lactobacillus*
2774	*Firmicutes*	*Bacilli*	*Lactobacillales*	*Lactobacillaceae*	*Lactobacillus*	*Lactobacillus*
2775	*Firmicutes*	*Bacilli*	*Lactobacillales*	*Lactobacillaceae*	*Lactobacillus*	*Lactobacillus*
2776	*Firmicutes*	*Bacilli*	*Lactobacillales*	*Lactobacillaceae*	*Lactobacillus*	*Lactobacillus*
2777	*Firmicutes*	*Bacilli*	*Lactobacillales*	*Lactobacillaceae*	*Lactobacillus*	*Lactobacillus*
2778	*Firmicutes*	*Bacilli*	*Lactobacillales*	*Lactobacillaceae*	*Lactobacillus*	*Lactobacillus*
2779	*Firmicutes*	*Bacilli*	*Lactobacillales*	*Lactobacillaceae*	*Lactobacillus*	*Lactobacillus*
2780	*Firmicutes*	*Bacilli*	*Lactobacillales*	*Lactobacillaceae*	*Lactobacillus*	*Lactobacillus*
2781	*Firmicutes*	*Bacilli*	*Lactobacillales*	*Lactobacillaceae*	*Lactobacillus*	*Lactobacillus*
2782	*Firmicutes*	*Bacilli*	*Lactobacillales*	*Lactobacillaceae*	*Lactobacillus*	*Lactobacillus*
2783	*Firmicutes*	*Bacilli*	*Lactobacillales*	*Lactobacillaceae*	*Lactobacillus*	*Lactobacillus*
2784	*Firmicutes*	*Bacilli*	*Lactobacillales*	*Lactobacillaceae*	*Lactobacillus*	*Lactobacillus*
2785	*Firmicutes*	*Bacilli*	*Lactobacillales*	*Lactobacillaceae*	*Lactobacillus*	*Lactobacillus*
2786	*Firmicutes*	*Bacilli*	*Lactobacillales*	*Lactobacillaceae*	*Lactobacillus*	*Lactobacillus*
2787	*Firmicutes*	*Bacilli*	*Lactobacillales*	*Lactobacillaceae*	*Lactobacillus*	*Lactobacillus*
2788	*Firmicutes*	*Bacilli*	*Lactobacillales*	*Lactobacillaceae*	*Lactobacillus*	*Lactobacillus*
2789	*Firmicutes*	*Bacilli*	*Lactobacillales*	*Lactobacillaceae*	*Lactobacillus*	*Lactobacillus*
2790	*Firmicutes*	*Bacilli*	*Lactobacillales*	*Lactobacillaceae*	*Lactobacillus*	*Lactobacillus*
2791	*Firmicutes*	*Bacilli*	*Lactobacillales*	*Lactobacillaceae*	*Lactobacillus*	*Lactobacillus*
2792	*Firmicutes*	*Bacilli*	*Lactobacillales*	*Lactobacillaceae*	*Lactobacillus*	*Lactobacillus*
2793	*Firmicutes*	*Bacilli*	*Lactobacillales*	*Lactobacillaceae*	*Lactobacillus*	*Lactobacillus*
2794	*Firmicutes*	*Bacilli*	*Lactobacillales*	*Lactobacillaceae*	*Lactobacillus*	*Lactobacillus*
2795	*Firmicutes*	*Bacilli*	*Lactobacillales*	*Lactobacillaceae*	*Lactobacillus*	*Lactobacillus*
2796	*Firmicutes*	*Bacilli*	*Lactobacillales*	*Lactobacillaceae*	*Lactobacillus*	*Lactobacillus*
2797	*Firmicutes*	*Bacilli*	*Lactobacillales*	*Lactobacillaceae*	*Lactobacillus*	*Lactobacillus*
2798	*Firmicutes*	*Bacilli*	*Lactobacillales*	*Lactobacillaceae*	*Lactobacillus*	*Lactobacillus*
2799	*Firmicutes*	*Bacilli*	*Lactobacillales*	*Lactobacillaceae*	*Lactobacillus*	*Lactobacillus*
2800	*Firmicutes*	*Bacilli*	*Lactobacillales*	*Lactobacillaceae*	*Lactobacillus*	*Lactobacillus*

Table S4. Isolated strains by culturomics

bacteriaId	*phylum*	*class*	*order*	*family*	*genus*	*species*
2801	*Firmicutes*	*Bacilli*	*Lactobacillales*	*Lactobacillaceae*	*Lactobacillus*	*Lactobacillus*
2802	*Firmicutes*	*Bacilli*	*Lactobacillales*	*Lactobacillaceae*	*Lactobacillus*	*Lactobacillus*
2803	*Firmicutes*	*Bacilli*	*Lactobacillales*	*Lactobacillaceae*	*Lactobacillus*	*Lactobacillus*
2804	*Firmicutes*	*Bacilli*	*Lactobacillales*	*Lactobacillaceae*	*Lactobacillus*	*Lactobacillus*
2805	*Firmicutes*	*Bacilli*	*Lactobacillales*	*Lactobacillaceae*	*Lactobacillus*	*Lactobacillus*
2806	*Firmicutes*	*Bacilli*	*Lactobacillales*	*Lactobacillaceae*	*Lactobacillus*	*Lactobacillus*
2807	*Firmicutes*	*Bacilli*	*Lactobacillales*	*Lactobacillaceae*	*Lactobacillus*	*Lactobacillus*
2808	*Firmicutes*	*Bacilli*	*Lactobacillales*	*Lactobacillaceae*	*Lactobacillus*	*Lactobacillus*
2809	*Firmicutes*	*Bacilli*	*Lactobacillales*	*Lactobacillaceae*	*Lactobacillus*	*Lactobacillus*
2810	*Firmicutes*	*Bacilli*	*Lactobacillales*	*Lactobacillaceae*	*Lactobacillus*	*Lactobacillus*
2811	*Firmicutes*	*Bacilli*	*Lactobacillales*	*Lactobacillaceae*	*Lactobacillus*	*Lactobacillus*
2812	*Firmicutes*	*Bacilli*	*Lactobacillales*	*Lactobacillaceae*	*Lactobacillus*	*Lactobacillus*
2813	*Firmicutes*	*Bacilli*	*Lactobacillales*	*Lactobacillaceae*	*Lactobacillus*	*Lactobacillus*
2814	*Firmicutes*	*Bacilli*	*Lactobacillales*	*Lactobacillaceae*	*Lactobacillus*	*Lactobacillus*
2815	*Firmicutes*	*Bacilli*	*Lactobacillales*	*Lactobacillaceae*	*Lactobacillus*	*Lactobacillus*
2816	*Firmicutes*	*Bacilli*	*Lactobacillales*	*Lactobacillaceae*	*Lactobacillus*	*Lactobacillus*
2817	*Firmicutes*	*Bacilli*	*Lactobacillales*	*Lactobacillaceae*	*Lactobacillus*	*Lactobacillus*
2818	*Firmicutes*	*Bacilli*	*Lactobacillales*	*Lactobacillaceae*	*Lactobacillus*	*Lactobacillus*
2819	*Firmicutes*	*Bacilli*	*Lactobacillales*	*Lactobacillaceae*	*Lactobacillus*	*Lactobacillus*
2820	*Firmicutes*	*Bacilli*	*Lactobacillales*	*Lactobacillaceae*	*Lactobacillus*	*Lactobacillus*
2821	*Firmicutes*	*Bacilli*	*Lactobacillales*	*Lactobacillaceae*	*Lactobacillus*	*Lactobacillus*
2822	*Firmicutes*	*Bacilli*	*Lactobacillales*	*Lactobacillaceae*	*Lactobacillus*	*Lactobacillus*
2823	*Firmicutes*	*Bacilli*	*Lactobacillales*	*Lactobacillaceae*	*Lactobacillus*	*Lactobacillus*
2824	*Firmicutes*	*Bacilli*	*Lactobacillales*	*Lactobacillaceae*	*Lactobacillus*	*Lactobacillus*
2825	*Firmicutes*	*Bacilli*	*Lactobacillales*	*Lactobacillaceae*	*Lactobacillus*	*Lactobacillus*
2826	*Firmicutes*	*Bacilli*	*Lactobacillales*	*Lactobacillaceae*	*Lactobacillus*	*Lactobacillus*
2827	*Firmicutes*	*Bacilli*	*Lactobacillales*	*Lactobacillaceae*	*Lactobacillus*	*Lactobacillus*
2828	*Firmicutes*	*Bacilli*	*Lactobacillales*	*Lactobacillaceae*	*Lactobacillus*	*Lactobacillus*

Table S4. Isolated strains by culturomics

bacteriaId	*phylum*	*class*	*order*	*family*	*genus*	*species*
2829	*Firmicutes*	*Bacilli*	*Lactobacillales*	*Lactobacillaceae*	*Lactobacillus*	*Lactobacillus*
2830	*Firmicutes*	*Bacilli*	*Lactobacillales*	*Lactobacillaceae*	*Lactobacillus*	*Lactobacillus*
2831	*Firmicutes*	*Bacilli*	*Lactobacillales*	*Lactobacillaceae*	*Lactobacillus*	*Lactobacillus*
2832	*Firmicutes*	*Bacilli*	*Lactobacillales*	*Lactobacillaceae*	*Lactobacillus*	*Lactobacillus*
2833	*Firmicutes*	*Bacilli*	*Lactobacillales*	*Lactobacillaceae*	*Lactobacillus*	*Lactobacillus*
2834	*Firmicutes*	*Bacilli*	*Lactobacillales*	*Lactobacillaceae*	*Lactobacillus*	*Lactobacillus*
2835	*Firmicutes*	*Bacilli*	*Lactobacillales*	*Lactobacillaceae*	*Lactobacillus*	*Lactobacillus*
2836	*Firmicutes*	*Bacilli*	*Lactobacillales*	*Lactobacillaceae*	*Lactobacillus*	*Lactobacillus*
2837	*Firmicutes*	*Bacilli*	*Lactobacillales*	*Lactobacillaceae*	*Lactobacillus*	*Lactobacillus*
2838	*Firmicutes*	*Bacilli*	*Lactobacillales*	*Lactobacillaceae*	*Lactobacillus*	*Lactobacillus*
2839	*Firmicutes*	*Bacilli*	*Lactobacillales*	*Lactobacillaceae*	*Lactobacillus*	*Lactobacillus*
2840	*Firmicutes*	*Bacilli*	*Lactobacillales*	*Lactobacillaceae*	*Lactobacillus*	*Lactobacillus*
2841	*Firmicutes*	*Bacilli*	*Lactobacillales*	*Lactobacillaceae*	*Lactobacillus*	*Lactobacillus*
2842	*Firmicutes*	*Bacilli*	*Lactobacillales*	*Lactobacillaceae*	*Lactobacillus*	*Lactobacillus*
2843	*Firmicutes*	*Bacilli*	*Lactobacillales*	*Lactobacillaceae*	*Lactobacillus*	*Lactobacillus*
2844	*Firmicutes*	*Bacilli*	*Lactobacillales*	*Lactobacillaceae*	*Lactobacillus*	*Lactobacillus*
2845	*Firmicutes*	*Bacilli*	*Lactobacillales*	*Lactobacillaceae*	*Lactobacillus*	*Lactobacillus*
2846	*Firmicutes*	*Bacilli*	*Lactobacillales*	*Lactobacillaceae*	*Lactobacillus*	*Lactobacillus*
2847	*Firmicutes*	*Bacilli*	*Lactobacillales*	*Lactobacillaceae*	*Lactobacillus*	*Lactobacillus*
2848	*Firmicutes*	*Bacilli*	*Lactobacillales*	*Lactobacillaceae*	*Lactobacillus*	*Lactobacillus*
2849	*Firmicutes*	*Bacilli*	*Lactobacillales*	*Lactobacillaceae*	*Lactobacillus*	*Lactobacillus*
2850	*Firmicutes*	*Bacilli*	*Lactobacillales*	*Lactobacillaceae*	*Lactobacillus*	*Lactobacillus*
2851	*Firmicutes*	*Bacilli*	*Lactobacillales*	*Lactobacillaceae*	*Lactobacillus*	*Lactobacillus*
2852	*Firmicutes*	*Bacilli*	*Lactobacillales*	*Lactobacillaceae*	*Lactobacillus*	*Lactobacillus*
2853	*Firmicutes*	*Bacilli*	*Lactobacillales*	*Lactobacillaceae*	*Lactobacillus*	*Lactobacillus*
2854	*Firmicutes*	*Bacilli*	*Lactobacillales*	*Lactobacillaceae*	*Lactobacillus*	*Lactobacillus*
2855	*Firmicutes*	*Bacilli*	*Lactobacillales*	*Lactobacillaceae*	*Lactobacillus*	*Lactobacillus*
2856	*Firmicutes*	*Bacilli*	*Lactobacillales*	*Lactobacillaceae*	*Lactobacillus*	*Lactobacillus*

Table S4. Isolated strains by culturomics

bacteriaId	*phylum*	*class*	*order*	*family*	*genus*	*species*
2857	*Firmicutes*	*Bacilli*	*Lactobacillales*	*Lactobacillaceae*	*Lactobacillus*	*Lactobacillus*
2858	*Firmicutes*	*Bacilli*	*Lactobacillales*	*Lactobacillaceae*	*Lactobacillus*	*Lactobacillus*
2859	*Firmicutes*	*Bacilli*	*Lactobacillales*	*Lactobacillaceae*	*Lactobacillus*	*Lactobacillus*
2860	*Firmicutes*	*Bacilli*	*Lactobacillales*	*Lactobacillaceae*	*Lactobacillus*	*Lactobacillus*
2861	*Firmicutes*	*Bacilli*	*Lactobacillales*	*Lactobacillaceae*	*Lactobacillus*	*Lactobacillus*
2862	*Firmicutes*	*Bacilli*	*Lactobacillales*	*Lactobacillaceae*	*Lactobacillus*	*Lactobacillus*
2863	*Firmicutes*	*Bacilli*	*Lactobacillales*	*Lactobacillaceae*	*Lactobacillus*	*Lactobacillus*
2864	*Firmicutes*	*Bacilli*	*Lactobacillales*	*Lactobacillaceae*	*Lactobacillus*	*Lactobacillus*
2865	*Firmicutes*	*Bacilli*	*Lactobacillales*	*Lactobacillaceae*	*Lactobacillus*	*Lactobacillus*
2866	*Firmicutes*	*Bacilli*	*Lactobacillales*	*Lactobacillaceae*	*Lactobacillus*	*Lactobacillus*
2867	*Firmicutes*	*Bacilli*	*Lactobacillales*	*Lactobacillaceae*	*Lactobacillus*	*Lactobacillus*
2868	*Firmicutes*	*Bacilli*	*Lactobacillales*	*Lactobacillaceae*	*Lactobacillus*	*Lactobacillus*
2869	*Firmicutes*	*Bacilli*	*Lactobacillales*	*Lactobacillaceae*	*Lactobacillus*	*Lactobacillus*
2870	*Firmicutes*	*Bacilli*	*Lactobacillales*	*Lactobacillaceae*	*Lactobacillus*	*Lactobacillus*
2871	*Firmicutes*	*Bacilli*	*Lactobacillales*	*Lactobacillaceae*	*Lactobacillus*	*Lactobacillus*
2872	*Firmicutes*	*Bacilli*	*Lactobacillales*	*Lactobacillaceae*	*Lactobacillus*	*Lactobacillus*
2873	*Firmicutes*	*Bacilli*	*Lactobacillales*	*Lactobacillaceae*	*Lactobacillus*	*Lactobacillus*
2874	*Firmicutes*	*Bacilli*	*Lactobacillales*	*Lactobacillaceae*	*Lactobacillus*	*Lactobacillus*
2875	*Firmicutes*	*Bacilli*	*Lactobacillales*	*Lactobacillaceae*	*Lactobacillus*	*Lactobacillus*
2876	*Firmicutes*	*Bacilli*	*Lactobacillales*	*Lactobacillaceae*	*Lactobacillus*	*Lactobacillus*
2877	*Firmicutes*	*Bacilli*	*Lactobacillales*	*Lactobacillaceae*	*Lactobacillus*	*Lactobacillus*
2878	*Firmicutes*	*Bacilli*	*Lactobacillales*	*Lactobacillaceae*	*Lactobacillus*	*Lactobacillus*
2879	*Firmicutes*	*Bacilli*	*Lactobacillales*	*Lactobacillaceae*	*Lactobacillus*	*Lactobacillus*
2880	*Firmicutes*	*Bacilli*	*Lactobacillales*	*Lactobacillaceae*	*Lactobacillus*	*Lactobacillus*
2881	*Firmicutes*	*Bacilli*	*Lactobacillales*	*Lactobacillaceae*	*Lactobacillus*	*Lactobacillus*
2882	*Firmicutes*	*Bacilli*	*Lactobacillales*	*Lactobacillaceae*	*Lactobacillus*	*Lactobacillus*
2883	*Firmicutes*	*Bacilli*	*Lactobacillales*	*Lactobacillaceae*	*Lactobacillus*	*Lactobacillus*
2884	*Firmicutes*	*Bacilli*	*Lactobacillales*	*Lactobacillaceae*	*Lactobacillus*	*Lactobacillus*

Table S4. Isolated strains by culturomics

bacteriaId	*phylum*	*class*	*order*	*family*	*genus*	*species*
2885	*Firmicutes*	*Bacilli*	*Lactobacillales*	*Lactobacillaceae*	*Lactobacillus*	*Lactobacillus*
2886	*Firmicutes*	*Bacilli*	*Lactobacillales*	*Lactobacillaceae*	*Lactobacillus*	*Lactobacillus*
2887	*Firmicutes*	*Bacilli*	*Lactobacillales*	*Lactobacillaceae*	*Lactobacillus*	*Lactobacillus*
2888	*Firmicutes*	*Bacilli*	*Lactobacillales*	*Lactobacillaceae*	*Lactobacillus*	*Lactobacillus*
2889	*Firmicutes*	*Bacilli*	*Lactobacillales*	*Lactobacillaceae*	*Lactobacillus*	*Lactobacillus*
2890	*Firmicutes*	*Bacilli*	*Lactobacillales*	*Lactobacillaceae*	*Lactobacillus*	*Lactobacillus*
2891	*Firmicutes*	*Bacilli*	*Lactobacillales*	*Lactobacillaceae*	*Lactobacillus*	*Lactobacillus*
2892	*Firmicutes*	*Bacilli*	*Lactobacillales*	*Lactobacillaceae*	*Lactobacillus*	*Lactobacillus*
2893	*Firmicutes*	*Bacilli*	*Lactobacillales*	*Lactobacillaceae*	*Lactobacillus*	*Lactobacillus*
2894	*Firmicutes*	*Bacilli*	*Lactobacillales*	*Lactobacillaceae*	*Lactobacillus*	*Lactobacillus*
2895	*Firmicutes*	*Bacilli*	*Lactobacillales*	*Lactobacillaceae*	*Lactobacillus*	*Lactobacillus*
2896	*Firmicutes*	*Bacilli*	*Lactobacillales*	*Lactobacillaceae*	*Lactobacillus*	*Lactobacillus*
2897	*Firmicutes*	*Bacilli*	*Lactobacillales*	*Lactobacillaceae*	*Lactobacillus*	*Lactobacillus*
2898	*Firmicutes*	*Bacilli*	*Lactobacillales*	*Lactobacillaceae*	*Lactobacillus*	*Lactobacillus*
2899	*Firmicutes*	*Bacilli*	*Lactobacillales*	*Lactobacillaceae*	*Lactobacillus*	*Lactobacillus*
2900	*Firmicutes*	*Bacilli*	*Lactobacillales*	*Lactobacillaceae*	*Lactobacillus*	*Lactobacillus*
2901	*Firmicutes*	*Bacilli*	*Lactobacillales*	*Lactobacillaceae*	*Lactobacillus*	*Lactobacillus*
2902	*Firmicutes*	*Bacilli*	*Lactobacillales*	*Lactobacillaceae*	*Lactobacillus*	*Lactobacillus*
2903	*Firmicutes*	*Bacilli*	*Lactobacillales*	*Lactobacillaceae*	*Lactobacillus*	*Lactobacillus*
2904	*Firmicutes*	*Bacilli*	*Lactobacillales*	*Lactobacillaceae*	*Lactobacillus*	*Lactobacillus*
2905	*Firmicutes*	*Bacilli*	*Lactobacillales*	*Lactobacillaceae*	*Lactobacillus*	*Lactobacillus*
2906	*Firmicutes*	*Bacilli*	*Lactobacillales*	*Lactobacillaceae*	*Lactobacillus*	*Lactobacillus*
2907	*Firmicutes*	*Bacilli*	*Lactobacillales*	*Lactobacillaceae*	*Lactobacillus*	*Lactobacillus*
2908	*Firmicutes*	*Bacilli*	*Lactobacillales*	*Lactobacillaceae*	*Lactobacillus*	*Lactobacillus*
2909	*Firmicutes*	*Bacilli*	*Lactobacillales*	*Lactobacillaceae*	*Lactobacillus*	*Lactobacillus*
2910	*Firmicutes*	*Bacilli*	*Lactobacillales*	*Lactobacillaceae*	*Lactobacillus*	*Lactobacillus*
2911	*Firmicutes*	*Bacilli*	*Lactobacillales*	*Lactobacillaceae*	*Lactobacillus*	*Lactobacillus*
2912	*Firmicutes*	*Bacilli*	*Lactobacillales*	*Lactobacillaceae*	*Lactobacillus*	*Lactobacillus*

Table S4. Isolated strains by culturomics

bacteriaId	*phylum*	*class*	*order*	*family*	*genus*	*species*
2913	*Firmicutes*	*Bacilli*	*Lactobacillales*	*Lactobacillaceae*	*Lactobacillus*	*Lactobacillus*
2914	*Firmicutes*	*Bacilli*	*Lactobacillales*	*Lactobacillaceae*	*Lactobacillus*	*Lactobacillus*
2915	*Firmicutes*	*Bacilli*	*Lactobacillales*	*Lactobacillaceae*	*Lactobacillus*	*Lactobacillus*
2916	*Firmicutes*	*Bacilli*	*Lactobacillales*	*Lactobacillaceae*	*Lactobacillus*	*Lactobacillus*
2917	*Firmicutes*	*Bacilli*	*Lactobacillales*	*Lactobacillaceae*	*Lactobacillus*	*Lactobacillus*
2918	*Firmicutes*	*Bacilli*	*Lactobacillales*	*Lactobacillaceae*	*Lactobacillus*	*Lactobacillus*
2919	*Firmicutes*	*Bacilli*	*Lactobacillales*	*Lactobacillaceae*	*Lactobacillus*	*Lactobacillus*
2920	*Firmicutes*	*Bacilli*	*Lactobacillales*	*Lactobacillaceae*	*Lactobacillus*	*Lactobacillus*
2921	*Firmicutes*	*Bacilli*	*Lactobacillales*	*Lactobacillaceae*	*Lactobacillus*	*Lactobacillus*
2922	*Firmicutes*	*Bacilli*	*Lactobacillales*	*Lactobacillaceae*	*Lactobacillus*	*Lactobacillus*
2923	*Firmicutes*	*Bacilli*	*Lactobacillales*	*Lactobacillaceae*	*Lactobacillus*	*Lactobacillus*
2924	*Firmicutes*	*Bacilli*	*Lactobacillales*	*Lactobacillaceae*	*Lactobacillus*	*Lactobacillus*
2925	*Firmicutes*	*Bacilli*	*Lactobacillales*	*Lactobacillaceae*	*Lactobacillus*	*Lactobacillus*
2926	*Firmicutes*	*Bacilli*	*Lactobacillales*	*Lactobacillaceae*	*Lactobacillus*	*Lactobacillus*
2927	*Firmicutes*	*Bacilli*	*Lactobacillales*	*Lactobacillaceae*	*Lactobacillus*	*Lactobacillus*
2928	*Firmicutes*	*Bacilli*	*Lactobacillales*	*Lactobacillaceae*	*Lactobacillus*	*Lactobacillus*
2929	*Firmicutes*	*Bacilli*	*Lactobacillales*	*Lactobacillaceae*	*Lactobacillus*	*Lactobacillus*
2930	*Firmicutes*	*Bacilli*	*Lactobacillales*	*Lactobacillaceae*	*Lactobacillus*	*Lactobacillus*
2931	*Firmicutes*	*Bacilli*	*Lactobacillales*	*Lactobacillaceae*	*Lactobacillus*	*Lactobacillus*
2932	*Firmicutes*	*Bacilli*	*Lactobacillales*	*Lactobacillaceae*	*Lactobacillus*	*Lactobacillus*
2933	*Firmicutes*	*Bacilli*	*Lactobacillales*	*Lactobacillaceae*	*Lactobacillus*	*Lactobacillus*
2934	*Firmicutes*	*Bacilli*	*Lactobacillales*	*Lactobacillaceae*	*Lactobacillus*	*Lactobacillus*
2935	*Firmicutes*	*Bacilli*	*Lactobacillales*	*Lactobacillaceae*	*Lactobacillus*	*Lactobacillus*
2936	*Firmicutes*	*Bacilli*	*Lactobacillales*	*Lactobacillaceae*	*Lactobacillus*	*Lactobacillus*
2937	*Firmicutes*	*Bacilli*	*Lactobacillales*	*Lactobacillaceae*	*Lactobacillus*	*Lactobacillus*
2938	*Firmicutes*	*Bacilli*	*Lactobacillales*	*Lactobacillaceae*	*Lactobacillus*	*Lactobacillus*
2939	*Firmicutes*	*Bacilli*	*Lactobacillales*	*Lactobacillaceae*	*Lactobacillus*	*Lactobacillus*
2940	*Firmicutes*	*Bacilli*	*Lactobacillales*	*Lactobacillaceae*	*Lactobacillus*	*Lactobacillus*

Table S4. Isolated strains by culturomics

bacteriaId	*phylum*	*class*	*order*	*family*	*genus*	*species*
2941	*Firmicutes*	*Bacilli*	*Lactobacillales*	*Lactobacillaceae*	*Lactobacillus*	*Lactobacillus*
2942	*Firmicutes*	*Bacilli*	*Lactobacillales*	*Lactobacillaceae*	*Lactobacillus*	*Lactobacillus*
2943	*Firmicutes*	*Bacilli*	*Lactobacillales*	*Lactobacillaceae*	*Lactobacillus*	*Lactobacillus*
2944	*Firmicutes*	*Bacilli*	*Lactobacillales*	*Lactobacillaceae*	*Lactobacillus*	*Lactobacillus*
2945	*Firmicutes*	*Bacilli*	*Lactobacillales*	*Lactobacillaceae*	*Lactobacillus*	*Lactobacillus*
2946	*Firmicutes*	*Bacilli*	*Lactobacillales*	*Lactobacillaceae*	*Lactobacillus*	*Lactobacillus*
2947	*Firmicutes*	*Bacilli*	*Lactobacillales*	*Lactobacillaceae*	*Lactobacillus*	*Lactobacillus*
2948	*Firmicutes*	*Bacilli*	*Lactobacillales*	*Lactobacillaceae*	*Lactobacillus*	*Lactobacillus*
2949	*Firmicutes*	*Bacilli*	*Lactobacillales*	*Lactobacillaceae*	*Lactobacillus*	*Lactobacillus*
2950	*Firmicutes*	*Bacilli*	*Lactobacillales*	*Lactobacillaceae*	*Lactobacillus*	*Lactobacillus*
2951	*Firmicutes*	*Bacilli*	*Lactobacillales*	*Lactobacillaceae*	*Lactobacillus*	*Lactobacillus*
2952	*Firmicutes*	*Bacilli*	*Lactobacillales*	*Lactobacillaceae*	*Lactobacillus*	*Lactobacillus*
2953	*Firmicutes*	*Bacilli*	*Lactobacillales*	*Lactobacillaceae*	*Lactobacillus*	*Lactobacillus*
2954	*Firmicutes*	*Bacilli*	*Lactobacillales*	*Lactobacillaceae*	*Lactobacillus*	*Lactobacillus*
2955	*Firmicutes*	*Bacilli*	*Lactobacillales*	*Lactobacillaceae*	*Lactobacillus*	*Lactobacillus*
2956	*Firmicutes*	*Bacilli*	*Lactobacillales*	*Lactobacillaceae*	*Lactobacillus*	*Lactobacillus*
2957	*Firmicutes*	*Bacilli*	*Lactobacillales*	*Lactobacillaceae*	*Lactobacillus*	*Lactobacillus*
2958	*Firmicutes*	*Bacilli*	*Lactobacillales*	*Lactobacillaceae*	*Lactobacillus*	*Lactobacillus*
2959	*Firmicutes*	*Bacilli*	*Lactobacillales*	*Lactobacillaceae*	*Lactobacillus*	*Lactobacillus*
2960	*Firmicutes*	*Bacilli*	*Lactobacillales*	*Lactobacillaceae*	*Lactobacillus*	*Lactobacillus*
2961	*Firmicutes*	*Bacilli*	*Lactobacillales*	*Lactobacillaceae*	*Lactobacillus*	*Lactobacillus*
2962	*Firmicutes*	*Bacilli*	*Lactobacillales*	*Lactobacillaceae*	*Lactobacillus*	*Lactobacillus*
2963	*Firmicutes*	*Bacilli*	*Lactobacillales*	*Lactobacillaceae*	*Lactobacillus*	*Lactobacillus*
2964	*Firmicutes*	*Bacilli*	*Lactobacillales*	*Lactobacillaceae*	*Lactobacillus*	*Lactobacillus*
2965	*Firmicutes*	*Bacilli*	*Lactobacillales*	*Lactobacillaceae*	*Lactobacillus*	*Lactobacillus*
2966	*Firmicutes*	*Bacilli*	*Lactobacillales*	*Lactobacillaceae*	*Lactobacillus*	*Lactobacillus*
2967	*Firmicutes*	*Bacilli*	*Lactobacillales*	*Lactobacillaceae*	*Lactobacillus*	*Lactobacillus*
2968	*Firmicutes*	*Bacilli*	*Lactobacillales*	*Lactobacillaceae*	*Lactobacillus*	*Lactobacillus*

Table S4. Isolated strains by culturomics

bacteriaId	*phylum*	*class*	*order*	*family*	*genus*	*species*
2969	*Firmicutes*	*Bacilli*	*Lactobacillales*	*Lactobacillaceae*	*Lactobacillus*	*Lactobacillus*
2970	*Firmicutes*	*Bacilli*	*Lactobacillales*	*Lactobacillaceae*	*Lactobacillus*	*Lactobacillus*
2971	*Firmicutes*	*Bacilli*	*Lactobacillales*	*Lactobacillaceae*	*Lactobacillus*	*Lactobacillus*
2972	*Firmicutes*	*Bacilli*	*Lactobacillales*	*Lactobacillaceae*	*Lactobacillus*	*Lactobacillus*
2973	*Firmicutes*	*Bacilli*	*Lactobacillales*	*Lactobacillaceae*	*Lactobacillus*	*Lactobacillus*
2974	*Firmicutes*	*Bacilli*	*Lactobacillales*	*Lactobacillaceae*	*Lactobacillus*	*Lactobacillus*
2975	*Firmicutes*	*Bacilli*	*Lactobacillales*	*Lactobacillaceae*	*Lactobacillus*	*Lactobacillus*
2976	*Firmicutes*	*Bacilli*	*Lactobacillales*	*Lactobacillaceae*	*Lactobacillus*	*Lactobacillus*
2977	*Firmicutes*	*Bacilli*	*Lactobacillales*	*Lactobacillaceae*	*Lactobacillus*	*Lactobacillus*
2978	*Firmicutes*	*Bacilli*	*Lactobacillales*	*Lactobacillaceae*	*Lactobacillus*	*Lactobacillus*
2979	*Firmicutes*	*Bacilli*	*Lactobacillales*	*Lactobacillaceae*	*Lactobacillus*	*Lactobacillus*
2980	*Firmicutes*	*Bacilli*	*Lactobacillales*	*Lactobacillaceae*	*Lactobacillus*	*Lactobacillus*
2981	*Firmicutes*	*Bacilli*	*Lactobacillales*	*Lactobacillaceae*	*Lactobacillus*	*Lactobacillus*
2982	*Firmicutes*	*Bacilli*	*Lactobacillales*	*Lactobacillaceae*	*Lactobacillus*	*Lactobacillus*
2983	*Firmicutes*	*Bacilli*	*Lactobacillales*	*Lactobacillaceae*	*Lactobacillus*	*Lactobacillus*
2984	*Firmicutes*	*Bacilli*	*Lactobacillales*	*Lactobacillaceae*	*Lactobacillus*	*Lactobacillus*
2985	*Firmicutes*	*Bacilli*	*Lactobacillales*	*Lactobacillaceae*	*Lactobacillus*	*Lactobacillus*
2986	*Firmicutes*	*Bacilli*	*Lactobacillales*	*Lactobacillaceae*	*Lactobacillus*	*Lactobacillus*
2987	*Firmicutes*	*Bacilli*	*Lactobacillales*	*Lactobacillaceae*	*Lactobacillus*	*Lactobacillus*
2988	*Firmicutes*	*Bacilli*	*Lactobacillales*	*Lactobacillaceae*	*Lactobacillus*	*Lactobacillus*
2989	*Firmicutes*	*Bacilli*	*Lactobacillales*	*Lactobacillaceae*	*Lactobacillus*	*Lactobacillus*
2990	*Firmicutes*	*Bacilli*	*Lactobacillales*	*Lactobacillaceae*	*Lactobacillus*	*Lactobacillus*
2991	*Firmicutes*	*Bacilli*	*Lactobacillales*	*Lactobacillaceae*	*Lactobacillus*	*Lactobacillus*
2992	*Firmicutes*	*Bacilli*	*Lactobacillales*	*Lactobacillaceae*	*Lactobacillus*	*Lactobacillus*
2993	*Firmicutes*	*Bacilli*	*Lactobacillales*	*Lactobacillaceae*	*Lactobacillus*	*Lactobacillus*
2994	*Firmicutes*	*Bacilli*	*Lactobacillales*	*Lactobacillaceae*	*Lactobacillus*	*Lactobacillus*
2995	*Firmicutes*	*Bacilli*	*Lactobacillales*	*Lactobacillaceae*	*Lactobacillus*	*Lactobacillus*
2996	*Firmicutes*	*Bacilli*	*Lactobacillales*	*Lactobacillaceae*	*Lactobacillus*	*Lactobacillus*

Table S4. Isolated strains by culturomics

bacteriaId	*phylum*	*class*	*order*	*family*	*genus*	*species*
2997	*Firmicutes*	*Bacilli*	*Lactobacillales*	*Lactobacillaceae*	*Lactobacillus*	*Lactobacillus*
2998	*Firmicutes*	*Bacilli*	*Lactobacillales*	*Lactobacillaceae*	*Lactobacillus*	*Lactobacillus*
2999	*Firmicutes*	*Bacilli*	*Lactobacillales*	*Lactobacillaceae*	*Lactobacillus*	*Lactobacillus*
3000	*Firmicutes*	*Bacilli*	*Lactobacillales*	*Lactobacillaceae*	*Lactobacillus*	*Lactobacillus*
3001	*Firmicutes*	*Bacilli*	*Lactobacillales*	*Lactobacillaceae*	*Lactobacillus*	*Lactobacillus*
3002	*Firmicutes*	*Bacilli*	*Lactobacillales*	*Lactobacillaceae*	*Lactobacillus*	*Lactobacillus*
3003	*Firmicutes*	*Bacilli*	*Lactobacillales*	*Lactobacillaceae*	*Lactobacillus*	*Lactobacillus*
3004	*Firmicutes*	*Bacilli*	*Lactobacillales*	*Lactobacillaceae*	*Lactobacillus*	*Lactobacillus*
3005	*Firmicutes*	*Bacilli*	*Lactobacillales*	*Lactobacillaceae*	*Lactobacillus*	*Lactobacillus*
3006	*Firmicutes*	*Bacilli*	*Lactobacillales*	*Lactobacillaceae*	*Lactobacillus*	*Lactobacillus*
3007	*Firmicutes*	*Bacilli*	*Lactobacillales*	*Lactobacillaceae*	*Lactobacillus*	*Lactobacillus*
3008	*Firmicutes*	*Bacilli*	*Lactobacillales*	*Lactobacillaceae*	*Lactobacillus*	*Lactobacillus*
3009	*Firmicutes*	*Bacilli*	*Lactobacillales*	*Lactobacillaceae*	*Lactobacillus*	*Lactobacillus*
3010	*Firmicutes*	*Bacilli*	*Lactobacillales*	*Lactobacillaceae*	*Lactobacillus*	*Lactobacillus*
3011	*Firmicutes*	*Bacilli*	*Lactobacillales*	*Lactobacillaceae*	*Lactobacillus*	*Lactobacillus*
3012	*Firmicutes*	*Bacilli*	*Lactobacillales*	*Lactobacillaceae*	*Lactobacillus*	*Lactobacillus*
3013	*Firmicutes*	*Bacilli*	*Lactobacillales*	*Lactobacillaceae*	*Lactobacillus*	*Lactobacillus*
3014	*Firmicutes*	*Bacilli*	*Lactobacillales*	*Lactobacillaceae*	*Lactobacillus*	*Lactobacillus*
3015	*Firmicutes*	*Bacilli*	*Lactobacillales*	*Lactobacillaceae*	*Lactobacillus*	*Lactobacillus*
3016	*Firmicutes*	*Bacilli*	*Lactobacillales*	*Lactobacillaceae*	*Lactobacillus*	*Lactobacillus*
3017	*Firmicutes*	*Bacilli*	*Lactobacillales*	*Lactobacillaceae*	*Lactobacillus*	*Lactobacillus*
3018	*Firmicutes*	*Bacilli*	*Lactobacillales*	*Lactobacillaceae*	*Lactobacillus*	*Lactobacillus*
3019	*Firmicutes*	*Bacilli*	*Lactobacillales*	*Lactobacillaceae*	*Lactobacillus*	*Lactobacillus*
3020	*Firmicutes*	*Bacilli*	*Lactobacillales*	*Lactobacillaceae*	*Lactobacillus*	*Lactobacillus*
3021	*Firmicutes*	*Bacilli*	*Lactobacillales*	*Lactobacillaceae*	*Lactobacillus*	*Lactobacillus*
3022	*Firmicutes*	*Bacilli*	*Lactobacillales*	*Lactobacillaceae*	*Lactobacillus*	*Lactobacillus*
3023	*Firmicutes*	*Bacilli*	*Lactobacillales*	*Lactobacillaceae*	*Lactobacillus*	*Lactobacillus*
3024	*Firmicutes*	*Bacilli*	*Lactobacillales*	*Lactobacillaceae*	*Lactobacillus*	*Lactobacillus*

Table S4. Isolated strains by culturomics

bacteriaId	*phylum*	*class*	*order*	*family*	*genus*	*species*
3025	*Firmicutes*	*Bacilli*	*Lactobacillales*	*Lactobacillaceae*	*Lactobacillus*	*Lactobacillus*
3026	*Firmicutes*	*Bacilli*	*Lactobacillales*	*Lactobacillaceae*	*Lactobacillus*	*Lactobacillus*
3027	*Firmicutes*	*Bacilli*	*Lactobacillales*	*Lactobacillaceae*	*Lactobacillus*	*Lactobacillus*
3028	*Firmicutes*	*Bacilli*	*Lactobacillales*	*Lactobacillaceae*	*Lactobacillus*	*Lactobacillus*
3029	*Firmicutes*	*Bacilli*	*Lactobacillales*	*Lactobacillaceae*	*Lactobacillus*	*Lactobacillus*
3030	*Firmicutes*	*Bacilli*	*Lactobacillales*	*Lactobacillaceae*	*Lactobacillus*	*Lactobacillus*
3031	*Firmicutes*	*Bacilli*	*Lactobacillales*	*Lactobacillaceae*	*Lactobacillus*	*Lactobacillus*
3032	*Firmicutes*	*Bacilli*	*Lactobacillales*	*Lactobacillaceae*	*Lactobacillus*	*Lactobacillus*
3033	*Firmicutes*	*Bacilli*	*Lactobacillales*	*Lactobacillaceae*	*Lactobacillus*	*Lactobacillus*
3034	*Firmicutes*	*Bacilli*	*Lactobacillales*	*Lactobacillaceae*	*Lactobacillus*	*Lactobacillus*
3035	*Firmicutes*	*Bacilli*	*Lactobacillales*	*Lactobacillaceae*	*Lactobacillus*	*Lactobacillus*
3036	*Firmicutes*	*Bacilli*	*Lactobacillales*	*Lactobacillaceae*	*Lactobacillus*	*Lactobacillus*
3037	*Firmicutes*	*Bacilli*	*Lactobacillales*	*Lactobacillaceae*	*Lactobacillus*	*Lactobacillus*
3038	*Firmicutes*	*Bacilli*	*Lactobacillales*	*Lactobacillaceae*	*Lactobacillus*	*Lactobacillus*
3039	*Firmicutes*	*Bacilli*	*Lactobacillales*	*Lactobacillaceae*	*Lactobacillus*	*Lactobacillus*
3040	*Firmicutes*	*Bacilli*	*Lactobacillales*	*Lactobacillaceae*	*Lactobacillus*	*Lactobacillus*
3041	*Firmicutes*	*Bacilli*	*Lactobacillales*	*Lactobacillaceae*	*Lactobacillus*	*Lactobacillus*
3042	*Firmicutes*	*Bacilli*	*Lactobacillales*	*Lactobacillaceae*	*Lactobacillus*	*Lactobacillus*
3043	*Firmicutes*	*Bacilli*	*Lactobacillales*	*Lactobacillaceae*	*Lactobacillus*	*Lactobacillus*
3044	*Firmicutes*	*Bacilli*	*Lactobacillales*	*Lactobacillaceae*	*Lactobacillus*	*Lactobacillus*
3045	*Firmicutes*	*Bacilli*	*Lactobacillales*	*Lactobacillaceae*	*Lactobacillus*	*Lactobacillus*
3046	*Firmicutes*	*Bacilli*	*Lactobacillales*	*Lactobacillaceae*	*Lactobacillus*	*Lactobacillus*
3047	*Firmicutes*	*Bacilli*	*Lactobacillales*	*Lactobacillaceae*	*Lactobacillus*	*Lactobacillus*
3048	*Firmicutes*	*Bacilli*	*Lactobacillales*	*Lactobacillaceae*	*Lactobacillus*	*Lactobacillus*
3049	*Firmicutes*	*Bacilli*	*Lactobacillales*	*Lactobacillaceae*	*Lactobacillus*	*Lactobacillus*
3050	*Firmicutes*	*Bacilli*	*Lactobacillales*	*Lactobacillaceae*	*Lactobacillus*	*Lactobacillus*
3051	*Firmicutes*	*Bacilli*	*Lactobacillales*	*Lactobacillaceae*	*Lactobacillus*	*Lactobacillus*
3052	*Firmicutes*	*Bacilli*	*Lactobacillales*	*Lactobacillaceae*	*Lactobacillus*	*Lactobacillus*

Table S4. Isolated strains by culturomics

bacteriaId	*phylum*	*class*	*order*	*family*	*genus*	*species*
3053	*Firmicutes*	*Bacilli*	*Lactobacillales*	*Lactobacillaceae*	*Lactobacillus*	*Lactobacillus*
3054	*Firmicutes*	*Bacilli*	*Lactobacillales*	*Lactobacillaceae*	*Lactobacillus*	*Lactobacillus*
3055	*Firmicutes*	*Bacilli*	*Lactobacillales*	*Lactobacillaceae*	*Lactobacillus*	*Lactobacillus*
3056	*Firmicutes*	*Bacilli*	*Lactobacillales*	*Lactobacillaceae*	*Lactobacillus*	*Lactobacillus*
3057	*Firmicutes*	*Bacilli*	*Lactobacillales*	*Lactobacillaceae*	*Lactobacillus*	*Lactobacillus*
3058	*Firmicutes*	*Bacilli*	*Lactobacillales*	*Lactobacillaceae*	*Lactobacillus*	*Lactobacillus*
3059	*Firmicutes*	*Bacilli*	*Lactobacillales*	*Lactobacillaceae*	*Lactobacillus*	*Lactobacillus*
3060	*Firmicutes*	*Bacilli*	*Lactobacillales*	*Lactobacillaceae*	*Lactobacillus*	*Lactobacillus*
3061	*Firmicutes*	*Bacilli*	*Lactobacillales*	*Lactobacillaceae*	*Lactobacillus*	*Lactobacillus*
3062	*Firmicutes*	*Bacilli*	*Lactobacillales*	*Lactobacillaceae*	*Lactobacillus*	*Lactobacillus*
3063	*Firmicutes*	*Bacilli*	*Lactobacillales*	*Lactobacillaceae*	*Lactobacillus*	*Lactobacillus*
3064	*Firmicutes*	*Bacilli*	*Lactobacillales*	*Lactobacillaceae*	*Lactobacillus*	*Lactobacillus*
3065	*Firmicutes*	*Bacilli*	*Lactobacillales*	*Lactobacillaceae*	*Lactobacillus*	*Lactobacillus*
3066	*Firmicutes*	*Bacilli*	*Lactobacillales*	*Lactobacillaceae*	*Lactobacillus*	*Lactobacillus*
3067	*Firmicutes*	*Bacilli*	*Lactobacillales*	*Lactobacillaceae*	*Lactobacillus*	*Lactobacillus*
3068	*Firmicutes*	*Bacilli*	*Lactobacillales*	*Lactobacillaceae*	*Lactobacillus*	*Lactobacillus*
3069	*Firmicutes*	*Bacilli*	*Lactobacillales*	*Lactobacillaceae*	*Lactobacillus*	*Lactobacillus*
3070	*Firmicutes*	*Bacilli*	*Lactobacillales*	*Lactobacillaceae*	*Lactobacillus*	*Lactobacillus*
3071	*Firmicutes*	*Bacilli*	*Lactobacillales*	*Lactobacillaceae*	*Lactobacillus*	*Lactobacillus*
3072	*Firmicutes*	*Bacilli*	*Lactobacillales*	*Lactobacillaceae*	*Lactobacillus*	*Lactobacillus*
3073	*Firmicutes*	*Bacilli*	*Lactobacillales*	*Lactobacillaceae*	*Lactobacillus*	*Lactobacillus*
3074	*Firmicutes*	*Bacilli*	*Lactobacillales*	*Lactobacillaceae*	*Lactobacillus*	*Lactobacillus*
3075	*Firmicutes*	*Bacilli*	*Lactobacillales*	*Lactobacillaceae*	*Lactobacillus*	*Lactobacillus*
3076	*Firmicutes*	*Bacilli*	*Lactobacillales*	*Lactobacillaceae*	*Lactobacillus*	*Lactobacillus*
3077	*Firmicutes*	*Bacilli*	*Lactobacillales*	*Lactobacillaceae*	*Lactobacillus*	*Lactobacillus*
3078	*Firmicutes*	*Bacilli*	*Lactobacillales*	*Lactobacillaceae*	*Lactobacillus*	*Lactobacillus*
3079	*Firmicutes*	*Bacilli*	*Lactobacillales*	*Lactobacillaceae*	*Lactobacillus*	*Lactobacillus*
3080	*Firmicutes*	*Bacilli*	*Lactobacillales*	*Lactobacillaceae*	*Lactobacillus*	*Lactobacillus*

Table S4. Isolated strains by culturomics

bacteriaId	*phylum*	*class*	*order*	*family*	*genus*	*species*
3081	*Firmicutes*	*Bacilli*	*Lactobacillales*	*Lactobacillaceae*	*Lactobacillus*	*Lactobacillus*
3082	*Firmicutes*	*Bacilli*	*Lactobacillales*	*Lactobacillaceae*	*Lactobacillus*	*Lactobacillus*
3083	*Firmicutes*	*Bacilli*	*Lactobacillales*	*Lactobacillaceae*	*Lactobacillus*	*Lactobacillus*
3084	*Firmicutes*	*Bacilli*	*Lactobacillales*	*Lactobacillaceae*	*Lactobacillus*	*Lactobacillus*
3085	*Firmicutes*	*Bacilli*	*Lactobacillales*	*Lactobacillaceae*	*Lactobacillus*	*Lactobacillus*
3086	*Firmicutes*	*Bacilli*	*Lactobacillales*	*Lactobacillaceae*	*Lactobacillus*	*Lactobacillus*
3087	*Firmicutes*	*Bacilli*	*Lactobacillales*	*Lactobacillaceae*	*Lactobacillus*	*Lactobacillus*
3088	*Firmicutes*	*Bacilli*	*Lactobacillales*	*Lactobacillaceae*	*Lactobacillus*	*Lactobacillus*
3089	*Firmicutes*	*Bacilli*	*Lactobacillales*	*Lactobacillaceae*	*Lactobacillus*	*Lactobacillus*
3090	*Firmicutes*	*Bacilli*	*Lactobacillales*	*Lactobacillaceae*	*Lactobacillus*	*Lactobacillus*
3091	*Firmicutes*	*Bacilli*	*Lactobacillales*	*Lactobacillaceae*	*Lactobacillus*	*Lactobacillus*
3092	*Firmicutes*	*Bacilli*	*Lactobacillales*	*Lactobacillaceae*	*Lactobacillus*	*Lactobacillus*
3093	*Firmicutes*	*Bacilli*	*Lactobacillales*	*Lactobacillaceae*	*Lactobacillus*	*Lactobacillus*
3094	*Firmicutes*	*Bacilli*	*Lactobacillales*	*Lactobacillaceae*	*Lactobacillus*	*Lactobacillus*
3095	*Firmicutes*	*Bacilli*	*Lactobacillales*	*Lactobacillaceae*	*Lactobacillus*	*Lactobacillus*
3096	*Firmicutes*	*Bacilli*	*Lactobacillales*	*Lactobacillaceae*	*Lactobacillus*	*Lactobacillus*
3097	*Firmicutes*	*Bacilli*	*Lactobacillales*	*Lactobacillaceae*	*Lactobacillus*	*Lactobacillus*
3098	*Firmicutes*	*Bacilli*	*Lactobacillales*	*Lactobacillaceae*	*Lactobacillus*	*Lactobacillus*
3099	*Firmicutes*	*Bacilli*	*Lactobacillales*	*Lactobacillaceae*	*Lactobacillus*	*Lactobacillus*
3100	*Firmicutes*	*Bacilli*	*Lactobacillales*	*Lactobacillaceae*	*Lactobacillus*	*Lactobacillus*
3101	*Firmicutes*	*Bacilli*	*Lactobacillales*	*Lactobacillaceae*	*Lactobacillus*	*Lactobacillus*
3102	*Firmicutes*	*Bacilli*	*Lactobacillales*	*Lactobacillaceae*	*Lactobacillus*	*Lactobacillus*
3103	*Firmicutes*	*Bacilli*	*Lactobacillales*	*Lactobacillaceae*	*Lactobacillus*	*Lactobacillus*
3104	*Firmicutes*	*Bacilli*	*Lactobacillales*	*Lactobacillaceae*	*Lactobacillus*	*Lactobacillus*
3105	*Firmicutes*	*Bacilli*	*Lactobacillales*	*Lactobacillaceae*	*Lactobacillus*	*Lactobacillus*
3106	*Firmicutes*	*Bacilli*	*Lactobacillales*	*Lactobacillaceae*	*Lactobacillus*	*Lactobacillus*
3107	*Firmicutes*	*Bacilli*	*Lactobacillales*	*Lactobacillaceae*	*Lactobacillus*	*Lactobacillus*
3108	*Firmicutes*	*Bacilli*	*Lactobacillales*	*Lactobacillaceae*	*Lactobacillus*	*Lactobacillus*

Table S4. Isolated strains by culturomics

bacteriaId	*phylum*	*class*	*order*	*family*	*genus*	*species*
3109	*Firmicutes*	*Bacilli*	*Lactobacillales*	*Lactobacillaceae*	*Lactobacillus*	*Lactobacillus*
3110	*Firmicutes*	*Bacilli*	*Lactobacillales*	*Lactobacillaceae*	*Lactobacillus*	*Lactobacillus*
3111	*Firmicutes*	*Bacilli*	*Lactobacillales*	*Lactobacillaceae*	*Lactobacillus*	*Lactobacillus*
3112	*Firmicutes*	*Bacilli*	*Lactobacillales*	*Lactobacillaceae*	*Lactobacillus*	*Lactobacillus*
3113	*Firmicutes*	*Bacilli*	*Lactobacillales*	*Lactobacillaceae*	*Lactobacillus*	*Lactobacillus*
3114	*Firmicutes*	*Bacilli*	*Lactobacillales*	*Lactobacillaceae*	*Lactobacillus*	*Lactobacillus*
3115	*Firmicutes*	*Bacilli*	*Lactobacillales*	*Lactobacillaceae*	*Lactobacillus*	*Lactobacillus*
3116	*Firmicutes*	*Bacilli*	*Lactobacillales*	*Lactobacillaceae*	*Lactobacillus*	*Lactobacillus*
3117	*Firmicutes*	*Bacilli*	*Lactobacillales*	*Lactobacillaceae*	*Lactobacillus*	*Lactobacillus*
3118	*Firmicutes*	*Bacilli*	*Lactobacillales*	*Lactobacillaceae*	*Lactobacillus*	*Lactobacillus*
3119	*Firmicutes*	*Bacilli*	*Lactobacillales*	*Lactobacillaceae*	*Lactobacillus*	*Lactobacillus*
3120	*Firmicutes*	*Bacilli*	*Lactobacillales*	*Lactobacillaceae*	*Lactobacillus*	*Lactobacillus*
3121	*Firmicutes*	*Bacilli*	*Lactobacillales*	*Lactobacillaceae*	*Lactobacillus*	*Lactobacillus*
3122	*Firmicutes*	*Bacilli*	*Lactobacillales*	*Lactobacillaceae*	*Lactobacillus*	*Lactobacillus*
3123	*Firmicutes*	*Bacilli*	*Lactobacillales*	*Lactobacillaceae*	*Lactobacillus*	*Lactobacillus*
3124	*Firmicutes*	*Bacilli*	*Lactobacillales*	*Lactobacillaceae*	*Lactobacillus*	*Lactobacillus*
3125	*Firmicutes*	*Bacilli*	*Lactobacillales*	*Lactobacillaceae*	*Lactobacillus*	*Lactobacillus*
3126	*Firmicutes*	*Bacilli*	*Lactobacillales*	*Lactobacillaceae*	*Lactobacillus*	*Lactobacillus*
3127	*Firmicutes*	*Bacilli*	*Lactobacillales*	*Lactobacillaceae*	*Lactobacillus*	*Lactobacillus*
3128	*Firmicutes*	*Bacilli*	*Lactobacillales*	*Lactobacillaceae*	*Lactobacillus*	*Lactobacillus*
3129	*Firmicutes*	*Bacilli*	*Lactobacillales*	*Lactobacillaceae*	*Lactobacillus*	*Lactobacillus*
3130	*Firmicutes*	*Bacilli*	*Lactobacillales*	*Lactobacillaceae*	*Lactobacillus*	*Lactobacillus*
3131	*Firmicutes*	*Bacilli*	*Lactobacillales*	*Lactobacillaceae*	*Lactobacillus*	*Lactobacillus*
3132	*Firmicutes*	*Bacilli*	*Lactobacillales*	*Lactobacillaceae*	*Lactobacillus*	*Lactobacillus*
3133	*Firmicutes*	*Bacilli*	*Lactobacillales*	*Lactobacillaceae*	*Lactobacillus*	*Lactobacillus*
3134	*Firmicutes*	*Bacilli*	*Lactobacillales*	*Lactobacillaceae*	*Lactobacillus*	*Lactobacillus*
3135	*Firmicutes*	*Bacilli*	*Lactobacillales*	*Lactobacillaceae*	*Lactobacillus*	*Lactobacillus*
3136	*Firmicutes*	*Bacilli*	*Lactobacillales*	*Lactobacillaceae*	*Lactobacillus*	*Lactobacillus*

Table S4. Isolated strains by culturomics

bacteriaId	*phylum*	*class*	*order*	*family*	*genus*	*species*
3137	*Firmicutes*	*Bacilli*	*Lactobacillales*	*Lactobacillaceae*	*Lactobacillus*	*Lactobacillus*
3138	*Firmicutes*	*Bacilli*	*Lactobacillales*	*Lactobacillaceae*	*Lactobacillus*	*Lactobacillus*
3139	*Firmicutes*	*Bacilli*	*Lactobacillales*	*Lactobacillaceae*	*Lactobacillus*	*Lactobacillus*
3140	*Firmicutes*	*Bacilli*	*Lactobacillales*	*Lactobacillaceae*	*Lactobacillus*	*Lactobacillus*
3141	*Firmicutes*	*Bacilli*	*Lactobacillales*	*Lactobacillaceae*	*Lactobacillus*	*Lactobacillus*
3142	*Firmicutes*	*Bacilli*	*Lactobacillales*	*Lactobacillaceae*	*Lactobacillus*	*Lactobacillus*
3143	*Firmicutes*	*Bacilli*	*Lactobacillales*	*Lactobacillaceae*	*Lactobacillus*	*Lactobacillus*
3144	*Firmicutes*	*Bacilli*	*Lactobacillales*	*Lactobacillaceae*	*Lactobacillus*	*Lactobacillus*
3145	*Firmicutes*	*Bacilli*	*Lactobacillales*	*Lactobacillaceae*	*Lactobacillus*	*Lactobacillus*
3146	*Firmicutes*	*Bacilli*	*Lactobacillales*	*Lactobacillaceae*	*Lactobacillus*	*Lactobacillus*
3147	*Firmicutes*	*Bacilli*	*Lactobacillales*	*Lactobacillaceae*	*Lactobacillus*	*Lactobacillus*
3148	*Firmicutes*	*Bacilli*	*Lactobacillales*	*Lactobacillaceae*	*Lactobacillus*	*Lactobacillus*
3149	*Firmicutes*	*Bacilli*	*Lactobacillales*	*Lactobacillaceae*	*Lactobacillus*	*Lactobacillus*
3150	*Firmicutes*	*Bacilli*	*Lactobacillales*	*Lactobacillaceae*	*Lactobacillus*	*Lactobacillus*
3151	*Firmicutes*	*Bacilli*	*Lactobacillales*	*Lactobacillaceae*	*Lactobacillus*	*Lactobacillus*
3152	*Firmicutes*	*Bacilli*	*Lactobacillales*	*Lactobacillaceae*	*Lactobacillus*	*Lactobacillus*
3153	*Firmicutes*	*Bacilli*	*Lactobacillales*	*Lactobacillaceae*	*Lactobacillus*	*Lactobacillus*
3154	*Firmicutes*	*Bacilli*	*Lactobacillales*	*Lactobacillaceae*	*Lactobacillus*	*Lactobacillus*
3155	*Firmicutes*	*Bacilli*	*Lactobacillales*	*Lactobacillaceae*	*Lactobacillus*	*Lactobacillus*
3156	*Firmicutes*	*Bacilli*	*Lactobacillales*	*Lactobacillaceae*	*Lactobacillus*	*Lactobacillus*
3157	*Firmicutes*	*Bacilli*	*Lactobacillales*	*Lactobacillaceae*	*Lactobacillus*	*Lactobacillus*
3158	*Firmicutes*	*Bacilli*	*Lactobacillales*	*Lactobacillaceae*	*Lactobacillus*	*Lactobacillus*
3159	*Firmicutes*	*Bacilli*	*Lactobacillales*	*Lactobacillaceae*	*Lactobacillus*	*Lactobacillus*
3160	*Firmicutes*	*Bacilli*	*Lactobacillales*	*Lactobacillaceae*	*Lactobacillus*	*Lactobacillus*
3161	*Firmicutes*	*Bacilli*	*Lactobacillales*	*Lactobacillaceae*	*Lactobacillus*	*Lactobacillus*
3162	*Firmicutes*	*Bacilli*	*Lactobacillales*	*Lactobacillaceae*	*Lactobacillus*	*Lactobacillus*
3163	*Firmicutes*	*Bacilli*	*Lactobacillales*	*Lactobacillaceae*	*Lactobacillus*	*Lactobacillus*
3164	*Firmicutes*	*Bacilli*	*Lactobacillales*	*Lactobacillaceae*	*Lactobacillus*	*Lactobacillus*

Table S4. Isolated strains by culturomics

bacteriaId	*phylum*	*class*	*order*	*family*	*genus*	*species*
3165	*Firmicutes*	*Bacilli*	*Lactobacillales*	*Lactobacillaceae*	*Lactobacillus*	*Lactobacillus*
3166	*Firmicutes*	*Bacilli*	*Lactobacillales*	*Lactobacillaceae*	*Lactobacillus*	*Lactobacillus*
3167	*Firmicutes*	*Bacilli*	*Lactobacillales*	*Lactobacillaceae*	*Lactobacillus*	*Lactobacillus*
3168	*Firmicutes*	*Bacilli*	*Lactobacillales*	*Lactobacillaceae*	*Lactobacillus*	*Lactobacillus*
3169	*Firmicutes*	*Bacilli*	*Lactobacillales*	*Lactobacillaceae*	*Lactobacillus*	*Lactobacillus*
3170	*Firmicutes*	*Bacilli*	*Lactobacillales*	*Lactobacillaceae*	*Lactobacillus*	*Lactobacillus*
3171	*Firmicutes*	*Bacilli*	*Lactobacillales*	*Lactobacillaceae*	*Lactobacillus*	*Lactobacillus*
3172	*Firmicutes*	*Bacilli*	*Lactobacillales*	*Lactobacillaceae*	*Lactobacillus*	*Lactobacillus*
3173	*Firmicutes*	*Bacilli*	*Lactobacillales*	*Lactobacillaceae*	*Lactobacillus*	*Lactobacillus*
3174	*Firmicutes*	*Bacilli*	*Lactobacillales*	*Lactobacillaceae*	*Lactobacillus*	*Lactobacillus*
3175	*Firmicutes*	*Bacilli*	*Lactobacillales*	*Lactobacillaceae*	*Lactobacillus*	*Lactobacillus*
3176	*Firmicutes*	*Bacilli*	*Lactobacillales*	*Lactobacillaceae*	*Lactobacillus*	*Lactobacillus*
3177	*Firmicutes*	*Bacilli*	*Lactobacillales*	*Lactobacillaceae*	*Lactobacillus*	*Lactobacillus*
3178	*Firmicutes*	*Bacilli*	*Lactobacillales*	*Lactobacillaceae*	*Lactobacillus*	*Lactobacillus*
3179	*Firmicutes*	*Bacilli*	*Lactobacillales*	*Lactobacillaceae*	*Lactobacillus*	*Lactobacillus*
3180	*Firmicutes*	*Bacilli*	*Lactobacillales*	*Lactobacillaceae*	*Lactobacillus*	*Lactobacillus*
3181	*Firmicutes*	*Bacilli*	*Lactobacillales*	*Lactobacillaceae*	*Lactobacillus*	*Lactobacillus*
3182	*Firmicutes*	*Bacilli*	*Lactobacillales*	*Lactobacillaceae*	*Lactobacillus*	*Lactobacillus*
3183	*Firmicutes*	*Bacilli*	*Lactobacillales*	*Lactobacillaceae*	*Lactobacillus*	*Lactobacillus*
3184	*Firmicutes*	*Bacilli*	*Lactobacillales*	*Lactobacillaceae*	*Lactobacillus*	*Lactobacillus*
3185	*Firmicutes*	*Bacilli*	*Lactobacillales*	*Lactobacillaceae*	*Lactobacillus*	*Lactobacillus*
3186	*Firmicutes*	*Bacilli*	*Lactobacillales*	*Lactobacillaceae*	*Lactobacillus*	*Lactobacillus*
3187	*Firmicutes*	*Bacilli*	*Lactobacillales*	*Lactobacillaceae*	*Lactobacillus*	*Lactobacillus*
3188	*Firmicutes*	*Bacilli*	*Lactobacillales*	*Lactobacillaceae*	*Lactobacillus*	*Lactobacillus*
3189	*Firmicutes*	*Bacilli*	*Lactobacillales*	*Lactobacillaceae*	*Lactobacillus*	*Lactobacillus*
3190	*Firmicutes*	*Bacilli*	*Lactobacillales*	*Lactobacillaceae*	*Lactobacillus*	*Lactobacillus*
3191	*Firmicutes*	*Bacilli*	*Lactobacillales*	*Lactobacillaceae*	*Lactobacillus*	*Lactobacillus*
3192	*Firmicutes*	*Bacilli*	*Lactobacillales*	*Lactobacillaceae*	*Lactobacillus*	*Lactobacillus*

Table S4. Isolated strains by culturomics

bacteriaId	*phylum*	*class*	*order*	*family*	*genus*	*species*
3193	*Firmicutes*	*Bacilli*	*Lactobacillales*	*Lactobacillaceae*	*Lactobacillus*	*Lactobacillus*
3194	*Firmicutes*	*Bacilli*	*Lactobacillales*	*Lactobacillaceae*	*Lactobacillus*	*Lactobacillus*
3195	*Firmicutes*	*Bacilli*	*Lactobacillales*	*Lactobacillaceae*	*Lactobacillus*	*Lactobacillus*
3196	*Firmicutes*	*Bacilli*	*Lactobacillales*	*Lactobacillaceae*	*Lactobacillus*	*Lactobacillus*
3197	*Firmicutes*	*Bacilli*	*Lactobacillales*	*Lactobacillaceae*	*Lactobacillus*	*Lactobacillus*
3198	*Firmicutes*	*Bacilli*	*Lactobacillales*	*Lactobacillaceae*	*Lactobacillus*	*Lactobacillus*
3199	*Firmicutes*	*Bacilli*	*Lactobacillales*	*Lactobacillaceae*	*Lactobacillus*	*Lactobacillus*
3200	*Firmicutes*	*Bacilli*	*Lactobacillales*	*Lactobacillaceae*	*Lactobacillus*	*Lactobacillus*
3201	*Firmicutes*	*Bacilli*	*Lactobacillales*	*Lactobacillaceae*	*Lactobacillus*	*Lactobacillus*
3202	*Firmicutes*	*Bacilli*	*Lactobacillales*	*Lactobacillaceae*	*Lactobacillus*	*Lactobacillus*
3203	*Firmicutes*	*Bacilli*	*Lactobacillales*	*Lactobacillaceae*	*Lactobacillus*	*Lactobacillus*
3204	*Firmicutes*	*Bacilli*	*Lactobacillales*	*Lactobacillaceae*	*Lactobacillus*	*Lactobacillus*
3205	*Firmicutes*	*Bacilli*	*Lactobacillales*	*Lactobacillaceae*	*Lactobacillus*	*Lactobacillus*
3206	*Firmicutes*	*Bacilli*	*Lactobacillales*	*Lactobacillaceae*	*Lactobacillus*	*Lactobacillus*
3207	*Firmicutes*	*Bacilli*	*Lactobacillales*	*Lactobacillaceae*	*Lactobacillus*	*Lactobacillus*
3208	*Firmicutes*	*Bacilli*	*Lactobacillales*	*Lactobacillaceae*	*Lactobacillus*	*Lactobacillus*
3209	*Firmicutes*	*Bacilli*	*Lactobacillales*	*Lactobacillaceae*	*Lactobacillus*	*Lactobacillus*
3210	*Firmicutes*	*Bacilli*	*Lactobacillales*	*Lactobacillaceae*	*Lactobacillus*	*Lactobacillus*
3211	*Firmicutes*	*Bacilli*	*Lactobacillales*	*Lactobacillaceae*	*Lactobacillus*	*Lactobacillus*
3212	*Firmicutes*	*Bacilli*	*Lactobacillales*	*Lactobacillaceae*	*Lactobacillus*	*Lactobacillus*
3213	*Firmicutes*	*Bacilli*	*Lactobacillales*	*Lactobacillaceae*	*Lactobacillus*	*Lactobacillus*
3214	*Firmicutes*	*Bacilli*	*Lactobacillales*	*Lactobacillaceae*	*Lactobacillus*	*Lactobacillus*
3215	*Firmicutes*	*Bacilli*	*Lactobacillales*	*Lactobacillaceae*	*Lactobacillus*	*Lactobacillus*
3216	*Firmicutes*	*Bacilli*	*Lactobacillales*	*Lactobacillaceae*	*Lactobacillus*	*Lactobacillus*
3217	*Firmicutes*	*Bacilli*	*Lactobacillales*	*Lactobacillaceae*	*Lactobacillus*	*Lactobacillus*
3218	*Firmicutes*	*Bacilli*	*Lactobacillales*	*Lactobacillaceae*	*Lactobacillus*	*Lactobacillus*
3219	*Firmicutes*	*Bacilli*	*Lactobacillales*	*Lactobacillaceae*	*Lactobacillus*	*Lactobacillus*
3220	*Firmicutes*	*Bacilli*	*Lactobacillales*	*Lactobacillaceae*	*Lactobacillus*	*Lactobacillus*

Table S4. Isolated strains by culturomics

bacteriaId	*phylum*	*class*	*order*	*family*	*genus*	*species*
3221	*Firmicutes*	*Bacilli*	*Lactobacillales*	*Lactobacillaceae*	*Lactobacillus*	*Lactobacillus*
3222	*Firmicutes*	*Bacilli*	*Lactobacillales*	*Lactobacillaceae*	*Lactobacillus*	*Lactobacillus*
3223	*Firmicutes*	*Bacilli*	*Lactobacillales*	*Lactobacillaceae*	*Lactobacillus*	*Lactobacillus*
3224	*Firmicutes*	*Bacilli*	*Lactobacillales*	*Lactobacillaceae*	*Lactobacillus*	*Lactobacillus*
3225	*Firmicutes*	*Bacilli*	*Lactobacillales*	*Lactobacillaceae*	*Lactobacillus*	*Lactobacillus*
3226	*Firmicutes*	*Bacilli*	*Lactobacillales*	*Lactobacillaceae*	*Lactobacillus*	*Lactobacillus*
3227	*Firmicutes*	*Bacilli*	*Lactobacillales*	*Lactobacillaceae*	*Lactobacillus*	*Lactobacillus*
3228	*Firmicutes*	*Bacilli*	*Lactobacillales*	*Lactobacillaceae*	*Lactobacillus*	*Lactobacillus*
3229	*Firmicutes*	*Bacilli*	*Lactobacillales*	*Lactobacillaceae*	*Lactobacillus*	*Lactobacillus*
3230	*Firmicutes*	*Bacilli*	*Lactobacillales*	*Lactobacillaceae*	*Lactobacillus*	*Lactobacillus*
3231	*Firmicutes*	*Bacilli*	*Lactobacillales*	*Lactobacillaceae*	*Lactobacillus*	*Lactobacillus*
3232	*Firmicutes*	*Bacilli*	*Lactobacillales*	*Lactobacillaceae*	*Lactobacillus*	*Lactobacillus*
3233	*Firmicutes*	*Bacilli*	*Lactobacillales*	*Lactobacillaceae*	*Lactobacillus*	*Lactobacillus*
3234	*Firmicutes*	*Bacilli*	*Lactobacillales*	*Lactobacillaceae*	*Lactobacillus*	*Lactobacillus*
3235	*Firmicutes*	*Bacilli*	*Lactobacillales*	*Lactobacillaceae*	*Lactobacillus*	*Lactobacillus*
3236	*Firmicutes*	*Bacilli*	*Lactobacillales*	*Lactobacillaceae*	*Lactobacillus*	*Lactobacillus*
3237	*Firmicutes*	*Bacilli*	*Lactobacillales*	*Lactobacillaceae*	*Lactobacillus*	*Lactobacillus*
3238	*Firmicutes*	*Bacilli*	*Lactobacillales*	*Lactobacillaceae*	*Lactobacillus*	*Lactobacillus*
3239	*Firmicutes*	*Bacilli*	*Lactobacillales*	*Lactobacillaceae*	*Lactobacillus*	*Lactobacillus*
3240	*Firmicutes*	*Bacilli*	*Lactobacillales*	*Lactobacillaceae*	*Lactobacillus*	*Lactobacillus*
3241	*Firmicutes*	*Bacilli*	*Lactobacillales*	*Lactobacillaceae*	*Lactobacillus*	*Lactobacillus*
3242	*Firmicutes*	*Bacilli*	*Lactobacillales*	*Lactobacillaceae*	*Lactobacillus*	*Lactobacillus*
3243	*Firmicutes*	*Bacilli*	*Lactobacillales*	*Lactobacillaceae*	*Lactobacillus*	*Lactobacillus*
3244	*Firmicutes*	*Bacilli*	*Lactobacillales*	*Lactobacillaceae*	*Lactobacillus*	*Lactobacillus*
3245	*Firmicutes*	*Bacilli*	*Lactobacillales*	*Lactobacillaceae*	*Lactobacillus*	*Lactobacillus*
3246	*Firmicutes*	*Bacilli*	*Lactobacillales*	*Lactobacillaceae*	*Lactobacillus*	*Lactobacillus*
3247	*Firmicutes*	*Bacilli*	*Lactobacillales*	*Lactobacillaceae*	*Lactobacillus*	*Lactobacillus*
3248	*Firmicutes*	*Bacilli*	*Lactobacillales*	*Lactobacillaceae*	*Lactobacillus*	*Lactobacillus*

Table S4. Isolated strains by culturomics

bacteriaId	*phylum*	*class*	*order*	*family*	*genus*	*species*
3249	*Firmicutes*	*Bacilli*	*Lactobacillales*	*Lactobacillaceae*	*Lactobacillus*	*Lactobacillus*
3250	*Firmicutes*	*Bacilli*	*Lactobacillales*	*Lactobacillaceae*	*Lactobacillus*	*Lactobacillus*
3251	*Firmicutes*	*Bacilli*	*Lactobacillales*	*Lactobacillaceae*	*Lactobacillus*	*Lactobacillus*
3252	*Firmicutes*	*Bacilli*	*Lactobacillales*	*Lactobacillaceae*	*Lactobacillus*	*Lactobacillus*
3253	*Firmicutes*	*Bacilli*	*Lactobacillales*	*Lactobacillaceae*	*Lactobacillus*	*Lactobacillus*
3254	*Firmicutes*	*Bacilli*	*Lactobacillales*	*Lactobacillaceae*	*Lactobacillus*	*Lactobacillus*
3255	*Firmicutes*	*Bacilli*	*Lactobacillales*	*Lactobacillaceae*	*Lactobacillus*	*Lactobacillus*
3256	*Firmicutes*	*Bacilli*	*Lactobacillales*	*Lactobacillaceae*	*Lactobacillus*	*Lactobacillus*
3257	*Firmicutes*	*Bacilli*	*Lactobacillales*	*Lactobacillaceae*	*Lactobacillus*	*Lactobacillus*
3258	*Firmicutes*	*Bacilli*	*Lactobacillales*	*Lactobacillaceae*	*Lactobacillus*	*Lactobacillus*
3259	*Firmicutes*	*Bacilli*	*Lactobacillales*	*Lactobacillaceae*	*Lactobacillus*	*Lactobacillus*
3260	*Firmicutes*	*Bacilli*	*Lactobacillales*	*Lactobacillaceae*	*Lactobacillus*	*Lactobacillus*
3261	*Firmicutes*	*Bacilli*	*Lactobacillales*	*Lactobacillaceae*	*Lactobacillus*	*Lactobacillus*
3262	*Firmicutes*	*Bacilli*	*Lactobacillales*	*Lactobacillaceae*	*Lactobacillus*	*Lactobacillus*
3263	*Firmicutes*	*Bacilli*	*Lactobacillales*	*Lactobacillaceae*	*Lactobacillus*	*Lactobacillus*
3264	*Firmicutes*	*Bacilli*	*Lactobacillales*	*Lactobacillaceae*	*Lactobacillus*	*Lactobacillus*
3265	*Firmicutes*	*Bacilli*	*Lactobacillales*	*Lactobacillaceae*	*Lactobacillus*	*Lactobacillus*
3266	*Firmicutes*	*Bacilli*	*Lactobacillales*	*Lactobacillaceae*	*Lactobacillus*	*Lactobacillus*
3267	*Firmicutes*	*Bacilli*	*Lactobacillales*	*Lactobacillaceae*	*Lactobacillus*	*Lactobacillus*
3268	*Firmicutes*	*Bacilli*	*Lactobacillales*	*Lactobacillaceae*	*Lactobacillus*	*Lactobacillus*
3269	*Firmicutes*	*Bacilli*	*Lactobacillales*	*Lactobacillaceae*	*Lactobacillus*	*Lactobacillus*
3270	*Firmicutes*	*Bacilli*	*Lactobacillales*	*Lactobacillaceae*	*Lactobacillus*	*Lactobacillus*
3271	*Firmicutes*	*Bacilli*	*Lactobacillales*	*Lactobacillaceae*	*Lactobacillus*	*Lactobacillus*
3272	*Firmicutes*	*Bacilli*	*Lactobacillales*	*Lactobacillaceae*	*Lactobacillus*	*Lactobacillus*
3273	*Firmicutes*	*Bacilli*	*Lactobacillales*	*Lactobacillaceae*	*Lactobacillus*	*Lactobacillus*
3274	*Firmicutes*	*Bacilli*	*Lactobacillales*	*Lactobacillaceae*	*Lactobacillus*	*Lactobacillus*
3275	*Firmicutes*	*Bacilli*	*Lactobacillales*	*Lactobacillaceae*	*Lactobacillus*	*Lactobacillus*
3276	*Firmicutes*	*Bacilli*	*Lactobacillales*	*Lactobacillaceae*	*Lactobacillus*	*Lactobacillus*

Table S4. Isolated strains by culturomics

bacteriaId	*phylum*	*class*	*order*	*family*	*genus*	*species*
3277	*Firmicutes*	*Bacilli*	*Lactobacillales*	*Lactobacillaceae*	*Lactobacillus*	*Lactobacillus*
3278	*Firmicutes*	*Bacilli*	*Lactobacillales*	*Lactobacillaceae*	*Lactobacillus*	*Lactobacillus*
3279	*Firmicutes*	*Bacilli*	*Lactobacillales*	*Lactobacillaceae*	*Lactobacillus*	*Lactobacillus*
3280	*Firmicutes*	*Bacilli*	*Lactobacillales*	*Lactobacillaceae*	*Lactobacillus*	*Lactobacillus*
3281	*Firmicutes*	*Bacilli*	*Lactobacillales*	*Lactobacillaceae*	*Lactobacillus*	*Lactobacillus*
3282	*Firmicutes*	*Bacilli*	*Lactobacillales*	*Lactobacillaceae*	*Lactobacillus*	*Lactobacillus*
3283	*Firmicutes*	*Bacilli*	*Lactobacillales*	*Lactobacillaceae*	*Lactobacillus*	*Lactobacillus*
3284	*Firmicutes*	*Bacilli*	*Lactobacillales*	*Lactobacillaceae*	*Lactobacillus*	*Lactobacillus*
3285	*Firmicutes*	*Bacilli*	*Lactobacillales*	*Lactobacillaceae*	*Lactobacillus*	*Lactobacillus*
3286	*Firmicutes*	*Bacilli*	*Lactobacillales*	*Lactobacillaceae*	*Lactobacillus*	*Lactobacillus*
3287	*Firmicutes*	*Bacilli*	*Lactobacillales*	*Lactobacillaceae*	*Lactobacillus*	*Lactobacillus*
3288	*Firmicutes*	*Bacilli*	*Lactobacillales*	*Lactobacillaceae*	*Lactobacillus*	*Lactobacillus*
3289	*Firmicutes*	*Bacilli*	*Lactobacillales*	*Lactobacillaceae*	*Lactobacillus*	*Lactobacillus*
3290	*Firmicutes*	*Bacilli*	*Lactobacillales*	*Lactobacillaceae*	*Lactobacillus*	*Lactobacillus*
3291	*Firmicutes*	*Bacilli*	*Lactobacillales*	*Lactobacillaceae*	*Lactobacillus*	*Lactobacillus*
3292	*Firmicutes*	*Bacilli*	*Lactobacillales*	*Lactobacillaceae*	*Lactobacillus*	*Lactobacillus*
3293	*Firmicutes*	*Bacilli*	*Lactobacillales*	*Lactobacillaceae*	*Lactobacillus*	*Lactobacillus*
3294	*Firmicutes*	*Bacilli*	*Lactobacillales*	*Lactobacillaceae*	*Lactobacillus*	*Lactobacillus*
3295	*Firmicutes*	*Bacilli*	*Lactobacillales*	*Lactobacillaceae*	*Lactobacillus*	*Lactobacillus*
3296	*Firmicutes*	*Bacilli*	*Lactobacillales*	*Lactobacillaceae*	*Lactobacillus*	*Lactobacillus*
3297	*Firmicutes*	*Bacilli*	*Lactobacillales*	*Lactobacillaceae*	*Lactobacillus*	*Lactobacillus*
3298	*Firmicutes*	*Bacilli*	*Lactobacillales*	*Lactobacillaceae*	*Lactobacillus*	*Lactobacillus*
3299	*Firmicutes*	*Bacilli*	*Lactobacillales*	*Lactobacillaceae*	*Lactobacillus*	*Lactobacillus*
3300	*Firmicutes*	*Bacilli*	*Lactobacillales*	*Lactobacillaceae*	*Lactobacillus*	*Lactobacillus*
3301	*Firmicutes*	*Bacilli*	*Lactobacillales*	*Lactobacillaceae*	*Lactobacillus*	*Lactobacillus*
3302	*Firmicutes*	*Bacilli*	*Lactobacillales*	*Lactobacillaceae*	*Lactobacillus*	*Lactobacillus*
3303	*Firmicutes*	*Bacilli*	*Lactobacillales*	*Lactobacillaceae*	*Lactobacillus*	*Lactobacillus*
3304	*Firmicutes*	*Bacilli*	*Lactobacillales*	*Lactobacillaceae*	*Lactobacillus*	*Lactobacillus*

Table S4. Isolated strains by culturomics

bacteriaId	*phylum*	*class*	*order*	*family*	*genus*	*species*
3305	*Firmicutes*	*Bacilli*	*Lactobacillales*	*Lactobacillaceae*	*Lactobacillus*	*Lactobacillus*
3306	*Firmicutes*	*Bacilli*	*Lactobacillales*	*Lactobacillaceae*	*Lactobacillus*	*Lactobacillus*
3307	*Firmicutes*	*Bacilli*	*Lactobacillales*	*Lactobacillaceae*	*Lactobacillus*	*Lactobacillus*
3308	*Firmicutes*	*Bacilli*	*Lactobacillales*	*Lactobacillaceae*	*Lactobacillus*	*Lactobacillus*
3309	*Firmicutes*	*Bacilli*	*Lactobacillales*	*Lactobacillaceae*	*Lactobacillus*	*Lactobacillus*
3310	*Firmicutes*	*Bacilli*	*Lactobacillales*	*Lactobacillaceae*	*Lactobacillus*	*Lactobacillus*
3311	*Firmicutes*	*Bacilli*	*Lactobacillales*	*Lactobacillaceae*	*Lactobacillus*	*Lactobacillus*
3312	*Firmicutes*	*Bacilli*	*Lactobacillales*	*Lactobacillaceae*	*Lactobacillus*	*Lactobacillus*
3313	*Firmicutes*	*Bacilli*	*Lactobacillales*	*Lactobacillaceae*	*Lactobacillus*	*Lactobacillus*
3314	*Firmicutes*	*Bacilli*	*Lactobacillales*	*Lactobacillaceae*	*Lactobacillus*	*Lactobacillus*
3315	*Firmicutes*	*Bacilli*	*Lactobacillales*	*Lactobacillaceae*	*Lactobacillus*	*Lactobacillus*
3316	*Firmicutes*	*Bacilli*	*Lactobacillales*	*Lactobacillaceae*	*Lactobacillus*	*Lactobacillus*
3317	*Firmicutes*	*Bacilli*	*Lactobacillales*	*Lactobacillaceae*	*Lactobacillus*	*Lactobacillus*
3318	*Firmicutes*	*Bacilli*	*Lactobacillales*	*Lactobacillaceae*	*Lactobacillus*	*Lactobacillus*
3319	*Firmicutes*	*Bacilli*	*Lactobacillales*	*Lactobacillaceae*	*Lactobacillus*	*Lactobacillus*
3320	*Firmicutes*	*Bacilli*	*Lactobacillales*	*Lactobacillaceae*	*Lactobacillus*	*Lactobacillus*
3321	*Firmicutes*	*Bacilli*	*Lactobacillales*	*Lactobacillaceae*	*Lactobacillus*	*Lactobacillus*
3322	*Firmicutes*	*Bacilli*	*Lactobacillales*	*Lactobacillaceae*	*Lactobacillus*	*Lactobacillus*
3323	*Firmicutes*	*Bacilli*	*Lactobacillales*	*Lactobacillaceae*	*Lactobacillus*	*Lactobacillus*
3324	*Firmicutes*	*Bacilli*	*Lactobacillales*	*Lactobacillaceae*	*Lactobacillus*	*Lactobacillus*
3325	*Firmicutes*	*Bacilli*	*Lactobacillales*	*Lactobacillaceae*	*Lactobacillus*	*Lactobacillus*
3326	*Firmicutes*	*Bacilli*	*Lactobacillales*	*Lactobacillaceae*	*Lactobacillus*	*Lactobacillus*
3327	*Firmicutes*	*Bacilli*	*Lactobacillales*	*Lactobacillaceae*	*Lactobacillus*	*Lactobacillus*
3328	*Firmicutes*	*Bacilli*	*Lactobacillales*	*Lactobacillaceae*	*Lactobacillus*	*Lactobacillus*
3329	*Firmicutes*	*Bacilli*	*Lactobacillales*	*Lactobacillaceae*	*Lactobacillus*	*Lactobacillus*
3330	*Firmicutes*	*Bacilli*	*Lactobacillales*	*Lactobacillaceae*	*Lactobacillus*	*Lactobacillus*
3331	*Firmicutes*	*Bacilli*	*Lactobacillales*	*Lactobacillaceae*	*Lactobacillus*	*Lactobacillus*
3332	*Firmicutes*	*Bacilli*	*Lactobacillales*	*Lactobacillaceae*	*Lactobacillus*	*Lactobacillus*

Table S4. Isolated strains by culturomics

bacteriaId	*phylum*	*class*	*order*	*family*	*genus*	*species*
3333	*Firmicutes*	*Bacilli*	*Lactobacillales*	*Lactobacillaceae*	*Lactobacillus*	*Lactobacillus*
3334	*Firmicutes*	*Bacilli*	*Lactobacillales*	*Lactobacillaceae*	*Lactobacillus*	*Lactobacillus*
3335	*Firmicutes*	*Bacilli*	*Lactobacillales*	*Lactobacillaceae*	*Lactobacillus*	*Lactobacillus*
3336	*Firmicutes*	*Bacilli*	*Lactobacillales*	*Lactobacillaceae*	*Lactobacillus*	*Lactobacillus*
3337	*Firmicutes*	*Bacilli*	*Lactobacillales*	*Lactobacillaceae*	*Lactobacillus*	*Lactobacillus*
3338	*Firmicutes*	*Bacilli*	*Lactobacillales*	*Lactobacillaceae*	*Lactobacillus*	*Lactobacillus*
3339	*Firmicutes*	*Bacilli*	*Lactobacillales*	*Lactobacillaceae*	*Lactobacillus*	*Lactobacillus*
3340	*Firmicutes*	*Bacilli*	*Lactobacillales*	*Lactobacillaceae*	*Lactobacillus*	*Lactobacillus*
3341	*Firmicutes*	*Bacilli*	*Lactobacillales*	*Lactobacillaceae*	*Lactobacillus*	*Lactobacillus*
3342	*Firmicutes*	*Bacilli*	*Lactobacillales*	*Lactobacillaceae*	*Lactobacillus*	*Lactobacillus*
3343	*Firmicutes*	*Bacilli*	*Lactobacillales*	*Lactobacillaceae*	*Lactobacillus*	*Lactobacillus*
3344	*Firmicutes*	*Bacilli*	*Lactobacillales*	*Lactobacillaceae*	*Lactobacillus*	*Lactobacillus*
3345	*Firmicutes*	*Bacilli*	*Lactobacillales*	*Lactobacillaceae*	*Lactobacillus*	*Lactobacillus*
3346	*Firmicutes*	*Bacilli*	*Lactobacillales*	*Lactobacillaceae*	*Lactobacillus*	*Lactobacillus*
3347	*Firmicutes*	*Bacilli*	*Lactobacillales*	*Lactobacillaceae*	*Lactobacillus*	*Lactobacillus*
3348	*Firmicutes*	*Bacilli*	*Lactobacillales*	*Lactobacillaceae*	*Lactobacillus*	*Lactobacillus*
3349	*Firmicutes*	*Bacilli*	*Lactobacillales*	*Lactobacillaceae*	*Lactobacillus*	*Lactobacillus*
3350	*Firmicutes*	*Bacilli*	*Lactobacillales*	*Lactobacillaceae*	*Lactobacillus*	*Lactobacillus*
3351	*Firmicutes*	*Bacilli*	*Lactobacillales*	*Lactobacillaceae*	*Lactobacillus*	*Lactobacillus*
3352	*Firmicutes*	*Bacilli*	*Lactobacillales*	*Lactobacillaceae*	*Lactobacillus*	*Lactobacillus*
3353	*Firmicutes*	*Bacilli*	*Lactobacillales*	*Lactobacillaceae*	*Lactobacillus*	*Lactobacillus*
3354	*Firmicutes*	*Bacilli*	*Lactobacillales*	*Lactobacillaceae*	*Lactobacillus*	*Lactobacillus*
3355	*Firmicutes*	*Bacilli*	*Lactobacillales*	*Lactobacillaceae*	*Lactobacillus*	*Lactobacillus*
3356	*Firmicutes*	*Bacilli*	*Lactobacillales*	*Lactobacillaceae*	*Lactobacillus*	*Lactobacillus*
3357	*Firmicutes*	*Bacilli*	*Lactobacillales*	*Lactobacillaceae*	*Lactobacillus*	*Lactobacillus*
3358	*Firmicutes*	*Bacilli*	*Lactobacillales*	*Lactobacillaceae*	*Lactobacillus*	*Lactobacillus*
3359	*Firmicutes*	*Bacilli*	*Lactobacillales*	*Lactobacillaceae*	*Lactobacillus*	*Lactobacillus*
3360	*Firmicutes*	*Bacilli*	*Lactobacillales*	*Lactobacillaceae*	*Lactobacillus*	*Lactobacillus*

Table S4. Isolated strains by culturomics

bacteriaId	*phylum*	*class*	*order*	*family*	*genus*	*species*
3361	*Firmicutes*	*Bacilli*	*Lactobacillales*	*Lactobacillaceae*	*Lactobacillus*	*Lactobacillus*
3362	*Firmicutes*	*Bacilli*	*Lactobacillales*	*Lactobacillaceae*	*Lactobacillus*	*Lactobacillus*
3363	*Firmicutes*	*Bacilli*	*Lactobacillales*	*Lactobacillaceae*	*Lactobacillus*	*Lactobacillus*
3364	*Firmicutes*	*Bacilli*	*Lactobacillales*	*Lactobacillaceae*	*Lactobacillus*	*Lactobacillus*
3365	*Firmicutes*	*Bacilli*	*Lactobacillales*	*Lactobacillaceae*	*Lactobacillus*	*Lactobacillus*
3366	*Firmicutes*	*Bacilli*	*Lactobacillales*	*Lactobacillaceae*	*Lactobacillus*	*Lactobacillus*
3367	*Firmicutes*	*Bacilli*	*Lactobacillales*	*Lactobacillaceae*	*Lactobacillus*	*Lactobacillus*
3368	*Firmicutes*	*Bacilli*	*Lactobacillales*	*Lactobacillaceae*	*Lactobacillus*	*Lactobacillus*
3369	*Firmicutes*	*Bacilli*	*Lactobacillales*	*Lactobacillaceae*	*Lactobacillus*	*Lactobacillus*
3370	*Firmicutes*	*Bacilli*	*Lactobacillales*	*Lactobacillaceae*	*Lactobacillus*	*Lactobacillus*
3371	*Firmicutes*	*Bacilli*	*Lactobacillales*	*Lactobacillaceae*	*Lactobacillus*	*Lactobacillus*
3372	*Firmicutes*	*Bacilli*	*Lactobacillales*	*Lactobacillaceae*	*Lactobacillus*	*Lactobacillus*
3373	*Firmicutes*	*Bacilli*	*Lactobacillales*	*Lactobacillaceae*	*Lactobacillus*	*Lactobacillus*
3374	*Firmicutes*	*Bacilli*	*Lactobacillales*	*Lactobacillaceae*	*Lactobacillus*	*Lactobacillus*
3375	*Firmicutes*	*Bacilli*	*Lactobacillales*	*Lactobacillaceae*	*Lactobacillus*	*Lactobacillus*
3376	*Firmicutes*	*Bacilli*	*Lactobacillales*	*Lactobacillaceae*	*Lactobacillus*	*Lactobacillus*
3377	*Firmicutes*	*Bacilli*	*Lactobacillales*	*Lactobacillaceae*	*Lactobacillus*	*Lactobacillus*
3378	*Firmicutes*	*Bacilli*	*Lactobacillales*	*Lactobacillaceae*	*Lactobacillus*	*Lactobacillus*
3379	*Firmicutes*	*Bacilli*	*Lactobacillales*	*Lactobacillaceae*	*Lactobacillus*	*Lactobacillus*
3380	*Firmicutes*	*Bacilli*	*Lactobacillales*	*Lactobacillaceae*	*Lactobacillus*	*Lactobacillus*
3381	*Firmicutes*	*Bacilli*	*Lactobacillales*	*Lactobacillaceae*	*Lactobacillus*	*Lactobacillus*
3382	*Firmicutes*	*Bacilli*	*Lactobacillales*	*Lactobacillaceae*	*Lactobacillus*	*Lactobacillus*
3383	*Firmicutes*	*Bacilli*	*Lactobacillales*	*Lactobacillaceae*	*Lactobacillus*	*Lactobacillus*
3384	*Firmicutes*	*Bacilli*	*Lactobacillales*	*Lactobacillaceae*	*Lactobacillus*	*Lactobacillus*
3385	*Firmicutes*	*Bacilli*	*Lactobacillales*	*Lactobacillaceae*	*Lactobacillus*	*Lactobacillus*
3386	*Firmicutes*	*Bacilli*	*Lactobacillales*	*Lactobacillaceae*	*Lactobacillus*	*Lactobacillus*
3387	*Firmicutes*	*Bacilli*	*Lactobacillales*	*Lactobacillaceae*	*Lactobacillus*	*Lactobacillus*
3388	*Firmicutes*	*Bacilli*	*Lactobacillales*	*Lactobacillaceae*	*Lactobacillus*	*Lactobacillus*

Table S4. Isolated strains by culturomics

bacterialId	*phylum*	*class*	*order*	*family*	*genus*	*species*
3389	*Firmicutes*	*Bacilli*	*Lactobacillales*	*Lactobacillaceae*	*Lactobacillus*	*Lactobacillus*
3390	*Firmicutes*	*Bacilli*	*Lactobacillales*	*Lactobacillaceae*	*Lactobacillus*	*Lactobacillus*
3391	*Firmicutes*	*Bacilli*	*Lactobacillales*	*Lactobacillaceae*	*Lactobacillus*	*Lactobacillus*
3392	*Firmicutes*	*Bacilli*	*Lactobacillales*	*Lactobacillaceae*	*Lactobacillus*	*Lactobacillus*
3393	*Firmicutes*	*Bacilli*	*Lactobacillales*	*Lactobacillaceae*	*Lactobacillus*	*Lactobacillus*
3394	*Firmicutes*	*Bacilli*	*Lactobacillales*	*Lactobacillaceae*	*Lactobacillus*	*Lactobacillus*
3395	*Firmicutes*	*Bacilli*	*Lactobacillales*	*Lactobacillaceae*	*Lactobacillus*	*Lactobacillus*
3396	*Firmicutes*	*Bacilli*	*Lactobacillales*	*Lactobacillaceae*	*Lactobacillus*	*Lactobacillus*
3397	*Firmicutes*	*Bacilli*	*Lactobacillales*	*Lactobacillaceae*	*Lactobacillus*	*Lactobacillus*
3398	*Firmicutes*	*Bacilli*	*Lactobacillales*	*Lactobacillaceae*	*Lactobacillus*	*Lactobacillus*
3399	*Firmicutes*	*Bacilli*	*Lactobacillales*	*Lactobacillaceae*	*Lactobacillus*	*Lactobacillus*
3400	*Firmicutes*	*Bacilli*	*Lactobacillales*	*Lactobacillaceae*	*Lactobacillus*	*Lactobacillus*
3401	*Firmicutes*	*Bacilli*	*Lactobacillales*	*Lactobacillaceae*	*Lactobacillus*	*Lactobacillus*
3402	*Firmicutes*	*Bacilli*	*Lactobacillales*	*Lactobacillaceae*	*Lactobacillus*	*Lactobacillus*
3403	*Firmicutes*	*Bacilli*	*Lactobacillales*	*Lactobacillaceae*	*Lactobacillus*	*Lactobacillus*
3404	*Firmicutes*	*Bacilli*	*Lactobacillales*	*Lactobacillaceae*	*Lactobacillus*	*Lactobacillus*
3405	*Firmicutes*	*Bacilli*	*Lactobacillales*	*Lactobacillaceae*	*Lactobacillus*	*Lactobacillus*
3406	*Firmicutes*	*Bacilli*	*Lactobacillales*	*Lactobacillaceae*	*Lactobacillus*	*Lactobacillus*
3407	*Firmicutes*	*Bacilli*	*Lactobacillales*	*Lactobacillaceae*	*Lactobacillus*	*Lactobacillus*
3408	*Firmicutes*	*Bacilli*	*Lactobacillales*	*Lactobacillaceae*	*Lactobacillus*	*Lactobacillus*
3409	*Firmicutes*	*Bacilli*	*Lactobacillales*	*Lactobacillaceae*	*Lactobacillus*	*Lactobacillus*
3410	*Firmicutes*	*Bacilli*	*Lactobacillales*	*Lactobacillaceae*	*Lactobacillus*	*Lactobacillus*
3411	*Firmicutes*	*Bacilli*	*Lactobacillales*	*Lactobacillaceae*	*Lactobacillus*	*Lactobacillus*
3412	*Firmicutes*	*Bacilli*	*Lactobacillales*	*Lactobacillaceae*	*Lactobacillus*	*Lactobacillus*
3413	*Firmicutes*	*Bacilli*	*Lactobacillales*	*Lactobacillaceae*	*Lactobacillus*	*Lactobacillus*
3414	*Firmicutes*	*Bacilli*	*Lactobacillales*	*Lactobacillaceae*	*Lactobacillus*	*Lactobacillus*
3415	*Firmicutes*	*Bacilli*	*Lactobacillales*	*Lactobacillaceae*	*Lactobacillus*	*Lactobacillus*
3416	*Firmicutes*	*Bacilli*	*Lactobacillales*	*Lactobacillaceae*	*Lactobacillus*	*Lactobacillus*

Table S4. Isolated strains by culturomics

bacteriaId	*phylum*	*class*	*order*	*family*	*genus*	*species*
3417	*Firmicutes*	*Bacilli*	*Lactobacillales*	*Lactobacillaceae*	*Lactobacillus*	*Lactobacillus*
3418	*Firmicutes*	*Bacilli*	*Lactobacillales*	*Lactobacillaceae*	*Lactobacillus*	*Lactobacillus*
3419	*Firmicutes*	*Bacilli*	*Lactobacillales*	*Lactobacillaceae*	*Lactobacillus*	*Lactobacillus*
3420	*Firmicutes*	*Bacilli*	*Lactobacillales*	*Lactobacillaceae*	*Lactobacillus*	*Lactobacillus*
3421	*Firmicutes*	*Bacilli*	*Lactobacillales*	*Lactobacillaceae*	*Lactobacillus*	*Lactobacillus*
3422	*Firmicutes*	*Bacilli*	*Lactobacillales*	*Lactobacillaceae*	*Lactobacillus*	*Lactobacillus*
3423	*Firmicutes*	*Bacilli*	*Lactobacillales*	*Lactobacillaceae*	*Lactobacillus*	*Lactobacillus*
3424	*Firmicutes*	*Bacilli*	*Lactobacillales*	*Lactobacillaceae*	*Lactobacillus*	*Lactobacillus*
3425	*Firmicutes*	*Bacilli*	*Lactobacillales*	*Lactobacillaceae*	*Lactobacillus*	*Lactobacillus*
3426	*Firmicutes*	*Bacilli*	*Lactobacillales*	*Lactobacillaceae*	*Lactobacillus*	*Lactobacillus*
3427	*Firmicutes*	*Bacilli*	*Lactobacillales*	*Lactobacillaceae*	*Lactobacillus*	*Lactobacillus*
3428	*Firmicutes*	*Bacilli*	*Lactobacillales*	*Lactobacillaceae*	*Lactobacillus*	*Lactobacillus*
3429	*Firmicutes*	*Bacilli*	*Lactobacillales*	*Lactobacillaceae*	*Lactobacillus*	*Lactobacillus*
3430	*Firmicutes*	*Bacilli*	*Lactobacillales*	*Lactobacillaceae*	*Lactobacillus*	*Lactobacillus*
3431	*Firmicutes*	*Bacilli*	*Lactobacillales*	*Lactobacillaceae*	*Lactobacillus*	*Lactobacillus*
3432	*Firmicutes*	*Bacilli*	*Lactobacillales*	*Lactobacillaceae*	*Lactobacillus*	*Lactobacillus*
3433	*Firmicutes*	*Bacilli*	*Lactobacillales*	*Lactobacillaceae*	*Lactobacillus*	*Lactobacillus*
3434	*Firmicutes*	*Bacilli*	*Lactobacillales*	*Lactobacillaceae*	*Lactobacillus*	*Lactobacillus*
3435	*Firmicutes*	*Bacilli*	*Lactobacillales*	*Lactobacillaceae*	*Lactobacillus*	*Lactobacillus*
3436	*Firmicutes*	*Bacilli*	*Lactobacillales*	*Lactobacillaceae*	*Lactobacillus*	*Lactobacillus*
3437	*Firmicutes*	*Bacilli*	*Lactobacillales*	*Lactobacillaceae*	*Lactobacillus*	*Lactobacillus*
3438	*Firmicutes*	*Bacilli*	*Lactobacillales*	*Lactobacillaceae*	*Lactobacillus*	*Lactobacillus*
3439	*Firmicutes*	*Bacilli*	*Lactobacillales*	*Lactobacillaceae*	*Lactobacillus*	*Lactobacillus*
3440	*Firmicutes*	*Bacilli*	*Lactobacillales*	*Lactobacillaceae*	*Lactobacillus*	*Lactobacillus*
3441	*Firmicutes*	*Bacilli*	*Lactobacillales*	*Lactobacillaceae*	*Lactobacillus*	*Lactobacillus*
3442	*Firmicutes*	*Bacilli*	*Lactobacillales*	*Lactobacillaceae*	*Lactobacillus*	*Lactobacillus*
3443	*Firmicutes*	*Bacilli*	*Lactobacillales*	*Lactobacillaceae*	*Lactobacillus*	*Lactobacillus*
3444	*Firmicutes*	*Bacilli*	*Lactobacillales*	*Lactobacillaceae*	*Lactobacillus*	*Lactobacillus*

Table S4. Isolated strains by culturomics

bacteriaId	*phylum*	*class*	*order*	*family*	*genus*	*species*
3445	*Firmicutes*	*Bacilli*	*Lactobacillales*	*Lactobacillaceae*	*Lactobacillus*	*Lactobacillus*
3446	*Firmicutes*	*Bacilli*	*Lactobacillales*	*Lactobacillaceae*	*Lactobacillus*	*Lactobacillus*
3447	*Firmicutes*	*Bacilli*	*Lactobacillales*	*Lactobacillaceae*	*Lactobacillus*	*Lactobacillus*
3448	*Firmicutes*	*Bacilli*	*Lactobacillales*	*Lactobacillaceae*	*Lactobacillus*	*Lactobacillus*
3449	*Firmicutes*	*Bacilli*	*Lactobacillales*	*Lactobacillaceae*	*Lactobacillus*	*Lactobacillus*
3450	*Firmicutes*	*Bacilli*	*Lactobacillales*	*Lactobacillaceae*	*Lactobacillus*	*Lactobacillus*
3451	*Firmicutes*	*Bacilli*	*Lactobacillales*	*Lactobacillaceae*	*Lactobacillus*	*Lactobacillus*
3452	*Firmicutes*	*Bacilli*	*Lactobacillales*	*Lactobacillaceae*	*Lactobacillus*	*Lactobacillus*
3453	*Firmicutes*	*Bacilli*	*Lactobacillales*	*Lactobacillaceae*	*Lactobacillus*	*Lactobacillus*
3454	*Firmicutes*	*Bacilli*	*Lactobacillales*	*Lactobacillaceae*	*Lactobacillus*	*Lactobacillus*
3455	*Firmicutes*	*Bacilli*	*Lactobacillales*	*Lactobacillaceae*	*Lactobacillus*	*Lactobacillus*
3456	*Firmicutes*	*Bacilli*	*Lactobacillales*	*Lactobacillaceae*	*Lactobacillus*	*Lactobacillus*
3457	*Firmicutes*	*Bacilli*	*Lactobacillales*	*Lactobacillaceae*	*Lactobacillus*	*Lactobacillus*
3458	*Firmicutes*	*Bacilli*	*Lactobacillales*	*Lactobacillaceae*	*Lactobacillus*	*Lactobacillus*
3459	*Firmicutes*	*Bacilli*	*Lactobacillales*	*Lactobacillaceae*	*Lactobacillus*	*Lactobacillus*
3460	*Firmicutes*	*Bacilli*	*Lactobacillales*	*Lactobacillaceae*	*Lactobacillus*	*Lactobacillus*
3461	*Firmicutes*	*Bacilli*	*Lactobacillales*	*Lactobacillaceae*	*Lactobacillus*	*Lactobacillus*
3462	*Firmicutes*	*Bacilli*	*Lactobacillales*	*Lactobacillaceae*	*Lactobacillus*	*Lactobacillus*
3463	*Firmicutes*	*Bacilli*	*Lactobacillales*	*Lactobacillaceae*	*Lactobacillus*	*Lactobacillus*
3464	*Firmicutes*	*Bacilli*	*Lactobacillales*	*Lactobacillaceae*	*Lactobacillus*	*Lactobacillus*
3465	*Firmicutes*	*Bacilli*	*Lactobacillales*	*Lactobacillaceae*	*Lactobacillus*	*Lactobacillus*
3466	*Firmicutes*	*Bacilli*	*Lactobacillales*	*Lactobacillaceae*	*Lactobacillus*	*Lactobacillus*
3467	*Firmicutes*	*Bacilli*	*Lactobacillales*	*Lactobacillaceae*	*Lactobacillus*	*Lactobacillus*
3468	*Firmicutes*	*Bacilli*	*Lactobacillales*	*Lactobacillaceae*	*Lactobacillus*	*Lactobacillus*
3469	*Firmicutes*	*Bacilli*	*Lactobacillales*	*Lactobacillaceae*	*Lactobacillus*	*Lactobacillus*
3470	*Firmicutes*	*Bacilli*	*Lactobacillales*	*Lactobacillaceae*	*Lactobacillus*	*Lactobacillus*
3471	*Firmicutes*	*Bacilli*	*Lactobacillales*	*Lactobacillaceae*	*Lactobacillus*	*Lactobacillus*
3472	*Firmicutes*	*Bacilli*	*Lactobacillales*	*Lactobacillaceae*	*Lactobacillus*	*Lactobacillus*

Table S4. Isolated strains by culturomics

bacteriaId	*phylum*	*class*	*order*	*family*	*genus*	*species*
3473	*Firmicutes*	*Bacilli*	*Lactobacillales*	*Lactobacillaceae*	*Lactobacillus*	*Lactobacillus*
3474	*Firmicutes*	*Bacilli*	*Lactobacillales*	*Lactobacillaceae*	*Lactobacillus*	*Lactobacillus*
3475	*Firmicutes*	*Bacilli*	*Lactobacillales*	*Lactobacillaceae*	*Lactobacillus*	*Lactobacillus*
3476	*Firmicutes*	*Bacilli*	*Lactobacillales*	*Lactobacillaceae*	*Lactobacillus*	*Lactobacillus*
3477	*Firmicutes*	*Bacilli*	*Lactobacillales*	*Lactobacillaceae*	*Lactobacillus*	*Lactobacillus*
3478	*Firmicutes*	*Bacilli*	*Lactobacillales*	*Lactobacillaceae*	*Lactobacillus*	*Lactobacillus*
3479	*Firmicutes*	*Bacilli*	*Lactobacillales*	*Lactobacillaceae*	*Lactobacillus*	*Lactobacillus*
3480	*Firmicutes*	*Bacilli*	*Lactobacillales*	*Lactobacillaceae*	*Lactobacillus*	*Lactobacillus*
3481	*Firmicutes*	*Bacilli*	*Lactobacillales*	*Lactobacillaceae*	*Lactobacillus*	*Lactobacillus*
3482	*Firmicutes*	*Bacilli*	*Lactobacillales*	*Lactobacillaceae*	*Lactobacillus*	*Lactobacillus*
3483	*Firmicutes*	*Bacilli*	*Lactobacillales*	*Lactobacillaceae*	*Lactobacillus*	*Lactobacillus*
3484	*Firmicutes*	*Bacilli*	*Lactobacillales*	*Lactobacillaceae*	*Lactobacillus*	*Lactobacillus*
3485	*Firmicutes*	*Bacilli*	*Lactobacillales*	*Lactobacillaceae*	*Lactobacillus*	*Lactobacillus*
3486	*Firmicutes*	*Bacilli*	*Lactobacillales*	*Lactobacillaceae*	*Lactobacillus*	*Lactobacillus*
3487	*Firmicutes*	*Bacilli*	*Lactobacillales*	*Lactobacillaceae*	*Lactobacillus*	*Lactobacillus*
3488	*Firmicutes*	*Bacilli*	*Lactobacillales*	*Lactobacillaceae*	*Lactobacillus*	*Lactobacillus*
3489	*Firmicutes*	*Bacilli*	*Lactobacillales*	*Lactobacillaceae*	*Lactobacillus*	*Lactobacillus*
3490	*Firmicutes*	*Bacilli*	*Lactobacillales*	*Lactobacillaceae*	*Lactobacillus*	*Lactobacillus*
3491	*Firmicutes*	*Bacilli*	*Lactobacillales*	*Lactobacillaceae*	*Lactobacillus*	*Lactobacillus*
3492	*Firmicutes*	*Bacilli*	*Lactobacillales*	*Lactobacillaceae*	*Lactobacillus*	*Lactobacillus oris*
3493	*Firmicutes*	*Bacilli*	*Lactobacillales*	*Lactobacillaceae*	*Lactobacillus*	*Lactobacillus oris*
3494	*Firmicutes*	*Bacilli*	*Lactobacillales*	*Lactobacillaceae*	*Lactobacillus*	*Lactobacillus oris*
3495	*Firmicutes*	*Bacilli*	*Lactobacillales*	*Lactobacillaceae*	*Lactobacillus*	*Lactobacillus oris*
3496	*Firmicutes*	*Bacilli*	*Lactobacillales*	*Lactobacillaceae*	*Lactobacillus*	*Lactobacillus oris*
3497	*Firmicutes*	*Bacilli*	*Lactobacillales*	*Lactobacillaceae*	*Lactobacillus*	*Lactobacillus oris*
3498	*Firmicutes*	*Bacilli*	*Lactobacillales*	*Lactobacillaceae*	*Lactobacillus*	*Lactobacillus oris*
3499	*Firmicutes*	*Bacilli*	*Lactobacillales*	*Lactobacillaceae*	*Lactobacillus*	*Lactobacillus oris*
3500	*Firmicutes*	*Bacilli*	*Lactobacillales*	*Lactobacillaceae*	*Lactobacillus*	*Lactobacillus oris*

Table S4. Isolated strains by culturomics

bacteriaId	*phylum*	*class*	*order*	*family*	*genus*	*species*
3501	*Firmicutes*	*Bacilli*	*Lactobacillales*	*Lactobacillaceae*	*Lactobacillus*	*Lactobacillus oris*
3502	*Firmicutes*	*Bacilli*	*Lactobacillales*	*Lactobacillaceae*	*Lactobacillus*	*Lactobacillus oris*
3503	*Firmicutes*	*Bacilli*	*Lactobacillales*	*Lactobacillaceae*	*Lactobacillus*	*Lactobacillus oris*
3504	*Firmicutes*	*Bacilli*	*Lactobacillales*	*Lactobacillaceae*	*Lactobacillus*	*Lactobacillus oris*
3505	*Firmicutes*	*Bacilli*	*Lactobacillales*	*Lactobacillaceae*	*Lactobacillus*	*Lactobacillus oris*
3506	*Firmicutes*	*Bacilli*	*Lactobacillales*	*Lactobacillaceae*	*Lactobacillus*	*Lactobacillus oris*
3507	*Firmicutes*	*Bacilli*	*Lactobacillales*	*Lactobacillaceae*	*Lactobacillus*	*Lactobacillus oris*
3508	*Firmicutes*	*Bacilli*	*Lactobacillales*	*Lactobacillaceae*	*Lactobacillus*	*Lactobacillus oris*
3509	*Firmicutes*	*Bacilli*	*Lactobacillales*	*Lactobacillaceae*	*Lactobacillus*	*Lactobacillus oris*
3510	*Firmicutes*	*Bacilli*	*Lactobacillales*	*Lactobacillaceae*	*Lactobacillus*	*Lactobacillus oris*
3511	*Firmicutes*	*Bacilli*	*Lactobacillales*	*Lactobacillaceae*	*Lactobacillus*	*Lactobacillus oris*
3512	*Firmicutes*	*Bacilli*	*Lactobacillales*	*Lactobacillaceae*	*Lactobacillus*	*Lactobacillus oris*
3513	*Firmicutes*	*Bacilli*	*Lactobacillales*	*Lactobacillaceae*	*Lactobacillus*	*Lactobacillus oris*
3514	*Firmicutes*	*Bacilli*	*Lactobacillales*	*Lactobacillaceae*	*Lactobacillus*	*Lactobacillus oris*
3515	*Firmicutes*	*Bacilli*	*Lactobacillales*	*Lactobacillaceae*	*Lactobacillus*	*Lactobacillus oris*
3516	*Firmicutes*	*Bacilli*	*Lactobacillales*	*Lactobacillaceae*	*Lactobacillus*	*Lactobacillus oris*
3517	*Firmicutes*	*Bacilli*	*Lactobacillales*	*Lactobacillaceae*	*Lactobacillus*	*Lactobacillus oris*
3518	*Firmicutes*	*Bacilli*	*Lactobacillales*	*Lactobacillaceae*	*Lactobacillus*	*Lactobacillus oris*
3519	*Firmicutes*	*Bacilli*	*Lactobacillales*	*Lactobacillaceae*	*Lactobacillus*	*Lactobacillus oris*
3520	*Firmicutes*	*Bacilli*	*Lactobacillales*	*Lactobacillaceae*	*Lactobacillus*	*Lactobacillus oris*
3521	*Firmicutes*	*Bacilli*	*Lactobacillales*	*Lactobacillaceae*	*Lactobacillus*	*Lactobacillus oris*
3522	*Firmicutes*	*Bacilli*	*Lactobacillales*	*Lactobacillaceae*	*Lactobacillus*	*Lactobacillus oris*
3523	*Firmicutes*	*Bacilli*	*Lactobacillales*	*Lactobacillaceae*	*Lactobacillus*	*Lactobacillus oris*
3524	*Firmicutes*	*Bacilli*	*Lactobacillales*	*Lactobacillaceae*	*Lactobacillus*	*Lactobacillus oris*
3525	*Firmicutes*	*Bacilli*	*Lactobacillales*	*Lactobacillaceae*	*Lactobacillus*	*Lactobacillus oris*
3526	*Firmicutes*	*Bacilli*	*Lactobacillales*	*Lactobacillaceae*	*Lactobacillus*	*Lactobacillus oris*
3527	*Firmicutes*	*Bacilli*	*Lactobacillales*	*Lactobacillaceae*	*Lactobacillus*	*Lactobacillus oris*
3528	*Firmicutes*	*Bacilli*	*Lactobacillales*	*Lactobacillaceae*	*Lactobacillus*	*Lactobacillus oris*

Table S4. Isolated strains by culturomics

bacteriaId	*phylum*	*class*	*order*	*family*	*genus*	*species*
3529	*Firmicutes*	*Bacilli*	*Lactobacillales*	*Lactobacillaceae*	*Lactobacillus*	*Lactobacillus oris*
3530	*Firmicutes*	*Bacilli*	*Lactobacillales*	*Lactobacillaceae*	*Lactobacillus*	*Lactobacillus oris*
3531	*Firmicutes*	*Bacilli*	*Lactobacillales*	*Lactobacillaceae*	*Lactobacillus*	*Lactobacillus*
3532	*Firmicutes*	*Bacilli*	*Lactobacillales*	*Lactobacillaceae*	*Lactobacillus*	*Lactobacillus*
3533	*Firmicutes*	*Bacilli*	*Lactobacillales*	*Lactobacillaceae*	*Lactobacillus*	*Lactobacillus*
3534	*Firmicutes*	*Bacilli*	*Lactobacillales*	*Lactobacillaceae*	*Lactobacillus*	*Lactobacillus*
3535	*Firmicutes*	*Bacilli*	*Lactobacillales*	*Lactobacillaceae*	*Lactobacillus*	*Lactobacillus*
3536	*Firmicutes*	*Bacilli*	*Lactobacillales*	*Lactobacillaceae*	*Lactobacillus*	*Lactobacillus*
3537	*Firmicutes*	*Bacilli*	*Lactobacillales*	*Lactobacillaceae*	*Lactobacillus*	*Lactobacillus*
3538	*Firmicutes*	*Bacilli*	*Lactobacillales*	*Lactobacillaceae*	*Lactobacillus*	*Lactobacillus*
3539	*Firmicutes*	*Bacilli*	*Lactobacillales*	*Lactobacillaceae*	*Lactobacillus*	*Lactobacillus*
3540	*Firmicutes*	*Bacilli*	*Lactobacillales*	*Lactobacillaceae*	*Lactobacillus*	*Lactobacillus*
3541	*Firmicutes*	*Bacilli*	*Lactobacillales*	*Lactobacillaceae*	*Lactobacillus*	*Lactobacillus*
3542	*Firmicutes*	*Bacilli*	*Lactobacillales*	*Lactobacillaceae*	*Lactobacillus*	*Lactobacillus*
3543	*Firmicutes*	*Bacilli*	*Lactobacillales*	*Lactobacillaceae*	*Lactobacillus*	*Lactobacillus*
3544	*Firmicutes*	*Bacilli*	*Lactobacillales*	*Lactobacillaceae*	*Lactobacillus*	*Lactobacillus*
3545	*Firmicutes*	*Bacilli*	*Lactobacillales*	*Lactobacillaceae*	*Lactobacillus*	*Lactobacillus*
3546	*Firmicutes*	*Bacilli*	*Lactobacillales*	*Lactobacillaceae*	*Lactobacillus*	*Lactobacillus*
3547	*Firmicutes*	*Bacilli*	*Lactobacillales*	*Lactobacillaceae*	*Lactobacillus*	*Lactobacillus*
3548	*Firmicutes*	*Bacilli*	*Lactobacillales*	*Lactobacillaceae*	*Lactobacillus*	*Lactobacillus*
3549	*Firmicutes*	*Bacilli*	*Lactobacillales*	*Lactobacillaceae*	*Lactobacillus*	*Lactobacillus*
3550	*Firmicutes*	*Bacilli*	*Lactobacillales*	*Lactobacillaceae*	*Lactobacillus*	*Lactobacillus*
3551	*Firmicutes*	*Bacilli*	*Lactobacillales*	*Lactobacillaceae*	*Lactobacillus*	*Lactobacillus*
3552	*Firmicutes*	*Bacilli*	*Lactobacillales*	*Lactobacillaceae*	*Lactobacillus*	*Lactobacillus*
3553	*Firmicutes*	*Bacilli*	*Lactobacillales*	*Lactobacillaceae*	*Lactobacillus*	*Lactobacillus*
3554	*Firmicutes*	*Bacilli*	*Lactobacillales*	*Lactobacillaceae*	*Lactobacillus*	*Lactobacillus*
3555	*Firmicutes*	*Bacilli*	*Lactobacillales*	*Lactobacillaceae*	*Lactobacillus*	*Lactobacillus*
3556	*Firmicutes*	*Bacilli*	*Lactobacillales*	*Lactobacillaceae*	*Lactobacillus*	*Lactobacillus*

Table S4. Isolated strains by culturomics

bacteriaId	*phylum*	*class*	*order*	*family*	*genus*	*species*
3557	*Firmicutes*	*Bacilli*	*Lactobacillales*	*Lactobacillaceae*	*Lactobacillus*	*Lactobacillus*
3558	*Firmicutes*	*Bacilli*	*Lactobacillales*	*Lactobacillaceae*	*Lactobacillus*	*Lactobacillus*
3559	*Firmicutes*	*Bacilli*	*Lactobacillales*	*Lactobacillaceae*	*Lactobacillus*	*Lactobacillus*
3560	*Firmicutes*	*Bacilli*	*Lactobacillales*	*Lactobacillaceae*	*Lactobacillus*	*Lactobacillus*
3561	*Firmicutes*	*Bacilli*	*Lactobacillales*	*Lactobacillaceae*	*Lactobacillus*	*Lactobacillus*
3562	*Firmicutes*	*Bacilli*	*Lactobacillales*	*Lactobacillaceae*	*Lactobacillus*	*Lactobacillus*
3563	*Firmicutes*	*Bacilli*	*Lactobacillales*	*Lactobacillaceae*	*Lactobacillus*	*Lactobacillus*
3564	*Firmicutes*	*Bacilli*	*Lactobacillales*	*Lactobacillaceae*	*Lactobacillus*	*Lactobacillus*
3565	*Firmicutes*	*Bacilli*	*Lactobacillales*	*Lactobacillaceae*	*Lactobacillus*	*Lactobacillus*
3566	*Firmicutes*	*Bacilli*	*Lactobacillales*	*Lactobacillaceae*	*Lactobacillus*	*Lactobacillus*
3567	*Firmicutes*	*Bacilli*	*Lactobacillales*	*Lactobacillaceae*	*Lactobacillus*	*Lactobacillus*
3568	*Firmicutes*	*Bacilli*	*Lactobacillales*	*Lactobacillaceae*	*Lactobacillus*	*Lactobacillus*
3569	*Firmicutes*	*Bacilli*	*Lactobacillales*	*Lactobacillaceae*	*Lactobacillus*	*Lactobacillus*
3570	*Firmicutes*	*Bacilli*	*Lactobacillales*	*Lactobacillaceae*	*Lactobacillus*	*Lactobacillus*
3571	*Firmicutes*	*Bacilli*	*Lactobacillales*	*Lactobacillaceae*	*Lactobacillus*	*Lactobacillus*
3572	*Firmicutes*	*Bacilli*	*Lactobacillales*	*Lactobacillaceae*	*Lactobacillus*	*Lactobacillus*
3573	*Firmicutes*	*Bacilli*	*Lactobacillales*	*Lactobacillaceae*	*Lactobacillus*	*Lactobacillus*
3574	*Firmicutes*	*Bacilli*	*Lactobacillales*	*Lactobacillaceae*	*Lactobacillus*	*Lactobacillus*
3575	*Firmicutes*	*Bacilli*	*Lactobacillales*	*Lactobacillaceae*	*Lactobacillus*	*Lactobacillus*
3576	*Firmicutes*	*Bacilli*	*Lactobacillales*	*Lactobacillaceae*	*Lactobacillus*	*Lactobacillus*
3577	*Firmicutes*	*Bacilli*	*Lactobacillales*	*Lactobacillaceae*	*Lactobacillus*	*Lactobacillus*
3578	*Firmicutes*	*Bacilli*	*Lactobacillales*	*Lactobacillaceae*	*Lactobacillus*	*Lactobacillus*
3579	*Firmicutes*	*Bacilli*	*Lactobacillales*	*Lactobacillaceae*	*Lactobacillus*	*Lactobacillus*
3580	*Firmicutes*	*Bacilli*	*Lactobacillales*	*Lactobacillaceae*	*Lactobacillus*	*Lactobacillus*
3581	*Firmicutes*	*Bacilli*	*Lactobacillales*	*Lactobacillaceae*	*Lactobacillus*	*Lactobacillus*
3582	*Firmicutes*	*Bacilli*	*Lactobacillales*	*Lactobacillaceae*	*Lactobacillus*	*Lactobacillus*
3583	*Firmicutes*	*Bacilli*	*Lactobacillales*	*Lactobacillaceae*	*Lactobacillus*	*Lactobacillus*
3584	*Firmicutes*	*Bacilli*	*Lactobacillales*	*Lactobacillaceae*	*Lactobacillus*	*Lactobacillus*

Table S4. Isolated strains by culturomics

bacteriaId	*phylum*	*class*	*order*	*family*	*genus*	*species*
3585	*Firmicutes*	*Bacilli*	*Lactobacillales*	*Lactobacillaceae*	*Lactobacillus*	*Lactobacillus*
3586	*Firmicutes*	*Bacilli*	*Lactobacillales*	*Lactobacillaceae*	*Lactobacillus*	*Lactobacillus*
3587	*Firmicutes*	*Bacilli*	*Lactobacillales*	*Lactobacillaceae*	*Lactobacillus*	*Lactobacillus*
3588	*Firmicutes*	*Bacilli*	*Lactobacillales*	*Lactobacillaceae*	*Lactobacillus*	*Lactobacillus*
3589	*Firmicutes*	*Bacilli*	*Lactobacillales*	*Lactobacillaceae*	*Lactobacillus*	*Lactobacillus*
3590	*Firmicutes*	*Bacilli*	*Lactobacillales*	*Lactobacillaceae*	*Lactobacillus*	*Lactobacillus*
3591	*Firmicutes*	*Bacilli*	*Lactobacillales*	*Lactobacillaceae*	*Lactobacillus*	*Lactobacillus*
3592	*Firmicutes*	*Bacilli*	*Lactobacillales*	*Lactobacillaceae*	*Lactobacillus*	*Lactobacillus*
3593	*Firmicutes*	*Bacilli*	*Lactobacillales*	*Lactobacillaceae*	*Lactobacillus*	*Lactobacillus*
3594	*Firmicutes*	*Bacilli*	*Lactobacillales*	*Lactobacillaceae*	*Lactobacillus*	*Lactobacillus*
3595	*Firmicutes*	*Bacilli*	*Lactobacillales*	*Lactobacillaceae*	*Lactobacillus*	*Lactobacillus*
3596	*Firmicutes*	*Bacilli*	*Lactobacillales*	*Lactobacillaceae*	*Lactobacillus*	*Lactobacillus*
3597	*Firmicutes*	*Bacilli*	*Lactobacillales*	*Lactobacillaceae*	*Lactobacillus*	*Lactobacillus*
3598	*Firmicutes*	*Bacilli*	*Lactobacillales*	*Lactobacillaceae*	*Lactobacillus*	*Lactobacillus*
3599	*Firmicutes*	*Bacilli*	*Lactobacillales*	*Lactobacillaceae*	*Lactobacillus*	*Lactobacillus*
3600	*Firmicutes*	*Bacilli*	*Lactobacillales*	*Lactobacillaceae*	*Lactobacillus*	*Lactobacillus*
3601	*Firmicutes*	*Bacilli*	*Lactobacillales*	*Lactobacillaceae*	*Lactobacillus*	*Lactobacillus*
3602	*Firmicutes*	*Bacilli*	*Lactobacillales*	*Lactobacillaceae*	*Lactobacillus*	*Lactobacillus*
3603	*Firmicutes*	*Bacilli*	*Lactobacillales*	*Lactobacillaceae*	*Lactobacillus*	*Lactobacillus*
3604	*Firmicutes*	*Bacilli*	*Lactobacillales*	*Lactobacillaceae*	*Lactobacillus*	*Lactobacillus*
3605	*Firmicutes*	*Bacilli*	*Lactobacillales*	*Lactobacillaceae*	*Lactobacillus*	*Lactobacillus*
3606	*Firmicutes*	*Bacilli*	*Lactobacillales*	*Lactobacillaceae*	*Lactobacillus*	*Lactobacillus*
3607	*Firmicutes*	*Bacilli*	*Lactobacillales*	*Lactobacillaceae*	*Lactobacillus*	*Lactobacillus*
3608	*Firmicutes*	*Bacilli*	*Lactobacillales*	*Lactobacillaceae*	*Lactobacillus*	*Lactobacillus*
3609	*Firmicutes*	*Bacilli*	*Lactobacillales*	*Lactobacillaceae*	*Lactobacillus*	*Lactobacillus*
3610	*Firmicutes*	*Bacilli*	*Lactobacillales*	*Lactobacillaceae*	*Lactobacillus*	*Lactobacillus*
3611	*Firmicutes*	*Bacilli*	*Lactobacillales*	*Lactobacillaceae*	*Lactobacillus*	*Lactobacillus*
3612	*Firmicutes*	*Bacilli*	*Lactobacillales*	*Lactobacillaceae*	*Lactobacillus*	*Lactobacillus*

Table S4. Isolated strains by culturomics

bacteriaId	*phylum*	*class*	*order*	*family*	*genus*	*species*
3613	*Firmicutes*	*Bacilli*	*Lactobacillales*	*Lactobacillaceae*	*Lactobacillus*	*Lactobacillus*
3614	*Firmicutes*	*Bacilli*	*Lactobacillales*	*Lactobacillaceae*	*Lactobacillus*	*Lactobacillus*
3615	*Firmicutes*	*Bacilli*	*Lactobacillales*	*Lactobacillaceae*	*Lactobacillus*	*Lactobacillus*
3616	*Firmicutes*	*Bacilli*	*Lactobacillales*	*Lactobacillaceae*	*Lactobacillus*	*Lactobacillus*
3617	*Firmicutes*	*Bacilli*	*Lactobacillales*	*Lactobacillaceae*	*Lactobacillus*	*Lactobacillus*
3618	*Firmicutes*	*Bacilli*	*Lactobacillales*	*Lactobacillaceae*	*Lactobacillus*	*Lactobacillus*
3619	*Firmicutes*	*Bacilli*	*Lactobacillales*	*Lactobacillaceae*	*Lactobacillus*	*Lactobacillus*
3620	*Firmicutes*	*Bacilli*	*Lactobacillales*	*Lactobacillaceae*	*Lactobacillus*	*Lactobacillus*
3621	*Firmicutes*	*Bacilli*	*Lactobacillales*	*Lactobacillaceae*	*Lactobacillus*	*Lactobacillus*
3622	*Firmicutes*	*Bacilli*	*Lactobacillales*	*Lactobacillaceae*	*Lactobacillus*	*Lactobacillus*
3623	*Firmicutes*	*Bacilli*	*Lactobacillales*	*Lactobacillaceae*	*Lactobacillus*	*Lactobacillus*
3624	*Firmicutes*	*Bacilli*	*Lactobacillales*	*Lactobacillaceae*	*Lactobacillus*	*Lactobacillus*
3625	*Firmicutes*	*Bacilli*	*Lactobacillales*	*Lactobacillaceae*	*Lactobacillus*	*Lactobacillus*
3626	*Firmicutes*	*Bacilli*	*Lactobacillales*	*Lactobacillaceae*	*Lactobacillus*	*Lactobacillus*
3627	*Firmicutes*	*Bacilli*	*Lactobacillales*	*Lactobacillaceae*	*Lactobacillus*	*Lactobacillus*
3628	*Firmicutes*	*Bacilli*	*Lactobacillales*	*Lactobacillaceae*	*Lactobacillus*	*Lactobacillus*
3629	*Firmicutes*	*Bacilli*	*Lactobacillales*	*Lactobacillaceae*	*Lactobacillus*	*Lactobacillus*
3630	*Firmicutes*	*Bacilli*	*Lactobacillales*	*Lactobacillaceae*	*Lactobacillus*	*Lactobacillus*
3631	*Firmicutes*	*Bacilli*	*Lactobacillales*	*Lactobacillaceae*	*Lactobacillus*	*Lactobacillus*
3632	*Firmicutes*	*Bacilli*	*Lactobacillales*	*Lactobacillaceae*	*Lactobacillus*	*Lactobacillus*
3633	*Firmicutes*	*Bacilli*	*Lactobacillales*	*Lactobacillaceae*	*Lactobacillus*	*Lactobacillus*
3634	*Firmicutes*	*Bacilli*	*Lactobacillales*	*Lactobacillaceae*	*Lactobacillus*	*Lactobacillus*
3635	*Firmicutes*	*Bacilli*	*Lactobacillales*	*Lactobacillaceae*	*Lactobacillus*	*Lactobacillus*
3636	*Firmicutes*	*Bacilli*	*Lactobacillales*	*Lactobacillaceae*	*Lactobacillus*	*Lactobacillus*
3637	*Firmicutes*	*Bacilli*	*Lactobacillales*	*Lactobacillaceae*	*Lactobacillus*	*Lactobacillus*
3638	*Firmicutes*	*Bacilli*	*Lactobacillales*	*Lactobacillaceae*	*Lactobacillus*	*Lactobacillus*
3639	*Firmicutes*	*Bacilli*	*Lactobacillales*	*Lactobacillaceae*	*Lactobacillus*	*Lactobacillus*
3640	*Firmicutes*	*Bacilli*	*Lactobacillales*	*Lactobacillaceae*	*Lactobacillus*	*Lactobacillus*

Table S4. Isolated strains by culturomics

bacteriaId	*phylum*	*class*	*order*	*family*	*genus*	*species*
3641	*Firmicutes*	*Bacilli*	*Lactobacillales*	*Lactobacillaceae*	*Lactobacillus*	*Lactobacillus*
3642	*Firmicutes*	*Bacilli*	*Lactobacillales*	*Lactobacillaceae*	*Lactobacillus*	*Lactobacillus*
3643	*Firmicutes*	*Bacilli*	*Lactobacillales*	*Lactobacillaceae*	*Lactobacillus*	*Lactobacillus*
3644	*Firmicutes*	*Bacilli*	*Lactobacillales*	*Lactobacillaceae*	*Lactobacillus*	*Lactobacillus*
3645	*Firmicutes*	*Bacilli*	*Lactobacillales*	*Lactobacillaceae*	*Lactobacillus*	*Lactobacillus*
3646	*Firmicutes*	*Bacilli*	*Lactobacillales*	*Lactobacillaceae*	*Lactobacillus*	*Lactobacillus*
3647	*Firmicutes*	*Bacilli*	*Lactobacillales*	*Lactobacillaceae*	*Lactobacillus*	*Lactobacillus*
3648	*Firmicutes*	*Bacilli*	*Lactobacillales*	*Lactobacillaceae*	*Lactobacillus*	*Lactobacillus*
3649	*Firmicutes*	*Bacilli*	*Lactobacillales*	*Lactobacillaceae*	*Lactobacillus*	*Lactobacillus*
3650	*Firmicutes*	*Bacilli*	*Lactobacillales*	*Lactobacillaceae*	*Lactobacillus*	*Lactobacillus*
3651	*Firmicutes*	*Bacilli*	*Lactobacillales*	*Lactobacillaceae*	*Lactobacillus*	*Lactobacillus*
3652	*Firmicutes*	*Bacilli*	*Lactobacillales*	*Lactobacillaceae*	*Lactobacillus*	*Lactobacillus*
3653	*Firmicutes*	*Bacilli*	*Lactobacillales*	*Lactobacillaceae*	*Lactobacillus*	*Lactobacillus*
3654	*Firmicutes*	*Bacilli*	*Lactobacillales*	*Lactobacillaceae*	*Lactobacillus*	*Lactobacillus*
3655	*Firmicutes*	*Bacilli*	*Lactobacillales*	*Lactobacillaceae*	*Lactobacillus*	*Lactobacillus*
3656	*Firmicutes*	*Bacilli*	*Lactobacillales*	*Lactobacillaceae*	*Lactobacillus*	*Lactobacillus*
3657	*Firmicutes*	*Bacilli*	*Lactobacillales*	*Lactobacillaceae*	*Lactobacillus*	*Lactobacillus*
3658	*Firmicutes*	*Bacilli*	*Lactobacillales*	*Lactobacillaceae*	*Lactobacillus*	*Lactobacillus*
3659	*Firmicutes*	*Bacilli*	*Lactobacillales*	*Lactobacillaceae*	*Lactobacillus*	*Lactobacillus*
3660	*Firmicutes*	*Bacilli*	*Lactobacillales*	*Lactobacillaceae*	*Lactobacillus*	*Lactobacillus*
3661	*Firmicutes*	*Bacilli*	*Lactobacillales*	*Lactobacillaceae*	*Lactobacillus*	*Lactobacillus*
3662	*Firmicutes*	*Bacilli*	*Lactobacillales*	*Lactobacillaceae*	*Lactobacillus*	*Lactobacillus*
3663	*Firmicutes*	*Bacilli*	*Lactobacillales*	*Lactobacillaceae*	*Lactobacillus*	*Lactobacillus*
3664	*Firmicutes*	*Bacilli*	*Lactobacillales*	*Lactobacillaceae*	*Lactobacillus*	*Lactobacillus*
3665	*Firmicutes*	*Bacilli*	*Lactobacillales*	*Lactobacillaceae*	*Lactobacillus*	*Lactobacillus*
3666	*Firmicutes*	*Bacilli*	*Lactobacillales*	*Lactobacillaceae*	*Lactobacillus*	*Lactobacillus*
3667	*Firmicutes*	*Bacilli*	*Lactobacillales*	*Lactobacillaceae*	*Lactobacillus*	*Lactobacillus*
3668	*Firmicutes*	*Bacilli*	*Lactobacillales*	*Lactobacillaceae*	*Lactobacillus*	*Lactobacillus*

Table S4. Isolated strains by culturomics

bacteriaId	*phylum*	*class*	*order*	*family*	*genus*	*species*
3669	*Firmicutes*	*Bacilli*	*Lactobacillales*	*Lactobacillaceae*	*Lactobacillus*	*Lactobacillus*
3670	*Firmicutes*	*Bacilli*	*Lactobacillales*	*Lactobacillaceae*	*Lactobacillus*	*Lactobacillus*
3671	*Firmicutes*	*Bacilli*	*Lactobacillales*	*Lactobacillaceae*	*Lactobacillus*	*Lactobacillus*
3672	*Firmicutes*	*Bacilli*	*Lactobacillales*	*Lactobacillaceae*	*Lactobacillus*	*Lactobacillus*
3673	*Firmicutes*	*Bacilli*	*Lactobacillales*	*Lactobacillaceae*	*Lactobacillus*	*Lactobacillus*
3674	*Firmicutes*	*Bacilli*	*Lactobacillales*	*Lactobacillaceae*	*Lactobacillus*	*Lactobacillus*
3675	*Firmicutes*	*Bacilli*	*Lactobacillales*	*Lactobacillaceae*	*Lactobacillus*	*Lactobacillus*
3676	*Firmicutes*	*Bacilli*	*Lactobacillales*	*Lactobacillaceae*	*Lactobacillus*	*Lactobacillus*
3677	*Firmicutes*	*Bacilli*	*Lactobacillales*	*Lactobacillaceae*	*Lactobacillus*	*Lactobacillus*
3678	*Firmicutes*	*Bacilli*	*Lactobacillales*	*Lactobacillaceae*	*Lactobacillus*	*Lactobacillus*
3679	*Firmicutes*	*Bacilli*	*Lactobacillales*	*Lactobacillaceae*	*Lactobacillus*	*Lactobacillus*
3680	*Firmicutes*	*Bacilli*	*Lactobacillales*	*Lactobacillaceae*	*Lactobacillus*	*Lactobacillus*
3681	*Firmicutes*	*Bacilli*	*Lactobacillales*	*Lactobacillaceae*	*Lactobacillus*	*Lactobacillus*
3682	*Firmicutes*	*Bacilli*	*Lactobacillales*	*Lactobacillaceae*	*Lactobacillus*	*Lactobacillus*
3683	*Firmicutes*	*Bacilli*	*Lactobacillales*	*Lactobacillaceae*	*Lactobacillus*	*Lactobacillus*
3684	*Firmicutes*	*Bacilli*	*Lactobacillales*	*Lactobacillaceae*	*Lactobacillus*	*Lactobacillus*
3685	*Firmicutes*	*Bacilli*	*Lactobacillales*	*Lactobacillaceae*	*Lactobacillus*	*Lactobacillus*
3686	*Firmicutes*	*Bacilli*	*Lactobacillales*	*Lactobacillaceae*	*Lactobacillus*	*Lactobacillus*
3687	*Firmicutes*	*Bacilli*	*Lactobacillales*	*Lactobacillaceae*	*Lactobacillus*	*Lactobacillus*
3688	*Firmicutes*	*Bacilli*	*Lactobacillales*	*Lactobacillaceae*	*Lactobacillus*	*Lactobacillus*
3689	*Firmicutes*	*Bacilli*	*Lactobacillales*	*Lactobacillaceae*	*Lactobacillus*	*Lactobacillus*
3690	*Firmicutes*	*Bacilli*	*Lactobacillales*	*Lactobacillaceae*	*Lactobacillus*	*Lactobacillus*
3691	*Firmicutes*	*Bacilli*	*Lactobacillales*	*Lactobacillaceae*	*Lactobacillus*	*Lactobacillus*
3692	*Firmicutes*	*Bacilli*	*Lactobacillales*	*Lactobacillaceae*	*Lactobacillus*	*Lactobacillus*
3693	*Firmicutes*	*Bacilli*	*Lactobacillales*	*Lactobacillaceae*	*Lactobacillus*	*Lactobacillus*
3694	*Firmicutes*	*Bacilli*	*Lactobacillales*	*Lactobacillaceae*	*Lactobacillus*	*Lactobacillus*
3695	*Firmicutes*	*Bacilli*	*Lactobacillales*	*Lactobacillaceae*	*Lactobacillus*	*Lactobacillus*
3696	*Firmicutes*	*Bacilli*	*Lactobacillales*	*Lactobacillaceae*	*Lactobacillus*	*Lactobacillus*

Table S4. Isolated strains by culturomics

bacteriaId	*phylum*	*class*	*order*	*family*	*genus*	*species*
3697	*Firmicutes*	*Bacilli*	*Lactobacillales*	*Lactobacillaceae*	*Lactobacillus*	*Lactobacillus*
3698	*Firmicutes*	*Bacilli*	*Lactobacillales*	*Lactobacillaceae*	*Lactobacillus*	*Lactobacillus*
3699	*Firmicutes*	*Bacilli*	*Lactobacillales*	*Lactobacillaceae*	*Lactobacillus*	*Lactobacillus*
3700	*Firmicutes*	*Bacilli*	*Lactobacillales*	*Lactobacillaceae*	*Lactobacillus*	*Lactobacillus*
3701	*Firmicutes*	*Bacilli*	*Lactobacillales*	*Lactobacillaceae*	*Lactobacillus*	*Lactobacillus*
3702	*Firmicutes*	*Bacilli*	*Lactobacillales*	*Lactobacillaceae*	*Lactobacillus*	*Lactobacillus*
3703	*Firmicutes*	*Bacilli*	*Lactobacillales*	*Lactobacillaceae*	*Lactobacillus*	*Lactobacillus*
3704	*Firmicutes*	*Bacilli*	*Lactobacillales*	*Lactobacillaceae*	*Lactobacillus*	*Lactobacillus*
3705	*Firmicutes*	*Bacilli*	*Lactobacillales*	*Lactobacillaceae*	*Lactobacillus*	*Lactobacillus*
3706	*Firmicutes*	*Bacilli*	*Lactobacillales*	*Lactobacillaceae*	*Lactobacillus*	*Lactobacillus*
3707	*Firmicutes*	*Bacilli*	*Lactobacillales*	*Lactobacillaceae*	*Lactobacillus*	*Lactobacillus*
3708	*Firmicutes*	*Bacilli*	*Lactobacillales*	*Lactobacillaceae*	*Lactobacillus*	*Lactobacillus*
3709	*Firmicutes*	*Bacilli*	*Lactobacillales*	*Lactobacillaceae*	*Lactobacillus*	*Lactobacillus*
3710	*Firmicutes*	*Bacilli*	*Lactobacillales*	*Lactobacillaceae*	*Lactobacillus*	*Lactobacillus*
3711	*Firmicutes*	*Bacilli*	*Lactobacillales*	*Lactobacillaceae*	*Lactobacillus*	*Lactobacillus*
3712	*Firmicutes*	*Bacilli*	*Lactobacillales*	*Lactobacillaceae*	*Lactobacillus*	*Lactobacillus*
3713	*Firmicutes*	*Bacilli*	*Lactobacillales*	*Lactobacillaceae*	*Lactobacillus*	*Lactobacillus*
3714	*Firmicutes*	*Bacilli*	*Lactobacillales*	*Lactobacillaceae*	*Lactobacillus*	*Lactobacillus*
3715	*Firmicutes*	*Bacilli*	*Lactobacillales*	*Lactobacillaceae*	*Lactobacillus*	*Lactobacillus*
3716	*Firmicutes*	*Bacilli*	*Lactobacillales*	*Lactobacillaceae*	*Lactobacillus*	*Lactobacillus*
3717	*Firmicutes*	*Bacilli*	*Lactobacillales*	*Lactobacillaceae*	*Lactobacillus*	*Lactobacillus*
3718	*Firmicutes*	*Bacilli*	*Lactobacillales*	*Lactobacillaceae*	*Lactobacillus*	*Lactobacillus*
3719	*Firmicutes*	*Bacilli*	*Lactobacillales*	*Lactobacillaceae*	*Lactobacillus*	*Lactobacillus*
3720	*Firmicutes*	*Bacilli*	*Lactobacillales*	*Lactobacillaceae*	*Lactobacillus*	*Lactobacillus*
3721	*Firmicutes*	*Bacilli*	*Lactobacillales*	*Lactobacillaceae*	*Lactobacillus*	*Lactobacillus*
3722	*Firmicutes*	*Bacilli*	*Lactobacillales*	*Lactobacillaceae*	*Lactobacillus*	*Lactobacillus*
3723	*Firmicutes*	*Bacilli*	*Lactobacillales*	*Lactobacillaceae*	*Lactobacillus*	*Lactobacillus*
3724	*Firmicutes*	*Bacilli*	*Lactobacillales*	*Lactobacillaceae*	*Lactobacillus*	*Lactobacillus*

Table S4. Isolated strains by culturomics

bacteriaId	*phylum*	*class*	*order*	*family*	*genus*	*species*
3725	*Firmicutes*	*Bacilli*	*Lactobacillales*	*Lactobacillaceae*	*Lactobacillus*	*Lactobacillus*
3726	*Firmicutes*	*Bacilli*	*Lactobacillales*	*Lactobacillaceae*	*Lactobacillus*	*Lactobacillus*
3727	*Firmicutes*	*Bacilli*	*Lactobacillales*	*Lactobacillaceae*	*Lactobacillus*	*Lactobacillus*
3728	*Firmicutes*	*Bacilli*	*Lactobacillales*	*Lactobacillaceae*	*Lactobacillus*	*Lactobacillus*
3729	*Firmicutes*	*Bacilli*	*Lactobacillales*	*Lactobacillaceae*	*Lactobacillus*	*Lactobacillus*
3730	*Firmicutes*	*Bacilli*	*Lactobacillales*	*Lactobacillaceae*	*Lactobacillus*	*Lactobacillus*
3731	*Firmicutes*	*Bacilli*	*Lactobacillales*	*Lactobacillaceae*	*Lactobacillus*	*Lactobacillus*
3732	*Firmicutes*	*Bacilli*	*Lactobacillales*	*Lactobacillaceae*	*Lactobacillus*	*Lactobacillus*
3733	*Firmicutes*	*Bacilli*	*Lactobacillales*	*Lactobacillaceae*	*Lactobacillus*	*Lactobacillus*
3734	*Firmicutes*	*Bacilli*	*Lactobacillales*	*Lactobacillaceae*	*Lactobacillus*	*Lactobacillus*
3735	*Firmicutes*	*Bacilli*	*Lactobacillales*	*Lactobacillaceae*	*Lactobacillus*	*Lactobacillus*
3736	*Firmicutes*	*Bacilli*	*Lactobacillales*	*Lactobacillaceae*	*Lactobacillus*	*Lactobacillus*
3737	*Firmicutes*	*Bacilli*	*Lactobacillales*	*Lactobacillaceae*	*Lactobacillus*	*Lactobacillus*
3738	*Firmicutes*	*Bacilli*	*Lactobacillales*	*Lactobacillaceae*	*Lactobacillus*	*Lactobacillus*
3739	*Firmicutes*	*Bacilli*	*Lactobacillales*	*Lactobacillaceae*	*Lactobacillus*	*Lactobacillus*
3740	*Firmicutes*	*Bacilli*	*Lactobacillales*	*Lactobacillaceae*	*Lactobacillus*	*Lactobacillus*
3741	*Firmicutes*	*Bacilli*	*Lactobacillales*	*Lactobacillaceae*	*Lactobacillus*	*Lactobacillus*
3742	*Firmicutes*	*Bacilli*	*Lactobacillales*	*Lactobacillaceae*	*Lactobacillus*	*Lactobacillus*
3743	*Firmicutes*	*Bacilli*	*Lactobacillales*	*Lactobacillaceae*	*Lactobacillus*	*Lactobacillus*
3744	*Firmicutes*	*Bacilli*	*Lactobacillales*	*Lactobacillaceae*	*Lactobacillus*	*Lactobacillus*
3745	*Firmicutes*	*Bacilli*	*Lactobacillales*	*Lactobacillaceae*	*Lactobacillus*	*Lactobacillus*
3746	*Firmicutes*	*Bacilli*	*Lactobacillales*	*Lactobacillaceae*	*Lactobacillus*	*Lactobacillus*
3747	*Firmicutes*	*Bacilli*	*Lactobacillales*	*Lactobacillaceae*	*Lactobacillus*	*Lactobacillus*
3748	*Firmicutes*	*Bacilli*	*Lactobacillales*	*Lactobacillaceae*	*Lactobacillus*	*Lactobacillus*
3749	*Firmicutes*	*Bacilli*	*Lactobacillales*	*Lactobacillaceae*	*Lactobacillus*	*Lactobacillus*
3750	*Firmicutes*	*Bacilli*	*Lactobacillales*	*Lactobacillaceae*	*Lactobacillus*	*Lactobacillus*
3751	*Firmicutes*	*Bacilli*	*Lactobacillales*	*Lactobacillaceae*	*Lactobacillus*	*Lactobacillus*
3752	*Firmicutes*	*Bacilli*	*Lactobacillales*	*Lactobacillaceae*	*Lactobacillus*	*Lactobacillus*

Table S4. Isolated strains by culturomics

bacteriald	*phylum*	*class*	*order*	*family*	*genus*	*species*
3753	*Firmicutes*	*Bacilli*	*Lactobacillales*	*Lactobacillaceae*	*Lactobacillus*	*Lactobacillus*
3754	*Firmicutes*	*Bacilli*	*Lactobacillales*	*Lactobacillaceae*	*Lactobacillus*	*Lactobacillus*
3755	*Firmicutes*	*Bacilli*	*Lactobacillales*	*Lactobacillaceae*	*Lactobacillus*	*Lactobacillus*
3756	*Firmicutes*	*Bacilli*	*Lactobacillales*	*Lactobacillaceae*	*Lactobacillus*	*Lactobacillus*
3757	*Firmicutes*	*Bacilli*	*Lactobacillales*	*Lactobacillaceae*	*Lactobacillus*	*Lactobacillus*
3758	*Firmicutes*	*Bacilli*	*Lactobacillales*	*Lactobacillaceae*	*Lactobacillus*	*Lactobacillus*
3759	*Firmicutes*	*Bacilli*	*Lactobacillales*	*Lactobacillaceae*	*Lactobacillus*	*Lactobacillus*
3760	*Firmicutes*	*Bacilli*	*Lactobacillales*	*Lactobacillaceae*	*Lactobacillus*	*Lactobacillus*
3761	*Firmicutes*	*Bacilli*	*Lactobacillales*	*Lactobacillaceae*	*Lactobacillus*	*Lactobacillus*
3762	*Firmicutes*	*Bacilli*	*Lactobacillales*	*Lactobacillaceae*	*Lactobacillus*	*Lactobacillus*
3763	*Firmicutes*	*Bacilli*	*Lactobacillales*	*Lactobacillaceae*	*Lactobacillus*	*Lactobacillus*
3764	*Firmicutes*	*Bacilli*	*Lactobacillales*	*Lactobacillaceae*	*Lactobacillus*	*Lactobacillus*
3765	*Firmicutes*	*Bacilli*	*Lactobacillales*	*Lactobacillaceae*	*Lactobacillus*	*Lactobacillus*
3766	*Firmicutes*	*Bacilli*	*Lactobacillales*	*Lactobacillaceae*	*Lactobacillus*	*Lactobacillus*
3767	*Firmicutes*	*Bacilli*	*Lactobacillales*	*Lactobacillaceae*	*Lactobacillus*	*Lactobacillus*
3768	*Firmicutes*	*Bacilli*	*Lactobacillales*	*Lactobacillaceae*	*Lactobacillus*	*Lactobacillus*
3769	*Firmicutes*	*Bacilli*	*Lactobacillales*	*Lactobacillaceae*	*Lactobacillus*	*Lactobacillus*
3770	*Firmicutes*	*Bacilli*	*Lactobacillales*	*Lactobacillaceae*	*Lactobacillus*	*Lactobacillus*
3771	*Firmicutes*	*Bacilli*	*Lactobacillales*	*Lactobacillaceae*	*Lactobacillus*	*Lactobacillus*
3772	*Firmicutes*	*Bacilli*	*Lactobacillales*	*Lactobacillaceae*	*Lactobacillus*	*Lactobacillus*
3773	*Firmicutes*	*Bacilli*	*Lactobacillales*	*Lactobacillaceae*	*Lactobacillus*	*Lactobacillus*
3774	*Firmicutes*	*Bacilli*	*Lactobacillales*	*Lactobacillaceae*	*Lactobacillus*	*Lactobacillus*
3775	*Firmicutes*	*Bacilli*	*Lactobacillales*	*Lactobacillaceae*	*Lactobacillus*	*Lactobacillus*
3776	*Firmicutes*	*Bacilli*	*Lactobacillales*	*Lactobacillaceae*	*Lactobacillus*	*Lactobacillus*
3777	*Firmicutes*	*Bacilli*	*Lactobacillales*	*Lactobacillaceae*	*Lactobacillus*	*Lactobacillus*
3778	*Firmicutes*	*Bacilli*	*Lactobacillales*	*Lactobacillaceae*	*Lactobacillus*	*Lactobacillus*
3779	*Firmicutes*	*Bacilli*	*Lactobacillales*	*Lactobacillaceae*	*Lactobacillus*	*Lactobacillus*
3780	*Firmicutes*	*Bacilli*	*Lactobacillales*	*Lactobacillaceae*	*Lactobacillus*	*Lactobacillus*

Table S4. Isolated strains by culturomics

bacteriaId	*phylum*	*class*	*order*	*family*	*genus*	*species*
3781	*Firmicutes*	*Bacilli*	*Lactobacillales*	*Lactobacillaceae*	*Lactobacillus*	*Lactobacillus*
3782	*Firmicutes*	*Bacilli*	*Lactobacillales*	*Lactobacillaceae*	*Lactobacillus*	*Lactobacillus*
3783	*Firmicutes*	*Bacilli*	*Lactobacillales*	*Lactobacillaceae*	*Lactobacillus*	*Lactobacillus*
3784	*Firmicutes*	*Bacilli*	*Lactobacillales*	*Lactobacillaceae*	*Lactobacillus*	*Lactobacillus*
3785	*Firmicutes*	*Bacilli*	*Lactobacillales*	*Lactobacillaceae*	*Lactobacillus*	*Lactobacillus*
3786	*Firmicutes*	*Bacilli*	*Lactobacillales*	*Lactobacillaceae*	*Lactobacillus*	*Lactobacillus*
3787	*Firmicutes*	*Bacilli*	*Lactobacillales*	*Lactobacillaceae*	*Lactobacillus*	*Lactobacillus*
3788	*Firmicutes*	*Bacilli*	*Lactobacillales*	*Lactobacillaceae*	*Lactobacillus*	*Lactobacillus*
3789	*Firmicutes*	*Bacilli*	*Lactobacillales*	*Lactobacillaceae*	*Lactobacillus*	*Lactobacillus*
3790	*Firmicutes*	*Bacilli*	*Lactobacillales*	*Lactobacillaceae*	*Lactobacillus*	*Lactobacillus*
3791	*Firmicutes*	*Bacilli*	*Lactobacillales*	*Lactobacillaceae*	*Lactobacillus*	*Lactobacillus*
3792	*Firmicutes*	*Bacilli*	*Lactobacillales*	*Lactobacillaceae*	*Lactobacillus*	*Lactobacillus*
3793	*Firmicutes*	*Bacilli*	*Lactobacillales*	*Lactobacillaceae*	*Lactobacillus*	*Lactobacillus*
3794	*Firmicutes*	*Bacilli*	*Lactobacillales*	*Lactobacillaceae*	*Lactobacillus*	*Lactobacillus*
3795	*Firmicutes*	*Bacilli*	*Lactobacillales*	*Lactobacillaceae*	*Lactobacillus*	*Lactobacillus*
3796	*Firmicutes*	*Bacilli*	*Lactobacillales*	*Lactobacillaceae*	*Lactobacillus*	*Lactobacillus*
3797	*Firmicutes*	*Bacilli*	*Lactobacillales*	*Lactobacillaceae*	*Lactobacillus*	*Lactobacillus*
3798	*Firmicutes*	*Bacilli*	*Lactobacillales*	*Lactobacillaceae*	*Lactobacillus*	*Lactobacillus*
3799	*Firmicutes*	*Bacilli*	*Lactobacillales*	*Lactobacillaceae*	*Lactobacillus*	*Lactobacillus*
3800	*Firmicutes*	*Bacilli*	*Lactobacillales*	*Lactobacillaceae*	*Lactobacillus*	*Lactobacillus*
3801	*Firmicutes*	*Bacilli*	*Lactobacillales*	*Lactobacillaceae*	*Lactobacillus*	*Lactobacillus*
3802	*Firmicutes*	*Bacilli*	*Lactobacillales*	*Lactobacillaceae*	*Lactobacillus*	*Lactobacillus*
3803	*Firmicutes*	*Bacilli*	*Lactobacillales*	*Lactobacillaceae*	*Lactobacillus*	*Lactobacillus*
3804	*Firmicutes*	*Bacilli*	*Lactobacillales*	*Lactobacillaceae*	*Lactobacillus*	*Lactobacillus*
3805	*Firmicutes*	*Bacilli*	*Lactobacillales*	*Lactobacillaceae*	*Lactobacillus*	*Lactobacillus*
3806	*Firmicutes*	*Bacilli*	*Lactobacillales*	*Lactobacillaceae*	*Lactobacillus*	*Lactobacillus*
3807	*Firmicutes*	*Bacilli*	*Lactobacillales*	*Lactobacillaceae*	*Lactobacillus*	*Lactobacillus*
3808	*Firmicutes*	*Bacilli*	*Lactobacillales*	*Lactobacillaceae*	*Lactobacillus*	*Lactobacillus*

Table S4. Isolated strains by culturomics

bacteriaId	*phylum*	*class*	*order*	*family*	*genus*	*species*
3809	*Firmicutes*	*Bacilli*	*Lactobacillales*	*Lactobacillaceae*	*Lactobacillus*	*Lactobacillus*
3810	*Firmicutes*	*Bacilli*	*Lactobacillales*	*Lactobacillaceae*	*Lactobacillus*	*Lactobacillus*
3811	*Firmicutes*	*Bacilli*	*Lactobacillales*	*Lactobacillaceae*	*Lactobacillus*	*Lactobacillus*
3812	*Firmicutes*	*Bacilli*	*Lactobacillales*	*Lactobacillaceae*	*Lactobacillus*	*Lactobacillus*
3813	*Firmicutes*	*Bacilli*	*Lactobacillales*	*Lactobacillaceae*	*Lactobacillus*	*Lactobacillus*
3814	*Firmicutes*	*Bacilli*	*Lactobacillales*	*Lactobacillaceae*	*Lactobacillus*	*Lactobacillus*
3815	*Firmicutes*	*Bacilli*	*Lactobacillales*	*Lactobacillaceae*	*Lactobacillus*	*Lactobacillus*
3816	*Firmicutes*	*Bacilli*	*Lactobacillales*	*Lactobacillaceae*	*Lactobacillus*	*Lactobacillus*
3817	*Firmicutes*	*Bacilli*	*Lactobacillales*	*Lactobacillaceae*	*Lactobacillus*	*Lactobacillus*
3818	*Firmicutes*	*Bacilli*	*Lactobacillales*	*Lactobacillaceae*	*Lactobacillus*	*Lactobacillus*
3819	*Firmicutes*	*Bacilli*	*Lactobacillales*	*Lactobacillaceae*	*Lactobacillus*	*Lactobacillus*
3820	*Firmicutes*	*Bacilli*	*Lactobacillales*	*Lactobacillaceae*	*Lactobacillus*	*Lactobacillus*
3821	*Firmicutes*	*Bacilli*	*Lactobacillales*	*Lactobacillaceae*	*Lactobacillus*	*Lactobacillus*
3822	*Firmicutes*	*Bacilli*	*Lactobacillales*	*Lactobacillaceae*	*Lactobacillus*	*Lactobacillus*
3823	*Firmicutes*	*Bacilli*	*Lactobacillales*	*Lactobacillaceae*	*Lactobacillus*	*Lactobacillus*
3824	*Firmicutes*	*Bacilli*	*Lactobacillales*	*Lactobacillaceae*	*Lactobacillus*	*Lactobacillus*
3825	*Firmicutes*	*Bacilli*	*Lactobacillales*	*Lactobacillaceae*	*Lactobacillus*	*Lactobacillus*
3826	*Firmicutes*	*Bacilli*	*Lactobacillales*	*Lactobacillaceae*	*Lactobacillus*	*Lactobacillus*
3827	*Firmicutes*	*Bacilli*	*Lactobacillales*	*Lactobacillaceae*	*Lactobacillus*	*Lactobacillus*
3828	*Firmicutes*	*Bacilli*	*Lactobacillales*	*Lactobacillaceae*	*Lactobacillus*	*Lactobacillus*
3829	*Firmicutes*	*Bacilli*	*Lactobacillales*	*Lactobacillaceae*	*Lactobacillus*	*Lactobacillus*
3830	*Firmicutes*	*Bacilli*	*Lactobacillales*	*Lactobacillaceae*	*Lactobacillus*	*Lactobacillus*
3831	*Firmicutes*	*Bacilli*	*Lactobacillales*	*Lactobacillaceae*	*Lactobacillus*	*Lactobacillus*
3832	*Firmicutes*	*Bacilli*	*Lactobacillales*	*Lactobacillaceae*	*Lactobacillus*	*Lactobacillus*
3833	*Firmicutes*	*Bacilli*	*Lactobacillales*	*Lactobacillaceae*	*Lactobacillus*	*Lactobacillus*
3834	*Firmicutes*	*Bacilli*	*Lactobacillales*	*Lactobacillaceae*	*Lactobacillus*	*Lactobacillus*
3835	*Firmicutes*	*Bacilli*	*Lactobacillales*	*Lactobacillaceae*	*Lactobacillus*	*Lactobacillus*
3836	*Firmicutes*	*Bacilli*	*Lactobacillales*	*Lactobacillaceae*	*Lactobacillus*	*Lactobacillus*

Table S4. Isolated strains by culturomics

bacteriaId	*phylum*	*class*	*order*	*family*	*genus*	*species*
3837	*Firmicutes*	*Bacilli*	*Lactobacillales*	*Lactobacillaceae*	*Lactobacillus*	*Lactobacillus*
3838	*Firmicutes*	*Bacilli*	*Lactobacillales*	*Lactobacillaceae*	*Lactobacillus*	*Lactobacillus*
3839	*Firmicutes*	*Bacilli*	*Lactobacillales*	*Lactobacillaceae*	*Lactobacillus*	*Lactobacillus*
3840	*Firmicutes*	*Bacilli*	*Lactobacillales*	*Lactobacillaceae*	*Lactobacillus*	*Lactobacillus*
3841	*Firmicutes*	*Bacilli*	*Lactobacillales*	*Lactobacillaceae*	*Lactobacillus*	*Lactobacillus*
3842	*Firmicutes*	*Bacilli*	*Lactobacillales*	*Lactobacillaceae*	*Lactobacillus*	*Lactobacillus*
3843	*Firmicutes*	*Bacilli*	*Lactobacillales*	*Lactobacillaceae*	*Lactobacillus*	*Lactobacillus*
3844	*Firmicutes*	*Bacilli*	*Lactobacillales*	*Lactobacillaceae*	*Lactobacillus*	*Lactobacillus*
3845	*Firmicutes*	*Bacilli*	*Lactobacillales*	*Lactobacillaceae*	*Lactobacillus*	*Lactobacillus*
3846	*Firmicutes*	*Bacilli*	*Lactobacillales*	*Lactobacillaceae*	*Lactobacillus*	*Lactobacillus*
3847	*Firmicutes*	*Bacilli*	*Lactobacillales*	*Lactobacillaceae*	*Lactobacillus*	*Lactobacillus*
3848	*Firmicutes*	*Bacilli*	*Lactobacillales*	*Lactobacillaceae*	*Lactobacillus*	*Lactobacillus*
3849	*Firmicutes*	*Bacilli*	*Lactobacillales*	*Lactobacillaceae*	*Lactobacillus*	*Lactobacillus*
3850	*Firmicutes*	*Bacilli*	*Lactobacillales*	*Lactobacillaceae*	*Lactobacillus*	*Lactobacillus*
3851	*Firmicutes*	*Bacilli*	*Lactobacillales*	*Lactobacillaceae*	*Lactobacillus*	*Lactobacillus*
3852	*Firmicutes*	*Bacilli*	*Lactobacillales*	*Lactobacillaceae*	*Lactobacillus*	*Lactobacillus*
3853	*Firmicutes*	*Bacilli*	*Lactobacillales*	*Lactobacillaceae*	*Lactobacillus*	*Lactobacillus*
3854	*Firmicutes*	*Bacilli*	*Lactobacillales*	*Lactobacillaceae*	*Lactobacillus*	*Lactobacillus*
3855	*Firmicutes*	*Bacilli*	*Lactobacillales*	*Lactobacillaceae*	*Lactobacillus*	*Lactobacillus*
3856	*Firmicutes*	*Bacilli*	*Lactobacillales*	*Lactobacillaceae*	*Lactobacillus*	*Lactobacillus*
3857	*Firmicutes*	*Bacilli*	*Lactobacillales*	*Lactobacillaceae*	*Lactobacillus*	*Lactobacillus*
3858	*Firmicutes*	*Bacilli*	*Lactobacillales*	*Lactobacillaceae*	*Lactobacillus*	*Lactobacillus*
3859	*Firmicutes*	*Bacilli*	*Lactobacillales*	*Lactobacillaceae*	*Lactobacillus*	*Lactobacillus*
3860	*Firmicutes*	*Bacilli*	*Lactobacillales*	*Lactobacillaceae*	*Lactobacillus*	*Lactobacillus*
3861	*Firmicutes*	*Bacilli*	*Lactobacillales*	*Lactobacillaceae*	*Lactobacillus*	*Lactobacillus*
3862	*Firmicutes*	*Bacilli*	*Lactobacillales*	*Lactobacillaceae*	*Lactobacillus*	*Lactobacillus*
3863	*Firmicutes*	*Bacilli*	*Lactobacillales*	*Lactobacillaceae*	*Lactobacillus*	*Lactobacillus sakei*
3864	*Firmicutes*	*Bacilli*	*Lactobacillales*	*Lactobacillaceae*	*Lactobacillus*	*Lactobacillus*

Table S4. Isolated strains by culturomics

bacterialId	*phylum*	*class*	*order*	*family*	*genus*	*species*
3865	*Firmicutes*	*Bacilli*	*Lactobacillales*	*Lactobacillaceae*	*Lactobacillus*	*Lactobacillus*
3866	*Firmicutes*	*Bacilli*	*Lactobacillales*	*Lactobacillaceae*	*Lactobacillus*	*Lactobacillus*
3867	*Firmicutes*	*Bacilli*	*Lactobacillales*	*Lactobacillaceae*	*Lactobacillus*	*Lactobacillus*
3868	*Firmicutes*	*Bacilli*	*Lactobacillales*	*Lactobacillaceae*	*Lactobacillus*	*Lactobacillus*
3869	*Firmicutes*	*Bacilli*	*Lactobacillales*	*Lactobacillaceae*	*Lactobacillus*	*Lactobacillus*
3870	*Firmicutes*	*Bacilli*	*Lactobacillales*	*Lactobacillaceae*	*Lactobacillus*	*Lactobacillus*
3871	*Firmicutes*	*Bacilli*	*Lactobacillales*	*Lactobacillaceae*	*Lactobacillus*	*Lactobacillus*
3872	*Firmicutes*	*Bacilli*	*Lactobacillales*	*Lactobacillaceae*	*Lactobacillus*	*Lactobacillus*
3873	*Firmicutes*	*Bacilli*	*Lactobacillales*	*Lactobacillaceae*	*Lactobacillus*	*Lactobacillus*
3874	*Firmicutes*	*Bacilli*	*Lactobacillales*	*Lactobacillaceae*	*Lactobacillus*	*Lactobacillus*
3875	*Firmicutes*	*Bacilli*	*Lactobacillales*	*Lactobacillaceae*	*Lactobacillus*	*Lactobacillus*
3876	*Firmicutes*	*Bacilli*	*Lactobacillales*	*Lactobacillaceae*	*Lactobacillus*	*Lactobacillus*
3877	*Firmicutes*	*Bacilli*	*Lactobacillales*	*Lactobacillaceae*	*Lactobacillus*	*Lactobacillus*
3878	*Firmicutes*	*Bacilli*	*Lactobacillales*	*Lactobacillaceae*	*Lactobacillus*	*Lactobacillus*
3879	*Firmicutes*	*Bacilli*	*Lactobacillales*	*Lactobacillaceae*	*Lactobacillus*	*Lactobacillus*
3880	*Firmicutes*	*Bacilli*	*Lactobacillales*	*Lactobacillaceae*	*Lactobacillus*	*Lactobacillus*
3881	*Firmicutes*	*Bacilli*	*Lactobacillales*	*Lactobacillaceae*	*Lactobacillus*	*Lactobacillus*
3882	*Firmicutes*	*Bacilli*	*Lactobacillales*	*Lactobacillaceae*	*Lactobacillus*	*Lactobacillus*
3883	*Firmicutes*	*Bacilli*	*Lactobacillales*	*Lactobacillaceae*	*Lactobacillus*	*Lactobacillus*
3884	*Firmicutes*	*Bacilli*	*Lactobacillales*	*Lactobacillaceae*	*Lactobacillus*	*Lactobacillus*
3885	*Firmicutes*	*Bacilli*	*Lactobacillales*	*Lactobacillaceae*	*Lactobacillus*	*Lactobacillus*
3886	*Firmicutes*	*Bacilli*	*Lactobacillales*	*Lactobacillaceae*	*Lactobacillus*	*Lactobacillus*
3887	*Firmicutes*	*Bacilli*	*Lactobacillales*	*Lactobacillaceae*	*Lactobacillus*	*Lactobacillus*
3888	*Firmicutes*	*Bacilli*	*Lactobacillales*	*Lactobacillaceae*	*Lactobacillus*	*Lactobacillus*
3889	*Firmicutes*	*Bacilli*	*Lactobacillales*	*Lactobacillaceae*	*Lactobacillus*	*Lactobacillus*
3890	*Firmicutes*	*Bacilli*	*Lactobacillales*	*Lactobacillaceae*	*Lactobacillus*	*Lactobacillus*
3891	*Firmicutes*	*Bacilli*	*Lactobacillales*	*Lactobacillaceae*	*Lactobacillus*	*Lactobacillus*
3892	*Firmicutes*	*Bacilli*	*Lactobacillales*	*Lactobacillaceae*	*Lactobacillus*	*Lactobacillus*

Table S4. Isolated strains by culturomics

bacteriaId	*phylum*	*class*	*order*	*family*	*genus*	*species*
3893	*Firmicutes*	*Bacilli*	*Lactobacillales*	*Lactobacillaceae*	*Lactobacillus*	*Lactobacillus*
3894	*Firmicutes*	*Bacilli*	*Lactobacillales*	*Lactobacillaceae*	*Lactobacillus*	*Lactobacillus*
3895	*Firmicutes*	*Bacilli*	*Lactobacillales*	*Lactobacillaceae*	*Lactobacillus*	*Lactobacillus*
3896	*Firmicutes*	*Bacilli*	*Lactobacillales*	*Lactobacillaceae*	*Lactobacillus*	*Lactobacillus*
3897	*Firmicutes*	*Bacilli*	*Lactobacillales*	*Lactobacillaceae*	*Lactobacillus*	*Lactobacillus*
3898	*Firmicutes*	*Bacilli*	*Lactobacillales*	*Lactobacillaceae*	*Lactobacillus*	*Lactobacillus*
3899	*Firmicutes*	*Bacilli*	*Lactobacillales*	*Lactobacillaceae*	*Lactobacillus*	*Lactobacillus*
3900	*Firmicutes*	*Bacilli*	*Lactobacillales*	*Lactobacillaceae*	*Lactobacillus*	*Lactobacillus*
3901	*Firmicutes*	*Bacilli*	*Lactobacillales*	*Lactobacillaceae*	*Lactobacillus*	*Lactobacillus*
3902	*Firmicutes*	*Bacilli*	*Lactobacillales*	*Lactobacillaceae*	*Lactobacillus*	*Lactobacillus*
3903	*Firmicutes*	*Bacilli*	*Lactobacillales*	*Lactobacillaceae*	*Lactobacillus*	*Lactobacillus*
3904	*Firmicutes*	*Bacilli*	*Lactobacillales*	*Lactobacillaceae*	*Lactobacillus*	*Lactobacillus*
3905	*Firmicutes*	*Bacilli*	*Lactobacillales*	*Lactobacillaceae*	*Lactobacillus*	*Lactobacillus*
3906	*Firmicutes*	*Bacilli*	*Lactobacillales*	*Lactobacillaceae*	*Lactobacillus*	*Lactobacillus*
3907	*Firmicutes*	*Bacilli*	*Lactobacillales*	*Lactobacillaceae*	*Lactobacillus*	*Lactobacillus*
3908	*Firmicutes*	*Bacilli*	*Lactobacillales*	*Lactobacillaceae*	*Lactobacillus*	*Lactobacillus*
3909	*Firmicutes*	*Bacilli*	*Lactobacillales*	*Lactobacillaceae*	*Lactobacillus*	*Lactobacillus*
3910	*Firmicutes*	*Bacilli*	*Lactobacillales*	*Lactobacillaceae*	*Lactobacillus*	*Lactobacillus*
3911	*Firmicutes*	*Bacilli*	*Lactobacillales*	*Lactobacillaceae*	*Lactobacillus*	*Lactobacillus*
3912	*Firmicutes*	*Bacilli*	*Lactobacillales*	*Lactobacillaceae*	*Lactobacillus*	*Lactobacillus*
3913	*Firmicutes*	*Bacilli*	*Lactobacillales*	*Lactobacillaceae*	*Lactobacillus*	*Lactobacillus*
3914	*Firmicutes*	*Bacilli*	*Lactobacillales*	*Lactobacillaceae*	*Lactobacillus*	*Lactobacillus*
3915	*Firmicutes*	*Bacilli*	*Lactobacillales*	*Lactobacillaceae*	*Lactobacillus*	*Lactobacillus*
3916	*Firmicutes*	*Bacilli*	*Lactobacillales*	*Lactobacillaceae*	*Lactobacillus*	*Lactobacillus*
3917	*Firmicutes*	*Bacilli*	*Lactobacillales*	*Lactobacillaceae*	*Lactobacillus*	*Lactobacillus*
3918	*Firmicutes*	*Bacilli*	*Lactobacillales*	*Lactobacillaceae*	*Lactobacillus*	*Lactobacillus*
3919	*Firmicutes*	*Bacilli*	*Lactobacillales*	*Lactobacillaceae*	*Lactobacillus*	*Lactobacillus*
3920	*Firmicutes*	*Bacilli*	*Lactobacillales*	*Lactobacillaceae*	*Lactobacillus*	*Lactobacillus*

Table S4. Isolated strains by culturomics

bacterialId	*phylum*	*class*	*order*	*family*	*genus*	*species*
3921	*Firmicutes*	*Bacilli*	*Lactobacillales*	*Lactobacillaceae*	*Lactobacillus*	*Lactobacillus*
3922	*Firmicutes*	*Bacilli*	*Lactobacillales*	*Lactobacillaceae*	*Lactobacillus*	*Lactobacillus*
3923	*Firmicutes*	*Bacilli*	*Lactobacillales*	*Lactobacillaceae*	*Lactobacillus*	*Lactobacillus*
3924	*Firmicutes*	*Bacilli*	*Lactobacillales*	*Lactobacillaceae*	*Lactobacillus*	*Lactobacillus*
3925	*Firmicutes*	*Bacilli*	*Lactobacillales*	*Lactobacillaceae*	*Lactobacillus*	*Lactobacillus*
3926	*Firmicutes*	*Bacilli*	*Lactobacillales*	*Lactobacillaceae*	*Lactobacillus*	*Lactobacillus*
3927	*Firmicutes*	*Bacilli*	*Lactobacillales*	*Lactobacillaceae*	*Lactobacillus*	*Lactobacillus*
3928	*Firmicutes*	*Bacilli*	*Lactobacillales*	*Lactobacillaceae*	*Lactobacillus*	*Lactobacillus*
3929	*Firmicutes*	*Bacilli*	*Lactobacillales*	*Lactobacillaceae*	*Lactobacillus*	*Lactobacillus*
3930	*Firmicutes*	*Bacilli*	*Lactobacillales*	*Lactobacillaceae*	*Lactobacillus*	*Lactobacillus*
3931	*Firmicutes*	*Bacilli*	*Lactobacillales*	*Lactobacillaceae*	*Lactobacillus*	*Lactobacillus*
3932	*Firmicutes*	*Bacilli*	*Lactobacillales*	*Lactobacillaceae*	*Lactobacillus*	*Lactobacillus*
3933	*Firmicutes*	*Bacilli*	*Lactobacillales*	*Lactobacillaceae*	*Lactobacillus*	*Lactobacillus*
3934	*Firmicutes*	*Bacilli*	*Lactobacillales*	*Lactobacillaceae*	*Lactobacillus*	*Lactobacillus*
3935	*Firmicutes*	*Bacilli*	*Lactobacillales*	*Lactobacillaceae*	*Lactobacillus*	*Lactobacillus*
3936	*Firmicutes*	*Bacilli*	*Lactobacillales*	*Lactobacillaceae*	*Lactobacillus*	*Lactobacillus*
3937	*Firmicutes*	*Bacilli*	*Lactobacillales*	*Lactobacillaceae*	*Lactobacillus*	*Lactobacillus*
3938	*Firmicutes*	*Bacilli*	*Lactobacillales*	*Lactobacillaceae*	*Lactobacillus*	*Lactobacillus*
3939	*Firmicutes*	*Bacilli*	*Lactobacillales*	*Lactobacillaceae*	*Lactobacillus*	*Lactobacillus*
3940	*Firmicutes*	*Bacilli*	*Lactobacillales*	*Lactobacillaceae*	*Lactobacillus*	*Lactobacillus*
3941	*Firmicutes*	*Bacilli*	*Lactobacillales*	*Lactobacillaceae*	*Lactobacillus*	*Lactobacillus*
3942	*Firmicutes*	*Bacilli*	*Lactobacillales*	*Lactobacillaceae*	*Lactobacillus*	*Lactobacillus*
3943	*Firmicutes*	*Bacilli*	*Lactobacillales*	*Lactobacillaceae*	*Lactobacillus*	*Lactobacillus*
3944	*Firmicutes*	*Bacilli*	*Lactobacillales*	*Lactobacillaceae*	*Lactobacillus*	*Lactobacillus*
3945	*Firmicutes*	*Bacilli*	*Lactobacillales*	*Lactobacillaceae*	*Lactobacillus*	*Lactobacillus*
3946	*Firmicutes*	*Bacilli*	*Lactobacillales*	*Lactobacillaceae*	*Lactobacillus*	*Lactobacillus*
3947	*Firmicutes*	*Bacilli*	*Lactobacillales*	*Lactobacillaceae*	*Lactobacillus*	*Lactobacillus*
3948	*Firmicutes*	*Bacilli*	*Lactobacillales*	*Lactobacillaceae*	*Lactobacillus*	*Lactobacillus*

Table S4. Isolated strains by culturomics

bacteriaId	*phylum*	*class*	*order*	*family*	*genus*	*species*
3949	*Firmicutes*	*Bacilli*	*Lactobacillales*	*Lactobacillaceae*	*Lactobacillus*	*Lactobacillus*
3950	*Firmicutes*	*Bacilli*	*Lactobacillales*	*Lactobacillaceae*	*Lactobacillus*	*Lactobacillus*
3951	*Firmicutes*	*Bacilli*	*Lactobacillales*	*Lactobacillaceae*	*Lactobacillus*	*Lactobacillus*
3952	*Firmicutes*	*Bacilli*	*Lactobacillales*	*Lactobacillaceae*	*Lactobacillus*	*Lactobacillus*
3953	*Firmicutes*	*Bacilli*	*Lactobacillales*	*Lactobacillaceae*	*Lactobacillus*	*Lactobacillus*
3954	*Firmicutes*	*Bacilli*	*Lactobacillales*	*Lactobacillaceae*	*Lactobacillus*	*Lactobacillus*
3955	*Firmicutes*	*Bacilli*	*Lactobacillales*	*Lactobacillaceae*	*Lactobacillus*	*Lactobacillus*
3956	*Firmicutes*	*Bacilli*	*Lactobacillales*	*Lactobacillaceae*	*Lactobacillus*	*Lactobacillus*
3957	*Firmicutes*	*Bacilli*	*Lactobacillales*	*Lactobacillaceae*	*Lactobacillus*	*Lactobacillus*
3958	*Firmicutes*	*Bacilli*	*Lactobacillales*	*Lactobacillaceae*	*Lactobacillus*	*Lactobacillus*
3959	*Firmicutes*	*Bacilli*	*Lactobacillales*	*Lactobacillaceae*	*Lactobacillus*	*Lactobacillus*
3960	*Firmicutes*	*Bacilli*	*Lactobacillales*	*Lactobacillaceae*	*Lactobacillus*	*Lactobacillus*
3961	*Firmicutes*	*Bacilli*	*Lactobacillales*	*Lactobacillaceae*	*Lactobacillus*	*Lactobacillus*
3962	*Firmicutes*	*Bacilli*	*Lactobacillales*	*Lactobacillaceae*	*Lactobacillus*	*Lactobacillus*
3963	*Firmicutes*	*Bacilli*	*Lactobacillales*	*Lactobacillaceae*	*Lactobacillus*	*Lactobacillus*
3964	*Firmicutes*	*Bacilli*	*Lactobacillales*	*Lactobacillaceae*	*Lactobacillus*	*Lactobacillus*
3965	*Firmicutes*	*Bacilli*	*Lactobacillales*	*Lactobacillaceae*	*Lactobacillus*	*Lactobacillus*
3966	*Firmicutes*	*Bacilli*	*Lactobacillales*	*Lactobacillaceae*	*Lactobacillus*	*Lactobacillus*
3967	*Firmicutes*	*Bacilli*	*Lactobacillales*	*Lactobacillaceae*	*Lactobacillus*	*Lactobacillus*
3968	*Firmicutes*	*Bacilli*	*Lactobacillales*	*Lactobacillaceae*	*Lactobacillus*	*Lactobacillus*
3969	*Firmicutes*	*Bacilli*	*Lactobacillales*	*Lactobacillaceae*	*Lactobacillus*	*Lactobacillus*
3970	*Firmicutes*	*Bacilli*	*Lactobacillales*	*Lactobacillaceae*	*Lactobacillus*	*Lactobacillus*
3971	*Firmicutes*	*Bacilli*	*Lactobacillales*	*Lactobacillaceae*	*Lactobacillus*	*Lactobacillus*
3972	*Firmicutes*	*Bacilli*	*Lactobacillales*	*Lactobacillaceae*	*Lactobacillus*	*Lactobacillus*
3973	*Firmicutes*	*Bacilli*	*Lactobacillales*	*Lactobacillaceae*	*Lactobacillus*	*Lactobacillus*
3974	*Firmicutes*	*Bacilli*	*Lactobacillales*	*Lactobacillaceae*	*Lactobacillus*	*Lactobacillus*
3975	*Firmicutes*	*Bacilli*	*Lactobacillales*	*Lactobacillaceae*	*Lactobacillus*	*Lactobacillus*
3976	*Firmicutes*	*Bacilli*	*Lactobacillales*	*Lactobacillaceae*	*Lactobacillus*	*Lactobacillus*

Table S4. Isolated strains by culturomics

bacteriaId	*phylum*	*class*	*order*	*family*	*genus*	*species*
3977	*Firmicutes*	*Bacilli*	*Lactobacillales*	*Lactobacillaceae*	*Lactobacillus*	*Lactobacillus*
3978	*Firmicutes*	*Bacilli*	*Lactobacillales*	*Lactobacillaceae*	*Lactobacillus*	*Lactobacillus*
3979	*Firmicutes*	*Bacilli*	*Lactobacillales*	*Lactobacillaceae*	*Lactobacillus*	*Lactobacillus*
3980	*Firmicutes*	*Bacilli*	*Lactobacillales*	*Lactobacillaceae*	*Lactobacillus*	*Lactobacillus*
3981	*Firmicutes*	*Bacilli*	*Lactobacillales*	*Lactobacillaceae*	*Lactobacillus*	*Lactobacillus*
3982	*Firmicutes*	*Bacilli*	*Lactobacillales*	*Lactobacillaceae*	*Lactobacillus*	*Lactobacillus*
3983	*Firmicutes*	*Bacilli*	*Lactobacillales*	*Lactobacillaceae*	*Lactobacillus*	*Lactobacillus*
3984	*Firmicutes*	*Bacilli*	*Lactobacillales*	*Lactobacillaceae*	*Lactobacillus*	*Lactobacillus*
3985	*Firmicutes*	*Bacilli*	*Lactobacillales*	*Lactobacillaceae*	*Lactobacillus*	*Lactobacillus*
3986	*Firmicutes*	*Bacilli*	*Lactobacillales*	*Lactobacillaceae*	*Lactobacillus*	*Lactobacillus*
3987	*Firmicutes*	*Bacilli*	*Lactobacillales*	*Lactobacillaceae*	*Lactobacillus*	*Lactobacillus*
3988	*Firmicutes*	*Bacilli*	*Lactobacillales*	*Lactobacillaceae*	*Lactobacillus*	*Lactobacillus*
3989	*Firmicutes*	*Bacilli*	*Lactobacillales*	*Lactobacillaceae*	*Lactobacillus*	*Lactobacillus*
3990	*Firmicutes*	*Bacilli*	*Lactobacillales*	*Lactobacillaceae*	*Lactobacillus*	*Lactobacillus*
3991	*Firmicutes*	*Bacilli*	*Lactobacillales*	*Lactobacillaceae*	*Lactobacillus*	*Lactobacillus*
3992	*Firmicutes*	*Bacilli*	*Lactobacillales*	*Lactobacillaceae*	*Lactobacillus*	*Lactobacillus*
3993	*Firmicutes*	*Bacilli*	*Lactobacillales*	*Lactobacillaceae*	*Lactobacillus*	*Lactobacillus*
3994	*Firmicutes*	*Bacilli*	*Lactobacillales*	*Lactobacillaceae*	*Lactobacillus*	*Lactobacillus*
3995	*Firmicutes*	*Bacilli*	*Lactobacillales*	*Lactobacillaceae*	*Lactobacillus*	*Lactobacillus*
3996	*Firmicutes*	*Bacilli*	*Lactobacillales*	*Lactobacillaceae*	*Lactobacillus*	*Lactobacillus*
3997	*Firmicutes*	*Bacilli*	*Lactobacillales*	*Lactobacillaceae*	*Lactobacillus*	*Lactobacillus*
3998	*Firmicutes*	*Bacilli*	*Lactobacillales*	*Lactobacillaceae*	*Lactobacillus*	*Lactobacillus*
3999	*Firmicutes*	*Bacilli*	*Lactobacillales*	*Lactobacillaceae*	*Lactobacillus*	*Lactobacillus*
4000	*Firmicutes*	*Bacilli*	*Lactobacillales*	*Lactobacillaceae*	*Lactobacillus*	*Lactobacillus*
4001	*Firmicutes*	*Bacilli*	*Lactobacillales*	*Lactobacillaceae*	*Lactobacillus*	*Lactobacillus*
4002	*Firmicutes*	*Bacilli*	*Lactobacillales*	*Lactobacillaceae*	*Lactobacillus*	*Lactobacillus*
4003	*Firmicutes*	*Bacilli*	*Lactobacillales*	*Lactobacillaceae*	*Lactobacillus*	*Lactobacillus*
4004	*Firmicutes*	*Bacilli*	*Lactobacillales*	*Lactobacillaceae*	*Lactobacillus*	*Lactobacillus*

Table S4. Isolated strains by culturomics

bacteriaId	*phylum*	*class*	*order*	*family*	*genus*	*species*
4005	*Firmicutes*	*Bacilli*	*Lactobacillales*	*Lactobacillaceae*	*Lactobacillus*	*Lactobacillus*
4006	*Firmicutes*	*Bacilli*	*Lactobacillales*	*Lactobacillaceae*	*Lactobacillus*	*Lactobacillus*
4007	*Firmicutes*	*Bacilli*	*Lactobacillales*	*Lactobacillaceae*	*Lactobacillus*	*Lactobacillus*
4008	*Firmicutes*	*Bacilli*	*Lactobacillales*	*Lactobacillaceae*	*Lactobacillus*	*Lactobacillus*
4009	*Firmicutes*	*Bacilli*	*Lactobacillales*	*Lactobacillaceae*	*Lactobacillus*	*Lactobacillus*
4010	*Firmicutes*	*Bacilli*	*Lactobacillales*	*Lactobacillaceae*	*Lactobacillus*	*Lactobacillus*
4011	*Firmicutes*	*Bacilli*	*Lactobacillales*	*Lactobacillaceae*	*Lactobacillus*	*Lactobacillus*
4012	*Firmicutes*	*Bacilli*	*Lactobacillales*	*Lactobacillaceae*	*Lactobacillus*	*Lactobacillus*
4013	*Firmicutes*	*Bacilli*	*Lactobacillales*	*Lactobacillaceae*	*Lactobacillus*	*Lactobacillus*
4014	*Firmicutes*	*Bacilli*	*Lactobacillales*	*Lactobacillaceae*	*Lactobacillus*	*Lactobacillus*
4015	*Firmicutes*	*Bacilli*	*Lactobacillales*	*Lactobacillaceae*	*Lactobacillus*	*Lactobacillus*
4016	*Firmicutes*	*Bacilli*	*Lactobacillales*	*Lactobacillaceae*	*Lactobacillus*	*Lactobacillus*
4017	*Firmicutes*	*Bacilli*	*Lactobacillales*	*Lactobacillaceae*	*Lactobacillus*	*Lactobacillus*
4018	*Firmicutes*	*Bacilli*	*Lactobacillales*	*Lactobacillaceae*	*Lactobacillus*	*Lactobacillus*
4019	*Firmicutes*	*Bacilli*	*Lactobacillales*	*Lactobacillaceae*	*Lactobacillus*	*Lactobacillus*
4020	*Firmicutes*	*Bacilli*	*Lactobacillales*	*Lactobacillaceae*	*Lactobacillus*	*Lactobacillus*
4021	*Firmicutes*	*Bacilli*	*Lactobacillales*	*Lactobacillaceae*	*Lactobacillus*	*Lactobacillus*
4022	*Firmicutes*	*Bacilli*	*Lactobacillales*	*Lactobacillaceae*	*Lactobacillus*	*Lactobacillus*
4023	*Firmicutes*	*Bacilli*	*Lactobacillales*	*Lactobacillaceae*	*Lactobacillus*	*Lactobacillus*
4024	*Firmicutes*	*Bacilli*	*Lactobacillales*	*Lactobacillaceae*	*Lactobacillus*	*Lactobacillus*
4025	*Firmicutes*	*Bacilli*	*Lactobacillales*	*Lactobacillaceae*	*Lactobacillus*	*Lactobacillus*
4026	*Firmicutes*	*Bacilli*	*Lactobacillales*	*Lactobacillaceae*	*Lactobacillus*	*Lactobacillus*
4027	*Firmicutes*	*Bacilli*	*Lactobacillales*	*Lactobacillaceae*	*Lactobacillus*	*Lactobacillus*
4028	*Firmicutes*	*Bacilli*	*Lactobacillales*	*Lactobacillaceae*	*Lactobacillus*	*Lactobacillus*
4029	*Firmicutes*	*Bacilli*	*Lactobacillales*	*Lactobacillaceae*	*Lactobacillus*	*Lactobacillus*
4030	*Firmicutes*	*Bacilli*	*Lactobacillales*	*Lactobacillaceae*	*Lactobacillus*	*Lactobacillus*
4031	*Firmicutes*	*Bacilli*	*Lactobacillales*	*Lactobacillaceae*	*Lactobacillus*	*Lactobacillus*
4032	*Firmicutes*	*Bacilli*	*Lactobacillales*	*Lactobacillaceae*	*Lactobacillus*	*Lactobacillus*

Table S4. Isolated strains by culturomics

bacteriaId	*phylum*	*class*	*order*	*family*	*genus*	*species*
4033	*Firmicutes*	*Bacilli*	*Lactobacillales*	*Lactobacillaceae*	*Lactobacillus*	*Lactobacillus*
4034	*Firmicutes*	*Bacilli*	*Lactobacillales*	*Lactobacillaceae*	*Lactobacillus*	*Lactobacillus*
4035	*Firmicutes*	*Bacilli*	*Lactobacillales*	*Lactobacillaceae*	*Lactobacillus*	*Lactobacillus*
4036	*Firmicutes*	*Bacilli*	*Lactobacillales*	*Lactobacillaceae*	*Lactobacillus*	*Lactobacillus*
4037	*Firmicutes*	*Bacilli*	*Lactobacillales*	*Lactobacillaceae*	*Lactobacillus*	*Lactobacillus*
4038	*Firmicutes*	*Bacilli*	*Lactobacillales*	*Lactobacillaceae*	*Lactobacillus*	*Lactobacillus*
4039	*Firmicutes*	*Bacilli*	*Lactobacillales*	*Lactobacillaceae*	*Lactobacillus*	*Lactobacillus*
4040	*Firmicutes*	*Bacilli*	*Lactobacillales*	*Lactobacillaceae*	*Lactobacillus*	*Lactobacillus*
4041	*Firmicutes*	*Bacilli*	*Lactobacillales*	*Lactobacillaceae*	*Lactobacillus*	*Lactobacillus*
4042	*Firmicutes*	*Bacilli*	*Lactobacillales*	*Lactobacillaceae*	*Lactobacillus*	*Lactobacillus*
4043	*Firmicutes*	*Bacilli*	*Lactobacillales*	*Lactobacillaceae*	*Lactobacillus*	*Lactobacillus*
4044	*Firmicutes*	*Bacilli*	*Lactobacillales*	*Lactobacillaceae*	*Lactobacillus*	*Lactobacillus*
4045	*Firmicutes*	*Bacilli*	*Lactobacillales*	*Lactobacillaceae*	*Lactobacillus*	*Lactobacillus*
4046	*Firmicutes*	*Bacilli*	*Lactobacillales*	*Lactobacillaceae*	*Lactobacillus*	*Lactobacillus*
4047	*Firmicutes*	*Bacilli*	*Lactobacillales*	*Lactobacillaceae*	*Lactobacillus*	*Lactobacillus*
4048	*Firmicutes*	*Bacilli*	*Lactobacillales*	*Lactobacillaceae*	*Lactobacillus*	*Lactobacillus*
4049	*Firmicutes*	*Bacilli*	*Lactobacillales*	*Lactobacillaceae*	*Lactobacillus*	*Lactobacillus*
4050	*Firmicutes*	*Bacilli*	*Lactobacillales*	*Lactobacillaceae*	*Lactobacillus*	*Lactobacillus*
4051	*Firmicutes*	*Bacilli*	*Lactobacillales*	*Lactobacillaceae*	*Lactobacillus*	*Lactobacillus*
4052	*Firmicutes*	*Bacilli*	*Lactobacillales*	*Lactobacillaceae*	*Lactobacillus*	*Lactobacillus*
4053	*Firmicutes*	*Bacilli*	*Lactobacillales*	*Lactobacillaceae*	*Lactobacillus*	*Lactobacillus*
4054	*Firmicutes*	*Bacilli*	*Lactobacillales*	*Lactobacillaceae*	*Lactobacillus*	*Lactobacillus*
4055	*Firmicutes*	*Bacilli*	*Lactobacillales*	*Lactobacillaceae*	*Lactobacillus*	*Lactobacillus*
4056	*Firmicutes*	*Bacilli*	*Lactobacillales*	*Lactobacillaceae*	*Lactobacillus*	*Lactobacillus*
4057	*Firmicutes*	*Bacilli*	*Lactobacillales*	*Lactobacillaceae*	*Lactobacillus*	*Lactobacillus*
4058	*Firmicutes*	*Bacilli*	*Lactobacillales*	*Lactobacillaceae*	*Lactobacillus*	*Lactobacillus*
4059	*Firmicutes*	*Bacilli*	*Lactobacillales*	*Lactobacillaceae*	*Lactobacillus*	*Lactobacillus*
4060	*Firmicutes*	*Bacilli*	*Lactobacillales*	*Lactobacillaceae*	*Lactobacillus*	*Lactobacillus*

Table S4. Isolated strains by culturomics

bacteriaId	*phylum*	*class*	*order*	*family*	*genus*	*species*
4061	*Firmicutes*	*Bacilli*	*Lactobacillales*	*Lactobacillaceae*	*Lactobacillus*	*Lactobacillus*
4062	*Firmicutes*	*Bacilli*	*Lactobacillales*	*Lactobacillaceae*	*Lactobacillus*	*Lactobacillus*
4063	*Firmicutes*	*Bacilli*	*Lactobacillales*	*Lactobacillaceae*	*Lactobacillus*	*Lactobacillus*
4064	*Firmicutes*	*Bacilli*	*Lactobacillales*	*Lactobacillaceae*	*Lactobacillus*	*Lactobacillus*
4065	*Firmicutes*	*Bacilli*	*Lactobacillales*	*Lactobacillaceae*	*Lactobacillus*	*Lactobacillus*
4066	*Firmicutes*	*Bacilli*	*Lactobacillales*	*Lactobacillaceae*	*Lactobacillus*	*Lactobacillus*
4067	*Firmicutes*	*Bacilli*	*Lactobacillales*	*Lactobacillaceae*	*Lactobacillus*	*Lactobacillus*
4068	*Firmicutes*	*Bacilli*	*Lactobacillales*	*Lactobacillaceae*	*Lactobacillus*	*Lactobacillus*
4069	*Firmicutes*	*Bacilli*	*Lactobacillales*	*Lactobacillaceae*	*Lactobacillus*	*Lactobacillus*
4070	*Firmicutes*	*Bacilli*	*Lactobacillales*	*Lactobacillaceae*	*Lactobacillus*	*Lactobacillus*
4071	*Firmicutes*	*Bacilli*	*Lactobacillales*	*Lactobacillaceae*	*Lactobacillus*	*Lactobacillus*
4072	*Firmicutes*	*Bacilli*	*Lactobacillales*	*Lactobacillaceae*	*Lactobacillus*	*Lactobacillus*
4073	*Firmicutes*	*Bacilli*	*Lactobacillales*	*Lactobacillaceae*	*Lactobacillus*	*Lactobacillus*
4074	*Firmicutes*	*Bacilli*	*Lactobacillales*	*Lactobacillaceae*	*Lactobacillus*	*Lactobacillus*
4075	*Firmicutes*	*Bacilli*	*Lactobacillales*	*Lactobacillaceae*	*Lactobacillus*	*Lactobacillus*
4076	*Firmicutes*	*Bacilli*	*Lactobacillales*	*Lactobacillaceae*	*Lactobacillus*	*Lactobacillus*
4077	*Firmicutes*	*Bacilli*	*Lactobacillales*	*Lactobacillaceae*	*Lactobacillus*	*Lactobacillus*
4078	*Firmicutes*	*Bacilli*	*Lactobacillales*	*Lactobacillaceae*	*Lactobacillus*	*Lactobacillus*
4079	*Firmicutes*	*Bacilli*	*Lactobacillales*	*Lactobacillaceae*	*Lactobacillus*	*Lactobacillus*
4080	*Firmicutes*	*Bacilli*	*Lactobacillales*	*Lactobacillaceae*	*Lactobacillus*	*Lactobacillus*
4081	*Firmicutes*	*Bacilli*	*Lactobacillales*	*Lactobacillaceae*	*Lactobacillus*	*Lactobacillus*
4082	*Firmicutes*	*Bacilli*	*Lactobacillales*	*Lactobacillaceae*	*Lactobacillus*	*Lactobacillus*
4083	*Firmicutes*	*Bacilli*	*Lactobacillales*	*Lactobacillaceae*	*Lactobacillus*	*Lactobacillus*
4084	*Firmicutes*	*Bacilli*	*Lactobacillales*	*Lactobacillaceae*	*Lactobacillus*	*Lactobacillus*
4085	*Firmicutes*	*Bacilli*	*Lactobacillales*	*Lactobacillaceae*	*Lactobacillus*	*Lactobacillus*
4086	*Firmicutes*	*Bacilli*	*Lactobacillales*	*Lactobacillaceae*	*Lactobacillus*	*Lactobacillus*
4087	*Firmicutes*	*Bacilli*	*Lactobacillales*	*Lactobacillaceae*	*Lactobacillus*	*Lactobacillus*
4088	*Firmicutes*	*Bacilli*	*Lactobacillales*	*Lactobacillaceae*	*Lactobacillus*	*Lactobacillus*

Table S4. Isolated strains by culturomics

bacteriaId	*phylum*	*class*	*order*	*family*	*genus*	*species*
4089	*Firmicutes*	*Bacilli*	*Lactobacillales*	*Lactobacillaceae*	*Lactobacillus*	*Lactobacillus*
4090	*Firmicutes*	*Bacilli*	*Lactobacillales*	*Lactobacillaceae*	*Lactobacillus*	*Lactobacillus*
4091	*Firmicutes*	*Bacilli*	*Lactobacillales*	*Lactobacillaceae*	*Lactobacillus*	*Lactobacillus*
4092	*Firmicutes*	*Bacilli*	*Lactobacillales*	*Lactobacillaceae*	*Lactobacillus*	*Lactobacillus*
4093	*Firmicutes*	*Bacilli*	*Lactobacillales*	*Lactobacillaceae*	*Lactobacillus*	*Lactobacillus*
4094	*Firmicutes*	*Bacilli*	*Lactobacillales*	*Lactobacillaceae*	*Lactobacillus*	*Lactobacillus*
4095	*Firmicutes*	*Bacilli*	*Lactobacillales*	*Lactobacillaceae*	*Lactobacillus*	*Lactobacillus*
4096	*Firmicutes*	*Bacilli*	*Lactobacillales*	*Lactobacillaceae*	*Lactobacillus*	*Lactobacillus*
4097	*Firmicutes*	*Bacilli*	*Lactobacillales*	*Lactobacillaceae*	*Lactobacillus*	*Lactobacillus*
4098	*Firmicutes*	*Bacilli*	*Lactobacillales*	*Lactobacillaceae*	*Lactobacillus*	*Lactobacillus*
4099	*Firmicutes*	*Bacilli*	*Lactobacillales*	*Lactobacillaceae*	*Lactobacillus*	*Lactobacillus*
4100	*Firmicutes*	*Bacilli*	*Lactobacillales*	*Lactobacillaceae*	*Lactobacillus*	*Lactobacillus*
4101	*Firmicutes*	*Bacilli*	*Lactobacillales*	*Lactobacillaceae*	*Lactobacillus*	*Lactobacillus*
4102	*Firmicutes*	*Bacilli*	*Lactobacillales*	*Lactobacillaceae*	*Lactobacillus*	*Lactobacillus*
4103	*Firmicutes*	*Bacilli*	*Lactobacillales*	*Lactobacillaceae*	*Lactobacillus*	*Lactobacillus*
4104	*Firmicutes*	*Bacilli*	*Lactobacillales*	*Lactobacillaceae*	*Lactobacillus*	*Lactobacillus*
4105	*Firmicutes*	*Bacilli*	*Lactobacillales*	*Lactobacillaceae*	*Lactobacillus*	*Lactobacillus*
4106	*Firmicutes*	*Bacilli*	*Lactobacillales*	*Lactobacillaceae*	*Lactobacillus*	*Lactobacillus*
4107	*Firmicutes*	*Bacilli*	*Lactobacillales*	*Lactobacillaceae*	*Lactobacillus*	*Lactobacillus*
4108	*Firmicutes*	*Bacilli*	*Lactobacillales*	*Lactobacillaceae*	*Lactobacillus*	*Lactobacillus*
4109	*Firmicutes*	*Bacilli*	*Lactobacillales*	*Lactobacillaceae*	*Lactobacillus*	*Lactobacillus*
4110	*Firmicutes*	*Bacilli*	*Lactobacillales*	*Lactobacillaceae*	*Lactobacillus*	*Lactobacillus*
4111	*Firmicutes*	*Bacilli*	*Lactobacillales*	*Lactobacillaceae*	*Lactobacillus*	*Lactobacillus*
4112	*Firmicutes*	*Bacilli*	*Lactobacillales*	*Lactobacillaceae*	*Lactobacillus*	*Lactobacillus*
4113	*Firmicutes*	*Bacilli*	*Lactobacillales*	*Lactobacillaceae*	*Lactobacillus*	*Lactobacillus*
4114	*Firmicutes*	*Bacilli*	*Lactobacillales*	*Lactobacillaceae*	*Lactobacillus*	*Lactobacillus*
4115	*Firmicutes*	*Bacilli*	*Lactobacillales*	*Lactobacillaceae*	*Lactobacillus*	*Lactobacillus*
4116	*Firmicutes*	*Bacilli*	*Lactobacillales*	*Lactobacillaceae*	*Lactobacillus*	*Lactobacillus*

Table S4. Isolated strains by culturomics

bacteriaId	*phylum*	*class*	*order*	*family*	*genus*	*species*
4117	*Firmicutes*	*Bacilli*	*Lactobacillales*	*Lactobacillaceae*	*Lactobacillus*	*Lactobacillus*
4118	*Firmicutes*	*Bacilli*	*Lactobacillales*	*Lactobacillaceae*	*Lactobacillus*	*Lactobacillus*
4119	*Firmicutes*	*Bacilli*	*Lactobacillales*	*Lactobacillaceae*	*Lactobacillus*	*Lactobacillus*
4120	*Firmicutes*	*Bacilli*	*Lactobacillales*	*Lactobacillaceae*	*Lactobacillus*	*Lactobacillus*
4121	*Firmicutes*	*Bacilli*	*Lactobacillales*	*Lactobacillaceae*	*Lactobacillus*	*Lactobacillus*
4122	*Firmicutes*	*Bacilli*	*Lactobacillales*	*Lactobacillaceae*	*Lactobacillus*	*Lactobacillus*
4123	*Firmicutes*	*Bacilli*	*Lactobacillales*	*Lactobacillaceae*	*Lactobacillus*	*Lactobacillus*
4124	*Firmicutes*	*Bacilli*	*Lactobacillales*	*Lactobacillaceae*	*Lactobacillus*	*Lactobacillus*
4125	*Firmicutes*	*Bacilli*	*Lactobacillales*	*Lactobacillaceae*	*Lactobacillus*	*Lactobacillus*
4126	*Firmicutes*	*Bacilli*	*Lactobacillales*	*Lactobacillaceae*	*Lactobacillus*	*Lactobacillus*
4127	*Firmicutes*	*Bacilli*	*Lactobacillales*	*Lactobacillaceae*	*Lactobacillus*	*Lactobacillus*
4128	*Firmicutes*	*Bacilli*	*Lactobacillales*	*Lactobacillaceae*	*Lactobacillus*	*Lactobacillus*
4129	*Firmicutes*	*Bacilli*	*Lactobacillales*	*Lactobacillaceae*	*Lactobacillus*	*Lactobacillus*
4130	*Firmicutes*	*Bacilli*	*Lactobacillales*	*Lactobacillaceae*	*Lactobacillus*	*Lactobacillus*
4131	*Firmicutes*	*Bacilli*	*Lactobacillales*	*Lactobacillaceae*	*Lactobacillus*	*Lactobacillus*
4132	*Firmicutes*	*Bacilli*	*Lactobacillales*	*Lactobacillaceae*	*Lactobacillus*	*Lactobacillus*
4133	*Firmicutes*	*Bacilli*	*Lactobacillales*	*Lactobacillaceae*	*Lactobacillus*	*Lactobacillus*
4134	*Firmicutes*	*Bacilli*	*Lactobacillales*	*Lactobacillaceae*	*Lactobacillus*	*Lactobacillus*
4135	*Firmicutes*	*Bacilli*	*Lactobacillales*	*Lactobacillaceae*	*Lactobacillus*	*Lactobacillus*
4136	*Firmicutes*	*Bacilli*	*Lactobacillales*	*Lactobacillaceae*	*Lactobacillus*	*Lactobacillus*
4137	*Firmicutes*	*Bacilli*	*Lactobacillales*	*Lactobacillaceae*	*Lactobacillus*	*Lactobacillus*
4138	*Firmicutes*	*Bacilli*	*Lactobacillales*	*Lactobacillaceae*	*Lactobacillus*	*Lactobacillus*
4139	*Firmicutes*	*Bacilli*	*Lactobacillales*	*Lactobacillaceae*	*Lactobacillus*	*Lactobacillus*
4140	*Firmicutes*	*Bacilli*	*Lactobacillales*	*Lactobacillaceae*	*Lactobacillus*	*Lactobacillus*
4141	*Firmicutes*	*Bacilli*	*Lactobacillales*	*Lactobacillaceae*	*Lactobacillus*	*Lactobacillus*
4142	*Firmicutes*	*Bacilli*	*Lactobacillales*	*Lactobacillaceae*	*Lactobacillus*	*Lactobacillus*
4143	*Firmicutes*	*Bacilli*	*Lactobacillales*	*Lactobacillaceae*	*Lactobacillus*	*Lactobacillus*
4144	*Firmicutes*	*Bacilli*	*Lactobacillales*	*Lactobacillaceae*	*Lactobacillus*	*Lactobacillus*

Table S4. Isolated strains by culturomics

bacteriaId	*phylum*	*class*	*order*	*family*	*genus*	*species*
4145	*Firmicutes*	*Bacilli*	*Lactobacillales*	*Lactobacillaceae*	*Lactobacillus*	*Lactobacillus*
4146	*Firmicutes*	*Bacilli*	*Lactobacillales*	*Lactobacillaceae*	*Lactobacillus*	*Lactobacillus*
4147	*Firmicutes*	*Bacilli*	*Lactobacillales*	*Lactobacillaceae*	*Lactobacillus*	*Lactobacillus*
4148	*Firmicutes*	*Bacilli*	*Lactobacillales*	*Lactobacillaceae*	*Lactobacillus*	*Lactobacillus*
4149	*Firmicutes*	*Bacilli*	*Lactobacillales*	*Lactobacillaceae*	*Lactobacillus*	*Lactobacillus*
4150	*Firmicutes*	*Bacilli*	*Lactobacillales*	*Lactobacillaceae*	*Lactobacillus*	*Lactobacillus*
4151	*Firmicutes*	*Bacilli*	*Lactobacillales*	*Lactobacillaceae*	*Lactobacillus*	*Lactobacillus*
4152	*Firmicutes*	*Bacilli*	*Lactobacillales*	*Lactobacillaceae*	*Lactobacillus*	*Lactobacillus*
4153	*Firmicutes*	*Bacilli*	*Lactobacillales*	*Lactobacillaceae*	*Lactobacillus*	*Lactobacillus*
4154	*Firmicutes*	*Bacilli*	*Lactobacillales*	*Lactobacillaceae*	*Lactobacillus*	*Lactobacillus*
4155	*Firmicutes*	*Bacilli*	*Lactobacillales*	*Lactobacillaceae*	*Lactobacillus*	*Lactobacillus*
4156	*Firmicutes*	*Bacilli*	*Lactobacillales*	*Lactobacillaceae*	*Lactobacillus*	*Lactobacillus*
4157	*Firmicutes*	*Bacilli*	*Lactobacillales*	*Lactobacillaceae*	*Lactobacillus*	*Lactobacillus*
4158	*Firmicutes*	*Bacilli*	*Lactobacillales*	*Lactobacillaceae*	*Lactobacillus*	*Lactobacillus*
4159	*Firmicutes*	*Bacilli*	*Lactobacillales*	*Lactobacillaceae*	*Lactobacillus*	*Lactobacillus*
4160	*Firmicutes*	*Bacilli*	*Lactobacillales*	*Lactobacillaceae*	*Lactobacillus*	*Lactobacillus*
4161	*Firmicutes*	*Bacilli*	*Lactobacillales*	*Lactobacillaceae*	*Lactobacillus*	*Lactobacillus*
4162	*Firmicutes*	*Bacilli*	*Lactobacillales*	*Lactobacillaceae*	*Lactobacillus*	*Lactobacillus*
4163	*Firmicutes*	*Bacilli*	*Lactobacillales*	*Lactobacillaceae*	*Lactobacillus*	*Lactobacillus*
4164	*Firmicutes*	*Bacilli*	*Lactobacillales*	*Lactobacillaceae*	*Lactobacillus*	*Lactobacillus*
4165	*Firmicutes*	*Bacilli*	*Lactobacillales*	*Lactobacillaceae*	*Lactobacillus*	*Lactobacillus*
4166	*Firmicutes*	*Bacilli*	*Lactobacillales*	*Lactobacillaceae*	*Lactobacillus*	*Lactobacillus*
4167	*Firmicutes*	*Bacilli*	*Lactobacillales*	*Lactobacillaceae*	*Lactobacillus*	*Lactobacillus*
4168	*Firmicutes*	*Bacilli*	*Lactobacillales*	*Lactobacillaceae*	*Lactobacillus*	*Lactobacillus*
4169	*Firmicutes*	*Bacilli*	*Lactobacillales*	*Lactobacillaceae*	*Lactobacillus*	*Lactobacillus*
4170	*Firmicutes*	*Bacilli*	*Lactobacillales*	*Lactobacillaceae*	*Lactobacillus*	*Lactobacillus*
4171	*Firmicutes*	*Bacilli*	*Lactobacillales*	*Lactobacillaceae*	*Lactobacillus*	*Lactobacillus*
4172	*Firmicutes*	*Bacilli*	*Lactobacillales*	*Lactobacillaceae*	*Lactobacillus*	*Lactobacillus*

Table S4. Isolated strains by culturomics

bacteriald	*phylum*	*class*	*order*	*family*	*genus*	*species*
4173	*Firmicutes*	*Bacilli*	*Lactobacillales*	*Lactobacillaceae*	*Lactobacillus*	*Lactobacillus*
4174	*Firmicutes*	*Bacilli*	*Lactobacillales*	*Lactobacillaceae*	*Lactobacillus*	*Lactobacillus*
4175	*Firmicutes*	*Bacilli*	*Lactobacillales*	*Lactobacillaceae*	*Lactobacillus*	*Lactobacillus*
4176	*Firmicutes*	*Bacilli*	*Lactobacillales*	*Lactobacillaceae*	*Lactobacillus*	*Lactobacillus*
4177	*Firmicutes*	*Bacilli*	*Lactobacillales*	*Lactobacillaceae*	*Lactobacillus*	*Lactobacillus*
4178	*Firmicutes*	*Bacilli*	*Lactobacillales*	*Lactobacillaceae*	*Lactobacillus*	*Lactobacillus*
4179	*Firmicutes*	*Bacilli*	*Lactobacillales*	*Lactobacillaceae*	*Lactobacillus*	*Lactobacillus*
4180	*Firmicutes*	*Bacilli*	*Lactobacillales*	*Lactobacillaceae*	*Lactobacillus*	*Lactobacillus*
4181	*Firmicutes*	*Bacilli*	*Lactobacillales*	*Lactobacillaceae*	*Lactobacillus*	*Lactobacillus*
4182	*Firmicutes*	*Bacilli*	*Lactobacillales*	*Lactobacillaceae*	*Lactobacillus*	*Lactobacillus*
4183	*Firmicutes*	*Bacilli*	*Lactobacillales*	*Lactobacillaceae*	*Lactobacillus*	*Lactobacillus*
4184	*Firmicutes*	*Bacilli*	*Lactobacillales*	*Lactobacillaceae*	*Lactobacillus*	*Lactobacillus*
4185	*Firmicutes*	*Bacilli*	*Lactobacillales*	*Lactobacillaceae*	*Lactobacillus*	*Lactobacillus*
4186	*Firmicutes*	*Bacilli*	*Lactobacillales*	*Lactobacillaceae*	*Lactobacillus*	*Lactobacillus*
4187	*Firmicutes*	*Bacilli*	*Lactobacillales*	*Lactobacillaceae*	*Lactobacillus*	*Lactobacillus*
4188	*Firmicutes*	*Bacilli*	*Lactobacillales*	*Lactobacillaceae*	*Lactobacillus*	*Lactobacillus*
4189	*Firmicutes*	*Bacilli*	*Lactobacillales*	*Lactobacillaceae*	*Lactobacillus*	*Lactobacillus*
4190	*Firmicutes*	*Bacilli*	*Lactobacillales*	*Lactobacillaceae*	*Lactobacillus*	*Lactobacillus*
4191	*Firmicutes*	*Bacilli*	*Lactobacillales*	*Lactobacillaceae*	*Lactobacillus*	*Lactobacillus*
4192	*Firmicutes*	*Bacilli*	*Lactobacillales*	*Lactobacillaceae*	*Lactobacillus*	*Lactobacillus*
4193	*Firmicutes*	*Bacilli*	*Lactobacillales*	*Lactobacillaceae*	*Lactobacillus*	*Lactobacillus*
4194	*Firmicutes*	*Bacilli*	*Lactobacillales*	*Lactobacillaceae*	*Lactobacillus*	*Lactobacillus*
4195	*Firmicutes*	*Bacilli*	*Lactobacillales*	*Lactobacillaceae*	*Lactobacillus*	*Lactobacillus*
4196	*Firmicutes*	*Bacilli*	*Lactobacillales*	*Lactobacillaceae*	*Lactobacillus*	*Lactobacillus*
4197	*Firmicutes*	*Bacilli*	*Lactobacillales*	*Lactobacillaceae*	*Lactobacillus*	*Lactobacillus*
4198	*Firmicutes*	*Bacilli*	*Lactobacillales*	*Lactobacillaceae*	*Lactobacillus*	*Lactobacillus*
4199	*Firmicutes*	*Bacilli*	*Lactobacillales*	*Lactobacillaceae*	*Lactobacillus*	*Lactobacillus*
4200	*Firmicutes*	*Bacilli*	*Lactobacillales*	*Lactobacillaceae*	*Lactobacillus*	*Lactobacillus*

Table S4. Isolated strains by culturomics

bacteriaId	*phylum*	*class*	*order*	*family*	*genus*	*species*
4201	*Firmicutes*	*Bacilli*	*Lactobacillales*	*Lactobacillaceae*	*Lactobacillus*	*Lactobacillus*
4202	*Firmicutes*	*Bacilli*	*Lactobacillales*	*Lactobacillaceae*	*Lactobacillus*	*Lactobacillus*
4203	*Firmicutes*	*Bacilli*	*Lactobacillales*	*Lactobacillaceae*	*Lactobacillus*	*Lactobacillus*
4204	*Firmicutes*	*Bacilli*	*Lactobacillales*	*Lactobacillaceae*	*Lactobacillus*	*Lactobacillus*
4205	*Firmicutes*	*Bacilli*	*Lactobacillales*	*Lactobacillaceae*	*Lactobacillus*	*Lactobacillus*
4206	*Firmicutes*	*Bacilli*	*Lactobacillales*	*Lactobacillaceae*	*Lactobacillus*	*Lactobacillus*
4207	*Firmicutes*	*Bacilli*	*Lactobacillales*	*Lactobacillaceae*	*Lactobacillus*	*Lactobacillus*
4208	*Firmicutes*	*Bacilli*	*Lactobacillales*	*Lactobacillaceae*	*Lactobacillus*	*Lactobacillus*
4209	*Firmicutes*	*Bacilli*	*Lactobacillales*	*Lactobacillaceae*	*Lactobacillus*	*Lactobacillus*
4210	*Firmicutes*	*Bacilli*	*Lactobacillales*	*Lactobacillaceae*	*Lactobacillus*	*Lactobacillus*
4211	*Firmicutes*	*Bacilli*	*Lactobacillales*	*Lactobacillaceae*	*Lactobacillus*	*Lactobacillus*
4212	*Firmicutes*	*Bacilli*	*Lactobacillales*	*Lactobacillaceae*	*Lactobacillus*	*Lactobacillus*
4213	*Firmicutes*	*Bacilli*	*Lactobacillales*	*Lactobacillaceae*	*Lactobacillus*	*Lactobacillus*
4214	*Firmicutes*	*Bacilli*	*Lactobacillales*	*Lactobacillaceae*	*Lactobacillus*	*Lactobacillus*
4215	*Firmicutes*	*Bacilli*	*Lactobacillales*	*Lactobacillaceae*	*Lactobacillus*	*Lactobacillus*
4216	*Firmicutes*	*Bacilli*	*Lactobacillales*	*Lactobacillaceae*	*Lactobacillus*	*Lactobacillus*
4217	*Firmicutes*	*Bacilli*	*Lactobacillales*	*Lactobacillaceae*	*Lactobacillus*	*Lactobacillus*
4218	*Firmicutes*	*Bacilli*	*Lactobacillales*	*Lactobacillaceae*	*Lactobacillus*	*Lactobacillus*
4219	*Firmicutes*	*Bacilli*	*Lactobacillales*	*Lactobacillaceae*	*Lactobacillus*	*Lactobacillus*
4220	*Firmicutes*	*Bacilli*	*Lactobacillales*	*Lactobacillaceae*	*Lactobacillus*	*Lactobacillus*
4221	*Firmicutes*	*Bacilli*	*Lactobacillales*	*Lactobacillaceae*	*Lactobacillus*	*Lactobacillus*
4222	*Firmicutes*	*Bacilli*	*Lactobacillales*	*Lactobacillaceae*	*Lactobacillus*	*Lactobacillus*
4223	*Firmicutes*	*Bacilli*	*Lactobacillales*	*Lactobacillaceae*	*Lactobacillus*	*Lactobacillus*
4224	*Firmicutes*	*Bacilli*	*Lactobacillales*	*Lactobacillaceae*	*Lactobacillus*	*Lactobacillus*
4225	*Firmicutes*	*Bacilli*	*Lactobacillales*	*Lactobacillaceae*	*Lactobacillus*	*Lactobacillus*
4226	*Firmicutes*	*Bacilli*	*Lactobacillales*	*Lactobacillaceae*	*Lactobacillus*	*Lactobacillus*
4227	*Firmicutes*	*Bacilli*	*Lactobacillales*	*Lactobacillaceae*	*Lactobacillus*	*Lactobacillus*
4228	*Firmicutes*	*Bacilli*	*Lactobacillales*	*Lactobacillaceae*	*Lactobacillus*	*Lactobacillus*

Table S4. Isolated strains by culturomics

bacteriaId	*phylum*	*class*	*order*	*family*	*genus*	*species*
4229	*Firmicutes*	*Bacilli*	*Lactobacillales*	*Lactobacillaceae*	*Lactobacillus*	*Lactobacillus*
4230	*Firmicutes*	*Bacilli*	*Lactobacillales*	*Lactobacillaceae*	*Lactobacillus*	*Lactobacillus*
4231	*Firmicutes*	*Bacilli*	*Lactobacillales*	*Lactobacillaceae*	*Lactobacillus*	*Lactobacillus*
4232	*Firmicutes*	*Bacilli*	*Lactobacillales*	*Lactobacillaceae*	*Lactobacillus*	*Lactobacillus*
4233	*Firmicutes*	*Bacilli*	*Lactobacillales*	*Lactobacillaceae*	*Lactobacillus*	*Lactobacillus*
4234	*Firmicutes*	*Bacilli*	*Lactobacillales*	*Lactobacillaceae*	*Lactobacillus*	*Lactobacillus*
4235	*Firmicutes*	*Bacilli*	*Lactobacillales*	*Lactobacillaceae*	*Lactobacillus*	*Lactobacillus*
4236	*Firmicutes*	*Bacilli*	*Lactobacillales*	*Lactobacillaceae*	*Lactobacillus*	*Lactobacillus*
4237	*Firmicutes*	*Bacilli*	*Lactobacillales*	*Lactobacillaceae*	*Lactobacillus*	*Lactobacillus*
4238	*Firmicutes*	*Bacilli*	*Lactobacillales*	*Lactobacillaceae*	*Lactobacillus*	*Lactobacillus*
4239	*Firmicutes*	*Bacilli*	*Lactobacillales*	*Lactobacillaceae*	*Lactobacillus*	*Lactobacillus*
4240	*Firmicutes*	*Bacilli*	*Lactobacillales*	*Lactobacillaceae*	*Lactobacillus*	*Lactobacillus*
4241	*Firmicutes*	*Bacilli*	*Lactobacillales*	*Lactobacillaceae*	*Lactobacillus*	*Lactobacillus*
4242	*Firmicutes*	*Bacilli*	*Lactobacillales*	*Lactobacillaceae*	*Lactobacillus*	*Lactobacillus*
4243	*Firmicutes*	*Bacilli*	*Lactobacillales*	*Lactobacillaceae*	*Lactobacillus*	*Lactobacillus*
4244	*Firmicutes*	*Bacilli*	*Lactobacillales*	*Lactobacillaceae*	*Lactobacillus*	*Lactobacillus*
4245	*Firmicutes*	*Bacilli*	*Lactobacillales*	*Lactobacillaceae*	*Lactobacillus*	*Lactobacillus*
4246	*Firmicutes*	*Bacilli*	*Lactobacillales*	*Lactobacillaceae*	*Lactobacillus*	*Lactobacillus*
4247	*Firmicutes*	*Bacilli*	*Lactobacillales*	*Lactobacillaceae*	*Lactobacillus*	*Lactobacillus*
4248	*Firmicutes*	*Bacilli*	*Lactobacillales*	*Lactobacillaceae*	*Lactobacillus*	*Lactobacillus*
4249	*Firmicutes*	*Bacilli*	*Lactobacillales*	*Lactobacillaceae*	*Lactobacillus*	*Lactobacillus*
4250	*Firmicutes*	*Bacilli*	*Lactobacillales*	*Lactobacillaceae*	*Lactobacillus*	*Lactobacillus*
4251	*Firmicutes*	*Bacilli*	*Lactobacillales*	*Lactobacillaceae*	*Lactobacillus*	*Lactobacillus*
4252	*Firmicutes*	*Bacilli*	*Lactobacillales*	*Lactobacillaceae*	*Lactobacillus*	*Lactobacillus*
4253	*Firmicutes*	*Bacilli*	*Lactobacillales*	*Lactobacillaceae*	*Lactobacillus*	*Lactobacillus*
4254	*Firmicutes*	*Bacilli*	*Lactobacillales*	*Lactobacillaceae*	*Lactobacillus*	*Lactobacillus*
4255	*Firmicutes*	*Bacilli*	*Lactobacillales*	*Lactobacillaceae*	*Lactobacillus*	*Lactobacillus*
4256	*Firmicutes*	*Bacilli*	*Lactobacillales*	*Lactobacillaceae*	*Lactobacillus*	*Lactobacillus*

Table S4. Isolated strains by culturomics

bacteriaId	*phylum*	*class*	*order*	*family*	*genus*	*species*
4257	*Firmicutes*	*Bacilli*	*Lactobacillales*	*Lactobacillaceae*	*Lactobacillus*	*Lactobacillus*
4258	*Firmicutes*	*Bacilli*	*Lactobacillales*	*Lactobacillaceae*	*Lactobacillus*	*Lactobacillus*
4259	*Firmicutes*	*Bacilli*	*Lactobacillales*	*Lactobacillaceae*	*Lactobacillus*	*Lactobacillus*
4260	*Firmicutes*	*Bacilli*	*Lactobacillales*	*Lactobacillaceae*	*Lactobacillus*	*Lactobacillus*
4261	*Firmicutes*	*Bacilli*	*Lactobacillales*	*Lactobacillaceae*	*Lactobacillus*	*Lactobacillus*
4262	*Firmicutes*	*Bacilli*	*Lactobacillales*	*Lactobacillaceae*	*Lactobacillus*	*Lactobacillus*
4263	*Firmicutes*	*Bacilli*	*Lactobacillales*	*Lactobacillaceae*	*Lactobacillus*	*Lactobacillus*
4264	*Firmicutes*	*Bacilli*	*Lactobacillales*	*Lactobacillaceae*	*Lactobacillus*	*Lactobacillus*
4265	*Firmicutes*	*Bacilli*	*Lactobacillales*	*Lactobacillaceae*	*Lactobacillus*	*Lactobacillus*
4266	*Firmicutes*	*Bacilli*	*Lactobacillales*	*Lactobacillaceae*	*Lactobacillus*	*Lactobacillus*
4267	*Firmicutes*	*Bacilli*	*Lactobacillales*	*Lactobacillaceae*	*Lactobacillus*	*Lactobacillus*
4268	*Firmicutes*	*Bacilli*	*Lactobacillales*	*Lactobacillaceae*	*Lactobacillus*	*Lactobacillus*
4269	*Firmicutes*	*Bacilli*	*Lactobacillales*	*Lactobacillaceae*	*Lactobacillus*	*Lactobacillus*
4270	*Firmicutes*	*Bacilli*	*Lactobacillales*	*Lactobacillaceae*	*Lactobacillus*	*Lactobacillus*
4271	*Firmicutes*	*Bacilli*	*Lactobacillales*	*Lactobacillaceae*	*Lactobacillus*	*Lactobacillus*
4272	*Firmicutes*	*Bacilli*	*Lactobacillales*	*Lactobacillaceae*	*Lactobacillus*	*Lactobacillus*
4273	*Firmicutes*	*Bacilli*	*Lactobacillales*	*Lactobacillaceae*	*Lactobacillus*	*Lactobacillus*
4274	*Firmicutes*	*Bacilli*	*Lactobacillales*	*Lactobacillaceae*	*Lactobacillus*	*Lactobacillus*
4275	*Firmicutes*	*Bacilli*	*Lactobacillales*	*Lactobacillaceae*	*Lactobacillus*	*Lactobacillus*
4276	*Firmicutes*	*Bacilli*	*Lactobacillales*	*Lactobacillaceae*	*Lactobacillus*	*Lactobacillus*
4277	*Firmicutes*	*Bacilli*	*Lactobacillales*	*Lactobacillaceae*	*Lactobacillus*	*Lactobacillus*
4278	*Firmicutes*	*Bacilli*	*Lactobacillales*	*Lactobacillaceae*	*Lactobacillus*	*Lactobacillus*
4279	*Firmicutes*	*Bacilli*	*Lactobacillales*	*Lactobacillaceae*	*Lactobacillus*	*Lactobacillus*
4280	*Firmicutes*	*Bacilli*	*Lactobacillales*	*Lactobacillaceae*	*Lactobacillus*	*Lactobacillus*
4281	*Firmicutes*	*Bacilli*	*Lactobacillales*	*Lactobacillaceae*	*Lactobacillus*	*Lactobacillus*
4282	*Firmicutes*	*Bacilli*	*Lactobacillales*	*Lactobacillaceae*	*Lactobacillus*	*Lactobacillus*
4283	*Firmicutes*	*Bacilli*	*Lactobacillales*	*Lactobacillaceae*	*Lactobacillus*	*Lactobacillus*
4284	*Firmicutes*	*Bacilli*	*Lactobacillales*	*Lactobacillaceae*	*Lactobacillus*	*Lactobacillus*

Table S4. Isolated strains by culturomics

bacteriaId	*phylum*	*class*	*order*	*family*	*genus*	*species*
4285	*Firmicutes*	*Bacilli*	*Lactobacillales*	*Lactobacillaceae*	*Lactobacillus*	*Lactobacillus*
4286	*Firmicutes*	*Bacilli*	*Lactobacillales*	*Lactobacillaceae*	*Lactobacillus*	*Lactobacillus*
4287	*Firmicutes*	*Bacilli*	*Lactobacillales*	*Lactobacillaceae*	*Lactobacillus*	*Lactobacillus*
4288	*Firmicutes*	*Bacilli*	*Lactobacillales*	*Lactobacillaceae*	*Lactobacillus*	*Lactobacillus*
4289	*Firmicutes*	*Bacilli*	*Lactobacillales*	*Lactobacillaceae*	*Lactobacillus*	*Lactobacillus*
4290	*Firmicutes*	*Bacilli*	*Lactobacillales*	*Lactobacillaceae*	*Lactobacillus*	*Lactobacillus*
4291	*Firmicutes*	*Bacilli*	*Lactobacillales*	*Lactobacillaceae*	*Lactobacillus*	*Lactobacillus*
4292	*Firmicutes*	*Bacilli*	*Lactobacillales*	*Lactobacillaceae*	*Lactobacillus*	*Lactobacillus*
4293	*Firmicutes*	*Bacilli*	*Lactobacillales*	*Lactobacillaceae*	*Lactobacillus*	*Lactobacillus*
4294	*Firmicutes*	*Bacilli*	*Lactobacillales*	*Lactobacillaceae*	*Lactobacillus*	*Lactobacillus*
4295	*Firmicutes*	*Bacilli*	*Lactobacillales*	*Lactobacillaceae*	*Lactobacillus*	*Lactobacillus*
4296	*Firmicutes*	*Bacilli*	*Lactobacillales*	*Lactobacillaceae*	*Lactobacillus*	*Lactobacillus*
4297	*Firmicutes*	*Bacilli*	*Lactobacillales*	*Lactobacillaceae*	*Lactobacillus*	*Lactobacillus*
4298	*Firmicutes*	*Bacilli*	*Lactobacillales*	*Lactobacillaceae*	*Lactobacillus*	*Lactobacillus*
4299	*Firmicutes*	*Bacilli*	*Lactobacillales*	*Lactobacillaceae*	*Lactobacillus*	*Lactobacillus*
4300	*Firmicutes*	*Bacilli*	*Lactobacillales*	*Lactobacillaceae*	*Lactobacillus*	*Lactobacillus*
4301	*Firmicutes*	*Bacilli*	*Lactobacillales*	*Lactobacillaceae*	*Lactobacillus*	*Lactobacillus*
4302	*Firmicutes*	*Bacilli*	*Lactobacillales*	*Lactobacillaceae*	*Lactobacillus*	*Lactobacillus*
4303	*Firmicutes*	*Bacilli*	*Lactobacillales*	*Lactobacillaceae*	*Lactobacillus*	*Lactobacillus*
4304	*Firmicutes*	*Bacilli*	*Lactobacillales*	*Lactobacillaceae*	*Lactobacillus*	*Lactobacillus*
4305	*Firmicutes*	*Bacilli*	*Lactobacillales*	*Lactobacillaceae*	*Lactobacillus*	*Lactobacillus*
4306	*Firmicutes*	*Bacilli*	*Lactobacillales*	*Lactobacillaceae*	*Lactobacillus*	*Lactobacillus*
4307	*Firmicutes*	*Bacilli*	*Lactobacillales*	*Lactobacillaceae*	*Lactobacillus*	*Lactobacillus*
4308	*Firmicutes*	*Bacilli*	*Lactobacillales*	*Lactobacillaceae*	*Lactobacillus*	*Lactobacillus*
4309	*Firmicutes*	*Bacilli*	*Lactobacillales*	*Lactobacillaceae*	*Lactobacillus*	*Lactobacillus*
4310	*Firmicutes*	*Bacilli*	*Lactobacillales*	*Lactobacillaceae*	*Lactobacillus*	*Lactobacillus*
4311	*Firmicutes*	*Bacilli*	*Lactobacillales*	*Lactobacillaceae*	*Lactobacillus*	*Lactobacillus*
4312	*Firmicutes*	*Bacilli*	*Lactobacillales*	*Lactobacillaceae*	*Lactobacillus*	*Lactobacillus*

Table S4. Isolated strains by culturomics

bacteriaId	*phylum*	*class*	*order*	*family*	*genus*	*species*
4313	*Firmicutes*	*Bacilli*	*Lactobacillales*	*Lactobacillaceae*	*Lactobacillus*	*Lactobacillus*
4314	*Firmicutes*	*Bacilli*	*Lactobacillales*	*Lactobacillaceae*	*Lactobacillus*	*Lactobacillus*
4315	*Firmicutes*	*Bacilli*	*Lactobacillales*	*Lactobacillaceae*	*Lactobacillus*	*Lactobacillus*
4316	*Firmicutes*	*Bacilli*	*Lactobacillales*	*Lactobacillaceae*	*Lactobacillus*	*Lactobacillus*
4317	*Firmicutes*	*Bacilli*	*Lactobacillales*	*Lactobacillaceae*	*Lactobacillus*	*Lactobacillus*
4318	*Firmicutes*	*Bacilli*	*Lactobacillales*	*Lactobacillaceae*	*Lactobacillus*	*Lactobacillus*
4319	*Firmicutes*	*Bacilli*	*Lactobacillales*	*Lactobacillaceae*	*Lactobacillus*	*Lactobacillus*
4320	*Firmicutes*	*Bacilli*	*Lactobacillales*	*Lactobacillaceae*	*Lactobacillus*	*Lactobacillus*
4321	*Firmicutes*	*Bacilli*	*Lactobacillales*	*Lactobacillaceae*	*Lactobacillus*	*Lactobacillus*
4322	*Firmicutes*	*Bacilli*	*Lactobacillales*	*Lactobacillaceae*	*Lactobacillus*	*Lactobacillus*
4323	*Firmicutes*	*Bacilli*	*Lactobacillales*	*Lactobacillaceae*	*Lactobacillus*	*Lactobacillus*
4324	*Firmicutes*	*Bacilli*	*Lactobacillales*	*Lactobacillaceae*	*Lactobacillus*	*Lactobacillus*
4325	*Firmicutes*	*Bacilli*	*Lactobacillales*	*Lactobacillaceae*	*Lactobacillus*	*Lactobacillus*
4326	*Firmicutes*	*Bacilli*	*Lactobacillales*	*Lactobacillaceae*	*Lactobacillus*	*Lactobacillus*
4327	*Firmicutes*	*Bacilli*	*Lactobacillales*	*Lactobacillaceae*	*Lactobacillus*	*Lactobacillus*
4328	*Firmicutes*	*Bacilli*	*Lactobacillales*	*Lactobacillaceae*	*Lactobacillus*	*Lactobacillus*
4329	*Firmicutes*	*Bacilli*	*Lactobacillales*	*Lactobacillaceae*	*Lactobacillus*	*Lactobacillus*
4330	*Firmicutes*	*Bacilli*	*Lactobacillales*	*Lactobacillaceae*	*Lactobacillus*	*Lactobacillus*
4331	*Firmicutes*	*Bacilli*	*Lactobacillales*	*Lactobacillaceae*	*Lactobacillus*	*Lactobacillus*
4332	*Firmicutes*	*Bacilli*	*Lactobacillales*	*Lactobacillaceae*	*Lactobacillus*	*Lactobacillus*
4333	*Firmicutes*	*Bacilli*	*Lactobacillales*	*Lactobacillaceae*	*Lactobacillus*	*Lactobacillus*
4334	*Firmicutes*	*Bacilli*	*Lactobacillales*	*Lactobacillaceae*	*Lactobacillus*	*Lactobacillus*
4335	*Firmicutes*	*Bacilli*	*Lactobacillales*	*Lactobacillaceae*	*Lactobacillus*	*Lactobacillus*
4336	*Firmicutes*	*Bacilli*	*Lactobacillales*	*Lactobacillaceae*	*Lactobacillus*	*Lactobacillus*
4337	*Firmicutes*	*Bacilli*	*Lactobacillales*	*Lactobacillaceae*	*Lactobacillus*	*Lactobacillus*
4338	*Firmicutes*	*Bacilli*	*Lactobacillales*	*Lactobacillaceae*	*Lactobacillus*	*Lactobacillus*
4339	*Firmicutes*	*Bacilli*	*Lactobacillales*	*Lactobacillaceae*	*Lactobacillus*	*Lactobacillus*
4340	*Firmicutes*	*Bacilli*	*Lactobacillales*	*Lactobacillaceae*	*Lactobacillus*	*Lactobacillus*

Table S4. Isolated strains by culturomics

bacteriaId	*phylum*	*class*	*order*	*family*	*genus*	*species*
4341	*Firmicutes*	*Bacilli*	*Lactobacillales*	*Lactobacillaceae*	*Lactobacillus*	*Lactobacillus*
4342	*Firmicutes*	*Bacilli*	*Lactobacillales*	*Lactobacillaceae*	*Lactobacillus*	*Lactobacillus*
4343	*Firmicutes*	*Bacilli*	*Lactobacillales*	*Lactobacillaceae*	*Lactobacillus*	*Lactobacillus*
4344	*Firmicutes*	*Bacilli*	*Lactobacillales*	*Lactobacillaceae*	*Lactobacillus*	*Lactobacillus*
4345	*Firmicutes*	*Bacilli*	*Lactobacillales*	*Lactobacillaceae*	*Lactobacillus*	*Lactobacillus*
4346	*Firmicutes*	*Bacilli*	*Lactobacillales*	*Lactobacillaceae*	*Lactobacillus*	*Lactobacillus*
4347	*Firmicutes*	*Bacilli*	*Lactobacillales*	*Lactobacillaceae*	*Lactobacillus*	*Lactobacillus*
4348	*Firmicutes*	*Bacilli*	*Lactobacillales*	*Lactobacillaceae*	*Lactobacillus*	*Lactobacillus*
4349	*Firmicutes*	*Bacilli*	*Lactobacillales*	*Lactobacillaceae*	*Lactobacillus*	*Lactobacillus*
4350	*Firmicutes*	*Bacilli*	*Lactobacillales*	*Lactobacillaceae*	*Lactobacillus*	*Lactobacillus*
4351	*Firmicutes*	*Bacilli*	*Lactobacillales*	*Lactobacillaceae*	*Lactobacillus*	*Lactobacillus*
4352	*Firmicutes*	*Bacilli*	*Lactobacillales*	*Lactobacillaceae*	*Lactobacillus*	*Lactobacillus*
4353	*Firmicutes*	*Bacilli*	*Lactobacillales*	*Lactobacillaceae*	*Lactobacillus*	*Lactobacillus*
4354	*Firmicutes*	*Bacilli*	*Lactobacillales*	*Lactobacillaceae*	*Lactobacillus*	*Lactobacillus*
4355	*Firmicutes*	*Bacilli*	*Lactobacillales*	*Lactobacillaceae*	*Lactobacillus*	*Lactobacillus*
4356	*Firmicutes*	*Bacilli*	*Lactobacillales*	*Lactobacillaceae*	*Lactobacillus*	*Lactobacillus*
4357	*Firmicutes*	*Bacilli*	*Lactobacillales*	*Lactobacillaceae*	*Lactobacillus*	*Lactobacillus*
4358	*Firmicutes*	*Bacilli*	*Lactobacillales*	*Lactobacillaceae*	*Lactobacillus*	*Lactobacillus*
4359	*Firmicutes*	*Bacilli*	*Lactobacillales*	*Lactobacillaceae*	*Lactobacillus*	*Lactobacillus*
4360	*Firmicutes*	*Bacilli*	*Lactobacillales*	*Lactobacillaceae*	*Lactobacillus*	*Lactobacillus*
4361	*Firmicutes*	*Bacilli*	*Lactobacillales*	*Lactobacillaceae*	*Lactobacillus*	*Lactobacillus*
4362	*Firmicutes*	*Bacilli*	*Lactobacillales*	*Lactobacillaceae*	*Lactobacillus*	*Lactobacillus*
4363	*Firmicutes*	*Bacilli*	*Lactobacillales*	*Lactobacillaceae*	*Lactobacillus*	*Lactobacillus*
4364	*Firmicutes*	*Bacilli*	*Lactobacillales*	*Lactobacillaceae*	*Lactobacillus*	*Lactobacillus*
4365	*Firmicutes*	*Bacilli*	*Lactobacillales*	*Lactobacillaceae*	*Lactobacillus*	*Lactobacillus*
4366	*Firmicutes*	*Bacilli*	*Lactobacillales*	*Lactobacillaceae*	*Lactobacillus*	*Lactobacillus*
4367	*Firmicutes*	*Bacilli*	*Lactobacillales*	*Lactobacillaceae*	*Lactobacillus*	*Lactobacillus*
4368	*Firmicutes*	*Bacilli*	*Lactobacillales*	*Lactobacillaceae*	*Lactobacillus*	*Lactobacillus*

Table S4. Isolated strains by culturomics

bacteriaId	*phylum*	*class*	*order*	*family*	*genus*	*species*
4369	*Firmicutes*	*Bacilli*	*Lactobacillales*	*Lactobacillaceae*	*Lactobacillus*	*Lactobacillus*
4370	*Firmicutes*	*Bacilli*	*Lactobacillales*	*Lactobacillaceae*	*Lactobacillus*	*Lactobacillus*
4371	*Firmicutes*	*Bacilli*	*Lactobacillales*	*Lactobacillaceae*	*Lactobacillus*	*Lactobacillus*
4372	*Firmicutes*	*Bacilli*	*Lactobacillales*	*Lactobacillaceae*	*Lactobacillus*	*Lactobacillus*
4373	*Firmicutes*	*Bacilli*	*Lactobacillales*	*Lactobacillaceae*	*Lactobacillus*	*Lactobacillus*
4374	*Firmicutes*	*Bacilli*	*Lactobacillales*	*Lactobacillaceae*	*Lactobacillus*	*Lactobacillus*
4375	*Firmicutes*	*Bacilli*	*Lactobacillales*	*Lactobacillaceae*	*Lactobacillus*	*Lactobacillus*
4376	*Firmicutes*	*Bacilli*	*Lactobacillales*	*Lactobacillaceae*	*Lactobacillus*	*Lactobacillus*
4377	*Firmicutes*	*Bacilli*	*Lactobacillales*	*Lactobacillaceae*	*Lactobacillus*	*Lactobacillus*
4378	*Firmicutes*	*Bacilli*	*Lactobacillales*	*Lactobacillaceae*	*Lactobacillus*	*Lactobacillus*
4379	*Firmicutes*	*Bacilli*	*Lactobacillales*	*Lactobacillaceae*	*Lactobacillus*	*Lactobacillus*
4380	*Firmicutes*	*Bacilli*	*Lactobacillales*	*Lactobacillaceae*	*Lactobacillus*	*Lactobacillus*
4381	*Firmicutes*	*Bacilli*	*Lactobacillales*	*Lactobacillaceae*	*Lactobacillus*	*Lactobacillus*
4382	*Firmicutes*	*Bacilli*	*Lactobacillales*	*Lactobacillaceae*	*Lactobacillus*	*Lactobacillus*
4383	*Firmicutes*	*Bacilli*	*Lactobacillales*	*Lactobacillaceae*	*Lactobacillus*	*Lactobacillus*
4384	*Firmicutes*	*Bacilli*	*Lactobacillales*	*Lactobacillaceae*	*Lactobacillus*	*Lactobacillus*
4385	*Firmicutes*	*Bacilli*	*Lactobacillales*	*Lactobacillaceae*	*Lactobacillus*	*Lactobacillus*
4386	*Firmicutes*	*Bacilli*	*Lactobacillales*	*Lactobacillaceae*	*Lactobacillus*	*Lactobacillus*
4387	*Firmicutes*	*Bacilli*	*Lactobacillales*	*Lactobacillaceae*	*Lactobacillus*	*Lactobacillus*
4388	*Firmicutes*	*Bacilli*	*Lactobacillales*	*Lactobacillaceae*	*Lactobacillus*	*Lactobacillus*
4389	*Firmicutes*	*Bacilli*	*Lactobacillales*	*Lactobacillaceae*	*Lactobacillus*	*Lactobacillus*
4390	*Firmicutes*	*Bacilli*	*Lactobacillales*	*Lactobacillaceae*	*Lactobacillus*	*Lactobacillus*
4391	*Firmicutes*	*Bacilli*	*Lactobacillales*	*Lactobacillaceae*	*Lactobacillus*	*Lactobacillus*
4392	*Firmicutes*	*Bacilli*	*Lactobacillales*	*Lactobacillaceae*	*Lactobacillus*	*Lactobacillus*
4393	*Firmicutes*	*Bacilli*	*Lactobacillales*	*Lactobacillaceae*	*Lactobacillus*	*Lactobacillus*
4394	*Firmicutes*	*Bacilli*	*Lactobacillales*	*Lactobacillaceae*	*Lactobacillus*	*Lactobacillus*
4395	*Firmicutes*	*Bacilli*	*Lactobacillales*	*Lactobacillaceae*	*Lactobacillus*	*Lactobacillus*
4396	*Firmicutes*	*Bacilli*	*Lactobacillales*	*Lactobacillaceae*	*Lactobacillus*	*Lactobacillus*

Table S4. Isolated strains by culturomics

bacteriaId	*phylum*	*class*	*order*	*family*	*genus*	*species*
4397	*Firmicutes*	*Bacilli*	*Lactobacillales*	*Lactobacillaceae*	*Lactobacillus*	*Lactobacillus*
4398	*Firmicutes*	*Bacilli*	*Lactobacillales*	*Lactobacillaceae*	*Lactobacillus*	*Lactobacillus*
4399	*Firmicutes*	*Bacilli*	*Lactobacillales*	*Lactobacillaceae*	*Lactobacillus*	*Lactobacillus*
4400	*Firmicutes*	*Bacilli*	*Lactobacillales*	*Lactobacillaceae*	*Lactobacillus*	*Lactobacillus*
4401	*Firmicutes*	*Bacilli*	*Lactobacillales*	*Lactobacillaceae*	*Lactobacillus*	*Lactobacillus*
4402	*Firmicutes*	*Bacilli*	*Lactobacillales*	*Lactobacillaceae*	*Lactobacillus*	*Lactobacillus*
4403	*Firmicutes*	*Bacilli*	*Lactobacillales*	*Lactobacillaceae*	*Lactobacillus*	*Lactobacillus*
4404	*Firmicutes*	*Bacilli*	*Lactobacillales*	*Lactobacillaceae*	*Lactobacillus*	*Lactobacillus*
4405	*Firmicutes*	*Bacilli*	*Lactobacillales*	*Lactobacillaceae*	*Lactobacillus*	*Lactobacillus*
4406	*Firmicutes*	*Bacilli*	*Lactobacillales*	*Lactobacillaceae*	*Lactobacillus*	*Lactobacillus*
4407	*Firmicutes*	*Bacilli*	*Lactobacillales*	*Lactobacillaceae*	*Lactobacillus*	*Lactobacillus*
4408	*Firmicutes*	*Bacilli*	*Lactobacillales*	*Lactobacillaceae*	*Lactobacillus*	*Lactobacillus*
4409	*Firmicutes*	*Bacilli*	*Lactobacillales*	*Lactobacillaceae*	*Lactobacillus*	*Lactobacillus*
4410	*Firmicutes*	*Bacilli*	*Lactobacillales*	*Lactobacillaceae*	*Lactobacillus*	*Lactobacillus*
4411	*Firmicutes*	*Bacilli*	*Lactobacillales*	*Lactobacillaceae*	*Lactobacillus*	*Lactobacillus*
4412	*Firmicutes*	*Bacilli*	*Lactobacillales*	*Lactobacillaceae*	*Lactobacillus*	*Lactobacillus*
4413	*Firmicutes*	*Bacilli*	*Lactobacillales*	*Lactobacillaceae*	*Lactobacillus*	*Lactobacillus*
4414	*Firmicutes*	*Bacilli*	*Lactobacillales*	*Lactobacillaceae*	*Lactobacillus*	*Lactobacillus*
4415	*Firmicutes*	*Bacilli*	*Lactobacillales*	*Lactobacillaceae*	*Lactobacillus*	*Lactobacillus*
4416	*Firmicutes*	*Bacilli*	*Lactobacillales*	*Lactobacillaceae*	*Lactobacillus*	*Lactobacillus*
4417	*Firmicutes*	*Bacilli*	*Lactobacillales*	*Lactobacillaceae*	*Lactobacillus*	*Lactobacillus*
4418	*Firmicutes*	*Bacilli*	*Lactobacillales*	*Lactobacillaceae*	*Lactobacillus*	*Lactobacillus*
4419	*Firmicutes*	*Bacilli*	*Lactobacillales*	*Lactobacillaceae*	*Lactobacillus*	*Lactobacillus*
4420	*Firmicutes*	*Bacilli*	*Lactobacillales*	*Lactobacillaceae*	*Lactobacillus*	*Lactobacillus*
4421	*Firmicutes*	*Bacilli*	*Lactobacillales*	*Lactobacillaceae*	*Lactobacillus*	*Lactobacillus*
4422	*Firmicutes*	*Bacilli*	*Lactobacillales*	*Lactobacillaceae*	*Lactobacillus*	*Lactobacillus*
4423	*Firmicutes*	*Bacilli*	*Lactobacillales*	*Lactobacillaceae*	*Lactobacillus*	*Lactobacillus*
4424	*Firmicutes*	*Bacilli*	*Lactobacillales*	*Lactobacillaceae*	*Lactobacillus*	*Lactobacillus*

Table S4. Isolated strains by culturomics

bacteriaId	*phylum*	*class*	*order*	*family*	*genus*	*species*
4425	*Firmicutes*	*Bacilli*	*Lactobacillales*	*Lactobacillaceae*	*Lactobacillus*	*Lactobacillus*
4426	*Firmicutes*	*Bacilli*	*Lactobacillales*	*Lactobacillaceae*	*Lactobacillus*	*Lactobacillus*
4427	*Firmicutes*	*Bacilli*	*Lactobacillales*	*Lactobacillaceae*	*Lactobacillus*	*Lactobacillus*
4428	*Firmicutes*	*Bacilli*	*Lactobacillales*	*Lactobacillaceae*	*Lactobacillus*	*Lactobacillus*
4429	*Firmicutes*	*Bacilli*	*Lactobacillales*	*Lactobacillaceae*	*Lactobacillus*	*Lactobacillus*
4430	*Firmicutes*	*Bacilli*	*Lactobacillales*	*Lactobacillaceae*	*Lactobacillus*	*Lactobacillus*
4431	*Firmicutes*	*Bacilli*	*Lactobacillales*	*Lactobacillaceae*	*Lactobacillus*	*Lactobacillus*
4432	*Firmicutes*	*Bacilli*	*Lactobacillales*	*Lactobacillaceae*	*Lactobacillus*	*Lactobacillus*
4433	*Firmicutes*	*Bacilli*	*Lactobacillales*	*Lactobacillaceae*	*Lactobacillus*	*Lactobacillus*
4434	*Firmicutes*	*Bacilli*	*Lactobacillales*	*Lactobacillaceae*	*Lactobacillus*	*Lactobacillus*
4435	*Firmicutes*	*Bacilli*	*Lactobacillales*	*Lactobacillaceae*	*Lactobacillus*	*Lactobacillus*
4436	*Firmicutes*	*Bacilli*	*Lactobacillales*	*Lactobacillaceae*	*Lactobacillus*	*Lactobacillus*
4437	*Firmicutes*	*Bacilli*	*Lactobacillales*	*Lactobacillaceae*	*Lactobacillus*	*Lactobacillus*
4438	*Firmicutes*	*Bacilli*	*Lactobacillales*	*Lactobacillaceae*	*Lactobacillus*	*Lactobacillus*
4439	*Firmicutes*	*Bacilli*	*Lactobacillales*	*Lactobacillaceae*	*Lactobacillus*	*Lactobacillus*
4440	*Firmicutes*	*Bacilli*	*Lactobacillales*	*Lactobacillaceae*	*Lactobacillus*	*Lactobacillus*
4441	*Firmicutes*	*Bacilli*	*Lactobacillales*	*Lactobacillaceae*	*Lactobacillus*	*Lactobacillus*
4442	*Firmicutes*	*Bacilli*	*Lactobacillales*	*Lactobacillaceae*	*Lactobacillus*	*Lactobacillus*
4443	*Firmicutes*	*Bacilli*	*Lactobacillales*	*Lactobacillaceae*	*Lactobacillus*	*Lactobacillus*
4444	*Firmicutes*	*Bacilli*	*Lactobacillales*	*Lactobacillaceae*	*Lactobacillus*	*Lactobacillus*
4445	*Firmicutes*	*Bacilli*	*Lactobacillales*	*Lactobacillaceae*	*Lactobacillus*	*Lactobacillus*
4446	*Firmicutes*	*Bacilli*	*Lactobacillales*	*Lactobacillaceae*	*Lactobacillus*	*Lactobacillus*
4447	*Firmicutes*	*Bacilli*	*Lactobacillales*	*Lactobacillaceae*	*Lactobacillus*	*Lactobacillus*
4448	*Firmicutes*	*Bacilli*	*Lactobacillales*	*Lactobacillaceae*	*Lactobacillus*	*Lactobacillus*
4449	*Firmicutes*	*Bacilli*	*Lactobacillales*	*Lactobacillaceae*	*Lactobacillus*	*Lactobacillus*
4450	*Firmicutes*	*Bacilli*	*Lactobacillales*	*Lactobacillaceae*	*Lactobacillus*	*Lactobacillus*
4451	*Firmicutes*	*Bacilli*	*Lactobacillales*	*Lactobacillaceae*	*Lactobacillus*	*Lactobacillus*
4452	*Firmicutes*	*Bacilli*	*Lactobacillales*	*Lactobacillaceae*	*Lactobacillus*	*Lactobacillus*

Table S4. Isolated strains by culturomics

bacteriaId	*phylum*	*class*	*order*	*family*	*genus*	*species*
4453	*Firmicutes*	*Bacilli*	*Lactobacillales*	*Lactobacillaceae*	*Lactobacillus*	*Lactobacillus*
4454	*Firmicutes*	*Bacilli*	*Lactobacillales*	*Lactobacillaceae*	*Lactobacillus*	*Lactobacillus*
4455	*Firmicutes*	*Bacilli*	*Lactobacillales*	*Lactobacillaceae*	*Lactobacillus*	*Lactobacillus*
4456	*Firmicutes*	*Bacilli*	*Lactobacillales*	*Lactobacillaceae*	*Lactobacillus*	*Lactobacillus*
4457	*Firmicutes*	*Bacilli*	*Lactobacillales*	*Lactobacillaceae*	*Lactobacillus*	*Lactobacillus*
4458	*Firmicutes*	*Bacilli*	*Lactobacillales*	*Lactobacillaceae*	*Lactobacillus*	*Lactobacillus*
4459	*Firmicutes*	*Bacilli*	*Lactobacillales*	*Lactobacillaceae*	*Lactobacillus*	*Lactobacillus*
4460	*Firmicutes*	*Bacilli*	*Lactobacillales*	*Lactobacillaceae*	*Lactobacillus*	*Lactobacillus*
4461	*Firmicutes*	*Bacilli*	*Lactobacillales*	*Lactobacillaceae*	*Lactobacillus*	*Lactobacillus*
4462	*Firmicutes*	*Bacilli*	*Lactobacillales*	*Lactobacillaceae*	*Lactobacillus*	*Lactobacillus*
4463	*Firmicutes*	*Bacilli*	*Lactobacillales*	*Lactobacillaceae*	*Lactobacillus*	*Lactobacillus*
4464	*Firmicutes*	*Bacilli*	*Lactobacillales*	*Lactobacillaceae*	*Lactobacillus*	*Lactobacillus*
4465	*Firmicutes*	*Bacilli*	*Lactobacillales*	*Lactobacillaceae*	*Lactobacillus*	*Lactococcus lactis*
4466	*Firmicutes*	*Bacilli*	*Lactobacillales*	*Lactobacillaceae*	*Pediococcus*	*Pediococcus*
4467	*Firmicutes*	*Bacilli*	*Lactobacillales*	*Lactobacillaceae*	*Pediococcus*	*Pediococcus*
4468	*Firmicutes*	*Bacilli*	*Lactobacillales*	*Leuconostocaceae*	*Weissella*	*Weissella cibaria*
4469	*Firmicutes*	*Bacilli*	*Lactobacillales*	*Leuconostocaceae*	*Weissella*	*Weissella confusa*
4470	*Firmicutes*	*Bacilli*	*Lactobacillales*	*Leuconostocaceae*	*Weissella*	*Weissella confusa*
4471	*Firmicutes*	*Bacilli*	*Lactobacillales*	*Leuconostocaceae*	*Weissella*	*Weissella confusa*
4472	*Firmicutes*	*Bacilli*	*Lactobacillales*	*Leuconostocaceae*	*Weissella*	*Weissella confusa*
4473	*Firmicutes*	*Bacilli*	*Lactobacillales*	*Leuconostocaceae*	*Weissella*	*Weissella confusa*
4474	*Firmicutes*	*Bacilli*	*Lactobacillales*	*Leuconostocaceae*	*Weissella*	*Weissella confusa*
4475	*Firmicutes*	*Bacilli*	*Lactobacillales*	*Leuconostocaceae*	*Weissella*	*Weissella confusa*
4476	*Firmicutes*	*Bacilli*	*Lactobacillales*	*Leuconostocaceae*	*Weissella*	*Weissella confusa*
4477	*Firmicutes*	*Bacilli*	*Lactobacillales*	*Leuconostocaceae*	*Weissella*	*Weissella confusa*
4478	*Firmicutes*	*Bacilli*	*Lactobacillales*	*Leuconostocaceae*	*Weissella*	*Weissella confusa*
4479	*Firmicutes*	*Bacilli*	*Lactobacillales*	*Leuconostocaceae*	*Weissella*	*Weissella confusa*
4480	*Firmicutes*	*Bacilli*	*Lactobacillales*	*Leuconostocaceae*	*Weissella*	*Weissella confusa*

Table S4. Isolated strains by culturomics

bacteriaId	*phylum*	*class*	*order*	*family*	*genus*	*species*
4481	*Firmicutes*	*Bacilli*	*Lactobacillales*	*Leuconostocaceae*	*Weissella*	*Weissella confusa*
4482	*Firmicutes*	*Bacilli*	*Lactobacillales*	*Leuconostocaceae*	*Weissella*	*Weissella confusa*
4483	*Firmicutes*	*Bacilli*	*Lactobacillales*	*Leuconostocaceae*	*Weissella*	*Weissella confusa*
4484	*Firmicutes*	*Bacilli*	*Lactobacillales*	*Leuconostocaceae*	*Weissella*	*Weissella confusa*
4485	*Firmicutes*	*Bacilli*	*Lactobacillales*	*Leuconostocaceae*	*Weissella*	*Weissella confusa*
4486	*Firmicutes*	*Bacilli*	*Lactobacillales*	*Leuconostocaceae*	*Weissella*	*Weissella confusa*
4487	*Firmicutes*	*Bacilli*	*Lactobacillales*	*Leuconostocaceae*	*Weissella*	*Weissella confusa*
4488	*Firmicutes*	*Bacilli*	*Lactobacillales*	*Leuconostocaceae*	*Weissella*	*Weissella confusa*
4489	*Firmicutes*	*Bacilli*	*Lactobacillales*	*Leuconostocaceae*	*Weissella*	*Weissella confusa*
4490	*Firmicutes*	*Bacilli*	*Lactobacillales*	*Leuconostocaceae*	*Weissella*	*Weissella confusa*
4491	*Firmicutes*	*Bacilli*	*Lactobacillales*	*Leuconostocaceae*	*Weissella*	*Weissella confusa*
4492	*Firmicutes*	*Bacilli*	*Lactobacillales*	*Leuconostocaceae*	*Weissella*	*Weissella confusa*
4493	*Firmicutes*	*Bacilli*	*Lactobacillales*	*Leuconostocaceae*	*Weissella*	*Weissella confusa*
4494	*Firmicutes*	*Bacilli*	*Lactobacillales*	*Streptococcaceae*	*Lactococcus*	*Lactococcus*
4495	*Firmicutes*	*Bacilli*	*Lactobacillales*	*Streptococcaceae*	*Lactococcus*	*Lactococcus*
4496	*Firmicutes*	*Bacilli*	*Lactobacillales*	*Streptococcaceae*	*Lactococcus*	*Lactococcus*
4497	*Firmicutes*	*Bacilli*	*Lactobacillales*	*Streptococcaceae*	*Lactococcus*	*Lactococcus*
4498	*Firmicutes*	*Bacilli*	*Lactobacillales*	*Streptococcaceae*	*Lactococcus*	*Lactococcus*
4499	*Firmicutes*	*Bacilli*	*Lactobacillales*	*Streptococcaceae*	*Lactococcus*	*Lactococcus*
4500	*Firmicutes*	*Bacilli*	*Lactobacillales*	*Streptococcaceae*	*Lactococcus*	*Lactococcus*
4501	*Firmicutes*	*Bacilli*	*Lactobacillales*	*Streptococcaceae*	*Lactococcus*	*Lactococcus*
4502	*Firmicutes*	*Bacilli*	*Lactobacillales*	*Streptococcaceae*	*Lactococcus*	*Lactococcus*
4503	*Firmicutes*	*Bacilli*	*Lactobacillales*	*Streptococcaceae*	*Lactococcus*	*Lactococcus*
4504	*Firmicutes*	*Bacilli*	*Lactobacillales*	*Streptococcaceae*	*Lactococcus*	*Lactococcus*
4505	*Firmicutes*	*Bacilli*	*Lactobacillales*	*Streptococcaceae*	*Lactococcus*	*Lactococcus*
4506	*Firmicutes*	*Bacilli*	*Lactobacillales*	*Streptococcaceae*	*Lactococcus*	*Lactococcus lactis*
4507	*Firmicutes*	*Bacilli*	*Lactobacillales*	*Streptococcaceae*	*Lactococcus*	*Lactococcus lactis*
4508	*Firmicutes*	*Bacilli*	*Lactobacillales*	*Streptococcaceae*	*Lactococcus*	*Lactococcus lactis*

Table S4. Isolated strains by culturomics

bacteriaId	*phylum*	*class*	*order*	*family*	*genus*	*species*
4509	*Firmicutes*	*Bacilli*	*Lactobacillales*	*Streptococcaceae*	*Lactococcus*	*Lactococcus lactis*
4510	*Firmicutes*	*Bacilli*	*Lactobacillales*	*Streptococcaceae*	*Lactococcus*	*Lactococcus lactis*
4511	*Firmicutes*	*Bacilli*	*Lactobacillales*	*Streptococcaceae*	*Lactococcus*	*Lactococcus lactis*
4512	*Firmicutes*	*Bacilli*	*Lactobacillales*	*Streptococcaceae*	*Lactococcus*	*Lactococcus lactis*
4513	*Firmicutes*	*Bacilli*	*Lactobacillales*	*Streptococcaceae*	*Lactococcus*	*Lactococcus lactis*
4514	*Firmicutes*	*Bacilli*	*Lactobacillales*	*Streptococcaceae*	*Lactococcus*	*Lactococcus lactis*
4515	*Firmicutes*	*Bacilli*	*Lactobacillales*	*Streptococcaceae*	*Lactococcus*	*Lactococcus lactis*
4516	*Firmicutes*	*Bacilli*	*Lactobacillales*	*Streptococcaceae*	*Lactococcus*	*Lactococcus lactis*
4517	*Firmicutes*	*Bacilli*	*Lactobacillales*	*Streptococcaceae*	*Lactococcus*	*Lactococcus lactis*
4518	*Firmicutes*	*Bacilli*	*Lactobacillales*	*Streptococcaceae*	*Lactococcus*	*Lactococcus lactis*
4519	*Firmicutes*	*Bacilli*	*Lactobacillales*	*Streptococcaceae*	*Lactococcus*	*Lactococcus lactis*
4520	*Firmicutes*	*Bacilli*	*Lactobacillales*	*Streptococcaceae*	*Lactococcus*	*Lactococcus lactis*
4521	*Firmicutes*	*Bacilli*	*Lactobacillales*	*Streptococcaceae*	*Streptococcus*	*Streptococcus*
4522	*Firmicutes*	*Bacilli*	*Lactobacillales*	*Streptococcaceae*	*Streptococcus*	*Streptococcus*
4523	*Firmicutes*	*Bacilli*	*Lactobacillales*	*Streptococcaceae*	*Streptococcus*	*Streptococcus*
4524	*Firmicutes*	*Bacilli*	*Lactobacillales*	*Streptococcaceae*	*Streptococcus*	*Streptococcus*
4525	*Firmicutes*	*Bacilli*	*Lactobacillales*	*Streptococcaceae*	*Streptococcus*	*Streptococcus*
4526	*Firmicutes*	*Bacilli*	*Lactobacillales*	*Streptococcaceae*	*Streptococcus*	*Streptococcus*
4527	*Firmicutes*	*Bacilli*	*Lactobacillales*	*Streptococcaceae*	*Streptococcus*	*Streptococcus*
4528	*Firmicutes*	*Bacilli*	*Lactobacillales*	*Streptococcaceae*	*Streptococcus*	*Streptococcus*
4529	*Firmicutes*	*Bacilli*	*Lactobacillales*	*Streptococcaceae*	*Streptococcus*	*Streptococcus*
4530	*Firmicutes*	*Bacilli*	*Lactobacillales*	*Streptococcaceae*	*Streptococcus*	*Streptococcus*
4531	*Firmicutes*	*Bacilli*	*Lactobacillales*	*Streptococcaceae*	*Streptococcus*	*Streptococcus*
4532	*Firmicutes*	*Bacilli*	*Lactobacillales*	*Streptococcaceae*	*Streptococcus*	*Streptococcus*
4533	*Firmicutes*	*Bacilli*	*Lactobacillales*	*Streptococcaceae*	*Streptococcus*	*Streptococcus*
4534	*Firmicutes*	*Bacilli*	*Lactobacillales*	*Streptococcaceae*	*Streptococcus*	*Streptococcus*
4535	*Firmicutes*	*Bacilli*	*Lactobacillales*	*Streptococcaceae*	*Streptococcus*	*Streptococcus*
4536	*Firmicutes*	*Bacilli*	*Lactobacillales*	*Streptococcaceae*	*Streptococcus*	*Streptococcus*

Table S4. Isolated strains by culturomics

bacteriaId	*phylum*	*class*	*order*	*family*	*genus*	*species*
4537	*Firmicutes*	*Bacilli*	*Lactobacillales*	*Streptococcaceae*	*Streptococcus*	*Streptococcus*
4538	*Firmicutes*	*Bacilli*	*Lactobacillales*	*Streptococcaceae*	*Streptococcus*	*Streptococcus*
4539	*Firmicutes*	*Bacilli*	*Lactobacillales*	*Streptococcaceae*	*Streptococcus*	*Streptococcus*
4540	*Firmicutes*	*Bacilli*	*Lactobacillales*	*Streptococcaceae*	*Streptococcus*	*Streptococcus*
4541	*Firmicutes*	*Bacilli*	*Lactobacillales*	*Streptococcaceae*	*Streptococcus*	*Streptococcus*
4542	*Firmicutes*	*Bacilli*	*Lactobacillales*	*Streptococcaceae*	*Streptococcus*	*Streptococcus*
4543	*Firmicutes*	*Bacilli*	*Lactobacillales*	*Streptococcaceae*	*Streptococcus*	*Streptococcus*
4544	*Firmicutes*	*Bacilli*	*Lactobacillales*	*Streptococcaceae*	*Streptococcus*	*Streptococcus*
4545	*Firmicutes*	*Bacilli*	*Lactobacillales*	*Streptococcaceae*	*Streptococcus*	*Streptococcus*
4546	*Firmicutes*	*Bacilli*	*Lactobacillales*	*Streptococcaceae*	*Streptococcus*	*Streptococcus*
4547	*Firmicutes*	*Bacilli*	*Lactobacillales*	*Streptococcaceae*	*Streptococcus*	*Streptococcus*
4548	*Firmicutes*	*Bacilli*	*Lactobacillales*	*Streptococcaceae*	*Streptococcus*	*Streptococcus*
4549	*Firmicutes*	*Bacilli*	*Lactobacillales*	*Streptococcaceae*	*Streptococcus*	*Streptococcus*
4550	*Firmicutes*	*Bacilli*	*Lactobacillales*	*Streptococcaceae*	*Streptococcus*	*Streptococcus*
4551	*Firmicutes*	*Bacilli*	*Lactobacillales*	*Streptococcaceae*	*Streptococcus*	*Streptococcus*
4552	*Firmicutes*	*Bacilli*	*Lactobacillales*	*Streptococcaceae*	*Streptococcus*	*Streptococcus*
4553	*Firmicutes*	*Bacilli*	*Lactobacillales*	*Streptococcaceae*	*Streptococcus*	*Streptococcus*
4554	*Firmicutes*	*Bacilli*	*Lactobacillales*	*Streptococcaceae*	*Streptococcus*	*Streptococcus*
4555	*Firmicutes*	*Bacilli*	*Lactobacillales*	*Streptococcaceae*	*Streptococcus*	*Streptococcus*
4556	*Firmicutes*	*Bacilli*	*Lactobacillales*	*Streptococcaceae*	*Streptococcus*	*Streptococcus*
4557	*Firmicutes*	*Bacilli*	*Lactobacillales*	*Streptococcaceae*	*Streptococcus*	*Streptococcus*
4558	*Firmicutes*	*Bacilli*	*Lactobacillales*	*Streptococcaceae*	*Streptococcus*	*Streptococcus*
4559	*Firmicutes*	*Bacilli*	*Lactobacillales*	*Streptococcaceae*	*Streptococcus*	*Streptococcus*
4560	*Firmicutes*	*Bacilli*	*Lactobacillales*	*Streptococcaceae*	*Streptococcus*	*Streptococcus*
4561	*Firmicutes*	*Bacilli*	*Lactobacillales*	*Streptococcaceae*	*Streptococcus*	*Streptococcus*
4562	*Firmicutes*	*Bacilli*	*Lactobacillales*	*Streptococcaceae*	*Streptococcus*	*Streptococcus*
4563	*Firmicutes*	*Bacilli*	*Lactobacillales*	*Streptococcaceae*	*Streptococcus*	*Streptococcus*
4564	*Firmicutes*	*Bacilli*	*Lactobacillales*	*Streptococcaceae*	*Streptococcus*	*Streptococcus*

Table S4. Isolated strains by culturomics

bacteriaId	*phylum*	*class*	*order*	*family*	*genus*	*species*
4565	*Firmicutes*	*Bacilli*	*Lactobacillales*	*Streptococcaceae*	*Streptococcus*	*Streptococcus*
4566	*Firmicutes*	*Bacilli*	*Lactobacillales*	*Streptococcaceae*	*Streptococcus*	*Streptococcus*
4567	*Firmicutes*	*Bacilli*	*Lactobacillales*	*Streptococcaceae*	*Streptococcus*	*Streptococcus*
4568	*Firmicutes*	*Bacilli*	*Lactobacillales*	*Streptococcaceae*	*Streptococcus*	*Streptococcus*
4569	*Firmicutes*	*Bacilli*	*Lactobacillales*	*Streptococcaceae*	*Streptococcus*	*Streptococcus*
4570	*Firmicutes*	*Bacilli*	*Lactobacillales*	*Streptococcaceae*	*Streptococcus*	*Streptococcus*
4571	*Firmicutes*	*Bacilli*	*Lactobacillales*	*Streptococcaceae*	*Streptococcus*	*Streptococcus*
4572	*Firmicutes*	*Bacilli*	*Lactobacillales*	*Streptococcaceae*	*Streptococcus*	*Streptococcus*
4573	*Firmicutes*	*Bacilli*	*Lactobacillales*	*Streptococcaceae*	*Streptococcus*	*Streptococcus*
4574	*Firmicutes*	*Bacilli*	*Lactobacillales*	*Streptococcaceae*	*Streptococcus*	*Streptococcus*
4575	*Firmicutes*	*Bacilli*	*Lactobacillales*	*Streptococcaceae*	*Streptococcus*	*Streptococcus*
4576	*Firmicutes*	*Bacilli*	*Lactobacillales*	*Streptococcaceae*	*Streptococcus*	*Streptococcus*
4577	*Firmicutes*	*Bacilli*	*Lactobacillales*	*Streptococcaceae*	*Streptococcus*	*Streptococcus*
4578	*Firmicutes*	*Bacilli*	*Lactobacillales*	*Streptococcaceae*	*Streptococcus*	*Streptococcus*
4579	*Firmicutes*	*Bacilli*	*Lactobacillales*	*Streptococcaceae*	*Streptococcus*	*Streptococcus*
4580	*Firmicutes*	*Bacilli*	*Lactobacillales*	*Streptococcaceae*	*Streptococcus*	*Streptococcus*
4581	*Firmicutes*	*Bacilli*	*Lactobacillales*	*Streptococcaceae*	*Streptococcus*	*Streptococcus*
4582	*Firmicutes*	*Bacilli*	*Lactobacillales*	*Streptococcaceae*	*Streptococcus*	*Streptococcus*
4583	*Firmicutes*	*Bacilli*	*Lactobacillales*	*Streptococcaceae*	*Streptococcus*	*Streptococcus*
4584	*Firmicutes*	*Bacilli*	*Lactobacillales*	*Streptococcaceae*	*Streptococcus*	*Streptococcus*
4585	*Firmicutes*	*Bacilli*	*Lactobacillales*	*Streptococcaceae*	*Streptococcus*	*Streptococcus*
4586	*Firmicutes*	*Bacilli*	*Lactobacillales*	*Streptococcaceae*	*Streptococcus*	*Streptococcus*
4587	*Firmicutes*	*Bacilli*	*Lactobacillales*	*Streptococcaceae*	*Streptococcus*	*Streptococcus*
4588	*Firmicutes*	*Bacilli*	*Lactobacillales*	*Streptococcaceae*	*Streptococcus*	*Streptococcus*
4589	*Firmicutes*	*Bacilli*	*Lactobacillales*	*Streptococcaceae*	*Streptococcus*	*Streptococcus*
4590	*Firmicutes*	*Bacilli*	*Lactobacillales*	*Streptococcaceae*	*Streptococcus*	*Streptococcus*
4591	*Firmicutes*	*Bacilli*	*Lactobacillales*	*Streptococcaceae*	*Streptococcus*	*Streptococcus*
4592	*Firmicutes*	*Bacilli*	*Lactobacillales*	*Streptococcaceae*	*Streptococcus*	*Streptococcus*

Table S4. Isolated strains by culturomics

bacteriaId	*phylum*	*class*	*order*	*family*	*genus*	*species*
4593	*Firmicutes*	*Bacilli*	*Lactobacillales*	*Streptococcaceae*	*Streptococcus*	*Streptococcus*
4594	*Firmicutes*	*Bacilli*	*Lactobacillales*	*Streptococcaceae*	*Streptococcus*	*Streptococcus*
4595	*Firmicutes*	*Bacilli*	*Lactobacillales*	*Streptococcaceae*	*Streptococcus*	*Streptococcus*
4596	*Firmicutes*	*Bacilli*	*Lactobacillales*	*Streptococcaceae*	*Streptococcus*	*Streptococcus*
4597	*Firmicutes*	*Bacilli*	*Lactobacillales*	*Streptococcaceae*	*Streptococcus*	*Streptococcus*
4598	*Firmicutes*	*Bacilli*	*Lactobacillales*	*Streptococcaceae*	*Streptococcus*	*Streptococcus*
4599	*Firmicutes*	*Bacilli*	*Lactobacillales*	*Streptococcaceae*	*Streptococcus*	*Streptococcus*
4600	*Firmicutes*	*Bacilli*	*Lactobacillales*	*Streptococcaceae*	*Streptococcus*	*Streptococcus*
4601	*Firmicutes*	*Bacilli*	*Lactobacillales*	*Streptococcaceae*	*Streptococcus*	*Streptococcus*
4602	*Firmicutes*	*Bacilli*	*Lactobacillales*	*Streptococcaceae*	*Streptococcus*	*Streptococcus*
4603	*Firmicutes*	*Bacilli*	*Lactobacillales*	*Streptococcaceae*	*Streptococcus*	*Streptococcus*
4604	*Firmicutes*	*Bacilli*	*Lactobacillales*	*Streptococcaceae*	*Streptococcus*	*Streptococcus*
4605	*Firmicutes*	*Bacilli*	*Lactobacillales*	*Streptococcaceae*	*Streptococcus*	*Streptococcus*
4606	*Firmicutes*	*Bacilli*	*Lactobacillales*	*Streptococcaceae*	*Streptococcus*	*Streptococcus*
4607	*Firmicutes*	*Bacilli*	*Lactobacillales*	*Streptococcaceae*	*Streptococcus*	*Streptococcus*
4608	*Firmicutes*	*Bacilli*	*Lactobacillales*	*Streptococcaceae*	*Streptococcus*	*Streptococcus*
4609	*Firmicutes*	*Bacilli*	*Lactobacillales*	*Streptococcaceae*	*Streptococcus*	*Streptococcus*
4610	*Firmicutes*	*Bacilli*	*Lactobacillales*	*Streptococcaceae*	*Streptococcus*	*Streptococcus*
4611	*Firmicutes*	*Bacilli*	*Lactobacillales*	*Streptococcaceae*	*Streptococcus*	*Streptococcus*
4612	*Firmicutes*	*Bacilli*	*Lactobacillales*	*Streptococcaceae*	*Streptococcus*	*Streptococcus*
4613	*Firmicutes*	*Bacilli*	*Lactobacillales*	*Streptococcaceae*	*Streptococcus*	*Streptococcus*
4614	*Firmicutes*	*Bacilli*	*Lactobacillales*	*Streptococcaceae*	*Streptococcus*	*Streptococcus*
4615	*Firmicutes*	*Bacilli*	*Lactobacillales*	*Streptococcaceae*	*Streptococcus*	*Streptococcus*
4616	*Firmicutes*	*Bacilli*	*Lactobacillales*	*Streptococcaceae*	*Streptococcus*	*Streptococcus*
4617	*Firmicutes*	*Bacilli*	*Lactobacillales*	*Streptococcaceae*	*Streptococcus*	*Streptococcus*
4618	*Firmicutes*	*Bacilli*	*Lactobacillales*	*Streptococcaceae*	*Streptococcus*	*Streptococcus*
4619	*Firmicutes*	*Bacilli*	*Lactobacillales*	*Streptococcaceae*	*Streptococcus*	*Streptococcus*
4620	*Firmicutes*	*Bacilli*	*Lactobacillales*	*Streptococcaceae*	*Streptococcus*	*Streptococcus*

Table S4. Isolated strains by culturomics

bacteriaId	*phylum*	*class*	*order*	*family*	*genus*	*species*
4621	*Firmicutes*	*Bacilli*	*Lactobacillales*	*Streptococcaceae*	*Streptococcus*	*Streptococcus*
4622	*Firmicutes*	*Bacilli*	*Lactobacillales*	*Streptococcaceae*	*Streptococcus*	*Streptococcus*
4623	*Firmicutes*	*Bacilli*	*Lactobacillales*	*Streptococcaceae*	*Streptococcus*	*Streptococcus*
4624	*Firmicutes*	*Bacilli*	*Lactobacillales*	*Streptococcaceae*	*Streptococcus*	*Streptococcus*
4625	*Firmicutes*	*Bacilli*	*Lactobacillales*	*Streptococcaceae*	*Streptococcus*	*Streptococcus*
4626	*Firmicutes*	*Bacilli*	*Lactobacillales*	*Streptococcaceae*	*Streptococcus*	*Streptococcus*
4627	*Firmicutes*	*Bacilli*	*Lactobacillales*	*Streptococcaceae*	*Streptococcus*	*Streptococcus*
4628	*Firmicutes*	*Bacilli*	*Lactobacillales*	*Streptococcaceae*	*Streptococcus*	*Streptococcus*
4629	*Firmicutes*	*Bacilli*	*Lactobacillales*	*Streptococcaceae*	*Streptococcus*	*Streptococcus*
4630	*Firmicutes*	*Bacilli*	*Lactobacillales*	*Streptococcaceae*	*Streptococcus*	*Streptococcus*
4631	*Firmicutes*	*Bacilli*	*Lactobacillales*	*Streptococcaceae*	*Streptococcus*	*Streptococcus*
4632	*Firmicutes*	*Bacilli*	*Lactobacillales*	*Streptococcaceae*	*Streptococcus*	*Streptococcus*
4633	*Firmicutes*	*Bacilli*	*Lactobacillales*	*Streptococcaceae*	*Streptococcus*	*Streptococcus*
4634	*Firmicutes*	*Bacilli*	*Lactobacillales*	*Streptococcaceae*	*Streptococcus*	*Streptococcus*
4635	*Firmicutes*	*Bacilli*	*Lactobacillales*	*Streptococcaceae*	*Streptococcus*	*Streptococcus*
4636	*Firmicutes*	*Bacilli*	*Lactobacillales*	*Streptococcaceae*	*Streptococcus*	*Streptococcus*
4637	*Firmicutes*	*Bacilli*	*Lactobacillales*	*Streptococcaceae*	*Streptococcus*	*Streptococcus*
4638	*Firmicutes*	*Bacilli*	*Lactobacillales*	*Streptococcaceae*	*Streptococcus*	*Streptococcus*
4639	*Firmicutes*	*Bacilli*	*Lactobacillales*	*Streptococcaceae*	*Streptococcus*	*Streptococcus*
4640	*Firmicutes*	*Bacilli*	*Lactobacillales*	*Streptococcaceae*	*Streptococcus*	*Streptococcus*
4641	*Firmicutes*	*Bacilli*	*Lactobacillales*	*Streptococcaceae*	*Streptococcus*	*Streptococcus*
4642	*Firmicutes*	*Bacilli*	*Lactobacillales*	*Streptococcaceae*	*Streptococcus*	*Streptococcus*
4643	*Firmicutes*	*Bacilli*	*Lactobacillales*	*Streptococcaceae*	*Streptococcus*	*Streptococcus*
4644	*Firmicutes*	*Bacilli*	*Lactobacillales*	*Streptococcaceae*	*Streptococcus*	*Streptococcus*
4645	*Firmicutes*	*Bacilli*	*Lactobacillales*	*Streptococcaceae*	*Streptococcus*	*Streptococcus*
4646	*Firmicutes*	*Bacilli*	*Lactobacillales*	*Streptococcaceae*	*Streptococcus*	*Streptococcus*
4647	*Firmicutes*	*Bacilli*	*Lactobacillales*	*Streptococcaceae*	*Streptococcus*	*Streptococcus*
4648	*Firmicutes*	*Bacilli*	*Lactobacillales*	*Streptococcaceae*	*Streptococcus*	*Streptococcus*

Table S4. Isolated strains by culturomics

bacteriaId	*phylum*	*class*	*order*	*family*	*genus*	*species*
4649	*Firmicutes*	*Bacilli*	*Lactobacillales*	*Streptococcaceae*	*Streptococcus*	*Streptococcus*
4650	*Firmicutes*	*Bacilli*	*Lactobacillales*	*Streptococcaceae*	*Streptococcus*	*Streptococcus*
4651	*Firmicutes*	*Bacilli*	*Lactobacillales*	*Streptococcaceae*	*Streptococcus*	*Streptococcus*
4652	*Firmicutes*	*Bacilli*	*Lactobacillales*	*Streptococcaceae*	*Streptococcus*	*Streptococcus*
4653	*Firmicutes*	*Bacilli*	*Lactobacillales*	*Streptococcaceae*	*Streptococcus*	*Streptococcus*
4654	*Firmicutes*	*Bacilli*	*Lactobacillales*	*Streptococcaceae*	*Streptococcus*	*Streptococcus*
4655	*Firmicutes*	*Bacilli*	*Lactobacillales*	*Streptococcaceae*	*Streptococcus*	*Streptococcus*
4656	*Firmicutes*	*Bacilli*	*Lactobacillales*	*Streptococcaceae*	*Streptococcus*	*Streptococcus*
4657	*Firmicutes*	*Bacilli*	*Lactobacillales*	*Streptococcaceae*	*Streptococcus*	*Streptococcus*
4658	*Firmicutes*	*Bacilli*	*Lactobacillales*	*Streptococcaceae*	*Streptococcus*	*Streptococcus*
4659	*Firmicutes*	*Bacilli*	*Lactobacillales*	*Streptococcaceae*	*Streptococcus*	*Streptococcus*
4660	*Firmicutes*	*Bacilli*	*Lactobacillales*	*Streptococcaceae*	*Streptococcus*	*Streptococcus*
4661	*Firmicutes*	*Bacilli*	*Lactobacillales*	*Streptococcaceae*	*Streptococcus*	*Streptococcus*
4662	*Firmicutes*	*Bacilli*	*Lactobacillales*	*Streptococcaceae*	*Streptococcus*	*Streptococcus*
4663	*Firmicutes*	*Bacilli*	*Lactobacillales*	*Streptococcaceae*	*Streptococcus*	*Streptococcus*
4664	*Firmicutes*	*Bacilli*	*Lactobacillales*	*Streptococcaceae*	*Streptococcus*	*Streptococcus*
4665	*Firmicutes*	*Bacilli*	*Lactobacillales*	*Streptococcaceae*	*Streptococcus*	*Streptococcus*
4666	*Firmicutes*	*Bacilli*	*Lactobacillales*	*Streptococcaceae*	*Streptococcus*	*Streptococcus*
4667	*Firmicutes*	*Bacilli*	*Lactobacillales*	*Streptococcaceae*	*Streptococcus*	*Streptococcus*
4668	*Firmicutes*	*Bacilli*	*Lactobacillales*	*Streptococcaceae*	*Streptococcus*	*Streptococcus*
4669	*Firmicutes*	*Bacilli*	*Lactobacillales*	*Streptococcaceae*	*Streptococcus*	*Streptococcus*
4670	*Firmicutes*	*Bacilli*	*Lactobacillales*	*Streptococcaceae*	*Streptococcus*	*Streptococcus*
4671	*Firmicutes*	*Bacilli*	*Lactobacillales*	*Streptococcaceae*	*Streptococcus*	*Streptococcus*
4672	*Firmicutes*	*Bacilli*	*Lactobacillales*	*Streptococcaceae*	*Streptococcus*	*Streptococcus*
4673	*Firmicutes*	*Bacilli*	*Lactobacillales*	*Streptococcaceae*	*Streptococcus*	*Streptococcus*
4674	*Firmicutes*	*Bacilli*	*Lactobacillales*	*Streptococcaceae*	*Streptococcus*	*Streptococcus*
4675	*Firmicutes*	*Bacilli*	*Lactobacillales*	*Streptococcaceae*	*Streptococcus*	*Streptococcus*
4676	*Firmicutes*	*Bacilli*	*Lactobacillales*	*Streptococcaceae*	*Streptococcus*	*Streptococcus*

Table S4. Isolated strains by culturomics

bacteriaId	*phylum*	*class*	*order*	*family*	*genus*	*species*
4677	*Firmicutes*	*Bacilli*	*Lactobacillales*	*Streptococcaceae*	*Streptococcus*	*Streptococcus*
4678	*Firmicutes*	*Bacilli*	*Lactobacillales*	*Streptococcaceae*	*Streptococcus*	*Streptococcus*
4679	*Firmicutes*	*Bacilli*	*Lactobacillales*	*Streptococcaceae*	*Streptococcus*	*Streptococcus*
4680	*Firmicutes*	*Bacilli*	*Lactobacillales*	*Streptococcaceae*	*Streptococcus*	*Streptococcus*
4681	*Firmicutes*	*Bacilli*	*Lactobacillales*	*Streptococcaceae*	*Streptococcus*	*Streptococcus*
4682	*Firmicutes*	*Bacilli*	*Lactobacillales*	*Streptococcaceae*	*Streptococcus*	*Streptococcus*
4683	*Firmicutes*	*Bacilli*	*Lactobacillales*	*Streptococcaceae*	*Streptococcus*	*Streptococcus*
4684	*Firmicutes*	*Bacilli*	*Lactobacillales*	*Streptococcaceae*	*Streptococcus*	*Streptococcus*
4685	*Firmicutes*	*Bacilli*	*Lactobacillales*	*Streptococcaceae*	*Streptococcus*	*Streptococcus*
4686	*Firmicutes*	*Bacilli*	*Lactobacillales*	*Streptococcaceae*	*Streptococcus*	*Streptococcus*
4687	*Firmicutes*	*Bacilli*	*Lactobacillales*	*Streptococcaceae*	*Streptococcus*	*Streptococcus*
4688	*Firmicutes*	*Bacilli*	*Lactobacillales*	*Streptococcaceae*	*Streptococcus*	*Streptococcus*
4689	*Firmicutes*	*Bacilli*	*Lactobacillales*	*Streptococcaceae*	*Streptococcus*	*Streptococcus*
4690	*Firmicutes*	*Bacilli*	*Lactobacillales*	*Streptococcaceae*	*Streptococcus*	*Streptococcus*
4691	*Firmicutes*	*Bacilli*	*Lactobacillales*	*Streptococcaceae*	*Streptococcus*	*Streptococcus*
4692	*Firmicutes*	*Bacilli*	*Lactobacillales*	*Streptococcaceae*	*Streptococcus*	*Streptococcus*
4693	*Firmicutes*	*Bacilli*	*Lactobacillales*	*Streptococcaceae*	*Streptococcus*	*Streptococcus*
4694	*Firmicutes*	*Bacilli*	*Lactobacillales*	*Streptococcaceae*	*Streptococcus*	*Streptococcus*
4695	*Firmicutes*	*Bacilli*	*Lactobacillales*	*Streptococcaceae*	*Streptococcus*	*Streptococcus*
4696	*Firmicutes*	*Bacilli*	*Lactobacillales*	*Streptococcaceae*	*Streptococcus*	*Streptococcus*
4697	*Firmicutes*	*Bacilli*	*Lactobacillales*	*Streptococcaceae*	*Streptococcus*	*Streptococcus*
4698	*Firmicutes*	*Bacilli*	*Lactobacillales*	*Streptococcaceae*	*Streptococcus*	*Streptococcus*
4699	*Firmicutes*	*Bacilli*	*Lactobacillales*	*Streptococcaceae*	*Streptococcus*	*Streptococcus*
4700	*Firmicutes*	*Bacilli*	*Lactobacillales*	*Streptococcaceae*	*Streptococcus*	*Streptococcus*
4701	*Firmicutes*	*Bacilli*	*Lactobacillales*	*Streptococcaceae*	*Streptococcus*	*Streptococcus*
4702	*Firmicutes*	*Bacilli*	*Lactobacillales*	*Streptococcaceae*	*Streptococcus*	*Streptococcus*
4703	*Firmicutes*	*Bacilli*	*Lactobacillales*	*Streptococcaceae*	*Streptococcus*	*Streptococcus*
4704	*Firmicutes*	*Bacilli*	*Lactobacillales*	*Streptococcaceae*	*Streptococcus*	*Streptococcus*

Table S4. Isolated strains by culturomics

bacteriaId	*phylum*	*class*	*order*	*family*	*genus*	*species*
4705	*Firmicutes*	*Bacilli*	*Lactobacillales*	*Streptococcaceae*	*Streptococcus*	*Streptococcus*
4706	*Firmicutes*	*Bacilli*	*Lactobacillales*	*Streptococcaceae*	*Streptococcus*	*Streptococcus*
4707	*Firmicutes*	*Bacilli*	*Lactobacillales*	*Streptococcaceae*	*Streptococcus*	*Streptococcus*
4708	*Firmicutes*	*Bacilli*	*Lactobacillales*	*Streptococcaceae*	*Streptococcus*	*Streptococcus*
4709	*Firmicutes*	*Bacilli*	*Lactobacillales*	*Streptococcaceae*	*Streptococcus*	*Streptococcus*
4710	*Firmicutes*	*Bacilli*	*Lactobacillales*	*Streptococcaceae*	*Streptococcus*	*Streptococcus*
4711	*Firmicutes*	*Bacilli*	*Lactobacillales*	*Streptococcaceae*	*Streptococcus*	*Streptococcus*
4712	*Firmicutes*	*Bacilli*	*Lactobacillales*	*Streptococcaceae*	*Streptococcus*	*Streptococcus*
4713	*Firmicutes*	*Bacilli*	*Lactobacillales*	*Streptococcaceae*	*Streptococcus*	*Streptococcus*
4714	*Firmicutes*	*Bacilli*	*Lactobacillales*	*Streptococcaceae*	*Streptococcus*	*Streptococcus*
4715	*Firmicutes*	*Bacilli*	*Lactobacillales*	*Streptococcaceae*	*Streptococcus*	*Streptococcus*
4716	*Firmicutes*	*Bacilli*	*Lactobacillales*	*Streptococcaceae*	*Streptococcus*	*Streptococcus*
4717	*Firmicutes*	*Bacilli*	*Lactobacillales*	*Streptococcaceae*	*Streptococcus*	*Streptococcus*
4718	*Firmicutes*	*Bacilli*	*Lactobacillales*	*Streptococcaceae*	*Streptococcus*	*Streptococcus*
4719	*Firmicutes*	*Bacilli*	*Lactobacillales*	*Streptococcaceae*	*Streptococcus*	*Streptococcus*
4720	*Firmicutes*	*Bacilli*	*Lactobacillales*	*Streptococcaceae*	*Streptococcus*	*Streptococcus*
4721	*Firmicutes*	*Bacilli*	*Lactobacillales*	*Streptococcaceae*	*Streptococcus*	*Streptococcus*
4722	*Firmicutes*	*Bacilli*	*Lactobacillales*	*Streptococcaceae*	*Streptococcus*	*Streptococcus*
4723	*Firmicutes*	*Bacilli*	*Lactobacillales*	*Streptococcaceae*	*Streptococcus*	*Streptococcus*
4724	*Firmicutes*	*Bacilli*	*Lactobacillales*	*Streptococcaceae*	*Streptococcus*	*Streptococcus*
4725	*Firmicutes*	*Bacilli*	*Lactobacillales*	*Streptococcaceae*	*Streptococcus*	*Streptococcus*
4726	*Firmicutes*	*Bacilli*	*Lactobacillales*	*Streptococcaceae*	*Streptococcus*	*Streptococcus*
4727	*Firmicutes*	*Bacilli*	*Lactobacillales*	*Streptococcaceae*	*Streptococcus*	*Streptococcus*
4728	*Firmicutes*	*Bacilli*	*Lactobacillales*	*Streptococcaceae*	*Streptococcus*	*Streptococcus*
4729	*Firmicutes*	*Bacilli*	*Lactobacillales*	*Streptococcaceae*	*Streptococcus*	*Streptococcus*
4730	*Firmicutes*	*Bacilli*	*Lactobacillales*	*Streptococcaceae*	*Streptococcus*	*Streptococcus*
4731	*Firmicutes*	*Bacilli*	*Lactobacillales*	*Streptococcaceae*	*Streptococcus*	*Streptococcus*
4732	*Firmicutes*	*Bacilli*	*Lactobacillales*	*Streptococcaceae*	*Streptococcus*	*Streptococcus*

Table S4. Isolated strains by culturomics

bacteriaId	*phylum*	*class*	*order*	*family*	*genus*	*species*
4733	*Firmicutes*	*Bacilli*	*Lactobacillales*	*Streptococcaceae*	*Streptococcus*	*Streptococcus*
4734	*Firmicutes*	*Bacilli*	*Lactobacillales*	*Streptococcaceae*	*Streptococcus*	*Streptococcus*
4735	*Firmicutes*	*Bacilli*	*Lactobacillales*	*Streptococcaceae*	*Streptococcus*	*Streptococcus*
4736	*Firmicutes*	*Bacilli*	*Lactobacillales*	*Streptococcaceae*	*Streptococcus*	*Streptococcus*
4737	*Firmicutes*	*Bacilli*	*Lactobacillales*	*Streptococcaceae*	*Streptococcus*	*Streptococcus*
4738	*Firmicutes*	*Bacilli*	*Lactobacillales*	*Streptococcaceae*	*Streptococcus*	*Streptococcus*
4739	*Firmicutes*	*Bacilli*	*Lactobacillales*	*Streptococcaceae*	*Streptococcus*	*Streptococcus*
4740	*Firmicutes*	*Bacilli*	*Lactobacillales*	*Streptococcaceae*	*Streptococcus*	*Streptococcus*
4741	*Firmicutes*	*Bacilli*	*Lactobacillales*	*Streptococcaceae*	*Streptococcus*	*Streptococcus*
4742	*Firmicutes*	*Bacilli*	*Lactobacillales*	*Streptococcaceae*	*Streptococcus*	*Streptococcus*
4743	*Firmicutes*	*Bacilli*	*Lactobacillales*	*Streptococcaceae*	*Streptococcus*	*Streptococcus*
4744	*Firmicutes*	*Bacilli*	*Lactobacillales*	*Streptococcaceae*	*Streptococcus*	*Streptococcus*
4745	*Firmicutes*	*Bacilli*	*Lactobacillales*	*Streptococcaceae*	*Streptococcus*	*Streptococcus*
4746	*Firmicutes*	*Bacilli*	*Lactobacillales*	*Streptococcaceae*	*Streptococcus*	*Streptococcus*
4747	*Firmicutes*	*Bacilli*	*Lactobacillales*	*Streptococcaceae*	*Streptococcus*	*Streptococcus*
4748	*Firmicutes*	*Bacilli*	*Lactobacillales*	*Streptococcaceae*	*Streptococcus*	*Streptococcus*
4749	*Firmicutes*	*Bacilli*	*Lactobacillales*	*Streptococcaceae*	*Streptococcus*	*Streptococcus*
4750	*Firmicutes*	*Bacilli*	*Lactobacillales*	*Streptococcaceae*	*Streptococcus*	*Streptococcus*
4751	*Firmicutes*	*Bacilli*	*Lactobacillales*	*Streptococcaceae*	*Streptococcus*	*Streptococcus*
4752	*Firmicutes*	*Bacilli*	*Lactobacillales*	*Streptococcaceae*	*Streptococcus*	*Streptococcus*
4753	*Firmicutes*	*Bacilli*	*Lactobacillales*	*Streptococcaceae*	*Streptococcus*	*Streptococcus*
4754	*Firmicutes*	*Bacilli*	*Lactobacillales*	*Streptococcaceae*	*Streptococcus*	*Streptococcus*
4755	*Firmicutes*	*Bacilli*	*Lactobacillales*	*Streptococcaceae*	*Streptococcus*	*Streptococcus*
4756	*Firmicutes*	*Bacilli*	*Lactobacillales*	*Streptococcaceae*	*Streptococcus*	*Streptococcus*
4757	*Firmicutes*	*Bacilli*	*Lactobacillales*	*Streptococcaceae*	*Streptococcus*	*Streptococcus*
4758	*Firmicutes*	*Bacilli*	*Lactobacillales*	*Streptococcaceae*	*Streptococcus*	*Streptococcus*
4759	*Firmicutes*	*Bacilli*	*Lactobacillales*	*Streptococcaceae*	*Streptococcus*	*Streptococcus*
4760	*Firmicutes*	*Bacilli*	*Lactobacillales*	*Streptococcaceae*	*Streptococcus*	*Streptococcus*

Table S4. Isolated strains by culturomics

bacteriaId	*phylum*	*class*	*order*	*family*	*genus*	*species*
4761	*Firmicutes*	*Bacilli*	*Lactobacillales*	*Streptococcaceae*	*Streptococcus*	*Streptococcus*
4762	*Firmicutes*	*Bacilli*	*Lactobacillales*	*Streptococcaceae*	*Streptococcus*	*Streptococcus*
4763	*Firmicutes*	*Bacilli*	*Lactobacillales*	*Streptococcaceae*	*Streptococcus*	*Streptococcus*
4764	*Firmicutes*	*Bacilli*	*Lactobacillales*	*Streptococcaceae*	*Streptococcus*	*Streptococcus*
4765	*Firmicutes*	*Bacilli*	*Lactobacillales*	*Streptococcaceae*	*Streptococcus*	*Streptococcus*
4766	*Firmicutes*	*Bacilli*	*Lactobacillales*	*Streptococcaceae*	*Streptococcus*	*Streptococcus*
4767	*Firmicutes*	*Bacilli*	*Lactobacillales*	*Streptococcaceae*	*Streptococcus*	*Streptococcus*
4768	*Firmicutes*	*Bacilli*	*Lactobacillales*	*Streptococcaceae*	*Streptococcus*	*Streptococcus*
4769	*Firmicutes*	*Bacilli*	*Lactobacillales*	*Streptococcaceae*	*Streptococcus*	*Streptococcus*
4770	*Firmicutes*	*Bacilli*	*Lactobacillales*	*Streptococcaceae*	*Streptococcus*	*Streptococcus*
4771	*Firmicutes*	*Bacilli*	*Lactobacillales*	*Streptococcaceae*	*Streptococcus*	*Streptococcus*
4772	*Firmicutes*	*Bacilli*	*Lactobacillales*	*Streptococcaceae*	*Streptococcus*	*Streptococcus*
4773	*Firmicutes*	*Bacilli*	*Lactobacillales*	*Streptococcaceae*	*Streptococcus*	*Streptococcus*
4774	*Firmicutes*	*Bacilli*	*Lactobacillales*	*Streptococcaceae*	*Streptococcus*	*Streptococcus*
4775	*Firmicutes*	*Bacilli*	*Lactobacillales*	*Streptococcaceae*	*Streptococcus*	*Streptococcus*
4776	*Firmicutes*	*Bacilli*	*Lactobacillales*	*Streptococcaceae*	*Streptococcus*	*Streptococcus*
4777	*Firmicutes*	*Bacilli*	*Lactobacillales*	*Streptococcaceae*	*Streptococcus*	*Streptococcus*
4778	*Firmicutes*	*Bacilli*	*Lactobacillales*	*Streptococcaceae*	*Streptococcus*	*Streptococcus*
4779	*Firmicutes*	*Bacilli*	*Lactobacillales*	*Streptococcaceae*	*Streptococcus*	*Streptococcus*
4780	*Firmicutes*	*Bacilli*	*Lactobacillales*	*Streptococcaceae*	*Streptococcus*	*Streptococcus*
4781	*Firmicutes*	*Bacilli*	*Lactobacillales*	*Streptococcaceae*	*Streptococcus*	*Streptococcus*
4782	*Firmicutes*	*Bacilli*	*Lactobacillales*	*Streptococcaceae*	*Streptococcus*	*Streptococcus*
4783	*Firmicutes*	*Bacilli*	*Lactobacillales*	*Streptococcaceae*	*Streptococcus*	*Streptococcus*
4784	*Firmicutes*	*Bacilli*	*Lactobacillales*	*Streptococcaceae*	*Streptococcus*	*Streptococcus*
4785	*Firmicutes*	*Bacilli*	*Lactobacillales*	*Streptococcaceae*	*Streptococcus*	*Streptococcus*
4786	*Firmicutes*	*Bacilli*	*Lactobacillales*	*Streptococcaceae*	*Streptococcus*	*Streptococcus*
4787	*Firmicutes*	*Bacilli*	*Lactobacillales*	*Streptococcaceae*	*Streptococcus*	*Streptococcus*
4788	*Firmicutes*	*Bacilli*	*Lactobacillales*	*Streptococcaceae*	*Streptococcus*	*Streptococcus*

Table S4. Isolated strains by culturomics

bacteriaId	*phylum*	*class*	*order*	*family*	*genus*	*species*
4789	*Firmicutes*	*Bacilli*	*Lactobacillales*	*Streptococcaceae*	*Streptococcus*	*Streptococcus*
4790	*Firmicutes*	*Bacilli*	*Lactobacillales*	*Streptococcaceae*	*Streptococcus*	*Streptococcus*
4791	*Firmicutes*	*Bacilli*	*Lactobacillales*	*Streptococcaceae*	*Streptococcus*	*Streptococcus*
4792	*Firmicutes*	*Bacilli*	*Lactobacillales*	*Streptococcaceae*	*Streptococcus*	*Streptococcus*
4793	*Firmicutes*	*Bacilli*	*Lactobacillales*	*Streptococcaceae*	*Streptococcus*	*Streptococcus*
4794	*Firmicutes*	*Bacilli*	*Lactobacillales*	*Streptococcaceae*	*Streptococcus*	*Streptococcus*
4795	*Firmicutes*	*Bacilli*	*Lactobacillales*	*Streptococcaceae*	*Streptococcus*	*Streptococcus*
4796	*Firmicutes*	*Bacilli*	*Lactobacillales*	*Streptococcaceae*	*Streptococcus*	*Streptococcus*
4797	*Firmicutes*	*Bacilli*	*Lactobacillales*	*Streptococcaceae*	*Streptococcus*	*Streptococcus*
4798	*Firmicutes*	*Bacilli*	*Lactobacillales*	*Streptococcaceae*	*Streptococcus*	*Streptococcus*
4799	*Firmicutes*	*Bacilli*	*Lactobacillales*	*Streptococcaceae*	*Streptococcus*	*Streptococcus*
4800	*Firmicutes*	*Bacilli*	*Lactobacillales*	*Streptococcaceae*	*Streptococcus*	*Streptococcus*
4801	*Firmicutes*	*Bacilli*	*Lactobacillales*	*Streptococcaceae*	*Streptococcus*	*Streptococcus*
4802	*Firmicutes*	*Bacilli*	*Lactobacillales*	*Streptococcaceae*	*Streptococcus*	*Streptococcus*
4803	*Firmicutes*	*Bacilli*	*Lactobacillales*	*Streptococcaceae*	*Streptococcus*	*Streptococcus*
4804	*Firmicutes*	*Bacilli*	*Lactobacillales*	*Streptococcaceae*	*Streptococcus*	*Streptococcus*
4805	*Firmicutes*	*Bacilli*	*Lactobacillales*	*Streptococcaceae*	*Streptococcus*	*Streptococcus*
4806	*Firmicutes*	*Bacilli*	*Lactobacillales*	*Streptococcaceae*	*Streptococcus*	*Streptococcus*
4807	*Firmicutes*	*Bacilli*	*Lactobacillales*	*Streptococcaceae*	*Streptococcus*	*Streptococcus*
4808	*Firmicutes*	*Bacilli*	*Lactobacillales*	*Streptococcaceae*	*Streptococcus*	*Streptococcus*
4809	*Firmicutes*	*Bacilli*	*Lactobacillales*	*Streptococcaceae*	*Streptococcus*	*Streptococcus*
4810	*Firmicutes*	*Bacilli*	*Lactobacillales*	*Streptococcaceae*	*Streptococcus*	*Streptococcus*
4811	*Firmicutes*	*Bacilli*	*Lactobacillales*	*Streptococcaceae*	*Streptococcus*	*Streptococcus*
4812	*Firmicutes*	*Bacilli*	*Lactobacillales*	*Streptococcaceae*	*Streptococcus*	*Streptococcus*
4813	*Firmicutes*	*Bacilli*	*Lactobacillales*	*Streptococcaceae*	*Streptococcus*	*Streptococcus*
4814	*Firmicutes*	*Bacilli*	*Lactobacillales*	*Streptococcaceae*	*Streptococcus*	*Streptococcus*
4815	*Firmicutes*	*Bacilli*	*Lactobacillales*	*Streptococcaceae*	*Streptococcus*	*Streptococcus*
4816	*Firmicutes*	*Bacilli*	*Lactobacillales*	*Streptococcaceae*	*Streptococcus*	*Streptococcus*

Table S4. Isolated strains by culturomics

bacteriaId	*phylum*	*class*	*order*	*family*	*genus*	*species*
4817	*Firmicutes*	*Bacilli*	*Lactobacillales*	*Streptococcaceae*	*Streptococcus*	*Streptococcus*
4818	*Firmicutes*	*Bacilli*	*Lactobacillales*	*Streptococcaceae*	*Streptococcus*	*Streptococcus*
4819	*Firmicutes*	*Bacilli*	*Lactobacillales*	*Streptococcaceae*	*Streptococcus*	*Streptococcus*
4820	*Firmicutes*	*Bacilli*	*Lactobacillales*	*Streptococcaceae*	*Streptococcus*	*Streptococcus oralis*
4821	*Firmicutes*	*Bacilli*	*Lactobacillales*	*Streptococcaceae*	*Streptococcus*	*Streptococcus oralis*
4822	*Firmicutes*	*Bacilli*	*Lactobacillales*	*Streptococcaceae*	*Streptococcus*	*Streptococcus oralis*
4823	*Firmicutes*	*Bacilli*	*Lactobacillales*	*Streptococcaceae*	*Streptococcus*	*Streptococcus oralis*
4824	*Firmicutes*	*Bacilli*	*Lactobacillales*	*Streptococcaceae*	*Streptococcus*	*Streptococcus oralis*
4825	*Firmicutes*	*Bacilli*	*Lactobacillales*	*Streptococcaceae*	*Streptococcus*	*Streptococcus oralis*
4826	*Firmicutes*	*Bacilli*	*Lactobacillales*	*Streptococcaceae*	*Streptococcus*	*Streptococcus oralis*
4827	*Firmicutes*	*Bacilli*	*Lactobacillales*	*Streptococcaceae*	*Streptococcus*	*Streptococcus oralis*
4828	*Firmicutes*	*Bacilli*	*Lactobacillales*	*Streptococcaceae*	*Streptococcus*	*Streptococcus oralis*
4829	*Firmicutes*	*Bacilli*	*Lactobacillales*	*Streptococcaceae*	*Streptococcus*	*Streptococcus*
4830	*Firmicutes*	*Bacilli*	*Lactobacillales*	*Streptococcaceae*	*Streptococcus*	*Streptococcus*
4831	*Firmicutes*	*Bacilli*	*Lactobacillales*	*Streptococcaceae*	*Streptococcus*	*Streptococcus*
4832	*Firmicutes*	*Bacilli*	*Lactobacillales*	*Streptococcaceae*	*Streptococcus*	*Streptococcus*
4833	*Firmicutes*	*Bacilli*	*Lactobacillales*	*Streptococcaceae*	*Streptococcus*	*Streptococcus*
4834	*Firmicutes*	*Bacilli*	*Lactobacillales*	*Streptococcaceae*	*Streptococcus*	*Streptococcus*
4835	*Firmicutes*	*Bacilli*	*Lactobacillales*	*Streptococcaceae*	*Streptococcus*	*Streptococcus*
4836	*Firmicutes*	*Bacilli*	*Lactobacillales*	*Streptococcaceae*	*Streptococcus*	*Streptococcus*
4837	*Firmicutes*	*Bacilli*	*Lactobacillales*	*Streptococcaceae*	*Streptococcus*	*Streptococcus*
4838	*Firmicutes*	*Bacilli*	*Lactobacillales*	*Streptococcaceae*	*Streptococcus*	*Streptococcus*
4839	*Firmicutes*	*Bacilli*	*Lactobacillales*	*Streptococcaceae*	*Streptococcus*	*Streptococcus*
4840	*Firmicutes*	*Bacilli*	*Lactobacillales*	*Streptococcaceae*	*Streptococcus*	*Streptococcus*
4841	*Firmicutes*	*Clostridia*	*Clostridia*	*Clostridiaceae*	*Clostridium*	*Clostridium*
4842	*Firmicutes*	*Clostridia*	*Clostridia*	*Clostridiaceae*	*Clostridium*	*Clostridium*
4843	*Firmicutes*	*Clostridia*	*Clostridia*	*Clostridiaceae*	*Clostridium*	*Clostridium*
4844	*Firmicutes*	*Clostridia*	*Clostridia*	*Clostridiaceae*	*Clostridium*	*Clostridium*

Table S4. Isolated strains by culturomics

bacteriaId	*phylum*	*class*	*order*	*family*	*genus*	*species*
4845	*Firmicutes*	*Clostridia*	*Clostridia*	*Clostridiaceae*	*Clostridium*	*Clostridium*
4846	*Firmicutes*	*Clostridia*	*Clostridia*	*Clostridiaceae*	*Clostridium*	*Clostridium*
4847	*Firmicutes*	*Clostridia*	*Clostridia*	*Clostridiaceae*	*Clostridium*	*Clostridium*
4848	*Firmicutes*	*Clostridia*	*Clostridia*	*Clostridiaceae*	*Clostridium*	*Clostridium*
4849	*Firmicutes*	*Clostridia*	*Clostridia*	*Clostridiaceae*	*Clostridium*	*Clostridium*
4850	*Firmicutes*	*Clostridia*	*Clostridia*	*Clostridiaceae*	*Clostridium*	*Clostridium*
4851	*Firmicutes*	*Clostridia*	*Clostridia*	*Clostridiaceae*	*Clostridium*	*Clostridium*
4852	*Firmicutes*	*Clostridia*	*Clostridia*	*Clostridiaceae*	*Clostridium*	*Clostridium*
4853	*Firmicutes*	*Clostridia*	*Clostridia*	*Clostridiaceae*	*Clostridium*	*Clostridium*
4854	*Firmicutes*	*Clostridia*	*Clostridia*	*Clostridiaceae*	*Clostridium*	*Clostridium*
4855	*Firmicutes*	*Clostridia*	*Clostridia*	*Clostridiaceae*	*Clostridium*	*Clostridium*
4856	*Firmicutes*	*Clostridia*	*Clostridia*	*Clostridiaceae*	*Clostridium*	*Clostridium*
4857	*Firmicutes*	*Clostridia*	*Clostridia*	*Clostridiaceae*	*Clostridium*	*Clostridium*
4858	*Firmicutes*	*Clostridia*	*Clostridia*	*Clostridiaceae*	*Clostridium*	*Clostridium*
4859	*Firmicutes*	*Clostridia*	*Clostridia*	*Clostridiaceae*	*Clostridium*	*Clostridium*
4860	*Firmicutes*	*Clostridia*	*Clostridia*	*Clostridiaceae*	*Clostridium*	*Clostridium*
4861	*Firmicutes*	*Clostridia*	*Clostridia*	*Clostridiaceae*	*Clostridium*	*Clostridium*
4862	*Firmicutes*	*Clostridia*	*Clostridia*	*Clostridiaceae*	*Clostridium*	*Clostridium*
4863	*Firmicutes*	*Clostridia*	*Clostridia*	*Clostridiaceae*	*Clostridium*	*Clostridium*
4864	*Firmicutes*	*Clostridia*	*Clostridia*	*Clostridiaceae*	*Clostridium*	*Clostridium*
4865	*Firmicutes*	*Clostridia*	*Clostridia*	*Clostridiaceae*	*Clostridium*	*Clostridium*
4866	*Firmicutes*	*Clostridia*	*Clostridia*	*Clostridiaceae*	*Clostridium*	*Clostridium*
4867	*Firmicutes*	*Clostridia*	*Clostridia*	*Clostridiaceae*	*Clostridium*	*Clostridium*
4868	*Firmicutes*	*Clostridia*	*Clostridia*	*Clostridiaceae*	*Clostridium*	*Clostridium*
4869	*Firmicutes*	*Clostridia*	*Clostridia*	*Clostridiaceae*	*Clostridium*	*Clostridium*
4870	*Firmicutes*	*Clostridia*	*Clostridia*	*Clostridiaceae*	*Clostridium*	*Clostridium*
4871	*Firmicutes*	*Clostridia*	*Clostridia*	*Clostridiaceae*	*Clostridium*	*Clostridium*
4872	*Firmicutes*	*Clostridia*	*Clostridia*	*Clostridiaceae*	*Clostridium*	*Clostridium*

Table S4. Isolated strains by culturomics

bacteriaId	*phylum*	*class*	*order*	*family*	*genus*	*species*
4873	*Firmicutes*	*Clostridia*	*Clostridia*	*Clostridiaceae*	*Clostridium*	*Clostridium*
4874	*Firmicutes*	*Clostridia*	*Clostridia*	*Clostridiaceae*	*Clostridium*	*Clostridium*
4875	*Firmicutes*	*Clostridia*	*Clostridia*	*Clostridiaceae*	*Clostridium*	*Clostridium*
4876	*Firmicutes*	*Clostridia*	*Clostridia*	*Clostridiaceae*	*Clostridium*	*Clostridium*
4877	*Firmicutes*	*Clostridia*	*Clostridia*	*Clostridiaceae*	*Clostridium*	*Clostridium*
4878	*Firmicutes*	*Clostridia*	*Clostridia*	*Clostridiaceae*	*Clostridium*	*Clostridium*
4879	*Firmicutes*	*Clostridia*	*Clostridia*	*Clostridiaceae*	*Clostridium*	*Clostridium*
4880	*Firmicutes*	*Clostridia*	*Clostridia*	*Clostridiaceae*	*Clostridium*	*Clostridium*
4881	*Firmicutes*	*Clostridia*	*Clostridia*	*Clostridiaceae*	*Clostridium*	*Clostridium*
4882	*Firmicutes*	*Clostridia*	*Clostridia*	*Clostridiaceae*	*Clostridium*	*Clostridium*
4883	*Firmicutes*	*Clostridia*	*Clostridia*	*Clostridiaceae*	*Clostridium*	*Clostridium*
4884	*Firmicutes*	*Clostridia*	*Clostridia*	*Clostridiaceae*	*Clostridium*	*Clostridium*
4885	*Firmicutes*	*Clostridia*	*Clostridia*	*Clostridiaceae*	*Clostridium*	*Clostridium*
4886	*Firmicutes*	*Clostridia*	*Clostridia*	*Clostridiaceae*	*Clostridium*	*Clostridium*
4887	*Firmicutes*	*Clostridia*	*Clostridia*	*Clostridiaceae*	*Clostridium*	*Clostridium*
4888	*Firmicutes*	*Clostridia*	*Clostridia*	*Clostridiaceae*	*Clostridium*	*Clostridium*
4889	*Firmicutes*	*Clostridia*	*Clostridia*	*Clostridiaceae*	*Clostridium*	*Clostridium*
4890	*Firmicutes*	*Clostridia*	*Clostridia*	*Clostridiaceae*	*Clostridium*	*Clostridium*
4891	*Firmicutes*	*Clostridia*	*Clostridia*	*Clostridiaceae*	*Clostridium*	*Clostridium*
4892	*Firmicutes*	*Clostridia*	*Clostridia*	*Clostridiaceae*	*Clostridium*	*Clostridium*
4893	*Firmicutes*	*Clostridia*	*Clostridia*	*Clostridiaceae*	*Clostridium*	*Clostridium*
4894	*Firmicutes*	*Clostridia*	*Clostridia*	*Clostridiaceae*	*Clostridium*	*Clostridium*
4895	*Firmicutes*	*Clostridia*	*Clostridia*	*Clostridiaceae*	*Clostridium*	*Clostridium*
4896	*Firmicutes*	*Clostridia*	*Clostridia*	*Clostridiaceae*	*Clostridium*	*Clostridium*
4897	*Firmicutes*	*Clostridia*	*Clostridia*	*Clostridiaceae*	*Clostridium*	*Clostridium*
4898	*Firmicutes*	*Clostridia*	*Clostridia*	*Clostridiaceae*	*Clostridium*	*Clostridium*
4899	*Firmicutes*	*Clostridia*	*Clostridia*	*Clostridiaceae*	*Clostridium*	*Clostridium*
4900	*Firmicutes*	*Clostridia*	*Clostridia*	*Clostridiaceae*	*Clostridium*	*Clostridium*

Table S4. Isolated strains by culturomics

bacteriaId	*phylum*	*class*	*order*	*family*	*genus*	*species*
4901	*Firmicutes*	*Clostridia*	*Clostridia*	*Clostridiaceae*	*Clostridium*	*Clostridium*
4902	*Firmicutes*	*Clostridia*	*Clostridia*	*Clostridiaceae*	*Clostridium*	*Clostridium*
4903	*Firmicutes*	*Clostridia*	*Clostridia*	*Clostridiaceae*	*Clostridium*	*Clostridium*
4904	*Firmicutes*	*Clostridia*	*Clostridia*	*Clostridiaceae*	*Clostridium*	*Clostridium*
4905	*Firmicutes*	*Clostridia*	*Clostridia*	*Clostridiaceae*	*Clostridium*	*Clostridium*
4906	*Firmicutes*	*Clostridia*	*Clostridia*	*Clostridiaceae*	*Clostridium*	*Clostridium*
4907	*Firmicutes*	*Clostridia*	*Clostridia*	*Clostridiaceae*	*Clostridium*	*Clostridium*
4908	*Firmicutes*	*Clostridia*	*Clostridia*	*Clostridiaceae*	*Clostridium*	*Clostridium*
4909	*Firmicutes*	*Clostridia*	*Clostridia*	*Clostridiaceae*	*Clostridium*	*Clostridium*
4910	*Firmicutes*	*Clostridia*	*Clostridia*	*Clostridiaceae*	*Clostridium*	*Clostridium*
4911	*Firmicutes*	*Clostridia*	*Clostridia*	*Clostridiaceae*	*Clostridium*	*Clostridium*
4912	*Firmicutes*	*Clostridia*	*Clostridia*	*Clostridiaceae*	*Clostridium*	*Clostridium*
4913	*Firmicutes*	*Clostridia*	*Clostridia*	*Clostridiaceae*	*Clostridium*	*Clostridium*
4914	*Firmicutes*	*Clostridia*	*Clostridia*	*Clostridiaceae*	*Clostridium*	*Clostridium*
4915	*Firmicutes*	*Clostridia*	*Clostridia*	*Clostridiaceae*	*Clostridium*	*Clostridium*
4916	*Firmicutes*	*Clostridia*	*Clostridia*	*Clostridiaceae*	*Clostridium*	*Clostridium*
4917	*Firmicutes*	*Clostridia*	*Clostridia*	*Clostridiaceae*	*Clostridium*	*Clostridium*
4918	*Firmicutes*	*Clostridia*	*Clostridia*	*Clostridiaceae*	*Clostridium*	*Clostridium*
4919	*Firmicutes*	*Clostridia*	*Clostridia*	*Clostridiaceae*	*Clostridium*	*Clostridium*
4920	*Firmicutes*	*Clostridia*	*Clostridia*	*Clostridiaceae*	*Clostridium*	*Clostridium*
4921	*Firmicutes*	*Clostridia*	*Clostridia*	*Clostridiaceae*	*Clostridium*	*Clostridium*
4922	*Firmicutes*	*Clostridia*	*Clostridia*	*Clostridiaceae*	*Clostridium*	*Clostridium*
4923	*Firmicutes*	*Clostridia*	*Clostridia*	*Clostridiaceae*	*Clostridium*	*Clostridium*
4924	*Firmicutes*	*Clostridia*	*Clostridia*	*Clostridiaceae*	*Clostridium*	*Clostridium*
4925	*Firmicutes*	*Clostridia*	*Clostridia*	*Clostridiaceae*	*Clostridium*	*Clostridium*
4926	*Firmicutes*	*Clostridia*	*Clostridia*	*Clostridiaceae*	*Clostridium*	*Clostridium*
4927	*Firmicutes*	*Clostridia*	*Clostridia*	*Clostridiaceae*	*Clostridium*	*Clostridium*
4928	*Firmicutes*	*Clostridia*	*Clostridia*	*Clostridiaceae*	*Clostridium*	*Clostridium*

Table S4. Isolated strains by culturomics

bacteriaId	*phylum*	*class*	*order*	*family*	*genus*	*species*
4929	*Firmicutes*	*Clostridia*	*Clostridia*	*Clostridiaceae*	*Clostridium*	*Clostridium*
4930	*Firmicutes*	*Clostridia*	*Clostridia*	*Clostridiaceae*	*Clostridium*	*Clostridium*
4931	*Firmicutes*	*Clostridia*	*Clostridia*	*Clostridiaceae*	*Clostridium*	*Clostridium*
4932	*Firmicutes*	*Clostridia*	*Clostridia*	*Clostridiaceae*	*Clostridium*	*Clostridium*
4933	*Firmicutes*	*Clostridia*	*Clostridia*	*Clostridiaceae*	*Clostridium*	*Clostridium*
4934	*Firmicutes*	*Clostridia*	*Clostridia*	*Clostridiaceae*	*Clostridium*	*Clostridium*
4935	*Firmicutes*	*Clostridia*	*Clostridia*	*Clostridiaceae*	*Clostridium*	*Clostridium*
4936	*Firmicutes*	*Clostridia*	*Clostridia*	*Clostridiaceae*	*Clostridium*	*Clostridium*
4937	*Firmicutes*	*Clostridia*	*Clostridia*	*Clostridiaceae*	*Clostridium*	*Clostridium*
4938	*Firmicutes*	*Clostridia*	*Clostridia*	*Clostridiaceae*	*Clostridium*	*Clostridium*
4939	*Firmicutes*	*Clostridia*	*Clostridia*	*Clostridiaceae*	*Clostridium*	*Clostridium*
4940	*Firmicutes*	*Clostridia*	*Clostridia*	*Clostridiaceae*	*Clostridium*	*Clostridium*
4941	*Firmicutes*	*Clostridia*	*Clostridia*	*Clostridiaceae*	*Clostridium*	*Clostridium*
4942	*Firmicutes*	*Clostridia*	*Clostridia*	*Clostridiaceae*	*Clostridium*	*Clostridium*
4943	*Firmicutes*	*Clostridia*	*Clostridia*	*Clostridiaceae*	*Clostridium*	*Clostridium*
4944	*Firmicutes*	*Clostridia*	*Clostridia*	*Clostridiaceae*	*Clostridium*	*Clostridium*
4945	*Firmicutes*	*Clostridia*	*Clostridia*	*Clostridiaceae*	*Clostridium*	*Clostridium*
4946	*Firmicutes*	*Clostridia*	*Clostridia*	*Clostridiaceae*	*Clostridium*	*Clostridium*
4947	*Firmicutes*	*Clostridia*	*Clostridia*	*Clostridiaceae*	*Clostridium*	*Clostridium*
4948	*Firmicutes*	*Clostridia*	*Clostridia*	*Clostridiaceae*	*Clostridium*	*Clostridium*
4949	*Firmicutes*	*Clostridia*	*Clostridia*	*Clostridiaceae*	*Clostridium*	*Clostridium*
4950	*Firmicutes*	*Clostridia*	*Clostridia*	*Clostridiaceae*	*Clostridium*	*Clostridium*
4951	*Firmicutes*	*Clostridia*	*Clostridia*	*Clostridiaceae*	*Clostridium*	*Clostridium*
4952	*Firmicutes*	*Clostridia*	*Clostridia*	*Clostridiaceae*	*Clostridium*	*Clostridium*
4953	*Firmicutes*	*Clostridia*	*Clostridia*	*Clostridiaceae*	*Clostridium*	*Clostridium*
4954	*Firmicutes*	*Clostridia*	*Clostridia*	*Clostridiaceae*	*Clostridium*	*Clostridium*
4955	*Firmicutes*	*Clostridia*	*Clostridia*	*Clostridiaceae*	*Clostridium*	*Clostridium*
4956	*Firmicutes*	*Clostridia*	*Clostridia*	*Clostridiaceae*	*Clostridium*	*Clostridium*

Table S4. Isolated strains by culturomics

bacteriaId	*phylum*	*class*	*order*	*family*	*genus*	*species*
4957	*Firmicutes*	*Clostridia*	*Clostridia*	*Clostridiaceae*	*Clostridium*	*Clostridium*
4958	*Firmicutes*	*Clostridia*	*Clostridia*	*Clostridiaceae*	*Clostridium*	*Clostridium*
4959	*Firmicutes*	*Clostridia*	*Clostridia*	*Clostridiaceae*	*Clostridium*	*Clostridium*
4960	*Firmicutes*	*Clostridia*	*Clostridia*	*Clostridiaceae*	*Clostridium*	*Clostridium*
4961	*Firmicutes*	*Clostridia*	*Clostridia*	*Clostridiaceae*	*Clostridium*	*Clostridium*
4962	*Firmicutes*	*Clostridia*	*Clostridia*	*Clostridiaceae*	*Clostridium*	*Clostridium*
4963	*Firmicutes*	*Clostridia*	*Clostridia*	*Clostridiaceae*	*Clostridium*	*Clostridium*
4964	*Firmicutes*	*Clostridia*	*Clostridia*	*Clostridiaceae*	*Clostridium*	*Clostridium*
4965	*Firmicutes*	*Clostridia*	*Clostridia*	*Clostridiaceae*	*Clostridium*	*Clostridium*
4966	*Firmicutes*	*Clostridia*	*Clostridia*	*Clostridiaceae*	*Clostridium*	*Clostridium*
4967	*Firmicutes*	*Clostridia*	*Clostridia*	*Clostridiaceae*	*Clostridium*	*Clostridium*
4968	*Firmicutes*	*Clostridia*	*Clostridia*	*Clostridiaceae*	*Clostridium*	*Clostridium*
4969	*Firmicutes*	*Clostridia*	*Clostridia*	*Clostridiaceae*	*Clostridium*	*Clostridium*
4970	*Firmicutes*	*Clostridia*	*Clostridia*	*Clostridiaceae*	*Clostridium*	*Clostridium*
4971	*Firmicutes*	*Clostridia*	*Clostridia*	*Clostridiaceae*	*Clostridium*	*Clostridium*
4972	*Firmicutes*	*Clostridia*	*Clostridia*	*Clostridiaceae*	*Clostridium*	*Clostridium*
4973	*Firmicutes*	*Clostridia*	*Clostridia*	*Clostridiaceae*	*Clostridium*	*Clostridium*
4974	*Firmicutes*	*Clostridia*	*Clostridia*	*Clostridiaceae*	*Clostridium*	*Clostridium*
4975	*Firmicutes*	*Clostridia*	*Clostridia*	*Clostridiaceae*	*Clostridium*	*Clostridium*
4976	*Firmicutes*	*Clostridia*	*Clostridia*	*Clostridiaceae*	*Clostridium*	*Clostridium*
4977	*Firmicutes*	*Clostridia*	*Clostridia*	*Clostridiaceae*	*Clostridium*	*Clostridium*
4978	*Firmicutes*	*Clostridia*	*Clostridia*	*Clostridiaceae*	*Clostridium*	*Clostridium*
4979	*Firmicutes*	*Clostridia*	*Clostridia*	*Clostridiaceae*	*Clostridium*	*Clostridium*
4980	*Firmicutes*	*Clostridia*	*Clostridia*	*Clostridiaceae*	*Clostridium*	*Clostridium*
4981	*Firmicutes*	*Clostridia*	*Clostridia*	*Clostridiaceae*	*Clostridium*	*Clostridium*
4982	*Firmicutes*	*Clostridia*	*Clostridia*	*Clostridiaceae*	*Clostridium*	*Clostridium*
4983	*Firmicutes*	*Clostridia*	*Clostridia*	*Clostridiaceae*	*Clostridium*	*Clostridium*
4984	*Firmicutes*	*Clostridia*	*Clostridia*	*Clostridiaceae*	*Clostridium*	*Clostridium*

Table S4. Isolated strains by culturomics

bacteriaId	*phylum*	*class*	*order*	*family*	*genus*	*species*
4985	*Firmicutes*	*Clostridia*	*Clostridia*	*Clostridiaceae*	*Clostridium*	*Clostridium*
4986	*Firmicutes*	*Clostridia*	*Clostridia*	*Clostridiaceae*	*Clostridium*	*Clostridium*
4987	*Firmicutes*	*Clostridia*	*Clostridia*	*Clostridiaceae*	*Clostridium*	*Clostridium*
4988	*Firmicutes*	*Clostridia*	*Clostridia*	*Clostridiaceae*	*Clostridium*	*Clostridium*
4989	*Firmicutes*	*Clostridia*	*Clostridia*	*Clostridiaceae*	*Clostridium*	*Clostridium*
4990	*Firmicutes*	*Clostridia*	*Clostridia*	*Clostridiaceae*	*Clostridium*	*Clostridium*
4991	*Firmicutes*	*Clostridia*	*Clostridia*	*Clostridiaceae*	*Clostridium*	*Clostridium*
4992	*Firmicutes*	*Clostridia*	*Clostridia*	*Clostridiaceae*	*Clostridium*	*Clostridium*
4993	*Firmicutes*	*Clostridia*	*Clostridia*	*Clostridiaceae*	*Clostridium*	*Clostridium*
4994	*Firmicutes*	*Clostridia*	*Clostridia*	*Clostridiaceae*	*Clostridium*	*Clostridium*
4995	*Firmicutes*	*Clostridia*	*Clostridia*	*Clostridiaceae*	*Clostridium*	*Clostridium*
4996	*Firmicutes*	*Clostridia*	*Clostridia*	*Clostridiaceae*	*Clostridium*	*Clostridium*
4997	*Firmicutes*	*Clostridia*	*Clostridia*	*Clostridiaceae*	*Clostridium*	*Clostridium*
4998	*Firmicutes*	*Clostridia*	*Clostridia*	*Clostridiaceae*	*Clostridium*	*Clostridium*
4999	*Firmicutes*	*Clostridia*	*Clostridia*	*Clostridiaceae*	*Clostridium*	*Clostridium*
5000	*Firmicutes*	*Clostridia*	*Clostridia*	*Clostridiaceae*	*Clostridium*	*Clostridium*
5001	*Firmicutes*	*Clostridia*	*Clostridia*	*Clostridiaceae*	*Clostridium*	*Clostridium*
5002	*Firmicutes*	*Clostridia*	*Clostridia*	*Clostridiaceae*	*Clostridium*	*Clostridium*
5003	*Firmicutes*	*Clostridia*	*Clostridia*	*Clostridiaceae*	*Clostridium*	*Clostridium*
5004	*Firmicutes*	*Clostridia*	*Clostridia*	*Clostridiaceae*	*Clostridium*	*Clostridium*
5005	*Firmicutes*	*Clostridia*	*Clostridia*	*Clostridiaceae*	*Clostridium*	*Clostridium*
5006	*Firmicutes*	*Clostridia*	*Clostridia*	*Clostridiaceae*	*Clostridium*	*Clostridium*
5007	*Firmicutes*	*Clostridia*	*Clostridia*	*Clostridiaceae*	*Clostridium*	*Clostridium*
5008	*Firmicutes*	*Clostridia*	*Clostridia*	*Clostridiaceae*	*Clostridium*	*Clostridium*
5009	*Firmicutes*	*Clostridia*	*Clostridia*	*Clostridiaceae*	*Clostridium*	*Clostridium*
5010	*Firmicutes*	*Clostridia*	*Clostridia*	*Clostridiaceae*	*Clostridium*	*Clostridium*
5011	*Firmicutes*	*Clostridia*	*Clostridia*	*Clostridiaceae*	*Clostridium*	*Clostridium*
5012	*Firmicutes*	*Clostridia*	*Clostridia*	*Clostridiaceae*	*Clostridium*	*Clostridium*

Table S4. Isolated strains by culturomics

bacteriaId	*phylum*	*class*	*order*	*family*	*genus*	*species*
5013	*Firmicutes*	*Clostridia*	*Clostridia*	*Clostridiaceae*	*Clostridium*	*Clostridium*
5014	*Firmicutes*	*Clostridia*	*Clostridia*	*Clostridiaceae*	*Clostridium*	*Clostridium*
5015	*Firmicutes*	*Clostridia*	*Clostridia*	*Clostridiaceae*	*Clostridium*	*Clostridium*
5016	*Firmicutes*	*Clostridia*	*Clostridia*	*Clostridiaceae*	*Clostridium*	*Clostridium*
5017	*Firmicutes*	*Clostridia*	*Clostridia*	*Clostridiaceae*	*Clostridium*	*Clostridium*
5018	*Firmicutes*	*Clostridia*	*Clostridia*	*Clostridiaceae*	*Clostridium*	*Clostridium*
5019	*Firmicutes*	*Clostridia*	*Clostridia*	*Clostridiaceae*	*Clostridium*	*Clostridium*
5020	*Firmicutes*	*Clostridia*	*Clostridia*	*Clostridiaceae*	*Clostridium*	*Clostridium*
5021	*Firmicutes*	*Clostridia*	*Clostridia*	*Clostridiaceae*	*Clostridium*	*Clostridium*
5022	*Firmicutes*	*Clostridia*	*Clostridia*	*Clostridiaceae*	*Clostridium*	*Clostridium*
5023	*Firmicutes*	*Clostridia*	*Clostridia*	*Clostridiaceae*	*Clostridium*	*Clostridium*
5024	*Firmicutes*	*Clostridia*	*Clostridia*	*Clostridiaceae*	*Clostridium*	*Clostridium*
5025	*Firmicutes*	*Clostridia*	*Clostridia*	*Clostridiaceae*	*Clostridium*	*Clostridium*
5026	*Firmicutes*	*Clostridia*	*Clostridia*	*Clostridiaceae*	*Clostridium*	*Clostridium*
5027	*Firmicutes*	*Clostridia*	*Clostridia*	*Clostridiaceae*	*Clostridium*	*Clostridium*
5028	*Firmicutes*	*Clostridia*	*Clostridia*	*Clostridiaceae*	*Clostridium*	*Clostridium*
5029	*Firmicutes*	*Clostridia*	*Clostridia*	*Clostridiaceae*	*Clostridium*	*Clostridium*
5030	*Firmicutes*	*Clostridia*	*Clostridia*	*Clostridiaceae*	*Clostridium*	*Clostridium*
5031	*Firmicutes*	*Clostridia*	*Clostridia*	*Clostridiaceae*	*Clostridium*	*Clostridium*
5032	*Firmicutes*	*Clostridia*	*Clostridia*	*Clostridiaceae*	*Clostridium*	*Clostridium*
5033	*Firmicutes*	*Clostridia*	*Clostridia*	*Clostridiaceae*	*Clostridium*	*Clostridium*
5034	*Firmicutes*	*Clostridia*	*Clostridia*	*Clostridiaceae*	*Clostridium*	*Clostridium*
5035	*Firmicutes*	*Clostridia*	*Clostridia*	*Clostridiaceae*	*Clostridium*	*Clostridium*
5036	*Firmicutes*	*Clostridia*	*Clostridia*	*Clostridiaceae*	*Clostridium*	*Clostridium*
5037	*Firmicutes*	*Clostridia*	*Clostridia*	*Clostridiaceae*	*Clostridium*	*Clostridium*
5038	*Firmicutes*	*Clostridia*	*Clostridia*	*Clostridiaceae*	*Clostridium*	*Clostridium*
5039	*Firmicutes*	*Clostridia*	*Clostridia*	*Clostridiaceae*	*Clostridium*	*Clostridium*
5040	*Firmicutes*	*Clostridia*	*Clostridia*	*Clostridiaceae*	*Clostridium*	*Clostridium*

Table S4. Isolated strains by culturomics

bacteriaId	*phylum*	*class*	*order*	*family*	*genus*	*species*
5041	*Firmicutes*	*Clostridia*	*Clostridia*	*Clostridiaceae*	*Clostridium*	*Clostridium*
5042	*Firmicutes*	*Clostridia*	*Clostridia*	*Clostridiaceae*	*Clostridium*	*Clostridium*
5043	*Firmicutes*	*Clostridia*	*Clostridia*	*Clostridiaceae*	*Clostridium*	*Clostridium*
5044	*Firmicutes*	*Clostridia*	*Clostridia*	*Clostridiaceae*	*Clostridium*	*Clostridium*
5045	*Firmicutes*	*Clostridia*	*Clostridia*	*Clostridiaceae*	*Clostridium*	*Clostridium*
5046	*Firmicutes*	*Clostridia*	*Clostridia*	*Clostridiaceae*	*Clostridium*	*Clostridium*
5047	*Firmicutes*	*Clostridia*	*Clostridia*	*Clostridiaceae*	*Clostridium*	*Clostridium*
5048	*Firmicutes*	*Clostridia*	*Clostridia*	*Clostridiaceae*	*Clostridium*	*Clostridium*
5049	*Firmicutes*	*Clostridia*	*Clostridia*	*Clostridiaceae*	*Clostridium*	*Clostridium*
5050	*Firmicutes*	*Clostridia*	*Clostridia*	*Clostridiaceae*	*Clostridium*	*Clostridium*
5051	*Firmicutes*	*Clostridia*	*Clostridia*	*Clostridiaceae*	*Clostridium*	*Clostridium*
5052	*Firmicutes*	*Clostridia*	*Clostridia*	*Clostridiaceae*	*Clostridium*	*Clostridium*
5053	*Firmicutes*	*Clostridia*	*Clostridia*	*Clostridiaceae*	*Clostridium*	*Clostridium*
5054	*Firmicutes*	*Clostridia*	*Clostridia*	*Clostridiaceae*	*Clostridium*	*Clostridium*
5055	*Firmicutes*	*Clostridia*	*Clostridia*	*Clostridiaceae*	*Clostridium*	*Clostridium*
5056	*Firmicutes*	*Clostridia*	*Clostridia*	*Clostridiaceae*	*Clostridium*	*Clostridium*
5057	*Firmicutes*	*Clostridia*	*Clostridia*	*Clostridiaceae*	*Clostridium*	*Clostridium*
5058	*Firmicutes*	*Clostridia*	*Clostridia*	*Clostridiaceae*	*Clostridium*	*Clostridium*
5059	*Firmicutes*	*Clostridia*	*Clostridia*	*Clostridiaceae*	*Clostridium*	*Clostridium*
5060	*Firmicutes*	*Clostridia*	*Clostridia*	*Clostridiaceae*	*Clostridium*	*Clostridium*
5061	*Firmicutes*	*Clostridia*	*Clostridia*	*Clostridiaceae*	*Clostridium*	*Clostridium*
5062	*Firmicutes*	*Clostridia*	*Clostridia*	*Clostridiaceae*	*Clostridium*	*Clostridium*
5063	*Firmicutes*	*Clostridia*	*Clostridia*	*Clostridiaceae*	*Clostridium*	*Clostridium*
5064	*Firmicutes*	*Clostridia*	*Clostridia*	*Clostridiaceae*	*Clostridium*	*Clostridium*
5065	*Firmicutes*	*Clostridia*	*Clostridia*	*Clostridiaceae*	*Clostridium*	*Clostridium*
5066	*Firmicutes*	*Clostridia*	*Clostridia*	*Clostridiaceae*	*Clostridium*	*Clostridium*
5067	*Firmicutes*	*Clostridia*	*Clostridia*	*Clostridiaceae*	*Clostridium*	*Clostridium*
5068	*Firmicutes*	*Clostridia*	*Clostridia*	*Clostridiaceae*	*Clostridium*	*Clostridium*

Table S4. Isolated strains by culturomics

bacteriaId	*phylum*	*class*	*order*	*family*	*genus*	*species*
5069	*Firmicutes*	*Clostridia*	*Clostridia*	*Clostridiaceae*	*Clostridium*	*Clostridium*
5070	*Firmicutes*	*Clostridia*	*Clostridia*	*Clostridiaceae*	*Clostridium*	*Clostridium*
5071	*Firmicutes*	*Clostridia*	*Clostridia*	*Clostridiaceae*	*Clostridium*	*Clostridium*
5072	*Firmicutes*	*Clostridia*	*Clostridia*	*Clostridiaceae*	*Clostridium*	*Clostridium*
5073	*Firmicutes*	*Clostridia*	*Clostridia*	*Clostridiaceae*	*Clostridium*	*Clostridium*
5074	*Firmicutes*	*Clostridia*	*Clostridia*	*Clostridiaceae*	*Clostridium*	*Clostridium*
5075	*Firmicutes*	*Clostridia*	*Clostridia*	*Clostridiaceae*	*Clostridium*	*Clostridium*
5076	*Firmicutes*	*Clostridia*	*Clostridia*	*Clostridiaceae*	*Clostridium*	*Clostridium*
5077	*Firmicutes*	*Clostridia*	*Clostridia*	*Clostridiaceae*	*Clostridium*	*Clostridium*
5078	*Firmicutes*	*Clostridia*	*Clostridia*	*Clostridiaceae*	*Clostridium*	*Clostridium*
5079	*Firmicutes*	*Clostridia*	*Clostridia*	*Clostridiaceae*	*Clostridium*	*Clostridium*
5080	*Firmicutes*	*Clostridia*	*Clostridia*	*Clostridiaceae*	*Clostridium*	*Clostridium*
5081	*Firmicutes*	*Clostridia*	*Clostridia*	*Clostridiaceae*	*Clostridium*	*Clostridium*
5082	*Firmicutes*	*Clostridia*	*Clostridia*	*Clostridiaceae*	*Clostridium*	*Clostridium*
5083	*Firmicutes*	*Clostridia*	*Clostridia*	*Clostridiaceae*	*Clostridium*	*Clostridium*
5084	*Firmicutes*	*Clostridia*	*Clostridia*	*Clostridiaceae*	*Clostridium*	*Clostridium*
5085	*Firmicutes*	*Clostridia*	*Clostridia*	*Clostridiaceae*	*Clostridium*	*Clostridium*
5086	*Firmicutes*	*Clostridia*	*Clostridia*	*Clostridiaceae*	*Clostridium*	*Clostridium*
5087	*Firmicutes*	*Clostridia*	*Clostridia*	*Clostridiaceae*	*Clostridium*	*Clostridium*
5088	*Firmicutes*	*Clostridia*	*Clostridia*	*Clostridiaceae*	*Clostridium*	*Clostridium*
5089	*Firmicutes*	*Clostridia*	*Clostridia*	*Clostridiaceae*	*Clostridium*	*Clostridium*
5090	*Firmicutes*	*Clostridia*	*Clostridia*	*Clostridiaceae*	*Clostridium*	*Clostridium*
5091	*Firmicutes*	*Clostridia*	*Clostridia*	*Clostridiaceae*	*Clostridium*	*Clostridium*
5092	*Firmicutes*	*Clostridia*	*Clostridia*	*Clostridiaceae*	*Clostridium*	*Clostridium*
5093	*Firmicutes*	*Clostridia*	*Clostridia*	*Clostridiaceae*	*Clostridium*	*Clostridium*
5094	*Firmicutes*	*Clostridia*	*Clostridia*	*Clostridiaceae*	*Clostridium*	*Clostridium*
5095	*Firmicutes*	*Clostridia*	*Clostridia*	*Clostridiaceae*	*Clostridium*	*Clostridium*
5096	*Firmicutes*	*Clostridia*	*Clostridia*	*Clostridiaceae*	*Clostridium*	*Clostridium*

Table S4. Isolated strains by culturomics

bacteriaId	*phylum*	*class*	*order*	*family*	*genus*	*species*
5097	*Firmicutes*	*Clostridia*	*Clostridia*	*Clostridiaceae*	*Clostridium*	*Clostridium*
5098	*Firmicutes*	*Clostridia*	*Clostridia*	*Clostridiaceae*	*Clostridium*	*Clostridium*
5099	*Firmicutes*	*Clostridia*	*Clostridia*	*Clostridiaceae*	*Clostridium*	*Clostridium*
5100	*Firmicutes*	*Clostridia*	*Clostridia*	*Clostridiaceae*	*Clostridium*	*Clostridium*
5101	*Firmicutes*	*Clostridia*	*Clostridia*	*Clostridiaceae*	*Clostridium*	*Clostridium*
5102	*Firmicutes*	*Clostridia*	*Clostridia*	*Clostridiaceae*	*Clostridium*	*Clostridium*
5103	*Firmicutes*	*Clostridia*	*Clostridia*	*Clostridiaceae*	*Clostridium*	*Clostridium*
5104	*Firmicutes*	*Clostridia*	*Clostridia*	*Clostridiaceae*	*Clostridium*	*Clostridium*
5105	*Firmicutes*	*Clostridia*	*Clostridia*	*Clostridiaceae*	*Clostridium*	*Clostridium*
5106	*Firmicutes*	*Clostridia*	*Clostridia*	*Clostridiaceae*	*Clostridium*	*Clostridium*
5107	*Firmicutes*	*Clostridia*	*Clostridia*	*Clostridiaceae*	*Clostridium*	*Clostridium*
5108	*Firmicutes*	*Clostridia*	*Clostridia*	*Clostridiaceae*	*Clostridium*	*Clostridium*
5109	*Firmicutes*	*Clostridia*	*Clostridia*	*Clostridiaceae*	*Clostridium*	*Clostridium*
5110	*Firmicutes*	*Clostridia*	*Clostridia*	*Clostridiaceae*	*Clostridium*	*Clostridium*
5111	*Firmicutes*	*Clostridia*	*Clostridia*	*Clostridiaceae*	*Clostridium*	*Clostridium*
5112	*Firmicutes*	*Clostridia*	*Clostridia*	*Clostridiaceae*	*Clostridium*	*Clostridium*
5113	*Firmicutes*	*Clostridia*	*Clostridia*	*Clostridiaceae*	*Clostridium*	*Clostridium*
5114	*Firmicutes*	*Clostridia*	*Clostridia*	*Clostridiaceae*	*Clostridium*	*Clostridium*
5115	*Firmicutes*	*Clostridia*	*Clostridia*	*Clostridiaceae*	*Clostridium*	*Clostridium*
5116	*Firmicutes*	*Clostridia*	*Clostridia*	*Clostridiaceae*	*Clostridium*	*Clostridium*
5117	*Firmicutes*	*Clostridia*	*Clostridia*	*Clostridiaceae*	*Clostridium*	*Clostridium*
5118	*Firmicutes*	*Clostridia*	*Clostridia*	*Clostridiaceae*	*Clostridium*	*Clostridium*
5119	*Firmicutes*	*Clostridia*	*Clostridia*	*Clostridiaceae*	*Clostridium*	*Clostridium*
5120	*Firmicutes*	*Clostridia*	*Clostridia*	*Clostridiaceae*	*Clostridium*	*Clostridium*
5121	*Firmicutes*	*Clostridia*	*Clostridia*	*Clostridiaceae*	*Clostridium*	*Clostridium*
5122	*Firmicutes*	*Clostridia*	*Clostridia*	*Clostridiaceae*	*Clostridium*	*Clostridium*
5123	*Firmicutes*	*Clostridia*	*Clostridia*	*Clostridiaceae*	*Clostridium*	*Clostridium*
5124	*Firmicutes*	*Clostridia*	*Clostridia*	*Clostridiaceae*	*Clostridium*	*Clostridium*

Table S4. Isolated strains by culturomics

bacteriaId	*phylum*	*class*	*order*	*family*	*genus*	*species*
5125	*Firmicutes*	*Clostridia*	*Clostridia*	*Clostridiaceae*	*Clostridium*	*Clostridium*
5126	*Firmicutes*	*Clostridia*	*Clostridia*	*Clostridiaceae*	*Clostridium*	*Clostridium*
5127	*Firmicutes*	*Clostridia*	*Clostridia*	*Clostridiaceae*	*Clostridium*	*Clostridium*
5128	*Firmicutes*	*Clostridia*	*Clostridia*	*Clostridiaceae*	*Clostridium*	*Clostridium*
5129	*Firmicutes*	*Clostridia*	*Clostridia*	*Clostridiaceae*	*Clostridium*	*Clostridium*
5130	*Firmicutes*	*Clostridia*	*Clostridia*	*Clostridiaceae*	*Clostridium*	*Clostridium*
5131	*Firmicutes*	*Clostridia*	*Clostridia*	*Clostridiaceae*	*Clostridium*	*Clostridium*
5132	*Firmicutes*	*Clostridia*	*Clostridia*	*Clostridiaceae*	*Clostridium*	*Clostridium*
5133	*Firmicutes*	*Clostridia*	*Clostridia*	*Clostridiaceae*	*Clostridium*	*Clostridium*
5134	*Firmicutes*	*Clostridia*	*Clostridia*	*Clostridiaceae*	*Clostridium*	*Clostridium*
5135	*Firmicutes*	*Clostridia*	*Clostridia*	*Clostridiaceae*	*Clostridium*	*Clostridium*
5136	*Firmicutes*	*Clostridia*	*Clostridia*	*Clostridiaceae*	*Clostridium*	*Clostridium*
5137	*Firmicutes*	*Clostridia*	*Clostridia*	*Clostridiaceae*	*Clostridium*	*Clostridium*
5138	*Firmicutes*	*Clostridia*	*Clostridia*	*Clostridiaceae*	*Clostridium*	*Clostridium*
5139	*Firmicutes*	*Clostridia*	*Clostridia*	*Clostridiaceae*	*Clostridium*	*Clostridium*
5140	*Firmicutes*	*Clostridia*	*Clostridia*	*Clostridiaceae*	*Clostridium*	*Clostridium*
5141	*Firmicutes*	*Clostridia*	*Clostridia*	*Clostridiaceae*	*Clostridium*	*Clostridium*
5142	*Firmicutes*	*Clostridia*	*Clostridia*	*Clostridiaceae*	*Clostridium*	*Clostridium*
5143	*Firmicutes*	*Clostridia*	*Clostridia*	*Clostridiaceae*	*Clostridium*	*Clostridium*
5144	*Firmicutes*	*Clostridia*	*Clostridia*	*Clostridiaceae*	*Clostridium*	*Clostridium*
5145	*Firmicutes*	*Clostridia*	*Clostridia*	*Clostridiaceae*	*Clostridium*	*Clostridium*
5146	*Firmicutes*	*Clostridia*	*Clostridia*	*Clostridiaceae*	*Clostridium*	*Clostridium*
5147	*Firmicutes*	*Clostridia*	*Clostridia*	*Clostridiaceae*	*Clostridium*	*Clostridium*
5148	*Firmicutes*	*Clostridia*	*Clostridia*	*Clostridiaceae*	*Clostridium*	*Clostridium*
5149	*Firmicutes*	*Clostridia*	*Clostridia*	*Clostridiaceae*	*Clostridium*	*Clostridium*
5150	*Firmicutes*	*Clostridia*	*Clostridia*	*Clostridiaceae*	*Clostridium*	*Clostridium*
5151	*Firmicutes*	*Clostridia*	*Clostridia*	*Clostridiaceae*	*Clostridium*	*Clostridium*
5152	*Firmicutes*	*Clostridia*	*Clostridia*	*Clostridiaceae*	*Clostridium*	*Clostridium*

Table S4. Isolated strains by culturomics

bacteriaId	*phylum*	*class*	*order*	*family*	*genus*	*species*
5153	*Firmicutes*	*Clostridia*	*Clostridia*	*Clostridiaceae*	*Clostridium*	*Clostridium*
5154	*Firmicutes*	*Clostridia*	*Clostridia*	*Clostridiaceae*	*Clostridium*	*Clostridium*
5155	*Firmicutes*	*Clostridia*	*Clostridia*	*Clostridiaceae*	*Clostridium*	*Clostridium*
5156	*Firmicutes*	*Clostridia*	*Clostridia*	*Clostridiaceae*	*Clostridium*	*Clostridium*
5157	*Firmicutes*	*Clostridia*	*Clostridia*	*Clostridiaceae*	*Clostridium*	*Clostridium*
5158	*Firmicutes*	*Clostridia*	*Clostridia*	*Clostridiaceae*	*Clostridium*	*Clostridium*
5159	*Firmicutes*	*Clostridia*	*Clostridia*	*Clostridiaceae*	*Clostridium*	*Clostridium*
5160	*Firmicutes*	*Clostridia*	*Clostridia*	*Clostridiaceae*	*Clostridium*	*Clostridium*
5161	*Firmicutes*	*Clostridia*	*Clostridia*	*Clostridiaceae*	*Clostridium*	*Clostridium*
5162	*Firmicutes*	*Clostridia*	*Clostridia*	*Clostridiaceae*	*Clostridium*	*Clostridium*
5163	*Firmicutes*	*Clostridia*	*Clostridia*	*Clostridiaceae*	*Clostridium*	*Clostridium*
5164	*Firmicutes*	*Clostridia*	*Clostridia*	*Clostridiaceae*	*Clostridium*	*Clostridium*
5165	*Firmicutes*	*Clostridia*	*Clostridia*	*Clostridiaceae*	*Clostridium*	*Clostridium*
5166	*Firmicutes*	*Clostridia*	*Clostridia*	*Clostridiaceae*	*Clostridium*	*Clostridium*
5167	*Firmicutes*	*Clostridia*	*Clostridia*	*Clostridiaceae*	*Clostridium*	*Clostridium*
5168	*Firmicutes*	*Clostridia*	*Clostridia*	*Clostridiaceae*	*Clostridium*	*Clostridium*
5169	*Firmicutes*	*Clostridia*	*Clostridia*	*Clostridiaceae*	*Clostridium*	*Clostridium*
5170	*Firmicutes*	*Clostridia*	*Clostridia*	*Clostridiaceae*	*Clostridium*	*Clostridium*
5171	*Firmicutes*	*Clostridia*	*Clostridia*	*Clostridiaceae*	*Clostridium*	*Clostridium*
5172	*Firmicutes*	*Clostridia*	*Clostridia*	*Clostridiaceae*	*Clostridium*	*Clostridium*
5173	*Firmicutes*	*Clostridia*	*Clostridia*	*Clostridiaceae*	*Clostridium*	*Clostridium*
5174	*Firmicutes*	*Clostridia*	*Clostridia*	*Clostridiaceae*	*Clostridium*	*Clostridium*
5175	*Firmicutes*	*Clostridia*	*Clostridia*	*Clostridiaceae*	*Clostridium*	*Clostridium*
5176	*Firmicutes*	*Clostridia*	*Clostridia*	*Clostridiaceae*	*Clostridium*	*Clostridium*
5177	*Firmicutes*	*Clostridia*	*Clostridia*	*Clostridiaceae*	*Clostridium*	*Clostridium*
5178	*Firmicutes*	*Clostridia*	*Clostridia*	*Clostridiaceae*	*Clostridium*	*Clostridium*
5179	*Firmicutes*	*Clostridia*	*Clostridia*	*Clostridiaceae*	*Clostridium*	*Clostridium*
5180	*Firmicutes*	*Clostridia*	*Clostridia*	*Clostridiaceae*	*Clostridium*	*Clostridium*

Table S4. Isolated strains by culturomics

bacteriaId	*phylum*	*class*	*order*	*family*	*genus*	*species*
5181	*Firmicutes*	*Clostridia*	*Clostridia*	*Clostridiaceae*	*Clostridium*	*Clostridium*
5182	*Firmicutes*	*Clostridia*	*Clostridia*	*Clostridiaceae*	*Clostridium*	*Clostridium*
5183	*Firmicutes*	*Clostridia*	*Clostridia*	*Clostridiaceae*	*Clostridium*	*Clostridium*
5184	*Firmicutes*	*Clostridia*	*Clostridia*	*Clostridiaceae*	*Clostridium*	*Clostridium*
5185	*Firmicutes*	*Clostridia*	*Clostridia*	*Clostridiaceae*	*Clostridium*	*Clostridium*
5186	*Firmicutes*	*Clostridia*	*Clostridia*	*Clostridiaceae*	*Clostridium*	*Clostridium*
5187	*Firmicutes*	*Clostridia*	*Clostridia*	*Clostridiaceae*	*Clostridium*	*Clostridium*
5188	*Firmicutes*	*Clostridia*	*Clostridia*	*Clostridiaceae*	*Clostridium*	*Clostridium*
5189	*Firmicutes*	*Clostridia*	*Clostridia*	*Clostridiaceae*	*Clostridium*	*Clostridium*
5190	*Firmicutes*	*Clostridia*	*Clostridia*	*Clostridiaceae*	*Clostridium*	*Clostridium*
5191	*Firmicutes*	*Clostridia*	*Clostridia*	*Clostridiaceae*	*Clostridium*	*Clostridium*
5192	*Firmicutes*	*Clostridia*	*Clostridia*	*Clostridiaceae*	*Clostridium*	*Clostridium*
5193	*Firmicutes*	*Clostridia*	*Clostridia*	*Clostridiaceae*	*Clostridium*	*Clostridium*
5194	*Firmicutes*	*Clostridia*	*Clostridia*	*Clostridiaceae*	*Clostridium*	*Clostridium*
5195	*Firmicutes*	*Clostridia*	*Clostridia*	*Clostridiaceae*	*Clostridium*	*Clostridium*
5196	*Firmicutes*	*Clostridia*	*Clostridia*	*Clostridiaceae*	*Clostridium*	*Clostridium*
5197	*Firmicutes*	*Clostridia*	*Clostridia*	*Clostridiaceae*	*Clostridium*	*Clostridium*
5198	*Firmicutes*	*Clostridia*	*Clostridia*	*Clostridiaceae*	*Clostridium*	*Clostridium*
5199	*Firmicutes*	*Clostridia*	*Clostridia*	*Clostridiaceae*	*Clostridium*	*Clostridium*
5200	*Firmicutes*	*Clostridia*	*Clostridia*	*Clostridiaceae*	*Clostridium*	*Clostridium*
5201	*Firmicutes*	*Clostridia*	*Clostridia*	*Clostridiaceae*	*Clostridium*	*Clostridium*
5202	*Firmicutes*	*Clostridia*	*Clostridia*	*Clostridiaceae*	*Clostridium*	*Clostridium*
5203	*Firmicutes*	*Clostridia*	*Clostridia*	*Clostridiaceae*	*Clostridium*	*Clostridium*
5204	*Firmicutes*	*Clostridia*	*Clostridia*	*Clostridiaceae*	*Clostridium*	*Clostridium*
5205	*Firmicutes*	*Clostridia*	*Clostridia*	*Clostridiaceae*	*Clostridium*	*Clostridium*
5206	*Firmicutes*	*Clostridia*	*Clostridia*	*Clostridiaceae*	*Clostridium*	*Clostridium*
5207	*Firmicutes*	*Clostridia*	*Clostridia*	*Clostridiaceae*	*Clostridium*	*Clostridium*
5208	*Firmicutes*	*Clostridia*	*Clostridia*	*Clostridiaceae*	*Clostridium*	*Clostridium*

Table S4. Isolated strains by culturomics

bacteriaId	*phylum*	*class*	*order*	*family*	*genus*	*species*
5209	*Firmicutes*	*Clostridia*	*Clostridia*	*Clostridiaceae*	*Clostridium*	*Clostridium*
5210	*Firmicutes*	*Clostridia*	*Clostridia*	*Clostridiaceae*	*Clostridium*	*Clostridium*
5211	*Firmicutes*	*Clostridia*	*Clostridia*	*Clostridiaceae*	*Clostridium*	*Clostridium*
5212	*Firmicutes*	*Clostridia*	*Clostridia*	*Clostridiaceae*	*Clostridium*	*Clostridium*
5213	*Firmicutes*	*Clostridia*	*Clostridia*	*Clostridiaceae*	*Clostridium*	*Clostridium*
5214	*Firmicutes*	*Clostridia*	*Clostridia*	*Clostridiaceae*	*Clostridium*	*Clostridium*
5215	*Firmicutes*	*Clostridia*	*Clostridia*	*Clostridiaceae*	*Clostridium*	*Clostridium*
5216	*Firmicutes*	*Clostridia*	*Clostridia*	*Clostridiaceae*	*Clostridium*	*Clostridium*
5217	*Firmicutes*	*Clostridia*	*Clostridia*	*Clostridiaceae*	*Clostridium*	*Clostridium*
5218	*Firmicutes*	*Clostridia*	*Clostridia*	*Clostridiaceae*	*Clostridium*	*Clostridium*
5219	*Firmicutes*	*Clostridia*	*Clostridia*	*Clostridiaceae*	*Clostridium*	*Clostridium*
5220	*Firmicutes*	*Clostridia*	*Clostridia*	*Clostridiaceae*	*Clostridium*	*Clostridium*
5221	*Firmicutes*	*Clostridia*	*Clostridia*	*Clostridiaceae*	*Clostridium*	*Clostridium*
5222	*Firmicutes*	*Clostridia*	*Clostridia*	*Clostridiaceae*	*Clostridium*	*Clostridium*
5223	*Firmicutes*	*Clostridia*	*Clostridia*	*Clostridiaceae*	*Clostridium*	*Clostridium*
5224	*Firmicutes*	*Clostridia*	*Clostridia*	*Clostridiaceae*	*Clostridium*	*Clostridium*
5225	*Firmicutes*	*Clostridia*	*Clostridia*	*Clostridiaceae*	*Clostridium*	*Clostridium*
5226	*Firmicutes*	*Clostridia*	*Clostridia*	*Clostridiaceae*	*Clostridium*	*Clostridium*
5227	*Firmicutes*	*Clostridia*	*Clostridia*	*Clostridiaceae*	*Clostridium*	*Clostridium*
5228	*Firmicutes*	*Clostridia*	*Clostridia*	*Clostridiaceae*	*Clostridium*	*Clostridium*
5229	*Firmicutes*	*Clostridia*	*Clostridia*	*Clostridiaceae*	*Clostridium*	*Clostridium*
5230	*Firmicutes*	*Clostridia*	*Clostridia*	*Clostridiaceae*	*Clostridium*	*Clostridium*
5231	*Firmicutes*	*Clostridia*	*Clostridia*	*Clostridiaceae*	*Clostridium*	*Clostridium*
5232	*Firmicutes*	*Clostridia*	*Clostridia*	*Clostridiaceae*	*Clostridium*	*Clostridium*
5233	*Firmicutes*	*Clostridia*	*Clostridia*	*Clostridiaceae*	*Clostridium*	*Clostridium*
5234	*Firmicutes*	*Clostridia*	*Clostridia*	*Clostridiaceae*	*Clostridium*	*Clostridium*
5235	*Firmicutes*	*Clostridia*	*Clostridia*	*Clostridiaceae*	*Clostridium*	*Clostridium*
5236	*Firmicutes*	*Clostridia*	*Clostridia*	*Clostridiaceae*	*Clostridium*	*Clostridium*

Table S4. Isolated strains by culturomics

bacteriaId	*phylum*	*class*	*order*	*family*	*genus*	*species*
5237	*Firmicutes*	*Clostridia*	*Clostridia*	*Clostridiaceae*	*Clostridium*	*Clostridium*
5238	*Firmicutes*	*Clostridia*	*Clostridia*	*Clostridiaceae*	*Clostridium*	*Clostridium*
5239	*Firmicutes*	*Clostridia*	*Clostridia*	*Clostridiaceae*	*Clostridium*	*Clostridium*
5240	*Firmicutes*	*Clostridia*	*Clostridia*	*Clostridiaceae*	*Clostridium*	*Clostridium*
5241	*Firmicutes*	*Clostridia*	*Clostridia*	*Clostridiaceae*	*Clostridium*	*Clostridium*
5242	*Firmicutes*	*Clostridia*	*Clostridia*	*Clostridiaceae*	*Clostridium*	*Clostridium*
5243	*Firmicutes*	*Clostridia*	*Clostridia*	*Clostridiaceae*	*Clostridium*	*Clostridium*
5244	*Firmicutes*	*Clostridia*	*Clostridia*	*Clostridiaceae*	*Clostridium*	*Clostridium*
5245	*Firmicutes*	*Clostridia*	*Clostridia*	*Clostridiaceae*	*Clostridium*	*Clostridium*
5246	*Firmicutes*	*Clostridia*	*Clostridia*	*Clostridiaceae*	*Clostridium*	*Clostridium*
5247	*Firmicutes*	*Clostridia*	*Clostridia*	*Clostridiaceae*	*Clostridium*	*Clostridium*
5248	*Firmicutes*	*Clostridia*	*Clostridia*	*Clostridiaceae*	*Clostridium*	*Clostridium*
5249	*Firmicutes*	*Clostridia*	*Clostridia*	*Clostridiaceae*	*Clostridium*	*Clostridium*
5250	*Firmicutes*	*Clostridia*	*Clostridia*	*Clostridiaceae*	*Clostridium*	*Clostridium*
5251	*Firmicutes*	*Clostridia*	*Clostridia*	*Clostridiaceae*	*Clostridium*	*Clostridium*
5252	*Firmicutes*	*Clostridia*	*Clostridia*	*Clostridiaceae*	*Clostridium*	*Clostridium*
5253	*Firmicutes*	*Clostridia*	*Clostridia*	*Clostridiaceae*	*Clostridium*	*Clostridium*
5254	*Firmicutes*	*Clostridia*	*Clostridia*	*Clostridiaceae*	*Clostridium*	*Clostridium*
5255	*Firmicutes*	*Clostridia*	*Clostridia*	*Clostridiaceae*	*Clostridium*	*Clostridium*
5256	*Firmicutes*	*Clostridia*	*Clostridia*	*Clostridiaceae*	*Clostridium*	*Clostridium*
5257	*Firmicutes*	*Clostridia*	*Clostridia*	*Clostridiaceae*	*Clostridium*	*Clostridium*
5258	*Firmicutes*	*Clostridia*	*Clostridia*	*Clostridiaceae*	*Clostridium*	*Clostridium bolteae*
5259	*Firmicutes*	*Clostridia*	*Clostridia*	*Clostridiaceae*	*Clostridium*	*Clostridium ihumii*
5260	*Firmicutes*	*Clostridia*	*Clostridia*	*Clostridiaceae*	*Clostridium*	*Clostridium*
5261	*Firmicutes*	*Clostridia*	*Clostridia*	*Clostridiaceae*	*Clostridium*	*Clostridium*
5262	*Firmicutes*	*Clostridia*	*Clostridia*	*Clostridiaceae*	*Clostridium*	*Clostridium*
5263	*Firmicutes*	*Clostridia*	*Clostridia*	*Clostridiaceae*	*Clostridium*	*Clostridium*
5264	*Firmicutes*	*Clostridia*	*Clostridia*	*Clostridiaceae*	*Clostridium*	*Clostridium*

Table S4. Isolated strains by culturomics

bacteriaId	*phylum*	*class*	*order*	*family*	*genus*	*species*
5265	*Firmicutes*	*Clostridia*	*Clostridia*	*Clostridiaceae*	*Clostridium*	*Clostridium*
5266	*Firmicutes*	*Clostridia*	*Clostridia*	*Clostridiaceae*	*Clostridium*	*Clostridium_biferm*
5267	*Firmicutes*	*Clostridia*	*Clostridia*	*Clostridiaceae*	*Clostridium*	*Clostridium_biferm*
5268	*Firmicutes*	*Clostridia*	*Clostridia*	*Clostridiaceae*	*Clostridium*	*Clostridium_biferm*
5269	*Firmicutes*	*Clostridia*	*Clostridia*	*Clostridiaceae*	*Clostridium*	*Clostridium_biferm*
5270	*Firmicutes*	*Clostridia*	*Clostridia*	*Clostridiaceae*	*Clostridium*	*Clostridium_sp_7_2*
5271	*Firmicutes*	*Clostridia*	*Clostridia*	*Clostridiaceae*	*Clostridium*	*Clostridium_sp_7_2*
5272	*Firmicutes*	*Clostridia*	*Clostridia*	*Clostridiaceae*	*Clostridium*	*Clostridium_sp_7_2*
5273	*Firmicutes*	*Clostridia*	*Clostridia*	*Clostridiaceae*	*Clostridium*	*Clostridium_sp_7_2*
5274	*Firmicutes*	*Clostridia*	*Clostridia*	*Clostridiaceae*	*Clostridium*	*Clostridium_sp_7_2*
5275	*Firmicutes*	*Clostridia*	*Clostridia*	*Clostridiaceae*	*Clostridium*	*Clostridium_sp_7_2*
5276	*Firmicutes*	*Clostridia*	*Clostridia*	*Clostridiaceae*	*Clostridium*	*Clostridium_sp_7_2*
5277	*Firmicutes*	*Clostridia*	*Clostridia*	*Clostridiaceae*	*Clostridium*	*Clostridium_sp_7_2*
5278	*Firmicutes*	*Clostridia*	*Clostridia*	*Clostridiaceae*	*Clostridium*	*Clostridium_sp_7_2*
5279	*Firmicutes*	*Clostridia*	*Clostridiales*	*Clostridiaceae*	*Clostridium*	*Clostridium baratii*
5280	*Firmicutes*	*Clostridia*	*Clostridiales*	*Clostridiaceae*	*Clostridium*	*Clostridium baratii*
5281	*Firmicutes*	*Clostridia*	*Clostridiales*	*Clostridiaceae*	*Clostridium*	*Clostridium baratii*
5282	*Firmicutes*	*Clostridia*	*Clostridiales*	*Clostridiaceae*	*Clostridium*	*Clostridium baratii*
5283	*Firmicutes*	*Clostridia*	*Clostridiales*	*Clostridiaceae*	*Clostridium*	*Clostridium baratii*
5284	*Firmicutes*	*Clostridia*	*Clostridiales*	*Clostridiaceae*	*Clostridium*	*Clostridium baratii*
5285	*Firmicutes*	*Clostridia*	*Clostridiales*	*Clostridiaceae*	*Clostridium*	*Clostridium baratii*
5286	*Firmicutes*	*Clostridia*	*Clostridiales*	*Clostridiaceae*	*Clostridium*	*Clostridium baratii*
5287	*Firmicutes*	*Clostridia*	*Clostridiales*	*Clostridiaceae*	*Clostridium*	*Clostridium baratii*
5288	*Firmicutes*	*Clostridia*	*Clostridiales*	*Clostridiaceae*	*Clostridium*	*Clostridium baratii*
5289	*Firmicutes*	*Clostridia*	*Clostridiales*	*Clostridiaceae*	*Clostridium*	*Clostridium*
5290	*Firmicutes*	*Clostridia*	*Clostridiales*	*Clostridiaceae*	*Clostridium*	*Clostridium*
5291	*Firmicutes*	*Clostridia*	*Clostridiales*	*Clostridiaceae*	*Clostridium*	*Clostridium*
5292	*Firmicutes*	*Clostridia*	*Clostridiales*	*Clostridiaceae*	*Clostridium*	*Clostridium*

Table S4. Isolated strains by culturomics

bacteriaId	*phylum*	*class*	*order*	*family*	*genus*	*species*
5293	*Firmicutes*	*Clostridia*	*Clostridiales*	*Clostridiaceae*	*Clostridium*	*Clostridium*
5294	*Firmicutes*	*Clostridia*	*Clostridiales*	*Clostridiaceae*	*Clostridium*	*Clostridium*
5295	*Firmicutes*	*Clostridia*	*Clostridiales*	*Clostridiaceae*	*Clostridium*	*Clostridium*
5296	*Firmicutes*	*Clostridia*	*Clostridiales*	*Clostridiaceae*	*Clostridium*	*Clostridium*
5297	*Firmicutes*	*Clostridia*	*Clostridiales*	*Clostridiaceae*	*Clostridium*	*Clostridium*
5298	*Firmicutes*	*Clostridia*	*Clostridiales*	*Clostridiaceae*	*Clostridium*	*Clostridium*
5299	*Firmicutes*	*Clostridia*	*Clostridiales*	*Clostridiaceae*	*Clostridium*	*Clostridium*
5300	*Firmicutes*	*Clostridia*	*Clostridiales*	*Clostridiaceae*	*Clostridium*	*Clostridium*
5301	*Firmicutes*	*Clostridia*	*Clostridiales*	*Clostridiaceae*	*Clostridium*	*Clostridium*
5302	*Firmicutes*	*Clostridia*	*Clostridiales*	*Clostridiaceae*	*Clostridium*	*Clostridium*
5303	*Firmicutes*	*Clostridia*	*Clostridiales*	*Clostridiaceae*	*Clostridium*	*Clostridium*
5304	*Firmicutes*	*Clostridia*	*Clostridiales*	*Clostridiaceae*	*Clostridium*	*Clostridium*
5305	*Firmicutes*	*Clostridia*	*Clostridiales*	*Clostridiaceae*	*Clostridium*	*Clostridium*
5306	*Firmicutes*	*Clostridia*	*Clostridiales*	*Clostridiaceae*	*Clostridium*	*Clostridium*
5307	*Firmicutes*	*Clostridia*	*Clostridiales*	*Clostridiaceae*	*Clostridium*	*Clostridium*
5308	*Firmicutes*	*Clostridia*	*Clostridiales*	*Clostridiaceae*	*Clostridium*	*Clostridium*
5309	*Firmicutes*	*Clostridia*	*Clostridiales*	*Clostridiaceae*	*Clostridium*	*Clostridium*
5310	*Firmicutes*	*Clostridia*	*Clostridiales*	*Clostridiaceae*	*Clostridium*	*Clostridium*
5311	*Firmicutes*	*Clostridia*	*Clostridiales*	*Clostridiaceae*	*Clostridium*	*Clostridium*
5312	*Firmicutes*	*Clostridia*	*Clostridiales*	*Clostridiaceae*	*Clostridium*	*Clostridium*
5313	*Firmicutes*	*Clostridia*	*Clostridiales*	*Clostridiaceae*	*Clostridium*	*Clostridium*
5314	*Firmicutes*	*Clostridia*	*Clostridiales*	*Clostridiaceae*	*Clostridium*	*Clostridium*
5315	*Firmicutes*	*Clostridia*	*Clostridiales*	*Clostridiaceae*	*Clostridium*	*Clostridium*
5316	*Firmicutes*	*Clostridia*	*Clostridiales*	*Clostridiaceae*	*Clostridium*	*Clostridium*
5317	*Firmicutes*	*Clostridia*	*Clostridiales*	*Clostridiaceae*	*Clostridium*	*Clostridium*
5318	*Firmicutes*	*Clostridia*	*Clostridiales*	*Clostridiaceae*	*Clostridium*	*Clostridium*
5319	*Firmicutes*	*Clostridia*	*Clostridiales*	*Clostridiaceae*	*Clostridium*	*Clostridium*
5320	*Firmicutes*	*Clostridia*	*Clostridiales*	*Clostridiaceae*	*Clostridium*	*Clostridium*

Table S4. Isolated strains by culturomics

bacteriaId	*phylum*	*class*	*order*	*family*	*genus*	*species*
5321	*Firmicutes*	*Clostridia*	*Clostridiales*	*Clostridiaceae*	*Clostridium*	*Clostridium*
5322	*Firmicutes*	*Clostridia*	*Clostridiales*	*Clostridiaceae*	*Clostridium*	*Clostridium*
5323	*Firmicutes*	*Clostridia*	*Clostridiales*	*Clostridiaceae*	*Clostridium*	*Clostridium*
5324	*Firmicutes*	*Clostridia*	*Clostridiales*	*Clostridiaceae*	*Clostridium*	*Clostridium*
5325	*Firmicutes*	*Clostridia*	*Clostridiales*	*Clostridiaceae*	*Clostridium*	*Clostridium*
5326	*Firmicutes*	*Clostridia*	*Clostridiales*	*Clostridiaceae*	*Clostridium*	*Clostridium*
5327	*Firmicutes*	*Clostridia*	*Clostridiales*	*Clostridiaceae*	*Clostridium*	*Clostridium*
5328	*Firmicutes*	*Clostridia*	*Clostridiales*	*Clostridiaceae*	*Clostridium*	*Clostridium*
5329	*Firmicutes*	*Clostridia*	*Clostridiales*	*Clostridiaceae*	*Clostridium*	*Clostridium*
5330	*Firmicutes*	*Clostridia*	*Clostridiales*	*Clostridiaceae*	*Clostridium*	*Clostridium*
5331	*Firmicutes*	*Clostridia*	*Clostridiales*	*Clostridiaceae*	*Clostridium*	*Clostridium*
5332	*Firmicutes*	*Clostridia*	*Clostridiales*	*Clostridiaceae*	*Clostridium*	*Clostridium*
5333	*Firmicutes*	*Clostridia*	*Clostridiales*	*Clostridiaceae*	*Clostridium*	*Clostridium*
5334	*Firmicutes*	*Clostridia*	*Clostridiales*	*Clostridiaceae*	*Clostridium*	*Clostridium*
5335	*Firmicutes*	*Clostridia*	*Clostridiales*	*Clostridiaceae*	*Clostridium*	*Clostridium*
5336	*Firmicutes*	*Clostridia*	*Clostridiales*	*Clostridiaceae*	*Clostridium*	*Clostridium*
5337	*Firmicutes*	*Clostridia*	*Clostridiales*	*Clostridiaceae*	*Clostridium*	*Clostridium*
5338	*Firmicutes*	*Clostridia*	*Clostridiales*	*Clostridiaceae*	*Clostridium*	*Clostridium*
5339	*Firmicutes*	*Clostridia*	*Clostridiales*	*Clostridiaceae*	*Clostridium*	*Clostridium*
5340	*Firmicutes*	*Clostridia*	*Clostridiales*	*Clostridiaceae*	*Clostridium*	*Clostridium*
5341	*Firmicutes*	*Clostridia*	*Clostridiales*	*Clostridiaceae*	*Clostridium*	*Clostridium*
5342	*Firmicutes*	*Clostridia*	*Clostridiales*	*Clostridiaceae*	*Clostridium*	*Clostridium*
5343	*Firmicutes*	*Clostridia*	*Clostridiales*	*Clostridiaceae*	*Clostridium*	*Clostridium*
5344	*Firmicutes*	*Clostridia*	*Clostridiales*	*Clostridiaceae*	*Clostridium*	*Clostridium*
5345	*Firmicutes*	*Clostridia*	*Clostridiales*	*Clostridiaceae*	*Clostridium*	*Clostridium*
5346	*Firmicutes*	*Clostridia*	*Clostridiales*	*Clostridiaceae*	*Clostridium*	*Clostridium*
5347	*Firmicutes*	*Clostridia*	*Clostridiales*	*Clostridiaceae*	*Clostridium*	*Clostridium*
5348	*Firmicutes*	*Clostridia*	*Clostridiales*	*Clostridiaceae*	*Clostridium*	*Clostridium*

Table S4. Isolated strains by culturomics

bacteriaId	*phylum*	*class*	*order*	*family*	*genus*	*species*
5349	*Firmicutes*	*Clostridia*	*Clostridiales*	*Clostridiaceae*	*Clostridium*	*Clostridium*
5350	*Firmicutes*	*Clostridia*	*Clostridiales*	*Clostridiaceae*	*Clostridium*	*Clostridium*
5351	*Firmicutes*	*Clostridia*	*Clostridiales*	*Clostridiaceae*	*Clostridium*	*Clostridium*
5352	*Firmicutes*	*Clostridia*	*Clostridiales*	*Clostridiaceae*	*Clostridium*	*Clostridium*
5353	*Firmicutes*	*Clostridia*	*Clostridiales*	*Clostridiaceae*	*Clostridium*	*Clostridium*
5354	*Firmicutes*	*Clostridia*	*Clostridiales*	*Clostridiaceae*	*Clostridium*	*Clostridium*
5355	*Firmicutes*	*Clostridia*	*Clostridiales*	*Clostridiaceae*	*Clostridium*	*Clostridium*
5356	*Firmicutes*	*Clostridia*	*Clostridiales*	*Clostridiaceae*	*Clostridium*	*Clostridium*
5357	*Firmicutes*	*Clostridia*	*Clostridiales*	*Clostridiaceae*	*Clostridium*	*Clostridium*
5358	*Firmicutes*	*Clostridia*	*Clostridiales*	*Clostridiaceae*	*Clostridium*	*Clostridium*
5359	*Firmicutes*	*Clostridia*	*Clostridiales*	*Clostridiaceae*	*Clostridium*	*Clostridium*
5360	*Firmicutes*	*Clostridia*	*Clostridiales*	*Clostridiaceae*	*Clostridium*	*Clostridium*
5361	*Firmicutes*	*Clostridia*	*Clostridiales*	*Clostridiaceae*	*Clostridium*	*Clostridium*
5362	*Firmicutes*	*Clostridia*	*Clostridiales*	*Clostridiaceae*	*Clostridium*	*Clostridium*
5363	*Firmicutes*	*Clostridia*	*Clostridiales*	*Clostridiaceae*	*Clostridium*	*Clostridium*
5364	*Firmicutes*	*Clostridia*	*Clostridiales*	*Clostridiaceae*	*Clostridium*	*Clostridium*
5365	*Firmicutes*	*Clostridia*	*Clostridiales*	*Clostridiaceae*	*Clostridium*	*Clostridium*
5366	*Firmicutes*	*Clostridia*	*Clostridiales*	*Clostridiaceae*	*Clostridium*	*Clostridium*
5367	*Firmicutes*	*Clostridia*	*Clostridiales*	*Clostridiaceae*	*Clostridium*	*Clostridium*
5368	*Firmicutes*	*Clostridia*	*Clostridiales*	*Clostridiaceae*	*Clostridium*	*Clostridium*
5369	*Firmicutes*	*Clostridia*	*Clostridiales*	*Clostridiaceae*	*Clostridium*	*Clostridium*
5370	*Firmicutes*	*Clostridia*	*Clostridiales*	*Clostridiaceae*	*Clostridium*	*Clostridium*
5371	*Firmicutes*	*Clostridia*	*Clostridiales*	*Clostridiaceae*	*Clostridium*	*Clostridium*
5372	*Firmicutes*	*Clostridia*	*Clostridiales*	*Clostridiaceae*	*Clostridium*	*Clostridium*
5373	*Firmicutes*	*Clostridia*	*Clostridiales*	*Clostridiaceae*	*Clostridium*	*Clostridium*
5374	*Firmicutes*	*Clostridia*	*Clostridiales*	*Clostridiaceae*	*Clostridium*	*Clostridium difficile*
5375	*Firmicutes*	*Clostridia*	*Clostridiales*	*Clostridiaceae*	*Clostridium*	*Clostridium difficile*
5376	*Firmicutes*	*Clostridia*	*Clostridiales*	*Clostridiaceae*	*Clostridium*	*Clostridium difficile*

Table S4. Isolated strains by culturomics

bacteriaId	*phylum*	*class*	*order*	*family*	*genus*	*species*
5377	*Firmicutes*	*Clostridia*	*Clostridiales*	*Clostridiaceae*	*Clostridium*	*Clostridium*
5378	*Firmicutes*	*Clostridia*	*Clostridiales*	*Clostridiaceae*	*Clostridium*	*Clostridium*
5379	*Firmicutes*	*Clostridia*	*Clostridiales*	*Clostridiaceae*	*Clostridium*	*Clostridium*
5380	*Firmicutes*	*Clostridia*	*Clostridiales*	*Clostridiaceae*	*Clostridium*	*Clostridium*
5381	*Firmicutes*	*Clostridia*	*Clostridiales*	*Clostridiaceae*	*Clostridium*	*Clostridium*
5382	*Firmicutes*	*Clostridia*	*Clostridiales*	*Clostridiaceae*	*Clostridium*	*Clostridium*
5383	*Firmicutes*	*Clostridia*	*Clostridiales*	*Clostridiaceae*	*Clostridium*	*Clostridium*
5384	*Firmicutes*	*Clostridia*	*Clostridiales*	*Clostridiaceae*	*Clostridium*	*Clostridium*
5385	*Firmicutes*	*Clostridia*	*Clostridiales*	*Clostridiaceae*	*Clostridium*	*Clostridium*
5386	*Firmicutes*	*Clostridia*	*Clostridiales*	*Clostridiaceae*	*Clostridium*	*Clostridium*
5387	*Firmicutes*	*Clostridia*	*Clostridiales*	*Clostridiaceae*	*Clostridium*	*Clostridium*
5388	*Firmicutes*	*Clostridia*	*Clostridiales*	*Clostridiaceae*	*Clostridium*	*Clostridium*
5389	*Firmicutes*	*Clostridia*	*Clostridiales*	*Clostridiaceae*	*Clostridium*	*Clostridium*
5390	*Firmicutes*	*Clostridia*	*Clostridiales*	*Clostridiaceae*	*Clostridium*	*Clostridium*
5391	*Firmicutes*	*Clostridia*	*Clostridiales*	*Clostridiaceae*	*Clostridium*	*Clostridium*
5392	*Firmicutes*	*Clostridia*	*Clostridiales*	*Clostridiaceae*	*Clostridium*	*Clostridium*
5393	*Firmicutes*	*Clostridia*	*Clostridiales*	*Clostridiaceae*	*Clostridium*	*Clostridium*
5394	*Firmicutes*	*Clostridia*	*Clostridiales*	*Clostridiaceae*	*Clostridium*	*Clostridium*
5395	*Firmicutes*	*Clostridia*	*Clostridiales*	*Clostridiaceae*	*Clostridium*	*Clostridium ihumii*
5396	*Firmicutes*	*Clostridia*	*Clostridiales*	*Clostridiaceae*	*Clostridium*	*Clostridium ihumii*
5397	*Firmicutes*	*Clostridia*	*Clostridiales*	*Clostridiaceae*	*Clostridium*	*Clostridium ihumii*
5398	*Firmicutes*	*Clostridia*	*Clostridiales*	*Clostridiaceae*	*Clostridium*	*Clostridium ihumii*
5399	*Firmicutes*	*Clostridia*	*Clostridiales*	*Clostridiaceae*	*Clostridium*	*Clostridium ihumii*
5400	*Firmicutes*	*Clostridia*	*Clostridiales*	*Clostridiaceae*	*Clostridium*	*Clostridium ihumii*
5401	*Firmicutes*	*Clostridia*	*Clostridiales*	*Clostridiaceae*	*Clostridium*	*Clostridium ihumii*
5402	*Firmicutes*	*Clostridia*	*Clostridiales*	*Clostridiaceae*	*Clostridium*	*Clostridium ihumii*
5403	*Firmicutes*	*Clostridia*	*Clostridiales*	*Clostridiaceae*	*Clostridium*	*Clostridium ihumii*
5404	*Firmicutes*	*Clostridia*	*Clostridiales*	*Clostridiaceae*	*Clostridium*	*Clostridium ihumii*

Table S4. Isolated strains by culturomics

bacteriaId	*phylum*	*class*	*order*	*family*	*genus*	*species*
5405	*Firmicutes*	*Clostridia*	*Clostridiales*	*Clostridiaceae*	*Clostridium*	*Clostridium ihumii*
5406	*Firmicutes*	*Clostridia*	*Clostridiales*	*Clostridiaceae*	*Clostridium*	*Clostridium*
5407	*Firmicutes*	*Clostridia*	*Clostridiales*	*Clostridiaceae*	*Clostridium*	*Clostridium*
5408	*Firmicutes*	*Clostridia*	*Clostridiales*	*Clostridiaceae*	*Clostridium*	*Clostridium*
5409	*Firmicutes*	*Clostridia*	*Clostridiales*	*Clostridiaceae*	*Clostridium*	*Clostridium*
5410	*Firmicutes*	*Clostridia*	*Clostridiales*	*Clostridiaceae*	*Clostridium*	*Clostridium*
5411	*Firmicutes*	*Clostridia*	*Clostridiales*	*Clostridiaceae*	*Clostridium*	*Clostridium*
5412	*Firmicutes*	*Clostridia*	*Clostridiales*	*Clostridiaceae*	*Clostridium*	*Clostridium*
5413	*Firmicutes*	*Clostridia*	*Clostridiales*	*Clostridiaceae*	*Clostridium*	*Clostridium*
5414	*Firmicutes*	*Clostridia*	*Clostridiales*	*Clostridiaceae*	*Clostridium*	*Clostridium*
5415	*Firmicutes*	*Clostridia*	*Clostridiales*	*Clostridiaceae*	*Clostridium*	*Clostridium*
5416	*Firmicutes*	*Clostridia*	*Clostridiales*	*Clostridiaceae*	*Clostridium*	*Clostridium*
5417	*Firmicutes*	*Clostridia*	*Clostridiales*	*Clostridiaceae*	*Clostridium*	*Clostridium*
5418	*Firmicutes*	*Clostridia*	*Clostridiales*	*Clostridiaceae*	*Clostridium*	*Clostridium*
5419	*Firmicutes*	*Clostridia*	*Clostridiales*	*Clostridiaceae*	*Clostridium*	*Clostridium*
5420	*Firmicutes*	*Clostridia*	*Clostridiales*	*Clostridiaceae*	*Clostridium*	*Clostridium*
5421	*Firmicutes*	*Clostridia*	*Clostridiales*	*Clostridiaceae*	*Clostridium*	*Clostridium*
5422	*Firmicutes*	*Clostridia*	*Clostridiales*	*Clostridiaceae*	*Clostridium*	*Clostridium*
5423	*Firmicutes*	*Clostridia*	*Clostridiales*	*Clostridiaceae*	*Clostridium*	*Clostridium*
5424	*Firmicutes*	*Clostridia*	*Clostridiales*	*Clostridiaceae*	*Clostridium*	*Clostridium*
5425	*Firmicutes*	*Clostridia*	*Clostridiales*	*Clostridiaceae*	*Clostridium*	*Clostridium*
5426	*Firmicutes*	*Clostridia*	*Clostridiales*	*Clostridiaceae*	*Clostridium*	*Clostridium*
5427	*Firmicutes*	*Clostridia*	*Clostridiales*	*Clostridiaceae*	*Clostridium*	*Clostridium*
5428	*Firmicutes*	*Clostridia*	*Clostridiales*	*Clostridiaceae*	*Clostridium*	*Clostridium*
5429	*Firmicutes*	*Clostridia*	*Clostridiales*	*Clostridiaceae*	*Clostridium*	*Clostridium*
5430	*Firmicutes*	*Clostridia*	*Clostridiales*	*Clostridiaceae*	*Clostridium*	*Clostridium*
5431	*Firmicutes*	*Clostridia*	*Clostridiales*	*Clostridiaceae*	*Clostridium*	*Clostridium*
5432	*Firmicutes*	*Clostridia*	*Clostridiales*	*Clostridiaceae*	*Clostridium*	*Clostridium*

Table S4. Isolated strains by culturomics

bacteriaId	*phylum*	*class*	*order*	*family*	*genus*	*species*
5433	*Firmicutes*	*Clostridia*	*Clostridiales*	*Clostridiaceae*	*Clostridium*	*Clostridium*
5434	*Firmicutes*	*Clostridia*	*Clostridiales*	*Clostridiaceae*	*Clostridium*	*Clostridium*
5435	*Firmicutes*	*Clostridia*	*Clostridiales*	*Clostridiaceae*	*Clostridium*	*Clostridium*
5436	*Firmicutes*	*Clostridia*	*Clostridiales*	*Clostridiaceae*	*Clostridium*	*Clostridium*
5437	*Firmicutes*	*Clostridia*	*Clostridiales*	*Clostridiaceae*	*Clostridium*	*Clostridium*
5438	*Firmicutes*	*Clostridia*	*Clostridiales*	*Clostridiaceae*	*Clostridium*	*Clostridium*
5439	*Firmicutes*	*Clostridia*	*Clostridiales*	*Clostridiaceae*	*Clostridium*	*Clostridium*
5440	*Firmicutes*	*Clostridia*	*Clostridiales*	*Clostridiaceae*	*Clostridium*	*Clostridium*
5441	*Firmicutes*	*Clostridia*	*Clostridiales*	*Clostridiaceae*	*Clostridium*	*Clostridium*
5442	*Firmicutes*	*Clostridia*	*Clostridiales*	*Clostridiaceae*	*Clostridium*	*Clostridium*
5443	*Firmicutes*	*Clostridia*	*Clostridiales*	*Clostridiaceae*	*Clostridium*	*Clostridium*
5444	*Firmicutes*	*Clostridia*	*Clostridiales*	*Clostridiaceae*	*Clostridium*	*Clostridium*
5445	*Firmicutes*	*Clostridia*	*Clostridiales*	*Clostridiaceae*	*Clostridium*	*Clostridium*
5446	*Firmicutes*	*Clostridia*	*Clostridiales*	*Clostridiaceae*	*Clostridium*	*Clostridium*
5447	*Firmicutes*	*Clostridia*	*Clostridiales*	*Clostridiaceae*	*Clostridium*	*Clostridium*
5448	*Firmicutes*	*Clostridia*	*Clostridiales*	*Clostridiaceae*	*Clostridium*	*Clostridium*
5449	*Firmicutes*	*Clostridia*	*Clostridiales*	*Clostridiaceae*	*Clostridium*	*Clostridium*
5450	*Firmicutes*	*Clostridia*	*Clostridiales*	*Clostridiaceae*	*Clostridium*	*Clostridium*
5451	*Firmicutes*	*Clostridia*	*Clostridiales*	*Clostridiaceae*	*Clostridium*	*Clostridium*
5452	*Firmicutes*	*Clostridia*	*Clostridiales*	*Clostridiaceae*	*Clostridium*	*Clostridium*
5453	*Firmicutes*	*Clostridia*	*Clostridiales*	*Clostridiaceae*	*Clostridium*	*Clostridium*
5454	*Firmicutes*	*Clostridia*	*Clostridiales*	*Clostridiaceae*	*Clostridium*	*Clostridium*
5455	*Firmicutes*	*Clostridia*	*Clostridiales*	*Clostridiaceae*	*Clostridium*	*Clostridium*
5456	*Firmicutes*	*Clostridia*	*Clostridiales*	*Clostridiaceae*	*Clostridium*	*Clostridium*
5457	*Firmicutes*	*Clostridia*	*Clostridiales*	*Clostridiaceae*	*Clostridium*	*Clostridium*
5458	*Firmicutes*	*Clostridia*	*Clostridiales*	*Clostridiaceae*	*Clostridium*	*Clostridium*
5459	*Firmicutes*	*Clostridia*	*Clostridiales*	*Clostridiaceae*	*Clostridium*	*Clostridium*
5460	*Firmicutes*	*Clostridia*	*Clostridiales*	*Clostridiaceae*	*Clostridium*	*Clostridium*

Table S4. Isolated strains by culturomics

bacteriaId	*phylum*	*class*	*order*	*family*	*genus*	*species*
5461	*Firmicutes*	*Clostridia*	*Clostridiales*	*Clostridiaceae*	*Clostridium*	*Clostridium*
5462	*Firmicutes*	*Clostridia*	*Clostridiales*	*Clostridiaceae*	*Clostridium*	*Clostridium*
5463	*Firmicutes*	*Clostridia*	*Clostridiales*	*Clostridiaceae*	*Clostridium*	*Clostridium*
5464	*Firmicutes*	*Clostridia*	*Clostridiales*	*Clostridiaceae*	*Clostridium*	*Clostridium*
5465	*Firmicutes*	*Clostridia*	*Clostridiales*	*Clostridiaceae*	*Clostridium*	*Clostridium*
5466	*Firmicutes*	*Clostridia*	*Clostridiales*	*Clostridiaceae*	*Clostridium*	*Clostridium*
5467	*Firmicutes*	*Clostridia*	*Clostridiales*	*Clostridiaceae*	*Clostridium*	*Clostridium*
5468	*Firmicutes*	*Clostridia*	*Clostridiales*	*Clostridiaceae*	*Clostridium*	*Clostridium*
5469	*Firmicutes*	*Clostridia*	*Clostridiales*	*Clostridiaceae*	*Clostridium*	*Clostridium*
5470	*Firmicutes*	*Clostridia*	*Clostridiales*	*Clostridiaceae*	*Clostridium*	*Clostridium*
5471	*Firmicutes*	*Clostridia*	*Clostridiales*	*Clostridiaceae*	*Clostridium*	*Clostridium*
5472	*Firmicutes*	*Clostridia*	*Clostridiales*	*Clostridiaceae*	*Clostridium*	*Clostridium*
5473	*Firmicutes*	*Clostridia*	*Clostridiales*	*Clostridiaceae*	*Clostridium*	*Clostridium*
5474	*Firmicutes*	*Clostridia*	*Clostridiales*	*Clostridiaceae*	*Clostridium*	*Clostridium*
5475	*Firmicutes*	*Clostridia*	*Clostridiales*	*Clostridiaceae*	*Clostridium*	*Clostridium*
5476	*Firmicutes*	*Clostridia*	*Clostridiales*	*Clostridiaceae*	*Clostridium*	*Clostridium*
5477	*Firmicutes*	*Clostridia*	*Clostridiales*	*Clostridiaceae*	*Clostridium*	*Clostridium*
5478	*Firmicutes*	*Clostridia*	*Clostridiales*	*Clostridiaceae*	*Clostridium*	*Clostridium*
5479	*Firmicutes*	*Clostridia*	*Clostridiales*	*Clostridiaceae*	*Clostridium*	*Clostridium*
5480	*Firmicutes*	*Clostridia*	*Clostridiales*	*Clostridiaceae*	*Clostridium*	*Clostridium*
5481	*Firmicutes*	*Clostridia*	*Clostridiales*	*Clostridiaceae*	*Clostridium*	*Clostridium*
5482	*Firmicutes*	*Clostridia*	*Clostridiales*	*Clostridiaceae*	*Clostridium*	*Clostridium*
5483	*Firmicutes*	*Clostridia*	*Clostridiales*	*Clostridiaceae*	*Clostridium*	*Clostridium*
5484	*Firmicutes*	*Clostridia*	*Clostridiales*	*Clostridiaceae*	*Clostridium*	*Clostridium*
5485	*Firmicutes*	*Clostridia*	*Clostridiales*	*Clostridiaceae*	*Clostridium*	*Clostridium*
5486	*Firmicutes*	*Clostridia*	*Clostridiales*	*Clostridiaceae*	*Clostridium*	*Clostridium*
5487	*Firmicutes*	*Clostridia*	*Clostridiales*	*Clostridiaceae*	*Clostridium*	*Clostridium*
5488	*Firmicutes*	*Clostridia*	*Clostridiales*	*Clostridiaceae*	*Clostridium*	*Clostridium*

Table S4. Isolated strains by culturomics

bacterialId	*phylum*	*class*	*order*	*family*	*genus*	*species*
5489	*Firmicutes*	*Clostridia*	*Clostridiales*	*Clostridiaceae*	*Clostridium*	*Clostridium*
5490	*Firmicutes*	*Clostridia*	*Clostridiales*	*Clostridiaceae*	*Clostridium*	*Clostridium*
5491	*Firmicutes*	*Clostridia*	*Clostridiales*	*Clostridiaceae*	*Clostridium*	*Clostridium*
5492	*Firmicutes*	*Clostridia*	*Clostridiales*	*Clostridiaceae*	*Clostridium*	*Clostridium*
5493	*Firmicutes*	*Clostridia*	*Clostridiales*	*Clostridiaceae*	*Clostridium*	*Clostridium*
5494	*Firmicutes*	*Clostridia*	*Clostridiales*	*Clostridiaceae*	*Clostridium*	*Clostridium*
5495	*Firmicutes*	*Clostridia*	*Clostridiales*	*Clostridiaceae*	*Clostridium*	*Clostridium*
5496	*Firmicutes*	*Clostridia*	*Clostridiales*	*Clostridiaceae*	*Clostridium*	*Clostridium*
5497	*Firmicutes*	*Clostridia*	*Clostridiales*	*Clostridiaceae*	*Clostridium*	*Clostridium*
5498	*Firmicutes*	*Clostridia*	*Clostridiales*	*Clostridiaceae*	*Clostridium*	*Clostridium*
5499	*Firmicutes*	*Clostridia*	*Clostridiales*	*Clostridiaceae*	*Clostridium*	*Clostridium*
5500	*Firmicutes*	*Clostridia*	*Clostridiales*	*Clostridiaceae*	*Clostridium*	*Clostridium*
5501	*Firmicutes*	*Clostridia*	*Clostridiales*	*Clostridiaceae*	*Clostridium*	*Clostridium*
5502	*Firmicutes*	*Clostridia*	*Clostridiales*	*Clostridiaceae*	*Clostridium*	*Clostridium*
5503	*Firmicutes*	*Clostridia*	*Clostridiales*	*Clostridiaceae*	*Clostridium*	*Clostridium*
5504	*Firmicutes*	*Clostridia*	*Clostridiales*	*Clostridiaceae*	*Clostridium*	*Clostridium*
5505	*Firmicutes*	*Clostridia*	*Clostridiales*	*Clostridiaceae*	*Clostridium*	*Clostridium*
5506	*Firmicutes*	*Clostridia*	*Clostridiales*	*Clostridiaceae*	*Clostridium*	*Clostridium*
5507	*Firmicutes*	*Clostridia*	*Clostridiales*	*Clostridiaceae*	*Clostridium*	*Clostridium*
5508	*Firmicutes*	*Clostridia*	*Clostridiales*	*Clostridiaceae*	*Clostridium*	*Clostridium*
5509	*Firmicutes*	*Clostridia*	*Clostridiales*	*Clostridiaceae*	*Clostridium*	*Clostridium*
5510	*Firmicutes*	*Clostridia*	*Clostridiales*	*Clostridiaceae*	*Clostridium*	*Clostridium*
5511	*Firmicutes*	*Clostridia*	*Clostridiales*	*Clostridiaceae*	*Clostridium*	*Clostridium*
5512	*Firmicutes*	*Clostridia*	*Clostridiales*	*Clostridiaceae*	*Clostridium*	*Clostridium*
5513	*Firmicutes*	*Clostridia*	*Clostridiales*	*Clostridiaceae*	*Clostridium*	*Clostridium*
5514	*Firmicutes*	*Clostridia*	*Clostridiales*	*Clostridiaceae*	*Clostridium*	*Clostridium*
5515	*Firmicutes*	*Clostridia*	*Clostridiales*	*Clostridiaceae*	*Clostridium*	*Clostridium*
5516	*Firmicutes*	*Clostridia*	*Clostridiales*	*Clostridiaceae*	*Clostridium*	*Clostridium*

Table S4. Isolated strains by culturomics

bacteriaId	*phylum*	*class*	*order*	*family*	*genus*	*species*
5517	*Firmicutes*	*Clostridia*	*Clostridiales*	*Clostridiaceae*	*Clostridium*	*Clostridium*
5518	*Firmicutes*	*Clostridia*	*Clostridiales*	*Clostridiaceae*	*Clostridium*	*Clostridium*
5519	*Firmicutes*	*Clostridia*	*Clostridiales*	*Clostridiaceae*	*Clostridium*	*Clostridium*
5520	*Firmicutes*	*Clostridia*	*Clostridiales*	*Clostridiaceae*	*Clostridium*	*Clostridium*
5521	*Firmicutes*	*Clostridia*	*Clostridiales*	*Clostridiaceae*	*Clostridium*	*Clostridium*
5522	*Firmicutes*	*Clostridia*	*Clostridiales*	*Clostridiaceae*	*Clostridium*	*Clostridium*
5523	*Firmicutes*	*Clostridia*	*Clostridiales*	*Clostridiaceae*	*Clostridium*	*Clostridium*
5524	*Firmicutes*	*Clostridia*	*Clostridiales*	*Clostridiaceae*	*Clostridium*	*Clostridium*
5525	*Firmicutes*	*Clostridia*	*Clostridiales*	*Clostridiaceae*	*Clostridium*	*Clostridium*
5526	*Firmicutes*	*Clostridia*	*Clostridiales*	*Clostridiaceae*	*Clostridium*	*Clostridium*
5527	*Firmicutes*	*Clostridia*	*Clostridiales*	*Clostridiaceae*	*Clostridium*	*Clostridium*
5528	*Firmicutes*	*Clostridia*	*Clostridiales*	*Clostridiaceae*	*Clostridium*	*Clostridium*
5529	*Firmicutes*	*Clostridia*	*Clostridiales*	*Clostridiaceae*	*Clostridium*	*Clostridium*
5530	*Firmicutes*	*Clostridia*	*Clostridiales*	*Clostridiaceae*	*Clostridium*	*Clostridium*
5531	*Firmicutes*	*Clostridia*	*Clostridiales*	*Clostridiaceae*	*Clostridium*	*Clostridium*
5532	*Firmicutes*	*Clostridia*	*Clostridiales*	*Clostridiaceae*	*Clostridium*	*Clostridium*
5533	*Firmicutes*	*Clostridia*	*Clostridiales*	*Clostridiaceae*	*Clostridium*	*Clostridium*
5534	*Firmicutes*	*Clostridia*	*Clostridiales*	*Clostridiaceae*	*Clostridium*	*Clostridium*
5535	*Firmicutes*	*Clostridia*	*Clostridiales*	*Clostridiaceae*	*Clostridium*	*Clostridium*
5536	*Firmicutes*	*Clostridia*	*Clostridiales*	*Clostridiaceae*	*Clostridium*	*Clostridium*
5537	*Firmicutes*	*Clostridia*	*Clostridiales*	*Clostridiaceae*	*Clostridium*	*Clostridium*
5538	*Firmicutes*	*Clostridia*	*Clostridiales*	*Clostridiaceae*	*Clostridium*	*Clostridium*
5539	*Firmicutes*	*Clostridia*	*Clostridiales*	*Clostridiaceae*	*Clostridium*	*Clostridium*
5540	*Firmicutes*	*Clostridia*	*Clostridiales*	*Clostridiaceae*	*Clostridium*	*Clostridium*
5541	*Firmicutes*	*Clostridia*	*Clostridiales*	*Clostridiaceae*	*Clostridium*	*Clostridium*
5542	*Firmicutes*	*Clostridia*	*Clostridiales*	*Clostridiaceae*	*Clostridium*	*Clostridium*
5543	*Firmicutes*	*Clostridia*	*Clostridiales*	*Clostridiaceae*	*Clostridium*	*Clostridium*
5544	*Firmicutes*	*Clostridia*	*Clostridiales*	*Clostridiaceae*	*Clostridium*	*Clostridium*

Table S4. Isolated strains by culturomics

bacteriaId	*phylum*	*class*	*order*	*family*	*genus*	*species*
5545	*Firmicutes*	*Clostridia*	*Clostridiales*	*Clostridiaceae*	*Clostridium*	*Clostridium*
5546	*Firmicutes*	*Clostridia*	*Clostridiales*	*Clostridiaceae*	*Clostridium*	*Clostridium*
5547	*Firmicutes*	*Clostridia*	*Clostridiales*	*Clostridiaceae*	*Clostridium*	*Clostridium*
5548	*Firmicutes*	*Clostridia*	*Clostridiales*	*Clostridiaceae*	*Clostridium*	*Clostridium*
5549	*Firmicutes*	*Clostridia*	*Clostridiales*	*Clostridiaceae*	*Clostridium*	*Clostridium*
5550	*Firmicutes*	*Clostridia*	*Clostridiales*	*Clostridiaceae*	*Clostridium*	*Clostridium*
5551	*Firmicutes*	*Clostridia*	*Clostridiales*	*Clostridiaceae*	*Clostridium*	*Clostridium*
5552	*Firmicutes*	*Clostridia*	*Clostridiales*	*Clostridiaceae*	*Clostridium*	*Clostridium*
5553	*Firmicutes*	*Clostridia*	*Clostridiales*	*Clostridiaceae*	*Clostridium*	*Clostridium*
5554	*Firmicutes*	*Clostridia*	*Clostridiales*	*Clostridiaceae*	*Clostridium*	*Clostridium*
5555	*Firmicutes*	*Clostridia*	*Clostridiales*	*Clostridiaceae*	*Clostridium*	*Clostridium*
5556	*Firmicutes*	*Clostridia*	*Clostridiales*	*Clostridiaceae*	*Clostridium*	*Clostridium*
5557	*Firmicutes*	*Clostridia*	*Clostridiales*	*Clostridiaceae*	*Clostridium*	*Clostridium*
5558	*Firmicutes*	*Clostridia*	*Clostridiales*	*Clostridiaceae*	*Clostridium*	*Clostridium*
5559	*Firmicutes*	*Clostridia*	*Clostridiales*	*Clostridiaceae*	*Clostridium*	*Clostridium*
5560	*Firmicutes*	*Clostridia*	*Clostridiales*	*Clostridiaceae*	*Clostridium*	*Clostridium*
5561	*Firmicutes*	*Clostridia*	*Clostridiales*	*Clostridiaceae*	*Clostridium*	*Clostridium*
5562	*Firmicutes*	*Clostridia*	*Clostridiales*	*Clostridiaceae*	*Clostridium*	*Clostridium*
5563	*Firmicutes*	*Clostridia*	*Clostridiales*	*Clostridiaceae*	*Clostridium*	*Clostridium*
5564	*Firmicutes*	*Clostridia*	*Clostridiales*	*Clostridiaceae*	*Clostridium*	*Clostridium*
5565	*Firmicutes*	*Clostridia*	*Clostridiales*	*Clostridiaceae*	*Clostridium*	*Clostridium*
5566	*Firmicutes*	*Clostridia*	*Clostridiales*	*Clostridiaceae*	*Clostridium*	*Clostridium*
5567	*Firmicutes*	*Clostridia*	*Clostridiales*	*Clostridiaceae*	*Clostridium*	*Clostridium*
5568	*Firmicutes*	*Clostridia*	*Clostridiales*	*Clostridiaceae*	*Clostridium*	*Clostridium*
5569	*Firmicutes*	*Clostridia*	*Clostridiales*	*Clostridiaceae*	*Clostridium*	*Clostridium*
5570	*Firmicutes*	*Clostridia*	*Clostridiales*	*Clostridiaceae*	*Clostridium*	*Clostridium*
5571	*Firmicutes*	*Clostridia*	*Clostridiales*	*Clostridiaceae*	*Clostridium*	*Clostridium*
5572	*Firmicutes*	*Clostridia*	*Clostridiales*	*Clostridiaceae*	*Clostridium*	*Clostridium*

Table S4. Isolated strains by culturomics

bacteriaId	*phylum*	*class*	*order*	*family*	*genus*	*species*
5573	*Firmicutes*	*Clostridia*	*Clostridiales*	*Clostridiaceae*	*Clostridium*	*Clostridium*
5574	*Firmicutes*	*Clostridia*	*Clostridiales*	*Clostridiaceae*	*Clostridium*	*Clostridium*
5575	*Firmicutes*	*Clostridia*	*Clostridiales*	*Clostridiaceae*	*Clostridium*	*Clostridium*
5576	*Firmicutes*	*Clostridia*	*Clostridiales*	*Clostridiaceae*	*Clostridium*	*Clostridium*
5577	*Firmicutes*	*Clostridia*	*Clostridiales*	*Clostridiaceae*	*Clostridium*	*Clostridium*
5578	*Firmicutes*	*Clostridia*	*Clostridiales*	*Clostridiaceae*	*Clostridium*	*Clostridium*
5579	*Firmicutes*	*Clostridia*	*Clostridiales*	*Clostridiaceae*	*Clostridium*	*Clostridium*
5580	*Firmicutes*	*Clostridia*	*Clostridiales*	*Clostridiaceae*	*Clostridium*	*Clostridium*
5581	*Firmicutes*	*Clostridia*	*Clostridiales*	*Clostridiaceae*	*Clostridium*	*Clostridium*
5582	*Firmicutes*	*Clostridia*	*Clostridiales*	*Clostridiaceae*	*Clostridium*	*Clostridium*
5583	*Firmicutes*	*Clostridia*	*Clostridiales*	*Clostridiaceae*	*Clostridium*	*Clostridium*
5584	*Firmicutes*	*Clostridia*	*Clostridiales*	*Clostridiaceae*	*Clostridium*	*Clostridium*
5585	*Firmicutes*	*Clostridia*	*Clostridiales*	*Clostridiaceae*	*Clostridium*	*Clostridium*
5586	*Firmicutes*	*Clostridia*	*Clostridiales*	*Clostridiaceae*	*Clostridium*	*Clostridium*
5587	*Firmicutes*	*Clostridia*	*Clostridiales*	*Clostridiaceae*	*Clostridium*	*Clostridium*
5588	*Firmicutes*	*Clostridia*	*Clostridiales*	*Clostridiaceae*	*Clostridium*	*Clostridium*
5589	*Firmicutes*	*Clostridia*	*Clostridiales*	*Clostridiaceae*	*Clostridium*	*Clostridium*
5590	*Firmicutes*	*Clostridia*	*Clostridiales*	*Clostridiaceae*	*Clostridium*	*Clostridium*
5591	*Firmicutes*	*Clostridia*	*Clostridiales*	*Clostridiaceae*	*Clostridium*	*Clostridium*
5592	*Firmicutes*	*Clostridia*	*Clostridiales*	*Clostridiaceae*	*Clostridium*	*Clostridium*
5593	*Firmicutes*	*Clostridia*	*Clostridiales*	*Clostridiaceae*	*Clostridium*	*Clostridium*
5594	*Firmicutes*	*Clostridia*	*Clostridiales*	*Clostridiaceae*	*Clostridium*	*Clostridium*
5595	*Firmicutes*	*Clostridia*	*Clostridiales*	*Clostridiaceae*	*Clostridium*	*Clostridium*
5596	*Firmicutes*	*Clostridia*	*Clostridiales*	*Clostridiaceae*	*Clostridium*	*Clostridium*
5597	*Firmicutes*	*Clostridia*	*Clostridiales*	*Clostridiaceae*	*Clostridium*	*Clostridium*
5598	*Firmicutes*	*Clostridia*	*Clostridiales*	*Clostridiaceae*	*Clostridium*	*Clostridium*
5599	*Firmicutes*	*Clostridia*	*Clostridiales*	*Clostridiaceae*	*Clostridium*	*Clostridium*
5600	*Firmicutes*	*Clostridia*	*Clostridiales*	*Clostridiaceae*	*Clostridium*	*Clostridium*

Table S4. Isolated strains by culturomics

bacteriaId	*phylum*	*class*	*order*	*family*	*genus*	*species*
5601	*Firmicutes*	*Clostridia*	*Clostridiales*	*Clostridiaceae*	*Clostridium*	*Clostridium*
5602	*Firmicutes*	*Clostridia*	*Clostridiales*	*Clostridiaceae*	*Clostridium*	*Clostridium*
5603	*Firmicutes*	*Clostridia*	*Clostridiales*	*Clostridiaceae*	*Clostridium*	*Clostridium*
5604	*Firmicutes*	*Clostridia*	*Clostridiales*	*Clostridiaceae*	*Clostridium*	*Clostridium*
5605	*Firmicutes*	*Clostridia*	*Clostridiales*	*Clostridiaceae*	*Clostridium*	*Clostridium*
5606	*Firmicutes*	*Clostridia*	*Clostridiales*	*Clostridiaceae*	*Clostridium*	*Clostridium*
5607	*Firmicutes*	*Clostridia*	*Clostridiales*	*Clostridiaceae*	*Clostridium*	*Clostridium*
5608	*Firmicutes*	*Clostridia*	*Clostridiales*	*Clostridiaceae*	*Clostridium*	*Clostridium*
5609	*Firmicutes*	*Clostridia*	*Clostridiales*	*Clostridiaceae*	*Clostridium*	*Clostridium*
5610	*Firmicutes*	*Clostridia*	*Clostridiales*	*Clostridiaceae*	*Clostridium*	*Clostridium*
5611	*Firmicutes*	*Clostridia*	*Clostridiales*	*Clostridiaceae*	*Clostridium*	*Clostridium*
5612	*Firmicutes*	*Clostridia*	*Clostridiales*	*Clostridiaceae*	*Clostridium*	*Clostridium*
5613	*Firmicutes*	*Clostridia*	*Clostridiales*	*Clostridiaceae*	*Clostridium*	*Clostridium*
5614	*Firmicutes*	*Clostridia*	*Clostridiales*	*Clostridiaceae*	*Clostridium*	*Clostridium*
5615	*Firmicutes*	*Clostridia*	*Clostridiales*	*Clostridiaceae*	*Clostridium*	*Clostridium*
5616	*Firmicutes*	*Clostridia*	*Clostridiales*	*Clostridiaceae*	*Clostridium*	*Clostridium*
5617	*Firmicutes*	*Clostridia*	*Clostridiales*	*Clostridiaceae*	*Clostridium*	*Clostridium*
5618	*Firmicutes*	*Clostridia*	*Clostridiales*	*Clostridiaceae*	*Clostridium*	*Clostridium*
5619	*Firmicutes*	*Clostridia*	*Clostridiales*	*Clostridiaceae*	*Clostridium*	*Clostridium*
5620	*Firmicutes*	*Clostridia*	*Clostridiales*	*Clostridiaceae*	*Clostridium*	*Clostridium*
5621	*Firmicutes*	*Clostridia*	*Clostridiales*	*Clostridiaceae*	*Clostridium*	*Clostridium*
5622	*Firmicutes*	*Clostridia*	*Clostridiales*	*Clostridiaceae*	*Clostridium*	*Clostridium*
5623	*Firmicutes*	*Clostridia*	*Clostridiales*	*Clostridiaceae*	*Clostridium*	*Clostridium*
5624	*Firmicutes*	*Clostridia*	*Clostridiales*	*Clostridiaceae*	*Clostridium*	*Clostridium*
5625	*Firmicutes*	*Clostridia*	*Clostridiales*	*Clostridiaceae*	*Clostridium*	*Clostridium*
5626	*Firmicutes*	*Clostridia*	*Clostridiales*	*Clostridiaceae*	*Clostridium*	*Clostridium*
5627	*Firmicutes*	*Clostridia*	*Clostridiales*	*Clostridiaceae*	*Clostridium*	*Clostridium*
5628	*Firmicutes*	*Clostridia*	*Clostridiales*	*Clostridiaceae*	*Clostridium*	*Clostridium*

Table S4. Isolated strains by culturomics

bacteriaId	*phylum*	*class*	*order*	*family*	*genus*	*species*
5629	*Firmicutes*	*Clostridia*	*Clostridiales*	*Clostridiaceae*	*Clostridium*	*Clostridium*
5630	*Firmicutes*	*Clostridia*	*Clostridiales*	*Clostridiaceae*	*Clostridium*	*Clostridium*
5631	*Firmicutes*	*Clostridia*	*Clostridiales*	*Clostridiaceae*	*Clostridium*	*Clostridium*
5632	*Firmicutes*	*Clostridia*	*Clostridiales*	*Clostridiaceae*	*Clostridium*	*Clostridium*
5633	*Firmicutes*	*Clostridia*	*Clostridiales*	*Clostridiaceae*	*Clostridium*	*Clostridium*
5634	*Firmicutes*	*Clostridia*	*Clostridiales*	*Clostridiaceae*	*Clostridium*	*Clostridium*
5635	*Firmicutes*	*Clostridia*	*Clostridiales*	*Clostridiaceae*	*Clostridium*	*Clostridium*
5636	*Firmicutes*	*Clostridia*	*Clostridiales*	*Clostridiaceae*	*Clostridium*	*Clostridium*
5637	*Firmicutes*	*Clostridia*	*Clostridiales*	*Clostridiaceae*	*Clostridium*	*Clostridium*
5638	*Firmicutes*	*Clostridia*	*Clostridiales*	*Clostridiaceae*	*Clostridium*	*Clostridium*
5639	*Firmicutes*	*Clostridia*	*Clostridiales*	*Clostridiaceae*	*Clostridium*	*Clostridium*
5640	*Firmicutes*	*Clostridia*	*Clostridiales*	*Clostridiaceae*	*Clostridium*	*Clostridium*
5641	*Firmicutes*	*Clostridia*	*Clostridiales*	*Clostridiaceae*	*Clostridium*	*Clostridium*
5642	*Firmicutes*	*Clostridia*	*Clostridiales*	*Clostridiaceae*	*Clostridium*	*Clostridium*
5643	*Firmicutes*	*Clostridia*	*Clostridiales*	*Clostridiaceae*	*Clostridium*	*Clostridium*
5644	*Firmicutes*	*Clostridia*	*Clostridiales*	*Clostridiaceae*	*Clostridium*	*Clostridium*
5645	*Firmicutes*	*Clostridia*	*Clostridiales*	*Clostridiaceae*	*Clostridium*	*Clostridium*
5646	*Firmicutes*	*Clostridia*	*Clostridiales*	*Clostridiaceae*	*Clostridium*	*Clostridium*
5647	*Firmicutes*	*Clostridia*	*Clostridiales*	*Clostridiaceae*	*Clostridium*	*Clostridium*
5648	*Firmicutes*	*Clostridia*	*Clostridiales*	*Clostridiaceae*	*Clostridium*	*Clostridium*
5649	*Firmicutes*	*Clostridia*	*Clostridiales*	*Clostridiaceae*	*Clostridium*	*Clostridium*
5650	*Firmicutes*	*Clostridia*	*Clostridiales*	*Clostridiaceae*	*Clostridium*	*Clostridium*
5651	*Firmicutes*	*Clostridia*	*Clostridiales*	*Clostridiaceae*	*Clostridium*	*Clostridium*
5652	*Firmicutes*	*Clostridia*	*Clostridiales*	*Clostridiaceae*	*Clostridium*	*Clostridium*
5653	*Firmicutes*	*Clostridia*	*Clostridiales*	*Clostridiaceae*	*Clostridium*	*Clostridium*
5654	*Firmicutes*	*Clostridia*	*Clostridiales*	*Clostridiaceae*	*Clostridium*	*Clostridium*
5655	*Firmicutes*	*Clostridia*	*Clostridiales*	*Clostridiaceae*	*Clostridium*	*Clostridium*
5656	*Firmicutes*	*Clostridia*	*Clostridiales*	*Clostridiaceae*	*Clostridium*	*Clostridium*

Table S4. Isolated strains by culturomics

bacteriaId	*phylum*	*class*	*order*	*family*	*genus*	*species*
5657	*Firmicutes*	*Clostridia*	*Clostridiales*	*Clostridiaceae*	*Clostridium*	*Clostridium*
5658	*Firmicutes*	*Clostridia*	*Clostridiales*	*Clostridiaceae*	*Clostridium*	*Clostridium*
5659	*Firmicutes*	*Clostridia*	*Clostridiales*	*Clostridiaceae*	*Clostridium*	*Clostridium*
5660	*Firmicutes*	*Clostridia*	*Clostridiales*	*Clostridiaceae*	*Clostridium*	*Clostridium*
5661	*Firmicutes*	*Clostridia*	*Clostridiales*	*Clostridiaceae*	*Clostridium*	*Clostridium*
5662	*Firmicutes*	*Clostridia*	*Clostridiales*	*Clostridiaceae*	*Clostridium*	*Clostridium*
5663	*Firmicutes*	*Clostridia*	*Clostridiales*	*Clostridiaceae*	*Clostridium*	*Clostridium*
5664	*Firmicutes*	*Clostridia*	*Clostridiales*	*Clostridiaceae*	*Clostridium*	*Clostridium*
5665	*Firmicutes*	*Clostridia*	*Clostridiales*	*Clostridiaceae*	*Clostridium*	*Clostridium*
5666	*Firmicutes*	*Clostridia*	*Clostridiales*	*Clostridiaceae*	*Clostridium*	*Clostridium*
5667	*Firmicutes*	*Clostridia*	*Clostridiales*	*Clostridiaceae*	*Clostridium*	*Clostridium*
5668	*Firmicutes*	*Clostridia*	*Clostridiales*	*Clostridiaceae*	*Clostridium*	*Clostridium*
5669	*Firmicutes*	*Clostridia*	*Clostridiales*	*Clostridiaceae*	*Clostridium*	*Clostridium*
5670	*Firmicutes*	*Clostridia*	*Clostridiales*	*Clostridiaceae*	*Clostridium*	*Clostridium*
5671	*Firmicutes*	*Clostridia*	*Clostridiales*	*Clostridiaceae*	*Clostridium*	*Clostridium*
5672	*Firmicutes*	*Clostridia*	*Clostridiales*	*Clostridiaceae*	*Clostridium*	*Clostridium*
5673	*Firmicutes*	*Clostridia*	*Clostridiales*	*Clostridiaceae*	*Clostridium*	*Clostridium*
5674	*Firmicutes*	*Clostridia*	*Clostridiales*	*Clostridiaceae*	*Clostridium*	*Clostridium*
5675	*Firmicutes*	*Clostridia*	*Clostridiales*	*Clostridiaceae*	*Clostridium*	*Clostridium*
5676	*Firmicutes*	*Clostridia*	*Clostridiales*	*Clostridiaceae*	*Clostridium*	*Clostridium*
5677	*Firmicutes*	*Clostridia*	*Clostridiales*	*Clostridiaceae*	*Clostridium*	*Clostridium*
5678	*Firmicutes*	*Clostridia*	*Clostridiales*	*Clostridiaceae*	*Clostridium*	*Clostridium*
5679	*Firmicutes*	*Clostridia*	*Clostridiales*	*Clostridiaceae*	*Clostridium*	*Clostridium*
5680	*Firmicutes*	*Clostridia*	*Clostridiales*	*Clostridiaceae*	*Clostridium*	*Clostridium*
5681	*Firmicutes*	*Clostridia*	*Clostridiales*	*Clostridiaceae*	*Clostridium*	*Clostridium*
5682	*Firmicutes*	*Clostridia*	*Clostridiales*	*Clostridiaceae*	*Clostridium*	*Clostridium*
5683	*Firmicutes*	*Clostridia*	*Clostridiales*	*Clostridiaceae*	*Clostridium*	*Clostridium*
5684	*Firmicutes*	*Clostridia*	*Clostridiales*	*Clostridiaceae*	*Clostridium*	*Clostridium*

Table S4. Isolated strains by culturomics

bacteriaId	*phylum*	*class*	*order*	*family*	*genus*	*species*
5685	*Firmicutes*	*Clostridia*	*Clostridiales*	*Clostridiaceae*	*Clostridium*	*Clostridium*
5686	*Firmicutes*	*Clostridia*	*Clostridiales*	*Clostridiaceae*	*Clostridium*	*Clostridium*
5687	*Firmicutes*	*Clostridia*	*Clostridiales*	*Clostridiaceae*	*Clostridium*	*Clostridium*
5688	*Firmicutes*	*Clostridia*	*Clostridiales*	*Clostridiaceae*	*Clostridium*	*Clostridium*
5689	*Firmicutes*	*Clostridia*	*Clostridiales*	*Clostridiaceae*	*Clostridium*	*Clostridium*
5690	*Firmicutes*	*Clostridia*	*Clostridiales*	*Clostridiaceae*	*Clostridium*	*Clostridium*
5691	*Firmicutes*	*Clostridia*	*Clostridiales*	*Clostridiaceae*	*Clostridium*	*Clostridium*
5692	*Firmicutes*	*Clostridia*	*Clostridiales*	*Clostridiaceae*	*Clostridium*	*Clostridium*
5693	*Firmicutes*	*Clostridia*	*Clostridiales*	*Clostridiaceae*	*Clostridium*	*Clostridium*
5694	*Firmicutes*	*Clostridia*	*Clostridiales*	*Clostridiaceae*	*Clostridium*	*Clostridium*
5695	*Firmicutes*	*Clostridia*	*Clostridiales*	*Clostridiaceae*	*Clostridium*	*Clostridium*
5696	*Firmicutes*	*Clostridia*	*Clostridiales*	*Clostridiaceae*	*Clostridium*	*Clostridium*
5697	*Firmicutes*	*Clostridia*	*Clostridiales*	*Clostridiaceae*	*Clostridium*	*Clostridium*
5698	*Firmicutes*	*Clostridia*	*Clostridiales*	*Clostridiaceae*	*Clostridium*	*Clostridium*
5699	*Firmicutes*	*Clostridia*	*Clostridiales*	*Clostridiaceae*	*Clostridium*	*Clostridium*
5700	*Firmicutes*	*Clostridia*	*Clostridiales*	*Clostridiaceae*	*Clostridium*	*Clostridium*
5701	*Firmicutes*	*Clostridia*	*Clostridiales*	*Clostridiaceae*	*Clostridium*	*Clostridium*
5702	*Firmicutes*	*Clostridia*	*Clostridiales*	*Clostridiaceae*	*Clostridium*	*Clostridium*
5703	*Firmicutes*	*Clostridia*	*Clostridiales*	*Clostridiaceae*	*Clostridium*	*Clostridium*
5704	*Firmicutes*	*Clostridia*	*Clostridiales*	*Clostridiaceae*	*Clostridium*	*Clostridium*
5705	*Firmicutes*	*Clostridia*	*Clostridiales*	*Clostridiaceae*	*Clostridium*	*Clostridium*
5706	*Firmicutes*	*Clostridia*	*Clostridiales*	*Clostridiaceae*	*Clostridium*	*Clostridium*
5707	*Firmicutes*	*Clostridia*	*Clostridiales*	*Clostridiaceae*	*Clostridium*	*Clostridium*
5708	*Firmicutes*	*Clostridia*	*Clostridiales*	*Clostridiaceae*	*Clostridium*	*Clostridium*
5709	*Firmicutes*	*Clostridia*	*Clostridiales*	*Clostridiaceae*	*Clostridium*	*Clostridium*
5710	*Firmicutes*	*Clostridia*	*Clostridiales*	*Clostridiaceae*	*Clostridium*	*Clostridium*
5711	*Firmicutes*	*Clostridia*	*Clostridiales*	*Clostridiaceae*	*Clostridium*	*Clostridium*
5712	*Firmicutes*	*Clostridia*	*Clostridiales*	*Clostridiaceae*	*Clostridium*	*Clostridium*

Table S4. Isolated strains by culturomics

bacteriaId	*phylum*	*class*	*order*	*family*	*genus*	*species*
5713	*Firmicutes*	*Clostridia*	*Clostridiales*	*Clostridiaceae*	*Clostridium*	*Clostridium*
5714	*Firmicutes*	*Clostridia*	*Clostridiales*	*Clostridiaceae*	*Clostridium*	*Clostridium*
5715	*Firmicutes*	*Clostridia*	*Clostridiales*	*Clostridiaceae*	*Clostridium*	*Clostridium*
5716	*Firmicutes*	*Clostridia*	*Clostridiales*	*Clostridiaceae*	*Clostridium*	*Clostridium*
5717	*Firmicutes*	*Clostridia*	*Clostridiales*	*Clostridiaceae*	*Clostridium*	*Clostridium*
5718	*Firmicutes*	*Clostridia*	*Clostridiales*	*Clostridiaceae*	*Clostridium*	*Clostridium*
5719	*Firmicutes*	*Clostridia*	*Clostridiales*	*Clostridiaceae*	*Clostridium*	*Clostridium*
5720	*Firmicutes*	*Clostridia*	*Clostridiales*	*Clostridiaceae*	*Clostridium*	*Clostridium*
5721	*Firmicutes*	*Clostridia*	*Clostridiales*	*Clostridiaceae*	*Clostridium*	*Clostridium*
5722	*Firmicutes*	*Clostridia*	*Clostridiales*	*Clostridiaceae*	*Clostridium*	*Clostridium*
5723	*Firmicutes*	*Clostridia*	*Clostridiales*	*Clostridiaceae*	*Clostridium*	*Clostridium*
5724	*Firmicutes*	*Clostridia*	*Clostridiales*	*Clostridiaceae*	*Clostridium*	*Clostridium*
5725	*Firmicutes*	*Clostridia*	*Clostridiales*	*Clostridiaceae*	*Clostridium*	*Clostridium*
5726	*Firmicutes*	*Clostridia*	*Clostridiales*	*Clostridiaceae*	*Clostridium*	*Clostridium*
5727	*Firmicutes*	*Clostridia*	*Clostridiales*	*Clostridiaceae*	*Clostridium*	*Clostridium*
5728	*Firmicutes*	*Clostridia*	*Clostridiales*	*Clostridiaceae*	*Clostridium*	*Clostridium*
5729	*Firmicutes*	*Clostridia*	*Clostridiales*	*Clostridiaceae*	*Clostridium*	*Clostridium*
5730	*Firmicutes*	*Clostridia*	*Clostridiales*	*Clostridiaceae*	*Clostridium*	*Clostridium*
5731	*Firmicutes*	*Clostridia*	*Clostridiales*	*Clostridiaceae*	*Clostridium*	*Clostridium*
5732	*Firmicutes*	*Clostridia*	*Clostridiales*	*Clostridiaceae*	*Clostridium*	*Clostridium*
5733	*Firmicutes*	*Clostridia*	*Clostridiales*	*Clostridiaceae*	*Clostridium*	*Clostridium*
5734	*Firmicutes*	*Clostridia*	*Clostridiales*	*Clostridiaceae*	*Clostridium*	*Clostridium*
5735	*Firmicutes*	*Clostridia*	*Clostridiales*	*Clostridiaceae*	*Clostridium*	*Clostridium*
5736	*Firmicutes*	*Clostridia*	*Clostridiales*	*Clostridiaceae*	*Clostridium*	*Clostridium*
5737	*Firmicutes*	*Clostridia*	*Clostridiales*	*Clostridiaceae*	*Clostridium*	*Clostridium*
5738	*Firmicutes*	*Clostridia*	*Clostridiales*	*Clostridiaceae*	*Clostridium*	*Clostridium*
5739	*Firmicutes*	*Clostridia*	*Clostridiales*	*Clostridiaceae*	*Clostridium*	*Clostridium*
5740	*Firmicutes*	*Clostridia*	*Clostridiales*	*Clostridiaceae*	*Clostridium*	*Clostridium*

Table S4. Isolated strains by culturomics

bacteriaId	*phylum*	*class*	*order*	*family*	*genus*	*species*
5741	*Firmicutes*	*Clostridia*	*Clostridiales*	*Clostridiaceae*	*Clostridium*	*Clostridium*
5742	*Firmicutes*	*Clostridia*	*Clostridiales*	*Clostridiaceae*	*Clostridium*	*Clostridium*
5743	*Firmicutes*	*Clostridia*	*Clostridiales*	*Clostridiaceae*	*Clostridium*	*Clostridium*
5744	*Firmicutes*	*Clostridia*	*Clostridiales*	*Clostridiaceae*	*Clostridium*	*Clostridium*
5745	*Firmicutes*	*Clostridia*	*Clostridiales*	*Clostridiaceae*	*Clostridium*	*Clostridium*
5746	*Firmicutes*	*Clostridia*	*Clostridiales*	*Clostridiaceae*	*Clostridium*	*Clostridium*
5747	*Firmicutes*	*Clostridia*	*Clostridiales*	*Clostridiaceae*	*Clostridium*	*Clostridium*
5748	*Firmicutes*	*Clostridia*	*Clostridiales*	*Clostridiaceae*	*Clostridium*	*Clostridium*
5749	*Firmicutes*	*Clostridia*	*Clostridiales*	*Clostridiaceae*	*Clostridium*	*Clostridium*
5750	*Firmicutes*	*Clostridia*	*Clostridiales*	*Clostridiaceae*	*Clostridium*	*Clostridium*
5751	*Firmicutes*	*Clostridia*	*Clostridiales*	*Clostridiaceae*	*Clostridium*	*Clostridium*
5752	*Firmicutes*	*Clostridia*	*Clostridiales*	*Clostridiaceae*	*Clostridium*	*Clostridium*
5753	*Firmicutes*	*Clostridia*	*Clostridiales*	*Clostridiaceae*	*Clostridium*	*Clostridium*
5754	*Firmicutes*	*Clostridia*	*Clostridiales*	*Clostridiaceae*	*Clostridium*	*Clostridium*
5755	*Firmicutes*	*Clostridia*	*Clostridiales*	*Clostridiaceae*	*Clostridium*	*Clostridium*
5756	*Firmicutes*	*Clostridia*	*Clostridiales*	*Clostridiaceae*	*Clostridium*	*Clostridium*
5757	*Firmicutes*	*Clostridia*	*Clostridiales*	*Clostridiaceae*	*Clostridium*	*Clostridium*
5758	*Firmicutes*	*Clostridia*	*Clostridiales*	*Clostridiaceae*	*Clostridium*	*Clostridium*
5759	*Firmicutes*	*Clostridia*	*Clostridiales*	*Clostridiaceae*	*Clostridium*	*Clostridium*
5760	*Firmicutes*	*Clostridia*	*Clostridiales*	*Clostridiaceae*	*Clostridium*	*Clostridium*
5761	*Firmicutes*	*Clostridia*	*Clostridiales*	*Clostridiaceae*	*Clostridium*	*Clostridium*
5762	*Firmicutes*	*Clostridia*	*Clostridiales*	*Clostridiaceae*	*Clostridium*	*Clostridium*
5763	*Firmicutes*	*Clostridia*	*Clostridiales*	*Clostridiaceae*	*Clostridium*	*Clostridium*
5764	*Firmicutes*	*Clostridia*	*Clostridiales*	*Clostridiaceae*	*Clostridium*	*Clostridium*
5765	*Firmicutes*	*Clostridia*	*Clostridiales*	*Clostridiaceae*	*Clostridium*	*Clostridium*
5766	*Firmicutes*	*Clostridia*	*Clostridiales*	*Clostridiaceae*	*Clostridium*	*Clostridium*
5767	*Firmicutes*	*Clostridia*	*Clostridiales*	*Clostridiaceae*	*Clostridium*	*Clostridium*
5768	*Firmicutes*	*Clostridia*	*Clostridiales*	*Clostridiaceae*	*Clostridium*	*Clostridium*

Table S4. Isolated strains by culturomics

bacteriaId	*phylum*	*class*	*order*	*family*	*genus*	*species*
5769	*Firmicutes*	*Clostridia*	*Clostridiales*	*Clostridiaceae*	*Clostridium*	*Clostridium*
5770	*Firmicutes*	*Clostridia*	*Clostridiales*	*Clostridiaceae*	*Clostridium*	*Clostridium*
5771	*Firmicutes*	*Clostridia*	*Clostridiales*	*Clostridiaceae*	*Clostridium*	*Clostridium*
5772	*Firmicutes*	*Clostridia*	*Clostridiales*	*Clostridiaceae*	*Clostridium*	*Clostridium*
5773	*Firmicutes*	*Clostridia*	*Clostridiales*	*Clostridiaceae*	*Clostridium*	*Clostridium*
5774	*Firmicutes*	*Clostridia*	*Clostridiales*	*Clostridiaceae*	*Clostridium*	*Clostridium*
5775	*Firmicutes*	*Clostridia*	*Clostridiales*	*Clostridiaceae*	*Clostridium*	*Clostridium*
5776	*Firmicutes*	*Clostridia*	*Clostridiales*	*Clostridiaceae*	*Clostridium*	*Clostridium*
5777	*Firmicutes*	*Clostridia*	*Clostridiales*	*Clostridiaceae*	*Clostridium*	*Clostridium*
5778	*Firmicutes*	*Clostridia*	*Clostridiales*	*Clostridiaceae*	*Clostridium*	*Clostridium*
5779	*Firmicutes*	*Clostridia*	*Clostridiales*	*Clostridiaceae*	*Clostridium*	*Clostridium*
5780	*Firmicutes*	*Clostridia*	*Clostridiales*	*Clostridiaceae*	*Clostridium*	*Clostridium*
5781	*Firmicutes*	*Clostridia*	*Clostridiales*	*Clostridiaceae*	*Clostridium*	*Clostridium*
5782	*Firmicutes*	*Clostridia*	*Clostridiales*	*Clostridiaceae*	*Clostridium*	*Clostridium*
5783	*Firmicutes*	*Clostridia*	*Clostridiales*	*Clostridiaceae*	*Clostridium*	*Clostridium*
5784	*Firmicutes*	*Clostridia*	*Clostridiales*	*Clostridiaceae*	*Clostridium*	*Clostridium*
5785	*Firmicutes*	*Clostridia*	*Clostridiales*	*Clostridiaceae*	*Clostridium*	*Clostridium*
5786	*Firmicutes*	*Clostridia*	*Clostridiales*	*Clostridiaceae*	*Clostridium*	*Clostridium*
5787	*Firmicutes*	*Clostridia*	*Clostridiales*	*Clostridiaceae*	*Clostridium*	*Clostridium*
5788	*Firmicutes*	*Clostridia*	*Clostridiales*	*Clostridiaceae*	*Clostridium*	*Clostridium*
5789	*Firmicutes*	*Clostridia*	*Clostridiales*	*Clostridiaceae*	*Clostridium*	*Clostridium*
5790	*Firmicutes*	*Clostridia*	*Clostridiales*	*Clostridiaceae*	*Clostridium*	*Clostridium tertium*
5791	*Firmicutes*	*Clostridia*	*Clostridiales*	*Clostridiaceae*	*Clostridium*	*Clostridium tertium*
5792	*Firmicutes*	*Clostridia*	*Clostridiales*	*Clostridiaceae*	*Clostridium*	*Clostridium tertium*
5793	*Firmicutes*	*Clostridia*	*Clostridiales*	*Clostridiaceae*	*Clostridium*	*Clostridium tertium*
5794	*Firmicutes*	*Clostridia*	*Clostridiales*	*Clostridiaceae*	*Clostridium*	*Clostridium tertium*
5795	*Firmicutes*	*Clostridia*	*Clostridiales*	*Clostridiaceae*	*Clostridium*	*Clostridium tertium*
5796	*Firmicutes*	*Clostridia*	*Clostridiales*	*Clostridiaceae*	*Clostridium*	*Clostridium tertium*

Table S4. Isolated strains by culturomics

bacteriaId	*phylum*	*class*	*order*	*family*	*genus*	*species*
5797	*Firmicutes*	*Clostridia*	*Clostridiales*	*Clostridiaceae*	*Clostridium*	*Clostridium tertium*
5798	*Firmicutes*	*Clostridia*	*Clostridiales*	*Clostridiaceae*	*Clostridium*	*Clostridium tertium*
5799	*Firmicutes*	*Clostridia*	*Clostridiales*	*Clostridiaceae*	*Clostridium*	*Clostridium tertium*
5800	*Firmicutes*	*Clostridia*	*Clostridiales*	*Clostridiaceae*	*Clostridium*	*Clostridium tertium*
5801	*Firmicutes*	*Clostridia*	*Clostridiales*	*Clostridiaceae*	*Clostridium*	*Clostridium tertium*
5802	*Firmicutes*	*Clostridia*	*Clostridiales*	*Clostridiaceae*	*Clostridium*	*Clostridium tertium*
5803	*Firmicutes*	*Clostridia*	*Clostridiales*	*Clostridiaceae*	*Clostridium*	*Clostridium tertium*
5804	*Firmicutes*	*Clostridia*	*Clostridiales*	*Clostridiaceae*	*Clostridium*	*Clostridium tertium*
5805	*Firmicutes*	*Clostridia*	*Clostridiales*	*Clostridiaceae*	*Clostridium*	*Clostridium tertium*
5806	*Firmicutes*	*Clostridia*	*Clostridiales*	*Clostridiaceae*	*Clostridium*	*Clostridium tertium*
5807	*Firmicutes*	*Clostridia*	*Clostridiales*	*Clostridiaceae*	*Clostridium*	*Clostridium tertium*
5808	*Firmicutes*	*Clostridia*	*Clostridiales*	*Clostridiaceae*	*Clostridium*	*Clostridium tertium*
5809	*Firmicutes*	*Clostridia*	*Clostridiales*	*Clostridiaceae*	*Clostridium*	*Clostridium tertium*
5810	*Firmicutes*	*Clostridia*	*Clostridiales*	*Clostridiaceae*	*Clostridium*	*Clostridium tertium*
5811	*Firmicutes*	*Clostridia*	*Clostridiales*	*Clostridiaceae*	*Clostridium*	*Clostridium tertium*
5812	*Firmicutes*	*Clostridia*	*Clostridiales*	*Clostridiaceae*	*Clostridium*	*Clostridium tertium*
5813	*Firmicutes*	*Clostridia*	*Clostridiales*	*Clostridiaceae*	*Clostridium*	*Clostridium tertium*
5814	*Firmicutes*	*Clostridia*	*Clostridiales*	*Clostridiaceae*	*Clostridium*	*Clostridium tertium*
5815	*Firmicutes*	*Clostridia*	*Clostridiales*	*Clostridiaceae*	*Clostridium*	*Clostridium tertium*
5816	*Firmicutes*	*Clostridia*	*Clostridiales*	*Clostridiaceae*	*Clostridium*	*Clostridium tertium*
5817	*Firmicutes*	*Clostridia*	*Clostridiales*	*Clostridiaceae*	*Clostridium*	*Clostridium tertium*
5818	*Firmicutes*	*Clostridia*	*Clostridiales*	*Clostridiaceae*	*Clostridium*	*Clostridium tertium*
5819	*Firmicutes*	*Clostridia*	*Clostridiales*	*Clostridiaceae*	*Clostridium*	*Clostridium tertium*
5820	*Firmicutes*	*Clostridia*	*Clostridiales*	*Clostridiaceae*	*Clostridium*	*Clostridium tertium*
5821	*Firmicutes*	*Clostridia*	*Clostridiales*	*Clostridiaceae*	*Clostridium*	*Clostridium tertium*
5822	*Firmicutes*	*Clostridia*	*Clostridiales*	*Clostridiaceae*	*Clostridium*	*Clostridium tertium*
5823	*Firmicutes*	*Clostridia*	*Clostridiales*	*Clostridiaceae*	*Clostridium*	*Clostridium tertium*
5824	*Firmicutes*	*Clostridia*	*Clostridiales*	*Clostridiaceae*	*Clostridium*	*Clostridium tertium*

Table S4. Isolated strains by culturomics

bacteriaId	*phylum*	*class*	*order*	*family*	*genus*	*species*
5825	*Firmicutes*	*Clostridia*	*Clostridiales*	*Clostridiaceae*	*Clostridium*	*Clostridium tertium*
5826	*Firmicutes*	*Clostridia*	*Clostridiales*	*Clostridiaceae*	*Clostridium*	*Clostridium tertium*
5827	*Firmicutes*	*Clostridia*	*Clostridiales*	*Clostridiaceae*	*Clostridium*	*Clostridium tertium*
5828	*Firmicutes*	*Clostridia*	*Clostridiales*	*Clostridiaceae*	*Clostridium*	*Clostridium tertium*
5829	*Firmicutes*	*Clostridia*	*Clostridiales*	*Clostridiaceae*	*Clostridium*	*Clostridium tertium*
5830	*Firmicutes*	*Clostridia*	*Clostridiales*	*Clostridiaceae*	*Clostridium*	*Clostridium tertium*
5831	*Firmicutes*	*Clostridia*	*Clostridiales*	*Clostridiaceae*	*Clostridium*	*Clostridium tertium*
5832	*Firmicutes*	*Clostridia*	*Clostridiales*	*Clostridiaceae*	*Clostridium*	*Clostridium tertium*
5833	*Firmicutes*	*Clostridia*	*Clostridiales*	*Clostridiaceae*	*Clostridium*	*Clostridium tertium*
5834	*Firmicutes*	*Clostridia*	*Clostridiales*	*Clostridiaceae*	*Clostridium*	*Clostridium tertium*
5835	*Firmicutes*	*Clostridia*	*Clostridiales*	*Clostridiaceae*	*Clostridium*	*Clostridium tertium*
5836	*Firmicutes*	*Clostridia*	*Clostridiales*	*Clostridiaceae*	*Clostridium*	*Clostridium tertium*
5837	*Firmicutes*	*Clostridia*	*Clostridiales*	*Clostridiaceae*	*Clostridium*	*Clostridium tertium*
5838	*Firmicutes*	*Clostridia*	*Clostridiales*	*Clostridiaceae*	*Clostridium*	*Clostridium tertium*
5839	*Firmicutes*	*Clostridia*	*Clostridiales*	*Clostridiaceae*	*Clostridium*	*Clostridium tertium*
5840	*Firmicutes*	*Clostridia*	*Clostridiales*	*Clostridiaceae*	*Clostridium*	*Clostridium tertium*
5841	*Firmicutes*	*Clostridia*	*Clostridiales*	*Clostridiaceae*	*Clostridium*	*Clostridium tertium*
5842	*Firmicutes*	*Clostridia*	*Clostridiales*	*Clostridiaceae*	*Clostridium*	*Clostridium tertium*
5843	*Firmicutes*	*Clostridia*	*Clostridiales*	*Clostridiaceae*	*Clostridium*	*Clostridium tertium*
5844	*Firmicutes*	*Clostridia*	*Clostridiales*	*Clostridiaceae*	*Clostridium*	*Clostridium tertium*
5845	*Firmicutes*	*Clostridia*	*Clostridiales*	*Clostridiaceae*	*Clostridium*	*Clostridium tertium*
5846	*Firmicutes*	*Clostridia*	*Clostridiales*	*Clostridiaceae*	*Clostridium*	*Clostridium tertium*
5847	*Firmicutes*	*Clostridia*	*Clostridiales*	*Clostridiaceae*	*Clostridium*	*Clostridium tertium*
5848	*Firmicutes*	*Clostridia*	*Clostridiales*	*Clostridiaceae*	*Clostridium*	*Clostridium tertium*
5849	*Firmicutes*	*Clostridia*	*Clostridiales*	*Clostridiaceae*	*Clostridium*	*Clostridium tertium*
5850	*Firmicutes*	*Clostridia*	*Clostridiales*	*Clostridiaceae*	*Clostridium*	*Clostridium tertium*
5851	*Firmicutes*	*Clostridia*	*Clostridiales*	*Clostridiaceae*	*Clostridium*	*Clostridium tertium*
5852	*Firmicutes*	*Clostridia*	*Clostridiales*	*Clostridiaceae*	*Clostridium*	*Clostridium tertium*

Table S4. Isolated strains by culturomics

bacteriaId	*phylum*	*class*	*order*	*family*	*genus*	*species*
5853	*Firmicutes*	*Clostridia*	*Clostridiales*	*Clostridiaceae*	*Clostridium*	*Clostridium tertium*
5854	*Firmicutes*	*Clostridia*	*Clostridiales*	*Clostridiaceae*	*Clostridium*	*Clostridium tertium*
5855	*Firmicutes*	*Clostridia*	*Clostridiales*	*Clostridiaceae*	*Clostridium*	*Clostridium tertium*
5856	*Firmicutes*	*Clostridia*	*Clostridiales*	*Clostridiaceae*	*Clostridium*	*Clostridium tertium*
5857	*Firmicutes*	*Clostridia*	*Clostridiales*	*Clostridiaceae*	*Clostridium*	*Clostridium tertium*
5858	*Firmicutes*	*Clostridia*	*Clostridiales*	*Clostridiaceae*	*Clostridium*	*Clostridium tertium*
5859	*Firmicutes*	*Clostridia*	*Clostridiales*	*Clostridiaceae*	*Clostridium*	*Clostridium tertium*
5860	*Firmicutes*	*Clostridia*	*Clostridiales*	*Clostridiaceae*	*Clostridium*	*Clostridium tertium*
5861	*Firmicutes*	*Clostridia*	*Clostridiales*	*Clostridiaceae*	*Clostridium*	*Clostridium tertium*
5862	*Firmicutes*	*Clostridia*	*Clostridiales*	*Clostridiaceae*	*Clostridium*	*Clostridium tertium*
5863	*Firmicutes*	*Clostridia*	*Clostridiales*	*Clostridiaceae*	*Clostridium*	*Clostridium tertium*
5864	*Firmicutes*	*Clostridia*	*Clostridiales*	*Clostridiaceae*	*Clostridium*	*Clostridium tertium*
5865	*Firmicutes*	*Clostridia*	*Clostridiales*	*Clostridiaceae*	*Clostridium*	*Clostridium tertium*
5866	*Firmicutes*	*Clostridia*	*Clostridiales*	*Clostridiaceae*	*Clostridium*	*Clostridium tertium*
5867	*Firmicutes*	*Clostridia*	*Clostridiales*	*Clostridiaceae*	*Clostridium*	*Clostridium tertium*
5868	*Firmicutes*	*Clostridia*	*Clostridiales*	*Clostridiaceae*	*Clostridium*	*Clostridium tertium*
5869	*Firmicutes*	*Clostridia*	*Clostridiales*	*Clostridiaceae*	*Clostridium*	*Clostridium tertium*
5870	*Firmicutes*	*Clostridia*	*Clostridiales*	*Clostridiaceae*	*Clostridium*	*Clostridium tertium*
5871	*Firmicutes*	*Clostridia*	*Clostridiales*	*Clostridiaceae*	*Clostridium*	*Clostridium tertium*
5872	*Firmicutes*	*Clostridia*	*Clostridiales*	*Clostridiaceae*	*Clostridium*	*Clostridium tertium*
5873	*Firmicutes*	*Clostridia*	*Clostridiales*	*Clostridiaceae*	*Clostridium*	*Clostridium tertium*
5874	*Firmicutes*	*Clostridia*	*Clostridiales*	*Clostridiaceae*	*Clostridium*	*Clostridium tertium*
5875	*Firmicutes*	*Clostridia*	*Clostridiales*	*Clostridiaceae*	*Clostridium*	*Clostridium tertium*
5876	*Firmicutes*	*Clostridia*	*Clostridiales*	*Clostridiaceae*	*Clostridium*	*Clostridium tertium*
5877	*Firmicutes*	*Clostridia*	*Clostridiales*	*Clostridiaceae*	*Clostridium*	*Clostridium tertium*
5878	*Firmicutes*	*Clostridia*	*Clostridiales*	*Clostridiaceae*	*Clostridium*	*Clostridium tertium*
5879	*Firmicutes*	*Clostridia*	*Clostridiales*	*Clostridiaceae*	*Clostridium*	*Clostridium tertium*
5880	*Firmicutes*	*Clostridia*	*Clostridiales*	*Clostridiaceae*	*Clostridium*	*Clostridium tertium*

Table S4. Isolated strains by culturomics

bacteriaId	*phylum*	*class*	*order*	*family*	*genus*	*species*
5881	*Firmicutes*	*Clostridia*	*Clostridiales*	*Clostridiaceae*	*Clostridium*	*Clostridium tertium*
5882	*Firmicutes*	*Clostridia*	*Clostridiales*	*Clostridiaceae*	*Clostridium*	*Clostridium tertium*
5883	*Firmicutes*	*Clostridia*	*Clostridiales*	*Clostridiaceae*	*Clostridium*	*Clostridium tertium*
5884	*Firmicutes*	*Clostridia*	*Clostridiales*	*Clostridiaceae*	*Clostridium*	*Clostridium tertium*
5885	*Firmicutes*	*Clostridia*	*Clostridiales*	*Clostridiaceae*	*Clostridium*	*Clostridium tertium*
5886	*Firmicutes*	*Clostridia*	*Clostridiales*	*Clostridiaceae*	*Clostridium*	*Clostridium tertium*
5887	*Firmicutes*	*Clostridia*	*Clostridiales*	*Clostridiaceae*	*Clostridium*	*Clostridium tertium*
5888	*Firmicutes*	*Clostridia*	*Clostridiales*	*Clostridiaceae*	*Clostridium*	*Clostridium tertium*
5889	*Firmicutes*	*Clostridia*	*Clostridiales*	*Clostridiaceae*	*Clostridium*	*Clostridium tertium*
5890	*Firmicutes*	*Clostridia*	*Clostridiales*	*Clostridiaceae*	*Clostridium*	*Clostridium tertium*
5891	*Firmicutes*	*Clostridia*	*Clostridiales*	*Clostridiaceae*	*Clostridium*	*Clostridium tertium*
5892	*Firmicutes*	*Clostridia*	*Clostridiales*	*Clostridiaceae*	*Clostridium*	*Clostridium tertium*
5893	*Firmicutes*	*Clostridia*	*Clostridiales*	*Clostridiaceae*	*Clostridium*	*Clostridium tertium*
5894	*Firmicutes*	*Clostridia*	*Clostridiales*	*Clostridiaceae*	*Clostridium*	*Clostridium tertium*
5895	*Firmicutes*	*Clostridia*	*Clostridiales*	*Clostridiaceae*	*Clostridium*	*Clostridium tertium*
5896	*Firmicutes*	*Clostridia*	*Clostridiales*	*Clostridiaceae*	*Clostridium*	*Clostridium tertium*
5897	*Firmicutes*	*Clostridia*	*Clostridiales*	*Clostridiaceae*	*Clostridium*	*Clostridium tertium*
5898	*Firmicutes*	*Clostridia*	*Clostridiales*	*Clostridiaceae*	*Clostridium*	*Clostridium tertium*
5899	*Firmicutes*	*Clostridia*	*Clostridiales*	*Clostridiaceae*	*Clostridium*	*Clostridium tertium*
5900	*Firmicutes*	*Clostridia*	*Clostridiales*	*Clostridiaceae*	*Clostridium*	*Clostridium tertium*
5901	*Firmicutes*	*Clostridia*	*Clostridiales*	*Clostridiaceae*	*Clostridium*	*Clostridium tertium*
5902	*Firmicutes*	*Clostridia*	*Clostridiales*	*Clostridiaceae*	*Clostridium*	*Clostridium tertium*
5903	*Firmicutes*	*Clostridia*	*Clostridiales*	*Clostridiaceae*	*Clostridium*	*Clostridium tertium*
5904	*Firmicutes*	*Clostridia*	*Clostridiales*	*Clostridiaceae*	*Clostridium*	*Clostridium tertium*
5905	*Firmicutes*	*Clostridia*	*Clostridiales*	*Clostridiaceae*	*Clostridium*	*Clostridium tertium*
5906	*Firmicutes*	*Clostridia*	*Clostridiales*	*Clostridiaceae*	*Clostridium*	*Clostridium tertium*
5907	*Firmicutes*	*Clostridia*	*Clostridiales*	*Clostridiaceae*	*Clostridium*	*Clostridium tertium*
5908	*Firmicutes*	*Clostridia*	*Clostridiales*	*Clostridiaceae*	*Clostridium*	*Clostridium tertium*

Table S4. Isolated strains by culturomics

bacteriaId	*phylum*	*class*	*order*	*family*	*genus*	*species*
5909	*Firmicutes*	*Clostridia*	*Clostridiales*	*Clostridiaceae*	*Clostridium*	*Clostridium tertium*
5910	*Firmicutes*	*Clostridia*	*Clostridiales*	*Clostridiaceae*	*Clostridium*	*Clostridium tertium*
5911	*Firmicutes*	*Clostridia*	*Clostridiales*	*Clostridiaceae*	*Clostridium*	*Clostridium tertium*
5912	*Firmicutes*	*Clostridia*	*Clostridiales*	*Clostridiaceae*	*Clostridium*	*Clostridium tertium*
5913	*Firmicutes*	*Clostridia*	*Clostridiales*	*Clostridiaceae*	*Clostridium*	*Clostridium tertium*
5914	*Firmicutes*	*Clostridia*	*Clostridiales*	*Clostridiaceae*	*Clostridium*	*Clostridium tertium*
5915	*Firmicutes*	*Clostridia*	*Clostridiales*	*Clostridiaceae*	*Clostridium*	*Clostridium tertium*
5916	*Firmicutes*	*Clostridia*	*Clostridiales*	*Clostridiaceae*	*Clostridium*	*Clostridium tertium*
5917	*Firmicutes*	*Clostridia*	*Clostridiales*	*Clostridiaceae*	*Clostridium*	*Clostridium tertium*
5918	*Firmicutes*	*Clostridia*	*Clostridiales*	*Clostridiaceae*	*Clostridium*	*Clostridium tertium*
5919	*Firmicutes*	*Clostridia*	*Clostridiales*	*Clostridiaceae*	*Clostridium*	*Clostridium tertium*
5920	*Firmicutes*	*Clostridia*	*Clostridiales*	*Clostridiaceae*	*Clostridium*	*Clostridium tertium*
5921	*Firmicutes*	*Clostridia*	*Clostridiales*	*Clostridiaceae*	*Clostridium*	*Clostridium tertium*
5922	*Firmicutes*	*Clostridia*	*Clostridiales*	*Clostridiaceae*	*Clostridium*	*Clostridium tertium*
5923	*Firmicutes*	*Clostridia*	*Clostridiales*	*Clostridiaceae*	*Clostridium*	*Clostridium tertium*
5924	*Firmicutes*	*Clostridia*	*Clostridiales*	*Clostridiaceae*	*Clostridium*	*Clostridium tertium*
5925	*Firmicutes*	*Clostridia*	*Clostridiales*	*Clostridiaceae*	*Clostridium*	*Clostridium tertium*
5926	*Firmicutes*	*Clostridia*	*Clostridiales*	*Clostridiaceae*	*Clostridium*	*Clostridium tertium*
5927	*Firmicutes*	*Clostridia*	*Clostridiales*	*Clostridiaceae*	*Clostridium*	*Clostridium tertium*
5928	*Firmicutes*	*Clostridia*	*Clostridiales*	*Clostridiaceae*	*Clostridium*	*Clostridium tertium*
5929	*Firmicutes*	*Clostridia*	*Clostridiales*	*Clostridiaceae*	*Clostridium*	*Clostridium tertium*
5930	*Firmicutes*	*Clostridia*	*Clostridiales*	*Clostridiaceae*	*Clostridium*	*Clostridium tertium*
5931	*Firmicutes*	*Clostridia*	*Clostridiales*	*Clostridiaceae*	*Clostridium*	*Clostridium tertium*
5932	*Firmicutes*	*Clostridia*	*Clostridiales*	*Clostridiaceae*	*Clostridium*	*Clostridium tertium*
5933	*Firmicutes*	*Clostridia*	*Clostridiales*	*Clostridiaceae*	*Clostridium*	*Clostridium tertium*
5934	*Firmicutes*	*Clostridia*	*Clostridiales*	*Clostridiaceae*	*Clostridium*	*Clostridium tertium*
5935	*Firmicutes*	*Clostridia*	*Clostridiales*	*Clostridiaceae*	*Clostridium*	*Clostridium tertium*
5936	*Firmicutes*	*Clostridia*	*Clostridiales*	*Clostridiaceae*	*Clostridium*	*Clostridium tertium*

Table S4. Isolated strains by culturomics

bacteriaId	*phylum*	*class*	*order*	*family*	*genus*	*species*
5937	*Firmicutes*	*Clostridia*	*Clostridiales*	*Clostridiaceae*	*Clostridium*	*Clostridium tertium*
5938	*Firmicutes*	*Clostridia*	*Clostridiales*	*Clostridiaceae*	*Clostridium*	*Clostridium tertium*
5939	*Firmicutes*	*Clostridia*	*Clostridiales*	*Clostridiaceae*	*Clostridium*	*Clostridium tertium*
5940	*Firmicutes*	*Clostridia*	*Clostridiales*	*Clostridiaceae*	*Clostridium*	*Clostridium tertium*
5941	*Firmicutes*	*Clostridia*	*Clostridiales*	*Clostridiaceae*	*Clostridium*	*Clostridium tertium*
5942	*Firmicutes*	*Clostridia*	*Clostridiales*	*Clostridiaceae*	*Clostridium*	*Clostridium tertium*
5943	*Firmicutes*	*Clostridia*	*Clostridiales*	*Clostridiaceae*	*Clostridium*	*Clostridium tertium*
5944	*Firmicutes*	*Clostridia*	*Clostridiales*	*Clostridiaceae*	*Clostridium*	*Clostridium tertium*
5945	*Firmicutes*	*Clostridia*	*Clostridiales*	*Clostridiaceae*	*Clostridium*	*Clostridium tertium*
5946	*Firmicutes*	*Clostridia*	*Clostridiales*	*Clostridiaceae*	*Clostridium*	*Clostridium tertium*
5947	*Firmicutes*	*Clostridia*	*Clostridiales*	*Clostridiaceae*	*Clostridium*	*Clostridium tertium*
5948	*Firmicutes*	*Clostridia*	*Clostridiales*	*Clostridiaceae*	*Clostridium*	*Clostridium tertium*
5949	*Firmicutes*	*Clostridia*	*Clostridiales*	*Clostridiaceae*	*Clostridium*	*Hungatella*
5950	*Firmicutes*	*Clostridia*	*Clostridiales*	*Eubacteriaceae*	*Eubacterium*	*Eubacterium*
5951	*Firmicutes*	*Clostridia*	*Clostridiales*	*Eubacteriaceae*	*Eubacterium*	*Eubacterium*
5952	*Firmicutes*	*Clostridia*	*Clostridiales*	*Eubacteriaceae*	*Eubacterium*	*Eubacterium*
5953	*Firmicutes*	*Clostridia*	*Clostridiales*	*Eubacteriaceae*	*Eubacterium*	*Eubacterium*
5954	*Firmicutes*	*Clostridia*	*Clostridiales*	*Eubacteriaceae*	*Eubacterium*	*Eubacterium*
5955	*Firmicutes*	*Clostridia*	*Clostridiales*	*Eubacteriaceae*	*Eubacterium*	*Eubacterium*
5956	*Firmicutes*	*Clostridia*	*Clostridiales*	*Eubacteriaceae*	*Eubacterium*	*Eubacterium*
5957	*Firmicutes*	*Clostridia*	*Clostridiales*	*Eubacteriaceae*	*Eubacterium*	*Eubacterium*
5958	*Firmicutes*	*Clostridia*	*Clostridiales*	*Eubacteriaceae*	*Eubacterium*	*Eubacterium*
5959	*Firmicutes*	*Clostridia*	*Clostridiales*	*Lachnospiraceae*	*Anaerostipes*	*Anaerostipes caccae*
5960	*Firmicutes*	*Clostridia*	*Clostridiales*	*Lachnospiraceae*	*Lachnoclostridium*	*Clostridium*
5961	*Firmicutes*	*Clostridia*	*Clostridiales*	*Lachnospiraceae*	*Lachnoclostridium*	*Clostridium*
5962	*Firmicutes*	*Clostridia*	*Clostridiales*	*Lachnospiraceae*	*Lachnoclostridium*	*Clostridium*
5963	*Firmicutes*	*Clostridia*	*Clostridiales*	*Lachnospiraceae*	*Lachnoclostridium*	*Clostridium*
5964	*Firmicutes*	*Clostridia*	*Clostridiales*	*Lachnospiraceae*	*Lachnoclostridium*	*Clostridium*

Table S4. Isolated strains by culturomics

bacterialId	*phylum*	*class*	*order*	*family*	*genus*	*species*
5965	*Firmicutes*	*Clostridia*	*Clostridiales*	*Lachnospiraceae*	*Lachnoclostridium*	*Clostridium*
5966	*Firmicutes*	*Clostridia*	*Clostridiales*	*Lachnospiraceae*	*Lachnoclostridium*	*Clostridium*
5967	*Firmicutes*	*Clostridia*	*Clostridiales*	*Lachnospiraceae*	*Lachnoclostridium*	*Clostridium*
5968	*Firmicutes*	*Clostridia*	*Clostridiales*	*Lachnospiraceae*	*Lachnoclostridium*	*Clostridium*
5969	*Firmicutes*	*Clostridia*	*Clostridiales*	*Lachnospiraceae*	*Lachnoclostridium*	*Clostridium*
5970	*Firmicutes*	*Clostridia*	*Clostridiales*	*Lachnospiraceae*	*Lachnoclostridium*	*Clostridium*
5971	*Firmicutes*	*Clostridia*	*Clostridiales*	*Lachnospiraceae*	*Lachnoclostridium*	*Clostridium*
5972	*Firmicutes*	*Clostridia*	*Clostridiales*	*Lachnospiraceae*	*Lachnoclostridium*	*Clostridium*
5973	*Firmicutes*	*Clostridia*	*Clostridiales*	*Lachnospiraceae*	*Lachnoclostridium*	*Clostridium*
5974	*Firmicutes*	*Clostridia*	*Clostridiales*	*Lachnospiraceae*	*Lachnoclostridium*	*Clostridium*
5975	*Firmicutes*	*Clostridia*	*Clostridiales*	*Lachnospiraceae*	*Lachnoclostridium*	*Clostridium*
5976	*Firmicutes*	*Clostridia*	*Clostridiales*	*Lachnospiraceae*	*Lachnoclostridium*	*Clostridium*
5977	*Firmicutes*	*Clostridia*	*Clostridiales*	*Peptostreptococcac*	*Paeniclostridium*	*Paeniclostridium*
5978	*Firmicutes*	*Clostridia*	*Clostridiales*	*Peptostreptococcac*	*Paeniclostridium*	*Paeniclostridium*
5979	*Firmicutes*	*Clostridia*	*Clostridiales*	*Peptostreptococcac*	*Peptostreptococcac*	*Peptostreptococcac*
5980	*Firmicutes*	*Clostridia*	*Clostridiales*	*Peptostreptococcac*	*Peptostreptococcac*	*Peptostreptococcac*
5981	*Firmicutes*	*Clostridia*	*Clostridiales*	*Peptostreptococcac*	*Peptostreptococcac*	*Peptostreptococcac*
5982	*Firmicutes*	*Clostridia*	*Clostridiales*	*Peptostreptococcac*	*Peptostreptococcac*	*Peptostreptococcac*
5983	*Firmicutes*	*Clostridia*	*Clostridiales*	*Peptostreptococcac*	*Peptostreptococcac*	*Peptostreptococcac*
5984	*Firmicutes*	*Clostridia*	*Clostridiales*	*Peptostreptococcac*	*Peptostreptococcac*	*Peptostreptococcac*
5985	*Firmicutes*	*Clostridia*	*Clostridiales*	*Peptostreptococcac*	*Terrisporobacter*	*Terrisporobacter*
5986	*Firmicutes*	*Clostridia*	*Clostridiales*	*Ruminococcaceae*	*Flavonifractor*	*Flavonifractor*
5987	*Firmicutes*	*Clostridia*	*Clostridiales*	*Lachnospiraceae*	*Lachnospiraceae*	*Lachnospiraceae*
5988	*Firmicutes*	*Clostridia*	*Clostridiales*	*Lachnospiraceae*	*Lachnospiraceae*	*Lachnospiraceae*
5989	*Firmicutes*	*Clostridia*	*Peptostreptococcale*	*Peptostreptococcac*	*Paraclostridium*	*Paraclostridium*
5990	*Firmicutes*	*Clostridia*	*Peptostreptococcale*	*Peptostreptococcac*	*Paraclostridium*	*Paraclostridium*
5991	*Firmicutes*	*Clostridia*	*Peptostreptococcale*	*Peptostreptococcac*	*Paraclostridium*	*Paraclostridium*
5992	*Firmicutes*	*Clostridia*	*Peptostreptococcale*	*Peptostreptococcac*	*Paraclostridium*	*Paraclostridium*

Table S4. Isolated strains by culturomics

bacteriaId	*phylum*	*class*	*order*	*family*	*genus*	*species*
5993	*Firmicutes*	*Erysipelotrichia*	*Erysipelotrichales*	*Erysipelotrichaceae*	*Dielma*	*Dielma fastidiosa*
5994	*Firmicutes*	*Erysipelotrichia*	*Erysipelotrichales*	*Erysipelotrichaceae*	*Erysipelatoclostridi*	*Clostridium*
5995	*Firmicutes*	*Erysipelotrichia*	*Erysipelotrichales*	*Erysipelotrichaceae*	*Erysipelatoclostridi*	*Clostridium*
5996	*Firmicutes*	*Erysipelotrichia*	*Erysipelotrichales*	*Erysipelotrichaceae*	*Erysipelatoclostridi*	*Clostridium*
5997	*Firmicutes*	*Erysipelotrichia*	*Erysipelotrichales*	*Erysipelotrichaceae*	*Erysipelatoclostridi*	*Clostridium*
5998	*Firmicutes*	*Erysipelotrichia*	*Erysipelotrichales*	*Erysipelotrichaceae*	*Erysipelatoclostridi*	*Clostridium*
5999	*Firmicutes*	*Erysipelotrichia*	*Erysipelotrichales*	*Erysipelotrichaceae*	*Erysipelatoclostridi*	*Clostridium*
6000	*Firmicutes*	*Erysipelotrichia*	*Erysipelotrichales*	*Erysipelotrichaceae*	*Erysipelatoclostridi*	*Clostridium*
6001	*Firmicutes*	*Erysipelotrichia*	*Erysipelotrichales*	*Erysipelotrichaceae*	*Erysipelatoclostridi*	*Clostridium*
6002	*Firmicutes*	*Erysipelotrichia*	*Erysipelotrichales*	*Erysipelotrichaceae*	*Erysipelatoclostridi*	*Clostridium*
6003	*Firmicutes*	*Erysipelotrichia*	*Erysipelotrichales*	*Erysipelotrichaceae*	*Erysipelatoclostridi*	*Clostridium*
6004	*Firmicutes*	*Erysipelotrichia*	*Erysipelotrichales*	*Erysipelotrichaceae*	*Erysipelatoclostridi*	*Clostridium*
6005	*Firmicutes*	*Erysipelotrichia*	*Erysipelotrichales*	*Erysipelotrichaceae*	*Erysipelatoclostridi*	*Clostridium*
6006	*Firmicutes*	*Erysipelotrichia*	*Erysipelotrichales*	*Erysipelotrichaceae*	*Erysipelatoclostridi*	*Clostridium*
6007	*Firmicutes*	*Erysipelotrichia*	*Erysipelotrichales*	*Erysipelotrichaceae*	*Erysipelatoclostridi*	*Erysipelatoclostridi*
6008	*Firmicutes*	*Negativicutes*	*Acidaminococcales*	*Acidaminococcacea*	*Acidaminococcus*	*Acidaminococcus*
6009	*Firmicutes*	*Negativicutes*	*Acidaminococcales*	*Acidaminococcacea*	*Acidaminococcus*	*Acidaminococcus*
6010	*Firmicutes*	*Negativicutes*	*Acidaminococcales*	*Acidaminococcacea*	*Acidaminococcus*	*Acidaminococcus*
6011	*Firmicutes*	*Negativicutes*	*Acidaminococcales*	*Acidaminococcacea*	*Acidaminococcus*	*Acidaminococcus*
6012	*Firmicutes*	*Negativicutes*	*Acidaminococcales*	*Acidaminococcacea*	*Acidaminococcus*	*Acidaminococcus*
6013	*Firmicutes*	*Negativicutes*	*Acidaminococcales*	*Acidaminococcacea*	*Acidaminococcus*	*Acidaminococcus*
6014	*Firmicutes*	*Negativicutes*	*Acidaminococcales*	*Acidaminococcacea*	*Acidaminococcus*	*Acidaminococcus*
6015	*Firmicutes*	*Negativicutes*	*Acidaminococcales*	*Acidaminococcacea*	*Acidaminococcus*	*Acidaminococcus*
6016	*Firmicutes*	*Negativicutes*	*Acidaminococcales*	*Acidaminococcacea*	*Acidaminococcus*	*Acidaminococcus*
6017	*Firmicutes*	*Negativicutes*	*Acidaminococcales*	*Acidaminococcacea*	*Acidaminococcus*	*Acidaminococcus*
6018	*Firmicutes*	*Negativicutes*	*Acidaminococcales*	*Acidaminococcacea*	*Acidaminococcus*	*Acidaminococcus*
6019	*Firmicutes*	*Negativicutes*	*Acidaminococcales*	*Acidaminococcacea*	*Acidaminococcus*	*Acidaminococcus*
6020	*Firmicutes*	*Negativicutes*	*Acidaminococcales*	*Acidaminococcacea*	*Acidaminococcus*	*Acidaminococcus*

Table S4. Isolated strains by culturomics

bacteriaId	*phylum*	*class*	*order*	*family*	*genus*	*species*
6021	*Firmicutes*	*Negativicutes*	*Acidaminococcales*	*Acidaminococcacea*	*Acidaminococcus*	*Acidaminococcus*
6022	*Firmicutes*	*Negativicutes*	*Acidaminococcales*	*Acidaminococcacea*	*Acidaminococcus*	*Acidaminococcus*
6023	*Firmicutes*	*Negativicutes*	*Acidaminococcales*	*Acidaminococcacea*	*Acidaminococcus*	*Acidaminococcus*
6024	*Firmicutes*	*Negativicutes*	*Acidaminococcales*	*Acidaminococcacea*	*Acidaminococcus*	*Acidaminococcus*
6025	*Firmicutes*	*Negativicutes*	*Acidaminococcales*	*Acidaminococcacea*	*Acidaminococcus*	*Acidaminococcus*
6026	*Firmicutes*	*Negativicutes*	*Acidaminococcales*	*Acidaminococcacea*	*Acidaminococcus*	*Acidaminococcus*
6027	*Firmicutes*	*Negativicutes*	*Acidaminococcales*	*Acidaminococcacea*	*Acidaminococcus*	*Acidaminococcus*
6028	*Firmicutes*	*Negativicutes*	*Acidaminococcales*	*Acidaminococcacea*	*Acidaminococcus*	*Acidaminococcus*
6029	*Firmicutes*	*Negativicutes*	*Acidaminococcales*	*Acidaminococcacea*	*Acidaminococcus*	*Acidaminococcus*
6030	*Firmicutes*	*Negativicutes*	*Acidaminococcales*	*Acidaminococcacea*	*Acidaminococcus*	*Acidaminococcus*
6031	*Firmicutes*	*Negativicutes*	*Acidaminococcales*	*Acidaminococcacea*	*Acidaminococcus*	*Acidaminococcus*
6032	*Firmicutes*	*Negativicutes*	*Acidaminococcales*	*Acidaminococcacea*	*Acidaminococcus*	*Acidaminococcus*
6033	*Firmicutes*	*Negativicutes*	*Acidaminococcales*	*Acidaminococcacea*	*Acidaminococcus*	*Acidaminococcus*
6034	*Firmicutes*	*Negativicutes*	*Acidaminococcales*	*Acidaminococcacea*	*Acidaminococcus*	*Acidaminococcus*
6035	*Firmicutes*	*Negativicutes*	*Acidaminococcales*	*Acidaminococcacea*	*Acidaminococcus*	*Acidaminococcus*
6036	*Firmicutes*	*Negativicutes*	*Acidaminococcales*	*Acidaminococcacea*	*Acidaminococcus*	*Acidaminococcus*
6037	*Firmicutes*	*Negativicutes*	*Acidaminococcales*	*Acidaminococcacea*	*Acidaminococcus*	*Acidaminococcus*
6038	*Firmicutes*	*Negativicutes*	*Acidaminococcales*	*Acidaminococcacea*	*Acidaminococcus*	*Acidaminococcus*
6039	*Firmicutes*	*Negativicutes*	*Acidaminococcales*	*Acidaminococcacea*	*Acidaminococcus*	*Acidaminococcus*
6040	*Firmicutes*	*Negativicutes*	*Acidaminococcales*	*Acidaminococcacea*	*Acidaminococcus*	*Acidaminococcus*
6041	*Firmicutes*	*Negativicutes*	*Acidaminococcales*	*Acidaminococcacea*	*Acidaminococcus*	*Acidaminococcus*
6042	*Firmicutes*	*Negativicutes*	*Acidaminococcales*	*Acidaminococcacea*	*Acidaminococcus*	*Acidaminococcus*
6043	*Firmicutes*	*Negativicutes*	*Acidaminococcales*	*Acidaminococcacea*	*Acidaminococcus*	*Acidaminococcus*
6044	*Firmicutes*	*Negativicutes*	*Selenomonadales*	*Selenomonadaceae*	*Megamonas*	*Megamonas sp.*
6045	*Firmicutes*	*Negativicutes*	*Selenomonadales*	*Selenomonadaceae*	*Mitsuokella*	*Mitsuokella*
6046	*Firmicutes*	*Negativicutes*	*Selenomonadales*	*Selenomonadaceae*	*Mitsuokella*	*Mitsuokella*
6047	*Firmicutes*	*Negativicutes*	*Selenomonadales*	*Selenomonadaceae*	*Mitsuokella*	*Mitsuokella*
6048	*Firmicutes*	*Negativicutes*	*Selenomonadales*	*Selenomonadaceae*	*Mitsuokella*	*Mitsuokella*

Table S4. Isolated strains by culturomics

bacteriaId	*phylum*	*class*	*order*	*family*	*genus*	*species*
6049	*Firmicutes*	*Negativicutes*	*Selenomonadales*	*Selenomonadaceae*	*Mitsuokella*	*Mitsuokella*
6050	*Firmicutes*	*Negativicutes*	*Selenomonadales*	*Selenomonadaceae*	*Mitsuokella*	*Mitsuokella*
6051	*Firmicutes*	*Negativicutes*	*Selenomonadales*	*Selenomonadaceae*	*Mitsuokella*	*Mitsuokella*
6052	*Firmicutes*	*Negativicutes*	*Selenomonadales*	*Selenomonadaceae*	*Mitsuokella*	*Mitsuokella*
6053	*Firmicutes*	*Negativicutes*	*Selenomonadales*	*Selenomonadaceae*	*Mitsuokella*	*Mitsuokella*
6054	*Firmicutes*	*Negativicutes*	*Selenomonadales*	*Selenomonadaceae*	*Mitsuokella*	*Mitsuokella*
6055	*Firmicutes*	*Negativicutes*	*Selenomonadales*	*Selenomonadaceae*	*Mitsuokella*	*Mitsuokella*
6056	*Firmicutes*	*Negativicutes*	*Selenomonadales*	*Selenomonadaceae*	*Mitsuokella*	*Mitsuokella*
6057	*Firmicutes*	*Negativicutes*	*Selenomonadales*	*Selenomonadaceae*	*Mitsuokella*	*Mitsuokella*
6058	*Firmicutes*	*Negativicutes*	*Selenomonadales*	*Selenomonadaceae*	*Mitsuokella*	*Mitsuokella*
6059	*Firmicutes*	*Negativicutes*	*Selenomonadales*	*Selenomonadaceae*	*Mitsuokella*	*Mitsuokella*
6060	*Firmicutes*	*Negativicutes*	*Selenomonadales*	*Selenomonadaceae*	*Mitsuokella*	*Mitsuokella*
6061	*Firmicutes*	*Negativicutes*	*Selenomonadales*	*Selenomonadaceae*	*Mitsuokella*	*Mitsuokella*
6062	*Firmicutes*	*Negativicutes*	*Selenomonadales*	*Selenomonadaceae*	*Mitsuokella*	*Mitsuokella*
6063	*Firmicutes*	*Negativicutes*	*Selenomonadales*	*Selenomonadaceae*	*Mitsuokella*	*Mitsuokella*
6064	*Firmicutes*	*Negativicutes*	*Selenomonadales*	*Selenomonadaceae*	*Mitsuokella*	*Mitsuokella*
6065	*Firmicutes*	*Negativicutes*	*Selenomonadales*	*Selenomonadaceae*	*Mitsuokella*	*Mitsuokella*
6066	*Firmicutes*	*Negativicutes*	*Selenomonadales*	*Selenomonadaceae*	*Mitsuokella*	*Mitsuokella*
6067	*Firmicutes*	*Negativicutes*	*Selenomonadales*	*Selenomonadaceae*	*Mitsuokella*	*Mitsuokella*
6068	*Firmicutes*	*Negativicutes*	*Selenomonadales*	*Selenomonadaceae*	*Mitsuokella*	*Mitsuokella*
6069	*Firmicutes*	*Negativicutes*	*Selenomonadales*	*Selenomonadaceae*	*Mitsuokella*	*Mitsuokella*
6070	*Firmicutes*	*Negativicutes*	*Selenomonadales*	*Selenomonadaceae*	*Mitsuokella*	*Mitsuokella*
6071	*Firmicutes*	*Negativicutes*	*Selenomonadales*	*Selenomonadaceae*	*Mitsuokella*	*Mitsuokella*
6072	*Firmicutes*	*Negativicutes*	*Selenomonadales*	*Selenomonadaceae*	*Mitsuokella*	*Mitsuokella*
6073	*Firmicutes*	*Negativicutes*	*Selenomonadales*	*Selenomonadaceae*	*Mitsuokella*	*Mitsuokella*
6074	*Firmicutes*	*Negativicutes*	*Selenomonadales*	*Selenomonadaceae*	*Mitsuokella*	*Mitsuokella*
6075	*Firmicutes*	*Negativicutes*	*Selenomonadales*	*Selenomonadaceae*	*Mitsuokella*	*Mitsuokella*
6076	*Firmicutes*	*Negativicutes*	*Selenomonadales*	*Selenomonadaceae*	*Mitsuokella*	*Mitsuokella*

Table S4. Isolated strains by culturomics

bacteriaId	*phylum*	*class*	*order*	*family*	*genus*	*species*
6077	*Firmicutes*	*Negativicutes*	*Selenomonadales*	*Selenomonadaceae*	*Mitsuokella*	*Mitsuokella*
6078	*Firmicutes*	*Negativicutes*	*Selenomonadales*	*Selenomonadaceae*	*Mitsuokella*	*Mitsuokella*
6079	*Firmicutes*	*Negativicutes*	*Selenomonadales*	*Selenomonadaceae*	*Mitsuokella*	*Mitsuokella*
6080	*Firmicutes*	*Negativicutes*	*Selenomonadales*	*Selenomonadaceae*	*Mitsuokella*	*Mitsuokella*
6081	*Firmicutes*	*Negativicutes*	*Selenomonadales*	*Selenomonadaceae*	*Mitsuokella*	*Mitsuokella*
6082	*Firmicutes*	*Negativicutes*	*Selenomonadales*	*Selenomonadaceae*	*Mitsuokella*	*Mitsuokella*
6083	*Firmicutes*	*Negativicutes*	*Selenomonadales*	*Selenomonadaceae*	*Mitsuokella*	*Mitsuokella*
6084	*Firmicutes*	*Negativicutes*	*Selenomonadales*	*Selenomonadaceae*	*Mitsuokella*	*Mitsuokella*
6085	*Firmicutes*	*Negativicutes*	*Selenomonadales*	*Selenomonadaceae*	*Mitsuokella*	*Mitsuokella*
6086	*Firmicutes*	*Negativicutes*	*Selenomonadales*	*Selenomonadaceae*	*Mitsuokella*	*Mitsuokella*
6087	*Firmicutes*	*Negativicutes*	*Selenomonadales*	*Selenomonadaceae*	*Mitsuokella*	*Mitsuokella*
6088	*Firmicutes*	*Negativicutes*	*Selenomonadales*	*Selenomonadaceae*	*Mitsuokella*	*Mitsuokella*
6089	*Firmicutes*	*Negativicutes*	*Selenomonadales*	*Selenomonadaceae*	*Mitsuokella*	*Mitsuokella*
6090	*Firmicutes*	*Negativicutes*	*Selenomonadales*	*Selenomonadaceae*	*Mitsuokella*	*Mitsuokella*
6091	*Firmicutes*	*Negativicutes*	*Selenomonadales*	*Selenomonadaceae*	*Mitsuokella*	*Mitsuokella*
6092	*Firmicutes*	*Negativicutes*	*Selenomonadales*	*Selenomonadaceae*	*Mitsuokella*	*Mitsuokella*
6093	*Firmicutes*	*Negativicutes*	*Selenomonadales*	*Selenomonadaceae*	*Mitsuokella*	*Mitsuokella*
6094	*Firmicutes*	*Negativicutes*	*Selenomonadales*	*Selenomonadaceae*	*Mitsuokella*	*Mitsuokella*
6095	*Firmicutes*	*Negativicutes*	*Selenomonadales*	*Selenomonadaceae*	*Mitsuokella*	*Mitsuokella*
6096	*Firmicutes*	*Negativicutes*	*Selenomonadales*	*Selenomonadaceae*	*Mitsuokella*	*Mitsuokella*
6097	*Firmicutes*	*Negativicutes*	*Selenomonadales*	*Selenomonadaceae*	*Mitsuokella*	*Mitsuokella*
6098	*Firmicutes*	*Negativicutes*	*Selenomonadales*	*Selenomonadaceae*	*Mitsuokella*	*Mitsuokella*
6099	*Firmicutes*	*Negativicutes*	*Selenomonadales*	*Selenomonadaceae*	*Mitsuokella*	*Mitsuokella*
6100	*Firmicutes*	*Negativicutes*	*Selenomonadales*	*Selenomonadaceae*	*Mitsuokella*	*Mitsuokella*
6101	*Firmicutes*	*Negativicutes*	*Selenomonadales*	*Selenomonadaceae*	*Mitsuokella*	*Mitsuokella*
6102	*Firmicutes*	*Negativicutes*	*Selenomonadales*	*Selenomonadaceae*	*Mitsuokella*	*Mitsuokella*
6103	*Firmicutes*	*Negativicutes*	*Selenomonadales*	*Selenomonadaceae*	*Mitsuokella*	*Mitsuokella*
6104	*Firmicutes*	*Negativicutes*	*Selenomonadales*	*Selenomonadaceae*	*Mitsuokella*	*Mitsuokella*

Table S4. Isolated strains by culturomics

bacteriaId	*phylum*	*class*	*order*	*family*	*genus*	*species*
6105	*Firmicutes*	*Negativicutes*	*Selenomonadales*	*Selenomonadaceae*	*Mitsuokella*	*Mitsuokella*
6106	*Firmicutes*	*Negativicutes*	*Selenomonadales*	*Selenomonadaceae*	*Mitsuokella*	*Mitsuokella*
6107	*Firmicutes*	*Negativicutes*	*Selenomonadales*	*Selenomonadaceae*	*Mitsuokella*	*Mitsuokella*
6108	*Firmicutes*	*Negativicutes*	*Selenomonadales*	*Selenomonadaceae*	*Mitsuokella*	*Mitsuokella*
6109	*Firmicutes*	*Negativicutes*	*Selenomonadales*	*Selenomonadaceae*	*Mitsuokella*	*Mitsuokella*
6110	*Firmicutes*	*Negativicutes*	*Selenomonadales*	*Selenomonadaceae*	*Mitsuokella*	*Mitsuokella*
6111	*Firmicutes*	*Negativicutes*	*Selenomonadales*	*Selenomonadaceae*	*Mitsuokella*	*Mitsuokella*
6112	*Firmicutes*	*Negativicutes*	*Selenomonadales*	*Selenomonadaceae*	*Mitsuokella*	*Mitsuokella*
6113	*Firmicutes*	*Negativicutes*	*Selenomonadales*	*Selenomonadaceae*	*Mitsuokella*	*Mitsuokella*
6114	*Firmicutes*	*Negativicutes*	*Selenomonadales*	*Selenomonadaceae*	*Mitsuokella*	*Mitsuokella*
6115	*Firmicutes*	*Negativicutes*	*Selenomonadales*	*Selenomonadaceae*	*Mitsuokella*	*Mitsuokella*
6116	*Firmicutes*	*Negativicutes*	*Selenomonadales*	*Selenomonadaceae*	*Mitsuokella*	*Mitsuokella*
6117	*Firmicutes*	*Negativicutes*	*Selenomonadales*	*Selenomonadaceae*	*Mitsuokella*	*Mitsuokella*
6118	*Firmicutes*	*Negativicutes*	*Selenomonadales*	*Selenomonadaceae*	*Mitsuokella*	*Mitsuokella*
6119	*Firmicutes*	*Negativicutes*	*Selenomonadales*	*Selenomonadaceae*	*Mitsuokella*	*unknown BDW2*
6120	*Firmicutes*	*Negativicutes*	*Veillonellales*	*Veillonellaceae*	*Megasphaera*	*Megasphaera*
6121	*Firmicutes*	*Negativicutes*	*Veillonellales*	*Veillonellaceae*	*Megasphaera*	*unknown B14W6*
6122	*Firmicutes*	*Negativicutes*	*Veillonellales*	*Veillonellaceae*	*Veillonella*	*Veillonella atypica*
6123	*Firmicutes*	*Negativicutes*	*Veillonellales*	*Veillonellaceae*	*Veillonella*	*Veillonella atypica*
6124	*Firmicutes*	*Negativicutes*	*Veillonellales*	*Veillonellaceae*	*Veillonella*	*Veillonella atypica*
6125	*Firmicutes*	*Negativicutes*	*Veillonellales*	*Veillonellaceae*	*Veillonella*	*Veillonella atypica*
6126	*Firmicutes*	*Negativicutes*	*Veillonellales*	*Veillonellaceae*	*Veillonella*	*Veillonella magna*
6127	*Firmicutes*	*Negativicutes*	*Veillonellales*	*Veillonellaceae*	*Veillonella*	*Veillonella magna*
6128	*Firmicutes*	*Negativicutes*	*Veillonellales*	*Veillonellaceae*	*Veillonella*	*Veillonella magna*
6129	*Firmicutes*	*Negativicutes*	*Veillonellales*	*Veillonellaceae*	*Veillonella*	*Veillonella magna*
6130	*Firmicutes*	*Negativicutes*	*Veillonellales*	*Veillonellaceae*	*Veillonella*	*Veillonella parvula*
6131	*Firmicutes*	*Negativicutes*	*Veillonellales*	*Veillonellaceae*	*Veillonella*	*Veillonella parvula*
6132	*Firmicutes*	*Negativicutes*	*Veillonellales*	*Veillonellaceae*	*Veillonella*	*Veillonella parvula*

Table S4. Isolated strains by culturomics

bacteriaId	*phylum*	*class*	*order*	*family*	*genus*	*species*
6133	*Firmicutes*	*Tissierellia*	*Tissierellales*	*Peptoniphilaceae*	*Anaerococcus*	*Anaerococcus*
6134	*Firmicutes*	*Tissierellia*	*Tissierellales*	*Peptoniphilaceae*	*Finegoldia*	*Finegoldia magna*
6135	*Firmicutes*	*Tissierellia*	*Tissierellales*	*Peptoniphilaceae*	*Finegoldia*	*Finegoldia magna*
6136	*Firmicutes*	*Tissierellia*	*Tissierellales*	*Peptoniphilaceae*	*Finegoldia*	*Finegoldia magna*
6137	*Firmicutes*	*Tissierellia*	*Tissierellales*	*Peptoniphilaceae*	*Finegoldia*	*Finegoldia magna*
6138	*Firmicutes*	*unknown*	*unknown*	*unknown*	*unknown*	*unknown B13BH*
6139	*Fusobacteria*	*Fusobacteriia*	*Fusobacteriales*	*Fusobacteriaceae*	*Fusobacterium*	*Fusobacterium*
6140	*Fusobacteria*	*Fusobacteriia*	*Fusobacteriales*	*Fusobacteriaceae*	*Fusobacterium*	*Fusobacterium*
6141	*Fusobacteria*	*Fusobacteriia*	*Fusobacteriales*	*Fusobacteriaceae*	*Fusobacterium*	*Fusobacterium*
6142	*Fusobacteria*	*Fusobacteriia*	*Fusobacteriales*	*Fusobacteriaceae*	*Fusobacterium*	*Fusobacterium*
6143	*Fusobacteria*	*Fusobacteriia*	*Fusobacteriales*	*Fusobacteriaceae*	*Fusobacterium*	*Fusobacterium*
6144	*Fusobacteria*	*Fusobacteriia*	*Fusobacteriales*	*Fusobacteriaceae*	*Fusobacterium*	*Fusobacterium*
6145	*Fusobacteria*	*Fusobacteriia*	*Fusobacteriales*	*Fusobacteriaceae*	*Fusobacterium*	*Fusobacterium*
6146	*Fusobacteria*	*Fusobacteriia*	*Fusobacteriales*	*Fusobacteriaceae*	*Fusobacterium*	*Fusobacterium*
6147	*Fusobacteria*	*Fusobacteriia*	*Fusobacteriales*	*Fusobacteriaceae*	*Fusobacterium*	*Fusobacterium*
6148	*Fusobacteria*	*Fusobacteriia*	*Fusobacteriales*	*Fusobacteriaceae*	*Fusobacterium*	*Fusobacterium*
6149	*Fusobacteria*	*Fusobacteriia*	*Fusobacteriales*	*Fusobacteriaceae*	*Fusobacterium*	*Fusobacterium*
6150	*Fusobacteria*	*Fusobacteriia*	*Fusobacteriales*	*Fusobacteriaceae*	*Fusobacterium*	*Fusobacterium*
6151	*Fusobacteria*	*Fusobacteriia*	*Fusobacteriales*	*Fusobacteriaceae*	*Fusobacterium*	*Fusobacterium*
6152	*Fusobacteria*	*Fusobacteriia*	*Fusobacteriales*	*Fusobacteriaceae*	*Fusobacterium*	*Fusobacterium*
6153	*Fusobacteria*	*Fusobacteriia*	*Fusobacteriales*	*Fusobacteriaceae*	*Fusobacterium*	*Fusobacterium*
6154	*Fusobacteria*	*Fusobacteriia*	*Fusobacteriales*	*Fusobacteriaceae*	*Fusobacterium*	*Fusobacterium*
6155	*Fusobacteria*	*Fusobacteriia*	*Fusobacteriales*	*Fusobacteriaceae*	*Fusobacterium*	*Fusobacterium*
6156	*Fusobacteria*	*Fusobacteriia*	*Fusobacteriales*	*Fusobacteriaceae*	*Fusobacterium*	*Fusobacterium*
6157	*Fusobacteria*	*Fusobacteriia*	*Fusobacteriales*	*Fusobacteriaceae*	*Fusobacterium*	*Fusobacterium*
6158	*Fusobacteria*	*Fusobacteriia*	*Fusobacteriales*	*Fusobacteriaceae*	*Fusobacterium*	*Fusobacterium*
6159	*Fusobacteria*	*Fusobacteriia*	*Fusobacteriales*	*Fusobacteriaceae*	*Fusobacterium*	*Fusobacterium*
6160	*Fusobacteria*	*Fusobacteriia*	*Fusobacteriales*	*Fusobacteriaceae*	*Fusobacterium*	*Fusobacterium*

Table S4. Isolated strains by culturomics

bacteriaId	*phylum*	*class*	*order*	*family*	*genus*	*species*
6161	*Fusobacteria*	*Fusobacteriia*	*Fusobacteriales*	*Fusobacteriaceae*	*Fusobacterium*	*Fusobacterium*
6162	*Fusobacteria*	*Fusobacteriia*	*Fusobacteriales*	*Fusobacteriaceae*	*Fusobacterium*	*Fusobacterium*
6163	*Fusobacteria*	*Fusobacteriia*	*Fusobacteriales*	*Fusobacteriaceae*	*Fusobacterium*	*Fusobacterium*
6164	*Fusobacteria*	*Fusobacteriia*	*Fusobacteriales*	*Fusobacteriaceae*	*Fusobacterium*	*Fusobacterium*
6165	*Fusobacteria*	*Fusobacteriia*	*Fusobacteriales*	*Fusobacteriaceae*	*Fusobacterium*	*Fusobacterium*
6166	*Fusobacteria*	*Fusobacteriia*	*Fusobacteriales*	*Fusobacteriaceae*	*Fusobacterium*	*Fusobacterium*
6167	*Fusobacteria*	*Fusobacteriia*	*Fusobacteriales*	*Fusobacteriaceae*	*Fusobacterium*	*Fusobacterium*
6168	*Fusobacteria*	*Fusobacteriia*	*Fusobacteriales*	*Fusobacteriaceae*	*Fusobacterium*	*Fusobacterium*
6169	*Fusobacteria*	*Fusobacteriia*	*Fusobacteriales*	*Fusobacteriaceae*	*Fusobacterium*	*Fusobacterium*
6170	*Fusobacteria*	*Fusobacteriia*	*Fusobacteriales*	*Fusobacteriaceae*	*Fusobacterium*	*Fusobacterium*
6171	*Fusobacteria*	*Fusobacteriia*	*Fusobacteriales*	*Fusobacteriaceae*	*Fusobacterium*	*Fusobacterium*
6172	*Fusobacteria*	*Fusobacteriia*	*Fusobacteriales*	*Fusobacteriaceae*	*Fusobacterium*	*Fusobacterium*
6173	*Fusobacteria*	*Fusobacteriia*	*Fusobacteriales*	*Fusobacteriaceae*	*Fusobacterium*	*Fusobacterium*
6174	*Fusobacteria*	*Fusobacteriia*	*Fusobacteriales*	*Fusobacteriaceae*	*Fusobacterium*	*Fusobacterium*
6175	*Fusobacteria*	*Fusobacteriia*	*Fusobacteriales*	*Fusobacteriaceae*	*Fusobacterium*	*Fusobacterium*
6176	*Proteobacteria*	*Betaproteobacteria*	*Burkholderiales*	*Burkholderiaceae*	*Ralstonia*	*Ralstonia insidiosa*
6177	*Proteobacteria*	*Betaproteobacteria*	*Burkholderiales*	*Comamonadaceae*	*Comamonas*	*Comamonas*
6178	*Proteobacteria*	*Betaproteobacteria*	*Burkholderiales*	*Comamonadaceae*	*Comamonas*	*Comamonas*
6179	*Proteobacteria*	*Betaproteobacteria*	*Burkholderiales*	*Comamonadaceae*	*Comamonas*	*Comamonas*
6180	*Proteobacteria*	*Betaproteobacteria*	*Burkholderiales*	*Comamonadaceae*	*Comamonas*	*Comamonas*
6181	*Proteobacteria*	*Betaproteobacteria*	*Burkholderiales*	*Comamonadaceae*	*Comamonas*	*Comamonas*
6182	*Proteobacteria*	*Betaproteobacteria*	*Burkholderiales*	*Comamonadaceae*	*Comamonas*	*Comamonas*
6183	*Proteobacteria*	*Betaproteobacteria*	*Burkholderiales*	*Comamonadaceae*	*Comamonas*	*Comamonas*
6184	*Proteobacteria*	*Betaproteobacteria*	*Burkholderiales*	*Comamonadaceae*	*Comamonas*	*Comamonas*
6185	*Proteobacteria*	*Betaproteobacteria*	*Burkholderiales*	*Comamonadaceae*	*Comamonas*	*Comamonas*
6186	*Proteobacteria*	*Betaproteobacteria*	*Burkholderiales*	*Comamonadaceae*	*Comamonas*	*Comamonas*
6187	*Proteobacteria*	*Betaproteobacteria*	*Burkholderiales*	*Comamonadaceae*	*Comamonas*	*Comamonas*
6188	*Proteobacteria*	*Betaproteobacteria*	*Burkholderiales*	*Comamonadaceae*	*Comamonas*	*Comamonas*

Table S4. Isolated strains by culturomics

bacteriaId	*phylum*	*class*	*order*	*family*	*genus*	*species*
6189	*Proteobacteria*	*Betaproteobacteria*	*Burkholderiales*	*Comamonadaceae*	*Comamonas*	*Comamonas*
6190	*Proteobacteria*	*Betaproteobacteria*	*Burkholderiales*	*Comamonadaceae*	*Comamonas*	*Comamonas*
6191	*Proteobacteria*	*Betaproteobacteria*	*Burkholderiales*	*Comamonadaceae*	*Comamonas*	*Comamonas*
6192	*Proteobacteria*	*Betaproteobacteria*	*Burkholderiales*	*Comamonadaceae*	*Comamonas*	*Comamonas*
6193	*Proteobacteria*	*Betaproteobacteria*	*Burkholderiales*	*Comamonadaceae*	*Comamonas*	*Comamonas*
6194	*Proteobacteria*	*Betaproteobacteria*	*Burkholderiales*	*Comamonadaceae*	*Comamonas*	*Comamonas*
6195	*Proteobacteria*	*Betaproteobacteria*	*Burkholderiales*	*Comamonadaceae*	*Comamonas*	*Comamonas*
6196	*Proteobacteria*	*Betaproteobacteria*	*Burkholderiales*	*Comamonadaceae*	*Comamonas*	*Comamonas*
6197	*Proteobacteria*	*Betaproteobacteria*	*Burkholderiales*	*Comamonadaceae*	*Comamonas*	*Comamonas*
6198	*Proteobacteria*	*Betaproteobacteria*	*Burkholderiales*	*Comamonadaceae*	*Comamonas*	*Comamonas*
6199	*Proteobacteria*	*Betaproteobacteria*	*Burkholderiales*	*Comamonadaceae*	*Comamonas*	*Comamonas*
6200	*Proteobacteria*	*Betaproteobacteria*	*Burkholderiales*	*Comamonadaceae*	*Comamonas*	*Comamonas*
6201	*Proteobacteria*	*Betaproteobacteria*	*Burkholderiales*	*Comamonadaceae*	*Comamonas*	*Comamonas*
6202	*Proteobacteria*	*Betaproteobacteria*	*Burkholderiales*	*Comamonadaceae*	*Comamonas*	*Comamonas*
6203	*Proteobacteria*	*Betaproteobacteria*	*Burkholderiales*	*Comamonadaceae*	*Comamonas*	*Comamonas*
6204	*Proteobacteria*	*Betaproteobacteria*	*Burkholderiales*	*Comamonadaceae*	*Comamonas*	*Comamonas*
6205	*Proteobacteria*	*Betaproteobacteria*	*Burkholderiales*	*Comamonadaceae*	*Comamonas*	*Comamonas*
6206	*Proteobacteria*	*Betaproteobacteria*	*Burkholderiales*	*Comamonadaceae*	*Comamonas*	*Comamonas*
6207	*Proteobacteria*	*Betaproteobacteria*	*Burkholderiales*	*Comamonadaceae*	*Comamonas*	*Comamonas*
6208	*Proteobacteria*	*Betaproteobacteria*	*Burkholderiales*	*Comamonadaceae*	*Comamonas*	*Comamonas*
6209	*Proteobacteria*	*Betaproteobacteria*	*Burkholderiales*	*Comamonadaceae*	*Comamonas*	*Comamonas*
6210	*Proteobacteria*	*Betaproteobacteria*	*Burkholderiales*	*Comamonadaceae*	*Comamonas*	*Comamonas*
6211	*Proteobacteria*	*Betaproteobacteria*	*Burkholderiales*	*Comamonadaceae*	*Comamonas*	*Comamonas*
6212	*Proteobacteria*	*Betaproteobacteria*	*Burkholderiales*	*Comamonadaceae*	*Comamonas*	*Comamonas*
6213	*Proteobacteria*	*Betaproteobacteria*	*Burkholderiales*	*Comamonadaceae*	*Comamonas*	*Comamonas*
6214	*Proteobacteria*	*Betaproteobacteria*	*Burkholderiales*	*Comamonadaceae*	*Comamonas*	*Comamonas*
6215	*Proteobacteria*	*Betaproteobacteria*	*Burkholderiales*	*Comamonadaceae*	*Comamonas*	*Comamonas*
6216	*Proteobacteria*	*Betaproteobacteria*	*Burkholderiales*	*Comamonadaceae*	*Comamonas*	*Comamonas*

Table S4. Isolated strains by culturomics

bacteriaId	*phylum*	*class*	*order*	*family*	*genus*	*species*
6217	*Proteobacteria*	*Betaproteobacteria*	*Burkholderiales*	*Comamonadaceae*	*Comamonas*	*Comamonas*
6218	*Proteobacteria*	*Betaproteobacteria*	*Burkholderiales*	*Comamonadaceae*	*Comamonas*	*Comamonas*
6219	*Proteobacteria*	*Betaproteobacteria*	*Burkholderiales*	*Comamonadaceae*	*Comamonas*	*Comamonas*
6220	*Proteobacteria*	*Betaproteobacteria*	*Burkholderiales*	*Comamonadaceae*	*Comamonas*	*Comamonas*
6221	*Proteobacteria*	*Betaproteobacteria*	*Burkholderiales*	*Comamonadaceae*	*Comamonas*	*Comamonas*
6222	*Proteobacteria*	*Betaproteobacteria*	*Burkholderiales*	*Comamonadaceae*	*Comamonas*	*Comamonas*
6223	*Proteobacteria*	*Betaproteobacteria*	*Burkholderiales*	*Comamonadaceae*	*Comamonas*	*Comamonas*
6224	*Proteobacteria*	*Betaproteobacteria*	*Burkholderiales*	*Comamonadaceae*	*Comamonas*	*Comamonas*
6225	*Proteobacteria*	*Betaproteobacteria*	*Burkholderiales*	*Comamonadaceae*	*Comamonas*	*Comamonas*
6226	*Proteobacteria*	*Betaproteobacteria*	*Burkholderiales*	*Comamonadaceae*	*Comamonas*	*Comamonas*
6227	*Proteobacteria*	*Betaproteobacteria*	*Burkholderiales*	*Comamonadaceae*	*Comamonas*	*Comamonas*
6228	*Proteobacteria*	*Betaproteobacteria*	*Burkholderiales*	*Comamonadaceae*	*Comamonas*	*Comamonas*
6229	*Proteobacteria*	*Betaproteobacteria*	*Burkholderiales*	*Comamonadaceae*	*Comamonas*	*Comamonas*
6230	*Proteobacteria*	*Betaproteobacteria*	*Burkholderiales*	*Comamonadaceae*	*Comamonas*	*Comamonas*
6231	*Proteobacteria*	*Betaproteobacteria*	*Burkholderiales*	*Comamonadaceae*	*Comamonas*	*Comamonas*
6232	*Proteobacteria*	*Betaproteobacteria*	*Burkholderiales*	*Comamonadaceae*	*Comamonas*	*Comamonas*
6233	*Proteobacteria*	*Betaproteobacteria*	*Burkholderiales*	*Comamonadaceae*	*Comamonas*	*Comamonas*
6234	*Proteobacteria*	*Betaproteobacteria*	*Burkholderiales*	*Comamonadaceae*	*Comamonas*	*Comamonas*
6235	*Proteobacteria*	*Betaproteobacteria*	*Burkholderiales*	*Comamonadaceae*	*Comamonas*	*Comamonas*
6236	*Proteobacteria*	*Betaproteobacteria*	*Burkholderiales*	*Comamonadaceae*	*Comamonas*	*Comamonas*
6237	*Proteobacteria*	*Betaproteobacteria*	*Burkholderiales*	*Comamonadaceae*	*Comamonas*	*Comamonas*
6238	*Proteobacteria*	*Betaproteobacteria*	*Burkholderiales*	*Comamonadaceae*	*Comamonas*	*Comamonas*
6239	*Proteobacteria*	*Betaproteobacteria*	*Burkholderiales*	*Comamonadaceae*	*Comamonas*	*Comamonas*
6240	*Proteobacteria*	*Betaproteobacteria*	*Burkholderiales*	*Comamonadaceae*	*Comamonas*	*Comamonas*
6241	*Proteobacteria*	*Betaproteobacteria*	*Burkholderiales*	*Comamonadaceae*	*Comamonas*	*Comamonas*
6242	*Proteobacteria*	*Betaproteobacteria*	*Burkholderiales*	*Comamonadaceae*	*Comamonas*	*Comamonas*
6243	*Proteobacteria*	*Betaproteobacteria*	*Burkholderiales*	*Comamonadaceae*	*Comamonas*	*Comamonas*
6244	*Proteobacteria*	*Deltaproteobacteria*	*Myxococcales*	*Cystobacterineae*	*Myxococcaceae*	*Corallococcus*

Table S4. Isolated strains by culturomics

bacteriaId	*phylum*	*class*	*order*	*family*	*genus*	*species*
6245	*Proteobacteria*	*Epsilonproteobacter*	*Campylobacterales*	*Campylobacteracea*	*Campylobacter*	*Campylobacter*
6246	*Proteobacteria*	*Gammaproteobacte*	*Enterobacterales*	*Enterobacteriaceae*	*Citrobacter*	*Citrobacter braakii*
6247	*Proteobacteria*	*Gammaproteobacte*	*Enterobacterales*	*Enterobacteriaceae*	*Citrobacter*	*Citrobacter braakii*
6248	*Proteobacteria*	*Gammaproteobacte*	*Enterobacterales*	*Enterobacteriaceae*	*Citrobacter*	*Citrobacter braakii*
6249	*Proteobacteria*	*Gammaproteobacte*	*Enterobacterales*	*Enterobacteriaceae*	*Citrobacter*	*Citrobacter braakii*
6250	*Proteobacteria*	*Gammaproteobacte*	*Enterobacterales*	*Enterobacteriaceae*	*Citrobacter*	*Citrobacter braakii*
6251	*Proteobacteria*	*Gammaproteobacte*	*Enterobacterales*	*Enterobacteriaceae*	*Citrobacter*	*Citrobacter freundii*
6252	*Proteobacteria*	*Gammaproteobacte*	*Enterobacterales*	*Enterobacteriaceae*	*Citrobacter*	*Citrobacter freundii*
6253	*Proteobacteria*	*Gammaproteobacte*	*Enterobacterales*	*Enterobacteriaceae*	*Citrobacter*	*Citrobacter freundii*
6254	*Proteobacteria*	*Gammaproteobacte*	*Enterobacterales*	*Enterobacteriaceae*	*Citrobacter*	*Citrobacter freundii*
6255	*Proteobacteria*	*Gammaproteobacte*	*Enterobacterales*	*Enterobacteriaceae*	*Citrobacter*	*Citrobacter freundii*
6256	*Proteobacteria*	*Gammaproteobacte*	*Enterobacterales*	*Enterobacteriaceae*	*Citrobacter*	*Citrobacter freundii*
6257	*Proteobacteria*	*Gammaproteobacte*	*Enterobacterales*	*Enterobacteriaceae*	*Citrobacter*	*Citrobacter freundii*
6258	*Proteobacteria*	*Gammaproteobacte*	*Enterobacterales*	*Enterobacteriaceae*	*Citrobacter*	*Citrobacter freundii*
6259	*Proteobacteria*	*Gammaproteobacte*	*Enterobacterales*	*Enterobacteriaceae*	*Citrobacter*	*Citrobacter freundii*
6260	*Proteobacteria*	*Gammaproteobacte*	*Enterobacterales*	*Enterobacteriaceae*	*Citrobacter*	*Citrobacter freundii*
6261	*Proteobacteria*	*Gammaproteobacte*	*Enterobacterales*	*Enterobacteriaceae*	*Citrobacter*	*Citrobacter freundii*
6262	*Proteobacteria*	*Gammaproteobacte*	*Enterobacterales*	*Enterobacteriaceae*	*Citrobacter*	*Citrobacter freundii*
6263	*Proteobacteria*	*Gammaproteobacte*	*Enterobacterales*	*Enterobacteriaceae*	*Citrobacter*	*Citrobacter freundii*
6264	*Proteobacteria*	*Gammaproteobacte*	*Enterobacterales*	*Enterobacteriaceae*	*Citrobacter*	*Citrobacter freundii*
6265	*Proteobacteria*	*Gammaproteobacte*	*Enterobacterales*	*Enterobacteriaceae*	*Citrobacter*	*Citrobacter freundii*
6266	*Proteobacteria*	*Gammaproteobacte*	*Enterobacterales*	*Enterobacteriaceae*	*Citrobacter*	*Citrobacter freundii*
6267	*Proteobacteria*	*Gammaproteobacte*	*Enterobacterales*	*Enterobacteriaceae*	*Citrobacter*	*Citrobacter freundii*
6268	*Proteobacteria*	*Gammaproteobacte*	*Enterobacterales*	*Enterobacteriaceae*	*Citrobacter*	*Citrobacter freundii*
6269	*Proteobacteria*	*Gammaproteobacte*	*Enterobacterales*	*Enterobacteriaceae*	*Citrobacter*	*Citrobacter freundii*
6270	*Proteobacteria*	*Gammaproteobacte*	*Enterobacterales*	*Enterobacteriaceae*	*Citrobacter*	*Citrobacter freundii*
6271	*Proteobacteria*	*Gammaproteobacte*	*Enterobacterales*	*Enterobacteriaceae*	*Citrobacter*	*Citrobacter freundii*
6272	*Proteobacteria*	*Gammaproteobacte*	*Enterobacterales*	*Enterobacteriaceae*	*Citrobacter*	*Citrobacter freundii*

Table S4. Isolated strains by culturomics

bacteriaId	*phylum*	*class*	*order*	*family*	*genus*	*species*
6273	*Proteobacteria*	*Gammaproteobacte*	*Enterobacterales*	*Enterobacteriaceae*	*Citrobacter*	*Citrobacter freundii*
6274	*Proteobacteria*	*Gammaproteobacte*	*Enterobacterales*	*Enterobacteriaceae*	*Citrobacter*	*Citrobacter freundii*
6275	*Proteobacteria*	*Gammaproteobacte*	*Enterobacterales*	*Enterobacteriaceae*	*Citrobacter*	*Citrobacter freundii*
6276	*Proteobacteria*	*Gammaproteobacte*	*Enterobacterales*	*Enterobacteriaceae*	*Citrobacter*	*Citrobacter freundii*
6277	*Proteobacteria*	*Gammaproteobacte*	*Enterobacterales*	*Enterobacteriaceae*	*Citrobacter*	*Citrobacter freundii*
6278	*Proteobacteria*	*Gammaproteobacte*	*Enterobacterales*	*Enterobacteriaceae*	*Citrobacter*	*Citrobacter freundii*
6279	*Proteobacteria*	*Gammaproteobacte*	*Enterobacterales*	*Enterobacteriaceae*	*Citrobacter*	*Citrobacter freundii*
6280	*Proteobacteria*	*Gammaproteobacte*	*Enterobacterales*	*Enterobacteriaceae*	*Citrobacter*	*Citrobacter freundii*
6281	*Proteobacteria*	*Gammaproteobacte*	*Enterobacterales*	*Enterobacteriaceae*	*Citrobacter*	*Citrobacter freundii*
6282	*Proteobacteria*	*Gammaproteobacte*	*Enterobacterales*	*Enterobacteriaceae*	*Citrobacter*	*Citrobacter freundii*
6283	*Proteobacteria*	*Gammaproteobacte*	*Enterobacterales*	*Enterobacteriaceae*	*Citrobacter*	*Citrobacter freundii*
6284	*Proteobacteria*	*Gammaproteobacte*	*Enterobacterales*	*Enterobacteriaceae*	*Citrobacter*	*Citrobacter freundii*
6285	*Proteobacteria*	*Gammaproteobacte*	*Enterobacterales*	*Enterobacteriaceae*	*Citrobacter*	*Citrobacter freundii*
6286	*Proteobacteria*	*Gammaproteobacte*	*Enterobacterales*	*Enterobacteriaceae*	*Citrobacter*	*Citrobacter freundii*
6287	*Proteobacteria*	*Gammaproteobacte*	*Enterobacterales*	*Enterobacteriaceae*	*Citrobacter*	*Citrobacter freundii*
6288	*Proteobacteria*	*Gammaproteobacte*	*Enterobacterales*	*Enterobacteriaceae*	*Citrobacter*	*Citrobacter koseri*
6289	*Proteobacteria*	*Gammaproteobacte*	*Enterobacterales*	*Enterobacteriaceae*	*Citrobacter*	*Citrobacter koseri*
6290	*Proteobacteria*	*Gammaproteobacte*	*Enterobacterales*	*Enterobacteriaceae*	*Citrobacter*	*Citrobacter koseri*
6291	*Proteobacteria*	*Gammaproteobacte*	*Enterobacterales*	*Enterobacteriaceae*	*Citrobacter*	*Citrobacter koseri*
6292	*Proteobacteria*	*Gammaproteobacte*	*Enterobacterales*	*Enterobacteriaceae*	*Citrobacter*	*Citrobacter koseri*
6293	*Proteobacteria*	*Gammaproteobacte*	*Enterobacterales*	*Enterobacteriaceae*	*Citrobacter*	*Citrobacter koseri*
6294	*Proteobacteria*	*Gammaproteobacte*	*Enterobacterales*	*Enterobacteriaceae*	*Citrobacter*	*Citrobacter koseri*
6295	*Proteobacteria*	*Gammaproteobacte*	*Enterobacterales*	*Enterobacteriaceae*	*Citrobacter*	*Citrobacter koseri*
6296	*Proteobacteria*	*Gammaproteobacte*	*Enterobacterales*	*Enterobacteriaceae*	*Citrobacter*	*Citrobacter koseri*
6297	*Proteobacteria*	*Gammaproteobacte*	*Enterobacterales*	*Enterobacteriaceae*	*Citrobacter*	*Citrobacter koseri*
6298	*Proteobacteria*	*Gammaproteobacte*	*Enterobacterales*	*Enterobacteriaceae*	*Citrobacter*	*Citrobacter koseri*
6299	*Proteobacteria*	*Gammaproteobacte*	*Enterobacterales*	*Enterobacteriaceae*	*Citrobacter*	*Citrobacter koseri*
6300	*Proteobacteria*	*Gammaproteobacte*	*Enterobacterales*	*Enterobacteriaceae*	*Citrobacter*	*Citrobacter koseri*

Table S4. Isolated strains by culturomics

bacteriaId	*phylum*	*class*	*order*	*family*	*genus*	*species*
6301	*Proteobacteria*	*Gammaproteobacte*	*Enterobacterales*	*Enterobacteriaceae*	*Citrobacter*	*Citrobacter koseri*
6302	*Proteobacteria*	*Gammaproteobacte*	*Enterobacterales*	*Enterobacteriaceae*	*Citrobacter*	*Citrobacter koseri*
6303	*Proteobacteria*	*Gammaproteobacte*	*Enterobacterales*	*Enterobacteriaceae*	*Citrobacter*	*Citrobacter koseri*
6304	*Proteobacteria*	*Gammaproteobacte*	*Enterobacterales*	*Enterobacteriaceae*	*Citrobacter*	*Citrobacter koseri*
6305	*Proteobacteria*	*Gammaproteobacte*	*Enterobacterales*	*Enterobacteriaceae*	*Citrobacter*	*Citrobacter koseri*
6306	*Proteobacteria*	*Gammaproteobacte*	*Enterobacterales*	*Enterobacteriaceae*	*Citrobacter*	*Citrobacter koseri*
6307	*Proteobacteria*	*Gammaproteobacte*	*Enterobacterales*	*Enterobacteriaceae*	*Citrobacter*	*Citrobacter koseri*
6308	*Proteobacteria*	*Gammaproteobacte*	*Enterobacterales*	*Enterobacteriaceae*	*Citrobacter*	*Citrobacter koseri*
6309	*Proteobacteria*	*Gammaproteobacte*	*Enterobacterales*	*Enterobacteriaceae*	*Citrobacter*	*Citrobacter koseri*
6310	*Proteobacteria*	*Gammaproteobacte*	*Enterobacterales*	*Enterobacteriaceae*	*Citrobacter*	*Citrobacter koseri*
6311	*Proteobacteria*	*Gammaproteobacte*	*Enterobacterales*	*Enterobacteriaceae*	*Citrobacter*	*Citrobacter koseri*
6312	*Proteobacteria*	*Gammaproteobacte*	*Enterobacterales*	*Enterobacteriaceae*	*Citrobacter*	*Citrobacter koseri*
6313	*Proteobacteria*	*Gammaproteobacte*	*Enterobacterales*	*Enterobacteriaceae*	*Citrobacter*	*Citrobacter koseri*
6314	*Proteobacteria*	*Gammaproteobacte*	*Enterobacterales*	*Enterobacteriaceae*	*Citrobacter*	*Citrobacter koseri*
6315	*Proteobacteria*	*Gammaproteobacte*	*Enterobacterales*	*Enterobacteriaceae*	*Citrobacter*	*Citrobacter koseri*
6316	*Proteobacteria*	*Gammaproteobacte*	*Enterobacterales*	*Enterobacteriaceae*	*Citrobacter*	*Citrobacter koseri*
6317	*Proteobacteria*	*Gammaproteobacte*	*Enterobacterales*	*Enterobacteriaceae*	*Citrobacter*	*Citrobacter koseri*
6318	*Proteobacteria*	*Gammaproteobacte*	*Enterobacterales*	*Enterobacteriaceae*	*Citrobacter*	*Citrobacter koseri*
6319	*Proteobacteria*	*Gammaproteobacte*	*Enterobacterales*	*Enterobacteriaceae*	*Citrobacter*	*Citrobacter koseri*
6320	*Proteobacteria*	*Gammaproteobacte*	*Enterobacterales*	*Enterobacteriaceae*	*Citrobacter*	*Citrobacter koseri*
6321	*Proteobacteria*	*Gammaproteobacte*	*Enterobacterales*	*Enterobacteriaceae*	*Citrobacter*	*Citrobacter koseri*
6322	*Proteobacteria*	*Gammaproteobacte*	*Enterobacterales*	*Enterobacteriaceae*	*Citrobacter*	*Citrobacter koseri*
6323	*Proteobacteria*	*Gammaproteobacte*	*Enterobacterales*	*Enterobacteriaceae*	*Citrobacter*	*Citrobacter koseri*
6324	*Proteobacteria*	*Gammaproteobacte*	*Enterobacterales*	*Enterobacteriaceae*	*Citrobacter*	*Citrobacter koseri*
6325	*Proteobacteria*	*Gammaproteobacte*	*Enterobacterales*	*Enterobacteriaceae*	*Citrobacter*	*Citrobacter koseri*
6326	*Proteobacteria*	*Gammaproteobacte*	*Enterobacterales*	*Enterobacteriaceae*	*Citrobacter*	*Citrobacter koseri*
6327	*Proteobacteria*	*Gammaproteobacte*	*Enterobacterales*	*Enterobacteriaceae*	*Citrobacter*	*Citrobacter koseri*
6328	*Proteobacteria*	*Gammaproteobacte*	*Enterobacterales*	*Enterobacteriaceae*	*Citrobacter*	*Citrobacter koseri*

Table S4. Isolated strains by culturomics

bacteriaId	*phylum*	*class*	*order*	*family*	*genus*	*species*
6329	*Proteobacteria*	*Gammaproteobacte*	*Enterobacterales*	*Enterobacteriaceae*	*Citrobacter*	*Citrobacter koseri*
6330	*Proteobacteria*	*Gammaproteobacte*	*Enterobacterales*	*Enterobacteriaceae*	*Citrobacter*	*Citrobacter koseri*
6331	*Proteobacteria*	*Gammaproteobacte*	*Enterobacterales*	*Enterobacteriaceae*	*Citrobacter*	*Citrobacter koseri*
6332	*Proteobacteria*	*Gammaproteobacte*	*Enterobacterales*	*Enterobacteriaceae*	*Citrobacter*	*Citrobacter*
6333	*Proteobacteria*	*Gammaproteobacte*	*Enterobacterales*	*Enterobacteriaceae*	*Cronobacter*	*Cronobacter*
6334	*Proteobacteria*	*Gammaproteobacte*	*Enterobacterales*	*Enterobacteriaceae*	*Cronobacter*	*Cronobacter*
6335	*Proteobacteria*	*Gammaproteobacte*	*Enterobacterales*	*Enterobacteriaceae*	*Enterobacter*	*Enterobacter*
6336	*Proteobacteria*	*Gammaproteobacte*	*Enterobacterales*	*Enterobacteriaceae*	*Enterobacter*	*Enterobacter*
6337	*Proteobacteria*	*Gammaproteobacte*	*Enterobacterales*	*Enterobacteriaceae*	*Enterobacter*	*Enterobacter*
6338	*Proteobacteria*	*Gammaproteobacte*	*Enterobacterales*	*Enterobacteriaceae*	*Enterobacter*	*Enterobacter*
6339	*Proteobacteria*	*Gammaproteobacte*	*Enterobacterales*	*Enterobacteriaceae*	*Enterobacter*	*Enterobacter*
6340	*Proteobacteria*	*Gammaproteobacte*	*Enterobacterales*	*Enterobacteriaceae*	*Enterobacter*	*Enterobacter*
6341	*Proteobacteria*	*Gammaproteobacte*	*Enterobacterales*	*Enterobacteriaceae*	*Enterobacter*	*Enterobacter*
6342	*Proteobacteria*	*Gammaproteobacte*	*Enterobacterales*	*Enterobacteriaceae*	*Enterobacter*	*Enterobacter*
6343	*Proteobacteria*	*Gammaproteobacte*	*Enterobacterales*	*Enterobacteriaceae*	*Enterobacter*	*Enterobacter*
6344	*Proteobacteria*	*Gammaproteobacte*	*Enterobacterales*	*Enterobacteriaceae*	*Enterobacter*	*Enterobacter*
6345	*Proteobacteria*	*Gammaproteobacte*	*Enterobacterales*	*Enterobacteriaceae*	*Enterobacter*	*Enterobacter*
6346	*Proteobacteria*	*Gammaproteobacte*	*Enterobacterales*	*Enterobacteriaceae*	*Enterobacter*	*Enterobacter*
6347	*Proteobacteria*	*Gammaproteobacte*	*Enterobacterales*	*Enterobacteriaceae*	*Enterobacter*	*Enterobacter*
6348	*Proteobacteria*	*Gammaproteobacte*	*Enterobacterales*	*Enterobacteriaceae*	*Enterobacter*	*Enterobacter*
6349	*Proteobacteria*	*Gammaproteobacte*	*Enterobacterales*	*Enterobacteriaceae*	*Enterobacter*	*Enterobacter*
6350	*Proteobacteria*	*Gammaproteobacte*	*Enterobacterales*	*Enterobacteriaceae*	*Enterobacter*	*Enterobacter*
6351	*Proteobacteria*	*Gammaproteobacte*	*Enterobacterales*	*Enterobacteriaceae*	*Enterobacter*	*Enterobacter*
6352	*Proteobacteria*	*Gammaproteobacte*	*Enterobacterales*	*Enterobacteriaceae*	*Enterobacter*	*Enterobacter*
6353	*Proteobacteria*	*Gammaproteobacte*	*Enterobacterales*	*Enterobacteriaceae*	*Enterobacter*	*Enterobacter*
6354	*Proteobacteria*	*Gammaproteobacte*	*Enterobacterales*	*Enterobacteriaceae*	*Enterobacter*	*Enterobacter*
6355	*Proteobacteria*	*Gammaproteobacte*	*Enterobacterales*	*Enterobacteriaceae*	*Enterobacter*	*Enterobacter*
6356	*Proteobacteria*	*Gammaproteobacte*	*Enterobacterales*	*Enterobacteriaceae*	*Enterobacter*	*Enterobacter*

Table S4. Isolated strains by culturomics

bacteriaId	phylum	class	order	family	genus	species
6357	*Proteobacteria*	*Gammaproteobacte*	*Enterobacterales*	*Enterobacteriaceae*	*Enterobacter*	*Enterobacter*
6358	*Proteobacteria*	*Gammaproteobacte*	*Enterobacterales*	*Enterobacteriaceae*	*Enterobacter*	*Enterobacter*
6359	*Proteobacteria*	*Gammaproteobacte*	*Enterobacterales*	*Enterobacteriaceae*	*Enterobacter*	*Enterobacter*
6360	*Proteobacteria*	*Gammaproteobacte*	*Enterobacterales*	*Enterobacteriaceae*	*Enterobacter*	*Enterobacter*
6361	*Proteobacteria*	*Gammaproteobacte*	*Enterobacterales*	*Enterobacteriaceae*	*Enterobacter*	*Enterobacter*
6362	*Proteobacteria*	*Gammaproteobacte*	*Enterobacterales*	*Enterobacteriaceae*	*Enterobacter*	*Enterobacter*
6363	*Proteobacteria*	*Gammaproteobacte*	*Enterobacterales*	*Enterobacteriaceae*	*Enterobacter*	*Enterobacter*
6364	*Proteobacteria*	*Gammaproteobacte*	*Enterobacterales*	*Enterobacteriaceae*	*Enterobacter*	*Enterobacter*
6365	*Proteobacteria*	*Gammaproteobacte*	*Enterobacterales*	*Enterobacteriaceae*	*Enterobacter*	*Enterobacter*
6366	*Proteobacteria*	*Gammaproteobacte*	*Enterobacterales*	*Enterobacteriaceae*	*Enterobacter*	*Enterobacter*
6367	*Proteobacteria*	*Gammaproteobacte*	*Enterobacterales*	*Enterobacteriaceae*	*Enterobacter*	*Enterobacter kobei*
6368	*Proteobacteria*	*Gammaproteobacte*	*Enterobacterales*	*Enterobacteriaceae*	*Enterobacter*	*Enterobacter kobei*
6369	*Proteobacteria*	*Gammaproteobacte*	*Enterobacterales*	*Enterobacteriaceae*	*Enterobacter*	*Enterobacter*
6370	*Proteobacteria*	*Gammaproteobacte*	*Enterobacterales*	*Enterobacteriaceae*	*Enterobacter*	*Enterobacter*
6371	*Proteobacteria*	*Gammaproteobacte*	*Enterobacterales*	*Enterobacteriaceae*	*Enterobacter*	*Enterobacter*
6372	*Proteobacteria*	*Gammaproteobacte*	*Enterobacterales*	*Enterobacteriaceae*	*Enterobacter*	*Enterobacter*
6373	*Proteobacteria*	*Gammaproteobacte*	*Enterobacterales*	*Enterobacteriaceae*	*Enterobacter*	*Enterobacter*
6374	*Proteobacteria*	*Gammaproteobacte*	*Enterobacterales*	*Enterobacteriaceae*	*Enterobacter*	*Enterobacter*
6375	*Proteobacteria*	*Gammaproteobacte*	*Enterobacterales*	*Enterobacteriaceae*	*Enterobacter*	*Enterobacter*
6376	*Proteobacteria*	*Gammaproteobacte*	*Enterobacterales*	*Enterobacteriaceae*	*Enterobacter*	*Enterobacter*
6377	*Proteobacteria*	*Gammaproteobacte*	*Enterobacterales*	*Enterobacteriaceae*	*Enterobacter*	*Enterobacter*
6378	*Proteobacteria*	*Gammaproteobacte*	*Enterobacterales*	*Enterobacteriaceae*	*Enterobacter*	*Enterobacter*
6379	*Proteobacteria*	*Gammaproteobacte*	*Enterobacterales*	*Enterobacteriaceae*	*Enterobacter*	*Enterobacter*
6380	*Proteobacteria*	*Gammaproteobacte*	*Enterobacterales*	*Enterobacteriaceae*	*Enterobacter*	*Enterobacter*
6381	*Proteobacteria*	*Gammaproteobacte*	*Enterobacterales*	*Enterobacteriaceae*	*Enterobacter*	*Enterobacter*
6382	*Proteobacteria*	*Gammaproteobacte*	*Enterobacterales*	*Enterobacteriaceae*	*Enterobacter*	*Enterobacter*
6383	*Proteobacteria*	*Gammaproteobacte*	*Enterobacterales*	*Enterobacteriaceae*	*Enterobacter*	*Enterobacter*
6384	*Proteobacteria*	*Gammaproteobacte*	*Enterobacterales*	*Enterobacteriaceae*	*Enterobacter*	*Enterobacter*

Table S4. Isolated strains by culturomics

bacteriaId	*phylum*	*class*	*order*	*family*	*genus*	*species*
6385	*Proteobacteria*	*Gammaproteobacte*	*Enterobacterales*	*Enterobacteriaceae*	*Enterobacter*	*Enterobacter*
6386	*Proteobacteria*	*Gammaproteobacte*	*Enterobacterales*	*Enterobacteriaceae*	*Enterobacter*	*Enterobacter*
6387	*Proteobacteria*	*Gammaproteobacte*	*Enterobacterales*	*Enterobacteriaceae*	*Enterobacter*	*Enterobacter*
6388	*Proteobacteria*	*Gammaproteobacte*	*Enterobacterales*	*Enterobacteriaceae*	*Enterobacter*	*Enterobacter*
6389	*Proteobacteria*	*Gammaproteobacte*	*Enterobacterales*	*Enterobacteriaceae*	*Enterobacter*	*Enterobacter_cloac*
6390	*Proteobacteria*	*Gammaproteobacte*	*Enterobacterales*	*Enterobacteriaceae*	*Enterobacter*	*Enterobacter_cloac*
6391	*Proteobacteria*	*Gammaproteobacte*	*Enterobacterales*	*Enterobacteriaceae*	*Enterobacter*	*Enterobacter_cloac*
6392	*Proteobacteria*	*Gammaproteobacte*	*Enterobacterales*	*Enterobacteriaceae*	*Enterobacter*	*Enterobacter_cloac*
6393	*Proteobacteria*	*Gammaproteobacte*	*Enterobacterales*	*Enterobacteriaceae*	*Enterobacter*	*Enterobacter_cloac*
6394	*Proteobacteria*	*Gammaproteobacte*	*Enterobacterales*	*Enterobacteriaceae*	*Enterobacteriaceae*	*Enterobacteriaceae*
6395	*Proteobacteria*	*Gammaproteobacte*	*Enterobacterales*	*Enterobacteriaceae*	*Escherichia*	*Escherichia coli*
6396	*Proteobacteria*	*Gammaproteobacte*	*Enterobacterales*	*Enterobacteriaceae*	*Escherichia*	*Escherichia coli*
6397	*Proteobacteria*	*Gammaproteobacte*	*Enterobacterales*	*Enterobacteriaceae*	*Escherichia*	*Escherichia coli*
6398	*Proteobacteria*	*Gammaproteobacte*	*Enterobacterales*	*Enterobacteriaceae*	*Escherichia*	*Escherichia coli*
6399	*Proteobacteria*	*Gammaproteobacte*	*Enterobacterales*	*Enterobacteriaceae*	*Escherichia*	*Escherichia coli*
6400	*Proteobacteria*	*Gammaproteobacte*	*Enterobacterales*	*Enterobacteriaceae*	*Escherichia*	*Escherichia coli*
6401	*Proteobacteria*	*Gammaproteobacte*	*Enterobacterales*	*Enterobacteriaceae*	*Escherichia*	*Escherichia coli*
6402	*Proteobacteria*	*Gammaproteobacte*	*Enterobacterales*	*Enterobacteriaceae*	*Escherichia*	*Escherichia coli*
6403	*Proteobacteria*	*Gammaproteobacte*	*Enterobacterales*	*Enterobacteriaceae*	*Escherichia*	*Escherichia coli*
6404	*Proteobacteria*	*Gammaproteobacte*	*Enterobacterales*	*Enterobacteriaceae*	*Escherichia*	*Escherichia coli*
6405	*Proteobacteria*	*Gammaproteobacte*	*Enterobacterales*	*Enterobacteriaceae*	*Escherichia*	*Escherichia coli*
6406	*Proteobacteria*	*Gammaproteobacte*	*Enterobacterales*	*Enterobacteriaceae*	*Escherichia*	*Escherichia coli*
6407	*Proteobacteria*	*Gammaproteobacte*	*Enterobacterales*	*Enterobacteriaceae*	*Escherichia*	*Escherichia coli*
6408	*Proteobacteria*	*Gammaproteobacte*	*Enterobacterales*	*Enterobacteriaceae*	*Escherichia*	*Escherichia coli*
6409	*Proteobacteria*	*Gammaproteobacte*	*Enterobacterales*	*Enterobacteriaceae*	*Escherichia*	*Escherichia coli*
6410	*Proteobacteria*	*Gammaproteobacte*	*Enterobacterales*	*Enterobacteriaceae*	*Escherichia*	*Escherichia coli*
6411	*Proteobacteria*	*Gammaproteobacte*	*Enterobacterales*	*Enterobacteriaceae*	*Escherichia*	*Escherichia coli*
6412	*Proteobacteria*	*Gammaproteobacte*	*Enterobacterales*	*Enterobacteriaceae*	*Escherichia*	*Escherichia coli*

Table S4. Isolated strains by culturomics

bacteriaId	*phylum*	*class*	*order*	*family*	*genus*	*species*
6413	*Proteobacteria*	*Gammaproteobacte*	*Enterobacterales*	*Enterobacteriaceae*	*Escherichia*	*Escherichia coli*
6414	*Proteobacteria*	*Gammaproteobacte*	*Enterobacterales*	*Enterobacteriaceae*	*Escherichia*	*Escherichia coli*
6415	*Proteobacteria*	*Gammaproteobacte*	*Enterobacterales*	*Enterobacteriaceae*	*Escherichia*	*Escherichia coli*
6416	*Proteobacteria*	*Gammaproteobacte*	*Enterobacterales*	*Enterobacteriaceae*	*Escherichia*	*Escherichia coli*
6417	*Proteobacteria*	*Gammaproteobacte*	*Enterobacterales*	*Enterobacteriaceae*	*Escherichia*	*Escherichia coli*
6418	*Proteobacteria*	*Gammaproteobacte*	*Enterobacterales*	*Enterobacteriaceae*	*Escherichia*	*Escherichia coli*
6419	*Proteobacteria*	*Gammaproteobacte*	*Enterobacterales*	*Enterobacteriaceae*	*Escherichia*	*Escherichia coli*
6420	*Proteobacteria*	*Gammaproteobacte*	*Enterobacterales*	*Enterobacteriaceae*	*Escherichia*	*Escherichia coli*
6421	*Proteobacteria*	*Gammaproteobacte*	*Enterobacterales*	*Enterobacteriaceae*	*Escherichia*	*Escherichia coli*
6422	*Proteobacteria*	*Gammaproteobacte*	*Enterobacterales*	*Enterobacteriaceae*	*Escherichia*	*Escherichia coli*
6423	*Proteobacteria*	*Gammaproteobacte*	*Enterobacterales*	*Enterobacteriaceae*	*Escherichia*	*Escherichia coli*
6424	*Proteobacteria*	*Gammaproteobacte*	*Enterobacterales*	*Enterobacteriaceae*	*Escherichia*	*Escherichia coli*
6425	*Proteobacteria*	*Gammaproteobacte*	*Enterobacterales*	*Enterobacteriaceae*	*Escherichia*	*Escherichia coli*
6426	*Proteobacteria*	*Gammaproteobacte*	*Enterobacterales*	*Enterobacteriaceae*	*Escherichia*	*Escherichia coli*
6427	*Proteobacteria*	*Gammaproteobacte*	*Enterobacterales*	*Enterobacteriaceae*	*Escherichia*	*Escherichia coli*
6428	*Proteobacteria*	*Gammaproteobacte*	*Enterobacterales*	*Enterobacteriaceae*	*Escherichia*	*Escherichia coli*
6429	*Proteobacteria*	*Gammaproteobacte*	*Enterobacterales*	*Enterobacteriaceae*	*Escherichia*	*Escherichia coli*
6430	*Proteobacteria*	*Gammaproteobacte*	*Enterobacterales*	*Enterobacteriaceae*	*Escherichia*	*Escherichia coli*
6431	*Proteobacteria*	*Gammaproteobacte*	*Enterobacterales*	*Enterobacteriaceae*	*Escherichia*	*Escherichia coli*
6432	*Proteobacteria*	*Gammaproteobacte*	*Enterobacterales*	*Enterobacteriaceae*	*Escherichia*	*Escherichia coli*
6433	*Proteobacteria*	*Gammaproteobacte*	*Enterobacterales*	*Enterobacteriaceae*	*Escherichia*	*Escherichia coli*
6434	*Proteobacteria*	*Gammaproteobacte*	*Enterobacterales*	*Enterobacteriaceae*	*Escherichia*	*Escherichia coli*
6435	*Proteobacteria*	*Gammaproteobacte*	*Enterobacterales*	*Enterobacteriaceae*	*Escherichia*	*Escherichia coli*
6436	*Proteobacteria*	*Gammaproteobacte*	*Enterobacterales*	*Enterobacteriaceae*	*Escherichia*	*Escherichia coli*
6437	*Proteobacteria*	*Gammaproteobacte*	*Enterobacterales*	*Enterobacteriaceae*	*Escherichia*	*Escherichia coli*
6438	*Proteobacteria*	*Gammaproteobacte*	*Enterobacterales*	*Enterobacteriaceae*	*Escherichia*	*Escherichia coli*
6439	*Proteobacteria*	*Gammaproteobacte*	*Enterobacterales*	*Enterobacteriaceae*	*Escherichia*	*Escherichia coli*
6440	*Proteobacteria*	*Gammaproteobacte*	*Enterobacterales*	*Enterobacteriaceae*	*Escherichia*	*Escherichia coli*

Table S4. Isolated strains by culturomics

bacteriaId	*phylum*	*class*	*order*	*family*	*genus*	*species*
6441	*Proteobacteria*	*Gammaproteobacte*	*Enterobacterales*	*Enterobacteriaceae*	*Escherichia*	*Escherichia coli*
6442	*Proteobacteria*	*Gammaproteobacte*	*Enterobacterales*	*Enterobacteriaceae*	*Escherichia*	*Escherichia coli*
6443	*Proteobacteria*	*Gammaproteobacte*	*Enterobacterales*	*Enterobacteriaceae*	*Escherichia*	*Escherichia coli*
6444	*Proteobacteria*	*Gammaproteobacte*	*Enterobacterales*	*Enterobacteriaceae*	*Escherichia*	*Escherichia coli*
6445	*Proteobacteria*	*Gammaproteobacte*	*Enterobacterales*	*Enterobacteriaceae*	*Escherichia*	*Escherichia coli*
6446	*Proteobacteria*	*Gammaproteobacte*	*Enterobacterales*	*Enterobacteriaceae*	*Escherichia*	*Escherichia coli*
6447	*Proteobacteria*	*Gammaproteobacte*	*Enterobacterales*	*Enterobacteriaceae*	*Escherichia*	*Escherichia coli*
6448	*Proteobacteria*	*Gammaproteobacte*	*Enterobacterales*	*Enterobacteriaceae*	*Escherichia*	*Escherichia coli*
6449	*Proteobacteria*	*Gammaproteobacte*	*Enterobacterales*	*Enterobacteriaceae*	*Escherichia*	*Escherichia coli*
6450	*Proteobacteria*	*Gammaproteobacte*	*Enterobacterales*	*Enterobacteriaceae*	*Escherichia*	*Escherichia coli*
6451	*Proteobacteria*	*Gammaproteobacte*	*Enterobacterales*	*Enterobacteriaceae*	*Escherichia*	*Escherichia coli*
6452	*Proteobacteria*	*Gammaproteobacte*	*Enterobacterales*	*Enterobacteriaceae*	*Escherichia*	*Escherichia coli*
6453	*Proteobacteria*	*Gammaproteobacte*	*Enterobacterales*	*Enterobacteriaceae*	*Escherichia*	*Escherichia coli*
6454	*Proteobacteria*	*Gammaproteobacte*	*Enterobacterales*	*Enterobacteriaceae*	*Escherichia*	*Escherichia coli*
6455	*Proteobacteria*	*Gammaproteobacte*	*Enterobacterales*	*Enterobacteriaceae*	*Escherichia*	*Escherichia coli*
6456	*Proteobacteria*	*Gammaproteobacte*	*Enterobacterales*	*Enterobacteriaceae*	*Escherichia*	*Escherichia coli*
6457	*Proteobacteria*	*Gammaproteobacte*	*Enterobacterales*	*Enterobacteriaceae*	*Escherichia*	*Escherichia coli*
6458	*Proteobacteria*	*Gammaproteobacte*	*Enterobacterales*	*Enterobacteriaceae*	*Escherichia*	*Escherichia coli*
6459	*Proteobacteria*	*Gammaproteobacte*	*Enterobacterales*	*Enterobacteriaceae*	*Escherichia*	*Escherichia coli*
6460	*Proteobacteria*	*Gammaproteobacte*	*Enterobacterales*	*Enterobacteriaceae*	*Escherichia*	*Escherichia coli*
6461	*Proteobacteria*	*Gammaproteobacte*	*Enterobacterales*	*Enterobacteriaceae*	*Escherichia*	*Escherichia coli*
6462	*Proteobacteria*	*Gammaproteobacte*	*Enterobacterales*	*Enterobacteriaceae*	*Escherichia*	*Escherichia coli*
6463	*Proteobacteria*	*Gammaproteobacte*	*Enterobacterales*	*Enterobacteriaceae*	*Escherichia*	*Escherichia coli*
6464	*Proteobacteria*	*Gammaproteobacte*	*Enterobacterales*	*Enterobacteriaceae*	*Escherichia*	*Escherichia coli*
6465	*Proteobacteria*	*Gammaproteobacte*	*Enterobacterales*	*Enterobacteriaceae*	*Escherichia*	*Escherichia coli*
6466	*Proteobacteria*	*Gammaproteobacte*	*Enterobacterales*	*Enterobacteriaceae*	*Escherichia*	*Escherichia coli*
6467	*Proteobacteria*	*Gammaproteobacte*	*Enterobacterales*	*Enterobacteriaceae*	*Escherichia*	*Escherichia coli*
6468	*Proteobacteria*	*Gammaproteobacte*	*Enterobacterales*	*Enterobacteriaceae*	*Escherichia*	*Escherichia coli*

Table S4. Isolated strains by culturomics

bacteriaId	*phylum*	*class*	*order*	*family*	*genus*	*species*
6469	*Proteobacteria*	*Gammaproteobacte*	*Enterobacterales*	*Enterobacteriaceae*	*Escherichia*	*Escherichia coli*
6470	*Proteobacteria*	*Gammaproteobacte*	*Enterobacterales*	*Enterobacteriaceae*	*Escherichia*	*Escherichia coli*
6471	*Proteobacteria*	*Gammaproteobacte*	*Enterobacterales*	*Enterobacteriaceae*	*Escherichia*	*Escherichia coli*
6472	*Proteobacteria*	*Gammaproteobacte*	*Enterobacterales*	*Enterobacteriaceae*	*Escherichia*	*Escherichia coli*
6473	*Proteobacteria*	*Gammaproteobacte*	*Enterobacterales*	*Enterobacteriaceae*	*Escherichia*	*Escherichia coli*
6474	*Proteobacteria*	*Gammaproteobacte*	*Enterobacterales*	*Enterobacteriaceae*	*Escherichia*	*Escherichia coli*
6475	*Proteobacteria*	*Gammaproteobacte*	*Enterobacterales*	*Enterobacteriaceae*	*Escherichia*	*Escherichia coli*
6476	*Proteobacteria*	*Gammaproteobacte*	*Enterobacterales*	*Enterobacteriaceae*	*Escherichia*	*Escherichia coli*
6477	*Proteobacteria*	*Gammaproteobacte*	*Enterobacterales*	*Enterobacteriaceae*	*Escherichia*	*Escherichia coli*
6478	*Proteobacteria*	*Gammaproteobacte*	*Enterobacterales*	*Enterobacteriaceae*	*Escherichia*	*Escherichia coli*
6479	*Proteobacteria*	*Gammaproteobacte*	*Enterobacterales*	*Enterobacteriaceae*	*Escherichia*	*Escherichia coli*
6480	*Proteobacteria*	*Gammaproteobacte*	*Enterobacterales*	*Enterobacteriaceae*	*Escherichia*	*Escherichia coli*
6481	*Proteobacteria*	*Gammaproteobacte*	*Enterobacterales*	*Enterobacteriaceae*	*Escherichia*	*Escherichia coli*
6482	*Proteobacteria*	*Gammaproteobacte*	*Enterobacterales*	*Enterobacteriaceae*	*Escherichia*	*Escherichia coli*
6483	*Proteobacteria*	*Gammaproteobacte*	*Enterobacterales*	*Enterobacteriaceae*	*Escherichia*	*Escherichia coli*
6484	*Proteobacteria*	*Gammaproteobacte*	*Enterobacterales*	*Enterobacteriaceae*	*Escherichia*	*Escherichia coli*
6485	*Proteobacteria*	*Gammaproteobacte*	*Enterobacterales*	*Enterobacteriaceae*	*Escherichia*	*Escherichia coli*
6486	*Proteobacteria*	*Gammaproteobacte*	*Enterobacterales*	*Enterobacteriaceae*	*Escherichia*	*Escherichia coli*
6487	*Proteobacteria*	*Gammaproteobacte*	*Enterobacterales*	*Enterobacteriaceae*	*Escherichia*	*Escherichia coli*
6488	*Proteobacteria*	*Gammaproteobacte*	*Enterobacterales*	*Enterobacteriaceae*	*Escherichia*	*Escherichia coli*
6489	*Proteobacteria*	*Gammaproteobacte*	*Enterobacterales*	*Enterobacteriaceae*	*Escherichia*	*Escherichia coli*
6490	*Proteobacteria*	*Gammaproteobacte*	*Enterobacterales*	*Enterobacteriaceae*	*Escherichia*	*Escherichia coli*
6491	*Proteobacteria*	*Gammaproteobacte*	*Enterobacterales*	*Enterobacteriaceae*	*Escherichia*	*Escherichia coli*
6492	*Proteobacteria*	*Gammaproteobacte*	*Enterobacterales*	*Enterobacteriaceae*	*Escherichia*	*Escherichia coli*
6493	*Proteobacteria*	*Gammaproteobacte*	*Enterobacterales*	*Enterobacteriaceae*	*Escherichia*	*Escherichia coli*
6494	*Proteobacteria*	*Gammaproteobacte*	*Enterobacterales*	*Enterobacteriaceae*	*Escherichia*	*Escherichia coli*
6495	*Proteobacteria*	*Gammaproteobacte*	*Enterobacterales*	*Enterobacteriaceae*	*Escherichia*	*Escherichia coli*
6496	*Proteobacteria*	*Gammaproteobacte*	*Enterobacterales*	*Enterobacteriaceae*	*Escherichia*	*Escherichia coli*

Table S4. Isolated strains by culturomics

bacterialId	*phylum*	*class*	*order*	*family*	*genus*	*species*
6497	*Proteobacteria*	*Gammaproteobacte*	*Enterobacterales*	*Enterobacteriaceae*	*Escherichia*	*Escherichia coli*
6498	*Proteobacteria*	*Gammaproteobacte*	*Enterobacterales*	*Enterobacteriaceae*	*Escherichia*	*Escherichia coli*
6499	*Proteobacteria*	*Gammaproteobacte*	*Enterobacterales*	*Enterobacteriaceae*	*Escherichia*	*Escherichia coli*
6500	*Proteobacteria*	*Gammaproteobacte*	*Enterobacterales*	*Enterobacteriaceae*	*Escherichia*	*Escherichia coli*
6501	*Proteobacteria*	*Gammaproteobacte*	*Enterobacterales*	*Enterobacteriaceae*	*Escherichia*	*Escherichia coli*
6502	*Proteobacteria*	*Gammaproteobacte*	*Enterobacterales*	*Enterobacteriaceae*	*Escherichia*	*Escherichia coli*
6503	*Proteobacteria*	*Gammaproteobacte*	*Enterobacterales*	*Enterobacteriaceae*	*Escherichia*	*Escherichia coli*
6504	*Proteobacteria*	*Gammaproteobacte*	*Enterobacterales*	*Enterobacteriaceae*	*Escherichia*	*Escherichia coli*
6505	*Proteobacteria*	*Gammaproteobacte*	*Enterobacterales*	*Enterobacteriaceae*	*Escherichia*	*Escherichia coli*
6506	*Proteobacteria*	*Gammaproteobacte*	*Enterobacterales*	*Enterobacteriaceae*	*Escherichia*	*Escherichia coli*
6507	*Proteobacteria*	*Gammaproteobacte*	*Enterobacterales*	*Enterobacteriaceae*	*Escherichia*	*Escherichia coli*
6508	*Proteobacteria*	*Gammaproteobacte*	*Enterobacterales*	*Enterobacteriaceae*	*Escherichia*	*Escherichia coli*
6509	*Proteobacteria*	*Gammaproteobacte*	*Enterobacterales*	*Enterobacteriaceae*	*Escherichia*	*Escherichia coli*
6510	*Proteobacteria*	*Gammaproteobacte*	*Enterobacterales*	*Enterobacteriaceae*	*Escherichia*	*Escherichia coli*
6511	*Proteobacteria*	*Gammaproteobacte*	*Enterobacterales*	*Enterobacteriaceae*	*Escherichia*	*Escherichia coli*
6512	*Proteobacteria*	*Gammaproteobacte*	*Enterobacterales*	*Enterobacteriaceae*	*Escherichia*	*Escherichia coli*
6513	*Proteobacteria*	*Gammaproteobacte*	*Enterobacterales*	*Enterobacteriaceae*	*Escherichia*	*Escherichia coli*
6514	*Proteobacteria*	*Gammaproteobacte*	*Enterobacterales*	*Enterobacteriaceae*	*Escherichia*	*Escherichia coli*
6515	*Proteobacteria*	*Gammaproteobacte*	*Enterobacterales*	*Enterobacteriaceae*	*Escherichia*	*Escherichia coli*
6516	*Proteobacteria*	*Gammaproteobacte*	*Enterobacterales*	*Enterobacteriaceae*	*Escherichia*	*Escherichia coli*
6517	*Proteobacteria*	*Gammaproteobacte*	*Enterobacterales*	*Enterobacteriaceae*	*Escherichia*	*Escherichia coli*
6518	*Proteobacteria*	*Gammaproteobacte*	*Enterobacterales*	*Enterobacteriaceae*	*Escherichia*	*Escherichia coli*
6519	*Proteobacteria*	*Gammaproteobacte*	*Enterobacterales*	*Enterobacteriaceae*	*Escherichia*	*Escherichia coli*
6520	*Proteobacteria*	*Gammaproteobacte*	*Enterobacterales*	*Enterobacteriaceae*	*Escherichia*	*Escherichia coli*
6521	*Proteobacteria*	*Gammaproteobacte*	*Enterobacterales*	*Enterobacteriaceae*	*Escherichia*	*Escherichia coli*
6522	*Proteobacteria*	*Gammaproteobacte*	*Enterobacterales*	*Enterobacteriaceae*	*Escherichia*	*Escherichia coli*
6523	*Proteobacteria*	*Gammaproteobacte*	*Enterobacterales*	*Enterobacteriaceae*	*Escherichia*	*Escherichia coli*
6524	*Proteobacteria*	*Gammaproteobacte*	*Enterobacterales*	*Enterobacteriaceae*	*Escherichia*	*Escherichia coli*

Table S4. Isolated strains by culturomics

bacteriaId	*phylum*	*class*	*order*	*family*	*genus*	*species*
6525	*Proteobacteria*	*Gammaproteobacte*	*Enterobacterales*	*Enterobacteriaceae*	*Escherichia*	*Escherichia coli*
6526	*Proteobacteria*	*Gammaproteobacte*	*Enterobacterales*	*Enterobacteriaceae*	*Escherichia*	*Escherichia coli*
6527	*Proteobacteria*	*Gammaproteobacte*	*Enterobacterales*	*Enterobacteriaceae*	*Escherichia*	*Escherichia coli*
6528	*Proteobacteria*	*Gammaproteobacte*	*Enterobacterales*	*Enterobacteriaceae*	*Escherichia*	*Escherichia coli*
6529	*Proteobacteria*	*Gammaproteobacte*	*Enterobacterales*	*Enterobacteriaceae*	*Escherichia*	*Escherichia coli*
6530	*Proteobacteria*	*Gammaproteobacte*	*Enterobacterales*	*Enterobacteriaceae*	*Escherichia*	*Escherichia coli*
6531	*Proteobacteria*	*Gammaproteobacte*	*Enterobacterales*	*Enterobacteriaceae*	*Escherichia*	*Escherichia coli*
6532	*Proteobacteria*	*Gammaproteobacte*	*Enterobacterales*	*Enterobacteriaceae*	*Escherichia*	*Escherichia coli*
6533	*Proteobacteria*	*Gammaproteobacte*	*Enterobacterales*	*Enterobacteriaceae*	*Escherichia*	*Escherichia coli*
6534	*Proteobacteria*	*Gammaproteobacte*	*Enterobacterales*	*Enterobacteriaceae*	*Escherichia*	*Escherichia coli*
6535	*Proteobacteria*	*Gammaproteobacte*	*Enterobacterales*	*Enterobacteriaceae*	*Escherichia*	*Escherichia coli*
6536	*Proteobacteria*	*Gammaproteobacte*	*Enterobacterales*	*Enterobacteriaceae*	*Escherichia*	*Escherichia coli*
6537	*Proteobacteria*	*Gammaproteobacte*	*Enterobacterales*	*Enterobacteriaceae*	*Escherichia*	*Escherichia coli*
6538	*Proteobacteria*	*Gammaproteobacte*	*Enterobacterales*	*Enterobacteriaceae*	*Escherichia*	*Escherichia coli*
6539	*Proteobacteria*	*Gammaproteobacte*	*Enterobacterales*	*Enterobacteriaceae*	*Escherichia*	*Escherichia coli*
6540	*Proteobacteria*	*Gammaproteobacte*	*Enterobacterales*	*Enterobacteriaceae*	*Escherichia*	*Escherichia coli*
6541	*Proteobacteria*	*Gammaproteobacte*	*Enterobacterales*	*Enterobacteriaceae*	*Escherichia*	*Escherichia coli*
6542	*Proteobacteria*	*Gammaproteobacte*	*Enterobacterales*	*Enterobacteriaceae*	*Escherichia*	*Escherichia coli*
6543	*Proteobacteria*	*Gammaproteobacte*	*Enterobacterales*	*Enterobacteriaceae*	*Escherichia*	*Escherichia coli*
6544	*Proteobacteria*	*Gammaproteobacte*	*Enterobacterales*	*Enterobacteriaceae*	*Escherichia*	*Escherichia coli*
6545	*Proteobacteria*	*Gammaproteobacte*	*Enterobacterales*	*Enterobacteriaceae*	*Escherichia*	*Escherichia coli*
6546	*Proteobacteria*	*Gammaproteobacte*	*Enterobacterales*	*Enterobacteriaceae*	*Escherichia*	*Escherichia coli*
6547	*Proteobacteria*	*Gammaproteobacte*	*Enterobacterales*	*Enterobacteriaceae*	*Escherichia*	*Escherichia coli*
6548	*Proteobacteria*	*Gammaproteobacte*	*Enterobacterales*	*Enterobacteriaceae*	*Escherichia*	*Escherichia coli*
6549	*Proteobacteria*	*Gammaproteobacte*	*Enterobacterales*	*Enterobacteriaceae*	*Escherichia*	*Escherichia coli*
6550	*Proteobacteria*	*Gammaproteobacte*	*Enterobacterales*	*Enterobacteriaceae*	*Escherichia*	*Escherichia coli*
6551	*Proteobacteria*	*Gammaproteobacte*	*Enterobacterales*	*Enterobacteriaceae*	*Escherichia*	*Escherichia coli*
6552	*Proteobacteria*	*Gammaproteobacte*	*Enterobacterales*	*Enterobacteriaceae*	*Escherichia*	*Escherichia coli*

Table S4. Isolated strains by culturomics

bacteriaId	phylum	class	order	family	genus	species
6553	*Proteobacteria*	*Gammaproteobacte*	*Enterobacterales*	*Enterobacteriaceae*	*Escherichia*	*Escherichia coli*
6554	*Proteobacteria*	*Gammaproteobacte*	*Enterobacterales*	*Enterobacteriaceae*	*Escherichia*	*Escherichia coli*
6555	*Proteobacteria*	*Gammaproteobacte*	*Enterobacterales*	*Enterobacteriaceae*	*Escherichia*	*Escherichia coli*
6556	*Proteobacteria*	*Gammaproteobacte*	*Enterobacterales*	*Enterobacteriaceae*	*Escherichia*	*Escherichia coli*
6557	*Proteobacteria*	*Gammaproteobacte*	*Enterobacterales*	*Enterobacteriaceae*	*Escherichia*	*Escherichia coli*
6558	*Proteobacteria*	*Gammaproteobacte*	*Enterobacterales*	*Enterobacteriaceae*	*Escherichia*	*Escherichia coli*
6559	*Proteobacteria*	*Gammaproteobacte*	*Enterobacterales*	*Enterobacteriaceae*	*Escherichia*	*Escherichia coli*
6560	*Proteobacteria*	*Gammaproteobacte*	*Enterobacterales*	*Enterobacteriaceae*	*Escherichia*	*Escherichia coli*
6561	*Proteobacteria*	*Gammaproteobacte*	*Enterobacterales*	*Enterobacteriaceae*	*Escherichia*	*Escherichia coli*
6562	*Proteobacteria*	*Gammaproteobacte*	*Enterobacterales*	*Enterobacteriaceae*	*Escherichia*	*Escherichia coli*
6563	*Proteobacteria*	*Gammaproteobacte*	*Enterobacterales*	*Enterobacteriaceae*	*Escherichia*	*Escherichia coli*
6564	*Proteobacteria*	*Gammaproteobacte*	*Enterobacterales*	*Enterobacteriaceae*	*Escherichia*	*Escherichia coli*
6565	*Proteobacteria*	*Gammaproteobacte*	*Enterobacterales*	*Enterobacteriaceae*	*Escherichia*	*Escherichia coli*
6566	*Proteobacteria*	*Gammaproteobacte*	*Enterobacterales*	*Enterobacteriaceae*	*Escherichia*	*Escherichia coli*
6567	*Proteobacteria*	*Gammaproteobacte*	*Enterobacterales*	*Enterobacteriaceae*	*Escherichia*	*Escherichia coli*
6568	*Proteobacteria*	*Gammaproteobacte*	*Enterobacterales*	*Enterobacteriaceae*	*Escherichia*	*Escherichia coli*
6569	*Proteobacteria*	*Gammaproteobacte*	*Enterobacterales*	*Enterobacteriaceae*	*Escherichia*	*Escherichia coli*
6570	*Proteobacteria*	*Gammaproteobacte*	*Enterobacterales*	*Enterobacteriaceae*	*Escherichia*	*Escherichia coli*
6571	*Proteobacteria*	*Gammaproteobacte*	*Enterobacterales*	*Enterobacteriaceae*	*Escherichia*	*Escherichia coli*
6572	*Proteobacteria*	*Gammaproteobacte*	*Enterobacterales*	*Enterobacteriaceae*	*Escherichia*	*Escherichia coli*
6573	*Proteobacteria*	*Gammaproteobacte*	*Enterobacterales*	*Enterobacteriaceae*	*Escherichia*	*Escherichia coli*
6574	*Proteobacteria*	*Gammaproteobacte*	*Enterobacterales*	*Enterobacteriaceae*	*Escherichia*	*Escherichia coli*
6575	*Proteobacteria*	*Gammaproteobacte*	*Enterobacterales*	*Enterobacteriaceae*	*Escherichia*	*Escherichia coli*
6576	*Proteobacteria*	*Gammaproteobacte*	*Enterobacterales*	*Enterobacteriaceae*	*Escherichia*	*Escherichia coli*
6577	*Proteobacteria*	*Gammaproteobacte*	*Enterobacterales*	*Enterobacteriaceae*	*Escherichia*	*Escherichia coli*
6578	*Proteobacteria*	*Gammaproteobacte*	*Enterobacterales*	*Enterobacteriaceae*	*Escherichia*	*Escherichia coli*
6579	*Proteobacteria*	*Gammaproteobacte*	*Enterobacterales*	*Enterobacteriaceae*	*Escherichia*	*Escherichia coli*
6580	*Proteobacteria*	*Gammaproteobacte*	*Enterobacterales*	*Enterobacteriaceae*	*Escherichia*	*Escherichia coli*

Table S4. Isolated strains by culturomics

bacteriaId	*phylum*	*class*	*order*	*family*	*genus*	*species*
6581	*Proteobacteria*	*Gammaproteobacte*	*Enterobacterales*	*Enterobacteriaceae*	*Escherichia*	*Escherichia coli*
6582	*Proteobacteria*	*Gammaproteobacte*	*Enterobacterales*	*Enterobacteriaceae*	*Escherichia*	*Escherichia coli*
6583	*Proteobacteria*	*Gammaproteobacte*	*Enterobacterales*	*Enterobacteriaceae*	*Escherichia*	*Escherichia coli*
6584	*Proteobacteria*	*Gammaproteobacte*	*Enterobacterales*	*Enterobacteriaceae*	*Escherichia*	*Escherichia coli*
6585	*Proteobacteria*	*Gammaproteobacte*	*Enterobacterales*	*Enterobacteriaceae*	*Escherichia*	*Escherichia coli*
6586	*Proteobacteria*	*Gammaproteobacte*	*Enterobacterales*	*Enterobacteriaceae*	*Escherichia*	*Escherichia coli*
6587	*Proteobacteria*	*Gammaproteobacte*	*Enterobacterales*	*Enterobacteriaceae*	*Escherichia*	*Escherichia coli*
6588	*Proteobacteria*	*Gammaproteobacte*	*Enterobacterales*	*Enterobacteriaceae*	*Escherichia*	*Escherichia coli*
6589	*Proteobacteria*	*Gammaproteobacte*	*Enterobacterales*	*Enterobacteriaceae*	*Escherichia*	*Escherichia coli*
6590	*Proteobacteria*	*Gammaproteobacte*	*Enterobacterales*	*Enterobacteriaceae*	*Escherichia*	*Escherichia coli*
6591	*Proteobacteria*	*Gammaproteobacte*	*Enterobacterales*	*Enterobacteriaceae*	*Escherichia*	*Escherichia coli*
6592	*Proteobacteria*	*Gammaproteobacte*	*Enterobacterales*	*Enterobacteriaceae*	*Escherichia*	*Escherichia coli*
6593	*Proteobacteria*	*Gammaproteobacte*	*Enterobacterales*	*Enterobacteriaceae*	*Escherichia*	*Escherichia coli*
6594	*Proteobacteria*	*Gammaproteobacte*	*Enterobacterales*	*Enterobacteriaceae*	*Escherichia*	*Escherichia coli*
6595	*Proteobacteria*	*Gammaproteobacte*	*Enterobacterales*	*Enterobacteriaceae*	*Escherichia*	*Escherichia coli*
6596	*Proteobacteria*	*Gammaproteobacte*	*Enterobacterales*	*Enterobacteriaceae*	*Escherichia*	*Escherichia coli*
6597	*Proteobacteria*	*Gammaproteobacte*	*Enterobacterales*	*Enterobacteriaceae*	*Escherichia*	*Escherichia coli*
6598	*Proteobacteria*	*Gammaproteobacte*	*Enterobacterales*	*Enterobacteriaceae*	*Escherichia*	*Escherichia coli*
6599	*Proteobacteria*	*Gammaproteobacte*	*Enterobacterales*	*Enterobacteriaceae*	*Escherichia*	*Escherichia coli*
6600	*Proteobacteria*	*Gammaproteobacte*	*Enterobacterales*	*Enterobacteriaceae*	*Escherichia*	*Escherichia coli*
6601	*Proteobacteria*	*Gammaproteobacte*	*Enterobacterales*	*Enterobacteriaceae*	*Escherichia*	*Escherichia coli*
6602	*Proteobacteria*	*Gammaproteobacte*	*Enterobacterales*	*Enterobacteriaceae*	*Escherichia*	*Escherichia coli*
6603	*Proteobacteria*	*Gammaproteobacte*	*Enterobacterales*	*Enterobacteriaceae*	*Escherichia*	*Escherichia coli*
6604	*Proteobacteria*	*Gammaproteobacte*	*Enterobacterales*	*Enterobacteriaceae*	*Escherichia*	*Escherichia coli*
6605	*Proteobacteria*	*Gammaproteobacte*	*Enterobacterales*	*Enterobacteriaceae*	*Escherichia*	*Escherichia coli*
6606	*Proteobacteria*	*Gammaproteobacte*	*Enterobacterales*	*Enterobacteriaceae*	*Escherichia*	*Escherichia coli*
6607	*Proteobacteria*	*Gammaproteobacte*	*Enterobacterales*	*Enterobacteriaceae*	*Escherichia*	*Escherichia coli*
6608	*Proteobacteria*	*Gammaproteobacte*	*Enterobacterales*	*Enterobacteriaceae*	*Escherichia*	*Escherichia coli*

Table S4. Isolated strains by culturomics

bacteriaId	*phylum*	*class*	*order*	*family*	*genus*	*species*
6609	*Proteobacteria*	*Gammaproteobacte*	*Enterobacterales*	*Enterobacteriaceae*	*Escherichia*	*Escherichia coli*
6610	*Proteobacteria*	*Gammaproteobacte*	*Enterobacterales*	*Enterobacteriaceae*	*Escherichia*	*Escherichia coli*
6611	*Proteobacteria*	*Gammaproteobacte*	*Enterobacterales*	*Enterobacteriaceae*	*Escherichia*	*Escherichia coli*
6612	*Proteobacteria*	*Gammaproteobacte*	*Enterobacterales*	*Enterobacteriaceae*	*Escherichia*	*Escherichia coli*
6613	*Proteobacteria*	*Gammaproteobacte*	*Enterobacterales*	*Enterobacteriaceae*	*Escherichia*	*Escherichia coli*
6614	*Proteobacteria*	*Gammaproteobacte*	*Enterobacterales*	*Enterobacteriaceae*	*Escherichia*	*Escherichia coli*
6615	*Proteobacteria*	*Gammaproteobacte*	*Enterobacterales*	*Enterobacteriaceae*	*Escherichia*	*Escherichia coli*
6616	*Proteobacteria*	*Gammaproteobacte*	*Enterobacterales*	*Enterobacteriaceae*	*Escherichia*	*Escherichia coli*
6617	*Proteobacteria*	*Gammaproteobacte*	*Enterobacterales*	*Enterobacteriaceae*	*Escherichia*	*Escherichia coli*
6618	*Proteobacteria*	*Gammaproteobacte*	*Enterobacterales*	*Enterobacteriaceae*	*Escherichia*	*Escherichia coli*
6619	*Proteobacteria*	*Gammaproteobacte*	*Enterobacterales*	*Enterobacteriaceae*	*Escherichia*	*Escherichia coli*
6620	*Proteobacteria*	*Gammaproteobacte*	*Enterobacterales*	*Enterobacteriaceae*	*Escherichia*	*Escherichia coli*
6621	*Proteobacteria*	*Gammaproteobacte*	*Enterobacterales*	*Enterobacteriaceae*	*Escherichia*	*Escherichia coli*
6622	*Proteobacteria*	*Gammaproteobacte*	*Enterobacterales*	*Enterobacteriaceae*	*Escherichia*	*Escherichia coli*
6623	*Proteobacteria*	*Gammaproteobacte*	*Enterobacterales*	*Enterobacteriaceae*	*Escherichia*	*Escherichia coli*
6624	*Proteobacteria*	*Gammaproteobacte*	*Enterobacterales*	*Enterobacteriaceae*	*Escherichia*	*Escherichia coli*
6625	*Proteobacteria*	*Gammaproteobacte*	*Enterobacterales*	*Enterobacteriaceae*	*Escherichia*	*Escherichia coli*
6626	*Proteobacteria*	*Gammaproteobacte*	*Enterobacterales*	*Enterobacteriaceae*	*Escherichia*	*Escherichia coli*
6627	*Proteobacteria*	*Gammaproteobacte*	*Enterobacterales*	*Enterobacteriaceae*	*Escherichia*	*Escherichia coli*
6628	*Proteobacteria*	*Gammaproteobacte*	*Enterobacterales*	*Enterobacteriaceae*	*Escherichia*	*Escherichia coli*
6629	*Proteobacteria*	*Gammaproteobacte*	*Enterobacterales*	*Enterobacteriaceae*	*Escherichia*	*Escherichia coli*
6630	*Proteobacteria*	*Gammaproteobacte*	*Enterobacterales*	*Enterobacteriaceae*	*Escherichia*	*Escherichia coli*
6631	*Proteobacteria*	*Gammaproteobacte*	*Enterobacterales*	*Enterobacteriaceae*	*Escherichia*	*Escherichia coli*
6632	*Proteobacteria*	*Gammaproteobacte*	*Enterobacterales*	*Enterobacteriaceae*	*Escherichia*	*Escherichia coli*
6633	*Proteobacteria*	*Gammaproteobacte*	*Enterobacterales*	*Enterobacteriaceae*	*Escherichia*	*Escherichia coli*
6634	*Proteobacteria*	*Gammaproteobacte*	*Enterobacterales*	*Enterobacteriaceae*	*Escherichia*	*Escherichia coli*
6635	*Proteobacteria*	*Gammaproteobacte*	*Enterobacterales*	*Enterobacteriaceae*	*Escherichia*	*Escherichia coli*
6636	*Proteobacteria*	*Gammaproteobacte*	*Enterobacterales*	*Enterobacteriaceae*	*Escherichia*	*Escherichia coli*

Table S4. Isolated strains by culturomics

bacteriaId	*phylum*	*class*	*order*	*family*	*genus*	*species*
6637	*Proteobacteria*	*Gammaproteobacte*	*Enterobacterales*	*Enterobacteriaceae*	*Escherichia*	*Escherichia coli*
6638	*Proteobacteria*	*Gammaproteobacte*	*Enterobacterales*	*Enterobacteriaceae*	*Escherichia*	*Escherichia coli*
6639	*Proteobacteria*	*Gammaproteobacte*	*Enterobacterales*	*Enterobacteriaceae*	*Escherichia*	*Escherichia coli*
6640	*Proteobacteria*	*Gammaproteobacte*	*Enterobacterales*	*Enterobacteriaceae*	*Escherichia*	*Escherichia coli*
6641	*Proteobacteria*	*Gammaproteobacte*	*Enterobacterales*	*Enterobacteriaceae*	*Escherichia*	*Escherichia coli*
6642	*Proteobacteria*	*Gammaproteobacte*	*Enterobacterales*	*Enterobacteriaceae*	*Escherichia*	*Escherichia coli*
6643	*Proteobacteria*	*Gammaproteobacte*	*Enterobacterales*	*Enterobacteriaceae*	*Escherichia*	*Escherichia coli*
6644	*Proteobacteria*	*Gammaproteobacte*	*Enterobacterales*	*Enterobacteriaceae*	*Escherichia*	*Escherichia coli*
6645	*Proteobacteria*	*Gammaproteobacte*	*Enterobacterales*	*Enterobacteriaceae*	*Escherichia*	*Escherichia coli*
6646	*Proteobacteria*	*Gammaproteobacte*	*Enterobacterales*	*Enterobacteriaceae*	*Escherichia*	*Escherichia coli*
6647	*Proteobacteria*	*Gammaproteobacte*	*Enterobacterales*	*Enterobacteriaceae*	*Escherichia*	*Escherichia coli*
6648	*Proteobacteria*	*Gammaproteobacte*	*Enterobacterales*	*Enterobacteriaceae*	*Escherichia*	*Escherichia coli*
6649	*Proteobacteria*	*Gammaproteobacte*	*Enterobacterales*	*Enterobacteriaceae*	*Escherichia*	*Escherichia coli*
6650	*Proteobacteria*	*Gammaproteobacte*	*Enterobacterales*	*Enterobacteriaceae*	*Escherichia*	*Escherichia coli*
6651	*Proteobacteria*	*Gammaproteobacte*	*Enterobacterales*	*Enterobacteriaceae*	*Escherichia*	*Escherichia coli*
6652	*Proteobacteria*	*Gammaproteobacte*	*Enterobacterales*	*Enterobacteriaceae*	*Escherichia*	*Escherichia coli*
6653	*Proteobacteria*	*Gammaproteobacte*	*Enterobacterales*	*Enterobacteriaceae*	*Escherichia*	*Escherichia coli*
6654	*Proteobacteria*	*Gammaproteobacte*	*Enterobacterales*	*Enterobacteriaceae*	*Escherichia*	*Escherichia coli*
6655	*Proteobacteria*	*Gammaproteobacte*	*Enterobacterales*	*Enterobacteriaceae*	*Escherichia*	*Escherichia coli*
6656	*Proteobacteria*	*Gammaproteobacte*	*Enterobacterales*	*Enterobacteriaceae*	*Escherichia*	*Escherichia coli*
6657	*Proteobacteria*	*Gammaproteobacte*	*Enterobacterales*	*Enterobacteriaceae*	*Escherichia*	*Escherichia coli*
6658	*Proteobacteria*	*Gammaproteobacte*	*Enterobacterales*	*Enterobacteriaceae*	*Escherichia*	*Escherichia coli*
6659	*Proteobacteria*	*Gammaproteobacte*	*Enterobacterales*	*Enterobacteriaceae*	*Escherichia*	*Escherichia coli*
6660	*Proteobacteria*	*Gammaproteobacte*	*Enterobacterales*	*Enterobacteriaceae*	*Escherichia*	*Escherichia coli*
6661	*Proteobacteria*	*Gammaproteobacte*	*Enterobacterales*	*Enterobacteriaceae*	*Escherichia*	*Escherichia coli*
6662	*Proteobacteria*	*Gammaproteobacte*	*Enterobacterales*	*Enterobacteriaceae*	*Escherichia*	*Escherichia coli*
6663	*Proteobacteria*	*Gammaproteobacte*	*Enterobacterales*	*Enterobacteriaceae*	*Escherichia*	*Escherichia coli*
6664	*Proteobacteria*	*Gammaproteobacte*	*Enterobacterales*	*Enterobacteriaceae*	*Escherichia*	*Escherichia coli*

Table S4. Isolated strains by culturomics

bacteriaId	*phylum*	*class*	*order*	*family*	*genus*	*species*
6665	*Proteobacteria*	*Gammaproteobacte*	*Enterobacterales*	*Enterobacteriaceae*	*Escherichia*	*Escherichia coli*
6666	*Proteobacteria*	*Gammaproteobacte*	*Enterobacterales*	*Enterobacteriaceae*	*Escherichia*	*Escherichia coli*
6667	*Proteobacteria*	*Gammaproteobacte*	*Enterobacterales*	*Enterobacteriaceae*	*Escherichia*	*Escherichia coli*
6668	*Proteobacteria*	*Gammaproteobacte*	*Enterobacterales*	*Enterobacteriaceae*	*Escherichia*	*Escherichia coli*
6669	*Proteobacteria*	*Gammaproteobacte*	*Enterobacterales*	*Enterobacteriaceae*	*Escherichia*	*Escherichia coli*
6670	*Proteobacteria*	*Gammaproteobacte*	*Enterobacterales*	*Enterobacteriaceae*	*Escherichia*	*Escherichia coli*
6671	*Proteobacteria*	*Gammaproteobacte*	*Enterobacterales*	*Enterobacteriaceae*	*Escherichia*	*Escherichia coli*
6672	*Proteobacteria*	*Gammaproteobacte*	*Enterobacterales*	*Enterobacteriaceae*	*Escherichia*	*Escherichia coli*
6673	*Proteobacteria*	*Gammaproteobacte*	*Enterobacterales*	*Enterobacteriaceae*	*Escherichia*	*Escherichia coli*
6674	*Proteobacteria*	*Gammaproteobacte*	*Enterobacterales*	*Enterobacteriaceae*	*Escherichia*	*Escherichia coli*
6675	*Proteobacteria*	*Gammaproteobacte*	*Enterobacterales*	*Enterobacteriaceae*	*Escherichia*	*Escherichia coli*
6676	*Proteobacteria*	*Gammaproteobacte*	*Enterobacterales*	*Enterobacteriaceae*	*Escherichia*	*Escherichia coli*
6677	*Proteobacteria*	*Gammaproteobacte*	*Enterobacterales*	*Enterobacteriaceae*	*Escherichia*	*Escherichia coli*
6678	*Proteobacteria*	*Gammaproteobacte*	*Enterobacterales*	*Enterobacteriaceae*	*Escherichia*	*Escherichia coli*
6679	*Proteobacteria*	*Gammaproteobacte*	*Enterobacterales*	*Enterobacteriaceae*	*Escherichia*	*Escherichia coli*
6680	*Proteobacteria*	*Gammaproteobacte*	*Enterobacterales*	*Enterobacteriaceae*	*Escherichia*	*Escherichia coli*
6681	*Proteobacteria*	*Gammaproteobacte*	*Enterobacterales*	*Enterobacteriaceae*	*Escherichia*	*Escherichia coli*
6682	*Proteobacteria*	*Gammaproteobacte*	*Enterobacterales*	*Enterobacteriaceae*	*Escherichia*	*Escherichia coli*
6683	*Proteobacteria*	*Gammaproteobacte*	*Enterobacterales*	*Enterobacteriaceae*	*Escherichia*	*Escherichia coli*
6684	*Proteobacteria*	*Gammaproteobacte*	*Enterobacterales*	*Enterobacteriaceae*	*Escherichia*	*Escherichia coli*
6685	*Proteobacteria*	*Gammaproteobacte*	*Enterobacterales*	*Enterobacteriaceae*	*Escherichia*	*Escherichia coli*
6686	*Proteobacteria*	*Gammaproteobacte*	*Enterobacterales*	*Enterobacteriaceae*	*Escherichia*	*Escherichia coli*
6687	*Proteobacteria*	*Gammaproteobacte*	*Enterobacterales*	*Enterobacteriaceae*	*Escherichia*	*Escherichia coli*
6688	*Proteobacteria*	*Gammaproteobacte*	*Enterobacterales*	*Enterobacteriaceae*	*Escherichia*	*Escherichia coli*
6689	*Proteobacteria*	*Gammaproteobacte*	*Enterobacterales*	*Enterobacteriaceae*	*Escherichia*	*Escherichia coli*
6690	*Proteobacteria*	*Gammaproteobacte*	*Enterobacterales*	*Enterobacteriaceae*	*Escherichia*	*Escherichia coli*
6691	*Proteobacteria*	*Gammaproteobacte*	*Enterobacterales*	*Enterobacteriaceae*	*Escherichia*	*Escherichia coli*
6692	*Proteobacteria*	*Gammaproteobacte*	*Enterobacterales*	*Enterobacteriaceae*	*Escherichia*	*Escherichia coli*

Table S4. Isolated strains by culturomics

bacteriaId	*phylum*	*class*	*order*	*family*	*genus*	*species*
6693	*Proteobacteria*	*Gammaproteobacte*	*Enterobacterales*	*Enterobacteriaceae*	*Escherichia*	*Escherichia coli*
6694	*Proteobacteria*	*Gammaproteobacte*	*Enterobacterales*	*Enterobacteriaceae*	*Escherichia*	*Escherichia coli*
6695	*Proteobacteria*	*Gammaproteobacte*	*Enterobacterales*	*Enterobacteriaceae*	*Escherichia*	*Escherichia coli*
6696	*Proteobacteria*	*Gammaproteobacte*	*Enterobacterales*	*Enterobacteriaceae*	*Escherichia*	*Escherichia coli*
6697	*Proteobacteria*	*Gammaproteobacte*	*Enterobacterales*	*Enterobacteriaceae*	*Escherichia*	*Escherichia coli*
6698	*Proteobacteria*	*Gammaproteobacte*	*Enterobacterales*	*Enterobacteriaceae*	*Escherichia*	*Escherichia coli*
6699	*Proteobacteria*	*Gammaproteobacte*	*Enterobacterales*	*Enterobacteriaceae*	*Escherichia*	*Escherichia coli*
6700	*Proteobacteria*	*Gammaproteobacte*	*Enterobacterales*	*Enterobacteriaceae*	*Escherichia*	*Escherichia coli*
6701	*Proteobacteria*	*Gammaproteobacte*	*Enterobacterales*	*Enterobacteriaceae*	*Escherichia*	*Escherichia coli*
6702	*Proteobacteria*	*Gammaproteobacte*	*Enterobacterales*	*Enterobacteriaceae*	*Escherichia*	*Escherichia coli*
6703	*Proteobacteria*	*Gammaproteobacte*	*Enterobacterales*	*Enterobacteriaceae*	*Escherichia*	*Escherichia coli*
6704	*Proteobacteria*	*Gammaproteobacte*	*Enterobacterales*	*Enterobacteriaceae*	*Escherichia*	*Escherichia coli*
6705	*Proteobacteria*	*Gammaproteobacte*	*Enterobacterales*	*Enterobacteriaceae*	*Escherichia*	*Escherichia coli*
6706	*Proteobacteria*	*Gammaproteobacte*	*Enterobacterales*	*Enterobacteriaceae*	*Escherichia*	*Escherichia coli*
6707	*Proteobacteria*	*Gammaproteobacte*	*Enterobacterales*	*Enterobacteriaceae*	*Escherichia*	*Escherichia coli*
6708	*Proteobacteria*	*Gammaproteobacte*	*Enterobacterales*	*Enterobacteriaceae*	*Escherichia*	*Escherichia coli*
6709	*Proteobacteria*	*Gammaproteobacte*	*Enterobacterales*	*Enterobacteriaceae*	*Escherichia*	*Escherichia coli*
6710	*Proteobacteria*	*Gammaproteobacte*	*Enterobacterales*	*Enterobacteriaceae*	*Escherichia*	*Escherichia coli*
6711	*Proteobacteria*	*Gammaproteobacte*	*Enterobacterales*	*Enterobacteriaceae*	*Escherichia*	*Escherichia coli*
6712	*Proteobacteria*	*Gammaproteobacte*	*Enterobacterales*	*Enterobacteriaceae*	*Escherichia*	*Escherichia coli*
6713	*Proteobacteria*	*Gammaproteobacte*	*Enterobacterales*	*Enterobacteriaceae*	*Escherichia*	*Escherichia coli*
6714	*Proteobacteria*	*Gammaproteobacte*	*Enterobacterales*	*Enterobacteriaceae*	*Escherichia*	*Escherichia coli*
6715	*Proteobacteria*	*Gammaproteobacte*	*Enterobacterales*	*Enterobacteriaceae*	*Escherichia*	*Escherichia coli*
6716	*Proteobacteria*	*Gammaproteobacte*	*Enterobacterales*	*Enterobacteriaceae*	*Escherichia*	*Escherichia coli*
6717	*Proteobacteria*	*Gammaproteobacte*	*Enterobacterales*	*Enterobacteriaceae*	*Escherichia*	*Escherichia coli*
6718	*Proteobacteria*	*Gammaproteobacte*	*Enterobacterales*	*Enterobacteriaceae*	*Escherichia*	*Escherichia coli*
6719	*Proteobacteria*	*Gammaproteobacte*	*Enterobacterales*	*Enterobacteriaceae*	*Escherichia*	*Escherichia coli*
6720	*Proteobacteria*	*Gammaproteobacte*	*Enterobacterales*	*Enterobacteriaceae*	*Escherichia*	*Escherichia coli*

Table S4. Isolated strains by culturomics

bacteriaId	phylum	class	order	family	genus	species
6721	*Proteobacteria*	*Gammaproteobacte*	*Enterobacterales*	*Enterobacteriaceae*	*Escherichia*	*Escherichia coli*
6722	*Proteobacteria*	*Gammaproteobacte*	*Enterobacterales*	*Enterobacteriaceae*	*Escherichia*	*Escherichia coli*
6723	*Proteobacteria*	*Gammaproteobacte*	*Enterobacterales*	*Enterobacteriaceae*	*Escherichia*	*Escherichia coli*
6724	*Proteobacteria*	*Gammaproteobacte*	*Enterobacterales*	*Enterobacteriaceae*	*Escherichia*	*Escherichia coli*
6725	*Proteobacteria*	*Gammaproteobacte*	*Enterobacterales*	*Enterobacteriaceae*	*Escherichia*	*Escherichia coli*
6726	*Proteobacteria*	*Gammaproteobacte*	*Enterobacterales*	*Enterobacteriaceae*	*Escherichia*	*Escherichia coli*
6727	*Proteobacteria*	*Gammaproteobacte*	*Enterobacterales*	*Enterobacteriaceae*	*Escherichia*	*Escherichia coli*
6728	*Proteobacteria*	*Gammaproteobacte*	*Enterobacterales*	*Enterobacteriaceae*	*Escherichia*	*Escherichia coli*
6729	*Proteobacteria*	*Gammaproteobacte*	*Enterobacterales*	*Enterobacteriaceae*	*Escherichia*	*Escherichia coli*
6730	*Proteobacteria*	*Gammaproteobacte*	*Enterobacterales*	*Enterobacteriaceae*	*Escherichia*	*Escherichia coli*
6731	*Proteobacteria*	*Gammaproteobacte*	*Enterobacterales*	*Enterobacteriaceae*	*Escherichia*	*Escherichia coli*
6732	*Proteobacteria*	*Gammaproteobacte*	*Enterobacterales*	*Enterobacteriaceae*	*Escherichia*	*Escherichia coli*
6733	*Proteobacteria*	*Gammaproteobacte*	*Enterobacterales*	*Enterobacteriaceae*	*Escherichia*	*Escherichia coli*
6734	*Proteobacteria*	*Gammaproteobacte*	*Enterobacterales*	*Enterobacteriaceae*	*Escherichia*	*Escherichia coli*
6735	*Proteobacteria*	*Gammaproteobacte*	*Enterobacterales*	*Enterobacteriaceae*	*Escherichia*	*Escherichia coli*
6736	*Proteobacteria*	*Gammaproteobacte*	*Enterobacterales*	*Enterobacteriaceae*	*Escherichia*	*Escherichia coli*
6737	*Proteobacteria*	*Gammaproteobacte*	*Enterobacterales*	*Enterobacteriaceae*	*Escherichia*	*Escherichia coli*
6738	*Proteobacteria*	*Gammaproteobacte*	*Enterobacterales*	*Enterobacteriaceae*	*Escherichia*	*Escherichia coli*
6739	*Proteobacteria*	*Gammaproteobacte*	*Enterobacterales*	*Enterobacteriaceae*	*Escherichia*	*Escherichia coli*
6740	*Proteobacteria*	*Gammaproteobacte*	*Enterobacterales*	*Enterobacteriaceae*	*Escherichia*	*Escherichia coli*
6741	*Proteobacteria*	*Gammaproteobacte*	*Enterobacterales*	*Enterobacteriaceae*	*Escherichia*	*Escherichia coli*
6742	*Proteobacteria*	*Gammaproteobacte*	*Enterobacterales*	*Enterobacteriaceae*	*Escherichia*	*Escherichia coli*
6743	*Proteobacteria*	*Gammaproteobacte*	*Enterobacterales*	*Enterobacteriaceae*	*Escherichia*	*Escherichia coli*
6744	*Proteobacteria*	*Gammaproteobacte*	*Enterobacterales*	*Enterobacteriaceae*	*Escherichia*	*Escherichia coli*
6745	*Proteobacteria*	*Gammaproteobacte*	*Enterobacterales*	*Enterobacteriaceae*	*Escherichia*	*Escherichia coli*
6746	*Proteobacteria*	*Gammaproteobacte*	*Enterobacterales*	*Enterobacteriaceae*	*Escherichia*	*Escherichia coli*
6747	*Proteobacteria*	*Gammaproteobacte*	*Enterobacterales*	*Enterobacteriaceae*	*Escherichia*	*Escherichia coli*
6748	*Proteobacteria*	*Gammaproteobacte*	*Enterobacterales*	*Enterobacteriaceae*	*Escherichia*	*Escherichia coli*

Table S4. Isolated strains by culturomics

bacteriaId	*phylum*	*class*	*order*	*family*	*genus*	*species*
6749	*Proteobacteria*	*Gammaproteobacte*	*Enterobacterales*	*Enterobacteriaceae*	*Escherichia*	*Escherichia coli*
6750	*Proteobacteria*	*Gammaproteobacte*	*Enterobacterales*	*Enterobacteriaceae*	*Escherichia*	*Escherichia coli*
6751	*Proteobacteria*	*Gammaproteobacte*	*Enterobacterales*	*Enterobacteriaceae*	*Escherichia*	*Escherichia coli*
6752	*Proteobacteria*	*Gammaproteobacte*	*Enterobacterales*	*Enterobacteriaceae*	*Escherichia*	*Escherichia coli*
6753	*Proteobacteria*	*Gammaproteobacte*	*Enterobacterales*	*Enterobacteriaceae*	*Escherichia*	*Escherichia coli*
6754	*Proteobacteria*	*Gammaproteobacte*	*Enterobacterales*	*Enterobacteriaceae*	*Escherichia*	*Escherichia coli*
6755	*Proteobacteria*	*Gammaproteobacte*	*Enterobacterales*	*Enterobacteriaceae*	*Escherichia*	*Escherichia coli*
6756	*Proteobacteria*	*Gammaproteobacte*	*Enterobacterales*	*Enterobacteriaceae*	*Escherichia*	*Escherichia coli*
6757	*Proteobacteria*	*Gammaproteobacte*	*Enterobacterales*	*Enterobacteriaceae*	*Escherichia*	*Escherichia coli*
6758	*Proteobacteria*	*Gammaproteobacte*	*Enterobacterales*	*Enterobacteriaceae*	*Escherichia*	*Escherichia coli*
6759	*Proteobacteria*	*Gammaproteobacte*	*Enterobacterales*	*Enterobacteriaceae*	*Escherichia*	*Escherichia coli*
6760	*Proteobacteria*	*Gammaproteobacte*	*Enterobacterales*	*Enterobacteriaceae*	*Escherichia*	*Escherichia coli*
6761	*Proteobacteria*	*Gammaproteobacte*	*Enterobacterales*	*Enterobacteriaceae*	*Escherichia*	*Escherichia coli*
6762	*Proteobacteria*	*Gammaproteobacte*	*Enterobacterales*	*Enterobacteriaceae*	*Escherichia*	*Escherichia coli*
6763	*Proteobacteria*	*Gammaproteobacte*	*Enterobacterales*	*Enterobacteriaceae*	*Escherichia*	*Escherichia coli*
6764	*Proteobacteria*	*Gammaproteobacte*	*Enterobacterales*	*Enterobacteriaceae*	*Escherichia*	*Escherichia coli*
6765	*Proteobacteria*	*Gammaproteobacte*	*Enterobacterales*	*Enterobacteriaceae*	*Escherichia*	*Escherichia coli*
6766	*Proteobacteria*	*Gammaproteobacte*	*Enterobacterales*	*Enterobacteriaceae*	*Escherichia*	*Escherichia coli*
6767	*Proteobacteria*	*Gammaproteobacte*	*Enterobacterales*	*Enterobacteriaceae*	*Escherichia*	*Escherichia coli*
6768	*Proteobacteria*	*Gammaproteobacte*	*Enterobacterales*	*Enterobacteriaceae*	*Escherichia*	*Escherichia coli*
6769	*Proteobacteria*	*Gammaproteobacte*	*Enterobacterales*	*Enterobacteriaceae*	*Escherichia*	*Escherichia coli*
6770	*Proteobacteria*	*Gammaproteobacte*	*Enterobacterales*	*Enterobacteriaceae*	*Escherichia*	*Escherichia coli*
6771	*Proteobacteria*	*Gammaproteobacte*	*Enterobacterales*	*Enterobacteriaceae*	*Escherichia*	*Escherichia coli*
6772	*Proteobacteria*	*Gammaproteobacte*	*Enterobacterales*	*Enterobacteriaceae*	*Escherichia*	*Escherichia coli*
6773	*Proteobacteria*	*Gammaproteobacte*	*Enterobacterales*	*Enterobacteriaceae*	*Escherichia*	*Escherichia coli*
6774	*Proteobacteria*	*Gammaproteobacte*	*Enterobacterales*	*Enterobacteriaceae*	*Escherichia*	*Escherichia coli*
6775	*Proteobacteria*	*Gammaproteobacte*	*Enterobacterales*	*Enterobacteriaceae*	*Escherichia*	*Escherichia coli*
6776	*Proteobacteria*	*Gammaproteobacte*	*Enterobacterales*	*Enterobacteriaceae*	*Escherichia*	*Escherichia coli*

Table S4. Isolated strains by culturomics

bacteriaId	*phylum*	*class*	*order*	*family*	*genus*	*species*
6777	*Proteobacteria*	*Gammaproteobacte*	*Enterobacterales*	*Enterobacteriaceae*	*Escherichia*	*Escherichia coli*
6778	*Proteobacteria*	*Gammaproteobacte*	*Enterobacterales*	*Enterobacteriaceae*	*Escherichia*	*Escherichia coli*
6779	*Proteobacteria*	*Gammaproteobacte*	*Enterobacterales*	*Enterobacteriaceae*	*Escherichia*	*Escherichia coli*
6780	*Proteobacteria*	*Gammaproteobacte*	*Enterobacterales*	*Enterobacteriaceae*	*Escherichia*	*Escherichia coli*
6781	*Proteobacteria*	*Gammaproteobacte*	*Enterobacterales*	*Enterobacteriaceae*	*Escherichia*	*Escherichia coli*
6782	*Proteobacteria*	*Gammaproteobacte*	*Enterobacterales*	*Enterobacteriaceae*	*Escherichia*	*Escherichia coli*
6783	*Proteobacteria*	*Gammaproteobacte*	*Enterobacterales*	*Enterobacteriaceae*	*Escherichia*	*Escherichia coli*
6784	*Proteobacteria*	*Gammaproteobacte*	*Enterobacterales*	*Enterobacteriaceae*	*Escherichia*	*Escherichia coli*
6785	*Proteobacteria*	*Gammaproteobacte*	*Enterobacterales*	*Enterobacteriaceae*	*Escherichia*	*Escherichia coli*
6786	*Proteobacteria*	*Gammaproteobacte*	*Enterobacterales*	*Enterobacteriaceae*	*Escherichia*	*Escherichia coli*
6787	*Proteobacteria*	*Gammaproteobacte*	*Enterobacterales*	*Enterobacteriaceae*	*Escherichia*	*Escherichia coli*
6788	*Proteobacteria*	*Gammaproteobacte*	*Enterobacterales*	*Enterobacteriaceae*	*Escherichia*	*Escherichia coli*
6789	*Proteobacteria*	*Gammaproteobacte*	*Enterobacterales*	*Enterobacteriaceae*	*Escherichia*	*Escherichia coli*
6790	*Proteobacteria*	*Gammaproteobacte*	*Enterobacterales*	*Enterobacteriaceae*	*Escherichia*	*Escherichia coli*
6791	*Proteobacteria*	*Gammaproteobacte*	*Enterobacterales*	*Enterobacteriaceae*	*Escherichia*	*Escherichia coli*
6792	*Proteobacteria*	*Gammaproteobacte*	*Enterobacterales*	*Enterobacteriaceae*	*Escherichia*	*Escherichia coli*
6793	*Proteobacteria*	*Gammaproteobacte*	*Enterobacterales*	*Enterobacteriaceae*	*Escherichia*	*Escherichia coli*
6794	*Proteobacteria*	*Gammaproteobacte*	*Enterobacterales*	*Enterobacteriaceae*	*Escherichia*	*Escherichia coli*
6795	*Proteobacteria*	*Gammaproteobacte*	*Enterobacterales*	*Enterobacteriaceae*	*Escherichia*	*Escherichia coli*
6796	*Proteobacteria*	*Gammaproteobacte*	*Enterobacterales*	*Enterobacteriaceae*	*Escherichia*	*Escherichia coli*
6797	*Proteobacteria*	*Gammaproteobacte*	*Enterobacterales*	*Enterobacteriaceae*	*Escherichia*	*Escherichia coli*
6798	*Proteobacteria*	*Gammaproteobacte*	*Enterobacterales*	*Enterobacteriaceae*	*Escherichia*	*Escherichia coli*
6799	*Proteobacteria*	*Gammaproteobacte*	*Enterobacterales*	*Enterobacteriaceae*	*Escherichia*	*Escherichia coli*
6800	*Proteobacteria*	*Gammaproteobacte*	*Enterobacterales*	*Enterobacteriaceae*	*Escherichia*	*Escherichia coli*
6801	*Proteobacteria*	*Gammaproteobacte*	*Enterobacterales*	*Enterobacteriaceae*	*Escherichia*	*Escherichia coli*
6802	*Proteobacteria*	*Gammaproteobacte*	*Enterobacterales*	*Enterobacteriaceae*	*Escherichia*	*Escherichia coli*
6803	*Proteobacteria*	*Gammaproteobacte*	*Enterobacterales*	*Enterobacteriaceae*	*Escherichia*	*Escherichia coli*
6804	*Proteobacteria*	*Gammaproteobacte*	*Enterobacterales*	*Enterobacteriaceae*	*Escherichia*	*Escherichia coli*

Table S4. Isolated strains by culturomics

bacteriaId	*phylum*	*class*	*order*	*family*	*genus*	*species*
6805	*Proteobacteria*	*Gammaproteobacte*	*Enterobacterales*	*Enterobacteriaceae*	*Escherichia*	*Escherichia coli*
6806	*Proteobacteria*	*Gammaproteobacte*	*Enterobacterales*	*Enterobacteriaceae*	*Escherichia*	*Escherichia coli*
6807	*Proteobacteria*	*Gammaproteobacte*	*Enterobacterales*	*Enterobacteriaceae*	*Escherichia*	*Escherichia coli*
6808	*Proteobacteria*	*Gammaproteobacte*	*Enterobacterales*	*Enterobacteriaceae*	*Escherichia*	*Escherichia coli*
6809	*Proteobacteria*	*Gammaproteobacte*	*Enterobacterales*	*Enterobacteriaceae*	*Escherichia*	*Escherichia coli*
6810	*Proteobacteria*	*Gammaproteobacte*	*Enterobacterales*	*Enterobacteriaceae*	*Escherichia*	*Escherichia coli*
6811	*Proteobacteria*	*Gammaproteobacte*	*Enterobacterales*	*Enterobacteriaceae*	*Escherichia*	*Escherichia coli*
6812	*Proteobacteria*	*Gammaproteobacte*	*Enterobacterales*	*Enterobacteriaceae*	*Escherichia*	*Escherichia coli*
6813	*Proteobacteria*	*Gammaproteobacte*	*Enterobacterales*	*Enterobacteriaceae*	*Escherichia*	*Escherichia coli*
6814	*Proteobacteria*	*Gammaproteobacte*	*Enterobacterales*	*Enterobacteriaceae*	*Escherichia*	*Escherichia coli*
6815	*Proteobacteria*	*Gammaproteobacte*	*Enterobacterales*	*Enterobacteriaceae*	*Escherichia*	*Escherichia coli*
6816	*Proteobacteria*	*Gammaproteobacte*	*Enterobacterales*	*Enterobacteriaceae*	*Escherichia*	*Escherichia coli*
6817	*Proteobacteria*	*Gammaproteobacte*	*Enterobacterales*	*Enterobacteriaceae*	*Escherichia*	*Escherichia coli*
6818	*Proteobacteria*	*Gammaproteobacte*	*Enterobacterales*	*Enterobacteriaceae*	*Escherichia*	*Escherichia coli*
6819	*Proteobacteria*	*Gammaproteobacte*	*Enterobacterales*	*Enterobacteriaceae*	*Escherichia*	*Escherichia coli*
6820	*Proteobacteria*	*Gammaproteobacte*	*Enterobacterales*	*Enterobacteriaceae*	*Escherichia*	*Escherichia coli*
6821	*Proteobacteria*	*Gammaproteobacte*	*Enterobacterales*	*Enterobacteriaceae*	*Escherichia*	*Escherichia coli*
6822	*Proteobacteria*	*Gammaproteobacte*	*Enterobacterales*	*Enterobacteriaceae*	*Escherichia*	*Escherichia coli*
6823	*Proteobacteria*	*Gammaproteobacte*	*Enterobacterales*	*Enterobacteriaceae*	*Escherichia*	*Escherichia coli*
6824	*Proteobacteria*	*Gammaproteobacte*	*Enterobacterales*	*Enterobacteriaceae*	*Escherichia*	*Escherichia coli*
6825	*Proteobacteria*	*Gammaproteobacte*	*Enterobacterales*	*Enterobacteriaceae*	*Escherichia*	*Escherichia coli*
6826	*Proteobacteria*	*Gammaproteobacte*	*Enterobacterales*	*Enterobacteriaceae*	*Escherichia*	*Escherichia coli*
6827	*Proteobacteria*	*Gammaproteobacte*	*Enterobacterales*	*Enterobacteriaceae*	*Escherichia*	*Escherichia coli*
6828	*Proteobacteria*	*Gammaproteobacte*	*Enterobacterales*	*Enterobacteriaceae*	*Escherichia*	*Escherichia coli*
6829	*Proteobacteria*	*Gammaproteobacte*	*Enterobacterales*	*Enterobacteriaceae*	*Escherichia*	*Escherichia coli*
6830	*Proteobacteria*	*Gammaproteobacte*	*Enterobacterales*	*Enterobacteriaceae*	*Escherichia*	*Escherichia coli*
6831	*Proteobacteria*	*Gammaproteobacte*	*Enterobacterales*	*Enterobacteriaceae*	*Escherichia*	*Escherichia coli*
6832	*Proteobacteria*	*Gammaproteobacte*	*Enterobacterales*	*Enterobacteriaceae*	*Escherichia*	*Escherichia coli*

Table S4. Isolated strains by culturomics

bacterialId	*phylum*	*class*	*order*	*family*	*genus*	*species*
6833	*Proteobacteria*	*Gammaproteobacte*	*Enterobacterales*	*Enterobacteriaceae*	*Escherichia*	*Escherichia coli*
6834	*Proteobacteria*	*Gammaproteobacte*	*Enterobacterales*	*Enterobacteriaceae*	*Escherichia*	*Escherichia coli*
6835	*Proteobacteria*	*Gammaproteobacte*	*Enterobacterales*	*Enterobacteriaceae*	*Escherichia*	*Escherichia coli*
6836	*Proteobacteria*	*Gammaproteobacte*	*Enterobacterales*	*Enterobacteriaceae*	*Escherichia*	*Escherichia coli*
6837	*Proteobacteria*	*Gammaproteobacte*	*Enterobacterales*	*Enterobacteriaceae*	*Escherichia*	*Escherichia coli*
6838	*Proteobacteria*	*Gammaproteobacte*	*Enterobacterales*	*Enterobacteriaceae*	*Escherichia*	*Escherichia coli*
6839	*Proteobacteria*	*Gammaproteobacte*	*Enterobacterales*	*Enterobacteriaceae*	*Escherichia*	*Escherichia coli*
6840	*Proteobacteria*	*Gammaproteobacte*	*Enterobacterales*	*Enterobacteriaceae*	*Escherichia*	*Escherichia coli*
6841	*Proteobacteria*	*Gammaproteobacte*	*Enterobacterales*	*Enterobacteriaceae*	*Escherichia*	*Escherichia coli*
6842	*Proteobacteria*	*Gammaproteobacte*	*Enterobacterales*	*Enterobacteriaceae*	*Escherichia*	*Escherichia coli*
6843	*Proteobacteria*	*Gammaproteobacte*	*Enterobacterales*	*Enterobacteriaceae*	*Escherichia*	*Escherichia coli*
6844	*Proteobacteria*	*Gammaproteobacte*	*Enterobacterales*	*Enterobacteriaceae*	*Escherichia*	*Escherichia coli*
6845	*Proteobacteria*	*Gammaproteobacte*	*Enterobacterales*	*Enterobacteriaceae*	*Escherichia*	*Escherichia coli*
6846	*Proteobacteria*	*Gammaproteobacte*	*Enterobacterales*	*Enterobacteriaceae*	*Escherichia*	*Escherichia coli*
6847	*Proteobacteria*	*Gammaproteobacte*	*Enterobacterales*	*Enterobacteriaceae*	*Escherichia*	*Escherichia coli*
6848	*Proteobacteria*	*Gammaproteobacte*	*Enterobacterales*	*Enterobacteriaceae*	*Escherichia*	*Escherichia coli*
6849	*Proteobacteria*	*Gammaproteobacte*	*Enterobacterales*	*Enterobacteriaceae*	*Escherichia*	*Escherichia coli*
6850	*Proteobacteria*	*Gammaproteobacte*	*Enterobacterales*	*Enterobacteriaceae*	*Escherichia*	*Escherichia coli*
6851	*Proteobacteria*	*Gammaproteobacte*	*Enterobacterales*	*Enterobacteriaceae*	*Escherichia*	*Escherichia coli*
6852	*Proteobacteria*	*Gammaproteobacte*	*Enterobacterales*	*Enterobacteriaceae*	*Escherichia*	*Escherichia coli*
6853	*Proteobacteria*	*Gammaproteobacte*	*Enterobacterales*	*Enterobacteriaceae*	*Escherichia*	*Escherichia coli*
6854	*Proteobacteria*	*Gammaproteobacte*	*Enterobacterales*	*Enterobacteriaceae*	*Escherichia*	*Escherichia coli*
6855	*Proteobacteria*	*Gammaproteobacte*	*Enterobacterales*	*Enterobacteriaceae*	*Escherichia*	*Escherichia coli*
6856	*Proteobacteria*	*Gammaproteobacte*	*Enterobacterales*	*Enterobacteriaceae*	*Escherichia*	*Escherichia coli*
6857	*Proteobacteria*	*Gammaproteobacte*	*Enterobacterales*	*Enterobacteriaceae*	*Escherichia*	*Escherichia coli*
6858	*Proteobacteria*	*Gammaproteobacte*	*Enterobacterales*	*Enterobacteriaceae*	*Escherichia*	*Escherichia coli*
6859	*Proteobacteria*	*Gammaproteobacte*	*Enterobacterales*	*Enterobacteriaceae*	*Escherichia*	*Escherichia coli*
6860	*Proteobacteria*	*Gammaproteobacte*	*Enterobacterales*	*Enterobacteriaceae*	*Escherichia*	*Escherichia coli*

Table S4. Isolated strains by culturomics

bacteriaId	*phylum*	*class*	*order*	*family*	*genus*	*species*
6861	*Proteobacteria*	*Gammaproteobacte*	*Enterobacterales*	*Enterobacteriaceae*	*Escherichia*	*Escherichia coli*
6862	*Proteobacteria*	*Gammaproteobacte*	*Enterobacterales*	*Enterobacteriaceae*	*Escherichia*	*Escherichia coli*
6863	*Proteobacteria*	*Gammaproteobacte*	*Enterobacterales*	*Enterobacteriaceae*	*Escherichia*	*Escherichia coli*
6864	*Proteobacteria*	*Gammaproteobacte*	*Enterobacterales*	*Enterobacteriaceae*	*Escherichia*	*Escherichia coli*
6865	*Proteobacteria*	*Gammaproteobacte*	*Enterobacterales*	*Enterobacteriaceae*	*Escherichia*	*Escherichia coli*
6866	*Proteobacteria*	*Gammaproteobacte*	*Enterobacterales*	*Enterobacteriaceae*	*Escherichia*	*Escherichia coli*
6867	*Proteobacteria*	*Gammaproteobacte*	*Enterobacterales*	*Enterobacteriaceae*	*Escherichia*	*Escherichia coli*
6868	*Proteobacteria*	*Gammaproteobacte*	*Enterobacterales*	*Enterobacteriaceae*	*Escherichia*	*Escherichia coli*
6869	*Proteobacteria*	*Gammaproteobacte*	*Enterobacterales*	*Enterobacteriaceae*	*Escherichia*	*Escherichia coli*
6870	*Proteobacteria*	*Gammaproteobacte*	*Enterobacterales*	*Enterobacteriaceae*	*Escherichia*	*Escherichia coli*
6871	*Proteobacteria*	*Gammaproteobacte*	*Enterobacterales*	*Enterobacteriaceae*	*Escherichia*	*Escherichia coli*
6872	*Proteobacteria*	*Gammaproteobacte*	*Enterobacterales*	*Enterobacteriaceae*	*Escherichia*	*Escherichia coli*
6873	*Proteobacteria*	*Gammaproteobacte*	*Enterobacterales*	*Enterobacteriaceae*	*Escherichia*	*Escherichia coli*
6874	*Proteobacteria*	*Gammaproteobacte*	*Enterobacterales*	*Enterobacteriaceae*	*Escherichia*	*Escherichia coli*
6875	*Proteobacteria*	*Gammaproteobacte*	*Enterobacterales*	*Enterobacteriaceae*	*Escherichia*	*Escherichia coli*
6876	*Proteobacteria*	*Gammaproteobacte*	*Enterobacterales*	*Enterobacteriaceae*	*Escherichia*	*Escherichia coli*
6877	*Proteobacteria*	*Gammaproteobacte*	*Enterobacterales*	*Enterobacteriaceae*	*Escherichia*	*Escherichia coli*
6878	*Proteobacteria*	*Gammaproteobacte*	*Enterobacterales*	*Enterobacteriaceae*	*Escherichia*	*Escherichia coli*
6879	*Proteobacteria*	*Gammaproteobacte*	*Enterobacterales*	*Enterobacteriaceae*	*Escherichia*	*Escherichia coli*
6880	*Proteobacteria*	*Gammaproteobacte*	*Enterobacterales*	*Enterobacteriaceae*	*Escherichia*	*Escherichia coli*
6881	*Proteobacteria*	*Gammaproteobacte*	*Enterobacterales*	*Enterobacteriaceae*	*Escherichia*	*Escherichia coli*
6882	*Proteobacteria*	*Gammaproteobacte*	*Enterobacterales*	*Enterobacteriaceae*	*Escherichia*	*Escherichia coli*
6883	*Proteobacteria*	*Gammaproteobacte*	*Enterobacterales*	*Enterobacteriaceae*	*Escherichia*	*Escherichia coli*
6884	*Proteobacteria*	*Gammaproteobacte*	*Enterobacterales*	*Enterobacteriaceae*	*Escherichia*	*Escherichia coli*
6885	*Proteobacteria*	*Gammaproteobacte*	*Enterobacterales*	*Enterobacteriaceae*	*Escherichia*	*Escherichia coli*
6886	*Proteobacteria*	*Gammaproteobacte*	*Enterobacterales*	*Enterobacteriaceae*	*Escherichia*	*Escherichia coli*
6887	*Proteobacteria*	*Gammaproteobacte*	*Enterobacterales*	*Enterobacteriaceae*	*Escherichia*	*Escherichia coli*
6888	*Proteobacteria*	*Gammaproteobacte*	*Enterobacterales*	*Enterobacteriaceae*	*Escherichia*	*Escherichia coli*

Table S4. Isolated strains by culturomics

bacteriaId	*phylum*	*class*	*order*	*family*	*genus*	*species*
6889	*Proteobacteria*	*Gammaproteobacte*	*Enterobacterales*	*Enterobacteriaceae*	*Escherichia*	*Escherichia coli*
6890	*Proteobacteria*	*Gammaproteobacte*	*Enterobacterales*	*Enterobacteriaceae*	*Escherichia*	*Escherichia coli*
6891	*Proteobacteria*	*Gammaproteobacte*	*Enterobacterales*	*Enterobacteriaceae*	*Escherichia*	*Escherichia coli*
6892	*Proteobacteria*	*Gammaproteobacte*	*Enterobacterales*	*Enterobacteriaceae*	*Escherichia*	*Escherichia coli*
6893	*Proteobacteria*	*Gammaproteobacte*	*Enterobacterales*	*Enterobacteriaceae*	*Escherichia*	*Escherichia coli*
6894	*Proteobacteria*	*Gammaproteobacte*	*Enterobacterales*	*Enterobacteriaceae*	*Escherichia*	*Escherichia coli*
6895	*Proteobacteria*	*Gammaproteobacte*	*Enterobacterales*	*Enterobacteriaceae*	*Escherichia*	*Escherichia coli*
6896	*Proteobacteria*	*Gammaproteobacte*	*Enterobacterales*	*Enterobacteriaceae*	*Escherichia*	*Escherichia coli*
6897	*Proteobacteria*	*Gammaproteobacte*	*Enterobacterales*	*Enterobacteriaceae*	*Escherichia*	*Escherichia coli*
6898	*Proteobacteria*	*Gammaproteobacte*	*Enterobacterales*	*Enterobacteriaceae*	*Escherichia*	*Escherichia coli*
6899	*Proteobacteria*	*Gammaproteobacte*	*Enterobacterales*	*Enterobacteriaceae*	*Escherichia*	*Escherichia coli*
6900	*Proteobacteria*	*Gammaproteobacte*	*Enterobacterales*	*Enterobacteriaceae*	*Escherichia*	*Escherichia coli*
6901	*Proteobacteria*	*Gammaproteobacte*	*Enterobacterales*	*Enterobacteriaceae*	*Escherichia*	*Escherichia coli*
6902	*Proteobacteria*	*Gammaproteobacte*	*Enterobacterales*	*Enterobacteriaceae*	*Escherichia*	*Escherichia coli*
6903	*Proteobacteria*	*Gammaproteobacte*	*Enterobacterales*	*Enterobacteriaceae*	*Escherichia*	*Escherichia coli*
6904	*Proteobacteria*	*Gammaproteobacte*	*Enterobacterales*	*Enterobacteriaceae*	*Escherichia*	*Escherichia coli*
6905	*Proteobacteria*	*Gammaproteobacte*	*Enterobacterales*	*Enterobacteriaceae*	*Escherichia*	*Escherichia coli*
6906	*Proteobacteria*	*Gammaproteobacte*	*Enterobacterales*	*Enterobacteriaceae*	*Escherichia*	*Escherichia coli*
6907	*Proteobacteria*	*Gammaproteobacte*	*Enterobacterales*	*Enterobacteriaceae*	*Escherichia*	*Escherichia coli*
6908	*Proteobacteria*	*Gammaproteobacte*	*Enterobacterales*	*Enterobacteriaceae*	*Escherichia*	*Escherichia coli*
6909	*Proteobacteria*	*Gammaproteobacte*	*Enterobacterales*	*Enterobacteriaceae*	*Escherichia*	*Escherichia coli*
6910	*Proteobacteria*	*Gammaproteobacte*	*Enterobacterales*	*Enterobacteriaceae*	*Escherichia*	*Escherichia coli*
6911	*Proteobacteria*	*Gammaproteobacte*	*Enterobacterales*	*Enterobacteriaceae*	*Escherichia*	*Escherichia coli*
6912	*Proteobacteria*	*Gammaproteobacte*	*Enterobacterales*	*Enterobacteriaceae*	*Escherichia*	*Escherichia coli*
6913	*Proteobacteria*	*Gammaproteobacte*	*Enterobacterales*	*Enterobacteriaceae*	*Escherichia*	*Escherichia coli*
6914	*Proteobacteria*	*Gammaproteobacte*	*Enterobacterales*	*Enterobacteriaceae*	*Escherichia*	*Escherichia coli*
6915	*Proteobacteria*	*Gammaproteobacte*	*Enterobacterales*	*Enterobacteriaceae*	*Escherichia*	*Escherichia coli*
6916	*Proteobacteria*	*Gammaproteobacte*	*Enterobacterales*	*Enterobacteriaceae*	*Escherichia*	*Escherichia coli*

Table S4. Isolated strains by culturomics

bacteriaId	*phylum*	*class*	*order*	*family*	*genus*	*species*
6917	*Proteobacteria*	*Gammaproteobacte*	*Enterobacterales*	*Enterobacteriaceae*	*Escherichia*	*Escherichia coli*
6918	*Proteobacteria*	*Gammaproteobacte*	*Enterobacterales*	*Enterobacteriaceae*	*Escherichia*	*Escherichia coli*
6919	*Proteobacteria*	*Gammaproteobacte*	*Enterobacterales*	*Enterobacteriaceae*	*Escherichia*	*Escherichia coli*
6920	*Proteobacteria*	*Gammaproteobacte*	*Enterobacterales*	*Enterobacteriaceae*	*Escherichia*	*Escherichia coli*
6921	*Proteobacteria*	*Gammaproteobacte*	*Enterobacterales*	*Enterobacteriaceae*	*Escherichia*	*Escherichia coli*
6922	*Proteobacteria*	*Gammaproteobacte*	*Enterobacterales*	*Enterobacteriaceae*	*Escherichia*	*Escherichia coli*
6923	*Proteobacteria*	*Gammaproteobacte*	*Enterobacterales*	*Enterobacteriaceae*	*Escherichia*	*Escherichia coli*
6924	*Proteobacteria*	*Gammaproteobacte*	*Enterobacterales*	*Enterobacteriaceae*	*Escherichia*	*Escherichia coli*
6925	*Proteobacteria*	*Gammaproteobacte*	*Enterobacterales*	*Enterobacteriaceae*	*Escherichia*	*Escherichia coli*
6926	*Proteobacteria*	*Gammaproteobacte*	*Enterobacterales*	*Enterobacteriaceae*	*Escherichia*	*Escherichia coli*
6927	*Proteobacteria*	*Gammaproteobacte*	*Enterobacterales*	*Enterobacteriaceae*	*Escherichia*	*Escherichia coli*
6928	*Proteobacteria*	*Gammaproteobacte*	*Enterobacterales*	*Enterobacteriaceae*	*Escherichia*	*Escherichia coli*
6929	*Proteobacteria*	*Gammaproteobacte*	*Enterobacterales*	*Enterobacteriaceae*	*Escherichia*	*Escherichia coli*
6930	*Proteobacteria*	*Gammaproteobacte*	*Enterobacterales*	*Enterobacteriaceae*	*Escherichia*	*Escherichia coli*
6931	*Proteobacteria*	*Gammaproteobacte*	*Enterobacterales*	*Enterobacteriaceae*	*Escherichia*	*Escherichia coli*
6932	*Proteobacteria*	*Gammaproteobacte*	*Enterobacterales*	*Enterobacteriaceae*	*Escherichia*	*Escherichia coli*
6933	*Proteobacteria*	*Gammaproteobacte*	*Enterobacterales*	*Enterobacteriaceae*	*Escherichia*	*Escherichia coli*
6934	*Proteobacteria*	*Gammaproteobacte*	*Enterobacterales*	*Enterobacteriaceae*	*Escherichia*	*Escherichia coli*
6935	*Proteobacteria*	*Gammaproteobacte*	*Enterobacterales*	*Enterobacteriaceae*	*Escherichia*	*Escherichia coli*
6936	*Proteobacteria*	*Gammaproteobacte*	*Enterobacterales*	*Enterobacteriaceae*	*Escherichia*	*Escherichia coli*
6937	*Proteobacteria*	*Gammaproteobacte*	*Enterobacterales*	*Enterobacteriaceae*	*Escherichia*	*Escherichia coli*
6938	*Proteobacteria*	*Gammaproteobacte*	*Enterobacterales*	*Enterobacteriaceae*	*Escherichia*	*Escherichia coli*
6939	*Proteobacteria*	*Gammaproteobacte*	*Enterobacterales*	*Enterobacteriaceae*	*Escherichia*	*Escherichia coli*
6940	*Proteobacteria*	*Gammaproteobacte*	*Enterobacterales*	*Enterobacteriaceae*	*Escherichia*	*Escherichia coli*
6941	*Proteobacteria*	*Gammaproteobacte*	*Enterobacterales*	*Enterobacteriaceae*	*Escherichia*	*Escherichia coli*
6942	*Proteobacteria*	*Gammaproteobacte*	*Enterobacterales*	*Enterobacteriaceae*	*Escherichia*	*Escherichia coli*
6943	*Proteobacteria*	*Gammaproteobacte*	*Enterobacterales*	*Enterobacteriaceae*	*Escherichia*	*Escherichia coli*
6944	*Proteobacteria*	*Gammaproteobacte*	*Enterobacterales*	*Enterobacteriaceae*	*Escherichia*	*Escherichia coli*

Table S4. Isolated strains by culturomics

bacteriaId	*phylum*	*class*	*order*	*family*	*genus*	*species*
6945	*Proteobacteria*	*Gammaproteobacte*	*Enterobacterales*	*Enterobacteriaceae*	*Escherichia*	*Escherichia coli*
6946	*Proteobacteria*	*Gammaproteobacte*	*Enterobacterales*	*Enterobacteriaceae*	*Escherichia*	*Escherichia coli*
6947	*Proteobacteria*	*Gammaproteobacte*	*Enterobacterales*	*Enterobacteriaceae*	*Escherichia*	*Escherichia coli*
6948	*Proteobacteria*	*Gammaproteobacte*	*Enterobacterales*	*Enterobacteriaceae*	*Escherichia*	*Escherichia coli*
6949	*Proteobacteria*	*Gammaproteobacte*	*Enterobacterales*	*Enterobacteriaceae*	*Escherichia*	*Escherichia coli*
6950	*Proteobacteria*	*Gammaproteobacte*	*Enterobacterales*	*Enterobacteriaceae*	*Escherichia*	*Escherichia coli*
6951	*Proteobacteria*	*Gammaproteobacte*	*Enterobacterales*	*Enterobacteriaceae*	*Escherichia*	*Escherichia coli*
6952	*Proteobacteria*	*Gammaproteobacte*	*Enterobacterales*	*Enterobacteriaceae*	*Escherichia*	*Escherichia coli*
6953	*Proteobacteria*	*Gammaproteobacte*	*Enterobacterales*	*Enterobacteriaceae*	*Escherichia*	*Escherichia coli*
6954	*Proteobacteria*	*Gammaproteobacte*	*Enterobacterales*	*Enterobacteriaceae*	*Escherichia*	*Escherichia coli*
6955	*Proteobacteria*	*Gammaproteobacte*	*Enterobacterales*	*Enterobacteriaceae*	*Escherichia*	*Escherichia coli*
6956	*Proteobacteria*	*Gammaproteobacte*	*Enterobacterales*	*Enterobacteriaceae*	*Escherichia*	*Escherichia coli*
6957	*Proteobacteria*	*Gammaproteobacte*	*Enterobacterales*	*Enterobacteriaceae*	*Escherichia*	*Escherichia coli*
6958	*Proteobacteria*	*Gammaproteobacte*	*Enterobacterales*	*Enterobacteriaceae*	*Escherichia*	*Escherichia coli*
6959	*Proteobacteria*	*Gammaproteobacte*	*Enterobacterales*	*Enterobacteriaceae*	*Escherichia*	*Escherichia coli*
6960	*Proteobacteria*	*Gammaproteobacte*	*Enterobacterales*	*Enterobacteriaceae*	*Escherichia*	*Escherichia coli*
6961	*Proteobacteria*	*Gammaproteobacte*	*Enterobacterales*	*Enterobacteriaceae*	*Escherichia*	*Escherichia coli*
6962	*Proteobacteria*	*Gammaproteobacte*	*Enterobacterales*	*Enterobacteriaceae*	*Escherichia*	*Escherichia coli*
6963	*Proteobacteria*	*Gammaproteobacte*	*Enterobacterales*	*Enterobacteriaceae*	*Escherichia*	*Escherichia coli*
6964	*Proteobacteria*	*Gammaproteobacte*	*Enterobacterales*	*Enterobacteriaceae*	*Escherichia*	*Escherichia coli*
6965	*Proteobacteria*	*Gammaproteobacte*	*Enterobacterales*	*Enterobacteriaceae*	*Escherichia*	*Escherichia coli*
6966	*Proteobacteria*	*Gammaproteobacte*	*Enterobacterales*	*Enterobacteriaceae*	*Escherichia*	*Escherichia coli*
6967	*Proteobacteria*	*Gammaproteobacte*	*Enterobacterales*	*Enterobacteriaceae*	*Escherichia*	*Escherichia coli*
6968	*Proteobacteria*	*Gammaproteobacte*	*Enterobacterales*	*Enterobacteriaceae*	*Escherichia*	*Escherichia coli*
6969	*Proteobacteria*	*Gammaproteobacte*	*Enterobacterales*	*Enterobacteriaceae*	*Escherichia*	*Escherichia coli*
6970	*Proteobacteria*	*Gammaproteobacte*	*Enterobacterales*	*Enterobacteriaceae*	*Escherichia*	*Escherichia coli*
6971	*Proteobacteria*	*Gammaproteobacte*	*Enterobacterales*	*Enterobacteriaceae*	*Escherichia*	*Escherichia coli*
6972	*Proteobacteria*	*Gammaproteobacte*	*Enterobacterales*	*Enterobacteriaceae*	*Escherichia*	*Escherichia coli*

Table S4. Isolated strains by culturomics

bacteriald	*phylum*	*class*	*order*	*family*	*genus*	*species*
6973	*Proteobacteria*	*Gammaproteobacte*	*Enterobacterales*	*Enterobacteriaceae*	*Escherichia*	*Escherichia coli*
6974	*Proteobacteria*	*Gammaproteobacte*	*Enterobacterales*	*Enterobacteriaceae*	*Escherichia*	*Escherichia coli*
6975	*Proteobacteria*	*Gammaproteobacte*	*Enterobacterales*	*Enterobacteriaceae*	*Escherichia*	*Escherichia coli*
6976	*Proteobacteria*	*Gammaproteobacte*	*Enterobacterales*	*Enterobacteriaceae*	*Escherichia*	*Escherichia coli*
6977	*Proteobacteria*	*Gammaproteobacte*	*Enterobacterales*	*Enterobacteriaceae*	*Escherichia*	*Escherichia coli*
6978	*Proteobacteria*	*Gammaproteobacte*	*Enterobacterales*	*Enterobacteriaceae*	*Escherichia*	*Escherichia coli*
6979	*Proteobacteria*	*Gammaproteobacte*	*Enterobacterales*	*Enterobacteriaceae*	*Escherichia*	*Escherichia coli*
6980	*Proteobacteria*	*Gammaproteobacte*	*Enterobacterales*	*Enterobacteriaceae*	*Escherichia*	*Escherichia coli*
6981	*Proteobacteria*	*Gammaproteobacte*	*Enterobacterales*	*Enterobacteriaceae*	*Escherichia*	*Escherichia coli*
6982	*Proteobacteria*	*Gammaproteobacte*	*Enterobacterales*	*Enterobacteriaceae*	*Escherichia*	*Escherichia coli*
6983	*Proteobacteria*	*Gammaproteobacte*	*Enterobacterales*	*Enterobacteriaceae*	*Escherichia*	*Escherichia coli*
6984	*Proteobacteria*	*Gammaproteobacte*	*Enterobacterales*	*Enterobacteriaceae*	*Escherichia*	*Escherichia coli*
6985	*Proteobacteria*	*Gammaproteobacte*	*Enterobacterales*	*Enterobacteriaceae*	*Escherichia*	*Escherichia coli*
6986	*Proteobacteria*	*Gammaproteobacte*	*Enterobacterales*	*Enterobacteriaceae*	*Escherichia*	*Escherichia coli*
6987	*Proteobacteria*	*Gammaproteobacte*	*Enterobacterales*	*Enterobacteriaceae*	*Escherichia*	*Escherichia coli*
6988	*Proteobacteria*	*Gammaproteobacte*	*Enterobacterales*	*Enterobacteriaceae*	*Escherichia*	*Escherichia coli*
6989	*Proteobacteria*	*Gammaproteobacte*	*Enterobacterales*	*Enterobacteriaceae*	*Escherichia*	*Escherichia coli*
6990	*Proteobacteria*	*Gammaproteobacte*	*Enterobacterales*	*Enterobacteriaceae*	*Escherichia*	*Escherichia coli*
6991	*Proteobacteria*	*Gammaproteobacte*	*Enterobacterales*	*Enterobacteriaceae*	*Escherichia*	*Escherichia coli*
6992	*Proteobacteria*	*Gammaproteobacte*	*Enterobacterales*	*Enterobacteriaceae*	*Escherichia*	*Escherichia coli*
6993	*Proteobacteria*	*Gammaproteobacte*	*Enterobacterales*	*Enterobacteriaceae*	*Escherichia*	*Escherichia coli*
6994	*Proteobacteria*	*Gammaproteobacte*	*Enterobacterales*	*Enterobacteriaceae*	*Escherichia*	*Escherichia coli*
6995	*Proteobacteria*	*Gammaproteobacte*	*Enterobacterales*	*Enterobacteriaceae*	*Escherichia*	*Escherichia coli*
6996	*Proteobacteria*	*Gammaproteobacte*	*Enterobacterales*	*Enterobacteriaceae*	*Escherichia*	*Escherichia coli*
6997	*Proteobacteria*	*Gammaproteobacte*	*Enterobacterales*	*Enterobacteriaceae*	*Escherichia*	*Escherichia coli*
6998	*Proteobacteria*	*Gammaproteobacte*	*Enterobacterales*	*Enterobacteriaceae*	*Escherichia*	*Escherichia coli*
6999	*Proteobacteria*	*Gammaproteobacte*	*Enterobacterales*	*Enterobacteriaceae*	*Escherichia*	*Escherichia coli*
7000	*Proteobacteria*	*Gammaproteobacte*	*Enterobacterales*	*Enterobacteriaceae*	*Escherichia*	*Escherichia coli*

Table S4. Isolated strains by culturomics

bacteriaId	*phylum*	*class*	*order*	*family*	*genus*	*species*
7001	*Proteobacteria*	*Gammaproteobacte*	*Enterobacterales*	*Enterobacteriaceae*	*Escherichia*	*Escherichia coli*
7002	*Proteobacteria*	*Gammaproteobacte*	*Enterobacterales*	*Enterobacteriaceae*	*Escherichia*	*Escherichia coli*
7003	*Proteobacteria*	*Gammaproteobacte*	*Enterobacterales*	*Enterobacteriaceae*	*Escherichia*	*Escherichia coli*
7004	*Proteobacteria*	*Gammaproteobacte*	*Enterobacterales*	*Enterobacteriaceae*	*Escherichia*	*Escherichia coli*
7005	*Proteobacteria*	*Gammaproteobacte*	*Enterobacterales*	*Enterobacteriaceae*	*Escherichia*	*Escherichia coli*
7006	*Proteobacteria*	*Gammaproteobacte*	*Enterobacterales*	*Enterobacteriaceae*	*Escherichia*	*Escherichia coli*
7007	*Proteobacteria*	*Gammaproteobacte*	*Enterobacterales*	*Enterobacteriaceae*	*Escherichia*	*Escherichia coli*
7008	*Proteobacteria*	*Gammaproteobacte*	*Enterobacterales*	*Enterobacteriaceae*	*Escherichia*	*Escherichia coli*
7009	*Proteobacteria*	*Gammaproteobacte*	*Enterobacterales*	*Enterobacteriaceae*	*Escherichia*	*Escherichia coli*
7010	*Proteobacteria*	*Gammaproteobacte*	*Enterobacterales*	*Enterobacteriaceae*	*Escherichia*	*Escherichia coli*
7011	*Proteobacteria*	*Gammaproteobacte*	*Enterobacterales*	*Enterobacteriaceae*	*Escherichia*	*Escherichia coli*
7012	*Proteobacteria*	*Gammaproteobacte*	*Enterobacterales*	*Enterobacteriaceae*	*Escherichia*	*Escherichia coli*
7013	*Proteobacteria*	*Gammaproteobacte*	*Enterobacterales*	*Enterobacteriaceae*	*Escherichia*	*Escherichia coli*
7014	*Proteobacteria*	*Gammaproteobacte*	*Enterobacterales*	*Enterobacteriaceae*	*Escherichia*	*Escherichia coli*
7015	*Proteobacteria*	*Gammaproteobacte*	*Enterobacterales*	*Enterobacteriaceae*	*Escherichia*	*Escherichia coli*
7016	*Proteobacteria*	*Gammaproteobacte*	*Enterobacterales*	*Enterobacteriaceae*	*Escherichia*	*Escherichia coli*
7017	*Proteobacteria*	*Gammaproteobacte*	*Enterobacterales*	*Enterobacteriaceae*	*Escherichia*	*Escherichia coli*
7018	*Proteobacteria*	*Gammaproteobacte*	*Enterobacterales*	*Enterobacteriaceae*	*Escherichia*	*Escherichia coli*
7019	*Proteobacteria*	*Gammaproteobacte*	*Enterobacterales*	*Enterobacteriaceae*	*Escherichia*	*Escherichia coli*
7020	*Proteobacteria*	*Gammaproteobacte*	*Enterobacterales*	*Enterobacteriaceae*	*Escherichia*	*Escherichia coli*
7021	*Proteobacteria*	*Gammaproteobacte*	*Enterobacterales*	*Enterobacteriaceae*	*Escherichia*	*Escherichia coli*
7022	*Proteobacteria*	*Gammaproteobacte*	*Enterobacterales*	*Enterobacteriaceae*	*Escherichia*	*Escherichia coli*
7023	*Proteobacteria*	*Gammaproteobacte*	*Enterobacterales*	*Enterobacteriaceae*	*Escherichia*	*Escherichia coli*
7024	*Proteobacteria*	*Gammaproteobacte*	*Enterobacterales*	*Enterobacteriaceae*	*Escherichia*	*Escherichia coli*
7025	*Proteobacteria*	*Gammaproteobacte*	*Enterobacterales*	*Enterobacteriaceae*	*Escherichia*	*Escherichia coli*
7026	*Proteobacteria*	*Gammaproteobacte*	*Enterobacterales*	*Enterobacteriaceae*	*Escherichia*	*Escherichia coli*
7027	*Proteobacteria*	*Gammaproteobacte*	*Enterobacterales*	*Enterobacteriaceae*	*Escherichia*	*Escherichia coli*
7028	*Proteobacteria*	*Gammaproteobacte*	*Enterobacterales*	*Enterobacteriaceae*	*Escherichia*	*Escherichia coli*

Table S4. Isolated strains by culturomics

bacteriaId	*phylum*	*class*	*order*	*family*	*genus*	*species*
7029	*Proteobacteria*	*Gammaproteobacte*	*Enterobacterales*	*Enterobacteriaceae*	*Escherichia*	*Escherichia coli*
7030	*Proteobacteria*	*Gammaproteobacte*	*Enterobacterales*	*Enterobacteriaceae*	*Escherichia*	*Escherichia coli*
7031	*Proteobacteria*	*Gammaproteobacte*	*Enterobacterales*	*Enterobacteriaceae*	*Escherichia*	*Escherichia coli*
7032	*Proteobacteria*	*Gammaproteobacte*	*Enterobacterales*	*Enterobacteriaceae*	*Escherichia*	*Escherichia coli*
7033	*Proteobacteria*	*Gammaproteobacte*	*Enterobacterales*	*Enterobacteriaceae*	*Escherichia*	*Escherichia coli*
7034	*Proteobacteria*	*Gammaproteobacte*	*Enterobacterales*	*Enterobacteriaceae*	*Escherichia*	*Escherichia coli*
7035	*Proteobacteria*	*Gammaproteobacte*	*Enterobacterales*	*Enterobacteriaceae*	*Escherichia*	*Escherichia coli*
7036	*Proteobacteria*	*Gammaproteobacte*	*Enterobacterales*	*Enterobacteriaceae*	*Escherichia*	*Escherichia coli*
7037	*Proteobacteria*	*Gammaproteobacte*	*Enterobacterales*	*Enterobacteriaceae*	*Escherichia*	*Escherichia coli*
7038	*Proteobacteria*	*Gammaproteobacte*	*Enterobacterales*	*Enterobacteriaceae*	*Escherichia*	*Escherichia coli*
7039	*Proteobacteria*	*Gammaproteobacte*	*Enterobacterales*	*Enterobacteriaceae*	*Escherichia*	*Escherichia coli*
7040	*Proteobacteria*	*Gammaproteobacte*	*Enterobacterales*	*Enterobacteriaceae*	*Escherichia*	*Escherichia coli*
7041	*Proteobacteria*	*Gammaproteobacte*	*Enterobacterales*	*Enterobacteriaceae*	*Escherichia*	*Escherichia coli*
7042	*Proteobacteria*	*Gammaproteobacte*	*Enterobacterales*	*Enterobacteriaceae*	*Escherichia*	*Escherichia coli*
7043	*Proteobacteria*	*Gammaproteobacte*	*Enterobacterales*	*Enterobacteriaceae*	*Escherichia*	*Escherichia coli*
7044	*Proteobacteria*	*Gammaproteobacte*	*Enterobacterales*	*Enterobacteriaceae*	*Escherichia*	*Escherichia coli*
7045	*Proteobacteria*	*Gammaproteobacte*	*Enterobacterales*	*Enterobacteriaceae*	*Escherichia*	*Escherichia coli*
7046	*Proteobacteria*	*Gammaproteobacte*	*Enterobacterales*	*Enterobacteriaceae*	*Escherichia*	*Escherichia coli*
7047	*Proteobacteria*	*Gammaproteobacte*	*Enterobacterales*	*Enterobacteriaceae*	*Escherichia*	*Escherichia coli*
7048	*Proteobacteria*	*Gammaproteobacte*	*Enterobacterales*	*Enterobacteriaceae*	*Escherichia*	*Escherichia coli*
7049	*Proteobacteria*	*Gammaproteobacte*	*Enterobacterales*	*Enterobacteriaceae*	*Escherichia*	*Escherichia coli*
7050	*Proteobacteria*	*Gammaproteobacte*	*Enterobacterales*	*Enterobacteriaceae*	*Escherichia*	*Escherichia coli*
7051	*Proteobacteria*	*Gammaproteobacte*	*Enterobacterales*	*Enterobacteriaceae*	*Escherichia*	*Escherichia coli*
7052	*Proteobacteria*	*Gammaproteobacte*	*Enterobacterales*	*Enterobacteriaceae*	*Escherichia*	*Escherichia coli*
7053	*Proteobacteria*	*Gammaproteobacte*	*Enterobacterales*	*Enterobacteriaceae*	*Escherichia*	*Escherichia coli*
7054	*Proteobacteria*	*Gammaproteobacte*	*Enterobacterales*	*Enterobacteriaceae*	*Escherichia*	*Escherichia coli*
7055	*Proteobacteria*	*Gammaproteobacte*	*Enterobacterales*	*Enterobacteriaceae*	*Escherichia*	*Escherichia coli*
7056	*Proteobacteria*	*Gammaproteobacte*	*Enterobacterales*	*Enterobacteriaceae*	*Escherichia*	*Escherichia coli*

Table S4. Isolated strains by culturomics

bacteriaId	*phylum*	*class*	*order*	*family*	*genus*	*species*
7057	*Proteobacteria*	*Gammaproteobacte*	*Enterobacterales*	*Enterobacteriaceae*	*Escherichia*	*Escherichia coli*
7058	*Proteobacteria*	*Gammaproteobacte*	*Enterobacterales*	*Enterobacteriaceae*	*Escherichia*	*Escherichia coli*
7059	*Proteobacteria*	*Gammaproteobacte*	*Enterobacterales*	*Enterobacteriaceae*	*Escherichia*	*Escherichia coli*
7060	*Proteobacteria*	*Gammaproteobacte*	*Enterobacterales*	*Enterobacteriaceae*	*Escherichia*	*Escherichia coli*
7061	*Proteobacteria*	*Gammaproteobacte*	*Enterobacterales*	*Enterobacteriaceae*	*Escherichia*	*Escherichia coli*
7062	*Proteobacteria*	*Gammaproteobacte*	*Enterobacterales*	*Enterobacteriaceae*	*Escherichia*	*Escherichia coli*
7063	*Proteobacteria*	*Gammaproteobacte*	*Enterobacterales*	*Enterobacteriaceae*	*Escherichia*	*Escherichia coli*
7064	*Proteobacteria*	*Gammaproteobacte*	*Enterobacterales*	*Enterobacteriaceae*	*Escherichia*	*Escherichia coli*
7065	*Proteobacteria*	*Gammaproteobacte*	*Enterobacterales*	*Enterobacteriaceae*	*Escherichia*	*Escherichia coli*
7066	*Proteobacteria*	*Gammaproteobacte*	*Enterobacterales*	*Enterobacteriaceae*	*Escherichia*	*Escherichia coli*
7067	*Proteobacteria*	*Gammaproteobacte*	*Enterobacterales*	*Enterobacteriaceae*	*Escherichia*	*Escherichia coli*
7068	*Proteobacteria*	*Gammaproteobacte*	*Enterobacterales*	*Enterobacteriaceae*	*Escherichia*	*Escherichia coli*
7069	*Proteobacteria*	*Gammaproteobacte*	*Enterobacterales*	*Enterobacteriaceae*	*Escherichia*	*Escherichia coli*
7070	*Proteobacteria*	*Gammaproteobacte*	*Enterobacterales*	*Enterobacteriaceae*	*Escherichia*	*Escherichia coli*
7071	*Proteobacteria*	*Gammaproteobacte*	*Enterobacterales*	*Enterobacteriaceae*	*Escherichia*	*Escherichia coli*
7072	*Proteobacteria*	*Gammaproteobacte*	*Enterobacterales*	*Enterobacteriaceae*	*Escherichia*	*Escherichia coli*
7073	*Proteobacteria*	*Gammaproteobacte*	*Enterobacterales*	*Enterobacteriaceae*	*Escherichia*	*Escherichia coli*
7074	*Proteobacteria*	*Gammaproteobacte*	*Enterobacterales*	*Enterobacteriaceae*	*Escherichia*	*Escherichia coli*
7075	*Proteobacteria*	*Gammaproteobacte*	*Enterobacterales*	*Enterobacteriaceae*	*Escherichia*	*Escherichia coli*
7076	*Proteobacteria*	*Gammaproteobacte*	*Enterobacterales*	*Enterobacteriaceae*	*Escherichia*	*Escherichia coli*
7077	*Proteobacteria*	*Gammaproteobacte*	*Enterobacterales*	*Enterobacteriaceae*	*Escherichia*	*Escherichia coli*
7078	*Proteobacteria*	*Gammaproteobacte*	*Enterobacterales*	*Enterobacteriaceae*	*Escherichia*	*Escherichia coli*
7079	*Proteobacteria*	*Gammaproteobacte*	*Enterobacterales*	*Enterobacteriaceae*	*Escherichia*	*Escherichia coli*
7080	*Proteobacteria*	*Gammaproteobacte*	*Enterobacterales*	*Enterobacteriaceae*	*Escherichia*	*Escherichia coli*
7081	*Proteobacteria*	*Gammaproteobacte*	*Enterobacterales*	*Enterobacteriaceae*	*Escherichia*	*Escherichia coli*
7082	*Proteobacteria*	*Gammaproteobacte*	*Enterobacterales*	*Enterobacteriaceae*	*Escherichia*	*Escherichia coli*
7083	*Proteobacteria*	*Gammaproteobacte*	*Enterobacterales*	*Enterobacteriaceae*	*Escherichia*	*Escherichia coli*
7084	*Proteobacteria*	*Gammaproteobacte*	*Enterobacterales*	*Enterobacteriaceae*	*Escherichia*	*Escherichia coli*

Table S4. Isolated strains by culturomics

bacteriaId	*phylum*	*class*	*order*	*family*	*genus*	*species*
7085	*Proteobacteria*	*Gammaproteobacte*	*Enterobacterales*	*Enterobacteriaceae*	*Escherichia*	*Escherichia coli*
7086	*Proteobacteria*	*Gammaproteobacte*	*Enterobacterales*	*Enterobacteriaceae*	*Escherichia*	*Escherichia coli*
7087	*Proteobacteria*	*Gammaproteobacte*	*Enterobacterales*	*Enterobacteriaceae*	*Escherichia*	*Escherichia coli*
7088	*Proteobacteria*	*Gammaproteobacte*	*Enterobacterales*	*Enterobacteriaceae*	*Escherichia*	*Escherichia coli*
7089	*Proteobacteria*	*Gammaproteobacte*	*Enterobacterales*	*Enterobacteriaceae*	*Escherichia*	*Escherichia coli*
7090	*Proteobacteria*	*Gammaproteobacte*	*Enterobacterales*	*Enterobacteriaceae*	*Escherichia*	*Escherichia coli*
7091	*Proteobacteria*	*Gammaproteobacte*	*Enterobacterales*	*Enterobacteriaceae*	*Escherichia*	*Escherichia coli*
7092	*Proteobacteria*	*Gammaproteobacte*	*Enterobacterales*	*Enterobacteriaceae*	*Escherichia*	*Escherichia coli*
7093	*Proteobacteria*	*Gammaproteobacte*	*Enterobacterales*	*Enterobacteriaceae*	*Escherichia*	*Escherichia coli*
7094	*Proteobacteria*	*Gammaproteobacte*	*Enterobacterales*	*Enterobacteriaceae*	*Escherichia*	*Escherichia coli*
7095	*Proteobacteria*	*Gammaproteobacte*	*Enterobacterales*	*Enterobacteriaceae*	*Escherichia*	*Escherichia coli*
7096	*Proteobacteria*	*Gammaproteobacte*	*Enterobacterales*	*Enterobacteriaceae*	*Escherichia*	*Escherichia coli*
7097	*Proteobacteria*	*Gammaproteobacte*	*Enterobacterales*	*Enterobacteriaceae*	*Escherichia*	*Escherichia coli*
7098	*Proteobacteria*	*Gammaproteobacte*	*Enterobacterales*	*Enterobacteriaceae*	*Escherichia*	*Escherichia coli*
7099	*Proteobacteria*	*Gammaproteobacte*	*Enterobacterales*	*Enterobacteriaceae*	*Escherichia*	*Escherichia coli*
7100	*Proteobacteria*	*Gammaproteobacte*	*Enterobacterales*	*Enterobacteriaceae*	*Escherichia*	*Escherichia coli*
7101	*Proteobacteria*	*Gammaproteobacte*	*Enterobacterales*	*Enterobacteriaceae*	*Escherichia*	*Escherichia coli*
7102	*Proteobacteria*	*Gammaproteobacte*	*Enterobacterales*	*Enterobacteriaceae*	*Escherichia*	*Escherichia coli*
7103	*Proteobacteria*	*Gammaproteobacte*	*Enterobacterales*	*Enterobacteriaceae*	*Escherichia*	*Escherichia coli*
7104	*Proteobacteria*	*Gammaproteobacte*	*Enterobacterales*	*Enterobacteriaceae*	*Escherichia*	*Escherichia coli*
7105	*Proteobacteria*	*Gammaproteobacte*	*Enterobacterales*	*Enterobacteriaceae*	*Escherichia*	*Escherichia coli*
7106	*Proteobacteria*	*Gammaproteobacte*	*Enterobacterales*	*Enterobacteriaceae*	*Escherichia*	*Escherichia coli*
7107	*Proteobacteria*	*Gammaproteobacte*	*Enterobacterales*	*Enterobacteriaceae*	*Escherichia*	*Escherichia coli*
7108	*Proteobacteria*	*Gammaproteobacte*	*Enterobacterales*	*Enterobacteriaceae*	*Escherichia*	*Escherichia coli*
7109	*Proteobacteria*	*Gammaproteobacte*	*Enterobacterales*	*Enterobacteriaceae*	*Escherichia*	*Escherichia coli*
7110	*Proteobacteria*	*Gammaproteobacte*	*Enterobacterales*	*Enterobacteriaceae*	*Escherichia*	*Escherichia coli*
7111	*Proteobacteria*	*Gammaproteobacte*	*Enterobacterales*	*Enterobacteriaceae*	*Escherichia*	*Escherichia coli*
7112	*Proteobacteria*	*Gammaproteobacte*	*Enterobacterales*	*Enterobacteriaceae*	*Escherichia*	*Escherichia coli*

Table S4. Isolated strains by culturomics

bacteriaId	*phylum*	*class*	*order*	*family*	*genus*	*species*
7113	*Proteobacteria*	*Gammaproteobacte*	*Enterobacterales*	*Enterobacteriaceae*	*Escherichia*	*Escherichia coli*
7114	*Proteobacteria*	*Gammaproteobacte*	*Enterobacterales*	*Enterobacteriaceae*	*Escherichia*	*Escherichia coli*
7115	*Proteobacteria*	*Gammaproteobacte*	*Enterobacterales*	*Enterobacteriaceae*	*Escherichia*	*Escherichia coli*
7116	*Proteobacteria*	*Gammaproteobacte*	*Enterobacterales*	*Enterobacteriaceae*	*Escherichia*	*Escherichia coli*
7117	*Proteobacteria*	*Gammaproteobacte*	*Enterobacterales*	*Enterobacteriaceae*	*Escherichia*	*Escherichia coli*
7118	*Proteobacteria*	*Gammaproteobacte*	*Enterobacterales*	*Enterobacteriaceae*	*Escherichia*	*Escherichia coli*
7119	*Proteobacteria*	*Gammaproteobacte*	*Enterobacterales*	*Enterobacteriaceae*	*Escherichia*	*Escherichia coli*
7120	*Proteobacteria*	*Gammaproteobacte*	*Enterobacterales*	*Enterobacteriaceae*	*Escherichia*	*Escherichia coli*
7121	*Proteobacteria*	*Gammaproteobacte*	*Enterobacterales*	*Enterobacteriaceae*	*Escherichia*	*Escherichia coli*
7122	*Proteobacteria*	*Gammaproteobacte*	*Enterobacterales*	*Enterobacteriaceae*	*Escherichia*	*Escherichia coli*
7123	*Proteobacteria*	*Gammaproteobacte*	*Enterobacterales*	*Enterobacteriaceae*	*Escherichia*	*Escherichia coli*
7124	*Proteobacteria*	*Gammaproteobacte*	*Enterobacterales*	*Enterobacteriaceae*	*Escherichia*	*Escherichia coli*
7125	*Proteobacteria*	*Gammaproteobacte*	*Enterobacterales*	*Enterobacteriaceae*	*Escherichia*	*Escherichia coli*
7126	*Proteobacteria*	*Gammaproteobacte*	*Enterobacterales*	*Enterobacteriaceae*	*Escherichia*	*Escherichia coli*
7127	*Proteobacteria*	*Gammaproteobacte*	*Enterobacterales*	*Enterobacteriaceae*	*Escherichia*	*Escherichia coli*
7128	*Proteobacteria*	*Gammaproteobacte*	*Enterobacterales*	*Enterobacteriaceae*	*Escherichia*	*Escherichia coli*
7129	*Proteobacteria*	*Gammaproteobacte*	*Enterobacterales*	*Enterobacteriaceae*	*Escherichia*	*Escherichia coli*
7130	*Proteobacteria*	*Gammaproteobacte*	*Enterobacterales*	*Enterobacteriaceae*	*Escherichia*	*Escherichia coli*
7131	*Proteobacteria*	*Gammaproteobacte*	*Enterobacterales*	*Enterobacteriaceae*	*Escherichia*	*Escherichia coli*
7132	*Proteobacteria*	*Gammaproteobacte*	*Enterobacterales*	*Enterobacteriaceae*	*Escherichia*	*Escherichia coli*
7133	*Proteobacteria*	*Gammaproteobacte*	*Enterobacterales*	*Enterobacteriaceae*	*Escherichia*	*Escherichia coli*
7134	*Proteobacteria*	*Gammaproteobacte*	*Enterobacterales*	*Enterobacteriaceae*	*Escherichia*	*Escherichia coli*
7135	*Proteobacteria*	*Gammaproteobacte*	*Enterobacterales*	*Enterobacteriaceae*	*Escherichia*	*Escherichia coli*
7136	*Proteobacteria*	*Gammaproteobacte*	*Enterobacterales*	*Enterobacteriaceae*	*Escherichia*	*Escherichia coli*
7137	*Proteobacteria*	*Gammaproteobacte*	*Enterobacterales*	*Enterobacteriaceae*	*Escherichia*	*Escherichia coli*
7138	*Proteobacteria*	*Gammaproteobacte*	*Enterobacterales*	*Enterobacteriaceae*	*Escherichia*	*Escherichia coli*
7139	*Proteobacteria*	*Gammaproteobacte*	*Enterobacterales*	*Enterobacteriaceae*	*Escherichia*	*Escherichia coli*
7140	*Proteobacteria*	*Gammaproteobacte*	*Enterobacterales*	*Enterobacteriaceae*	*Escherichia*	*Escherichia coli*

Table S4. Isolated strains by culturomics

bacteriaId	*phylum*	*class*	*order*	*family*	*genus*	*species*
7141	*Proteobacteria*	*Gammaproteobacte*	*Enterobacterales*	*Enterobacteriaceae*	*Escherichia*	*Escherichia coli*
7142	*Proteobacteria*	*Gammaproteobacte*	*Enterobacterales*	*Enterobacteriaceae*	*Escherichia*	*Escherichia coli*
7143	*Proteobacteria*	*Gammaproteobacte*	*Enterobacterales*	*Enterobacteriaceae*	*Escherichia*	*Escherichia coli*
7144	*Proteobacteria*	*Gammaproteobacte*	*Enterobacterales*	*Enterobacteriaceae*	*Escherichia*	*Escherichia coli*
7145	*Proteobacteria*	*Gammaproteobacte*	*Enterobacterales*	*Enterobacteriaceae*	*Escherichia*	*Escherichia coli*
7146	*Proteobacteria*	*Gammaproteobacte*	*Enterobacterales*	*Enterobacteriaceae*	*Escherichia*	*Escherichia coli*
7147	*Proteobacteria*	*Gammaproteobacte*	*Enterobacterales*	*Enterobacteriaceae*	*Escherichia*	*Escherichia coli*
7148	*Proteobacteria*	*Gammaproteobacte*	*Enterobacterales*	*Enterobacteriaceae*	*Escherichia*	*Escherichia coli*
7149	*Proteobacteria*	*Gammaproteobacte*	*Enterobacterales*	*Enterobacteriaceae*	*Escherichia*	*Escherichia coli*
7150	*Proteobacteria*	*Gammaproteobacte*	*Enterobacterales*	*Enterobacteriaceae*	*Escherichia*	*Escherichia coli*
7151	*Proteobacteria*	*Gammaproteobacte*	*Enterobacterales*	*Enterobacteriaceae*	*Escherichia*	*Escherichia coli*
7152	*Proteobacteria*	*Gammaproteobacte*	*Enterobacterales*	*Enterobacteriaceae*	*Escherichia*	*Escherichia coli*
7153	*Proteobacteria*	*Gammaproteobacte*	*Enterobacterales*	*Enterobacteriaceae*	*Escherichia*	*Escherichia coli*
7154	*Proteobacteria*	*Gammaproteobacte*	*Enterobacterales*	*Enterobacteriaceae*	*Escherichia*	*Escherichia coli*
7155	*Proteobacteria*	*Gammaproteobacte*	*Enterobacterales*	*Enterobacteriaceae*	*Escherichia*	*Escherichia coli*
7156	*Proteobacteria*	*Gammaproteobacte*	*Enterobacterales*	*Enterobacteriaceae*	*Escherichia*	*Escherichia coli*
7157	*Proteobacteria*	*Gammaproteobacte*	*Enterobacterales*	*Enterobacteriaceae*	*Escherichia*	*Escherichia coli*
7158	*Proteobacteria*	*Gammaproteobacte*	*Enterobacterales*	*Enterobacteriaceae*	*Escherichia*	*Escherichia coli*
7159	*Proteobacteria*	*Gammaproteobacte*	*Enterobacterales*	*Enterobacteriaceae*	*Escherichia*	*Escherichia coli*
7160	*Proteobacteria*	*Gammaproteobacte*	*Enterobacterales*	*Enterobacteriaceae*	*Escherichia*	*Escherichia coli*
7161	*Proteobacteria*	*Gammaproteobacte*	*Enterobacterales*	*Enterobacteriaceae*	*Escherichia*	*Escherichia coli*
7162	*Proteobacteria*	*Gammaproteobacte*	*Enterobacterales*	*Enterobacteriaceae*	*Escherichia*	*Escherichia coli*
7163	*Proteobacteria*	*Gammaproteobacte*	*Enterobacterales*	*Enterobacteriaceae*	*Escherichia*	*Escherichia coli*
7164	*Proteobacteria*	*Gammaproteobacte*	*Enterobacterales*	*Enterobacteriaceae*	*Escherichia*	*Escherichia coli*
7165	*Proteobacteria*	*Gammaproteobacte*	*Enterobacterales*	*Enterobacteriaceae*	*Escherichia*	*Escherichia coli*
7166	*Proteobacteria*	*Gammaproteobacte*	*Enterobacterales*	*Enterobacteriaceae*	*Escherichia*	*Escherichia coli*
7167	*Proteobacteria*	*Gammaproteobacte*	*Enterobacterales*	*Enterobacteriaceae*	*Escherichia*	*Escherichia coli*
7168	*Proteobacteria*	*Gammaproteobacte*	*Enterobacterales*	*Enterobacteriaceae*	*Escherichia*	*Escherichia coli*

Table S4. Isolated strains by culturomics

bacteriaId	phylum	class	order	family	genus	species
7169	*Proteobacteria*	*Gammaproteobacte*	*Enterobacterales*	*Enterobacteriaceae*	*Escherichia*	*Escherichia coli*
7170	*Proteobacteria*	*Gammaproteobacte*	*Enterobacterales*	*Enterobacteriaceae*	*Escherichia*	*Escherichia coli*
7171	*Proteobacteria*	*Gammaproteobacte*	*Enterobacterales*	*Enterobacteriaceae*	*Escherichia*	*Escherichia coli*
7172	*Proteobacteria*	*Gammaproteobacte*	*Enterobacterales*	*Enterobacteriaceae*	*Escherichia*	*Escherichia coli*
7173	*Proteobacteria*	*Gammaproteobacte*	*Enterobacterales*	*Enterobacteriaceae*	*Escherichia*	*Escherichia coli*
7174	*Proteobacteria*	*Gammaproteobacte*	*Enterobacterales*	*Enterobacteriaceae*	*Escherichia*	*Escherichia coli*
7175	*Proteobacteria*	*Gammaproteobacte*	*Enterobacterales*	*Enterobacteriaceae*	*Escherichia*	*Escherichia coli*
7176	*Proteobacteria*	*Gammaproteobacte*	*Enterobacterales*	*Enterobacteriaceae*	*Escherichia*	*Escherichia coli*
7177	*Proteobacteria*	*Gammaproteobacte*	*Enterobacterales*	*Enterobacteriaceae*	*Escherichia*	*Escherichia coli*
7178	*Proteobacteria*	*Gammaproteobacte*	*Enterobacterales*	*Enterobacteriaceae*	*Escherichia*	*Escherichia coli*
7179	*Proteobacteria*	*Gammaproteobacte*	*Enterobacterales*	*Enterobacteriaceae*	*Escherichia*	*Escherichia coli*
7180	*Proteobacteria*	*Gammaproteobacte*	*Enterobacterales*	*Enterobacteriaceae*	*Escherichia*	*Escherichia coli*
7181	*Proteobacteria*	*Gammaproteobacte*	*Enterobacterales*	*Enterobacteriaceae*	*Escherichia*	*Escherichia coli*
7182	*Proteobacteria*	*Gammaproteobacte*	*Enterobacterales*	*Enterobacteriaceae*	*Escherichia*	*Escherichia coli*
7183	*Proteobacteria*	*Gammaproteobacte*	*Enterobacterales*	*Enterobacteriaceae*	*Escherichia*	*Escherichia coli*
7184	*Proteobacteria*	*Gammaproteobacte*	*Enterobacterales*	*Enterobacteriaceae*	*Escherichia*	*Escherichia coli*
7185	*Proteobacteria*	*Gammaproteobacte*	*Enterobacterales*	*Enterobacteriaceae*	*Escherichia*	*Escherichia coli*
7186	*Proteobacteria*	*Gammaproteobacte*	*Enterobacterales*	*Enterobacteriaceae*	*Escherichia*	*Escherichia coli*
7187	*Proteobacteria*	*Gammaproteobacte*	*Enterobacterales*	*Enterobacteriaceae*	*Escherichia*	*Escherichia coli*
7188	*Proteobacteria*	*Gammaproteobacte*	*Enterobacterales*	*Enterobacteriaceae*	*Escherichia*	*Escherichia coli*
7189	*Proteobacteria*	*Gammaproteobacte*	*Enterobacterales*	*Enterobacteriaceae*	*Escherichia*	*Escherichia coli*
7190	*Proteobacteria*	*Gammaproteobacte*	*Enterobacterales*	*Enterobacteriaceae*	*Escherichia*	*Escherichia coli*
7191	*Proteobacteria*	*Gammaproteobacte*	*Enterobacterales*	*Enterobacteriaceae*	*Escherichia*	*Escherichia coli*
7192	*Proteobacteria*	*Gammaproteobacte*	*Enterobacterales*	*Enterobacteriaceae*	*Escherichia*	*Escherichia coli*
7193	*Proteobacteria*	*Gammaproteobacte*	*Enterobacterales*	*Enterobacteriaceae*	*Escherichia*	*Escherichia coli*
7194	*Proteobacteria*	*Gammaproteobacte*	*Enterobacterales*	*Enterobacteriaceae*	*Escherichia*	*Escherichia coli*
7195	*Proteobacteria*	*Gammaproteobacte*	*Enterobacterales*	*Enterobacteriaceae*	*Escherichia*	*Escherichia coli*
7196	*Proteobacteria*	*Gammaproteobacte*	*Enterobacterales*	*Enterobacteriaceae*	*Escherichia*	*Escherichia coli*

Table S4. Isolated strains by culturomics

bacteriaId	*phylum*	*class*	*order*	*family*	*genus*	*species*
7197	*Proteobacteria*	*Gammaproteobacte*	*Enterobacterales*	*Enterobacteriaceae*	*Escherichia*	*Escherichia coli*
7198	*Proteobacteria*	*Gammaproteobacte*	*Enterobacterales*	*Enterobacteriaceae*	*Escherichia*	*Escherichia coli*
7199	*Proteobacteria*	*Gammaproteobacte*	*Enterobacterales*	*Enterobacteriaceae*	*Escherichia*	*Escherichia coli*
7200	*Proteobacteria*	*Gammaproteobacte*	*Enterobacterales*	*Enterobacteriaceae*	*Escherichia*	*Escherichia coli*
7201	*Proteobacteria*	*Gammaproteobacte*	*Enterobacterales*	*Enterobacteriaceae*	*Escherichia*	*Escherichia coli*
7202	*Proteobacteria*	*Gammaproteobacte*	*Enterobacterales*	*Enterobacteriaceae*	*Escherichia*	*Escherichia coli*
7203	*Proteobacteria*	*Gammaproteobacte*	*Enterobacterales*	*Enterobacteriaceae*	*Escherichia*	*Escherichia coli*
7204	*Proteobacteria*	*Gammaproteobacte*	*Enterobacterales*	*Enterobacteriaceae*	*Escherichia*	*Escherichia coli*
7205	*Proteobacteria*	*Gammaproteobacte*	*Enterobacterales*	*Enterobacteriaceae*	*Escherichia*	*Escherichia coli*
7206	*Proteobacteria*	*Gammaproteobacte*	*Enterobacterales*	*Enterobacteriaceae*	*Escherichia*	*Escherichia coli*
7207	*Proteobacteria*	*Gammaproteobacte*	*Enterobacterales*	*Enterobacteriaceae*	*Escherichia*	*Escherichia coli*
7208	*Proteobacteria*	*Gammaproteobacte*	*Enterobacterales*	*Enterobacteriaceae*	*Escherichia*	*Escherichia coli*
7209	*Proteobacteria*	*Gammaproteobacte*	*Enterobacterales*	*Enterobacteriaceae*	*Escherichia*	*Escherichia coli*
7210	*Proteobacteria*	*Gammaproteobacte*	*Enterobacterales*	*Enterobacteriaceae*	*Escherichia*	*Escherichia coli*
7211	*Proteobacteria*	*Gammaproteobacte*	*Enterobacterales*	*Enterobacteriaceae*	*Escherichia*	*Escherichia coli*
7212	*Proteobacteria*	*Gammaproteobacte*	*Enterobacterales*	*Enterobacteriaceae*	*Escherichia*	*Escherichia coli*
7213	*Proteobacteria*	*Gammaproteobacte*	*Enterobacterales*	*Enterobacteriaceae*	*Escherichia*	*Escherichia coli*
7214	*Proteobacteria*	*Gammaproteobacte*	*Enterobacterales*	*Enterobacteriaceae*	*Escherichia*	*Escherichia coli*
7215	*Proteobacteria*	*Gammaproteobacte*	*Enterobacterales*	*Enterobacteriaceae*	*Escherichia*	*Escherichia coli*
7216	*Proteobacteria*	*Gammaproteobacte*	*Enterobacterales*	*Enterobacteriaceae*	*Escherichia*	*Escherichia coli*
7217	*Proteobacteria*	*Gammaproteobacte*	*Enterobacterales*	*Enterobacteriaceae*	*Escherichia*	*Escherichia coli*
7218	*Proteobacteria*	*Gammaproteobacte*	*Enterobacterales*	*Enterobacteriaceae*	*Escherichia*	*Escherichia coli*
7219	*Proteobacteria*	*Gammaproteobacte*	*Enterobacterales*	*Enterobacteriaceae*	*Escherichia*	*Escherichia coli*
7220	*Proteobacteria*	*Gammaproteobacte*	*Enterobacterales*	*Enterobacteriaceae*	*Escherichia*	*Escherichia coli*
7221	*Proteobacteria*	*Gammaproteobacte*	*Enterobacterales*	*Enterobacteriaceae*	*Escherichia*	*Escherichia coli*
7222	*Proteobacteria*	*Gammaproteobacte*	*Enterobacterales*	*Enterobacteriaceae*	*Escherichia*	*Escherichia coli*
7223	*Proteobacteria*	*Gammaproteobacte*	*Enterobacterales*	*Enterobacteriaceae*	*Escherichia*	*Escherichia coli*
7224	*Proteobacteria*	*Gammaproteobacte*	*Enterobacterales*	*Enterobacteriaceae*	*Escherichia*	*Escherichia coli*

Table S4. Isolated strains by culturomics

bacteriaId	*phylum*	*class*	*order*	*family*	*genus*	*species*
7225	*Proteobacteria*	*Gammaproteobacte*	*Enterobacterales*	*Enterobacteriaceae*	*Escherichia*	*Escherichia coli*
7226	*Proteobacteria*	*Gammaproteobacte*	*Enterobacterales*	*Enterobacteriaceae*	*Escherichia*	*Escherichia coli*
7227	*Proteobacteria*	*Gammaproteobacte*	*Enterobacterales*	*Enterobacteriaceae*	*Escherichia*	*Escherichia coli*
7228	*Proteobacteria*	*Gammaproteobacte*	*Enterobacterales*	*Enterobacteriaceae*	*Escherichia*	*Escherichia coli*
7229	*Proteobacteria*	*Gammaproteobacte*	*Enterobacterales*	*Enterobacteriaceae*	*Escherichia*	*Escherichia coli*
7230	*Proteobacteria*	*Gammaproteobacte*	*Enterobacterales*	*Enterobacteriaceae*	*Escherichia*	*Escherichia coli*
7231	*Proteobacteria*	*Gammaproteobacte*	*Enterobacterales*	*Enterobacteriaceae*	*Escherichia*	*Escherichia coli*
7232	*Proteobacteria*	*Gammaproteobacte*	*Enterobacterales*	*Enterobacteriaceae*	*Escherichia*	*Escherichia coli*
7233	*Proteobacteria*	*Gammaproteobacte*	*Enterobacterales*	*Enterobacteriaceae*	*Escherichia*	*Escherichia coli*
7234	*Proteobacteria*	*Gammaproteobacte*	*Enterobacterales*	*Enterobacteriaceae*	*Escherichia*	*Escherichia coli*
7235	*Proteobacteria*	*Gammaproteobacte*	*Enterobacterales*	*Enterobacteriaceae*	*Escherichia*	*Escherichia coli*
7236	*Proteobacteria*	*Gammaproteobacte*	*Enterobacterales*	*Enterobacteriaceae*	*Escherichia*	*Escherichia coli*
7237	*Proteobacteria*	*Gammaproteobacte*	*Enterobacterales*	*Enterobacteriaceae*	*Escherichia*	*Escherichia coli*
7238	*Proteobacteria*	*Gammaproteobacte*	*Enterobacterales*	*Enterobacteriaceae*	*Escherichia*	*Escherichia coli*
7239	*Proteobacteria*	*Gammaproteobacte*	*Enterobacterales*	*Enterobacteriaceae*	*Escherichia*	*Escherichia coli*
7240	*Proteobacteria*	*Gammaproteobacte*	*Enterobacterales*	*Enterobacteriaceae*	*Escherichia*	*Escherichia coli*
7241	*Proteobacteria*	*Gammaproteobacte*	*Enterobacterales*	*Enterobacteriaceae*	*Escherichia*	*Escherichia coli*
7242	*Proteobacteria*	*Gammaproteobacte*	*Enterobacterales*	*Enterobacteriaceae*	*Escherichia*	*Escherichia coli*
7243	*Proteobacteria*	*Gammaproteobacte*	*Enterobacterales*	*Enterobacteriaceae*	*Escherichia*	*Escherichia coli*
7244	*Proteobacteria*	*Gammaproteobacte*	*Enterobacterales*	*Enterobacteriaceae*	*Escherichia*	*Escherichia coli*
7245	*Proteobacteria*	*Gammaproteobacte*	*Enterobacterales*	*Enterobacteriaceae*	*Escherichia*	*Escherichia coli*
7246	*Proteobacteria*	*Gammaproteobacte*	*Enterobacterales*	*Enterobacteriaceae*	*Escherichia*	*Escherichia coli*
7247	*Proteobacteria*	*Gammaproteobacte*	*Enterobacterales*	*Enterobacteriaceae*	*Escherichia*	*Escherichia coli*
7248	*Proteobacteria*	*Gammaproteobacte*	*Enterobacterales*	*Enterobacteriaceae*	*Escherichia*	*Escherichia coli*
7249	*Proteobacteria*	*Gammaproteobacte*	*Enterobacterales*	*Enterobacteriaceae*	*Escherichia*	*Escherichia coli*
7250	*Proteobacteria*	*Gammaproteobacte*	*Enterobacterales*	*Enterobacteriaceae*	*Escherichia*	*Escherichia coli*
7251	*Proteobacteria*	*Gammaproteobacte*	*Enterobacterales*	*Enterobacteriaceae*	*Escherichia*	*Escherichia coli*
7252	*Proteobacteria*	*Gammaproteobacte*	*Enterobacterales*	*Enterobacteriaceae*	*Escherichia*	*Escherichia coli*

Table S4. Isolated strains by culturomics

bacteriaId	phylum	class	order	family	genus	species
7253	*Proteobacteria*	*Gammaproteobacte*	*Enterobacterales*	*Enterobacteriaceae*	*Escherichia*	*Escherichia coli*
7254	*Proteobacteria*	*Gammaproteobacte*	*Enterobacterales*	*Enterobacteriaceae*	*Escherichia*	*Escherichia coli*
7255	*Proteobacteria*	*Gammaproteobacte*	*Enterobacterales*	*Enterobacteriaceae*	*Escherichia*	*Escherichia coli*
7256	*Proteobacteria*	*Gammaproteobacte*	*Enterobacterales*	*Enterobacteriaceae*	*Escherichia*	*Escherichia coli*
7257	*Proteobacteria*	*Gammaproteobacte*	*Enterobacterales*	*Enterobacteriaceae*	*Escherichia*	*Escherichia coli*
7258	*Proteobacteria*	*Gammaproteobacte*	*Enterobacterales*	*Enterobacteriaceae*	*Escherichia*	*Escherichia coli*
7259	*Proteobacteria*	*Gammaproteobacte*	*Enterobacterales*	*Enterobacteriaceae*	*Escherichia*	*Escherichia coli*
7260	*Proteobacteria*	*Gammaproteobacte*	*Enterobacterales*	*Enterobacteriaceae*	*Escherichia*	*Escherichia coli*
7261	*Proteobacteria*	*Gammaproteobacte*	*Enterobacterales*	*Enterobacteriaceae*	*Escherichia*	*Escherichia coli*
7262	*Proteobacteria*	*Gammaproteobacte*	*Enterobacterales*	*Enterobacteriaceae*	*Escherichia*	*Escherichia coli*
7263	*Proteobacteria*	*Gammaproteobacte*	*Enterobacterales*	*Enterobacteriaceae*	*Escherichia*	*Escherichia coli*
7264	*Proteobacteria*	*Gammaproteobacte*	*Enterobacterales*	*Enterobacteriaceae*	*Escherichia*	*Escherichia coli*
7265	*Proteobacteria*	*Gammaproteobacte*	*Enterobacterales*	*Enterobacteriaceae*	*Escherichia*	*Escherichia coli*
7266	*Proteobacteria*	*Gammaproteobacte*	*Enterobacterales*	*Enterobacteriaceae*	*Escherichia*	*Escherichia coli*
7267	*Proteobacteria*	*Gammaproteobacte*	*Enterobacterales*	*Enterobacteriaceae*	*Escherichia*	*Escherichia coli*
7268	*Proteobacteria*	*Gammaproteobacte*	*Enterobacterales*	*Enterobacteriaceae*	*Escherichia*	*Escherichia coli*
7269	*Proteobacteria*	*Gammaproteobacte*	*Enterobacterales*	*Enterobacteriaceae*	*Escherichia*	*Escherichia coli*
7270	*Proteobacteria*	*Gammaproteobacte*	*Enterobacterales*	*Enterobacteriaceae*	*Escherichia*	*Escherichia coli*
7271	*Proteobacteria*	*Gammaproteobacte*	*Enterobacterales*	*Enterobacteriaceae*	*Escherichia*	*Escherichia coli*
7272	*Proteobacteria*	*Gammaproteobacte*	*Enterobacterales*	*Enterobacteriaceae*	*Escherichia*	*Escherichia coli*
7273	*Proteobacteria*	*Gammaproteobacte*	*Enterobacterales*	*Enterobacteriaceae*	*Escherichia*	*Escherichia coli*
7274	*Proteobacteria*	*Gammaproteobacte*	*Enterobacterales*	*Enterobacteriaceae*	*Escherichia*	*Escherichia coli*
7275	*Proteobacteria*	*Gammaproteobacte*	*Enterobacterales*	*Enterobacteriaceae*	*Escherichia*	*Escherichia coli*
7276	*Proteobacteria*	*Gammaproteobacte*	*Enterobacterales*	*Enterobacteriaceae*	*Escherichia*	*Escherichia coli*
7277	*Proteobacteria*	*Gammaproteobacte*	*Enterobacterales*	*Enterobacteriaceae*	*Escherichia*	*Escherichia coli*
7278	*Proteobacteria*	*Gammaproteobacte*	*Enterobacterales*	*Enterobacteriaceae*	*Escherichia*	*Escherichia coli*
7279	*Proteobacteria*	*Gammaproteobacte*	*Enterobacterales*	*Enterobacteriaceae*	*Escherichia*	*Escherichia coli*
7280	*Proteobacteria*	*Gammaproteobacte*	*Enterobacterales*	*Enterobacteriaceae*	*Escherichia*	*Escherichia coli*

Table S4. Isolated strains by culturomics

bacteriaId	*phylum*	*class*	*order*	*family*	*genus*	*species*
7281	*Proteobacteria*	*Gammaproteobacte*	*Enterobacterales*	*Enterobacteriaceae*	*Escherichia*	*Escherichia coli*
7282	*Proteobacteria*	*Gammaproteobacte*	*Enterobacterales*	*Enterobacteriaceae*	*Escherichia*	*Escherichia coli*
7283	*Proteobacteria*	*Gammaproteobacte*	*Enterobacterales*	*Enterobacteriaceae*	*Escherichia*	*Escherichia coli*
7284	*Proteobacteria*	*Gammaproteobacte*	*Enterobacterales*	*Enterobacteriaceae*	*Escherichia*	*Escherichia coli*
7285	*Proteobacteria*	*Gammaproteobacte*	*Enterobacterales*	*Enterobacteriaceae*	*Escherichia*	*Escherichia coli*
7286	*Proteobacteria*	*Gammaproteobacte*	*Enterobacterales*	*Enterobacteriaceae*	*Escherichia*	*Escherichia coli*
7287	*Proteobacteria*	*Gammaproteobacte*	*Enterobacterales*	*Enterobacteriaceae*	*Escherichia*	*Escherichia coli*
7288	*Proteobacteria*	*Gammaproteobacte*	*Enterobacterales*	*Enterobacteriaceae*	*Escherichia*	*Escherichia coli*
7289	*Proteobacteria*	*Gammaproteobacte*	*Enterobacterales*	*Enterobacteriaceae*	*Escherichia*	*Escherichia coli*
7290	*Proteobacteria*	*Gammaproteobacte*	*Enterobacterales*	*Enterobacteriaceae*	*Escherichia*	*Escherichia coli*
7291	*Proteobacteria*	*Gammaproteobacte*	*Enterobacterales*	*Enterobacteriaceae*	*Escherichia*	*Escherichia coli*
7292	*Proteobacteria*	*Gammaproteobacte*	*Enterobacterales*	*Enterobacteriaceae*	*Escherichia*	*Escherichia coli*
7293	*Proteobacteria*	*Gammaproteobacte*	*Enterobacterales*	*Enterobacteriaceae*	*Escherichia*	*Escherichia coli*
7294	*Proteobacteria*	*Gammaproteobacte*	*Enterobacterales*	*Enterobacteriaceae*	*Escherichia*	*Escherichia coli*
7295	*Proteobacteria*	*Gammaproteobacte*	*Enterobacterales*	*Enterobacteriaceae*	*Escherichia*	*Escherichia coli*
7296	*Proteobacteria*	*Gammaproteobacte*	*Enterobacterales*	*Enterobacteriaceae*	*Escherichia*	*Escherichia coli*
7297	*Proteobacteria*	*Gammaproteobacte*	*Enterobacterales*	*Enterobacteriaceae*	*Escherichia*	*Escherichia coli*
7298	*Proteobacteria*	*Gammaproteobacte*	*Enterobacterales*	*Enterobacteriaceae*	*Escherichia*	*Escherichia coli*
7299	*Proteobacteria*	*Gammaproteobacte*	*Enterobacterales*	*Enterobacteriaceae*	*Escherichia*	*Escherichia coli*
7300	*Proteobacteria*	*Gammaproteobacte*	*Enterobacterales*	*Enterobacteriaceae*	*Escherichia*	*Escherichia coli*
7301	*Proteobacteria*	*Gammaproteobacte*	*Enterobacterales*	*Enterobacteriaceae*	*Escherichia*	*Escherichia coli*
7302	*Proteobacteria*	*Gammaproteobacte*	*Enterobacterales*	*Enterobacteriaceae*	*Escherichia*	*Escherichia coli*
7303	*Proteobacteria*	*Gammaproteobacte*	*Enterobacterales*	*Enterobacteriaceae*	*Escherichia*	*Escherichia coli*
7304	*Proteobacteria*	*Gammaproteobacte*	*Enterobacterales*	*Enterobacteriaceae*	*Escherichia*	*Escherichia coli*
7305	*Proteobacteria*	*Gammaproteobacte*	*Enterobacterales*	*Enterobacteriaceae*	*Escherichia*	*Escherichia coli*
7306	*Proteobacteria*	*Gammaproteobacte*	*Enterobacterales*	*Enterobacteriaceae*	*Escherichia*	*Escherichia coli*
7307	*Proteobacteria*	*Gammaproteobacte*	*Enterobacterales*	*Enterobacteriaceae*	*Escherichia*	*Escherichia coli*
7308	*Proteobacteria*	*Gammaproteobacte*	*Enterobacterales*	*Enterobacteriaceae*	*Escherichia*	*Escherichia coli*

Table S4. Isolated strains by culturomics

bacteriaId	*phylum*	*class*	*order*	*family*	*genus*	*species*
7309	*Proteobacteria*	*Gammaproteobacte*	*Enterobacterales*	*Enterobacteriaceae*	*Escherichia*	*Escherichia coli*
7310	*Proteobacteria*	*Gammaproteobacte*	*Enterobacterales*	*Enterobacteriaceae*	*Escherichia*	*Escherichia coli*
7311	*Proteobacteria*	*Gammaproteobacte*	*Enterobacterales*	*Enterobacteriaceae*	*Escherichia*	*Escherichia coli*
7312	*Proteobacteria*	*Gammaproteobacte*	*Enterobacterales*	*Enterobacteriaceae*	*Escherichia*	*Escherichia coli*
7313	*Proteobacteria*	*Gammaproteobacte*	*Enterobacterales*	*Enterobacteriaceae*	*Escherichia*	*Escherichia coli*
7314	*Proteobacteria*	*Gammaproteobacte*	*Enterobacterales*	*Enterobacteriaceae*	*Escherichia*	*Escherichia coli*
7315	*Proteobacteria*	*Gammaproteobacte*	*Enterobacterales*	*Enterobacteriaceae*	*Escherichia*	*Escherichia coli*
7316	*Proteobacteria*	*Gammaproteobacte*	*Enterobacterales*	*Enterobacteriaceae*	*Escherichia*	*Escherichia coli*
7317	*Proteobacteria*	*Gammaproteobacte*	*Enterobacterales*	*Enterobacteriaceae*	*Escherichia*	*Escherichia coli*
7318	*Proteobacteria*	*Gammaproteobacte*	*Enterobacterales*	*Enterobacteriaceae*	*Escherichia*	*Escherichia coli*
7319	*Proteobacteria*	*Gammaproteobacte*	*Enterobacterales*	*Enterobacteriaceae*	*Escherichia*	*Escherichia coli*
7320	*Proteobacteria*	*Gammaproteobacte*	*Enterobacterales*	*Enterobacteriaceae*	*Escherichia*	*Escherichia coli*
7321	*Proteobacteria*	*Gammaproteobacte*	*Enterobacterales*	*Enterobacteriaceae*	*Escherichia*	*Escherichia coli*
7322	*Proteobacteria*	*Gammaproteobacte*	*Enterobacterales*	*Enterobacteriaceae*	*Escherichia*	*Escherichia coli*
7323	*Proteobacteria*	*Gammaproteobacte*	*Enterobacterales*	*Enterobacteriaceae*	*Escherichia*	*Escherichia coli*
7324	*Proteobacteria*	*Gammaproteobacte*	*Enterobacterales*	*Enterobacteriaceae*	*Escherichia*	*Escherichia coli*
7325	*Proteobacteria*	*Gammaproteobacte*	*Enterobacterales*	*Enterobacteriaceae*	*Escherichia*	*Escherichia coli*
7326	*Proteobacteria*	*Gammaproteobacte*	*Enterobacterales*	*Enterobacteriaceae*	*Escherichia*	*Escherichia coli*
7327	*Proteobacteria*	*Gammaproteobacte*	*Enterobacterales*	*Enterobacteriaceae*	*Escherichia*	*Escherichia coli*
7328	*Proteobacteria*	*Gammaproteobacte*	*Enterobacterales*	*Enterobacteriaceae*	*Escherichia*	*Escherichia coli*
7329	*Proteobacteria*	*Gammaproteobacte*	*Enterobacterales*	*Enterobacteriaceae*	*Escherichia*	*Escherichia coli*
7330	*Proteobacteria*	*Gammaproteobacte*	*Enterobacterales*	*Enterobacteriaceae*	*Escherichia*	*Escherichia coli*
7331	*Proteobacteria*	*Gammaproteobacte*	*Enterobacterales*	*Enterobacteriaceae*	*Escherichia*	*Escherichia coli*
7332	*Proteobacteria*	*Gammaproteobacte*	*Enterobacterales*	*Enterobacteriaceae*	*Escherichia*	*Escherichia coli*
7333	*Proteobacteria*	*Gammaproteobacte*	*Enterobacterales*	*Enterobacteriaceae*	*Escherichia*	*Escherichia coli*
7334	*Proteobacteria*	*Gammaproteobacte*	*Enterobacterales*	*Enterobacteriaceae*	*Escherichia*	*Escherichia coli*
7335	*Proteobacteria*	*Gammaproteobacte*	*Enterobacterales*	*Enterobacteriaceae*	*Escherichia*	*Escherichia coli*
7336	*Proteobacteria*	*Gammaproteobacte*	*Enterobacterales*	*Enterobacteriaceae*	*Escherichia*	*Escherichia coli*

Table S4. Isolated strains by culturomics

bacteriaId	*phylum*	*class*	*order*	*family*	*genus*	*species*
7337	*Proteobacteria*	*Gammaproteobacte*	*Enterobacterales*	*Enterobacteriaceae*	*Escherichia*	*Escherichia coli*
7338	*Proteobacteria*	*Gammaproteobacte*	*Enterobacterales*	*Enterobacteriaceae*	*Escherichia*	*Escherichia coli*
7339	*Proteobacteria*	*Gammaproteobacte*	*Enterobacterales*	*Enterobacteriaceae*	*Escherichia*	*Escherichia coli*
7340	*Proteobacteria*	*Gammaproteobacte*	*Enterobacterales*	*Enterobacteriaceae*	*Escherichia*	*Escherichia coli*
7341	*Proteobacteria*	*Gammaproteobacte*	*Enterobacterales*	*Enterobacteriaceae*	*Escherichia*	*Escherichia coli*
7342	*Proteobacteria*	*Gammaproteobacte*	*Enterobacterales*	*Enterobacteriaceae*	*Escherichia*	*Escherichia coli*
7343	*Proteobacteria*	*Gammaproteobacte*	*Enterobacterales*	*Enterobacteriaceae*	*Escherichia*	*Escherichia coli*
7344	*Proteobacteria*	*Gammaproteobacte*	*Enterobacterales*	*Enterobacteriaceae*	*Escherichia*	*Escherichia coli*
7345	*Proteobacteria*	*Gammaproteobacte*	*Enterobacterales*	*Enterobacteriaceae*	*Escherichia*	*Escherichia coli*
7346	*Proteobacteria*	*Gammaproteobacte*	*Enterobacterales*	*Enterobacteriaceae*	*Escherichia*	*Escherichia coli*
7347	*Proteobacteria*	*Gammaproteobacte*	*Enterobacterales*	*Enterobacteriaceae*	*Escherichia*	*Escherichia coli*
7348	*Proteobacteria*	*Gammaproteobacte*	*Enterobacterales*	*Enterobacteriaceae*	*Escherichia*	*Escherichia coli*
7349	*Proteobacteria*	*Gammaproteobacte*	*Enterobacterales*	*Enterobacteriaceae*	*Escherichia*	*Escherichia coli*
7350	*Proteobacteria*	*Gammaproteobacte*	*Enterobacterales*	*Enterobacteriaceae*	*Escherichia*	*Escherichia coli*
7351	*Proteobacteria*	*Gammaproteobacte*	*Enterobacterales*	*Enterobacteriaceae*	*Escherichia*	*Escherichia coli*
7352	*Proteobacteria*	*Gammaproteobacte*	*Enterobacterales*	*Enterobacteriaceae*	*Escherichia*	*Escherichia coli*
7353	*Proteobacteria*	*Gammaproteobacte*	*Enterobacterales*	*Enterobacteriaceae*	*Escherichia*	*Escherichia coli*
7354	*Proteobacteria*	*Gammaproteobacte*	*Enterobacterales*	*Enterobacteriaceae*	*Escherichia*	*Escherichia coli*
7355	*Proteobacteria*	*Gammaproteobacte*	*Enterobacterales*	*Enterobacteriaceae*	*Escherichia*	*Escherichia coli*
7356	*Proteobacteria*	*Gammaproteobacte*	*Enterobacterales*	*Enterobacteriaceae*	*Escherichia*	*Escherichia coli*
7357	*Proteobacteria*	*Gammaproteobacte*	*Enterobacterales*	*Enterobacteriaceae*	*Escherichia*	*Escherichia coli*
7358	*Proteobacteria*	*Gammaproteobacte*	*Enterobacterales*	*Enterobacteriaceae*	*Escherichia*	*Escherichia coli*
7359	*Proteobacteria*	*Gammaproteobacte*	*Enterobacterales*	*Enterobacteriaceae*	*Escherichia*	*Escherichia coli*
7360	*Proteobacteria*	*Gammaproteobacte*	*Enterobacterales*	*Enterobacteriaceae*	*Escherichia*	*Escherichia coli*
7361	*Proteobacteria*	*Gammaproteobacte*	*Enterobacterales*	*Enterobacteriaceae*	*Escherichia*	*Escherichia coli*
7362	*Proteobacteria*	*Gammaproteobacte*	*Enterobacterales*	*Enterobacteriaceae*	*Escherichia*	*Escherichia coli*
7363	*Proteobacteria*	*Gammaproteobacte*	*Enterobacterales*	*Enterobacteriaceae*	*Escherichia*	*Escherichia coli*
7364	*Proteobacteria*	*Gammaproteobacte*	*Enterobacterales*	*Enterobacteriaceae*	*Escherichia*	*Escherichia coli*

Table S4. Isolated strains by culturomics

bacterialId	*phylum*	*class*	*order*	*family*	*genus*	*species*
7365	*Proteobacteria*	*Gammaproteobacte*	*Enterobacterales*	*Enterobacteriaceae*	*Escherichia*	*Escherichia coli*
7366	*Proteobacteria*	*Gammaproteobacte*	*Enterobacterales*	*Enterobacteriaceae*	*Escherichia*	*Escherichia coli*
7367	*Proteobacteria*	*Gammaproteobacte*	*Enterobacterales*	*Enterobacteriaceae*	*Escherichia*	*Escherichia coli*
7368	*Proteobacteria*	*Gammaproteobacte*	*Enterobacterales*	*Enterobacteriaceae*	*Escherichia*	*Escherichia coli*
7369	*Proteobacteria*	*Gammaproteobacte*	*Enterobacterales*	*Enterobacteriaceae*	*Escherichia*	*Escherichia coli*
7370	*Proteobacteria*	*Gammaproteobacte*	*Enterobacterales*	*Enterobacteriaceae*	*Escherichia*	*Escherichia coli*
7371	*Proteobacteria*	*Gammaproteobacte*	*Enterobacterales*	*Enterobacteriaceae*	*Escherichia*	*Escherichia coli*
7372	*Proteobacteria*	*Gammaproteobacte*	*Enterobacterales*	*Enterobacteriaceae*	*Escherichia*	*Escherichia coli*
7373	*Proteobacteria*	*Gammaproteobacte*	*Enterobacterales*	*Enterobacteriaceae*	*Escherichia*	*Escherichia coli*
7374	*Proteobacteria*	*Gammaproteobacte*	*Enterobacterales*	*Enterobacteriaceae*	*Escherichia*	*Escherichia coli*
7375	*Proteobacteria*	*Gammaproteobacte*	*Enterobacterales*	*Enterobacteriaceae*	*Escherichia*	*Escherichia coli*
7376	*Proteobacteria*	*Gammaproteobacte*	*Enterobacterales*	*Enterobacteriaceae*	*Escherichia*	*Escherichia coli*
7377	*Proteobacteria*	*Gammaproteobacte*	*Enterobacterales*	*Enterobacteriaceae*	*Escherichia*	*Escherichia coli*
7378	*Proteobacteria*	*Gammaproteobacte*	*Enterobacterales*	*Enterobacteriaceae*	*Escherichia*	*Escherichia coli*
7379	*Proteobacteria*	*Gammaproteobacte*	*Enterobacterales*	*Enterobacteriaceae*	*Escherichia*	*Escherichia coli*
7380	*Proteobacteria*	*Gammaproteobacte*	*Enterobacterales*	*Enterobacteriaceae*	*Escherichia*	*Escherichia coli*
7381	*Proteobacteria*	*Gammaproteobacte*	*Enterobacterales*	*Enterobacteriaceae*	*Escherichia*	*Escherichia coli*
7382	*Proteobacteria*	*Gammaproteobacte*	*Enterobacterales*	*Enterobacteriaceae*	*Escherichia*	*Escherichia coli*
7383	*Proteobacteria*	*Gammaproteobacte*	*Enterobacterales*	*Enterobacteriaceae*	*Escherichia*	*Escherichia coli*
7384	*Proteobacteria*	*Gammaproteobacte*	*Enterobacterales*	*Enterobacteriaceae*	*Escherichia*	*Escherichia coli*
7385	*Proteobacteria*	*Gammaproteobacte*	*Enterobacterales*	*Enterobacteriaceae*	*Escherichia*	*Escherichia coli*
7386	*Proteobacteria*	*Gammaproteobacte*	*Enterobacterales*	*Enterobacteriaceae*	*Escherichia*	*Escherichia coli*
7387	*Proteobacteria*	*Gammaproteobacte*	*Enterobacterales*	*Enterobacteriaceae*	*Escherichia*	*Escherichia coli*
7388	*Proteobacteria*	*Gammaproteobacte*	*Enterobacterales*	*Enterobacteriaceae*	*Escherichia*	*Escherichia coli*
7389	*Proteobacteria*	*Gammaproteobacte*	*Enterobacterales*	*Enterobacteriaceae*	*Escherichia*	*Escherichia coli*
7390	*Proteobacteria*	*Gammaproteobacte*	*Enterobacterales*	*Enterobacteriaceae*	*Escherichia*	*Escherichia coli*
7391	*Proteobacteria*	*Gammaproteobacte*	*Enterobacterales*	*Enterobacteriaceae*	*Escherichia*	*Escherichia coli*
7392	*Proteobacteria*	*Gammaproteobacte*	*Enterobacterales*	*Enterobacteriaceae*	*Escherichia*	*Escherichia coli*

Table S4. Isolated strains by culturomics

bacteriaId	*phylum*	*class*	*order*	*family*	*genus*	*species*
7393	*Proteobacteria*	*Gammaproteobacte*	*Enterobacterales*	*Enterobacteriaceae*	*Klebsiella*	*Klebsiella oxytoca*
7394	*Proteobacteria*	*Gammaproteobacte*	*Enterobacterales*	*Enterobacteriaceae*	*Klebsiella*	*Klebsiella oxytoca*
7395	*Proteobacteria*	*Gammaproteobacte*	*Enterobacterales*	*Enterobacteriaceae*	*Klebsiella*	*Klebsiella oxytoca*
7396	*Proteobacteria*	*Gammaproteobacte*	*Enterobacterales*	*Enterobacteriaceae*	*Klebsiella*	*Klebsiella*
7397	*Proteobacteria*	*Gammaproteobacte*	*Enterobacterales*	*Enterobacteriaceae*	*Klebsiella*	*Klebsiella*
7398	*Proteobacteria*	*Gammaproteobacte*	*Enterobacterales*	*Enterobacteriaceae*	*Klebsiella*	*Klebsiella*
7399	*Proteobacteria*	*Gammaproteobacte*	*Enterobacterales*	*Enterobacteriaceae*	*Klebsiella*	*Klebsiella*
7400	*Proteobacteria*	*Gammaproteobacte*	*Enterobacterales*	*Enterobacteriaceae*	*Klebsiella*	*Klebsiella*
7401	*Proteobacteria*	*Gammaproteobacte*	*Enterobacterales*	*Enterobacteriaceae*	*Klebsiella*	*Klebsiella*
7402	*Proteobacteria*	*Gammaproteobacte*	*Enterobacterales*	*Enterobacteriaceae*	*Klebsiella*	*Klebsiella*
7403	*Proteobacteria*	*Gammaproteobacte*	*Enterobacterales*	*Enterobacteriaceae*	*Klebsiella*	*Klebsiella*
7404	*Proteobacteria*	*Gammaproteobacte*	*Enterobacterales*	*Enterobacteriaceae*	*Klebsiella*	*Klebsiella*
7405	*Proteobacteria*	*Gammaproteobacte*	*Enterobacterales*	*Enterobacteriaceae*	*Klebsiella*	*Klebsiella*
7406	*Proteobacteria*	*Gammaproteobacte*	*Enterobacterales*	*Enterobacteriaceae*	*Klebsiella*	*Klebsiella*
7407	*Proteobacteria*	*Gammaproteobacte*	*Enterobacterales*	*Enterobacteriaceae*	*Klebsiella*	*Klebsiella*
7408	*Proteobacteria*	*Gammaproteobacte*	*Enterobacterales*	*Enterobacteriaceae*	*Klebsiella*	*Klebsiella*
7409	*Proteobacteria*	*Gammaproteobacte*	*Enterobacterales*	*Enterobacteriaceae*	*Klebsiella*	*Klebsiella*
7410	*Proteobacteria*	*Gammaproteobacte*	*Enterobacterales*	*Enterobacteriaceae*	*Klebsiella*	*Klebsiella*
7411	*Proteobacteria*	*Gammaproteobacte*	*Enterobacterales*	*Enterobacteriaceae*	*Klebsiella*	*Klebsiella*
7412	*Proteobacteria*	*Gammaproteobacte*	*Enterobacterales*	*Enterobacteriaceae*	*Klebsiella*	*Klebsiella*
7413	*Proteobacteria*	*Gammaproteobacte*	*Enterobacterales*	*Enterobacteriaceae*	*Klebsiella*	*Klebsiella*
7414	*Proteobacteria*	*Gammaproteobacte*	*Enterobacterales*	*Enterobacteriaceae*	*Klebsiella*	*Klebsiella*
7415	*Proteobacteria*	*Gammaproteobacte*	*Enterobacterales*	*Enterobacteriaceae*	*Klebsiella*	*Klebsiella*
7416	*Proteobacteria*	*Gammaproteobacte*	*Enterobacterales*	*Enterobacteriaceae*	*Klebsiella*	*Klebsiella*
7417	*Proteobacteria*	*Gammaproteobacte*	*Enterobacterales*	*Enterobacteriaceae*	*Klebsiella*	*Klebsiella*
7418	*Proteobacteria*	*Gammaproteobacte*	*Enterobacterales*	*Enterobacteriaceae*	*Klebsiella*	*Klebsiella*
7419	*Proteobacteria*	*Gammaproteobacte*	*Enterobacterales*	*Enterobacteriaceae*	*Klebsiella*	*Klebsiella*
7420	*Proteobacteria*	*Gammaproteobacte*	*Enterobacterales*	*Enterobacteriaceae*	*Klebsiella*	*Klebsiella*

Table S4. Isolated strains by culturomics

bacteriald	*phylum*	*class*	*order*	*family*	*genus*	*species*
7421	*Proteobacteria*	*Gammaproteobacte*	*Enterobacterales*	*Enterobacteriaceae*	*Klebsiella*	*Klebsiella*
7422	*Proteobacteria*	*Gammaproteobacte*	*Enterobacterales*	*Enterobacteriaceae*	*Klebsiella*	*Klebsiella*
7423	*Proteobacteria*	*Gammaproteobacte*	*Enterobacterales*	*Enterobacteriaceae*	*Klebsiella*	*Klebsiella*
7424	*Proteobacteria*	*Gammaproteobacte*	*Enterobacterales*	*Enterobacteriaceae*	*Klebsiella*	*Klebsiella*
7425	*Proteobacteria*	*Gammaproteobacte*	*Enterobacterales*	*Enterobacteriaceae*	*Klebsiella*	*Klebsiella*
7426	*Proteobacteria*	*Gammaproteobacte*	*Enterobacterales*	*Enterobacteriaceae*	*Klebsiella*	*Klebsiella*
7427	*Proteobacteria*	*Gammaproteobacte*	*Enterobacterales*	*Enterobacteriaceae*	*Klebsiella*	*Klebsiella*
7428	*Proteobacteria*	*Gammaproteobacte*	*Enterobacterales*	*Enterobacteriaceae*	*Klebsiella*	*Klebsiella*
7429	*Proteobacteria*	*Gammaproteobacte*	*Enterobacterales*	*Enterobacteriaceae*	*Klebsiella*	*Klebsiella*
7430	*Proteobacteria*	*Gammaproteobacte*	*Enterobacterales*	*Enterobacteriaceae*	*Klebsiella*	*Klebsiella*
7431	*Proteobacteria*	*Gammaproteobacte*	*Enterobacterales*	*Enterobacteriaceae*	*Klebsiella*	*Klebsiella*
7432	*Proteobacteria*	*Gammaproteobacte*	*Enterobacterales*	*Enterobacteriaceae*	*Klebsiella*	*Klebsiella*
7433	*Proteobacteria*	*Gammaproteobacte*	*Enterobacterales*	*Enterobacteriaceae*	*Klebsiella*	*Klebsiella*
7434	*Proteobacteria*	*Gammaproteobacte*	*Enterobacterales*	*Enterobacteriaceae*	*Klebsiella*	*Klebsiella*
7435	*Proteobacteria*	*Gammaproteobacte*	*Enterobacterales*	*Enterobacteriaceae*	*Klebsiella*	*Klebsiella*
7436	*Proteobacteria*	*Gammaproteobacte*	*Enterobacterales*	*Enterobacteriaceae*	*Klebsiella*	*Klebsiella*
7437	*Proteobacteria*	*Gammaproteobacte*	*Enterobacterales*	*Enterobacteriaceae*	*Klebsiella*	*Klebsiella*
7438	*Proteobacteria*	*Gammaproteobacte*	*Enterobacterales*	*Enterobacteriaceae*	*Klebsiella*	*Klebsiella*
7439	*Proteobacteria*	*Gammaproteobacte*	*Enterobacterales*	*Enterobacteriaceae*	*Klebsiella*	*Klebsiella*
7440	*Proteobacteria*	*Gammaproteobacte*	*Enterobacterales*	*Enterobacteriaceae*	*Klebsiella*	*Klebsiella*
7441	*Proteobacteria*	*Gammaproteobacte*	*Enterobacterales*	*Enterobacteriaceae*	*Klebsiella*	*Klebsiella*
7442	*Proteobacteria*	*Gammaproteobacte*	*Enterobacterales*	*Enterobacteriaceae*	*Klebsiella*	*Klebsiella*
7443	*Proteobacteria*	*Gammaproteobacte*	*Enterobacterales*	*Enterobacteriaceae*	*Klebsiella*	*Klebsiella*
7444	*Proteobacteria*	*Gammaproteobacte*	*Enterobacterales*	*Enterobacteriaceae*	*Klebsiella*	*Klebsiella*
7445	*Proteobacteria*	*Gammaproteobacte*	*Enterobacterales*	*Enterobacteriaceae*	*Klebsiella*	*Klebsiella*
7446	*Proteobacteria*	*Gammaproteobacte*	*Enterobacterales*	*Enterobacteriaceae*	*Klebsiella*	*Klebsiella*
7447	*Proteobacteria*	*Gammaproteobacte*	*Enterobacterales*	*Enterobacteriaceae*	*Klebsiella*	*Klebsiella*
7448	*Proteobacteria*	*Gammaproteobacte*	*Enterobacterales*	*Enterobacteriaceae*	*Klebsiella*	*Klebsiella*

Table S4. Isolated strains by culturomics

bacteriaId	*phylum*	*class*	*order*	*family*	*genus*	*species*
7449	*Proteobacteria*	*Gammaproteobacte*	*Enterobacterales*	*Enterobacteriaceae*	*Klebsiella*	*Klebsiella*
7450	*Proteobacteria*	*Gammaproteobacte*	*Enterobacterales*	*Enterobacteriaceae*	*Klebsiella*	*Klebsiella*
7451	*Proteobacteria*	*Gammaproteobacte*	*Enterobacterales*	*Enterobacteriaceae*	*Klebsiella*	*Klebsiella*
7452	*Proteobacteria*	*Gammaproteobacte*	*Enterobacterales*	*Enterobacteriaceae*	*Klebsiella*	*Klebsiella*
7453	*Proteobacteria*	*Gammaproteobacte*	*Enterobacterales*	*Enterobacteriaceae*	*Klebsiella*	*Klebsiella*
7454	*Proteobacteria*	*Gammaproteobacte*	*Enterobacterales*	*Enterobacteriaceae*	*Klebsiella*	*Klebsiella*
7455	*Proteobacteria*	*Gammaproteobacte*	*Enterobacterales*	*Enterobacteriaceae*	*Klebsiella*	*Klebsiella*
7456	*Proteobacteria*	*Gammaproteobacte*	*Enterobacterales*	*Enterobacteriaceae*	*Klebsiella*	*Klebsiella*
7457	*Proteobacteria*	*Gammaproteobacte*	*Enterobacterales*	*Enterobacteriaceae*	*Klebsiella*	*Klebsiella*
7458	*Proteobacteria*	*Gammaproteobacte*	*Enterobacterales*	*Enterobacteriaceae*	*Klebsiella*	*Klebsiella*
7459	*Proteobacteria*	*Gammaproteobacte*	*Enterobacterales*	*Enterobacteriaceae*	*Klebsiella*	*Klebsiella*
7460	*Proteobacteria*	*Gammaproteobacte*	*Enterobacterales*	*Enterobacteriaceae*	*Klebsiella*	*Klebsiella*
7461	*Proteobacteria*	*Gammaproteobacte*	*Enterobacterales*	*Enterobacteriaceae*	*Klebsiella*	*Klebsiella*
7462	*Proteobacteria*	*Gammaproteobacte*	*Enterobacterales*	*Enterobacteriaceae*	*Klebsiella*	*Klebsiella*
7463	*Proteobacteria*	*Gammaproteobacte*	*Enterobacterales*	*Enterobacteriaceae*	*Klebsiella*	*Klebsiella*
7464	*Proteobacteria*	*Gammaproteobacte*	*Enterobacterales*	*Enterobacteriaceae*	*Klebsiella*	*Klebsiella*
7465	*Proteobacteria*	*Gammaproteobacte*	*Enterobacterales*	*Enterobacteriaceae*	*Klebsiella*	*Klebsiella*
7466	*Proteobacteria*	*Gammaproteobacte*	*Enterobacterales*	*Enterobacteriaceae*	*Klebsiella*	*Klebsiella*
7467	*Proteobacteria*	*Gammaproteobacte*	*Enterobacterales*	*Enterobacteriaceae*	*Klebsiella*	*Klebsiella*
7468	*Proteobacteria*	*Gammaproteobacte*	*Enterobacterales*	*Enterobacteriaceae*	*Klebsiella*	*Klebsiella*
7469	*Proteobacteria*	*Gammaproteobacte*	*Enterobacterales*	*Enterobacteriaceae*	*Klebsiella*	*Klebsiella*
7470	*Proteobacteria*	*Gammaproteobacte*	*Enterobacterales*	*Enterobacteriaceae*	*Klebsiella*	*Klebsiella variicola*
7471	*Proteobacteria*	*Gammaproteobacte*	*Enterobacterales*	*Enterobacteriaceae*	*Raoultella*	*Raoultella*
7472	*Proteobacteria*	*Gammaproteobacte*	*Enterobacterales*	*Enterobacteriaceae*	*Salmonella*	*Salmonella sp.*
7473	*Proteobacteria*	*Gammaproteobacte*	*Enterobacterales*	*Enterobacteriaceae*	*Salmonella*	*Salmonella sp.*
7474	*Proteobacteria*	*Gammaproteobacte*	*Enterobacterales*	*Enterobacteriaceae*	*Salmonella*	*Salmonella sp.*
7475	*Proteobacteria*	*Gammaproteobacte*	*Enterobacterales*	*Enterobacteriaceae*	*Salmonella*	*Salmonella sp.*
7476	*Proteobacteria*	*Gammaproteobacte*	*Enterobacterales*	*Enterobacteriaceae*	*Salmonella*	*Salmonella sp.*

Table S4. Isolated strains by culturomics

bacteriaId	*phylum*	*class*	*order*	*family*	*genus*	*species*
7477	*Proteobacteria*	*Gammaproteobacte*	*Enterobacterales*	*Enterobacteriaceae*	*Salmonella*	*Salmonella sp.*
7478	*Proteobacteria*	*Gammaproteobacte*	*Enterobacterales*	*Enterobacteriaceae*	*Salmonella*	*Salmonella sp.*
7479	*Proteobacteria*	*Gammaproteobacte*	*Enterobacterales*	*Enterobacteriaceae*	*Salmonella*	*Salmonella sp.*
7480	*Proteobacteria*	*Gammaproteobacte*	*Enterobacterales*	*Enterobacteriaceae*	*Salmonella*	*Salmonella sp.*
7481	*Proteobacteria*	*Gammaproteobacte*	*Enterobacterales*	*Enterobacteriaceae*	*Trabulsiella*	*Trabulsiella*
7482	*Proteobacteria*	*Gammaproteobacte*	*Enterobacterales*	*Hafniaceae*	*Hafnia*	*Hafnia alvei*
7483	*Proteobacteria*	*Gammaproteobacte*	*Enterobacterales*	*Hafniaceae*	*Hafnia*	*Hafnia alvei*
7484	*Proteobacteria*	*Gammaproteobacte*	*Enterobacterales*	*Hafniaceae*	*Hafnia*	*Hafnia alvei*
7485	*Proteobacteria*	*Gammaproteobacte*	*Enterobacterales*	*Hafniaceae*	*Hafnia*	*Hafnia alvei*
7486	*Proteobacteria*	*Gammaproteobacte*	*Enterobacterales*	*Hafniaceae*	*Hafnia*	*Hafnia alvei*
7487	*Proteobacteria*	*Gammaproteobacte*	*Enterobacterales*	*Hafniaceae*	*Hafnia*	*Hafnia alvei*
7488	*Proteobacteria*	*Gammaproteobacte*	*Enterobacterales*	*Hafniaceae*	*Hafnia*	*Hafnia alvei*
7489	*Proteobacteria*	*Gammaproteobacte*	*Enterobacterales*	*Hafniaceae*	*Hafnia*	*Hafnia alvei*
7490	*Proteobacteria*	*Gammaproteobacte*	*Enterobacterales*	*Hafniaceae*	*Hafnia*	*Hafnia alvei*
7491	*Proteobacteria*	*Gammaproteobacte*	*Enterobacterales*	*Hafniaceae*	*Hafnia*	*Hafnia alvei*
7492	*Proteobacteria*	*Gammaproteobacte*	*Enterobacterales*	*Hafniaceae*	*Hafnia*	*Hafnia alvei*
7493	*Proteobacteria*	*Gammaproteobacte*	*Enterobacterales*	*Hafniaceae*	*Hafnia*	*Hafnia alvei*
7494	*Proteobacteria*	*Gammaproteobacte*	*Enterobacterales*	*Hafniaceae*	*Hafnia*	*Hafnia alvei*
7495	*Proteobacteria*	*Gammaproteobacte*	*Enterobacterales*	*Hafniaceae*	*Hafnia*	*Hafnia alvei*
7496	*Proteobacteria*	*Gammaproteobacte*	*Enterobacterales*	*Hafniaceae*	*Hafnia*	*Hafnia alvei*
7497	*Proteobacteria*	*Gammaproteobacte*	*Enterobacterales*	*Hafniaceae*	*Hafnia*	*Hafnia alvei*
7498	*Proteobacteria*	*Gammaproteobacte*	*Enterobacterales*	*Hafniaceae*	*Hafnia*	*Hafnia alvei*
7499	*Proteobacteria*	*Gammaproteobacte*	*Enterobacterales*	*Hafniaceae*	*Hafnia*	*Hafnia alvei*
7500	*Proteobacteria*	*Gammaproteobacte*	*Enterobacterales*	*Hafniaceae*	*Hafnia*	*Hafnia alvei*
7501	*Proteobacteria*	*Gammaproteobacte*	*Enterobacterales*	*Hafniaceae*	*Hafnia*	*Hafnia alvei*
7502	*Proteobacteria*	*Gammaproteobacte*	*Enterobacterales*	*Hafniaceae*	*Hafnia*	*Hafnia alvei*
7503	*Proteobacteria*	*Gammaproteobacte*	*Enterobacterales*	*Hafniaceae*	*Hafnia*	*Hafnia alvei*
7504	*Proteobacteria*	*Gammaproteobacte*	*Enterobacterales*	*Hafniaceae*	*Hafnia*	*Hafnia alvei*

Table S4. Isolated strains by culturomics

bacteriaId	*phylum*	*class*	*order*	*family*	*genus*	*species*
7505	*Proteobacteria*	*Gammaproteobacte*	*Enterobacterales*	*Hafniaceae*	*Hafnia*	*Hafnia alvei*
7506	*Proteobacteria*	*Gammaproteobacte*	*Enterobacterales*	*Hafniaceae*	*Hafnia*	*Hafnia alvei*
7507	*Proteobacteria*	*Gammaproteobacte*	*Enterobacterales*	*Hafniaceae*	*Hafnia*	*Hafnia alvei*
7508	*Proteobacteria*	*Gammaproteobacte*	*Enterobacterales*	*Hafniaceae*	*Hafnia*	*Hafnia alvei*
7509	*Proteobacteria*	*Gammaproteobacte*	*Enterobacterales*	*Hafniaceae*	*Hafnia*	*Hafnia alvei*
7510	*Proteobacteria*	*Gammaproteobacte*	*Enterobacterales*	*Hafniaceae*	*Hafnia*	*Hafnia alvei*
7511	*Proteobacteria*	*Gammaproteobacte*	*Enterobacterales*	*Hafniaceae*	*Hafnia*	*Hafnia alvei*
7512	*Proteobacteria*	*Gammaproteobacte*	*Enterobacterales*	*Hafniaceae*	*Hafnia*	*Hafnia alvei*
7513	*Proteobacteria*	*Gammaproteobacte*	*Enterobacterales*	*Hafniaceae*	*Hafnia*	*Hafnia alvei*
7514	*Proteobacteria*	*Gammaproteobacte*	*Enterobacterales*	*Hafniaceae*	*Hafnia*	*Hafnia alvei*
7515	*Proteobacteria*	*Gammaproteobacte*	*Enterobacterales*	*Hafniaceae*	*Hafnia*	*Hafnia alvei*
7516	*Proteobacteria*	*Gammaproteobacte*	*Enterobacterales*	*Hafniaceae*	*Hafnia*	*Hafnia alvei*
7517	*Proteobacteria*	*Gammaproteobacte*	*Enterobacterales*	*Hafniaceae*	*Hafnia*	*Hafnia alvei*
7518	*Proteobacteria*	*Gammaproteobacte*	*Enterobacterales*	*Hafniaceae*	*Hafnia*	*Hafnia alvei*
7519	*Proteobacteria*	*Gammaproteobacte*	*Enterobacterales*	*Hafniaceae*	*Hafnia*	*Hafnia alvei*
7520	*Proteobacteria*	*Gammaproteobacte*	*Enterobacterales*	*Hafniaceae*	*Hafnia*	*Hafnia alvei*
7521	*Proteobacteria*	*Gammaproteobacte*	*Enterobacterales*	*Hafniaceae*	*Hafnia*	*Hafnia alvei*
7522	*Proteobacteria*	*Gammaproteobacte*	*Enterobacterales*	*Hafniaceae*	*Hafnia*	*Hafnia alvei*
7523	*Proteobacteria*	*Gammaproteobacte*	*Enterobacterales*	*Hafniaceae*	*Hafnia*	*Hafnia alvei*
7524	*Proteobacteria*	*Gammaproteobacte*	*Enterobacterales*	*Hafniaceae*	*Hafnia*	*Hafnia alvei*
7525	*Proteobacteria*	*Gammaproteobacte*	*Enterobacterales*	*Hafniaceae*	*Hafnia*	*Hafnia alvei*
7526	*Proteobacteria*	*Gammaproteobacte*	*Enterobacterales*	*Hafniaceae*	*Hafnia*	*Hafnia alvei*
7527	*Proteobacteria*	*Gammaproteobacte*	*Enterobacterales*	*Hafniaceae*	*Hafnia*	*Hafnia alvei*
7528	*Proteobacteria*	*Gammaproteobacte*	*Enterobacterales*	*Hafniaceae*	*Hafnia*	*Hafnia alvei*
7529	*Proteobacteria*	*Gammaproteobacte*	*Enterobacterales*	*Hafniaceae*	*Hafnia*	*Hafnia alvei*
7530	*Proteobacteria*	*Gammaproteobacte*	*Enterobacterales*	*Hafniaceae*	*Hafnia*	*Hafnia alvei*
7531	*Proteobacteria*	*Gammaproteobacte*	*Enterobacterales*	*Hafniaceae*	*Hafnia*	*Hafnia alvei*
7532	*Proteobacteria*	*Gammaproteobacte*	*Enterobacterales*	*Hafniaceae*	*Hafnia*	*Hafnia alvei*

Table S4. Isolated strains by culturomics

bacteriaId	*phylum*	*class*	*order*	*family*	*genus*	*species*
7533	*Proteobacteria*	*Gammaproteobacte*	*Enterobacterales*	*Hafniaceae*	*Hafnia*	*Hafnia alvei*
7534	*Proteobacteria*	*Gammaproteobacte*	*Enterobacterales*	*Hafniaceae*	*Hafnia*	*Hafnia alvei*
7535	*Proteobacteria*	*Gammaproteobacte*	*Enterobacterales*	*Hafniaceae*	*Hafnia*	*Hafnia alvei*
7536	*Proteobacteria*	*Gammaproteobacte*	*Enterobacterales*	*Hafniaceae*	*Hafnia*	*Hafnia alvei*
7537	*Proteobacteria*	*Gammaproteobacte*	*Enterobacterales*	*Hafniaceae*	*Hafnia*	*Hafnia alvei*
7538	*Proteobacteria*	*Gammaproteobacte*	*Enterobacterales*	*Hafniaceae*	*Hafnia*	*Hafnia alvei*
7539	*Proteobacteria*	*Gammaproteobacte*	*Enterobacterales*	*Hafniaceae*	*Hafnia*	*Hafnia alvei*
7540	*Proteobacteria*	*Gammaproteobacte*	*Enterobacterales*	*Hafniaceae*	*Hafnia*	*Hafnia alvei*
7541	*Proteobacteria*	*Gammaproteobacte*	*Enterobacterales*	*Hafniaceae*	*Hafnia*	*Hafnia alvei*
7542	*Proteobacteria*	*Gammaproteobacte*	*Enterobacterales*	*Hafniaceae*	*Hafnia*	*Hafnia alvei*
7543	*Proteobacteria*	*Gammaproteobacte*	*Enterobacterales*	*Hafniaceae*	*Hafnia*	*Hafnia alvei*
7544	*Proteobacteria*	*Gammaproteobacte*	*Enterobacterales*	*Hafniaceae*	*Hafnia*	*Hafnia alvei*
7545	*Proteobacteria*	*Gammaproteobacte*	*Enterobacterales*	*Hafniaceae*	*Hafnia*	*Hafnia alvei*
7546	*Proteobacteria*	*Gammaproteobacte*	*Enterobacterales*	*Hafniaceae*	*Hafnia*	*Hafnia alvei*
7547	*Proteobacteria*	*Gammaproteobacte*	*Enterobacterales*	*Hafniaceae*	*Hafnia*	*Hafnia alvei*
7548	*Proteobacteria*	*Gammaproteobacte*	*Enterobacterales*	*Hafniaceae*	*Hafnia*	*Hafnia alvei*
7549	*Proteobacteria*	*Gammaproteobacte*	*Enterobacterales*	*Hafniaceae*	*Hafnia*	*Hafnia alvei*
7550	*Proteobacteria*	*Gammaproteobacte*	*Enterobacterales*	*Hafniaceae*	*Hafnia*	*Hafnia alvei*
7551	*Proteobacteria*	*Gammaproteobacte*	*Enterobacterales*	*Hafniaceae*	*Hafnia*	*Hafnia alvei*
7552	*Proteobacteria*	*Gammaproteobacte*	*Enterobacterales*	*Hafniaceae*	*Hafnia*	*Hafnia alvei*
7553	*Proteobacteria*	*Gammaproteobacte*	*Enterobacterales*	*Hafniaceae*	*Hafnia*	*Hafnia alvei*
7554	*Proteobacteria*	*Gammaproteobacte*	*Enterobacterales*	*Hafniaceae*	*Hafnia*	*Hafnia alvei*
7555	*Proteobacteria*	*Gammaproteobacte*	*Enterobacterales*	*Hafniaceae*	*Hafnia*	*Hafnia alvei*
7556	*Proteobacteria*	*Gammaproteobacte*	*Enterobacterales*	*Hafniaceae*	*Hafnia*	*Hafnia alvei*
7557	*Proteobacteria*	*Gammaproteobacte*	*Enterobacterales*	*Hafniaceae*	*Hafnia*	*Hafnia alvei*
7558	*Proteobacteria*	*Gammaproteobacte*	*Enterobacterales*	*Hafniaceae*	*Hafnia*	*Hafnia alvei*
7559	*Proteobacteria*	*Gammaproteobacte*	*Enterobacterales*	*Hafniaceae*	*Hafnia*	*Hafnia alvei*
7560	*Proteobacteria*	*Gammaproteobacte*	*Enterobacterales*	*Hafniaceae*	*Hafnia*	*Hafnia alvei*

Table S4. Isolated strains by culturomics

bacteriaId	*phylum*	*class*	*order*	*family*	*genus*	*species*
7561	*Proteobacteria*	*Gammaproteobacte*	*Enterobacterales*	*Hafniaceae*	*Hafnia*	*Hafnia alvei*
7562	*Proteobacteria*	*Gammaproteobacte*	*Enterobacterales*	*Hafniaceae*	*Hafnia*	*Hafnia alvei*
7563	*Proteobacteria*	*Gammaproteobacte*	*Enterobacterales*	*Hafniaceae*	*Hafnia*	*Hafnia alvei*
7564	*Proteobacteria*	*Gammaproteobacte*	*Enterobacterales*	*Hafniaceae*	*Hafnia*	*Hafnia alvei*
7565	*Proteobacteria*	*Gammaproteobacte*	*Enterobacterales*	*Hafniaceae*	*Hafnia*	*Hafnia alvei*
7566	*Proteobacteria*	*Gammaproteobacte*	*Enterobacterales*	*Hafniaceae*	*Hafnia*	*Hafnia alvei*
7567	*Proteobacteria*	*Gammaproteobacte*	*Enterobacterales*	*Hafniaceae*	*Hafnia*	*Hafnia alvei*
7568	*Proteobacteria*	*Gammaproteobacte*	*Enterobacterales*	*Hafniaceae*	*Hafnia*	*Hafnia alvei*
7569	*Proteobacteria*	*Gammaproteobacte*	*Enterobacterales*	*Hafniaceae*	*Hafnia*	*Hafnia alvei*
7570	*Proteobacteria*	*Gammaproteobacte*	*Enterobacterales*	*Hafniaceae*	*Hafnia*	*Hafnia alvei*
7571	*Proteobacteria*	*Gammaproteobacte*	*Enterobacterales*	*Hafniaceae*	*Hafnia*	*Hafnia alvei*
7572	*Proteobacteria*	*Gammaproteobacte*	*Enterobacterales*	*Hafniaceae*	*Hafnia*	*Hafnia alvei*
7573	*Proteobacteria*	*Gammaproteobacte*	*Enterobacterales*	*Hafniaceae*	*Hafnia*	*Hafnia alvei*
7574	*Proteobacteria*	*Gammaproteobacte*	*Enterobacterales*	*Hafniaceae*	*Hafnia*	*Hafnia alvei*
7575	*Proteobacteria*	*Gammaproteobacte*	*Enterobacterales*	*Hafniaceae*	*Hafnia*	*Hafnia alvei*
7576	*Proteobacteria*	*Gammaproteobacte*	*Enterobacterales*	*Hafniaceae*	*Hafnia*	*Hafnia alvei*
7577	*Proteobacteria*	*Gammaproteobacte*	*Enterobacterales*	*Hafniaceae*	*Hafnia*	*Hafnia alvei*
7578	*Proteobacteria*	*Gammaproteobacte*	*Enterobacterales*	*Hafniaceae*	*Hafnia*	*Hafnia alvei*
7579	*Proteobacteria*	*Gammaproteobacte*	*Enterobacterales*	*Hafniaceae*	*Hafnia*	*Hafnia alvei*
7580	*Proteobacteria*	*Gammaproteobacte*	*Enterobacterales*	*Hafniaceae*	*Hafnia*	*Hafnia alvei*
7581	*Proteobacteria*	*Gammaproteobacte*	*Enterobacterales*	*Hafniaceae*	*Hafnia*	*Hafnia alvei*
7582	*Proteobacteria*	*Gammaproteobacte*	*Enterobacterales*	*Hafniaceae*	*Hafnia*	*Hafnia alvei*
7583	*Proteobacteria*	*Gammaproteobacte*	*Enterobacterales*	*Hafniaceae*	*Hafnia*	*Hafnia alvei*
7584	*Proteobacteria*	*Gammaproteobacte*	*Enterobacterales*	*Hafniaceae*	*Hafnia*	*Hafnia alvei*
7585	*Proteobacteria*	*Gammaproteobacte*	*Enterobacterales*	*Hafniaceae*	*Hafnia*	*Hafnia alvei*
7586	*Proteobacteria*	*Gammaproteobacte*	*Enterobacterales*	*Hafniaceae*	*Hafnia*	*Hafnia alvei*
7587	*Proteobacteria*	*Gammaproteobacte*	*Enterobacterales*	*Hafniaceae*	*Hafnia*	*Hafnia alvei*
7588	*Proteobacteria*	*Gammaproteobacte*	*Enterobacterales*	*Hafniaceae*	*Hafnia*	*Hafnia alvei*

Table S4. Isolated strains by culturomics

bacterialId	*phylum*	*class*	*order*	*family*	*genus*	*species*
7589	*Proteobacteria*	*Gammaproteobacte*	*Enterobacterales*	*Hafniaceae*	*Hafnia*	*Hafnia alvei*
7590	*Proteobacteria*	*Gammaproteobacte*	*Enterobacterales*	*Hafniaceae*	*Hafnia*	*Hafnia alvei*
7591	*Proteobacteria*	*Gammaproteobacte*	*Enterobacterales*	*Hafniaceae*	*Hafnia*	*Hafnia alvei*
7592	*Proteobacteria*	*Gammaproteobacte*	*Enterobacterales*	*Hafniaceae*	*Hafnia*	*Hafnia alvei*
7593	*Proteobacteria*	*Gammaproteobacte*	*Enterobacterales*	*Hafniaceae*	*Hafnia*	*Hafnia alvei*
7594	*Proteobacteria*	*Gammaproteobacte*	*Enterobacterales*	*Hafniaceae*	*Hafnia*	*Hafnia alvei*
7595	*Proteobacteria*	*Gammaproteobacte*	*Enterobacterales*	*Hafniaceae*	*Hafnia*	*Hafnia alvei*
7596	*Proteobacteria*	*Gammaproteobacte*	*Enterobacterales*	*Hafniaceae*	*Hafnia*	*Hafnia alvei*
7597	*Proteobacteria*	*Gammaproteobacte*	*Enterobacterales*	*Hafniaceae*	*Hafnia*	*Hafnia alvei*
7598	*Proteobacteria*	*Gammaproteobacte*	*Enterobacterales*	*Hafniaceae*	*Hafnia*	*Hafnia alvei*
7599	*Proteobacteria*	*Gammaproteobacte*	*Enterobacterales*	*Hafniaceae*	*Hafnia*	*Hafnia alvei*
7600	*Proteobacteria*	*Gammaproteobacte*	*Enterobacterales*	*Hafniaceae*	*Hafnia*	*Hafnia alvei*
7601	*Proteobacteria*	*Gammaproteobacte*	*Enterobacterales*	*Hafniaceae*	*Hafnia*	*Hafnia alvei*
7602	*Proteobacteria*	*Gammaproteobacte*	*Enterobacterales*	*Hafniaceae*	*Hafnia*	*Hafnia alvei*
7603	*Proteobacteria*	*Gammaproteobacte*	*Enterobacterales*	*Hafniaceae*	*Hafnia*	*Hafnia alvei*
7604	*Proteobacteria*	*Gammaproteobacte*	*Enterobacterales*	*Hafniaceae*	*Hafnia*	*Hafnia alvei*
7605	*Proteobacteria*	*Gammaproteobacte*	*Enterobacterales*	*Hafniaceae*	*Hafnia*	*Hafnia alvei*
7606	*Proteobacteria*	*Gammaproteobacte*	*Enterobacterales*	*Hafniaceae*	*Hafnia*	*Hafnia alvei*
7607	*Proteobacteria*	*Gammaproteobacte*	*Enterobacterales*	*Hafniaceae*	*Hafnia*	*Hafnia alvei*
7608	*Proteobacteria*	*Gammaproteobacte*	*Enterobacterales*	*Hafniaceae*	*Hafnia*	*Hafnia alvei*
7609	*Proteobacteria*	*Gammaproteobacte*	*Enterobacterales*	*Hafniaceae*	*Hafnia*	*Hafnia alvei*
7610	*Proteobacteria*	*Gammaproteobacte*	*Enterobacterales*	*Hafniaceae*	*Hafnia*	*Hafnia alvei*
7611	*Proteobacteria*	*Gammaproteobacte*	*Enterobacterales*	*Hafniaceae*	*Hafnia*	*Hafnia alvei*
7612	*Proteobacteria*	*Gammaproteobacte*	*Enterobacterales*	*Hafniaceae*	*Hafnia*	*Hafnia alvei*
7613	*Proteobacteria*	*Gammaproteobacte*	*Enterobacterales*	*Hafniaceae*	*Hafnia*	*Hafnia alvei*
7614	*Proteobacteria*	*Gammaproteobacte*	*Enterobacterales*	*Hafniaceae*	*Hafnia*	*Hafnia alvei*
7615	*Proteobacteria*	*Gammaproteobacte*	*Enterobacterales*	*Hafniaceae*	*Hafnia*	*Hafnia alvei*
7616	*Proteobacteria*	*Gammaproteobacte*	*Enterobacterales*	*Hafniaceae*	*Hafnia*	*Hafnia alvei*

Table S4. Isolated strains by culturomics

bacteriaId	*phylum*	*class*	*order*	*family*	*genus*	*species*
7617	*Proteobacteria*	*Gammaproteobacte*	*Enterobacterales*	*Hafniaceae*	*Hafnia*	*Hafnia alvei*
7618	*Proteobacteria*	*Gammaproteobacte*	*Enterobacterales*	*Hafniaceae*	*Hafnia*	*Hafnia alvei*
7619	*Proteobacteria*	*Gammaproteobacte*	*Enterobacterales*	*Hafniaceae*	*Hafnia*	*Hafnia alvei*
7620	*Proteobacteria*	*Gammaproteobacte*	*Enterobacterales*	*Hafniaceae*	*Hafnia*	*Hafnia alvei*
7621	*Proteobacteria*	*Gammaproteobacte*	*Enterobacterales*	*Hafniaceae*	*Hafnia*	*Hafnia alvei*
7622	*Proteobacteria*	*Gammaproteobacte*	*Enterobacterales*	*Hafniaceae*	*Hafnia*	*Hafnia alvei*
7623	*Proteobacteria*	*Gammaproteobacte*	*Enterobacterales*	*Hafniaceae*	*Hafnia*	*Hafnia alvei*
7624	*Proteobacteria*	*Gammaproteobacte*	*Enterobacterales*	*Hafniaceae*	*Hafnia*	*Hafnia alvei*
7625	*Proteobacteria*	*Gammaproteobacte*	*Enterobacterales*	*Hafniaceae*	*Hafnia*	*Hafnia alvei*
7626	*Proteobacteria*	*Gammaproteobacte*	*Enterobacterales*	*Hafniaceae*	*Hafnia*	*Hafnia alvei*
7627	*Proteobacteria*	*Gammaproteobacte*	*Enterobacterales*	*Hafniaceae*	*Hafnia*	*Hafnia alvei*
7628	*Proteobacteria*	*Gammaproteobacte*	*Enterobacterales*	*Hafniaceae*	*Hafnia*	*Hafnia alvei*
7629	*Proteobacteria*	*Gammaproteobacte*	*Enterobacterales*	*Hafniaceae*	*Hafnia*	*Hafnia alvei*
7630	*Proteobacteria*	*Gammaproteobacte*	*Enterobacterales*	*Hafniaceae*	*Hafnia*	*Hafnia alvei*
7631	*Proteobacteria*	*Gammaproteobacte*	*Enterobacterales*	*Hafniaceae*	*Hafnia*	*Hafnia alvei*
7632	*Proteobacteria*	*Gammaproteobacte*	*Enterobacterales*	*Hafniaceae*	*Hafnia*	*Hafnia alvei*
7633	*Proteobacteria*	*Gammaproteobacte*	*Enterobacterales*	*Hafniaceae*	*Hafnia*	*Hafnia alvei*
7634	*Proteobacteria*	*Gammaproteobacte*	*Enterobacterales*	*Hafniaceae*	*Hafnia*	*Hafnia alvei*
7635	*Proteobacteria*	*Gammaproteobacte*	*Enterobacterales*	*Hafniaceae*	*Hafnia*	*Hafnia alvei*
7636	*Proteobacteria*	*Gammaproteobacte*	*Enterobacterales*	*Hafniaceae*	*Hafnia*	*Hafnia alvei*
7637	*Proteobacteria*	*Gammaproteobacte*	*Enterobacterales*	*Hafniaceae*	*Hafnia*	*Hafnia alvei*
7638	*Proteobacteria*	*Gammaproteobacte*	*Enterobacterales*	*Hafniaceae*	*Hafnia*	*Hafnia alvei*
7639	*Proteobacteria*	*Gammaproteobacte*	*Enterobacterales*	*Hafniaceae*	*Hafnia*	*Hafnia alvei*
7640	*Proteobacteria*	*Gammaproteobacte*	*Enterobacterales*	*Hafniaceae*	*Hafnia*	*Hafnia alvei*
7641	*Proteobacteria*	*Gammaproteobacte*	*Enterobacterales*	*Hafniaceae*	*Hafnia*	*Hafnia alvei*
7642	*Proteobacteria*	*Gammaproteobacte*	*Enterobacterales*	*Hafniaceae*	*Hafnia*	*Hafnia alvei*
7643	*Proteobacteria*	*Gammaproteobacte*	*Enterobacterales*	*Hafniaceae*	*Hafnia*	*Hafnia alvei*
7644	*Proteobacteria*	*Gammaproteobacte*	*Enterobacterales*	*Morganellaceae*	*Morganella*	*Morganella*

Table S4. Isolated strains by culturomics

bacterialId	*phylum*	*class*	*order*	*family*	*genus*	*species*
7645	*Proteobacteria*	*Gammaproteobacte*	*Enterobacterales*	*Morganellaceae*	*Morganella*	*Morganella*
7646	*Proteobacteria*	*Gammaproteobacte*	*Enterobacterales*	*Morganellaceae*	*Morganella*	*Morganella*
7647	*Proteobacteria*	*Gammaproteobacte*	*Enterobacterales*	*Morganellaceae*	*Proteus*	*Proteus mirabilis*
7648	*Proteobacteria*	*Gammaproteobacte*	*Enterobacterales*	*Morganellaceae*	*Proteus*	*Proteus mirabilis*
7649	*Proteobacteria*	*Gammaproteobacte*	*Enterobacterales*	*Morganellaceae*	*Proteus*	*Proteus mirabilis*
7650	*Proteobacteria*	*Gammaproteobacte*	*Enterobacterales*	*Morganellaceae*	*Proteus*	*Proteus mirabilis*
7651	*Proteobacteria*	*Gammaproteobacte*	*Enterobacterales*	*Morganellaceae*	*Proteus*	*Proteus mirabilis*
7652	*Proteobacteria*	*Gammaproteobacte*	*Enterobacterales*	*Morganellaceae*	*Proteus*	*Proteus mirabilis*
7653	*Proteobacteria*	*Gammaproteobacte*	*Enterobacterales*	*Morganellaceae*	*Proteus*	*Proteus mirabilis*
7654	*Proteobacteria*	*Gammaproteobacte*	*Enterobacterales*	*Morganellaceae*	*Proteus*	*Proteus mirabilis*
7655	*Proteobacteria*	*Gammaproteobacte*	*Enterobacterales*	*Morganellaceae*	*Proteus*	*Proteus mirabilis*
7656	*Proteobacteria*	*Gammaproteobacte*	*Enterobacterales*	*Morganellaceae*	*Proteus*	*Proteus mirabilis*
7657	*Proteobacteria*	*Gammaproteobacte*	*Enterobacterales*	*Morganellaceae*	*Proteus*	*Proteus mirabilis*
7658	*Proteobacteria*	*Gammaproteobacte*	*Enterobacterales*	*Morganellaceae*	*Proteus*	*Proteus mirabilis*
7659	*Proteobacteria*	*Gammaproteobacte*	*Enterobacterales*	*Morganellaceae*	*Proteus*	*Proteus mirabilis*
7660	*Proteobacteria*	*Gammaproteobacte*	*Enterobacterales*	*Morganellaceae*	*Proteus*	*Proteus mirabilis*
7661	*Proteobacteria*	*Gammaproteobacte*	*Enterobacterales*	*Morganellaceae*	*Proteus*	*Proteus mirabilis*
7662	*Proteobacteria*	*Gammaproteobacte*	*Enterobacterales*	*Morganellaceae*	*Proteus*	*Proteus mirabilis*
7663	*Proteobacteria*	*Gammaproteobacte*	*Enterobacterales*	*Morganellaceae*	*Proteus*	*Proteus mirabilis*
7664	*Proteobacteria*	*Gammaproteobacte*	*Enterobacterales*	*Morganellaceae*	*Proteus*	*Proteus mirabilis*
7665	*Proteobacteria*	*Gammaproteobacte*	*Enterobacterales*	*Morganellaceae*	*Proteus*	*Proteus mirabilis*
7666	*Proteobacteria*	*Gammaproteobacte*	*Enterobacterales*	*Morganellaceae*	*Proteus*	*Proteus mirabilis*
7667	*Proteobacteria*	*Gammaproteobacte*	*Enterobacterales*	*Morganellaceae*	*Proteus*	*Proteus mirabilis*
7668	*Proteobacteria*	*Gammaproteobacte*	*Enterobacterales*	*Morganellaceae*	*Proteus*	*Proteus mirabilis*
7669	*Proteobacteria*	*Gammaproteobacte*	*Enterobacterales*	*Morganellaceae*	*Proteus*	*Proteus mirabilis*
7670	*Proteobacteria*	*Gammaproteobacte*	*Enterobacterales*	*Morganellaceae*	*Proteus*	*Proteus mirabilis*
7671	*Proteobacteria*	*Gammaproteobacte*	*Enterobacterales*	*Morganellaceae*	*Proteus*	*Proteus mirabilis*
7672	*Proteobacteria*	*Gammaproteobacte*	*Enterobacterales*	*Morganellaceae*	*Proteus*	*Proteus mirabilis*

Table S4. Isolated strains by culturomics

bacteriaId	*phylum*	*class*	*order*	*family*	*genus*	*species*
7673	*Proteobacteria*	*Gammaproteobacte*	*Enterobacterales*	*Morganellaceae*	*Proteus*	*Proteus mirabilis*
7674	*Proteobacteria*	*Gammaproteobacte*	*Enterobacterales*	*Morganellaceae*	*Proteus*	*Proteus mirabilis*
7675	*Proteobacteria*	*Gammaproteobacte*	*Enterobacterales*	*Morganellaceae*	*Proteus*	*Proteus mirabilis*
7676	*Proteobacteria*	*Gammaproteobacte*	*Enterobacterales*	*Morganellaceae*	*Proteus*	*Proteus mirabilis*
7677	*Proteobacteria*	*Gammaproteobacte*	*Enterobacterales*	*Morganellaceae*	*Proteus*	*Proteus mirabilis*
7678	*Proteobacteria*	*Gammaproteobacte*	*Enterobacterales*	*Morganellaceae*	*Proteus*	*Proteus mirabilis*
7679	*Proteobacteria*	*Gammaproteobacte*	*Enterobacterales*	*Morganellaceae*	*Proteus*	*Proteus mirabilis*
7680	*Proteobacteria*	*Gammaproteobacte*	*Enterobacterales*	*Morganellaceae*	*Proteus*	*Proteus mirabilis*
7681	*Proteobacteria*	*Gammaproteobacte*	*Enterobacterales*	*Morganellaceae*	*Proteus*	*Proteus mirabilis*
7682	*Proteobacteria*	*Gammaproteobacte*	*Enterobacterales*	*Morganellaceae*	*Proteus*	*Proteus mirabilis*
7683	*Proteobacteria*	*Gammaproteobacte*	*Enterobacterales*	*Morganellaceae*	*Proteus*	*Proteus mirabilis*
7684	*Proteobacteria*	*Gammaproteobacte*	*Enterobacterales*	*Morganellaceae*	*Proteus*	*Proteus mirabilis*
7685	*Proteobacteria*	*Gammaproteobacte*	*Enterobacterales*	*Morganellaceae*	*Proteus*	*Proteus mirabilis*
7686	*Proteobacteria*	*Gammaproteobacte*	*Enterobacterales*	*Morganellaceae*	*Proteus*	*Proteus mirabilis*
7687	*Proteobacteria*	*Gammaproteobacte*	*Enterobacterales*	*Morganellaceae*	*Proteus*	*Proteus mirabilis*
7688	*Proteobacteria*	*Gammaproteobacte*	*Enterobacterales*	*Morganellaceae*	*Proteus*	*Proteus mirabilis*
7689	*Proteobacteria*	*Gammaproteobacte*	*Enterobacterales*	*Morganellaceae*	*Proteus*	*Proteus mirabilis*
7690	*Proteobacteria*	*Gammaproteobacte*	*Enterobacterales*	*Morganellaceae*	*Proteus*	*Proteus mirabilis*
7691	*Proteobacteria*	*Gammaproteobacte*	*Enterobacterales*	*Morganellaceae*	*Proteus*	*Proteus mirabilis*
7692	*Proteobacteria*	*Gammaproteobacte*	*Enterobacterales*	*Morganellaceae*	*Proteus*	*Proteus mirabilis*
7693	*Proteobacteria*	*Gammaproteobacte*	*Enterobacterales*	*Morganellaceae*	*Proteus*	*Proteus mirabilis*
7694	*Proteobacteria*	*Gammaproteobacte*	*Enterobacterales*	*Morganellaceae*	*Proteus*	*Proteus mirabilis*
7695	*Proteobacteria*	*Gammaproteobacte*	*Enterobacterales*	*Morganellaceae*	*Proteus*	*Proteus mirabilis*
7696	*Proteobacteria*	*Gammaproteobacte*	*Enterobacterales*	*Morganellaceae*	*Proteus*	*Proteus mirabilis*
7697	*Proteobacteria*	*Gammaproteobacte*	*Enterobacterales*	*Morganellaceae*	*Proteus*	*Proteus mirabilis*
7698	*Proteobacteria*	*Gammaproteobacte*	*Enterobacterales*	*Morganellaceae*	*Proteus*	*Proteus mirabilis*
7699	*Proteobacteria*	*Gammaproteobacte*	*Enterobacterales*	*Morganellaceae*	*Proteus*	*Proteus mirabilis*
7700	*Proteobacteria*	*Gammaproteobacte*	*Enterobacterales*	*Morganellaceae*	*Proteus*	*Proteus mirabilis*

Table S4. Isolated strains by culturomics

bacteriaId	*phylum*	*class*	*order*	*family*	*genus*	*species*
7701	*Proteobacteria*	*Gammaproteobacte*	*Enterobacterales*	*Morganellaceae*	*Proteus*	*Proteus mirabilis*
7702	*Proteobacteria*	*Gammaproteobacte*	*Enterobacterales*	*Morganellaceae*	*Proteus*	*Proteus mirabilis*
7703	*Proteobacteria*	*Gammaproteobacte*	*Enterobacterales*	*Morganellaceae*	*Proteus*	*Proteus mirabilis*
7704	*Proteobacteria*	*Gammaproteobacte*	*Enterobacterales*	*Morganellaceae*	*Proteus*	*Proteus mirabilis*
7705	*Proteobacteria*	*Gammaproteobacte*	*Enterobacterales*	*Morganellaceae*	*Proteus*	*Proteus mirabilis*
7706	*Proteobacteria*	*Gammaproteobacte*	*Enterobacterales*	*Morganellaceae*	*Proteus*	*Proteus mirabilis*
7707	*Proteobacteria*	*Gammaproteobacte*	*Enterobacterales*	*Morganellaceae*	*Proteus*	*Proteus mirabilis*
7708	*Proteobacteria*	*Gammaproteobacte*	*Enterobacterales*	*Morganellaceae*	*Proteus*	*Proteus mirabilis*
7709	*Proteobacteria*	*Gammaproteobacte*	*Enterobacterales*	*Morganellaceae*	*Proteus*	*Proteus mirabilis*
7710	*Proteobacteria*	*Gammaproteobacte*	*Enterobacterales*	*Morganellaceae*	*Proteus*	*Proteus mirabilis*
7711	*Proteobacteria*	*Gammaproteobacte*	*Enterobacterales*	*Morganellaceae*	*Proteus*	*Proteus mirabilis*
7712	*Proteobacteria*	*Gammaproteobacte*	*Enterobacterales*	*Morganellaceae*	*Proteus*	*Proteus mirabilis*
7713	*Proteobacteria*	*Gammaproteobacte*	*Enterobacterales*	*Morganellaceae*	*Proteus*	*Proteus mirabilis*
7714	*Proteobacteria*	*Gammaproteobacte*	*Enterobacterales*	*Morganellaceae*	*Proteus*	*Proteus mirabilis*
7715	*Proteobacteria*	*Gammaproteobacte*	*Enterobacterales*	*Morganellaceae*	*Proteus*	*Proteus mirabilis*
7716	*Proteobacteria*	*Gammaproteobacte*	*Enterobacterales*	*Morganellaceae*	*Proteus*	*Proteus mirabilis*
7717	*Proteobacteria*	*Gammaproteobacte*	*Enterobacterales*	*Morganellaceae*	*Proteus*	*Proteus mirabilis*
7718	*Proteobacteria*	*Gammaproteobacte*	*Enterobacterales*	*Morganellaceae*	*Proteus*	*Proteus mirabilis*
7719	*Proteobacteria*	*Gammaproteobacte*	*Enterobacterales*	*Morganellaceae*	*Proteus*	*Proteus mirabilis*
7720	*Proteobacteria*	*Gammaproteobacte*	*Enterobacterales*	*Morganellaceae*	*Proteus*	*Proteus mirabilis*
7721	*Proteobacteria*	*Gammaproteobacte*	*Enterobacterales*	*Morganellaceae*	*Proteus*	*Proteus mirabilis*
7722	*Proteobacteria*	*Gammaproteobacte*	*Enterobacterales*	*Morganellaceae*	*Proteus*	*Proteus mirabilis*
7723	*Proteobacteria*	*Gammaproteobacte*	*Enterobacterales*	*Morganellaceae*	*Proteus*	*Proteus mirabilis*
7724	*Proteobacteria*	*Gammaproteobacte*	*Enterobacterales*	*Morganellaceae*	*Proteus*	*Proteus mirabilis*
7725	*Proteobacteria*	*Gammaproteobacte*	*Enterobacterales*	*Morganellaceae*	*Proteus*	*Proteus mirabilis*
7726	*Proteobacteria*	*Gammaproteobacte*	*Enterobacterales*	*Morganellaceae*	*Proteus*	*Proteus mirabilis*
7727	*Proteobacteria*	*Gammaproteobacte*	*Enterobacterales*	*Morganellaceae*	*Proteus*	*Proteus mirabilis*
7728	*Proteobacteria*	*Gammaproteobacte*	*Enterobacterales*	*Morganellaceae*	*Proteus*	*Proteus mirabilis*

Table S4. Isolated strains by culturomics

bacteriaId	*phylum*	*class*	*order*	*family*	*genus*	*species*
7729	*Proteobacteria*	*Gammaproteobacte*	*Enterobacterales*	*Morganellaceae*	*Proteus*	*Proteus mirabilis*
7730	*Proteobacteria*	*Gammaproteobacte*	*Enterobacterales*	*Morganellaceae*	*Proteus*	*Proteus mirabilis*
7731	*Proteobacteria*	*Gammaproteobacte*	*Enterobacterales*	*Morganellaceae*	*Proteus*	*Proteus mirabilis*
7732	*Proteobacteria*	*Gammaproteobacte*	*Enterobacterales*	*Morganellaceae*	*Proteus*	*Proteus mirabilis*
7733	*Proteobacteria*	*Gammaproteobacte*	*Enterobacterales*	*Morganellaceae*	*Proteus*	*Proteus mirabilis*
7734	*Proteobacteria*	*Gammaproteobacte*	*Enterobacterales*	*Morganellaceae*	*Proteus*	*Proteus mirabilis*
7735	*Proteobacteria*	*Gammaproteobacte*	*Enterobacterales*	*Morganellaceae*	*Proteus*	*Proteus mirabilis*
7736	*Proteobacteria*	*Gammaproteobacte*	*Enterobacterales*	*Morganellaceae*	*Proteus*	*Proteus mirabilis*
7737	*Proteobacteria*	*Gammaproteobacte*	*Enterobacterales*	*Morganellaceae*	*Proteus*	*Proteus mirabilis*
7738	*Proteobacteria*	*Gammaproteobacte*	*Enterobacterales*	*Morganellaceae*	*Proteus*	*Proteus mirabilis*
7739	*Proteobacteria*	*Gammaproteobacte*	*Enterobacterales*	*Morganellaceae*	*Proteus*	*Proteus mirabilis*
7740	*Proteobacteria*	*Gammaproteobacte*	*Enterobacterales*	*Morganellaceae*	*Proteus*	*Proteus mirabilis*
7741	*Proteobacteria*	*Gammaproteobacte*	*Enterobacterales*	*Morganellaceae*	*Proteus*	*Proteus mirabilis*
7742	*Proteobacteria*	*Gammaproteobacte*	*Enterobacterales*	*Morganellaceae*	*Proteus*	*Proteus mirabilis*
7743	*Proteobacteria*	*Gammaproteobacte*	*Enterobacterales*	*Morganellaceae*	*Proteus*	*Proteus mirabilis*
7744	*Proteobacteria*	*Gammaproteobacte*	*Enterobacterales*	*Morganellaceae*	*Proteus*	*Proteus mirabilis*
7745	*Proteobacteria*	*Gammaproteobacte*	*Enterobacterales*	*Morganellaceae*	*Proteus*	*Proteus mirabilis*
7746	*Proteobacteria*	*Gammaproteobacte*	*Enterobacterales*	*Morganellaceae*	*Proteus*	*Proteus mirabilis*
7747	*Proteobacteria*	*Gammaproteobacte*	*Enterobacterales*	*Morganellaceae*	*Proteus*	*Proteus mirabilis*
7748	*Proteobacteria*	*Gammaproteobacte*	*Enterobacterales*	*Morganellaceae*	*Proteus*	*Proteus mirabilis*
7749	*Proteobacteria*	*Gammaproteobacte*	*Enterobacterales*	*Morganellaceae*	*Proteus*	*Proteus mirabilis*
7750	*Proteobacteria*	*Gammaproteobacte*	*Enterobacterales*	*Morganellaceae*	*Proteus*	*Proteus mirabilis*
7751	*Proteobacteria*	*Gammaproteobacte*	*Enterobacterales*	*Morganellaceae*	*Proteus*	*Proteus mirabilis*
7752	*Proteobacteria*	*Gammaproteobacte*	*Enterobacterales*	*Morganellaceae*	*Proteus*	*Proteus mirabilis*
7753	*Proteobacteria*	*Gammaproteobacte*	*Enterobacterales*	*Morganellaceae*	*Proteus*	*Proteus mirabilis*
7754	*Proteobacteria*	*Gammaproteobacte*	*Enterobacterales*	*Morganellaceae*	*Proteus*	*Proteus mirabilis*
7755	*Proteobacteria*	*Gammaproteobacte*	*Enterobacterales*	*Morganellaceae*	*Proteus*	*Proteus mirabilis*
7756	*Proteobacteria*	*Gammaproteobacte*	*Enterobacterales*	*Morganellaceae*	*Proteus*	*Proteus mirabilis*

Table S4. Isolated strains by culturomics

bacteriald	*phylum*	*class*	*order*	*family*	*genus*	*species*
7757	*Proteobacteria*	*Gammaproteobacte*	*Enterobacterales*	*Morganellaceae*	*Proteus*	*Proteus mirabilis*
7758	*Proteobacteria*	*Gammaproteobacte*	*Enterobacterales*	*Morganellaceae*	*Proteus*	*Proteus mirabilis*
7759	*Proteobacteria*	*Gammaproteobacte*	*Enterobacterales*	*Morganellaceae*	*Proteus*	*Proteus mirabilis*
7760	*Proteobacteria*	*Gammaproteobacte*	*Enterobacterales*	*Morganellaceae*	*Proteus*	*Proteus mirabilis*
7761	*Proteobacteria*	*Gammaproteobacte*	*Enterobacterales*	*Morganellaceae*	*Proteus*	*Proteus mirabilis*
7762	*Proteobacteria*	*Gammaproteobacte*	*Enterobacterales*	*Morganellaceae*	*Proteus*	*Proteus mirabilis*
7763	*Proteobacteria*	*Gammaproteobacte*	*Enterobacterales*	*Morganellaceae*	*Proteus*	*Proteus mirabilis*
7764	*Proteobacteria*	*Gammaproteobacte*	*Enterobacterales*	*Morganellaceae*	*Proteus*	*Proteus mirabilis*
7765	*Proteobacteria*	*Gammaproteobacte*	*Enterobacterales*	*Morganellaceae*	*Proteus*	*Proteus mirabilis*
7766	*Proteobacteria*	*Gammaproteobacte*	*Enterobacterales*	*Morganellaceae*	*Proteus*	*Proteus mirabilis*
7767	*Proteobacteria*	*Gammaproteobacte*	*Enterobacterales*	*Morganellaceae*	*Proteus*	*Proteus mirabilis*
7768	*Proteobacteria*	*Gammaproteobacte*	*Enterobacterales*	*Morganellaceae*	*Proteus*	*Proteus mirabilis*
7769	*Proteobacteria*	*Gammaproteobacte*	*Enterobacterales*	*Morganellaceae*	*Proteus*	*Proteus penneri*
7770	*Proteobacteria*	*Gammaproteobacte*	*Enterobacterales*	*Morganellaceae*	*Proteus*	*Proteus penneri*
7771	*Proteobacteria*	*Gammaproteobacte*	*Enterobacterales*	*Morganellaceae*	*Proteus*	*Proteus penneri*
7772	*Proteobacteria*	*Gammaproteobacte*	*Enterobacterales*	*Morganellaceae*	*Proteus*	*Proteus vulgaris*
7773	*Proteobacteria*	*Gammaproteobacte*	*Enterobacterales*	*Morganellaceae*	*Proteus*	*Proteus vulgaris*
7774	*Proteobacteria*	*Gammaproteobacte*	*Enterobacterales*	*Morganellaceae*	*Proteus*	*Proteus vulgaris*
7775	*Proteobacteria*	*Gammaproteobacte*	*Enterobacterales*	*Morganellaceae*	*Providencia*	*Providencia*
7776	*Proteobacteria*	*Gammaproteobacte*	*Enterobacterales*	*Morganellaceae*	*Providencia*	*Providencia rettgeri*
7777	*Proteobacteria*	*Gammaproteobacte*	*Enterobacterales*	*Morganellaceae*	*Providencia*	*Providencia rettgeri*
7778	*Proteobacteria*	*Gammaproteobacte*	*Enterobacterales*	*Morganellaceae*	*Providencia*	*Providencia rettgeri*
7779	*Proteobacteria*	*Gammaproteobacte*	*Enterobacterales*	*Morganellaceae*	*Providencia*	*Providencia rettgeri*
7780	*Proteobacteria*	*Gammaproteobacte*	*Enterobacterales*	*Morganellaceae*	*Providencia*	*Providencia rettgeri*
7781	*Proteobacteria*	*Gammaproteobacte*	*Enterobacterales*	*Morganellaceae*	*Providencia*	*Providencia rettgeri*
7782	*Proteobacteria*	*Gammaproteobacte*	*Enterobacterales*	*Morganellaceae*	*Providencia*	*Providencia rettgeri*
7783	*Proteobacteria*	*Gammaproteobacte*	*Enterobacterales*	*Morganellaceae*	*Providencia*	*Providencia rettgeri*
7784	*Proteobacteria*	*Gammaproteobacte*	*Enterobacterales*	*Morganellaceae*	*Providencia*	*Providencia rettgeri*

Table S4. Isolated strains by culturomics

bacteriaId	*phylum*	*class*	*order*	*family*	*genus*	*species*
7785	*Proteobacteria*	*Gammaproteobacte*	*Enterobacterales*	*Morganellaceae*	*Providencia*	*Providencia rettgeri*
7786	*Proteobacteria*	*Gammaproteobacte*	*Enterobacterales*	*Morganellaceae*	*Providencia*	*Providencia rettgeri*
7787	*Proteobacteria*	*Gammaproteobacte*	*Enterobacterales*	*Morganellaceae*	*Providencia*	*Providencia stuartii*
7788	*Proteobacteria*	*Gammaproteobacte*	*Enterobacterales*	*Morganellaceae*	*Providencia*	*Providencia stuartii*
7789	*Proteobacteria*	*Gammaproteobacte*	*Enterobacterales*	*Morganellaceae*	*Providencia*	*Providencia stuartii*
7790	*Proteobacteria*	*Gammaproteobacte*	*Enterobacterales*	*Morganellaceae*	*Providencia*	*Providencia stuartii*
7791	*Proteobacteria*	*Gammaproteobacte*	*Pseudomonadales*	*Moraxellaceae*	*Acinetobacter*	*Acinetobacter*
7792	*Proteobacteria*	*Gammaproteobacte*	*Pseudomonadales*	*Moraxellaceae*	*Acinetobacter*	*Acinetobacter*
7793	*Proteobacteria*	*Gammaproteobacte*	*Pseudomonadales*	*Moraxellaceae*	*Moraxella*	*Moraxella sg*
7794	*Proteobacteria*	*Gammaproteobacte*	*Pseudomonadales*	*Pseudomonadaceae*	*Pseudomonas*	*Pseudomonas*
7795	*Proteobacteria*	*Gammaproteobacte*	*Pseudomonadales*	*Pseudomonadaceae*	*Pseudomonas*	*Pseudomonas*
7796	*Proteobacteria*	*Gammaproteobacte*	*Pseudomonadales*	*Pseudomonadaceae*	*Pseudomonas*	*Pseudomonas*
7797	*Proteobacteria*	*Gammaproteobacte*	*Pseudomonadales*	*Pseudomonadaceae*	*Pseudomonas*	*Pseudomonas*
7798	*Proteobacteria*	*Gammaproteobacte*	*Pseudomonadales*	*Pseudomonadaceae*	*Pseudomonas*	*Pseudomonas*
7799	*Proteobacteria*	*Gammaproteobacte*	*Pseudomonadales*	*Pseudomonadaceae*	*Pseudomonas*	*Pseudomonas*
7800	*Proteobacteria*	*Gammaproteobacte*	*Pseudomonadales*	*Pseudomonadaceae*	*Pseudomonas*	*Pseudomonas*
7801	*Proteobacteria*	*Gammaproteobacte*	*Pseudomonadales*	*Pseudomonadaceae*	*Pseudomonas*	*Pseudomonas*
7802	*Proteobacteria*	*Gammaproteobacte*	*Pseudomonadales*	*Pseudomonadaceae*	*Pseudomonas*	*Pseudomonas*
7803	*Proteobacteria*	*Gammaproteobacte*	*Pseudomonadales*	*Pseudomonadaceae*	*Pseudomonas*	*Pseudomonas*
7804	*Proteobacteria*	*Gammaproteobacte*	*Pseudomonadales*	*Pseudomonadaceae*	*Pseudomonas*	*Pseudomonas*
7805	*Proteobacteria*	*Gammaproteobacte*	*Pseudomonadales*	*Pseudomonadaceae*	*Pseudomonas*	*Pseudomonas*
7806	*Proteobacteria*	*Gammaproteobacte*	*Pseudomonadales*	*Pseudomonadaceae*	*Pseudomonas*	*Pseudomonas*
7807	*Proteobacteria*	*Gammaproteobacte*	*Pseudomonadales*	*Pseudomonadaceae*	*Pseudomonas*	*Pseudomonas*
7808	*Proteobacteria*	*Gammaproteobacte*	*Pseudomonadales*	*Pseudomonadaceae*	*Pseudomonas*	*Pseudomonas*
7809	*Proteobacteria*	*Gammaproteobacte*	*Pseudomonadales*	*Pseudomonadaceae*	*Pseudomonas*	*Pseudomonas*
7810	*Proteobacteria*	*Gammaproteobacte*	*Pseudomonadales*	*Pseudomonadaceae*	*Pseudomonas*	*Pseudomonas*
7811	*Proteobacteria*	*Gammaproteobacte*	*Pseudomonadales*	*Pseudomonadaceae*	*Pseudomonas*	*Pseudomonas*
7812	*Proteobacteria*	*Gammaproteobacte*	*Pseudomonadales*	*Pseudomonadaceae*	*Pseudomonas*	*Pseudomonas*

Table S4. Isolated strains by culturomics

bacteriaId	*phylum*	*class*	*order*	*family*	*genus*	*species*
7813	*Proteobacteria*	*Gammaproteobacte*	*Pseudomonadales*	*Pseudomonadaceae*	*Pseudomonas*	*Pseudomonas*
7814	*Proteobacteria*	*Gammaproteobacte*	*Pseudomonadales*	*Pseudomonadaceae*	*Pseudomonas*	*Pseudomonas*
7815	*Proteobacteria*	*Gammaproteobacte*	*Pseudomonadales*	*Pseudomonadaceae*	*Pseudomonas*	*Pseudomonas*
7816	*Proteobacteria*	*Gammaproteobacte*	*Pseudomonadales*	*Pseudomonadaceae*	*Pseudomonas*	*Pseudomonas*
7817	*Proteobacteria*	*Gammaproteobacte*	*Pseudomonadales*	*Pseudomonadaceae*	*Pseudomonas*	*Pseudomonas*
7818	*Proteobacteria*	*Gammaproteobacte*	*Pseudomonadales*	*Pseudomonadaceae*	*Pseudomonas*	*Pseudomonas*
7819	*Proteobacteria*	*Gammaproteobacte*	*Pseudomonadales*	*Pseudomonadaceae*	*Pseudomonas*	*Pseudomonas*
7820	*Proteobacteria*	*Gammaproteobacte*	*Pseudomonadales*	*Pseudomonadaceae*	*Pseudomonas*	*Pseudomonas*
7821	*Proteobacteria*	*Gammaproteobacte*	*Pseudomonadales*	*Pseudomonadaceae*	*Pseudomonas*	*Pseudomonas*
7822	*Proteobacteria*	*Gammaproteobacte*	*Pseudomonadales*	*Pseudomonadaceae*	*Pseudomonas*	*Pseudomonas*
7823	*Proteobacteria*	*Gammaproteobacte*	*Pseudomonadales*	*Pseudomonadaceae*	*Pseudomonas*	*Pseudomonas*
7824	*Proteobacteria*	*Gammaproteobacte*	*Pseudomonadales*	*Pseudomonadaceae*	*Pseudomonas*	*Pseudomonas*
7825	*Proteobacteria*	*Gammaproteobacte*	*Pseudomonadales*	*Pseudomonadaceae*	*Pseudomonas*	*Pseudomonas*
7826	*Proteobacteria*	*Gammaproteobacte*	*Pseudomonadales*	*Pseudomonadaceae*	*Pseudomonas*	*Pseudomonas*
7827	*Proteobacteria*	*Gammaproteobacte*	*Pseudomonadales*	*Pseudomonadaceae*	*Pseudomonas*	*Pseudomonas*
7828	*Proteobacteria*	*Gammaproteobacte*	*Pseudomonadales*	*Pseudomonadaceae*	*Pseudomonas*	*Pseudomonas*
7829	*Proteobacteria*	*Gammaproteobacte*	*Pseudomonadales*	*Pseudomonadaceae*	*Pseudomonas*	*Pseudomonas*
7830	*Proteobacteria*	*Gammaproteobacte*	*Pseudomonadales*	*Pseudomonadaceae*	*Pseudomonas*	*Pseudomonas*
7831	*Proteobacteria*	*Gammaproteobacte*	*Pseudomonadales*	*Pseudomonadaceae*	*Pseudomonas*	*Pseudomonas*
7832	*Proteobacteria*	*Gammaproteobacte*	*Pseudomonadales*	*Pseudomonadaceae*	*Pseudomonas*	*Pseudomonas*
7833	*Proteobacteria*	*Gammaproteobacte*	*Pseudomonadales*	*Pseudomonadaceae*	*Pseudomonas*	*Pseudomonas*
7834	*Proteobacteria*	*Gammaproteobacte*	*Pseudomonadales*	*Pseudomonadaceae*	*Pseudomonas*	*Pseudomonas*
7835	*Proteobacteria*	*Gammaproteobacte*	*Pseudomonadales*	*Pseudomonadaceae*	*Pseudomonas*	*Pseudomonas*
7836	*Proteobacteria*	*Gammaproteobacte*	*Pseudomonadales*	*Pseudomonadaceae*	*Pseudomonas*	*Pseudomonas*
7837	*Proteobacteria*	*Gammaproteobacte*	*Pseudomonadales*	*Pseudomonadaceae*	*Pseudomonas*	*Pseudomonas*
7838	*Proteobacteria*	*Gammaproteobacte*	*Pseudomonadales*	*Pseudomonadaceae*	*Pseudomonas*	*Pseudomonas*
7839	*Proteobacteria*	*Gammaproteobacte*	*Pseudomonadales*	*Pseudomonadaceae*	*Pseudomonas*	*Pseudomonas*
7840	*Proteobacteria*	*Gammaproteobacte*	*Pseudomonadales*	*Pseudomonadaceae*	*Pseudomonas*	*Pseudomonas*

Table S4. Isolated strains by culturomics

bacteriaId	*phylum*	*class*	*order*	*family*	*genus*	*species*
7841	*Proteobacteria*	*Gammaproteobacte*	*Pseudomonadales*	*Pseudomonadaceae*	*Pseudomonas*	*Pseudomonas*
7842	*Proteobacteria*	*Gammaproteobacte*	*Pseudomonadales*	*Pseudomonadaceae*	*Pseudomonas*	*Pseudomonas*
7843	*Proteobacteria*	*Gammaproteobacte*	*Pseudomonadales*	*Pseudomonadaceae*	*Pseudomonas*	*Pseudomonas*
7844	*Proteobacteria*	*Gammaproteobacte*	*Pseudomonadales*	*Pseudomonadaceae*	*Pseudomonas*	*Pseudomonas*
7845	*Proteobacteria*	*Gammaproteobacte*	*Pseudomonadales*	*Pseudomonadaceae*	*Pseudomonas*	*Pseudomonas*
7846	*Proteobacteria*	*Gammaproteobacte*	*Pseudomonadales*	*Pseudomonadaceae*	*Pseudomonas*	*Pseudomonas*
7847	*Proteobacteria*	*Gammaproteobacte*	*Pseudomonadales*	*Pseudomonadaceae*	*Pseudomonas*	*Pseudomonas*
7848	*Proteobacteria*	*Gammaproteobacte*	*Pseudomonadales*	*Pseudomonadaceae*	*Pseudomonas*	*Pseudomonas*
7849	*Proteobacteria*	*Gammaproteobacte*	*Pseudomonadales*	*Pseudomonadaceae*	*Pseudomonas*	*Pseudomonas*
7850	*Proteobacteria*	*Gammaproteobacte*	*Pseudomonadales*	*Pseudomonadaceae*	*Pseudomonas*	*Pseudomonas*
7851	*Proteobacteria*	*Gammaproteobacte*	*Pseudomonadales*	*Pseudomonadaceae*	*Pseudomonas*	*Pseudomonas*
7852	*Proteobacteria*	*Gammaproteobacte*	*Pseudomonadales*	*Pseudomonadaceae*	*Pseudomonas*	*Pseudomonas*
7853	*Proteobacteria*	*Gammaproteobacte*	*Pseudomonadales*	*Pseudomonadaceae*	*Pseudomonas*	*Pseudomonas*
7854	*Proteobacteria*	*Gammaproteobacte*	*Pseudomonadales*	*Pseudomonadaceae*	*Pseudomonas*	*Pseudomonas*
7855	*Proteobacteria*	*Gammaproteobacte*	*Pseudomonadales*	*Pseudomonadaceae*	*Pseudomonas*	*Pseudomonas*
7856	*Proteobacteria*	*Gammaproteobacte*	*Pseudomonadales*	*Pseudomonadaceae*	*Pseudomonas*	*Pseudomonas*
7857	*Proteobacteria*	*Gammaproteobacte*	*Pseudomonadales*	*Pseudomonadaceae*	*Pseudomonas*	*Pseudomonas*
7858	*Proteobacteria*	*Gammaproteobacte*	*Pseudomonadales*	*Pseudomonadaceae*	*Pseudomonas*	*Pseudomonas*
7859	*Proteobacteria*	*Gammaproteobacte*	*Pseudomonadales*	*Pseudomonadaceae*	*Pseudomonas*	*Pseudomonas*
7860	*Proteobacteria*	*Gammaproteobacte*	*Pseudomonadales*	*Pseudomonadaceae*	*Pseudomonas*	*Pseudomonas*
7861	*Proteobacteria*	*Gammaproteobacte*	*Pseudomonadales*	*Pseudomonadaceae*	*Pseudomonas*	*Pseudomonas*
7862	*Proteobacteria*	*Gammaproteobacte*	*Pseudomonadales*	*Pseudomonadaceae*	*Pseudomonas*	*Pseudomonas*
7863	*Proteobacteria*	*Gammaproteobacte*	*Pseudomonadales*	*Pseudomonadaceae*	*Pseudomonas*	*Pseudomonas*
7864	*Proteobacteria*	*Gammaproteobacte*	*Pseudomonadales*	*Pseudomonadaceae*	*Pseudomonas*	*Pseudomonas*
7865	*Proteobacteria*	*Gammaproteobacte*	*Pseudomonadales*	*Pseudomonadaceae*	*Pseudomonas*	*Pseudomonas*
7866	*Proteobacteria*	*Gammaproteobacte*	*Pseudomonadales*	*Pseudomonadaceae*	*Pseudomonas*	*Pseudomonas*
7867	*Proteobacteria*	*Gammaproteobacte*	*Pseudomonadales*	*Pseudomonadaceae*	*Pseudomonas*	*Pseudomonas*
7868	*Proteobacteria*	*Gammaproteobacte*	*Pseudomonadales*	*Pseudomonadaceae*	*Pseudomonas*	*Pseudomonas*

Table S4. Isolated strains by culturomics

bacteriaId	*phylum*	*class*	*order*	*family*	*genus*	*species*
7869	*Proteobacteria*	*Gammaproteobacte*	*Pseudomonadales*	*Pseudomonadaceae*	*Pseudomonas*	*Pseudomonas*
7870	*Proteobacteria*	*Gammaproteobacte*	*Pseudomonadales*	*Pseudomonadaceae*	*Pseudomonas*	*Pseudomonas*
7871	*Proteobacteria*	*Gammaproteobacte*	*Pseudomonadales*	*Pseudomonadaceae*	*Pseudomonas*	*Pseudomonas*
7872	*Proteobacteria*	*Gammaproteobacte*	*Pseudomonadales*	*Pseudomonadaceae*	*Pseudomonas*	*Pseudomonas*
7873	*Proteobacteria*	*Gammaproteobacte*	*Pseudomonadales*	*Pseudomonadaceae*	*Pseudomonas*	*Pseudomonas*
7874	*Proteobacteria*	*Gammaproteobacte*	*Pseudomonadales*	*Pseudomonadaceae*	*Pseudomonas*	*Pseudomonas*
7875	*Proteobacteria*	*Gammaproteobacte*	*Pseudomonadales*	*Pseudomonadaceae*	*Pseudomonas*	*Pseudomonas*
7876	*Proteobacteria*	*Gammaproteobacte*	*Pseudomonadales*	*Pseudomonadaceae*	*Pseudomonas*	*Pseudomonas*
7877	*Proteobacteria*	*Gammaproteobacte*	*Pseudomonadales*	*Pseudomonadaceae*	*Pseudomonas*	*Pseudomonas*
7878	*Proteobacteria*	*Gammaproteobacte*	*Pseudomonadales*	*Pseudomonadaceae*	*Pseudomonas*	*Pseudomonas*
7879	*Proteobacteria*	*Gammaproteobacte*	*Pseudomonadales*	*Pseudomonadaceae*	*Pseudomonas*	*Pseudomonas*
7880	*Proteobacteria*	*Gammaproteobacte*	*Pseudomonadales*	*Pseudomonadaceae*	*Pseudomonas*	*Pseudomonas*
7881	*Proteobacteria*	*Gammaproteobacte*	*Pseudomonadales*	*Pseudomonadaceae*	*Pseudomonas*	*Pseudomonas*
7882	*Proteobacteria*	*Gammaproteobacte*	*Pseudomonadales*	*Pseudomonadaceae*	*Pseudomonas*	*Pseudomonas*
7883	*Proteobacteria*	*Gammaproteobacte*	*Pseudomonadales*	*Pseudomonadaceae*	*Pseudomonas*	*Pseudomonas*
7884	*Proteobacteria*	*Gammaproteobacte*	*Pseudomonadales*	*Pseudomonadaceae*	*Pseudomonas*	*Pseudomonas*
7885	*Proteobacteria*	*Gammaproteobacte*	*Pseudomonadales*	*Pseudomonadaceae*	*Pseudomonas*	*Pseudomonas*
7886	*Proteobacteria*	*Gammaproteobacte*	*Pseudomonadales*	*Pseudomonadaceae*	*Pseudomonas*	*Pseudomonas*
7887	*Proteobacteria*	*Gammaproteobacte*	*Pseudomonadales*	*Pseudomonadaceae*	*Pseudomonas*	*Pseudomonas*
7888	*Proteobacteria*	*Gammaproteobacte*	*Pseudomonadales*	*Pseudomonadaceae*	*Pseudomonas*	*Pseudomonas*
7889	*Proteobacteria*	*Gammaproteobacte*	*Pseudomonadales*	*Pseudomonadaceae*	*Pseudomonas*	*Pseudomonas*
7890	*Proteobacteria*	*Gammaproteobacte*	*Pseudomonadales*	*Pseudomonadaceae*	*Pseudomonas*	*Pseudomonas*
7891	*Proteobacteria*	*Gammaproteobacte*	*Pseudomonadales*	*Pseudomonadaceae*	*Pseudomonas*	*Pseudomonas*
7892	*Proteobacteria*	*Gammaproteobacte*	*Pseudomonadales*	*Pseudomonadaceae*	*Pseudomonas*	*Pseudomonas*
7893	*Proteobacteria*	*Gammaproteobacte*	*Pseudomonadales*	*Pseudomonadaceae*	*Pseudomonas*	*Pseudomonas*
7894	*Proteobacteria*	*Gammaproteobacte*	*Pseudomonadales*	*Pseudomonadaceae*	*Pseudomonas*	*Pseudomonas*
7895	*Proteobacteria*	*Gammaproteobacte*	*Pseudomonadales*	*Pseudomonadaceae*	*Pseudomonas*	*Pseudomonas*
7896	*Proteobacteria*	*Gammaproteobacte*	*Pseudomonadales*	*Pseudomonadaceae*	*Pseudomonas*	*Pseudomonas*

Table S4. Isolated strains by culturomics

bacteriaId	*phylum*	*class*	*order*	*family*	*genus*	*species*
7897	*Proteobacteria*	*Gammaproteobacte*	*Pseudomonadales*	*Pseudomonadaceae*	*Pseudomonas*	*Pseudomonas*
7898	*Proteobacteria*	*Gammaproteobacte*	*Pseudomonadales*	*Pseudomonadaceae*	*Pseudomonas*	*Pseudomonas*
7899	*Proteobacteria*	*Gammaproteobacte*	*Pseudomonadales*	*Pseudomonadaceae*	*Pseudomonas*	*Pseudomonas*
7900	*Proteobacteria*	*Gammaproteobacte*	*Pseudomonadales*	*Pseudomonadaceae*	*Pseudomonas*	*Pseudomonas*
7901	*Proteobacteria*	*Gammaproteobacte*	*Pseudomonadales*	*Pseudomonadaceae*	*Pseudomonas*	*Pseudomonas*
7902	*Proteobacteria*	*Gammaproteobacte*	*Pseudomonadales*	*Pseudomonadaceae*	*Pseudomonas*	*Pseudomonas*
7903	*Proteobacteria*	*Gammaproteobacte*	*Pseudomonadales*	*Pseudomonadaceae*	*Pseudomonas*	*Pseudomonas*
7904	*Proteobacteria*	*Gammaproteobacte*	*Pseudomonadales*	*Pseudomonadaceae*	*Pseudomonas*	*Pseudomonas*
7905	*Proteobacteria*	*Gammaproteobacte*	*Pseudomonadales*	*Pseudomonadaceae*	*Pseudomonas*	*Pseudomonas*
7906	*Proteobacteria*	*Gammaproteobacte*	*Pseudomonadales*	*Pseudomonadaceae*	*Pseudomonas*	*Pseudomonas*
7907	*Proteobacteria*	*Gammaproteobacte*	*Pseudomonadales*	*Pseudomonadaceae*	*Pseudomonas*	*Pseudomonas*
7908	*Proteobacteria*	*Gammaproteobacte*	*Pseudomonadales*	*Pseudomonadaceae*	*Pseudomonas*	*Pseudomonas*
7909	*Proteobacteria*	*Gammaproteobacte*	*Pseudomonadales*	*Pseudomonadaceae*	*Pseudomonas*	*Pseudomonas*
7910	*Proteobacteria*	*Gammaproteobacte*	*Pseudomonadales*	*Pseudomonadaceae*	*Pseudomonas*	*Pseudomonas*
7911	*Proteobacteria*	*Gammaproteobacte*	*Pseudomonadales*	*Pseudomonadaceae*	*Pseudomonas*	*Pseudomonas*
7912	*Proteobacteria*	*Gammaproteobacte*	*Pseudomonadales*	*Pseudomonadaceae*	*Pseudomonas*	*Pseudomonas*
7913	*Proteobacteria*	*Gammaproteobacte*	*Pseudomonadales*	*Pseudomonadaceae*	*Pseudomonas*	*Pseudomonas*
7914	*Proteobacteria*	*Gammaproteobacte*	*Pseudomonadales*	*Pseudomonadaceae*	*Pseudomonas*	*Pseudomonas*
7915	*Proteobacteria*	*Gammaproteobacte*	*Pseudomonadales*	*Pseudomonadaceae*	*Pseudomonas*	*Pseudomonas*
7916	*Proteobacteria*	*Gammaproteobacte*	*Pseudomonadales*	*Pseudomonadaceae*	*Pseudomonas*	*Pseudomonas*
7917	*Proteobacteria*	*Gammaproteobacte*	*Pseudomonadales*	*Pseudomonadaceae*	*Pseudomonas*	*Pseudomonas*
7918	*Proteobacteria*	*Gammaproteobacte*	*Pseudomonadales*	*Pseudomonadaceae*	*Pseudomonas*	*Pseudomonas*
7919	*Proteobacteria*	*Gammaproteobacte*	*Pseudomonadales*	*Pseudomonadaceae*	*Pseudomonas*	*Pseudomonas*
7920	*Proteobacteria*	*Gammaproteobacte*	*Pseudomonadales*	*Pseudomonadaceae*	*Pseudomonas*	*Pseudomonas*
7921	*Proteobacteria*	*Gammaproteobacte*	*Pseudomonadales*	*Pseudomonadaceae*	*Pseudomonas*	*Pseudomonas*
7922	*Proteobacteria*	*Gammaproteobacte*	*Pseudomonadales*	*Pseudomonadaceae*	*Pseudomonas*	*Pseudomonas*
7923	*Proteobacteria*	*Gammaproteobacte*	*Pseudomonadales*	*Pseudomonadaceae*	*Pseudomonas*	*Pseudomonas*
7924	*Proteobacteria*	*Gammaproteobacte*	*Pseudomonadales*	*Pseudomonadaceae*	*Pseudomonas*	*Pseudomonas*

Table S4. Isolated strains by culturomics

bacteriaId	*phylum*	*class*	*order*	*family*	*genus*	*species*
7925	*Proteobacteria*	*Gammaproteobacte*	*Pseudomonadales*	*Pseudomonadaceae*	*Pseudomonas*	*Pseudomonas*
7926	*Proteobacteria*	*Gammaproteobacte*	*Pseudomonadales*	*Pseudomonadaceae*	*Pseudomonas*	*Pseudomonas*
7927	*Proteobacteria*	*Gammaproteobacte*	*Pseudomonadales*	*Pseudomonadaceae*	*Pseudomonas*	*Pseudomonas*
7928	*Proteobacteria*	*Gammaproteobacte*	*Pseudomonadales*	*Pseudomonadaceae*	*Pseudomonas*	*Pseudomonas*
7929	*Proteobacteria*	*Gammaproteobacte*	*Pseudomonadales*	*Pseudomonadaceae*	*Pseudomonas*	*Pseudomonas*
7930	*Proteobacteria*	*Gammaproteobacte*	*Pseudomonadales*	*Pseudomonadaceae*	*Pseudomonas*	*Pseudomonas*
7931	*Proteobacteria*	*Gammaproteobacte*	*Pseudomonadales*	*Pseudomonadaceae*	*Pseudomonas*	*Pseudomonas*
7932	*Proteobacteria*	*Gammaproteobacte*	*Pseudomonadales*	*Pseudomonadaceae*	*Pseudomonas*	*Pseudomonas*
7933	*Proteobacteria*	*Gammaproteobacte*	*Pseudomonadales*	*Pseudomonadaceae*	*Pseudomonas*	*Pseudomonas*
7934	*Proteobacteria*	*Gammaproteobacte*	*Pseudomonadales*	*Pseudomonadaceae*	*Pseudomonas*	*Pseudomonas*
7935	*Proteobacteria*	*Gammaproteobacte*	*Pseudomonadales*	*Pseudomonadaceae*	*Pseudomonas*	*Pseudomonas*
7936	*Proteobacteria*	*Gammaproteobacte*	*Pseudomonadales*	*Pseudomonadaceae*	*Pseudomonas*	*Pseudomonas*
7937	*Proteobacteria*	*Gammaproteobacte*	*Pseudomonadales*	*Pseudomonadaceae*	*Pseudomonas*	*Pseudomonas*
7938	*Proteobacteria*	*Gammaproteobacte*	*Pseudomonadales*	*Pseudomonadaceae*	*Pseudomonas*	*Pseudomonas*
7939	*Proteobacteria*	*Gammaproteobacte*	*Pseudomonadales*	*Pseudomonadaceae*	*Pseudomonas*	*Pseudomonas*
7940	*Proteobacteria*	*Gammaproteobacte*	*Pseudomonadales*	*Pseudomonadaceae*	*Pseudomonas*	*Pseudomonas*
7941	*Proteobacteria*	*Gammaproteobacte*	*Pseudomonadales*	*Pseudomonadaceae*	*Pseudomonas*	*Pseudomonas*
7942	*Proteobacteria*	*Gammaproteobacte*	*Pseudomonadales*	*Pseudomonadaceae*	*Pseudomonas*	*Pseudomonas*
7943	*Proteobacteria*	*Gammaproteobacte*	*Pseudomonadales*	*Pseudomonadaceae*	*Pseudomonas*	*Pseudomonas*
7944	*Proteobacteria*	*Gammaproteobacte*	*Pseudomonadales*	*Pseudomonadaceae*	*Pseudomonas*	*Pseudomonas*
7945	*Proteobacteria*	*Gammaproteobacte*	*Pseudomonadales*	*Pseudomonadaceae*	*Pseudomonas*	*Pseudomonas*
7946	*Proteobacteria*	*Gammaproteobacte*	*Pseudomonadales*	*Pseudomonadaceae*	*Pseudomonas*	*Pseudomonas*
7947	*Proteobacteria*	*Gammaproteobacte*	*Pseudomonadales*	*Pseudomonadaceae*	*Pseudomonas*	*Pseudomonas*
7948	*Proteobacteria*	*Gammaproteobacte*	*Pseudomonadales*	*Pseudomonadaceae*	*Pseudomonas*	*Pseudomonas*
7949	*Proteobacteria*	*Gammaproteobacte*	*Pseudomonadales*	*Pseudomonadaceae*	*Pseudomonas*	*Pseudomonas*
7950	*Proteobacteria*	*Gammaproteobacte*	*Pseudomonadales*	*Pseudomonadaceae*	*Pseudomonas*	*Pseudomonas*
7951	*Proteobacteria*	*Gammaproteobacte*	*Pseudomonadales*	*Pseudomonadaceae*	*Pseudomonas*	*Pseudomonas*
7952	*Proteobacteria*	*Gammaproteobacte*	*Pseudomonadales*	*Pseudomonadaceae*	*Pseudomonas*	*Pseudomonas*

Table S4. Isolated strains by culturomics

bacteriaId	*phylum*	*class*	*order*	*family*	*genus*	*species*
7953	*Proteobacteria*	*Gammaproteobacte*	*Pseudomonadales*	*Pseudomonadaceae*	*Pseudomonas*	*Pseudomonas*
7954	*Proteobacteria*	*Gammaproteobacte*	*Pseudomonadales*	*Pseudomonadaceae*	*Pseudomonas*	*Pseudomonas*
7955	*Proteobacteria*	*Gammaproteobacte*	*Pseudomonadales*	*Pseudomonadaceae*	*Pseudomonas*	*Pseudomonas*
7956	*Proteobacteria*	*Gammaproteobacte*	*Pseudomonadales*	*Pseudomonadaceae*	*Pseudomonas*	*Pseudomonas*
7957	*Proteobacteria*	*Gammaproteobacte*	*Pseudomonadales*	*Pseudomonadaceae*	*Pseudomonas*	*Pseudomonas*
7958	*Proteobacteria*	*Gammaproteobacte*	*Pseudomonadales*	*Pseudomonadaceae*	*Pseudomonas*	*Pseudomonas*
7959	*Proteobacteria*	*Gammaproteobacte*	*Pseudomonadales*	*Pseudomonadaceae*	*Pseudomonas*	*Pseudomonas*
7960	*Proteobacteria*	*Gammaproteobacte*	*Pseudomonadales*	*Pseudomonadaceae*	*Pseudomonas*	*Pseudomonas*
7961	*Proteobacteria*	*Gammaproteobacte*	*Pseudomonadales*	*Pseudomonadaceae*	*Pseudomonas*	*Pseudomonas*
7962	*Proteobacteria*	*Gammaproteobacte*	*Pseudomonadales*	*Pseudomonadaceae*	*Pseudomonas*	*Pseudomonas*
7963	*Proteobacteria*	*Gammaproteobacte*	*Pseudomonadales*	*Pseudomonadaceae*	*Pseudomonas*	*Pseudomonas*
7964	*Proteobacteria*	*Gammaproteobacte*	*Pseudomonadales*	*Pseudomonadaceae*	*Pseudomonas*	*Pseudomonas*
7965	*Proteobacteria*	*Gammaproteobacte*	*Pseudomonadales*	*Pseudomonadaceae*	*Pseudomonas*	*Pseudomonas*
7966	*Proteobacteria*	*Gammaproteobacte*	*Pseudomonadales*	*Pseudomonadaceae*	*Pseudomonas*	*Pseudomonas*
7967	*Proteobacteria*	*Gammaproteobacte*	*Pseudomonadales*	*Pseudomonadaceae*	*Pseudomonas*	*Pseudomonas*
7968	*Proteobacteria*	*Gammaproteobacte*	*Pseudomonadales*	*Pseudomonadaceae*	*Pseudomonas*	*Pseudomonas*
7969	*Proteobacteria*	*Gammaproteobacte*	*Pseudomonadales*	*Pseudomonadaceae*	*Pseudomonas*	*Pseudomonas*
7970	*Proteobacteria*	*Gammaproteobacte*	*Pseudomonadales*	*Pseudomonadaceae*	*Pseudomonas*	*Pseudomonas*
7971	*Proteobacteria*	*Gammaproteobacte*	*Pseudomonadales*	*Pseudomonadaceae*	*Pseudomonas*	*Pseudomonas*
7972	*Proteobacteria*	*Gammaproteobacte*	*Pseudomonadales*	*Pseudomonadaceae*	*Pseudomonas*	*Pseudomonas*
7973	*Proteobacteria*	*Gammaproteobacte*	*Pseudomonadales*	*Pseudomonadaceae*	*Pseudomonas*	*Pseudomonas*
7974	*Proteobacteria*	*Gammaproteobacte*	*Pseudomonadales*	*Pseudomonadaceae*	*Pseudomonas*	*Pseudomonas*
7975	*Proteobacteria*	*Gammaproteobacte*	*Pseudomonadales*	*Pseudomonadaceae*	*Pseudomonas*	*Pseudomonas*
7976	*Proteobacteria*	*Gammaproteobacte*	*Pseudomonadales*	*Pseudomonadaceae*	*Pseudomonas*	*Pseudomonas*
7977	*Proteobacteria*	*Gammaproteobacte*	*Pseudomonadales*	*Pseudomonadaceae*	*Pseudomonas*	*Pseudomonas*
7978	*Proteobacteria*	*Gammaproteobacte*	*Pseudomonadales*	*Pseudomonadaceae*	*Pseudomonas*	*Pseudomonas*
7979	*Proteobacteria*	*Gammaproteobacte*	*Pseudomonadales*	*Pseudomonadaceae*	*Pseudomonas*	*Pseudomonas*
7980	*Proteobacteria*	*Gammaproteobacte*	*Pseudomonadales*	*Pseudomonadaceae*	*Pseudomonas*	*Pseudomonas*

Table S4. Isolated strains by culturomics

bacteriald	*phylum*	*class*	*order*	*family*	*genus*	*species*
7981	*Proteobacteria*	*Gammaproteobacte*	*Pseudomonadales*	*Pseudomonadaceae*	*Pseudomonas*	*Pseudomonas*
7982	*Proteobacteria*	*Gammaproteobacte*	*Pseudomonadales*	*Pseudomonadaceae*	*Pseudomonas*	*Pseudomonas*
7983	*Proteobacteria*	*Gammaproteobacte*	*Pseudomonadales*	*Pseudomonadaceae*	*Pseudomonas*	*Pseudomonas*
7984	*Proteobacteria*	*Gammaproteobacte*	*Pseudomonadales*	*Pseudomonadaceae*	*Pseudomonas*	*Pseudomonas*
7985	*Proteobacteria*	*Gammaproteobacte*	*Pseudomonadales*	*Pseudomonadaceae*	*Pseudomonas*	*Pseudomonas*
7986	*Proteobacteria*	*Gammaproteobacte*	*Pseudomonadales*	*Pseudomonadaceae*	*Pseudomonas*	*Pseudomonas*
7987	*Proteobacteria*	*Gammaproteobacte*	*Pseudomonadales*	*Pseudomonadaceae*	*Pseudomonas*	*Pseudomonas*
7988	*Proteobacteria*	*Gammaproteobacte*	*Pseudomonadales*	*Pseudomonadaceae*	*Pseudomonas*	*Pseudomonas*
7989	*Proteobacteria*	*Gammaproteobacte*	*Pseudomonadales*	*Pseudomonadaceae*	*Pseudomonas*	*Pseudomonas*
7990	*Proteobacteria*	*Gammaproteobacte*	*Pseudomonadales*	*Pseudomonadaceae*	*Pseudomonas*	*Pseudomonas*
7991	*Proteobacteria*	*Gammaproteobacte*	*Pseudomonadales*	*Pseudomonadaceae*	*Pseudomonas*	*Pseudomonas*
7992	*Proteobacteria*	*Gammaproteobacte*	*Pseudomonadales*	*Pseudomonadaceae*	*Pseudomonas*	*Pseudomonas*
7993	*Proteobacteria*	*Gammaproteobacte*	*Pseudomonadales*	*Pseudomonadaceae*	*Pseudomonas*	*Pseudomonas*
7994	*Proteobacteria*	*Gammaproteobacte*	*Pseudomonadales*	*Pseudomonadaceae*	*Pseudomonas*	*Pseudomonas*
7995	*Proteobacteria*	*Gammaproteobacte*	*Pseudomonadales*	*Pseudomonadaceae*	*Pseudomonas*	*Pseudomonas*
7996	*Proteobacteria*	*Gammaproteobacte*	*Pseudomonadales*	*Pseudomonadaceae*	*Pseudomonas*	*Pseudomonas*
7997	*Proteobacteria*	*Gammaproteobacte*	*Pseudomonadales*	*Pseudomonadaceae*	*Pseudomonas*	*Pseudomonas*
7998	*Proteobacteria*	*Gammaproteobacte*	*Pseudomonadales*	*Pseudomonadaceae*	*Pseudomonas*	*Pseudomonas*
7999	*Proteobacteria*	*Gammaproteobacte*	*Pseudomonadales*	*Pseudomonadaceae*	*Pseudomonas*	*Pseudomonas*
8000	*Proteobacteria*	*Gammaproteobacte*	*Pseudomonadales*	*Pseudomonadaceae*	*Pseudomonas*	*Pseudomonas*
8001	*Proteobacteria*	*Gammaproteobacte*	*Pseudomonadales*	*Pseudomonadaceae*	*Pseudomonas*	*Pseudomonas*
8002	*Proteobacteria*	*Gammaproteobacte*	*Pseudomonadales*	*Pseudomonadaceae*	*Pseudomonas*	*Pseudomonas*
8003	*Proteobacteria*	*Gammaproteobacte*	*Pseudomonadales*	*Pseudomonadaceae*	*Pseudomonas*	*Pseudomonas*
8004	*Proteobacteria*	*Gammaproteobacte*	*Pseudomonadales*	*Pseudomonadaceae*	*Pseudomonas*	*Pseudomonas*
8005	*Proteobacteria*	*Gammaproteobacte*	*Pseudomonadales*	*Pseudomonadaceae*	*Pseudomonas*	*Pseudomonas*
8006	*Proteobacteria*	*Gammaproteobacte*	*Pseudomonadales*	*Pseudomonadaceae*	*Pseudomonas*	*Pseudomonas*
8007	*Proteobacteria*	*Gammaproteobacte*	*Pseudomonadales*	*Pseudomonadaceae*	*Pseudomonas*	*Pseudomonas*
8008	*Proteobacteria*	*Gammaproteobacte*	*Pseudomonadales*	*Pseudomonadaceae*	*Pseudomonas*	*Pseudomonas*

Table S4. Isolated strains by culturomics

bacteriaId	*phylum*	*class*	*order*	*family*	*genus*	*species*
8009	*Proteobacteria*	*Gammaproteobacte*	*Pseudomonadales*	*Pseudomonadaceae*	*Pseudomonas*	*Pseudomonas*
8010	*Proteobacteria*	*Gammaproteobacte*	*Pseudomonadales*	*Pseudomonadaceae*	*Pseudomonas*	*Pseudomonas*
8011	*Proteobacteria*	*Gammaproteobacte*	*Pseudomonadales*	*Pseudomonadaceae*	*Pseudomonas*	*Pseudomonas*
8012	*Proteobacteria*	*Gammaproteobacte*	*Pseudomonadales*	*Pseudomonadaceae*	*Pseudomonas*	*Pseudomonas*
8013	*Proteobacteria*	*Gammaproteobacte*	*Pseudomonadales*	*Pseudomonadaceae*	*Pseudomonas*	*Pseudomonas*
8014	*Proteobacteria*	*Gammaproteobacte*	*Pseudomonadales*	*Pseudomonadaceae*	*Pseudomonas*	*Pseudomonas*
8015	*Proteobacteria*	*Gammaproteobacte*	*Pseudomonadales*	*Pseudomonadaceae*	*Pseudomonas*	*Pseudomonas*
8016	*Proteobacteria*	*Gammaproteobacte*	*Pseudomonadales*	*Pseudomonadaceae*	*Pseudomonas*	*Pseudomonas*
8017	*Proteobacteria*	*Gammaproteobacte*	*Pseudomonadales*	*Pseudomonadaceae*	*Pseudomonas*	*Pseudomonas*
8018	*Proteobacteria*	*Gammaproteobacte*	*Pseudomonadales*	*Pseudomonadaceae*	*Pseudomonas*	*Pseudomonas*
8019	*Proteobacteria*	*Gammaproteobacte*	*Pseudomonadales*	*Pseudomonadaceae*	*Pseudomonas*	*Pseudomonas*
8020	*Proteobacteria*	*Gammaproteobacte*	*Pseudomonadales*	*Pseudomonadaceae*	*Pseudomonas*	*Pseudomonas*
8021	*Proteobacteria*	*Gammaproteobacte*	*Pseudomonadales*	*Pseudomonadaceae*	*Pseudomonas*	*Pseudomonas*
8022	*Proteobacteria*	*Gammaproteobacte*	*Pseudomonadales*	*Pseudomonadaceae*	*Pseudomonas*	*Pseudomonas*
8023	*Proteobacteria*	*Gammaproteobacte*	*Pseudomonadales*	*Pseudomonadaceae*	*Pseudomonas*	*Pseudomonas*
8024	*Proteobacteria*	*Gammaproteobacte*	*Pseudomonadales*	*Pseudomonadaceae*	*Pseudomonas*	*Pseudomonas*
8025	*Proteobacteria*	*Gammaproteobacte*	*Pseudomonadales*	*Pseudomonadaceae*	*Pseudomonas*	*Pseudomonas*
8026	*Proteobacteria*	*Gammaproteobacte*	*Pseudomonadales*	*Pseudomonadaceae*	*Pseudomonas*	*Pseudomonas*
8027	*Proteobacteria*	*Gammaproteobacte*	*Pseudomonadales*	*Pseudomonadaceae*	*Pseudomonas*	*Pseudomonas*
8028	*Proteobacteria*	*Gammaproteobacte*	*Pseudomonadales*	*Pseudomonadaceae*	*Pseudomonas*	*Pseudomonas*
8029	*Proteobacteria*	*Gammaproteobacte*	*Pseudomonadales*	*Pseudomonadaceae*	*Pseudomonas*	*Pseudomonas*
8030	*Proteobacteria*	*Gammaproteobacte*	*Pseudomonadales*	*Pseudomonadaceae*	*Pseudomonas*	*Pseudomonas*
8031	*Proteobacteria*	*Gammaproteobacte*	*Pseudomonadales*	*Pseudomonadaceae*	*Pseudomonas*	*Pseudomonas*
8032	*Proteobacteria*	*Gammaproteobacte*	*Pseudomonadales*	*Pseudomonadaceae*	*Pseudomonas*	*Pseudomonas*
8033	*Proteobacteria*	*Gammaproteobacte*	*Pseudomonadales*	*Pseudomonadaceae*	*Pseudomonas*	*Pseudomonas*
8034	*Proteobacteria*	*Gammaproteobacte*	*Pseudomonadales*	*Pseudomonadaceae*	*Pseudomonas*	*Pseudomonas*
8035	*Proteobacteria*	*Gammaproteobacte*	*Pseudomonadales*	*Pseudomonadaceae*	*Pseudomonas*	*Pseudomonas*
8036	*Proteobacteria*	*Gammaproteobacte*	*Pseudomonadales*	*Pseudomonadaceae*	*Pseudomonas*	*Pseudomonas*

Table S4. Isolated strains by culturomics

bacteriaId	*phylum*	*class*	*order*	*family*	*genus*	*species*
8037	*Proteobacteria*	*Gammaproteobacte*	*Pseudomonadales*	*Pseudomonadaceae*	*Pseudomonas*	*Pseudomonas*
8038	*Proteobacteria*	*Gammaproteobacte*	*Pseudomonadales*	*Pseudomonadaceae*	*Pseudomonas*	*Pseudomonas*
8039	*Proteobacteria*	*Gammaproteobacte*	*Pseudomonadales*	*Pseudomonadaceae*	*Pseudomonas*	*Pseudomonas*
8040	*Proteobacteria*	*Gammaproteobacte*	*Pseudomonadales*	*Pseudomonadaceae*	*Pseudomonas*	*Pseudomonas*
8041	*Proteobacteria*	*Gammaproteobacte*	*Pseudomonadales*	*Pseudomonadaceae*	*Pseudomonas*	*Pseudomonas*
8042	*Proteobacteria*	*Gammaproteobacte*	*Pseudomonadales*	*Pseudomonadaceae*	*Pseudomonas*	*Pseudomonas*
8043	*Proteobacteria*	*Gammaproteobacte*	*Pseudomonadales*	*Pseudomonadaceae*	*Pseudomonas*	*Pseudomonas*
8044	*Proteobacteria*	*Gammaproteobacte*	*Pseudomonadales*	*Pseudomonadaceae*	*Pseudomonas*	*Pseudomonas*
8045	*Proteobacteria*	*Gammaproteobacte*	*Pseudomonadales*	*Pseudomonadaceae*	*Pseudomonas*	*Pseudomonas*
8046	*Proteobacteria*	*Gammaproteobacte*	*Pseudomonadales*	*Pseudomonadaceae*	*Pseudomonas*	*Pseudomonas*
8047	*Proteobacteria*	*Gammaproteobacte*	*Pseudomonadales*	*Pseudomonadaceae*	*Pseudomonas*	*Pseudomonas*
8048	*Proteobacteria*	*Gammaproteobacte*	*Pseudomonadales*	*Pseudomonadaceae*	*Pseudomonas*	*Pseudomonas*
8049	*Proteobacteria*	*Gammaproteobacte*	*Pseudomonadales*	*Pseudomonadaceae*	*Pseudomonas*	*Pseudomonas*
8050	*Proteobacteria*	*Gammaproteobacte*	*Pseudomonadales*	*Pseudomonadaceae*	*Pseudomonas*	*Pseudomonas*
8051	*Proteobacteria*	*Gammaproteobacte*	*Pseudomonadales*	*Pseudomonadaceae*	*Pseudomonas*	*Pseudomonas*
8052	*Proteobacteria*	*Gammaproteobacte*	*Pseudomonadales*	*Pseudomonadaceae*	*Pseudomonas*	*Pseudomonas*
8053	*Proteobacteria*	*Gammaproteobacte*	*Pseudomonadales*	*Pseudomonadaceae*	*Pseudomonas*	*Pseudomonas*
8054	*Proteobacteria*	*Gammaproteobacte*	*Pseudomonadales*	*Pseudomonadaceae*	*Pseudomonas*	*Pseudomonas*
8055	*Proteobacteria*	*Gammaproteobacte*	*Pseudomonadales*	*Pseudomonadaceae*	*Pseudomonas*	*Pseudomonas*
8056	*Proteobacteria*	*Gammaproteobacte*	*Pseudomonadales*	*Pseudomonadaceae*	*Pseudomonas*	*Pseudomonas*
8057	*Proteobacteria*	*Gammaproteobacte*	*Pseudomonadales*	*Pseudomonadaceae*	*Pseudomonas*	*Pseudomonas*
8058	*Proteobacteria*	*Gammaproteobacte*	*Pseudomonadales*	*Pseudomonadaceae*	*Pseudomonas*	*Pseudomonas*
8059	*Proteobacteria*	*Gammaproteobacte*	*Pseudomonadales*	*Pseudomonadaceae*	*Pseudomonas*	*Pseudomonas*
8060	*Proteobacteria*	*Gammaproteobacte*	*Pseudomonadales*	*Pseudomonadaceae*	*Pseudomonas*	*Pseudomonas*
8061	*Proteobacteria*	*Gammaproteobacte*	*Pseudomonadales*	*Pseudomonadaceae*	*Pseudomonas*	*Pseudomonas*
8062	*Proteobacteria*	*Gammaproteobacte*	*Pseudomonadales*	*Pseudomonadaceae*	*Pseudomonas*	*Pseudomonas*
8063	*Proteobacteria*	*Gammaproteobacte*	*Pseudomonadales*	*Pseudomonadaceae*	*Pseudomonas*	*Pseudomonas*
8064	*Proteobacteria*	*Gammaproteobacte*	*Pseudomonadales*	*Pseudomonadaceae*	*Pseudomonas*	*Pseudomonas*

Table S4. Isolated strains by culturomics

bacteriaId	phylum	class	order	family	genus	species
8065	*Proteobacteria*	*Gammaproteobacte*	*Pseudomonadales*	*Pseudomonadaceae*	*Pseudomonas*	*Pseudomonas*
8066	*Proteobacteria*	*Gammaproteobacte*	*Pseudomonadales*	*Pseudomonadaceae*	*Pseudomonas*	*Pseudomonas*
8067	*Proteobacteria*	*Gammaproteobacte*	*Pseudomonadales*	*Pseudomonadaceae*	*Pseudomonas*	*Pseudomonas*
8068	*Proteobacteria*	*Gammaproteobacte*	*Pseudomonadales*	*Pseudomonadaceae*	*Pseudomonas*	*Pseudomonas*
8069	*Proteobacteria*	*Gammaproteobacte*	*Pseudomonadales*	*Pseudomonadaceae*	*Pseudomonas*	*Pseudomonas*
8070	*Proteobacteria*	*Gammaproteobacte*	*Pseudomonadales*	*Pseudomonadaceae*	*Pseudomonas*	*Pseudomonas*
8071	*Proteobacteria*	*Gammaproteobacte*	*Pseudomonadales*	*Pseudomonadaceae*	*Pseudomonas*	*Pseudomonas*
8072	*Proteobacteria*	*Gammaproteobacte*	*Pseudomonadales*	*Pseudomonadaceae*	*Pseudomonas*	*Pseudomonas*
8073	*Proteobacteria*	*Gammaproteobacte*	*Pseudomonadales*	*Pseudomonadaceae*	*Pseudomonas*	*Pseudomonas*
8074	*Proteobacteria*	*Gammaproteobacte*	*Pseudomonadales*	*Pseudomonadaceae*	*Pseudomonas*	*Pseudomonas*
8075	*Proteobacteria*	*Gammaproteobacte*	*Pseudomonadales*	*Pseudomonadaceae*	*Pseudomonas*	*Pseudomonas*
8076	*Proteobacteria*	*Gammaproteobacte*	*Pseudomonadales*	*Pseudomonadaceae*	*Pseudomonas*	*Pseudomonas*
8077	*Proteobacteria*	*Gammaproteobacte*	*Pseudomonadales*	*Pseudomonadaceae*	*Pseudomonas*	*Pseudomonas*
8078	*Proteobacteria*	*Gammaproteobacte*	*Pseudomonadales*	*Pseudomonadaceae*	*Pseudomonas*	*Pseudomonas*
8079	*Proteobacteria*	*Gammaproteobacte*	*Pseudomonadales*	*Pseudomonadaceae*	*Pseudomonas*	*Pseudomonas*
8080	*Proteobacteria*	*Gammaproteobacte*	*Pseudomonadales*	*Pseudomonadaceae*	*Pseudomonas*	*Pseudomonas*
8081	*Proteobacteria*	*Gammaproteobacte*	*Pseudomonadales*	*Pseudomonadaceae*	*Pseudomonas*	*Pseudomonas*
8082	*Proteobacteria*	*Gammaproteobacte*	*Pseudomonadales*	*Pseudomonadaceae*	*Pseudomonas*	*Pseudomonas*
8083	*Proteobacteria*	*Gammaproteobacte*	*Pseudomonadales*	*Pseudomonadaceae*	*Pseudomonas*	*Pseudomonas*
8084	*Proteobacteria*	*Gammaproteobacte*	*Pseudomonadales*	*Pseudomonadaceae*	*Pseudomonas*	*Pseudomonas*
8085	*Proteobacteria*	*Gammaproteobacte*	*Pseudomonadales*	*Pseudomonadaceae*	*Pseudomonas*	*Pseudomonas*
8086	*Proteobacteria*	*Gammaproteobacte*	*Pseudomonadales*	*Pseudomonadaceae*	*Pseudomonas*	*Pseudomonas*
8087	*Proteobacteria*	*Gammaproteobacte*	*Pseudomonadales*	*Pseudomonadaceae*	*Pseudomonas*	*Pseudomonas*
8088	*Proteobacteria*	*Gammaproteobacte*	*Pseudomonadales*	*Pseudomonadaceae*	*Pseudomonas*	*Pseudomonas*
8089	*Proteobacteria*	*Gammaproteobacte*	*Pseudomonadales*	*Pseudomonadaceae*	*Pseudomonas*	*Pseudomonas*
8090	*Proteobacteria*	*Gammaproteobacte*	*Pseudomonadales*	*Pseudomonadaceae*	*Pseudomonas*	*Pseudomonas*
8091	*Proteobacteria*	*Gammaproteobacte*	*Pseudomonadales*	*Pseudomonadaceae*	*Pseudomonas*	*Pseudomonas*
8092	*Proteobacteria*	*Gammaproteobacte*	*Pseudomonadales*	*Pseudomonadaceae*	*Pseudomonas*	*Pseudomonas*

Table S4. Isolated strains by culturomics

bacteriaId	*phylum*	*class*	*order*	*family*	*genus*	*species*
8093	*Proteobacteria*	*Gammaproteobacte*	*Pseudomonadales*	*Pseudomonadaceae*	*Pseudomonas*	*Pseudomonas*
8094	*Proteobacteria*	*Gammaproteobacte*	*Pseudomonadales*	*Pseudomonadaceae*	*Pseudomonas*	*Pseudomonas*
8095	*Proteobacteria*	*Gammaproteobacte*	*Pseudomonadales*	*Pseudomonadaceae*	*Pseudomonas*	*Pseudomonas*
8096	*Proteobacteria*	*Gammaproteobacte*	*Pseudomonadales*	*Pseudomonadaceae*	*Pseudomonas*	*Pseudomonas*
8097	*Proteobacteria*	*Gammaproteobacte*	*Pseudomonadales*	*Pseudomonadaceae*	*Pseudomonas*	*Pseudomonas*
8098	*Proteobacteria*	*Gammaproteobacte*	*Pseudomonadales*	*Pseudomonadaceae*	*Pseudomonas*	*Pseudomonas*
8099	*Proteobacteria*	*Gammaproteobacte*	*Pseudomonadales*	*Pseudomonadaceae*	*Pseudomonas*	*Pseudomonas*
8100	*Proteobacteria*	*Gammaproteobacte*	*Pseudomonadales*	*Pseudomonadaceae*	*Pseudomonas*	*Pseudomonas*
8101	*Proteobacteria*	*Gammaproteobacte*	*Pseudomonadales*	*Pseudomonadaceae*	*Pseudomonas*	*Pseudomonas*
8102	*Proteobacteria*	*Gammaproteobacte*	*Pseudomonadales*	*Pseudomonadaceae*	*Pseudomonas*	*Pseudomonas*
8103	*Proteobacteria*	*Gammaproteobacte*	*Pseudomonadales*	*Pseudomonadaceae*	*Pseudomonas*	*Pseudomonas*
8104	*Proteobacteria*	*Gammaproteobacte*	*Pseudomonadales*	*Pseudomonadaceae*	*Pseudomonas*	*Pseudomonas*
8105	*Proteobacteria*	*Gammaproteobacte*	*Pseudomonadales*	*Pseudomonadaceae*	*Pseudomonas*	*Pseudomonas*
8106	*Proteobacteria*	*Gammaproteobacte*	*Pseudomonadales*	*Pseudomonadaceae*	*Pseudomonas*	*Pseudomonas*
8107	*Proteobacteria*	*Gammaproteobacte*	*Pseudomonadales*	*Pseudomonadaceae*	*Pseudomonas*	*Pseudomonas*
8108	*Proteobacteria*	*Gammaproteobacte*	*Pseudomonadales*	*Pseudomonadaceae*	*Pseudomonas*	*Pseudomonas*
8109	*Proteobacteria*	*Gammaproteobacte*	*Pseudomonadales*	*Pseudomonadaceae*	*Pseudomonas*	*Pseudomonas*
8110	*Proteobacteria*	*Gammaproteobacte*	*Pseudomonadales*	*Pseudomonadaceae*	*Pseudomonas*	*Pseudomonas*
8111	*Proteobacteria*	*Gammaproteobacte*	*Pseudomonadales*	*Pseudomonadaceae*	*Pseudomonas*	*Pseudomonas*
8112	*Proteobacteria*	*Gammaproteobacte*	*Pseudomonadales*	*Pseudomonadaceae*	*Pseudomonas*	*Pseudomonas*
8113	*Proteobacteria*	*Gammaproteobacte*	*Pseudomonadales*	*Pseudomonadaceae*	*Pseudomonas*	*Pseudomonas*
8114	*Proteobacteria*	*Gammaproteobacte*	*Pseudomonadales*	*Pseudomonadaceae*	*Pseudomonas*	*Pseudomonas*
8115	*Proteobacteria*	*Gammaproteobacte*	*Pseudomonadales*	*Pseudomonadaceae*	*Pseudomonas*	*Pseudomonas*
8116	*Proteobacteria*	*Gammaproteobacte*	*Pseudomonadales*	*Pseudomonadaceae*	*Pseudomonas*	*Pseudomonas*
8117	*Proteobacteria*	*Gammaproteobacte*	*Pseudomonadales*	*Pseudomonadaceae*	*Pseudomonas*	*Pseudomonas*
8118	*Proteobacteria*	*Gammaproteobacte*	*Pseudomonadales*	*Pseudomonadaceae*	*Pseudomonas*	*Pseudomonas*
8119	*Proteobacteria*	*Gammaproteobacte*	*Pseudomonadales*	*Pseudomonadaceae*	*Pseudomonas*	*Pseudomonas*
8120	*Proteobacteria*	*Gammaproteobacte*	*Pseudomonadales*	*Pseudomonadaceae*	*Pseudomonas*	*Pseudomonas*

Table S4. Isolated strains by culturomics

bacteriaId	phylum	class	order	family	genus	species
8121	*Proteobacteria*	*Gammaproteobacte*	*Pseudomonadales*	*Pseudomonadaceae*	*Pseudomonas*	*Pseudomonas*
8122	*Proteobacteria*	*Gammaproteobacte*	*Pseudomonadales*	*Pseudomonadaceae*	*Pseudomonas*	*Pseudomonas*
8123	*Proteobacteria*	*Gammaproteobacte*	*Pseudomonadales*	*Pseudomonadaceae*	*Pseudomonas*	*Pseudomonas*
8124	*Proteobacteria*	*Gammaproteobacte*	*Pseudomonadales*	*Pseudomonadaceae*	*Pseudomonas*	*Pseudomonas*
8125	*Proteobacteria*	*Gammaproteobacte*	*Pseudomonadales*	*Pseudomonadaceae*	*Pseudomonas*	*Pseudomonas*
8126	*Proteobacteria*	*Gammaproteobacte*	*Xanthomonadales*	*Xanthomonadaceae*	*Stenotrophomonas*	*Stenotrophomonas*
8127	*undefined*	*undefined*	*undefined*	*undefined*	*undefined*	*undefined*
8128	*undefined*	*undefined*	*undefined*	*undefined*	*undefined*	*undefined*
8129	*undefined*	*undefined*	*undefined*	*undefined*	*undefined*	*undefined*
8130	*undefined*	*undefined*	*undefined*	*undefined*	*undefined*	*undefined*
8131	*unknown*	*unknown*	*unknown*	*unknown*	*unknown*	*agfgfdhgfjhagfj*
8132	*unknown*	*unknown*	*unknown*	*unknown*	*unknown*	*agfh*
8133	*unknown*	*unknown*	*unknown*	*unknown*	*unknown*	*agfh*
8134	*unknown*	*unknown*	*unknown*	*unknown*	*unknown*	*agfhgfhgjkjl*
8135	*unknown*	*unknown*	*unknown*	*unknown*	*unknown*	*DFJDDGJFK*
8136	*unknown*	*unknown*	*unknown*	*unknown*	*unknown*	*SHGJGJ*
8137	*unknown*	*unknown*	*unknown*	*unknown*	*unknown*	*unknown B1351*
8138	*unknown*	*unknown*	*unknown*	*unknown*	*unknown*	*unknown B1352*
8139	*unknown*	*unknown*	*unknown*	*unknown*	*unknown*	*unknown B1352*
8140	*unknown*	*unknown*	*unknown*	*unknown*	*unknown*	*unknown B1352*
8141	*unknown*	*unknown*	*unknown*	*unknown*	*unknown*	*unknown B1352*
8142	*unknown*	*unknown*	*unknown*	*unknown*	*unknown*	*unknown B135E*
8143	*unknown*	*unknown*	*unknown*	*unknown*	*unknown*	*unknown B139D*
8144	*unknown*	*unknown*	*unknown*	*unknown*	*unknown*	*unknown B139E*
8145	*unknown*	*unknown*	*unknown*	*unknown*	*unknown*	*unknown B13FN*
8146	*unknown*	*unknown*	*unknown*	*unknown*	*unknown*	*unknown B13G8*
8147	*unknown*	*unknown*	*unknown*	*unknown*	*unknown*	*unknown B13R2*
8148	*unknown*	*unknown*	*unknown*	*unknown*	*unknown*	*unknown B13T6*

Table S4. Isolated strains by culturomics

bacteriaId	*phylum*	*class*	*order*	*family*	*genus*	*species*
8149	*unknown*	*unknown*	*unknown*	*unknown*	*unknown*	*unknown B13TC*
8150	*unknown*	*unknown*	*unknown*	*unknown*	*unknown*	*unknown B1410*
8151	*unknown*	*unknown*	*unknown*	*unknown*	*unknown*	*unknown B14J5*
8152	*unknown*	*unknown*	*unknown*	*unknown*	*unknown*	*unknown B14RH*
8153	*unknown*	*unknown*	*unknown*	*unknown*	*unknown*	*unknown B14RH*
8154	*unknown*	*unknown*	*unknown*	*unknown*	*unknown*	*unknown B14RH*
8155	*unknown*	*unknown*	*unknown*	*unknown*	*unknown*	*unknown BCUC*
8156	*unknown*	*unknown*	*unknown*	*unknown*	*unknown*	*unknown BJGU*
8157	*unknown*	*unknown*	*unknown*	*unknown*	*unknown*	*unknown BJGU*
8158	*unknown*	*unknown*	*unknown*	*unknown*	*unknown*	*unknown BJU5*
8159	*unknown*	*unknown*	*unknown*	*unknown*	*unknown*	*unknownB11UH*
8160	*unknown*	*unknown*	*unknown*	*unknown*	*unknown*	*unknownB140R*
8161	*unknown*	*unknown*	*unknown*	*unknown*	*unknown*	*unknown*
8162	*unknown*	*unknown*	*unknown*	*unknown*	*unknown*	*unknown*
8163	*unknown*	*unknown*	*unknown*	*unknown*	*unknown*	*unknown*
8164	*unknown*	*unknown*	*unknown*	*unknown*	*unknown*	*unknown B14RH*
8165	*unknown*	*unknown*	*unknown*	*unknown*	*unknown*	*unknown B1EHT*

Table S5. The cultured strains and their culture atmospheres

**Atmosphere**	***species***
anaerobic/42 degree	*Acidaminococcus fermentans*
anaerobic/42 degree	*Acidaminococcus fermentans*
anaerobic/37 degree	*Acidaminococcus intestini*
anaerobic/37 degree	*Acidaminococcus intestini*
anaerobic/37 degree	*Acidaminococcus intestini*
anaerobic/37 degree	*Acidaminococcus intestini*
anaerobic/37 degree	*Acidaminococcus intestini*
anaerobic/37 degree	*Acidaminococcus intestini*
anaerobic/37 degree	*Acidaminococcus intestini*
anaerobic/37 degree	*Acidaminococcus intestini*
anaerobic/37 degree	*Acidaminococcus intestini*
anaerobic/37 degree	*Acidaminococcus intestini*
anaerobic/37 degree	*Acidaminococcus intestini*
anaerobic/40-50 degree	*Acidaminococcus intestini*
anaerobic/40-50 degree	*Acidaminococcus intestini*
anaerobic/40-50 degree	*Acidaminococcus intestini*
anaerobic/40-50 degree	*Acidaminococcus intestini*
anaerobic/40-50 degree	*Acidaminococcus intestini*
anaerobic/40-50 degree	*Acidaminococcus intestini*
anaerobic/40-50 degree	*Acidaminococcus intestini*
anaerobic/42 degree	*Acidaminococcus intestini*
anaerobic/42 degree	*Acidaminococcus intestini*
anaerobic/42 degree	*Acidaminococcus intestini*
anaerobic/42 degree	*Acidaminococcus intestini*
anaerobic/42 degree	*Acidaminococcus intestini*
anaerobic/42 degree	*Acidaminococcus intestini*
anaerobic/42 degree	*Acidaminococcus intestini*
anaerobic/42 degree	*Acidaminococcus intestini*
anaerobic/42 degree	*Acidaminococcus intestini*
anaerobic/42 degree	*Acidaminococcus intestini*
anaerobic/42 degree	*Acidaminococcus intestini*
anaerobic/42 degree	*Acidaminococcus intestini*
anaerobic/42 degree	*Acidaminococcus intestini*
anaerobic/42 degree	*Acidaminococcus intestini*
anaerobic/42 degree	*Acidaminococcus intestini*
anaerobic/42 degree	*Acidaminococcus intestini*
anaerobic/37 degree	*Acinetobacter baumannii*
anaerobic/37 degree	*Alistipes finegoldii*
anaerobic/37 degree	*Alistipes onderdonkii*
anaerobic/37 degree	*Alistipes onderdonkii*
anaerobic/37 degree	*Alistipes onderdonkii*
anaerobic/37 degree	*Alistipes onderdonkii*
anaerobic/37 degree	*Alistipes onderdonkii*
anaerobic/37 degree	*Alistipes onderdonkii*

Table S5. The cultured strains and their culture atmospheres

Atmosphere	*species*
anaerobic/37 degree	*Alistipes shahii*
anaerobic/37 degree	*Anaerococcus vaginalis*
anaerobic/37 degree	*Anaerostipes caccae*
anaerobic/42 degree	*Aneurinibacillus aneurinilyticus*
anaerobic/35 degree	*Bacillus altitudinis*
anaerobic/35 degree	*Bacillus altitudinis*
anaerobic/37 degree	*Bacillus altitudinis*
anaerobic/37 degree	*Bacillus altitudinis*
anaerobic/37 degree	*Bacillus altitudinis*
anaerobic/37 degree	*Bacillus altitudinis*
anaerobic/37 degree	*Bacillus altitudinis*
anaerobic/37 degree	*Bacillus altitudinis*
anaerobic/37 degree	*Bacillus altitudinis*
anaerobic/37 degree	*Bacillus altitudinis*
anaerobic/37 degree	*Bacillus altitudinis*
anaerobic/37 degree	*Bacillus altitudinis*
anaerobic/37 degree	*Bacillus altitudinis*
anaerobic/37 degree	*Bacillus altitudinis*
anaerobic/37 degree	*Bacillus altitudinis*
anaerobic/37 degree	*Bacillus altitudinis*
anaerobic/37 degree	*Bacillus altitudinis*
anaerobic/37 degree	*Bacillus altitudinis*
anaerobic/37 degree	*Bacillus altitudinis*
anaerobic/37 degree	*Bacillus altitudinis*
anaerobic/37 degree	*Bacillus altitudinis*
anaerobic/37 degree	*Bacillus altitudinis*
anaerobic/42 degree	*Bacillus altitudinis*
anaerobic/37 degree	*Bacillus amyloliquefaciens*
anaerobic/42 degree	*Bacillus amyloliquefaciens*
anaerobic/37 degree	*Bacillus badius*
anaerobic/37 degree	*Bacillus cereus*
anaerobic/37 degree	*Bacillus cereus*
anaerobic/37 degree	*Bacillus cereus*
anaerobic/37 degree	*Bacillus cereus*
anaerobic/37 degree	*Bacillus cereus*
anaerobic/37 degree	*Bacillus cereus*
anaerobic/37 degree	*Bacillus cereus*
anaerobic/37 degree	*Bacillus cereus*
anaerobic/37 degree	*Bacillus cereus*
anaerobic/37 degree	*Bacillus cereus*
anaerobic/37 degree	*Bacillus cereus*
anaerobic/37 degree	*Bacillus cereus*
anaerobic/37 degree	*Bacillus cereus*
anaerobic/37 degree	*Bacillus cereus*

Table S5. The cultured strains and their culture atmospheres

**Atmosphere**	***species***
anaerobic/37 degree	*Bacillus cereus*
anaerobic/37 degree	*Bacillus cereus*
anaerobic/37 degree	*Bacillus cereus*
anaerobic/42 degree	*Bacillus cereus*
anaerobic/42 degree	*Bacillus cereus*
anaerobic/42 degree	*Bacillus cereus*
anaerobic/42 degree	*Bacillus cereus*
anaerobic/42 degree	*Bacillus cereus*
anaerobic/42 degree	*Bacillus cereus*
anaerobic/42 degree	*Bacillus cereus*
anaerobic/42 degree	*Bacillus cereus*
anaerobic/42 degree	*Bacillus cereus*
anaerobic/42 degree	*Bacillus cereus*
anaerobic/42 degree	*Bacillus cereus*
anaerobic/42 degree	*Bacillus cereus*
anaerobic/42 degree	*Bacillus cereus*
anaerobic/37 degree	*Bacillus coagulans*
anaerobic/37 degree	*Bacillus infantis*
anaerobic/37 degree	*Bacillus licheniformis*
anaerobic/37 degree	*Bacillus licheniformis*
anaerobic/37 degree	*Bacillus licheniformis*
anaerobic/37 degree	*Bacillus licheniformis*
anaerobic/37 degree	*Bacillus licheniformis*
anaerobic/37 degree	*Bacillus licheniformis*
anaerobic/37 degree	*Bacillus licheniformis*
anaerobic/37 degree	*Bacillus licheniformis*
anaerobic/42 degree	*Bacillus licheniformis*
anaerobic/37 degree	*Bacillus megaterium*
anaerobic/37 degree	*Bacillus mojavensis*
anaerobic/35 degree	*Bacillus pumilus*
anaerobic/35 degree	*Bacillus pumilus*
anaerobic/35 degree	*Bacillus pumilus*
anaerobic/35 degree	*Bacillus pumilus*
anaerobic/35 degree	*Bacillus pumilus*
anaerobic/37 degree	*Bacillus pumilus*
anaerobic/37 degree	*Bacillus pumilus*
anaerobic/37 degree	*Bacillus pumilus*
anaerobic/37 degree	*Bacillus pumilus*
anaerobic/37 degree	*Bacillus pumilus*
anaerobic/37 degree	*Bacillus pumilus*
anaerobic/37 degree	*Bacillus pumilus*
anaerobic/37 degree	*Bacillus pumilus*
anaerobic/37 degree	*Bacillus pumilus*
anaerobic/37 degree	*Bacillus pumilus*

Table S5. The cultured strains and their culture atmospheres

**Atmosphere**	***species***
anaerobic/37 degree	*Bacillus pumilus*
anaerobic/37 degree	*Bacillus pumilus*
anaerobic/37 degree	*Bacillus pumilus*
anaerobic/37 degree	*Bacillus pumilus*
anaerobic/37 degree	*Bacillus pumilus*
anaerobic/37 degree	*Bacillus pumilus*
anaerobic/37 degree	*Bacillus pumilus*
anaerobic/37 degree	*Bacillus pumilus*
anaerobic/37 degree	*Bacillus pumilus*
anaerobic/37 degree	*Bacillus pumilus*
anaerobic/37 degree	*Bacillus pumilus*
anaerobic/37 degree	*Bacillus pumilus*
anaerobic/37 degree	*Bacillus pumilus*
anaerobic/37 degree	*Bacillus pumilus*
anaerobic/37 degree	*Bacillus pumilus*
anaerobic/37 degree	*Bacillus pumilus*
anaerobic/37 degree	*Bacillus pumilus*
anaerobic/37 degree	*Bacillus pumilus*
anaerobic/37 degree	*Bacillus pumilus*
anaerobic/37 degree	*Bacillus pumilus*
anaerobic/37 degree	*Bacillus pumilus*
anaerobic/37 degree	*Bacillus pumilus*
anaerobic/37 degree	*Bacillus pumilus*
anaerobic/37 degree	*Bacillus pumilus*
anaerobic/37 degree	*Bacillus pumilus*
anaerobic/37 degree	*Bacillus pumilus*
anaerobic/37 degree	*Bacillus pumilus*
anaerobic/37 degree	*Bacillus pumilus*
anaerobic/37 degree	*Bacillus pumilus*
anaerobic/37 degree	*Bacillus pumilus*
anaerobic/37 degree	*Bacillus pumilus*
anaerobic/37 degree	*Bacillus pumilus*
anaerobic/37 degree	*Bacillus pumilus*
anaerobic/37 degree	*Bacillus pumilus*
anaerobic/37 degree	*Bacillus pumilus*
anaerobic/37 degree	*Bacillus pumilus*
anaerobic/37 degree	*Bacillus pumilus*
anaerobic/37 degree	*Bacillus pumilus*
anaerobic/37 degree	*Bacillus pumilus*
anaerobic/37 degree	*Bacillus pumilus*
anaerobic/37 degree	*Bacillus pumilus*
anaerobic/37 degree	*Bacillus pumilus*
anaerobic/37 degree	*Bacillus pumilus*
anaerobic/37 degree	*Bacillus pumilus*

Table S5. The cultured strains and their culture atmospheres

**Atmosphere**	***species***
anaerobic/37 degree	*Bacillus pumilus*
anaerobic/37 degree	*Bacillus pumilus*
anaerobic/37 degree	*Bacillus pumilus*
anaerobic/37 degree	*Bacillus pumilus*
anaerobic/37 degree	*Bacillus pumilus*
anaerobic/37 degree	*Bacillus pumilus*
anaerobic/37 degree	*Bacillus pumilus*
anaerobic/37 degree	*Bacillus pumilus*
anaerobic/37 degree	*Bacillus pumilus*
anaerobic/37 degree	*Bacillus pumilus*
anaerobic/37 degree	*Bacillus pumilus*
anaerobic/37 degree	*Bacillus pumilus*
anaerobic/37 degree	*Bacillus pumilus*
anaerobic/37 degree	*Bacillus pumilus*
anaerobic/37 degree	*Bacillus pumilus*
anaerobic/37 degree	*Bacillus pumilus*
anaerobic/37 degree	*Bacillus pumilus*
anaerobic/37 degree	*Bacillus pumilus*
anaerobic/37 degree	*Bacillus pumilus*
anaerobic/37 degree	*Bacillus pumilus*
anaerobic/37 degree	*Bacillus pumilus*
anaerobic/37 degree	*Bacillus pumilus*
anaerobic/37 degree	*Bacillus pumilus*
anaerobic/37 degree	*Bacillus pumilus*
anaerobic/37 degree	*Bacillus pumilus*
anaerobic/37 degree	*Bacillus pumilus*
anaerobic/37 degree	*Bacillus pumilus*
anaerobic/37 degree	*Bacillus pumilus*
anaerobic/37 degree	*Bacillus pumilus*
anaerobic/42 degree	*Bacillus pumilus*
anaerobic/42 degree	*Bacillus pumilus*
anaerobic/42 degree	*Bacillus pumilus*
anaerobic/42 degree	*Bacillus pumilus*
anaerobic/42 degree	*Bacillus pumilus*
anaerobic/42 degree	*Bacillus pumilus*
anaerobic/42 degree	*Bacillus pumilus*
anaerobic/42 degree	*Bacillus pumilus*
anaerobic/37 degree	*Bacillus safensis*
anaerobic/37 degree	*Bacillus safensis*
anaerobic/37 degree	*Bacillus safensis*
anaerobic/37 degree	*Bacillus safensis*
anaerobic/37 degree	*Bacillus safensis*
anaerobic/37 degree	*Bacillus safensis*
anaerobic/37 degree	*Bacillus safensis*

Table S5. The cultured strains and their culture atmospheres

Atmosphere	*species*
anaerobic/37 degree	*Bacillus safensis*
anaerobic/37 degree	*Bacillus safensis*
anaerobic/37 degree	*Bacillus safensis*
anaerobic/37 degree	*Bacillus safensis*
anaerobic/37 degree	*Bacillus safensis*
anaerobic/37 degree	*Bacillus safensis*
anaerobic/37 degree	*Bacillus safensis*
anaerobic/37 degree	*Bacillus safensis*
anaerobic/37 degree	*Bacillus safensis*
anaerobic/37 degree	*Bacillus safensis*
anaerobic/37 degree	*Bacillus safensis*
anaerobic/37 degree	*Bacillus safensis*
anaerobic/37 degree	*Bacillus safensis*
anaerobic/37 degree	*Bacillus safensis*
anaerobic/37 degree	*Bacillus safensis*
anaerobic/37 degree	*Bacillus safensis*
anaerobic/37 degree	*Bacillus safensis*
anaerobic/37 degree	*Bacillus safensis*
anaerobic/37 degree	*Bacillus safensis*
anaerobic/37 degree	*Bacillus safensis*
anaerobic/37 degree	*Bacillus safensis*
anaerobic/37 degree	*Bacillus safensis*
anaerobic/37 degree	*Bacillus safensis*
anaerobic/37 degree	*Bacillus safensis*
anaerobic/37 degree	*Bacillus safensis*
anaerobic/37 degree	*Bacillus safensis*
anaerobic/37 degree	*Bacillus safensis*
anaerobic/37 degree	*Bacillus safensis*
anaerobic/37 degree	*Bacillus safensis*
anaerobic/37 degree	*Bacillus safensis*
anaerobic/37 degree	*Bacillus safensis*
anaerobic/37 degree	*Bacillus safensis*
anaerobic/37 degree	*Bacillus safensis*
anaerobic/37 degree	*Bacillus safensis*
anaerobic/37 degree	*Bacillus safensis*
anaerobic/42 degree	*Bacillus safensis*
anaerobic/42 degree	*Bacillus safensis*
anaerobic/42 degree	*Bacillus safensis*
anaerobic/42 degree	*Bacillus safensis*
anaerobic/37 degree	*Bacillus sonorensis*
anaerobic/37 degree	*Bacillus sp*
anaerobic/37 degree	*Bacillus sp*
anaerobic/37 degree	*Bacillus sp*
anaerobic/37 degree	*Bacillus sp.*

Table S5. The cultured strains and their culture atmospheres

Atmosphere	species
anaerobic/42 degree	*Bacillus sp.*
anaerobic/42 degree	*Bacillus sp.*
anaerobic/42 degree	*Bacillus sp.*
anaerobic/37 degree	*Bacillus subtilis*
anaerobic/42 degree	*Bacillus subtilis*
anaerobic/37 degree	*Bacillus velezensis*
anaerobic/37 degree	*Bacillus xiamenensis*
anaerobic/37 degree	*Bacillus xiamenensis*
anaerobic/37 degree	*Bacteroides caccae*
anaerobic/37 degree	*Bacteroides caccae*
anaerobic/37 degree	*Bacteroides caccae*
anaerobic/37 degree	*Bacteroides caccae*
anaerobic/37 degree	*Bacteroides caccae*
anaerobic/37 degree	*Bacteroides caccae*
anaerobic/37 degree	*Bacteroides caccae*
anaerobic/37 degree	*Bacteroides caccae*
anaerobic/37 degree	*Bacteroides caccae*
anaerobic/37 degree	*Bacteroides caccae*
anaerobic/42 degree	*Bacteroides caccae*
anaerobic/42 degree	*Bacteroides caccae*
anaerobic/42 degree	*Bacteroides caccae*
anaerobic/42 degree	*Bacteroides caccae*
anaerobic/37 degree	*Bacteroides caccae CAG:21*
anaerobic/37 degree	*Bacteroides coagulans*
anaerobic/35 degree	*Bacteroides dorei*
anaerobic/37 degree	*Bacteroides dorei*
anaerobic/37 degree	*Bacteroides dorei*
anaerobic/42 degree	*Bacteroides dorei*
anaerobic/42 degree	*Bacteroides dorei*
anaerobic/42 degree	*Bacteroides dorei*
anaerobic/42 degree	*Bacteroides dorei*
anaerobic/42 degree	*Bacteroides dorei*
anaerobic/37 degree	*Bacteroides fluxus*
anaerobic/37 degree	*Bacteroides fragilis*
anaerobic/37 degree	*Bacteroides fragilis*
anaerobic/37 degree	*Bacteroides fragilis*
anaerobic/37 degree	*Bacteroides fragilis*
anaerobic/37 degree	*Bacteroides fragilis*
anaerobic/37 degree	*Bacteroides fragilis*
anaerobic/37 degree	*Bacteroides fragilis*
anaerobic/37 degree	*Bacteroides fragilis*
anaerobic/37 degree	*Bacteroides fragilis*
anaerobic/37 degree	*Bacteroides fragilis*
anaerobic/37 degree	*Bacteroides fragilis*

Table S5. The cultured strains and their culture atmospheres

**Atmosphere**	***species***
anaerobic/37 degree	*Bacteroides fragilis*
anaerobic/37 degree	*Bacteroides fragilis*
anaerobic/37 degree	*Bacteroides fragilis*
anaerobic/37 degree	*Bacteroides fragilis*
anaerobic/37 degree	*Bacteroides fragilis*
anaerobic/37 degree	*Bacteroides fragilis*
anaerobic/37 degree	*Bacteroides fragilis*
anaerobic/37 degree	*Bacteroides fragilis*
anaerobic/37 degree	*Bacteroides fragilis*
anaerobic/37 degree	*Bacteroides fragilis*
anaerobic/37 degree	*Bacteroides fragilis*
anaerobic/37 degree	*Bacteroides fragilis*
anaerobic/37 degree	*Bacteroides fragilis*
anaerobic/37 degree	*Bacteroides fragilis*
anaerobic/37 degree	*Bacteroides fragilis*
anaerobic/37 degree	*Bacteroides fragilis*
anaerobic/37 degree	*Bacteroides fragilis*
anaerobic/37 degree	*Bacteroides fragilis*
anaerobic/37 degree	*Bacteroides fragilis*
anaerobic/37 degree	*Bacteroides fragilis*
anaerobic/37 degree	*Bacteroides fragilis*
anaerobic/37 degree	*Bacteroides fragilis*
anaerobic/37 degree	*Bacteroides fragilis*
anaerobic/37 degree	*Bacteroides fragilis*
anaerobic/37 degree	*Bacteroides fragilis*
anaerobic/37 degree	*Bacteroides fragilis*
anaerobic/37 degree	*Bacteroides fragilis*
anaerobic/37 degree	*Bacteroides fragilis*
anaerobic/37 degree	*Bacteroides fragilis*
anaerobic/37 degree	*Bacteroides fragilis*
anaerobic/37 degree	*Bacteroides fragilis*
anaerobic/37 degree	*Bacteroides fragilis*
anaerobic/37 degree	*Bacteroides fragilis*
anaerobic/37 degree	*Bacteroides fragilis*
anaerobic/37 degree	*Bacteroides fragilis*
anaerobic/37 degree	*Bacteroides fragilis*
anaerobic/37 degree	*Bacteroides fragilis*
anaerobic/37 degree	*Bacteroides fragilis*
anaerobic/37 degree	*Bacteroides fragilis*
anaerobic/37 degree	*Bacteroides fragilis*
anaerobic/37 degree	*Bacteroides fragilis*
anaerobic/37 degree	*Bacteroides fragilis*
anaerobic/37 degree	*Bacteroides fragilis*
anaerobic/37 degree	*Bacteroides fragilis*

Table S5. The cultured strains and their culture atmospheres

**Atmosphere**	***species***
anaerobic/37 degree	*Bacteroides fragilis*
anaerobic/37 degree	*Bacteroides fragilis*
anaerobic/37 degree	*Bacteroides fragilis*
anaerobic/37 degree	*Bacteroides fragilis*
anaerobic/37 degree	*Bacteroides fragilis*
anaerobic/37 degree	*Bacteroides fragilis*
anaerobic/37 degree	*Bacteroides fragilis*
anaerobic/37 degree	*Bacteroides fragilis*
anaerobic/37 degree	*Bacteroides fragilis*
anaerobic/42 degree	*Bacteroides fragilis*
anaerobic/42 degree	*Bacteroides fragilis*
anaerobic/42 degree	*Bacteroides fragilis*
anaerobic/42 degree	*Bacteroides fragilis*
anaerobic/42 degree	*Bacteroides fragilis*
anaerobic/42 degree	*Bacteroides fragilis*
anaerobic/42 degree	*Bacteroides fragilis*
anaerobic/42 degree	*Bacteroides fragilis*
anaerobic/42 degree	*Bacteroides fragilis*
anaerobic/42 degree	*Bacteroides fragilis*
anaerobic/42 degree	*Bacteroides fragilis*
anaerobic/42 degree	*Bacteroides fragilis*
anaerobic/42 degree	*Bacteroides fragilis*
anaerobic/42 degree	*Bacteroides fragilis*
anaerobic/42 degree	*Bacteroides fragilis*
anaerobic/42 degree	*Bacteroides fragilis*
anaerobic/37 degree	*Bacteroides intestinalis*
anaerobic/37 degree	*Bacteroides intestinalis*
anaerobic/37 degree	*Bacteroides intestinalis*
anaerobic/37 degree	*Bacteroides intestinalis*
anaerobic/37 degree	*Bacteroides intestinalis*
anaerobic/37 degree	*Bacteroides intestinalis*
anaerobic/37 degree	*Bacteroides intestinalis*
anaerobic/37 degree	*Bacteroides nordii*
anaerobic/37 degree	*Bacteroides nordii*
anaerobic/37 degree	*Bacteroides nordii*
anaerobic/37 degree	*Bacteroides nordii*
anaerobic/37 degree	*Bacteroides nordii*
anaerobic/37 degree	*Bacteroides nordii*
anaerobic/37 degree	*Bacteroides nordii*
anaerobic/37 degree	*Bacteroides nordii*
anaerobic/37 degree	*Bacteroides nordii*
anaerobic/37 degree	*Bacteroides nordii*
anaerobic/37 degree	*Bacteroides nordii*
anaerobic/37 degree	*Bacteroides nordii*

Table S5. The cultured strains and their culture atmospheres

Atmosphere	*species*
anaerobic/37 degree	*Bacteroides nordii*
anaerobic/37 degree	*Bacteroides nordii*
anaerobic/37 degree	*Bacteroides nordii*
anaerobic/37 degree	*Bacteroides nordii*
anaerobic/37 degree	*Bacteroides nordii*
anaerobic/37 degree	*Bacteroides nordii*
anaerobic/37 degree	*Bacteroides nordii*
anaerobic/37 degree	*Bacteroides ovatus*
anaerobic/37 degree	*Bacteroides ovatus*
anaerobic/37 degree	*Bacteroides ovatus*
anaerobic/37 degree	*Bacteroides ovatus*
anaerobic/37 degree	*Bacteroides ovatus*
anaerobic/37 degree	*Bacteroides ovatus*
anaerobic/37 degree	*Bacteroides ovatus*
anaerobic/42 degree	*Bacteroides ovatus*
anaerobic/42 degree	*Bacteroides ovatus*
anaerobic/42 degree	*Bacteroides ovatus*
anaerobic/42 degree	*Bacteroides ovatus*
anaerobic/42 degree	*Bacteroides ovatus*
anaerobic/42 degree	*Bacteroides ovatus*
anaerobic/37 degree	*Bacteroides salyersiae*
anaerobic/37 degree	*Bacteroides salyersiae*
anaerobic/37 degree	*Bacteroides salyersiae*
anaerobic/37 degree	*Bacteroides stercoris*
anaerobic/37 degree	*Bacteroides stercoris*
anaerobic/37 degree	*Bacteroides stercoris*
anaerobic/37 degree	*Bacteroides stercoris*
anaerobic/37 degree	*Bacteroides stercoris*
anaerobic/37 degree	*Bacteroides stercoris*
anaerobic/37 degree	*Bacteroides stercoris*
anaerobic/37 degree	*Bacteroides stercoris*
anaerobic/37 degree	*Bacteroides stercoris*
anaerobic/37 degree	*Bacteroides thetaiotaomicron*
anaerobic/37 degree	*Bacteroides thetaiotaomicron*
anaerobic/37 degree	*Bacteroides thetaiotaomicron*
anaerobic/37 degree	*Bacteroides thetaiotaomicron*
anaerobic/37 degree	*Bacteroides thetaiotaomicron*
anaerobic/37 degree	*Bacteroides thetaiotaomicron*
anaerobic/37 degree	*Bacteroides thetaiotaomicron*
anaerobic/37 degree	*Bacteroides thetaiotaomicron*
anaerobic/37 degree	*Bacteroides thetaiotaomicron*
anaerobic/37 degree	*Bacteroides thetaiotaomicron*
anaerobic/37 degree	*Bacteroides thetaiotaomicron*
anaerobic/37 degree	*Bacteroides thetaiotaomicron*

Table S5. The cultured strains and their culture atmospheres

**Atmosphere**	***species***
anaerobic/37 degree	*Bacteroides thetaiotaomicron*
anaerobic/37 degree	*Bacteroides uniformis*
anaerobic/37 degree	*Bacteroides uniformis*
anaerobic/37 degree	*Bacteroides uniformis*
anaerobic/37 degree	*Bacteroides uniformis*
anaerobic/37 degree	*Bacteroides uniformis*
anaerobic/37 degree	*Bacteroides uniformis*
anaerobic/37 degree	*Bacteroides uniformis*
anaerobic/37 degree	*Bacteroides uniformis*
anaerobic/37 degree	*Bacteroides uniformis*
anaerobic/37 degree	*Bacteroides uniformis*
anaerobic/37 degree	*Bacteroides uniformis*
anaerobic/37 degree	*Bacteroides uniformis*
anaerobic/37 degree	*Bacteroides uniformis*
anaerobic/37 degree	*Bacteroides uniformis*
anaerobic/37 degree	*Bacteroides uniformis*
anaerobic/37 degree	*Bacteroides uniformis*
anaerobic/37 degree	*Bacteroides uniformis*
anaerobic/37 degree	*Bacteroides uniformis*
anaerobic/37 degree	*Bacteroides uniformis*
anaerobic/37 degree	*Bacteroides uniformis*
anaerobic/37 degree	*Bacteroides uniformis*
anaerobic/37 degree	*Bacteroides uniformis*
anaerobic/37 degree	*Bacteroides uniformis*
anaerobic/37 degree	*Bacteroides uniformis*
anaerobic/37 degree	*Bacteroides uniformis*
anaerobic/37 degree	*Bacteroides uniformis*
anaerobic/37 degree	*Bacteroides uniformis*
anaerobic/37 degree	*Bacteroides uniformis*
anaerobic/37 degree	*Bacteroides uniformis*
anaerobic/37 degree	*Bacteroides uniformis*
anaerobic/37 degree	*Bacteroides uniformis*
anaerobic/37 degree	*Bacteroides uniformis*
anaerobic/37 degree	*Bacteroides uniformis*
anaerobic/37 degree	*Bacteroides uniformis*
anaerobic/37 degree	*Bacteroides uniformis*
anaerobic/37 degree	*Bacteroides uniformis*
anaerobic/37 degree	*Bacteroides uniformis*
anaerobic/37 degree	*Bacteroides uniformis*
anaerobic/37 degree	*Bacteroides uniformis*
anaerobic/37 degree	*Bacteroides uniformis*
anaerobic/37 degree	*Bacteroides uniformis*
anaerobic/37 degree	*Bacteroides uniformis*
anaerobic/37 degree	*Bacteroides uniformis*

Table S5. The cultured strains and their culture atmospheres

**Atmosphere**	***species***
anaerobic/37 degree	*Bacteroides uniformis*
anaerobic/37 degree	*Bacteroides uniformis*
anaerobic/37 degree	*Bacteroides uniformis*
anaerobic/37 degree	*Bacteroides uniformis*
anaerobic/37 degree	*Bacteroides uniformis*
anaerobic/37 degree	*Bacteroides uniformis*
anaerobic/37 degree	*Bacteroides uniformis*
anaerobic/37 degree	*Bacteroides uniformis*
anaerobic/37 degree	*Bacteroides uniformis*
anaerobic/37 degree	*Bacteroides uniformis*
anaerobic/37 degree	*Bacteroides uniformis*
anaerobic/37 degree	*Bacteroides uniformis*
anaerobic/37 degree	*Bacteroides uniformis*
anaerobic/37 degree	*Bacteroides uniformis*
anaerobic/42 degree	*Bacteroides uniformis*
anaerobic/42 degree	*Bacteroides uniformis*
anaerobic/42 degree	*Bacteroides uniformis*
anaerobic/42 degree	*Bacteroides uniformis*
anaerobic/42 degree	*Bacteroides uniformis*
anaerobic/37 degree	*Bacteroides vulgatus*
anaerobic/37 degree	*Bacteroides vulgatus*
anaerobic/37 degree	*Bacteroides vulgatus*
anaerobic/37 degree	*Bacteroides vulgatus*
anaerobic/37 degree	*Bacteroides vulgatus*
anaerobic/37 degree	*Bacteroides vulgatus*
anaerobic/37 degree	*Bacteroides vulgatus*
anaerobic/37 degree	*Bacteroides vulgatus*
anaerobic/37 degree	*Bacteroides vulgatus*
anaerobic/37 degree	*Bacteroides vulgatus*
anaerobic/37 degree	*Bacteroides vulgatus*
anaerobic/37 degree	*Bacteroides vulgatus*
anaerobic/37 degree	*Bacteroides vulgatus*
anaerobic/37 degree	*Bacteroides vulgatus*
anaerobic/37 degree	*Bacteroides vulgatus*
anaerobic/37 degree	*Bacteroides vulgatus*
anaerobic/37 degree	*Bacteroides vulgatus*
anaerobic/37 degree	*Bacteroides vulgatus*
anaerobic/37 degree	*Bacteroides vulgatus*
anaerobic/37 degree	*Bacteroides vulgatus*
anaerobic/37 degree	*Bacteroides vulgatus*
anaerobic/37 degree	*Bacteroides vulgatus*
anaerobic/37 degree	*Bacteroides vulgatus*
anaerobic/37 degree	*Bacteroides vulgatus*
anaerobic/37 degree	*Bacteroides vulgatus*

Table S5. The cultured strains and their culture atmospheres

Atmosphere	*species*
anaerobic/37 degree	*Bacteroides vulgatus*
anaerobic/37 degree	*Bacteroides vulgatus*
anaerobic/37 degree	*Bacteroides vulgatus*
anaerobic/37 degree	*Bacteroides vulgatus*
anaerobic/37 degree	*Bacteroides vulgatus*
anaerobic/37 degree	*Bacteroides vulgatus*
anaerobic/37 degree	*Bacteroides vulgatus*
anaerobic/37 degree	*Bacteroides vulgatus*
anaerobic/37 degree	*Bacteroides vulgatus*
anaerobic/37 degree	*Bacteroides vulgatus*
anaerobic/37 degree	*Bacteroides vulgatus*
anaerobic/37 degree	*Bacteroides vulgatus*
anaerobic/37 degree	*Bacteroides vulgatus*
anaerobic/37 degree	*Bacteroides vulgatus*
anaerobic/37 degree	*Bacteroides vulgatus*
anaerobic/37 degree	*Bacteroides vulgatus*
anaerobic/42 degree	*Bacteroides vulgatus*
anaerobic/37 degree	*Bacteroides xylanisolvens*
anaerobic/37 degree	*Bacteroides xylanisolvens*
anaerobic/37 degree	*Bacteroides xylanisolvens*
anaerobic/37 degree	*Bacteroides xylanisolvens*
anaerobic/37 degree	*Bacteroides xylanisolvens*
anaerobic/37 degree	*Bacteroides xylanisolvens*
anaerobic/37 degree	*Bacteroides xylanisolvens*
anaerobic/37 degree	*Bifidobacterium adolescentis*
anaerobic/37 degree	*Bifidobacterium animalis*
anaerobic/37 degree	*Bifidobacterium bifidum*
anaerobic/37 degree	*Bifidobacterium bifidum*
anaerobic/37 degree	*Bifidobacterium bifidum*
anaerobic/37 degree	*Bifidobacterium breve*
anaerobic/37 degree	*Bifidobacterium breve*
anaerobic/40-50 degree	*Bifidobacterium breve*
anaerobic/40-50 degree	*Bifidobacterium breve*
anaerobic/37 degree	*Bifidobacterium catenulatum*
anaerobic/40-50 degree	*Bifidobacterium catenulatum*
anaerobic/37 degree	*Bifidobacterium dentium*
anaerobic/37 degree	*Bifidobacterium dentium*
anaerobic/37 degree	*Bifidobacterium dentium*
anaerobic/37 degree	*Bifidobacterium dentium*
anaerobic/37 degree	*Bifidobacterium dentium*
anaerobic/37 degree	*Bifidobacterium dentium*
anaerobic/37 degree	*Bifidobacterium dentium*
anaerobic/42 degree	*Bifidobacterium dentium*
anaerobic/42 degree	*Bifidobacterium dentium*

Table S5. The cultured strains and their culture atmospheres

Atmosphere	*species*
anaerobic/42 degree	*Bifidobacterium dentium*
anaerobic/42 degree	*Bifidobacterium dentium*
anaerobic/42 degree	*Bifidobacterium dentium*
anaerobic/42 degree	*Bifidobacterium dentium*
anaerobic/42 degree	*Bifidobacterium dentium*
anaerobic/37 degree	*Bifidobacterium longum*
anaerobic/37 degree	*Bifidobacterium longum*
anaerobic/37 degree	*Bifidobacterium longum*
anaerobic/37 degree	*Bifidobacterium longum*
anaerobic/37 degree	*Bifidobacterium longum*
anaerobic/37 degree	*Bifidobacterium longum*
anaerobic/37 degree	*Bifidobacterium longum*
anaerobic/37 degree	*Bifidobacterium longum*
anaerobic/37 degree	*Bifidobacterium longum*
anaerobic/37 degree	*Bifidobacterium longum*
anaerobic/37 degree	*Bifidobacterium longum*
anaerobic/37 degree	*Bifidobacterium longum*
anaerobic/37 degree	*Bifidobacterium longum*
anaerobic/37 degree	*Bifidobacterium longum*
anaerobic/37 degree	*Bifidobacterium longum*
anaerobic/37 degree	*Bifidobacterium longum*
anaerobic/37 degree	*Bifidobacterium longum*
anaerobic/37 degree	*Bifidobacterium longum*
anaerobic/40-50 degree	*Bifidobacterium longum*
anaerobic/40-50 degree	*Bifidobacterium longum*
anaerobic/40-50 degree	*Bifidobacterium longum*
anaerobic/40-50 degree	*Bifidobacterium longum*
anaerobic/40-50 degree	*Bifidobacterium longum*
anaerobic/42 degree	*Bifidobacterium longum*
anaerobic/37 degree	*Bifidobacterium pseudocatenulatum*
anaerobic/37 degree	*Bifidobacterium pseudocatenulatum*
anaerobic/37 degree	*Bifidobacterium pseudocatenulatum*
anaerobic/37 degree	*Bifidobacterium pseudocatenulatum*
anaerobic/37 degree	*Bifidobacterium pseudocatenulatum*
anaerobic/37 degree	*Bifidobacterium pseudocatenulatum*
anaerobic/37 degree	*Bifidobacterium pseudolongum*
anaerobic/37 degree	*Bifidobacterium pseudolongum*
anaerobic/37 degree	*Bifidobacterium pseudolongum*
anaerobic/37 degree	*Bifidobacterium pseudolongum*
anaerobic/37 degree	*Bifidobacterium pseudolongum*
anaerobic/37 degree	*Bifidobacterium pseudolongum*
anaerobic/37 degree	*Bifidobacterium pseudolongum*
anaerobic/37 degree	*Bifidobacterium pseudolongum*
anaerobic/37 degree	*Bifidobacterium pseudolongum*

Table S5. The cultured strains and their culture atmospheres

Atmosphere	*species*
anaerobic/37 degree	*Bifidobacterium pseudolongum*
anaerobic/37 degree	*Bifidobacterium pseudolongum*
anaerobic/37 degree	*Bifidobacterium pseudolongum*
anaerobic/37 degree	*Bifidobacterium pseudolongum*
anaerobic/37 degree	*Bifidobacterium pseudolongum*
anaerobic/37 degree	*Bifidobacterium pseudolongum*
anaerobic/37 degree	*Bifidobacterium pseudolongum*
anaerobic/37 degree	*Bifidobacterium pseudolongum*
anaerobic/37 degree	*Bifidobacterium pseudolongum*
anaerobic/37 degree	*Bifidobacterium pseudolongum*
anaerobic/37 degree	*Bifidobacterium pseudolongum*
anaerobic/37 degree	*Bifidobacterium pseudolongum*
anaerobic/37 degree	*Bifidobacterium pseudolongum*
anaerobic/37 degree	*Bifidobacterium pseudolongum*
anaerobic/37 degree	*Bifidobacterium pseudolongum*
anaerobic/37 degree	*Bifidobacterium pseudolongum*
anaerobic/37 degree	*Bifidobacterium pseudolongum*
anaerobic/37 degree	*Bifidobacterium pseudolongum*
anaerobic/37 degree	*Bifidobacterium pseudolongum*
anaerobic/37 degree	*Bifidobacterium pseudolongum*
anaerobic/37 degree	*Bifidobacterium pseudolongum*
anaerobic/37 degree	*Bifidobacterium pseudolongum*
anaerobic/37 degree	*Bifidobacterium pseudolongum*
anaerobic/37 degree	*Bifidobacterium pseudolongum*
anaerobic/37 degree	*Bifidobacterium pseudolongum*
anaerobic/37 degree	*Bifidobacterium pseudolongum*
anaerobic/37 degree	*Bifidobacterium pseudolongum*
anaerobic/37 degree	*Campylobacter ureolyticus*
anaerobic/37 degree	*Citrobacter braakii*
anaerobic/37 degree	*Citrobacter braakii*
anaerobic/42 degree	*Citrobacter braakii*
anaerobic/42 degree	*Citrobacter braakii*
anaerobic/42 degree	*Citrobacter braakii*
anaerobic/37 degree	*Citrobacter freundii*
anaerobic/37 degree	*Citrobacter freundii*
anaerobic/37 degree	*Citrobacter freundii*
anaerobic/37 degree	*Citrobacter freundii*
anaerobic/37 degree	*Citrobacter freundii*
anaerobic/37 degree	*Citrobacter freundii*
anaerobic/37 degree	*Citrobacter freundii*
anaerobic/37 degree	*Citrobacter freundii*
anaerobic/37 degree	*Citrobacter freundii*
anaerobic/37 degree	*Citrobacter freundii*
anaerobic/37 degree	*Citrobacter freundii*

Table S5. The cultured strains and their culture atmospheres

Atmosphere	*species*
anaerobic/37 degree	*Citrobacter freundii*
anaerobic/37 degree	*Citrobacter freundii*
anaerobic/37 degree	*Citrobacter freundii*
anaerobic/37 degree	*Citrobacter freundii*
anaerobic/37 degree	*Citrobacter freundii*
anaerobic/37 degree	*Citrobacter freundii*
anaerobic/37 degree	*Citrobacter freundii*
anaerobic/37 degree	*Citrobacter freundii*
anaerobic/37 degree	*Citrobacter freundii*
anaerobic/37 degree	*Citrobacter freundii*
anaerobic/37 degree	*Citrobacter freundii*
anaerobic/37 degree	*Citrobacter freundii*
anaerobic/37 degree	*Citrobacter freundii*
anaerobic/37 degree	*Citrobacter freundii*
anaerobic/37 degree	*Citrobacter freundii*
anaerobic/37 degree	*Citrobacter freundii*
anaerobic/37 degree	*Citrobacter freundii*
anaerobic/37 degree	*Citrobacter freundii*
anaerobic/37 degree	*Citrobacter freundii*
anaerobic/37 degree	*Citrobacter freundii*
anaerobic/42 degree	*Citrobacter freundii*
anaerobic/42 degree	*Citrobacter freundii*
anaerobic/35 degree	*Citrobacter koseri*
anaerobic/37 degree	*Citrobacter koseri*
anaerobic/37 degree	*Citrobacter koseri*
anaerobic/37 degree	*Citrobacter koseri*
anaerobic/37 degree	*Citrobacter koseri*
anaerobic/37 degree	*Citrobacter koseri*
anaerobic/37 degree	*Citrobacter koseri*
anaerobic/37 degree	*Citrobacter koseri*
anaerobic/37 degree	*Citrobacter koseri*
anaerobic/37 degree	*Citrobacter koseri*
anaerobic/37 degree	*Citrobacter koseri*
anaerobic/37 degree	*Citrobacter koseri*
anaerobic/37 degree	*Citrobacter koseri*
anaerobic/37 degree	*Citrobacter koseri*
anaerobic/37 degree	*Citrobacter koseri*
anaerobic/37 degree	*Citrobacter koseri*
anaerobic/37 degree	*Citrobacter koseri*
anaerobic/37 degree	*Citrobacter koseri*
anaerobic/37 degree	*Citrobacter koseri*
anaerobic/37 degree	*Citrobacter koseri*
anaerobic/37 degree	*Citrobacter koseri*
anaerobic/37 degree	*Citrobacter koseri*
anaerobic/37 degree	*Citrobacter koseri*

Table S5. The cultured strains and their culture atmospheres

**Atmosphere**	***species***
anaerobic/37 degree	*Citrobacter koseri*
anaerobic/42 degree	*Citrobacter koseri*
anaerobic/42 degree	*Citrobacter werkmanii*
anaerobic/37 degree	*Clostridium baratii*
anaerobic/37 degree	*Clostridium baratii*
anaerobic/37 degree	*Clostridium baratii*
anaerobic/37 degree	*Clostridium baratii*
anaerobic/37 degree	*Clostridium baratii*
anaerobic/37 degree	*Clostridium baratii*
anaerobic/42 degree	*Clostridium baratii*
anaerobic/42 degree	*Clostridium baratii*
anaerobic/42 degree	*Clostridium baratii*
anaerobic/42 degree	*Clostridium baratii*
anaerobic/35 degree	*Clostridium bifermentans*
anaerobic/35 degree	*Clostridium bifermentans*
anaerobic/35 degree	*Clostridium bifermentans*
anaerobic/35 degree	*Clostridium bifermentans*
anaerobic/35 degree	*Clostridium bifermentans*
anaerobic/35 degree	*Clostridium bifermentans*
anaerobic/37 degree	*Clostridium bifermentans*
anaerobic/37 degree	*Clostridium bifermentans*
anaerobic/37 degree	*Clostridium bifermentans*
anaerobic/37 degree	*Clostridium bifermentans*
anaerobic/37 degree	*Clostridium bifermentans*
anaerobic/37 degree	*Clostridium bifermentans*
anaerobic/37 degree	*Clostridium bifermentans*
anaerobic/37 degree	*Clostridium bifermentans*
anaerobic/37 degree	*Clostridium bifermentans*
anaerobic/37 degree	*Clostridium bifermentans*
anaerobic/37 degree	*Clostridium bifermentans*
anaerobic/37 degree	*Clostridium bifermentans*
anaerobic/37 degree	*Clostridium bifermentans*
anaerobic/37 degree	*Clostridium bifermentans*
anaerobic/37 degree	*Clostridium bifermentans*
anaerobic/37 degree	*Clostridium bifermentans*
anaerobic/37 degree	*Clostridium bifermentans*
anaerobic/37 degree	*Clostridium bifermentans*
anaerobic/37 degree	*Clostridium bifermentans*
anaerobic/37 degree	*Clostridium bifermentans*
anaerobic/37 degree	*Clostridium bifermentans*
anaerobic/37 degree	*Clostridium bifermentans*
anaerobic/37 degree	*Clostridium bifermentans*
anaerobic/37 degree	*Clostridium bifermentans*
anaerobic/37 degree	*Clostridium bifermentans*

Table S5. The cultured strains and their culture atmospheres

**Atmosphere**	***species***
anaerobic/37 degree	*Clostridium bifermentans*
anaerobic/37 degree	*Clostridium bifermentans*
anaerobic/37 degree	*Clostridium bifermentans*
anaerobic/37 degree	*Clostridium bifermentans*
anaerobic/37 degree	*Clostridium bifermentans*
anaerobic/37 degree	*Clostridium bifermentans*
anaerobic/37 degree	*Clostridium bifermentans*
anaerobic/37 degree	*Clostridium bifermentans*
anaerobic/37 degree	*Clostridium bifermentans*
anaerobic/37 degree	*Clostridium bifermentans*
anaerobic/37 degree	*Clostridium bifermentans*
anaerobic/37 degree	*Clostridium bifermentans*
anaerobic/37 degree	*Clostridium bifermentans*
anaerobic/37 degree	*Clostridium bifermentans*
anaerobic/37 degree	*Clostridium bifermentans*
anaerobic/37 degree	*Clostridium bifermentans*
anaerobic/37 degree	*Clostridium bifermentans*
anaerobic/37 degree	*Clostridium bifermentans*
anaerobic/37 degree	*Clostridium bifermentans*
anaerobic/37 degree	*Clostridium bifermentans*
anaerobic/37 degree	*Clostridium bifermentans*
anaerobic/37 degree	*Clostridium bifermentans*
anaerobic/37 degree	*Clostridium bifermentans*
anaerobic/37 degree	*Clostridium bifermentans*
anaerobic/37 degree	*Clostridium bifermentans*
anaerobic/37 degree	*Clostridium bifermentans*
anaerobic/37 degree	*Clostridium bifermentans*
anaerobic/37 degree	*Clostridium bifermentans*
anaerobic/37 degree	*Clostridium bifermentans*
anaerobic/37 degree	*Clostridium bifermentans*
anaerobic/37 degree	*Clostridium bifermentans*
anaerobic/37 degree	*Clostridium bifermentans*
anaerobic/37 degree	*Clostridium bifermentans*
anaerobic/37 degree	*Clostridium bifermentans*
anaerobic/37 degree	*Clostridium bifermentans*
anaerobic/37 degree	*Clostridium bifermentans*
anaerobic/37 degree	*Clostridium bifermentans*
anaerobic/37 degree	*Clostridium bifermentans*
anaerobic/37 degree	*Clostridium bifermentans*
anaerobic/37 degree	*Clostridium bifermentans*
anaerobic/37 degree	*Clostridium bifermentans*
anaerobic/37 degree	*Clostridium bifermentans*
anaerobic/37 degree	*Clostridium bifermentans*
anaerobic/37 degree	*Clostridium bifermentans*

Table S5. The cultured strains and their culture atmospheres

**Atmosphere**	***species***
anaerobic/37 degree	*Clostridium bifermentans*
anaerobic/37 degree	*Clostridium bifermentans*
anaerobic/37 degree	*Clostridium bifermentans*
anaerobic/37 degree	*Clostridium bifermentans*
anaerobic/37 degree	*Clostridium bifermentans*
anaerobic/37 degree	*Clostridium bifermentans*
anaerobic/37 degree	*Clostridium bifermentans*
anaerobic/37 degree	*Clostridium bifermentans*
anaerobic/37 degree	*Clostridium bifermentans*
anaerobic/37 degree	*Clostridium bifermentans*
anaerobic/37 degree	*Clostridium bifermentans*
anaerobic/37 degree	*Clostridium bifermentans*
anaerobic/37 degree	*Clostridium bifermentans*
anaerobic/37 degree	*Clostridium bifermentans*
anaerobic/37 degree	*Clostridium bifermentans*
anaerobic/37 degree	*Clostridium bifermentans*
anaerobic/37 degree	*Clostridium bifermentans*
anaerobic/37 degree	*Clostridium bifermentans*
anaerobic/37 degree	*Clostridium bifermentans*
anaerobic/37 degree	*Clostridium bifermentans*
anaerobic/37 degree	*Clostridium bifermentans*
anaerobic/37 degree	*Clostridium bifermentans*
anaerobic/37 degree	*Clostridium bifermentans*
anaerobic/37 degree	*Clostridium bifermentans*
anaerobic/37 degree	*Clostridium bifermentans*
anaerobic/37 degree	*Clostridium bifermentans*
anaerobic/37 degree	*Clostridium bifermentans*
anaerobic/37 degree	*Clostridium bifermentans*
anaerobic/37 degree	*Clostridium bifermentans*
anaerobic/37 degree	*Clostridium bifermentans*
anaerobic/37 degree	*Clostridium bifermentans*
anaerobic/37 degree	*Clostridium bifermentans*
anaerobic/37 degree	*Clostridium bifermentans*
anaerobic/37 degree	*Clostridium bifermentans*
anaerobic/37 degree	*Clostridium bifermentans*
anaerobic/37 degree	*Clostridium bifermentans*
anaerobic/37 degree	*Clostridium bifermentans*
anaerobic/37 degree	*Clostridium bifermentans*
anaerobic/37 degree	*Clostridium bifermentans*
anaerobic/37 degree	*Clostridium bifermentans*
anaerobic/37 degree	*Clostridium bifermentans*
anaerobic/37 degree	*Clostridium bifermentans*
anaerobic/37 degree	*Clostridium bifermentans*
anaerobic/37 degree	*Clostridium bifermentans*

Table S5. The cultured strains and their culture atmospheres

**Atmosphere**	***species***
anaerobic/37 degree	*Clostridium bifermentans*
anaerobic/37 degree	*Clostridium bifermentans*
anaerobic/37 degree	*Clostridium bifermentans*
anaerobic/37 degree	*Clostridium bifermentans*
anaerobic/37 degree	*Clostridium bifermentans*
anaerobic/37 degree	*Clostridium bifermentans*
anaerobic/37 degree	*Clostridium bifermentans*
anaerobic/37 degree	*Clostridium bifermentans*
anaerobic/37 degree	*Clostridium bifermentans*
anaerobic/37 degree	*Clostridium bifermentans*
anaerobic/37 degree	*Clostridium bifermentans*
anaerobic/37 degree	*Clostridium bifermentans*
anaerobic/37 degree	*Clostridium bifermentans*
anaerobic/37 degree	*Clostridium bifermentans*
anaerobic/37 degree	*Clostridium bifermentans*
anaerobic/37 degree	*Clostridium bifermentans*
anaerobic/37 degree	*Clostridium bifermentans*
anaerobic/37 degree	*Clostridium bifermentans*
anaerobic/37 degree	*Clostridium bifermentans*
anaerobic/37 degree	*Clostridium bifermentans*
anaerobic/37 degree	*Clostridium bifermentans*
anaerobic/37 degree	*Clostridium bifermentans*
anaerobic/37 degree	*Clostridium bifermentans*
anaerobic/37 degree	*Clostridium bifermentans*
anaerobic/37 degree	*Clostridium bifermentans*
anaerobic/37 degree	*Clostridium bifermentans*
anaerobic/37 degree	*Clostridium bifermentans*
anaerobic/37 degree	*Clostridium bifermentans*
anaerobic/37 degree	*Clostridium bifermentans*
anaerobic/37 degree	*Clostridium bifermentans*
anaerobic/37 degree	*Clostridium bifermentans*
anaerobic/37 degree	*Clostridium bifermentans*
anaerobic/37 degree	*Clostridium bifermentans*
anaerobic/37 degree	*Clostridium bifermentans*
anaerobic/37 degree	*Clostridium bifermentans*
anaerobic/37 degree	*Clostridium bifermentans*
anaerobic/37 degree	*Clostridium bifermentans*
anaerobic/37 degree	*Clostridium bifermentans*
anaerobic/37 degree	*Clostridium bifermentans*
anaerobic/37 degree	*Clostridium bifermentans*
anaerobic/37 degree	*Clostridium bifermentans*
anaerobic/37 degree	*Clostridium bifermentans*
anaerobic/37 degree	*Clostridium bifermentans*
anaerobic/37 degree	*Clostridium bifermentans*

Table S5. The cultured strains and their culture atmospheres

**Atmosphere**	***species***
anaerobic/37 degree	*Clostridium bifermentans*
anaerobic/37 degree	*Clostridium bifermentans*
anaerobic/37 degree	*Clostridium bifermentans*
anaerobic/37 degree	*Clostridium bifermentans*
anaerobic/37 degree	*Clostridium bifermentans*
anaerobic/37 degree	*Clostridium bifermentans*
anaerobic/37 degree	*Clostridium bifermentans*
anaerobic/37 degree	*Clostridium bifermentans*
anaerobic/37 degree	*Clostridium bifermentans*
anaerobic/37 degree	*Clostridium bifermentans*
anaerobic/37 degree	*Clostridium bifermentans*
anaerobic/37 degree	*Clostridium bifermentans*
anaerobic/37 degree	*Clostridium bifermentans*
anaerobic/37 degree	*Clostridium bifermentans*
anaerobic/37 degree	*Clostridium bifermentans*
anaerobic/37 degree	*Clostridium bifermentans*
anaerobic/37 degree	*Clostridium bifermentans*
anaerobic/37 degree	*Clostridium bifermentans*
anaerobic/37 degree	*Clostridium bifermentans*
anaerobic/37 degree	*Clostridium bifermentans*
anaerobic/37 degree	*Clostridium bifermentans*
anaerobic/37 degree	*Clostridium bifermentans*
anaerobic/37 degree	*Clostridium bifermentans*
anaerobic/37 degree	*Clostridium bifermentans*
anaerobic/37 degree	*Clostridium bifermentans*
anaerobic/37 degree	*Clostridium bifermentans*
anaerobic/37 degree	*Clostridium bifermentans*
anaerobic/37 degree	*Clostridium bifermentans*
anaerobic/37 degree	*Clostridium bifermentans*
anaerobic/37 degree	*Clostridium bifermentans*
anaerobic/37 degree	*Clostridium bifermentans*
anaerobic/37 degree	*Clostridium bifermentans*
anaerobic/37 degree	*Clostridium bifermentans*
anaerobic/37 degree	*Clostridium bifermentans*
anaerobic/37 degree	*Clostridium bifermentans*
anaerobic/37 degree	*Clostridium bifermentans*
anaerobic/37 degree	*Clostridium bifermentans*
anaerobic/37 degree	*Clostridium bifermentans*
anaerobic/37 degree	*Clostridium bifermentans*
anaerobic/37 degree	*Clostridium bifermentans*
anaerobic/37 degree	*Clostridium bifermentans*
anaerobic/37 degree	*Clostridium bifermentans*
anaerobic/37 degree	*Clostridium bifermentans*
anaerobic/37 degree	*Clostridium bifermentans*

Table S5. The cultured strains and their culture atmospheres

**Atmosphere**	***species***
anaerobic/37 degree	*Clostridium bifermentans*
anaerobic/37 degree	*Clostridium bifermentans*
anaerobic/37 degree	*Clostridium bifermentans*
anaerobic/37 degree	*Clostridium bifermentans*
anaerobic/37 degree	*Clostridium bifermentans*
anaerobic/37 degree	*Clostridium bifermentans*
anaerobic/37 degree	*Clostridium bifermentans*
anaerobic/37 degree	*Clostridium bifermentans*
anaerobic/37 degree	*Clostridium bifermentans*
anaerobic/37 degree	*Clostridium bifermentans*
anaerobic/37 degree	*Clostridium bifermentans*
anaerobic/37 degree	*Clostridium bifermentans*
anaerobic/37 degree	*Clostridium bifermentans*
anaerobic/37 degree	*Clostridium bifermentans*
anaerobic/37 degree	*Clostridium bifermentans*
anaerobic/37 degree	*Clostridium bifermentans*
anaerobic/37 degree	*Clostridium bifermentans*
anaerobic/37 degree	*Clostridium bifermentans*
anaerobic/37 degree	*Clostridium bifermentans*
anaerobic/37 degree	*Clostridium bifermentans*
anaerobic/37 degree	*Clostridium bifermentans*
anaerobic/37 degree	*Clostridium bifermentans*
anaerobic/37 degree	*Clostridium bifermentans*
anaerobic/37 degree	*Clostridium bifermentans*
anaerobic/37 degree	*Clostridium bifermentans*
anaerobic/37 degree	*Clostridium bifermentans*
anaerobic/37 degree	*Clostridium bifermentans*
anaerobic/37 degree	*Clostridium bifermentans*
anaerobic/37 degree	*Clostridium bifermentans*
anaerobic/37 degree	*Clostridium bifermentans*
anaerobic/37 degree	*Clostridium bifermentans*
anaerobic/37 degree	*Clostridium bifermentans*
anaerobic/37 degree	*Clostridium bifermentans*
anaerobic/37 degree	*Clostridium bifermentans*
anaerobic/37 degree	*Clostridium bifermentans*
anaerobic/37 degree	*Clostridium bifermentans*
anaerobic/37 degree	*Clostridium bifermentans*
anaerobic/37 degree	*Clostridium bifermentans*
anaerobic/37 degree	*Clostridium bifermentans*
anaerobic/37 degree	*Clostridium bifermentans*
anaerobic/37 degree	*Clostridium bifermentans*
anaerobic/37 degree	*Clostridium bifermentans*
anaerobic/37 degree	*Clostridium bifermentans*
anaerobic/37 degree	*Clostridium bifermentans*

Table S5. The cultured strains and their culture atmospheres

**Atmosphere**	***species***
anaerobic/37 degree	*Clostridium bifermentans*
anaerobic/37 degree	*Clostridium bifermentans*
anaerobic/37 degree	*Clostridium bifermentans*
anaerobic/37 degree	*Clostridium bifermentans*
anaerobic/37 degree	*Clostridium bifermentans*
anaerobic/37 degree	*Clostridium bifermentans*
anaerobic/37 degree	*Clostridium bifermentans*
anaerobic/37 degree	*Clostridium bifermentans*
anaerobic/37 degree	*Clostridium bifermentans*
anaerobic/37 degree	*Clostridium bifermentans*
anaerobic/37 degree	*Clostridium bifermentans*
anaerobic/37 degree	*Clostridium bifermentans*
anaerobic/37 degree	*Clostridium bifermentans*
anaerobic/37 degree	*Clostridium bifermentans*
anaerobic/37 degree	*Clostridium bifermentans*
anaerobic/37 degree	*Clostridium bifermentans*
anaerobic/37 degree	*Clostridium bifermentans*
anaerobic/37 degree	*Clostridium bifermentans*
anaerobic/37 degree	*Clostridium bifermentans*
anaerobic/37 degree	*Clostridium bifermentans*
anaerobic/37 degree	*Clostridium bifermentans*
anaerobic/37 degree	*Clostridium bifermentans*
anaerobic/37 degree	*Clostridium bifermentans*
anaerobic/37 degree	*Clostridium bifermentans*
anaerobic/37 degree	*Clostridium bifermentans*
anaerobic/37 degree	*Clostridium bifermentans*
anaerobic/37 degree	*Clostridium bifermentans*
anaerobic/37 degree	*Clostridium bifermentans*
anaerobic/37 degree	*Clostridium bifermentans*
anaerobic/37 degree	*Clostridium bifermentans*
anaerobic/37 degree	*Clostridium bifermentans*
anaerobic/37 degree	*Clostridium bifermentans*
anaerobic/37 degree	*Clostridium bifermentans*
anaerobic/37 degree	*Clostridium bifermentans*
anaerobic/37 degree	*Clostridium bifermentans*
anaerobic/37 degree	*Clostridium bifermentans*
anaerobic/37 degree	*Clostridium bifermentans*
anaerobic/37 degree	*Clostridium bifermentans*
anaerobic/37 degree	*Clostridium bifermentans*
anaerobic/37 degree	*Clostridium bifermentans*
anaerobic/37 degree	*Clostridium bifermentans*
anaerobic/37 degree	*Clostridium bifermentans*
anaerobic/37 degree	*Clostridium bifermentans*
anaerobic/37 degree	*Clostridium bifermentans*

Table S5. The cultured strains and their culture atmospheres

**Atmosphere**	***species***
anaerobic/37 degree	*Clostridium bifermentans*
anaerobic/37 degree	*Clostridium bifermentans*
anaerobic/37 degree	*Clostridium bifermentans*
anaerobic/37 degree	*Clostridium bifermentans*
anaerobic/37 degree	*Clostridium bifermentans*
anaerobic/37 degree	*Clostridium bifermentans*
anaerobic/37 degree	*Clostridium bifermentans*
anaerobic/37 degree	*Clostridium bifermentans*
anaerobic/37 degree	*Clostridium bifermentans*
anaerobic/37 degree	*Clostridium bifermentans*
anaerobic/37 degree	*Clostridium bifermentans*
anaerobic/37 degree	*Clostridium bifermentans*
anaerobic/37 degree	*Clostridium bifermentans*
anaerobic/37 degree	*Clostridium bifermentans*
anaerobic/37 degree	*Clostridium bifermentans*
anaerobic/37 degree	*Clostridium bifermentans*
anaerobic/37 degree	*Clostridium bifermentans*
anaerobic/37 degree	*Clostridium bifermentans*
anaerobic/37 degree	*Clostridium bifermentans*
anaerobic/37 degree	*Clostridium bifermentans*
anaerobic/37 degree	*Clostridium bifermentans*
anaerobic/37 degree	*Clostridium bifermentans*
anaerobic/37 degree	*Clostridium bifermentans*
anaerobic/37 degree	*Clostridium bifermentans*
anaerobic/37 degree	*Clostridium bifermentans*
anaerobic/37 degree	*Clostridium bifermentans*
anaerobic/37 degree	*Clostridium bifermentans*
anaerobic/37 degree	*Clostridium bifermentans*
anaerobic/37 degree	*Clostridium bifermentans*
anaerobic/37 degree	*Clostridium bifermentans*
anaerobic/37 degree	*Clostridium bifermentans*
anaerobic/37 degree	*Clostridium bifermentans*
anaerobic/37 degree	*Clostridium bifermentans*
anaerobic/37 degree	*Clostridium bifermentans*
anaerobic/37 degree	*Clostridium bifermentans*
anaerobic/37 degree	*Clostridium bifermentans*
anaerobic/37 degree	*Clostridium bifermentans*
anaerobic/37 degree	*Clostridium bifermentans*
anaerobic/37 degree	*Clostridium bifermentans*
anaerobic/37 degree	*Clostridium bifermentans*
anaerobic/37 degree	*Clostridium bifermentans*
anaerobic/37 degree	*Clostridium bifermentans*
anaerobic/37 degree	*Clostridium bifermentans*
anaerobic/37 degree	*Clostridium bifermentans*

Table S5. The cultured strains and their culture atmospheres

**Atmosphere**	***species***
anaerobic/37 degree	*Clostridium bifermentans*
anaerobic/37 degree	*Clostridium bifermentans*
anaerobic/37 degree	*Clostridium bifermentans*
anaerobic/37 degree	*Clostridium bifermentans*
anaerobic/37 degree	*Clostridium bifermentans*
anaerobic/37 degree	*Clostridium bifermentans*
anaerobic/37 degree	*Clostridium bifermentans*
anaerobic/37 degree	*Clostridium bifermentans*
anaerobic/37 degree	*Clostridium bifermentans*
anaerobic/37 degree	*Clostridium bifermentans*
anaerobic/37 degree	*Clostridium bifermentans*
anaerobic/37 degree	*Clostridium bifermentans*
anaerobic/37 degree	*Clostridium bifermentans*
anaerobic/37 degree	*Clostridium bifermentans*
anaerobic/37 degree	*Clostridium bifermentans*
anaerobic/37 degree	*Clostridium bifermentans*
anaerobic/37 degree	*Clostridium bifermentans*
anaerobic/37 degree	*Clostridium bifermentans*
anaerobic/37 degree	*Clostridium bifermentans*
anaerobic/37 degree	*Clostridium bifermentans*
anaerobic/37 degree	*Clostridium bifermentans*
anaerobic/37 degree	*Clostridium bifermentans*
anaerobic/37 degree	*Clostridium bifermentans*
anaerobic/37 degree	*Clostridium bifermentans*
anaerobic/37 degree	*Clostridium bifermentans*
anaerobic/37 degree	*Clostridium bifermentans*
anaerobic/37 degree	*Clostridium bifermentans*
anaerobic/37 degree	*Clostridium bifermentans*
anaerobic/37 degree	*Clostridium bifermentans*
anaerobic/37 degree	*Clostridium bifermentans*
anaerobic/37 degree	*Clostridium bifermentans*
anaerobic/37 degree	*Clostridium bifermentans*
anaerobic/37 degree	*Clostridium bifermentans*
anaerobic/37 degree	*Clostridium bifermentans*
anaerobic/37 degree	*Clostridium bifermentans*
anaerobic/37 degree	*Clostridium bifermentans*
anaerobic/37 degree	*Clostridium bifermentans*
anaerobic/37 degree	*Clostridium bifermentans*
anaerobic/37 degree	*Clostridium bifermentans*
anaerobic/37 degree	*Clostridium bifermentans*
anaerobic/37 degree	*Clostridium bifermentans*
anaerobic/37 degree	*Clostridium bifermentans*
anaerobic/37 degree	*Clostridium bifermentans*
anaerobic/37 degree	*Clostridium bifermentans*

Table S5. The cultured strains and their culture atmospheres

Atmosphere	*species*
anaerobic/37 degree	*Clostridium bifermentans*
anaerobic/37 degree	*Clostridium bifermentans*
anaerobic/37 degree	*Clostridium bifermentans*
anaerobic/37 degree	*Clostridium bifermentans*
anaerobic/37 degree	*Clostridium bifermentans*
anaerobic/40-50 degree	*Clostridium bifermentans*
anaerobic/42 degree	*Clostridium bifermentans*
anaerobic/42 degree	*Clostridium bifermentans*
anaerobic/42 degree	*Clostridium bifermentans*
anaerobic/42 degree	*Clostridium bifermentans*
anaerobic/42 degree	*Clostridium bifermentans*
anaerobic/42 degree	*Clostridium bifermentans*
anaerobic/42 degree	*Clostridium bifermentans*
anaerobic/42 degree	*Clostridium bifermentans*
anaerobic/42 degree	*Clostridium bifermentans*
anaerobic/42 degree	*Clostridium bifermentans*
anaerobic/42 degree	*Clostridium bifermentans*
anaerobic/42 degree	*Clostridium bifermentans*
anaerobic/42 degree	*Clostridium bifermentans*
anaerobic/42 degree	*Clostridium bifermentans*
anaerobic/42 degree	*Clostridium bifermentans*
anaerobic/42 degree	*Clostridium bifermentans*
anaerobic/42 degree	*Clostridium bifermentans*
anaerobic/42 degree	*Clostridium bifermentans*
anaerobic/42 degree	*Clostridium bifermentans*
anaerobic/42 degree	*Clostridium bifermentans*
anaerobic/42 degree	*Clostridium bifermentans*
anaerobic/42 degree	*Clostridium bifermentans*
anaerobic/42 degree	*Clostridium bifermentans*
anaerobic/42 degree	*Clostridium bifermentans*
anaerobic/42 degree	*Clostridium bifermentans*
anaerobic/37 degree	*Clostridium bolteae*
anaerobic/35 degree	*Clostridium butyricum*
anaerobic/35 degree	*Clostridium butyricum*
anaerobic/35 degree	*Clostridium butyricum*
anaerobic/35 degree	*Clostridium butyricum*
anaerobic/35 degree	*Clostridium butyricum*
anaerobic/37 degree	*Clostridium butyricum*
anaerobic/37 degree	*Clostridium butyricum*
anaerobic/37 degree	*Clostridium butyricum*
anaerobic/37 degree	*Clostridium butyricum*
anaerobic/37 degree	*Clostridium butyricum*
anaerobic/37 degree	*Clostridium butyricum*
anaerobic/37 degree	*Clostridium butyricum*

Table S5. The cultured strains and their culture atmospheres

Atmosphere	*species*
anaerobic/37 degree	*Clostridium butyricum*
anaerobic/37 degree	*Clostridium butyricum*
anaerobic/37 degree	*Clostridium butyricum*
anaerobic/37 degree	*Clostridium butyricum*
anaerobic/37 degree	*Clostridium butyricum*
anaerobic/37 degree	*Clostridium butyricum*
anaerobic/37 degree	*Clostridium butyricum*
anaerobic/37 degree	*Clostridium butyricum*
anaerobic/37 degree	*Clostridium butyricum*
anaerobic/37 degree	*Clostridium butyricum*
anaerobic/37 degree	*Clostridium butyricum*
anaerobic/37 degree	*Clostridium butyricum*
anaerobic/37 degree	*Clostridium butyricum*
anaerobic/37 degree	*Clostridium butyricum*
anaerobic/37 degree	*Clostridium butyricum*
anaerobic/37 degree	*Clostridium butyricum*
anaerobic/37 degree	*Clostridium butyricum*
anaerobic/37 degree	*Clostridium butyricum*
anaerobic/37 degree	*Clostridium butyricum*
anaerobic/37 degree	*Clostridium butyricum*
anaerobic/37 degree	*Clostridium butyricum*
anaerobic/37 degree	*Clostridium butyricum*
anaerobic/37 degree	*Clostridium butyricum*
anaerobic/37 degree	*Clostridium butyricum*
anaerobic/37 degree	*Clostridium butyricum*
anaerobic/42 degree	*Clostridium butyricum*
anaerobic/42 degree	*Clostridium butyricum*
anaerobic/42 degree	*Clostridium butyricum*
anaerobic/42 degree	*Clostridium butyricum*
anaerobic/42 degree	*Clostridium butyricum*
anaerobic/42 degree	*Clostridium butyricum*
anaerobic/42 degree	*Clostridium butyricum*
anaerobic/35 degree	*Clostridium cadaveris*
anaerobic/35 degree	*Clostridium cadaveris*
anaerobic/37 degree	*Clostridium cadaveris*
anaerobic/37 degree	*Clostridium cadaveris*
anaerobic/37 degree	*Clostridium cadaveris*
anaerobic/37 degree	*Clostridium cadaveris*
anaerobic/37 degree	*Clostridium cadaveris*
anaerobic/37 degree	*Clostridium cadaveris*
anaerobic/37 degree	*Clostridium cadaveris*
anaerobic/37 degree	*Clostridium cadaveris*
anaerobic/37 degree	*Clostridium cadaveris*
anaerobic/37 degree	*Clostridium cadaveris*

Table S5. The cultured strains and their culture atmospheres

**Atmosphere**	***species***
anaerobic/37 degree	*Clostridium cadaveris*
anaerobic/37 degree	*Clostridium cadaveris*
anaerobic/37 degree	*Clostridium cadaveris*
anaerobic/37 degree	*Clostridium cadaveris*
anaerobic/37 degree	*Clostridium cadaveris*
anaerobic/37 degree	*Clostridium cadaveris*
anaerobic/37 degree	*Clostridium cadaveris*
anaerobic/37 degree	*Clostridium cadaveris*
anaerobic/37 degree	*Clostridium cadaveris*
anaerobic/37 degree	*Clostridium cadaveris*
anaerobic/37 degree	*Clostridium cadaveris*
anaerobic/37 degree	*Clostridium cadaveris*
anaerobic/37 degree	*Clostridium cadaveris*
anaerobic/37 degree	*Clostridium cadaveris*
anaerobic/37 degree	*Clostridium cadaveris*
anaerobic/37 degree	*Clostridium cadaveris*
anaerobic/37 degree	*Clostridium cadaveris*
anaerobic/37 degree	*Clostridium cadaveris*
anaerobic/37 degree	*Clostridium cadaveris*
anaerobic/42 degree	*Clostridium cadaveris*
anaerobic/42 degree	*Clostridium cadaveris*
anaerobic/42 degree	*Clostridium cadaveris*
anaerobic/42 degree	*Clostridium cadaveris*
anaerobic/42 degree	*Clostridium cadaveris*
anaerobic/42 degree	*Clostridium cadaveris*
anaerobic/42 degree	*Clostridium cadaveris*
anaerobic/42 degree	*Clostridium cadaveris*
anaerobic/42 degree	*Clostridium cadaveris*
anaerobic/37 degree	*Clostridium difficile*
anaerobic/37 degree	*Clostridium difficile*
anaerobic/37 degree	*Clostridium disporicum*
anaerobic/37 degree	*Clostridium disporicum*
anaerobic/37 degree	*Clostridium disporicum*
anaerobic/37 degree	*Clostridium disporicum*
anaerobic/37 degree	*Clostridium disporicum*
anaerobic/37 degree	*Clostridium disporicum*
anaerobic/37 degree	*Clostridium disporicum*
anaerobic/37 degree	*Clostridium hathewayi*
anaerobic/37 degree	*Clostridium hathewayi*
anaerobic/37 degree	*Clostridium hathewayi*
anaerobic/37 degree	*Clostridium hathewayi*
anaerobic/37 degree	*Clostridium hathewayi*
anaerobic/37 degree	*Clostridium hathewayi*
anaerobic/37 degree	*Clostridium hathewayi*

Table S5. The cultured strains and their culture atmospheres

Atmosphere	*species*
anaerobic/37 degree	*Clostridium hathewayi*
anaerobic/37 degree	*Clostridium hathewayi*
anaerobic/37 degree	*Clostridium hathewayi*
anaerobic/42 degree	*Clostridium hathewayi*
anaerobic/37 degree	*Clostridium ihumii*
anaerobic/37 degree	*Clostridium ihumii*
anaerobic/37 degree	*Clostridium ihumii*
anaerobic/37 degree	*Clostridium ihumii*
anaerobic/37 degree	*Clostridium ihumii*
anaerobic/37 degree	*Clostridium ihumii*
anaerobic/37 degree	*Clostridium ihumii*
anaerobic/37 degree	*Clostridium ihumii*
anaerobic/37 degree	*Clostridium ihumii*
anaerobic/37 degree	*Clostridium ihumii*
anaerobic/37 degree	*Clostridium ihumii*
anaerobic/35 degree	*Clostridium innocuum*
anaerobic/37 degree	*Clostridium innocuum*
anaerobic/37 degree	*Clostridium innocuum*
anaerobic/37 degree	*Clostridium innocuum*
anaerobic/37 degree	*Clostridium innocuum*
anaerobic/37 degree	*Clostridium innocuum*
anaerobic/37 degree	*Clostridium innocuum*
anaerobic/37 degree	*Clostridium innocuum*
anaerobic/37 degree	*Clostridium innocuum*
anaerobic/37 degree	*Clostridium innocuum*
anaerobic/37 degree	*Clostridium innocuum*
anaerobic/37 degree	*Clostridium innocuum*
anaerobic/37 degree	*Clostridium paraputrificum*
anaerobic/37 degree	*Clostridium paraputrificum*
anaerobic/37 degree	*Clostridium paraputrificum*
anaerobic/37 degree	*Clostridium paraputrificum*
anaerobic/37 degree	*Clostridium paraputrificum*
anaerobic/37 degree	*Clostridium paraputrificum*
anaerobic/37 degree	*Clostridium paraputrificum*
anaerobic/37 degree	*Clostridium paraputrificum*
anaerobic/37 degree	*Clostridium paraputrificum*
anaerobic/37 degree	*Clostridium paraputrificum*
anaerobic/37 degree	*Clostridium paraputrificum*
anaerobic/37 degree	*Clostridium paraputrificum*
anaerobic/37 degree	*Clostridium paraputrificum*
anaerobic/37 degree	*Clostridium paraputrificum*
anaerobic/37 degree	*Clostridium paraputrificum*
anaerobic/37 degree	*Clostridium paraputrificum*
anaerobic/37 degree	*Clostridium paraputrificum*

Table S5. The cultured strains and their culture atmospheres

**Atmosphere**	***species***
anaerobic/37 degree	*Clostridium paraputrificum*
anaerobic/37 degree	*Clostridium paraputrificum*
anaerobic/37 degree	*Clostridium paraputrificum*
anaerobic/37 degree	*Clostridium paraputrificum*
anaerobic/42 degree	*Clostridium paraputrificum*
anaerobic/35 degree	*Clostridium perfringens*
anaerobic/35 degree	*Clostridium perfringens*
anaerobic/35 degree	*Clostridium perfringens*
anaerobic/35 degree	*Clostridium perfringens*
anaerobic/35 degree	*Clostridium perfringens*
anaerobic/37 degree	*Clostridium perfringens*
anaerobic/37 degree	*Clostridium perfringens*
anaerobic/37 degree	*Clostridium perfringens*
anaerobic/37 degree	*Clostridium perfringens*
anaerobic/37 degree	*Clostridium perfringens*
anaerobic/37 degree	*Clostridium perfringens*
anaerobic/37 degree	*Clostridium perfringens*
anaerobic/37 degree	*Clostridium perfringens*
anaerobic/37 degree	*Clostridium perfringens*
anaerobic/37 degree	*Clostridium perfringens*
anaerobic/37 degree	*Clostridium perfringens*
anaerobic/37 degree	*Clostridium perfringens*
anaerobic/37 degree	*Clostridium perfringens*
anaerobic/37 degree	*Clostridium perfringens*
anaerobic/37 degree	*Clostridium perfringens*
anaerobic/37 degree	*Clostridium perfringens*
anaerobic/37 degree	*Clostridium perfringens*
anaerobic/37 degree	*Clostridium perfringens*
anaerobic/37 degree	*Clostridium perfringens*
anaerobic/37 degree	*Clostridium perfringens*
anaerobic/37 degree	*Clostridium perfringens*
anaerobic/37 degree	*Clostridium perfringens*
anaerobic/37 degree	*Clostridium perfringens*
anaerobic/37 degree	*Clostridium perfringens*
anaerobic/37 degree	*Clostridium perfringens*
anaerobic/37 degree	*Clostridium perfringens*
anaerobic/37 degree	*Clostridium perfringens*
anaerobic/37 degree	*Clostridium perfringens*
anaerobic/37 degree	*Clostridium perfringens*
anaerobic/37 degree	*Clostridium perfringens*
anaerobic/37 degree	*Clostridium perfringens*
anaerobic/37 degree	*Clostridium perfringens*
anaerobic/37 degree	*Clostridium perfringens*
anaerobic/37 degree	*Clostridium perfringens*
anaerobic/37 degree	*Clostridium perfringens*

Table S5. The cultured strains and their culture atmospheres

**Atmosphere**	***species***
anaerobic/37 degree	*Clostridium perfringens*
anaerobic/37 degree	*Clostridium perfringens*
anaerobic/37 degree	*Clostridium perfringens*
anaerobic/37 degree	*Clostridium perfringens*
anaerobic/37 degree	*Clostridium perfringens*
anaerobic/37 degree	*Clostridium perfringens*
anaerobic/37 degree	*Clostridium perfringens*
anaerobic/37 degree	*Clostridium perfringens*
anaerobic/37 degree	*Clostridium perfringens*
anaerobic/37 degree	*Clostridium perfringens*
anaerobic/37 degree	*Clostridium perfringens*
anaerobic/37 degree	*Clostridium perfringens*
anaerobic/37 degree	*Clostridium perfringens*
anaerobic/37 degree	*Clostridium perfringens*
anaerobic/37 degree	*Clostridium perfringens*
anaerobic/37 degree	*Clostridium perfringens*
anaerobic/37 degree	*Clostridium perfringens*
anaerobic/37 degree	*Clostridium perfringens*
anaerobic/37 degree	*Clostridium perfringens*
anaerobic/37 degree	*Clostridium perfringens*
anaerobic/37 degree	*Clostridium perfringens*
anaerobic/37 degree	*Clostridium perfringens*
anaerobic/37 degree	*Clostridium perfringens*
anaerobic/37 degree	*Clostridium perfringens*
anaerobic/37 degree	*Clostridium perfringens*
anaerobic/37 degree	*Clostridium perfringens*
anaerobic/37 degree	*Clostridium perfringens*
anaerobic/37 degree	*Clostridium perfringens*
anaerobic/37 degree	*Clostridium perfringens*
anaerobic/37 degree	*Clostridium perfringens*
anaerobic/37 degree	*Clostridium perfringens*
anaerobic/37 degree	*Clostridium perfringens*
anaerobic/37 degree	*Clostridium perfringens*
anaerobic/37 degree	*Clostridium perfringens*
anaerobic/37 degree	*Clostridium perfringens*
anaerobic/37 degree	*Clostridium perfringens*
anaerobic/37 degree	*Clostridium perfringens*
anaerobic/37 degree	*Clostridium perfringens*
anaerobic/37 degree	*Clostridium perfringens*
anaerobic/37 degree	*Clostridium perfringens*
anaerobic/37 degree	*Clostridium perfringens*
anaerobic/37 degree	*Clostridium perfringens*
anaerobic/37 degree	*Clostridium perfringens*
anaerobic/37 degree	*Clostridium perfringens*

Table S5. The cultured strains and their culture atmospheres

**Atmosphere**	***species***
anaerobic/37 degree	*Clostridium perfringens*
anaerobic/37 degree	*Clostridium perfringens*
anaerobic/37 degree	*Clostridium perfringens*
anaerobic/37 degree	*Clostridium perfringens*
anaerobic/37 degree	*Clostridium perfringens*
anaerobic/37 degree	*Clostridium perfringens*
anaerobic/37 degree	*Clostridium perfringens*
anaerobic/37 degree	*Clostridium perfringens*
anaerobic/37 degree	*Clostridium perfringens*
anaerobic/37 degree	*Clostridium perfringens*
anaerobic/37 degree	*Clostridium perfringens*
anaerobic/37 degree	*Clostridium perfringens*
anaerobic/37 degree	*Clostridium perfringens*
anaerobic/37 degree	*Clostridium perfringens*
anaerobic/37 degree	*Clostridium perfringens*
anaerobic/37 degree	*Clostridium perfringens*
anaerobic/37 degree	*Clostridium perfringens*
anaerobic/37 degree	*Clostridium perfringens*
anaerobic/37 degree	*Clostridium perfringens*
anaerobic/37 degree	*Clostridium perfringens*
anaerobic/37 degree	*Clostridium perfringens*
anaerobic/37 degree	*Clostridium perfringens*
anaerobic/37 degree	*Clostridium perfringens*
anaerobic/37 degree	*Clostridium perfringens*
anaerobic/37 degree	*Clostridium perfringens*
anaerobic/37 degree	*Clostridium perfringens*
anaerobic/37 degree	*Clostridium perfringens*
anaerobic/37 degree	*Clostridium perfringens*
anaerobic/37 degree	*Clostridium perfringens*
anaerobic/37 degree	*Clostridium perfringens*
anaerobic/37 degree	*Clostridium perfringens*
anaerobic/37 degree	*Clostridium perfringens*
anaerobic/37 degree	*Clostridium perfringens*
anaerobic/37 degree	*Clostridium perfringens*
anaerobic/37 degree	*Clostridium perfringens*
anaerobic/37 degree	*Clostridium perfringens*
anaerobic/37 degree	*Clostridium perfringens*
anaerobic/37 degree	*Clostridium perfringens*
anaerobic/37 degree	*Clostridium perfringens*
anaerobic/37 degree	*Clostridium perfringens*
anaerobic/37 degree	*Clostridium perfringens*
anaerobic/37 degree	*Clostridium perfringens*
anaerobic/37 degree	*Clostridium perfringens*
anaerobic/37 degree	*Clostridium perfringens*

Table S5. The cultured strains and their culture atmospheres

**Atmosphere**	***species***
anaerobic/37 degree	*Clostridium perfringens*
anaerobic/37 degree	*Clostridium perfringens*
anaerobic/37 degree	*Clostridium perfringens*
anaerobic/37 degree	*Clostridium perfringens*
anaerobic/37 degree	*Clostridium perfringens*
anaerobic/37 degree	*Clostridium perfringens*
anaerobic/37 degree	*Clostridium perfringens*
anaerobic/37 degree	*Clostridium perfringens*
anaerobic/37 degree	*Clostridium perfringens*
anaerobic/37 degree	*Clostridium perfringens*
anaerobic/37 degree	*Clostridium perfringens*
anaerobic/37 degree	*Clostridium perfringens*
anaerobic/37 degree	*Clostridium perfringens*
anaerobic/37 degree	*Clostridium perfringens*
anaerobic/37 degree	*Clostridium perfringens*
anaerobic/37 degree	*Clostridium perfringens*
anaerobic/37 degree	*Clostridium perfringens*
anaerobic/37 degree	*Clostridium perfringens*
anaerobic/37 degree	*Clostridium perfringens*
anaerobic/37 degree	*Clostridium perfringens*
anaerobic/37 degree	*Clostridium perfringens*
anaerobic/37 degree	*Clostridium perfringens*
anaerobic/37 degree	*Clostridium perfringens*
anaerobic/37 degree	*Clostridium perfringens*
anaerobic/37 degree	*Clostridium perfringens*
anaerobic/37 degree	*Clostridium perfringens*
anaerobic/37 degree	*Clostridium perfringens*
anaerobic/37 degree	*Clostridium perfringens*
anaerobic/37 degree	*Clostridium perfringens*
anaerobic/37 degree	*Clostridium perfringens*
anaerobic/37 degree	*Clostridium perfringens*
anaerobic/37 degree	*Clostridium perfringens*
anaerobic/37 degree	*Clostridium perfringens*
anaerobic/37 degree	*Clostridium perfringens*
anaerobic/37 degree	*Clostridium perfringens*
anaerobic/37 degree	*Clostridium perfringens*
anaerobic/37 degree	*Clostridium perfringens*
anaerobic/37 degree	*Clostridium perfringens*
anaerobic/37 degree	*Clostridium perfringens*
anaerobic/37 degree	*Clostridium perfringens*
anaerobic/37 degree	*Clostridium perfringens*
anaerobic/37 degree	*Clostridium perfringens*
anaerobic/37 degree	*Clostridium perfringens*
anaerobic/37 degree	*Clostridium perfringens*

Table S5. The cultured strains and their culture atmospheres

**Atmosphere**	***species***
anaerobic/37 degree	*Clostridium perfringens*
anaerobic/37 degree	*Clostridium perfringens*
anaerobic/37 degree	*Clostridium perfringens*
anaerobic/37 degree	*Clostridium perfringens*
anaerobic/37 degree	*Clostridium perfringens*
anaerobic/37 degree	*Clostridium perfringens*
anaerobic/37 degree	*Clostridium perfringens*
anaerobic/37 degree	*Clostridium perfringens*
anaerobic/37 degree	*Clostridium perfringens*
anaerobic/37 degree	*Clostridium perfringens*
anaerobic/37 degree	*Clostridium perfringens*
anaerobic/37 degree	*Clostridium perfringens*
anaerobic/37 degree	*Clostridium perfringens*
anaerobic/37 degree	*Clostridium perfringens*
anaerobic/37 degree	*Clostridium perfringens*
anaerobic/37 degree	*Clostridium perfringens*
anaerobic/37 degree	*Clostridium perfringens*
anaerobic/37 degree	*Clostridium perfringens*
anaerobic/37 degree	*Clostridium perfringens*
anaerobic/37 degree	*Clostridium perfringens*
anaerobic/37 degree	*Clostridium perfringens*
anaerobic/37 degree	*Clostridium perfringens*
anaerobic/37 degree	*Clostridium perfringens*
anaerobic/37 degree	*Clostridium perfringens*
anaerobic/37 degree	*Clostridium perfringens*
anaerobic/37 degree	*Clostridium perfringens*
anaerobic/37 degree	*Clostridium perfringens*
anaerobic/37 degree	*Clostridium perfringens*
anaerobic/37 degree	*Clostridium perfringens*
anaerobic/37 degree	*Clostridium perfringens*
anaerobic/37 degree	*Clostridium perfringens*
anaerobic/37 degree	*Clostridium perfringens*
anaerobic/37 degree	*Clostridium perfringens*
anaerobic/37 degree	*Clostridium perfringens*
anaerobic/37 degree	*Clostridium perfringens*
anaerobic/37 degree	*Clostridium perfringens*
anaerobic/37 degree	*Clostridium perfringens*
anaerobic/37 degree	*Clostridium perfringens*
anaerobic/37 degree	*Clostridium perfringens*
anaerobic/37 degree	*Clostridium perfringens*
anaerobic/37 degree	*Clostridium perfringens*
anaerobic/37 degree	*Clostridium perfringens*
anaerobic/37 degree	*Clostridium perfringens*
anaerobic/37 degree	*Clostridium perfringens*

Table S5. The cultured strains and their culture atmospheres

**Atmosphere**	***species***
anaerobic/37 degree	*Clostridium perfringens*
anaerobic/37 degree	*Clostridium perfringens*
anaerobic/37 degree	*Clostridium perfringens*
anaerobic/37 degree	*Clostridium perfringens*
anaerobic/37 degree	*Clostridium perfringens*
anaerobic/37 degree	*Clostridium perfringens*
anaerobic/37 degree	*Clostridium perfringens*
anaerobic/37 degree	*Clostridium perfringens*
anaerobic/37 degree	*Clostridium perfringens*
anaerobic/37 degree	*Clostridium perfringens*
anaerobic/37 degree	*Clostridium perfringens*
anaerobic/37 degree	*Clostridium perfringens*
anaerobic/37 degree	*Clostridium perfringens*
anaerobic/37 degree	*Clostridium perfringens*
anaerobic/37 degree	*Clostridium perfringens*
anaerobic/37 degree	*Clostridium perfringens*
anaerobic/37 degree	*Clostridium perfringens*
anaerobic/37 degree	*Clostridium perfringens*
anaerobic/37 degree	*Clostridium perfringens*
anaerobic/37 degree	*Clostridium perfringens*
anaerobic/37 degree	*Clostridium perfringens*
anaerobic/37 degree	*Clostridium perfringens*
anaerobic/37 degree	*Clostridium perfringens*
anaerobic/37 degree	*Clostridium perfringens*
anaerobic/37 degree	*Clostridium perfringens*
anaerobic/37 degree	*Clostridium perfringens*
anaerobic/37 degree	*Clostridium perfringens*
anaerobic/37 degree	*Clostridium perfringens*
anaerobic/37 degree	*Clostridium perfringens*
anaerobic/37 degree	*Clostridium perfringens*
anaerobic/37 degree	*Clostridium perfringens*
anaerobic/37 degree	*Clostridium perfringens*
anaerobic/37 degree	*Clostridium perfringens*
anaerobic/37 degree	*Clostridium perfringens*
anaerobic/37 degree	*Clostridium perfringens*
anaerobic/37 degree	*Clostridium perfringens*
anaerobic/37 degree	*Clostridium perfringens*
anaerobic/37 degree	*Clostridium perfringens*
anaerobic/37 degree	*Clostridium perfringens*
anaerobic/37 degree	*Clostridium perfringens*
anaerobic/37 degree	*Clostridium perfringens*
anaerobic/37 degree	*Clostridium perfringens*
anaerobic/37 degree	*Clostridium perfringens*
anaerobic/37 degree	*Clostridium perfringens*

Table S5. The cultured strains and their culture atmospheres

Atmosphere	*species*
anaerobic/37 degree	*Clostridium perfringens*
anaerobic/37 degree	*Clostridium perfringens*
anaerobic/37 degree	*Clostridium perfringens*
anaerobic/37 degree	*Clostridium perfringens*
anaerobic/37 degree	*Clostridium perfringens*
anaerobic/37 degree	*Clostridium perfringens*
anaerobic/37 degree	*Clostridium perfringens*
anaerobic/37 degree	*Clostridium perfringens*
anaerobic/37 degree	*Clostridium perfringens*
anaerobic/37 degree	*Clostridium perfringens*
anaerobic/37 degree	*Clostridium perfringens*
anaerobic/37 degree	*Clostridium perfringens*
anaerobic/37 degree	*Clostridium perfringens*
anaerobic/37 degree	*Clostridium perfringens*
anaerobic/37 degree	*Clostridium perfringens*
anaerobic/37 degree	*Clostridium perfringens*
anaerobic/37 degree	*Clostridium perfringens*
anaerobic/37 degree	*Clostridium perfringens*
anaerobic/37 degree	*Clostridium perfringens*
anaerobic/37 degree	*Clostridium perfringens*
anaerobic/37 degree	*Clostridium perfringens*
anaerobic/37 degree	*Clostridium perfringens*
anaerobic/37 degree	*Clostridium perfringens*
anaerobic/37 degree	*Clostridium perfringens*
anaerobic/37 degree	*Clostridium perfringens*
anaerobic/37 degree	*Clostridium perfringens*
anaerobic/37 degree	*Clostridium perfringens*
anaerobic/37 degree	*Clostridium perfringens*
anaerobic/37 degree	*Clostridium perfringens*
anaerobic/37 degree	*Clostridium perfringens*
anaerobic/37 degree	*Clostridium perfringens*
anaerobic/37 degree	*Clostridium perfringens*
anaerobic/37 degree	*Clostridium perfringens*
anaerobic/42 degree	*Clostridium perfringens*
anaerobic/42 degree	*Clostridium perfringens*
anaerobic/42 degree	*Clostridium perfringens*
anaerobic/42 degree	*Clostridium perfringens*
anaerobic/42 degree	*Clostridium perfringens*
anaerobic/42 degree	*Clostridium perfringens*
anaerobic/42 degree	*Clostridium perfringens*
anaerobic/42 degree	*Clostridium perfringens*
anaerobic/42 degree	*Clostridium perfringens*
anaerobic/42 degree	*Clostridium perfringens*
anaerobic/37 degree	*Clostridium ramosum*

Table S5. The cultured strains and their culture atmospheres

Atmosphere	*species*
anaerobic/37 degree	*Clostridium ramosum*
anaerobic/37 degree	*Clostridium ramosum*
anaerobic/37 degree	*Clostridium ramosum*
anaerobic/37 degree	*Clostridium scindens*
anaerobic/37 degree	*Clostridium scindens*
anaerobic/37 degree	*Clostridium scindens*
anaerobic/37 degree	*Clostridium scindens*
anaerobic/37 degree	*Clostridium scindens*
anaerobic/37 degree	*Clostridium scindens*
anaerobic/37 degree	*Clostridium scindens*
anaerobic/37 degree	*Clostridium scindens*
anaerobic/42 degree	*Clostridium scindens*
anaerobic/37 degree	*Clostridium sordellii*
anaerobic/37 degree	*Clostridium sordellii*
anaerobic/37 degree	*Clostridium sordellii*
anaerobic/37 degree	*Clostridium sordellii*
anaerobic/37 degree	*Clostridium sordellii*
anaerobic/37 degree	*Clostridium sordellii*
anaerobic/37 degree	*Clostridium sordellii*
anaerobic/37 degree	*Clostridium sordellii*
anaerobic/37 degree	*Clostridium sordellii*
anaerobic/37 degree	*Clostridium sordellii*
anaerobic/37 degree	*Clostridium sordellii*
anaerobic/37 degree	*Clostridium sordellii*
anaerobic/37 degree	*Clostridium sordellii*
anaerobic/37 degree	*Clostridium sordellii*
anaerobic/37 degree	*Clostridium sordellii*
anaerobic/37 degree	*Clostridium sordellii*
anaerobic/37 degree	*Clostridium sordellii*
anaerobic/37 degree	*Clostridium sordellii*
anaerobic/37 degree	*Clostridium sordellii*
anaerobic/37 degree	*Clostridium sordellii*
anaerobic/37 degree	*Clostridium sordellii*
anaerobic/37 degree	*Clostridium sordellii*
anaerobic/37 degree	*Clostridium sordellii*
anaerobic/37 degree	*Clostridium sordellii*
anaerobic/37 degree	*Clostridium sordellii*
anaerobic/37 degree	*Clostridium sordellii*
anaerobic/37 degree	*Clostridium sordellii*
anaerobic/37 degree	*Clostridium sordellii*
anaerobic/37 degree	*Clostridium sordellii*
anaerobic/37 degree	*Clostridium sordellii*
anaerobic/37 degree	*Clostridium sordellii*
anaerobic/37 degree	*Clostridium sordellii*
anaerobic/37 degree	*Clostridium sordellii*
anaerobic/37 degree	*Clostridium sordellii*

Table S5. The cultured strains and their culture atmospheres

**Atmosphere**	***species***
anaerobic/37 degree	*Clostridium sordellii*
anaerobic/37 degree	*Clostridium sordellii*
anaerobic/37 degree	*Clostridium sordellii*
anaerobic/37 degree	*Clostridium sordellii*
anaerobic/37 degree	*Clostridium sordellii*
anaerobic/37 degree	*Clostridium sordellii*
anaerobic/37 degree	*Clostridium sordellii*
anaerobic/37 degree	*Clostridium sordellii*
anaerobic/37 degree	*Clostridium sordellii*
anaerobic/37 degree	*Clostridium sordellii*
anaerobic/37 degree	*Clostridium sordellii*
anaerobic/37 degree	*Clostridium sordellii*
anaerobic/37 degree	*Clostridium sordellii*
anaerobic/37 degree	*Clostridium sordellii*
anaerobic/37 degree	*Clostridium sordellii*
anaerobic/37 degree	*Clostridium sordellii*
anaerobic/37 degree	*Clostridium sordellii*
anaerobic/37 degree	*Clostridium sordellii*
anaerobic/37 degree	*Clostridium sordellii*
anaerobic/37 degree	*Clostridium sordellii*
anaerobic/37 degree	*Clostridium sordellii*
anaerobic/37 degree	*Clostridium sordellii*
anaerobic/37 degree	*Clostridium sordellii*
anaerobic/37 degree	*Clostridium sordellii*
anaerobic/37 degree	*Clostridium sordellii*
anaerobic/37 degree	*Clostridium sordellii*
anaerobic/37 degree	*Clostridium symbiosum*
anaerobic/37 degree	*Clostridium symbiosum*
anaerobic/37 degree	*Clostridium symbiosum*
anaerobic/37 degree	*Clostridium symbiosum*
anaerobic/37 degree	*Clostridium symbiosum*
anaerobic/37 degree	*Clostridium symbiosum*
anaerobic/37 degree	*Clostridium symbiosum*
anaerobic/37 degree	*Clostridium symbiosum*
anaerobic/37 degree	*Clostridium symbiosum*
anaerobic/37 degree	*Clostridium symbiosum*
anaerobic/35 degree	*Clostridium tertium*
anaerobic/37 degree	*Clostridium tertium*
anaerobic/37 degree	*Clostridium tertium*
anaerobic/37 degree	*Clostridium tertium*
anaerobic/37 degree	*Clostridium tertium*
anaerobic/37 degree	*Clostridium tertium*
anaerobic/37 degree	*Clostridium tertium*
anaerobic/37 degree	*Clostridium tertium*

Table S5. The cultured strains and their culture atmospheres

**Atmosphere**	***species***
anaerobic/37 degree	*Clostridium tertium*
anaerobic/37 degree	*Clostridium tertium*
anaerobic/37 degree	*Clostridium tertium*
anaerobic/37 degree	*Clostridium tertium*
anaerobic/37 degree	*Clostridium tertium*
anaerobic/37 degree	*Clostridium tertium*
anaerobic/37 degree	*Clostridium tertium*
anaerobic/37 degree	*Clostridium tertium*
anaerobic/37 degree	*Clostridium tertium*
anaerobic/37 degree	*Clostridium tertium*
anaerobic/37 degree	*Clostridium tertium*
anaerobic/37 degree	*Clostridium tertium*
anaerobic/37 degree	*Clostridium tertium*
anaerobic/37 degree	*Clostridium tertium*
anaerobic/37 degree	*Clostridium tertium*
anaerobic/37 degree	*Clostridium tertium*
anaerobic/37 degree	*Clostridium tertium*
anaerobic/37 degree	*Clostridium tertium*
anaerobic/37 degree	*Clostridium tertium*
anaerobic/37 degree	*Clostridium tertium*
anaerobic/37 degree	*Clostridium tertium*
anaerobic/37 degree	*Clostridium tertium*
anaerobic/37 degree	*Clostridium tertium*
anaerobic/37 degree	*Clostridium tertium*
anaerobic/37 degree	*Clostridium tertium*
anaerobic/37 degree	*Clostridium tertium*
anaerobic/37 degree	*Clostridium tertium*
anaerobic/37 degree	*Clostridium tertium*
anaerobic/37 degree	*Clostridium tertium*
anaerobic/37 degree	*Clostridium tertium*
anaerobic/37 degree	*Clostridium tertium*
anaerobic/37 degree	*Clostridium tertium*
anaerobic/37 degree	*Clostridium tertium*
anaerobic/37 degree	*Clostridium tertium*
anaerobic/37 degree	*Clostridium tertium*
anaerobic/37 degree	*Clostridium tertium*
anaerobic/37 degree	*Clostridium tertium*
anaerobic/37 degree	*Clostridium tertium*
anaerobic/37 degree	*Clostridium tertium*
anaerobic/37 degree	*Clostridium tertium*
anaerobic/37 degree	*Clostridium tertium*
anaerobic/37 degree	*Clostridium tertium*
anaerobic/37 degree	*Clostridium tertium*
anaerobic/37 degree	*Clostridium tertium*

Table S5. The cultured strains and their culture atmospheres

**Atmosphere**	***species***
anaerobic/37 degree	*Clostridium tertium*
anaerobic/37 degree	*Clostridium tertium*
anaerobic/37 degree	*Clostridium tertium*
anaerobic/37 degree	*Clostridium tertium*
anaerobic/37 degree	*Clostridium tertium*
anaerobic/37 degree	*Clostridium tertium*
anaerobic/37 degree	*Clostridium tertium*
anaerobic/37 degree	*Clostridium tertium*
anaerobic/37 degree	*Clostridium tertium*
anaerobic/37 degree	*Clostridium tertium*
anaerobic/37 degree	*Clostridium tertium*
anaerobic/37 degree	*Clostridium tertium*
anaerobic/37 degree	*Clostridium tertium*
anaerobic/37 degree	*Clostridium tertium*
anaerobic/37 degree	*Clostridium tertium*
anaerobic/37 degree	*Clostridium tertium*
anaerobic/37 degree	*Clostridium tertium*
anaerobic/37 degree	*Clostridium tertium*
anaerobic/37 degree	*Clostridium tertium*
anaerobic/37 degree	*Clostridium tertium*
anaerobic/37 degree	*Clostridium tertium*
anaerobic/37 degree	*Clostridium tertium*
anaerobic/37 degree	*Clostridium tertium*
anaerobic/37 degree	*Clostridium tertium*
anaerobic/37 degree	*Clostridium tertium*
anaerobic/37 degree	*Clostridium tertium*
anaerobic/37 degree	*Clostridium tertium*
anaerobic/37 degree	*Clostridium tertium*
anaerobic/37 degree	*Clostridium tertium*
anaerobic/37 degree	*Clostridium tertium*
anaerobic/37 degree	*Clostridium tertium*
anaerobic/37 degree	*Clostridium tertium*
anaerobic/37 degree	*Clostridium tertium*
anaerobic/37 degree	*Clostridium tertium*
anaerobic/37 degree	*Clostridium tertium*
anaerobic/37 degree	*Clostridium tertium*
anaerobic/37 degree	*Clostridium tertium*
anaerobic/37 degree	*Clostridium tertium*
anaerobic/37 degree	*Clostridium tertium*
anaerobic/37 degree	*Clostridium tertium*
anaerobic/37 degree	*Clostridium tertium*
anaerobic/37 degree	*Clostridium tertium*
anaerobic/37 degree	*Clostridium tertium*
anaerobic/37 degree	*Clostridium tertium*

Table S5. The cultured strains and their culture atmospheres

**Atmosphere**	***species***
anaerobic/37 degree	*Clostridium tertium*
anaerobic/37 degree	*Clostridium tertium*
anaerobic/37 degree	*Clostridium tertium*
anaerobic/37 degree	*Clostridium tertium*
anaerobic/37 degree	*Clostridium tertium*
anaerobic/37 degree	*Clostridium tertium*
anaerobic/37 degree	*Clostridium tertium*
anaerobic/37 degree	*Clostridium tertium*
anaerobic/37 degree	*Clostridium tertium*
anaerobic/37 degree	*Clostridium tertium*
anaerobic/37 degree	*Clostridium tertium*
anaerobic/37 degree	*Clostridium tertium*
anaerobic/37 degree	*Clostridium tertium*
anaerobic/37 degree	*Clostridium tertium*
anaerobic/37 degree	*Clostridium tertium*
anaerobic/37 degree	*Clostridium tertium*
anaerobic/37 degree	*Clostridium tertium*
anaerobic/37 degree	*Clostridium tertium*
anaerobic/37 degree	*Clostridium tertium*
anaerobic/37 degree	*Clostridium tertium*
anaerobic/37 degree	*Clostridium tertium*
anaerobic/37 degree	*Clostridium tertium*
anaerobic/37 degree	*Clostridium tertium*
anaerobic/37 degree	*Clostridium tertium*
anaerobic/37 degree	*Clostridium tertium*
anaerobic/37 degree	*Clostridium tertium*
anaerobic/37 degree	*Clostridium tertium*
anaerobic/37 degree	*Clostridium tertium*
anaerobic/37 degree	*Clostridium tertium*
anaerobic/37 degree	*Clostridium tertium*
anaerobic/37 degree	*Clostridium tertium*
anaerobic/37 degree	*Clostridium tertium*
anaerobic/37 degree	*Clostridium tertium*
anaerobic/37 degree	*Clostridium tertium*
anaerobic/37 degree	*Clostridium tertium*
anaerobic/37 degree	*Clostridium tertium*
anaerobic/37 degree	*Clostridium tertium*
anaerobic/37 degree	*Clostridium tertium*
anaerobic/37 degree	*Clostridium tertium*
anaerobic/37 degree	*Clostridium tertium*
anaerobic/37 degree	*Clostridium tertium*
anaerobic/37 degree	*Clostridium tertium*
anaerobic/37 degree	*Clostridium tertium*
anaerobic/37 degree	*Clostridium tertium*

Table S5. The cultured strains and their culture atmospheres

**Atmosphere**	***species***
anaerobic/37 degree	*Clostridium tertium*
anaerobic/37 degree	*Clostridium tertium*
anaerobic/37 degree	*Clostridium tertium*
anaerobic/37 degree	*Clostridium tertium*
anaerobic/37 degree	*Clostridium tertium*
anaerobic/37 degree	*Clostridium tertium*
anaerobic/37 degree	*Clostridium tertium*
anaerobic/37 degree	*Clostridium tertium*
anaerobic/37 degree	*Clostridium tertium*
anaerobic/37 degree	*Clostridium tertium*
anaerobic/37 degree	*Clostridium tertium*
anaerobic/37 degree	*Clostridium tertium*
anaerobic/37 degree	*Clostridium tertium*
anaerobic/37 degree	*Clostridium_bifermentans*
anaerobic/37 degree	*Clostridium_bifermentans*
anaerobic/37 degree	*Clostridium_bifermentans*
anaerobic/37 degree	*Clostridium_bifermentans*
anaerobic/37 degree	*Clostridium_sp_7_2_43FAA*
anaerobic/37 degree	*Clostridium_sp_7_2_43FAA*
anaerobic/37 degree	*Clostridium_sp_7_2_43FAA*
anaerobic/37 degree	*Clostridium_sp_7_2_43FAA*
anaerobic/37 degree	*Clostridium_sp_7_2_43FAA*
anaerobic/37 degree	*Clostridium_sp_7_2_43FAA*
anaerobic/37 degree	*Clostridium_sp_7_2_43FAA*
anaerobic/37 degree	*Clostridium_sp_7_2_43FAA*
anaerobic/37 degree	*Clostridium_sp_7_2_43FAA*
anaerobic/37 degree	*Collinsella aerofaciens*
anaerobic/37 degree	*Collinsella aerofaciens*
anaerobic/37 degree	*Collinsella aerofaciens*
anaerobic/37 degree	*Collinsella aerofaciens*
anaerobic/37 degree	*Collinsella aerofaciens*
anaerobic/37 degree	*Collinsella aerofaciens*
anaerobic/37 degree	*Collinsella aerofaciens*
anaerobic/37 degree	*Collinsella aerofaciens*
anaerobic/37 degree	*Collinsella aerofaciens*
anaerobic/37 degree	*Collinsella aerofaciens*
anaerobic/37 degree	*Collinsella aerofaciens*
anaerobic/37 degree	*Collinsella aerofaciens*
anaerobic/37 degree	*Collinsella aerofaciens*
anaerobic/37 degree	*Collinsella aerofaciens*
anaerobic/37 degree	*Collinsella aerofaciens*
anaerobic/37 degree	*Collinsella aerofaciens*
anaerobic/37 degree	*Collinsella aerofaciens*
anaerobic/37 degree	*Collinsella aerofaciens*

Table S5. The cultured strains and their culture atmospheres

**Atmosphere**	***species***
anaerobic/37 degree	*Collinsella aerofaciens*
anaerobic/37 degree	*Collinsella aerofaciens*
anaerobic/37 degree	*Collinsella aerofaciens*
anaerobic/37 degree	*Collinsella aerofaciens*
anaerobic/37 degree	*Collinsella aerofaciens*
anaerobic/37 degree	*Collinsella aerofaciens*
anaerobic/37 degree	*Collinsella aerofaciens*
anaerobic/37 degree	*Collinsella aerofaciens*
anaerobic/37 degree	*Collinsella aerofaciens*
anaerobic/37 degree	*Collinsella aerofaciens*
anaerobic/37 degree	*Collinsella aerofaciens*
anaerobic/37 degree	*Collinsella aerofaciens*
anaerobic/37 degree	*Collinsella aerofaciens*
anaerobic/37 degree	*Collinsella aerofaciens*
anaerobic/37 degree	*Collinsella aerofaciens*
anaerobic/37 degree	*Collinsella aerofaciens*
anaerobic/37 degree	*Collinsella aerofaciens*
anaerobic/37 degree	*Collinsella aerofaciens*
anaerobic/37 degree	*Collinsella aerofaciens*
anaerobic/37 degree	*Collinsella aerofaciens*
anaerobic/37 degree	*Collinsella aerofaciens*
anaerobic/37 degree	*Collinsella aerofaciens*
anaerobic/37 degree	*Collinsella aerofaciens*
anaerobic/37 degree	*Collinsella aerofaciens*
anaerobic/37 degree	*Collinsella aerofaciens*
anaerobic/37 degree	*Collinsella aerofaciens*
anaerobic/37 degree	*Collinsella aerofaciens*
anaerobic/37 degree	*Collinsella aerofaciens*
anaerobic/37 degree	*Collinsella aerofaciens*
anaerobic/37 degree	*Collinsella aerofaciens*
anaerobic/37 degree	*Collinsella aerofaciens*
anaerobic/37 degree	*Collinsella aerofaciens*
anaerobic/37 degree	*Collinsella aerofaciens*
anaerobic/37 degree	*Collinsella aerofaciens*
anaerobic/42 degree	*Collinsella aerofaciens*
anaerobic/42 degree	*Collinsella aerofaciens*
anaerobic/42 degree	*Collinsella aerofaciens*
anaerobic/42 degree	*Collinsella aerofaciens*
anaerobic/42 degree	*Collinsella aerofaciens*
anaerobic/42 degree	*Collinsella aerofaciens*
anaerobic/42 degree	*Collinsella aerofaciens*
anaerobic/42 degree	*Collinsella massiliensis*
anaerobic/37 degree	*Collinsella tanakaei*
anaerobic/37 degree	*Collinsella tanakaei*

Table S5. The cultured strains and their culture atmospheres

**Atmosphere**	***species***
anaerobic/37 degree	*Collinsella tanakaei*
anaerobic/37 degree	*Collinsella tanakaei*
anaerobic/37 degree	*Collinsella tanakaei*
anaerobic/37 degree	*Comamonas kerstersii*
anaerobic/37 degree	*Comamonas kerstersii*
anaerobic/37 degree	*Comamonas kerstersii*
anaerobic/37 degree	*Comamonas kerstersii*
anaerobic/37 degree	*Comamonas kerstersii*
anaerobic/37 degree	*Comamonas kerstersii*
anaerobic/37 degree	*Comamonas kerstersii*
anaerobic/37 degree	*Comamonas kerstersii*
anaerobic/37 degree	*Comamonas kerstersii*
anaerobic/37 degree	*Comamonas kerstersii*
anaerobic/37 degree	*Comamonas kerstersii*
anaerobic/37 degree	*Comamonas kerstersii*
anaerobic/37 degree	*Comamonas kerstersii*
anaerobic/37 degree	*Comamonas kerstersii*
anaerobic/37 degree	*Comamonas kerstersii*
anaerobic/37 degree	*Comamonas kerstersii*
anaerobic/37 degree	*Comamonas kerstersii*
anaerobic/37 degree	*Comamonas kerstersii*
anaerobic/37 degree	*Comamonas kerstersii*
anaerobic/37 degree	*Comamonas kerstersii*
anaerobic/37 degree	*Comamonas kerstersii*
anaerobic/37 degree	*Comamonas kerstersii*
anaerobic/37 degree	*Comamonas kerstersii*
anaerobic/37 degree	*Comamonas kerstersii*
anaerobic/37 degree	*Comamonas kerstersii*
anaerobic/37 degree	*Comamonas kerstersii*
anaerobic/37 degree	*Comamonas kerstersii*
anaerobic/37 degree	*Comamonas kerstersii*
anaerobic/37 degree	*Comamonas kerstersii*
anaerobic/37 degree	*Comamonas kerstersii*
anaerobic/37 degree	*Comamonas kerstersii*
anaerobic/37 degree	*Comamonas kerstersii*
anaerobic/37 degree	*Comamonas kerstersii*
anaerobic/37 degree	*Comamonas kerstersii*
anaerobic/37 degree	*Comamonas kerstersii*
anaerobic/37 degree	*Comamonas kerstersii*
anaerobic/37 degree	*Comamonas kerstersii*
anaerobic/37 degree	*Comamonas kerstersii*
anaerobic/37 degree	*Comamonas kerstersii*
anaerobic/37 degree	*Comamonas kerstersii*
anaerobic/37 degree	*Comamonas kerstersii*

Table S5. The cultured strains and their culture atmospheres

Atmosphere	*species*
anaerobic/37 degree	*Comamonas kerstersii*
anaerobic/37 degree	*Comamonas kerstersii*
anaerobic/37 degree	*Comamonas kerstersii*
anaerobic/37 degree	*Comamonas kerstersii*
anaerobic/37 degree	*Comamonas kerstersii*
anaerobic/37 degree	*Comamonas kerstersii*
anaerobic/42 degree	*Corynebacterium kroppenstedtii*
anaerobic/37 degree	*Cronobacter sakazakii*
anaerobic/42 degree	*Cronobacter sakazakii*
anaerobic/42 degree	*Cryptococcus gattii(Filobasidiella bacillispora)*
anaerobic/37 degree	*Dielma fastidiosa*
anaerobic/37 degree	*Dysgonomonas unclassified*
anaerobic/37 degree	*Eggerthella lenta*
anaerobic/37 degree	*Eggerthella lenta*
anaerobic/37 degree	*Eggerthella lenta*
anaerobic/37 degree	*Eggerthella lenta*
anaerobic/37 degree	*Eggerthella lenta*
anaerobic/37 degree	*Eggerthella lenta*
anaerobic/37 degree	*Eggerthella lenta*
anaerobic/37 degree	*Eggerthella lenta*
anaerobic/37 degree	*Eggerthella lenta*
anaerobic/37 degree	*Eggerthella lenta*
anaerobic/37 degree	*Eggerthella lenta*
anaerobic/37 degree	*Eggerthella lenta*
anaerobic/37 degree	*Eggerthella lenta*
anaerobic/37 degree	*Eggerthella lenta*
anaerobic/37 degree	*Eggerthella lenta*
anaerobic/37 degree	*Eggerthella lenta*
anaerobic/37 degree	*Eggerthella lenta*
anaerobic/37 degree	*Eggerthella lenta*
anaerobic/37 degree	*Eggerthella lenta*
anaerobic/42 degree	*Enorma timonensis*
anaerobic/42 degree	*Enorma timonensis*
anaerobic/42 degree	*Enorma timonensis*
anaerobic/37 degree	*Enterobacter aerogenes*
anaerobic/35 degree	*Enterobacter cloacae*
anaerobic/35 degree	*Enterobacter cloacae*
anaerobic/35 degree	*Enterobacter cloacae*
anaerobic/35 degree	*Enterobacter cloacae*
anaerobic/37 degree	*Enterobacter cloacae*
anaerobic/37 degree	*Enterobacter cloacae*
anaerobic/37 degree	*Enterobacter cloacae*
anaerobic/37 degree	*Enterobacter cloacae*

Table S5. The cultured strains and their culture atmospheres

**Atmosphere**	***species***
anaerobic/37 degree	*Enterobacter cowanii*
anaerobic/37 degree	*Enterobacter kobei*
anaerobic/37 degree	*Enterobacter kobei*
anaerobic/35 degree	*Enterobacter ludwigii*
anaerobic/37 degree	*Enterobacter ludwigii*
anaerobic/37 degree	*Enterobacter ludwigii*
anaerobic/37 degree	*Enterobacter ludwigii*
anaerobic/37 degree	*Enterobacter ludwigii*
anaerobic/37 degree	*Enterobacter ludwigii*
anaerobic/37 degree	*Enterobacter ludwigii*
anaerobic/37 degree	*Enterobacter ludwigii*
anaerobic/37 degree	*Enterobacter ludwigii*
anaerobic/42 degree	*Enterobacter ludwigii*
anaerobic/42 degree	*Enterobacter ludwigii*
anaerobic/37 degree	*Enterobacter roggenkampii*
anaerobic/37 degree	*Enterobacter_cloacae*
anaerobic/37 degree	*Enterobacter_cloacae*
anaerobic/37 degree	*Enterobacter_cloacae*
anaerobic/37 degree	*Enterobacter_cloacae*
anaerobic/42 degree	*Enterobacteriaceae bacterium 9_2_54FAA*
anaerobic/37 degree	*Enterococcus asini*
anaerobic/37 degree	*Enterococcus asini*
anaerobic/37 degree	*Enterococcus asini*
anaerobic/37 degree	*Enterococcus asini*
anaerobic/37 degree	*Enterococcus asini*
anaerobic/37 degree	*Enterococcus asini*
anaerobic/37 degree	*Enterococcus asini*
anaerobic/37 degree	*Enterococcus asini*
anaerobic/37 degree	*Enterococcus asini*
anaerobic/37 degree	*Enterococcus asini*
anaerobic/37 degree	*Enterococcus asini*
anaerobic/37 degree	*Enterococcus asini*
anaerobic/37 degree	*Enterococcus asini*
anaerobic/37 degree	*Enterococcus asini*
anaerobic/37 degree	*Enterococcus asini*
anaerobic/42 degree	*Enterococcus asini*
anaerobic/42 degree	*Enterococcus asini*
anaerobic/42 degree	*Enterococcus asini*
anaerobic/42 degree	*Enterococcus asini*
anaerobic/37 degree	*Enterococcus avium*
anaerobic/37 degree	*Enterococcus avium*
anaerobic/37 degree	*Enterococcus avium*
anaerobic/37 degree	*Enterococcus avium*
anaerobic/37 degree	*Enterococcus avium*

Table S5. The cultured strains and their culture atmospheres

**Atmosphere**	***species***
anaerobic/37 degree	*Enterococcus avium*
anaerobic/37 degree	*Enterococcus avium*
anaerobic/37 degree	*Enterococcus avium*
anaerobic/37 degree	*Enterococcus avium*
anaerobic/37 degree	*Enterococcus avium*
anaerobic/37 degree	*Enterococcus avium*
anaerobic/37 degree	*Enterococcus avium*
anaerobic/37 degree	*Enterococcus avium*
anaerobic/37 degree	*Enterococcus avium*
anaerobic/37 degree	*Enterococcus avium*
anaerobic/37 degree	*Enterococcus avium*
anaerobic/40-50 degree	*Enterococcus avium*
anaerobic/42 degree	*Enterococcus avium*
anaerobic/42 degree	*Enterococcus avium*
anaerobic/42 degree	*Enterococcus avium*
anaerobic/42 degree	*Enterococcus avium*
anaerobic/42 degree	*Enterococcus avium*
anaerobic/42 degree	*Enterococcus avium*
anaerobic/42 degree	*Enterococcus avium*
anaerobic/42 degree	*Enterococcus avium*
anaerobic/42 degree	*Enterococcus avium*
anaerobic/42 degree	*Enterococcus avium*
anaerobic/37 degree	*Enterococcus canintestini*
anaerobic/37 degree	*Enterococcus canintestini*
anaerobic/35 degree	*Enterococcus casseliflavus*
anaerobic/35 degree	*Enterococcus casseliflavus*
anaerobic/37 degree	*Enterococcus casseliflavus*
anaerobic/37 degree	*Enterococcus casseliflavus*
anaerobic/37 degree	*Enterococcus casseliflavus*
anaerobic/37 degree	*Enterococcus casseliflavus*
anaerobic/37 degree	*Enterococcus casseliflavus*
anaerobic/37 degree	*Enterococcus casseliflavus*
anaerobic/37 degree	*Enterococcus casseliflavus*
anaerobic/37 degree	*Enterococcus casseliflavus*
anaerobic/37 degree	*Enterococcus casseliflavus*
anaerobic/37 degree	*Enterococcus casseliflavus*
anaerobic/37 degree	*Enterococcus casseliflavus*
anaerobic/37 degree	*Enterococcus casseliflavus*
anaerobic/37 degree	*Enterococcus casseliflavus*
anaerobic/37 degree	*Enterococcus casseliflavus*
anaerobic/37 degree	*Enterococcus casseliflavus*
anaerobic/37 degree	*Enterococcus casseliflavus*
anaerobic/37 degree	*Enterococcus casseliflavus*
anaerobic/37 degree	*Enterococcus casseliflavus*
anaerobic/37 degree	*Enterococcus casseliflavus*

Table S5. The cultured strains and their culture atmospheres

**Atmosphere**	***species***
anaerobic/42 degree	*Enterococcus casseliflavus*
anaerobic/42 degree	*Enterococcus casseliflavus*
anaerobic/42 degree	*Enterococcus casseliflavus*
anaerobic/42 degree	*Enterococcus casseliflavus*
anaerobic/42 degree	*Enterococcus casseliflavus*
anaerobic/42 degree	*Enterococcus casseliflavus*
anaerobic/37 degree	*Enterococcus cecorum*
anaerobic/37 degree	*Enterococcus cecorum*
anaerobic/37 degree	*Enterococcus cecorum*
anaerobic/37 degree	*Enterococcus cecorum*
anaerobic/37 degree	*Enterococcus cecorum*
anaerobic/37 degree	*Enterococcus cecorum*
anaerobic/37 degree	*Enterococcus cecorum*
anaerobic/37 degree	*Enterococcus cecorum*
anaerobic/37 degree	*Enterococcus cecorum*
anaerobic/37 degree	*Enterococcus cecorum*
anaerobic/37 degree	*Enterococcus cecorum*
anaerobic/37 degree	*Enterococcus cecorum*
anaerobic/37 degree	*Enterococcus cecorum*
anaerobic/37 degree	*Enterococcus cecorum*
anaerobic/37 degree	*Enterococcus cecorum*
anaerobic/37 degree	*Enterococcus cecorum*
anaerobic/37 degree	*Enterococcus cecorum*
anaerobic/37 degree	*Enterococcus cecorum*
anaerobic/40-50 degree	*Enterococcus cecorum*
anaerobic/40-50 degree	*Enterococcus cecorum*
anaerobic/40-50 degree	*Enterococcus cecorum*
anaerobic/40-50 degree	*Enterococcus cecorum*
anaerobic/40-50 degree	*Enterococcus cecorum*
anaerobic/40-50 degree	*Enterococcus cecorum*
anaerobic/40-50 degree	*Enterococcus cecorum*
anaerobic/40-50 degree	*Enterococcus cecorum*
anaerobic/40-50 degree	*Enterococcus cecorum*
anaerobic/40-50 degree	*Enterococcus cecorum*
anaerobic/40-50 degree	*Enterococcus cecorum*
anaerobic/40-50 degree	*Enterococcus cecorum*
anaerobic/40-50 degree	*Enterococcus cecorum*
anaerobic/40-50 degree	*Enterococcus cecorum*
anaerobic/40-50 degree	*Enterococcus cecorum*
anaerobic/42 degree	*Enterococcus cecorum*
anaerobic/42 degree	*Enterococcus cecorum*
anaerobic/42 degree	*Enterococcus cecorum*
anaerobic/42 degree	*Enterococcus cecorum*
anaerobic/42 degree	*Enterococcus cecorum*

Table S5. The cultured strains and their culture atmospheres

**Atmosphere**	***species***
anaerobic/37 degree	*Enterococcus dispar*
anaerobic/37 degree	*Enterococcus dispar*
anaerobic/37 degree	*Enterococcus dispar*
anaerobic/37 degree	*Enterococcus dispar*
anaerobic/37 degree	*Enterococcus dispar*
anaerobic/37 degree	*Enterococcus dispar*
anaerobic/37 degree	*Enterococcus dispar*
anaerobic/37 degree	*Enterococcus dispar*
anaerobic/37 degree	*Enterococcus dispar*
anaerobic/37 degree	*Enterococcus dispar*
anaerobic/37 degree	*Enterococcus dispar*
anaerobic/37 degree	*Enterococcus dispar*
anaerobic/37 degree	*Enterococcus dispar*
anaerobic/37 degree	*Enterococcus dispar*
anaerobic/37 degree	*Enterococcus dispar*
anaerobic/37 degree	*Enterococcus dispar*
anaerobic/37 degree	*Enterococcus dispar*
anaerobic/37 degree	*Enterococcus dispar*
anaerobic/37 degree	*Enterococcus dispar*
anaerobic/37 degree	*Enterococcus dispar*
anaerobic/37 degree	*Enterococcus dispar*
anaerobic/37 degree	*Enterococcus dispar*
anaerobic/37 degree	*Enterococcus dispar*
anaerobic/37 degree	*Enterococcus dispar*
anaerobic/37 degree	*Enterococcus dispar*
anaerobic/37 degree	*Enterococcus dispar*
anaerobic/37 degree	*Enterococcus dispar*
anaerobic/37 degree	*Enterococcus dispar*
anaerobic/37 degree	*Enterococcus dispar*
anaerobic/37 degree	*Enterococcus dispar*
anaerobic/37 degree	*Enterococcus dispar*
anaerobic/37 degree	*Enterococcus dispar*
anaerobic/37 degree	*Enterococcus dispar*
anaerobic/37 degree	*Enterococcus dispar*
anaerobic/37 degree	*Enterococcus dispar*
anaerobic/37 degree	*Enterococcus dispar*
anaerobic/37 degree	*Enterococcus dispar*
anaerobic/37 degree	*Enterococcus dispar*
anaerobic/37 degree	*Enterococcus dispar*
anaerobic/37 degree	*Enterococcus dispar*
anaerobic/37 degree	*Enterococcus dispar*
anaerobic/37 degree	*Enterococcus dispar*
anaerobic/37 degree	*Enterococcus dispar*
anaerobic/37 degree	*Enterococcus dispar*

Table S5. The cultured strains and their culture atmospheres

**Atmosphere**	***species***
anaerobic/37 degree	*Enterococcus dispar*
anaerobic/37 degree	*Enterococcus dispar*
anaerobic/37 degree	*Enterococcus dispar*
anaerobic/37 degree	*Enterococcus dispar*
anaerobic/37 degree	*Enterococcus dispar*
anaerobic/37 degree	*Enterococcus dispar*
anaerobic/37 degree	*Enterococcus dispar*
anaerobic/37 degree	*Enterococcus dispar*
anaerobic/37 degree	*Enterococcus dispar*
anaerobic/37 degree	*Enterococcus dispar*
anaerobic/37 degree	*Enterococcus dispar*
anaerobic/37 degree	*Enterococcus dispar*
anaerobic/37 degree	*Enterococcus dispar*
anaerobic/37 degree	*Enterococcus dispar*
anaerobic/37 degree	*Enterococcus dispar*
anaerobic/37 degree	*Enterococcus dispar*
anaerobic/37 degree	*Enterococcus dispar*
anaerobic/37 degree	*Enterococcus dispar*
anaerobic/37 degree	*Enterococcus dispar*
anaerobic/37 degree	*Enterococcus dispar*
anaerobic/37 degree	*Enterococcus dispar*
anaerobic/37 degree	*Enterococcus dispar*
anaerobic/37 degree	*Enterococcus dispar*
anaerobic/37 degree	*Enterococcus dispar*
anaerobic/37 degree	*Enterococcus dispar*
anaerobic/37 degree	*Enterococcus dispar*
anaerobic/37 degree	*Enterococcus dispar*
anaerobic/37 degree	*Enterococcus dispar*
anaerobic/37 degree	*Enterococcus dispar*
anaerobic/37 degree	*Enterococcus dispar*
anaerobic/37 degree	*Enterococcus dispar*
anaerobic/37 degree	*Enterococcus dispar*
anaerobic/37 degree	*Enterococcus dispar*
anaerobic/37 degree	*Enterococcus dispar*
anaerobic/37 degree	*Enterococcus dispar*
anaerobic/37 degree	*Enterococcus dispar*
anaerobic/37 degree	*Enterococcus dispar*
anaerobic/37 degree	*Enterococcus dispar*
anaerobic/37 degree	*Enterococcus dispar*
anaerobic/37 degree	*Enterococcus dispar*
anaerobic/37 degree	*Enterococcus dispar*
anaerobic/37 degree	*Enterococcus dispar*
anaerobic/37 degree	*Enterococcus dispar*
anaerobic/37 degree	*Enterococcus dispar*

Table S5. The cultured strains and their culture atmospheres

**Atmosphere**	***species***
anaerobic/37 degree	*Enterococcus dispar*
anaerobic/37 degree	*Enterococcus dispar*
anaerobic/37 degree	*Enterococcus dispar*
anaerobic/37 degree	*Enterococcus dispar*
anaerobic/37 degree	*Enterococcus dispar*
anaerobic/37 degree	*Enterococcus dispar*
anaerobic/37 degree	*Enterococcus dispar*
anaerobic/37 degree	*Enterococcus dispar*
anaerobic/37 degree	*Enterococcus dispar*
anaerobic/37 degree	*Enterococcus dispar*
anaerobic/37 degree	*Enterococcus dispar*
anaerobic/37 degree	*Enterococcus dispar*
anaerobic/37 degree	*Enterococcus dispar*
anaerobic/37 degree	*Enterococcus dispar*
anaerobic/37 degree	*Enterococcus dispar*
anaerobic/37 degree	*Enterococcus dispar*
anaerobic/37 degree	*Enterococcus dispar*
anaerobic/37 degree	*Enterococcus dispar*
anaerobic/37 degree	*Enterococcus dispar*
anaerobic/37 degree	*Enterococcus dispar*
anaerobic/37 degree	*Enterococcus dispar*
anaerobic/37 degree	*Enterococcus dispar*
anaerobic/37 degree	*Enterococcus dispar*
anaerobic/37 degree	*Enterococcus dispar*
anaerobic/37 degree	*Enterococcus dispar*
anaerobic/37 degree	*Enterococcus dispar*
anaerobic/37 degree	*Enterococcus dispar*
anaerobic/37 degree	*Enterococcus dispar*
anaerobic/37 degree	*Enterococcus dispar*
anaerobic/37 degree	*Enterococcus dispar*
anaerobic/37 degree	*Enterococcus dispar*
anaerobic/37 degree	*Enterococcus dispar*
anaerobic/37 degree	*Enterococcus dispar*
anaerobic/37 degree	*Enterococcus dispar*
anaerobic/37 degree	*Enterococcus dispar*
anaerobic/37 degree	*Enterococcus dispar*
anaerobic/37 degree	*Enterococcus dispar*
anaerobic/37 degree	*Enterococcus dispar*
anaerobic/37 degree	*Enterococcus dispar*
anaerobic/37 degree	*Enterococcus dispar*
anaerobic/37 degree	*Enterococcus dispar*
anaerobic/37 degree	*Enterococcus dispar*
anaerobic/37 degree	*Enterococcus dispar*
anaerobic/37 degree	*Enterococcus dispar*

Table S5. The cultured strains and their culture atmospheres

Atmosphere	*species*
anaerobic/37 degree	*Enterococcus dispar*
anaerobic/37 degree	*Enterococcus dispar*
anaerobic/37 degree	*Enterococcus dispar*
anaerobic/37 degree	*Enterococcus dispar*
anaerobic/37 degree	*Enterococcus dispar*
anaerobic/37 degree	*Enterococcus dispar*
anaerobic/37 degree	*Enterococcus dispar*
anaerobic/37 degree	*Enterococcus dispar*
anaerobic/37 degree	*Enterococcus dispar*
anaerobic/37 degree	*Enterococcus dispar*
anaerobic/37 degree	*Enterococcus dispar*
anaerobic/37 degree	*Enterococcus dispar*
anaerobic/37 degree	*Enterococcus dispar*
anaerobic/37 degree	*Enterococcus dispar*
anaerobic/37 degree	*Enterococcus dispar*
anaerobic/37 degree	*Enterococcus dispar*
anaerobic/37 degree	*Enterococcus dispar*
anaerobic/37 degree	*Enterococcus dispar*
anaerobic/37 degree	*Enterococcus dispar*
anaerobic/37 degree	*Enterococcus dispar*
anaerobic/37 degree	*Enterococcus dispar*
anaerobic/42 degree	*Enterococcus dispar*
anaerobic/42 degree	*Enterococcus dispar*
anaerobic/37 degree	*Enterococcus durans*
anaerobic/37 degree	*Enterococcus durans*
anaerobic/37 degree	*Enterococcus durans*
anaerobic/35 degree	*Enterococcus faecalis*
anaerobic/35 degree	*Enterococcus faecalis*
anaerobic/35 degree	*Enterococcus faecalis*
anaerobic/35 degree	*Enterococcus faecalis*
anaerobic/37 degree	*Enterococcus faecalis*
anaerobic/37 degree	*Enterococcus faecalis*
anaerobic/37 degree	*Enterococcus faecalis*
anaerobic/37 degree	*Enterococcus faecalis*
anaerobic/37 degree	*Enterococcus faecalis*
anaerobic/37 degree	*Enterococcus faecalis*
anaerobic/37 degree	*Enterococcus faecalis*
anaerobic/37 degree	*Enterococcus faecalis*
anaerobic/37 degree	*Enterococcus faecalis*
anaerobic/37 degree	*Enterococcus faecalis*
anaerobic/37 degree	*Enterococcus faecalis*
anaerobic/37 degree	*Enterococcus faecalis*
anaerobic/37 degree	*Enterococcus faecalis*
anaerobic/37 degree	*Enterococcus faecalis*

Table S5. The cultured strains and their culture atmospheres

**Atmosphere**	***species***
anaerobic/37 degree	*Enterococcus faecalis*
anaerobic/37 degree	*Enterococcus faecalis*
anaerobic/37 degree	*Enterococcus faecalis*
anaerobic/37 degree	*Enterococcus faecalis*
anaerobic/37 degree	*Enterococcus faecalis*
anaerobic/37 degree	*Enterococcus faecalis*
anaerobic/37 degree	*Enterococcus faecalis*
anaerobic/37 degree	*Enterococcus faecalis*
anaerobic/37 degree	*Enterococcus faecalis*
anaerobic/37 degree	*Enterococcus faecalis*
anaerobic/37 degree	*Enterococcus faecalis*
anaerobic/37 degree	*Enterococcus faecalis*
anaerobic/37 degree	*Enterococcus faecalis*
anaerobic/37 degree	*Enterococcus faecalis*
anaerobic/37 degree	*Enterococcus faecalis*
anaerobic/37 degree	*Enterococcus faecalis*
anaerobic/37 degree	*Enterococcus faecalis*
anaerobic/37 degree	*Enterococcus faecalis*
anaerobic/37 degree	*Enterococcus faecalis*
anaerobic/37 degree	*Enterococcus faecalis*
anaerobic/37 degree	*Enterococcus faecalis*
anaerobic/37 degree	*Enterococcus faecalis*
anaerobic/37 degree	*Enterococcus faecalis*
anaerobic/37 degree	*Enterococcus faecalis*
anaerobic/37 degree	*Enterococcus faecalis*
anaerobic/37 degree	*Enterococcus faecalis*
anaerobic/37 degree	*Enterococcus faecalis*
anaerobic/37 degree	*Enterococcus faecalis*
anaerobic/37 degree	*Enterococcus faecalis*
anaerobic/37 degree	*Enterococcus faecalis*
anaerobic/37 degree	*Enterococcus faecalis*
anaerobic/37 degree	*Enterococcus faecalis*
anaerobic/37 degree	*Enterococcus faecalis*
anaerobic/37 degree	*Enterococcus faecalis*
anaerobic/37 degree	*Enterococcus faecalis*
anaerobic/37 degree	*Enterococcus faecalis*
anaerobic/37 degree	*Enterococcus faecalis*
anaerobic/37 degree	*Enterococcus faecalis*
anaerobic/37 degree	*Enterococcus faecalis*
anaerobic/37 degree	*Enterococcus faecalis*
anaerobic/37 degree	*Enterococcus faecalis*
anaerobic/37 degree	*Enterococcus faecalis*
anaerobic/37 degree	*Enterococcus faecalis*
anaerobic/37 degree	*Enterococcus faecalis*

Table S5. The cultured strains and their culture atmospheres

**Atmosphere**	***species***
anaerobic/37 degree	*Enterococcus faecalis*
anaerobic/37 degree	*Enterococcus faecalis*
anaerobic/37 degree	*Enterococcus faecalis*
anaerobic/37 degree	*Enterococcus faecalis*
anaerobic/37 degree	*Enterococcus faecalis*
anaerobic/37 degree	*Enterococcus faecalis*
anaerobic/37 degree	*Enterococcus faecalis*
anaerobic/37 degree	*Enterococcus faecalis*
anaerobic/37 degree	*Enterococcus faecalis*
anaerobic/37 degree	*Enterococcus faecalis*
anaerobic/37 degree	*Enterococcus faecalis*
anaerobic/37 degree	*Enterococcus faecalis*
anaerobic/37 degree	*Enterococcus faecalis*
anaerobic/42 degree	*Enterococcus faecalis*
anaerobic/42 degree	*Enterococcus faecalis*
anaerobic/42 degree	*Enterococcus faecalis*
anaerobic/42 degree	*Enterococcus faecalis*
anaerobic/42 degree	*Enterococcus faecalis*
anaerobic/42 degree	*Enterococcus faecalis*
anaerobic/42 degree	*Enterococcus faecalis*
anaerobic/42 degree	*Enterococcus faecalis*
anaerobic/42 degree	*Enterococcus faecalis*
anaerobic/42 degree	*Enterococcus faecalis*
anaerobic/42 degree	*Enterococcus faecalis*
anaerobic/42 degree	*Enterococcus faecalis*
anaerobic/42 degree	*Enterococcus faecalis*
anaerobic/42 degree	*Enterococcus faecalis*
anaerobic/42 degree	*Enterococcus faecalis*
anaerobic/42 degree	*Enterococcus faecalis*
anaerobic/42 degree	*Enterococcus faecalis*
anaerobic/42 degree	*Enterococcus faecalis*
anaerobic/42 degree	*Enterococcus faecalis*
anaerobic/42 degree	*Enterococcus faecalis*
anaerobic/42 degree	*Enterococcus faecalis*
anaerobic/42 degree	*Enterococcus faecalis*
anaerobic/42 degree	*Enterococcus faecalis*
anaerobic/42 degree	*Enterococcus faecalis*
anaerobic/42 degree	*Enterococcus faecalis*
anaerobic/42 degree	*Enterococcus faecalis*
anaerobic/42 degree	*Enterococcus faecalis*
anaerobic/42 degree	*Enterococcus faecalis*
anaerobic/42 degree	*Enterococcus faecalis*
anaerobic/42 degree	*Enterococcus faecalis*
anaerobic/42 degree	*Enterococcus faecalis*
anaerobic/42 degree	*Enterococcus faecalis*

Table S5. The cultured strains and their culture atmospheres

**Atmosphere**	***species***
anaerobic/42 degree	*Enterococcus faecalis*
anaerobic/42 degree	*Enterococcus faecalis*
anaerobic/42 degree	*Enterococcus faecalis*
anaerobic/42 degree	*Enterococcus faecalis*
anaerobic/42 degree	*Enterococcus faecalis*
anaerobic/42 degree	*Enterococcus faecalis*
anaerobic/42 degree	*Enterococcus faecalis*
anaerobic/35 degree	*Enterococcus faecium*
anaerobic/35 degree	*Enterococcus faecium*
anaerobic/35 degree	*Enterococcus faecium*
anaerobic/35 degree	*Enterococcus faecium*
anaerobic/35 degree	*Enterococcus faecium*
anaerobic/37 degree	*Enterococcus faecium*
anaerobic/37 degree	*Enterococcus faecium*
anaerobic/37 degree	*Enterococcus faecium*
anaerobic/37 degree	*Enterococcus faecium*
anaerobic/37 degree	*Enterococcus faecium*
anaerobic/37 degree	*Enterococcus faecium*
anaerobic/37 degree	*Enterococcus faecium*
anaerobic/37 degree	*Enterococcus faecium*
anaerobic/37 degree	*Enterococcus faecium*
anaerobic/37 degree	*Enterococcus faecium*
anaerobic/37 degree	*Enterococcus faecium*
anaerobic/37 degree	*Enterococcus faecium*
anaerobic/37 degree	*Enterococcus faecium*
anaerobic/37 degree	*Enterococcus faecium*
anaerobic/37 degree	*Enterococcus faecium*
anaerobic/37 degree	*Enterococcus faecium*
anaerobic/37 degree	*Enterococcus faecium*
anaerobic/37 degree	*Enterococcus faecium*
anaerobic/37 degree	*Enterococcus faecium*
anaerobic/37 degree	*Enterococcus faecium*
anaerobic/37 degree	*Enterococcus faecium*
anaerobic/37 degree	*Enterococcus faecium*
anaerobic/37 degree	*Enterococcus faecium*
anaerobic/37 degree	*Enterococcus faecium*
anaerobic/37 degree	*Enterococcus faecium*
anaerobic/37 degree	*Enterococcus faecium*
anaerobic/37 degree	*Enterococcus faecium*
anaerobic/37 degree	*Enterococcus faecium*
anaerobic/37 degree	*Enterococcus faecium*
anaerobic/37 degree	*Enterococcus faecium*
anaerobic/37 degree	*Enterococcus faecium*
anaerobic/37 degree	*Enterococcus faecium*
anaerobic/37 degree	*Enterococcus faecium*

Table S5. The cultured strains and their culture atmospheres

**Atmosphere**	***species***
anaerobic/37 degree	*Enterococcus faecium*
anaerobic/37 degree	*Enterococcus faecium*
anaerobic/37 degree	*Enterococcus faecium*
anaerobic/37 degree	*Enterococcus faecium*
anaerobic/37 degree	*Enterococcus faecium*
anaerobic/37 degree	*Enterococcus faecium*
anaerobic/37 degree	*Enterococcus faecium*
anaerobic/37 degree	*Enterococcus faecium*
anaerobic/37 degree	*Enterococcus faecium*
anaerobic/37 degree	*Enterococcus faecium*
anaerobic/37 degree	*Enterococcus faecium*
anaerobic/37 degree	*Enterococcus faecium*
anaerobic/37 degree	*Enterococcus faecium*
anaerobic/37 degree	*Enterococcus faecium*
anaerobic/37 degree	*Enterococcus faecium*
anaerobic/37 degree	*Enterococcus faecium*
anaerobic/37 degree	*Enterococcus faecium*
anaerobic/37 degree	*Enterococcus faecium*
anaerobic/37 degree	*Enterococcus faecium*
anaerobic/37 degree	*Enterococcus faecium*
anaerobic/37 degree	*Enterococcus faecium*
anaerobic/37 degree	*Enterococcus faecium*
anaerobic/37 degree	*Enterococcus faecium*
anaerobic/37 degree	*Enterococcus faecium*
anaerobic/37 degree	*Enterococcus faecium*
anaerobic/37 degree	*Enterococcus faecium*
anaerobic/37 degree	*Enterococcus faecium*
anaerobic/37 degree	*Enterococcus faecium*
anaerobic/37 degree	*Enterococcus faecium*
anaerobic/37 degree	*Enterococcus faecium*
anaerobic/37 degree	*Enterococcus faecium*
anaerobic/37 degree	*Enterococcus faecium*
anaerobic/37 degree	*Enterococcus faecium*
anaerobic/37 degree	*Enterococcus faecium*
anaerobic/37 degree	*Enterococcus faecium*
anaerobic/37 degree	*Enterococcus faecium*
anaerobic/37 degree	*Enterococcus faecium*
anaerobic/37 degree	*Enterococcus faecium*
anaerobic/37 degree	*Enterococcus faecium*
anaerobic/37 degree	*Enterococcus faecium*
anaerobic/37 degree	*Enterococcus faecium*
anaerobic/37 degree	*Enterococcus faecium*
anaerobic/37 degree	*Enterococcus faecium*
anaerobic/37 degree	*Enterococcus faecium*

Table S5. The cultured strains and their culture atmospheres

**Atmosphere**	***species***
anaerobic/37 degree	*Enterococcus faecium*
anaerobic/37 degree	*Enterococcus faecium*
anaerobic/37 degree	*Enterococcus faecium*
anaerobic/37 degree	*Enterococcus faecium*
anaerobic/37 degree	*Enterococcus faecium*
anaerobic/37 degree	*Enterococcus faecium*
anaerobic/37 degree	*Enterococcus faecium*
anaerobic/37 degree	*Enterococcus faecium*
anaerobic/37 degree	*Enterococcus faecium*
anaerobic/37 degree	*Enterococcus faecium*
anaerobic/37 degree	*Enterococcus faecium*
anaerobic/37 degree	*Enterococcus faecium*
anaerobic/37 degree	*Enterococcus faecium*
anaerobic/37 degree	*Enterococcus faecium*
anaerobic/37 degree	*Enterococcus faecium*
anaerobic/37 degree	*Enterococcus faecium*
anaerobic/37 degree	*Enterococcus faecium*
anaerobic/37 degree	*Enterococcus faecium*
anaerobic/37 degree	*Enterococcus faecium*
anaerobic/37 degree	*Enterococcus faecium*
anaerobic/37 degree	*Enterococcus faecium*
anaerobic/37 degree	*Enterococcus faecium*
anaerobic/37 degree	*Enterococcus faecium*
anaerobic/37 degree	*Enterococcus faecium*
anaerobic/37 degree	*Enterococcus faecium*
anaerobic/37 degree	*Enterococcus faecium*
anaerobic/37 degree	*Enterococcus faecium*
anaerobic/37 degree	*Enterococcus faecium*
anaerobic/37 degree	*Enterococcus faecium*
anaerobic/37 degree	*Enterococcus faecium*
anaerobic/37 degree	*Enterococcus faecium*
anaerobic/37 degree	*Enterococcus faecium*
anaerobic/37 degree	*Enterococcus faecium*
anaerobic/37 degree	*Enterococcus faecium*
anaerobic/37 degree	*Enterococcus faecium*
anaerobic/37 degree	*Enterococcus faecium*
anaerobic/37 degree	*Enterococcus faecium*
anaerobic/37 degree	*Enterococcus faecium*
anaerobic/37 degree	*Enterococcus faecium*
anaerobic/37 degree	*Enterococcus faecium*
anaerobic/37 degree	*Enterococcus faecium*
anaerobic/37 degree	*Enterococcus faecium*
anaerobic/37 degree	*Enterococcus faecium*
anaerobic/37 degree	*Enterococcus faecium*

Table S5. The cultured strains and their culture atmospheres

**Atmosphere**	***species***
anaerobic/37 degree	*Enterococcus faecium*
anaerobic/37 degree	*Enterococcus faecium*
anaerobic/37 degree	*Enterococcus faecium*
anaerobic/37 degree	*Enterococcus faecium*
anaerobic/37 degree	*Enterococcus faecium*
anaerobic/37 degree	*Enterococcus faecium*
anaerobic/37 degree	*Enterococcus faecium*
anaerobic/37 degree	*Enterococcus faecium*
anaerobic/37 degree	*Enterococcus faecium*
anaerobic/37 degree	*Enterococcus faecium*
anaerobic/37 degree	*Enterococcus faecium*
anaerobic/37 degree	*Enterococcus faecium*
anaerobic/37 degree	*Enterococcus faecium*
anaerobic/37 degree	*Enterococcus faecium*
anaerobic/37 degree	*Enterococcus faecium*
anaerobic/37 degree	*Enterococcus faecium*
anaerobic/37 degree	*Enterococcus faecium*
anaerobic/37 degree	*Enterococcus faecium*
anaerobic/37 degree	*Enterococcus faecium*
anaerobic/37 degree	*Enterococcus faecium*
anaerobic/37 degree	*Enterococcus faecium*
anaerobic/37 degree	*Enterococcus faecium*
anaerobic/37 degree	*Enterococcus faecium*
anaerobic/37 degree	*Enterococcus faecium*
anaerobic/37 degree	*Enterococcus faecium*
anaerobic/37 degree	*Enterococcus faecium*
anaerobic/37 degree	*Enterococcus faecium*
anaerobic/37 degree	*Enterococcus faecium*
anaerobic/37 degree	*Enterococcus faecium*
anaerobic/37 degree	*Enterococcus faecium*
anaerobic/37 degree	*Enterococcus faecium*
anaerobic/37 degree	*Enterococcus faecium*
anaerobic/37 degree	*Enterococcus faecium*
anaerobic/37 degree	*Enterococcus faecium*
anaerobic/37 degree	*Enterococcus faecium*
anaerobic/37 degree	*Enterococcus faecium*
anaerobic/37 degree	*Enterococcus faecium*
anaerobic/37 degree	*Enterococcus faecium*
anaerobic/37 degree	*Enterococcus faecium*
anaerobic/37 degree	*Enterococcus faecium*
anaerobic/37 degree	*Enterococcus faecium*
anaerobic/37 degree	*Enterococcus faecium*
anaerobic/37 degree	*Enterococcus faecium*
anaerobic/37 degree	*Enterococcus faecium*

Table S5. The cultured strains and their culture atmospheres

**Atmosphere**	***species***
anaerobic/37 degree	*Enterococcus faecium*
anaerobic/37 degree	*Enterococcus faecium*
anaerobic/37 degree	*Enterococcus faecium*
anaerobic/37 degree	*Enterococcus faecium*
anaerobic/37 degree	*Enterococcus faecium*
anaerobic/37 degree	*Enterococcus faecium*
anaerobic/37 degree	*Enterococcus faecium*
anaerobic/37 degree	*Enterococcus faecium*
anaerobic/37 degree	*Enterococcus faecium*
anaerobic/37 degree	*Enterococcus faecium*
anaerobic/37 degree	*Enterococcus faecium*
anaerobic/37 degree	*Enterococcus faecium*
anaerobic/37 degree	*Enterococcus faecium*
anaerobic/37 degree	*Enterococcus faecium*
anaerobic/37 degree	*Enterococcus faecium*
anaerobic/37 degree	*Enterococcus faecium*
anaerobic/37 degree	*Enterococcus faecium*
anaerobic/37 degree	*Enterococcus faecium*
anaerobic/37 degree	*Enterococcus faecium*
anaerobic/37 degree	*Enterococcus faecium*
anaerobic/37 degree	*Enterococcus faecium*
anaerobic/37 degree	*Enterococcus faecium*
anaerobic/37 degree	*Enterococcus faecium*
anaerobic/37 degree	*Enterococcus faecium*
anaerobic/37 degree	*Enterococcus faecium*
anaerobic/37 degree	*Enterococcus faecium*
anaerobic/37 degree	*Enterococcus faecium*
anaerobic/37 degree	*Enterococcus faecium*
anaerobic/37 degree	*Enterococcus faecium*
anaerobic/37 degree	*Enterococcus faecium*
anaerobic/37 degree	*Enterococcus faecium*
anaerobic/37 degree	*Enterococcus faecium*
anaerobic/37 degree	*Enterococcus faecium*
anaerobic/37 degree	*Enterococcus faecium*
anaerobic/37 degree	*Enterococcus faecium*
anaerobic/37 degree	*Enterococcus faecium*
anaerobic/37 degree	*Enterococcus faecium*
anaerobic/37 degree	*Enterococcus faecium*
anaerobic/37 degree	*Enterococcus faecium*
anaerobic/37 degree	*Enterococcus faecium*
anaerobic/37 degree	*Enterococcus faecium*
anaerobic/37 degree	*Enterococcus faecium*
anaerobic/37 degree	*Enterococcus faecium*
anaerobic/37 degree	*Enterococcus faecium*

Table S5. The cultured strains and their culture atmospheres

**Atmosphere**	***species***
anaerobic/37 degree	*Enterococcus faecium*
anaerobic/37 degree	*Enterococcus faecium*
anaerobic/37 degree	*Enterococcus faecium*
anaerobic/37 degree	*Enterococcus faecium*
anaerobic/37 degree	*Enterococcus faecium*
anaerobic/37 degree	*Enterococcus faecium*
anaerobic/37 degree	*Enterococcus faecium*
anaerobic/37 degree	*Enterococcus faecium*
anaerobic/37 degree	*Enterococcus faecium*
anaerobic/37 degree	*Enterococcus faecium*
anaerobic/37 degree	*Enterococcus faecium*
anaerobic/37 degree	*Enterococcus faecium*
anaerobic/37 degree	*Enterococcus faecium*
anaerobic/37 degree	*Enterococcus faecium*
anaerobic/37 degree	*Enterococcus faecium*
anaerobic/37 degree	*Enterococcus faecium*
anaerobic/37 degree	*Enterococcus faecium*
anaerobic/37 degree	*Enterococcus faecium*
anaerobic/37 degree	*Enterococcus faecium*
anaerobic/37 degree	*Enterococcus faecium*
anaerobic/37 degree	*Enterococcus faecium*
anaerobic/37 degree	*Enterococcus faecium*
anaerobic/37 degree	*Enterococcus faecium*
anaerobic/37 degree	*Enterococcus faecium*
anaerobic/37 degree	*Enterococcus faecium*
anaerobic/37 degree	*Enterococcus faecium*
anaerobic/37 degree	*Enterococcus faecium*
anaerobic/37 degree	*Enterococcus faecium*
anaerobic/37 degree	*Enterococcus faecium*
anaerobic/37 degree	*Enterococcus faecium*
anaerobic/37 degree	*Enterococcus faecium*
anaerobic/37 degree	*Enterococcus faecium*
anaerobic/37 degree	*Enterococcus faecium*
anaerobic/37 degree	*Enterococcus faecium*
anaerobic/37 degree	*Enterococcus faecium*
anaerobic/37 degree	*Enterococcus faecium*
anaerobic/37 degree	*Enterococcus faecium*
anaerobic/37 degree	*Enterococcus faecium*
anaerobic/37 degree	*Enterococcus faecium*
anaerobic/37 degree	*Enterococcus faecium*
anaerobic/37 degree	*Enterococcus faecium*
anaerobic/37 degree	*Enterococcus faecium*
anaerobic/37 degree	*Enterococcus faecium*
anaerobic/37 degree	*Enterococcus faecium*

Table S5. The cultured strains and their culture atmospheres

**Atmosphere**	***species***
anaerobic/37 degree	*Enterococcus faecium*
anaerobic/37 degree	*Enterococcus faecium*
anaerobic/37 degree	*Enterococcus faecium*
anaerobic/37 degree	*Enterococcus faecium*
anaerobic/37 degree	*Enterococcus faecium*
anaerobic/37 degree	*Enterococcus faecium*
anaerobic/37 degree	*Enterococcus faecium*
anaerobic/37 degree	*Enterococcus faecium*
anaerobic/37 degree	*Enterococcus faecium*
anaerobic/37 degree	*Enterococcus faecium*
anaerobic/37 degree	*Enterococcus faecium*
anaerobic/37 degree	*Enterococcus faecium*
anaerobic/40-50 degree	*Enterococcus faecium*
anaerobic/42 degree	*Enterococcus faecium*
anaerobic/42 degree	*Enterococcus faecium*
anaerobic/42 degree	*Enterococcus faecium*
anaerobic/42 degree	*Enterococcus faecium*
anaerobic/42 degree	*Enterococcus faecium*
anaerobic/42 degree	*Enterococcus faecium*
anaerobic/42 degree	*Enterococcus faecium*
anaerobic/42 degree	*Enterococcus faecium*
anaerobic/42 degree	*Enterococcus faecium*
anaerobic/42 degree	*Enterococcus faecium*
anaerobic/42 degree	*Enterococcus faecium*
anaerobic/42 degree	*Enterococcus faecium*
anaerobic/42 degree	*Enterococcus faecium*
anaerobic/42 degree	*Enterococcus faecium*
anaerobic/42 degree	*Enterococcus faecium*
anaerobic/42 degree	*Enterococcus faecium*
anaerobic/42 degree	*Enterococcus faecium*
anaerobic/42 degree	*Enterococcus faecium*
anaerobic/42 degree	*Enterococcus faecium*
anaerobic/42 degree	*Enterococcus faecium*
anaerobic/42 degree	*Enterococcus faecium*
anaerobic/42 degree	*Enterococcus faecium*
anaerobic/42 degree	*Enterococcus faecium*
anaerobic/42 degree	*Enterococcus faecium*
anaerobic/42 degree	*Enterococcus faecium*
anaerobic/42 degree	*Enterococcus faecium*
anaerobic/42 degree	*Enterococcus faecium*
anaerobic/42 degree	*Enterococcus faecium*
anaerobic/42 degree	*Enterococcus faecium*
anaerobic/42 degree	*Enterococcus faecium*
anaerobic/42 degree	*Enterococcus faecium*

Table S5. The cultured strains and their culture atmospheres

**Atmosphere**	***species***
anaerobic/42 degree	*Enterococcus faecium*
anaerobic/42 degree	*Enterococcus faecium*
anaerobic/42 degree	*Enterococcus faecium*
anaerobic/42 degree	*Enterococcus faecium*
anaerobic/42 degree	*Enterococcus faecium*
anaerobic/42 degree	*Enterococcus faecium*
anaerobic/42 degree	*Enterococcus faecium*
anaerobic/42 degree	*Enterococcus faecium*
anaerobic/35 degree	*Enterococcus gallinarum*
anaerobic/37 degree	*Enterococcus gallinarum*
anaerobic/37 degree	*Enterococcus gallinarum*
anaerobic/37 degree	*Enterococcus gallinarum*
anaerobic/37 degree	*Enterococcus gallinarum*
anaerobic/37 degree	*Enterococcus gallinarum*
anaerobic/37 degree	*Enterococcus gallinarum*
anaerobic/37 degree	*Enterococcus gallinarum*
anaerobic/37 degree	*Enterococcus gallinarum*
anaerobic/37 degree	*Enterococcus gallinarum*
anaerobic/37 degree	*Enterococcus gallinarum*
anaerobic/37 degree	*Enterococcus gallinarum*
anaerobic/37 degree	*Enterococcus gallinarum*
anaerobic/37 degree	*Enterococcus gallinarum*
anaerobic/37 degree	*Enterococcus gallinarum*
anaerobic/37 degree	*Enterococcus gallinarum*
anaerobic/37 degree	*Enterococcus gallinarum*
anaerobic/37 degree	*Enterococcus gallinarum*
anaerobic/37 degree	*Enterococcus gallinarum*
anaerobic/37 degree	*Enterococcus gallinarum*
anaerobic/37 degree	*Enterococcus gallinarum*
anaerobic/37 degree	*Enterococcus gallinarum*
anaerobic/37 degree	*Enterococcus gallinarum*
anaerobic/37 degree	*Enterococcus gallinarum*
anaerobic/37 degree	*Enterococcus gallinarum*
anaerobic/37 degree	*Enterococcus gallinarum*
anaerobic/37 degree	*Enterococcus gallinarum*
anaerobic/37 degree	*Enterococcus gallinarum*
anaerobic/37 degree	*Enterococcus gallinarum*
anaerobic/37 degree	*Enterococcus gallinarum*
anaerobic/37 degree	*Enterococcus gallinarum*
anaerobic/37 degree	*Enterococcus gallinarum*
anaerobic/37 degree	*Enterococcus gallinarum*
anaerobic/37 degree	*Enterococcus gallinarum*
anaerobic/37 degree	*Enterococcus gallinarum*
anaerobic/37 degree	*Enterococcus gallinarum*

Table S5. The cultured strains and their culture atmospheres

Atmosphere	*species*
anaerobic/37 degree	*Enterococcus gallinarum*
anaerobic/37 degree	*Enterococcus gallinarum*
anaerobic/37 degree	*Enterococcus gallinarum*
anaerobic/37 degree	*Enterococcus gallinarum*
anaerobic/37 degree	*Enterococcus gallinarum*
anaerobic/37 degree	*Enterococcus gallinarum*
anaerobic/37 degree	*Enterococcus gallinarum*
anaerobic/37 degree	*Enterococcus gallinarum*
anaerobic/37 degree	*Enterococcus gallinarum*
anaerobic/37 degree	*Enterococcus gallinarum*
anaerobic/37 degree	*Enterococcus gallinarum*
anaerobic/37 degree	*Enterococcus gallinarum*
anaerobic/37 degree	*Enterococcus gallinarum*
anaerobic/37 degree	*Enterococcus gallinarum*
anaerobic/37 degree	*Enterococcus gallinarum*
anaerobic/37 degree	*Enterococcus gallinarum*
anaerobic/37 degree	*Enterococcus gallinarum*
anaerobic/42 degree	*Enterococcus gallinarum*
anaerobic/42 degree	*Enterococcus gallinarum*
anaerobic/42 degree	*Enterococcus gallinarum*
anaerobic/42 degree	*Enterococcus gallinarum*
anaerobic/42 degree	*Enterococcus gallinarum*
anaerobic/42 degree	*Enterococcus gallinarum*
anaerobic/42 degree	*Enterococcus gallinarum*
anaerobic/42 degree	*Enterococcus gallinarum*
anaerobic/42 degree	*Enterococcus gallinarum*
anaerobic/42 degree	*Enterococcus gallinarum*
anaerobic/42 degree	*Enterococcus gallinarum*
anaerobic/42 degree	*Enterococcus gallinarum*
anaerobic/42 degree	*Enterococcus gallinarum*
anaerobic/42 degree	*Enterococcus gallinarum*
anaerobic/42 degree	*Enterococcus gallinarum*
anaerobic/42 degree	*Enterococcus gallinarum*
anaerobic/42 degree	*Enterococcus gallinarum*
anaerobic/37 degree	*Enterococcus gilvus*
anaerobic/37 degree	*Enterococcus gilvus*
anaerobic/37 degree	*Enterococcus gilvus*
anaerobic/37 degree	*Enterococcus hirae*
anaerobic/37 degree	*Enterococcus hirae*
anaerobic/37 degree	*Enterococcus hirae*
anaerobic/37 degree	*Enterococcus hirae*
anaerobic/37 degree	*Enterococcus hirae*
anaerobic/37 degree	*Enterococcus hirae*
anaerobic/37 degree	*Enterococcus hirae*

Table S5. The cultured strains and their culture atmospheres

**Atmosphere**	***species***
anaerobic/37 degree	*Enterococcus hirae*
anaerobic/37 degree	*Enterococcus hirae*
anaerobic/37 degree	*Enterococcus hirae*
anaerobic/37 degree	*Enterococcus hirae*
anaerobic/37 degree	*Enterococcus hirae*
anaerobic/37 degree	*Enterococcus hirae*
anaerobic/37 degree	*Enterococcus hirae*
anaerobic/37 degree	*Enterococcus hirae*
anaerobic/37 degree	*Enterococcus hirae*
anaerobic/37 degree	*Enterococcus hirae*
anaerobic/37 degree	*Enterococcus hirae*
anaerobic/37 degree	*Enterococcus hirae*
anaerobic/37 degree	*Enterococcus hirae*
anaerobic/37 degree	*Enterococcus hirae*
anaerobic/37 degree	*Enterococcus hirae*
anaerobic/37 degree	*Enterococcus hirae*
anaerobic/37 degree	*Enterococcus hirae*
anaerobic/37 degree	*Enterococcus hirae*
anaerobic/37 degree	*Enterococcus hirae*
anaerobic/37 degree	*Enterococcus hirae*
anaerobic/37 degree	*Enterococcus hirae*
anaerobic/37 degree	*Enterococcus hirae*
anaerobic/37 degree	*Enterococcus hirae*
anaerobic/37 degree	*Enterococcus hirae*
anaerobic/37 degree	*Enterococcus hirae*
anaerobic/37 degree	*Enterococcus hirae*
anaerobic/37 degree	*Enterococcus hirae*
anaerobic/37 degree	*Enterococcus hirae*
anaerobic/37 degree	*Enterococcus hirae*
anaerobic/37 degree	*Enterococcus hirae*
anaerobic/37 degree	*Enterococcus hirae*
anaerobic/37 degree	*Enterococcus hirae*
anaerobic/37 degree	*Enterococcus hirae*
anaerobic/37 degree	*Enterococcus hirae*
anaerobic/37 degree	*Enterococcus hirae*
anaerobic/37 degree	*Enterococcus hirae*
anaerobic/37 degree	*Enterococcus hirae*
anaerobic/37 degree	*Enterococcus hirae*
anaerobic/37 degree	*Enterococcus hirae*
anaerobic/37 degree	*Enterococcus hirae*
anaerobic/37 degree	*Enterococcus hirae*
anaerobic/37 degree	*Enterococcus hirae*
anaerobic/37 degree	*Enterococcus hirae*
anaerobic/37 degree	*Enterococcus hirae*

Table S5. The cultured strains and their culture atmospheres

**Atmosphere**	***species***
anaerobic/37 degree	*Enterococcus hirae*
anaerobic/37 degree	*Enterococcus hirae*
anaerobic/37 degree	*Enterococcus hirae*
anaerobic/37 degree	*Enterococcus hirae*
anaerobic/37 degree	*Enterococcus hirae*
anaerobic/37 degree	*Enterococcus hirae*
anaerobic/37 degree	*Enterococcus hirae*
anaerobic/37 degree	*Enterococcus hirae*
anaerobic/37 degree	*Enterococcus hirae*
anaerobic/37 degree	*Enterococcus hirae*
anaerobic/37 degree	*Enterococcus hirae*
anaerobic/37 degree	*Enterococcus hirae*
anaerobic/37 degree	*Enterococcus hirae*
anaerobic/37 degree	*Enterococcus hirae*
anaerobic/37 degree	*Enterococcus hirae*
anaerobic/37 degree	*Enterococcus hirae*
anaerobic/37 degree	*Enterococcus hirae*
anaerobic/37 degree	*Enterococcus hirae*
anaerobic/37 degree	*Enterococcus hirae*
anaerobic/37 degree	*Enterococcus hirae*
anaerobic/37 degree	*Enterococcus hirae*
anaerobic/37 degree	*Enterococcus hirae*
anaerobic/37 degree	*Enterococcus hirae*
anaerobic/37 degree	*Enterococcus hirae*
anaerobic/37 degree	*Enterococcus hirae*
anaerobic/37 degree	*Enterococcus hirae*
anaerobic/37 degree	*Enterococcus hirae*
anaerobic/37 degree	*Enterococcus hirae*
anaerobic/37 degree	*Enterococcus hirae*
anaerobic/37 degree	*Enterococcus hirae*
anaerobic/37 degree	*Enterococcus hirae*
anaerobic/37 degree	*Enterococcus hirae*
anaerobic/37 degree	*Enterococcus hirae*
anaerobic/37 degree	*Enterococcus hirae*
anaerobic/37 degree	*Enterococcus hirae*
anaerobic/37 degree	*Enterococcus hirae*
anaerobic/37 degree	*Enterococcus hirae*
anaerobic/37 degree	*Enterococcus hirae*
anaerobic/37 degree	*Enterococcus hirae*
anaerobic/37 degree	*Enterococcus hirae*
anaerobic/37 degree	*Enterococcus hirae*
anaerobic/37 degree	*Enterococcus hirae*
anaerobic/37 degree	*Enterococcus hirae*
anaerobic/37 degree	*Enterococcus hirae*

Table S5. The cultured strains and their culture atmospheres

Atmosphere	*species*
anaerobic/37 degree	*Enterococcus hirae*
anaerobic/37 degree	*Enterococcus hirae*
anaerobic/42 degree	*Enterococcus hirae*
anaerobic/42 degree	*Enterococcus hirae*
anaerobic/42 degree	*Enterococcus hirae*
anaerobic/42 degree	*Enterococcus hirae*
anaerobic/42 degree	*Enterococcus hirae*
anaerobic/37 degree	*Enterococcus mundtii*
anaerobic/42 degree	*Enterococcus raffinosus*
anaerobic/37 degree	*Enterococcus thailandicus*
anaerobic/37 degree	*Erysipelatoclostridium ramosum*
anaerobic/35 degree	*Escherichia coli*
anaerobic/35 degree	*Escherichia coli*
anaerobic/37 degree	*Escherichia coli*
anaerobic/37 degree	*Escherichia coli*
anaerobic/37 degree	*Escherichia coli*
anaerobic/37 degree	*Escherichia coli*
anaerobic/37 degree	*Escherichia coli*
anaerobic/37 degree	*Escherichia coli*
anaerobic/37 degree	*Escherichia coli*
anaerobic/37 degree	*Escherichia coli*
anaerobic/37 degree	*Escherichia coli*
anaerobic/37 degree	*Escherichia coli*
anaerobic/37 degree	*Escherichia coli*
anaerobic/37 degree	*Escherichia coli*
anaerobic/37 degree	*Escherichia coli*
anaerobic/37 degree	*Escherichia coli*
anaerobic/37 degree	*Escherichia coli*
anaerobic/37 degree	*Escherichia coli*
anaerobic/37 degree	*Escherichia coli*
anaerobic/37 degree	*Escherichia coli*
anaerobic/37 degree	*Escherichia coli*
anaerobic/37 degree	*Escherichia coli*
anaerobic/37 degree	*Escherichia coli*
anaerobic/37 degree	*Escherichia coli*
anaerobic/37 degree	*Escherichia coli*
anaerobic/37 degree	*Escherichia coli*
anaerobic/37 degree	*Escherichia coli*
anaerobic/37 degree	*Escherichia coli*
anaerobic/37 degree	*Escherichia coli*
anaerobic/37 degree	*Escherichia coli*
anaerobic/37 degree	*Escherichia coli*
anaerobic/37 degree	*Escherichia coli*
anaerobic/37 degree	*Escherichia coli*
anaerobic/37 degree	*Escherichia coli*

Table S5. The cultured strains and their culture atmospheres

Atmosphere	***species***
anaerobic/37 degree	*Escherichia coli*
anaerobic/37 degree	*Escherichia coli*
anaerobic/37 degree	*Escherichia coli*
anaerobic/37 degree	*Escherichia coli*
anaerobic/37 degree	*Escherichia coli*
anaerobic/37 degree	*Escherichia coli*
anaerobic/37 degree	*Escherichia coli*
anaerobic/37 degree	*Escherichia coli*
anaerobic/37 degree	*Escherichia coli*
anaerobic/37 degree	*Escherichia coli*
anaerobic/37 degree	*Escherichia coli*
anaerobic/37 degree	*Escherichia coli*
anaerobic/37 degree	*Escherichia coli*
anaerobic/37 degree	*Escherichia coli*
anaerobic/37 degree	*Escherichia coli*
anaerobic/37 degree	*Escherichia coli*
anaerobic/37 degree	*Escherichia coli*
anaerobic/37 degree	*Escherichia coli*
anaerobic/37 degree	*Escherichia coli*
anaerobic/37 degree	*Escherichia coli*
anaerobic/37 degree	*Escherichia coli*
anaerobic/37 degree	*Escherichia coli*
anaerobic/37 degree	*Escherichia coli*
anaerobic/37 degree	*Escherichia coli*
anaerobic/37 degree	*Escherichia coli*
anaerobic/37 degree	*Escherichia coli*
anaerobic/37 degree	*Escherichia coli*
anaerobic/37 degree	*Escherichia coli*
anaerobic/37 degree	*Escherichia coli*
anaerobic/37 degree	*Escherichia coli*
anaerobic/37 degree	*Escherichia coli*
anaerobic/37 degree	*Escherichia coli*
anaerobic/37 degree	*Escherichia coli*
anaerobic/37 degree	*Escherichia coli*
anaerobic/37 degree	*Escherichia coli*
anaerobic/37 degree	*Escherichia coli*
anaerobic/37 degree	*Escherichia coli*
anaerobic/37 degree	*Escherichia coli*
anaerobic/37 degree	*Escherichia coli*
anaerobic/37 degree	*Escherichia coli*
anaerobic/37 degree	*Escherichia coli*
anaerobic/37 degree	*Escherichia coli*
anaerobic/37 degree	*Escherichia coli*
anaerobic/37 degree	*Escherichia coli*

Table S5. The cultured strains and their culture atmospheres

**Atmosphere**	***species***
anaerobic/37 degree	*Escherichia coli*
anaerobic/37 degree	*Escherichia coli*
anaerobic/37 degree	*Escherichia coli*
anaerobic/37 degree	*Escherichia coli*
anaerobic/37 degree	*Escherichia coli*
anaerobic/37 degree	*Escherichia coli*
anaerobic/37 degree	*Escherichia coli*
anaerobic/37 degree	*Escherichia coli*
anaerobic/37 degree	*Escherichia coli*
anaerobic/37 degree	*Escherichia coli*
anaerobic/37 degree	*Escherichia coli*
anaerobic/37 degree	*Escherichia coli*
anaerobic/37 degree	*Escherichia coli*
anaerobic/37 degree	*Escherichia coli*
anaerobic/37 degree	*Escherichia coli*
anaerobic/37 degree	*Escherichia coli*
anaerobic/37 degree	*Escherichia coli*
anaerobic/37 degree	*Escherichia coli*
anaerobic/37 degree	*Escherichia coli*
anaerobic/37 degree	*Escherichia coli*
anaerobic/37 degree	*Escherichia coli*
anaerobic/37 degree	*Escherichia coli*
anaerobic/37 degree	*Escherichia coli*
anaerobic/37 degree	*Escherichia coli*
anaerobic/37 degree	*Escherichia coli*
anaerobic/37 degree	*Escherichia coli*
anaerobic/37 degree	*Escherichia coli*
anaerobic/37 degree	*Escherichia coli*
anaerobic/37 degree	*Escherichia coli*
anaerobic/37 degree	*Escherichia coli*
anaerobic/37 degree	*Escherichia coli*
anaerobic/37 degree	*Escherichia coli*
anaerobic/37 degree	*Escherichia coli*
anaerobic/37 degree	*Escherichia coli*
anaerobic/37 degree	*Escherichia coli*
anaerobic/37 degree	*Escherichia coli*
anaerobic/37 degree	*Escherichia coli*
anaerobic/37 degree	*Escherichia coli*
anaerobic/37 degree	*Escherichia coli*
anaerobic/37 degree	*Escherichia coli*
anaerobic/37 degree	*Escherichia coli*
anaerobic/37 degree	*Escherichia coli*
anaerobic/37 degree	*Escherichia coli*
anaerobic/37 degree	*Escherichia coli*

Table S5. The cultured strains and their culture atmospheres

**Atmosphere**	***species***
anaerobic/37 degree	*Escherichia coli*
anaerobic/37 degree	*Escherichia coli*
anaerobic/37 degree	*Escherichia coli*
anaerobic/37 degree	*Escherichia coli*
anaerobic/37 degree	*Escherichia coli*
anaerobic/37 degree	*Escherichia coli*
anaerobic/37 degree	*Escherichia coli*
anaerobic/37 degree	*Escherichia coli*
anaerobic/37 degree	*Escherichia coli*
anaerobic/37 degree	*Escherichia coli*
anaerobic/37 degree	*Escherichia coli*
anaerobic/37 degree	*Escherichia coli*
anaerobic/37 degree	*Escherichia coli*
anaerobic/37 degree	*Escherichia coli*
anaerobic/37 degree	*Escherichia coli*
anaerobic/37 degree	*Escherichia coli*
anaerobic/37 degree	*Escherichia coli*
anaerobic/37 degree	*Escherichia coli*
anaerobic/37 degree	*Escherichia coli*
anaerobic/37 degree	*Escherichia coli*
anaerobic/37 degree	*Escherichia coli*
anaerobic/37 degree	*Escherichia coli*
anaerobic/37 degree	*Escherichia coli*
anaerobic/37 degree	*Escherichia coli*
anaerobic/37 degree	*Escherichia coli*
anaerobic/37 degree	*Escherichia coli*
anaerobic/37 degree	*Escherichia coli*
anaerobic/37 degree	*Escherichia coli*
anaerobic/37 degree	*Escherichia coli*
anaerobic/37 degree	*Escherichia coli*
anaerobic/37 degree	*Escherichia coli*
anaerobic/37 degree	*Escherichia coli*
anaerobic/37 degree	*Escherichia coli*
anaerobic/37 degree	*Escherichia coli*
anaerobic/37 degree	*Escherichia coli*
anaerobic/37 degree	*Escherichia coli*
anaerobic/37 degree	*Escherichia coli*
anaerobic/37 degree	*Escherichia coli*
anaerobic/37 degree	*Escherichia coli*
anaerobic/37 degree	*Escherichia coli*
anaerobic/37 degree	*Escherichia coli*
anaerobic/37 degree	*Escherichia coli*
anaerobic/37 degree	*Escherichia coli*
anaerobic/37 degree	*Escherichia coli*

Table S5. The cultured strains and their culture atmospheres

**Atmosphere**	***species***
anaerobic/37 degree	*Escherichia coli*
anaerobic/37 degree	*Escherichia coli*
anaerobic/37 degree	*Escherichia coli*
anaerobic/37 degree	*Escherichia coli*
anaerobic/37 degree	*Escherichia coli*
anaerobic/37 degree	*Escherichia coli*
anaerobic/37 degree	*Escherichia coli*
anaerobic/37 degree	*Escherichia coli*
anaerobic/37 degree	*Escherichia coli*
anaerobic/37 degree	*Escherichia coli*
anaerobic/37 degree	*Escherichia coli*
anaerobic/37 degree	*Escherichia coli*
anaerobic/37 degree	*Escherichia coli*
anaerobic/37 degree	*Escherichia coli*
anaerobic/37 degree	*Escherichia coli*
anaerobic/37 degree	*Escherichia coli*
anaerobic/37 degree	*Escherichia coli*
anaerobic/37 degree	*Escherichia coli*
anaerobic/37 degree	*Escherichia coli*
anaerobic/37 degree	*Escherichia coli*
anaerobic/37 degree	*Escherichia coli*
anaerobic/37 degree	*Escherichia coli*
anaerobic/37 degree	*Escherichia coli*
anaerobic/37 degree	*Escherichia coli*
anaerobic/37 degree	*Escherichia coli*
anaerobic/37 degree	*Escherichia coli*
anaerobic/37 degree	*Escherichia coli*
anaerobic/37 degree	*Escherichia coli*
anaerobic/37 degree	*Escherichia coli*
anaerobic/37 degree	*Escherichia coli*
anaerobic/37 degree	*Escherichia coli*
anaerobic/37 degree	*Escherichia coli*
anaerobic/37 degree	*Escherichia coli*
anaerobic/37 degree	*Escherichia coli*
anaerobic/37 degree	*Escherichia coli*
anaerobic/37 degree	*Escherichia coli*
anaerobic/37 degree	*Escherichia coli*
anaerobic/37 degree	*Escherichia coli*
anaerobic/37 degree	*Escherichia coli*
anaerobic/37 degree	*Escherichia coli*
anaerobic/37 degree	*Escherichia coli*
anaerobic/37 degree	*Escherichia coli*
anaerobic/37 degree	*Escherichia coli*
anaerobic/37 degree	*Escherichia coli*

Table S5. The cultured strains and their culture atmospheres

**Atmosphere**	***species***
anaerobic/37 degree	*Escherichia coli*
anaerobic/37 degree	*Escherichia coli*
anaerobic/37 degree	*Escherichia coli*
anaerobic/37 degree	*Escherichia coli*
anaerobic/37 degree	*Escherichia coli*
anaerobic/37 degree	*Escherichia coli*
anaerobic/37 degree	*Escherichia coli*
anaerobic/37 degree	*Escherichia coli*
anaerobic/37 degree	*Escherichia coli*
anaerobic/37 degree	*Escherichia coli*
anaerobic/37 degree	*Escherichia coli*
anaerobic/37 degree	*Escherichia coli*
anaerobic/37 degree	*Escherichia coli*
anaerobic/37 degree	*Escherichia coli*
anaerobic/37 degree	*Escherichia coli*
anaerobic/37 degree	*Escherichia coli*
anaerobic/37 degree	*Escherichia coli*
anaerobic/37 degree	*Escherichia coli*
anaerobic/37 degree	*Escherichia coli*
anaerobic/37 degree	*Escherichia coli*
anaerobic/37 degree	*Escherichia coli*
anaerobic/37 degree	*Escherichia coli*
anaerobic/37 degree	*Escherichia coli*
anaerobic/37 degree	*Escherichia coli*
anaerobic/37 degree	*Escherichia coli*
anaerobic/37 degree	*Escherichia coli*
anaerobic/37 degree	*Escherichia coli*
anaerobic/37 degree	*Escherichia coli*
anaerobic/37 degree	*Escherichia coli*
anaerobic/37 degree	*Escherichia coli*
anaerobic/37 degree	*Escherichia coli*
anaerobic/37 degree	*Escherichia coli*
anaerobic/37 degree	*Escherichia coli*
anaerobic/37 degree	*Escherichia coli*
anaerobic/37 degree	*Escherichia coli*
anaerobic/37 degree	*Escherichia coli*
anaerobic/37 degree	*Escherichia coli*
anaerobic/37 degree	*Escherichia coli*
anaerobic/37 degree	*Escherichia coli*
anaerobic/37 degree	*Escherichia coli*
anaerobic/37 degree	*Escherichia coli*
anaerobic/37 degree	*Escherichia coli*
anaerobic/37 degree	*Escherichia coli*
anaerobic/37 degree	*Escherichia coli*

Table S5. The cultured strains and their culture atmospheres

**Atmosphere**	***species***
anaerobic/37 degree	*Escherichia coli*
anaerobic/37 degree	*Escherichia coli*
anaerobic/37 degree	*Escherichia coli*
anaerobic/37 degree	*Escherichia coli*
anaerobic/37 degree	*Escherichia coli*
anaerobic/37 degree	*Escherichia coli*
anaerobic/37 degree	*Escherichia coli*
anaerobic/37 degree	*Escherichia coli*
anaerobic/37 degree	*Escherichia coli*
anaerobic/37 degree	*Escherichia coli*
anaerobic/37 degree	*Escherichia coli*
anaerobic/37 degree	*Escherichia coli*
anaerobic/37 degree	*Escherichia coli*
anaerobic/37 degree	*Escherichia coli*
anaerobic/37 degree	*Escherichia coli*
anaerobic/37 degree	*Escherichia coli*
anaerobic/37 degree	*Escherichia coli*
anaerobic/37 degree	*Escherichia coli*
anaerobic/37 degree	*Escherichia coli*
anaerobic/37 degree	*Escherichia coli*
anaerobic/37 degree	*Escherichia coli*
anaerobic/37 degree	*Escherichia coli*
anaerobic/37 degree	*Escherichia coli*
anaerobic/37 degree	*Escherichia coli*
anaerobic/37 degree	*Escherichia coli*
anaerobic/37 degree	*Escherichia coli*
anaerobic/37 degree	*Escherichia coli*
anaerobic/37 degree	*Escherichia coli*
anaerobic/37 degree	*Escherichia coli*
anaerobic/37 degree	*Escherichia coli*
anaerobic/37 degree	*Escherichia coli*
anaerobic/37 degree	*Escherichia coli*
anaerobic/37 degree	*Escherichia coli*
anaerobic/37 degree	*Escherichia coli*
anaerobic/37 degree	*Escherichia coli*
anaerobic/37 degree	*Escherichia coli*
anaerobic/37 degree	*Escherichia coli*
anaerobic/37 degree	*Escherichia coli*
anaerobic/37 degree	*Escherichia coli*
anaerobic/37 degree	*Escherichia coli*
anaerobic/37 degree	*Escherichia coli*
anaerobic/37 degree	*Escherichia coli*
anaerobic/37 degree	*Escherichia coli*
anaerobic/37 degree	*Escherichia coli*

Table S5. The cultured strains and their culture atmospheres

**Atmosphere**	***species***
anaerobic/37 degree	*Escherichia coli*
anaerobic/37 degree	*Escherichia coli*
anaerobic/37 degree	*Escherichia coli*
anaerobic/37 degree	*Escherichia coli*
anaerobic/37 degree	*Escherichia coli*
anaerobic/37 degree	*Escherichia coli*
anaerobic/37 degree	*Escherichia coli*
anaerobic/37 degree	*Escherichia coli*
anaerobic/37 degree	*Escherichia coli*
anaerobic/37 degree	*Escherichia coli*
anaerobic/37 degree	*Escherichia coli*
anaerobic/37 degree	*Escherichia coli*
anaerobic/37 degree	*Escherichia coli*
anaerobic/37 degree	*Escherichia coli*
anaerobic/37 degree	*Escherichia coli*
anaerobic/37 degree	*Escherichia coli*
anaerobic/37 degree	*Escherichia coli*
anaerobic/37 degree	*Escherichia coli*
anaerobic/37 degree	*Escherichia coli*
anaerobic/37 degree	*Escherichia coli*
anaerobic/37 degree	*Escherichia coli*
anaerobic/37 degree	*Escherichia coli*
anaerobic/37 degree	*Escherichia coli*
anaerobic/37 degree	*Escherichia coli*
anaerobic/37 degree	*Escherichia coli*
anaerobic/37 degree	*Escherichia coli*
anaerobic/37 degree	*Escherichia coli*
anaerobic/37 degree	*Escherichia coli*
anaerobic/37 degree	*Escherichia coli*
anaerobic/37 degree	*Escherichia coli*
anaerobic/37 degree	*Escherichia coli*
anaerobic/37 degree	*Escherichia coli*
anaerobic/37 degree	*Escherichia coli*
anaerobic/37 degree	*Escherichia coli*
anaerobic/37 degree	*Escherichia coli*
anaerobic/37 degree	*Escherichia coli*
anaerobic/37 degree	*Escherichia coli*
anaerobic/37 degree	*Escherichia coli*
anaerobic/37 degree	*Escherichia coli*
anaerobic/37 degree	*Escherichia coli*
anaerobic/37 degree	*Escherichia coli*
anaerobic/37 degree	*Escherichia coli*
anaerobic/37 degree	*Escherichia coli*
anaerobic/37 degree	*Escherichia coli*

Table S5. The cultured strains and their culture atmospheres

**Atmosphere**	***species***
anaerobic/37 degree	*Escherichia coli*
anaerobic/37 degree	*Escherichia coli*
anaerobic/37 degree	*Escherichia coli*
anaerobic/37 degree	*Escherichia coli*
anaerobic/37 degree	*Escherichia coli*
anaerobic/37 degree	*Escherichia coli*
anaerobic/37 degree	*Escherichia coli*
anaerobic/37 degree	*Escherichia coli*
anaerobic/37 degree	*Escherichia coli*
anaerobic/37 degree	*Escherichia coli*
anaerobic/37 degree	*Escherichia coli*
anaerobic/37 degree	*Escherichia coli*
anaerobic/37 degree	*Escherichia coli*
anaerobic/37 degree	*Escherichia coli*
anaerobic/37 degree	*Escherichia coli*
anaerobic/37 degree	*Escherichia coli*
anaerobic/37 degree	*Escherichia coli*
anaerobic/37 degree	*Escherichia coli*
anaerobic/37 degree	*Escherichia coli*
anaerobic/37 degree	*Escherichia coli*
anaerobic/37 degree	*Escherichia coli*
anaerobic/37 degree	*Escherichia coli*
anaerobic/37 degree	*Escherichia coli*
anaerobic/37 degree	*Escherichia coli*
anaerobic/37 degree	*Escherichia coli*
anaerobic/37 degree	*Escherichia coli*
anaerobic/37 degree	*Escherichia coli*
anaerobic/37 degree	*Escherichia coli*
anaerobic/37 degree	*Escherichia coli*
anaerobic/37 degree	*Escherichia coli*
anaerobic/37 degree	*Escherichia coli*
anaerobic/37 degree	*Escherichia coli*
anaerobic/37 degree	*Escherichia coli*
anaerobic/37 degree	*Escherichia coli*
anaerobic/37 degree	*Escherichia coli*
anaerobic/37 degree	*Escherichia coli*
anaerobic/37 degree	*Escherichia coli*
anaerobic/37 degree	*Escherichia coli*
anaerobic/37 degree	*Escherichia coli*
anaerobic/37 degree	*Escherichia coli*
anaerobic/37 degree	*Escherichia coli*
anaerobic/37 degree	*Escherichia coli*
anaerobic/37 degree	*Escherichia coli*
anaerobic/37 degree	*Escherichia coli*

Table S5. The cultured strains and their culture atmospheres

**Atmosphere**	***species***
anaerobic/37 degree	*Escherichia coli*
anaerobic/37 degree	*Escherichia coli*
anaerobic/37 degree	*Escherichia coli*
anaerobic/37 degree	*Escherichia coli*
anaerobic/37 degree	*Escherichia coli*
anaerobic/37 degree	*Escherichia coli*
anaerobic/37 degree	*Escherichia coli*
anaerobic/37 degree	*Escherichia coli*
anaerobic/37 degree	*Escherichia coli*
anaerobic/37 degree	*Escherichia coli*
anaerobic/37 degree	*Escherichia coli*
anaerobic/37 degree	*Escherichia coli*
anaerobic/37 degree	*Escherichia coli*
anaerobic/37 degree	*Escherichia coli*
anaerobic/37 degree	*Escherichia coli*
anaerobic/37 degree	*Escherichia coli*
anaerobic/37 degree	*Escherichia coli*
anaerobic/37 degree	*Escherichia coli*
anaerobic/37 degree	*Escherichia coli*
anaerobic/37 degree	*Escherichia coli*
anaerobic/37 degree	*Escherichia coli*
anaerobic/37 degree	*Escherichia coli*
anaerobic/37 degree	*Escherichia coli*
anaerobic/37 degree	*Escherichia coli*
anaerobic/37 degree	*Escherichia coli*
anaerobic/37 degree	*Escherichia coli*
anaerobic/37 degree	*Escherichia coli*
anaerobic/37 degree	*Escherichia coli*
anaerobic/37 degree	*Escherichia coli*
anaerobic/37 degree	*Escherichia coli*
anaerobic/37 degree	*Escherichia coli*
anaerobic/37 degree	*Escherichia coli*
anaerobic/37 degree	*Escherichia coli*
anaerobic/37 degree	*Escherichia coli*
anaerobic/37 degree	*Escherichia coli*
anaerobic/37 degree	*Escherichia coli*
anaerobic/37 degree	*Escherichia coli*
anaerobic/37 degree	*Escherichia coli*
anaerobic/37 degree	*Escherichia coli*
anaerobic/37 degree	*Escherichia coli*
anaerobic/37 degree	*Escherichia coli*
anaerobic/37 degree	*Escherichia coli*
anaerobic/37 degree	*Escherichia coli*
anaerobic/37 degree	*Escherichia coli*

Table S5. The cultured strains and their culture atmospheres

**Atmosphere**	***species***
anaerobic/37 degree	*Escherichia coli*
anaerobic/37 degree	*Escherichia coli*
anaerobic/37 degree	*Escherichia coli*
anaerobic/37 degree	*Escherichia coli*
anaerobic/37 degree	*Escherichia coli*
anaerobic/37 degree	*Escherichia coli*
anaerobic/37 degree	*Escherichia coli*
anaerobic/37 degree	*Escherichia coli*
anaerobic/37 degree	*Escherichia coli*
anaerobic/37 degree	*Escherichia coli*
anaerobic/37 degree	*Escherichia coli*
anaerobic/37 degree	*Escherichia coli*
anaerobic/37 degree	*Escherichia coli*
anaerobic/37 degree	*Escherichia coli*
anaerobic/37 degree	*Escherichia coli*
anaerobic/37 degree	*Escherichia coli*
anaerobic/37 degree	*Escherichia coli*
anaerobic/37 degree	*Escherichia coli*
anaerobic/37 degree	*Escherichia coli*
anaerobic/37 degree	*Escherichia coli*
anaerobic/37 degree	*Escherichia coli*
anaerobic/37 degree	*Escherichia coli*
anaerobic/37 degree	*Escherichia coli*
anaerobic/37 degree	*Escherichia coli*
anaerobic/37 degree	*Escherichia coli*
anaerobic/37 degree	*Escherichia coli*
anaerobic/37 degree	*Escherichia coli*
anaerobic/37 degree	*Escherichia coli*
anaerobic/37 degree	*Escherichia coli*
anaerobic/37 degree	*Escherichia coli*
anaerobic/37 degree	*Escherichia coli*
anaerobic/37 degree	*Escherichia coli*
anaerobic/37 degree	*Escherichia coli*
anaerobic/37 degree	*Escherichia coli*
anaerobic/37 degree	*Escherichia coli*
anaerobic/37 degree	*Escherichia coli*
anaerobic/37 degree	*Escherichia coli*
anaerobic/37 degree	*Escherichia coli*
anaerobic/37 degree	*Escherichia coli*
anaerobic/37 degree	*Escherichia coli*
anaerobic/37 degree	*Escherichia coli*
anaerobic/37 degree	*Escherichia coli*
anaerobic/37 degree	*Escherichia coli*
anaerobic/37 degree	*Escherichia coli*

Table S5. The cultured strains and their culture atmospheres

**Atmosphere**	***species***
anaerobic/37 degree	*Escherichia coli*
anaerobic/37 degree	*Escherichia coli*
anaerobic/37 degree	*Escherichia coli*
anaerobic/37 degree	*Escherichia coli*
anaerobic/37 degree	*Escherichia coli*
anaerobic/37 degree	*Escherichia coli*
anaerobic/37 degree	*Escherichia coli*
anaerobic/37 degree	*Escherichia coli*
anaerobic/37 degree	*Escherichia coli*
anaerobic/37 degree	*Escherichia coli*
anaerobic/37 degree	*Escherichia coli*
anaerobic/37 degree	*Escherichia coli*
anaerobic/37 degree	*Escherichia coli*
anaerobic/37 degree	*Escherichia coli*
anaerobic/37 degree	*Escherichia coli*
anaerobic/37 degree	*Escherichia coli*
anaerobic/37 degree	*Escherichia coli*
anaerobic/37 degree	*Escherichia coli*
anaerobic/37 degree	*Escherichia coli*
anaerobic/37 degree	*Escherichia coli*
anaerobic/37 degree	*Escherichia coli*
anaerobic/37 degree	*Escherichia coli*
anaerobic/37 degree	*Escherichia coli*
anaerobic/37 degree	*Escherichia coli*
anaerobic/37 degree	*Escherichia coli*
anaerobic/37 degree	*Escherichia coli*
anaerobic/37 degree	*Escherichia coli*
anaerobic/37 degree	*Escherichia coli*
anaerobic/37 degree	*Escherichia coli*
anaerobic/37 degree	*Escherichia coli*
anaerobic/37 degree	*Escherichia coli*
anaerobic/37 degree	*Escherichia coli*
anaerobic/37 degree	*Escherichia coli*
anaerobic/37 degree	*Escherichia coli*
anaerobic/37 degree	*Escherichia coli*
anaerobic/37 degree	*Escherichia coli*
anaerobic/37 degree	*Escherichia coli*
anaerobic/37 degree	*Escherichia coli*
anaerobic/37 degree	*Escherichia coli*
anaerobic/37 degree	*Escherichia coli*
anaerobic/37 degree	*Escherichia coli*
anaerobic/37 degree	*Escherichia coli*
anaerobic/37 degree	*Escherichia coli*
anaerobic/37 degree	*Escherichia coli*

Table S5. The cultured strains and their culture atmospheres

Atmosphere	*species*
anaerobic/37 degree	*Escherichia coli*
anaerobic/37 degree	*Escherichia coli*
anaerobic/37 degree	*Escherichia coli*
anaerobic/37 degree	*Escherichia coli*
anaerobic/37 degree	*Escherichia coli*
anaerobic/37 degree	*Escherichia coli*
anaerobic/37 degree	*Escherichia coli*
anaerobic/37 degree	*Escherichia coli*
anaerobic/37 degree	*Escherichia coli*
anaerobic/37 degree	*Escherichia coli*
anaerobic/37 degree	*Escherichia coli*
anaerobic/37 degree	*Escherichia coli*
anaerobic/37 degree	*Escherichia coli*
anaerobic/37 degree	*Escherichia coli*
anaerobic/37 degree	*Escherichia coli*
anaerobic/37 degree	*Escherichia coli*
anaerobic/37 degree	*Escherichia coli*
anaerobic/37 degree	*Escherichia coli*
anaerobic/37 degree	*Escherichia coli*
anaerobic/37 degree	*Escherichia coli*
anaerobic/37 degree	*Escherichia coli*
anaerobic/37 degree	*Escherichia coli*
anaerobic/37 degree	*Escherichia coli*
anaerobic/37 degree	*Escherichia coli*
anaerobic/37 degree	*Escherichia coli*
anaerobic/37 degree	*Escherichia coli*
anaerobic/37 degree	*Escherichia coli*
anaerobic/37 degree	*Escherichia coli*
anaerobic/37 degree	*Escherichia coli*
anaerobic/37 degree	*Escherichia coli*
anaerobic/37 degree	*Escherichia coli*
anaerobic/37 degree	*Escherichia coli*
anaerobic/37 degree	*Escherichia coli*
anaerobic/37 degree	*Escherichia coli*
anaerobic/37 degree	*Escherichia coli*
anaerobic/37 degree	*Escherichia coli*
anaerobic/37 degree	*Escherichia coli*
anaerobic/37 degree	*Escherichia coli*
anaerobic/37 degree	*Escherichia coli*
anaerobic/37 degree	*Escherichia coli*
anaerobic/37 degree	*Escherichia coli*
anaerobic/37 degree	*Escherichia coli*
anaerobic/37 degree	*Escherichia coli*
anaerobic/37 degree	*Escherichia coli*

Table S5. The cultured strains and their culture atmospheres

**Atmosphere**	***species***
anaerobic/37 degree	*Escherichia coli*
anaerobic/37 degree	*Escherichia coli*
anaerobic/37 degree	*Escherichia coli*
anaerobic/37 degree	*Escherichia coli*
anaerobic/37 degree	*Escherichia coli*
anaerobic/37 degree	*Escherichia coli*
anaerobic/37 degree	*Escherichia coli*
anaerobic/37 degree	*Escherichia coli*
anaerobic/37 degree	*Escherichia coli*
anaerobic/37 degree	*Escherichia coli*
anaerobic/37 degree	*Escherichia coli*
anaerobic/37 degree	*Escherichia coli*
anaerobic/37 degree	*Escherichia coli*
anaerobic/37 degree	*Escherichia coli*
anaerobic/37 degree	*Escherichia coli*
anaerobic/37 degree	*Escherichia coli*
anaerobic/37 degree	*Escherichia coli*
anaerobic/37 degree	*Escherichia coli*
anaerobic/37 degree	*Escherichia coli*
anaerobic/37 degree	*Escherichia coli*
anaerobic/37 degree	*Escherichia coli*
anaerobic/37 degree	*Escherichia coli*
anaerobic/37 degree	*Escherichia coli*
anaerobic/37 degree	*Escherichia coli*
anaerobic/37 degree	*Escherichia coli*
anaerobic/37 degree	*Escherichia coli*
anaerobic/37 degree	*Escherichia coli*
anaerobic/37 degree	*Escherichia coli*
anaerobic/37 degree	*Escherichia coli*
anaerobic/37 degree	*Escherichia coli*
anaerobic/37 degree	*Escherichia coli*
anaerobic/37 degree	*Escherichia coli*
anaerobic/37 degree	*Escherichia coli*
anaerobic/37 degree	*Escherichia coli*
anaerobic/37 degree	*Escherichia coli*
anaerobic/37 degree	*Escherichia coli*
anaerobic/37 degree	*Escherichia coli*
anaerobic/37 degree	*Escherichia coli*
anaerobic/37 degree	*Escherichia coli*
anaerobic/37 degree	*Escherichia coli*
anaerobic/37 degree	*Escherichia coli*
anaerobic/37 degree	*Escherichia coli*
anaerobic/37 degree	*Escherichia coli*
anaerobic/37 degree	*Escherichia coli*

Table S5. The cultured strains and their culture atmospheres

**Atmosphere**	***species***
anaerobic/37 degree	*Escherichia coli*
anaerobic/37 degree	*Escherichia coli*
anaerobic/37 degree	*Escherichia coli*
anaerobic/37 degree	*Escherichia coli*
anaerobic/37 degree	*Escherichia coli*
anaerobic/37 degree	*Escherichia coli*
anaerobic/37 degree	*Escherichia coli*
anaerobic/37 degree	*Escherichia coli*
anaerobic/37 degree	*Escherichia coli*
anaerobic/37 degree	*Escherichia coli*
anaerobic/37 degree	*Escherichia coli*
anaerobic/37 degree	*Escherichia coli*
anaerobic/37 degree	*Escherichia coli*
anaerobic/37 degree	*Escherichia coli*
anaerobic/37 degree	*Escherichia coli*
anaerobic/37 degree	*Escherichia coli*
anaerobic/37 degree	*Escherichia coli*
anaerobic/37 degree	*Escherichia coli*
anaerobic/37 degree	*Escherichia coli*
anaerobic/37 degree	*Escherichia coli*
anaerobic/37 degree	*Escherichia coli*
anaerobic/37 degree	*Escherichia coli*
anaerobic/37 degree	*Escherichia coli*
anaerobic/37 degree	*Escherichia coli*
anaerobic/37 degree	*Escherichia coli*
anaerobic/37 degree	*Escherichia coli*
anaerobic/37 degree	*Escherichia coli*
anaerobic/37 degree	*Escherichia coli*
anaerobic/37 degree	*Escherichia coli*
anaerobic/37 degree	*Escherichia coli*
anaerobic/37 degree	*Escherichia coli*
anaerobic/37 degree	*Escherichia coli*
anaerobic/37 degree	*Escherichia coli*
anaerobic/37 degree	*Escherichia coli*
anaerobic/37 degree	*Escherichia coli*
anaerobic/37 degree	*Escherichia coli*
anaerobic/37 degree	*Escherichia coli*
anaerobic/37 degree	*Escherichia coli*
anaerobic/37 degree	*Escherichia coli*
anaerobic/37 degree	*Escherichia coli*
anaerobic/37 degree	*Escherichia coli*
anaerobic/37 degree	*Escherichia coli*
anaerobic/37 degree	*Escherichia coli*
anaerobic/37 degree	*Escherichia coli*

Table S5. The cultured strains and their culture atmospheres

**Atmosphere**	***species***
anaerobic/37 degree	*Escherichia coli*
anaerobic/37 degree	*Escherichia coli*
anaerobic/37 degree	*Escherichia coli*
anaerobic/37 degree	*Escherichia coli*
anaerobic/37 degree	*Escherichia coli*
anaerobic/37 degree	*Escherichia coli*
anaerobic/37 degree	*Escherichia coli*
anaerobic/37 degree	*Escherichia coli*
anaerobic/37 degree	*Escherichia coli*
anaerobic/37 degree	*Escherichia coli*
anaerobic/37 degree	*Escherichia coli*
anaerobic/37 degree	*Escherichia coli*
anaerobic/37 degree	*Escherichia coli*
anaerobic/37 degree	*Escherichia coli*
anaerobic/37 degree	*Escherichia coli*
anaerobic/37 degree	*Escherichia coli*
anaerobic/37 degree	*Escherichia coli*
anaerobic/37 degree	*Escherichia coli*
anaerobic/37 degree	*Escherichia coli*
anaerobic/37 degree	*Escherichia coli*
anaerobic/37 degree	*Escherichia coli*
anaerobic/37 degree	*Escherichia coli*
anaerobic/37 degree	*Escherichia coli*
anaerobic/37 degree	*Escherichia coli*
anaerobic/37 degree	*Escherichia coli*
anaerobic/37 degree	*Escherichia coli*
anaerobic/37 degree	*Escherichia coli*
anaerobic/37 degree	*Escherichia coli*
anaerobic/37 degree	*Escherichia coli*
anaerobic/37 degree	*Escherichia coli*
anaerobic/37 degree	*Escherichia coli*
anaerobic/37 degree	*Escherichia coli*
anaerobic/37 degree	*Escherichia coli*
anaerobic/37 degree	*Escherichia coli*
anaerobic/37 degree	*Escherichia coli*
anaerobic/37 degree	*Escherichia coli*
anaerobic/37 degree	*Escherichia coli*
anaerobic/37 degree	*Escherichia coli*
anaerobic/37 degree	*Escherichia coli*
anaerobic/37 degree	*Escherichia coli*
anaerobic/37 degree	*Escherichia coli*
anaerobic/37 degree	*Escherichia coli*
anaerobic/37 degree	*Escherichia coli*
anaerobic/37 degree	*Escherichia coli*

Table S5. The cultured strains and their culture atmospheres

**Atmosphere**	***species***
anaerobic/37 degree	*Escherichia coli*
anaerobic/37 degree	*Escherichia coli*
anaerobic/37 degree	*Escherichia coli*
anaerobic/37 degree	*Escherichia coli*
anaerobic/37 degree	*Escherichia coli*
anaerobic/37 degree	*Escherichia coli*
anaerobic/37 degree	*Escherichia coli*
anaerobic/37 degree	*Escherichia coli*
anaerobic/37 degree	*Escherichia coli*
anaerobic/37 degree	*Escherichia coli*
anaerobic/37 degree	*Escherichia coli*
anaerobic/37 degree	*Escherichia coli*
anaerobic/37 degree	*Escherichia coli*
anaerobic/37 degree	*Escherichia coli*
anaerobic/37 degree	*Escherichia coli*
anaerobic/37 degree	*Escherichia coli*
anaerobic/37 degree	*Escherichia coli*
anaerobic/37 degree	*Escherichia coli*
anaerobic/37 degree	*Escherichia coli*
anaerobic/37 degree	*Escherichia coli*
anaerobic/37 degree	*Escherichia coli*
anaerobic/37 degree	*Escherichia coli*
anaerobic/37 degree	*Escherichia coli*
anaerobic/37 degree	*Escherichia coli*
anaerobic/37 degree	*Escherichia coli*
anaerobic/37 degree	*Escherichia coli*
anaerobic/37 degree	*Escherichia coli*
anaerobic/37 degree	*Escherichia coli*
anaerobic/37 degree	*Escherichia coli*
anaerobic/37 degree	*Escherichia coli*
anaerobic/37 degree	*Escherichia coli*
anaerobic/37 degree	*Escherichia coli*
anaerobic/37 degree	*Escherichia coli*
anaerobic/37 degree	*Escherichia coli*
anaerobic/37 degree	*Escherichia coli*
anaerobic/37 degree	*Escherichia coli*
anaerobic/37 degree	*Escherichia coli*
anaerobic/37 degree	*Escherichia coli*
anaerobic/37 degree	*Escherichia coli*
anaerobic/37 degree	*Escherichia coli*
anaerobic/37 degree	*Escherichia coli*
anaerobic/37 degree	*Escherichia coli*
anaerobic/37 degree	*Escherichia coli*
anaerobic/37 degree	*Escherichia coli*

Table S5. The cultured strains and their culture atmospheres

**Atmosphere**	***species***
anaerobic/37 degree	*Escherichia coli*
anaerobic/37 degree	*Escherichia coli*
anaerobic/37 degree	*Escherichia coli*
anaerobic/37 degree	*Escherichia coli*
anaerobic/37 degree	*Escherichia coli*
anaerobic/37 degree	*Escherichia coli*
anaerobic/37 degree	*Escherichia coli*
anaerobic/37 degree	*Escherichia coli*
anaerobic/37 degree	*Escherichia coli*
anaerobic/37 degree	*Escherichia coli*
anaerobic/37 degree	*Escherichia coli*
anaerobic/37 degree	*Escherichia coli*
anaerobic/37 degree	*Escherichia coli*
anaerobic/37 degree	*Escherichia coli*
anaerobic/37 degree	*Escherichia coli*
anaerobic/37 degree	*Escherichia coli*
anaerobic/37 degree	*Escherichia coli*
anaerobic/37 degree	*Escherichia coli*
anaerobic/37 degree	*Escherichia coli*
anaerobic/37 degree	*Escherichia coli*
anaerobic/37 degree	*Escherichia coli*
anaerobic/37 degree	*Escherichia coli*
anaerobic/37 degree	*Escherichia coli*
anaerobic/37 degree	*Escherichia coli*
anaerobic/37 degree	*Escherichia coli*
anaerobic/37 degree	*Escherichia coli*
anaerobic/37 degree	*Escherichia coli*
anaerobic/37 degree	*Escherichia coli*
anaerobic/37 degree	*Escherichia coli*
anaerobic/37 degree	*Escherichia coli*
anaerobic/37 degree	*Escherichia coli*
anaerobic/37 degree	*Escherichia coli*
anaerobic/37 degree	*Escherichia coli*
anaerobic/37 degree	*Escherichia coli*
anaerobic/37 degree	*Escherichia coli*
anaerobic/37 degree	*Escherichia coli*
anaerobic/37 degree	*Escherichia coli*
anaerobic/37 degree	*Escherichia coli*
anaerobic/37 degree	*Escherichia coli*
anaerobic/37 degree	*Escherichia coli*
anaerobic/37 degree	*Escherichia coli*
anaerobic/37 degree	*Escherichia coli*
anaerobic/37 degree	*Escherichia coli*
anaerobic/37 degree	*Escherichia coli*

Table S5. The cultured strains and their culture atmospheres

**Atmosphere**	***species***
anaerobic/37 degree	*Escherichia coli*
anaerobic/37 degree	*Escherichia coli*
anaerobic/37 degree	*Escherichia coli*
anaerobic/37 degree	*Escherichia coli*
anaerobic/37 degree	*Escherichia coli*
anaerobic/37 degree	*Escherichia coli*
anaerobic/37 degree	*Escherichia coli*
anaerobic/37 degree	*Escherichia coli*
anaerobic/37 degree	*Escherichia coli*
anaerobic/37 degree	*Escherichia coli*
anaerobic/37 degree	*Escherichia coli*
anaerobic/37 degree	*Escherichia coli*
anaerobic/37 degree	*Escherichia coli*
anaerobic/37 degree	*Escherichia coli*
anaerobic/37 degree	*Escherichia coli*
anaerobic/37 degree	*Escherichia coli*
anaerobic/37 degree	*Escherichia coli*
anaerobic/37 degree	*Escherichia coli*
anaerobic/37 degree	*Escherichia coli*
anaerobic/37 degree	*Escherichia coli*
anaerobic/37 degree	*Escherichia coli*
anaerobic/37 degree	*Escherichia coli*
anaerobic/37 degree	*Escherichia coli*
anaerobic/37 degree	*Escherichia coli*
anaerobic/37 degree	*Escherichia coli*
anaerobic/37 degree	*Escherichia coli*
anaerobic/37 degree	*Escherichia coli*
anaerobic/37 degree	*Escherichia coli*
anaerobic/37 degree	*Escherichia coli*
anaerobic/37 degree	*Escherichia coli*
anaerobic/37 degree	*Escherichia coli*
anaerobic/37 degree	*Escherichia coli*
anaerobic/37 degree	*Escherichia coli*
anaerobic/37 degree	*Escherichia coli*
anaerobic/37 degree	*Escherichia coli*
anaerobic/37 degree	*Escherichia coli*
anaerobic/37 degree	*Escherichia coli*
anaerobic/37 degree	*Escherichia coli*
anaerobic/37 degree	*Escherichia coli*
anaerobic/37 degree	*Escherichia coli*
anaerobic/37 degree	*Escherichia coli*
anaerobic/37 degree	*Escherichia coli*
anaerobic/37 degree	*Escherichia coli*
anaerobic/37 degree	*Escherichia coli*

Table S5. The cultured strains and their culture atmospheres

**Atmosphere**	***species***
anaerobic/37 degree	*Escherichia coli*
anaerobic/37 degree	*Escherichia coli*
anaerobic/37 degree	*Escherichia coli*
anaerobic/37 degree	*Escherichia coli*
anaerobic/37 degree	*Escherichia coli*
anaerobic/37 degree	*Escherichia coli*
anaerobic/37 degree	*Escherichia coli*
anaerobic/37 degree	*Escherichia coli*
anaerobic/37 degree	*Escherichia coli*
anaerobic/37 degree	*Escherichia coli*
anaerobic/37 degree	*Escherichia coli*
anaerobic/37 degree	*Escherichia coli*
anaerobic/37 degree	*Escherichia coli*
anaerobic/37 degree	*Escherichia coli*
anaerobic/37 degree	*Escherichia coli*
anaerobic/37 degree	*Escherichia coli*
anaerobic/37 degree	*Escherichia coli*
anaerobic/37 degree	*Escherichia coli*
anaerobic/37 degree	*Escherichia coli*
anaerobic/37 degree	*Escherichia coli*
anaerobic/37 degree	*Escherichia coli*
anaerobic/40-50 degree	*Escherichia coli*
anaerobic/42 degree	*Escherichia coli*
anaerobic/42 degree	*Escherichia coli*
anaerobic/42 degree	*Escherichia coli*
anaerobic/42 degree	*Escherichia coli*
anaerobic/42 degree	*Escherichia coli*
anaerobic/42 degree	*Escherichia coli*
anaerobic/42 degree	*Escherichia coli*
anaerobic/42 degree	*Escherichia coli*
anaerobic/42 degree	*Escherichia coli*
anaerobic/42 degree	*Escherichia coli*
anaerobic/42 degree	*Escherichia coli*
anaerobic/42 degree	*Escherichia coli*
anaerobic/42 degree	*Escherichia coli*
anaerobic/42 degree	*Escherichia coli*
anaerobic/42 degree	*Escherichia coli*
anaerobic/42 degree	*Escherichia coli*
anaerobic/42 degree	*Escherichia coli*
anaerobic/42 degree	*Escherichia coli*
anaerobic/42 degree	*Escherichia coli*
anaerobic/42 degree	*Escherichia coli*
anaerobic/42 degree	*Escherichia coli*
anaerobic/42 degree	*Escherichia coli*
anaerobic/42 degree	*Escherichia coli*

Table S5. The cultured strains and their culture atmospheres

Atmosphere	*species*
anaerobic/42 degree	*Escherichia coli*
anaerobic/42 degree	*Escherichia coli*
anaerobic/42 degree	*Escherichia coli*
anaerobic/42 degree	*Escherichia coli*
anaerobic/42 degree	*Escherichia coli*
anaerobic/42 degree	*Escherichia coli*
anaerobic/42 degree	*Escherichia coli*
anaerobic/35 degree	*Eubacterium limosum*
anaerobic/37 degree	*Eubacterium limosum*
anaerobic/37 degree	*Eubacterium limosum*
anaerobic/37 degree	*Eubacterium limosum*
anaerobic/37 degree	*Eubacterium limosum*
anaerobic/37 degree	*Eubacterium limosum*
anaerobic/37 degree	*Eubacterium limosum*
anaerobic/37 degree	*Eubacterium limosum*
anaerobic/37 degree	*Exiguobacterium aurantiacum*
anaerobic/37 degree	*Finegoldia magna*
anaerobic/37 degree	*Finegoldia magna*
anaerobic/37 degree	*Finegoldia magna*
anaerobic/37 degree	*Finegoldia magna*
anaerobic/37 degree	*Flavonifractor plautii*
anaerobic/37 degree	*Fusobacterium mortiferum*
anaerobic/37 degree	*Fusobacterium mortiferum*
anaerobic/37 degree	*Fusobacterium mortiferum*
anaerobic/37 degree	*Fusobacterium mortiferum*
anaerobic/37 degree	*Fusobacterium mortiferum*
anaerobic/37 degree	*Fusobacterium mortiferum*
anaerobic/37 degree	*Fusobacterium mortiferum*
anaerobic/37 degree	*Fusobacterium mortiferum*
anaerobic/37 degree	*Fusobacterium mortiferum*
anaerobic/37 degree	*Fusobacterium mortiferum*
anaerobic/37 degree	*Fusobacterium mortiferum*
anaerobic/37 degree	*Fusobacterium mortiferum*
anaerobic/37 degree	*Fusobacterium mortiferum*
anaerobic/37 degree	*Fusobacterium mortiferum*
anaerobic/37 degree	*Fusobacterium mortiferum*
anaerobic/37 degree	*Fusobacterium mortiferum*
anaerobic/37 degree	*Fusobacterium mortiferum*
anaerobic/37 degree	*Fusobacterium mortiferum*
anaerobic/37 degree	*Fusobacterium mortiferum*
anaerobic/37 degree	*Fusobacterium mortiferum*
anaerobic/37 degree	*Fusobacterium mortiferum*
anaerobic/37 degree	*Fusobacterium mortiferum*
anaerobic/37 degree	*Fusobacterium mortiferum*
anaerobic/37 degree	*Fusobacterium mortiferum*

Table S5. The cultured strains and their culture atmospheres

**Atmosphere**	***species***
anaerobic/37 degree	*Fusobacterium mortiferum*
anaerobic/37 degree	*Fusobacterium mortiferum*
anaerobic/37 degree	*Fusobacterium mortiferum*
anaerobic/37 degree	*Fusobacterium mortiferum*
anaerobic/37 degree	*Fusobacterium mortiferum*
anaerobic/37 degree	*Fusobacterium mortiferum*
anaerobic/37 degree	*Fusobacterium mortiferum*
anaerobic/37 degree	*Fusobacterium mortiferum*
anaerobic/37 degree	*Fusobacterium mortiferum*
anaerobic/42 degree	*Fusobacterium mortiferum*
anaerobic/42 degree	*Fusobacterium mortiferum*
anaerobic/37 degree	*Fusobacterium varium*
anaerobic/37 degree	*Fusobacterium varium*
anaerobic/37 degree	*Fusobacterium varium*
anaerobic/35 degree	*Hafnia alvei*
anaerobic/35 degree	*Hafnia alvei*
anaerobic/35 degree	*Hafnia alvei*
anaerobic/35 degree	*Hafnia alvei*
anaerobic/35 degree	*Hafnia alvei*
anaerobic/35 degree	*Hafnia alvei*
anaerobic/35 degree	*Hafnia alvei*
anaerobic/35 degree	*Hafnia alvei*
anaerobic/35 degree	*Hafnia alvei*
anaerobic/35 degree	*Hafnia alvei*
anaerobic/35 degree	*Hafnia alvei*
anaerobic/35 degree	*Hafnia alvei*
anaerobic/35 degree	*Hafnia alvei*
anaerobic/35 degree	*Hafnia alvei*
anaerobic/35 degree	*Hafnia alvei*
anaerobic/35 degree	*Hafnia alvei*
anaerobic/35 degree	*Hafnia alvei*
anaerobic/35 degree	*Hafnia alvei*
anaerobic/35 degree	*Hafnia alvei*
anaerobic/35 degree	*Hafnia alvei*
anaerobic/35 degree	*Hafnia alvei*
anaerobic/35 degree	*Hafnia alvei*
anaerobic/35 degree	*Hafnia alvei*
anaerobic/35 degree	*Hafnia alvei*
anaerobic/35 degree	*Hafnia alvei*
anaerobic/35 degree	*Hafnia alvei*
anaerobic/35 degree	*Hafnia alvei*
anaerobic/35 degree	*Hafnia alvei*
anaerobic/35 degree	*Hafnia alvei*
anaerobic/35 degree	*Hafnia alvei*

Table S5. The cultured strains and their culture atmospheres

**Atmosphere**	***species***
anaerobic/35 degree	*Hafnia alvei*
anaerobic/35 degree	*Hafnia alvei*
anaerobic/35 degree	*Hafnia alvei*
anaerobic/35 degree	*Hafnia alvei*
anaerobic/35 degree	*Hafnia alvei*
anaerobic/35 degree	*Hafnia alvei*
anaerobic/35 degree	*Hafnia alvei*
anaerobic/35 degree	*Hafnia alvei*
anaerobic/35 degree	*Hafnia alvei*
anaerobic/35 degree	*Hafnia alvei*
anaerobic/35 degree	*Hafnia alvei*
anaerobic/35 degree	*Hafnia alvei*
anaerobic/35 degree	*Hafnia alvei*
anaerobic/35 degree	*Hafnia alvei*
anaerobic/35 degree	*Hafnia alvei*
anaerobic/35 degree	*Hafnia alvei*
anaerobic/35 degree	*Hafnia alvei*
anaerobic/35 degree	*Hafnia alvei*
anaerobic/35 degree	*Hafnia alvei*
anaerobic/35 degree	*Hafnia alvei*
anaerobic/35 degree	*Hafnia alvei*
anaerobic/35 degree	*Hafnia alvei*
anaerobic/35 degree	*Hafnia alvei*
anaerobic/35 degree	*Hafnia alvei*
anaerobic/35 degree	*Hafnia alvei*
anaerobic/35 degree	*Hafnia alvei*
anaerobic/35 degree	*Hafnia alvei*
anaerobic/35 degree	*Hafnia alvei*
anaerobic/35 degree	*Hafnia alvei*
anaerobic/35 degree	*Hafnia alvei*
anaerobic/35 degree	*Hafnia alvei*
anaerobic/35 degree	*Hafnia alvei*
anaerobic/35 degree	*Hafnia alvei*
anaerobic/35 degree	*Hafnia alvei*
anaerobic/37 degree	*Hafnia alvei*
anaerobic/37 degree	*Hafnia alvei*
anaerobic/37 degree	*Hafnia alvei*
anaerobic/37 degree	*Hafnia alvei*
anaerobic/37 degree	*Hafnia alvei*
anaerobic/37 degree	*Hafnia alvei*
anaerobic/37 degree	*Hafnia alvei*
anaerobic/37 degree	*Hafnia alvei*
anaerobic/40-50 degree	*Hafnia alvei*
anaerobic/40-50 degree	*Hafnia alvei*

Table S5. The cultured strains and their culture atmospheres

Atmosphere	*species*
anaerobic/42 degree	*Hafnia alvei*
anaerobic/42 degree	*Hafnia alvei*
anaerobic/42 degree	*Hafnia alvei*
anaerobic/42 degree	*Hafnia alvei*
anaerobic/42 degree	*Hafnia alvei*
anaerobic/42 degree	*Hafnia alvei*
anaerobic/42 degree	*Hafnia alvei*
anaerobic/42 degree	*Hafnia alvei*
anaerobic/42 degree	*Hafnia alvei*
anaerobic/42 degree	*Hafnia alvei*
anaerobic/42 degree	*Hafnia alvei*
anaerobic/42 degree	*Hafnia alvei*
anaerobic/42 degree	*Hafnia alvei*
anaerobic/42 degree	*Hafnia alvei*
anaerobic/42 degree	*Hafnia alvei*
anaerobic/42 degree	*Hafnia alvei*
anaerobic/42 degree	*Hafnia alvei*
anaerobic/42 degree	*Hafnia alvei*
anaerobic/42 degree	*Hafnia alvei*
anaerobic/42 degree	*Hafnia alvei*
anaerobic/42 degree	*Hafnia alvei*
anaerobic/42 degree	*Hafnia alvei*
anaerobic/42 degree	*Hafnia alvei*
anaerobic/42 degree	*Hafnia alvei*
anaerobic/42 degree	*Hafnia alvei*
anaerobic/42 degree	*Hafnia alvei*
anaerobic/42 degree	*Hafnia alvei*
anaerobic/42 degree	*Hafnia alvei*
anaerobic/42 degree	*Hafnia alvei*
anaerobic/42 degree	*Hafnia alvei*
anaerobic/42 degree	*Hafnia alvei*
anaerobic/42 degree	*Hafnia alvei*
anaerobic/42 degree	*Hafnia alvei*
anaerobic/42 degree	*Hafnia alvei*
anaerobic/42 degree	*Hafnia alvei*
anaerobic/42 degree	*Hafnia alvei*
anaerobic/42 degree	*Hafnia alvei*
anaerobic/42 degree	*Hafnia alvei*
anaerobic/42 degree	*Hafnia alvei*
anaerobic/42 degree	*Hafnia alvei*
anaerobic/42 degree	*Hafnia alvei*
anaerobic/42 degree	*Hafnia alvei*
anaerobic/42 degree	*Hafnia alvei*
anaerobic/42 degree	*Hafnia alvei*

Table S5. The cultured strains and their culture atmospheres

Atmosphere	*species*
anaerobic/42 degree	*Hafnia alvei*
anaerobic/42 degree	*Hafnia alvei*
anaerobic/42 degree	*Hafnia alvei*
anaerobic/42 degree	*Hafnia alvei*
anaerobic/42 degree	*Hafnia alvei*
anaerobic/42 degree	*Hafnia alvei*
anaerobic/42 degree	*Hafnia alvei*
anaerobic/42 degree	*Hafnia alvei*
anaerobic/42 degree	*Hafnia alvei*
anaerobic/42 degree	*Hafnia alvei*
anaerobic/42 degree	*Hafnia alvei*
anaerobic/42 degree	*Hafnia alvei*
anaerobic/42 degree	*Hafnia alvei*
anaerobic/42 degree	*Hafnia alvei*
anaerobic/42 degree	*Hafnia alvei*
anaerobic/42 degree	*Hafnia alvei*
anaerobic/42 degree	*Hafnia alvei*
anaerobic/42 degree	*Hafnia alvei*
anaerobic/42 degree	*Hafnia alvei*
anaerobic/42 degree	*Hafnia alvei*
anaerobic/42 degree	*Hafnia alvei*
anaerobic/42 degree	*Hafnia alvei*
anaerobic/42 degree	*Hafnia alvei*
anaerobic/42 degree	*Hafnia alvei*
anaerobic/42 degree	*Hafnia alvei*
anaerobic/42 degree	*Hafnia alvei*
anaerobic/42 degree	*Hafnia alvei*
anaerobic/42 degree	*Hafnia alvei*
anaerobic/42 degree	*Hafnia alvei*
anaerobic/42 degree	*Hafnia alvei*
anaerobic/42 degree	*Hafnia alvei*
anaerobic/42 degree	*Hafnia alvei*
anaerobic/42 degree	*Hafnia alvei*
anaerobic/42 degree	*Hafnia alvei*
anaerobic/42 degree	*Hafnia alvei*
anaerobic/42 degree	*Hafnia alvei*
anaerobic/42 degree	*Hafnia alvei*
anaerobic/42 degree	*Hafnia alvei*
anaerobic/42 degree	*Hafnia alvei*
anaerobic/42 degree	*Hafnia alvei*
anaerobic/42 degree	*Hafnia alvei*
anaerobic/42 degree	*Hafnia alvei*
anaerobic/42 degree	*Hafnia alvei*
anaerobic/42 degree	*Hafnia alvei*

Table S5. The cultured strains and their culture atmospheres

**Atmosphere**	***species***
anaerobic/37 degree	*Hungatella hathewayi*
anaerobic/37 degree	*Klebsiella oxytoca*
anaerobic/37 degree	*Klebsiella oxytoca*
anaerobic/37 degree	*Klebsiella oxytoca*
anaerobic/37 degree	*Klebsiella pneumoniae*
anaerobic/37 degree	*Klebsiella pneumoniae*
anaerobic/37 degree	*Klebsiella pneumoniae*
anaerobic/37 degree	*Klebsiella pneumoniae*
anaerobic/37 degree	*Klebsiella pneumoniae*
anaerobic/37 degree	*Klebsiella pneumoniae*
anaerobic/37 degree	*Klebsiella pneumoniae*
anaerobic/37 degree	*Klebsiella Pneumoniae*
anaerobic/37 degree	*Klebsiella pneumoniae*
anaerobic/37 degree	*Klebsiella pneumoniae*
anaerobic/37 degree	*Klebsiella pneumoniae*
anaerobic/37 degree	*Klebsiella pneumoniae*
anaerobic/37 degree	*Klebsiella pneumoniae*
anaerobic/37 degree	*Klebsiella pneumoniae*
anaerobic/37 degree	*Klebsiella pneumoniae*
anaerobic/37 degree	*Klebsiella pneumoniae*
anaerobic/37 degree	*Klebsiella pneumoniae*
anaerobic/37 degree	*Klebsiella pneumoniae*
anaerobic/37 degree	*Klebsiella pneumoniae*
anaerobic/37 degree	*Klebsiella pneumoniae*
anaerobic/37 degree	*Klebsiella pneumoniae*
anaerobic/37 degree	*Klebsiella pneumoniae*
anaerobic/37 degree	*Klebsiella pneumoniae*
anaerobic/37 degree	*Klebsiella pneumoniae*
anaerobic/37 degree	*Klebsiella pneumoniae*
anaerobic/37 degree	*Klebsiella pneumoniae*
anaerobic/37 degree	*Klebsiella pneumoniae*
anaerobic/37 degree	*Klebsiella pneumoniae*
anaerobic/37 degree	*Klebsiella pneumoniae*
anaerobic/37 degree	*Klebsiella pneumoniae*
anaerobic/37 degree	*Klebsiella pneumoniae*
anaerobic/37 degree	*Klebsiella pneumoniae*
anaerobic/37 degree	*Klebsiella pneumoniae*
anaerobic/37 degree	*Klebsiella pneumoniae*
anaerobic/37 degree	*Klebsiella pneumoniae*
anaerobic/37 degree	*Klebsiella pneumoniae*
anaerobic/37 degree	*Klebsiella pneumoniae*
anaerobic/37 degree	*Klebsiella pneumoniae*
anaerobic/37 degree	*Klebsiella pneumoniae*
anaerobic/37 degree	*Klebsiella pneumoniae*

Table S5. The cultured strains and their culture atmospheres

**Atmosphere**	***species***
anaerobic/37 degree	*Klebsiella pneumoniae*
anaerobic/37 degree	*Klebsiella pneumoniae*
anaerobic/37 degree	*Klebsiella pneumoniae*
anaerobic/37 degree	*Klebsiella pneumoniae*
anaerobic/37 degree	*Klebsiella pneumoniae*
anaerobic/37 degree	*Klebsiella Pneumoniae*
anaerobic/37 degree	*Klebsiella pneumoniae*
anaerobic/37 degree	*Klebsiella pneumoniae*
anaerobic/37 degree	*Klebsiella pneumoniae*
anaerobic/37 degree	*Klebsiella pneumoniae*
anaerobic/37 degree	*Klebsiella pneumoniae*
anaerobic/37 degree	*Klebsiella pneumoniae*
anaerobic/37 degree	*Klebsiella pneumoniae*
anaerobic/37 degree	*Klebsiella pneumoniae*
anaerobic/37 degree	*Klebsiella pneumoniae*
anaerobic/37 degree	*Klebsiella pneumoniae*
anaerobic/42 degree	*Klebsiella pneumoniae*
anaerobic/42 degree	*Klebsiella pneumoniae*
anaerobic/42 degree	*Klebsiella pneumoniae*
anaerobic/42 degree	*Klebsiella pneumoniae*
anaerobic/42 degree	*Klebsiella pneumoniae*
anaerobic/42 degree	*Klebsiella pneumoniae*
anaerobic/42 degree	*Klebsiella pneumoniae*
anaerobic/42 degree	*Klebsiella pneumoniae subsp.ozaenae*
anaerobic/37 degree	*Klebsiella quasipneumoniae*
anaerobic/37 degree	*Klebsiella quasipneumoniae*
anaerobic/37 degree	*Klebsiella quasipneumoniae*
anaerobic/37 degree	*Klebsiella variicola*
anaerobic/37 degree	*Kocuria kristinae*
anaerobic/37 degree	*Kocuria kristinae*
anaerobic/37 degree	*Lachnospiraceae bacterium_3_1_57FAA_CT1*
anaerobic/37 degree	*Lachnospiraceae bacterium_3_1_57FAA_CT1*
anaerobic/37 degree	*Lactobacillus agilis*
anaerobic/37 degree	*Lactobacillus agilis*
anaerobic/37 degree	*Lactobacillus agilis*
anaerobic/37 degree	*Lactobacillus agilis*
anaerobic/37 degree	*Lactobacillus agilis*
anaerobic/37 degree	*Lactobacillus agilis*
anaerobic/37 degree	*Lactobacillus agilis*
anaerobic/37 degree	*Lactobacillus agilis*
anaerobic/37 degree	*Lactobacillus agilis*
anaerobic/37 degree	*Lactobacillus agilis*
anaerobic/37 degree	*Lactobacillus agilis*
anaerobic/37 degree	*Lactobacillus agilis*

Table S5. The cultured strains and their culture atmospheres

**Atmosphere**	***species***
anaerobic/37 degree	*Lactobacillus agilis*
anaerobic/37 degree	*Lactobacillus agilis*
anaerobic/37 degree	*Lactobacillus agilis*
anaerobic/37 degree	*Lactobacillus agilis*
anaerobic/37 degree	*Lactobacillus agilis*
anaerobic/37 degree	*Lactobacillus agilis*
anaerobic/37 degree	*Lactobacillus agilis*
anaerobic/37 degree	*Lactobacillus agilis*
anaerobic/37 degree	*Lactobacillus agilis*
anaerobic/37 degree	*Lactobacillus agilis*
anaerobic/37 degree	*Lactobacillus agilis*
anaerobic/37 degree	*Lactobacillus agilis*
anaerobic/37 degree	*Lactobacillus agilis*
anaerobic/37 degree	*Lactobacillus agilis*
anaerobic/37 degree	*Lactobacillus agilis*
anaerobic/37 degree	*Lactobacillus agilis*
anaerobic/37 degree	*Lactobacillus agilis*
anaerobic/37 degree	*Lactobacillus agilis*
anaerobic/37 degree	*Lactobacillus agilis*
anaerobic/37 degree	*Lactobacillus agilis*
anaerobic/37 degree	*Lactobacillus agilis*
anaerobic/37 degree	*Lactobacillus agilis*
anaerobic/37 degree	*Lactobacillus agilis*
anaerobic/37 degree	*Lactobacillus agilis*
anaerobic/37 degree	*Lactobacillus agilis*
anaerobic/37 degree	*Lactobacillus agilis*
anaerobic/37 degree	*Lactobacillus agilis*
anaerobic/37 degree	*Lactobacillus agilis*
anaerobic/42 degree	*Lactobacillus agilis*
anaerobic/42 degree	*Lactobacillus agilis*
anaerobic/42 degree	*Lactobacillus agilis*
anaerobic/42 degree	*Lactobacillus agilis*
anaerobic/42 degree	*Lactobacillus agilis*
anaerobic/42 degree	*Lactobacillus agilis*
anaerobic/42 degree	*Lactobacillus agilis*
anaerobic/42 degree	*Lactobacillus agilis*
anaerobic/42 degree	*Lactobacillus agilis*
anaerobic/42 degree	*Lactobacillus agilis*
anaerobic/42 degree	*Lactobacillus agilis*
anaerobic/42 degree	*Lactobacillus agilis*
anaerobic/42 degree	*Lactobacillus agilis*
anaerobic/42 degree	*Lactobacillus agilis*
anaerobic/42 degree	*Lactobacillus agilis*
anaerobic/42 degree	*Lactobacillus agilis*

Table S5. The cultured strains and their culture atmospheres

Atmosphere	*species*
anaerobic/42 degree	*Lactobacillus agilis*
anaerobic/42 degree	*Lactobacillus agilis*
anaerobic/42 degree	*Lactobacillus agilis*
anaerobic/42 degree	*Lactobacillus agilis*
anaerobic/42 degree	*Lactobacillus agilis*
anaerobic/42 degree	*Lactobacillus agilis*
anaerobic/42 degree	*Lactobacillus agilis*
anaerobic/42 degree	*Lactobacillus agilis*
anaerobic/42 degree	*Lactobacillus agilis*
anaerobic/42 degree	*Lactobacillus agilis*
anaerobic/42 degree	*Lactobacillus agilis*
anaerobic/42 degree	*Lactobacillus agilis*
anaerobic/42 degree	*Lactobacillus agilis*
anaerobic/42 degree	*Lactobacillus agilis*
anaerobic/42 degree	*Lactobacillus agilis*
anaerobic/42 degree	*Lactobacillus agilis*
anaerobic/42 degree	*Lactobacillus agilis*
anaerobic/42 degree	*Lactobacillus agilis*
anaerobic/42 degree	*Lactobacillus agilis*
anaerobic/42 degree	*Lactobacillus agilis*
anaerobic/37 degree	*Lactobacillus amylovorus*
anaerobic/37 degree	*Lactobacillus amylovorus*
anaerobic/37 degree	*Lactobacillus amylovorus*
anaerobic/37 degree	*Lactobacillus amylovorus*
anaerobic/37 degree	*Lactobacillus amylovorus*
anaerobic/37 degree	*Lactobacillus antri*
anaerobic/42 degree	*Lactobacillus antri*
anaerobic/37 degree	*Lactobacillus brevis*
anaerobic/37 degree	*Lactobacillus brevis*
anaerobic/42 degree	*Lactobacillus brevis*
anaerobic/35 degree	*Lactobacillus casei paracasei*
anaerobic/35 degree	*Lactobacillus casei paracasei*
anaerobic/35 degree	*Lactobacillus casei paracasei*
anaerobic/35 degree	*Lactobacillus casei paracasei*
anaerobic/35 degree	*Lactobacillus casei paracasei*
anaerobic/35 degree	*Lactobacillus casei paracasei*
anaerobic/35 degree	*Lactobacillus casei paracasei*
anaerobic/35 degree	*Lactobacillus casei paracasei*
anaerobic/35 degree	*Lactobacillus casei paracasei*
anaerobic/37 degree	*Lactobacillus casei paracasei*
anaerobic/37 degree	*Lactobacillus casei paracasei*
anaerobic/37 degree	*Lactobacillus casei paracasei*
anaerobic/37 degree	*Lactobacillus casei paracasei*
anaerobic/37 degree	*Lactobacillus casei paracasei*

Table S5. The cultured strains and their culture atmospheres

**Atmosphere**	***species***
anaerobic/37 degree	*Lactobacillus casei paracasei*
anaerobic/37 degree	*Lactobacillus casei paracasei*
anaerobic/37 degree	*Lactobacillus casei paracasei*
anaerobic/37 degree	*Lactobacillus casei paracasei*
anaerobic/37 degree	*Lactobacillus casei paracasei*
anaerobic/37 degree	*Lactobacillus casei paracasei*
anaerobic/37 degree	*Lactobacillus casei paracasei*
anaerobic/37 degree	*Lactobacillus casei paracasei*
anaerobic/37 degree	*Lactobacillus casei paracasei*
anaerobic/37 degree	*Lactobacillus casei paracasei*
anaerobic/37 degree	*Lactobacillus casei paracasei*
anaerobic/37 degree	*Lactobacillus casei paracasei*
anaerobic/37 degree	*Lactobacillus casei paracasei*
anaerobic/37 degree	*Lactobacillus casei paracasei*
anaerobic/37 degree	*Lactobacillus casei paracasei*
anaerobic/37 degree	*Lactobacillus casei paracasei*
anaerobic/37 degree	*Lactobacillus casei paracasei*
anaerobic/37 degree	*Lactobacillus casei paracasei*
anaerobic/37 degree	*Lactobacillus casei paracasei*
anaerobic/37 degree	*Lactobacillus casei paracasei*
anaerobic/37 degree	*Lactobacillus casei paracasei*
anaerobic/37 degree	*Lactobacillus casei paracasei*
anaerobic/37 degree	*Lactobacillus casei paracasei*
anaerobic/37 degree	*Lactobacillus casei paracasei*
anaerobic/37 degree	*Lactobacillus casei paracasei*
anaerobic/37 degree	*Lactobacillus casei paracasei*
anaerobic/37 degree	*Lactobacillus casei paracasei*
anaerobic/37 degree	*Lactobacillus casei paracasei*
anaerobic/37 degree	*Lactobacillus casei paracasei*
anaerobic/37 degree	*Lactobacillus casei paracasei*
anaerobic/37 degree	*Lactobacillus casei paracasei*
anaerobic/37 degree	*Lactobacillus casei paracasei*
anaerobic/37 degree	*Lactobacillus casei paracasei*
anaerobic/37 degree	*Lactobacillus casei paracasei*
anaerobic/37 degree	*Lactobacillus casei paracasei*
anaerobic/37 degree	*Lactobacillus casei paracasei*
anaerobic/42 degree	*Lactobacillus casei paracasei*
anaerobic/42 degree	*Lactobacillus casei paracasei*
anaerobic/42 degree	*Lactobacillus casei paracasei*
anaerobic/42 degree	*Lactobacillus casei paracasei*
anaerobic/42 degree	*Lactobacillus casei paracasei*
anaerobic/42 degree	*Lactobacillus casei paracasei*
anaerobic/42 degree	*Lactobacillus casei paracasei*
anaerobic/42 degree	*Lactobacillus casei paracasei*

Table S5. The cultured strains and their culture atmospheres

**Atmosphere**	***species***
anaerobic/37 degree	*Lactobacillus crispatus*
anaerobic/37 degree	*Lactobacillus crispatus*
anaerobic/37 degree	*Lactobacillus crispatus*
anaerobic/37 degree	*Lactobacillus crispatus*
anaerobic/37 degree	*Lactobacillus crispatus*
anaerobic/37 degree	*Lactobacillus crispatus*
anaerobic/37 degree	*Lactobacillus crispatus*
anaerobic/37 degree	*Lactobacillus crispatus*
anaerobic/37 degree	*Lactobacillus crispatus*
anaerobic/37 degree	*Lactobacillus crispatus*
anaerobic/37 degree	*Lactobacillus crispatus*
anaerobic/37 degree	*Lactobacillus crispatus*
anaerobic/37 degree	*Lactobacillus crispatus*
anaerobic/37 degree	*Lactobacillus crispatus*
anaerobic/37 degree	*Lactobacillus crispatus*
anaerobic/37 degree	*Lactobacillus crispatus*
anaerobic/37 degree	*Lactobacillus crispatus*
anaerobic/37 degree	*Lactobacillus delbrueckii*
anaerobic/37 degree	*Lactobacillus delbrueckii*
anaerobic/37 degree	*Lactobacillus delbrueckii*
anaerobic/37 degree	*Lactobacillus delbrueckii*
anaerobic/37 degree	*Lactobacillus delbrueckii*
anaerobic/37 degree	*Lactobacillus delbrueckii*
anaerobic/37 degree	*Lactobacillus delbrueckii*
anaerobic/37 degree	*Lactobacillus delbrueckii*
anaerobic/37 degree	*Lactobacillus delbrueckii*
anaerobic/37 degree	*Lactobacillus delbrueckii*
anaerobic/37 degree	*Lactobacillus delbrueckii*
anaerobic/37 degree	*Lactobacillus delbrueckii*
anaerobic/42 degree	*Lactobacillus delbrueckii*
anaerobic/42 degree	*Lactobacillus delbrueckii*
anaerobic/35 degree	*Lactobacillus fermentum*
anaerobic/35 degree	*Lactobacillus fermentum*
anaerobic/35 degree	*Lactobacillus fermentum*
anaerobic/37 degree	*Lactobacillus fermentum*
anaerobic/37 degree	*Lactobacillus fermentum*
anaerobic/37 degree	*Lactobacillus fermentum*
anaerobic/37 degree	*Lactobacillus fermentum*
anaerobic/37 degree	*Lactobacillus fermentum*
anaerobic/37 degree	*Lactobacillus fermentum*
anaerobic/37 degree	*Lactobacillus fermentum*
anaerobic/37 degree	*Lactobacillus fermentum*
anaerobic/37 degree	*Lactobacillus fermentum*
anaerobic/37 degree	*Lactobacillus fermentum*

Table S5. The cultured strains and their culture atmospheres

**Atmosphere**	***species***
anaerobic/37 degree	*Lactobacillus fermentum*
anaerobic/37 degree	*Lactobacillus fermentum*
anaerobic/37 degree	*Lactobacillus fermentum*
anaerobic/37 degree	*Lactobacillus fermentum*
anaerobic/37 degree	*Lactobacillus fermentum*
anaerobic/37 degree	*Lactobacillus fermentum*
anaerobic/37 degree	*Lactobacillus fermentum*
anaerobic/37 degree	*Lactobacillus fermentum*
anaerobic/37 degree	*Lactobacillus fermentum*
anaerobic/37 degree	*Lactobacillus fermentum*
anaerobic/37 degree	*Lactobacillus fermentum*
anaerobic/37 degree	*Lactobacillus fermentum*
anaerobic/37 degree	*Lactobacillus fermentum*
anaerobic/37 degree	*Lactobacillus fermentum*
anaerobic/37 degree	*Lactobacillus fermentum*
anaerobic/37 degree	*Lactobacillus fermentum*
anaerobic/37 degree	*Lactobacillus fermentum*
anaerobic/37 degree	*Lactobacillus fermentum*
anaerobic/37 degree	*Lactobacillus fermentum*
anaerobic/37 degree	*Lactobacillus fermentum*
anaerobic/37 degree	*Lactobacillus fermentum*
anaerobic/37 degree	*Lactobacillus fermentum*
anaerobic/37 degree	*Lactobacillus fermentum*
anaerobic/37 degree	*Lactobacillus fermentum*
anaerobic/37 degree	*Lactobacillus fermentum*
anaerobic/37 degree	*Lactobacillus fermentum*
anaerobic/37 degree	*Lactobacillus fermentum*
anaerobic/37 degree	*Lactobacillus fermentum*
anaerobic/37 degree	*Lactobacillus fermentum*
anaerobic/37 degree	*Lactobacillus fermentum*
anaerobic/37 degree	*Lactobacillus fermentum*
anaerobic/37 degree	*Lactobacillus fermentum*
anaerobic/37 degree	*Lactobacillus fermentum*
anaerobic/37 degree	*Lactobacillus fermentum*
anaerobic/37 degree	*Lactobacillus fermentum*
anaerobic/37 degree	*Lactobacillus fermentum*
anaerobic/37 degree	*Lactobacillus fermentum*
anaerobic/37 degree	*Lactobacillus fermentum*
anaerobic/37 degree	*Lactobacillus fermentum*
anaerobic/37 degree	*Lactobacillus fermentum*
anaerobic/37 degree	*Lactobacillus fermentum*
anaerobic/37 degree	*Lactobacillus fermentum*
anaerobic/37 degree	*Lactobacillus fermentum*
anaerobic/37 degree	*Lactobacillus fermentum*

Table S5. The cultured strains and their culture atmospheres

**Atmosphere**	***species***
anaerobic/37 degree	*Lactobacillus fermentum*
anaerobic/37 degree	*Lactobacillus fermentum*
anaerobic/37 degree	*Lactobacillus fermentum*
anaerobic/37 degree	*Lactobacillus fermentum*
anaerobic/37 degree	*Lactobacillus fermentum*
anaerobic/37 degree	*Lactobacillus fermentum*
anaerobic/37 degree	*Lactobacillus fermentum*
anaerobic/37 degree	*Lactobacillus fermentum*
anaerobic/37 degree	*Lactobacillus fermentum*
anaerobic/37 degree	*Lactobacillus fermentum*
anaerobic/37 degree	*Lactobacillus fermentum*
anaerobic/37 degree	*Lactobacillus fermentum*
anaerobic/37 degree	*Lactobacillus fermentum*
anaerobic/37 degree	*Lactobacillus fermentum*
anaerobic/37 degree	*Lactobacillus fermentum*
anaerobic/37 degree	*Lactobacillus fermentum*
anaerobic/37 degree	*Lactobacillus fermentum*
anaerobic/37 degree	*Lactobacillus fermentum*
anaerobic/37 degree	*Lactobacillus fermentum*
anaerobic/37 degree	*Lactobacillus fermentum*
anaerobic/37 degree	*Lactobacillus fermentum*
anaerobic/37 degree	*Lactobacillus fermentum*
anaerobic/37 degree	*Lactobacillus fermentum*
anaerobic/37 degree	*Lactobacillus fermentum*
anaerobic/37 degree	*Lactobacillus fermentum*
anaerobic/37 degree	*Lactobacillus fermentum*
anaerobic/37 degree	*Lactobacillus fermentum*
anaerobic/37 degree	*Lactobacillus fermentum*
anaerobic/37 degree	*Lactobacillus fermentum*
anaerobic/37 degree	*Lactobacillus fermentum*
anaerobic/37 degree	*Lactobacillus fermentum*
anaerobic/37 degree	*Lactobacillus fermentum*
anaerobic/37 degree	*Lactobacillus fermentum*
anaerobic/37 degree	*Lactobacillus fermentum*
anaerobic/37 degree	*Lactobacillus fermentum*
anaerobic/37 degree	*Lactobacillus fermentum*
anaerobic/37 degree	*Lactobacillus fermentum*
anaerobic/37 degree	*Lactobacillus fermentum*
anaerobic/37 degree	*Lactobacillus fermentum*
anaerobic/37 degree	*Lactobacillus fermentum*
anaerobic/37 degree	*Lactobacillus fermentum*
anaerobic/37 degree	*Lactobacillus fermentum*
anaerobic/37 degree	*Lactobacillus fermentum*
anaerobic/37 degree	*Lactobacillus fermentum*

Table S5. The cultured strains and their culture atmospheres

**Atmosphere**	***species***
anaerobic/37 degree	*Lactobacillus fermentum*
anaerobic/37 degree	*Lactobacillus fermentum*
anaerobic/37 degree	*Lactobacillus fermentum*
anaerobic/37 degree	*Lactobacillus fermentum*
anaerobic/37 degree	*Lactobacillus fermentum*
anaerobic/37 degree	*Lactobacillus fermentum*
anaerobic/37 degree	*Lactobacillus fermentum*
anaerobic/37 degree	*Lactobacillus fermentum*
anaerobic/37 degree	*Lactobacillus fermentum*
anaerobic/37 degree	*Lactobacillus fermentum*
anaerobic/37 degree	*Lactobacillus fermentum*
anaerobic/37 degree	*Lactobacillus fermentum*
anaerobic/37 degree	*Lactobacillus fermentum*
anaerobic/37 degree	*Lactobacillus fermentum*
anaerobic/37 degree	*Lactobacillus fermentum*
anaerobic/37 degree	*Lactobacillus fermentum*
anaerobic/37 degree	*Lactobacillus fermentum*
anaerobic/37 degree	*Lactobacillus fermentum*
anaerobic/37 degree	*Lactobacillus fermentum*
anaerobic/37 degree	*Lactobacillus fermentum*
anaerobic/42 degree	*Lactobacillus fermentum*
anaerobic/42 degree	*Lactobacillus fermentum*
anaerobic/42 degree	*Lactobacillus fermentum*
anaerobic/42 degree	*Lactobacillus fermentum*
anaerobic/42 degree	*Lactobacillus fermentum*
anaerobic/42 degree	*Lactobacillus fermentum*
anaerobic/42 degree	*Lactobacillus fermentum*
anaerobic/42 degree	*Lactobacillus fermentum*
anaerobic/42 degree	*Lactobacillus fermentum*
anaerobic/42 degree	*Lactobacillus fermentum*
anaerobic/42 degree	*Lactobacillus fermentum*
anaerobic/42 degree	*Lactobacillus fermentum*
anaerobic/42 degree	*Lactobacillus fermentum*
anaerobic/42 degree	*Lactobacillus fermentum*
anaerobic/42 degree	*Lactobacillus fermentum*
anaerobic/42 degree	*Lactobacillus fermentum*
anaerobic/42 degree	*Lactobacillus fermentum*
anaerobic/42 degree	*Lactobacillus fermentum*
anaerobic/42 degree	*Lactobacillus fermentum*
anaerobic/42 degree	*Lactobacillus fermentum*
anaerobic/42 degree	*Lactobacillus fermentum*
anaerobic/42 degree	*Lactobacillus fermentum*
anaerobic/42 degree	*Lactobacillus fermentum*
anaerobic/42 degree	*Lactobacillus fermentum*

Table S5. The cultured strains and their culture atmospheres

**Atmosphere**	***species***
anaerobic/42 degree	*Lactobacillus fermentum*
anaerobic/42 degree	*Lactobacillus fermentum*
anaerobic/42 degree	*Lactobacillus fermentum*
anaerobic/42 degree	*Lactobacillus fermentum*
anaerobic/42 degree	*Lactobacillus fermentum*
anaerobic/42 degree	*Lactobacillus fermentum*
anaerobic/42 degree	*Lactobacillus fermentum*
anaerobic/42 degree	*Lactobacillus fermentum*
anaerobic/42 degree	*Lactobacillus fermentum*
anaerobic/42 degree	*Lactobacillus fermentum*
anaerobic/42 degree	*Lactobacillus fermentum*
anaerobic/42 degree	*Lactobacillus fermentum*
anaerobic/42 degree	*Lactobacillus fermentum*
anaerobic/42 degree	*Lactobacillus fermentum*
anaerobic/42 degree	*Lactobacillus fermentum*
anaerobic/42 degree	*Lactobacillus fermentum*
anaerobic/42 degree	*Lactobacillus fermentum*
anaerobic/42 degree	*Lactobacillus fermentum*
anaerobic/42 degree	*Lactobacillus fermentum*
anaerobic/42 degree	*Lactobacillus fermentum*
anaerobic/42 degree	*Lactobacillus fermentum*
anaerobic/42 degree	*Lactobacillus fermentum*
anaerobic/42 degree	*Lactobacillus fermentum*
anaerobic/42 degree	*Lactobacillus fermentum*
anaerobic/42 degree	*Lactobacillus fermentum*
anaerobic/42 degree	*Lactobacillus fermentum*
anaerobic/42 degree	*Lactobacillus fermentum*
anaerobic/42 degree	*Lactobacillus fermentum*
anaerobic/42 degree	*Lactobacillus fermentum*
anaerobic/42 degree	*Lactobacillus fermentum*
anaerobic/42 degree	*Lactobacillus fermentum*
anaerobic/42 degree	*Lactobacillus fermentum*
anaerobic/42 degree	*Lactobacillus fermentum*
anaerobic/42 degree	*Lactobacillus fermentum*
anaerobic/42 degree	*Lactobacillus fermentum*
anaerobic/42 degree	*Lactobacillus fermentum*
anaerobic/42 degree	*Lactobacillus fermentum*
anaerobic/42 degree	*Lactobacillus fermentum*
anaerobic/42 degree	*Lactobacillus fermentum*
anaerobic/42 degree	*Lactobacillus fermentum*
anaerobic/42 degree	*Lactobacillus fermentum*
anaerobic/42 degree	*Lactobacillus fermentum*
anaerobic/42 degree	*Lactobacillus fermentum*
anaerobic/42 degree	*Lactobacillus fermentum*

Table S5. The cultured strains and their culture atmospheres

**Atmosphere**	***species***
anaerobic/42 degree	*Lactobacillus fermentum*
anaerobic/35 degree	*Lactobacillus gasseri*
anaerobic/35 degree	*Lactobacillus gasseri*
anaerobic/35 degree	*Lactobacillus gasseri*
anaerobic/35 degree	*Lactobacillus gasseri*
anaerobic/37 degree	*Lactobacillus gasseri*
anaerobic/37 degree	*Lactobacillus gasseri*
anaerobic/37 degree	*Lactobacillus gasseri*
anaerobic/37 degree	*Lactobacillus gasseri*
anaerobic/37 degree	*Lactobacillus gasseri*
anaerobic/37 degree	*Lactobacillus gasseri*
anaerobic/37 degree	*Lactobacillus gasseri*
anaerobic/37 degree	*Lactobacillus gasseri*
anaerobic/37 degree	*Lactobacillus gasseri*
anaerobic/37 degree	*Lactobacillus gasseri*
anaerobic/37 degree	*Lactobacillus gasseri*
anaerobic/37 degree	*Lactobacillus gasseri*
anaerobic/37 degree	*Lactobacillus gasseri*
anaerobic/37 degree	*Lactobacillus gasseri*
anaerobic/37 degree	*Lactobacillus gasseri*
anaerobic/37 degree	*Lactobacillus gasseri*
anaerobic/37 degree	*Lactobacillus gasseri*
anaerobic/37 degree	*Lactobacillus gasseri*
anaerobic/37 degree	*Lactobacillus gasseri*
anaerobic/37 degree	*Lactobacillus gasseri*
anaerobic/37 degree	*Lactobacillus gasseri*
anaerobic/37 degree	*Lactobacillus gasseri*
anaerobic/37 degree	*Lactobacillus gasseri*
anaerobic/37 degree	*Lactobacillus gasseri*
anaerobic/37 degree	*Lactobacillus gasseri*
anaerobic/37 degree	*Lactobacillus gasseri*
anaerobic/37 degree	*Lactobacillus gasseri*
anaerobic/37 degree	*Lactobacillus gasseri*
anaerobic/37 degree	*Lactobacillus gasseri*
anaerobic/37 degree	*Lactobacillus gasseri*
anaerobic/37 degree	*Lactobacillus gasseri*
anaerobic/37 degree	*Lactobacillus gasseri*
anaerobic/37 degree	*Lactobacillus gasseri*
anaerobic/37 degree	*Lactobacillus gasseri*
anaerobic/37 degree	*Lactobacillus gasseri*
anaerobic/37 degree	*Lactobacillus gasseri*
anaerobic/37 degree	*Lactobacillus gasseri*
anaerobic/37 degree	*Lactobacillus gasseri*
anaerobic/37 degree	*Lactobacillus gasseri*
anaerobic/37 degree	*Lactobacillus gasseri*

Table S5. The cultured strains and their culture atmospheres

Atmosphere	***species***
anaerobic/37 degree	*Lactobacillus gasseri*
anaerobic/37 degree	*Lactobacillus gasseri*
anaerobic/37 degree	*Lactobacillus gasseri*
anaerobic/37 degree	*Lactobacillus gasseri*
anaerobic/37 degree	*Lactobacillus gasseri*
anaerobic/37 degree	*Lactobacillus gasseri*
anaerobic/37 degree	*Lactobacillus gasseri*
anaerobic/37 degree	*Lactobacillus gasseri*
anaerobic/37 degree	*Lactobacillus gasseri*
anaerobic/37 degree	*Lactobacillus gasseri*
anaerobic/37 degree	*Lactobacillus gasseri*
anaerobic/37 degree	*Lactobacillus gasseri*
anaerobic/37 degree	*Lactobacillus gasseri*
anaerobic/42 degree	*Lactobacillus gasseri*
anaerobic/42 degree	*Lactobacillus gasseri*
anaerobic/42 degree	*Lactobacillus gasseri*
anaerobic/42 degree	*Lactobacillus gasseri*
anaerobic/42 degree	*Lactobacillus gasseri*
anaerobic/42 degree	*Lactobacillus gasseri*
anaerobic/37 degree	*Lactobacillus gastricus*
anaerobic/37 degree	*Lactobacillus johnsonii*
anaerobic/37 degree	*Lactobacillus johnsonii*
anaerobic/37 degree	*Lactobacillus johnsonii*
anaerobic/37 degree	*Lactobacillus johnsonii*
anaerobic/37 degree	*Lactobacillus johnsonii*
anaerobic/37 degree	*Lactobacillus johnsonii*
anaerobic/37 degree	*Lactobacillus johnsonii*
anaerobic/37 degree	*Lactobacillus johnsonii*
anaerobic/37 degree	*Lactobacillus johnsonii*
anaerobic/37 degree	*Lactobacillus johnsonii*
anaerobic/37 degree	*Lactobacillus johnsonii*
anaerobic/37 degree	*Lactobacillus johnsonii*
anaerobic/37 degree	*Lactobacillus johnsonii*
anaerobic/37 degree	*Lactobacillus johnsonii*
anaerobic/37 degree	*Lactobacillus johnsonii*
anaerobic/37 degree	*Lactobacillus johnsonii*
anaerobic/37 degree	*Lactobacillus johnsonii*
anaerobic/37 degree	*Lactobacillus johnsonii*
anaerobic/37 degree	*Lactobacillus johnsonii*
anaerobic/37 degree	*Lactobacillus johnsonii*
anaerobic/37 degree	*Lactobacillus johnsonii*
anaerobic/37 degree	*Lactobacillus johnsonii*
anaerobic/37 degree	*Lactobacillus johnsonii*
anaerobic/42 degree	*Lactobacillus johnsonii*

Table S5. The cultured strains and their culture atmospheres

**Atmosphere**	***species***
anaerobic/42 degree	*Lactobacillus johnsonii*
anaerobic/42 degree	*Lactobacillus johnsonii*
anaerobic/42 degree	*Lactobacillus johnsonii*
anaerobic/42 degree	*Lactobacillus johnsonii*
anaerobic/42 degree	*Lactobacillus johnsonii*
anaerobic/42 degree	*Lactobacillus johnsonii*
anaerobic/42 degree	*Lactobacillus johnsonii*
anaerobic/42 degree	*Lactobacillus johnsonii*
anaerobic/42 degree	*Lactobacillus johnsonii*
anaerobic/42 degree	*Lactobacillus johnsonii*
anaerobic/42 degree	*Lactobacillus johnsonii*
anaerobic/42 degree	*Lactobacillus johnsonii*
anaerobic/42 degree	*Lactobacillus johnsonii*
anaerobic/42 degree	*Lactobacillus johnsonii*
anaerobic/37 degree	*Lactobacillus kalixensis*
anaerobic/37 degree	*Lactobacillus kalixensis*
anaerobic/37 degree	*Lactobacillus kalixensis*
anaerobic/37 degree	*Lactobacillus kalixensis*
anaerobic/37 degree	*Lactobacillus kalixensis*
anaerobic/37 degree	*Lactobacillus kalixensis*
anaerobic/37 degree	*Lactobacillus kalixensis*
anaerobic/37 degree	*Lactobacillus kalixensis*
anaerobic/37 degree	*Lactobacillus kalixensis*
anaerobic/37 degree	*Lactobacillus kalixensis*
anaerobic/37 degree	*Lactobacillus kalixensis*
anaerobic/37 degree	*Lactobacillus kalixensis*
anaerobic/37 degree	*Lactobacillus kalixensis*
anaerobic/37 degree	*Lactobacillus kalixensis*
anaerobic/37 degree	*Lactobacillus kalixensis*
anaerobic/37 degree	*Lactobacillus kalixensis*
anaerobic/37 degree	*Lactobacillus kalixensis*
anaerobic/37 degree	*Lactobacillus kalixensis*
anaerobic/37 degree	*Lactobacillus kalixensis*
anaerobic/37 degree	*Lactobacillus kalixensis*
anaerobic/37 degree	*Lactobacillus kalixensis*
anaerobic/37 degree	*Lactobacillus kalixensis*
anaerobic/37 degree	*Lactobacillus kalixensis*
anaerobic/37 degree	*Lactobacillus kalixensis*
anaerobic/37 degree	*Lactobacillus kalixensis*
anaerobic/37 degree	*Lactobacillus kalixensis*
anaerobic/37 degree	*Lactobacillus kalixensis*
anaerobic/37 degree	*Lactobacillus kalixensis*
anaerobic/37 degree	*Lactobacillus kalixensis*
anaerobic/37 degree	*Lactobacillus kalixensis*

Table S5. The cultured strains and their culture atmospheres

**Atmosphere**	***species***
anaerobic/37 degree	*Lactobacillus kalixensis*
anaerobic/37 degree	*Lactobacillus kalixensis*
anaerobic/37 degree	*Lactobacillus kalixensis*
anaerobic/37 degree	*Lactobacillus kalixensis*
anaerobic/37 degree	*Lactobacillus kalixensis*
anaerobic/37 degree	*Lactobacillus kalixensis*
anaerobic/37 degree	*Lactobacillus kalixensis*
anaerobic/42 degree	*Lactobacillus kalixensis*
anaerobic/42 degree	*Lactobacillus kalixensis*
anaerobic/42 degree	*Lactobacillus kalixensis*
anaerobic/42 degree	*Lactobacillus kalixensis*
anaerobic/42 degree	*Lactobacillus kalixensis*
anaerobic/42 degree	*Lactobacillus kalixensis*
anaerobic/42 degree	*Lactobacillus kalixensis*
anaerobic/42 degree	*Lactobacillus kalixensis*
anaerobic/42 degree	*Lactobacillus kalixensis*
anaerobic/42 degree	*Lactobacillus kalixensis*
anaerobic/42 degree	*Lactobacillus kalixensis*
anaerobic/42 degree	*Lactobacillus kalixensis*
anaerobic/42 degree	*Lactobacillus kalixensis*
anaerobic/42 degree	*Lactobacillus kalixensis*
anaerobic/42 degree	*Lactobacillus kalixensis*
anaerobic/42 degree	*Lactobacillus kalixensis*
anaerobic/42 degree	*Lactobacillus kalixensis*
anaerobic/42 degree	*Lactobacillus kalixensis*
anaerobic/42 degree	*Lactobacillus kalixensis*
anaerobic/42 degree	*Lactobacillus kalixensis*
anaerobic/37 degree	*Lactobacillus mucosae*
anaerobic/37 degree	*Lactobacillus mucosae*
anaerobic/37 degree	*Lactobacillus mucosae*
anaerobic/37 degree	*Lactobacillus mucosae*
anaerobic/37 degree	*Lactobacillus mucosae*
anaerobic/37 degree	*Lactobacillus mucosae*
anaerobic/37 degree	*Lactobacillus mucosae*
anaerobic/37 degree	*Lactobacillus mucosae*
anaerobic/37 degree	*Lactobacillus mucosae*
anaerobic/37 degree	*Lactobacillus mucosae*
anaerobic/37 degree	*Lactobacillus mucosae*
anaerobic/37 degree	*Lactobacillus mucosae*
anaerobic/37 degree	*Lactobacillus mucosae*
anaerobic/37 degree	*Lactobacillus mucosae*
anaerobic/37 degree	*Lactobacillus mucosae*
anaerobic/37 degree	*Lactobacillus mucosae*
anaerobic/37 degree	*Lactobacillus mucosae*

Table S5. The cultured strains and their culture atmospheres

**Atmosphere**	***species***
anaerobic/37 degree	*Lactobacillus mucosae*
anaerobic/37 degree	*Lactobacillus mucosae*
anaerobic/37 degree	*Lactobacillus mucosae*
anaerobic/37 degree	*Lactobacillus mucosae*
anaerobic/37 degree	*Lactobacillus mucosae*
anaerobic/37 degree	*Lactobacillus mucosae*
anaerobic/37 degree	*Lactobacillus mucosae*
anaerobic/37 degree	*Lactobacillus mucosae*
anaerobic/37 degree	*Lactobacillus mucosae*
anaerobic/37 degree	*Lactobacillus mucosae*
anaerobic/37 degree	*Lactobacillus mucosae*
anaerobic/37 degree	*Lactobacillus mucosae*
anaerobic/37 degree	*Lactobacillus mucosae*
anaerobic/37 degree	*Lactobacillus mucosae*
anaerobic/37 degree	*Lactobacillus mucosae*
anaerobic/37 degree	*Lactobacillus mucosae*
anaerobic/37 degree	*Lactobacillus mucosae*
anaerobic/37 degree	*Lactobacillus mucosae*
anaerobic/37 degree	*Lactobacillus mucosae*
anaerobic/37 degree	*Lactobacillus mucosae*
anaerobic/37 degree	*Lactobacillus mucosae*
anaerobic/37 degree	*Lactobacillus mucosae*
anaerobic/37 degree	*Lactobacillus mucosae*
anaerobic/37 degree	*Lactobacillus mucosae*
anaerobic/37 degree	*Lactobacillus mucosae*
anaerobic/37 degree	*Lactobacillus mucosae*
anaerobic/37 degree	*Lactobacillus mucosae*
anaerobic/37 degree	*Lactobacillus mucosae*
anaerobic/37 degree	*Lactobacillus mucosae*
anaerobic/37 degree	*Lactobacillus mucosae*
anaerobic/37 degree	*Lactobacillus mucosae*
anaerobic/37 degree	*Lactobacillus mucosae*
anaerobic/37 degree	*Lactobacillus mucosae*
anaerobic/37 degree	*Lactobacillus mucosae*
anaerobic/37 degree	*Lactobacillus mucosae*
anaerobic/37 degree	*Lactobacillus mucosae*
anaerobic/37 degree	*Lactobacillus mucosae*
anaerobic/37 degree	*Lactobacillus mucosae*
anaerobic/37 degree	*Lactobacillus mucosae*
anaerobic/37 degree	*Lactobacillus mucosae*
anaerobic/37 degree	*Lactobacillus mucosae*
anaerobic/37 degree	*Lactobacillus mucosae*
anaerobic/37 degree	*Lactobacillus mucosae*
anaerobic/37 degree	*Lactobacillus mucosae*

Table S5. The cultured strains and their culture atmospheres

**Atmosphere**	***species***
anaerobic/37 degree	*Lactobacillus mucosae*
anaerobic/37 degree	*Lactobacillus mucosae*
anaerobic/37 degree	*Lactobacillus mucosae*
anaerobic/37 degree	*Lactobacillus mucosae*
anaerobic/37 degree	*Lactobacillus mucosae*
anaerobic/37 degree	*Lactobacillus mucosae*
anaerobic/37 degree	*Lactobacillus mucosae*
anaerobic/37 degree	*Lactobacillus mucosae*
anaerobic/37 degree	*Lactobacillus mucosae*
anaerobic/37 degree	*Lactobacillus mucosae*
anaerobic/37 degree	*Lactobacillus mucosae*
anaerobic/37 degree	*Lactobacillus mucosae*
anaerobic/37 degree	*Lactobacillus mucosae*
anaerobic/37 degree	*Lactobacillus mucosae*
anaerobic/37 degree	*Lactobacillus mucosae*
anaerobic/37 degree	*Lactobacillus mucosae*
anaerobic/37 degree	*Lactobacillus mucosae*
anaerobic/37 degree	*Lactobacillus mucosae*
anaerobic/37 degree	*Lactobacillus mucosae*
anaerobic/37 degree	*Lactobacillus mucosae*
anaerobic/37 degree	*Lactobacillus mucosae*
anaerobic/37 degree	*Lactobacillus mucosae*
anaerobic/37 degree	*Lactobacillus mucosae*
anaerobic/37 degree	*Lactobacillus mucosae*
anaerobic/37 degree	*Lactobacillus mucosae*
anaerobic/37 degree	*Lactobacillus mucosae*
anaerobic/37 degree	*Lactobacillus mucosae*
anaerobic/37 degree	*Lactobacillus mucosae*
anaerobic/37 degree	*Lactobacillus mucosae*
anaerobic/37 degree	*Lactobacillus mucosae*
anaerobic/37 degree	*Lactobacillus mucosae*
anaerobic/37 degree	*Lactobacillus mucosae*
anaerobic/37 degree	*Lactobacillus mucosae*
anaerobic/37 degree	*Lactobacillus mucosae*
anaerobic/37 degree	*Lactobacillus mucosae*
anaerobic/37 degree	*Lactobacillus mucosae*
anaerobic/37 degree	*Lactobacillus mucosae*
anaerobic/37 degree	*Lactobacillus mucosae*
anaerobic/37 degree	*Lactobacillus mucosae*
anaerobic/37 degree	*Lactobacillus mucosae*
anaerobic/37 degree	*Lactobacillus mucosae*
anaerobic/37 degree	*Lactobacillus mucosae*
anaerobic/37 degree	*Lactobacillus mucosae*
anaerobic/37 degree	*Lactobacillus mucosae*

Table S5. The cultured strains and their culture atmospheres

**Atmosphere**	***species***
anaerobic/37 degree	*Lactobacillus mucosae*
anaerobic/37 degree	*Lactobacillus mucosae*
anaerobic/37 degree	*Lactobacillus mucosae*
anaerobic/37 degree	*Lactobacillus mucosae*
anaerobic/37 degree	*Lactobacillus mucosae*
anaerobic/37 degree	*Lactobacillus mucosae*
anaerobic/37 degree	*Lactobacillus mucosae*
anaerobic/37 degree	*Lactobacillus mucosae*
anaerobic/37 degree	*Lactobacillus mucosae*
anaerobic/37 degree	*Lactobacillus mucosae*
anaerobic/37 degree	*Lactobacillus mucosae*
anaerobic/37 degree	*Lactobacillus mucosae*
anaerobic/37 degree	*Lactobacillus mucosae*
anaerobic/37 degree	*Lactobacillus mucosae*
anaerobic/37 degree	*Lactobacillus mucosae*
anaerobic/37 degree	*Lactobacillus mucosae*
anaerobic/37 degree	*Lactobacillus mucosae*
anaerobic/37 degree	*Lactobacillus mucosae*
anaerobic/37 degree	*Lactobacillus mucosae*
anaerobic/37 degree	*Lactobacillus mucosae*
anaerobic/37 degree	*Lactobacillus mucosae*
anaerobic/37 degree	*Lactobacillus mucosae*
anaerobic/37 degree	*Lactobacillus mucosae*
anaerobic/37 degree	*Lactobacillus mucosae*
anaerobic/37 degree	*Lactobacillus mucosae*
anaerobic/37 degree	*Lactobacillus mucosae*
anaerobic/37 degree	*Lactobacillus mucosae*
anaerobic/37 degree	*Lactobacillus mucosae*
anaerobic/37 degree	*Lactobacillus mucosae*
anaerobic/37 degree	*Lactobacillus mucosae*
anaerobic/37 degree	*Lactobacillus mucosae*
anaerobic/37 degree	*Lactobacillus mucosae*
anaerobic/37 degree	*Lactobacillus mucosae*
anaerobic/37 degree	*Lactobacillus mucosae*
anaerobic/37 degree	*Lactobacillus mucosae*
anaerobic/37 degree	*Lactobacillus mucosae*
anaerobic/37 degree	*Lactobacillus mucosae*
anaerobic/37 degree	*Lactobacillus mucosae*
anaerobic/37 degree	*Lactobacillus mucosae*
anaerobic/37 degree	*Lactobacillus mucosae*
anaerobic/37 degree	*Lactobacillus mucosae*
anaerobic/37 degree	*Lactobacillus mucosae*
anaerobic/37 degree	*Lactobacillus mucosae*
anaerobic/37 degree	*Lactobacillus mucosae*

Table S5. The cultured strains and their culture atmospheres

Atmosphere	*species*
anaerobic/37 degree	*Lactobacillus mucosae*
anaerobic/37 degree	*Lactobacillus mucosae*
anaerobic/37 degree	*Lactobacillus mucosae*
anaerobic/37 degree	*Lactobacillus mucosae*
anaerobic/37 degree	*Lactobacillus mucosae*
anaerobic/37 degree	*Lactobacillus mucosae*
anaerobic/37 degree	*Lactobacillus mucosae*
anaerobic/37 degree	*Lactobacillus mucosae*
anaerobic/37 degree	*Lactobacillus mucosae*
anaerobic/37 degree	*Lactobacillus mucosae*
anaerobic/37 degree	*Lactobacillus mucosae*
anaerobic/37 degree	*Lactobacillus mucosae*
anaerobic/37 degree	*Lactobacillus mucosae*
anaerobic/37 degree	*Lactobacillus mucosae*
anaerobic/37 degree	*Lactobacillus mucosae*
anaerobic/37 degree	*Lactobacillus mucosae*
anaerobic/37 degree	*Lactobacillus mucosae*
anaerobic/37 degree	*Lactobacillus mucosae*
anaerobic/37 degree	*Lactobacillus mucosae*
anaerobic/37 degree	*Lactobacillus mucosae*
anaerobic/37 degree	*Lactobacillus mucosae*
anaerobic/37 degree	*Lactobacillus mucosae*
anaerobic/37 degree	*Lactobacillus mucosae*
anaerobic/37 degree	*Lactobacillus mucosae*
anaerobic/37 degree	*Lactobacillus mucosae*
anaerobic/37 degree	*Lactobacillus mucosae*
anaerobic/37 degree	*Lactobacillus mucosae*
anaerobic/37 degree	*Lactobacillus mucosae*
anaerobic/40-50 degree	*Lactobacillus mucosae*
anaerobic/42 degree	*Lactobacillus mucosae*
anaerobic/42 degree	*Lactobacillus mucosae*
anaerobic/42 degree	*Lactobacillus mucosae*
anaerobic/42 degree	*Lactobacillus mucosae*
anaerobic/42 degree	*Lactobacillus mucosae*
anaerobic/42 degree	*Lactobacillus mucosae*
anaerobic/42 degree	*Lactobacillus mucosae*
anaerobic/42 degree	*Lactobacillus mucosae*
anaerobic/42 degree	*Lactobacillus mucosae*
anaerobic/42 degree	*Lactobacillus mucosae*
anaerobic/42 degree	*Lactobacillus mucosae*
anaerobic/42 degree	*Lactobacillus mucosae*
anaerobic/42 degree	*Lactobacillus mucosae*
anaerobic/42 degree	*Lactobacillus mucosae*
anaerobic/42 degree	*Lactobacillus mucosae*

Table S5. The cultured strains and their culture atmospheres

**Atmosphere**	***species***
anaerobic/42 degree	*Lactobacillus mucosae*
anaerobic/42 degree	*Lactobacillus mucosae*
anaerobic/42 degree	*Lactobacillus mucosae*
anaerobic/42 degree	*Lactobacillus mucosae*
anaerobic/42 degree	*Lactobacillus mucosae*
anaerobic/42 degree	*Lactobacillus mucosae*
anaerobic/42 degree	*Lactobacillus mucosae*
anaerobic/42 degree	*Lactobacillus mucosae*
anaerobic/42 degree	*Lactobacillus mucosae*
anaerobic/42 degree	*Lactobacillus mucosae*
anaerobic/42 degree	*Lactobacillus mucosae*
anaerobic/42 degree	*Lactobacillus mucosae*
anaerobic/42 degree	*Lactobacillus mucosae*
anaerobic/42 degree	*Lactobacillus mucosae*
anaerobic/42 degree	*Lactobacillus mucosae*
anaerobic/42 degree	*Lactobacillus mucosae*
anaerobic/42 degree	*Lactobacillus mucosae*
anaerobic/42 degree	*Lactobacillus mucosae*
anaerobic/42 degree	*Lactobacillus mucosae*
anaerobic/42 degree	*Lactobacillus mucosae*
anaerobic/42 degree	*Lactobacillus mucosae*
anaerobic/42 degree	*Lactobacillus mucosae*
anaerobic/42 degree	*Lactobacillus mucosae*
anaerobic/42 degree	*Lactobacillus mucosae*
anaerobic/42 degree	*Lactobacillus mucosae*
anaerobic/42 degree	*Lactobacillus mucosae*
anaerobic/42 degree	*Lactobacillus mucosae*
anaerobic/42 degree	*Lactobacillus mucosae*
anaerobic/42 degree	*Lactobacillus mucosae*
anaerobic/42 degree	*Lactobacillus mucosae*
anaerobic/42 degree	*Lactobacillus mucosae*
anaerobic/42 degree	*Lactobacillus mucosae*
anaerobic/42 degree	*Lactobacillus mucosae*
anaerobic/42 degree	*Lactobacillus mucosae*
anaerobic/42 degree	*Lactobacillus mucosae*
anaerobic/42 degree	*Lactobacillus mucosae*
anaerobic/42 degree	*Lactobacillus mucosae*
anaerobic/42 degree	*Lactobacillus mucosae*
anaerobic/42 degree	*Lactobacillus mucosae*
anaerobic/42 degree	*Lactobacillus mucosae*
anaerobic/42 degree	*Lactobacillus mucosae*
anaerobic/42 degree	*Lactobacillus mucosae*
anaerobic/42 degree	*Lactobacillus mucosae*
anaerobic/42 degree	*Lactobacillus mucosae*

Table S5. The cultured strains and their culture atmospheres

**Atmosphere**	***species***
anaerobic/42 degree	*Lactobacillus mucosae*
anaerobic/42 degree	*Lactobacillus mucosae*
anaerobic/42 degree	*Lactobacillus mucosae*
anaerobic/42 degree	*Lactobacillus mucosae*
anaerobic/42 degree	*Lactobacillus mucosae*
anaerobic/42 degree	*Lactobacillus mucosae*
anaerobic/42 degree	*Lactobacillus mucosae*
anaerobic/42 degree	*Lactobacillus mucosae*
anaerobic/42 degree	*Lactobacillus mucosae*
anaerobic/42 degree	*Lactobacillus mucosae*
anaerobic/42 degree	*Lactobacillus mucosae*
anaerobic/42 degree	*Lactobacillus mucosae*
anaerobic/42 degree	*Lactobacillus mucosae*
anaerobic/42 degree	*Lactobacillus mucosae*
anaerobic/42 degree	*Lactobacillus mucosae*
anaerobic/42 degree	*Lactobacillus mucosae*
anaerobic/42 degree	*Lactobacillus mucosae*
anaerobic/42 degree	*Lactobacillus mucosae*
anaerobic/42 degree	*Lactobacillus mucosae*
anaerobic/42 degree	*Lactobacillus mucosae*
anaerobic/42 degree	*Lactobacillus mucosae*
anaerobic/42 degree	*Lactobacillus mucosae*
anaerobic/42 degree	*Lactobacillus mucosae*
anaerobic/42 degree	*Lactobacillus mucosae*
anaerobic/42 degree	*Lactobacillus mucosae*
anaerobic/42 degree	*Lactobacillus mucosae*
anaerobic/42 degree	*Lactobacillus mucosae*
anaerobic/42 degree	*Lactobacillus mucosae*
anaerobic/42 degree	*Lactobacillus mucosae*
anaerobic/42 degree	*Lactobacillus mucosae*
anaerobic/42 degree	*Lactobacillus mucosae*
anaerobic/42 degree	*Lactobacillus mucosae*
anaerobic/42 degree	*Lactobacillus mucosae*
anaerobic/42 degree	*Lactobacillus mucosae*
anaerobic/42 degree	*Lactobacillus mucosae*
anaerobic/42 degree	*Lactobacillus mucosae*
anaerobic/42 degree	*Lactobacillus mucosae*
anaerobic/42 degree	*Lactobacillus mucosae*
anaerobic/42 degree	*Lactobacillus mucosae*
anaerobic/42 degree	*Lactobacillus mucosae*
anaerobic/42 degree	*Lactobacillus mucosae*
anaerobic/42 degree	*Lactobacillus mucosae*
anaerobic/42 degree	*Lactobacillus mucosae*
anaerobic/42 degree	*Lactobacillus mucosae*

Table S5. The cultured strains and their culture atmospheres

**Atmosphere**	***species***
anaerobic/42 degree	*Lactobacillus mucosae*
anaerobic/42 degree	*Lactobacillus mucosae*
anaerobic/42 degree	*Lactobacillus mucosae*
anaerobic/42 degree	*Lactobacillus mucosae*
anaerobic/42 degree	*Lactobacillus mucosae*
anaerobic/42 degree	*Lactobacillus mucosae*
anaerobic/42 degree	*Lactobacillus mucosae*
anaerobic/42 degree	*Lactobacillus mucosae*
anaerobic/42 degree	*Lactobacillus mucosae*
anaerobic/42 degree	*Lactobacillus mucosae*
anaerobic/42 degree	*Lactobacillus mucosae*
anaerobic/42 degree	*Lactobacillus mucosae*
anaerobic/42 degree	*Lactobacillus mucosae*
anaerobic/42 degree	*Lactobacillus mucosae*
anaerobic/42 degree	*Lactobacillus mucosae*
anaerobic/42 degree	*Lactobacillus mucosae*
anaerobic/42 degree	*Lactobacillus mucosae*
anaerobic/42 degree	*Lactobacillus mucosae*
anaerobic/42 degree	*Lactobacillus mucosae*
anaerobic/42 degree	*Lactobacillus mucosae*
anaerobic/42 degree	*Lactobacillus mucosae*
anaerobic/42 degree	*Lactobacillus mucosae*
anaerobic/42 degree	*Lactobacillus mucosae*
anaerobic/42 degree	*Lactobacillus mucosae*
anaerobic/42 degree	*Lactobacillus mucosae*
anaerobic/42 degree	*Lactobacillus mucosae*
anaerobic/42 degree	*Lactobacillus mucosae*
anaerobic/42 degree	*Lactobacillus mucosae*
anaerobic/42 degree	*Lactobacillus mucosae*
anaerobic/42 degree	*Lactobacillus mucosae*
anaerobic/42 degree	*Lactobacillus mucosae*
anaerobic/42 degree	*Lactobacillus mucosae*
anaerobic/42 degree	*Lactobacillus mucosae*
anaerobic/42 degree	*Lactobacillus mucosae*
anaerobic/42 degree	*Lactobacillus mucosae*
anaerobic/42 degree	*Lactobacillus mucosae*
anaerobic/42 degree	*Lactobacillus mucosae*
anaerobic/42 degree	*Lactobacillus mucosae*
anaerobic/42 degree	*Lactobacillus mucosae*
anaerobic/42 degree	*Lactobacillus mucosae*
anaerobic/42 degree	*Lactobacillus mucosae*
anaerobic/42 degree	*Lactobacillus mucosae*
anaerobic/42 degree	*Lactobacillus mucosae*
anaerobic/42 degree	*Lactobacillus mucosae*

Table S5. The cultured strains and their culture atmospheres

Atmosphere	*species*
anaerobic/42 degree	*Lactobacillus mucosae*
anaerobic/42 degree	*Lactobacillus mucosae*
anaerobic/42 degree	*Lactobacillus mucosae*
anaerobic/42 degree	*Lactobacillus mucosae*
anaerobic/42 degree	*Lactobacillus mucosae*
anaerobic/42 degree	*Lactobacillus mucosae*
anaerobic/42 degree	*Lactobacillus mucosae*
anaerobic/42 degree	*Lactobacillus mucosae*
anaerobic/42 degree	*Lactobacillus mucosae*
anaerobic/35 degree	*Lactobacillus oris*
anaerobic/35 degree	*Lactobacillus oris*
anaerobic/35 degree	*Lactobacillus oris*
anaerobic/35 degree	*Lactobacillus oris*
anaerobic/35 degree	*Lactobacillus oris*
anaerobic/37 degree	*Lactobacillus oris*
anaerobic/37 degree	*Lactobacillus oris*
anaerobic/37 degree	*Lactobacillus oris*
anaerobic/37 degree	*Lactobacillus oris*
anaerobic/37 degree	*Lactobacillus oris*
anaerobic/37 degree	*Lactobacillus oris*
anaerobic/37 degree	*Lactobacillus oris*
anaerobic/37 degree	*Lactobacillus oris*
anaerobic/37 degree	*Lactobacillus oris*
anaerobic/37 degree	*Lactobacillus oris*
anaerobic/37 degree	*Lactobacillus oris*
anaerobic/37 degree	*Lactobacillus oris*
anaerobic/37 degree	*Lactobacillus oris*
anaerobic/37 degree	*Lactobacillus oris*
anaerobic/37 degree	*Lactobacillus oris*
anaerobic/37 degree	*Lactobacillus oris*
anaerobic/37 degree	*Lactobacillus oris*
anaerobic/37 degree	*Lactobacillus oris*
anaerobic/37 degree	*Lactobacillus oris*
anaerobic/37 degree	*Lactobacillus oris*
anaerobic/37 degree	*Lactobacillus oris*
anaerobic/37 degree	*Lactobacillus oris*
anaerobic/37 degree	*Lactobacillus oris*
anaerobic/37 degree	*Lactobacillus oris*
anaerobic/37 degree	*Lactobacillus oris*
anaerobic/37 degree	*Lactobacillus oris*
anaerobic/37 degree	*Lactobacillus oris*
anaerobic/37 degree	*Lactobacillus oris*
anaerobic/37 degree	*Lactobacillus oris*
anaerobic/42 degree	*Lactobacillus oris*

Table S5. The cultured strains and their culture atmospheres

Atmosphere	species
anaerobic/42 degree	*Lactobacillus oris*
anaerobic/42 degree	*Lactobacillus oris*
anaerobic/42 degree	*Lactobacillus oris*
anaerobic/42 degree	*Lactobacillus oris*
anaerobic/35 degree	*Lactobacillus paracasei*
anaerobic/35 degree	*Lactobacillus paracasei*
anaerobic/35 degree	*Lactobacillus paracasei*
anaerobic/37 degree	*Lactobacillus paracasei*
anaerobic/37 degree	*Lactobacillus paracasei*
anaerobic/37 degree	*Lactobacillus paracasei*
anaerobic/37 degree	*Lactobacillus paracasei*
anaerobic/37 degree	*Lactobacillus paracasei*
anaerobic/37 degree	*Lactobacillus paracasei*
anaerobic/37 degree	*Lactobacillus paracasei*
anaerobic/37 degree	*Lactobacillus paracasei*
anaerobic/37 degree	*Lactobacillus paracasei*
anaerobic/37 degree	*Lactobacillus paracasei*
anaerobic/37 degree	*Lactobacillus paracasei*
anaerobic/37 degree	*Lactobacillus paracasei*
anaerobic/42 degree	*Lactobacillus paracasei*
anaerobic/35 degree	*Lactobacillus paracasei subsp.paracasei*
anaerobic/35 degree	*Lactobacillus paracasei subsp.paracasei*
anaerobic/35 degree	*Lactobacillus paracasei subsp.paracasei*
anaerobic/35 degree	*Lactobacillus paracasei subsp.paracasei*
anaerobic/35 degree	*Lactobacillus paracasei subsp.paracasei*
anaerobic/35 degree	*Lactobacillus paracasei subsp.paracasei*
anaerobic/35 degree	*Lactobacillus paracasei subsp.paracasei*
anaerobic/35 degree	*Lactobacillus paracasei subsp.paracasei*
anaerobic/35 degree	*Lactobacillus paracasei subsp.paracasei*
anaerobic/35 degree	*Lactobacillus paracasei subsp.paracasei*
anaerobic/35 degree	*Lactobacillus paracasei subsp.paracasei*
anaerobic/35 degree	*Lactobacillus paracasei subsp.paracasei*
anaerobic/35 degree	*Lactobacillus paracasei subsp.paracasei*
anaerobic/35 degree	*Lactobacillus paracasei subsp.paracasei*
anaerobic/37 degree	*Lactobacillus paracasei subsp.paracasei*
anaerobic/37 degree	*Lactobacillus paracasei subsp.paracasei*
anaerobic/37 degree	*Lactobacillus paracasei subsp.paracasei*
anaerobic/37 degree	*Lactobacillus paracasei subsp.paracasei*
anaerobic/37 degree	*Lactobacillus paracasei subsp.paracasei*
anaerobic/37 degree	*Lactobacillus paracasei subsp.paracasei*
anaerobic/37 degree	*Lactobacillus paracasei subsp.paracasei*
anaerobic/37 degree	*Lactobacillus paracasei subsp.paracasei*
anaerobic/37 degree	*Lactobacillus paracasei subsp.paracasei*
anaerobic/37 degree	*Lactobacillus paracasei subsp.paracasei*

Table S5. The cultured strains and their culture atmospheres

**Atmosphere**	***species***
anaerobic/37 degree	*Lactobacillus paracasei subsp.paracasei*
anaerobic/37 degree	*Lactobacillus paracasei subsp.paracasei*
anaerobic/37 degree	*Lactobacillus paracasei subsp.paracasei*
anaerobic/37 degree	*Lactobacillus paracasei subsp.paracasei*
anaerobic/37 degree	*Lactobacillus paracasei subsp.paracasei*
anaerobic/37 degree	*Lactobacillus paracasei subsp.paracasei*
anaerobic/37 degree	*Lactobacillus paracasei subsp.paracasei*
anaerobic/37 degree	*Lactobacillus paracasei subsp.paracasei*
anaerobic/37 degree	*Lactobacillus paracasei subsp.paracasei*
anaerobic/37 degree	*Lactobacillus paracasei subsp.paracasei*
anaerobic/37 degree	*Lactobacillus paracasei subsp.paracasei*
anaerobic/37 degree	*Lactobacillus paracasei subsp.paracasei*
anaerobic/37 degree	*Lactobacillus paracasei subsp.paracasei*
anaerobic/37 degree	*Lactobacillus paracasei subsp.paracasei*
anaerobic/37 degree	*Lactobacillus paracasei subsp.paracasei*
anaerobic/37 degree	*Lactobacillus paracasei subsp.paracasei*
anaerobic/37 degree	*Lactobacillus paracasei subsp.paracasei*
anaerobic/37 degree	*Lactobacillus paracasei subsp.paracasei*
anaerobic/37 degree	*Lactobacillus paracasei subsp.paracasei*
anaerobic/37 degree	*Lactobacillus paracasei subsp.paracasei*
anaerobic/37 degree	*Lactobacillus paracasei subsp.paracasei*
anaerobic/37 degree	*Lactobacillus paracasei subsp.paracasei*
anaerobic/37 degree	*Lactobacillus paracasei subsp.paracasei*
anaerobic/37 degree	*Lactobacillus paracasei subsp.paracasei*
anaerobic/37 degree	*Lactobacillus paracasei subsp.paracasei*
anaerobic/37 degree	*Lactobacillus paracasei subsp.paracasei*
anaerobic/37 degree	*Lactobacillus paracasei subsp.paracasei*
anaerobic/37 degree	*Lactobacillus paracasei subsp.paracasei*
anaerobic/37 degree	*Lactobacillus paracasei subsp.paracasei*
anaerobic/37 degree	*Lactobacillus paracasei subsp.paracasei*
anaerobic/37 degree	*Lactobacillus paracasei subsp.paracasei*
anaerobic/37 degree	*Lactobacillus paracasei subsp.paracasei*
anaerobic/37 degree	*Lactobacillus paracasei subsp.paracasei*
anaerobic/37 degree	*Lactobacillus paracasei subsp.paracasei*
anaerobic/37 degree	*Lactobacillus paracasei subsp.paracasei*
anaerobic/37 degree	*Lactobacillus paracasei subsp.paracasei*
anaerobic/37 degree	*Lactobacillus paracasei subsp.paracasei*
anaerobic/37 degree	*Lactobacillus paracasei subsp.paracasei*
anaerobic/37 degree	*Lactobacillus paracasei subsp.paracasei*
anaerobic/42 degree	*Lactobacillus paracasei subsp.paracasei*
anaerobic/42 degree	*Lactobacillus paracasei subsp.paracasei*
anaerobic/42 degree	*Lactobacillus paracasei subsp.paracasei*
anaerobic/42 degree	*Lactobacillus paracasei subsp.paracasei*
anaerobic/42 degree	*Lactobacillus paracasei subsp.paracasei*

Table S5. The cultured strains and their culture atmospheres

**Atmosphere**	***species***
anaerobic/42 degree	*Lactobacillus paracasei subsp.paracasei*
anaerobic/37 degree	*Lactobacillus pentosus*
anaerobic/37 degree	*Lactobacillus pentosus*
anaerobic/37 degree	*Lactobacillus pentosus*
anaerobic/37 degree	*Lactobacillus pentosus*
anaerobic/37 degree	*Lactobacillus pentosus*
anaerobic/37 degree	*Lactobacillus pentosus*
anaerobic/37 degree	*Lactobacillus pentosus*
anaerobic/37 degree	*Lactobacillus pentosus*
anaerobic/37 degree	*Lactobacillus pentosus*
anaerobic/37 degree	*Lactobacillus pentosus*
anaerobic/37 degree	*Lactobacillus pentosus*
anaerobic/37 degree	*Lactobacillus pentosus*
anaerobic/37 degree	*Lactobacillus pentosus*
anaerobic/37 degree	*Lactobacillus pentosus*
anaerobic/37 degree	*Lactobacillus pentosus*
anaerobic/37 degree	*Lactobacillus pentosus*
anaerobic/37 degree	*Lactobacillus pentosus*
anaerobic/37 degree	*Lactobacillus pentosus*
anaerobic/37 degree	*Lactobacillus pentosus*
anaerobic/37 degree	*Lactobacillus pentosus*
anaerobic/37 degree	*Lactobacillus pentosus*
anaerobic/37 degree	*Lactobacillus pentosus*
anaerobic/37 degree	*Lactobacillus pentosus*
anaerobic/37 degree	*Lactobacillus pentosus*
anaerobic/37 degree	*Lactobacillus pentosus*
anaerobic/37 degree	*Lactobacillus pentosus*
anaerobic/37 degree	*Lactobacillus pentosus*
anaerobic/37 degree	*Lactobacillus pentosus*
anaerobic/37 degree	*Lactobacillus pentosus*
anaerobic/37 degree	*Lactobacillus pentosus*
anaerobic/37 degree	*Lactobacillus pentosus*
anaerobic/37 degree	*Lactobacillus pentosus*
anaerobic/37 degree	*Lactobacillus pentosus*
anaerobic/37 degree	*Lactobacillus pentosus*
anaerobic/37 degree	*Lactobacillus pentosus*
anaerobic/37 degree	*Lactobacillus pentosus*
anaerobic/37 degree	*Lactobacillus pentosus*
anaerobic/37 degree	*Lactobacillus pentosus*
anaerobic/37 degree	*Lactobacillus pentosus*
anaerobic/37 degree	*Lactobacillus pentosus*
anaerobic/37 degree	*Lactobacillus pentosus*
anaerobic/37 degree	*Lactobacillus pentosus*
anaerobic/37 degree	*Lactobacillus pentosus*

Table S5. The cultured strains and their culture atmospheres

**Atmosphere**	***species***
anaerobic/37 degree	*Lactobacillus pentosus*
anaerobic/37 degree	*Lactobacillus pentosus*
anaerobic/37 degree	*Lactobacillus pentosus*
anaerobic/37 degree	*Lactobacillus pentosus*
anaerobic/37 degree	*Lactobacillus pentosus*
anaerobic/37 degree	*Lactobacillus pentosus*
anaerobic/37 degree	*Lactobacillus pentosus*
anaerobic/37 degree	*Lactobacillus pentosus*
anaerobic/37 degree	*Lactobacillus pentosus*
anaerobic/37 degree	*Lactobacillus pentosus*
anaerobic/37 degree	*Lactobacillus pentosus*
anaerobic/37 degree	*Lactobacillus pentosus*
anaerobic/37 degree	*Lactobacillus pentosus*
anaerobic/37 degree	*Lactobacillus pentosus*
anaerobic/37 degree	*Lactobacillus pentosus*
anaerobic/37 degree	*Lactobacillus pentosus*
anaerobic/37 degree	*Lactobacillus pentosus*
anaerobic/37 degree	*Lactobacillus pentosus*
anaerobic/37 degree	*Lactobacillus pentosus*
anaerobic/37 degree	*Lactobacillus pentosus*
anaerobic/37 degree	*Lactobacillus pentosus*
anaerobic/37 degree	*Lactobacillus pentosus*
anaerobic/37 degree	*Lactobacillus pentosus*
anaerobic/37 degree	*Lactobacillus pentosus*
anaerobic/37 degree	*Lactobacillus pentosus*
anaerobic/37 degree	*Lactobacillus pentosus*
anaerobic/37 degree	*Lactobacillus pentosus*
anaerobic/37 degree	*Lactobacillus pentosus*
anaerobic/37 degree	*Lactobacillus pentosus*
anaerobic/37 degree	*Lactobacillus pentosus*
anaerobic/37 degree	*Lactobacillus pentosus*
anaerobic/37 degree	*Lactobacillus pentosus*
anaerobic/37 degree	*Lactobacillus pentosus*
anaerobic/37 degree	*Lactobacillus pentosus*
anaerobic/37 degree	*Lactobacillus pentosus*
anaerobic/37 degree	*Lactobacillus pentosus*
anaerobic/37 degree	*Lactobacillus pentosus*
anaerobic/35 degree	*Lactobacillus plantarum*
anaerobic/35 degree	*Lactobacillus plantarum*
anaerobic/35 degree	*Lactobacillus plantarum*
anaerobic/37 degree	*Lactobacillus plantarum*
anaerobic/37 degree	*Lactobacillus plantarum*
anaerobic/37 degree	*Lactobacillus plantarum*
anaerobic/37 degree	*Lactobacillus plantarum*

Table S5. The cultured strains and their culture atmospheres

Atmosphere	*species*
anaerobic/37 degree	*Lactobacillus plantarum*
anaerobic/37 degree	*Lactobacillus plantarum*
anaerobic/37 degree	*Lactobacillus plantarum*
anaerobic/37 degree	*Lactobacillus plantarum*
anaerobic/37 degree	*Lactobacillus plantarum*
anaerobic/42 degree	*Lactobacillus plantarum*
anaerobic/42 degree	*Lactobacillus plantarum*
anaerobic/42 degree	*Lactobacillus plantarum*
anaerobic/42 degree	*Lactobacillus plantarum*
anaerobic/42 degree	*Lactobacillus plantarum*
anaerobic/42 degree	*Lactobacillus plantarum*
anaerobic/42 degree	*Lactobacillus plantarum*
anaerobic/42 degree	*Lactobacillus plantarum*
anaerobic/42 degree	*Lactobacillus plantarum*
anaerobic/42 degree	*Lactobacillus plantarum*
anaerobic/42 degree	*Lactobacillus plantarum*
anaerobic/42 degree	*Lactobacillus plantarum*
anaerobic/42 degree	*Lactobacillus plantarum*
anaerobic/42 degree	*Lactobacillus plantarum*
anaerobic/42 degree	*Lactobacillus plantarum*
anaerobic/42 degree	*Lactobacillus plantarum*
anaerobic/42 degree	*Lactobacillus plantarum*
anaerobic/42 degree	*Lactobacillus plantarum*
anaerobic/42 degree	*Lactobacillus plantarum*
anaerobic/42 degree	*Lactobacillus plantarum*
anaerobic/42 degree	*Lactobacillus plantarum*
anaerobic/42 degree	*Lactobacillus plantarum subsp.plantarum*
anaerobic/42 degree	*Lactobacillus plantarum subsp.plantarum*
anaerobic/37 degree	*Lactobacillus reuteri*
anaerobic/37 degree	*Lactobacillus reuteri*
anaerobic/37 degree	*Lactobacillus reuteri*
anaerobic/37 degree	*Lactobacillus reuteri*
anaerobic/37 degree	*Lactobacillus reuteri*
anaerobic/37 degree	*Lactobacillus reuteri*
anaerobic/37 degree	*Lactobacillus reuteri*
anaerobic/37 degree	*Lactobacillus reuteri*
anaerobic/37 degree	*Lactobacillus reuteri*
anaerobic/37 degree	*Lactobacillus reuteri*
anaerobic/37 degree	*Lactobacillus reuteri*
anaerobic/37 degree	*Lactobacillus reuteri*
anaerobic/37 degree	*Lactobacillus reuteri*
anaerobic/37 degree	*Lactobacillus reuteri*
anaerobic/37 degree	*Lactobacillus reuteri*
anaerobic/37 degree	*Lactobacillus reuteri*

Table S5. The cultured strains and their culture atmospheres

**Atmosphere**	***species***
anaerobic/37 degree	*Lactobacillus reuteri*
anaerobic/37 degree	*Lactobacillus reuteri*
anaerobic/37 degree	*Lactobacillus reuteri*
anaerobic/37 degree	*Lactobacillus reuteri*
anaerobic/37 degree	*Lactobacillus reuteri*
anaerobic/37 degree	*Lactobacillus reuteri*
anaerobic/37 degree	*Lactobacillus reuteri*
anaerobic/37 degree	*Lactobacillus reuteri*
anaerobic/37 degree	*Lactobacillus reuteri*
anaerobic/37 degree	*Lactobacillus reuteri*
anaerobic/37 degree	*Lactobacillus reuteri*
anaerobic/37 degree	*Lactobacillus reuteri*
anaerobic/37 degree	*Lactobacillus reuteri*
anaerobic/37 degree	*Lactobacillus reuteri*
anaerobic/37 degree	*Lactobacillus reuteri*
anaerobic/37 degree	*Lactobacillus reuteri*
anaerobic/37 degree	*Lactobacillus reuteri*
anaerobic/37 degree	*Lactobacillus reuteri*
anaerobic/37 degree	*Lactobacillus reuteri*
anaerobic/37 degree	*Lactobacillus reuteri*
anaerobic/37 degree	*Lactobacillus reuteri*
anaerobic/37 degree	*Lactobacillus reuteri*
anaerobic/37 degree	*Lactobacillus reuteri*
anaerobic/37 degree	*Lactobacillus reuteri*
anaerobic/37 degree	*Lactobacillus reuteri*
anaerobic/37 degree	*Lactobacillus reuteri*
anaerobic/37 degree	*Lactobacillus reuteri*
anaerobic/37 degree	*Lactobacillus reuteri*
anaerobic/37 degree	*Lactobacillus reuteri*
anaerobic/37 degree	*Lactobacillus reuteri*
anaerobic/37 degree	*Lactobacillus reuteri*
anaerobic/37 degree	*Lactobacillus reuteri*
anaerobic/37 degree	*Lactobacillus reuteri*
anaerobic/37 degree	*Lactobacillus reuteri*
anaerobic/37 degree	*Lactobacillus reuteri*
anaerobic/37 degree	*Lactobacillus reuteri*
anaerobic/37 degree	*Lactobacillus reuteri*
anaerobic/37 degree	*Lactobacillus reuteri*
anaerobic/37 degree	*Lactobacillus reuteri*
anaerobic/40-50 degree	*Lactobacillus reuteri*
anaerobic/40-50 degree	*Lactobacillus reuteri*
anaerobic/40-50 degree	*Lactobacillus reuteri*
anaerobic/40-50 degree	*Lactobacillus reuteri*
anaerobic/40-50 degree	*Lactobacillus reuteri*

Table S5. The cultured strains and their culture atmospheres

Atmosphere	*species*
anaerobic/40-50 degree	*Lactobacillus reuteri*
anaerobic/40-50 degree	*Lactobacillus reuteri*
anaerobic/40-50 degree	*Lactobacillus reuteri*
anaerobic/40-50 degree	*Lactobacillus reuteri*
anaerobic/40-50 degree	*Lactobacillus reuteri*
anaerobic/40-50 degree	*Lactobacillus reuteri*
anaerobic/40-50 degree	*Lactobacillus reuteri*
anaerobic/40-50 degree	*Lactobacillus reuteri*
anaerobic/40-50 degree	*Lactobacillus reuteri*
anaerobic/40-50 degree	*Lactobacillus reuteri*
anaerobic/40-50 degree	*Lactobacillus reuteri*
anaerobic/40-50 degree	*Lactobacillus reuteri*
anaerobic/40-50 degree	*Lactobacillus reuteri*
anaerobic/40-50 degree	*Lactobacillus reuteri*
anaerobic/40-50 degree	*Lactobacillus reuteri*
anaerobic/40-50 degree	*Lactobacillus reuteri*
anaerobic/40-50 degree	*Lactobacillus reuteri*
anaerobic/40-50 degree	*Lactobacillus reuteri*
anaerobic/40-50 degree	*Lactobacillus reuteri*
anaerobic/40-50 degree	*Lactobacillus reuteri*
anaerobic/40-50 degree	*Lactobacillus reuteri*
anaerobic/40-50 degree	*Lactobacillus reuteri*
anaerobic/40-50 degree	*Lactobacillus reuteri*
anaerobic/40-50 degree	*Lactobacillus reuteri*
anaerobic/40-50 degree	*Lactobacillus reuteri*
anaerobic/42 degree	*Lactobacillus reuteri*
anaerobic/42 degree	*Lactobacillus reuteri*
anaerobic/42 degree	*Lactobacillus reuteri*
anaerobic/42 degree	*Lactobacillus reuteri*
anaerobic/42 degree	*Lactobacillus reuteri*
anaerobic/42 degree	*Lactobacillus reuteri*
anaerobic/42 degree	*Lactobacillus reuteri*
anaerobic/42 degree	*Lactobacillus reuteri*
anaerobic/42 degree	*Lactobacillus reuteri*
anaerobic/42 degree	*Lactobacillus reuteri*
anaerobic/42 degree	*Lactobacillus reuteri*
anaerobic/42 degree	*Lactobacillus reuteri*
anaerobic/42 degree	*Lactobacillus reuteri*
anaerobic/42 degree	*Lactobacillus reuteri*
anaerobic/42 degree	*Lactobacillus reuteri*
anaerobic/42 degree	*Lactobacillus reuteri*
anaerobic/42 degree	*Lactobacillus reuteri*
anaerobic/42 degree	*Lactobacillus reuteri*
anaerobic/42 degree	*Lactobacillus reuteri*

Table S5. The cultured strains and their culture atmospheres

**Atmosphere**	***species***
anaerobic/42 degree	*Lactobacillus reuteri*
anaerobic/42 degree	*Lactobacillus reuteri*
anaerobic/42 degree	*Lactobacillus reuteri*
anaerobic/42 degree	*Lactobacillus reuteri*
anaerobic/42 degree	*Lactobacillus reuteri*
anaerobic/42 degree	*Lactobacillus reuteri*
anaerobic/42 degree	*Lactobacillus reuteri*
anaerobic/42 degree	*Lactobacillus reuteri*
anaerobic/42 degree	*Lactobacillus reuteri*
anaerobic/42 degree	*Lactobacillus reuteri*
anaerobic/42 degree	*Lactobacillus reuteri*
anaerobic/42 degree	*Lactobacillus reuteri*
anaerobic/42 degree	*Lactobacillus reuteri*
anaerobic/42 degree	*Lactobacillus reuteri*
anaerobic/42 degree	*Lactobacillus reuteri*
anaerobic/42 degree	*Lactobacillus reuteri*
anaerobic/42 degree	*Lactobacillus reuteri*
anaerobic/42 degree	*Lactobacillus reuteri*
anaerobic/42 degree	*Lactobacillus reuteri*
anaerobic/37 degree	*Lactobacillus rhamnosus*
anaerobic/37 degree	*Lactobacillus rhamnosus*
anaerobic/37 degree	*Lactobacillus rhamnosus*
anaerobic/37 degree	*Lactobacillus sakei*
anaerobic/35 degree	*Lactobacillus salivarius*
anaerobic/35 degree	*Lactobacillus salivarius*
anaerobic/35 degree	*Lactobacillus salivarius*
anaerobic/35 degree	*Lactobacillus salivarius*
anaerobic/37 degree	*Lactobacillus salivarius*
anaerobic/37 degree	*Lactobacillus salivarius*
anaerobic/37 degree	*Lactobacillus salivarius*
[truncated: 193,033 more chars]
